# Causal evidence linking injury-associated DNA methylation to the risk of developing depression or post-traumatic stress disorder

**DOI:** 10.1097/MD.0000000000048310

**Published:** 2026-04-17

**Authors:** Lian Liu, Ziyu Zhu, Mengran Du, Xin Liu, Qiuhao Tan, Longwei Xiao, Xiaoyang Zheng, Zhijian Huang, Zhengbu Liao

**Affiliations:** a Department of Neurosurgery, The First Affiliated Hospital of Chongqing Medical University, Chongqing, China.

**Keywords:** co-methylation network, MDD, Mendelian randomization, PTSD

## Abstract

Depression and post-traumatic stress disorder (PTSD) are common psychiatric disorders following physical trauma. However, genetic evidence between these disorders is lacking. DNA methylation (DNAm) is implicated in the response to traumatic events and has been proposed as a promising therapeutic target. This study aimed to investigate the causal evidence of injury-associated DNAm and the subsequent onset of depression or PTSD. We performed co-methylation analysis of the whole-genome bisulfite sequencing (WGBS) data from physical trauma patients who developed subsequent depression or PTSD. DNA methylation quantitative trait loci (mQTL) from the GoDMC database, expression quantitative trait loci (eQTL) from the eQTLGen consortium (phase Ⅰ), and genome-wide association (GWAS) summary statistics from the FinnGen consortium were used for causal inference using the mendelian randomization (MR) framework. Our results showed significant causal genetic evidence for injury-associated DNAm changes at cg24526596 (DLGAP2), cg00157656 (ERICH1), cg12317217 (PCDHA2), and cg12661624 (PTPRN2) on depression onset. However, no strong causal evidence was found for injury-associated DNAm and PTSD. The present study provided genetic evidence supporting the causal role of DNAm in the onset of depression or PTSD after injury and identified DLGAP2, ERICH1, PCDHA2, and PTPRN2 as the candidate genes worthy of further exploratory investigation.

## 1. Introduction

Traumatic injury is a leading cause of death and disability, affecting millions of people per year. More than 30% of patients who suffered from traumatic injury may develop psychiatric disorders, especially depression and post-traumatic stress disorder (PTSD). Psychiatric disorders due to traumatic injury have posed an enormous burden on the patients and society.^[1,2]^ Among the broad range of psychiatric sequelae, depression and PTSD are the most frequent and widely studied psychiatric disorders that arise after the traumatic events such as traumatic burn, traffic accidents, and traumatic brain injury (TBI).^[3,4]^

The high comorbidity of physical trauma and subsequent psychiatric sequelae is an area of growing interest. Understanding this overlap can contribute to better clinical decision making, thereby improving patients’ life quality.^[5]^ Current management and treatment of acute traumatic injury is well established, but early identification and intervention of the risk of psychiatric sequelae has been infrequent and inadequate due to the challenge of diagnosis based on heterogeneous and common symptoms, as well as incongruent healthcare strategies.^[6,7]^ Although efforts have been directed at identification of the potential risk factors for prevention and early treatment of post-injury psychiatric sequelae over the past years.^[6]^ Nevertheless, the early recognition of patients at risk for pervasive mental disorders after injury remains an unresolved and urgent problem, and our understanding of the mechanistic basis of this comorbid situation is still in its infancy.

The co-occurrence of forms of physical injury and subsequent psychiatric disorders can be attributed to compromised neural circuits that subserve the normal function of brain networks as a result of molecular changes due to external insults.^[8]^ For example, recent studies have suggested that the hormone and neurotransmitter disruptions due to stress response or direct morphological changes caused by physical injury may lead to series of mental disorders following traumatic events.^[6,8]^ Such disruption can result from either direct brain injury or systemic stress responses to bodily trauma. In response to traumatic events, large-scale tissue and cell-specific DNA methylation (DNAm) patterns changes could occur and contribute to the pathophysiological processes via disturbing the dynamic balance between DNA methyltransferase (DNMT) and ten-eleven translocation (TET) family enzymes, resulting in altered gene expression profiles.^[9,10]^ Therefore, DNAm, as the major epigenetic modification, has been proposed to play a mechanistic role in the pathogenesis of psychiatric disorders following traumatic injury by selectively regulating the transcription and expression of certain genes.^[11]^ Unraveling the causal DNA methylation underlying these comorbid conditions could provide new insights into the pathways involved in the disrupted neurocircuitry and is essential for the future development of effective prevention and treatment strategies.^[12]^

Although mounting evidence has suggested the strong association between traumatic injury and the co-occurring psychiatric disorders; however, much of this evidence is undermined by the observational design nature, relatively small samplesize, and potential confounders.^[3,12]^ It is also unclear whether DNAm changes can cause the onset of these diseases. Mendelian randomization (MR) is a causal inference method that uses single nucleotide polymorphisms (SNPs) as the instrumental variables (IVs) to estimate the causal relationship between exposure and outcome. Due to the random allocation of alleles, MR is more resistant to confounding factors and other biases than traditional epidemiological studies.^[13]^

Herein, in the present study, we aim to investigate the DNAm patterns underlying the onset of depression and PTSD after injury and to probe the casual inference within the MR framework. Our results provided new insights into the association of DNAm with the onset of psychiatric disorders after the injury.

## 2. Materials and methods

### 2.1. Study design

**Figure 1.**
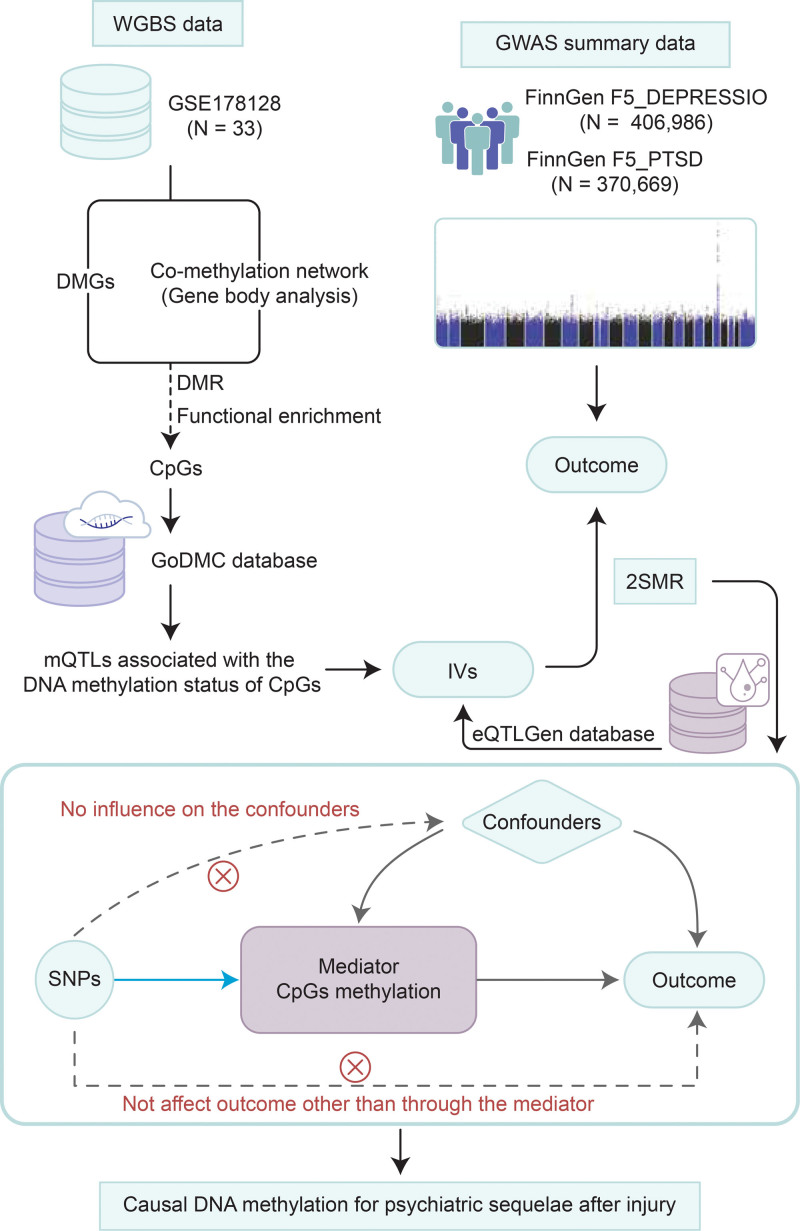
Workflow of the present study demonstrating the causal relationship between injury-associated DNAm changes and the onset of depression or PTSD. 2SMR = 2-sample Mendelian randomization, DMGs = differentially methylated genes, GWAS = genome-wide association, IVs = instrumental variables, SNP = single nucleotide polymorphism, WGBS = whole-genome bisulfite sequencing.

The design of the present study is shown in Figure 1. First, we investigated the potential DNAm patterns underlying the onset of depression or PTSD after injury using the co-methylation analysis. Then, to determine whether these DNAm predispose patients with higher liability to psychiatric sequelae, we applied MR analyses according to the Strengthening the Reporting of Observational Studies in Epidemiology using Mendelian randomization (STROBE-MR) guideline.^[14]^

### 2.2. Data source

Whole-genome bisulfite sequencing (WGBS) data were obtained from the GEO public database under accession number GSE178128,^[15]^ in which peripheral blood mononuclear cells from physical trauma patients with depression or PTSD onset were bisulfite sequenced after controlling for age, sex, race, and mechanism of injury (N = 33). The cohort encompassed patients with various injury mechanisms, primarily falls and motor vehicle accidents. To obtain DNA methylation quantitative trait loci (mQTL) as the genetic instrument for CpGs (cytosine-phosphate-guanine sites), the GoDMC database was accessed, which systematically mapped mQTL based on 32,851 blood samples.^[16]^ We drew genome-wide association studies (GWAS) summary statistics for depression (F5_DEPRESSIO, cases = 47,696, controls = 3,59,290) and PTSD (F5_PTSD, cases = 2615, controls = 3,68,054) from the FinnGen R10 release,^[17]^ a large nationwide biobank with genetic information available for over 4,30,000 individuals, the diagnoses were based on the International Classification of Diseases (ICD)-9 and ICD-10. Since DNAm can lead to altered transcript levels, the eQTLGen consortium (phase I), which contains cis-eQTL instruments from 31,684 blood samples,^[18]^ was accessed to perform additional MR analysis to validate the identified putative causal genes. All the GWAS summary statistics were restricted to European ancestry to minimize the bias caused by population stratification.

### 2.3. Co-methylation network and enrichment analyses

For raw bedgraph files processing, the methrix R package was used to generate Bismark CpG reports and transform them into a BSseq object for downstream analyses.^[19]^ During preprocessing, CpG sites with low sequencing coverage were filtered out, batch effects were assessed and adjusted where applicable, and cell-type heterogeneity was considered using reference-based estimates to minimize potential confounding. Comethyl R package was applied to perform the co-methylation analysis by constructing the weighted region co-methylation network from the processed WGBS data using CpGs grouped by gene body annotation, which reflects the expression status and allows a better understanding of the functional interpretation.^[20]^ Differentially methylated regions (DMRs) were identified using the dmrseq R package with a *q*-value < 0.05 as the significance threshold.^[21]^ The UCSC hg38 was used as the reference genome. Annotation of genomic regions was performed using the annotatr R package,^[22]^ and the circlize R package was used for genomic visualization. Functional enrichment analyses of the mapped module genes for gene ontology (GO) including biological process (BP), molecular function (MF), and cellular component (CC) were performed using the Genekitr and clusterProfiler R packages,^[23,24]^ with *P*-value and *q*-value cutoffs set at .01.

### 2.4. Genetic instruments selection

In addition to the previously identified differentially methylated genes (DMGs) that could be potential predictors of depression or PTSD onset following injury, the gene list of the clustered protocadherins (cPCDHs) family was retrieved from the HUGO Gene Nomenclature Committee (HGNC) database because the cPCDHs play important roles in the co-methylation network and were partially differentially methylated among the patients. The associated CpG sites were retrieved from the Illumina Infinium EPIC annotation. Related mQTLs of each CpG were selected based on genome-wide significance threshold (*P* < 5e−8) and low linkage disequilibrium (LD) threshold (*R*^2^ < 0.001 and clumping window size = 10,000 kb) using PLINK version 1.9. The exposure and outcome data were harmonized to remove palindromic SNPs. A phenome-wide scan was performed to remove SNPs associated with any confounding risk factors using the GWASATLAS^[25]^ (*P* < 5e−8).

### 2.5. Two-sample mendelian randomization (2SMR)

The TwoSampleMR^[26]^ R package version 0.5.9 was applied to evaluate the causal relationship between genetically predicted DNAm CpG sites and depression or PTSD outcomes, and the causal relationship between identified putative causal gene expression on depression or PTSD. The inverse-variance weighted (IVW) method was used as the main approach to evaluate the potential causal effect estimates when multiple SNPs were selected, and the Wald ratio was applied when a single SNP was available.

### 2.6. Statistical analyses

Euclidean distance was calculated for sample clustering, and a soft power threshold was determined using Pearson correlation to detect modules. Identified modules were correlated using Bicor correlation. The MR analyses of the present study were performed based on the 3 core IVs assumptions: The IVs should be highly associated with exposure; IVs should not be associated with any confounders; the causal effect of IVs should be through exposure only.^[13]^ The causal effect was calculated as:

β_DNAm-psychiatric_ = β_SNP-psychiatric_/β_SNP-DNAm_.

The 2SMR obtained the estimated effect size of DNA methylation on psychiatric sequelae (β_DNAm-psychiatric_) by dividing the estimated effect size of the SNP on psychiatric sequelae (β_SNP-psychiatric_) by the estimated effect size of the SNP on DNA methylation (β_SNP-DNAm_). *F*-statistics were calculated to reduce weak IV bias, and an *F*-statistic < 10 was considered weak IV and was therefore removed.^[27]^ As the sensitivity analyses, the potential presence of horizontal pleiotropy was evaluated using the MR-Egger intercept (*P* < .05 indicated significant horizontal pleiotropy). Heterogeneity tests were performed to evaluate the viability of the effects using Cochran *Q* test (*P* < .05 was considered significant heterogeneity). Leave-one-out analysis was performed to validate the robustness of the causal effect estimates. To detect reverse causality, the Steiger test was applied (*P* < .05 indicated no significant evidence of reverse causality). After removing cross-reactive CpGs,^[28]^ each included CpG site was treated as an independent exposure. To account for multiple testing, the Benjamini-Hochberg correction was implemented with a false discovery rate (FDR) < 0.05 for significance. Statistical significance is denoted as: *P < .05, **P < .01; ***P < .001, and ****P < .0001.

### 2.7. Ethics statement

All data used in this study were derived from previous studies conducted under the appropriate regulations and approvals. The present study was waived from ethical approval due to the use of previously published public data.

## 3. Results

### 3.1. Co-methylation analysis revealed cPCDHs as the key regulators

**Figure 2.**
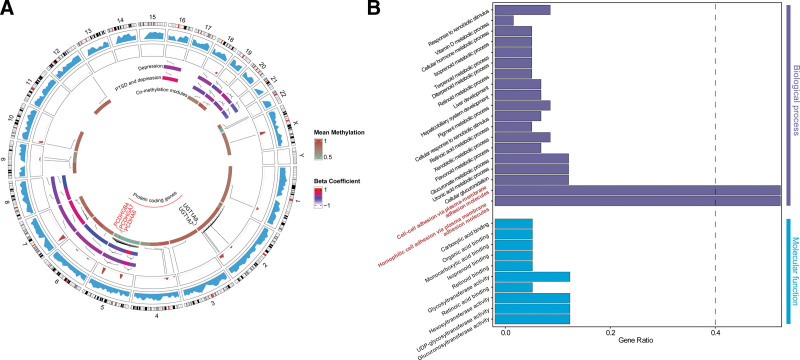
Co-methylation network analysis of depression or PTSD patients after physical injury. (A) Circular plot of the co-methylation network. The outermost track is chromosome bands of the whole genome, mapped by the genomic density of co-methylation regions in blue. The density plot of the identified co-methylation modules is shown in red. The DMRs were visualized with the inner track (depression and PTSD patients) and the outer track (depression patients) in beta value, which is the condition difference coefficient. The innermost track represents the mean methylation status of the co-methylation modules excluding the unassigned gray module for both patient groups. Hub regions were labeled and PCDHGB4, PCDHGA7, and PCDHA6 were also found within DMRs. (B) Bar plot showing the significant GO terms, including BP and MF. The CC category did not enrich any terms and is not shown. BP = biological process, DMRs = differentially methylated regions, GO = gene ontology, MF = molecular function, PTSD = post-traumatic stress disorder.

Five hub regions were identified from the co-methylation network of 19,241 regions and mapped to protein-coding genes: PCDHGB4 (turquoise module), PCDHGA7 (turquoise module), PCDHA6 (blue module), UGT1A7 (brown module), and UGT1A9 (brown module). Notably, the blue and turquoise modules were mainly mapped to the cPCDHs family, and the brown module was mainly mapped to the UGT1 family. The cPCDHs were of most interest, as a hub region mapped to PCDHA6 (*q* < 0.02; MDD_beta: 0.18; PTSD_MDD_beta: 0.07) was also found in the DMRs and none of the UGT1 family showed significant difference among the patients (see Fig. 2A and Tables S1 and S2, Supplemental Digital Content, https://links.lww.com/MD/R684). Consistently, the GO BP enrichment analysis for the whole co-methylation network suggested significant enrichment in “Homophilic cell adhesion via plasma membrane adhesion molecules,” “Cell − cell adhesion via plasma − membrane adhesion molecules,” followed by “Cellular glucuronidation” and “Uronic acid metabolic process” (see Fig. 2B). Taken together, the cPCDHs could be implicated in the biological processes underlying depression or PTSD onset after injury. Detailed gene body co-methylation analysis results, DMR statistics, the list of implicated genes, and functional enrichment results are available in Figure S1, Supplemental Digital Content, https://links.lww.com/MD/R683 and Tables S1–S7, Supplemental Digital Content, https://links.lww.com/MD/R684.

### 3.2. Causal injury-associated DNAm associated with depression and PTSD

**Figure 3.**
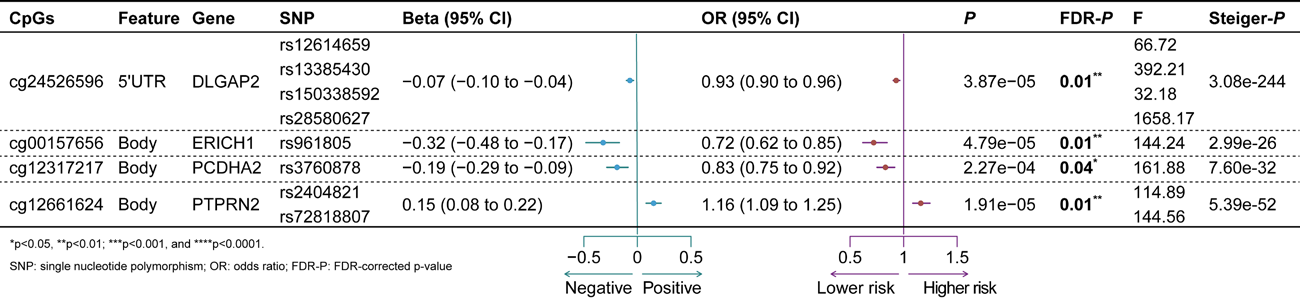
Significant 2SMR causal effect estimates for the association between injury-associated DNAm and onset of depression or PTSD. 2SMR = 2-sample Mendelian randomization, CI = confidence interval, DNAm = DNA methylation, FDR = false discovery rate, OR = odds ratio, PTSD = post-traumatic stress disorder, SNPs = single nucleotide polymorphisms. PTSD = post-traumatic stress disorder.

A total of 4407 relevant CpGs of 88 genes were retrieved, and 901 CpGs from 44 genes were included for 2SMR after mQTLs matching and clumping. The 2SMR analysis identified 4 significant CpGs (cg12661624: PTPRN2, cg24526596: DLGAP2, cg00157656: ERICH1, and cg12317217: PCDHA2) in depression outcome after FDR adjustment (FDR-*P*val < .05), and no significant causal associations were detected for PTSD after FDR adjustment (see Fig. 3). No evidence indicated weak instruments for all CpGs (smallest *F* statistic > 10). Sensitivity analyses of MR results, phenome-wide association scans for instrumental SNPs, and complementary eQTL validation analyses are presented in Figures S2–S4, Supplemental Digital Content, https://links.lww.com/MD/R683. The top 10 significant correlations between DNAm and depression or PTSD onset are shown in Table 1. Genome-wide association summary statistics of injury-associated DNAm at 901 unique CpGs and depression or PTSD are shown in Table S8, Supplemental Digital Content, https://links.lww.com/MD/R684. Specifically, the methylation at the CpG cg12661624 (PTPRN2, *P* < 5e−4, FDR: 0.04, IVW) significantly increased the risk of depression (OR: 1.16 [95% CI: 1.09–1.25]). However, methylation at the CpG cg00157656 (ERICH1, *P* < 5e−5, FDR: 0.01, Wald ratio) significantly decreased the risk of depression (OR: 0.72 [95% CI: 0.62–0.85]). For sensitivity analysis, no horizontal pleioptropy was detected for the causal estimates (MR Egger intercept *P* > .05), and no evidence of heterogeneity was found based on the Cochran *Q* test (*P* > .05) for cg12661624 and cg24526596. Leave-one-out analysis revealed a robust causal association between the methylation at the CpG cg24526596 (DLGAP2, *P* < 5e−5, FDR < 0.01, IVW) and depression. The results of the sensitivity analysis for cg24526596 were consistent with the main IVW estimate. To validate the mQTL MR results, MR analyses for eQTLs were performed and identified a significant causal association between PTPRN2 and depression (*P* < .05, OR: 1.02 [95% CI: 1.00–1.04]), no statistical significance was found for ERICH1, and no available eQTL information was available for DLGAP2 and PCDHA2 (see Tables 2, S9, and S10, Supplemental Digital Content, https://links.lww.com/MD/R684). However, the validity of the sensitivity analysis can be affected by the small number of IVs (n < 5), and we are unable to perform sensitivity analysis for cg00157656 and cg12317217 due to small number of SNPs. The Steiger tests suggested that the direction of association of all identified significant CpGs was from DNAm to depression (*P* < .05). Phenome-wide scan from GWASATLAS of the included SNPs did not detect any confounding factors (see Table S11, Supplemental Digital Content, https://links.lww.com/MD/R684).

**Table 1:** Two-sample mendelian randomization results of the top 10 correlations of the DNAm to depression or post-traumatic stress disorder.

CpGs	Feature	Chr	Gene	SE	Beta (95% CI)	OR (95% CI)	*P*	FDR-*P*	Outcome
cg12661624	Body	7	PTPRN2	3.53E−02	0.15 (0.08–0.22)	1.16 (1.09–1.25)	1.91E−05	9.04E−03	Depression
cg24526596	5′UTR	8	DLGAP2	1.67E−02	−0.07 (−0.1 to −0.04)	0.93 (0.9–0.96)	3.87E−05	9.04E−03	Depression
cg00157656	Body	8	ERICH1	7.98E−02	−0.32 (−0.48 to −0.17)	0.72 (0.62–0.85)	4.79E−05	9.60E−03	Depression
cg12317217	Body	5	PCDHA2	5.06E−02	−0.19 (−0.29 to −0.09)	0.83 (0.75–0.92)	2.27E−04	3.97E−02	Depression
cg23997185	1stExon	5	PCDHB2	7.50E−02	0.26 (0.12–0.41)	1.3 (1.12–1.51)	4.40E−04	6.86E−02	Depression
cg09040942	Body	7	PTPRN2	1.27E−01	−0.41 (−0.66 to −0.16)	0.66 (0.52–0.85)	1.11E−03	1.40E−01	Depression
cg17200577	Body	10	PARD3	1.07E−01	−0.35 (−0.56 to −0.14)	0.71 (0.57–0.87)	1.12E−03	1.40E−01	Depression
cg16010433	TSS1500	19	HCN2	1.12E−01	−0.36 (−0.58 to −0.14)	0.7 (0.56–0.87)	1.20E−03	1.40E−01	Depression
cg00716277	Body	7	PTPRN2	5.07E−02	−0.16 (−0.26 to −0.06)	0.85 (0.77–0.94)	1.62E−03	1.41E−01	Depression
cg22517801	Body	7	PTPRN2	8.17E−02	−0.26 (−0.42 to −0.1)	0.77 (0.66–0.91)	1.63E−03	1.41E−01	Depression
cg09742643	Body	7	PTPRN2	7.84E−02	0.29 (0.14–0.44)	1.34 (1.15–1.56)	2.11E−04	5.54E−02	PTSD
cg05328339	Body	7	PTPRN2	5.49E−02	−0.2 (−0.3 to −0.09)	0.82 (0.74–0.92)	3.48E−04	5.54E−02	PTSD
cg26114452	Body	5	PCDHGA4	7.41E−02	−0.26 (−0.41 to −0.12)	0.77 (0.66–0.89)	3.56E−04	5.54E−02	PTSD
cg22420514	Body	7	PTPRN2	3.97E−02	0.12 (0.04–0.2)	1.13 (1.05–1.22)	2.04E−03	1.70E−01	PTSD
cg23375968	Body	7	PTPRN2	3.96E−02	0.12 (0.04–0.2)	1.13 (1.04–1.22)	2.18E−03	1.70E−01	PTSD
cg08395122	Body	5	PCDHGA4	2.07E−01	−0.61 (−1.02 to −0.2)	0.54 (0.36–0.82)	3.24E−03	2.05E−01	PTSD
cg18878616	Body	5	PCDHGA1	9.59E−02	0.28 (0.09–0.47)	1.32 (1.1–1.6)	3.60E−03	2.05E−01	PTSD
cg05149776	Body	5	PCDHGA4	5.79E−02	0.17 (0.05–0.28)	1.18 (1.05–1.32)	4.09E−03	2.05E−01	PTSD
cg04214710	Body	7	PTPRN2	2.84E−01	−0.76 (−1.32 to −0.2)	0.47 (0.27–0.81)	7.34E−03	3.26E−01	PTSD
cg12877853	Body	7	PTPRN2	2.37E−01	−0.63 (−1.1 to −0.17)	0.53 (0.33–0.85)	7.56E−03	3.26E−01	PTSD

Chr = chromosome, CI = confidence interval, CpGs = cytosine-phosphate-guanine sites, DNAm = DNA methylation, FDR-*P* = false discovery rate-corrected *P* value, OR = odds ratio, PTSD = post-traumatic stress disorder, SE = standard error.

**Table 2:** Sensitivity analysis and expression quantitative trait loci mendelian randomization results of the 4 putative causal cytosine-phosphate-guanine sites.

CpG	Gene	*Q*-*P*	Egger-*P*	*P*† (depression)	Beta 95% CI† (depression)	*P*† (PTSD)	Beta 95% CI† (PTSD)
cg12317217	PCDHA2*	–	–	–	–	–	–
cg12661624	PTPRN2	0.70	–	.04‡	0.02 (0.0004–0.04)	.67	−0.02 (−0.12 to 0.08)
cg24526596	DLGAP2*	0.62	0.92	–	–	–	–
cg00157656	ERICH1	–	–	.91	−0.001 (−0.03 to 0.03)	.76	0.02 (−0.12 to 0.17)

CI = confidence interval, CpG = cytosine-phosphate-guanine, Egger-*P* = Mendelian randomization-Egger intercept test *P*-value, eQTL = expression quantitative trait loci, MR = Mendelian randomization, PTSD = post-traumatic stress disorder, *Q*-*P* = Cochran *Q* test *P*-value.

* Indicates no available information from the eQTLGen consortium (phase I) for this gene.

† Represents the eQTL MR analysis results.

‡ Indicates statistical significance.

## 4. Discussion

**Figure 4.**
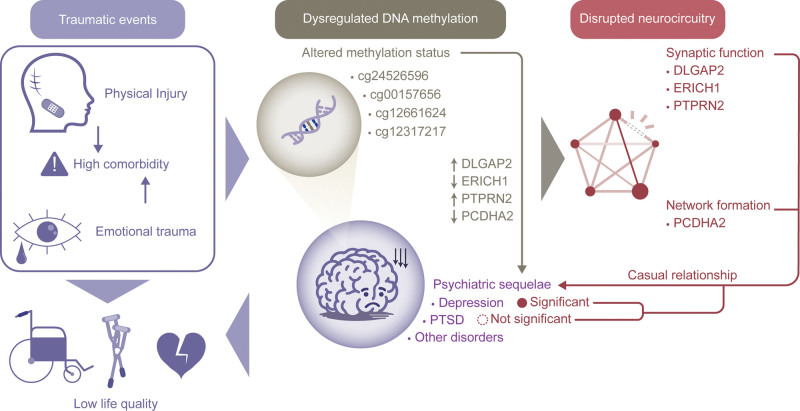
Schematic illustration of the main findings from the present study.

In this study, we investigated the DNAm patterns underlying the onset of depression or PTSD after injury and demonstrated that injury-associated DNAm alterations predispose patients to the occurrence of psychiatric sequelae after the injury. Overall, we identified a significant causal relationship between physical trauma-associated DNAm and depression at 4 distinct CpG sites mapped to PCDHA2, ERICH1, DLGAP2, and PTPRN2, respectively. However, the present study did not reveal substantial genetic evidence linking DNAm patterns to the onset of PTSD following injury. A schematic summary of the main findings of the present study is shown in Figure 4.

It may seem logically intuitive to associate the location of injury in a particular brain region with the occurrence of psychiatric sequelae. However, it is not the location of the injury, but rather the altered functional connectivity of specific brain networks that is responsible for the common phenomenon of co-occurring post-injury psychiatric disorders. This mechanism may extend beyond TBI, as even extracranial bodily injuries can precipitate psychiatric disorders through systemic maladaptation rather than localized structural damage.^[29,30]^ Interestingly, we observed a DNAm module composed mainly of cPCDHs from the co-methylation network, and DNAm at cg12317217 in the gene body of PCDHA2 was found to be protective against depression (OR: 0.83 [95% CI: 0.75–0.92]). The PCDH family is highly involved in the regulation of neural circuits and can be divided into cPCDHs and non-clustered PCDHs. While non-clustered PCDHs mainly modulate cell motility, cPCDHs can regulate neuronal survival, and both are involved in neural circuit formation.^[31]^ Notably, cPCDHs are known to be significantly influenced by epigenetic regulation and have already been reported to be associated with susceptibility to various mental disorders, including depression, autism, bipolar disorder, and schizophrenia. Furthermore, alpha-cPCDHs, including PCDHA2, are crucial for serotonergic neurocircuitry, which is even more interesting in the context of depression, given the well-known serotonin theory.^[32]^ Taken together, dysregulated DNAm of cPCDH after injury, especially the alpha cluster, may disrupt neural circuit function and could be the potential biomarkers for predicting psychiatric sequelae after injury.

Importantly, the 4 CpGs identified in our study were all mapped to genes involved in maintaining neurocircuitry integrity. ERICH1, which encodes the glutamate rich 1 protein, has previously been implicated in the regulation of the intracellular glutamate levels.^[33]^ In this study, our results suggest that cg00157656 methylation in the ERICH1 gene body, which is associated with active gene expression, has a negative causal relationship (OR: 0.72 [95% CI: 0.62–0.85]) with the development of depression. Therefore, we hypothesized that ERICH1 may help regulate the glutamate levels, thereby reducing excitotoxicity due to overactivation of *N*‑methyl‑D‑aspartate receptors (NMDARs) and alpha-amino-3-hydroxy-5-methyl-4-isoxazolepropionic acid receptors (AMPARs), and may represent a potential target for both depression and PTSD treatment.^[34,35]^ Unfortunately, few related studies have been conducted to delineate the role of ERICH1, and future research investigating the mechanisms of ERICH1 and targeting glutamate signaling may yield novel translational insights for potential treatments. In addition, revisiting the existing classical NMDR antagonists, such as ketamine, for their therapeutic potential, may also provide unexpected insights.

DLGAP2 is a postsynaptic density protein at dendrites and excitatory synapses and has been implicated in various mental disorders, especially schizophrenia.^[36]^ However, we only find significant negative causal relationship for depression at cg24526596 methylation (DLGAP2) in the 5′UTR (OR: 0.93 [95% CI: 0.90–0.96]) and the causal effect is not significant for PTSD (*P* = .24, OR: 0.93 [95% CI: 0.82–1.05]). Future clinical studies are needed to determine the influence of DLGAP2 in the vulnerability to PTSD.

PTPRN2, encoding protein tyrosine phosphatase receptor type N2, is a transmembrane protein required for normal accumulation of secretory vesicles in the hippocampus and has been implicated in neurotransmitter exocytosis.^[37]^ Our results showed that DNAm at cg12661624 (PTPRN2) in the gene body was associated with increased risk of depression (OR: 1.16 [95% CI: 1.09–1.25]), and is further supported by the eQTL MR result (OR: 1.02 [95% CI: 1.00–1.04]). Meanwhile, a recent epigenome-wide association study (EWAS) also reported that PTPRN2 is a potential biomarker for depression.^[38]^ These combined findings generate the hypothesis that upregulation of PTPRN2 due to injury-associated methylation at cg12661624 may disrupt synaptic transmission and thereby potentially contribute to the development of depression. Still, further research is warranted to elucidate the role of PTPRN2 in depression and PTSD.

DNAm alterations have been implicated in PTSD with regard to immune regulation, stress response, and neural development and plasticity.^[39,40]^ Unfortunately, the results of PTSD-related EWAS studies to date have been inconsistent^[41,42]^ and our study did not find a robust causal relationship between the injury-associated DNAm loci and PTSD. Importantly, this null finding should be interpreted with caution, as the statistical power of the MR analysis for PTSD was substantially lower than that for depression, largely due to the smaller number of cases available in the PTSD GWAS summary statistics. PTSD remains a particularly relevant psychiatric outcome following physical injury, especially TBI, because of the overlapping symptomatology and pathophysiology that inevitably involves DNAm alterations.^[8,43]^ Therefore, current causal evidence of DNAm and PTSD is still lacking, and it’s still an open issue to determine their interactions and requires further investigations.

To our knowledge, the present study is the first to investigate the causal relationship between injury-associated DNAm and the onset of depression or PTSD. The altered methylation levels at CpGs associated with ERICH1, PCDHA2, DLGAP2, and PTPRN2 may indicate disrupted neural circuitry, leading to psychiatric sequelae. Despite the insufficient robust causal evidence, the potential role of DNAm after injury in leading to PTSD should be recognized. Interestingly, a previous MR study examining the association between trauma exposure (child or adult trauma and PTSD-related trauma) and depression and suggested that the association between trauma and depression was not significant and remained unclear.^[44]^ Given that the common occurrence of PTSD and depression, future research and clinical management of patients should not overlook their potential interactions. Another previous MR study supported the causal effect of DNAm on depression, and is associated with immune responses and neural development.^[45]^ Unfortunately, there is no existing MR study investigating DNAm and PTSD.

Taken together, the onset of PTSD and depression after injury may differ, thereby highlighting the importance of early identification and classification of psychiatric disorders risk after injury. We acknowledge that several limitations of the present study should be considered. The WGBS data we used were from a single study with a relatively small sample size, which may affect the stability of the constructed co-methylation networks and limit the generalizability of the findings. We are optimistic that future larger multicenter studies should hopefully reduce this bias. Furthermore, the MR analysis for PTSD was likely underpowered due to the limited number of cases in the GWAS summary statistics. Finally, there is no GWAS study has been conducted specifically for certain post-injury psychiatric disorders, nor are there any QTL datasets tailored for this subpopulation of patients. Therefore, the validity of our results may be affected, and more targeted GWAS studies are warranted to reduce the influence of confounding factors.

## 5. Conclusion

This study investigated the causal DNAm underlying physical injury for the onset of depression or PTSD. Our results demonstrated that 4 unique CpGs mapped to PCDHA2, ERICH1, DLGAP2, and PTPRN2 were causative for depression. However, there was no robust evidence for a causal relationship between injury-associated DNAm changes and PTSD. Our results support the notion that the damaged functional neurocircuitry is crucial for the development of psychiatric disorders following injury. The methylation levels of the identified genes may serve as potential biomarkers for early identification and surveillance of injury patients, and further validation in larger independent cohorts are indispensable.

## Acknowledgments

We want to acknowledge all investigators and participants involved in the Martin CA et al study, the GoDMC database, the FinnGen study, and the eQTLGen consortium for sharing publicly available data.

## Author contributions

**Conceptualization:** Lian Liu, Ziyu Zhu.

**Data curation:** Lian Liu, Mengran Du, Xin Liu.

**Formal analysis:** Ziyu Zhu, Qiuhao Tan, Longwei Xiao, Xiaoyang Zheng.

**Funding acquisition:** Zhengbu Liao.

**Investigation:** Mengran Du, Xin Liu.

**Methodology:** Ziyu Zhu.

**Project administration:** Zhengbu Liao.

**Software:** Zhijian Huang,

**Supervision:** Zhengbu Liao.

**Validation:** Longwei Xiao, Zhijian Huang.

**Visualization:** Qiuhao Tan, Xiaoyang Zheng.

**Writing – original draft:** Lian Liu, Ziyu Zhu.

**Writing – review & editing:** Zhengbu Liao.

## Supplementary Material




