# Supplementary material for: Causal evidence linking injury-associated DNA methylation to the risk of developing depression or post-traumatic stress disorder
Source: Medicine (Baltimore). 2026 Apr 17;105(16):e48310. doi: 10.1097/MD.0000000000048310 (PMC13095310; doi:10.1097/MD.0000000000048310)
Supplement: Supplementary file 2 [file medi-105-e48310-s002.pdf]

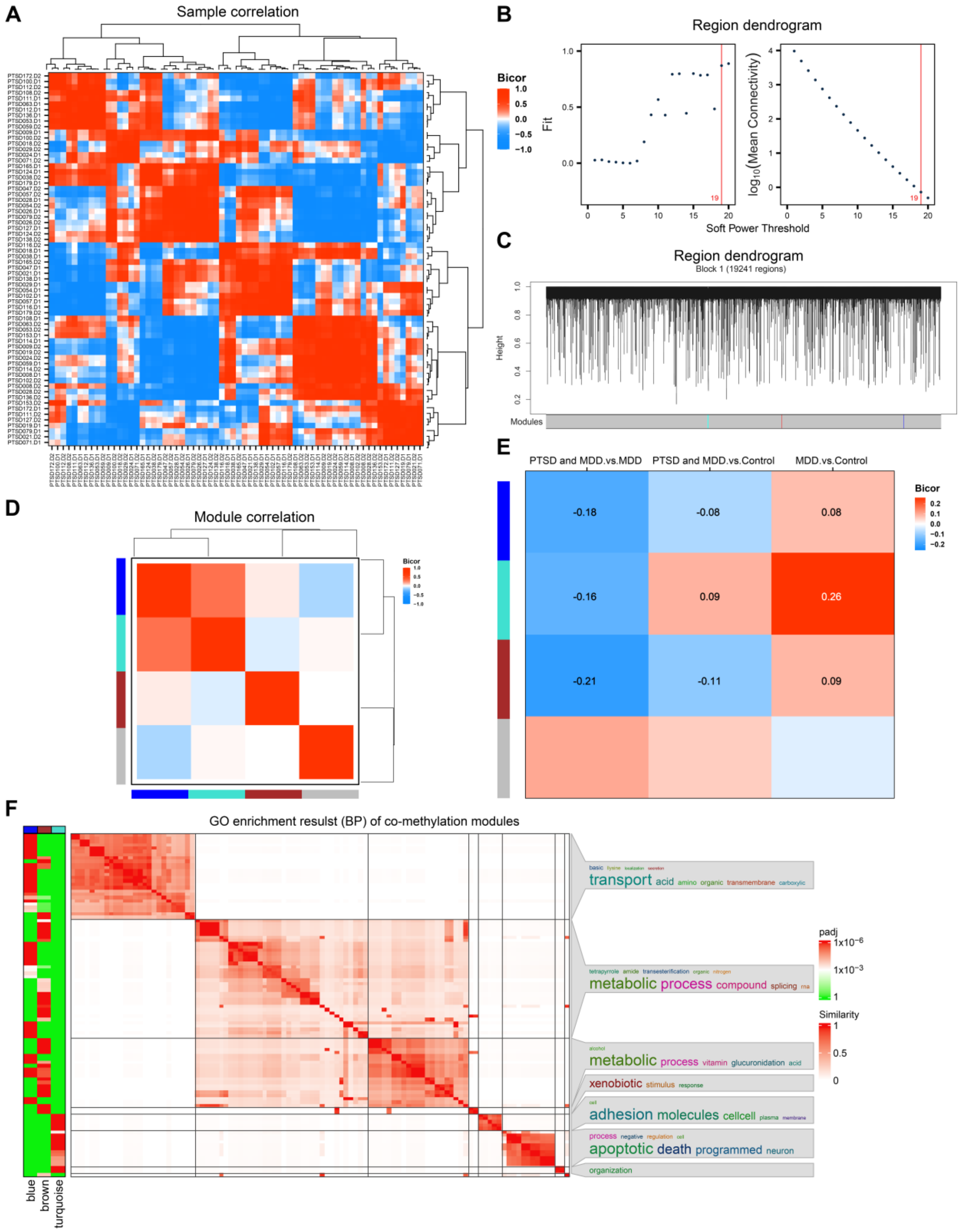

A
Sample correlation
B
Region dendrogram
Fit
log10(Mean Connectivity)
Soft Power Threshold
19
Bicor
C
Region dendrogram
Block 1 (19241 regions)
Height
Modules
D
Module correlation
E
PTSD and MDD.vs.MDD
PTSD and MDD.vs.Control
MDD.vs.Control
-0.18
-0.08
0.08
-0.16
0.09
0.26
-0.21
-0.11
0.09
F
GO enrichment resulst (BP) of co-methylation modules
transport acid amino organic transmembrane carboxylic
metabolic process compound splicing
metabolic process vitamin glucuronidation acid
xenobiotic stimulus response
adhesion molecules cellcell plasma membrane
process negative regulation cell
apoptotic death programmed neuron
organization
padj
Similarity
blue
brown
turquoise

Figure S1 Co-methylation network construction and module characterization.

Sample clustering analysis showed clear correlation patterns among patients (A). The optimal soft-thresholding power was determined as 19 based on Pearson correlation coefficients to achieve scale-free topology (B). Using this threshold, three co-methylation modules were identified from the full network of 19,241 genomic regions, including the blue (n = 17), turquoise (n = 35), and brown (n = 14) modules (C). Module eigengene correlation analysis using bicor correlation coefficients revealed no significant inter-module associations (D). Sample–module correlation analysis demonstrated that none of the modules reached statistical significance, although the turquoise module showed the strongest correlation with depression diagnosis (E). Gene Ontology biological process enrichment and semantic simplification results for each module are shown in (F).

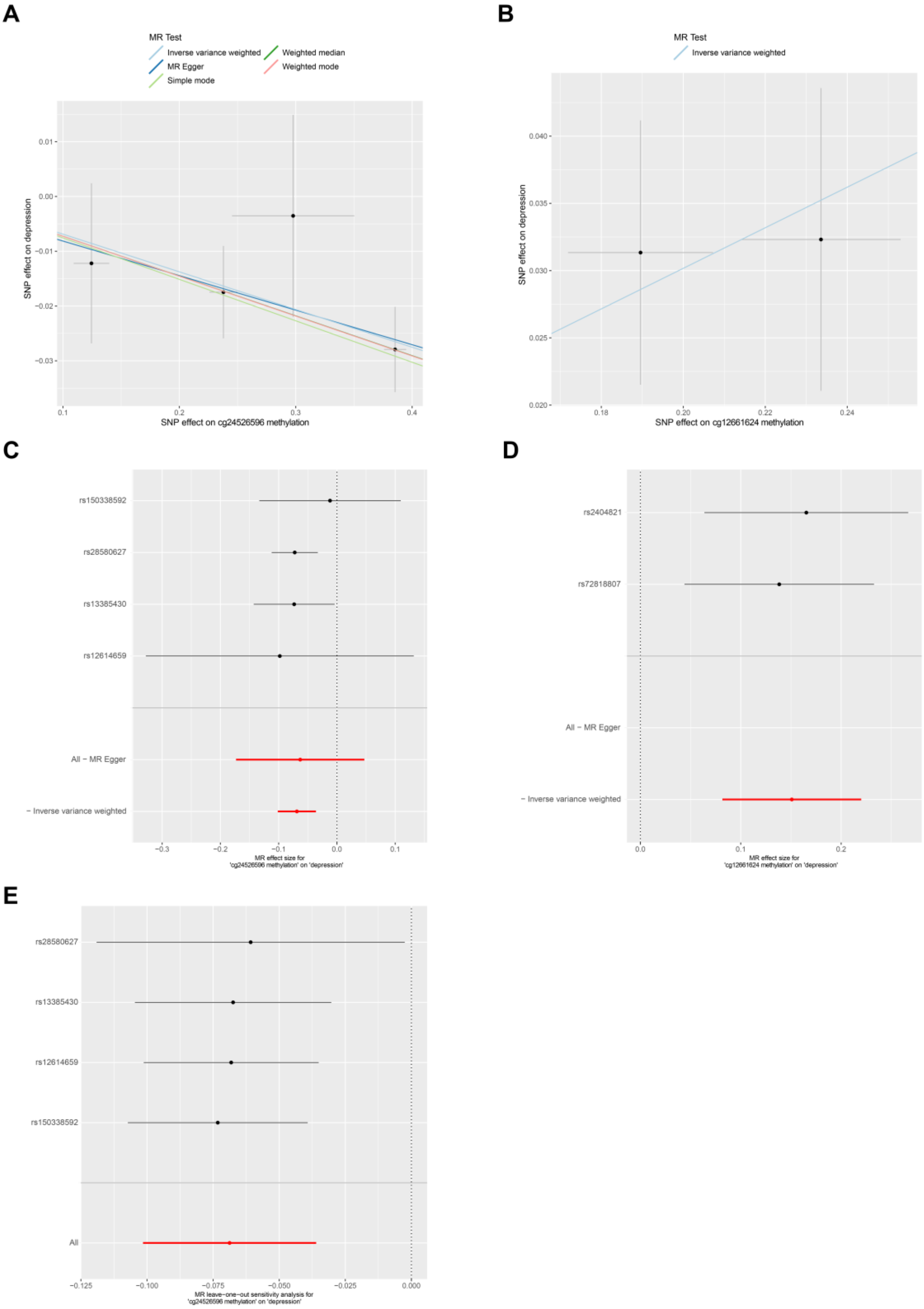

A
MR Test
Inverse variance weighted
MR Egger
Simple mode
Weighted median
Weighted mode
SNP effect on depression
SNP effect on cg24526596 methylation
B
MR Test
Inverse variance weighted
SNP effect on depression
SNP effect on cg12661624 methylation
C
rs150338592
rs28580627
rs13385430
rs12614659
All − MR Egger
− Inverse variance weighted
MR effect size for 'cg24526596 methylation' on 'depression'
D
rs2404821
rs72818807
All − MR Egger
− Inverse variance weighted
MR effect size for 'cg12661624 methylation' on 'depression'
E
rs28580627
rs13385430
rs12614659
rs150338592
All
MR leave−one−out sensitivity analysis for 'cg24526596 methylation' on 'depression'

Figure S2 Sensitivity analyses of Mendelian randomization (MR) results.

MR sensitivity analyses for cg24526596 (DLGAP2) and cg12661624 (PTPRN2) demonstrated robust causal estimates across multiple methods, supporting the stability of the primary MR findings.

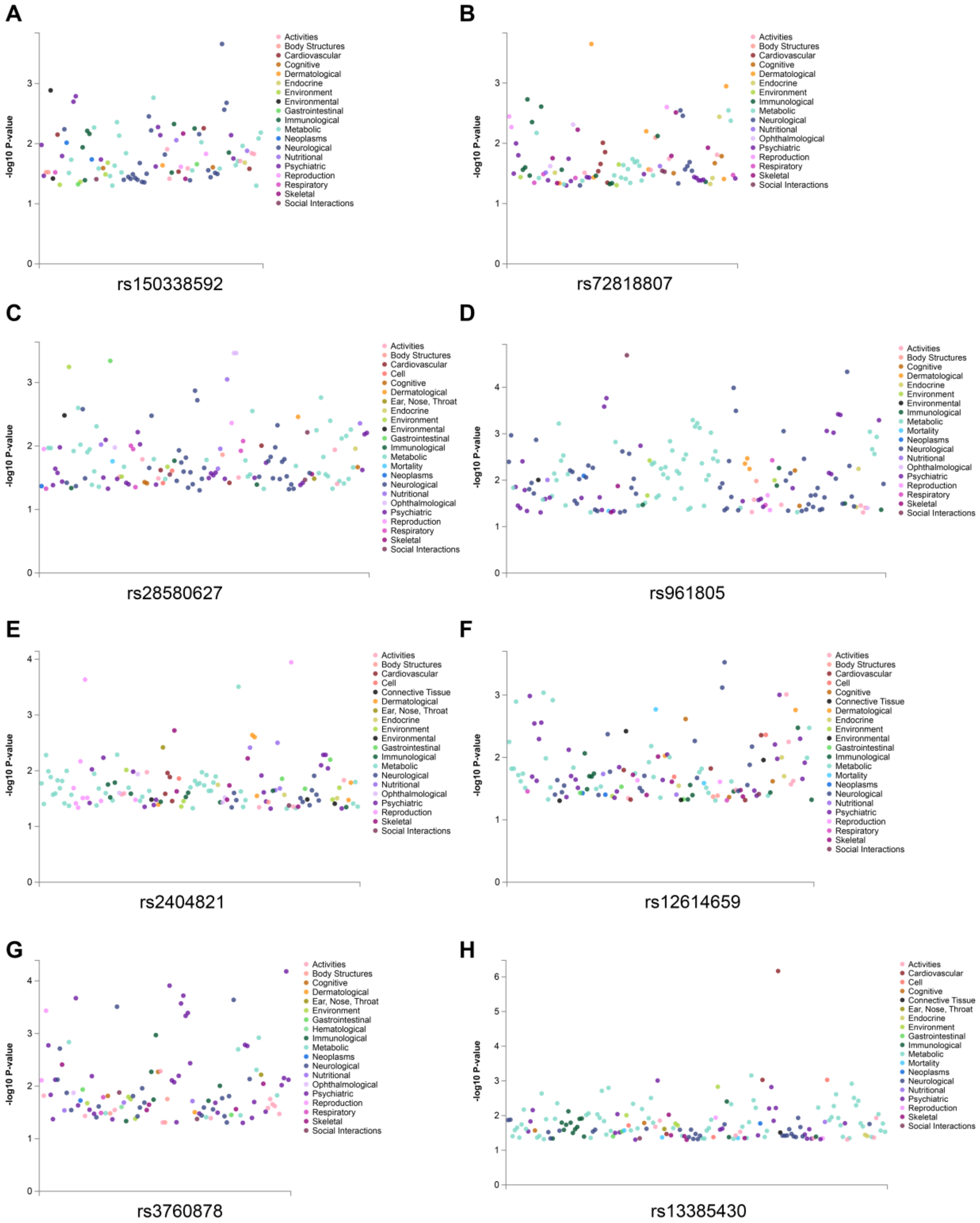
A
-log10 P-value
rs150338592
Activities
Body Structures
Cardiovascular
Cognitive
Dermatological
Endocrine
Environment
Environmental
Gastrointestinal
Immunological
Metabolic
Neoplasms
Neurological
Nutritional
Psychiatric
Reproduction
Respiratory
Skeletal
Social Interactions
B
-log10 P-value
rs72818807
Activities
Body Structures
Cardiovascular
Cognitive
Dermatological
Endocrine
Environment
Immunological
Metabolic
Neurological
Nutritional
Ophthalmological
Psychiatric
Reproduction
Respiratory
Skeletal
Social Interactions
C
-log10 P-value
rs28580627
Activities
Body Structures
Cardiovascular
Cell
Cognitive
Dermatological
Ear, Nose, Throat
Endocrine
Environment
Environmental
Gastrointestinal
Immunological
Metabolic
Mortality
Neoplasms
Neurological
Nutritional
Ophthalmological
Psychiatric
Reproduction
Respiratory
Skeletal
Social Interactions
D
-log10 P-value
rs961805
Activities
Body Structures
Cognitive
Dermatological
Endocrine
Environment
Environmental
Immunological
Metabolic
Mortality
Neoplasms
Neurological
Nutritional
Ophthalmological
Psychiatric
Reproduction
Respiratory
Skeletal
Social Interactions
E
-log10 P-value
rs2404821
Activities
Body Structures
Cardiovascular
Cell
Connective Tissue
Dermatological
Ear, Nose, Throat
Endocrine
Environment
Environmental
Gastrointestinal
Immunological
Metabolic
Neurological
Nutritional
Ophthalmological
Psychiatric
Reproduction
Skeletal
Social Interactions
F
-log10 P-value
rs12614659
Activities
Body Structures
Cardiovascular
Cell
Cognitive
Connective Tissue
Dermatological
Endocrine
Environment
Environmental
Gastrointestinal
Immunological
Metabolic
Mortality
Neoplasms
Neurological
Nutritional
Psychiatric
Reproduction
Respiratory
Skeletal
Social Interactions
G
-log10 P-value
rs3760878
Activities
Body Structures
Cognitive
Dermatological
Ear, Nose, Throat
Environment
Gastrointestinal
Hematological
Immunological
Metabolic
Neoplasms
Neurological
Nutritional
Ophthalmological
Psychiatric
Reproduction
Respiratory
Skeletal
Social Interactions
H
-log10 P-value
rs13385430
Activities
Cardiovascular
Cell
Cognitive
Connective Tissue
Ear, Nose, Throat
Endocrine
Environment
Gastrointestinal
Immunological
Metabolic
Mortality
Neoplasms
Neurological
Nutritional
Psychiatric
Reproduction
Skeletal
Social Interactions

Figure S3 Phenome-wide association scan of instrumental SNPs.

Phenome-wide association analyses were performed for all instrumental SNPs to assess potential associations with known confounders. No significant associations were detected, supporting the validity of the instrumental variables.

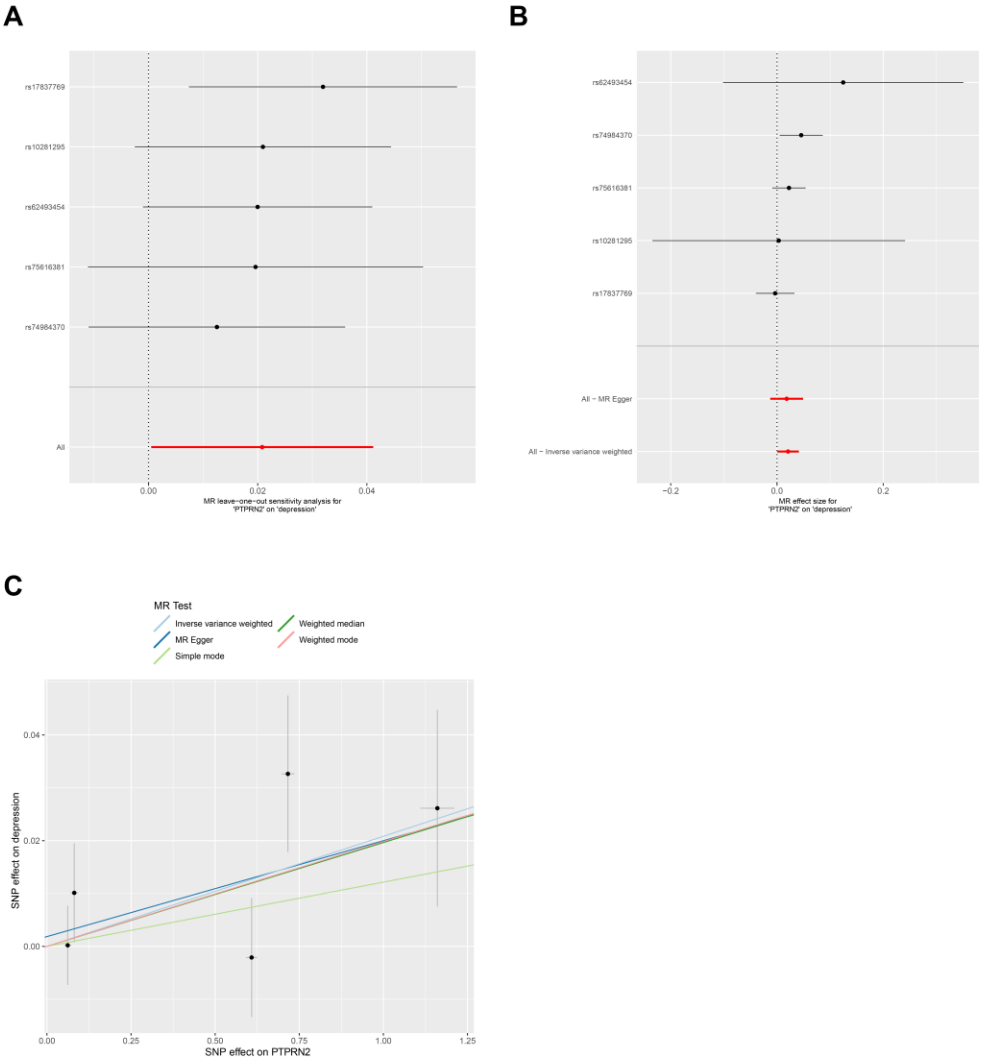
A
rs17837769
rs10281295
rs62493454
rs75616381
rs74984370
All
0.00
0.02
0.04
MR leave−one−out sensitivity analysis for
'PTPRN2' on 'depression'
B
rs62493454
rs74984370
rs75616381
rs10281295
rs17837769
All − MR Egger
All − Inverse variance weighted
−0.2
0.0
0.2
MR effect size for
'PTPRN2' on 'depression'
C
MR Test
Inverse variance weighted
MR Egger
Simple mode
Weighted median
Weighted mode
0.04
0.02
0.00
SNP effect on depression
0.00
0.25
0.50
0.75
1.00
1.25
SNP effect on PTPRN2

Figure S4 eQTL-based MR validation analyses.

Complementary MR analyses using expression quantitative trait loci (eQTLs) indicated a significant causal association between PTPRN2 expression and depression. No significant associations were observed for ERICH1, and eQTL data were unavailable for DLGAP2 and PCDHA2.
