# Supplementary material for: Causal evidence linking injury-associated DNA methylation to the risk of developing depression or post-traumatic stress disorder
Source: Medicine (Baltimore). 2026 Apr 17;105(16):e48310. doi: 10.1097/MD.0000000000048310 (PMC13095310; doi:10.1097/MD.0000000000048310)
Supplement: Supplementary file 1 [file medi-105-e48310-s001.pdf]

RegionID	chr	start	end	width	n	covMin	covMean	covSD	methMean	methSD	strand	gene_id	module	membership	hubRegion	SYMBOL
Region_1	chr1	89295	268816	179521	7	19	49.09090909	18.4396957	0.955160668	0.03912951	-	100996442	grey	0.070232961	FALSE	WASH9P
Region_2	chr1	586071	827796	241725	33	221	277.7272727	36.50734192	0.794134308	0.018303873	-	105378947	grey	-0.144636126	FALSE	LOC105378947
Region_3	chr1	586945	720194	133249	5	18	42.1969697	22.07136655	0.682508709	0.072931802	-	81399	grey	-0.133830794	FALSE	OR4F16
Region_4	chr1	711867	778663	66796	14	88	118.6969697	13.11132182	0.738067824	0.034984552	-	100288069	grey	-0.280891753	FALSE	LOC100288069
Region_5	chr1	778747	810065	31318	14	88	115.8484848	18.71835169	0.882824527	0.026905094	+	105378580	grey	0.174490214	FALSE	LINC01409
Region_6	chr1	825138	859446	34308	31	204	255.1060606	19.46604838	0.868431664	0.020889001	+	643837	grey	0.203758892	FALSE	LINC01128
Region_7	chr1	860226	868202	7976	9	39	59.21212121	8.690428247	0.888432085	0.060674747	+	107984850	grey	0.163742789	FALSE	LOC107984850
Region_8	chr1	868071	877234	9163	20	112	149.4848485	16.07888479	0.445845909	0.031890659	-	284593	grey	0.342561405	FALSE	FAM41C
Region_9	chr1	911435	914948	3513	16	87	128.7121212	16.78072626	0.602057787	0.031745679	+	284600	grey	-0.188621365	FALSE	LOC284600
Region_10	chr1	916870	919692	2822	13	70	90.1969697	12.9593681	0.819540843	0.041456899	-	100130417	grey	-0.016928215	FALSE	LINC02593
Region_11	chr1	923923	944575	20652	74	409	502.9848485	35.518356	0.431707914	0.04035833	+	148398	grey	-0.461491067	FALSE	SAMD11
Region_12	chr1	944203	959309	15106	32	197	234.151515	18.1084276	0.80286579	0.02050597	-	26155	grey	-0.00249023	FALSE	NOC2L

							2	4	9	8				7		
Region_13	chr1	960584	965719	5135	17	61	92.34848485	13.48445535	0.572181591	0.025281646	+	339451	grey	0.0198028	FALSE	KLHL17
Region_14	chr1	966482	975865	9383	26	169	207.1060606	17.78601766	0.787745226	0.026233089	+	84069	grey	-0.048062566	FALSE	PLEKHN1
Region_15	chr1	975198	982117	6919	34	181	228.6969697	22.61379409	0.687798814	0.035652331	-	84808	grey	-0.205298892	FALSE	PERM1
Region_16	chr1	998962	1000172	1210	14	60	94.16666667	17.26676072	0.006639292	0.010656378	-	57801	grey	0.034393309	FALSE	HES4
Region_17	chr1	1001138	1014540	13402	38	207	270.2272727	21.95085101	0.599672109	0.029616363	+	9636	grey	0.063463398	FALSE	ISG15
Region_18	chr1	1020120	1056118	35998	94	487	591.3939394	43.04304779	0.762023744	0.016263265	+	375790	grey	-0.064253151	FALSE	AGRN
Region_19	chr1	1059687	1069355	9668	30	173	207.8333333	17.21053643	0.487713624	0.033480626	+	100288175	grey	-0.019552793	FALSE	LOC100288175
Region_20	chr1	1070967	1074306	3339	14	64	84.28787879	10.39047663	0.888747426	0.045413355	-	401934	grey	-0.136825216	FALSE	RNF223
Region_21	chr1	1081818	1116361	34543	81	532	622.1060606	39.95810452	0.820673922	0.013105681	-	54991	grey	0.131379302	FALSE	C1orf159
Region_22	chr1	1137017	1144056	7039	14	52	73.25757576	11.03325129	0.729022649	0.036181062	+	254099	grey	-0.297818164	FALSE	LINC01342
Region_23	chr1	1173056	1179555	6499	24	132	182.030303	16.68345076	0.676320477	0.046854254	-	100506376	grey	0.061116843	FALSE	TTLL10-AS1
Region_24	chr1	1173880	1197936	24056	59	342	412.9848485	29.98024602	0.767579668	0.022843097	+	254173	grey	0.076756889	FALSE	TTLL10

Region_25	chr1	1203508	1206592	3084	7	26	40.27272727	6.001398438	0.572019854	0.057752207	-	8784	grey	-0.24782711	FALSE	TNFRSF18
Region_26	chr1	1211326	1214153	2827	5	18	36.18181818	6.679265485	0.296052378	0.060298576	-	7293	grey	0.082701662	FALSE	TNFRSF4
Region_27	chr1	1216931	1232031	15100	87	548	674.0606061	49.97164697	0.844749175	0.011784314	-	51150	grey	0.061343946	FALSE	SDF4
Region_28	chr1	1232237	1235041	2804	26	138	183.4848485	24.91724298	0.615723972	0.029940454	+	126792	grey	0.070179842	FALSE	B3GALT6
Region_29	chr1	1242446	1246722	4276	26	107	151.7575758	17.36934577	0.805670947	0.027565634	-	388581	grey	-0.021456702	FALSE	C1QTNF12
Region_30	chr1	1253909	1273864	19955	59	405	478.9393939	31.46616194	0.645714921	0.014347809	-	118424	grey	0.335214298	FALSE	UBE2J2
Region_31	chr1	1275223	1280420	5197	15	73	105.0454545	9.568267777	0.927354296	0.030120767	+	101928895	grey	0.013411177	FALSE	LINC01786
Region_32	chr1	1280436	1292029	11593	51	264	329.8939394	24.90605659	0.884674335	0.02179591	+	6339	grey	-0.136162389	FALSE	SCNN1D
Region_33	chr1	1292390	1309609	17219	40	176	232.8939394	23.17235009	0.496410959	0.021742281	-	116983	grey	-0.14246491	FALSE	ACAP3
Region_34	chr1	1308597	1311677	3080	25	101	151.8181818	15.95465603	0.459406389	0.028409323	+	126789	grey	0.0080309	FALSE	PUSL1
Region_35	chr1	1311585	1324687	13102	22	115	145.2878788	14.59058195	0.720306684	0.023294686	-	54973	grey	-0.040196681	FALSE	INTS11
Region_36	chr1	1324756	1328896	4140	5	18	31.22727273	6.345914384	0.420905395	0.058560382	+	80772	grey	0.196582113	FALSE	CPTP
Region_37	chr1	1331280	1335314	4034	29	173	205.545454	19.8580979	0.90874179	0.02344716	+	83756	grey	0.100144214	FALSE	TAS1R3

							5	9	4	7						
Region_38	chr1	1335276	1349418	14142	30	145	184.0757576	17.89225067	0.858566288	0.02160294	-	1855	grey	0.155519166	FALSE	DVL1
Region_39	chr1	1352689	1361777	9088	28	143	179.1363636	22.39566329	0.269162822	0.048989698	-	54587	grey	0.175407614	FALSE	MXRA8
Region_40	chr1	1373730	1375207	1477	22	84	130.8030303	21.70162681	0.098517386	0.017690664	-	54998	grey	0.27410088	FALSE	AURKAIP1
Region_41	chr1	1385711	1399335	13624	30	218	253.5	17.98995446	0.910089092	0.012999816	-	81669	grey	0.059949178	FALSE	CCNL2
Region_42	chr1	1399515	1402046	2531	8	38	56.10606061	7.799662296	0.373282773	0.022953528	+	148413	grey	0.035164417	FALSE	MRPL20-AS1
Region_43	chr1	1401909	1407293	5384	21	157	204.3030303	24.369571	0.227847312	0.013078593	-	55052	grey	0.200383011	FALSE	MRPL20
Region_44	chr1	1418420	1421769	3349	19	86	116.2727273	15.19670557	0.61619012	0.044175976	-	441869	grey	0.044653034	FALSE	ANKRD65
Region_45	chr1	1420245	1422691	2446	8	15	36.86363636	9.164960638	0.335197454	0.094746155	+	105378585	grey	0.036799214	FALSE	ANKRD65-AS1
Region_46	chr1	1430539	1434573	4034	5	23	33.72727273	5.345150079	0.436114785	0.055663266	-	102724312	grey	0.089749892	FALSE	LINC01770
Region_47	chr1	1434861	1442882	8021	17	73	102.2121212	12.39790944	0.698088443	0.05806547	+	64856	grey	-0.057306705	FALSE	VWA1
Region_48	chr1	1449689	1470163	20474	40	139	271.3181818	32.20566557	0.86547957	0.026056619	+	219293	grey	-0.164440611	FALSE	ATAD3C
Region_49	chr1	1471765	1509466	37701	67	300	463.7727273	43.52710754	0.850267122	0.011836681	+	83858	grey	-0.017522511	FALSE	ATAD3B

Region_50	chr1	1512162	1534685	22523	41	244	286.1666667	21.26650189	0.875659571	0.019546403	+	55210	grey	-0.001022611	FALSE	ATAD3A
Region_51	chr1	1534778	1540624	5846	29	119	163.6969697	20.97542129	0.907574838	0.028839501	-	339453	grey	0.033640173	FALSE	TMEM240
Region_52	chr1	1541673	1574863	33190	91	623	730.3636364	39.72003423	0.895564156	0.010419728	-	29101	grey	0.124852659	FALSE	SSU72
Region_53	chr1	1598012	1600135	2123	12	52	76.93939394	9.827557468	0.43201777	0.030472142	-	643988	grey	-0.278111046	FALSE	FNDC10
Region_54	chr1	1615454	1630605	15151	63	340	400.7121212	31.81763669	0.617049152	0.016307944	+	142678	grey	0.057294615	FALSE	MIB2
Region_55	chr1	1635225	1659012	23787	44	255	314.1515152	28.48278423	0.883646252	0.013829829	-	984	grey	-0.142442833	FALSE	CDK11B
Region_56	chr1	1659529	1692795	33266	81	486	584.4090909	54.74583996	0.793008168	0.020137332	-	728661	grey	0.080754671	FALSE	SLC35E2B
Region_57	chr1	1702379	1737688	35309	30	100	218.2424242	48.8202545	0.742587447	0.025357153	-	728642	grey	0.282313112	FALSE	CDK11A
Region_58	chr1	1724838	1745992	21154	14	48	108.1212121	21.42429617	0.920636076	0.042070473	-	9906	grey	0.223748891	FALSE	SLC35E2A
Region_59	chr1	1751232	1780457	29225	27	158	189.2727273	12.41406827	0.79524283	0.019277132	-	65220	grey	-0.240311316	FALSE	NADK
Region_60	chr1	1785285	1891117	105832	103	704	810.6666667	41.37458653	0.83415573	0.011650005	-	2782	grey	-0.070255954	FALSE	GNB1
Region_61	chr1	1915108	1917296	2188	6	28	41.71212121	7.991851911	0.946280688	0.03481401	+	163688	grey	-0.144115552	FALSE	CALML6
Region_62	chr1	1921951	2003837	81886	173	1098	1290.18181	78.9344244	0.86724862	0.01690186	-	85452	grey	0.124752475	FALSE	CFAP74

							8	1	6	6						
Region_63	chr1	2013213	2015639	2426	4	28	39.6363636 4	5.93201577 8	0.83347942 6	0.07344568 1	-	10537858 9	grey	0.097470329	FALSE	LOC105378589
Region_64	chr1	2019329	2030758	11429	30	160	190.136363 6	16.7533109 1	0.68561992	0.03514783 1	+	2563	grey	0.136204472	FALSE	GABRD
Region_65	chr1	2050411	2185395	134984	263	1735	1939.78787 9	84.1422815 4	0.85991217 8	0.00905153 7	+	5590	grey	0.067392597	FALSE	PRKCZ
Region_66	chr1	2181794	2184389	2595	8	42	65.4242424 2	9.01974007 1	0.96307415 2	0.02270094 8	-	10050650 4	grey	-0.09979771 7	FALSE	PRKCZ-AS1
Region_67	chr1	2184461	2212720	28259	49	295	342.742424 2	23.8432771 4	0.78711696 2	0.02531067	-	199990	grey	0.020391074	FALSE	FAAP20
Region_68	chr1	2228319	2310213	81894	167	1167	1287.74242 4	58.3227915 9	0.72416249 6	0.01172454 4	+	6497	grey	-0.37376491 1	FALSE	SKI
Region_69	chr1	2321253	2391707	70454	150	889	1001.48484 8	45.2498168	0.84971212 5	0.01856108 3	-	79906	grey	-0.29003225 2	FALSE	MORN1
Region_70	chr1	2350414	2352820	2406	4	11	25.5151515 2	5.46102955 7	0.93268485 5	0.05378899 1	-	10012953 4	grey	0.024150044	FALSE	LOC100129534
Region_71	chr1	2391775	2405442	13667	34	226	273.121212 1	21.5980876 3	0.79982144 2	0.01943978 3	+	11079	grey	0.081919248	FALSE	RER1
Region_72	chr1	2403964	2413797	9833	20	103	139.333333 3	15.8049002 5	0.76603320 4	0.01356095 7	-	5192	grey	0.116724843	FALSE	PEX10
Region_73	chr1	2768091	2784733	16642	13	70	92.5909090 9	11.8736648 1	0.73797067 2	0.04227761 6	+	10537859 8	grey	-0.18576407 8	FALSE	LOC105378598
Region_74	chr1	3059611	3069088	9477	23	121	155.757575 8	15.2659103 4	0.64903418 1	0.03315508 9	-	440556	grey	0.117330307	FALSE	PRDM16-DT

Region_75	chr1	3069168	3438621	369453	640	4272	4744.909091	196.2087371	0.794249332	0.009199187	+	63976	grey	0.236258491	FALSE	PRDM16
Region_76	chr1	3313052	3323149	10097	16	96	126.4848485	13.97717086	0.664991996	0.043618974	-	105378604	grey	0.267222627	FALSE	LOC105378604
Region_77	chr1	3454665	3481113	26448	42	241	292.0909091	21.8595005	0.811415885	0.020772	+	27237	grey	0.087754856	FALSE	ARHGEF16
Region_78	chr1	3487951	3624786	136835	271	1703	1874.954545	82.59007797	0.79695311	0.008909249	-	1953	grey	-0.097706175	FALSE	MEGF6
Region_79	chr1	3625015	3630127	5112	11	66	83.59090909	9.166791692	0.593170144	0.023311276	+	127262	grey	-0.116242583	FALSE	TPRG1L
Region_80	chr1	3630767	3652761	21994	41	248	294.6969697	23.32444719	0.677623068	0.013858476	-	49856	grey	-0.24798066	FALSE	WRAP73
Region_81	chr1	3652516	3736201	83685	99	637	715.1818182	37.16777309	0.807408704	0.016413809	+	7161	grey	-0.272416497	FALSE	TP73
Region_82	chr1	3658938	3668772	9834	8	46	62	7.07324319	0.86626004	0.049056351	-	105378610	grey	0.000770137	FALSE	TP73-AS3
Region_83	chr1	3735511	3747373	11862	23	168	205.6666667	16.32396382	0.712745967	0.018730599	-	57212	grey	-0.103362755	FALSE	GFOD3P
Region_84	chr1	3746460	3771645	25185	23	148	180.1818182	13.82184346	0.752401554	0.021871814	+	148870	grey	0.125633577	FALSE	CCDC27
Region_85	chr1	3772749	3775982	3233	11	49	68.72727273	8.583607731	0.546589565	0.053571959	+	388588	grey	0.161181543	FALSE	SMIM1
Region_86	chr1	3778559	3796498	17939	30	187	235.4242424	19.63753126	0.836354445	0.019016529	-	57470	grey	-0.034197534	FALSE	LRRC47
Region_87	chr1	3812086	3857396	45310	35	245	306.954545	21.4192090	0.87996531	0.03566721	-	9731	grey	-0.12708344	FALSE	CEP104

							5	8		6				2		
Region_88	chr1	3976133	4012704	36571	15	82	112.5454545	13.39940506	0.849557525	0.035612503	+	105376673	grey	0.031684035	FALSE	LINC02780
Region_89	chr1	4412027	4424689	12662	9	46	72.59090909	8.561051827	0.835888397	0.053743563	+	284661	grey	0.069141733	FALSE	LINC01777
Region_90	chr1	4571481	4594016	22535	11	77	100.8636364	9.645862188	0.874720474	0.035365608	+	105376679	grey	0.053327966	FALSE	LINC01646
Region_91	chr1	4654609	4792534	137925	115	816	929.4242424	41.30824489	0.698126508	0.017632758	+	55966	grey	0.012749583	FALSE	AJAP1
Region_92	chr1	4973381	4981568	8187	4	20	34.18181818	6.695369447	0.775324322	0.093619843	+	105376680	grey	-0.274816904	FALSE	LINC02781
Region_93	chr1	5478736	5493057	14321	15	91	122.8939394	13.09037084	0.743207119	0.039109851	-	105376686	grey	0.061850294	FALSE	LOC105376686
Region_94	chr1	5862811	5992473	129662	103	762	860.3484848	43.03576059	0.916698579	0.011843518	-	261734	grey	-0.162430573	FALSE	NPHP4
Region_95	chr1	5990927	6101193	110266	98	671	772.1666667	36.29646447	0.789264412	0.013798079	+	8514	grey	0.135041663	FALSE	KCNAB2
Region_96	chr1	6101787	6180321	78534	72	455	503.9848485	23.253448	0.765193804	0.014790523	-	26038	grey	0.092874527	FALSE	CHD5
Region_97	chr1	6159430	6209244	49814	54	328	374.2272727	22.66735739	0.419457322	0.014595776	-	6146	grey	0.146985851	FALSE	RPL22
Region_98	chr1	6192930	6209389	16459	22	136	166.3939394	14.16157182	0.176871136	0.018138956	-	107985733	grey	-0.024162976	FALSE	
Region_99	chr1	6205475	6221299	15824	26	155	195.5151515	17.60265926	0.493024702	0.017721153	+	388591	grey	-0.033420575	FALSE	RNF207

Region_100	chr1	6221193	6235972	14779	12	80	105.5757576	11.12178819	0.873170552	0.02029927	-	23463	grey	-0.047551862	FALSE	ICMT
Region_101	chr1	6247353	6261098	13745	16	91	118.0151515	11.3007725	0.634493441	0.043642353	-	387509	grey	0.159957643	FALSE	GPR153
Region_102	chr1	6264269	6393767	129498	188	1335	1464.181818	58.38622311	0.896226303	0.009503941	-	11332	grey	0.047576286	FALSE	ACOT7
Region_103	chr1	6412418	6424670	12252	17	103	121.8484848	10.50749538	0.446238352	0.030971151	-	54626	grey	-0.01057278	FALSE	HES2
Region_104	chr1	6424776	6461367	36591	55	316	369.0151515	28.16079786	0.409014677	0.025508489	+	83715	grey	0.162125648	FALSE	ESPN
Region_105	chr1	6460786	6466175	5389	6	16	31.25757576	5.93376416	0.83867257	0.079840334	-	8718	grey	0.002784818	FALSE	TNFRSF25
Region_106	chr1	6467122	6520074	52952	76	473	532.9393939	29.1952993	0.802878131	0.017151039	-	57449	grey	-0.214488661	FALSE	PLEKHG5
Region_107	chr1	6521347	6554513	33166	43	279	317.6363636	20.62831241	0.770426698	0.015218767	-	79707	grey	-0.133244214	FALSE	NOL9
Region_108	chr1	6555307	6579755	24448	28	169	203.9242424	16.25499744	0.920400677	0.019698527	+	80835	grey	-0.131895749	FALSE	TAS1R1
Region_109	chr1	6579994	6589280	9286	7	45	60.90909091	6.54388093	0.664630896	0.036460958	+	3104	grey	-0.104957385	FALSE	ZBTB48
Region_110	chr1	6590724	6614607	23883	28	196	236.4393939	17.36852041	0.307038316	0.012789904	-	9903	grey	-0.014994907	FALSE	KLHL21
Region_111	chr1	6613731	6624030	10299	11	51	74.34848485	9.30920541	0.85303523	0.036410059	+	148479	grey	0.305855052	FALSE	PHF13
Region_112	chr1	6624868	6635586	10718	15	81	105.848484	9.44334103	0.67094293	0.02399748	+	90326	grey	0.166900392	FALSE	THAP3

							8	9	3	4						
Region_113	chr1	6634168	6701924	67756	81	614	679.7878788	27.44702038	0.918115883	0.013758212	-	55735	grey	0.378700062	FALSE	DNAJC11
Region_114	chr1	6724637	6730012	5375	5	17	30.03030303	6.461649461	0.914029035	0.061911892	+	100505887	grey	0.160772388	FALSE	LINC01672
Region_115	chr1	6785454	7769706	984252	474	3636	3852.924242	107.4705413	0.822982157	0.009279239	+	23261	grey	0.173110872	FALSE	CAMTA1
Region_116	chr1	7771296	7781432	10136	4	26	35.74242424	5.517275264	0.925834645	0.048641893	+	9341	grey	-0.161256616	FALSE	VAMP3
Region_117	chr1	7784291	7845177	60886	38	267	318.8181818	23.01297901	0.905339217	0.015365462	+	8863	grey	-0.087442683	FALSE	PER3
Region_118	chr1	7843083	7853512	10429	13	79	108.2121212	13.29605511	0.924731381	0.032636633	-	10911	grey	-0.256551634	FALSE	UTS2
Region_119	chr1	7915871	7943165	27294	13	88	103.6666667	7.855097969	0.955130266	0.025435879	-	3604	grey	-0.029610419	FALSE	TNFRSF9
Region_120	chr1	7954291	7985505	31214	13	83	108.2272727	10.85965781	0.703856749	0.035225954	+	11315	grey	-0.044523935	FALSE	PARK7
Region_121	chr1	8004404	8026309	21905	8	41	58.74242424	8.027473223	0.642424995	0.051482582	-	54206	grey	-0.006601723	FALSE	ERRFI1
Region_122	chr1	8201518	8215210	13692	8	48	65.90909091	8.481159704	0.683329306	0.053412265	+	102724539	grey	0.036191579	FALSE	LINC01714
Region_123	chr1	8318114	8344167	26053	19	115	145.0757576	12.76571202	0.83479404	0.018682422	+	50651	grey	-0.055524673	FALSE	SLC45A1
Region_124	chr1	8352397	8848921	496524	172	1314	1402.075758	45.70921826	0.832867814	0.010330442	-	473	grey	-0.056033609	FALSE	RERE

Region_125	chr1	8424645	8435013	10368	3	18	27.03030303	4.547085954	0.662059216	0.090781598	+	102724552	grey	0.032940504	FALSE	RERE-AS1
Region_126	chr1	8861000	8879190	18190	26	153	188.6515152	17.82521328	0.142634303	0.035237497	-	2023	grey	-0.152724764	FALSE	ENO1
Region_127	chr1	8878835	8879894	1059	8	32	54.86363636	9.2800636	0.033705747	0.01880198	+	100505975	grey	-0.272884163	FALSE	ENO1-AS1
Region_128	chr1	8945867	8975092	29225	17	118	142.6212121	11.90315427	0.847220431	0.03043074	+	765	grey	-0.046258046	FALSE	CA6
Region_129	chr1	9002973	9026423	23450	16	88	113.3333333	10.61542178	0.865611623	0.03476122	-	155184	grey	-0.145612012	FALSE	SLC2A7
Region_130	chr1	9035106	9088478	53372	25	162	203.7878788	14.81217291	0.785258892	0.026816562	-	6518	grey	-0.015120982	FALSE	SLC2A5
Region_131	chr1	9100305	9129102	28797	16	84	113.2575758	11.91935802	0.795852811	0.02760021	-	80045	grey	0.174455385	FALSE	GPR157
Region_132	chr1	9148011	9182392	34381	21	150	179.4393939	12.64683455	0.833427924	0.027967442	-	106614088	grey	0.012040029	FALSE	MIR34AHG
Region_133	chr1	9182004	9196284	14280	15	78	103.8636364	10.9332047	0.816669075	0.024511861	+	102724571	grey	0.391564082	FALSE	LNCTAM34A
Region_134	chr1	9234774	9271337	36563	46	314	371.5454545	26.48144884	0.757033892	0.024907319	+	9563	grey	-0.036033637	FALSE	H6PD
Region_135	chr1	9292894	9369532	76638	78	522	585.3939394	31.33240092	0.831470675	0.013725285	+	80176	grey	-0.110053362	FALSE	SPSB1
Region_136	chr1	9425094	9440564	15470	13	88	114.030303	10.88254735	0.569181393	0.028934008	-	100506022	grey	0.014143795	FALSE	LINC02606
Region_137	chr1	9539465	9585173	45708	23	132	166.227272	14.6484921	0.87650862	0.02028605	+	84275	grey	-0.16644559	FALSE	SLC25A33

							7	3	2	7						
Region_138	chr1	9588911	9614877	25966	23	144	187.833333 3	15.3828909 3	0.79953865 8	0.01245204 7	+	199953	grey	-0.01068469 5	FALSE	TMEM201
Region_139	chr1	9629889	9729114	99225	92	594	675.227272 7	30.9549526 1	0.77725367 5	0.01342244 1	+	5293	grey	-0.08772389 4	FALSE	PIK3CD
Region_140	chr1	9652610	9654586	1976	4	13	26.3939393 9	5.08959357	0.01242039 2	0.02259505 4	-	644997	grey	0.271666093	FALSE	PIK3CD-AS1
Region_141	chr1	9672405	9687583	15178	11	52	67.2121212 1	7.85153615 8	0.63634338 7	0.04278288 2	-	10192907 4	grey	-0.02997017 5	FALSE	PIK3CD-AS2
Region_142	chr1	9728926	9823984	95058	93	665	725.196969 7	27.7761489 8	0.91005777 2	0.00814983 5	-	22883	grey	-0.27628794	FALSE	CLSTN1
Region_143	chr1	9848276	9910336	62060	52	334	376.151515 2	20.8424437	0.70862733 1	0.01945812	-	56998	grey	0.144093381	FALSE	CTNNBIP1
Region_144	chr1	9922113	9943407	21294	21	92	132.712121 2	14.8145096 8	0.78712319 7	0.01634080 3	-	84328	grey	0.050382661	FALSE	LZIC
Region_145	chr1	9942923	9985501	42578	31	180	225.060606 1	15.679363	0.80466767 3	0.01740205 3	+	64802	grey	-0.16701916 9	FALSE	NMNAT1
Region_146	chr1	9950572	9960787	10215	6	25	36.3484848 5	4.92869907 7	0.72990637 3	0.05507052	-	11339133 5	grey	-0.06626681 9	FALSE	TMEM274P
Region_147	chr1	9997206	10016021	18815	11	70	90.2424242 4	9.04528879 7	0.76397745 4	0.03237743 9	+	116362	grey	-0.12621848	FALSE	RBP7
Region_148	chr1	10032832	10181239	148407	73	517	601.181818 2	25.8390932 7	0.896936	0.01058144 4	+	10277	grey	0.123636669	FALSE	UBE4B
Region_149	chr1	10210570	10381603	171033	67	486	546.742424 2	29.9478061 9	0.86951316 6	0.01516918 3	+	23095	grey	0.060071152	FALSE	KIF1B

Region_150	chr1	10398592	10420511	21919	15	105	134.0757576	12.67621711	0.69698156	0.017565944	+	5226	grey	-0.216742155	FALSE	PGD
Region_151	chr1	10430102	10452153	22051	20	136	156.7121212	11.62856582	0.695594763	0.025765345	+	100526739	grey	-0.280896225	FALSE	CENPS-CORT
Region_152	chr1	10430433	10442808	12375	11	73	90.65151515	9.375077311	0.69105212	0.032026573	+	378708	grey	-0.079193406	FALSE	CENPS
Region_153	chr1	10456522	10472529	16007	10	60	79.63636364	8.972841697	0.972713731	0.021307958	-	1676	grey	0.117912536	FALSE	DFFA
Region_154	chr1	10472288	10630758	158470	97	668	752.6212121	35.09186412	0.850934319	0.013452735	+	5195	grey	0.221250142	FALSE	PEX14
Region_155	chr1	10636604	10796650	160046	187	1137	1311.409091	67.54621831	0.814122848	0.010871859	-	54897	grey	0.135013299	FALSE	CASZ1
Region_156	chr1	10946471	10982076	35605	36	246	284.3636364	18.28882497	0.758880348	0.022294107	-	148345	grey	0.036991385	FALSE	C1orf127
Region_157	chr1	11012344	11030528	18184	15	90	118.4090909	10.12685276	0.943629689	0.028553717	+	23435	grey	0.048102119	FALSE	TARDBP
Region_158	chr1	11022009	11047239	25230	18	111	137.0151515	11.57782021	0.885472028	0.024819528	-	10747	grey	-0.272540096	FALSE	MASP2
Region_159	chr1	11054584	11060020	5436	3	13	22.8030303	4.700964539	0.90433618	0.065147333	-	6723	grey	-0.00081316	FALSE	SRM
Region_160	chr1	11066618	11099869	33251	26	154	195.6363636	17.34415119	0.914807098	0.025331554	-	5394	grey	0.104967913	FALSE	EXOSC10
Region_161	chr1	11106535	11262556	156021	48	312	365.1363636	19.73274231	0.934337143	0.01590581	-	2475	grey	-0.039232516	FALSE	MTOR
Region_162	chr1	11189355	11195981	6626	4	19	38.1818181	6.61213125	0.97149325	0.02954088	+	10218	grey	0.100685815	FALSE	ANGPTL7

							8	4	9	4						
Region_163	chr1	11273198	11296049	22851	3	15	24.54545455	4.574786479	0.968680814	0.036170825	+	29914	grey	0.347014326	FALSE	UBIAD1
Region_164	chr1	11479155	11537551	58396	36	220	276	20.24237748	0.76853714	0.022421822	+	57540	grey	0.164544006	FALSE	DISP3
Region_165	chr1	11500803	11502016	1213	4	13	31.01515152	6.335473813	0.680434864	0.090239173	-	112268221	grey	0.240787582	FALSE	
Region_166	chr1	11637018	11655785	18767	11	60	75.74242424	8.411184141	0.626171142	0.044487432	-	26232	grey	0.105853158	FALSE	FBXO2
Region_167	chr1	11654375	11663327	8952	4	11	21.46969697	5.754515811	0.738380422	0.049598497	+	93611	grey	0.113378422	FALSE	FBXO44
Region_168	chr1	11658918	11691811	32893	35	211	250.8484848	19.63140531	0.598510776	0.027914721	-	10459	grey	0.20259773	FALSE	MAD2L2
Region_169	chr1	11664200	11674354	10154	16	85	110.030303	11.68163542	0.753582742	0.021904732	+	26270	grey	-0.112970408	FALSE	FBXO6
Region_170	chr1	11691710	11725857	34147	43	274	325.5757576	19.72274018	0.771379752	0.018474505	+	374946	grey	0.180443654	FALSE	DRAXIN
Region_171	chr1	11736084	11754802	18718	18	113	143.3484848	13.07080414	0.683853625	0.03542671	+	57085	grey	0.019223774	FALSE	AGTRAP
Region_172	chr1	11761787	11789585	27798	21	118	139.7575758	10.58890654	0.691638528	0.033978686	+	284498	grey	0.211051011	FALSE	C1orf167
Region_173	chr1	11785723	11806455	20732	23	143	169.2575758	14.57849903	0.752787449	0.016929942	-	4524	grey	0.064669535	FALSE	MTHFR
Region_174	chr1	11806096	11848079	41983	23	154	196.7878788	15.69763838	0.849447678	0.023165945	+	1185	grey	0.07359823	FALSE	CLCN6

Region_175	chr1	11919591	11926428	6837	8	42	64.21212121	8.606822252	0.442273452	0.027810132	-	90231	grey	-0.109997737	FALSE	KIAA2013
Region_176	chr1	11934205	11975538	41333	40	262	299.3939394	18.98005333	0.896222199	0.017542392	+	5351	grey	0.193919855	FALSE	PLOD1
Region_177	chr1	11980181	12015211	35030	34	232	269.0606061	19.69137163	0.803678924	0.023620268	+	9927	grey	-0.006073047	FALSE	MFN2
Region_178	chr1	12019466	12032045	12579	10	58	77.87878788	8.960623521	0.798224997	0.042386758	+	60672	grey	0.133012739	FALSE	MIIP
Region_179	chr1	12063303	12144207	80904	55	347	382.8484848	19.65490146	0.787524377	0.020035536	+	943	grey	0.117822255	FALSE	TNFRSF8
Region_180	chr1	12166991	12209228	42237	34	209	249.5151515	19.01759705	0.813820757	0.029188456	+	7133	grey	0.074718511	FALSE	TNFRSF1B
Region_181	chr1	12230030	12512047	282017	84	642	706.2424242	31.443263	0.896524277	0.00909529	+	55187	grey	-0.020529886	FALSE	VPS13D
Region_182	chr1	12567910	12618210	50300	33	223	268.1818182	19.91049904	0.787630201	0.022906312	-	9249	grey	0.053144711	FALSE	DHRS3
Region_183	chr1	12644085	12667076	22991	7	42	61.66666667	9.480884953	0.948229413	0.036011447	+	343066	grey	0.002525536	FALSE	AADACL4
Region_184	chr1	13474973	13514003	39030	22	131	164.1212121	12.97514209	0.838027314	0.037435307	-	126755	grey	0.044099568	FALSE	LRRC38
Region_185	chr1	13700188	13825079	124891	70	532	586.4545455	26.17995817	0.686797152	0.0124835	+	7799	grey	0.099441261	FALSE	PRDM2
Region_186	chr1	13892792	15118043	1225251	323	2520	2666.560606	84.0714218	0.808118088	0.011669976	+	23254	grey	-0.051283735	FALSE	KAZN
Region_187	chr1	14221887	14304445	82558	19	147	176.363636	12.5252193	0.81174162	0.03127273	-	10798546	grey	-0.26606100	FALSE	

							4	1				8		8		
Region_188	chr1	14338825	14419973	81148	13	86	109.4393939	9.101358796	0.738179497	0.047949856	-	105376762	grey	0.095831001	FALSE	KAZN-AS1
Region_189	chr1	15111815	15153618	41803	14	97	126.3030303	11.32182331	0.668220982	0.033692136	-	200197	grey	-0.013710024	FALSE	TMEM51-AS1
Region_190	chr1	15152532	15220478	67946	28	177	204.7121212	12.40070116	0.653570772	0.020708677	+	55092	grey	-0.310346321	FALSE	TMEM51
Region_191	chr1	15236521	15400283	163762	105	795	853.6212121	34.88740798	0.821482478	0.013538636	+	114827	grey	0.427683554	FALSE	FHAD1
Region_192	chr1	15326680	15343876	17196	14	95	119.0606061	11.1505485	0.733679114	0.040044733	-	101927417	grey	0.197544617	FALSE	FHAD1-AS1
Region_193	chr1	15409888	15430339	20451	17	87	109.969697	10.64962216	0.519858217	0.037620522	+	79180	grey	0.00666389	FALSE	EFHD2
Region_194	chr1	15438442	15449242	10800	7	34	54.68181818	6.594074475	0.805648003	0.067705281	+	11330	grey	-0.040213675	FALSE	CTRC
Region_195	chr1	15456728	15472091	15363	4	18	30.59090909	5.326211737	0.898613686	0.087066602	+	63036	grey	-0.074095883	FALSE	CELA2A
Region_196	chr1	15465909	15491395	25486	7	46	59.93939394	8.01705641	0.815616242	0.037766127	+	51032	grey	-0.105360644	FALSE	CELA2B
Region_197	chr1	15490832	15526534	35702	17	119	143.8636364	14.092973	0.642196688	0.024816559	-	842	grey	-0.288660323	FALSE	CASP9
Region_198	chr1	15526813	15592379	65566	28	169	211.8636364	17.21438251	0.86546504	0.016658208	+	23341	grey	0.135172791	FALSE	DNAJC16
Region_199	chr1	15571699	15585051	13352	4	20	31.25757576	5.858092643	0.702000309	0.063768488	-	79814	grey	-0.019756668	FALSE	AGMAT

Region_200	chr1	15617458	15669044	51586	24	175	196.1666667	10.4874772 7	0.875430959	0.020937127	+	84301	grey	0.162432623	FALSE	DDI2
Region_201	chr1	15684320	15734769	50449	30	176	222.5454545	16.5438176 4	0.848815535	0.018478044	+	23207	grey	-0.073880532	FALSE	PLEKHM2
Region_202	chr1	15736258	15741392	5134	9	41	65.33333333	10.6110730 7	0.959026912	0.026725506	+	284723	grey	-0.163653737	FALSE	SLC25A34
Region_203	chr1	15740048	15749896	9848	8	42	63.1969697	8.250351409	0.941345807	0.034443474	-	110806303	grey	-0.365863352	FALSE	SLC25A34-AS1
Region_204	chr1	15742499	15747982	5483	5	25	42.43939394	6.977168894	0.970491512	0.029326201	+	388595	grey	-0.135823519	FALSE	TMEM82
Region_205	chr1	15756607	15786594	29987	18	103	124.3181818	10.44275102	0.723862441	0.023180054	+	54751	grey	0.123826666	FALSE	FBLIM1
Region_206	chr1	15834474	15848147	13673	20	105	135.5757576	13.73379953	0.071509892	0.014185602	-	729614	grey	-0.202573404	FALSE	SPEN-AS1
Region_207	chr1	15836095	15940456	104361	66	429	490.1818182	26.16098627	0.679835061	0.012391845	+	23013	grey	-0.211649852	FALSE	SPEN
Region_208	chr1	15941869	15976132	34263	26	167	224.0909091	16.83378222	0.851371356	0.023510879	-	7709	grey	0.077499264	FALSE	ZBTB17
Region_209	chr1	16004236	16008807	4571	3	12	20.48484848	3.860021619	0.818212273	0.106133853	+	149563	grey	0.119562394	FALSE	SRARP
Region_210	chr1	16014028	16019594	5566	10	38	56.59090909	8.717757779	0.728305779	0.077725655	-	27129	grey	0.068804829	FALSE	HSPB7
Region_211	chr1	16018875	16034050	15175	9	38	59.59090909	9.136532372	0.690417184	0.05614485	+	1187	grey	0.019170492	FALSE	CLCNKA
Region_212	chr1	16040252	16057311	17059	10	53	75.7575757	14.7492483	0.92171507	0.04153385	+	1188	grey	0.046607374	FALSE	CLCNKB

							6	3	3	7						
Region_213	chr1	16057769	16073651	15882	10	40	65.53030303	10.4516759	0.690669206	0.051719426	-	348487	grey	-0.225747211	FALSE	FAM131C
Region_214	chr1	16124337	16156069	31732	35	216	282.6515152	22.55420304	0.721157111	0.017102476	-	1969	grey	0.228070991	FALSE	EPHA2
Region_215	chr1	16155176	16161883	6707	18	127	150.7121212	12.29229296	0.595038821	0.022170735	+	101927479	grey	0.059979612	FALSE	EPHA2-AS1
Region_216	chr1	16197854	16212652	14798	9	35	50.92424242	8.311827728	0.485709582	0.047621881	-	128272	grey	0.22558063	FALSE	ARHGEF19
Region_217	chr1	16231692	16237183	5491	3	15	23.72727273	4.149311174	0.970179428	0.04367334	-	79363	grey	-0.016845733	FALSE	CPLANE2
Region_218	chr1	16246840	16352480	105640	65	452	513.4848485	28.59329799	0.875617778	0.013351651	-	54455	grey	-0.008721361	FALSE	FBXO42
Region_219	chr1	16352575	16398145	45570	31	199	241.5151515	19.78285382	0.873833616	0.022013488	+	26099	grey	-0.18742538	FALSE	SZRD1
Region_220	chr1	16387117	16437424	50307	26	160	195.0151515	14.52689011	0.946219554	0.023470008	-	374955	grey	-0.161956682	FALSE	SPATA21
Region_221	chr1	16440721	16460078	19357	15	98	127.1666667	11.56597574	0.653954414	0.01826441	+	55707	grey	-0.163405774	FALSE	NECAP2
Region_222	chr1	16460948	16468481	7533	3	15	22.5	4.714135862	0.534066804	0.212880931	+	107984921	grey	-0.081394858	FALSE	LINC01772
Region_223	chr1	16467436	16499257	31821	27	177	210.4545455	17.04851161	0.740007115	0.01549398	-	114819	grey	0.139361212	FALSE	CROCCP3
Region_224	chr1	16533886	16535649	1763	4	13	32.59090909	6.537412503	0.328865354	0.072924451	-	100132147	grey	0.006160289	FALSE	LINC01783

Region_225	chr1	16562319	16613562	51243	3	27	50.5757575 8	11.2579006 2	0.92661268	0.04410209 4	-	55672	grey	0.001852907	FALSE	NBPF1
Region_226	chr1	16618253	16657232	38979	21	249	362.787878 8	49.2901972 3	0.76467863 9	0.01657654 3	-	84809	grey	-0.03554267 2	FALSE	CROCCP2
Region_227	chr1	16687339	16720157	32818	28	134	267.030303	73.8670259 9	0.50048886 4	0.04586286 8	-	284729	grey	-0.2300316	FALSE	ESPNP
Region_228	chr1	16740273	16972964	232691	103	757	993.454545 5	129.676898 1	0.76878744 3	0.01612732 9	+	9696	grey	-0.16901773 4	FALSE	CROCC
Region_229	chr1	16754910	16770237	15327	7	38	68.2272727 3	15.6665203 6	0.68508265 2	0.05733181 4	-	11223	grey	-0.09003767 7	FALSE	MST1L
Region_230	chr1	16870945	16883659	12714	13	84	129.075757 6	22.8374258 3	0.76117387	0.04226724 2	+	11226787 1	grey	-0.30115188 4	FALSE	LOC112267871
Region_231	chr1	16985958	17011928	25970	18	111	139.818181 8	14.3708134 7	0.85933250 2	0.02704386 6	-	23400	grey	-0.07821991 8	FALSE	ATP13A2
Region_232	chr1	17018722	17054032	35310	29	196	234.272727 3	17.4025156 9	0.87862541 2	0.01736309	-	6390	grey	0.182729166	FALSE	SDHB
Region_233	chr1	17066761	17119451	52690	22	144	175.363636 4	12.6072462 2	0.76361721 4	0.03001872 1	-	11240	grey	0.095341131	FALSE	PADI2
Region_234	chr1	17539698	17697874	158176	91	661	727.727272 7	30.7458792 5	0.80123124	0.01403384 2	+	55160	grey	0.005425981	FALSE	ARHGEF10L
Region_235	chr1	17717625	17749978	32353	21	127	156.242424 2	12.3426223 4	0.79128535 2	0.03849610 6	+	10798402 8	grey	-0.16413741 7	FALSE	LINC02810
Region_236	chr1	17755333	17827063	71730	34	212	267.666666 7	20.1244843 9	0.85584327 9	0.02841644	+	81569	grey	0.062977973	FALSE	ACTL8
Region_237	chr1	18015712	18045612	29900	13	79	98.7424242	9.64972794	0.75268888	0.03847716	+	10537681	grey	-0.16137084	FALSE	LOC105376812

							4	5		4		2		2		
Region_238	chr1	18065657	18074412	8755	3	28	36.93939394	5.110709082	0.854941197	0.053253166	+	101927876	grey	-0.07497911	FALSE	LINC01654
Region_239	chr1	18107798	18378483	270685	88	648	709.2272727	27.60423348	0.706341221	0.021692621	+	84966	grey	-0.100452586	FALSE	IGSF21
Region_240	chr1	18166929	18179346	12417	7	33	50.25757576	7.600223896	0.59777547	0.067919934	-	105376814	grey	-0.27732617	FALSE	IGSF21-AS1
Region_241	chr1	18480930	18485974	5044	6	26	45.95454545	7.8670331	0.914127535	0.053113797	+	127707	grey	0.204896076	FALSE	KLHDC7A
Region_242	chr1	18630846	18748866	118020	37	247	282.8939394	17.94361149	0.471067778	0.023163808	+	5081	grey	-0.186811994	FALSE	PAX7
Region_243	chr1	18839599	18859660	20061	6	28	51.86363636	8.318443574	0.882018021	0.045281421	-	80834	grey	-0.083734912	FALSE	TAS1R2
Region_244	chr1	18871430	18902724	31294	17	113	136.530303	10.79489865	0.668847238	0.027790454	-	8659	grey	-0.356828107	FALSE	ALDH4A1
Region_245	chr1	18904280	18956676	52396	30	188	222.3333333	12.68109839	0.592710114	0.025287648	-	126917	grey	0.060661047	FALSE	IFFO2
Region_246	chr1	19074510	19210266	135756	42	310	370.3030303	18.47490581	0.80517138	0.015685233	-	23352	grey	0.192409373	FALSE	UBR4
Region_247	chr1	19210348	19240709	30361	15	106	131.7575758	12.06018319	0.963625598	0.016429819	+	101927895	grey	-0.183514855	FALSE	EMC1-AS1
Region_248	chr1	19215660	19251552	35892	24	197	226.3030303	14.63816897	0.888385206	0.019100956	-	23065	grey	-0.028127689	FALSE	EMC1
Region_249	chr1	19251805	19260128	8323	3	14	23.48484848	4.78537009	0.293862871	0.045938437	+	51154	grey	-0.052221651	FALSE	MRTO4

Region_250	chr1	19282573	19288770	6197	4	15	25.5	4.598494737	0.735063432	0.03112706	-	22977	grey	0.357382791	FALSE	AKR7A3
Region_251	chr1	19312326	19329300	16974	7	28	42.3030303	6.307430775	0.694237366	0.074041488	+	54896	grey	0.023925053	FALSE	SLC66A1
Region_252	chr1	19338775	19485539	146764	81	619	690.7878788	37.32333366	0.83904037	0.014352975	-	832	grey	-0.148057692	FALSE	CAPZB
Region_253	chr1	19484403	19629821	145418	42	275	313.0151515	16.97100915	0.764683229	0.017949896	+	440574	grey	0.109424741	FALSE	MICOS10
Region_254	chr1	19591802	19596832	5030	3	21	30.15151515	4.507233244	0.646607994	0.030350069	-	105378614	grey	-0.265679859	FALSE	MICOS10-DT
Region_255	chr1	19596979	19658056	61077	34	219	252.1515152	15.95690583	0.697007321	0.020624481	+	100532736	grey	-0.007200031	FALSE	MICOS10-NBL1
Region_256	chr1	19640554	19658456	17902	17	96	121.1212121	10.73673085	0.703112241	0.033902418	+	4681	grey	0.03554036	FALSE	NBL1
Region_257	chr1	19664875	19680966	16091	17	94	121.6060606	12.57577499	0.454342142	0.033605937	+	3362	grey	-0.080246176	FALSE	HTR6
Region_258	chr1	19682240	19799945	117705	51	405	450.6060606	21.44537159	0.902029928	0.016378342	-	255104	grey	-0.221846917	FALSE	TMCO4
Region_259	chr1	19882395	19912945	30550	13	62	90.5	11.4330829	0.583379398	0.022239624	+	23252	grey	0.096076486	FALSE	OTUD3
Region_260	chr1	20028179	20091911	63732	18	122	146.530303	12.45820051	0.826077268	0.037806242	+	5322	grey	-0.125236262	FALSE	PLA2G5
Region_261	chr1	20139323	20150381	11058	7	32	55.72727273	7.952832983	0.774260895	0.056648212	+	64600	grey	-0.134939233	FALSE	PLA2G2F
Region_262	chr1	20161253	20186518	25265	8	31	53.1212121	7.19939132	0.82144218	0.03492955	-	391013	grey	0.24063953	FALSE	PLA2G2C

							2	4		5						
Region_263	chr1	20186096	20196050	9954	4	19	32.28787879	5.856659987	0.613214964	0.102718575	+	127733	grey	0.070972923	FALSE	UBXN10
Region_264	chr1	20290875	20359518	68643	22	138	166.2272727	13.59727965	0.790080508	0.031466936	+	127731	grey	-0.346581285	FALSE	VWA5B1
Region_265	chr1	20360579	20431234	70655	12	74	96.63636364	9.443982803	0.711722164	0.050800396	-	339505	grey	-0.004562974	FALSE	LINC01141
Region_266	chr1	20552573	20555020	2447	4	14	30.74242424	5.407432509	0.315553943	0.06973865	+	163933	grey	0.045972805	FALSE	FAM43B
Region_267	chr1	20589086	20618903	29817	9	50	69.10606061	8.781324217	0.914549982	0.05360176	+	978	grey	0.05668675	FALSE	CDA
Region_268	chr1	20633458	20651511	18053	10	61	75.96969697	7.881760243	0.747655531	0.040254344	+	65018	grey	0.062214525	FALSE	PINK1
Region_269	chr1	20642657	20652193	9536	6	34	48.15151515	6.476567049	0.915691019	0.059252532	-	100861548	grey	0.091717039	FALSE	PINK1-AS
Region_270	chr1	20651767	20661544	9777	5	27	39.27272727	6.508090715	0.509757848	0.079718941	-	1650	grey	-0.282857998	FALSE	DDOST
Region_271	chr1	20664014	20718017	54003	32	213	244.8787879	15.52220757	0.889469559	0.024674878	-	57576	grey	0.155034212	FALSE	KIF17
Region_272	chr1	20719731	20732837	13106	4	17	31.06060606	6.326692629	0.679296306	0.055001614	-	400745	grey	-0.142228474	FALSE	SH2D5
Region_273	chr1	20740266	20787323	47057	11	58	77.59090909	10.31500367	0.511438194	0.042471035	-	50809	grey	-0.020078446	FALSE	HP1BP3
Region_274	chr1	20806292	21177285	370993	88	642	690.6363636	26.13258288	0.865426253	0.011831411	-	8672	grey	-0.145615378	FALSE	EIF4G3

Region_275	chr1	21217247	21345572	128325	39	257	287.2575758	16.06937901	0.845046311	0.022900279	-	1889	grey	0.128083407	FALSE	ECE1
Region_276	chr1	21440128	21485005	44877	8	55	74.96969697	8.397746395	0.505983053	0.028864718	+	84224	grey	-0.138208752	FALSE	NBPF3
Region_277	chr1	21509397	21578410	69013	43	294	340.4545455	21.56933851	0.789968242	0.025924365	+	249	grey	0.002214157	FALSE	ALPL
Region_278	chr1	21586472	21591187	4715	6	28	41.62121212	6.923238796	0.510030948	0.074286632	+	105376834	grey	0.053394316	FALSE	LINC02596
Region_279	chr1	21596221	21669357	73136	45	280	320.1363636	20.23468946	0.651594219	0.023962479	-	5909	grey	0.246030656	FALSE	RAP1GAP
Region_280	chr1	21678298	21783606	105308	45	336	382.9242424	24.26349623	0.838226595	0.018080042	-	84196	grey	-0.042631282	FALSE	USP48
Region_281	chr1	21822244	21937310	115066	88	545	633.6818182	31.54930651	0.900105898	0.01436777	-	3339	grey	-0.295562063	FALSE	HSPG2
Region_282	chr1	21977022	21998642	21620	12	56	72.45454545	8.114909011	0.854056985	0.036863811	+	23436	grey	0.080797865	FALSE	CELA3B
Region_283	chr1	21983606	22030617	47011	22	120	147.1666667	12.45741464	0.729877208	0.032124939	+	10136	grey	0.109789017	FALSE	CELA3A
Region_284	chr1	22005305	22031223	25918	10	60	75.36363636	8.789217807	0.629743882	0.037649618	+	29092	grey	0.073285978	FALSE	LINC00339
Region_285	chr1	22025142	22101360	76218	26	174	200.5909091	15.56818973	0.715964794	0.034102053	+	998	grey	-0.154662473	FALSE	CDC42
Region_286	chr1	22028317	22052927	24610	9	50	73.54545455	8.915296825	0.848665036	0.031814394	-	112268223	grey	-0.256333432	FALSE	CDC42-AS1
Region_287	chr1	22117313	22143969	26656	26	166	194	14.5115338	0.65973552	0.02601765	-	54361	grey	-0.02113810	FALSE	WNT4

								7	2	4				9		
Region_288	chr1	22142850	22157401	14551	9	41	53	6.49733673 2	0.72893381 2	0.04197729 4	+	10537684 5	grey	-0.16367897	FALSE	LOC105376845
Region_289	chr1	22428838	22531157	102319	40	275	318.969697	19.9799666 6	0.87278835 7	0.02498076 2	+	9923	grey	-0.03940850 6	FALSE	ZBTB40
Region_290	chr1	22563489	22603595	40106	39	240	310.878787 9	24.5660899 2	0.78845445 5	0.02862758 1	+	2046	grey	-0.14991183 9	FALSE	EPHA8
Region_291	chr1	22635077	22644067	8990	5	20	34.0909090 9	6.05037131 1	0.53094613 7	0.06746189 3	+	712	grey	-0.24792763 5	FALSE	C1QA
Region_292	chr1	22710839	22921500	210661	86	585	665.151515 2	34.5076323 4	0.82717737 3	0.01789555 5	+	2048	grey	0.239693145	FALSE	EPHB2
Region_293	chr1	22953043	22972866	19823	13	87	109.969697	8.76701808 3	0.61872847 6	0.04142495 9	-	646262	grey	-0.11951511 3	FALSE	LACTBL1
Region_294	chr1	23019443	23083689	64246	20	131	160.893939 4	11.9552097 3	0.80066238 5	0.02094185 8	+	23028	grey	0.251492218	FALSE	KDM1A
Region_295	chr1	23084030	23177808	93778	33	227	254.075757 6	14.8171528 5	0.81928103 3	0.01698194 6	-	7798	grey	0.076946448	FALSE	LUZP1
Region_296	chr1	23191895	23217502	25607	9	45	61.6969697	7.27476078 8	0.87517276 3	0.03976967 4	-	3352	grey	-0.08131962 6	FALSE	HTR1D
Region_297	chr1	23576436	23593621	17185	4	23	37.1060606 1	6.00801717 7	0.90263788 9	0.06844223 7	-	10798492 8	grey	0.045226387	FALSE	LOC107984928
Region_298	chr1	23581495	23640568	59073	14	103	126.409090 9	11.7891439 9	0.66171725 8	0.05076858 7	+	259283	grey	-0.09958871 6	FALSE	MDS2
Region_299	chr1	23706901	23778325	71424	48	314	352.893939 4	22.6295441 9	0.81626411	0.01878063 1	-	10050696 3	grey	-0.14846141 3	FALSE	ELOA-AS1

Region_300	chr1	23743448	23762059	18611	13	63	78.09090909	8.200801598	0.730473576	0.036854538	+	6924	grey	0.019247586	FALSE	ELOA
Region_301	chr1	23801885	23838620	36735	18	98	126.2575758	11.67418006	0.838216836	0.024231325	-	3155	grey	-0.302888747	FALSE	HMGCL
Region_302	chr1	23845077	23868290	23213	22	137	174.7575758	16.47101006	0.707467914	0.012707455	-	2517	grey	-0.10099299	FALSE	FUCA1
Region_303	chr1	23870515	23913362	42847	22	124	153.6969697	13.30814637	0.764230083	0.03637747	-	1269	grey	-0.153072107	FALSE	CNR2
Region_304	chr1	23956839	23963462	6623	5	30	47.96969697	6.992236787	0.163928538	0.034073716	+	55629	grey	-0.061625876	FALSE	PNRC2
Region_305	chr1	23964347	23980927	16580	6	21	32.16666667	5.944831843	0.48713062	0.055289365	-	10772	grey	0.271152963	FALSE	SRSF10
Region_306	chr1	24040835	24086799	45964	19	108	145.2121212	15.03996231	0.764389549	0.040162981	+	107984931	grey	-0.115874358	FALSE	LOC107984931
Region_307	chr1	24056035	24112135	56100	37	226	267.1818182	19.27047091	0.859106077	0.024494743	-	127294	grey	-0.317222361	FALSE	MYOM3
Region_308	chr1	24066774	24083565	16791	10	50	68.87878788	8.594300708	0.807575098	0.065969971	+	107985737	grey	-0.150829745	FALSE	MYOM3-AS1
Region_309	chr1	24119771	24143140	23369	13	62	95.89393939	10.58609943	0.891967347	0.040498739	-	58985	grey	0.138334489	FALSE	IL22RA1
Region_310	chr1	24154168	24187959	33791	13	80	100.1818182	9.156601807	0.539211223	0.035033795	-	163702	grey	-0.132247533	FALSE	IFNLR1
Region_311	chr1	24199558	24364482	164924	59	393	473.6666667	28.54757612	0.801348287	0.01636745	+	57822	grey	-0.090249308	FALSE	GRHL3
Region_312	chr1	24200240	24211693	11453	5	26	40.8333333	5.51524693	0.91263189	0.05158516	+	284632	grey	0.174738848	FALSE	LINC02800

							3	2	2	4						
Region_313	chr1	24307556	24321901	14345	4	20	29.86363636	5.065376783	0.66535434	0.073130657	-	105376871	grey	-0.16046278	FALSE	GRHL3-AS1
Region_314	chr1	24356999	24416934	59935	28	203	238.5606061	15.36542451	0.654115863	0.020703497	-	90529	grey	0.052273736	FALSE	STPG1
Region_315	chr1	24415802	24472976	57174	20	150	173.9242424	11.77521596	0.872468341	0.019020047	+	57185	grey	-0.216349677	FALSE	NIPAL3
Region_316	chr1	24496254	24535400	39146	23	154	197.9848485	14.65027427	0.829379086	0.029189853	-	100750325	grey	0.011864451	FALSE	RCAN3AS
Region_317	chr1	24502351	24541040	38689	22	154	186.6515152	14.03456439	0.818888899	0.030805009	+	11123	grey	0.036271328	FALSE	RCAN3
Region_318	chr1	24538802	24556024	17222	6	21	38.25757576	7.31554847	0.52969733	0.050132736	-	100506985	grey	-0.074549158	FALSE	NCMAP-DT
Region_319	chr1	24556087	24609328	53241	18	108	132.2878788	9.805438289	0.678297985	0.02766503	+	400746	grey	-0.276101592	FALSE	NCMAP
Region_320	chr1	24633066	24673281	40215	17	94	121.8484848	10.09987422	0.640090801	0.021289062	+	10250	grey	0.137503829	FALSE	SRRM1
Region_321	chr1	24745382	24844321	98939	30	200	231.9242424	13.74639601	0.907807815	0.019303975	+	25932	grey	-0.032196427	FALSE	CLIC4
Region_322	chr1	24899511	24965121	65610	34	201	235.4848485	17.31450475	0.703748592	0.027563565	-	864	grey	-0.167285554	FALSE	RUNX3
Region_323	chr1	25222276	25232502	10226	5	27	40.74242424	7.353305078	0.932666398	0.039505456	-	25949	grey	-0.200307491	FALSE	SYF2
Region_324	chr1	25242249	25338213	95964	40	174	304.1969697	67.50754253	0.698262892	0.017082948	-	57035	grey	0.005123888	FALSE	RSRP1

Region_325	chr1	25338317	25362361	24044	9	44	66.04545455	9.132245184	0.708431005	0.040995054	+	23585	grey	0.014711016	FALSE	TMEM50A
Region_326	chr1	25362249	25430192	67943	12	64	89.31818182	9.36796356	0.824772087	0.030559405	-	6006	grey	0.199169573	FALSE	RHCE
Region_327	chr1	25430858	25500209	69351	15	94	117.7575758	12.0474199	0.74208682	0.020515658	+	55219	grey	-0.100743906	FALSE	MACO1
Region_328	chr1	25543606	25590356	46750	23	147	169.530303	11.46663346	0.669780542	0.035493665	+	26119	grey	0.04381558	FALSE	LDLRAP1
Region_329	chr1	25616791	25786206	169415	65	472	518.8787879	22.17796214	0.755851435	0.019374407	+	57134	grey	0.117325572	FALSE	MAN1C1
Region_330	chr1	25800193	25823308	23115	11	47	76.10606061	10.14681274	0.287651929	0.026081593	+	57190	grey	0.115156303	FALSE	SELENON
Region_331	chr1	25813973	25832942	18969	9	35	64	9.746005099	0.210618348	0.01829632	+	56181	grey	0.023561815	FALSE	MTFR1L
Region_332	chr1	25818491	25820797	2306	8	29	54.36363636	9.42767834	0.114944468	0.01952407	-	646471	grey	0.092571252	FALSE	LOC646471
Region_333	chr1	25831913	25859458	27545	16	106	133.3787879	11.05060409	0.946951494	0.025932511	-	79000	grey	-0.100081419	FALSE	AUNIP
Region_334	chr1	25861484	25875708	14224	9	47	69.57575758	8.74785355	0.692711309	0.034105211	-	164091	grey	0.117735721	FALSE	PAQR7
Region_335	chr1	25884181	25906991	22810	16	96	123.2424242	13.90855919	0.857228391	0.026446903	-	3925	grey	0.079310955	FALSE	STMN1
Region_336	chr1	25959767	25998117	38350	15	98	130.2878788	11.99585204	0.842141007	0.029455112	-	5051	grey	-0.421573671	FALSE	PAFAH2
Region_337	chr1	26019884	26036464	16580	12	50	78.2272727	10.3771871	0.50268756	0.03898396	+	2134	grey	0.063421928	FALSE	EXTL1

							3	9	5	2						
Region_338	chr1	26037252	26046118	8866	3	17	26.59090909	4.590884858	0.568088101	0.060235823	-	7780	grey	-0.023777639	FALSE	SLC30A2
Region_339	chr1	26051301	26068436	17135	12	72	92.01515152	9.402933134	0.846103738	0.036167113	-	84676	grey	0.198183267	FALSE	TRIM63
Region_340	chr1	26111165	26125555	14390	13	88	109.7272727	10.6273167	0.577049742	0.037360655	+	149420	grey	0.047261763	FALSE	PDIK1L
Region_341	chr1	26177484	26189884	12400	7	41	59.53030303	6.955285089	0.837935773	0.0569447	+	10256	grey	-0.097643507	FALSE	CNKSR1
Region_342	chr1	26190561	26202968	12407	10	51	65.39393939	9.301577679	0.849500459	0.05278466	+	378807	grey	0.320747767	FALSE	CATSPER4
Region_343	chr1	26209741	26229844	20103	8	57	73.25757576	7.6807664	0.879270518	0.029992543	+	101928303	grey	0.015282725	FALSE	ZPLD2P
Region_344	chr1	26234200	26278808	44608	15	90	118.4545455	10.87719112	0.89586854	0.017163128	+	64793	grey	0.066355817	FALSE	CEP85
Region_345	chr1	26280086	26281522	1436	3	10	22.21212121	6.075208516	0.004602756	0.012833203	+	83442	grey	-0.055133815	FALSE	SH3BGRL3
Region_346	chr1	26281328	26318363	37035	10	53	78.33333333	8.974036624	0.622086477	0.033450879	-	91544	grey	0.138074118	FALSE	UBXN11
Region_347	chr1	26317958	26320523	2565	3	14	26.74242424	4.8684561	0.329888952	0.107934939	+	1043	grey	-0.011455652	FALSE	CD52
Region_348	chr1	26321698	26360080	38382	18	89	123	11.95118275	0.862735327	0.030736901	-	55057	grey	-0.044968258	FALSE	CRYBG2
Region_349	chr1	26361634	26374522	12888	15	79	103.969697	11.73944003	0.702332253	0.03598089	-	257101	grey	0.127872514	FALSE	ZNF683

Region_350	chr1	26410817	26429728	18911	6	33	50.68181818	7.842996074	0.530368327	0.03784884	+	79727	grey	0.096543114	FALSE	LIN28A
Region_351	chr1	26432282	26471306	39024	17	109	133.6363636	12.25578214	0.961256302	0.020607579	+	79947	grey	0.050233597	FALSE	DHDDS
Region_352	chr1	26472440	26476642	4202	3	10	16.98484848	3.492816471	0.009493439	0.033795037	+	3151	grey	0.02438558	FALSE	HMGN2
Region_353	chr1	26693236	26782104	88868	34	235	269.2727273	16.64613187	0.701488017	0.017959964	+	8289	grey	0.218814518	FALSE	ARID1A
Region_354	chr1	26787054	26800659	13605	7	47	58.6969697	6.501998975	0.613003004	0.032892635	+	55650	grey	-0.033371412	FALSE	PIGV
Region_355	chr1	26826688	26857604	30916	28	139	177.9545455	16.02635504	0.5971185	0.022908043	+	84243	grey	-0.091411818	FALSE	ZDHHC18
Region_356	chr1	26876132	26890283	14151	7	39	55.3030303	6.69321056	0.704044039	0.024978039	-	54707	grey	-0.073032448	FALSE	GPN2
Region_357	chr1	26890488	26900467	9979	4	20	28.56060606	4.933804236	0.953975889	0.049270934	-	63906	grey	-0.174588831	FALSE	GPATCH3
Region_358	chr1	26900238	26946871	46633	23	161	186.2878788	11.20269906	0.785195264	0.023423141	+	10726	grey	0.396564653	FALSE	NUDC
Region_359	chr1	26949562	26960468	10906	7	29	42.15151515	6.349421353	0.537384648	0.032802811	-	126695	grey	-0.047786619	FALSE	KDF1
Region_360	chr1	26993692	27000886	7194	7	27	44.56060606	7.700407158	0.281235179	0.03316523	+	388610	grey	0.141479756	FALSE	TRNP1
Region_361	chr1	27005020	27012850	7830	4	19	33.1969697	6.021039259	0.563084166	0.0798719	-	115572	grey	0.110857619	FALSE	TENT5B
Region_362	chr1	27098809	27166981	68172	35	231	271.060606	18.3528482	0.91122943	0.01923976	-	6548	grey	-0.13111818	FALSE	SLC9A1

							1	7	8	7				2		
Region_363	chr1	27229106	27234352	5246	8	34	49.28787879	6.768172464	0.938230951	0.036516012	-	105376890	grey	-0.022953389	FALSE	WDTC1-DT
Region_364	chr1	27234632	27308636	74004	27	160	193.3333333	14.78079142	0.885520014	0.015755377	+	23038	grey	-0.083220638	FALSE	WDTC1
Region_365	chr1	27322145	27336400	14255	10	72	98.40909091	11.1050609	0.79911234	0.033294518	+	84065	grey	-0.119044705	FALSE	TMEM222
Region_366	chr1	27342020	27353937	11917	15	75	100.4848485	12.19614368	0.753375353	0.049582885	+	84958	grey	-0.05625	FALSE	SYTL1
Region_367	chr1	27355184	27366961	11777	19	97	130.3030303	13.73593792	0.373485926	0.025190582	-	9064	grey	-0.02538109	FALSE	MAP3K6
Region_368	chr1	27392622	27395814	3192	5	23	42.46969697	6.682283573	0.751561528	0.062792055	+	2827	grey	-0.172223544	FALSE	GPR3
Region_369	chr1	27404230	27490167	85937	35	211	256.0454545	15.24207922	0.783117804	0.020399691	-	10163	grey	0.094714367	FALSE	WASF2
Region_370	chr1	27525805	27530561	4756	3	16	26.62121212	5.456866251	0.373331768	0.050150081	+	105376892	grey	0.21911613	FALSE	LOC105376892
Region_371	chr1	27534035	27604431	70396	24	149	178	13.52945505	0.747610503	0.023696043	-	27245	grey	0.005719616	FALSE	AHDC1
Region_372	chr1	27612064	27635185	23121	13	85	104.530303	11.28677878	0.763899403	0.04232895	-	2268	grey	-0.089682089	FALSE	FGR
Region_373	chr1	27660328	27666279	5951	4	22	37.96969697	5.865071832	0.954093944	0.034047921	-	111216282	grey	-0.171913319	FALSE	LINC02574
Region_374	chr1	27666064	27672212	6148	8	35	57.68181818	7.680462909	0.534726439	0.040944116	-	2537	grey	0.225383203	FALSE	IFI6

Region_375	chr1	27725961	27763116	37155	22	142	181.4090909	17.1965804	0.685524221	0.015149544	+	199870	grey	0.133117542	FALSE	FAM76A
Region_376	chr1	27773219	27824443	51224	20	133	160.7424242	11.65571581	0.925728079	0.019131627	+	23673	grey	0.040370105	FALSE	STX12
Region_377	chr1	27830782	27851676	20894	11	67	84.60606061	8.666254249	0.87057379	0.024859837	+	5511	grey	0.166681263	FALSE	PPP1R8
Region_378	chr1	27872543	27886685	14142	14	89	114.1969697	11.45858052	0.644676911	0.037163093	+	9473	grey	0.099205135	FALSE	THEMIS2
Region_379	chr1	27891524	27914746	23222	15	93	121.030303	12.38854272	0.840701312	0.016998155	-	6118	grey	0.1844521	FALSE	RPA2
Region_380	chr1	27935000	27959157	24157	17	127	152.7424242	14.87518388	0.884155441	0.024433417	+	27293	grey	-0.009831462	FALSE	SMPDL3B
Region_381	chr1	27959588	27968093	8505	7	51	65.72727273	8.071879877	0.621024166	0.031331804	+	55113	grey	0.090483116	FALSE	XKR8
Region_382	chr1	27970344	28088637	118293	34	240	267.8181818	15.30199493	0.913219975	0.015419598	-	2140	grey	-0.040279737	FALSE	EYA3
Region_383	chr1	28147166	28193936	46770	31	196	221.6969697	13.70341866	0.861806461	0.02137639	-	5724	grey	-0.277048677	FALSE	PTAFR
Region_384	chr1	28199456	28233029	33573	15	85	108.7424242	10.58491031	0.912160191	0.022625379	-	22826	grey	-0.114320348	FALSE	DNAJC8
Region_385	chr1	28369582	28500364	130782	38	266	300.7878788	17.1325288	0.877363028	0.016720536	+	65979	grey	0.014876601	FALSE	PHACTR4
Region_386	chr1	28505943	28539300	33357	18	97	128.969697	12.43935405	0.817573914	0.029352646	+	1104	grey	0.348256162	FALSE	RCC1
Region_387	chr1	28505980	28510892	4912	3	18	29.0757575	4.67829885	0.28550107	0.08245011	+	8420	grey	0.513997105	FALSE	SNHG3

							8	6	4							
Region_388	chr1	28553085	28578545	25460	21	119	146.9545455	11.8975522	0.97248308	0.012101277	+	54952	grey	-0.241589491	FALSE	TRNAU1AP
Region_389	chr1	28578538	28582986	4448	7	58	75.60606061	8.845474789	0.012658539	0.015565433	-	85028	grey	-0.042539219	FALSE	SNHG12
Region_390	chr1	28587829	28648291	60462	32	211	246.6666667	15.49027818	0.741135658	0.017406401	-	6883	grey	0.104615277	FALSE	TAF12
Region_391	chr1	28643228	28648581	5353	4	19	32.56060606	5.096390239	0.431943246	0.081767899	+	105378616	grey	0.199701039	FALSE	TAF12-DT
Region_392	chr1	28668778	28719353	50575	29	178	220.4848485	16.79857359	0.909300092	0.011835757	+	10691	grey	0.131660687	FALSE	GMEB1
Region_393	chr1	28736621	28769775	33154	11	54	80.62121212	9.090595566	0.776905315	0.04498836	+	51441	grey	-0.043538368	FALSE	YTHDF2
Region_394	chr1	28812170	28871267	59097	36	224	260.5606061	17.50879233	0.79269332	0.022228558	+	4985	grey	-0.006995547	FALSE	OPRD1
Region_395	chr1	28887091	29120046	232955	110	752	820.7121212	32.13948693	0.933289491	0.008999471	+	2035	grey	0.045703562	FALSE	EPB41
Region_396	chr1	29119429	29123903	4474	8	38	51.59090909	7.242985514	0.016600272	0.01677123	-	399474	grey	-0.085352476	FALSE	TMEM200B
Region_397	chr1	29147743	29181900	34157	17	101	123.0454545	12.10267843	0.767259316	0.026126436	-	6429	grey	-0.026172184	FALSE	SRSF4
Region_398	chr1	29192657	29230942	38285	24	179	211.9242424	16.79680428	0.939698923	0.023637027	-	51102	grey	0.333245681	FALSE	MECR
Region_399	chr1	29236516	29326813	90297	33	209	253.8484848	20.60563211	0.743033667	0.020038275	+	10076	grey	-0.0306265	FALSE	PTPRU

Region_400	chr1	30711277	30723585	12308	4	17	26.31818182	5.41837072	0.280378885	0.044620247	-	4146	grey	-0.106202704	FALSE	MATN1
Region_401	chr1	30718504	30726827	8323	4	13	25.24242424	5.901917827	0.467525377	0.033563541	+	100129196	grey	0.0304011	FALSE	MATN1-AS1
Region_402	chr1	30732469	30757774	25305	7	35	51.65151515	6.599516123	0.402667826	0.050875722	-	7805	grey	0.057596018	FALSE	LAPTM5
Region_403	chr1	30869466	30908758	39292	11	61	89.03030303	9.844904495	0.819060184	0.043507437	-	9672	grey	-0.065753265	FALSE	SDC3
Region_404	chr1	30931506	31065991	134485	27	170	204.4242424	14.60880726	0.845546541	0.024349609	-	9698	grey	-0.325554141	FALSE	PUM1
Region_405	chr1	31179745	31239887	60142	27	177	210.0454545	16.25416568	0.745129419	0.027370925	-	79570	grey	-0.003393934	FALSE	NKAIN1
Region_406	chr1	31259568	31296788	37220	8	42	61.31818182	7.831217787	0.854492829	0.031668544	-	9410	grey	0.167110726	FALSE	SNRNP40
Region_407	chr1	31296982	31364953	67971	20	149	181.6969697	12.18065262	0.686790946	0.020067482	+	51538	grey	0.112604962	FALSE	ZCCHC17
Region_408	chr1	31365253	31376850	11597	3	15	26.8030303	4.691136313	0.327971477	0.0190452	-	2170	grey	0.186700608	FALSE	FABP3
Region_409	chr1	31409565	31434680	25115	9	45	64.13636364	8.076340305	0.689431785	0.037433735	+	347735	grey	-0.077013882	FALSE	SERINC2
Region_410	chr1	31500085	31583306	83221	31	238	273.5151515	17.67940342	0.807674259	0.022575703	+	149086	grey	-0.232801266	FALSE	LDC1P
Region_411	chr1	31506285	31526444	20159	9	68	94.96969697	11.26050921	0.814651806	0.039648855	+	284551	grey	-0.152607998	FALSE	LINC01226
Region_412	chr1	31571585	31577898	6313	3	11	20.2575757	4.75009507	0.93136687	0.05565322	-	10537862	grey	-0.09724973	FALSE	LOC105378626

							6	9	9			6		2		
Region_413	chr1	31576485	31587686	11201	4	17	29.98484848	6.008948261	0.779841849	0.087461764	+	64129	grey	-0.102337072	FALSE	TINAGL1
Region_414	chr1	31617686	31632518	14832	10	75	94.60606061	9.572201399	0.791457997	0.041371089	+	3061	grey	-0.341380904	FALSE	HCRTR1
Region_415	chr1	31629866	31644896	15030	12	96	112.6818182	9.535615975	0.562395175	0.015644805	-	553115	grey	-0.061460085	FALSE	PEF1
Region_416	chr1	31644049	31660162	16113	13	95	114.3636364	10.5752052	0.50705517	0.019725371	+	107985471	grey	0.064712934	FALSE	PEF1-AS1
Region_417	chr1	31652263	31704319	52056	17	94	114.5454545	11.08593262	0.774747982	0.031589901	-	1307	grey	-0.166211831	FALSE	COL16A1
Region_418	chr1	31727117	31764893	37776	14	84	105.0909091	10.02109663	0.796768998	0.033730678	-	576	grey	-0.009383467	FALSE	ADGRB2
Region_419	chr1	31790422	31816022	25600	4	30	42.1969697	6.791894937	0.772642809	0.058550917	-	90853	grey	0.2343855	FALSE	SPOCD1
Region_420	chr1	31906421	31944856	38435	11	68	89.10606061	8.904845333	0.339568389	0.032927155	-	8073	grey	0.163548758	FALSE	PTP4A2
Region_421	chr1	32013868	32060850	46982	20	131	158.7424242	13.80001182	0.816202555	0.016698874	+	10657	grey	-0.16240954	FALSE	KHDRBS1
Region_422	chr1	32072031	32102866	30835	18	103	130.0606061	12.32243581	0.884404883	0.027523609	+	55116	grey	-0.159584322	FALSE	TMEM39B
Region_423	chr1	32108056	32176563	68507	39	265	307.7272727	20.39120886	0.839814836	0.018976271	+	23633	grey	-0.198844625	FALSE	KPNA6
Region_424	chr1	32179675	32198285	18610	17	92	119.2121212	12.24055699	0.601201025	0.025760818	+	200081	grey	0.231938137	FALSE	TXLNA

Region_425	chr1	32200595	32205453	4858	7	26	40.3636363 6	6.59985166 2	0.77372701	0.04798482 8	+	79140	grey	0.098437438	FALSE	CCDC28B
Region_426	chr1	32205498	32222359	16861	10	61	82.3333333 3	10.8419886 5	0.34365495 9	0.03536360 4	-	56063	grey	0.367131131	FALSE	TMEM234
Region_427	chr1	32205671	32208682	3011	4	23	38.6969697	7.17253242 9	0.49643397 8	0.04319184 9	+	55721	grey	0.279293964	FALSE	IQCC
Region_428	chr1	32221077	32245397	24320	31	194	234.954545 5	17.0044626	0.72728027 3	0.01651614 3	+	8668	grey	0.261517003	FALSE	EIF3I
Region_429	chr1	32231656	32241710	10054	11	56	80.8181818 2	8.77392832 1	0.78246695 9	0.02392722 6	-	339483	grey	0.189719149	FALSE	MTMR9LP
Region_430	chr1	32251244	32286165	34921	22	143	181.333333 3	15.7659158 5	0.85344192 6	0.02631332 6	+	3932	grey	-0.04234616 5	FALSE	LCK
Region_431	chr1	32292083	32333635	41552	17	86	122.651515 2	11.8561797 2	0.86892583 9	0.02951816	+	3065	grey	0.015341523	FALSE	HDAC1
Region_432	chr1	32351521	32364312	12791	6	26	41.0909090 9	6.40847341 2	0.06154010 4	0.03726806 4	+	81629	grey	-0.07467422	FALSE	TSSK3
Region_433	chr1	32361270	32364278	3008	3	12	22.1969697	4.89966936 7	0.02974993 6	0.03871872	-	10012807 1	grey	0.091715862	FALSE	FAM229A
Region_434	chr1	32364633	32394731	30098	7	43	58.2424242 4	6.62874998 3	0.72381423 4	0.03320837 5	-	55108	grey	0.075175918	FALSE	BSDC1
Region_435	chr1	32465057	32605941	140884	71	497	553.030303	25.0838407 7	0.77329014 8	0.01526494	+	653121	grey	0.120125771	FALSE	ZBTB8A
Region_436	chr1	32465072	32496686	31614	14	93	115.166666 7	10.2620785 6	0.87373562 8	0.02984177 5	+	728116	grey	0.484665751	FALSE	ZBTB8B
Region_437	chr1	32600172	32650903	50731	20	137	158.242424	11.1507993	0.88128882	0.02103364	-	339487	grey	-0.20821404	FALSE	ZBTB8OS

							2	6	9					3		
Region_438	chr1	32651142	32686211	35069	16	101	120.484848<br>5	12.3627759<br>2	0.89528654<br>2	0.02652333<br>8	+	5928	grey	0.115838917	FALSE	RBBP4
Region_439	chr1	32679906	32703596	23690	16	96	121.136363<br>6	12.1501964<br>1	0.6957403	0.02587578<br>2	-	81493	grey	0.183391857	FALSE	SYNC
Region_440	chr1	32741830	32774970	33140	17	81	103.924242<br>4	10.1849219	0.75901358<br>9	0.03333276<br>4	+	57648	grey	0.288820488	FALSE	NHSL3
Region_441	chr1	32775237	32818031	42794	32	173	219.833333<br>3	19.337157	0.59086508	0.01842364<br>4	-	8565	grey	0.01425026	FALSE	YARS1
Region_442	chr1	32816767	32858875	42108	25	116	167.378787<br>9	15.2454433<br>6	0.50876684<br>1	0.01086700<br>2	+	64766	grey	-0.23846043<br>8	FALSE	S100PBP
Region_443	chr1	32862268	32872482	10214	6	30	41.9242424<br>2	6.33260331<br>9	0.52138447	0.04078506<br>3	-	252995	grey	0.118512719	FALSE	FNDC5
Region_444	chr1	32885994	32898441	12447	5	21	35.8181818<br>2	6.03810742<br>4	0.62256568<br>3	0.07781900<br>9	+	3208	grey	-0.25895164<br>3	FALSE	HPCA
Region_445	chr1	32936445	32964809	28364	8	55	71.4696969<br>7	7.92507160<br>3	0.67864955	0.04203394<br>6	-	127544	grey	0.097831139	FALSE	RNF19B
Region_446	chr1	33007986	33080996	73010	24	159	191.545454<br>5	15.0140260<br>8	0.69422838<br>9	0.03756439	-	204	grey	-0.41384338<br>5	FALSE	AK2
Region_447	chr1	33081104	33123492	42388	18	135	163.469697	12.1430191<br>4	0.78317286<br>9	0.02901982<br>1	+	113451	grey	-0.13780236<br>6	FALSE	AZIN2
Region_448	chr1	33145399	33182059	36660	27	191	227.575757<br>6	15.1708836<br>6	0.67801256<br>2	0.02606968<br>9	-	55223	grey	-0.0290485	FALSE	TRIM62
Region_449	chr1	33256492	33300719	44227	21	136	161.848484<br>8	12.5798892<br>3	0.81035593<br>7	0.01863734<br>5	+	149076	grey	-0.11699112<br>7	FALSE	ZNF362

Region_450	chr1	33306766	33321098	14332	8	49	78.98484848	11.53655384	0.526817552	0.037679852	-	127550	grey	0.014200971	FALSE	A3GALT2
Region_451	chr1	33323623	33431095	107472	51	335	374.0454545	20.53322397	0.579312995	0.017249236	-	1912	grey	-0.04963978	FALSE	PHC2
Region_452	chr1	33350352	33363245	12893	7	43	55.87878788	7.237753905	0.811255365	0.027316839	+	101929464	grey	0.237734053	FALSE	PHC2-AS1
Region_453	chr1	33472645	33501643	28998	8	39	62.72727273	9.127381383	0.966879472	0.027016101	+	7579	grey	-0.119693196	FALSE	ZSCAN20
Region_454	chr1	33513998	34165842	651844	142	1051	1122.166667	40.07054676	0.828499412	0.017429032	-	114784	grey	0.231390829	FALSE	CSMD2
Region_455	chr1	34166883	34219131	52248	11	78	96.42424242	9.326654287	0.760749657	0.036764773	+	84970	grey	-0.241423076	FALSE	C1orf94
Region_456	chr1	34640157	34685732	45575	6	31	46.33333333	7.892222722	0.83514652	0.075163341	-	105378641	grey	-0.084641333	FALSE	LOC105378641
Region_457	chr1	34712737	34859755	147018	20	108	142.2575758	13.52070845	0.620536792	0.030903797	-	113444	grey	-0.009573082	FALSE	SMIM12
Region_458	chr1	34865436	34929650	64214	12	69	91.42424242	9.633372452	0.546612546	0.050766365	-	58512	grey	-0.077677516	FALSE	DLGAP3
Region_459	chr1	34981533	35031945	50412	8	35	55.83333333	8.113774296	0.851453413	0.028572991	-	9204	grey	0.23919636	FALSE	ZMYM6
Region_460	chr1	35032172	35115859	83687	11	75	93.84848485	8.659796452	0.87798361	0.01918489	+	79830	grey	0.060402783	FALSE	ZMYM1
Region_461	chr1	35268709	35422058	153349	32	221	247.6666667	13.21459954	0.958596356	0.013772653	+	9202	grey	0.116051499	FALSE	ZMYM4
Region_462	chr1	35393883	35557950	164067	43	336	374.681818	17.5658401	0.82415387	0.01335012	-	79932	grey	0.13232024	FALSE	KIAA0319L

							2	8	4	9						
Region_463	chr1	35557473	35567274	9801	9	46	60.03030303	7.866129333	0.676651508	0.040706276	+	23154	grey	-0.117738983	FALSE	NCDN
Region_464	chr1	35569807	35577779	7972	4	14	25.78787879	4.662502304	0.920630349	0.064996818	-	105378645	grey	0.240057854	FALSE	TFAP2E-AS1
Region_465	chr1	35573314	35595328	22014	8	49	68.90909091	8.658881208	0.879831975	0.03780933	+	339488	grey	0.003651412	FALSE	TFAP2E
Region_466	chr1	35599541	35641526	41985	10	68	86.59090909	9.028122341	0.857543281	0.022180764	-	5690	grey	0.002683225	FALSE	PSMB2
Region_467	chr1	35720218	35769978	49760	16	80	114.9242424	13.46369546	0.658934897	0.019889969	-	63967	grey	-0.021815661	FALSE	CLSPN
Region_468	chr1	35808016	35857890	49874	11	58	78.42424242	9.29360509	0.750937282	0.034711814	+	192670	grey	0.173784239	FALSE	AGO4
Region_469	chr1	35869808	35930532	60724	15	90	110.7878788	10.05449024	0.797927357	0.025213448	+	26523	grey	0.246453972	FALSE	AGO1
Region_470	chr1	35930718	36072500	141782	22	150	173.2575758	13.7676439	0.926807976	0.012507091	+	192669	grey	0.107306929	FALSE	AGO3
Region_471	chr1	36084094	36088275	4181	3	24	32.28787879	4.559909417	0.729028063	0.071787969	+	27285	grey	-0.206467877	FALSE	TEKT2
Region_472	chr1	36095239	36125222	29983	17	109	126.8484848	8.638451471	0.684648162	0.044593733	-	1296	grey	-0.270258542	FALSE	COL8A2
Region_473	chr1	36136570	36156053	19483	7	42	61.42424242	9.138356368	0.550261991	0.023042749	-	27095	grey	-0.072642186	FALSE	TRAPPC3
Region_474	chr1	36155579	36180849	25270	8	31	42.53030303	5.210107623	0.731702646	0.040922944	+	55700	grey	-0.338703801	FALSE	MAP7D1

Region_475	chr1	36224432	36305357	80925	27	155	183.1363636	13.5406923 6	0.933205255	0.020854232	+	9967	grey	0.377081345	FALSE	THRAP3
Region_476	chr1	36306368	36329340	22972	20	97	133	12.35126901	0.386621874	0.022598573	+	79729	grey	0.080677225	FALSE	SH3D21
Region_477	chr1	36322030	36324154	2124	3	10	20.33333333	4.062334809	0.165510093	0.060080139	-	55194	grey	-0.054533534	FALSE	EVA1B
Region_478	chr1	36339624	36385924	46300	18	103	132.6969697	13.9581459	0.724816746	0.027180124	-	83931	grey	0.004981369	FALSE	STK40
Region_479	chr1	36391238	36397908	6670	6	31	45.43939394	7.542961646	0.895267979	0.073724044	-	84967	grey	-0.058317638	FALSE	LSM10
Region_480	chr1	36415827	36450451	34624	10	53	75.07575758	9.153915692	0.878042056	0.057705132	-	127700	grey	-0.11999802	FALSE	OSCP1
Region_481	chr1	36466043	36483278	17235	4	18	32.60606061	5.659243733	0.880532229	0.051233327	-	1441	grey	-0.148129358	FALSE	CSF3R
Region_482	chr1	36795527	37034515	238988	88	712	783.5	31.32325261	0.742144109	0.017641027	-	2899	grey	0.171304361	FALSE	GRIK3
Region_483	chr1	37350934	37474404	123470	22	160	193.969697	14.40616289	0.816909613	0.024898537	-	107984942	grey	0.020780754	FALSE	LOC107984942
Region_484	chr1	37454872	37474432	19560	5	32	44.96969697	6.560885721	0.77232422	0.030616999	-	728431	grey	0.11251237	FALSE	LITATS1
Region_485	chr1	37474580	37484377	9797	3	15	23.90909091	4.879241028	0.819199915	0.075236468	+	80149	grey	-0.165424455	FALSE	ZC3H12A
Region_486	chr1	37489993	37514766	24773	12	85	102.6060606	9.685641693	0.847178417	0.029933569	-	64769	grey	0.018485445	FALSE	MEAF6
Region_487	chr1	37534449	37554293	19844	11	72	88.5757575	9.21881121	0.96157527	0.02337700	-	79753	grey	-0.09919504	FALSE	SNIP1

							8	3	6	2				2		
Region_488	chr1	37556919	37566857	9938	4	20	32.6666666 7	5.75749233 2	0.78755171 6	0.08339703 8	+	7802	grey	-0.00251965 7	FALSE	DNALI1
Region_489	chr1	37566816	37595937	29121	8	47	62.9090909 1	7.85978522 5	0.96573014 8	0.02492646 8	-	29889	grey	-0.09577051 1	FALSE	GNL2
Region_490	chr1	37611350	37634892	23542	4	14	25.7121212 1	5.37162101 7	0.68134736 7	0.05340325 7	-	284654	grey	-0.00118580 9	FALSE	RSPO1
Region_491	chr1	37681570	37692249	10679	5	22	39.4242424 2	7.06684698 7	0.58510886 8	0.03065459 1	-	54955	grey	-0.31039652 1	FALSE	AIRIM
Region_492	chr1	37713880	37765133	51253	20	109	143.409090 9	13.0607777 7	0.67437440 8	0.03604300 5	-	284656	grey	-0.17490374 2	FALSE	EPHA10
Region_493	chr1	37793847	37801137	7290	5	15	35.6363636 4	8.11956111 7	0.06992316 6	0.04146334 8	+	149175	grey	0.162087814	FALSE	MANEAL
Region_494	chr1	37802945	37808208	5263	4	21	32.6666666 7	5.02710601 4	0.25093061	0.0270084	-	79693	grey	-0.21387573 3	FALSE	YRDC
Region_495	chr1	37806979	37809454	2475	4	21	28.9090909 1	4.74820540 3	0.00800901 2	0.01632409 4	+	127687	grey	-0.23589480 2	FALSE	C1orf122
Region_496	chr1	37809574	37859592	50018	14	92	116.636363 6	10.2801322 4	0.8725654	0.02331551 4	-	4520	grey	-0.00623047	FALSE	MTF1
Region_497	chr1	37860697	37947057	86360	29	171	218.151515 2	17.1179100 3	0.91941010 8	0.01895402 8	-	3633	grey	-0.29586583 6	FALSE	INPP5B
Region_498	chr1	37956975	37990075	33100	20	112	150.272727 3	16.1679126 2	0.81761764 1	0.01617070 8	-	10946	grey	0.111146724	FALSE	SF3A3
Region_499	chr1	38009258	38024820	15562	8	57	72.7575757 6	7.66823739 9	0.66118974 6	0.04468051 7	+	51118	grey	-0.02040062	FALSE	UTP11

Region_500	chr1	38043829	38046793	2964	6	26	39.25757576	5.583796905	0.041811372	0.0374376	-	5453	grey	0.015526922	FALSE	POU3F1
Region_501	chr1	38047314	38119025	71711	24	134	167.0151515	13.96753645	0.694391196	0.024812894	+	105378653	grey	-0.093066482	FALSE	MIR3659HG
Region_502	chr1	38149544	38210340	60796	14	89	112.3484848	11.06413808	0.922429231	0.023795748	+	105378654	grey	-0.060539322	FALSE	LOC105378654
Region_503	chr1	38474825	38516520	41695	20	121	154.469697	15.74432225	0.741473094	0.029324997	+	105378656	grey	-0.027504447	FALSE	LINC01685
Region_504	chr1	38838198	38874494	36296	7	44	60.28787879	8.030260368	0.656959081	0.046139025	-	64121	grey	0.090572495	FALSE	RRAGC
Region_505	chr1	38859912	38919526	59614	10	62	77.13636364	7.462493631	0.871395215	0.026169503	+	105378662	grey	0.195685737	FALSE	LOC105378662
Region_506	chr1	38885807	38941830	56023	12	75	97.1969697	9.103202643	0.962517132	0.023552924	-	54933	grey	0.241443049	FALSE	RHBDL2
Region_507	chr1	39081316	39487177	405861	84	629	680.8030303	29.0115201	0.794661693	0.012314673	+	23499	grey	0.05475689	FALSE	MACF1
Region_508	chr1	39491636	39529869	38233	26	120	156.0151515	15.7367697	0.446761895	0.036211179	+	353500	grey	0.20075934	FALSE	BMP8A
Region_509	chr1	39522280	39559698	37418	13	81	104.0454545	9.307452462	0.901182866	0.028177376	-	728448	grey	0.136792908	FALSE	PPIEL
Region_510	chr1	39560709	39576790	16081	7	58	74.87878788	7.956642414	0.956332764	0.024275907	-	8761	grey	-0.166569467	FALSE	PABPC4
Region_511	chr1	39565052	39573860	8808	5	39	51.42424242	6.414072524	0.952114352	0.032158185	+	101929516	grey	-0.150067014	FALSE	PABPC4-AS1
Region_512	chr1	39623435	39639643	16208	15	77	95.6212121	8.95246446	0.79963914	0.03531150	-	26508	grey	-0.07970288	FALSE	HEYL

							2	8	7	1				9		
Region_513	chr1	39651229	39672107	20878	5	15	29.57575758	6.755193116	0.949227978	0.054590024	-	84618	grey	0.15963682	FALSE	NT5C1A
Region_514	chr1	39678648	39691485	12837	4	13	20.43939394	4.648006315	0.714298696	0.086770851	-	51440	grey	0.258138881	FALSE	HPCAL4
Region_515	chr1	39692182	39763914	71732	14	87	113.5909091	10.41445338	0.779629297	0.032111982	+	10450	grey	-0.314909012	FALSE	PPIE
Region_516	chr1	39757182	39788865	31683	18	100	151.0909091	23.08027948	0.450894984	0.028857142	-	656	grey	0.07692704	FALSE	BMP8B
Region_517	chr1	39769523	39771348	1825	7	23	53.98484848	15.55782506	0.03149297	0.030807497	-	64064	grey	-0.166765473	FALSE	OXCT2
Region_518	chr1	39799422	39809450	10028	4	12	22.93939394	5.056233891	0.961189274	0.041804519	+	105378951	grey	-0.072041094	FALSE	
Region_519	chr1	39801414	39817460	16046	4	12	22.93939394	5.056233891	0.961189274	0.041804519	-	107985740	grey	-0.072041094	FALSE	LINC02811
Region_520	chr1	39838110	39883511	45401	10	60	81.81818182	8.457875876	0.955875543	0.025179003	-	54802	grey	-0.052958521	FALSE	TRIT1
Region_521	chr1	39955112	39969968	14856	10	43	65.24242424	8.401576054	0.705490195	0.04592667	+	84879	grey	0.098998634	FALSE	MFSD2A
Region_522	chr1	40040233	40072649	32416	28	163	201.0909091	15.04631544	0.593019306	0.032439041	+	10487	grey	0.010708105	FALSE	CAP1
Region_523	chr1	40072710	40097260	24550	11	60	88.77272727	12.4650841	0.69968793	0.027359666	-	5538	grey	-0.0416173	FALSE	PPT1
Region_524	chr1	40161387	40240921	79534	26	179	211.8333333	13.97204168	0.883707806	0.018063618	+	6018	grey	-0.226860414	FALSE	RLF

Region_525	chr1	40258041	40294180	36139	22	126	159.3787879	15.80240016	0.875085523	0.018747531	+	10269	grey	-0.056909997	FALSE	ZMPSTE24
Region_526	chr1	40300489	40317813	17324	6	26	41.48484848	6.828092254	0.623355708	0.084202596	-	1298	grey	0.152550016	FALSE	COL9A2
Region_527	chr1	40344850	40423326	78476	28	189	227.0151515	16.48355117	0.817475889	0.025936494	+	64744	grey	-0.169107231	FALSE	SMAP2
Region_528	chr1	40450102	40463718	13616	6	25	37.36363636	6.146008996	0.517760135	0.05072094	+	65243	grey	0.069060974	FALSE	ZFP69B
Region_529	chr1	40477290	40496343	19053	4	24	34.54545455	5.335459949	0.743517956	0.023775817	+	339559	grey	0.125548723	FALSE	ZFP69
Region_530	chr1	40493157	40508661	15504	3	13	22.87878788	4.72477969	0.662077863	0.025751814	-	105378672	grey	0.080466559	FALSE	EXO5-DT
Region_531	chr1	40508741	40516038	7297	4	28	40.28787879	5.771988463	0.492576108	0.015601189	+	64789	grey	0.008145879	FALSE	EXO5
Region_532	chr1	40531573	40548167	16594	3	15	23.27272727	4.269914265	0.938974248	0.063027095	+	127396	grey	-0.285057129	FALSE	ZNF684
Region_533	chr1	40620680	40665682	45002	12	63	86.71212121	11.56488738	0.866885008	0.042722028	-	9783	grey	-0.180382595	FALSE	RIMS3
Region_534	chr1	40669089	40687588	18499	6	24	39.77272727	7.511622696	0.956082863	0.035539747	-	105378674	grey	0.077922616	FALSE	
Region_535	chr1	40690380	40692074	1694	5	10	24.72727273	5.41094462	0.006698293	0.017097981	-	100130557	grey	-0.033542411	FALSE	NFYC-AS1
Region_536	chr1	40691648	40771603	79955	20	108	142.1363636	15.45105061	0.755412606	0.025056898	+	4802	grey	-0.042368542	FALSE	NFYC
Region_537	chr1	40783787	40840452	56665	29	155	191.878787	15.2965709	0.61780974	0.02230317	+	9132	grey	0.056704819	FALSE	KCNQ4

							9	1	9	3						
Region_538	chr1	40979300	41012565	33265	12	77	103.3181818	9.779348871	0.597510873	0.033786361	+	1503	grey	0.18434347	FALSE	CTPS1
Region_539	chr1	41014590	41043890	29300	10	58	76.21212121	8.917597385	0.919696905	0.036345258	+	100507178	grey	0.068382721	FALSE	SLFNL1-AS1
Region_540	chr1	41015589	41023237	7648	8	39	60.22727273	7.603228979	0.913672921	0.036770587	-	200172	grey	0.140531581	FALSE	SLFNL1
Region_541	chr1	41027202	41242306	215104	31	228	262	14.71367757	0.908569035	0.017454679	-	22955	grey	0.007097045	FALSE	SCMH1
Region_542	chr1	41478775	41484683	5908	5	25	39.72727273	6.643783571	0.858382532	0.06133179	-	1907	grey	-0.183120674	FALSE	EDN2
Region_543	chr1	41506365	42035925	529560	93	705	776.7424242	30.92189197	0.810753789	0.015473481	-	59269	grey	-0.107908904	FALSE	HIVEP3
Region_544	chr1	42176539	42335877	159338	20	127	155.1818182	12.7483442	0.833529582	0.024156623	-	22887	grey	0.231479958	FALSE	FOXJ3
Region_545	chr1	42380792	42424232	43440	14	84	117.8333333	11.17770216	0.908974265	0.032359444	+	284716	grey	0.09977511	FALSE	RIMKLA
Region_546	chr1	42430329	42456253	25924	6	31	40.63636364	5.267129896	0.934043608	0.056392144	-	84217	grey	0.130364245	FALSE	ZMYND12
Region_547	chr1	42463221	42657190	193969	31	188	228.4545455	16.75450049	0.957106859	0.014322484	+	728621	grey	0.140426883	FALSE	CCDC30
Region_548	chr1	42682418	42703805	21387	8	48	65.5	8.374411575	0.606898667	0.052020916	+	4904	grey	0.121069993	FALSE	YBX1
Region_549	chr1	42767245	42794493	27248	6	40	55.78787879	7.267451466	0.56137002	0.046068593	+	79078	grey	0.171936407	FALSE	C1orf50

Region_550	chr1	42784991	42816619	31628	7	46	62.8030303	8.367004237	0.949725916	0.023691677	+	100129924	grey	0.104598363	FALSE	TMEM269
Region_551	chr1	42846573	42852477	5904	9	31	69.13636364	13.05006497	0.110109335	0.013763504	+	51058	grey	-0.139464822	FALSE	ZNF691
Region_552	chr1	42925353	42958893	33540	13	83	102.3636364	11.1239677	0.716025878	0.043610998	-	6513	grey	-0.290375832	FALSE	SLC2A1
Region_553	chr1	42959049	42996814	37765	18	107	145.469697	12.93084529	0.739961514	0.025631942	+	440584	grey	-0.52747927	FALSE	SLC2A1-DT
Region_554	chr1	43145153	43156396	11243	10	67	93.09090909	10.70402839	0.44310732	0.042852888	+	440585	grey	-0.141070903	FALSE	CFAP144
Region_555	chr1	43164175	43270936	106761	40	253	296.7878788	17.27382649	0.816306458	0.019349399	-	10969	grey	0.065359443	FALSE	EBNA1BP2
Region_556	chr1	43172330	43254358	82028	33	206	246.0757576	15.87965375	0.786169517	0.022013112	+	149465	grey	0.063457849	FALSE	CFAP57
Region_557	chr1	43181682	43261910	80228	32	191	235.469697	16.82326201	0.810270728	0.022439057	-	105378685	grey	0.015445329	FALSE	LOC105378685
Region_558	chr1	43300982	43323108	22126	10	52	69.16666667	7.761013679	0.932169527	0.037723538	+	7075	grey	-0.056601741	FALSE	TIE1
Region_559	chr1	43337818	43354466	16648	9	41	64.6969697	8.120192679	0.85429546	0.054658241	+	4352	grey	-0.09290172	FALSE	MPL
Region_560	chr1	43389882	43454247	64365	42	307	354.6060606	23.12566259	0.773125906	0.015405408	+	23334	grey	-0.040406451	FALSE	SZT2
Region_561	chr1	43525187	43623666	98479	46	323	364.530303	21.81969449	0.822244396	0.016675703	+	5792	grey	-0.007660763	FALSE	PTPRF
Region_562	chr1	43650149	43931014	280865	78	564	629.848484	23.1584790	0.89783987	0.01228470	+	9682	grey	0.005788656	FALSE	KDM4A

							8	4	1	2						
Region_563	chr1	43685123	43708138	23015	9	49	69.5	8.73234482 6	0.73081655 7	0.02564449 9	-	10013277 4	grey	0.236679059	FALSE	KDM4A-AS1
Region_564	chr1	43705824	43931165	225341	59	436	490.590909 1	20.9019522 6	0.87784327 4	0.01436953 4	+	6487	grey	-0.07635548 9	FALSE	ST3GAL3
Region_565	chr1	43946950	43968022	21072	3	11	20.2878787 9	4.61023994 5	0.97339229 2	0.03546868 6	+	9670	grey	-0.08553927 3	FALSE	IPO13
Region_566	chr1	43970000	43973369	3369	3	10	19.6666666 7	4.34770791 4	0.65575828 5	0.03090055	+	1802	grey	0.257857706	FALSE	DPH2
Region_567	chr1	43978943	43991170	12227	4	15	28.0454545 5	5.62256940 5	0.97738524 1	0.02925731 4	+	8704	grey	0.199103294	FALSE	B4GALT2
Region_568	chr1	43991359	43996528	5169	4	17	31.4242424 2	6.11696406 9	0.44861665 1	0.04244696	+	149473	grey	-0.16785941 5	FALSE	CCDC24
Region_569	chr1	43991500	44031467	39967	39	234	276.303030 3	18.1421478 6	0.67858972 1	0.01520375	-	6536	grey	-0.04597731 1	FALSE	SLC6A9
Region_570	chr1	44030414	44041561	11147	11	54	74.5909090 9	9.97840325 2	0.29342898 5	0.02422155 1	+	10798494 8	grey	-0.06425436 8	FALSE	LOC107984948
Region_571	chr1	44036126	44135140	99014	41	256	316.878787 9	23.7114157 2	0.88709717 7	0.02042345 1	+	128209	grey	-0.40009867 3	FALSE	KLF17
Region_572	chr1	44221070	44355260	134190	25	165	204.015151 5	14.7037330 9	0.86664927 5	0.02504937	-	79033	grey	-0.01384745 1	FALSE	ERI3
Region_573	chr1	44405194	44651724	246530	100	687	780	35.4092556 3	0.66783663 5	0.01561006 3	+	55182	grey	-0.09216579 1	FALSE	RNF220
Region_574	chr1	44412698	44419872	7174	7	46	61.9242424 2	7.87070636 3	0.29531276 3	0.07315161 7	+	10798495 1	grey	-0.2524367	FALSE	

Region_575	chr1	44635238	44674477	39239	13	60	87.72727273	9.568450489	0.865718023	0.028278989	-	107984950	grey	-0.141591729	FALSE	LOC107984950
Region_576	chr1	44653247	44674481	21234	7	28	44.37878788	7.174238424	0.791235488	0.050109321	-	79639	grey	-0.050562618	FALSE	TMEM53
Region_577	chr1	44674692	44725591	50899	19	122	148.2575758	11.95158259	0.888255387	0.027299905	+	339541	grey	-0.183944574	FALSE	ARMH1
Region_578	chr1	44739818	44767767	27949	5	28	36.62121212	5.549123705	0.775037105	0.031131987	+	11004	grey	0.1599319	FALSE	KIF2C
Region_579	chr1	44783585	44788170	4585	3	10	19.18181818	4.332024188	0.039656906	0.045834795	-	266675	grey	-0.070494157	FALSE	BEST4
Region_580	chr1	44800377	44805990	5613	4	14	25.56060606	5.404069088	0.441791577	0.057363352	+	1263	grey	0.170050702	FALSE	PLK3
Region_581	chr1	44819844	44843253	23409	13	74	90.34848485	8.889912043	0.603035516	0.041503198	-	8643	grey	-0.138303538	FALSE	PTCH2
Region_582	chr1	44850522	44986722	136200	37	259	289.9393939	16.61035709	0.929026729	0.015690754	-	8891	grey	-0.0608433	FALSE	EIF2B3
Region_583	chr1	45002540	45011324	8784	7	38	52.01515152	7.177941475	0.696387056	0.019994785	-	79654	grey	-0.02877899	FALSE	HECTD3
Region_584	chr1	45016399	45306209	289810	95	703	773.3333333	28.98399205	0.787225487	0.011589045	-	57643	grey	-0.227048196	FALSE	ZSWIM5
Region_585	chr1	45303910	45305619	1709	5	26	40.48484848	4.78537009	0.204086662	0.062826342	+	400752	grey	-0.091919676	FALSE	LINC01144
Region_586	chr1	45329163	45491155	161992	53	341	385.0151515	18.03372262	0.886431031	0.017031651	-	4595	grey	-0.070425156	FALSE	MUTYH
Region_587	chr1	45343883	45491163	147280	49	310	354.454545	18.8207592	0.92822844	0.01659572	-	10420	grey	-0.02434568	FALSE	TESK2

							5	6	9	9				5		
Region_588	chr1	45500300	45513382	13082	6	31	44.54545455	7.213623475	0.760030797	0.066992393	+	25974	grey	-0.15098945	FALSE	MMACHC
Region_589	chr1	45510914	45542732	31818	6	34	45.46969697	6.69378517	0.770778943	0.057680453	-	5052	grey	-0.249640441	FALSE	PRDX1
Region_590	chr1	45550543	45570049	19506	7	38	57.56060606	7.662353099	0.56141246	0.027642946	+	10327	grey	0.325583631	FALSE	AKR1A1
Region_591	chr1	45583846	45618904	35058	6	38	52.75757576	6.568129592	0.659902552	0.026967967	+	4678	grey	-0.057149734	FALSE	NASP
Region_592	chr1	45620044	45624057	4013	3	15	24.53030303	5.23955282	0.935068341	0.053881972	-	149483	grey	0.11581102	FALSE	CCDC17
Region_593	chr1	45627304	45688113	60809	12	68	90.68181818	10.03096604	0.866773005	0.032637753	-	60313	grey	0.071615203	FALSE	GPBP1L1
Region_594	chr1	45688181	45694436	6255	5	25	35.77272727	5.758727035	0.475858027	0.091524606	+	51249	grey	-0.278100149	FALSE	TMEM69
Region_595	chr1	45694324	45750653	56329	8	45	63.16666667	8.864073266	0.740753058	0.018946687	-	3652	grey	-0.131086687	FALSE	IPP
Region_596	chr1	45786987	46036122	249135	68	502	565.2272727	23.38296254	0.946857333	0.010061151	+	23139	grey	-0.305243585	FALSE	MAST2
Region_597	chr1	46040140	46133036	92896	32	186	231.969697	17.97260282	0.892326217	0.018393233	-	8503	grey	-0.018886201	FALSE	PIK3R3
Region_598	chr1	46043661	46176488	132827	49	309	355.8181818	21.13612226	0.836075049	0.015180667	-	110117499	grey	-0.061904221	FALSE	P3R3URF-PIK3R3
Region_599	chr1	46188683	46220305	31622	23	130	171.4393939	14.27114225	0.875414836	0.027842177	-	55624	grey	-0.228274504	FALSE	POMGNT1

Region_600	chr1	46203334	46221256	17922	12	65	89.6969697	9.634824172	0.836308483	0.032356933	+	541468	grey	-0.198644136	FALSE	LURAP1
Region_601	chr1	46246461	46278480	32019	12	70	91.90909091	10.13861965	0.732196285	0.024879041	+	8438	grey	0.077723514	FALSE	RAD54L
Region_602	chr1	46261196	46303616	42420	14	90	109.4848485	10.05560299	0.760464467	0.017182629	-	10489	grey	0.036698142	FALSE	LRRC41
Region_603	chr1	46303698	46316776	13078	9	60	79.36363636	10.05314549	0.76364487	0.033069985	+	7388	grey	0.357639435	FALSE	UQCRH
Region_604	chr1	46340789	46365152	24363	11	60	87.15151515	9.754277049	0.539903524	0.059633401	+	387338	grey	0.055142953	FALSE	NSUN4
Region_605	chr1	46394317	46413848	19531	8	25	44.57575758	6.60084052	0.719421649	0.047575965	+	2166	grey	-0.014086309	FALSE	FAAH
Region_606	chr1	46432129	46482493	50364	15	72	104.7424242	9.371496237	0.706995666	0.043904725	+	729041	grey	0.041538375	FALSE	FAAHP1
Region_607	chr1	46489836	46516216	26380	11	61	86.45454545	9.02506223	0.715366387	0.039900933	+	127343	grey	-0.0351013	FALSE	DMBX1
Region_608	chr1	46538611	46570255	31644	9	58	71.51515152	6.90427387	0.794666284	0.044503531	+	100507423	grey	-0.135234501	FALSE	MKNK1-AS1
Region_609	chr1	46557407	46616843	59436	26	196	230.530303	16.23502358	0.809282798	0.013167731	-	8569	grey	-0.07397004	FALSE	MKNK1
Region_610	chr1	46607719	46616811	9092	9	57	78.36363636	9.406440287	0.630998856	0.024866943	-	148932	grey	-0.022170361	FALSE	MOB3C
Region_611	chr1	46632737	46673867	41130	9	50	66.10606061	7.889874305	0.72188674	0.043515295	-	64756	grey	-0.213536183	FALSE	ATPAF1
Region_612	chr1	46674659	46719146	44487	10	53	72.3484848	9.52162304	0.66639974	0.04523512	-	9813	grey	-0.03764007	FALSE	EFCAB14

							5	2	3	8				2		
Region_613	chr1	46757838	46810850	53012	14	82	111.7727273	10.30577949	0.908131797	0.027774694	+	105378699	grey	0.15230803	FALSE	LOC105378699
Region_614	chr1	46798998	46819413	20415	6	32	45.5	6.703041789	0.862673368	0.052615738	+	1580	grey	0.175416146	FALSE	CYP4B1
Region_615	chr1	46843095	46900437	57342	9	54	72.46969697	7.672567912	0.889507087	0.03699901	-	163720	grey	0.350621483	FALSE	CYP4Z2P
Region_616	chr1	46929177	46941484	12307	4	15	23.15151515	4.403643332	0.832338316	0.086508114	-	1579	grey	0.120458458	FALSE	CYP4A11
Region_617	chr1	47023669	47050751	27082	5	24	37.03030303	6.159344863	0.627295001	0.102039472	+	260293	grey	0.134410815	FALSE	CYP4X1
Region_618	chr1	47067231	47118318	51087	6	30	50.36363636	7.470220599	0.871530788	0.053124844	+	199974	grey	0.017794248	FALSE	CYP4Z1
Region_619	chr1	47250139	47314892	64753	10	58	88.03030303	10.31350078	0.587245482	0.015750917	-	6491	grey	-0.159739899	FALSE	STIL
Region_620	chr1	47333790	47394866	61076	8	47	61.96969697	6.836485145	0.952790561	0.028897524	+	51727	grey	-0.071215008	FALSE	CMPK1
Region_621	chr1	47380928	47408477	27549	4	21	33.83333333	5.512456756	0.843559259	0.066593886	-	102724077	grey	0.155379012	FALSE	LINC01389
Region_622	chr1	47432133	47434641	2508	3	15	22.3030303	3.683517119	0.108719703	0.080708507	-	84793	grey	0.016824146	FALSE	FOXD2-AS1
Region_623	chr1	47688463	47704029	15566	9	42	60.1969697	8.317210511	0.91599165	0.04570184	+	107984953	grey	0.066405842	FALSE	LINC01738
Region_624	chr1	47760528	47997385	236857	73	555	610.4393939	31.18585912	0.872315166	0.017970582	-	388630	grey	0.105737735	FALSE	TRABD2B

Region_625	chr1	48050659	48091736	41077	4	16	27.6969697	5.883404815	0.839728289	0.080799913	+	105379778	grey	0.116607686	FALSE	LINC02794
Region_626	chr1	48096092	48182428	86336	14	74	99.5	9.793640008	0.805798551	0.044516298	-	391037	grey	-0.288665817	FALSE	SKINT1L
Region_627	chr1	48295373	48472208	176835	19	137	169.1818182	14.77930892	0.882312542	0.035394321	-	54558	grey	0.041552465	FALSE	SPATA6
Region_628	chr1	48532854	50023954	1491100	106	862	934.3030303	34.40884041	0.83094911	0.021054203	-	84871	grey	-0.001053299	FALSE	AGBL4
Region_629	chr1	48727519	48776969	49450	6	30	45.42424242	6.802851452	0.642345302	0.049031768	-	79656	grey	-0.006270215	FALSE	BEND5
Region_630	chr1	49025595	49187585	161990	8	50	66.03030303	8.849110685	0.843303935	0.063987637	+	105378706	grey	-0.052488835	FALSE	LOC105378706
Region_631	chr1	49374201	49472085	97884	5	24	39.87878788	6.733418726	0.82874142	0.070505803	-	100874313	grey	-0.180336036	FALSE	AGBL4-IT1
Region_632	chr1	50024029	50203772	179743	12	76	96.21212121	9.982315365	0.76236781	0.029023499	+	1996	grey	-0.158074543	FALSE	ELAVL4
Region_633	chr1	50206084	50321161	115077	7	43	63.18181818	7.701965965	0.58630647	0.054651152	-	105378711	grey	0.067528017	FALSE	LOC105378711
Region_634	chr1	50265742	50321192	55450	3	18	28.96969697	5.092890057	0.430134212	0.075472654	-	105378712	grey	0.036766321	FALSE	LINC02808
Region_635	chr1	50437028	50960267	523239	68	493	541.6969697	27.34000714	0.949398265	0.009560438	-	11124	grey	0.149589849	FALSE	FAF1
Region_636	chr1	51102221	51148086	45865	8	47	65.72727273	7.70922615	0.877083772	0.042308761	+	284546	grey	0.104929848	FALSE	C1orf185
Region_637	chr1	51236273	51273447	37174	4	27	39.6666666	6.79441701	0.94817827	0.03812296	+	26994	grey	-0.32045132	FALSE	RNF11

							7	4	7	6				2		
Region_638	chr1	51287258	51345116	57858	9	50	64.86363636	8.701699858	0.800473831	0.044120816	-	22996	grey	-0.01994056	FALSE	TTC39A
Region_639	chr1	51354263	51519266	165003	36	244	281.2272727	14.82180873	0.840844547	0.019359946	-	2060	grey	-0.073515287	FALSE	EPS15
Region_640	chr1	51518288	51561651	43363	3	12	22.42424242	4.826403681	0.681845327	0.086942022	+	105378720	grey	0.158786307	FALSE	EPS15-AS1
Region_641	chr1	51561866	51594897	33031	7	36	52.86363636	6.943478099	0.974461292	0.027714072	-	441884	grey	0.161827993	FALSE	CALR4P
Region_642	chr1	51577179	51789219	212040	40	270	309.2121212	18.02008721	0.870099288	0.01506572	+	114883	grey	0.074468465	FALSE	OSBPL9
Region_643	chr1	51784575	51799154	14579	8	63	77.96969697	8.028736268	0.907985678	0.035303252	+	105378721	grey	0.218797128	FALSE	
Region_644	chr1	51789191	51878805	89614	21	155	180.4848485	12.69432632	0.916469891	0.023291465	-	4898	grey	-0.229877417	FALSE	NRDC
Region_645	chr1	51907956	51990700	82744	10	64	83.22727273	7.749531906	0.911027275	0.050453499	-	5865	grey	0.157960649	FALSE	RAB3B
Region_646	chr1	52020131	52055191	35060	9	59	76.75757576	10.35977518	0.692525846	0.057674569	-	51060	grey	0.1439641	FALSE	TXNDC12
Region_647	chr1	52056199	52090716	34517	12	72	93.89393939	11.50802902	0.611989234	0.027535442	+	91408	grey	0.062232789	FALSE	BTF3L4
Region_648	chr1	52142089	52348671	206582	44	328	362.7575758	18.53733082	0.930231486	0.015839517	+	9372	grey	0.085940186	FALSE	ZFYVE9
Region_649	chr1	52345723	52366205	20482	4	21	31.40909091	5.294343201	0.709210928	0.030728984	-	200014	grey	0.38424725	FALSE	CC2D1B

Region_650	chr1	52372829	52404423	31594	8	44	71.6060606 1	10.5653698 3	0.85324561	0.01713580 1	-	4998	grey	0.057692846	FALSE	ORC1
Region_651	chr1	52408282	52553487	145205	35	273	308.242424 2	16.6696966 9	0.97243131 5	0.01016673 7	-	23318	grey	0.003431113	FALSE	TUT4
Region_652	chr1	52602371	52609051	6680	10	53	70.5151515 2	10.0601918 1	0.74032968 5	0.03542791 4	+	2882	grey	-0.03422146 2	FALSE	GPX7
Region_653	chr1	52633168	52669683	36515	4	22	33.2121212 1	5.75797814 8	0.84824585 1	0.06040937 1	+	348378	grey	0.100182552	FALSE	SHISAL2A
Region_654	chr1	52726453	52827336	100883	27	179	218.121212 1	15.7338587 9	0.96154561	0.01296079 1	+	79699	grey	-0.27165349	FALSE	ZYG11B
Region_655	chr1	52842511	52894998	52487	16	90	118.545454 5	11.2867271 5	0.87350826 8	0.02719965 1	+	440590	grey	-0.03616534 1	FALSE	ZYG11A
Region_656	chr1	52895910	52927212	31302	7	48	66.1969697	7.71456105 1	0.51105393 3	0.03033758 4	-	55268	grey	-0.11398702 8	FALSE	ECHDC2
Region_657	chr1	52927276	53051698	124422	19	115	146.621212 1	13.3390867 5	0.89608218 4	0.02674906 3	+	6342	grey	0.086741657	FALSE	SCP2
Region_658	chr1	53062052	53085501	23449	13	72	95.2121212 1	8.93827577 9	0.74475391 7	0.03580475 1	+	127435	grey	-0.20886663	FALSE	PODN
Region_659	chr1	53087179	53142638	55459	30	158	207.045454 5	21.4228000 9	0.89146580 6	0.02234463 6	-	6512	grey	0.20602057	FALSE	SLC1A7
Region_660	chr1	53196792	53214197	17405	4	20	30.7272727 3	5.58444392 8	0.73184059 6	0.04362429 9	+	1376	grey	-0.02868796	FALSE	CPT2
Region_661	chr1	53226900	53238518	11618	7	31	48.2424242 4	6.47851028 9	0.56884590 7	0.05109967	-	4116	grey	0.022991434	FALSE	MAGOH
Region_662	chr1	53242364	53328469	86105	41	283	330.818181	19.7755942	0.79204257	0.02139838	-	7804	grey	0.314240633	FALSE	LRP8

							8		3	1						
Region_663	chr1	53328233	53336509	8276	4	23	35.71212121	5.763986739	0.78967535	0.077153734	+	105378732	grey	-0.358931976	FALSE	LRP8-DT
Region_664	chr1	53413149	53440020	26871	6	30	49.24242424	8.385079434	0.89695596	0.051478315	-	163742	grey	0.174254012	FALSE	SLC25A3P1
Region_665	chr1	53459399	53467488	8089	6	36	47.13636364	6.275437744	0.951415331	0.040331909	+	63948	grey	0.132907557	FALSE	DMRTB1
Region_666	chr1	53506237	53739164	232927	83	604	675	29.87409477	0.831829806	0.014779049	-	148979	grey	-0.001108632	FALSE	GLIS1
Region_667	chr1	53765478	53838463	72985	29	190	221.8787879	15.01334294	0.910326167	0.015084416	-	55706	grey	-0.00502617	FALSE	NDC1
Region_668	chr1	53851719	53889798	38079	9	57	73.96969697	7.538556681	0.776397547	0.031485283	-	54432	grey	0.272049694	FALSE	YIPF1
Region_669	chr1	53891239	53911086	19847	5	35	50.95454545	7.124026127	0.909072073	0.047415398	+	1733	grey	0.012572227	FALSE	DIO1
Region_670	chr1	53916574	53945699	29125	7	33	48.6969697	6.600628634	0.800151526	0.051888357	-	51668	grey	-0.029063806	FALSE	IFT25
Region_671	chr1	53946085	53968168	22083	10	56	77.12121212	9.781481957	0.959123302	0.025661603	+	115353	grey	0.365176051	FALSE	LRRC42
Region_672	chr1	54007298	54018186	10888	7	50	63.68181818	7.565425589	0.956474035	0.024047623	-	388633	grey	-0.060360913	FALSE	LDLRAD1
Region_673	chr1	54053584	54112519	58935	15	82	122.7727273	12.60988069	0.904405714	0.017749302	+	127428	grey	0.285482022	FALSE	TCEANC2
Region_674	chr1	54132686	54196433	63747	15	102	129.1212121	11.89895296	0.93504248	0.027491876	-	200008	grey	-0.042607834	FALSE	CDCP2

Region_675	chr1	54143392	54200073	56681	13	90	110.7272727	10.31585107	0.866196965	0.029480186	-	606495	grey	-0.145469055	FALSE	CYB5RL
Region_676	chr1	54184041	54225464	41423	12	93	114.0909091	10.40669499	0.700482314	0.029205808	+	51253	grey	-0.193502014	FALSE	MRPL37
Region_677	chr1	54225433	54413479	188046	74	530	623.6818182	34.67304832	0.791918897	0.016042808	-	23648	grey	-0.165646199	FALSE	SSBP3
Region_678	chr1	54542257	54639192	96935	49	325	375.0909091	21.16363156	0.908233354	0.022926482	+	26027	grey	0.209391883	FALSE	ACOT11
Region_679	chr1	54609181	54623556	14375	11	73	95.92424242	10.21207521	0.864933936	0.0492676	-	338094	grey	0.144608212	FALSE	FAM151A
Region_680	chr1	54641754	54742308	100554	30	219	260.3939394	15.68622991	0.917877236	0.015042249	+	100527960	grey	0.193547249	FALSE	MROH7-TTC4
Region_681	chr1	54641786	54710266	68480	18	128	159.3333333	12.01324056	0.93831687	0.021356129	+	374977	grey	0.168006633	FALSE	MROH7
Region_682	chr1	54715861	54742657	26796	12	82	99.96969697	8.767018083	0.963694936	0.018665634	+	7268	grey	0.258491807	FALSE	TTC4
Region_683	chr1	54756898	54764523	7625	10	58	82.5	10.50457776	0.49401602	0.027741855	-	25973	grey	-0.141808025	FALSE	PARS2
Region_684	chr1	54779712	54801323	21611	12	75	101.1363636	10.75587333	0.93011286	0.03417701	-	55001	grey	0.203485184	FALSE	TTC22
Region_685	chr1	54806063	54842252	36189	11	70	91.22727273	10.65803482	0.879993137	0.045904654	+	163747	grey	0.009696757	FALSE	CIMAP2
Region_686	chr1	54849627	54887195	37568	18	127	163.2575758	12.18739652	0.900812655	0.015850815	-	1718	grey	-0.285296128	FALSE	DHCR24
Region_687	chr1	54998933	55017172	18239	6	31	48.3787878	7.38556836	0.91729005	0.04266986	+	7809	grey	-0.18637361	FALSE	BSND

							8	3	3	9				3		
Region_688	chr1	55039447	55064852	25405	15	100	121.1515152	9.277337855	0.552945697	0.035035547	+	255738	grey	0.374576712	FALSE	PCSK9
Region_689	chr1	55066359	55215364	149005	36	251	310.8787879	21.57813363	0.852587224	0.017356544	-	23358	grey	-0.022688757	FALSE	USP24
Region_690	chr1	55217645	55324691	107046	31	232	263.6363636	14.73419504	0.868231735	0.017056008	+	109729135	grey	0.034301871	FALSE	MIR4422HG
Region_691	chr1	55823816	56070513	246697	12	85	109.5	10.92245394	0.911730379	0.038340152	+	105378737	grey	0.260529758	FALSE	LOC105378737
Region_692	chr1	55868254	55950119	81865	7	46	60.13636364	7.66588118	0.914340828	0.043607732	+	105378738	grey	0.167952702	FALSE	LINC01755
Region_693	chr1	56173433	56645301	471868	42	327	362.2575758	17.39081155	0.734857439	0.015478789	-	8613	grey	0.007060208	FALSE	PLPP3
Region_694	chr1	56579168	56645272	66104	6	44	57.6969697	6.194214911	0.644128709	0.027480919	-	101929935	grey	-0.176913111	FALSE	LOC101929935
Region_695	chr1	56645314	56715335	70021	3	17	26.78787879	4.36952788	0.739532781	0.137755882	+	5563	grey	0.284947217	FALSE	PRKAA2
Region_696	chr1	56718789	56819696	100907	8	44	64.71212121	7.534056322	0.832470607	0.054040814	-	199920	grey	0.049415771	FALSE	FYB2
Region_697	chr1	56854768	56918223	63455	3	18	29.48484848	5.135824177	0.835785818	0.082598869	+	731	grey	0.178929796	FALSE	C8A
Region_698	chr1	56929207	56974383	45176	5	26	41.18181818	6.916889232	0.768288137	0.07972759	-	732	grey	0.053617033	FALSE	C8B
Region_699	chr1	56994778	58546734	1551956	153	1219	1299.318182	42.7440177	0.846375886	0.015110473	-	1600	grey	0.035065875	FALSE	DAB1

Region_700	chr1	58415384	58546802	131418	17	114	139.1060606	11.44502416	0.960362798	0.022165757	-	115209	grey	-0.101867387	FALSE	OMA1
Region_701	chr1	58643440	58700090	56650	5	26	35.84848485	5.62752145	0.77699098	0.034571894	-	114803	grey	0.056019763	FALSE	MYSM1
Region_702	chr1	58715609	58759623	44014	7	44	61.25757576	7.067391219	0.812503062	0.046646306	+	112268263	grey	0.320802629	FALSE	LOC112268263
Region_703	chr1	58718420	58771295	52875	8	54	70.45454545	7.61007797	0.834799557	0.041864267	+	105378751	grey	0.347034666	FALSE	LOC105378751
Region_704	chr1	58785128	58901109	115981	9	61	78.98484848	8.646017179	0.737299122	0.038922659	+	100131060	grey	0.157635021	FALSE	JUN-DT
Region_705	chr1	58785156	58816042	30886	5	38	52.16666667	7.048367696	0.579050418	0.062293237	+	101926907	grey	-0.016172743	FALSE	LOC101926907
Region_706	chr1	58882868	58931897	49029	6	29	44.28787879	7.187093457	0.70724134	0.044454544	-	105378753	grey	0.218250505	FALSE	LINC02777
Region_707	chr1	58933643	59132803	199160	16	117	143.6515152	13.29371445	0.896402612	0.024218541	+	101926925	grey	0.027423506	FALSE	LINC01358
Region_708	chr1	59131932	59208121	76189	9	44	60.39393939	6.372727439	0.908070634	0.060613884	-	729467	grey	-0.151627009	FALSE	HSD52
Region_709	chr1	59131960	59296491	164531	20	132	157.0606061	11.06606564	0.929922709	0.030235412	-	105378756	grey	-0.084477702	FALSE	FGGY-DT
Region_710	chr1	59296638	59810647	514009	70	530	585.3939394	23.64668055	0.912694871	0.011443025	+	55277	grey	0.027396471	FALSE	FGGY
Region_711	chr1	59814786	59876322	61536	5	30	45.57575758	6.679614466	0.926032907	0.047000699	+	51361	grey	-0.384855964	FALSE	HOOK1
Region_712	chr1	59987269	60073770	86501	5	33	49.5303030	7.03008851	0.9269158	0.04608696	-	127795	grey	0.2853413	FALSE	C1orf87

							3	5		5						
Region_713	chr1	60515716	60640507	124791	8	47	67.89393939	9.026676342	0.874244021	0.039816925	-	105378763	grey	-0.165999582	FALSE	LINC01748
Region_714	chr1	60659631	60868009	208378	13	82	106.6666667	10.83489141	0.881587564	0.035783868	-	101926964	grey	0.125333851	FALSE	LOC101926964
Region_715	chr1	60865259	61462788	597529	68	482	530.2272727	22.39766159	0.768636018	0.019269693	+	4774	grey	0.19248989	FALSE	NFIA
Region_716	chr1	60912675	60970776	58101	3	16	25.86363636	4.597278003	0.529988591	0.115459651	-	100996570	grey	0.033759262	FALSE	NFIA-AS2
Region_717	chr1	60912791	61056885	144094	14	92	109.3333333	9.162353298	0.682431601	0.052726681	-	105378764	grey	0.367553944	FALSE	LOC105378764
Region_718	chr1	61681046	61725423	44377	9	49	67.18181818	8.44148928	0.854989989	0.032301168	-	83941	grey	0.173969255	FALSE	TM2D1
Region_719	chr1	61742477	62178675	436198	77	532	598.4545455	28.42435674	0.93635387	0.0103739	+	10207	grey	-0.027794531	FALSE	PATJ
Region_720	chr1	62194849	62212328	17479	12	75	101.6060606	11.86187012	0.874130547	0.025611727	+	54596	grey	0.260518009	FALSE	L1TD1
Region_721	chr1	62236165	62319434	83269	13	87	111.4393939	11.60388368	0.719075609	0.035226434	-	163782	grey	-0.164744747	FALSE	KANK4
Region_722	chr1	62436297	62451804	15507	6	27	44.40909091	7.423408449	0.602311008	0.05014401	+	7398	grey	0.024527305	FALSE	USP1
Region_723	chr1	62454298	62688386	234088	39	250	286.1060606	17.96075104	0.859145067	0.012015551	-	85440	grey	-0.1496465	FALSE	DOCK7
Region_724	chr1	62597520	62606313	8793	3	11	19.62121212	4.433318436	0.938286671	0.063007179	+	27329	grey	0.106822008	FALSE	ANGPTL3

Region_725	chr1	62784132	62865516	81384	13	74	102.469697	10.6268451	0.692720063	0.028461239	+	84938	grey	-0.113707957	FALSE	ATG4C
Region_726	chr1	62975751	63012471	36720	4	23	33.59090909	5.400789893	0.914865747	0.056237077	-	105378769	grey	0.020888091	FALSE	LINC01739
Region_727	chr1	63159083	63317274	158191	31	211	255.2121212	14.40994865	0.867992392	0.025364867	-	199899	grey	-0.191292608	FALSE	LINC00466
Region_728	chr1	63320878	63324441	3563	4	15	28.03030303	5.651329856	0.063485492	0.056024242	-	100996301	grey	-0.072273085	FALSE	FOXD3-AS1
Region_729	chr1	63367575	63438553	70978	9	52	69.87878788	8.115540935	0.732637012	0.030969917	+	29929	grey	-0.14786419	FALSE	ALG6
Region_730	chr1	63440770	63593721	152951	16	91	109.9090909	10.47300893	0.870966652	0.032055177	-	23421	grey	-0.172388329	FALSE	ITGB3BP
Region_731	chr1	63523372	63572693	49321	6	28	39.68181818	6.097058789	0.963512985	0.035609993	+	84455	grey	0.052955701	FALSE	EFCAB7
Region_732	chr1	63593411	63660245	66834	7	38	60.48484848	8.120709375	0.891097514	0.034109125	+	5236	grey	-0.009086858	FALSE	PGM1
Region_733	chr1	63774017	64181498	407481	64	486	552.0606061	26.09737905	0.768994125	0.016219613	+	4919	grey	-0.047415223	FALSE	ROR1
Region_734	chr1	64094379	64171342	76963	12	85	109.5	7.987008682	0.802348943	0.038799457	-	101927034	grey	0.059531452	FALSE	ROR1-AS1
Region_735	chr1	64203623	64267368	63745	7	36	61.75757576	9.679575016	0.927744452	0.035747273	+	148581	grey	0.133428536	FALSE	UBE2U
Region_736	chr1	64470129	64693058	222929	25	192	229.7575758	16.19217342	0.844879481	0.026304452	+	57685	grey	-0.113852855	FALSE	CACHD1
Region_737	chr1	64745075	64833232	88157	6	24	41.6969697	6.93923005	0.79904249	0.03983003	+	55225	grey	-0.23031710	FALSE	RAVER2

								5	8	6				7		
Region_738	chr1	64833223	65067754	234531	48	325	386.8030303	22.84005907	0.721313664	0.016312169	-	3716	grey	0.113187475	FALSE	JAK1
Region_739	chr1	65147549	65232145	84596	23	142	180.5	17.57642652	0.767430339	0.020195945	+	205	grey	-0.031958442	FALSE	AK4
Region_740	chr1	65248219	65415871	167652	18	116	137.3181818	12.41606787	0.675037233	0.028934289	+	9829	grey	-0.1343075	FALSE	DNAJC6
Region_741	chr1	65420587	65436007	15420	4	19	30.28787879	5.726497524	0.005590537	0.013518055	+	54741	grey	-0.249470647	FALSE	LEPROT
Region_742	chr1	65420652	65641559	220907	41	279	352.0909091	22.89959843	0.771959734	0.020426579	+	3953	grey	0.317692951	FALSE	LEPR
Region_743	chr1	65792514	66374579	582065	49	391	428.9393939	21.14265014	0.879382297	0.018922637	+	5142	grey	0.032522961	FALSE	PDE4B
Region_744	chr1	66533267	66751139	217872	17	130	162.3939394	14.21145669	0.80443767	0.021153173	+	84251	grey	0.148517129	FALSE	SGIP1
Region_745	chr1	66812885	66924856	111971	5	24	37.15151515	6.723649248	0.579347732	0.034878744	-	79819	grey	0.067468219	FALSE	DNAI4
Region_746	chr1	66924895	66988619	63724	6	39	53.90909091	6.940909434	0.643574201	0.025480611	+	57708	grey	0.04046248	FALSE	MIER1
Region_747	chr1	66999350	67054148	54798	4	18	33.65151515	6.232649577	0.498587443	0.018848181	-	23169	grey	0.117841792	FALSE	SLC35D1
Region_748	chr1	67092165	67231853	139688	12	93	113.0606061	10.44233806	0.944680467	0.024507216	-	400757	grey	0.108086956	FALSE	C1orf141
Region_749	chr1	67138907	67259979	121072	14	101	123.8030303	11.64371035	0.946209314	0.022283201	+	149233	grey	0.154371585	FALSE	IL23R

Region_750	chr1	67307364	67398724	91360	14	83	102.1818182	9.133049185	0.897656831	0.022915073	+	3595	grey	-0.070255632	FALSE	IL12RB2
Region_751	chr1	67407810	67430415	22605	7	36	51.62121212	8.037920036	0.347695517	0.039637221	-	26135	grey	-0.274819243	FALSE	SERBP1
Region_752	chr1	67701475	67833467	131992	17	109	155.5757576	13.93176846	0.814079437	0.035361657	-	55970	grey	-0.068902417	FALSE	GNG12
Region_753	chr1	67832293	68202987	370694	45	358	418.2424242	20.39310638	0.847797364	0.024461441	+	100289178	grey	0.222465011	FALSE	GNG12-AS1
Region_754	chr1	68098473	68233120	134647	20	134	156.4090909	10.930518	0.777119832	0.025991966	-	79971	grey	0.007197377	FALSE	WLS
Region_755	chr1	68496676	68538627	41951	3	21	33.13636364	5.601885297	0.900738059	0.055226324	+	101927220	grey	-0.146511041	FALSE	DEPDC1-AS1
Region_756	chr1	69055838	69229607	173769	7	42	59	8.028794334	0.902081113	0.04772419	+	105378787	grey	-0.016058183	FALSE	LOC105378787
Region_757	chr1	69055852	69219097	163245	7	42	59	8.028794334	0.902081113	0.04772419	+	105378785	grey	-0.016058183	FALSE	LINC01707
Region_758	chr1	69567922	70148005	580083	31	219	248.530303	16.11231702	0.827567182	0.024355249	+	57554	grey	0.047522294	FALSE	LRRC7
Region_759	chr1	70144805	70205579	60774	4	29	41.78787879	6.412764078	0.951754904	0.033961791	-	55631	grey	0.117711019	FALSE	LRRC40
Region_760	chr1	70205682	70253052	47370	3	10	17.34848485	3.288639767	0.966404121	0.046704598	+	9295	grey	0.234201623	FALSE	SRSF11
Region_761	chr1	70258999	70354734	95735	9	45	69.75757576	10.95594058	0.655304684	0.021799449	-	81573	grey	0.066619672	FALSE	ANKRD13C
Region_762	chr1	70354786	70385339	30553	3	17	24.9848484	4.32343319	0.94667374	0.05115073	+	11147	grey	0.092651927	FALSE	ANKRD13C-DT

							8	5	4	1						
Region_763	chr1	70411218	70439851	28633	4	17	30.09090909	4.904400764	0.958415869	0.038791369	+	1491	grey	0.010023916	FALSE	CTH
Region_764	chr1	70706441	70786468	80027	10	52	82.18181818	10.17675948	0.825742733	0.038388412	+	101927244	grey	0.101170814	FALSE	LINC01788
Region_765	chr1	70852353	71047816	195463	14	87	112.1666667	11.25588165	0.778923049	0.029935469	-	5733	grey	0.068834979	FALSE	PTGER3
Region_766	chr1	71063291	71081289	17998	3	12	22.01515152	4.508086492	0.966176227	0.047817153	-	9406	grey	-0.012852032	FALSE	ZRANB2
Region_767	chr1	71081324	71487723	406399	30	222	253.469697	15.2529641	0.822394631	0.027737503	+	100852410	grey	0.151171045	FALSE	ZRANB2-DT
Region_768	chr1	71395943	72282539	886596	74	585	631.8636364	21.3274012	0.84408809	0.019039163	-	257194	grey	-0.20297343	FALSE	NEGR1
Region_769	chr1	72283170	72753772	470602	18	114	142	12.37242592	0.925329024	0.024951336	+	105378797	grey	0.101942315	FALSE	LOC105378797
Region_770	chr1	72636547	72899236	262689	10	62	77	7.87596148	0.915277545	0.037994428	-	105378798	grey	0.087790391	FALSE	LOC105378798
Region_771	chr1	74026015	74198187	172172	7	40	58.62121212	7.647553961	0.844749336	0.064471376	-	127255	grey	-0.165260735	FALSE	LRRIQ3
Region_772	chr1	74198235	74544393	346158	13	86	115.6818182	11.15507348	0.870056946	0.035949441	+	100526835	grey	0.059768665	FALSE	FPGT-TNNI3K
Region_773	chr1	74235387	74544428	309041	12	78	105.3181818	10.69026745	0.8705632	0.037670901	+	51086	grey	0.016561054	FALSE	TNNI3K
Region_774	chr1	74469376	74512611	43235	3	20	31.28787879	5.400185614	0.753313053	0.084519359	-	105378803	grey	-0.001891089	FALSE	LRRC53

Region_775	chr1	74568117	74673792	105675	6	32	49.59090909	7.00763819	0.676819949	0.056889257	-	127254	grey	0.006905117	FALSE	ERICH3
Region_776	chr1	74705482	74733408	27926	3	14	25.27272727	4.672490538	0.956021483	0.045152305	-	1429	grey	-0.128674355	FALSE	CRYZ
Region_777	chr1	74733152	74766678	33526	4	27	45.83333333	6.555287551	0.951227219	0.033149572	+	127253	grey	0.230170344	FALSE	TYW3
Region_778	chr1	75128434	75161533	33099	8	62	76.40909091	8.154238676	0.343328254	0.040324661	+	431707	grey	-0.022223263	FALSE	LHX8
Region_779	chr1	75202129	75724049	521920	28	191	229.5454545	17.43840308	0.821202499	0.02400497	-	204962	grey	-0.240853094	FALSE	SLC44A5
Region_780	chr1	75724431	75763720	39289	5	29	40.75757576	6.104375747	0.849118504	0.048872135	+	34	grey	0.113838062	FALSE	ACADM
Region_781	chr1	75749497	75795086	45589	7	47	63.87878788	8.109851846	0.956389489	0.035146892	+	5876	grey	0.315200519	FALSE	RABGGTB
Region_782	chr1	75796882	75913242	116360	6	32	51.36363636	7.645282837	0.912862603	0.048237045	+	4438	grey	-0.104510989	FALSE	MSH4
Region_783	chr1	75926454	76019356	92902	9	66	84.45454545	8.518182938	0.798164316	0.042045987	+	101927342	grey	-0.269806334	FALSE	LOC101927342
Region_784	chr1	76074746	76634603	559857	59	473	522.0454545	20.51598388	0.877671029	0.018720928	+	256435	grey	0.301860747	FALSE	ST6GALNAC3
Region_785	chr1	76758124	76779267	21143	6	45	59.21212121	6.655281654	0.766554784	0.067578298	-	110806286	grey	-0.119821171	FALSE	LINC02567
Region_786	chr1	76867480	77078482	211002	17	125	143.469697	10.34068841	0.872536954	0.024392336	+	81849	grey	0.226995686	FALSE	ST6GALNAC5
Region_787	chr1	77088989	77219430	130441	6	43	62.8636363	7.96702118	0.93867795	0.03179615	-	10026	grey	0.275638981	FALSE	PIGK

							6	6		9						
Region_788	chr1	77282019	77559966	277947	31	228	266.1212121	16.31376498	0.827555759	0.024248154	+	26289	grey	6.01648E-05	FALSE	AK5
Region_789	chr1	77562416	77683419	121003	7	48	62.10606061	6.589335878	0.836920808	0.041709049	-	26009	grey	0.097692033	FALSE	ZZZ3
Region_790	chr1	77695987	77759852	63865	11	66	100.9393939	10.12670314	0.580713993	0.020954525	-	23032	grey	-0.133216421	FALSE	USP33
Region_791	chr1	77779624	77879540	99916	6	33	50.65151515	6.763004394	0.92174036	0.045813263	+	374986	grey	0.104529982	FALSE	MIGA1
Region_792	chr1	77881348	77889539	8191	4	16	24.65151515	4.909934746	0.266407775	0.044665256	-	374987	grey	-0.100425384	FALSE	NEXN-AS1
Region_793	chr1	77888513	77943895	55382	7	34	50.15151515	7.264756705	0.565853226	0.04086319	+	91624	grey	-0.160004818	FALSE	NEXN
Region_794	chr1	77944055	77979110	35055	14	78	103.5454545	11.65950496	0.861544809	0.026874385	-	8880	grey	-0.250189329	FALSE	FUBP1
Region_795	chr1	77979175	78017964	38789	9	63	85.03030303	9.268589963	0.651155807	0.016813551	+	11080	grey	-0.239591757	FALSE	DNAJB4
Region_796	chr1	77979542	78138444	158902	20	139	169.2272727	13.84729405	0.669778756	0.017704369	+	54810	grey	-0.023680685	FALSE	GIPC2
Region_797	chr1	78229599	78369464	139865	14	67	93.16666667	11.94420791	0.803780456	0.051134902	+	149047	grey	-0.120176817	FALSE	MGC27382
Region_798	chr1	78303884	78540701	236817	31	240	280.6363636	16.80992048	0.81121046	0.027757801	+	5737	grey	-0.374842394	FALSE	PTGFR
Region_799	chr1	78889764	79282124	392360	19	132	161.8333333	12.34078084	0.765733502	0.035447608	-	64123	grey	-0.041332476	FALSE	ADGRL4

Region_800	chr1	80535755	80646854	111099	4	24	35.34848485	5.304547901	0.774466093	0.077780598	+	101927412	grey	-0.261588432	FALSE	LINC01781
Region_801	chr1	81306147	81993932	687785	52	323	386.2424242	22.46300247	0.8246304	0.020552974	+	23266	grey	0.358953818	FALSE	ADGRL2
Region_802	chr1	81513880	81557702	43822	3	16	30.28787879	4.82227253	0.936819149	0.054191981	-	101927434	grey	0.075159101	FALSE	LOC101927434
Region_803	chr1	82903183	83166815	263632	14	98	129.0757576	10.48561008	0.845849678	0.0432251	+	103283057	grey	0.245961674	FALSE	LINC01362
Region_804	chr1	82970820	82986208	15388	4	27	39	5.925174454	0.864956686	0.069299814	-	101927498	grey	-0.287518298	FALSE	LINC01361
Region_805	chr1	83575776	83861053	285277	16	110	134.6060606	10.38175736	0.803592972	0.024074473	-	101927587	grey	-0.005178853	FALSE	LINC01725
Region_806	chr1	83801516	83861026	59510	6	33	53.65151515	7.890583335	0.716294458	0.046283706	-	101927560	grey	-0.042202388	FALSE	LOC101927560
Region_807	chr1	83865024	83999150	134126	7	40	55.37878788	7.980293327	0.935436962	0.039711787	-	79739	grey	-0.031403978	FALSE	TTLL7
Region_808	chr1	84078062	84238498	160436	9	47	67.34848485	8.213483144	0.881998235	0.033572333	+	5567	grey	-0.051628443	FALSE	PRKACB
Region_809	chr1	84298366	84389957	91591	3	14	21.83333333	4.175324339	0.883316697	0.07533685	+	148418	grey	0.28182973	FALSE	SAMD13
Region_810	chr1	84479259	84498352	19093	5	30	47.21212121	8.362029845	0.584016793	0.029335434	+	80135	grey	-0.018057566	FALSE	RPF1
Region_811	chr1	84498323	84556688	58365	9	61	82.93939394	9.761586863	0.744897372	0.023675899	-	2787	grey	0.024748114	FALSE	GNG5
Region_812	chr1	84506300	84567379	61079	9	62	83.7575757	8.65769663	0.96244283	0.0240383	+	10050574	grey	-0.10949083	FALSE	SPATA1

							6	1	6			1		6		
Region_813	chr1	84549611	84574480	24869	3	18	28.90909091	5.465211018	0.977953234	0.027763041	-	1486	grey	-0.342074586	FALSE	CTBS
Region_814	chr1	84811602	84893206	81604	5	31	47.8030303	6.707561054	0.778225548	0.023094036	-	23566	grey	0.006638987	FALSE	LPAR3
Region_815	chr1	84925583	84997113	71530	6	29	46.1969697	7.549263228	0.680444824	0.04812145	-	255231	grey	0.240616041	FALSE	MCOLN2
Region_816	chr1	84999147	85133138	133991	12	69	91.81818182	8.359074463	0.646285693	0.034264488	+	126820	grey	-0.200735486	FALSE	DNAI3
Region_817	chr1	85018082	85048500	30418	5	26	40.31818182	5.466426454	0.502147107	0.046667384	-	55283	grey	-0.178118602	FALSE	MCOLN3
Region_818	chr1	85156889	85201724	44835	11	72	87.98484848	8.40419753	0.584559763	0.028699116	-	84144	grey	0.10612799	FALSE	SYDE2
Region_819	chr1	85265776	85276632	10856	5	25	41.60606061	7.739765029	0.390132013	0.02767416	-	8915	grey	-0.11517543	FALSE	BCL10
Region_820	chr1	85276388	85399963	123575	11	79	103.6515152	8.736294628	0.85349662	0.028053057	+	646626	grey	0.014431453	FALSE	BCL10-AS1
Region_821	chr1	85318481	85578363	259882	43	324	359.2272727	19.62948759	0.742307843	0.01582135	-	23576	grey	0.357953102	FALSE	DDAH1
Region_822	chr1	85580761	85584589	3828	3	12	21.01515152	5.319029478	0.016255501	0.032414656	+	3491	grey	0.115969025	FALSE	CCN1
Region_823	chr1	85649417	85708433	59016	9	50	69.89393939	9.031787944	0.669167695	0.027636473	-	54680	grey	-0.2174558	FALSE	ZNHIT6
Region_824	chr1	85729233	86156943	427710	38	240	276.5454545	16.96256677	0.776083283	0.025067518	-	255631	grey	-0.108545403	FALSE	COL24A1

Region_825	chr1	86288704	86320711	32007	3	16	27.68181818	5.050998655	0.795057572	0.075091953	-	105378824	grey	0.124586676	FALSE	LINC02795
Region_826	chr1	86346824	86396342	49518	6	42	56.13636364	7.308343732	0.646223901	0.024147137	-	57489	grey	-0.047621495	FALSE	ODF2L
Region_827	chr1	86569024	86704484	135460	9	61	74.13636364	7.994009995	0.924533953	0.038582723	-	105378828	grey	0.056419468	FALSE	CLCA4-AS1
Region_828	chr1	86704570	86748184	43614	5	27	39.89393939	5.982355564	0.712897408	0.045464563	+	51100	grey	0.077069118	FALSE	SH3GLB1
Region_829	chr1	86862445	86914424	51979	6	33	46.21212121	6.082800852	0.963470981	0.028288691	-	9403	grey	0.06485176	FALSE	SELENOF
Region_830	chr1	86914635	87109982	195347	10	63	81.89393939	9.121620618	0.962932696	0.024414733	+	9653	grey	-0.045700573	FALSE	HS2ST1
Region_831	chr1	86993009	87169204	176195	12	84	101.6060606	8.371892145	0.944453707	0.023696601	+	339524	grey	0.125282048	FALSE	LINC01140
Region_832	chr1	87212669	87264741	52072	4	20	28.5	4.531089188	0.930316044	0.062158522	+	101927844	grey	-0.008133416	FALSE	LINC02801
Region_833	chr1	87328880	87348923	20043	9	49	71.84848485	8.811255781	0.417090732	0.021494875	+	8543	grey	0.052406229	FALSE	LMO4
Region_834	chr1	87353521	87371683	18162	5	29	42.72727273	6.049908975	0.869506329	0.114462267	-	100505768	grey	0.293406694	FALSE	LINC01364
Region_835	chr1	87620803	88228297	607494	47	348	388.7424242	18.92683126	0.926057179	0.016098371	-	107985518	grey	0.142713498	FALSE	LOC107985518
Region_836	chr1	87899454	88329083	429629	33	234	283.2575758	15.53884523	0.928156451	0.018055195	-	107985519	grey	0.038451589	FALSE	LINC01773
Region_837	chr1	88513130	88685204	172074	17	125	154.454545	13.0786753	0.80076650	0.02289111	-	10192789	grey	0.243892012	FALSE	PKN2-AS1

							5	2	4	9		1				
Region_838	chr1	88684222	88836255	152033	10	65	82.45454545	8.462008887	0.773002688	0.01641482	+	5586	grey	0.27811377	FALSE	PKN2
Region_839	chr1	88852633	88891944	39311	5	30	44.56060606	6.732535899	0.791856608	0.047056616	-	2959	grey	-0.121196635	FALSE	GTF2B
Region_840	chr1	88935773	88992953	57180	3	15	25.96969697	4.180429485	0.526492741	0.080262095	-	56267	grey	-0.128978527	FALSE	KYAT3
Region_841	chr1	89106132	89150456	44324	5	19	35.48484848	6.197375187	0.905441196	0.073299069	-	2634	grey	0.073991844	FALSE	GBP2
Region_842	chr1	89131742	89176009	44267	5	29	39.77272727	5.311223109	0.846524548	0.080155048	-	388646	grey	-0.136808978	FALSE	GBP7
Region_843	chr1	89256189	89272860	16671	4	22	38.16666667	5.944831843	0.515245802	0.0320522	-	115362	grey	0.067088325	FALSE	GBP5
Region_844	chr1	89427533	89525472	97939	11	68	91.37878788	8.517430366	0.702144851	0.037582666	-	105378844	grey	-0.009370584	FALSE	LOC105378844
Region_845	chr1	89524829	89597861	73032	8	52	66.66666667	8.111403812	0.520473858	0.032555374	+	23507	grey	-0.037778714	FALSE	LRRC8B
Region_846	chr1	89581291	89633003	51712	7	39	54.16666667	7.019934984	0.724545296	0.036590971	-	400761	grey	-0.050505254	FALSE	LRRC8C-DT
Region_847	chr1	89629725	89764518	134793	13	87	109.4545455	9.952474479	0.789590579	0.023982311	+	84230	grey	0.225699715	FALSE	LRRC8C
Region_848	chr1	89633140	89936611	303471	30	218	261.2272727	16.38554283	0.82681889	0.018259275	+	55144	grey	0.149442522	FALSE	LRRC8D
Region_849	chr1	90242088	90284188	42100	3	15	23.63636364	3.983181425	0.954015252	0.062585614	+	105378849	grey	0.01455577	FALSE	LOC105378849

Region_850	chr1	90711539	90717302	5763	3	11	20.84848485	3.91935012	0.11646133	0.080290599	-	343472	grey	-0.075215004	FALSE	BARHL2
Region_851	chr1	90769086	90851671	82585	13	73	97.13636364	11.10632026	0.464779128	0.043910235	-	105378853	grey	0.025414119	FALSE	LINC02609
Region_852	chr1	90835660	90849472	13812	5	13	23.53030303	5.177524496	0.220747342	0.082866502	+	105378855	grey	-0.073242825	FALSE	LINC02788
Region_853	chr1	90915298	91022272	106974	7	44	57.56060606	7.123371691	0.759916196	0.044345228	-	84146	grey	-0.26280831	FALSE	ZNF644
Region_854	chr1	91260766	91404856	144090	13	97	120.469697	10.88714089	0.889129605	0.029355628	-	164045	grey	0.048310908	FALSE	HFM1
Region_855	chr1	91500851	91525764	24913	3	11	24.95454545	4.504465539	0.64833654	0.03173067	+	8317	grey	0.060484418	FALSE	CDC7
Region_856	chr1	91680343	91906335	225992	36	258	295.6666667	18.30286513	0.752437462	0.01715105	-	7049	grey	0.121869225	FALSE	TGFBR3
Region_857	chr1	91949343	92014426	65083	9	51	69.15151515	8.476651052	0.88942204	0.043492689	+	676	grey	-0.193915094	FALSE	BRDT
Region_858	chr1	92029985	92063538	33553	4	18	29.48484848	5.221972584	0.915097602	0.051772215	+	253152	grey	0.113878883	FALSE	EPHX4
Region_859	chr1	92080305	92184725	104420	21	135	168.530303	16.08268988	0.709469096	0.018665519	+	284697	grey	0.073794734	FALSE	BTBD8
Region_860	chr1	92246402	92298987	52585	9	50	65.57575758	7.423581151	0.864591371	0.021024152	-	11146	grey	0.331215621	FALSE	GLMN
Region_861	chr1	92299059	92402056	102997	11	80	99.77272727	9.423041244	0.880590517	0.018617404	+	79871	grey	0.314409792	FALSE	RPAP2
Region_862	chr1	92473043	92486925	13882	5	23	38.3787878	6.52277702	0.38008401	0.04149370	-	2672	grey	0.154278457	FALSE	GFI1

							8		3	8						
Region_863	chr1	92508696	92792404	283708	43	314	360.045454 5	19.3378320 5	0.86587714 4	0.01429100 7	-	7813	grey	-0.11316925 5	FALSE	EVI5
Region_864	chr1	92832737	92961522	128785	20	131	151.575757 6	11.3056291	0.74398210 3	0.03545817 2	-	388650	grey	-0.00288211 4	FALSE	DIPK1A
Region_865	chr1	93079235	93139079	59844	8	55	73.8030303	8.88770922 6	0.72316532 1	0.02324108 4	+	22823	grey	-0.28832952 3	FALSE	MTF2
Region_866	chr1	93149742	93180516	30774	4	15	25.4545454 5	5.25702992 5	0.73476731 6	0.03137123 4	-	50999	grey	0.138827424	FALSE	TMED5
Region_867	chr1	93179919	93278730	98811	21	147	188.454545 5	13.7596755 5	0.67501727 7	0.02339408 3	+	343099	grey	-0.13383484 2	FALSE	CCDC18
Region_868	chr1	93262186	93346025	83839	7	29	49.7878787 9	7.75096053 9	0.98014769 5	0.01930463 6	-	10013156 4	grey	-0.42703382 5	FALSE	CCDC18-AS1
Region_869	chr1	93345907	93369493	23586	7	37	52.9848484 8	6.63671008 5	0.14292929 3	0.01192408 5	+	1810	grey	-0.06216528 2	FALSE	DR1
Region_870	chr1	93448118	93554661	106543	20	134	162.681818 2	12.6420045 8	0.86645785 7	0.02563585 4	+	54874	grey	-0.08775148 1	FALSE	FNBP1L
Region_871	chr1	93561741	93847150	285409	57	433	492.272727 3	23.4865234 4	0.83595223 1	0.01523173 7	-	8412	grey	0.194731591	FALSE	BCAR3
Region_872	chr1	93591966	93610605	18639	6	36	53.1212121 2	6.91601297 2	0.93247131 4	0.04072717 8	+	10012904 6	grey	0.129991706	FALSE	BCAR3-AS1
Region_873	chr1	93992834	94121148	128314	32	218	260.530303	17.1151931 6	0.87153094 3	0.02805908 8	-	24	grey	-0.27231766	FALSE	ABCA4
Region_874	chr1	94149847	94275068	125221	17	114	137.242424 2	10.8373437 1	0.75328391 1	0.02470022 8	-	9411	grey	0.391539896	FALSE	ARHGAP29

Region_875	chr1	94247819	94411036	163217	24	186	207.3787879	13.93365064	0.757969808	0.026933174	+	107985092	grey	0.317671825	FALSE	ARHGAP29-AS1
Region_876	chr1	94418389	94518666	100277	17	98	141.2575758	10.9815378	0.921723376	0.028020621	+	5825	grey	-0.02560411	FALSE	ABCD3
Region_877	chr1	94529173	94541759	12586	5	38	50.53030303	6.179917094	0.797589962	0.045875818	-	2152	grey	-0.001628156	FALSE	F3
Region_878	chr1	94613814	94855421	241607	40	295	350.6363636	22.23277993	0.768894136	0.021570884	-	101928079	grey	-0.241811384	FALSE	SLC44A3-AS1
Region_879	chr1	94820342	94895246	74904	19	116	141.7424242	12.45707782	0.72541191	0.029667132	+	126969	grey	-0.169280881	FALSE	SLC44A3
Region_880	chr1	94974405	95072951	98546	16	122	146.7878788	10.31729713	0.728187448	0.035579864	-	199857	grey	-0.137904427	FALSE	ALG14
Region_881	chr1	95117355	95197607	80252	8	37	53.07575758	7.23944453	0.699826883	0.040021201	+	148534	grey	0.272635181	FALSE	TLCD4
Region_882	chr1	95117923	95244955	127032	8	43	61.06060606	8.093451841	0.809596406	0.03908568	+	100527978	grey	0.228705516	FALSE	TLCD4-RWDD3
Region_883	chr1	95151377	95247225	95848	5	25	41.90909091	6.44438282	0.733101965	0.043627897	+	25950	grey	0.106050689	FALSE	RWDD3
Region_884	chr1	95161676	95233982	72306	5	25	41.90909091	6.44438282	0.733101965	0.043627897	-	101928118	grey	0.106050689	FALSE	RWDD3-DT
Region_885	chr1	95510167	95782342	272175	27	225	258.7424242	17.46760572	0.844493592	0.023013045	+	101928219	grey	-0.175474245	FALSE	LOC101928219
Region_886	chr1	95937901	96022890	84989	4	20	32.07575758	5.444293006	0.703309293	0.033529334	+	102723661	grey	0.124805374	FALSE	LINC02790
Region_887	chr1	96254069	96374125	120056	7	47	69.9393939	8.87672633	0.90788472	0.03674001	-	10192824	grey	-0.04482256	FALSE	LINC01787

							4	3	1	8		1		5		
Region_888	chr1	96721665	96823738	102073	8	30	49.6969697	6.745869841	0.852422669	0.025476661	+	58155	grey	0.096348744	FALSE	PTBP2
Region_889	chr1	97077743	97921049	843306	47	385	433.3636364	19.00011042	0.950247616	0.017684461	-	1806	grey	-0.103126147	FALSE	DPYD
Region_890	chr1	97095923	97322955	227032	19	159	183.8939394	12.78837066	0.962478731	0.021404324	+	100873932	grey	0.029778753	FALSE	DPYD-AS1
Region_891	chr1	97883264	98046147	162883	9	47	69.22727273	7.751516883	0.643545686	0.030070789	-	109729128	grey	0.000781452	FALSE	LINC01930
Region_892	chr1	97986740	98049871	63131	9	44	66.60606061	7.73379952	0.424095644	0.034872048	-	400765	grey	-0.031104452	FALSE	MIR137HG
Region_893	chr1	98661701	98760500	98799	5	22	35.87878788	6.982428829	0.873582701	0.062681473	+	51375	grey	-0.045839356	FALSE	SNX7
Region_894	chr1	98890245	99227929	337684	20	135	177.5909091	15.05781399	0.75501399	0.034131631	-	163404	grey	0.095365174	FALSE	PLPPR5
Region_895	chr1	99004276	99244794	240518	15	103	131.8787879	11.85231578	0.737627068	0.035133918	+	100129620	grey	0.223801956	FALSE	PLPPR5-AS1
Region_896	chr1	99264292	99309590	45298	3	14	29.63636364	5.822070379	0.603787428	0.056235169	+	9890	grey	0.035278192	FALSE	PLPPR4
Region_897	chr1	99472332	99600995	128663	5	21	41.10606061	6.9661352	0.880162369	0.058509183	-	101928270	grey	0.090364526	FALSE	LINC01708
Region_898	chr1	99646113	99694541	48428	3	24	34.15151515	5.600116549	0.667395014	0.079137533	+	54873	grey	-0.127363701	FALSE	PALMD
Region_899	chr1	99703970	99766635	62665	7	45	62.37878788	7.312297653	0.904275842	0.082987538	-	391059	grey	0.426594184	FALSE	FRRS1

Region_900	chr1	99850361	99924023	73662	10	59	76.93939394	8.652040734	0.905125182	0.034826313	+	178	grey	0.311551676	FALSE	AGL
Region_901	chr1	99969351	100035634	66283	7	32	48.12121212	7.378795298	0.975489205	0.025490993	+	23443	grey	0.047558562	FALSE	SLC35A3
Region_902	chr1	99970011	100083377	113366	13	73	102.6363636	10.97919202	0.873609744	0.027648149	+	64645	grey	0.224867465	FALSE	MFSD14A
Region_903	chr1	100083563	100132955	49392	4	18	31.24242424	5.123282019	0.977031449	0.037682571	-	163786	grey	-0.156975501	FALSE	SASS6
Region_904	chr1	100133150	100150496	17346	3	14	26.12121212	4.944352338	0.160774071	0.117309072	+	54482	grey	-0.203160457	FALSE	TRMT13
Region_905	chr1	100148448	100178273	29825	4	26	36.81818182	5.007407101	0.906305543	0.059708496	-	127495	grey	0.076818346	FALSE	LRRC39
Region_906	chr1	100186919	100249834	62915	10	65	84.09090909	8.482973486	0.964567027	0.020705261	-	1629	grey	0.345266756	FALSE	DBT
Region_907	chr1	100266216	100292769	26553	5	36	49.04545455	6.696570461	0.890976296	0.042729872	+	8634	grey	-0.045437041	FALSE	RTCA
Region_908	chr1	100345001	100520277	175276	19	133	162.8939394	13.84877533	0.939011796	0.016309236	+	8556	grey	0.087096945	FALSE	CDC14A
Region_909	chr1	100538139	100542021	3882	6	37	52.25757576	6.728379862	0.112522943	0.040973797	+	54112	grey	0.224098581	FALSE	GPR88
Region_910	chr1	100896076	100996260	100184	8	48	68.53030303	8.748133334	0.955296415	0.027231436	+	148867	grey	0.101762933	FALSE	SLC30A7
Region_911	chr1	100989623	101026088	36465	9	63	80.75757576	7.321329442	0.85514162	0.025778764	-	51611	grey	0.129281227	FALSE	DPH5
Region_912	chr1	10102584	10109051	64669	3	10	18.1515151	3.42498702	0.63651996	0.06709269	+	10260646	grey	-0.16472906	FALSE	DPH5-DT

		4	3				5	6	2	1		5		3		
Region_913	chr1	101323337	101390303	66966	9	50	62.98484848	6.448595373	0.906247975	0.045431684	+	104355286	grey	-0.201543064	FALSE	LINC01307
Region_914	chr1	101639509	101787572	148063	17	104	143.9848485	12.74422032	0.832229667	0.039112994	+	105378872	grey	0.02469148	FALSE	LINC01709
Region_915	chr1	101802560	101996926	194366	8	37	63.51515152	9.038792336	0.73971463	0.042564934	-	118427	grey	0.105719156	FALSE	OLFM3
Region_916	chr1	102763322	102853842	90520	3	13	28.16666667	4.972488414	0.836313948	0.064617917	-	112268286	grey	-0.030621332	FALSE	LOC112268286
Region_917	chr1	102876467	103108872	232405	10	60	76.46969697	7.927012624	0.806871265	0.056519133	-	1301	grey	-0.081543065	FALSE	COL11A1
Region_918	chr1	103415980	103525511	109531	6	34	48.71212121	7.15276203	0.893560708	0.052160851	-	101928436	grey	-0.0690828	FALSE	RNPC3-DT
Region_919	chr1	103525691	103555239	29548	5	20	32.24242424	5.953823868	0.982559797	0.035780115	+	55599	grey	-0.142445063	FALSE	RNPC3
Region_920	chr1	103553815	103579534	25719	5	25	40	9.275277313	0.947329472	0.050512502	+	280	grey	0.280307634	FALSE	AMY2B
Region_921	chr1	104072983	104226678	153695	10	59	78.21212121	8.297386338	0.890931217	0.040288638	+	100129138	grey	0.064375402	FALSE	LOC100129138
Region_922	chr1	104998406	105032365	33959	5	25	43.06060606	6.346116409	0.836304427	0.070415041	-	105378880	grey	0.100806952	FALSE	LOC105378880
Region_923	chr1	105927620	106028277	100657	5	22	34.06060606	5.971415984	0.908869973	0.057731157	-	105378884	grey	-0.116555552	FALSE	LINC01677
Region_924	chr1	105956694	106048753	92059	5	21	33.74242424	5.95188556	0.925798188	0.041916944	+	105378886	grey	0.049977691	FALSE	LOC105378886

Region_925	chr1	105956715	106124564	167849	10	47	70.48484848	10.04029179	0.88065316	0.045717139	+	107985116	grey	0.020181101	FALSE	LOC107985116
Region_926	chr1	107056674	107059294	2620	4	19	34.36363636	6.29929511	0.085396648	0.061567125	+	55170	grey	-0.169999748	FALSE	PRMT6
Region_927	chr1	107140007	107484923	344916	20	149	177.6363636	11.67065533	0.872116141	0.02491408	+	22854	grey	0.155642644	FALSE	NTNG1
Region_928	chr1	107571161	107965180	394019	39	289	330.9393939	20.80449566	0.84168504	0.021713111	-	10451	grey	-0.073341945	FALSE	VAV3
Region_929	chr1	108560089	108644900	84811	5	29	42.74242424	7.023719387	0.965080401	0.032376812	+	284611	grey	0.140887965	FALSE	EEIG2
Region_930	chr1	108661533	108666246	4713	3	13	20.71212121	4.384466116	0.758519343	0.133931982	+	105378890	grey	-0.023427265	FALSE	LOC105378890
Region_931	chr1	108746674	108809523	62849	4	16	24.74242424	3.820231995	0.955776379	0.054144643	+	6814	grey	-0.086969346	FALSE	STXBP3
Region_932	chr1	108815898	108963484	147586	23	133	173.1818182	18.2772739	0.63393017	0.018454744	-	254268	grey	-0.232855313	FALSE	AKNAD1
Region_933	chr1	108875350	108934545	59195	8	31	60.12121212	9.476310791	0.961592709	0.03170267	+	29899	grey	-0.292948603	FALSE	GPSM2
Region_934	chr1	108881885	108963527	81642	16	81	114.2878788	16.75411789	0.545609999	0.019365535	-	23155	grey	-0.094371441	FALSE	CLCC1
Region_935	chr1	108970214	109042113	71899	18	98	130.2575758	13.66782928	0.961370458	0.019531681	-	22911	grey	-0.197270075	FALSE	WDR47
Region_936	chr1	109105951	109113857	7906	3	14	25.40909091	5.400789893	0.331175896	0.072588064	-	127003	grey	-0.001771578	FALSE	CFAP276
Region_937	chr1	10911367	10920678	93102	18	116	139.196969	12.8664377	0.83908485	0.02919009	+	57535	grey	-0.24501801	FALSE	ELAPOR1

		9	1				7	9	6	9						
Region_938	chr1	109213918	109238182	24264	6	25	37.21212121	5.803222318	0.984570409	0.019861036	+	6301	grey	-0.010292566	FALSE	SARS1
Region_939	chr1	109249539	109275751	26212	13	71	92.96969697	10.9079642	0.731340826	0.043205202	+	1952	grey	0.153605766	FALSE	CELSR2
Region_940	chr1	109309568	109397918	88350	14	75	93.27272727	10.17167327	0.895984078	0.027822517	-	6272	grey	0.140935723	FALSE	SORT1
Region_941	chr1	109399042	109426448	27406	10	51	69.83333333	9.848987974	0.774945454	0.022302856	-	5686	grey	-0.176107133	FALSE	PSMA5
Region_942	chr1	109466546	109482134	15588	7	29	40.42424242	6.329565804	0.291231748	0.035707782	+	284612	grey	0.104432319	FALSE	SYPL2
Region_943	chr1	109483479	109492804	9325	3	14	22.81818182	4.209546261	0.947311336	0.047500477	+	127002	grey	-0.257189218	FALSE	ATXN7L2
Region_944	chr1	109494052	109502932	8880	3	17	27.45454545	4.749677943	0.93254165	0.055803154	+	284613	grey	-0.004081689	FALSE	CYB561D1
Region_945	chr1	109548615	109600195	51580	21	134	165.4242424	12.9710454	0.866293595	0.018130452	+	2773	grey	-0.169302481	FALSE	GNAI3
Region_946	chr1	109603091	109619929	16838	7	35	50.68181818	6.509541142	0.545884497	0.035138509	-	2780	grey	0.028245846	FALSE	GNAT2
Region_947	chr1	109616104	109632051	15947	11	59	81.62121212	9.671154814	0.574043221	0.034700346	+	271	grey	0.17526478	FALSE	AMPD2
Region_948	chr1	109656099	109674836	18737	12	47	66.34848485	8.948557983	0.389663223	0.042496963	+	2948	grey	-0.156372964	FALSE	GSTM4
Region_949	chr1	109668022	109709549	41527	7	31	42.89393939	5.602551034	0.747960482	0.058405525	+	2946	grey	0.163966678	FALSE	GSTM2

Region_950	chr1	109711780	109775428	63648	18	111	130.1818182	10.99637575	0.756286212	0.0381405	+	2949	grey	-0.094265665	FALSE	GSTM5
Region_951	chr1	109733932	109741038	7106	3	10	22.62121212	3.781595721	0.314077042	0.04219009	-	2947	grey	-0.066621388	FALSE	GSTM3
Region_952	chr1	109750080	109764027	13947	3	12	22.54545455	4.513848156	0.954709925	0.052678647	-	79574	grey	-0.011361293	FALSE	EPS8L3
Region_953	chr1	109828355	109871436	43081	9	63	78.22727273	8.204680568	0.82605102	0.057777816	+	107985167	grey	0.315593969	FALSE	LINC01768
Region_954	chr1	109910242	109930992	20750	6	30	48.59090909	6.822986652	0.650491049	0.046803922	+	1435	grey	0.104513733	FALSE	CSF1
Region_955	chr1	109984765	110023742	38977	11	82	98	9.808944113	0.767441746	0.022586243	+	10768	grey	0.091141302	FALSE	AHCYL1
Region_956	chr1	110031577	110074641	43064	21	159	196.5	15.15687201	0.622939456	0.022010386	+	85369	grey	0.002383419	FALSE	STRIP1
Region_957	chr1	110059870	110070672	10802	4	28	40.31818182	7.116955034	0.189821582	0.07571031	-	257	grey	0.051195537	FALSE	ALX3
Region_958	chr1	110082651	110109719	27068	8	53	66.63636364	7.550112304	0.644892033	0.042797539	+	104355139	grey	-0.138347608	FALSE	LINC01397
Region_959	chr1	110150494	110202202	51708	26	179	224.7575758	19.41376245	0.695689175	0.022915669	+	388662	grey	0.233869414	FALSE	SLC6A17
Region_960	chr1	110165948	110172489	6541	9	60	80.60606061	9.631484889	0.887903685	0.050451574	-	105378897	grey	0.06202794	FALSE	SLC6A17-AS1
Region_961	chr1	110210314	110283100	72786	31	208	236.6666667	15.8883928	0.53581982	0.0180188	+	3749	grey	0.08302215	FALSE	KCNC4
Region_962	chr1	11028637	11033917	52796	12	73	93.4545454	9.53081127	0.55407619	0.02202631	-	440600	grey	0.238735528	FALSE	RBM15-AS1

		5	1				5	9	5	8						
Region_963	chr1	110338506	110346681	8175	5	26	36.09090909	6.075745659	0.244851966	0.048180532	+	64783	grey	0.093399246	FALSE	RBM15
Region_964	chr1	110362851	110391082	28231	8	39	54.68181818	7.597892604	0.778187427	0.04210186	-	9122	grey	-0.197676454	FALSE	SLC16A4
Region_965	chr1	110382819	110432340	49521	13	78	100	10.35968517	0.90216929	0.024250513	+	101410535	grey	-0.090025333	FALSE	LAMTOR5-AS1
Region_966	chr1	110473756	110491277	17521	7	35	49.46969697	7.183784511	0.895184769	0.045899575	+	643160	grey	-0.005388932	FALSE	CYMP
Region_967	chr1	110519837	110631474	111637	30	237	282.6818182	20.68307304	0.742879984	0.021931418	-	3737	grey	-0.01596227	FALSE	KCNA2
Region_968	chr1	110653560	110674940	21380	4	27	37.5	5.180585058	0.088509286	0.067373038	-	3738	grey	0.125056092	FALSE	KCNA3
Region_969	chr1	110871188	110899922	28734	8	62	71.15151515	6.044049647	0.613414728	0.041310988	+	963	grey	0.007156769	FALSE	CD53
Region_970	chr1	110947190	110963965	16775	4	21	38.01515152	6.650604193	0.492049328	0.023668489	-	55791	grey	0.206614565	FALSE	LRIF1
Region_971	chr1	111139479	111185104	45625	7	46	63.81818182	7.266168371	0.814007386	0.030381184	+	10390	grey	0.121130524	FALSE	CEPT1
Region_972	chr1	111200771	111243440	42669	11	60	76.43939394	8.143196855	0.249977977	0.044699875	+	1117	grey	0.133899245	FALSE	CHI3L2
Region_973	chr1	111290851	111320566	29715	4	29	41.25757576	5.77344213	0.854938909	0.08730504	+	27159	grey	-0.105689921	FALSE	CHIA
Region_974	chr1	111483348	111563962	80614	6	37	48.92424242	6.538196896	0.92846389	0.03370005	-	57413	grey	0.032113304	FALSE	TMIGD3

Region_975	chr1	111542218	111716691	174473	14	78	105.2727273	11.50056247	0.798805318	0.031018256	+	5906	grey	0.479042585	FALSE	RAP1A
Region_976	chr1	111680630	111755824	75194	13	89	112.0606061	10.59445252	0.634521169	0.025747659	-	55924	grey	0.06618311	FALSE	INKA2
Region_977	chr1	111739579	111747798	8219	7	47	60.66666667	7.115169782	0.517790529	0.031834649	+	100506343	grey	-0.28743499	FALSE	INKA2-AS1
Region_978	chr1	111745299	111755509	10210	3	22	30.84848485	4.736900721	0.586122159	0.047036263	-	101928718	grey	0.048922805	FALSE	LOC101928718
Region_979	chr1	111754832	111775602	20770	5	13	28.16666667	5.172659882	0.152720058	0.055930049	+	11218	grey	0.034271596	FALSE	DDX20
Region_980	chr1	111770662	111989668	219006	31	225	261.5757576	15.53463684	0.831335197	0.029993793	-	3752	grey	-0.050066472	FALSE	KCND3
Region_981	chr1	111990536	111998842	8306	9	56	82.15151515	11.76466996	0.220699151	0.027979616	+	643355	grey	0.090132753	FALSE	LINC01750
Region_982	chr1	112176836	112360625	183789	28	192	227.7878788	13.92232866	0.796249345	0.030676051	-	105378909	grey	-0.279119812	FALSE	LINC02884
Region_983	chr1	112396214	112461164	64950	10	64	86.12121212	9.237907108	0.892783114	0.045951275	+	55917	grey	0.296260124	FALSE	CTTNBP2NL
Region_984	chr1	112466541	112530165	63624	20	123	146.8484848	11.69910243	0.841624236	0.024815532	+	7482	grey	-0.023865952	FALSE	WNT2B
Region_985	chr1	112523514	112620825	97311	20	123	152.3636364	11.741624	0.946671747	0.018197625	-	54879	grey	-0.153586532	FALSE	ST7L
Region_986	chr1	112619805	112671616	51811	4	20	33.59090909	5.256431292	0.78549567	0.055439474	+	829	grey	-0.142386117	FALSE	CAPZA1
Region_987	chr1	11267314	11270075	27615	3	15	25.5757575	4.64778063	0.65738390	0.09410543	+	4343	grey	-0.05357863	FALSE	MOV10

		1	6				8	2	4	8				3		
Region_988	chr1	112701127	112711224	10097	3	13	24.31818182	4.90261811	0.006600092	0.0174816	-	389	grey	0.143677228	FALSE	RHOC
Region_989	chr1	112702614	112715332	12718	7	34	51.48484848	7.26263869	0.276459288	0.022512822	-	333926	grey	0.080993546	FALSE	PPM1J
Region_990	chr1	112718905	112727235	8330	3	11	21.6969697	4.75546551	0.018513814	0.030897066	+	284467	grey	0.001265852	FALSE	TAFA3
Region_991	chr1	112820170	112850643	30473	8	44	63.81818182	7.959337218	0.799810691	0.044381079	-	100996702	grey	-0.102815849	FALSE	LINC01356
Region_992	chr1	112849821	112877878	28057	9	47	67.43939394	9.014737998	0.733341393	0.05125867	+	105378912	grey	-0.130214604	FALSE	LINC01357
Region_993	chr1	112956415	113047051	90636	16	92	120.8333333	11.57395396	0.771779016	0.03622338	+	100506392	grey	0.159876596	FALSE	SLC16A1-AS1
Region_994	chr1	113011687	113073113	61426	24	117	150.2424242	13.52724954	0.869764833	0.032267249	-	100996251	grey	0.12298263	FALSE	LRIG2-DT
Region_995	chr1	113073198	113132260	59062	11	74	90.74242424	8.418497228	0.891131632	0.018121105	+	9860	grey	0.021309791	FALSE	LRIG2
Region_996	chr1	113390515	113685923	295408	51	372	417.0757576	19.23483267	0.777938569	0.021336414	+	260425	grey	-0.093217102	FALSE	MAGI3
Region_997	chr1	113696831	113759489	62658	8	45	66.6969697	8.377570231	0.977458882	0.021447357	-	10745	grey	0.199378927	FALSE	PHTF1
Region_998	chr1	113761832	113812476	50644	6	31	45.89393939	6.00289363	0.757265135	0.037581258	-	54665	grey	-0.102334119	FALSE	RSBN1
Region_999	chr1	113812379	113901237	88858	13	85	108.3939394	9.891532957	0.920746022	0.035410253	+	100287722	grey	0.07453426	FALSE	AP4B1-AS1

Region_1000	chr1	113813811	113871753	57942	7	45	62.27272727	6.699128427	0.950065835	0.038278453	-	26191	grey	0.134708556	FALSE	PTPN22
Region_1001	chr1	113894194	113905201	11007	3	10	21.21212121	4.649284979	0.626522509	0.049229609	-	10717	grey	-0.161867364	FALSE	AP4B1
Region_1002	chr1	113929324	113977869	48545	4	16	30.07575758	5.517275264	0.605852078	0.057015679	+	204851	grey	0.020865973	FALSE	HIPK1
Region_1003	chr1	113979391	114035572	56181	4	16	30.60606061	5.582607022	0.711798737	0.074991538	+	56944	grey	-0.023683949	FALSE	OLFML3
Region_1004	chr1	114089291	114153880	64589	10	66	85.93939394	8.756323057	0.573858703	0.033764723	-	148281	grey	0.23423073	FALSE	SYT6
Region_1005	chr1	114206427	114262278	55851	6	38	53.83333333	6.956089382	0.88382522	0.037334578	-	107985443	grey	-0.101918289	FALSE	LOC107985443
Region_1006	chr1	114392790	114511203	118413	14	97	116.3181818	9.716218148	0.936918463	0.02265351	-	51592	grey	-0.000912068	FALSE	TRIM33
Region_1007	chr1	114567557	114581629	14072	5	30	42.78787879	5.765988211	0.968205684	0.030830186	-	10286	grey	0.198096419	FALSE	BCAS2
Region_1008	chr1	114582848	114670422	87574	15	96	114.030303	10.59022728	0.939707648	0.027003843	-	163259	grey	-0.181680508	FALSE	DENND2C
Region_1009	chr1	114673090	114695618	22528	3	16	26	5.692099788	0.959093951	0.047393606	-	270	grey	-0.141061218	FALSE	AMPD1
Region_1010	chr1	114716913	114758676	41763	8	51	68.33333333	7.326103662	0.869732316	0.043535264	-	7812	grey	0.033531045	FALSE	CSDE1
Region_1011	chr1	114854863	114995370	140507	7	43	58.59090909	7.464742301	0.87043634	0.051264848	+	6847	grey	-0.132486288	FALSE	SYCP1
Region_1012	chr1	11504801	11508950	41492	6	25	38.4545454	6.09206752	0.91824490	0.05410941	-	10100	grey	-0.08204997	FALSE	TSPAN2

		1	3				5	4	8	9				2		
Region_1013	chr1	115283034	115368072	85038	7	43	54.71212121	6.639659749	0.589975401	0.075602275	+	112840934	grey	-0.093477228	FALSE	NGF-AS1
Region_1014	chr1	115285904	115338770	52866	5	25	38.31818182	5.627293627	0.485506989	0.088001784	-	4803	grey	-0.101973294	FALSE	NGF
Region_1015	chr1	115641970	115698224	56254	5	24	37.15151515	5.762591362	0.579432425	0.035500804	+	81839	grey	0.100468662	FALSE	VANGL1
Region_1016	chr1	115700021	115768714	68693	8	41	61.83333333	8.075763042	0.948613515	0.033531367	-	845	grey	0.081267606	FALSE	CASQ2
Region_1017	chr1	115904855	115931616	26761	4	24	39.54545455	6.723510572	0.959847654	0.037690169	+	101928995	grey	-0.190917279	FALSE	LINC01649
Region_1018	chr1	115976513	116070054	93541	6	34	51.48484848	7.369881377	0.843264203	0.07140193	+	55356	grey	-0.430228205	FALSE	SLC22A15
Region_1019	chr1	116372668	116410261	37593	13	94	120.7575758	10.98118756	0.642650541	0.026138155	+	476	grey	0.183538408	FALSE	ATP1A1
Region_1020	chr1	116378437	116421301	42864	14	109	130.9393939	11.48896298	0.700149214	0.025878305	-	84852	grey	0.075469499	FALSE	ATP1A1-AS1
Region_1021	chr1	116423724	116478842	55118	7	38	54.48484848	8.26715917	0.915409694	0.045325113	-	101929023	grey	-0.389670712	FALSE	LINC01762
Region_1022	chr1	116493350	116502634	9284	3	18	27.57575758	4.246484903	0.956295248	0.059330071	+	112268235	grey	0.199422934	FALSE	LOC112268235
Region_1023	chr1	116514534	116571039	56505	10	60	75.37878788	10.01040251	0.87901447	0.043516995	-	965	grey	-0.124043214	FALSE	CD58
Region_1024	chr1	116574399	116667755	93356	18	116	150.1818182	13.23737028	0.777902507	0.030206101	-	3321	grey	-0.123757169	FALSE	IGSF3

Region_1025	chr1	116909916	116990353	80437	23	146	175.3787879	17.00385943	0.942537963	0.020451954	+	5738	grey	-0.222138451	FALSE	PTGFRN
Region_1026	chr1	117001750	117036552	34802	4	25	36.28787879	6.681236992	0.937615228	0.047026897	+	9398	grey	-0.226160328	FALSE	CD101
Region_1027	chr1	117025482	117059490	34008	4	22	33.37878788	5.91416852	0.698089595	0.053902004	-	101929099	grey	-0.296843145	FALSE	CD101-AS1
Region_1028	chr1	117060326	117107453	47127	7	45	60.21212121	7.553939678	0.964586841	0.03069322	+	8458	grey	0.098724857	FALSE	TTF2
Region_1029	chr1	117111060	117122587	11527	13	79	111.1212121	13.31802151	0.622279117	0.031052267	-	80263	grey	0.24653899	FALSE	TRIM45
Region_1030	chr1	117143587	117210960	67373	11	72	103.5	11.24722188	0.912578432	0.030187662	-	79679	grey	-0.016259831	FALSE	VTCN1
Region_1031	chr1	117272182	117321336	49154	7	46	60.25757576	7.944636519	0.886404132	0.058528103	+	104355292	grey	0.352774377	FALSE	LINC01525
Region_1032	chr1	117367449	117528872	161423	14	102	126.530303	11.16205068	0.971972776	0.015847829	+	10905	grey	0.000102908	FALSE	MAN1A2
Region_1033	chr1	117606048	117628389	22341	4	24	39.16666667	6.576375746	0.943936983	0.040279975	+	54855	grey	-0.145199663	FALSE	TENT5C
Region_1034	chr1	117863485	117929621	66136	5	38	58.72727273	8.177916708	0.976460764	0.025145535	-	54834	grey	-0.22811039	FALSE	GDAP2
Region_1035	chr1	117953590	118185228	231638	24	181	215.3484848	17.04698703	0.811632078	0.027256596	-	200162	grey	0.365751598	FALSE	SPAG17
Region_1036	chr1	118883046	118989556	106510	11	70	89.89393939	9.56295543	0.473486338	0.039428615	-	6913	grey	-0.024698141	FALSE	TBX15
Region_1037	chr1	11903121	11914065	109438	7	37	53.4545454	7.37601480	0.90750731	0.05894637	-	10352	grey	0.30591779	FALSE	WARS2

		6	4				5	5	2	9						
Region_1038	chr1	119140391	119275973	135582	9	62	85.74242424	9.633771697	0.929698711	0.02715818	+	101929147	grey	0.280532343	FALSE	WARS2-AS1
Region_1039	chr1	119368779	119394130	25351	3	17	25.54545455	4.654796759	0.861877797	0.087465225	+	51179	grey	-0.062671064	FALSE	HAO2
Region_1040	chr1	119619377	119648266	28889	6	32	44.46969697	6.702972238	0.782956248	0.043600336	-	90874	grey	-0.21574347	FALSE	ZNF697
Region_1041	chr1	119648411	119744218	95807	10	58	83.81818182	9.014363053	0.662559823	0.03848719	+	26227	grey	0.151304644	FALSE	PHGDH
Region_1042	chr1	119748002	119768905	20903	3	17	25.63636364	4.626466881	0.876236502	0.072026379	-	3158	grey	-0.18316528	FALSE	HMGCS2
Region_1043	chr1	119911553	120100688	189135	9	53	73.86363636	9.684065204	0.67468451	0.034722286	-	4853	grey	-0.097080072	FALSE	NOTCH2
Region_1044	chr1	120415035	120469676	54641	8	33	47.74242424	8.927590089	0.807106029	0.050008478	+	728841	grey	0.058635745	FALSE	NBPF8
Region_1045	chr1	121090289	121097655	7366	4	19	28.86363636	4.627298144	0.308179705	0.064863429	-	100996318	grey	-0.090093226	FALSE	LOC100996318
Region_1046	chr1	121184811	121392874	208063	9	37	53.59090909	7.08188865	0.832956991	0.060733008	+	653464	grey	0.092497813	FALSE	SRGAP2C
Region_1047	chr1	121360156	121398806	38650	10	40	57.83333333	8.33266664	0.483595212	0.068824293	-	100873165	grey	0.03646353	FALSE	SRGAP2-AS1
Region_1048	chr1	121396754	121463508	66754	30	185	223.3787879	15.97186483	0.785319275	0.031065505	+	107985194	grey	0.005872171	FALSE	LINC02798
Region_1049	chr1	143905487	143934776	29289	13	39	91.93939394	28.47448455	0.862838872	0.038126592	+	105371209	grey	0.207055744	FALSE	LOC105371209

Region_1050	chr1	144887265	145095528	208263	17	45	101.030303	15.75189336	0.897913975	0.027872508	-	647135	grey	-0.341917258	FALSE	SRGAP2B
Region_1051	chr1	145164099	145216058	51959	12	32	74.75757576	9.641354208	0.733386865	0.048097794	-	103091866	grey	0.061929718	FALSE	LINC01145
Region_1052	chr1	145289900	145425603	135703	11	75	92.16666667	9.117326419	0.816283315	0.040035259	-	100288142	grey	-0.145861385	FALSE	NBPF20
Region_1053	chr1	145572345	145607858	35513	12	77	94.09090909	11.38383101	0.834051249	0.02766298	-	101929780	grey	0.187079654	FALSE	NBPF25P
Region_1054	chr1	145607988	145670650	62662	14	91	113.0606061	13.57472628	0.881063003	0.032046741	+	653519	grey	0.206969478	FALSE	GPR89A
Region_1055	chr1	145670852	145708148	37296	5	28	39	6.002563555	0.946002515	0.040852766	-	5174	grey	-0.151582	FALSE	PDZK1
Region_1056	chr1	145719471	145739288	19817	7	45	60.27272727	9.120636709	0.784920015	0.051354912	+	11126	grey	0.004743842	FALSE	CD160
Region_1057	chr1	145738868	145824095	85227	8	41	55.60606061	9.2401778	0.763442572	0.052923078	-	27246	grey	-0.044513241	FALSE	RNF115
Region_1058	chr1	145824053	145844402	20349	7	45	62.21212121	8.031697113	0.975834392	0.027203776	+	10623	grey	-0.04722889	FALSE	POLR3C
Region_1059	chr1	145911350	145918717	7367	5	22	33.48484848	6.278761329	0.680234317	0.045902665	-	8799	grey	0.335370708	FALSE	PEX11B
Region_1060	chr1	145917714	145927678	9964	5	17	28.16666667	4.847150895	0.171632341	0.03314062	-	9939	grey	0.054156248	FALSE	RBM8A
Region_1061	chr1	145926590	145949043	22453	11	62	78.15151515	8.929195716	0.848298372	0.025471361	+	105371260	grey	0.145101504	FALSE	LIX1L-AS1
Region_1062	chr1	14592725	14597884	51590	18	114	134.621212	10.7785615	0.67995947	0.01722303	+	84265	grey	0.238602291	FALSE	POLR3GL

		8	8				1	6	4	1						
Region_1063	chr1	145933423	145958017	24594	6	28	38.84848485	5.797194081	0.944163342	0.037634263	-	128077	grey	0.078738595	FALSE	LIX1L
Region_1064	chr1	146017468	146036746	19278	3	17	24.59090909	4.513150947	0.888538314	0.060870395	-	148738	grey	0.113710533	FALSE	HJV
Region_1065	chr1	146321214	146401647	80433	17	94	130.9393939	15.24858509	0.682157821	0.028482433	-	102724364	grey	0.081247171	FALSE	SEC22B4P
Region_1066	chr1	146472566	146898974	426408	42	271	315.2575758	19.7883498	0.884186847	0.024264987	+	100288805	grey	0.350151201	FALSE	HYDIN2
Region_1067	chr1	146938324	146996202	57878	3	13	24.57575758	5.233209358	0.65675436	0.055543005	+	149013	grey	0.003940201	FALSE	NBPF12
Region_1068	chr1	147099482	147124285	24803	4	14	26.6969697	6.319614635	0.874968823	0.086938367	-	644861	grey	-0.136586572	FALSE	NBPF13P
Region_1069	chr1	147172744	147295765	123021	18	109	134.1060606	12.26142016	0.8527448	0.01893991	+	9557	grey	-0.092048697	FALSE	CHD1L
Region_1070	chr1	147175351	147243050	67699	14	81	103.6060606	10.6812255	0.819208327	0.021014014	-	2330	grey	-0.033065218	FALSE	FMO5
Region_1071	chr1	147258885	147517875	258990	47	368	414.0454545	23.47268908	0.90343079	0.017345838	-	100289211	grey	-0.164032926	FALSE	LINC00624
Region_1072	chr1	147541501	147626216	84715	23	157	184.2727273	14.13946518	0.902552066	0.019697934	+	607	grey	0.236820098	FALSE	BCL9
Region_1073	chr1	147629652	147670524	40872	9	68	86.43939394	10.46331142	0.792456593	0.031805576	-	51205	grey	0.148301856	FALSE	ACP6
Region_1074	chr1	147902795	147909269	6474	3	11	20.74242424	3.820231995	0.958290951	0.05277082	+	2703	grey	0.147174173	FALSE	GJA8

Region_1075	chr1	147928393	147993592	65199	5	17	29.42424242	5.516578109	0.746866571	0.040630226	+	51463	grey	0.350796245	FALSE	GPR89B
Region_1076	chr1	147993862	148014956	21094	4	22	31.93939394	5.621055986	0.855947331	0.070817514	-	100034743	grey	-0.087277193	FALSE	PDZK1P1
Region_1077	chr1	148159213	148255012	95799	14	65	94.34848485	12.93830618	0.87711841	0.031705475	-	101927468	grey	0.031054111	FALSE	LOC101927468
Region_1078	chr1	148290889	148459959	169070	23	187	216.2727273	14.36292819	0.506074244	0.034791377	-	105371225	grey	-0.103711459	FALSE	LOC105371225
Region_1079	chr1	148531385	148596011	64626	6	28	41.93939394	7.035905251	0.940641168	0.046011917	-	25832	grey	0.136483944	FALSE	NBPF14
Region_1080	chr1	148808140	149051273	243133	21	112	160.2272727	19.95903147	0.734643697	0.031791484	+	9659	grey	0.056047684	FALSE	PDE4DIP
Region_1081	chr1	149052186	149103561	51375	4	27	44.07575758	8.369009878	0.966423376	0.02823823	-	400818	grey	-0.08875731	FALSE	NBPF9
Region_1082	chr1	149197555	149321732	124177	7	16	35.1969697	9.199029542	0.794957616	0.087504964	-	653513	grey	0.170091693	FALSE	LOC653513
Region_1083	chr1	149606213	149679523	73310	16	92	124.3181818	14.89982869	0.459591274	0.028969336	+	57234	grey	-0.060588955	FALSE	LINC00869
Region_1084	chr1	149636538	149705716	69178	13	69	96.33333333	11.55100173	0.541028994	0.027131314	+	644634	grey	-0.118801062	FALSE	LOC644634
Region_1085	chr1	149676851	149747818	70967	9	33	70.83333333	12.35199558	0.419217208	0.06056759	-	102723955	grey	0.291771946	FALSE	PDE4DIPP7
Region_1086	chr1	149782689	149812373	29684	21	136	179.2424242	20.37121695	0.222063053	0.035912743	-	440689	grey	-0.316273956	FALSE	H2BC18
Region_1087	chr1	14981322	14981369	468	7	34	63.1060606	12.4444968	0.01970463	0.05222398	-	653604	grey	0.153639717	FALSE	H3C13

		5	3				1	2	1	3						
Region_1088	chr1	149887890	149900798	12908	10	70	96.51515152	11.50818094	0.273179125	0.031320519	+	51027	grey	0.227139245	FALSE	BOLA1
Region_1089	chr1	149937812	150010726	72914	9	42	61.87878788	8.260220059	0.600088912	0.026415129	-	56957	grey	-0.050257796	FALSE	OTUD7B
Region_1090	chr1	150045660	150067701	22041	3	13	22.71212121	4.653419426	0.669181226	0.050518933	-	105371427	grey	0.130957297	FALSE	
Region_1091	chr1	150067279	150145329	78050	20	121	152.6060606	12.86124625	0.914275831	0.024068453	+	11311	grey	-0.170881456	FALSE	VPS45
Region_1092	chr1	150149183	150160065	10882	6	35	48.43939394	6.440565641	0.636181126	0.029891456	+	51177	grey	-0.06491412	FALSE	PLEKHO1
Region_1093	chr1	150159405	150181429	22024	12	62	87.81818182	10.18431538	0.735282153	0.043920691	+	105371433	grey	-0.122472423	FALSE	LINC02988
Region_1094	chr1	150218417	150236156	17739	9	37	59.24242424	7.477269362	0.936834685	0.044812827	-	81611	grey	0.123733268	FALSE	ANP32E
Region_1095	chr1	150268200	150280916	12716	5	17	27.93939394	5.543891407	0.585805226	0.041651194	+	79630	grey	-0.380052317	FALSE	C1orf54
Region_1096	chr1	150282543	150287093	4550	4	11	21.78787879	4.60606674	0.647790233	0.088715345	+	148523	grey	-0.038359237	FALSE	CIART
Region_1097	chr1	150293861	150308979	15118	14	74	101.3636364	10.94410465	0.605400258	0.021497323	+	54460	grey	0.354787829	FALSE	MRPS21
Region_1098	chr1	150321479	150353233	31754	24	130	174.2878788	15.65273645	0.974248099	0.014071136	+	9129	grey	0.154678369	FALSE	PRPF3
Region_1099	chr1	150363091	150476566	113475	37	267	312.5909091	23.64869635	0.794570677	0.017012513	+	23248	grey	0.084959949	FALSE	RPRD2

Region_1100	chr1	150487414	150507602	20188	13	70	100.2727273	12.94906644	0.772903174	0.05331146	+	80222	grey	0.364146789	FALSE	TARS2
Region_1101	chr1	150548562	150557724	9162	13	63	93.90909091	10.89633287	0.52486483	0.033530645	-	100289061	grey	-0.144835022	FALSE	ADAMTSL4-AS2
Region_1102	chr1	150549369	150560937	11568	12	57	88.24242424	10.43376266	0.661327679	0.036999261	+	54507	grey	-0.098561314	FALSE	ADAMTSL4
Region_1103	chr1	150560895	150574552	13657	8	49	58.68181818	6.476369094	0.4964638	0.056593431	-	574406	grey	-0.215090211	FALSE	ADAMTSL4-AS1
Region_1104	chr1	150563102	150579738	16636	6	40	49.15151515	6.008308156	0.659797929	0.072991016	-	4170	grey	-0.256493711	FALSE	MCL1
Region_1105	chr1	150600851	150629612	28761	10	56	76.81818182	8.514077195	0.63947685	0.045616847	-	2029	grey	0.078570362	FALSE	ENSA
Region_1106	chr1	150646230	150697154	50924	13	75	95.1969697	10.02492232	0.875080052	0.035483917	-	55204	grey	0.269699762	FALSE	GOLPH3L
Region_1107	chr1	150698060	150720895	22835	5	23	37.18181818	6.370386031	0.807691433	0.132935792	-	84072	grey	0.068371202	FALSE	HORMAD1
Region_1108	chr1	150730079	150765957	35878	12	79	101.9393939	10.56973733	0.867168078	0.031689938	-	1520	grey	-0.22067906	FALSE	CTSS
Region_1109	chr1	150794880	150809577	14697	4	20	32.71212121	5.459684836	0.839322819	0.08403421	-	1513	grey	-0.259954256	FALSE	CTSK
Region_1110	chr1	150809713	150876708	66995	26	154	181.8939394	13.33143052	0.822139833	0.019779847	-	405	grey	-0.31127587	FALSE	ARNT
Region_1111	chr1	150887136	150913292	26156	6	34	48.01515152	5.931642461	0.868273494	0.064601067	+	100996521	grey	0.331799757	FALSE	CTXND2
Region_1112	chr1	15092626	15096474	38481	22	132	155.712121	12.3197966	0.92193119	0.01451422	+	9869	grey	-0.02547332	FALSE	SETDB1

		3	4				2	4	4	7				1		
Region_1113	chr1	15096058<br>3	15097500<br>3	14420	7	37	53.1212121<br>2	7.60063793<br>2	0.69406603<br>9	0.04510347<br>1	-	29956	grey	0.220948027	FALSE	CERS2
Region_1114	chr1	15098224<br>9	15099563<br>4	13385	9	48	70.3181818<br>2	9.19390950<br>7	0.90089224<br>2	0.04467890<br>4	+	8416	grey	-0.16274331<br>3	FALSE	ANXA9
Region_1115	chr1	15100842<br>0	15103571<br>3	27293	10	57	79.9545454<br>5	9.57469713<br>2	0.80645022<br>6	0.03129051<br>3	+	58497	grey	-0.09318623<br>3	FALSE	PRUNE1
Region_1116	chr1	15103632<br>1	15104772<br>0	11399	7	33	54.8181818<br>2	7.21090859<br>7	0.96681638<br>8	0.0265353	+	149428	grey	-0.13759351<br>3	FALSE	BNIPL
Region_1117	chr1	15105097<br>1	15107032<br>5	19354	10	53	69.0303030<br>3	8.70891620<br>4	0.55512809<br>1	0.03126019<br>8	-	56882	grey	-0.18905467<br>1	FALSE	CDC42SE1
Region_1118	chr1	15106039<br>7	15106954<br>4	9147	4	22	33.5909090<br>9	5.63226221<br>2	0.96655466<br>3	0.03300402<br>2	+	10962	grey	0.01050953	FALSE	MLLT11
Region_1119	chr1	15107057<br>8	15112554<br>2	54964	31	188	231.909090<br>9	16.0122330<br>9	0.88839141	0.01483117<br>2	+	126626	grey	-0.04653723<br>6	FALSE	GABPB2
Region_1120	chr1	15113168<br>5	15114663<br>1	14946	12	58	85.4242424<br>2	10.5787313<br>6	0.61293218<br>6	0.02377827<br>3	-	10500	grey	-0.07137657<br>7	FALSE	SEMA6C
Region_1121	chr1	15115666<br>4	15116913<br>3	12469	9	60	81.0303030<br>3	10.1919571<br>6	0.57882069<br>1	0.03656927<br>9	+	10053401<br>2	grey	-0.07636261<br>5	FALSE	TNFAIP8L2-SCNM1
Region_1122	chr1	15115974<br>8	15116594<br>8	6200	4	17	29.3030303	4.64416821<br>1	0.82005518<br>6	0.08131432<br>2	-	388695	grey	-0.07501954<br>4	FALSE	LYSMD1
Region_1123	chr1	15116602<br>2	15117029<br>6	4274	4	28	41.3636363<br>6	7.52153086<br>6	0.48260176<br>4	0.02516555	+	79005	grey	0.026856578	FALSE	SCNM1
Region_1124	chr1	15116998<br>6	15117598<br>1	5995	3	19	30.3030303	4.18042948<br>5	0.96255917	0.03925203<br>3	-	29765	grey	-0.15731072<br>1	FALSE	TMOD4

Region_1125	chr1	151176304	151195321	19017	14	69	89.15151515	9.351663652	0.973776377	0.019635754	-	6944	grey	-0.242622461	FALSE	VPS72
Region_1126	chr1	151197949	151249536	51587	28	176	206.3939394	15.45502536	0.923607053	0.013757017	+	8394	grey	-0.32662191	FALSE	PIP5K1A
Region_1127	chr1	151254709	151267479	12770	14	94	118.9242424	10.72209883	0.641267286	0.031211748	+	5710	grey	-0.135484511	FALSE	PSMD4
Region_1128	chr1	151279678	151282000	2322	4	18	34.21212121	6.680661302	0.969531367	0.035447559	-	100507670	grey	-0.058650247	FALSE	ZNF687-AS1
Region_1129	chr1	151281618	151292176	10558	12	74	90.48484848	9.627272837	0.622214988	0.041535363	+	57592	grey	-0.30025599	FALSE	ZNF687
Region_1130	chr1	151291797	151327715	35918	11	69	90.34848485	10.24393618	0.958735707	0.022945974	-	5298	grey	0.032767428	FALSE	PI4KB
Region_1131	chr1	151364304	151372707	8403	3	11	23.06060606	4.620618633	0.971114347	0.038794648	-	8991	grey	-0.256766442	FALSE	SELENBP1
Region_1132	chr1	151402724	151459494	56770	19	121	149.5	13.73177113	0.968627974	0.01616351	-	23126	grey	0.13453227	FALSE	POGZ
Region_1133	chr1	151510510	151538692	28182	10	63	82.65151515	9.381639063	0.918041246	0.040288853	+	57530	grey	0.231699286	FALSE	CGN
Region_1134	chr1	151540305	151583583	43278	12	84	102.7878788	8.085152872	0.935508011	0.028840877	+	7286	grey	0.041438881	FALSE	TUFT1
Region_1135	chr1	151612006	151699091	87085	24	165	193.8181818	14.47322099	0.939803444	0.019048618	+	81609	grey	-0.092197686	FALSE	SNX27
Region_1136	chr1	151700058	151716803	16745	4	12	24.93939394	4.979585364	0.580412094	0.110578954	-	11189	grey	0.113054007	FALSE	CELF3
Region_1137	chr1	15175964	15176349	3849	8	40	63.2121212	9.86604924	0.23850156	0.02432062	-	65005	grey	0.082387682	FALSE	MRPL9

		7	6				1	7	1	7						
Region_1138	chr1	151762899	151771334	8435	12	88	111.8939394	12.84718321	0.103722486	0.017023271	+	51686	grey	0.416045691	FALSE	OAZ3
Region_1139	chr1	151770107	151791416	21309	7	46	64.12121212	6.477214861	0.489780992	0.053043577	-	11022	grey	0.102590532	FALSE	TDRKH
Region_1140	chr1	151800264	151805419	5155	4	17	29.46969697	5.34133287	0.586172029	0.071580564	-	339398	grey	-0.107958038	FALSE	LINGO4
Region_1141	chr1	151806071	151831845	25774	11	67	85.71212121	9.439304328	0.726441807	0.054044399	-	6097	grey	0.003237605	FALSE	RORC
Region_1142	chr1	151837818	151840557	2739	10	39	59.8030303	10.23000067	0.008703775	0.013989992	-	100191040	grey	-0.098389545	FALSE	C2CD4D
Region_1143	chr1	151841877	151850385	8508	3	17	26.68181818	4.974269458	0.89806507	0.066346729	+	100132111	grey	0.194425442	FALSE	C2CD4D-AS1
Region_1144	chr1	151870866	151909637	38771	4	20	31.28787879	5.688761259	0.754600005	0.053496188	-	117145	grey	-0.060222465	FALSE	THEM4
Region_1145	chr1	151994531	152042774	48243	9	59	80.34848485	8.773197688	0.769851713	0.047743829	+	441908	grey	-0.147129754	FALSE	NBPF18P
Region_1146	chr1	152032506	152047907	15401	3	16	28.77272727	4.991671385	0.638345875	0.063860605	-	6282	grey	-0.164386448	FALSE	S100A11
Region_1147	chr1	152079557	152080903	1346	3	16	24.1969697	4.635049405	0.969469065	0.037879954	-	100131107	grey	0.036868047	FALSE	LOC100131107
Region_1148	chr1	152106317	152115444	9127	3	14	28.16666667	5.284471688	0.134185769	0.10833245	-	7062	grey	-0.111460388	FALSE	TCHH
Region_1149	chr1	152168125	152405050	236925	31	228	258.1969697	17.88443216	0.631438372	0.029233307	+	339400	grey	-0.212400158	FALSE	FLG-AS1

Region_1150	chr1	153174518	153191676	17158	3	19	30	5.357955982	0.94720523	0.050724142	+	101928009	grey	0.001404857	FALSE	LOC101928009
Region_1151	chr1	153606886	153613145	6259	6	27	38.04545455	5.364934061	0.711593184	0.080419371	-	140576	grey	0.290941869	FALSE	S100A16
Region_1152	chr1	153618787	153634397	15610	6	22	31.65151515	4.316310477	0.869642683	0.069330967	-	6284	grey	-0.251054776	FALSE	S100A13
Region_1153	chr1	153633982	153646306	12324	8	36	58.83333333	11.17081821	0.968129122	0.02529834	+	26097	grey	-0.399290696	FALSE	CHTOP
Region_1154	chr1	153661788	153671028	9240	7	30	44.22727273	6.616518849	0.448659421	0.037604813	-	3608	grey	-0.205808433	FALSE	ILF2
Region_1155	chr1	153678688	153693992	15304	11	78	96.78787879	10.87200378	0.820642177	0.030261276	+	4881	grey	0.02659583	FALSE	NPR1
Region_1156	chr1	153923284	153935245	11961	7	33	50.45454545	9.028470896	0.904401673	0.055156498	+	101928059	grey	-0.099749678	FALSE	LOC101928059
Region_1157	chr1	153929501	153946718	17217	19	122	147.5909091	11.29189944	0.868645068	0.02809085	-	9909	grey	-0.053692723	FALSE	DENND4B
Region_1158	chr1	153947669	153958615	10946	6	19	40.31818182	8.00607287	0.619081442	0.056972862	-	200186	grey	-0.156818891	FALSE	CRTC2
Region_1159	chr1	153959099	153975887	16788	16	85	129.9090909	16.35540586	0.141166249	0.01489787	-	27173	grey	-0.102166274	FALSE	SLC39A1
Region_1160	chr1	153967534	153974361	6827	3	16	23.53030303	4.243437275	0.092290236	0.063943044	+	148327	grey	-0.118986399	FALSE	CREB3L4
Region_1161	chr1	153975850	153986358	10508	5	18	32.46969697	5.114743988	0.458420788	0.056645071	-	5872	grey	-0.022248532	FALSE	RAB13
Region_1162	chr1	15399076	15399215	1393	3	14	24	4.93340263	0.28584633	0.06347214	+	6232	grey	0.053513185	FALSE	RPS27

		2	5					3		2						
Region_1163	chr1	153992685	154155116	162431	42	270	314.1060606	17.7106042	0.942848112	0.017986708	-	91181	grey	0.091870361	FALSE	NUP210L
Region_1164	chr1	154155304	154194648	39344	24	139	169.5909091	11.45825503	0.709155509	0.02121241	-	7170	grey	-0.017836522	FALSE	TPM3
Region_1165	chr1	154199085	154206333	7248	5	28	39.04545455	5.514824269	0.940756777	0.044694798	-	388701	grey	0.019736094	FALSE	CFAP141
Region_1166	chr1	154206696	154220637	13941	3	16	28.63636364	4.619811397	0.978782987	0.025149665	-	25912	grey	0.046044343	FALSE	C1orf43
Region_1167	chr1	154220179	154271510	51331	12	55	79.12121212	8.958906443	0.692291618	0.033908408	+	9898	grey	-0.141528344	FALSE	UBAP2L
Region_1168	chr1	154321090	154325325	4235	4	15	24.74242424	4.544855438	0.323326474	0.084318359	+	89872	grey	-0.17035362	FALSE	AQP10
Region_1169	chr1	154325525	154351304	25779	20	144	178.5606061	13.85321822	0.798553324	0.027653703	+	57198	grey	0.154453481	FALSE	ATP8B2
Region_1170	chr1	154402328	154406564	4236	8	40	59.13636364	8.057268794	0.080480126	0.028264532	-	101928101	grey	-0.142158854	FALSE	IL6R-AS1
Region_1171	chr1	154405193	154469450	64257	35	231	265.969697	15.62342393	0.713640621	0.019740608	+	3570	grey	-0.112467401	FALSE	IL6R
Region_1172	chr1	154469772	154502412	32640	16	91	106.0606061	8.437788241	0.563773515	0.046485837	-	126669	grey	0.191895225	FALSE	SHE
Region_1173	chr1	154502219	154548147	45928	36	219	261.7727273	20.10877761	0.885190515	0.018940031	+	126668	grey	-0.220668741	FALSE	TDRD10
Region_1174	chr1	154548577	154559028	10451	4	18	33.36363636	4.922902095	0.452964285	0.042050094	-	55585	grey	0.160430337	FALSE	UBE2Q1

Region_1175	chr1	154567778	154580013	12235	6	31	45.37878788	6.646607351	0.683800693	0.04487782	+	1141	grey	0.114544123	FALSE	CHRNB2
Region_1176	chr1	154581695	154628013	46318	10	62	78.28787879	8.696179801	0.889239406	0.027359788	-	103	grey	-0.174500932	FALSE	ADAR
Region_1177	chr1	154697455	154870281	172826	79	560	621.2878788	25.56967263	0.818009673	0.016571587	-	3782	grey	0.16138772	FALSE	KCNN3
Region_1178	chr1	154924740	154936719	11979	5	32	49.75757576	8.937493378	0.960901949	0.041423162	-	10654	grey	0.112209616	FALSE	PMVK
Region_1179	chr1	154944076	154956123	12047	5	26	38.13636364	5.785622402	0.855423873	0.046986367	-	57326	grey	0.138791798	FALSE	PBXIP1
Region_1180	chr1	154957026	154963853	6827	5	24	35.43939394	5.329536819	0.75313175	0.041806588	-	90780	grey	0.020929934	FALSE	PYGO2
Region_1181	chr1	154962298	154974395	12097	13	71	93.34848485	9.963768864	0.530351167	0.029639799	-	6464	grey	0.121710139	FALSE	SHC1
Region_1182	chr1	154974653	154979251	4598	8	48	63.21212121	6.958149956	0.090008928	0.027241142	+	1163	grey	-0.045848097	FALSE	CKS1B
Region_1183	chr1	154983338	154993111	9773	13	77	100.3636364	9.820913891	0.811185781	0.031547312	+	80308	grey	-0.211912993	FALSE	FLAD1
Region_1184	chr1	155002630	155018523	15893	13	43	63.13636364	7.755665648	0.380629125	0.054063082	+	51043	grey	-0.078345675	FALSE	ZBTB7B
Region_1185	chr1	155018520	155033781	15261	21	117	150.2575758	14.14791162	0.917062966	0.022951562	-	127579	grey	0.073113058	FALSE	DCST2
Region_1186	chr1	155033824	155050930	17106	10	43	59.1969697	8.482052903	0.864089606	0.043782513	+	149095	grey	0.024271867	FALSE	DCST1
Region_1187	chr1	15505056	15506277	12209	36	204	263.212121	19.0145815	0.47987775	0.01171176	+	8751	grey	0.088811568	FALSE	ADAM15

		6	5				2	7		7						
Region_1188	chr1	15506374<br>8	15508753<br>8	23790	23	111	136.939393<br>9	12.4738234<br>1	0.21734838	0.02181312<br>6	+	1944	grey	-0.03375176<br>1	FALSE	EFNA3
Region_1189	chr1	15512787<br>6	15513489<br>9	7023	3	19	26.3030303	4.32513120<br>1	0.65919799	0.02762520<br>1	+	1942	grey	0.142924138	FALSE	EFNA1
Region_1190	chr1	15513989<br>1	15514059<br>5	704	7	40	57.1060606<br>1	8.41816495<br>2	0.01151566<br>9	0.01493589<br>4	-	54344	grey	-0.50543244<br>7	FALSE	DPM3
Region_1191	chr1	15516940<br>8	15518727<br>2	17864	14	62	85.3787878<br>8	12.5939731	0.52429958<br>8	0.03158747	-	200185	grey	-0.02264884<br>1	FALSE	KRTCAP2
Region_1192	chr1	15517378<br>7	15518497<br>1	11184	8	33	50.8030303	8.44569931	0.70605554<br>3	0.04579445<br>1	+	80128	grey	0.05630229	FALSE	TRIM46
Region_1193	chr1	15518582<br>4	15519291<br>6	7092	3	12	24.0303030<br>3	5.33196369<br>4	0.78093534<br>7	0.09798917<br>6	-	4582	grey	-0.12440371<br>1	FALSE	MUC1
Region_1194	chr1	15519499<br>6	15520549<br>5	10499	5	23	35.7727272<br>7	6.39902746<br>1	0.77524527<br>4	0.06451560<br>9	+	10537145<br>0	grey	-0.01437363<br>6	FALSE	THBS3-AS1
Region_1195	chr1	15519558<br>8	15520905<br>1	13463	5	27	40.1515151<br>5	6.58493017<br>1	0.75583048<br>2	0.03110199	-	7059	grey	-0.19458825	FALSE	THBS3
Region_1196	chr1	15530874<br>8	15532066<br>6	11918	6	32	43.4393939<br>4	6.28338171<br>5	0.96311577<br>3	0.02778668<br>3	+	2224	grey	0.026720747	FALSE	FDPS
Region_1197	chr1	15531686<br>3	15532417<br>6	7313	4	14	24.9696969<br>7	4.46169921<br>7	0.25958186<br>8	0.07394734<br>7	-	284618	grey	-0.40736937<br>8	FALSE	RUSC1-AS1
Region_1198	chr1	15532089<br>4	15533111<br>4	10220	11	61	83.5454545<br>5	9.49037053<br>4	0.46432963<br>4	0.03963961	+	23623	grey	-0.27856643<br>4	FALSE	RUSC1
Region_1199	chr1	15533526<br>8	15556316<br>2	227894	71	495	541.590909<br>1	26.4511028	0.89510004<br>5	0.01247192<br>5	-	55870	grey	0.031793451	FALSE	ASH1L

Region_1200	chr1	155562026	155563944	1918	6	31	46.53030303	6.438393725	0.002440783	0.009157722	+	645676	grey	-0.104178975	FALSE	ASH1L-AS1
Region_1201	chr1	155563235	155614951	51716	23	150	178.2878788	12.64395891	0.753447336	0.018796896	+	55154	grey	0.011261405	FALSE	MSTO1
Region_1202	chr1	155659443	155689000	29557	11	82	102.1212121	8.65139411	0.756033189	0.03659124	-	55249	grey	-0.15351778	FALSE	YY1AP1
Region_1203	chr1	155687960	155739010	51050	25	168	202.8787879	16.75939704	0.928218661	0.015188385	+	7818	grey	-0.01457752	FALSE	DAP3
Region_1204	chr1	155749659	155859400	109741	42	291	325.3181818	18.79947552	0.862252213	0.020794261	-	54856	grey	-0.03708664	FALSE	GON4L
Region_1205	chr1	155859567	155885199	25632	14	72	91.63636364	9.229778502	0.831948714	0.038387527	+	23208	grey	0.394282741	FALSE	SYT11
Region_1206	chr1	155897808	155911404	13596	9	52	69.75757576	8.163823776	0.977629394	0.020624015	-	6016	grey	0.083424285	FALSE	RIT1
Region_1207	chr1	155913045	155934413	21368	16	82	107.5	10.96883698	0.773668851	0.032490556	-	22889	grey	-0.045603267	FALSE	KHDC4
Region_1208	chr1	155946851	155991212	44361	21	107	131.3787879	11.27797738	0.746501163	0.029634762	-	9181	grey	-0.123303335	FALSE	ARHGEF2
Region_1209	chr1	155946855	156002564	55709	24	122	152.530303	11.85582583	0.774537313	0.026417275	-	107985208	grey	-0.132815979	FALSE	
Region_1210	chr1	155991390	156001787	10397	3	12	21.15151515	4.050612202	0.969568245	0.039813766	+	107985210	grey	-0.06882072	FALSE	ARHGEF2-AS1
Region_1211	chr1	156001953	156033342	31389	20	130	162.7424242	15.59616383	0.867640509	0.03246798	+	105371729	grey	0.074458379	FALSE	LOC105371729
Region_1212	chr1	15600904	15602095	11903	6	35	52.6363636	9.90359121	0.94261936	0.04220091	-	6746	grey	0.117846528	FALSE	SSR2

		8	1				4	1	8	7						
Region_1213	chr1	156035299	156053798	18499	11	64	85.66666667	9.640193636	0.910096441	0.02694728	-	56893	grey	-0.006175598	FALSE	UBQLN4
Region_1214	chr1	156061160	156070504	9344	4	24	34.28787879	5.445577321	0.945818882	0.038101119	+	57111	grey	0.059199883	FALSE	RAB25
Region_1215	chr1	156082573	156140081	57508	35	206	244.8030303	17.23790073	0.712950546	0.023785656	+	4000	grey	-0.364036132	FALSE	LMNA
Region_1216	chr1	156147366	156177752	30386	16	83	109.3484848	11.54988168	0.743682902	0.037910807	+	64218	grey	-0.086329977	FALSE	SEMA4A
Region_1217	chr1	156193932	156212796	18864	16	96	125.3787879	11.39873833	0.773197163	0.022718955	+	9673	grey	0.353794722	FALSE	SLC25A44
Region_1218	chr1	156212982	156243332	30350	25	160	188.9393939	14.07548746	0.848840009	0.02086037	+	100527963	grey	0.080251709	FALSE	PMF1-BGLAP
Region_1219	chr1	156212993	156240042	27049	22	148	171.7727273	13.44481079	0.832645494	0.02167667	+	11243	grey	0.095660795	FALSE	PMF1
Region_1220	chr1	156249224	156291550	42326	15	93	114.0909091	11.21088382	0.763130486	0.029274214	-	23381	grey	0.278855388	FALSE	SMG5
Region_1221	chr1	156282935	156293185	10250	3	12	22.15151515	4.641758368	0.709997475	0.05116674	+	84283	grey	0.017287513	FALSE	TMEM79
Region_1222	chr1	156290089	156295689	5600	6	35	45.34848485	6.102943613	0.42968525	0.047262117	-	112770	grey	0.18637659	FALSE	GLMP
Region_1223	chr1	156308968	156367873	58905	31	181	206.3333333	13.46372143	0.729174005	0.025306541	-	7203	grey	-0.067696515	FALSE	CCT3
Region_1224	chr1	156337314	156346995	9681	11	52	74.15151515	9.167236685	0.521605469	0.045616637	+	128229	grey	-0.3530974	FALSE	TSACC

Region_1225	chr1	156369211	156385219	16008	11	43	66.36363636	9.772231245	0.76523999	0.031710475	+	57127	grey	-0.05481201	FALSE	RHBG
Region_1226	chr1	156404250	156456613	52363	18	115	138.5606061	12.50246596	0.488680631	0.030241969	-	10485	grey	-0.381513119	FALSE	MIR9-1HG
Region_1227	chr1	156463727	156500779	37052	18	95	119.469697	11.47468075	0.694070528	0.023571268	-	4209	grey	0.158304269	FALSE	MEF2D
Region_1228	chr1	156525405	156572604	47199	20	128	170.9242424	15.740502	0.753032643	0.017185675	-	128239	grey	-0.242076538	FALSE	IQGAP3
Region_1229	chr1	156591756	156609507	17751	14	98	123.9090909	11.57680343	0.656268959	0.029624321	+	128240	grey	-0.258751317	FALSE	NAXE
Region_1230	chr1	156594301	156601496	7195	8	55	71.59090909	9.04174468	0.678134566	0.036564312	-	54865	grey	-0.28639243	FALSE	GPATCH4
Region_1231	chr1	156641390	156659532	18142	20	99	153.4090909	18.43322859	0.295236222	0.037125989	+	63827	grey	-0.120755103	FALSE	BCAN
Region_1232	chr1	156646507	156661424	14917	21	103	162.2878788	19.36433292	0.376351309	0.036490858	-	105371453	grey	-0.034010799	FALSE	BCAN-AS1
Region_1233	chr1	156668763	156677407	8644	9	57	80.96969697	9.61084275	0.504985799	0.041241567	-	10763	grey	0.029962684	FALSE	NES
Region_1234	chr1	156699606	156705816	6210	6	26	42.66666667	7.349167056	0.250597974	0.061822118	-	1382	grey	-0.08494567	FALSE	CRABP2
Region_1235	chr1	156721891	156728766	6875	7	38	61.31818182	8.471053138	0.219596578	0.034483557	-	81875	grey	0.120385463	FALSE	ISG20L2
Region_1236	chr1	156728442	156736960	8518	3	18	30.71212121	4.519241432	0.326649849	0.017056013	+	51093	grey	-0.021169075	FALSE	METTL25B
Region_1237	chr1	15674210	15676692	24816	12	65	88.9242424	11.6754978	0.82274103	0.04562545	-	3068	grey	-0.04108951	FALSE	HDGF

		9	5				2	2	1	5				8		
Region_1238	chr1	156750610	156800815	50205	33	211	260.8484848	18.96007337	0.921872526	0.021107545	+	5546	grey	0.119723266	FALSE	PRCC
Region_1239	chr1	156806243	156816848	10605	5	31	45.93939394	6.103917501	0.682092852	0.05071405	-	9047	grey	0.135341311	FALSE	SH2D2A
Region_1240	chr1	156815640	156881850	66210	43	290	328.1212121	19.11867001	0.668743592	0.023398524	+	4914	grey	-0.123779382	FALSE	NTRK1
Region_1241	chr1	156840063	156859117	19054	12	71	91.43939394	10.14984469	0.424314525	0.05958373	-	3645	grey	0.05574557	FALSE	INSRR
Region_1242	chr1	156893698	156916429	22731	15	78	105.6363636	12.02258946	0.792951199	0.036858757	+	375033	grey	0.08457049	FALSE	PEAR1
Region_1243	chr1	156920632	156933094	12462	5	17	27.57575758	5.91493704	0.206477798	0.055814036	+	149499	grey	-0.271078046	FALSE	LRRC71
Region_1244	chr1	156934840	157045742	110902	16	104	135.530303	13.41895295	0.77548784	0.036507082	-	9826	grey	0.065566339	FALSE	ARHGEF11
Region_1245	chr1	157092043	157112412	20369	9	51	67.34848485	8.402366744	0.742334389	0.066974652	-	440695	grey	-0.084360511	FALSE	ETV3L
Region_1246	chr1	157121191	157138474	17283	5	28	42.6969697	6.92813594	0.580547186	0.034793047	-	2117	grey	0.088013698	FALSE	ETV3
Region_1247	chr1	157171116	157191963	20847	5	23	37.87878788	6.472462738	0.959918753	0.039622989	+	107985211	grey	-0.091238894	FALSE	LOC107985211
Region_1248	chr1	157513377	157552515	39138	4	22	38.37878788	6.350687792	0.724143107	0.065509504	-	83416	grey	0.17583203	FALSE	FCRL5
Region_1249	chr1	157745733	157777132	31399	3	22	33.90909091	5.6424961	0.326432616	0.039687501	-	79368	grey	0.040305179	FALSE	FCRL2

Region_1250	chr1	157830911	157898256	67345	4	23	34.83333333	6.294279047	0.920763304	0.053919986	-	922	grey	-0.199512036	FALSE	CD5L
Region_1251	chr1	157993273	158100262	106989	35	212	246.2424242	16.43173347	0.81153117	0.026947452	+	55243	grey	0.10641004	FALSE	KIRREL1
Region_1252	chr1	158131983	158149810	17827	5	33	49.95454545	7.724948231	0.634337655	0.050296499	-	646268	grey	0.19695656	FALSE	LINC01704
Region_1253	chr1	158930796	158977059	46263	5	35	50.71212121	5.790616153	0.82204088	0.04298827	+	149628	grey	0.131559713	FALSE	PYHIN1
Region_1254	chr1	158999968	159055155	55187	4	27	38.10606061	5.836565871	0.873621291	0.060590104	+	3428	grey	0.002858896	FALSE	IFI16
Region_1255	chr1	159061599	159187843	126244	14	90	120.1666667	11.069825	0.601105184	0.037522534	-	9447	grey	0.148476404	FALSE	AIM2
Region_1256	chr1	159171609	159203313	31704	3	20	31.60606061	5.734856414	0.321892326	0.072494972	+	57863	grey	0.113754363	FALSE	CADM3
Region_1257	chr1	159501664	159581497	79833	7	33	46.53030303	5.684580214	0.829115311	0.067938142	-	107985215	grey	-0.286842371	FALSE	
Region_1258	chr1	159834474	159873053	38579	13	76	94.78787879	8.836141085	0.797525886	0.028689686	-	284677	grey	0.082273688	FALSE	SNHG28
Region_1259	chr1	159854316	159873014	18698	7	40	51.1969697	6.86399681	0.664972096	0.044861513	-	391123	grey	-0.135271277	FALSE	VSIG8
Region_1260	chr1	159854870	159867685	12815	4	16	24.6969697	4.33933599	0.930269278	0.061691497	+	107985216	grey	-0.024719542	FALSE	LOC107985216
Region_1261	chr1	159872364	159900165	27801	11	72	96.07575758	8.545463225	0.896618994	0.027285203	-	25790	grey	0.177460627	FALSE	CFAP45
Region_1262	chr1	15991810	15992550	7400	4	20	31	5.47722557	0.47020654	0.04247943	-	8407	grey	0.063737054	FALSE	TAGLN2

		7	7					5		3						
Region_1263	chr1	159927039	159945613	18574	9	43	69.01515152	8.977737293	0.892236236	0.044248064	-	57549	grey	-0.258378898	FALSE	IGSF9
Region_1264	chr1	159998651	160070160	71509	16	91	118.4545455	11.29626462	0.708364874	0.033738862	-	3766	grey	-0.186304319	FALSE	KCNJ10
Region_1265	chr1	160081538	160090563	9025	3	15	24.21212121	4.77337214	0.85325142	0.07631283	+	3765	grey	-0.355211759	FALSE	KCNJ9
Region_1266	chr1	160115759	160143591	27832	7	27	42.98484848	6.962520375	0.885104309	0.060262608	+	477	grey	0.219470439	FALSE	ATP1A2
Region_1267	chr1	160151586	160186980	35394	11	73	95.22727273	8.96453775	0.799486897	0.046232988	+	480	grey	-0.129861374	FALSE	ATP1A4
Region_1268	chr1	160205380	160215376	9996	6	22	36.43939394	6.241619089	0.520907565	0.052323093	+	8682	grey	-0.080571563	FALSE	PEA15
Region_1269	chr1	160215715	160285130	69415	18	132	156.9242424	14.30794358	0.880616371	0.018255462	-	50717	grey	-0.055697989	FALSE	DCAF8
Region_1270	chr1	160240142	160286348	46206	11	74	94.92424242	10.39720466	0.871464835	0.021916069	-	5824	grey	-0.116188136	FALSE	PEX19
Region_1271	chr1	160288594	160343566	54972	15	92	119.9545455	11.05292418	0.958670856	0.020807558	-	1314	grey	0.153122994	FALSE	COPA
Region_1272	chr1	160367071	160372846	5775	3	10	19.09090909	5.223400816	0.882060504	0.114709416	+	4807	grey	0.066317447	FALSE	NHLH1
Region_1273	chr1	160400564	160428670	28106	4	17	28.96969697	5.380791183	0.785567017	0.092567577	+	57216	grey	-0.066307596	FALSE	VANGL2
Region_1274	chr1	160485030	160523262	38232	3	11	22.34848485	4.843687166	0.797311813	0.137080686	-	114836	grey	0.066625281	FALSE	SLAMF6

Region_1275	chr1	160608106	160647044	38938	8	41	55.22727273	6.676699909	0.771420096	0.040749159	-	6504	grey	0.243909964	FALSE	SLAMF1
Region_1276	chr1	160678746	160711831	33085	6	29	44.24242424	7.589589238	0.819923697	0.063233194	-	962	grey	-0.005104264	FALSE	CD48
Region_1277	chr1	160739057	160754821	15764	3	13	28.51515152	5.168670188	0.913676565	0.06087489	+	57823	grey	0.155254374	FALSE	SLAMF7
Region_1278	chr1	160796074	160828261	32187	7	41	56.24242424	6.809632558	0.93118463	0.041078866	+	4063	grey	0.100910139	FALSE	LY9
Region_1279	chr1	160830160	160862887	32727	4	20	31.31818182	5.326736888	0.981190926	0.023300326	-	51744	grey	-0.141074107	FALSE	CD244
Region_1280	chr1	160932465	160949922	17457	5	21	39.89393939	7.060461497	0.963560522	0.033254807	+	101928372	grey	0.15857565	FALSE	LOC101928372
Region_1281	chr1	160995211	161038962	43751	30	153	199.3181818	16.43182568	0.817163649	0.022153113	-	50848	grey	0.000720924	FALSE	F11R
Region_1282	chr1	161046946	161069970	23024	5	29	45.57575758	6.983630544	0.770093162	0.031417419	-	257106	grey	-0.016061902	FALSE	ARHGAP30
Region_1283	chr1	161070998	161089558	18560	9	39	59.87878788	7.584427673	0.825773523	0.053066226	-	81607	grey	-0.006474411	FALSE	NECTIN4
Region_1284	chr1	161100556	161118055	17499	10	56	76.6969697	8.807286663	0.859036224	0.035864564	-	5202	grey	-0.059838692	FALSE	PFDN2
Region_1285	chr1	161118086	161125445	7359	9	59	77.78787879	10.24547202	0.744830754	0.025679211	+	4817	grey	0.056658156	FALSE	NIT1
Region_1286	chr1	161120974	161132688	11714	8	52	72.39393939	9.065367383	0.967548097	0.025815377	-	9191	grey	0.089149212	FALSE	DEDD
Region_1287	chr1	16115945	16116572	6273	4	17	31.9090909	6.15624079	0.73570385	0.02516565	+	27005	grey	0.260045673	FALSE	USP21

		0	3				1	3	8	6						
Region_1288	chr1	161166056	161178013	11957	9	40	65	10.26120401	0.180343101	0.03209852	+	5498	grey	0.098655982	FALSE	PPOX
Region_1289	chr1	161171310	161177968	6658	5	17	33.51515152	6.286107819	0.316296893	0.055815675	-	8703	grey	0.015001133	FALSE	B4GALT3
Region_1290	chr1	161184302	161199054	14752	7	42	63.33333333	7.91752357	0.84892808	0.058963206	-	9507	grey	-0.110591595	FALSE	ADAMTS4
Region_1291	chr1	161197104	161214723	17619	8	46	62.96969697	6.985632942	0.671391065	0.040108774	+	4720	grey	-0.184151437	FALSE	NDUFS2
Region_1292	chr1	161229666	161238244	8578	6	41	54.25757576	6.703180889	0.943542345	0.043193418	-	9970	grey	-0.060699609	FALSE	NR1I3
Region_1293	chr1	161258745	161285450	26705	8	36	53.48484848	7.600085879	0.582464261	0.052152803	+	654790	grey	0.140046101	FALSE	PCP4L1
Region_1294	chr1	161314381	161363206	48825	21	138	167.9848485	12.35810842	0.934883298	0.02523219	+	6391	grey	-0.146259732	FALSE	SDHC
Region_1295	chr1	161505430	161605099	99669	16	74	113.8181818	16.0450415	0.607002312	0.038350817	+	2212	grey	-0.017239371	FALSE	FCGR2A
Region_1296	chr1	161541759	161631456	89697	15	52	102.8636364	19.54787918	0.622653108	0.033388592	-	2214	grey	0.01404535	FALSE	FCGR3A
Region_1297	chr1	161749452	161766227	16775	4	20	34.3030303	5.004520101	0.249700552	0.018118169	-	102724329	grey	-0.204029102	FALSE	ATF6-DT
Region_1298	chr1	161766298	161977574	211276	20	116	140.9090909	10.17194826	0.95719403	0.019226375	+	22926	grey	-0.199700739	FALSE	ATF6
Region_1299	chr1	161983192	162023869	40677	7	50	63.6969697	7.43374778	0.750135946	0.036261593	-	25903	grey	-0.12959695	FALSE	OLFML2B

Region_1300	chr1	162069691	162382115	312424	45	322	367.4090909	23.0837787	0.855773241	0.020453022	+	9722	grey	-0.155097409	FALSE	NOS1AP
Region_1301	chr1	162146709	162175243	28534	5	25	37.96969697	6.501998975	0.595314196	0.058773849	-	105371475	grey	0.21066386	FALSE	LOC105371475
Region_1302	chr1	162366908	162386812	19904	4	15	28.33333333	5.744116276	0.946571505	0.049482677	+	400793	grey	-0.173100407	FALSE	C1orf226
Region_1303	chr1	162395268	162412138	16870	6	29	40.62121212	6.348264818	0.950812297	0.035760138	-	117157	grey	0.073830613	FALSE	SH2D1B
Region_1304	chr1	162497251	162529631	32380	6	36	53.74242424	6.212420824	0.797806083	0.028930784	+	127933	grey	-0.332606338	FALSE	UHMK1
Region_1305	chr1	162561722	162601240	39518	6	34	47.07575758	6.608410972	0.644744548	0.037714631	+	6675	grey	-0.047629188	FALSE	UAP1
Region_1306	chr1	162631373	162787405	156032	15	105	126.469697	10.46050403	0.793178707	0.030385131	+	4921	grey	-0.096027981	FALSE	DDR2
Region_1307	chr1	162824458	162868761	44303	3	15	24.53030303	4.170296787	0.765541316	0.193253196	-	339512	grey	0.09988999	FALSE	CCDC190
Region_1308	chr1	163111121	163321894	210773	11	64	89.78787879	9.81132023	0.899294879	0.038399816	-	8490	grey	-0.028294306	FALSE	RGS5
Region_1309	chr1	163161675	163213023	51348	3	10	18	3.831047256	0.832435347	0.121982751	+	101928404	grey	0.016402791	FALSE	RGS5-AS1
Region_1310	chr1	163266576	163355764	89188	10	61	85.81818182	8.945835457	0.757559318	0.020639854	+	83540	grey	-0.020981894	FALSE	NUF2
Region_1311	chr1	164555584	164886007	330423	52	413	456.8333333	19.97427833	0.829069869	0.018862627	+	5087	grey	-0.2456562	FALSE	PBX1
Region_1312	chr1	16520186	16535671	154848	16	109	136.636363	12.0902202	0.43969811	0.02942380	-	4009	grey	0.433916974	FALSE	LMX1A

		7	5				6	9	4	8						
Region_1313	chr1	165476833	165582155	105322	13	69	105.2424242	10.98538975	0.814260828	0.039242314	-	400794	grey	-0.044131293	FALSE	LRRC52-AS1
Region_1314	chr1	165544000	165563957	19957	3	12	19.96969697	3.645733848	0.663408799	0.092934567	+	440699	grey	-0.099607203	FALSE	LRRC52
Region_1315	chr1	165631213	165661796	30583	7	56	76.04545455	9.322317008	0.768745708	0.036552806	+	4259	grey	0.078463754	FALSE	MGST3
Region_1316	chr1	165662216	165698562	36346	4	18	30.90909091	5.795826808	0.895521637	0.060239455	-	223	grey	-0.00221618	FALSE	ALDH9A1
Region_1317	chr1	165698750	165709968	11218	5	21	32.90909091	5.328377651	0.918791394	0.053443075	+	440700	grey	0.258176793	FALSE	LOC440700
Region_1318	chr1	165724293	165827755	103462	25	152	187.469697	16.67814756	0.859297025	0.023088478	-	54499	grey	0.189693565	FALSE	TMCO1
Region_1319	chr1	165827614	165911618	84004	25	177	205.0454545	14.87427497	0.868782288	0.024080276	+	7371	grey	0.168191769	FALSE	UCK2
Region_1320	chr1	166057426	166167001	109575	9	60	79.45454545	9.047195601	0.829295676	0.04793203	-	149297	grey	-0.029256315	FALSE	FAM78B
Region_1321	chr1	166165852	166340900	175048	18	117	143.7272727	9.719995971	0.907439884	0.025736146	+	112268276	grey	0.095645959	FALSE	LOC112268276
Region_1322	chr1	166839447	166856359	16912	3	20	28.53030303	4.469450825	0.957884659	0.042299173	+	57645	grey	-0.068835345	FALSE	POGK
Region_1323	chr1	166908187	166975540	67353	9	54	72	8.761805223	0.552689407	0.036149759	-	387597	grey	-0.013472142	FALSE	ILDR2
Region_1324	chr1	166975582	167022214	46632	3	15	27	5.474416021	0.632173389	0.040091922	+	84944	grey	0.332607769	FALSE	MAEL

Region_1325	chr1	167052836	167166479	113643	20	141	164.469697	13.6969617	0.869486292	0.034850997	-	10223	grey	0.063866733	FALSE	GPA33
Region_1326	chr1	167094075	167129165	35090	5	30	43.43939394	6.67746794	0.766805598	0.045648853	+	92235	grey	-0.205439642	FALSE	STYXL2
Region_1327	chr1	167175363	167195792	20429	7	34	46.86363636	5.885801334	0.959911003	0.041593327	-	101928484	grey	-0.280228412	FALSE	LINC01363
Region_1328	chr1	167220876	167427345	206469	24	187	216.8181818	13.46437932	0.903618423	0.016390251	+	5451	grey	0.283805844	FALSE	POU2F1
Region_1329	chr1	167425027	167518640	93613	36	270	309.2727273	18.95788487	0.779209312	0.029233774	-	919	grey	0.30688287	FALSE	CD247
Region_1330	chr1	167455195	167458646	3451	4	22	35.66666667	4.733947234	0.40141585	0.04542844	+	101928512	grey	-0.041501782	FALSE	LOC101928512
Region_1331	chr1	167529117	167553805	24688	7	50	66.43939394	8.237118219	0.654597764	0.039973392	-	107985224	grey	0.101163709	FALSE	LOC107985224
Region_1332	chr1	167541013	167553762	12749	4	23	35.93939394	5.984284002	0.43865942	0.05841677	-	8804	grey	-0.048320068	FALSE	CREG1
Region_1333	chr1	167630093	167708696	78603	29	214	248.1060606	17.57281886	0.819654776	0.023790085	+	92241	grey	-0.033820822	FALSE	RCSD1
Region_1334	chr1	167721950	167791919	69969	16	89	111.030303	10.80878517	0.897241137	0.025280569	+	9019	grey	-0.098062962	FALSE	MPZL1
Region_1335	chr1	167809386	167914215	104829	25	184	216.5606061	12.80520181	0.820936609	0.027870331	-	55811	grey	0.227337293	FALSE	ADCY10
Region_1336	chr1	167916675	167937072	20397	4	18	27.3030303	4.889453948	0.252049376	0.024261828	-	25874	grey	0.030847226	FALSE	MPC2
Region_1337	chr1	16793578	16807584	140060	20	137	166.212121	14.8515890	0.80738509	0.02408392	+	55827	grey	0.033724867	FALSE	DCAF6

		3	3				2	4	9							
Region_1338	chr1	168079542	168137667	58125	23	167	200.3939394	16.22522138	0.852840379	0.037855924	-	23432	grey	-0.148823426	FALSE	GPR161
Region_1339	chr1	168178962	168202109	23147	6	31	48.01515152	7.484327058	0.957898117	0.033096046	+	261726	grey	-0.123489089	FALSE	TIPRL
Region_1340	chr1	168280877	168314426	33549	7	32	54.21212121	8.649615646	0.771605223	0.072138489	+	9095	grey	-0.144022382	FALSE	TBX19
Region_1341	chr1	168400829	168495685	94856	7	42	62.65151515	9.43722975	0.784636956	0.031376914	-	100505918	grey	-0.023363107	FALSE	LOC100505918
Region_1342	chr1	168417663	168495692	78029	5	27	45.01515152	7.882780502	0.885225728	0.036183283	-	101928565	grey	-0.014851099	FALSE	LOC101928565
Region_1343	chr1	168695468	169103276	407808	35	239	292.7727273	19.85314969	0.931097134	0.020201522	-	1805	grey	-0.291413366	FALSE	DPT
Region_1344	chr1	168903905	169087005	183100	18	111	152.2878788	13.24882015	0.926832824	0.027257225	-	101978719	grey	-0.239437983	FALSE	LINC00970
Region_1345	chr1	169105697	169310992	205295	14	87	108.6363636	11.65086509	0.725530895	0.027571526	+	481	grey	0.119986729	FALSE	ATP1B1
Region_1346	chr1	169132531	169367948	235417	12	68	95.04545455	12.61919395	0.956536536	0.023682321	-	29922	grey	-0.052646378	FALSE	NME7
Region_1347	chr1	169394870	169460669	65799	3	10	17.92424242	3.767515427	0.928157126	0.111966794	-	57821	grey	0.147251639	FALSE	CCDC181
Region_1348	chr1	169511951	169586588	74637	4	17	30	5.457528322	0.634144737	0.053578352	-	2153	grey	0.037697054	FALSE	F5
Region_1349	chr1	169588849	169630193	41344	4	20	39.09090909	9.403317373	0.966200786	0.04245168	-	6403	grey	0.211681388	FALSE	SELP

Region_1350	chr1	169662007	169854080	192073	17	123	150.2878788	11.89927619	0.882962629	0.03121302	+	55732	grey	-0.49260343	FALSE	FIRRM
Region_1351	chr1	169733577	169764705	31128	3	12	22.5	4.924819403	0.927602173	0.06529808	-	101928628	grey	0.0464513	FALSE	LOC101928628
Region_1352	chr1	169849631	169894267	44636	9	63	83.33333333	10.04043109	0.909279339	0.048640172	-	57147	grey	-0.399908831	FALSE	SCYL3
Region_1353	chr1	169921326	170085208	163882	3	10	19.33333333	4.178086913	0.951262262	0.044137705	-	22920	grey	0.06591313	FALSE	KIFAP3
Region_1354	chr1	170173865	170304358	130493	6	26	42.59090909	6.811703216	0.897443724	0.0540027	+	105371609	grey	-0.297154795	FALSE	LINC01681
Region_1355	chr1	170460453	170532647	72194	7	48	66.18181818	8.15599655	0.814720418	0.044279128	-	101928650	grey	0.335765771	FALSE	GORAB-AS1
Region_1356	chr1	170662728	170739421	76693	11	80	97.10606061	9.143520258	0.425334229	0.031931032	+	5396	grey	-0.15570277	FALSE	PRRX1
Region_1357	chr1	170748573	171251857	503284	27	187	223.5909091	15.21939782	0.869827559	0.029727744	-	105371611	grey	0.30331021	FALSE	LOC105371611
Region_1358	chr1	170935526	171064765	129239	6	26	40.92424242	7.093465262	0.906009822	0.060033799	+	80133	grey	0.037527565	FALSE	MROH9
Region_1359	chr1	171137740	171161568	23828	3	10	22.24242424	4.867666021	0.91203895	0.053441511	+	388714	grey	-0.019569527	FALSE	FMO6P
Region_1360	chr1	171314183	171342084	27901	5	32	42.48484848	7.256280938	0.966531152	0.034194399	+	2329	grey	-0.170077051	FALSE	FMO4
Region_1361	chr1	171485530	171593511	107981	15	91	112.6666667	9.195874214	0.857257743	0.02199178	+	23215	grey	-0.219714758	FALSE	PRRC2C
Region_1362	chr1	17160062	17163879	38178	9	51	72.4848484	8.54353993	0.89145414	0.04353813	+	11080629	grey	-0.02839676	FALSE	MYOCOS

		1	9				8	3	1	9		0		8		
Region_1363	chr1	171635417	171652688	17271	3	12	24.09090909	5.53235872	0.89241384	0.103548313	-	4653	grey	-0.131769161	FALSE	MYOC
Region_1364	chr1	171700160	171742074	41914	7	43	59.16666667	9.08690238	0.811808209	0.042544913	-	8674	grey	-0.151343053	FALSE	VAMP4
Region_1365	chr1	171781660	171814023	32363	7	40	59.37878788	7.560555843	0.937043482	0.045686787	+	51603	grey	-0.297705587	FALSE	METTL13
Region_1366	chr1	171817887	172418466	600579	39	299	332.5	18.29859179	0.861751041	0.018254279	+	26052	grey	-0.345691355	FALSE	DNM3
Region_1367	chr1	172370189	172444083	73894	3	17	26.3030303	5.047374632	0.754197518	0.144949252	-	102724528	grey	-0.356206538	FALSE	LOC102724528
Region_1368	chr1	172420685	172468831	48146	3	21	31.09090909	5.235168861	0.963758866	0.038504543	+	92346	grey	-0.053511807	FALSE	C1orf105
Region_1369	chr1	172532349	172611833	79484	9	49	66.46969697	8.038789991	0.598839284	0.032216306	+	51430	grey	-0.205488181	FALSE	SUCO
Region_1370	chr1	173183731	173489407	305676	14	101	131.6515152	11.10854477	0.74473355	0.026651064	-	7292	grey	-0.349572076	FALSE	TNFSF4
Region_1371	chr1	173417793	173461362	43569	4	21	35.45454545	5.804989414	0.727924318	0.045503649	-	101928673	grey	-0.116158015	FALSE	PRDX6-AS1
Region_1372	chr1	173500460	173603072	102612	6	53	71.24242424	8.577359475	0.95254385	0.030875239	-	284525	grey	-0.00775017	FALSE	SLC9C2
Region_1373	chr1	173555251	173637130	81879	4	31	42.57575758	5.662455586	0.95603016	0.040063872	+	730159	grey	0.01068224	FALSE	TEX50
Region_1374	chr1	173596060	173669851	73791	3	13	24.63636364	5.088860727	0.813619899	0.079377479	-	339416	grey	-0.218109979	FALSE	ANKRD45

Region_1375	chr1	173714941	173786692	71751	13	72	99.62121212	10.79139993	0.899217714	0.016016173	+	27252	grey	0.044567473	FALSE	KLHL20
Region_1376	chr1	173799550	173824883	25333	7	42	59.24242424	9.387488574	0.557926723	0.023570827	-	91687	grey	0.025462212	FALSE	CENPL
Region_1377	chr1	173824653	173858808	34155	14	91	117.7121212	10.56666042	0.736249894	0.030230092	+	55157	grey	0.301411758	FALSE	DARS2
Region_1378	chr1	173858559	173868882	10323	5	30	44.22727273	6.032719643	0.507755868	0.060231908	-	60674	grey	0.0921991	FALSE	GAS5
Region_1379	chr1	173868082	173903547	35465	5	29	42.04545455	5.993411301	0.793703413	0.04715268	+	84614	grey	-0.006614659	FALSE	ZBTB37
Region_1380	chr1	173931084	174022357	91273	25	148	184.6212121	13.05345831	0.948321948	0.020434089	-	149041	grey	0.05919575	FALSE	RC3H1
Region_1381	chr1	174110268	174160165	49897	18	110	135.0757576	14.40973027	0.533989318	0.03021936	-	102724601	grey	-0.041585814	FALSE	RABGAP1L-DT
Region_1382	chr1	174159410	174995308	835898	72	541	604.9393939	23.17351193	0.856041792	0.012330443	+	9910	grey	-0.058045375	FALSE	RABGAP1L
Region_1383	chr1	174999163	175012027	12864	8	49	65.87878788	7.80239638	0.385951473	0.034599328	+	27101	grey	-0.095744642	FALSE	CACYBP
Region_1384	chr1	175067833	175148075	80242	16	101	123.3787879	11.42300668	0.825273484	0.044364366	+	63923	grey	-0.131814201	FALSE	TNN
Region_1385	chr1	175156986	175192999	36013	3	19	28.83333333	5.642103627	0.960258414	0.039823261	-	9674	grey	-0.106996652	FALSE	KIAA0040
Region_1386	chr1	175307218	175335459	28241	3	18	29.06060606	5.54666577	0.928948386	0.048224339	+	105371623	grey	0.143546591	FALSE	LOC105371623
Region_1387	chr1	17531519	17574361	428422	45	366	405.348484	20.8441044	0.85107733	0.01754662	-	7143	grey	-0.02618923	FALSE	TNR

		4	6				8	4	5	2				6		
Region_1388	chr1	175904762	175921057	16295	4	19	29.24242424	4.575378835	0.897447659	0.080418936	-	107985228	grey	-0.203695245	FALSE	LINC02803
Region_1389	chr1	175944831	176207286	262455	26	162	200.0909091	15.57683415	0.929025258	0.01949	-	64326	grey	-0.069435968	FALSE	COP1
Region_1390	chr1	176463171	176845601	382430	24	183	209.2121212	14.59453549	0.798605538	0.032155062	+	60676	grey	0.144753359	FALSE	PAPPA2
Region_1391	chr1	176857302	177164973	307671	34	270	304.2575758	20.53463162	0.811591289	0.026067463	-	460	grey	-0.071491431	FALSE	ASTN1
Region_1392	chr1	177170958	177282422	111464	15	105	128.9545455	12.0568293	0.750155691	0.046667684	+	57795	grey	0.109283427	FALSE	BRINP2
Region_1393	chr1	177351560	177459759	108199	11	69	89.54545455	8.875518305	0.739379949	0.038620091	+	102724661	grey	-0.036366503	FALSE	LINC01645
Region_1394	chr1	177927566	177984303	56737	12	80	105.5	10.72201187	0.863375053	0.02862402	-	89866	grey	0.178830852	FALSE	SEC16B
Region_1395	chr1	177928788	178038007	109219	18	134	160.4090909	12.00509798	0.890699258	0.022604394	-	111240474	grey	0.133314605	FALSE	CRYZL2P-SEC16B
Region_1396	chr1	178006136	178037950	31814	3	10	23.75757576	5.491421579	0.937511745	0.06825454	-	730102	grey	0.047058824	FALSE	CRYZL2P
Region_1397	chr1	178094104	178484147	390043	40	314	357.8030303	22.5260326	0.883566631	0.017528638	+	9462	grey	-0.044589391	FALSE	RASAL2
Region_1398	chr1	178479240	178499634	20394	6	32	49.10606061	7.664360655	0.940145409	0.031958457	-	400797	grey	-0.000286135	FALSE	CLEC20A
Region_1399	chr1	178513109	178548602	35493	5	23	40.16666667	6.286942105	0.740971626	0.043066021	+	84066	grey	0.098692532	FALSE	TEX35

Region_1400	chr1	178725165	178921842	196677	20	122	162.6515152	12.76352064	0.865186	0.018635649	+	55103	grey	0.012153927	FALSE	RALGPS2
Region_1401	chr1	179025804	179076567	50763	22	148	179.2878788	14.12228479	0.898181514	0.024834272	+	9917	grey	0.140537791	FALSE	FAM20B
Region_1402	chr1	179082070	179098023	15953	6	30	43.96969697	5.994791834	0.801023373	0.037578389	+	64222	grey	-0.116862365	FALSE	TOR3A
Region_1403	chr1	179099330	179229684	130354	38	237	286.2575758	18.52259378	0.78919195	0.019868185	-	27	grey	-0.06119529	FALSE	ABL2
Region_1404	chr1	179293714	179358680	64966	18	127	151.5606061	13.68562276	0.834459355	0.022020631	+	6646	grey	0.056037905	FALSE	SOAT1
Region_1405	chr1	179365720	179554735	189015	26	176	214.7727273	17.11930575	0.790622533	0.025401257	+	126859	grey	0.24608583	FALSE	AXDND1
Region_1406	chr1	179550539	179575952	25413	5	29	39.90909091	5.620641279	0.833580061	0.068562726	-	7827	grey	0.38566646	FALSE	NPHS2
Region_1407	chr1	179591613	179691272	99659	10	54	76.90909091	10.66947807	0.878321637	0.044803614	+	163589	grey	-0.162136327	FALSE	TDRD5
Region_1408	chr1	179730191	179816198	86007	24	172	201.1969697	13.85998079	0.756160719	0.018570887	+	148753	grey	-0.046864634	FALSE	FAM163A
Region_1409	chr1	179839967	179877803	37836	11	67	85.90909091	8.490224737	0.800922769	0.035375952	-	163590	grey	-0.304053722	FALSE	TOR1AIP2
Region_1410	chr1	179882042	179920077	38035	7	48	63.43939394	6.307818806	0.805877396	0.055077071	+	26092	grey	-0.272303965	FALSE	TOR1AIP1
Region_1411	chr1	179926641	179941796	15155	6	37	51.98484848	6.833159927	0.924479841	0.039509642	-	101928933	grey	0.239368278	FALSE	LOC101928933
Region_1412	chr1	17995477	18011487	160102	21	134	155.954545	12.5188249	0.96209246	0.01816502	+	9857	grey	0.233548373	FALSE	CEP350

		3	5				5	9	3	4						
Region_1413	chr1	180154869	180204030	49161	31	162	211.1515152	14.9071407	0.895218776	0.02786492	+	5768	grey	0.002970316	FALSE	QSOX1
Region_1414	chr1	180230264	180278984	48720	21	124	166.9848485	14.01262335	0.355225381	0.018749056	+	89884	grey	-0.274341785	FALSE	LHX4
Region_1415	chr1	180269653	180502954	233301	20	152	176.1666667	11.68112657	0.90342872	0.017674104	-	84320	grey	-0.096490118	FALSE	ACBD6
Region_1416	chr1	180509380	180566523	57143	10	63	86.78787879	9.040958342	0.888187401	0.023518413	+	148756	grey	0.062021449	FALSE	OVAAL
Region_1417	chr1	180632022	180890279	258257	26	193	221.4393939	14.02539322	0.892566736	0.019875799	+	9213	grey	-0.279662206	FALSE	XPR1
Region_1418	chr1	180912897	180951614	38717	31	208	243.8939394	16.80671	0.846570535	0.021541969	+	57710	grey	-0.301882302	FALSE	KIAA1614
Region_1419	chr1	180949699	180954887	5188	5	23	38.5	7.79990138	0.291736344	0.06990725	-	103344928	grey	0.099251502	FALSE	KIAA1614-AS1
Region_1420	chr1	180972712	181023121	50409	5	32	46.04545455	6.819501038	0.927787113	0.036865311	-	10228	grey	-0.016611222	FALSE	STX6
Region_1421	chr1	181033374	181061938	28564	9	54	66.71212121	7.879408708	0.956586308	0.028464684	+	3140	grey	-0.017849311	FALSE	MR1
Region_1422	chr1	181088700	181092900	4200	3	20	29.72727273	5.433642933	0.017863188	0.029284201	+	51278	grey	0.075424196	FALSE	IER5
Region_1423	chr1	181317690	181813262	495572	57	433	471.7121212	22.06128886	0.807084503	0.020339062	+	777	grey	0.011004121	FALSE	CACNA1E
Region_1424	chr1	182096338	182329213	232875	34	231	267.1818182	18.38218962	0.848932227	0.021423186	-	400799	grey	-0.044060077	FALSE	LINC01344

Region_1425	chr1	182409192	182560599	151407	17	113	140.7424242	11.64911434	0.895137487	0.029750022	+	353299	grey	-0.186290452	FALSE	RGSL1
Region_1426	chr1	182641816	182684587	42771	5	27	43.68181818	6.337092493	0.938100828	0.041662581	-	85397	grey	0.294191	FALSE	RGS8
Region_1427	chr1	182789293	182830384	41091	13	56	77.59090909	9.47868422	0.727261425	0.030173795	+	80896	grey	0.08956208	FALSE	NPL
Region_1428	chr1	182839347	182887982	48635	8	45	64.01515152	8.409687499	0.841350348	0.037147953	+	1660	grey	-0.228530538	FALSE	DHX9
Region_1429	chr1	182899865	182953525	53660	17	103	125	11.85424299	0.887776671	0.037658866	-	81626	grey	0.001696379	FALSE	SHCBP1L
Region_1430	chr1	183023420	183145592	122172	25	177	209.3030303	16.39696942	0.74845562	0.032243335	+	3915	grey	-0.038210523	FALSE	LAMC1
Region_1431	chr1	183186238	183245127	58889	13	74	100.030303	9.596425131	0.749018202	0.029139344	+	3918	grey	0.244221846	FALSE	LAMC2
Region_1432	chr1	183248237	183418380	170143	25	157	186.5454545	15.09883523	0.926136021	0.024456727	-	23057	grey	-0.021890793	FALSE	NMNAT2
Region_1433	chr1	183472216	183598246	126030	16	118	142.0757576	11.52563778	0.683953468	0.03272358	+	9887	grey	-0.286484036	FALSE	SMG7
Region_1434	chr1	183554461	183590905	36444	5	35	51.95454545	7.324210261	0.623684235	0.047589673	-	4688	grey	-0.253882153	FALSE	NCF2
Region_1435	chr1	183636085	183928532	292447	41	283	330.2272727	17.14534739	0.81040465	0.020208136	+	23179	grey	-0.0755085	FALSE	RGL1
Region_1436	chr1	183929854	184037729	107875	15	95	126.4090909	13.06313341	0.884620851	0.025303122	-	23127	grey	-0.182828473	FALSE	COLGALT2
Region_1437	chr1	18405172	18412397	72256	4	14	24.4848484	4.53445756	0.69183080	0.05340424	+	10272483	grey	-0.12628812	FALSE	LOC102724830

		2	8				8		8	5		0		2		
Region_1438	chr1	184387029	184629019	241990	23	166	203.530303	14.52977812	0.911842637	0.026689654	+	81563	grey	0.055057852	FALSE	C1orf21
Region_1439	chr1	184539993	184592248	52255	10	55	84.16666667	9.461799037	0.882221021	0.040137312	+	102724857	grey	0.067019626	FALSE	
Region_1440	chr1	184607599	184669603	62004	11	73	95.54545455	12.80166073	0.777880399	0.051158022	-	107985236	grey	-0.302226365	FALSE	LOC107985236
Region_1441	chr1	184690237	184754907	64670	5	37	50.81818182	7.312600486	0.628823974	0.036089488	-	80267	grey	-0.029205734	FALSE	EDEM3
Region_1442	chr1	184790724	184974508	183784	20	116	133.3030303	11.19611155	0.861774875	0.018939977	-	116496	grey	0.09010105	FALSE	NIBAN1
Region_1443	chr1	185045526	185102603	57077	17	92	129.1060606	14.46707539	0.842884692	0.020580249	+	6045	grey	-0.073470663	FALSE	RNF2
Region_1444	chr1	185118101	185157072	38971	5	29	42.74242424	6.320444498	0.568116696	0.024332449	-	81627	grey	0.041032747	FALSE	TRMT1L
Region_1445	chr1	185157080	185291781	134701	11	70	94.1969697	8.588399505	0.875755329	0.018831253	+	54823	grey	0.336332451	FALSE	SWT1
Region_1446	chr1	185558371	185628944	70573	5	31	42.68181818	6.488235723	0.740258456	0.056118204	-	101929093	grey	0.204564006	FALSE	LINC01350
Region_1447	chr1	185734391	186190949	456558	31	267	302.2575758	16.93272717	0.861560405	0.026910347	+	83872	grey	0.108698194	FALSE	HMCN1
Region_1448	chr1	186311652	186375693	64041	6	33	49.54545455	6.87958366	0.495614879	0.014511634	-	7175	grey	-0.011585888	FALSE	TPR
Region_1449	chr1	186423481	186521147	97666	7	41	60.24242424	7.49576415	0.957554998	0.038719273	+	102724919	grey	-0.043815641	FALSE	PDC-AS1

Region_1450	chr1	186828949	186988981	160032	19	126	156.4545455	12.9652575	0.877433003	0.039864214	+	5321	grey	0.058283963	FALSE	PLA2G4A
Region_1451	chr1	187070700	187686130	615430	41	303	344.8484848	18.87794189	0.870023594	0.026678576	+	106144535	grey	-0.021928564	FALSE	LINC01037
Region_1452	chr1	187072513	187480614	408101	26	177	210.3787879	14.20267055	0.864441835	0.03344501	+	104169671	grey	-0.174653644	FALSE	LINC01036
Region_1453	chr1	187228587	187330505	101918	9	61	74.66666667	7.440705784	0.882402174	0.040870003	+	105371655	grey	-0.023552598	FALSE	LOC105371655
Region_1454	chr1	190097658	190477864	380206	18	131	157.0606061	11.63929523	0.856829684	0.028056101	-	339479	grey	0.14340047	FALSE	BRINP3
Region_1455	chr1	190264898	190362270	97372	6	31	45.54545455	6.640625129	0.819534475	0.05642141	+	105371658	grey	0.180258713	FALSE	LOC105371658
Region_1456	chr1	190624890	190801658	176768	5	27	38.62121212	5.945302351	0.744231005	0.091162873	+	440704	grey	0.149511968	FALSE	LINC01720
Region_1457	chr1	191823432	192011260	187828	8	51	68.06060606	8.477805935	0.836928382	0.065610899	+	107985457	grey	0.114314326	FALSE	LINC02770
Region_1458	chr1	192158462	192185815	27353	3	12	20.92424242	3.884131845	0.958938714	0.044977966	+	64407	grey	0.254226294	FALSE	RGS18
Region_1459	chr1	192167786	192392914	225128	9	60	78.39393939	8.172898531	0.925581926	0.035391497	-	105371662	grey	0.321471452	FALSE	LOC105371662
Region_1460	chr1	192387632	192925804	538172	50	362	402.9848485	22.18002735	0.88466266	0.014626788	-	105371664	grey	-0.289495544	FALSE	LOC105371664
Region_1461	chr1	192491452	192925752	434300	42	293	335.0151515	20.45369436	0.883549635	0.015693476	-	105371665	grey	-0.261420248	FALSE	LOC105371665
Region_1462	chr1	19260935	19263454	25185	3	13	23.8636363	5.05929871	0.92366647	0.05798655	+	10798524	grey	0.012805228	FALSE	LOC107985241

		9	4				6	6	1			1				
Region_1463	chr1	192835405	192955037	119632	14	92	118.4848485	10.91539833	0.938400075	0.026178438	-	105371667	grey	-0.141464203	FALSE	LOC105371667
Region_1464	chr1	192935739	192957713	21974	5	31	44.81818182	6.527831754	0.925591941	0.038803858	-	102724954	grey	0.216495714	FALSE	RGS2-AS1
Region_1465	chr1	193012250	193060080	47830	7	36	57.01515152	6.904830916	0.558370144	0.01483337	-	51377	grey	0.082197408	FALSE	UCHL5
Region_1466	chr1	193059454	193091777	32323	7	33	50.12121212	7.455540328	0.68738844	0.030525092	+	6738	grey	-0.244645119	FALSE	RO60
Region_1467	chr1	193090866	193106114	15248	3	17	30.37878788	6.847268184	0.312110584	0.038662848	-	51022	grey	0.188602248	FALSE	GLRX2
Region_1468	chr1	193121983	193254815	132832	12	75	100.030303	10.02453866	0.898837588	0.022343849	+	79577	grey	0.033933916	FALSE	CDC73
Region_1469	chr1	193304745	193365953	61208	4	21	36.89393939	5.530694178	0.955155266	0.037085134	+	101929184	grey	-0.015802933	FALSE	LINC01031
Region_1470	chr1	196225779	196609225	383446	21	152	180.6969697	13.51349149	0.821146972	0.022409203	-	343450	grey	0.247813583	FALSE	KCNT2
Region_1471	chr1	196651754	196795393	143639	12	54	82.93939394	10.67113834	0.914213121	0.037564218	+	3075	grey	-0.139364634	FALSE	CFH
Region_1472	chr1	196975010	197009678	34668	6	36	50.03030303	6.867918052	0.862488218	0.04898134	+	81494	grey	-0.029640042	FALSE	CFHR5
Region_1473	chr1	197084121	197146694	62573	10	65	87.27272727	9.570058202	0.702698205	0.025907277	-	259266	grey	0.117071535	FALSE	ASPM
Region_1474	chr1	197153682	197201293	47611	3	17	28.90909091	5.459578098	0.931072618	0.054253402	-	360023	grey	-0.150109119	FALSE	ZBTB41

Region_1475	chr1	197268204	197478455	210251	16	87	112.5151515	11.08740439	0.922670194	0.031125182	+	23418	grey	-0.042267947	FALSE	CRB1
Region_1476	chr1	197504748	197775696	270948	23	154	190.3787879	14.74268022	0.786696405	0.017980081	-	163486	grey	0.18250439	FALSE	DENND1B
Region_1477	chr1	197911902	197935478	23576	8	39	54.1969697	6.344518402	0.260233269	0.037478921	+	56956	grey	0.148016623	FALSE	LHX9
Region_1478	chr1	198156994	198322420	165426	12	74	91.28787879	8.034091115	0.808283973	0.024319833	+	140609	grey	0.186365951	FALSE	NEK7
Region_1479	chr1	198450666	198519877	69211	4	23	36.65151515	4.678896728	0.909801783	0.062494508	-	105371677	grey	0.357828069	FALSE	LOC105371677
Region_1480	chr1	198777861	198937471	159610	13	92	110.9242424	9.43367227	0.900151561	0.04523722	-	100131234	grey	-0.170485778	FALSE	MIR181A1HG
Region_1481	chr1	199148598	199393307	244709	12	84	106.2272727	10.7270979	0.899488335	0.041840752	+	400800	grey	-0.183055143	FALSE	LINC02789
Region_1482	chr1	200027614	200177420	149806	24	176	216.2424242	16.9343171	0.707966533	0.032581864	+	2494	grey	0.064044602	FALSE	NR5A2
Region_1483	chr1	200253419	200400705	147286	28	219	253.7424242	17.7229192	0.909706035	0.023452952	-	554279	grey	0.036776346	FALSE	LINC00862
Region_1484	chr1	200404940	200410056	5116	4	12	21.39393939	5.471264199	0.003823954	0.013789086	-	23528	grey	0.103755069	FALSE	ZNF281
Region_1485	chr1	200551497	200620751	69254	11	54	70.18181818	7.822864104	0.970034102	0.02407105	-	9928	grey	0.134152481	FALSE	KIF14
Region_1486	chr1	200623896	200669907	46011	8	44	55.36363636	6.833483994	0.691708722	0.044118424	-	83479	grey	0.169462981	FALSE	DDX59
Region_1487	chr1	20066950	20069425	24743	5	21	33.3787878	6.73398990	0.78020804	0.05301524	+	10192922	grey	0.239826646	FALSE	DDX59-AS1

		7	0				8	5		9		4				
Region_1488	chr1	200738893	200860704	121811	18	102	133.9848485	14.4791547	0.82397438	0.030517378	+	23271	grey	0.045412768	FALSE	CAMSAP2
Region_1489	chr1	200872981	200874178	1197	4	29	44.93939394	8.701686464	0.091518021	0.059008115	+	2848	grey	-0.072487329	FALSE	GPR25
Region_1490	chr1	200891048	200915742	24694	9	52	66.71212121	7.806831627	0.743391518	0.037787969	+	55765	grey	0.039017488	FALSE	INAVA
Region_1491	chr1	200917460	200966668	49208	17	108	125.969697	9.907215237	0.902106362	0.032541507	+	647215	grey	-0.158056642	FALSE	MROH3P
Region_1492	chr1	200969390	201023714	54324	30	195	234.5757576	17.58851077	0.797352782	0.021623661	-	23046	grey	-0.247356098	FALSE	KIF21B
Region_1493	chr1	201023949	201043073	19124	8	41	54.43939394	7.038637954	0.829322262	0.040155849	+	101929305	grey	0.045452564	FALSE	LOC101929305
Region_1494	chr1	201039512	201112451	72939	22	137	156.9545455	10.04211412	0.887205473	0.033146075	-	779	grey	0.239126984	FALSE	CACNA1S
Region_1495	chr1	201113943	201127184	13241	6	29	42.45454545	6.334719515	0.482819436	0.052406421	-	647219	grey	-0.160628303	FALSE	ASCL5
Region_1496	chr1	201134772	201171574	36802	12	57	81.42424242	10.04614064	0.718408111	0.040597762	-	252839	grey	0.182976043	FALSE	TMEM9
Region_1497	chr1	201190824	201228952	38128	13	71	87.71212121	8.085628564	0.87097917	0.050060652	+	91156	grey	-0.047093553	FALSE	IGFN1
Region_1498	chr1	201283452	201332993	49541	17	119	140.1212121	11.89378008	0.704515778	0.033919028	+	5317	grey	-0.114615675	FALSE	PKP1
Region_1499	chr1	201359008	201382254	23246	6	26	43.13636364	6.876686062	0.659122507	0.072954105	-	7139	grey	0.186923861	FALSE	TNNT2

Region_1500	chr1	201373244	201399915	26671	11	55	77.42424242	9.752412887	0.507834078	0.044247627	-	3898	grey	0.076563653	FALSE	LAD1
Region_1501	chr1	201399633	201401190	1557	4	15	27.56060606	5.43246307	0.503051883	0.099589499	+	101929343	grey	0.02632373	FALSE	LOC101929343
Region_1502	chr1	201403768	201429866	26098	9	57	69.92424242	7.907757839	0.732168985	0.05450337	-	7135	grey	0.245562665	FALSE	TNNI1
Region_1503	chr1	201464278	201469237	4959	5	21	31.33333333	5.090967366	0.454313763	0.063168308	-	23612	grey	-0.06542122	FALSE	PHLDA3
Region_1504	chr1	201483530	201509456	25926	11	61	83.63636364	9.015604188	0.909615822	0.03380151	-	1465	grey	-0.138617624	FALSE	CSRP1
Region_1505	chr1	201507241	201534784	27543	9	55	79.12121212	9.086786944	0.887502962	0.042515725	+	107985246	grey	-0.020608737	FALSE	CSRP1-AS1
Region_1506	chr1	201539127	201826969	287842	58	337	410.4393939	23.94489108	0.779525747	0.020038485	+	89796	grey	-0.042684347	FALSE	NAV1
Region_1507	chr1	201688259	201829559	141300	26	170	199.3787879	14.93242744	0.849292839	0.017897711	-	100873949	grey	0.012559586	FALSE	IPO9-AS1
Region_1508	chr1	201829149	201884291	55142	14	105	126.9242424	10.78219416	0.959107922	0.01850794	+	55705	grey	0.278294958	FALSE	IPO9
Region_1509	chr1	201896456	201946588	50132	16	97	114.3939394	9.821293645	0.814607199	0.03998042	-	25802	grey	-0.406478562	FALSE	LMOD1
Region_1510	chr1	201955503	201970664	15161	13	66	100.0909091	11.46865598	0.819159157	0.023343577	+	10440	grey	0.027663938	FALSE	TIMM17A
Region_1511	chr1	201982372	202006147	23775	17	92	115.1060606	11.57600806	0.724862564	0.022995867	+	6051	grey	-0.030411513	FALSE	RNPEP
Region_1512	chr1	20199569	20201046	14767	9	46	58.4090909	7.09057288	0.83772952	0.03233630	-	10272346	grey	0.031942805	FALSE	ELF3-AS1

		6	3				1	1	4	3		5				
Region_1513	chr1	20200794<br>5	20201718<br>3	9238	4	16	26.4545454<br>5	5.13863740<br>7	0.67074226<br>8	0.08090215<br>2	+	1999	grey	0.082689919	FALSE	ELF3
Region_1514	chr1	20212288<br>6	20213359<br>2	10706	8	54	67.3030303	8.13533545<br>6	0.80520961<br>3	0.04817486<br>1	+	9283	grey	0.135096811	FALSE	GPR37L1
Region_1515	chr1	20213340<br>4	20214474<br>3	11339	6	31	47.4545454<br>5	7.65241403<br>5	0.64830840<br>6	0.03331363<br>6	-	127829	grey	0.006389388	FALSE	ARL8A
Region_1516	chr1	20214701<br>3	20216158<br>8	14575	7	34	47.6969697	7.25358202<br>6	0.48205341<br>3	0.05366401	-	5778	grey	-0.06502368<br>9	FALSE	PTPN7
Region_1517	chr1	20216805<br>1	20218945<br>5	21404	11	58	79.2727272<br>7	8.95724108<br>4	0.89526630<br>6	0.04127611<br>2	+	148713	grey	-0.25169464<br>3	FALSE	PTPRVP
Region_1518	chr1	20219379<br>9	20231978<br>1	125982	42	290	329.818181<br>8	20.3892196<br>9	0.80642234<br>9	0.02032318	+	59352	blue	0.531629938	FALSE	LGR6
Region_1519	chr1	20233154<br>4	20234198<br>4	10440	3	19	25.7575757<br>6	4.21009996<br>6	0.79692812<br>2	0.06267026<br>2	-	29089	grey	-0.22816159<br>5	FALSE	UBE2T
Region_1520	chr1	20234869<br>9	20259270<br>6	244007	24	158	189.212121<br>2	13.1961709<br>5	0.85317944<br>2	0.02088818<br>3	+	4660	grey	-0.00635941<br>6	FALSE	PPP1R12B
Region_1521	chr1	20259059<br>6	20271045<br>4	119858	46	334	384.833333<br>3	21.2542001<br>9	0.79813084<br>7	0.02022307<br>9	-	127833	grey	0.114036439	FALSE	SYT2
Region_1522	chr1	20272449<br>5	20280848<br>7	83992	23	141	166.439393<br>9	12.6893399<br>1	0.71554170<br>1	0.02035831<br>6	-	10765	grey	-0.04494359<br>5	FALSE	KDM5B
Region_1523	chr1	20282026<br>6	20282722<br>5	6959	3	19	33.7727272<br>7	5.11346775<br>2	0.72538061<br>1	0.06693629	+	641515	grey	-0.00394228<br>4	FALSE	MGAT4EP
Region_1524	chr1	20286175<br>4	20287524<br>1	13487	6	30	47.0303030<br>3	6.73445719<br>8	0.78690487<br>3	0.04287783<br>1	+	148709	grey	0.187219519	FALSE	ACTG1P25

Region_1525	chr1	202891116	202928636	37520	20	100	132.4545455	14.77230446	0.503462865	0.019323679	-	59349	grey	0.127180268	FALSE	KLHL12
Region_1526	chr1	202940826	202958572	17746	6	24	40.48484848	7.29223491	0.268448611	0.060269891	-	51094	grey	-0.086146877	FALSE	ADIPOR1
Region_1527	chr1	203007374	203024848	17474	6	27	43.74242424	5.263256103	0.970577146	0.025272498	+	92703	grey	0.313979431	FALSE	TMEM183A
Region_1528	chr1	203026491	203078740	52249	16	77	96.24242424	9.59977661	0.746598895	0.033573894	+	8497	grey	-0.267829861	FALSE	PPFIA4
Region_1529	chr1	203090654	203167405	76751	20	127	151.6363636	11.6204546	0.788855794	0.02854886	+	134	grey	-0.127040581	FALSE	ADORA1
Region_1530	chr1	203167811	203175826	8015	5	27	40.53030303	5.508649693	0.906520767	0.047314534	-	4608	grey	-0.345571661	FALSE	MYBPH
Region_1531	chr1	203178931	203186704	7773	3	14	23.89393939	4.38924836	0.949965799	0.051600729	-	1116	grey	-0.189013493	FALSE	CHI3L1
Region_1532	chr1	203212827	203272809	59982	10	68	81.07575758	7.516772233	0.699660193	0.049998276	-	1118	grey	0.083207857	FALSE	CHIT1
Region_1533	chr1	203273221	203288804	15583	8	52	85.37878788	9.693400057	0.404437446	0.04540795	+	100506775	grey	-0.003749683	FALSE	LINC01353
Region_1534	chr1	203475806	203491352	15546	9	48	68.90909091	9.758195408	0.673779045	0.035597754	+	5549	grey	0.039216985	FALSE	PRELP
Region_1535	chr1	203626787	203744081	117294	31	177	209.7121212	15.9622296	0.678448134	0.02070887	+	493	grey	-0.064896828	FALSE	ATP2B4
Region_1536	chr1	203765177	203776372	11195	6	28	51.89393939	7.082217792	0.748094701	0.048249468	+	54900	grey	0.01694736	FALSE	LAX1
Region_1537	chr1	20379562	20385412	58501	15	79	104.363636	11.7061934	0.84291967	0.03097572	+	9877	grey	-0.30049176	FALSE	ZC3H11A

		3	4				4	5	9	6				9		
Region_1538	chr1	203795623	203854999	59376	15	79	104.3636364	11.70619345	0.842919679	0.030975726	+	100381270	grey	-0.300491769	FALSE	ZBED6
Region_1539	chr1	203996532	204008930	12398	3	12	22.56060606	4.845707975	0.940634856	0.065376747	+	105371689	grey	0.122274654	FALSE	LOC105371689
Region_1540	chr1	204073115	204127743	54628	33	203	242.2272727	16.90952891	0.772299699	0.013868123	+	9580	grey	-0.042131496	FALSE	SOX13
Region_1541	chr1	204131062	204152044	20982	11	41	61.40909091	9.528866706	0.449036438	0.054662022	-	55224	grey	-0.119703976	FALSE	ETNK2
Region_1542	chr1	204154819	204190324	35505	10	58	79.59090909	10.45131905	0.956017877	0.032419528	-	5972	grey	-0.110594029	FALSE	REN
Region_1543	chr1	204198163	204213988	15825	4	27	38.86363636	7.09688201	0.951745602	0.037904541	-	127845	grey	-0.007311792	FALSE	GOLT1A
Region_1544	chr1	204218853	204377665	158812	49	351	395.4848485	22.79894898	0.765828092	0.019785393	-	22874	grey	-0.318913973	FALSE	PLEKHA6
Region_1545	chr1	204396492	204411887	15395	3	20	28.87878788	5.641917709	0.329686298	0.023518791	-	84919	grey	0.160753995	FALSE	PPP1R15B
Region_1546	chr1	204422628	204494805	72177	37	255	295.7878788	18.90259661	0.760334931	0.020021375	-	5287	grey	0.036508758	FALSE	PIK3C2B
Region_1547	chr1	204516379	204558120	41741	12	67	91.75757576	9.638162299	0.887020235	0.031343888	+	4194	grey	0.256690865	FALSE	MDM4
Region_1548	chr1	204603035	204629712	26677	7	47	62.01515152	8.014395556	0.952172599	0.035912425	+	105371692	grey	0.112484539	FALSE	LOC105371692
Region_1549	chr1	204617170	204685738	68568	25	170	212.3484848	16.61740739	0.781901709	0.024540457	-	10446	grey	0.150529548	FALSE	LRRN2

Region_1550	chr1	204828651	205022822	194171	56	382	432.1969697	22.84948725	0.822278929	0.019768309	+	23114	grey	0.084631149	FALSE	NFASC
Region_1551	chr1	205042937	205078289	35352	9	42	62.39393939	8.172898531	0.938165616	0.034110284	+	6900	grey	-0.278461168	FALSE	CNTN2
Region_1552	chr1	205086142	205122015	35873	8	38	56.74242424	7.642614547	0.838132752	0.025958802	-	5929	grey	-0.037052564	FALSE	RBBP5
Region_1553	chr1	205142505	205211702	69197	41	260	301.9090909	18.69243144	0.829137595	0.015250694	-	25778	grey	0.065514633	FALSE	DSTYK
Region_1554	chr1	205227946	205273343	45397	24	143	173.9090909	16.44265305	0.753397589	0.021828769	+	9911	grey	0.190862085	FALSE	TMCC2
Region_1555	chr1	205302063	205321745	19682	7	40	62.07575758	8.858391252	0.720638495	0.031807632	-	81788	grey	-0.052554148	FALSE	NUAK2
Region_1556	chr1	205336061	205357090	21029	7	36	49.66666667	6.318065267	0.666051742	0.036864416	-	55220	grey	0.315776792	FALSE	KLHDC8A
Region_1557	chr1	205373252	205387440	14188	4	20	36.72727273	7.195180982	0.93735353	0.042962977	+	284576	grey	0.009287492	FALSE	LEMD1-AS1
Region_1558	chr1	205381378	205455954	74576	22	125	156.8181818	13.72690686	0.657257314	0.032174033	-	93273	grey	0.180798559	FALSE	LEMD1
Region_1559	chr1	205434885	205457091	22206	15	64	86.95454545	10.7165319	0.278331881	0.040826003	-	101669762	grey	0.106741804	FALSE	BLACAT1
Region_1560	chr1	205455929	205469024	13095	8	36	61.81818182	8.465148626	0.367175881	0.032772215	+	284577	grey	-0.205496252	FALSE	LEMD1-DT
Region_1561	chr1	205504596	205532793	28197	5	21	33.18181818	5.835986743	0.479202879	0.063388581	+	5129	grey	-0.022429582	FALSE	CDK18
Region_1562	chr1	20556888	20560291	34033	17	108	131.545454	11.5973230	0.71553697	0.03097154	+	148808	grey	0.41221711	FALSE	MFSD4A

		5	8				5	6	6	3						
Region_1563	chr1	205597556	205632011	34455	16	94	121.2878788	13.1731255	0.642893114	0.028791305	-	2005	grey	0.154123814	FALSE	ELK4
Region_1564	chr1	205657851	205680509	22658	16	92	115.4848485	10.05713283	0.769065937	0.038385087	-	85414	grey	-0.025435362	FALSE	SLC45A3
Region_1565	chr1	205712822	205750182	37360	7	53	70.13636364	7.980526999	0.683987266	0.038126007	-	64710	grey	-0.253171755	FALSE	NUCKS1
Region_1566	chr1	205767986	205775482	7496	5	21	35.16666667	7.111384877	0.590498752	0.049475604	-	8934	grey	0.228683587	FALSE	RAB29
Region_1567	chr1	205775559	205783623	8064	5	26	38.16666667	5.864058278	0.926590838	0.058962278	+	105371702	grey	0.487750367	FALSE	
Region_1568	chr1	205813322	205896082	82760	10	68	87.37878788	10.06252018	0.806126631	0.031195472	+	284581	grey	0.058030099	FALSE	PM20D1-AS1
Region_1569	chr1	206052845	206102449	49604	4	20	32.53030303	6.478888074	0.75377784	0.04288518	+	440712	grey	-0.046768998	FALSE	RHEX
Region_1570	chr1	206106936	206117699	10763	5	21	31.71212121	6.128784925	0.539717561	0.057911572	-	553	grey	0.117140285	FALSE	AVPR1B
Region_1571	chr1	206203346	206464436	261090	9	48	62.8030303	7.690593049	0.952534126	0.025477229	+	23380	grey	-0.361516089	FALSE	SRGAP2
Region_1572	chr1	206507531	206589448	81917	12	73	98.03030303	10.49097736	0.772484353	0.045024069	+	83593	grey	-0.051419297	FALSE	RASSF5
Region_1573	chr1	206571292	206612465	41173	8	53	70.57575758	7.569291413	0.934327178	0.025058259	-	1939	grey	0.156082769	FALSE	EIF2D
Region_1574	chr1	206634184	206635714	1530	6	28	39.92424242	7.336548352	0.006943407	0.013308877	-	105372876	grey	-0.03720796	FALSE	DYRK3-AS1

Region_1575	chr1	206635536	206684419	48883	16	98	123.5909091	10.57279131	0.696467962	0.02070179	+	8444	grey	0.247368503	FALSE	DYRK3
Region_1576	chr1	206684905	206734281	49376	10	66	87.78787879	10.86917328	0.90767224	0.044972115	+	9261	grey	0.001308549	FALSE	MAPKAPK2
Region_1577	chr1	206770764	206842981	72217	16	121	142.2272727	11.17248744	0.743821319	0.032285953	+	29949	grey	0.03635758	FALSE	IL19
Region_1578	chr1	206903317	206923247	19930	5	32	48.18181818	7.208774762	0.507459687	0.049820872	-	9214	grey	-0.083267673	FALSE	FCMR
Region_1579	chr1	206928522	206946466	17944	6	36	52.18181818	6.961231303	0.921420976	0.050551731	-	5284	grey	0.075098892	FALSE	PIGR
Region_1580	chr1	207034366	207081024	46658	7	39	55.48484848	8.035005001	0.697527296	0.022813692	+	5208	grey	-0.147389358	FALSE	PFKFB2
Region_1581	chr1	207034416	207062014	27598	5	25	39.89393939	7.415428382	0.588780913	0.027724175	+	107984109	grey	0.109903372	FALSE	
Region_1582	chr1	207088860	207099993	11133	4	30	43.59090909	6.216546834	0.938318471	0.042113517	+	725	grey	0.154709751	FALSE	C4BPB
Region_1583	chr1	207104233	207144972	40739	9	50	65.25757576	7.931069588	0.836636265	0.05962288	+	722	grey	0.132093701	FALSE	C4BPA
Region_1584	chr1	207321519	207386804	65285	6	25	40.37878788	6.541618599	0.816317538	0.046493617	+	1604	grey	0.127776131	FALSE	CD55
Region_1585	chr1	207453024	207489895	36871	3	18	29.53030303	5.695395433	0.641623197	0.031796239	+	1380	grey	-0.034424351	FALSE	CR2
Region_1586	chr1	207496147	207641765	145618	18	111	144.8787879	13.43533247	0.940943844	0.027798739	+	1378	grey	-0.217836815	FALSE	CR1
Region_1587	chr1	20764511	20773841	93303	22	171	205.924242	17.6968581	0.52487222	0.03117186	+	1379	grey	-0.26446873	FALSE	CR1L

		3	6				4	4	2	1				5		
Region_1588	chr1	207752037	207795513	43476	10	63	85.09090909	9.015759318	0.675340637	0.022797892	+	4179	grey	0.298970757	FALSE	CD46
Region_1589	chr1	207801518	207879096	77578	23	128	162.1363636	14.94285151	0.683497148	0.035239515	-	100128537	grey	0.201050522	FALSE	MIR29B2CHG
Region_1590	chr1	207880972	207911402	30430	7	37	49.92424242	7.500380721	0.410713003	0.040684074	-	947	grey	-0.202822919	FALSE	CD34
Region_1591	chr1	207959292	207969329	10037	3	13	23.51515152	4.817412086	0.02095413	0.034715661	+	105372883	grey	0.03554795	FALSE	LINC02767
Region_1592	chr1	208022242	208244384	222142	61	443	505.7878788	28.81907229	0.863606312	0.017200463	-	5362	grey	0.241388518	FALSE	PLXNA2
Region_1593	chr1	209147267	209266758	119491	9	63	78.34848485	7.463742976	0.781733264	0.037032617	-	105372896	grey	0.067732881	FALSE	LOC105372896
Region_1594	chr1	209528455	209567673	39218	4	21	34.77272727	6.019955161	0.93910293	0.048288085	-	105372898	grey	-0.020026711	FALSE	LOC105372898
Region_1595	chr1	209583714	209613939	30225	3	16	26.75757576	4.651089553	0.927797751	0.062840165	+	57172	grey	-0.076134376	FALSE	CAMK1G
Region_1596	chr1	209614870	209652425	37555	7	38	55.8030303	6.325089715	0.813550662	0.047452145	-	3914	grey	0.277236269	FALSE	LAMB3
Region_1597	chr1	209661356	209724125	62769	3	14	24.63636364	4.793060251	0.831855097	0.08073224	-	101930114	grey	0.068993601	FALSE	HSD11B1-AS1
Region_1598	chr1	209686178	209734949	48771	3	14	27.74242424	5.762773387	0.835875053	0.074768633	+	3290	grey	0.009332548	FALSE	HSD11B1
Region_1599	chr1	209756032	209782320	26288	3	21	32.90909091	5.184970211	0.662669991	0.068459862	+	80342	grey	-0.140200679	FALSE	TRAF3IP3

Region_1600	chr1	209779208	209806179	26971	7	43	59.27272727	7.818214344	0.436898865	0.028321941	-	148304	grey	0.092152129	FALSE	C1orf74
Region_1601	chr1	209785617	209806175	20558	5	27	37.92424242	6.02127154	0.609388657	0.039292829	-	3664	grey	0.068149781	FALSE	IRF6
Region_1602	chr1	209827972	209857565	29593	6	24	46.89393939	6.879465068	0.717882728	0.072028996	+	27042	grey	0.013297007	FALSE	UTP25
Region_1603	chr1	209900923	210171389	270466	32	211	251.530303	15.45735543	0.709019119	0.024982033	+	255928	grey	0.060669961	FALSE	SYT14
Region_1604	chr1	210676823	211188533	511710	54	380	436.5909091	20.49313376	0.881912498	0.022459238	-	3756	grey	0.189066957	FALSE	KCNH1
Region_1605	chr1	211258377	211316385	58008	5	16	31.36363636	5.760973111	0.972296744	0.034777961	+	55758	grey	0.036041139	FALSE	RCOR3
Region_1606	chr1	211326615	211374946	48331	5	25	43.10606061	7.192151252	0.626161879	0.049893214	+	7188	grey	0.097425352	FALSE	TRAF5
Region_1607	chr1	211382736	211435333	52597	25	166	196.4090909	16.98504072	0.84937563	0.032476294	+	84791	grey	0.125143528	FALSE	LINC00467
Region_1608	chr1	211571568	211579161	7593	4	24	34.66666667	4.79850404	0.472716492	0.04426283	-	7779	grey	0.147074475	FALSE	SLC30A1
Region_1609	chr1	211658657	211675630	16973	8	50	72.12121212	8.927942569	0.94720244	0.043456735	-	4751	grey	0.220938842	FALSE	NEK2
Region_1610	chr1	211675749	211687459	11710	3	20	30.59090909	5.291436544	0.61198052	0.049594962	+	91548	grey	-0.194861946	FALSE	NEK2-DT
Region_1611	chr1	211743457	211830763	87306	23	143	176.8030303	16.09034082	0.900380129	0.027431893	-	9926	grey	-0.138288859	FALSE	LPGAT1
Region_1612	chr1	21182963	21185370	24067	5	29	39.7727272	5.53810702	0.75622605	0.04139968	+	10272372	grey	-0.14962608	FALSE	LPGAT1-AS1

		6	3				7	1	5	9		7				
Region_1613	chr1	211940399	212035557	95158	9	51	72.36363636	9.213094968	0.959445979	0.024128811	-	25896	grey	-0.030592245	FALSE	INTS7
Region_1614	chr1	212035553	212107400	71847	13	108	130.0757576	10.9899403	0.588375834	0.031288644	+	51514	grey	0.17977664	FALSE	DTL
Region_1615	chr1	212180141	212285084	104943	9	61	77	8.593378488	0.87727757	0.042463477	-	101929541	grey	0.05190389	FALSE	LINC02608
Region_1616	chr1	212285410	212361853	76443	15	74	106.4242424	10.43818523	0.691526461	0.028534965	+	5525	grey	-0.162630057	FALSE	PPP2R5A
Region_1617	chr1	212363928	212414901	50973	12	67	86.33333333	8.931075107	0.942916574	0.033687732	-	55248	grey	-0.040736641	FALSE	PACC1
Region_1618	chr1	212565334	212620775	55441	22	157	185.969697	11.44077687	0.561745876	0.021874877	+	467	grey	-0.302889616	FALSE	ATF3
Region_1619	chr1	212653305	212665638	12333	5	37	51.13636364	6.206414458	0.746955461	0.032975757	-	102723761	grey	0.067613942	FALSE	LINC02773
Region_1620	chr1	212686417	212699840	13423	4	12	26.09090909	5.535138863	0.343935361	0.063548422	-	55509	grey	-0.116245649	FALSE	BATF3
Region_1621	chr1	212726153	212791782	65629	16	112	136.3939394	11.19186351	0.7176325	0.036530625	-	25936	grey	0.047030897	FALSE	NSL1
Region_1622	chr1	212791828	212816830	25002	6	27	42.40909091	7.148915513	0.97365308	0.028513967	+	128387	grey	0.069563175	FALSE	TATDN3
Region_1623	chr1	212830141	212847649	17508	4	12	26.40909091	6.251097806	0.819927254	0.064603297	-	149643	grey	0.176540819	FALSE	SPATA45
Region_1624	chr1	212858275	212899363	41088	17	97	129.8484848	12.34284897	0.864575338	0.019676525	+	28982	grey	-0.003695316	FALSE	FLVCR1

Region_1625	chr1	212950520	212991585	41065	8	49	64.54545455	6.913046347	0.81926967	0.045445546	+	79805	grey	0.128445736	FALSE	VASH2
Region_1626	chr1	212992182	213015867	23685	11	74	95.68181818	8.980592906	0.787631015	0.017462666	-	90806	grey	0.071345832	FALSE	ANGEL2
Region_1627	chr1	213051233	213274774	223541	29	206	236.8484848	14.93806951	0.921904196	0.016468555	+	26750	grey	0.471925874	FALSE	RPS6KC1
Region_1628	chr1	213492288	213794587	302299	30	223	251.030303	14.95940116	0.853932899	0.032269671	+	105372912	grey	-0.116374781	FALSE	
Region_1629	chr1	213817751	213988508	170757	10	63	84.1969697	8.798985426	0.621444271	0.036589491	-	100505832	grey	-0.232853193	FALSE	PROX1-AS1
Region_1630	chr1	213983181	214041510	58329	14	96	119.3333333	10.49883998	0.713817632	0.03321045	+	5629	grey	0.23462582	FALSE	PROX1
Region_1631	chr1	214051194	214187773	136579	20	146	174.8030303	12.36025852	0.796140429	0.033828407	-	105372911	grey	0.05880852	FALSE	LINC02775
Region_1632	chr1	214281102	214337131	56029	14	100	139.2121212	11.59909168	0.89185784	0.022218211	+	56950	grey	-0.263326465	FALSE	SMYD2
Region_1633	chr1	214348700	214552449	203749	45	311	367.8636364	20.83701319	0.83673789	0.02158868	-	5784	grey	-0.045734676	FALSE	PTPN14
Region_1634	chr1	214603195	214664571	61376	13	87	105.6818182	8.679168856	0.954249718	0.026514242	+	1063	grey	-0.173786487	FALSE	CENPF
Region_1635	chr1	215005775	215237090	231315	22	148	177.8939394	13.97374328	0.695372144	0.034295263	+	3776	grey	0.111403676	FALSE	KCNK2
Region_1636	chr1	215567304	215621807	54503	6	20	33.57575758	5.854808953	0.648615	0.037771802	+	51133	grey	-0.013867499	FALSE	KCTD3
Region_1637	chr1	21562289	21642344	800557	70	549	592.348484	24.8109730	0.81966439	0.01914179	-	7399	grey	-0.07163879	FALSE	USH2A

		1	8				8	2	4	1						
Region_1638	chr1	216503246	217137755	634509	53	400	485.5454545	22.7786552	0.765264599	0.021383895	-	2104	grey	0.040783783	FALSE	ESRRG
Region_1639	chr1	217426992	217631090	204098	16	114	136.5909091	11.96016114	0.938572199	0.023791977	-	55105	grey	-0.132391744	FALSE	GPATCH2
Region_1640	chr1	217631324	217871696	240372	23	153	183.4242424	14.71792273	0.839067051	0.022644614	+	128153	grey	0.155156076	FALSE	SPATA17
Region_1641	chr1	217892900	217920804	27904	4	17	29.3030303	6.109185258	0.892917766	0.074337304	+	100885798	grey	0.224369142	FALSE	LINC00210
Region_1642	chr1	218043505	218059140	15635	4	24	38.57575758	5.291678826	0.70404263	0.11827467	+	101929631	grey	-0.062420811	FALSE	LINC01653
Region_1643	chr1	218285293	218337983	52690	7	35	51.1969697	6.249233053	0.952339961	0.031100395	+	51018	grey	0.043960849	FALSE	RRP15
Region_1644	chr1	218345336	218444619	99283	13	84	107.3636364	12.29963897	0.753375858	0.035983239	+	7042	grey	-0.333476671	FALSE	TGFB2
Region_1645	chr1	218459265	218525978	66713	7	50	66.89393939	8.21092853	0.91530195	0.035256464	+	440714	grey	-0.253623003	FALSE	LINC02869
Region_1646	chr1	218976672	219173961	197289	7	47	60.62121212	7.202061994	0.858710896	0.049910461	-	643723	grey	-0.052565909	FALSE	LYPLAL1-DT
Region_1647	chr1	219173869	219297668	123799	3	20	29.43939394	4.527280685	0.940970549	0.047578026	+	127018	grey	0.075842265	FALSE	LYPLAL1
Region_1648	chr1	219685427	219958647	273220	30	203	238.7424242	14.20975895	0.774286279	0.027224882	-	105372926	grey	-0.023985364	FALSE	LOC105372926
Region_1649	chr1	219910445	219959018	48573	11	69	93.8030303	9.935514693	0.703527406	0.036056506	-	55532	grey	0.18665937	FALSE	SLC30A10

Region_1650	chr1	219968600	220046530	77930	5	24	35.22727273	5.620827901	0.976695437	0.027081583	-	2058	grey	0.275111932	FALSE	EPRS1
Region_1651	chr1	220057482	220090462	32980	8	47	65.16666667	6.835615629	0.947094605	0.036205549	-	10380	grey	-0.193303865	FALSE	BPNT1
Region_1652	chr1	220094132	220148041	53909	18	113	146.2727273	13.53575943	0.967258054	0.017731274	+	55699	grey	0.102836919	FALSE	IARS2
Region_1653	chr1	220148293	220272529	124236	18	126	155.1212121	12.44002863	0.843958899	0.038572222	-	25782	grey	0.083068724	FALSE	RAB3GAP2
Region_1654	chr1	220528136	220664461	136325	11	86	106.4242424	8.35014621	0.906428289	0.030623263	+	4139	grey	0.117936256	FALSE	MARK1
Region_1655	chr1	220786352	220884503	98151	22	163	193.6060606	13.40479683	0.459869828	0.020377683	+	64757	grey	0.131369732	FALSE	MTARC1
Region_1656	chr1	220832763	220880140	47377	15	107	131.6060606	10.97672893	0.580559023	0.024845109	-	100873924	grey	0.202737367	FALSE	HLX-AS1
Region_1657	chr1	220879443	220885059	5616	4	14	34.65151515	7.320135401	0.074907876	0.057545103	+	3142	grey	0.043233641	FALSE	HLX
Region_1658	chr1	221555550	221563653	8103	3	25	36.93939394	5.656524586	0.902548863	0.052283343	-	105372937	grey	0.246495576	FALSE	LOC105372937
Region_1659	chr1	221701424	221742089	40665	7	50	69.87878788	8.271387478	0.730244538	0.034461377	-	11221	grey	0.023282957	FALSE	DUSP10
Region_1660	chr1	221819842	221840717	20875	3	17	28.42424242	5.162171893	0.801432582	0.091398153	-	101929771	grey	-0.03931013	FALSE	LINC01655
Region_1661	chr1	221880981	221978523	97542	13	92	113.7878788	10.70659775	0.921502084	0.036960703	-	105372950	grey	0.012130156	FALSE	LINC02257
Region_1662	chr1	22201082	22206477	53948	9	62	77.0303030	7.67607611	0.83263796	0.05979396	-	10798530	grey	0.042465468	FALSE	LINC01705

		5	3				3	5	3	1		7				
Region_1663	chr1	222486060	222548104	62044	8	25	56.31818182	9.114922829	0.938622426	0.040023037	-	79802	grey	0.045457677	FALSE	HHIPL2
Region_1664	chr1	222618097	222668007	49910	19	126	158	16.83128954	0.549946132	0.019840578	+	375056	grey	-0.145763709	FALSE	MIA3
Region_1665	chr1	222668013	222713210	45197	7	45	58.78787879	6.625162179	0.69556402	0.020003333	-	64853	grey	0.110208118	FALSE	AIDA
Region_1666	chr1	222712553	222735196	22643	7	37	50.21212121	7.252617886	0.697910311	0.021847278	+	148362	grey	-0.020508056	FALSE	BROX
Region_1667	chr1	222743356	222773149	29793	3	19	27.95454545	4.763424167	0.878032051	0.071631837	-	105372984	grey	0.050925305	FALSE	LOC105372984
Region_1668	chr1	222815022	223005995	190973	27	152	189.6212121	14.79788462	0.913993959	0.023074481	+	84976	grey	-0.042296429	FALSE	DISP1
Region_1669	chr1	223109404	223143248	33844	7	48	61.53030303	8.545299557	0.56127092	0.02835878	-	7100	grey	-0.064110651	FALSE	TLR5
Region_1670	chr1	223220819	223364233	143414	32	230	256.4848485	12.78249094	0.824254393	0.020811355	-	55061	grey	0.359297023	FALSE	SUSD4
Region_1671	chr1	223538007	223665723	127716	17	106	141.2878788	12.4650467	0.893956608	0.019918586	-	388743	grey	-0.023810863	FALSE	CAPN8
Region_1672	chr1	223701593	223776018	74425	28	145	170.8333333	12.28831006	0.724093324	0.02527913	+	824	grey	-0.421407406	FALSE	CAPN2
Region_1673	chr1	223779893	223845954	66061	10	59	77.96969697	8.298229092	0.683222934	0.043502661	-	7159	grey	0.189828416	FALSE	TP53BP2
Region_1674	chr1	223951479	223992603	41124	6	16	31.81818182	6.166682417	0.946429082	0.062326665	-	441124	grey	-0.213858937	FALSE	GTF2IP20

Region_1675	chr1	22399274<br>3	22401011<br>6	17373	5	25	41.3939393<br>9	6.11147417<br>2	0.96898800<br>3	0.03301256<br>4	+	10028749<br>7	grey	0.224814056	FALSE	SEPTIN7P13
Region_1676	chr1	22411411<br>1	22416204<br>7	47936	11	74	92.1363636<br>4	9.25350056<br>6	0.96747684<br>5	0.02371151<br>6	+	23219	grey	0.262955528	FALSE	FBXO28
Region_1677	chr1	22417575<br>6	22419344<br>1	17685	15	80	109.045454<br>5	14.2833219<br>8	0.76183086<br>4	0.03802406<br>5	+	8560	grey	0.062191988	FALSE	DEGS1
Region_1678	chr1	22421961<br>3	22422804<br>3	8430	4	14	27.2575757<br>6	4.75656827<br>2	0.87839015<br>5	0.10143140<br>9	+	10192716<br>4	grey	-0.13997544<br>5	FALSE	LOC101927164
Region_1679	chr1	22422733<br>4	22433018<br>9	102855	22	131	158.272727<br>3	14.8836983<br>1	0.80652248	0.03989287<br>2	-	4931	grey	0.011241497	FALSE	NVL
Region_1680	chr1	22435685<br>8	22437945<br>9	22601	14	85	118.409090<br>9	12.7785605	0.86803795<br>4	0.02126249<br>7	+	29097	grey	-0.39705574<br>9	FALSE	CNIH4
Region_1681	chr1	22438514<br>6	22443703<br>3	51887	23	135	180.530303	15.5456842<br>4	0.73921148<br>9	0.02538029<br>3	-	80232	grey	-0.06055465<br>6	FALSE	WDR26
Region_1682	chr1	22443466<br>0	22474055<br>4	305894	91	754	820.166666<br>7	31.3901421<br>7	0.81542996<br>7	0.01337696<br>5	+	149111	grey	0.050428601	FALSE	CNIH3
Region_1683	chr1	22470327<br>2	22473066<br>2	27390	6	43	61.6818181<br>8	7.73037782<br>1	0.92920493<br>1	0.03899393<br>8	-	10537310<br>6	grey	0.036244172	FALSE	CNIH3-AS1
Region_1684	chr1	22480295<br>9	22488135<br>5	78396	13	83	111.409090<br>9	11.5398670<br>5	0.92131072	0.03089006<br>9	-	10537310<br>8	grey	-0.11774802<br>1	FALSE	LOC105373108
Region_1685	chr1	22489626<br>2	22539929<br>2	503030	36	236	275.166666<br>7	15.7866383	0.87673894<br>1	0.02208824<br>1	+	127602	grey	-0.17550511<br>5	FALSE	DNAH14
Region_1686	chr1	22540150<br>2	22542892<br>5	27423	4	29	39.8333333<br>3	4.79930550<br>5	0.73644066<br>8	0.04536107<br>7	-	3930	grey	-0.04923390<br>4	FALSE	LBR
Region_1687	chr1	22544723	22546540	18167	3	15	23.8181818	4.26401432	0.75423710	0.11524580	-	10537311	grey	-0.00077878	FALSE	LINC02765

		3	0				2	7	7	1		0		1		
Region_1688	chr1	22548676<br>5	22565314<br>2	166377	43	258	297.030303	21.2971429<br>6	0.90035077<br>4	0.01585321<br>9	-	55740	grey	0.153935798	FALSE	ENAH
Region_1689	chr1	22570026<br>4	22575225<br>8	51994	10	58	79.0303030<br>3	8.89938579<br>9	0.93180992<br>7	0.04956096<br>7	+	10272383<br>4	grey	-0.54519880<br>9	FALSE	LOC102723834
Region_1690	chr1	22581012<br>4	22584556<br>3	35439	16	87	114.015151<br>5	11.5138611<br>1	0.96356179<br>5	0.01937299<br>9	+	2052	grey	-0.28629053<br>4	FALSE	EPHX1
Region_1691	chr1	22584088<br>3	22588238<br>0	41497	22	135	174.636363<br>6	13.4446287<br>4	0.89776375<br>8	0.01837947<br>2	-	9725	grey	-0.02002660<br>9	FALSE	TMEM63A
Region_1692	chr1	22588628<br>2	22592427<br>8	37996	18	109	135.621212<br>1	11.5422503<br>5	0.94866224<br>3	0.01897576<br>4	-	10637	grey	0.074142311	FALSE	LEFTY1
Region_1693	chr1	22591987<br>7	22592434<br>0	4463	4	18	31.3787878<br>8	5.94012471<br>1	0.69370898<br>8	0.04786206<br>7	-	29920	grey	0.303020163	FALSE	PYCR2
Region_1694	chr1	22593659<br>8	22594138<br>3	4785	4	25	38.6212121<br>2	5.83560728<br>2	0.96366836<br>6	0.03580624<br>8	-	7044	grey	0.037003956	FALSE	LEFTY2
Region_1695	chr1	22598270<br>2	22599934<br>3	16641	18	97	126.803030<br>3	13.4772968	0.71840105<br>7	0.03995335<br>5	-	163859	grey	-0.11095617<br>2	FALSE	SDE2
Region_1696	chr1	22606185<br>1	22607201<br>9	10168	11	56	75.1212121<br>2	8.40423913<br>5	0.60197256<br>6	0.04238539<br>5	+	3020	grey	-0.15099187<br>1	FALSE	H3-3A
Region_1697	chr1	22608359<br>0	22609046<br>5	6875	9	43	60.3939393<br>9	6.84077995<br>5	0.61461795<br>4	0.03200800<br>4	+	10537311<br>3	grey	0.016366778	FALSE	LINC01703
Region_1698	chr1	22614467<br>9	22618674<br>1	42062	14	79	102	9.13488663<br>6	0.79986755<br>4	0.02457524	-	64746	grey	0.048854249	FALSE	ACBD3
Region_1699	chr1	22623114<br>9	22630986<br>9	78720	54	326	377.818181<br>8	22.4245120<br>6	0.59265085<br>1	0.01393905<br>6	-	286826	grey	0.156523217	FALSE	LIN9

Region_1700	chr1	226360210	226408154	47944	19	128	150.6060606	12.69267358	0.813113621	0.015523368	-	142	grey	0.039081673	FALSE	PARP1
Region_1701	chr1	226548764	226609230	60466	23	148	176.0757576	14.07377332	0.773352475	0.029229535	+	375057	grey	0.075972503	FALSE	STUM
Region_1702	chr1	226631690	226739323	107633	63	416	491.6212121	25.18654412	0.629488351	0.030502705	-	3707	grey	-0.078944251	FALSE	ITPKB
Region_1703	chr1	226656080	226675092	19012	4	20	31.98484848	5.413636413	0.830680919	0.0630877	-	100506443	grey	-0.111867878	FALSE	ITPKB-IT1
Region_1704	chr1	226870184	226987545	117361	49	338	387.8939394	25.18982016	0.782766582	0.018540149	+	5664	grey	0.080034002	FALSE	PSEN2
Region_1705	chr1	226940286	226987544	47258	20	134	153.3333333	12.2616768	0.686025071	0.029228332	+	56997	grey	0.202103522	FALSE	COQ8A
Region_1706	chr1	226989865	227318492	328627	56	372	414.2575758	21.55587416	0.916758976	0.014156966	-	8476	grey	0.014433982	FALSE	CDC42BPA
Region_1707	chr1	227393554	227431035	37481	7	41	55.72727273	7.804427648	0.776013261	0.041151706	+	107985463	grey	0.060317545	FALSE	LINC01641
Region_1708	chr1	227563543	227677443	113900	10	75	101.6515152	10.41669138	0.926184393	0.032332719	+	339500	grey	0.313228649	FALSE	ZNF678
Region_1709	chr1	227728200	227781826	53626	27	194	229.1363636	16.71653844	0.679092937	0.022704581	+	116841	grey	0.131306143	FALSE	SNAP47
Region_1710	chr1	227730425	227735411	4986	3	15	31.15151515	5.569815421	0.32751082	0.015698213	-	65094	grey	0.08485261	FALSE	JMJD4
Region_1711	chr1	227786753	227792179	5426	4	24	36.53030303	5.671032187	0.178330511	0.079328925	+	105373289	grey	-0.192298791	FALSE	LOC105373289
Region_1712	chr1	22781567	22784647	30795	5	35	48.1818181	5.87539613	0.76801068	0.05096422	+	339501	grey	-0.14611736	FALSE	PRSS38

		5	0				8	3		2				5		
Region_1713	chr1	227918656	227947932	29276	33	203	246.6969697	17.8282142	0.737035276	0.024713781	-	7483	grey	0.200427314	FALSE	WNT9A
Region_1714	chr1	228006998	228061271	54273	22	138	176.6363636	14.68922825	0.741149176	0.031747369	+	89780	grey	-0.275330525	FALSE	WNT3A
Region_1715	chr1	228073909	228076550	2641	4	19	34.01515152	6.429481195	0.547923709	0.095064884	-	100506571	grey	-0.110553277	FALSE	LINC02809
Region_1716	chr1	228082708	228099212	16504	18	134	164.1515152	15.67481314	0.48025653	0.022350686	+	375	grey	0.030948426	FALSE	ARF1
Region_1717	chr1	228106679	228109312	2633	3	18	28.22727273	5.052936555	0.914636919	0.05429538	-	128308	grey	0.306621509	FALSE	MRPL55
Region_1718	chr1	228139962	228148984	9022	9	43	60.62121212	7.663630695	0.356752096	0.036848904	+	2987	grey	0.193210765	FALSE	GUK1
Region_1719	chr1	228149930	228159826	9896	20	103	136.3787879	16.26394327	0.89622692	0.027293552	+	57165	grey	-0.083113368	FALSE	GJC2
Region_1720	chr1	228165804	228182257	16453	6	26	45.48484848	7.862750398	0.780577566	0.042324817	+	200205	grey	-0.128805172	FALSE	IBA57
Region_1721	chr1	228203503	228213664	10161	7	31	50.48484848	8.080827117	0.896796028	0.047542975	-	574407	grey	-0.13628452	FALSE	OBSCN-AS1
Region_1722	chr1	228208044	228378876	170832	116	771	875.969697	46.28505979	0.885778077	0.011792906	+	84033	grey	-0.188379652	FALSE	OBSCN
Region_1723	chr1	228357012	228368554	11542	12	41	61.15151515	9.340140708	0.809514728	0.054640353	-	101927401	grey	-0.191200021	FALSE	LOC101927401
Region_1724	chr1	228393673	228406835	13162	16	98	127.5909091	12.38234136	0.784814756	0.020639342	-	81559	grey	0.022035532	FALSE	TRIM11

Region_1725	chr1	228407935	228416861	8926	7	41	60.93939394	7.075154335	0.681602199	0.032305883	-	51127	grey	0.062020205	FALSE	TRIM17
Region_1726	chr1	229022773	229038274	15501	5	27	41.66666667	6.6963442	0.888377414	0.06141508	-	105373158	grey	-0.149091376	FALSE	LINC02815
Region_1727	chr1	229087114	229205428	118314	28	184	212.4545455	12.83766312	0.82989553	0.02925833	-	112267873	grey	-0.002442821	FALSE	LINC02814
Region_1728	chr1	229223457	229242902	19445	4	15	26.62121212	5.134394284	0.943423123	0.0472662	-	102723935	grey	0.194971712	FALSE	LOC102723935
Region_1729	chr1	229256892	229271242	14350	4	22	34.03030303	6.334204332	0.631987812	0.108969253	-	105373159	grey	0.092350411	FALSE	RAB4A-AS1
Region_1730	chr1	229271062	229305894	34832	4	22	31.89393939	5.66535704	0.552427849	0.087120046	+	5867	grey	0.200372069	FALSE	RAB4A
Region_1731	chr1	229321011	229343294	22283	8	52	67.04545455	7.082876031	0.935404488	0.037791386	-	126731	grey	-0.049444652	FALSE	CCSAP
Region_1732	chr1	229430365	229434104	3739	4	19	33.59090909	6.775469947	0.114972258	0.064104256	-	58	grey	0.20076508	FALSE	ACTA1
Region_1733	chr1	229440259	229508341	68082	25	197	225.8333333	13.93013704	0.873986647	0.019467606	-	55746	grey	-0.177732549	FALSE	NUP133
Region_1734	chr1	229516582	229558707	42125	18	120	145.0606061	12.91258037	0.878982471	0.033440241	-	23456	grey	-0.08386109	FALSE	ABCB10
Region_1735	chr1	229593134	229626122	32988	8	49	70.87878788	8.258357355	0.778153759	0.05485364	-	27097	grey	-0.282602906	FALSE	TAF5L
Region_1736	chr1	229626247	229660200	33953	10	51	73.71212121	9.557542574	0.761242326	0.036243018	+	9816	grey	-0.134551721	FALSE	URB2
Region_1737	chr1	22981291	22989200	79091	15	83	114.681818	11.4194962	0.91504397	0.03257835	-	10537316	grey	0.056497869	FALSE	LOC105373162

		7	8				2	7	2	4		2				
Region_1738	chr1	229869435	229892046	22611	7	39	58.3030303	6.437796319	0.915433171	0.039993823	-	101927504	grey	-0.098331856	FALSE	LINC01682
Region_1739	chr1	230057990	230282122	224132	87	645	702.3787879	29.03725202	0.805766696	0.014820697	+	2590	grey	0.143632879	FALSE	GALNT2
Region_1740	chr1	230314490	230426332	111842	45	315	354.2575758	19.90539981	0.83637887	0.021970955	-	79605	grey	0.060371617	FALSE	PGBD5
Region_1741	chr1	230426498	230641806	215308	46	338	382.4545455	16.89258537	0.814181937	0.025230204	+	105373164	grey	-0.139027875	FALSE	LOC105373164
Region_1742	chr1	230642481	230693982	51501	3	12	24.68181818	5.280323682	0.963011997	0.049678076	+	22796	grey	-0.157513816	FALSE	COG2
Region_1743	chr1	230690776	230795492	104716	26	178	213.8484848	14.88028373	0.929776708	0.020128424	-	183	grey	0.004669375	FALSE	AGT
Region_1744	chr1	230747384	230802003	54619	13	77	102.1969697	10.56877796	0.909865416	0.030838004	+	10753	grey	0.092915215	FALSE	CAPN9
Region_1745	chr1	230776081	230795153	19072	7	44	57.04545455	6.839774666	0.938545783	0.038515664	-	107985359	grey	0.039379977	FALSE	LOC107985359
Region_1746	chr1	230837119	230869589	32470	13	76	99.63636364	10.31598664	0.731929583	0.026741496	-	84886	grey	0.049567347	FALSE	C1orf198
Region_1747	chr1	230868259	230879141	10882	6	39	50.89393939	6.753070613	0.614308367	0.034554081	+	101927604	grey	-0.063862998	FALSE	LOC101927604
Region_1748	chr1	230906243	230978875	72632	14	87	111.6818182	10.0202243	0.750061418	0.022676593	-	79573	grey	0.099955049	FALSE	TTC13
Region_1749	chr1	230978981	231000733	21752	11	63	97.36363636	11.44436222	0.448960841	0.019471407	+	64801	grey	-0.093717213	FALSE	ARV1

Region_1750	chr1	231018958	231040254	21296	7	34	50.93939394	7.144398102	0.512624988	0.06189041	-	375061	grey	-0.241060247	FALSE	FAM89A
Region_1751	chr1	231162058	231221565	59507	29	221	260.7121212	14.22323684	0.540937947	0.021913398	+	440730	grey	-0.134205504	FALSE	TRIM67
Region_1752	chr1	231223763	231241187	17424	6	40	56.1969697	7.16663143	0.650923362	0.027454605	-	128061	grey	0.269971456	FALSE	C1orf131
Region_1753	chr1	231241207	231277973	36766	8	49	63.45454545	8.458371944	0.735545813	0.021573637	+	8443	grey	-0.043863948	FALSE	GNPAT
Region_1754	chr1	231332753	231337852	5099	6	42	58.77272727	8.174623877	0.083542062	0.026188847	-	149371	grey	-0.034711553	FALSE	EXOC8
Region_1755	chr1	231337104	231355023	17919	17	110	136.8333333	11.51799039	0.673024058	0.01997407	+	83932	grey	-0.117586825	FALSE	SPRTN
Region_1756	chr1	231363751	231528603	164852	47	346	391.4848485	18.86979061	0.638988926	0.02423157	-	54583	grey	0.131545676	FALSE	EGLN1
Region_1757	chr1	231520729	231528618	7889	3	12	23.42424242	4.053718544	0.423382594	0.091189379	-	122526782	grey	-0.066023483	FALSE	LOC122526782
Region_1758	chr1	231528541	231819244	290703	39	314	343.2424242	16.71761909	0.741406498	0.025637128	+	100303453	grey	0.226732877	FALSE	TSNAX-DISC1
Region_1759	chr1	231528669	231566524	37855	4	22	33.24242424	5.449235955	0.875137466	0.08067525	+	7257	grey	-0.001844946	FALSE	TSNAX
Region_1760	chr1	231591292	231612090	20798	5	26	37.18181818	5.670189499	0.944337811	0.046002712	-	100287814	grey	-0.011320544	FALSE	LINC00582
Region_1761	chr1	231626790	232041272	414482	56	458	506.5757576	23.3416502	0.822448429	0.018602165	+	27185	grey	0.067919852	FALSE	DISC1
Region_1762	chr1	23176746	23184780	80339	9	51	74.3181818	9.46598621	0.95683336	0.02537055	-	10537317	grey	-0.04637625	FALSE	LOC105373170

		4	3				2	9	2	2		0		7		
Region_1763	chr1	231925834	231945233	19399	3	23	34.81818182	5.901522857	0.948788855	0.035655191	+	104472714	grey	-0.024966305	FALSE	DISC1-IT1
Region_1764	chr1	232397965	232630571	232606	49	362	410.0757576	21.12913454	0.874906234	0.017150958	-	57568	grey	0.156907879	FALSE	SIPA1L2
Region_1765	chr1	232727251	232751299	24048	8	41	59.43939394	8.095827582	0.829527513	0.055304193	-	101927683	grey	-0.135980828	FALSE	LINC01744
Region_1766	chr1	232843386	232897660	54274	20	138	165.9545455	13.29248697	0.82427419	0.025017895	+	101927711	grey	0.029733935	FALSE	LOC101927711
Region_1767	chr1	232950605	232983882	33277	3	15	27.60606061	5.232674821	0.874388305	0.087461117	+	84284	grey	0.220749445	FALSE	NTPCR
Region_1768	chr1	232983435	233295725	312290	47	351	403.4545455	18.50087883	0.874623849	0.018062042	-	80003	grey	0.120705398	FALSE	PCNX2
Region_1769	chr1	233327724	233385148	57424	5	29	47.62121212	6.398954606	0.643388373	0.040417889	+	84451	grey	0.082704507	FALSE	MAP3K21
Region_1770	chr1	233614106	233672514	58408	8	39	54.74242424	6.913332951	0.654750647	0.054853907	+	3775	grey	-0.198930903	FALSE	KCNK1
Region_1771	chr1	233904676	234324511	419835	51	365	411.7121212	20.84430574	0.817082974	0.019367591	+	148641	grey	0.225605433	FALSE	SLC35F3
Region_1772	chr1	234391313	234479179	87866	20	131	167.9545455	12.50652976	0.897592006	0.025853853	-	6894	grey	-0.175190847	FALSE	TARBP1
Region_1773	chr1	234527887	234531803	3916	3	14	22.07575758	4.008877096	0.903827857	0.064458039	-	100506795	grey	-0.110365109	FALSE	LINC01354
Region_1774	chr1	234604269	234610178	5909	4	24	36.18181818	5.76170138	0.006743383	0.016064556	-	359948	grey	0.082289775	FALSE	IRF2BP2

Region_1775	chr1	234646289	234660924	14635	4	21	29.78787879	4.962986544	0.940434975	0.049498412	+	101927787	grey	0.311065236	FALSE	LOC101927787
Region_1776	chr1	234656052	234683176	27124	6	24	40.15151515	6.843846027	0.663316288	0.051254586	+	105373209	grey	-0.243849336	FALSE	LOC105373209
Region_1777	chr1	234724042	234736720	12678	14	70	97.51515152	10.52066454	0.562235362	0.032604377	+	100506810	grey	-0.211625642	FALSE	LINC01132
Region_1778	chr1	234811052	234932653	121601	46	313	377.1515152	22.96434857	0.877182183	0.020206534	-	107985365	grey	0.244597614	FALSE	LOC107985365
Region_1779	chr1	234957342	234970062	12720	4	20	28.66666667	4.996408967	0.618688298	0.099002296	-	101927851	grey	0.110826089	FALSE	LNCATV
Region_1780	chr1	235064927	235069814	4887	5	26	38.46969697	6.154972216	0.84838946	0.048409695	-	731656	grey	0.133550793	FALSE	LINC01348
Region_1781	chr1	235065479	235074220	8741	6	35	52.60606061	7.242776323	0.837206518	0.043478922	-	107985746	grey	0.13040193	FALSE	
Region_1782	chr1	235109341	235128837	19496	9	54	76.56060606	9.535567085	0.812895025	0.043936032	-	9804	grey	0.142122473	FALSE	TOMM20
Region_1783	chr1	235131183	235161283	30100	10	57	80.83333333	10.51823813	0.848052792	0.028695014	-	23029	grey	0.12454781	FALSE	RBM34
Region_1784	chr1	235131634	235328219	196585	54	370	422.5454545	23.6735372	0.806320855	0.011829688	-	51742	grey	0.198842369	FALSE	ARID4B
Region_1785	chr1	235327350	235401587	74237	29	188	224.4393939	18.21168584	0.67809499	0.01332073	+	9453	grey	-0.054281763	FALSE	GGPS1
Region_1786	chr1	235328570	235452443	123873	43	321	364.1666667	20.93990179	0.818989368	0.01083572	+	6905	grey	0.050762242	FALSE	TBCE
Region_1787	chr1	23554768	23565075	103069	42	301	342.515151	19.8418690	0.80563579	0.02303532	-	2786	grey	0.149056301	FALSE	GNG4

		5	4				5	4	1	5						
Region_1788	chr1	235661041	235883724	222683	45	337	371.9242424	17.22548256	0.789835982	0.020841288	-	1130	grey	-0.007718133	FALSE	LYST
Region_1789	chr1	235975830	236065109	89279	27	195	235.1060606	16.80487913	0.899825609	0.015514645	-	4811	grey	0.095484349	FALSE	NID1
Region_1790	chr1	236142505	236221865	79360	23	146	172.9848485	16.05231105	0.74333426	0.027904213	+	7107	grey	0.129490202	FALSE	GPR137B
Region_1791	chr1	236214681	236282019	67338	18	100	121.7575758	10.75612256	0.747245224	0.035516824	-	56605	grey	0.041442018	FALSE	ERO1B
Region_1792	chr1	236348257	236502915	154658	50	348	391.5757576	21.10491055	0.898309931	0.014145972	+	128178	grey	0.090655784	FALSE	EDARADD
Region_1793	chr1	236518000	236552981	34981	12	73	94.54545455	9.882668307	0.607177415	0.045654102	+	3964	grey	-0.344801628	FALSE	LGALS8
Region_1794	chr1	236549005	236604516	55511	21	148	173.3939394	11.5783336	0.936301243	0.020254051	-	55127	grey	-0.302171804	FALSE	HEATR1
Region_1795	chr1	236664141	236764631	100490	41	292	325.7272727	19.95267127	0.747770725	0.018080143	+	88	grey	0.022463392	FALSE	ACTN2
Region_1796	chr1	236795260	236913424	118164	27	211	244.0454545	17.43332954	0.79034413	0.017209779	+	4548	grey	0.14330549	FALSE	MTR
Region_1797	chr1	236898523	236921278	22755	8	37	58.09090909	8.833027659	0.966542573	0.030042135	+	105373218	grey	0.288468003	FALSE	LOC105373218
Region_1798	chr1	237042184	237833988	791804	134	1010	1093.257576	32.62434702	0.851212146	0.016421536	+	6262	grey	0.016125948	FALSE	RYR2
Region_1799	chr1	237862175	237928321	66146	5	31	47.63636364	6.840234732	0.741487952	0.036779717	+	100130331	grey	0.24476155	FALSE	LOC100130331

Region_1800	chr1	238238943	238404906	165963	21	132	165.1060606	12.99364198	0.890414321	0.031785967	+	105373220	grey	0.02393322	FALSE	LOC105373220
Region_1801	chr1	239386565	239915452	528887	75	584	642.4090909	29.18059275	0.856519889	0.021635336	+	1131	grey	0.256552629	FALSE	CHRM3
Region_1802	chr1	239703892	239770130	66238	6	45	56.33333333	6.83280173	0.945393296	0.044258662	-	100506915	grey	-0.112663159	FALSE	CHRM3-AS2
Region_1803	chr1	240014348	240475187	460839	98	687	765.530303	32.56388736	0.84581269	0.016524664	+	56776	grey	0.521287226	FALSE	FMN2
Region_1804	chr1	240489573	240612155	122582	17	110	134.3939394	11.23987242	0.764486591	0.036052221	-	64388	grey	0.1149237	FALSE	GREM2
Region_1805	chr1	240767636	241357374	589738	116	896	972.8484848	30.90792401	0.856537522	0.013628174	-	6000	grey	0.046901718	FALSE	RGS7
Region_1806	chr1	241424628	241450593	25965	4	24	33.48484848	5.915409926	0.812408184	0.077335963	+	105373230	grey	-0.013299656	FALSE	
Region_1807	chr1	241497511	241519799	22288	7	40	65	12.60280799	0.144449824	0.013858308	-	2271	grey	0.118795845	FALSE	FH
Region_1808	chr1	241532134	241595642	63508	7	33	51	6.387487769	0.95231468	0.056083769	+	8564	grey	-0.187220443	FALSE	KMO
Region_1809	chr1	241590102	241677376	87274	13	83	106.8333333	11.8705626	0.899446251	0.030156082	-	23596	grey	-0.219988261	FALSE	OPN3
Region_1810	chr1	241652278	241802777	150499	15	102	128.5757576	12.13014123	0.832309398	0.033620304	+	128025	grey	-0.061093797	FALSE	WDR64
Region_1811	chr1	241847967	241895148	47181	8	45	63.77272727	7.356411028	0.749572231	0.027980199	+	9156	grey	0.186360548	FALSE	EXO1
Region_1812	chr1	24199549	24199909	3608	3	15	25.9090909	6.07321299	0.94841519	0.0420858	-	440738	grey	-0.05426382	FALSE	MAP1LC3C

		0	8				1	5	6					1		
Region_1813	chr1	242082986	242524697	441711	84	588	669.3333333	32.05780036	0.819906014	0.016620307	-	200150	grey	0.284945506	FALSE	PLD5
Region_1814	chr1	243917402	244047317	129915	26	205	237.030303	14.35909757	0.85889756	0.025975026	+	339529	grey	0.535054916	FALSE	LINC02774
Region_1815	chr1	244048547	244057476	8929	5	38	50.13636364	6.602764837	0.380906809	0.069713226	+	10472	grey	0.189460557	FALSE	ZBTB18
Region_1816	chr1	244068820	244093026	24206	10	63	90.33333333	9.87667545	0.902350458	0.050490663	-	112268257	grey	0.245967821	FALSE	
Region_1817	chr1	244308554	244325179	16625	4	23	31.37878788	4.806294435	0.812750625	0.064263686	-	105373262	grey	-0.012807857	FALSE	LOC105373262
Region_1818	chr1	244352635	244389663	37028	11	83	104.3333333	8.944845242	0.838267751	0.038990792	+	200159	grey	0.325128358	FALSE	SPMIP3
Region_1819	chr1	244408494	244451909	43415	9	66	84	8.816069244	0.872915024	0.02279126	-	159	grey	0.162596679	FALSE	ADSS2
Region_1820	chr1	244454377	244640504	186127	63	452	509.4848485	23.87096236	0.88518682	0.015096329	+	257044	grey	-0.076659736	FALSE	CATSPERE
Region_1821	chr1	244653103	244709033	55930	7	34	48.63636364	6.653249903	0.690348773	0.030638691	+	51029	grey	0.065905196	FALSE	DESI2
Region_1822	chr1	244840638	244864560	23922	7	39	56.40909091	7.702102156	0.750406297	0.054117336	-	3192	grey	0.061009022	FALSE	HNRNPU
Region_1823	chr1	244969682	245127164	157482	60	436	477.2272727	19.96596755	0.848766608	0.013481232	+	84288	grey	-0.300862114	FALSE	EFCAB2
Region_1824	chr1	245002153	245030831	28678	9	51	67.12121212	8.117436411	0.966103274	0.026111848	+	107985725	grey	0.178309216	FALSE	LOC107985725

Region_1825	chr1	245206444	245234501	28057	17	113	134.8787879	10.08199484	0.929563338	0.029117336	-	111216275	grey	0.195919736	FALSE	KIF26B-AS1
Region_1826	chr1	245749342	246507312	757970	170	1284	1393.151515	51.59166373	0.919468723	0.008860267	-	64754	grey	-0.116373909	FALSE	SMYD3
Region_1827	chr1	246540561	246566261	25700	11	78	95.10606061	9.609496566	0.868339351	0.02473208	-	64216	grey	-0.151051099	FALSE	TFB2M
Region_1828	chr1	246566444	246668595	102151	36	241	278.0151515	17.799302	0.859572231	0.02988899	+	163882	grey	-0.121309957	FALSE	CNST
Region_1829	chr1	246724409	246768137	43728	16	101	135.0606061	11.7628866	0.892089087	0.027130179	+	51097	grey	-0.226888273	FALSE	SCCPDH
Region_1830	chr1	246775880	246792385	16505	10	55	73.48484848	8.414716823	0.815534356	0.033233385	+	149134	grey	-0.000630662	FALSE	LINC01341
Region_1831	chr1	246839098	246931948	92850	29	209	241.1666667	13.8947509	0.833361694	0.01652805	-	25909	grey	0.246313624	FALSE	AHCTF1
Region_1832	chr1	246945547	247008093	62546	32	232	274.0151515	18.74976301	0.940379138	0.012996973	-	57116	grey	-0.087992926	FALSE	ZNF695
Region_1833	chr1	246945547	247078811	133264	57	395	447.1363636	24.95897333	0.900289931	0.016483792	-	100533111	grey	-0.479465233	FALSE	ZNF670-ZNF695
Region_1834	chr1	247034637	247078811	44174	15	71	101.4545455	11.19228006	0.805514979	0.040808426	-	93474	grey	-0.388686668	FALSE	ZNF670
Region_1835	chr1	247099962	247104372	4410	6	36	50.60606061	7.671884309	0.777439816	0.040520133	-	79862	grey	-0.101369141	FALSE	ZNF669
Region_1836	chr1	247110160	247112417	2257	6	26	37.63636364	5.267129896	0.462167799	0.052712984	-	388759	grey	-0.15727665	FALSE	LINC02897
Region_1837	chr1	24712197	24717202	50045	14	65	106.121212	12.5127813	0.85989595	0.04829728	-	7678	grey	0.114097514	FALSE	ZNF124

		5	0				1	4	4	9						
Region_1838	chr1	24718985<br>1	24721085<br>6	21005	7	39	51.4696969<br>7	6.75783237<br>2	0.98227315<br>6	0.02134014<br>6	-	11226825<br>9	grey	-0.01288653<br>7	FALSE	
Region_1839	chr1	24727338<br>5	24728648<br>9	13104	9	49	68.8939393<br>9	7.89767012<br>9	0.85157214<br>5	0.0487731	+	10537327<br>3	grey	-0.04176504<br>1	FALSE	LOC105373273
Region_1840	chr1	24729741<br>2	24733186<br>7	34455	6	30	49.6818181<br>8	8.15646811	0.72540391<br>9	0.05003160<br>6	-	84838	grey	0.119647277	FALSE	ZNF496
Region_1841	chr1	24733233<br>1	24744910<br>8	116777	37	225	286.772727<br>3	18.7368788<br>6	0.68493371<br>6	0.02794787<br>8	+	114548	grey	-0.14958711<br>2	FALSE	NLRP3
Region_1842	chr1	24733235<br>1	24739378<br>9	61438	21	133	165.757575<br>8	14.3752571<br>7	0.65522072<br>6	0.04269115	+	10798511<br>5	grey	-0.27605140<br>7	FALSE	ZNF496-DT
Region_1843	chr1	24733276<br>5	24739383<br>9	61074	21	133	165.757575<br>8	14.3752571<br>7	0.65522072<br>6	0.04269115	+	10798537<br>5	grey	-0.27605140<br>7	FALSE	LOC107985375
Region_1844	chr1	24744911<br>8	24745810<br>5	8987	6	34	46.6060606<br>1	6.06091840<br>7	0.65735871<br>3	0.06027937<br>4	-	127623	grey	0.084328616	FALSE	OR2B11
Region_1845	chr1	24750705<br>8	24757769<br>0	70632	12	69	89.5151515<br>2	9.94328907<br>7	0.86329027	0.04109322<br>9	+	148823	grey	-0.15663304<br>2	FALSE	GCSAML
Region_1846	chr1	24752467<br>7	24753644<br>0	11763	3	15	25.4848484<br>8	5.15675039<br>1	0.90237234<br>8	0.06226800<br>4	-	81472	grey	-0.04090935<br>6	FALSE	OR2C3
Region_1847	chr1	24756563<br>9	24764085<br>4	75215	13	92	113.787878<br>8	10.9635973<br>2	0.89528006	0.03733639<br>8	-	10272444<br>6	grey	-0.10821933	FALSE	LOC102724446
Region_1848	chr1	24771143<br>6	24772376<br>4	12328	3	13	30.8181818<br>2	6.44958935<br>1	0.93415498<br>8	0.06606039<br>9	-	343169	grey	-0.27853357	FALSE	OR6F1
Region_1849	chr1	24785718<br>7	24788013<br>8	22951	4	18	28.3939393<br>9	4.97958536<br>4	0.80300324<br>5	0.07295823<br>2	+	25893	grey	-0.31058097<br>5	FALSE	TRIM58

Region_1850	chr1	248046836	248063407	16571	4	21	35.28787879	6.055550922	0.912698305	0.057075332	+	391192	grey	0.002680166	FALSE	OR2L3
Region_1851	chr1	248095184	248101163	5979	3	12	23.27272727	4.913233166	0.673681811	0.098320664	+	284521	grey	-0.327227608	FALSE	OR2L13
Region_1852	chr1	248681249	248748349	67100	9	45	70.66666667	9.815738278	0.840144913	0.043158462	-	401994	grey	-0.164861575	FALSE	OR14I1
Region_1853	chr1	248691760	248716755	24995	5	21	38.8030303	7.890051568	0.750708806	0.071477221	+	101928226	grey	-0.148605638	FALSE	LYPD9P
Region_1854	chr1	248718649	248755759	37110	4	19	31.86363636	5.414755804	0.951940047	0.041712924	-	646627	grey	-0.108873346	FALSE	LYPD8
Region_1855	chr1	248810446	248825915	15469	7	39	53.3030303	6.950306461	0.846567799	0.026668558	-	80851	grey	-0.148155648	FALSE	SH3BP5L
Region_1856	chr1	248838210	248849517	11307	9	52	73.40909091	10.2326435	0.609082774	0.031185699	+	79894	grey	0.079333101	FALSE	ZNF672
Region_1857	chr1	248850006	248859144	9138	6	33	47.77272727	6.985249193	0.392120243	0.033875997	-	55657	grey	0.030171328	FALSE	ZNF692
Region_1858	chr10	46888	74163	27275	8	48	81.63636364	15.09522223	0.388987032	0.04676392	-	347688	grey	0.034291973	FALSE	TUBB8
Region_1859	chr10	134465	254637	120172	20	143	177.1818182	12.9399906	0.824021783	0.018440237	+	10771	grey	0.400185171	FALSE	ZMYND11
Region_1860	chr10	274190	689668	415478	393	2939	3212.757576	110.0288159	0.853350523	0.007370505	-	22982	grey	-0.144788633	FALSE	DIP2C
Region_1861	chr10	628633	631255	2622	5	23	35.84848485	6.170007915	0.746630886	0.044178455	+	101930421	grey	0.024199488	FALSE	LOC101930421
Region_1862	chr10	649948	669581	19633	22	161	188.818181	13.9646907	0.88617634	0.02390659	+	414235	grey	0.040558161	FALSE	DIP2C-AS1

	0						8	4	3	8						
Region_1863	chr10	806933	931821	124888	65	521	591.1060606	32.1061359	0.878687282	0.013718232	-	23185	grey	0.005722873	FALSE	LARP4B
Region_1864	chr10	933026	942743	9717	4	27	40.04545455	6.705753714	0.8260469	0.083257416	+	101927762	grey	-0.290121284	FALSE	LARP4B-DT
Region_1865	chr10	988434	1019932	31498	23	154	175.7272727	12.80690373	0.922147256	0.014054599	+	23560	grey	-0.125648793	FALSE	GTPBP4
Region_1866	chr10	1018910	1025859	6949	7	38	56.33333333	8.721914633	0.968322453	0.02544769	-	91734	grey	-0.089360296	FALSE	IDI2
Region_1867	chr10	1022618	1045425	22807	15	79	118.2121212	13.55162795	0.81358613	0.031879473	+	55853	grey	0.061472892	FALSE	IDI2-AS1
Region_1868	chr10	1039152	1049119	9967	7	53	67.36363636	6.774695823	0.462342343	0.024171954	-	3422	grey	-0.25144648	FALSE	IDI1
Region_1869	chr10	1049538	1132384	82846	67	495	560.5151515	30.57914947	0.840873042	0.015256615	+	22884	grey	0.161516828	FALSE	WDR37
Region_1870	chr10	1159768	1289426	129658	84	596	663.1363636	32.3913029	0.878326641	0.018475777	+	399706	grey	-0.006929678	FALSE	LINC00200
Region_1871	chr10	1177313	1737525	560212	330	2517	2756.151515	90.80397186	0.838619456	0.01355232	-	105	grey	-0.033182077	FALSE	ADARB2
Region_1872	chr10	1526637	1556988	30351	15	88	119.6212121	12.67676876	0.878882265	0.048235585	+	642394	grey	0.057321628	FALSE	ADARB2-AS1
Region_1873	chr10	1989076	2015005	25929	8	72	88.37878788	8.39004016	0.884646201	0.037412661	-	282980	grey	-0.03469163	FALSE	LINC00700
Region_1874	chr10	2446244	2502200	55956	9	62	76.62121212	8.242040732	0.898657053	0.040800967	-	105376351	grey	-0.071370663	FALSE	LINC02645

Region_1875	chr10	2501600	2618739	117139	33	228	264.1363636	16.0248714	0.889141827	0.024219113	+	105376350	grey	0.244231912	FALSE	LOC105376350
Region_1876	chr10	3066333	3137718	71385	86	529	604.3939394	39.88285878	0.810608727	0.012166812	+	5214	grey	-0.095727424	FALSE	PFKP
Region_1877	chr10	3134566	3167972	33406	42	300	348.1060606	18.60200881	0.947151766	0.016504402	+	100507034	grey	0.159396659	FALSE	PITRM1-AS1
Region_1878	chr10	3137728	3172841	35113	51	378	422.7878788	22.09804388	0.876612744	0.013806537	-	10531	grey	0.177439769	FALSE	PITRM1
Region_1879	chr10	3232264	3258897	26633	20	135	166.3636364	12.13340762	0.849445461	0.036811288	-	105376354	grey	0.376610127	FALSE	LINC02668
Region_1880	chr10	3432519	3502915	70396	29	219	250.8030303	16.44872658	0.935422676	0.018433492	-	101927880	grey	0.051245817	FALSE	LINC02669
Region_1881	chr10	3748966	3768751	19785	7	32	49.83333333	7.764977257	0.947385182	0.047880287	+	105376363	grey	-0.081848919	FALSE	LOC105376363
Region_1882	chr10	3775996	3785281	9285	5	22	36.90909091	5.901048858	0.410878843	0.056348461	-	1316	grey	0.237995122	FALSE	KLF6
Region_1883	chr10	3911889	3941506	29617	9	38	63.56060606	9.452925942	0.951608021	0.042002363	+	101927904	grey	0.180439808	FALSE	LINC02660
Region_1884	chr10	4024903	4053018	28115	6	44	59.56060606	6.407036487	0.713246398	0.044661001	+	107984195	grey	0.220313897	FALSE	LOC107984195
Region_1885	chr10	4051726	4089013	37287	9	45	68.83333333	8.831035018	0.767379101	0.041745758	+	101927964	grey	0.286049862	FALSE	
Region_1886	chr10	4201141	4243912	42771	7	46	65.09090909	9.373821604	0.958376233	0.025016495	-	100652988	grey	0.129276738	FALSE	LINC00702
Region_1887	chr1	4581295	4678216	96921	6	40	60.5909090	7.53040457	0.90333125	0.03856109	-	10021600	grey	0.210173805	FALSE	MANCR

	0						9	1	5			1				
Region_1888	chr10	4655427	4676981	21554	3	16	26.86363636	5.485326899	0.901827208	0.065403444	+	338588	grey	0.101628555	FALSE	LINC00705
Region_1889	chr10	4655463	4682722	27259	4	24	35.57575758	6.707196149	0.913653682	0.055612059	+	105376373	grey	-0.055012837	FALSE	LOC105376373
Region_1890	chr10	4786629	4848062	61433	13	81	98.89393939	10.28236547	0.795207373	0.034400074	+	83592	grey	-0.106376974	FALSE	AKR1E2
Region_1891	chr10	4963253	4983283	20030	5	28	48.06060606	7.542451729	0.810284735	0.074584776	+	1645	grey	0.09567989	FALSE	AKR1C1
Region_1892	chr10	5035354	5107686	72332	5	33	46.25757576	6.388235837	0.896855999	0.078833412	+	8644	grey	0.107212924	FALSE	AKR1C3
Region_1893	chr10	5035357	5097537	62180	3	18	30.01515152	5.196129993	0.940108688	0.047376018	+	107984198	grey	0.252709595	FALSE	LOC107984198
Region_1894	chr10	5153920	5185187	31267	5	27	41.45454545	6.246677439	0.899620931	0.066205629	-	340811	grey	-0.133975384	FALSE	AKR1C8
Region_1895	chr10	5412557	5459056	46499	8	52	72.07575758	9.20252575	0.953857699	0.020453005	+	10276	grey	0.047760868	FALSE	NET1
Region_1896	chr10	5510036	5526246	16210	12	62	87.75757576	10.73607951	0.934226815	0.037706144	-	100132159	grey	0.416016189	FALSE	CALML3-AS1
Region_1897	chr10	5549637	5554329	4692	6	33	49.27272727	7.252682166	0.78070691	0.055901171	-	105376380	grey	-0.240452342	FALSE	LOC105376380
Region_1898	chr10	5594984	5632459	37475	33	209	255.1666667	19.88086311	0.880886616	0.029646005	-	105376382	grey	0.425470149	FALSE	LASTR
Region_1899	chr10	5603308	5610878	7570	6	27	44.51515152	8.259712091	0.858574363	0.063175678	-	105376383	grey	0.186914114	FALSE	LINC02678

Region_1900	chr10	5616618	5632566	15948	17	111	145.030303	13.3646718	0.876797733	0.035152694	-	102723629	grey	0.341797722	FALSE	LINC02677
Region_1901	chr10	5638867	5666595	27728	21	148	177.8939394	16.21976836	0.758529019	0.028857262	-	79754	grey	-0.311825844	FALSE	ASB13
Region_1902	chr10	5684731	5763779	79048	23	142	187.3939394	14.29887439	0.793464902	0.021777394	+	54906	grey	0.060208291	FALSE	TASOR2
Region_1903	chr10	5765223	5842132	76909	25	159	188.5454545	14.08237504	0.860382497	0.018144388	-	2665	grey	-0.199812722	FALSE	GDI2
Region_1904	chr10	5861616	5889906	28290	13	83	105.1212121	9.563576981	0.753176801	0.025415504	-	54522	grey	-0.047033077	FALSE	ANKRD16
Region_1905	chr10	5890203	5937594	47391	35	246	294.2121212	20.82492231	0.812646735	0.017809411	+	84893	grey	-0.05256454	FALSE	FBH1
Region_1906	chr10	5934270	5945900	11630	11	71	84.62121212	7.449863502	0.924546843	0.02849295	-	105376384	grey	0.094304322	FALSE	LOC105376384
Region_1907	chr10	5943639	5978187	34548	22	116	150.1969697	13.51149903	0.678438683	0.020030539	-	3601	grey	0.31191958	FALSE	IL15RA
Region_1908	chr10	6010689	6062367	51678	15	73	99.46969697	9.076122006	0.801964661	0.041607333	-	3559	grey	0.37341264	FALSE	IL2RA
Region_1909	chr10	6089034	6117457	28423	17	120	141.7424242	11.55629779	0.683090163	0.018524489	+	84991	grey	0.01402681	FALSE	RBM17
Region_1910	chr10	6144934	6254644	109710	107	699	766.8333333	39.08711506	0.801708718	0.011590809	+	5209	grey	0.176875332	FALSE	PFKFB3
Region_1911	chr10	6271169	6353827	82658	27	193	218.7272727	13.98850677	0.868876376	0.023327355	+	399715	grey	-0.024843609	FALSE	LINC02649
Region_1912	chr1	6427143	6580301	153158	39	286	334.409090	19.5518854	0.84310407	0.02040894	-	5588	grey	0.23566152	FALSE	PRKCQ

	0						9	3	8	2						
Region_1913	chr10	6580419	6615954	35535	5	26	37.06060606	5.953354033	0.871522359	0.074733078	+	439949	grey	0.143297742	FALSE	PRKCQ-AS1
Region_1914	chr10	6682513	6877888	195375	30	228	254.6515152	15.21688579	0.854849831	0.02505167	+	100507127	grey	0.103708536	FALSE	LINC00707
Region_1915	chr10	6709530	6740532	31002	7	45	62.71212121	7.877455957	0.913773193	0.043943834	+	108570035	grey	-0.327741078	FALSE	LINP1
Region_1916	chr10	6824275	7118469	294194	32	233	271.8181818	15.42217094	0.860109934	0.02633322	-	107984203	grey	-0.009609333	FALSE	LOC107984203
Region_1917	chr10	6848870	7118432	269562	31	227	265.2272727	15.52942166	0.856780964	0.027085401	-	105376387	grey	0.024827548	FALSE	LOC105376387
Region_9818	chr19	55675226	55709858	34632	55	327	381.7121212	26.52242311	0.858922592	0.017653628	+	29924	blue	0.547362485	FALSE	EPN1
Region_1919	chr10	7444298	7472040	27742	6	36	56.6969697	7.992245659	0.682196004	0.067258595	-	105376388	grey	0.300952566	FALSE	LINC02642
Region_1920	chr10	7559270	7666998	107728	47	335	379.9545455	17.81911836	0.87387427	0.018762017	-	80760	grey	-0.09995552	FALSE	ITIH5
Region_1921	chr10	7703316	7749520	46204	12	84	104.3333333	9.600213673	0.963524104	0.0207933	+	3698	grey	-0.290145182	FALSE	ITIH2
Region_1922	chr10	7750962	7787993	37031	15	80	113.6666667	11.5776088	0.783605834	0.04080836	-	22944	grey	0.29954938	FALSE	KIN
Region_1923	chr10	7788147	7807815	19668	7	34	58.5	8.638464963	0.56586736	0.041536965	+	509	grey	-0.043015669	FALSE	ATP5F1C
Region_1924	chr10	7818497	8016631	198134	44	311	357.4242424	20.80536958	0.892023444	0.016266026	+	83860	grey	0.17344114	FALSE	TAF3

Region_1925	chr10	8045378	8075198	29820	27	152	190.8939394	16.14561506	0.270810412	0.019816866	+	2625	grey	0.271461494	FALSE	GATA3
Region_1926	chr10	8050450	8053484	3034	8	32	53.40909091	9.05704532	0.082756257	0.048183552	-	399717	grey	0.221945194	FALSE	GATA3-AS1
Region_1927	chr10	8400860	8461863	61003	10	67	88.87878788	11.00002119	0.837343312	0.044040827	+	105376396	grey	0.153058239	FALSE	LOC105376396
Region_1928	chr10	9758783	9878060	119277	6	24	40.24242424	6.066454055	0.747157907	0.057495775	-	105376402	grey	-0.035745773	FALSE	LINC02663
Region_1929	chr10	10326875	10337876	11001	5	20	30.87878788	5.845485178	0.790214972	0.090976828	-	107984207	grey	0.183620792	FALSE	LOC107984207
Region_1930	chr10	10369809	10462361	92552	12	90	112.4848485	10.37191834	0.915375171	0.030217794	-	101928322	grey	-0.008759857	FALSE	CELF2-DT
Region_1931	chr10	10784437	10795047	10610	3	15	24.98484848	4.612666255	0.898757927	0.055865992	-	207107	grey	0.066379591	FALSE	SFTA1P
Region_1932	chr10	10798397	11336675	538278	139	1060	1164.727273	44.46002369	0.827048948	0.014806882	+	10659	grey	0.523139186	FALSE	CELF2
Region_1933	chr10	11071541	11105773	34232	9	51	72.12121212	7.59658861	0.94539576	0.049915767	-	439950	grey	0.00402135	FALSE	CELF2-AS2
Region_1934	chr10	11460510	11611666	151156	26	187	230.5151515	17.38190255	0.798636647	0.024358243	-	9712	grey	0.187772837	FALSE	USP6NL
Region_1935	chr10	11742366	11764070	21704	11	60	82.22727273	9.146324112	0.874611708	0.028599104	+	79746	grey	0.126739357	FALSE	ECHDC3
Region_1936	chr10	11823339	11872277	48938	47	321	368.5454545	21.59357584	0.808824159	0.02054445	+	254427	grey	-0.150540772	FALSE	PROSER2
Region_1937	chr1	11849608	11894700	45092	40	268	300.303030	17.4664246	0.85033559	0.02324822	-	219731	grey	0.100519662	FALSE	PROSER2-AS1

	0						3	7	4	9						
Region_1938	chr10	11920022	12043170	123148	53	395	458.1212121	24.64549586	0.889607792	0.010992946	-	26019	grey	0.132895722	FALSE	UPF2
Region_1939	chr10	12068954	12123221	54267	25	164	191.030303	13.30120839	0.938011227	0.012845222	+	55526	grey	-0.045448274	FALSE	DHTKD1
Region_1940	chr10	12129637	12169961	40324	26	153	191.2121212	14.62507347	0.87504724	0.022692725	+	55176	grey	0.008600875	FALSE	SEC61A2
Region_1941	chr10	12165330	12196144	30814	21	162	193.2272727	13.57463183	0.598766507	0.022172731	-	11164	grey	-0.197198451	FALSE	NUDT5
Region_1942	chr10	12195965	12250589	54624	28	173	218.9242424	15.14779558	0.79797626	0.022343889	+	8872	grey	0.139438062	FALSE	CDC123
Region_1943	chr10	12349547	12835545	485998	240	1832	1946.893939	52.49794979	0.88113267	0.009597387	+	57118	grey	-0.194559129	FALSE	CAMK1D
Region_1944	chr10	12877459	12965275	87816	26	177	215.530303	15.35249386	0.917448221	0.019033079	+	105376418	grey	-0.011110266	FALSE	LOC105376418
Region_1945	chr10	12896625	13099652	203027	52	338	407.6363636	21.36469868	0.87466596	0.018478908	-	83643	grey	0.127234162	FALSE	CCDC3
Region_1946	chr10	13099449	13138308	38859	15	82	109.3484848	11.88210345	0.827133829	0.019306508	+	10133	grey	0.017604297	FALSE	OPTN
Region_1947	chr10	13161558	13211110	49552	22	169	205.7121212	14.71447759	0.754784365	0.022282933	+	55388	grey	0.254752345	FALSE	MCM10
Region_1948	chr10	13221766	13234374	12608	6	31	46.90909091	7.111696266	0.920087185	0.048616273	-	221044	grey	-0.09775574	FALSE	UCMA
Region_1949	chr10	13277796	13302412	24616	13	72	93.06060606	9.66337859	0.728865576	0.02984433	-	5264	grey	0.268276555	FALSE	PHYH

Region_1950	chr10	13317428	13348298	30870	20	116	148.6818182	13.3256942	0.737674386	0.028340078	-	22929	grey	0.233564886	FALSE	SEPHS1
Region_1951	chr10	13391347	13400706	9359	4	23	35.25757576	5.757431602	0.933743689	0.049103147	+	105376419	grey	-0.158769516	FALSE	LOC105376419
Region_1952	chr10	13415202	13423133	7931	4	20	31.18181818	4.758503614	0.908247993	0.055343104	+	105376420	grey	-0.044873436	FALSE	LOC105376420
Region_1953	chr10	13438484	13529014	90530	38	279	328.0909091	19.51940043	0.887013794	0.018967207	-	222389	grey	0.049532972	FALSE	BEND7
Region_1954	chr10	13586939	13668445	81506	16	106	134.9545455	12.06703305	0.902665916	0.024643047	+	8559	grey	0.004028605	FALSE	PRPF18
Region_1955	chr10	13643706	14462142	818436	221	1700	1808.545455	57.40897325	0.811504842	0.011674339	-	55691	grey	-0.175061357	FALSE	FRMD4A
Region_1956	chr10	13991815	14007526	15711	4	22	32.53030303	5.533727905	0.773591697	0.102495996	+	105376425	grey	-0.184083938	FALSE	LOC105376425
Region_1957	chr10	14518557	14774897	256340	85	655	724.1969697	29.80770148	0.766101438	0.01584384	-	83641	grey	-0.105385571	FALSE	FAM107B
Region_1958	chr10	14652689	14660581	7892	3	14	24.01515152	4.727960771	0.583938144	0.073167765	+	105376428	grey	0.064827049	FALSE	
Region_1959	chr10	14652742	14661691	8949	3	14	24.01515152	4.727960771	0.583938144	0.073167765	+	105376429	grey	0.064827049	FALSE	LOC105376429
Region_1960	chr10	14819245	14838575	19330	5	27	37.92424242	5.452763889	0.586821756	0.021503529	-	441549	grey	0.135960475	FALSE	CDNF
Region_1961	chr10	14838306	14871741	33435	4	21	33.86363636	6.141626854	0.732640098	0.035548868	+	51182	grey	-0.099994304	FALSE	HSPA14
Region_1962	chr1	14878820	14904315	25495	18	91	112.469697	11.7030169	0.48347775	0.01471879	+	79723	grey	-0.02281331	FALSE	SUV39H2

	0							6	8	3				9		
Region_1963	chr10	14897359	14954432	57073	23	162	200.9848485	15.70741366	0.972794664	0.013268256	-	64421	grey	0.06700156	FALSE	DCLRE1C
Region_1964	chr10	14959388	14988050	28662	17	100	131.3787879	13.30328491	0.884753537	0.031005003	+	644890	grey	-0.087698418	FALSE	MEIG1
Region_1965	chr10	15015370	15021863	6493	5	19	30.15151515	6.330007715	0.907448602	0.055671854	-	105376432	grey	-0.195601492	FALSE	DCLRE1CP1
Region_1966	chr10	15032227	15073853	41626	25	140	179.9848485	13.38482772	0.915708711	0.029161989	+	55301	grey	0.001097839	FALSE	OLAH
Region_1967	chr10	15075475	15088776	13301	5	22	35.87878788	5.895278819	0.768407982	0.045694119	-	414149	grey	-0.173925266	FALSE	ACBD7
Region_1968	chr10	15097180	15139818	42638	18	122	148.530303	10.17269301	0.858062601	0.022434692	+	10557	grey	0.095683094	FALSE	RPP38
Region_1969	chr10	15102584	15168693	66109	29	210	246.5606061	15.82512976	0.862501079	0.020783126	-	9397	grey	0.148784798	FALSE	NMT2
Region_1970	chr10	15211643	15371289	159646	52	366	425.6212121	20.81254441	0.745446943	0.018740248	-	221061	grey	-0.010276653	FALSE	FAM171A1
Region_1971	chr10	15513954	15719922	205968	36	249	296.5	17.59479818	0.831747671	0.031718472	-	8516	grey	-0.16824139	FALSE	ITGA8
Region_1972	chr10	15778170	15860507	82337	12	82	103.9242424	9.968119344	0.903535743	0.039855937	-	80013	grey	0.327468895	FALSE	MINDY3
Region_1973	chr10	16436943	16513745	76802	22	165	201.4393939	13.9847486	0.915644756	0.014285886	+	9317	grey	-0.088117553	FALSE	PTER
Region_1974	chr10	16521714	16539737	18023	4	23	35.07575758	5.072734374	0.89997166	0.06357539	+	101928701	grey	-0.096811164	FALSE	

Region_1975	chr10	16590611	16817463	226852	55	457	520.2272727	23.92089235	0.901623808	0.011271026	-	6251	grey	0.11072786	FALSE	RSU1
Region_1976	chr10	16823966	17129811	305845	46	369	414.8030303	21.8211046	0.867996045	0.01736458	-	8029	grey	-0.11466258	FALSE	CUBN
Region_1977	chr10	17137336	17202054	64718	7	41	55.04545455	7.563946504	0.896379699	0.058435388	-	1787	grey	0.073757289	FALSE	TRDMT1
Region_1978	chr10	17214239	17230018	15779	3	19	27.86363636	4.580515133	0.362137264	0.037281889	-	100507347	grey	-0.109007249	FALSE	VIM-AS1
Region_1979	chr10	17315421	17454595	139174	30	213	247.6969697	17.67655527	0.736448607	0.025530495	-	338596	grey	0.292179214	FALSE	ST8SIA6
Region_1980	chr10	17386919	17413503	26584	3	17	30.74242424	5.616762292	0.929972974	0.060704341	+	100128098	grey	0.350178047	FALSE	ST8SIA6-AS1
Region_1981	chr10	17386936	17445758	58822	14	93	120.9090909	10.55349344	0.645280377	0.035133613	+	105376436	grey	0.099705121	FALSE	LOC105376436
Region_1982	chr10	17589032	17617374	28342	7	41	57.22727273	6.965398999	0.958338229	0.028952603	-	9200	grey	-0.066714505	FALSE	HACD1
Region_1983	chr10	17644151	17716824	72673	24	183	212.2727273	16.66091276	0.756648217	0.028097064	+	8027	grey	0.037544692	FALSE	STAM
Region_1984	chr10	17752201	17800868	48667	9	68	85.57575758	8.447230964	0.928358637	0.050065393	+	653567	grey	-0.055063642	FALSE	TMEM236
Region_1985	chr10	17809348	17911164	101816	16	104	121.969697	10.24164905	0.811578295	0.042059543	+	4360	grey	-0.178122213	FALSE	MRC1
Region_1986	chr10	17951839	18043292	91453	9	52	70.10606061	7.907404102	0.873585104	0.049632289	+	221074	grey	0.005994621	FALSE	SLC39A12
Region_1987	chr1	18140424	18543557	403133	78	592	680.909090	34.8454730	0.89189264	0.01496803	+	783	grey	-0.15244550	FALSE	CACNB2

	0						9	6	1	8				5		
Region_1988	chr10	18206127	18261504	55377	14	110	133.969697	10.88820067	0.887011133	0.030695211	-	107984213	grey	0.117617731	FALSE	LOC107984213
Region_1989	chr10	18513115	18659285	146170	31	195	239.4090909	17.77463817	0.914602213	0.015897572	-	221078	grey	0.053136503	FALSE	NSUN6
Region_1990	chr10	18659431	18681639	22208	3	14	28.24242424	5.746063819	0.982507483	0.026747262	+	221079	grey	0.182233003	FALSE	ARL5B
Region_1991	chr10	18710269	18748342	38073	7	43	60.04545455	8.762563408	0.870718183	0.064152752	+	105376440	grey	-0.177124387	FALSE	LOC105376440
Region_1992	chr10	19048801	19790401	741600	61	439	501.530303	26.94736733	0.86417618	0.024494982	+	340895	grey	0.04973869	FALSE	MALRD1
Region_1993	chr10	19816239	20289856	473617	43	298	338.5606061	17.96075104	0.921602096	0.019166648	+	84898	grey	0.102935049	FALSE	PLXDC2
Region_1994	chr10	20779973	21293011	513038	72	549	604.4545455	22.72929781	0.818414345	0.021750318	-	10529	grey	0.391534944	FALSE	NEBL
Region_1995	chr10	21340233	21372950	32717	11	65	87.54545455	10.77915627	0.933628472	0.032526531	-	105376444	grey	-0.078143686	FALSE	LINC02643
Region_1996	chr10	21513475	21526368	12893	7	40	55.68181818	7.779972495	0.148816843	0.024945293	-	387640	grey	-0.121819944	FALSE	SKIDA1
Region_1997	chr10	21524646	21743630	218984	82	553	617.0909091	29.86210734	0.801940509	0.01229679	+	8028	grey	-0.032689257	FALSE	MLLT10
Region_1998	chr10	21756548	22003769	247221	58	387	443.1363636	23.99736291	0.891374811	0.013856217	-	64215	grey	0.144321643	FALSE	DNAJC1
Region_1999	chr10	22316386	22326521	10135	6	13	28.36363636	5.658584666	0.006254209	0.019470754	+	23412	grey	-0.014772267	FALSE	COMMD3

Region_2000	chr10	22316388	22329542	13154	6	13	28.36363636	5.658584666	0.006254209	0.019470754	+	100532731	grey	-0.014772267	FALSE	COMMD3-BMI1
Region_2001	chr10	22345445	22417610	72165	17	107	132.9848485	10.84930685	0.834596965	0.023102199	+	9576	grey	0.256408066	FALSE	SPAG6
Region_2002	chr10	22391805	22454224	62419	11	63	83.21212121	9.502250991	0.664069904	0.026478077	+	105376449	grey	0.160301708	FALSE	LOC105376449
Region_2003	chr10	22534854	22714578	179724	51	380	424.7727273	22.43609417	0.78027197	0.021715555	-	5305	grey	0.163976469	FALSE	PIP4K2A
Region_2004	chr10	22928024	23038523	110499	14	90	111.6363636	11.72064105	0.930560635	0.025287907	+	219681	grey	-0.130284106	FALSE	ARMC3
Region_2005	chr10	22964926	23095189	130263	20	135	162.9090909	14.07532185	0.932256155	0.03028774	-	107984215	grey	0.117659409	FALSE	LOC107984215
Region_2006	chr10	23095579	23122013	26434	5	31	44.48484848	6.90427387	0.771992772	0.02602878	+	22921	grey	0.189675432	FALSE	MSRB2
Region_2007	chr10	23192312	23194245	1933	4	16	28.12121212	5.636461387	0.047636238	0.044636043	+	256297	grey	0.00974826	FALSE	PTF1A
Region_2008	chr10	23202696	23344845	142149	16	120	137.1363636	10.17974817	0.815801271	0.035283212	-	256815	grey	0.305542975	FALSE	C10orf67
Region_2009	chr10	23439075	23442377	3302	8	15	40.1969697	10.20591035	0.005796124	0.013998065	+	220213	grey	0.066280821	FALSE	OTUD1
Region_2010	chr10	23694746	24547848	853102	102	799	876.6212121	34.17005669	0.823799751	0.020357167	+	56243	grey	0.349533306	FALSE	KIAA1217
Region_2011	chr10	24583609	24723887	140278	30	217	255.6666667	17.61409692	0.78888846	0.022048968	-	57584	grey	0.102206931	FALSE	ARHGAP21
Region_2012	chr1	24848614	24952606	103992	19	132	161	15.8686919	0.89739137	0.02934993	-	56952	grey	0.283479469	FALSE	PRTFDC1

	0							6	4	3						
Region_2013	chr1<br>0	24952350	25161236	208886	46	331	379.393939<br>4	21.0032076<br>6	0.71500390<br>5	0.01960545<br>1	+	10192902<br>5	grey	-0.07964912<br>2	FALSE	LINC01516
Region_2014	chr1<br>0	24981979	25062279	80300	16	103	126.666666<br>7	11.6992219<br>8	0.60031710<br>6	0.02531470<br>1	-	219670	grey	-0.03165471<br>2	FALSE	ENKUR
Region_2015	chr1<br>0	25158072	25176276	18204	6	34	47.4090909<br>1	6.37055068<br>9	0.30236309<br>2	0.06078223<br>6	-	10012881<br>1	grey	0.009419871	FALSE	GPR158-AS1
Region_2016	chr1<br>0	25174802	25602229	427427	50	356	413.833333<br>3	25.7183462<br>4	0.75877258<br>8	0.02687982<br>7	+	57512	grey	-0.0280809	FALSE	GPR158
Region_2017	chr1<br>0	25651712	25732935	81223	6	33	47.8636363<br>6	6.43998653<br>5	0.85762258<br>5	0.06029842<br>1	+	10192905<br>2	grey	-0.20220386<br>3	FALSE	LINC00836
Region_2018	chr1<br>0	25934229	26212532	278303	33	212	281.666666<br>7	22.3554043<br>3	0.81484527<br>5	0.04400559<br>5	+	53904	grey	0.202239742	FALSE	MYO3A
Region_2019	chr1<br>0	26216665	26304558	87893	10	62	87.1212121<br>2	9.17440444<br>8	0.81731350<br>1	0.05033280<br>7	+	2572	grey	-0.23112678<br>9	FALSE	GAD2
Region_2020	chr1<br>0	26337473	26380859	43386	6	39	55.5909090<br>9	7.05577182<br>8	0.80741297<br>8	0.03130093<br>6	-	10798417<br>6	grey	-0.00453551<br>9	FALSE	
Region_2021	chr1<br>0	26438341	26567803	129462	32	241	272.424242<br>4	15.3252537<br>7	0.90402520<br>3	0.01738768<br>1	+	54518	grey	0.03832547	FALSE	APBB1IP
Region_2022	chr1<br>0	26577676	26622338	44662	12	75	102.166666<br>7	10.4874772<br>7	0.94383566	0.03427758<br>6	-	10192911<br>7	grey	-0.34197628<br>5	FALSE	LINC03028
Region_2023	chr1<br>0	26664206	26697295	33089	4	15	25.7121212<br>1	4.66002691<br>1	0.93100246<br>2	0.08432117<br>7	-	10537646<br>2	grey	0.155441204	FALSE	SELENOOLP
Region_2024	chr1<br>0	26697701	26746798	49097	16	100	123.363636<br>4	13.3193166<br>4	0.88620542<br>5	0.03326497<br>7	+	23590	grey	0.285237976	FALSE	PDSS1

Region_2025	chr10	26746593	26861087	114494	16	91	115.4848485	12.29539304	0.939506057	0.025647466	-	10006	grey	-0.084331587	FALSE	ABI1
Region_2026	chr10	26931206	26944418	13212	8	47	68.07575758	9.333662165	0.782271944	0.051522671	-	387644	grey	-0.035339659	FALSE	FAM238C
Region_2027	chr10	26973793	27100494	126701	15	104	127.3181818	11.66738926	0.929419352	0.034017625	-	22852	grey	-0.287286327	FALSE	ANKRD26
Region_2028	chr10	27110111	27155266	45155	7	50	71.42424242	8.921987716	0.748831587	0.062341646	-	10730	grey	0.015759869	FALSE	YME1L1
Region_2029	chr10	27154824	27187953	33129	15	99	126.4090909	11.27963075	0.791341691	0.024089318	+	84930	grey	0.03694145	FALSE	MASTL
Region_2030	chr10	27168135	27243046	74911	26	150	202.8787879	16.40123368	0.958915623	0.019260532	-	91452	grey	-0.001969226	FALSE	ACBD5
Region_2031	chr10	27258762	27288824	30062	8	37	58.53030303	8.625935306	0.700984566	0.037400666	+	101060171	grey	0.004266402	FALSE	ODAD2P1
Region_2032	chr10	27504174	27543207	39033	6	30	48.40909091	7.327455793	0.484514142	0.033336698	+	22931	grey	-0.023601921	FALSE	RAB18
Region_2033	chr10	27564430	27594588	30158	3	13	27.04545455	5.671607609	0.966094773	0.042754085	+	105376463	grey	-0.175578862	FALSE	LINC02680
Region_2034	chr10	27672874	27746060	73186	13	78	98.39393939	10.79013622	0.692088207	0.032686528	-	283078	grey	-0.084418022	FALSE	MKX
Region_2035	chr10	27744786	27767794	23008	6	37	56.56060606	7.60592641	0.529518885	0.054298806	+	101929202	grey	0.212790361	FALSE	MKX-AS1
Region_2036	chr10	27775186	27999079	223893	34	234	278.2575758	16.13625694	0.910878983	0.025050418	-	55130	grey	-0.23758518	FALSE	ODAD2
Region_2037	chr1	28050993	28334486	283493	41	299	333.136363	17.7529077	0.89297661	0.02041682	-	143098	grey	0.230515641	FALSE	MPP7

	0						6	7	5	7						
Region_2038	chr10	28433008	28495813	62805	13	80	100.9545455	9.322317008	0.878729319	0.04917749	-	105376468	grey	-0.492935259	FALSE	LINC02652
Region_2039	chr10	28522652	28533066	10414	3	16	24.66666667	4.315743392	0.644433583	0.032291247	-	220906	grey	0.096096836	FALSE	WAC-AS1
Region_2040	chr10	28532493	28623112	90619	23	157	189.8333333	12.86567686	0.868921844	0.022732144	+	51322	grey	0.257902131	FALSE	WAC
Region_2041	chr10	28677510	28682932	5422	3	15	23.39393939	5.077488137	0.327469458	0.014638168	+	25805	grey	0.102674389	FALSE	BAMBI
Region_2042	chr10	28743506	28941910	198404	50	359	407	17.79801199	0.847529206	0.018692618	+	101929218	grey	-0.052674974	FALSE	LINC01517
Region_2043	chr10	28789188	28796113	6925	5	23	35.12121212	5.709681795	0.955545317	0.043779107	-	100507605	grey	-0.044850271	FALSE	LINC00837
Region_2044	chr10	28846408	28882311	35903	5	33	42.04545455	5.390680899	0.776061825	0.072038626	+	283080	grey	-0.100322099	FALSE	C10orf126
Region_2045	chr10	29289061	29318328	29267	8	47	70.34848485	7.492544857	0.930703544	0.034049689	+	84569	grey	0.273668218	FALSE	LYZL1
Region_2046	chr10	29373027	29409366	36339	4	22	31.95454545	5.002866311	0.893330885	0.064519406	-	105376473	grey	0.074386091	FALSE	LOC105376473
Region_2047	chr10	29409402	29512266	102864	27	189	231.7272727	17.85027081	0.815346388	0.026344551	+	102724316	grey	0.020273507	FALSE	SVIL-AS1
Region_2048	chr10	29457338	29736959	279621	75	594	643.8333333	26.51071385	0.818769365	0.014481537	-	6840	grey	-0.074232743	FALSE	SVIL
Region_2049	chr10	29991111	30001119	10008	6	34	47.81818182	5.875396133	0.830083607	0.037973866	-	107984218	grey	-0.131085927	FALSE	LOC107984218

Region_2050	chr10	30012803	30115494	102691	40	290	329.1969697	18.73147881	0.782834945	0.017340915	-	57608	grey	-0.036612709	FALSE	JCAD
Region_2051	chr10	30309801	30374441	64640	26	150	197.6060606	16.89230939	0.921376857	0.023377896	-	55149	grey	-0.200235586	FALSE	MTPAP
Region_2052	chr10	30434021	30461833	27812	4	19	35.83333333	5.779495143	0.480039584	0.044646883	+	1326	grey	0.040039308	FALSE	MAP3K8
Region_2053	chr10	30611779	30629762	17983	8	35	53.03030303	7.049210969	0.873724562	0.046421583	-	119180	grey	0.141938055	FALSE	LYZL2
Region_2054	chr10	30655651	30717266	61615	18	108	134.530303	12.13795027	0.939582663	0.028996843	+	645954	grey	0.029515954	FALSE	SVIL2P
Region_2055	chr10	30820207	31031937	211730	45	325	366.3636364	21.8974086	0.831903339	0.020220654	-	220929	grey	0.397717752	FALSE	ZNF438
Region_2056	chr10	31033089	31143813	110724	24	176	200.1969697	14.1467253	0.789588605	0.03481841	+	105376481	grey	0.326359782	FALSE	LOC105376481
Region_2057	chr10	31133864	31143842	9978	4	26	38.22727273	6.473128965	0.740770388	0.038236999	+	105376483	grey	0.261517525	FALSE	LOC105376483
Region_2058	chr10	31187715	31261910	74195	10	58	75.75757576	8.135507371	0.933830298	0.033501983	+	101929352	grey	-0.174642867	FALSE	LINC02664
Region_2059	chr10	31206278	31320447	114169	19	113	136.3181818	10.19978747	0.696994957	0.031209794	-	220930	grey	-0.064297309	FALSE	ZEB1-AS1
Region_2060	chr10	31318495	31529814	211319	33	210	241.1212121	17.13612033	0.454573605	0.013607936	+	6935	grey	0.088376837	FALSE	ZEB1
Region_2061	chr10	31693082	31707487	14405	4	22	36.04545455	6.675861957	0.570720078	0.078360469	-	101929951	grey	0.261687816	FALSE	MACORIS
Region_2062	chr1	31805398	31928876	123478	16	104	133.196969	12.7233518	0.89770502	0.01878870	-	94134	grey	0.08229585	FALSE	ARHGAP12

	0						7	8	7	7						
Region_2063	chr10	32009015	32056425	47410	13	78	103.5	10.10293179	0.75443884	0.032702026	-	3799	grey	-0.250669835	FALSE	KIF5B
Region_2064	chr10	32267751	32378798	111047	28	174	213.469697	16.46275021	0.82404037	0.020620034	-	80314	grey	-0.052531871	FALSE	EPC1
Region_2065	chr10	32413499	32446076	32577	3	12	25.40909091	4.995312488	0.688923413	0.127752551	-	101929431	grey	-0.323391266	FALSE	LOC101929431
Region_2066	chr10	32446140	32882874	436734	49	338	399.3787879	20.6090086	0.92147613	0.023449367	+	79741	grey	-0.034152351	FALSE	CCDC7
Region_2067	chr10	32887273	33005792	118519	13	88	113.9848485	10.16251396	0.788689626	0.024378846	-	3688	grey	-0.021849362	FALSE	ITGB1
Region_2068	chr10	32958437	33082409	123972	14	91	116.969697	11.2290416	0.720886663	0.02762336	+	101929475	grey	0.148736581	FALSE	ITGB1-DT
Region_2069	chr10	33073486	33116789	43303	3	17	26.03030303	4.392194815	0.531595901	0.081381611	-	101929447	grey	-0.260044286	FALSE	IATPR
Region_2070	chr10	33177492	33336262	158770	38	261	308.1969697	20.39624377	0.697870932	0.021543033	-	8829	grey	-0.147116581	FALSE	NRP1
Region_2071	chr10	33578931	33600040	21109	6	37	53.74242424	7.039035349	0.812335354	0.066012658	-	105376490	grey	-0.180276006	FALSE	LINC02628
Region_2072	chr10	34109560	34815325	705765	114	887	975.6212121	33.54251729	0.871796906	0.015571863	-	56288	grey	-0.157475314	FALSE	PARD3
Region_2073	chr10	35008504	35127020	118516	40	250	309.0151515	22.03039329	0.841373016	0.019384834	-	8453	grey	-0.152800714	FALSE	CUL2
Region_2074	chr10	35126791	35212958	86167	30	216	247.6212121	16.86759766	0.795129849	0.019899823	+	1390	grey	0.082921379	FALSE	CREM

Region_2075	chr10	35247025	35572669	325644	93	663	731.8636364	32.69339447	0.831620825	0.010786553	+	219771	grey	0.216314033	FALSE	CCNY
Region_2076	chr10	35314552	35336401	21849	5	27	41.25757576	7.305025891	0.781682417	0.032603757	-	107984220	grey	0.024086496	FALSE	LOC107984220
Region_2077	chr10	35605341	35608935	3594	4	14	31.75757576	6.901639952	0.335796103	0.075149279	+	219770	grey	0.122608354	FALSE	GJD4
Region_2078	chr10	35638247	35642296	4049	4	14	33.68181818	5.839281017	0.00714764	0.019930325	-	8325	grey	0.073087725	FALSE	FZD8
Region_2079	chr10	35641097	35647826	6729	6	31	44.3030303	6.184272103	0.25574848	0.041641091	+	105376494	grey	0.137687902	FALSE	LOC105376494
Region_2080	chr10	37125598	37315801	190203	14	102	122.2424242	11.34095452	0.836803437	0.041837657	+	91074	grey	-0.123117462	FALSE	ANKRD30A
Region_2081	chr10	37309185	37347031	37846	3	17	24.89393939	3.815469677	0.785708406	0.115789168	+	101929520	grey	0.155793735	FALSE	LINC00993
Region_2082	chr10	37775371	37858106	82735	7	39	58.45454545	7.876316629	0.824192718	0.06610439	-	57209	grey	-0.178835125	FALSE	ZNF248
Region_2083	chr10	37949573	37976647	27074	8	43	59.78787879	6.604229767	0.731342666	0.03906291	-	219749	grey	-0.23125589	FALSE	ZNF25
Region_2084	chr10	38010650	38065088	54438	9	69	85.36363636	8.17244218	0.873324062	0.027103887	+	7581	grey	-0.171274803	FALSE	ZNF33A
Region_2085	chr10	38094334	38360098	265764	35	211	261.7727273	20.50050315	0.825723637	0.029103334	+	7587	grey	-0.160486111	FALSE	ZNF37A
Region_2086	chr10	38164648	38212417	47769	8	40	64.72727273	9.991604868	0.904413532	0.04476448	+	100129055	grey	0.056453222	FALSE	CCNYL4
Region_2087	chr1	42254072	42367993	113921	5	20	32.8787878	5.55951063	0.77626566	0.07167700	-	441666	grey	0.036698003	FALSE	LOC441666

	0						8	6	2	5						
Region_2088	chr10	42408168	42475349	67181	7	35	56.33333333	8.263605719	0.826918484	0.058818212	-	414194	grey	-0.357303047	FALSE	CCNYL2
Region_2089	chr10	42475480	42495337	19857	3	15	26.28787879	5.504585925	0.702675813	0.078600898	+	84856	grey	0.075051163	FALSE	LINC00839
Region_2090	chr10	42513510	42552832	39322	7	42	55.27272727	7.31604234	0.833018104	0.031428548	-	100129482	grey	0.204070652	FALSE	ZNF37BP
Region_2091	chr10	42574185	42638570	64385	6	31	47.71212121	7.238285287	0.645325137	0.038419739	-	7582	grey	0.118288624	FALSE	ZNF33B
Region_2092	chr10	42638252	42645542	7290	6	39	60.01515152	8.278781821	0.5721332	0.061418429	+	105378268	grey	-0.191176265	FALSE	LOC105378268
Region_2093	chr10	42644445	42691723	47278	21	150	187.1818182	15.44509789	0.896296171	0.024746225	-	101929397	grey	-0.137158995	FALSE	LINC01518
Region_2094	chr10	42782795	42834937	52142	13	87	109.8484848	11.34933739	0.892169711	0.023110021	+	9790	grey	-0.057926493	FALSE	BMS1
Region_2095	chr10	43077064	43130351	53287	15	59	87.51515152	8.776265938	0.718052999	0.044275566	+	5979	grey	0.179784055	FALSE	RET
Region_2096	chr10	43138445	43185302	46857	8	48	65.6969697	8.249885215	0.879771606	0.04070015	+	55454	grey	-0.241311968	FALSE	CSGALNACT2
Region_2097	chr10	43194535	43267065	72530	28	154	191.6060606	13.19193085	0.634220774	0.027759099	-	221002	grey	-0.139671927	FALSE	RASGEF1A
Region_2098	chr10	43325833	43350792	24959	17	75	108.0909091	15.01663413	0.811218296	0.03819033	-	107984226	grey	0.101955349	FALSE	LOC107984226
Region_2099	chr10	43385617	43409186	23569	9	49	66.83333333	8.862337482	0.607800332	0.029271872	-	3185	grey	-0.101740128	FALSE	HNRNPF

Region_2100	chr10	43436841	43483181	46340	27	169	206	16.58358781	0.784219513	0.01868198	+	642819	grey	-0.014933424	FALSE	ZNF487
Region_2101	chr10	43556344	43574618	18274	15	102	130.7727273	11.52094475	0.598231937	0.024110031	-	8187	grey	0.041227995	FALSE	ZNF239
Region_2102	chr10	43628817	43674703	45886	18	115	140.1818182	13.3530856	0.551599063	0.034467224	+	414201	grey	0.083205691	FALSE	ZNF32-AS3
Region_2103	chr10	43778946	43862005	83059	27	179	213.2878788	15.65175355	0.86953746	0.026335137	+	105378275	grey	0.336962028	FALSE	LOC105378275
Region_2104	chr10	43845189	43901004	55815	16	110	143.8787879	13.62973576	0.832324835	0.039350218	+	100506835	grey	-0.040009368	FALSE	LINC00840
Region_2105	chr10	43900874	43912369	11495	7	52	66.90909091	7.316424669	0.916019456	0.044037532	-	105378277	grey	-0.123784797	FALSE	LINC02659
Region_2106	chr10	43909244	43981102	71858	20	151	178.9848485	13.55160215	0.836118781	0.037199801	+	283033	grey	-0.279076364	FALSE	LINC00841
Region_2107	chr10	44282489	44386493	104004	20	133	158.5909091	12.70006332	0.794087149	0.037530925	-	6387	grey	0.019471707	FALSE	CXCL12
Region_2108	chr10	44793119	44959724	166605	43	287	328.4090909	19.91673226	0.766577275	0.019213776	-	220980	grey	-0.26343145	FALSE	TMEM72-AS1
Region_2109	chr10	44911316	44937010	25694	5	34	50.04545455	7.043669975	0.693094739	0.042976386	+	643236	grey	-0.013526762	FALSE	TMEM72
Region_2110	chr10	44959407	44995891	36484	10	62	79.45454545	9.13687679	0.711651962	0.038791089	+	83937	grey	0.230530517	FALSE	RASSF4
Region_2111	chr10	44970981	44978809	7828	3	14	28.68181818	5.230558408	0.309540014	0.091667085	-	11067	grey	0.141415753	FALSE	DEPP1
Region_2112	chr1	45016080	45154606	138526	18	130	159.742424	13.9165762	0.90172788	0.03099056	-	10013330	grey	-0.20106540	FALSE	RSU1P2

	0						2	9	8	4		8		9		
Region_2113	chr10	45147394	45176925	29531	3	12	24.62121212	5.137389794	0.916042979	0.059761333	+	338579	grey	-0.210212082	FALSE	ANKRD30BP3
Region_2114	chr10	45615500	45672780	57280	12	72	91.33333333	9.27085314	0.975563465	0.016656417	-	93550	grey	-0.063033639	FALSE	ZFAND4
Region_2115	chr10	45678692	45700532	21840	4	20	28.62121212	6.494412158	0.965657547	0.050226612	+	653234	grey	0.004095975	FALSE	AGAP10P
Region_2116	chr10	45727200	45792964	65764	10	55	74.24242424	7.720224401	0.872886142	0.020805323	+	253725	grey	0.208132811	FALSE	WASHC2C
Region_2117	chr10	45825594	45853875	28281	5	20	34.77272727	8.380922392	0.977036185	0.02977395	-	119016	grey	0.169998467	FALSE	AGAP4
Region_2118	chr10	45854093	45892483	38390	14	71	91.39393939	12.21081335	0.970593141	0.019944922	-	114004351	grey	-0.390829332	FALSE	PARGP1-AGAP4
Region_2119	chr10	45862609	45972428	109819	29	159	201.6212121	15.1197936	0.906996701	0.016179908	-	728407	grey	-0.173729029	FALSE	PARGP1
Region_2120	chr10	45972489	46003742	31253	4	19	35.57575758	7.83887866	0.953719264	0.042611203	+	100287932	grey	-0.228229218	FALSE	TIMM23
Region_2121	chr10	46005088	46030623	25535	6	30	40.68181818	5.802278308	0.537443055	0.084667874	-	8031	grey	-0.227782411	FALSE	NCOA4
Region_2122	chr10	46033307	46048180	14873	3	14	20.36363636	3.79325865	0.961745676	0.045255571	-	4477	grey	-0.057920416	FALSE	MSMB
Region_2123	chr10	46233885	46273557	39672	6	26	44.98484848	9.474650266	0.766452379	0.065025131	-	100996567	grey	0.077049432	FALSE	ANTXRLP1
Region_2124	chr10	46286345	46330207	43862	7	41	55.95454545	9.949768947	0.575979555	0.070325279	+	195977	grey	-0.363114424	FALSE	ANTXRL

Region_2125	chr10	46375752	46537864	162112	12	100	142.0909091	25.82886118	0.869357997	0.047860254	+	643650	grey	-0.113916622	FALSE	LINC00842
Region_2126	chr10	46541736	46556658	14922	3	34	52.12121212	9.859809877	0.787838679	0.045008176	-	9721	grey	0.29858183	FALSE	GPRIN2
Region_2127	chr10	46759019	46786770	27751	20	38	120.0757576	35.47493616	0.824813104	0.069441833	-	414212	grey	-0.241103939	FALSE	GLUD1P2
Region_2128	chr10	46786674	46811989	25315	5	11	39.31818182	11.99892186	0.776955315	0.055957858	+	399761	grey	-0.21282479	FALSE	BMS1P1
Region_2129	chr10	46911396	47002488	91092	8	29	49.34848485	7.667097383	0.932038331	0.050997794	+	26095	grey	0.306713017	FALSE	PTPN20
Region_2130	chr10	47207445	47224994	17549	3	14	28.54545455	5.215898236	0.937651322	0.055788812	+	101929216	grey	0.248325164	FALSE	
Region_2131	chr10	47348363	47357881	9518	4	23	36.25757576	6.084812382	0.917566318	0.050558672	+	5949	grey	0.063647437	FALSE	RBP3
Region_2132	chr10	47460162	47991877	531715	43	251	384.4242424	76.02470461	0.750901731	0.020050608	-	653145	grey	0.130098453	FALSE	ANXA8
Region_2133	chr10	47689707	47730436	40729	5	16	34.98484848	9.445377256	0.30426466	0.07348232	+	439965	grey	-0.071836027	FALSE	SHLD2P3
Region_2134	chr10	48153088	48274696	121608	18	137	167.1818182	13.25014844	0.892821799	0.018559918	-	143162	grey	-0.102857857	FALSE	FRMPD2
Region_2135	chr10	48306639	48439360	132721	25	178	211.8939394	14.94516005	0.937900783	0.020916121	+	5599	grey	0.09578429	FALSE	MAPK8
Region_2136	chr10	48446036	48656265	210229	36	263	294.969697	17.2510599	0.670385581	0.021375464	-	58504	grey	-0.117600643	FALSE	ARHGAP22
Region_2137	chr1	48684873	48982956	298083	55	464	532.075757	25.1012170	0.88660072	0.01389328	+	57705	grey	0.252835247	FALSE	WDFY4

	0						6	1	4	2						
Region_2138	chr10	48883955	48939840	55885	12	86	115.6818182	13.31414412	0.939083816	0.028954597	-	474354	grey	-0.061051332	FALSE	LRRC18
Region_2139	chr10	49014236	49115522	101286	14	79	110.0606061	12.80850533	0.919327512	0.037772244	-	196740	grey	0.127362945	FALSE	VSTM4
Region_2140	chr10	49364066	49396089	32023	8	42	58.98484848	6.342754617	0.391830768	0.042263383	-	644168	grey	0.144716436	FALSE	DRGX
Region_2141	chr10	49454168	49539538	85370	17	137	170.5454545	14.25501997	0.763284323	0.024048341	-	2074	grey	0.35664809	FALSE	ERCC6
Region_2142	chr10	49609095	49667942	58847	23	168	199.3787879	13.61307646	0.664336559	0.022480138	+	1103	grey	0.066714261	FALSE	CHAT
Region_2143	chr10	49610310	49612720	2410	4	31	41.83333333	6.257508311	0.089706248	0.05896593	+	6572	grey	0.103766889	FALSE	SLC18A3
Region_2144	chr10	49679651	49710261	30610	6	44	56.77272727	6.621167581	0.74748083	0.064753885	+	282966	grey	0.214753494	FALSE	C10orf53
Region_2145	chr10	49734641	49762379	27738	14	72	91.18181818	8.5573344	0.865313285	0.045132645	-	55753	grey	0.195514338	FALSE	OGDHL
Region_2146	chr10	49818279	49970203	151924	30	182	219.3181818	14.42130805	0.904917926	0.013028918	-	8505	grey	0.044486076	FALSE	PARG
Region_2147	chr10	49942049	49974850	32801	5	28	40.45454545	5.550278768	0.766303094	0.034738079	+	100652748	grey	0.406722407	FALSE	TIMM23B
Region_2148	chr10	49942056	50011654	69598	11	64	80.71212121	8.58400149	0.881568555	0.021901827	+	113218477	grey	0.351415957	FALSE	TIMM23B-AGAP6
Region_2149	chr10	49988318	50010589	22271	3	10	18.98484848	4.104378241	0.974963743	0.045012614	+	414189	grey	0.280790719	FALSE	AGAP6

Region_2150	chr10	50021182	50067803	46621	7	28	46.24242424	8.980424191	0.933326417	0.037488381	-	100421577	grey	-0.098748449	FALSE	FAM21EP
Region_2151	chr10	50067888	50133509	65621	7	32	43.28787879	5.74527271	0.92972325	0.051091403	+	387680	grey	-0.299423834	FALSE	WASHC2A
Region_2152	chr10	50182778	50279720	96942	8	57	72.65151515	7.993251816	0.872083607	0.043723967	-	56624	grey	0.131156139	FALSE	ASAH2
Region_2153	chr10	50305586	50625163	319577	48	366	409.7575758	18.5389906	0.786931159	0.01929947	-	259230	grey	0.051976745	FALSE	SGMS1
Region_2154	chr10	50624943	50641451	16508	7	47	70	10.53930373	0.23952143	0.032140125	+	104355295	grey	0.215451617	FALSE	SGMS1-AS1
Region_2155	chr10	50739318	50816495	77177	6	35	54.93939394	8.968112378	0.453777712	0.043785112	+	653308	grey	0.316518526	FALSE	ASAH2B
Region_2156	chr10	50799409	50885675	86266	16	117	145.2424242	14.54548368	0.832106109	0.048790486	-	29974	grey	0.359544654	FALSE	A1CF
Region_2157	chr10	50990888	52298423	1307535	130	1042	1147.090909	36.25496349	0.845934834	0.016850704	+	5592	grey	0.085924681	FALSE	PRKG1
Region_2158	chr10	52230398	52314507	84109	7	42	62.24242424	7.561157026	0.643057081	0.049069768	-	100506939	grey	-0.233943699	FALSE	PRKG1-AS1
Region_2159	chr10	52450874	52755404	304530	14	99	120.8333333	11.86667387	0.893243235	0.037067636	-	101928687	grey	0.002474501	FALSE	LNCAROD
Region_2160	chr10	52556702	52755476	198774	6	31	48.16666667	7.954840487	0.909848212	0.060519984	-	105378305	grey	0.089813995	FALSE	LOC105378305
Region_2161	chr10	53802771	55627942	1825171	125	1047	1127.878788	41.88427836	0.798251121	0.019399921	-	65217	grey	0.33257921	FALSE	PCDH15
Region_2162	chr1	54486230	54656051	169821	20	147	180.136363	12.9209268	0.75099420	0.03270976	+	10537831	grey	0.222773546	FALSE	LOC105378311

	0						6	1	4	8		1				
Region_2163	chr10	58191517	58267894	76377	7	32	47.33333333	8.274768569	0.840831419	0.034099545	-	253430	grey	-0.158200479	FALSE	IPMK
Region_2164	chr10	58269162	58289586	20424	4	13	31.06060606	5.937828632	0.614713553	0.064359473	+	55847	grey	-0.137626647	FALSE	CISD1
Region_2165	chr10	58334979	58370751	35772	3	11	18.8030303	4.077026431	0.662281914	0.061342983	+	7321	grey	-0.070647801	FALSE	UBE2D1
Region_2166	chr10	58512872	58831435	318563	23	157	193.0454545	12.6386852	0.862616737	0.027403469	+	80114	grey	-0.055632736	FALSE	BICC1
Region_2167	chr10	58999482	59066396	66914	5	31	43.68181818	5.727555771	0.807345195	0.061452309	+	102724768	grey	-0.065323984	FALSE	LOC102724768
Region_2168	chr10	59176643	59247774	71131	3	12	20.63636364	4.327501904	0.884823159	0.084806581	+	84457	grey	-0.043789891	FALSE	PHYHIPL
Region_2169	chr10	59246130	59363181	117051	8	44	53.8030303	7.222229992	0.779141088	0.05634317	-	220965	grey	0.217904975	FALSE	FAM13C
Region_2170	chr10	59594952	59650345	55393	3	16	26.71212121	4.467885928	0.927460356	0.085336671	+	105378319	grey	0.217439409	FALSE	LOC105378319
Region_2171	chr10	59650764	59736002	85238	8	44	60.09090909	7.721552799	0.791735755	0.05829303	-	220963	grey	-0.021916061	FALSE	SLC16A9
Region_2172	chr10	59788747	59906556	117809	11	64	86.96969697	10.22812066	0.85506759	0.024924428	-	8030	grey	0.004396308	FALSE	CCDC6
Region_2173	chr10	60026298	60733490	707192	66	491	537.4848485	21.70802221	0.853658271	0.028496571	-	288	grey	0.348351579	FALSE	ANK3
Region_2174	chr10	60507501	60733324	225823	19	115	146.5606061	9.546854154	0.869396766	0.035396575	-	107984292	grey	0.139290524	FALSE	

Region_2175	chr10	60778331	60794852	16521	4	24	36.27272727	6.359839027	0.732238903	0.023969441	+	983	grey	0.144441868	FALSE	CDK1
Region_2176	chr10	60869438	61001440	132002	10	52	71.89393939	8.171486613	0.854367222	0.030924413	-	9886	grey	0.229750731	FALSE	RHOBTB1
Region_2177	chr10	61001117	61027604	26487	3	12	21.96969697	4.732765323	0.539222731	0.083523931	+	100507058	grey	0.18247086	FALSE	LINC00845
Region_2178	chr10	61406642	61453381	46739	3	23	32.54545455	5.692591186	0.900371002	0.064280305	-	219623	grey	0.213630449	FALSE	TMEM26
Region_2179	chr10	61452639	61481956	29317	8	56	76.10606061	9.00620092	0.758265036	0.048616258	+	101928781	grey	-0.380387983	FALSE	TMEM26-AS1
Region_2180	chr10	61662929	61766766	103837	14	82	104.8636364	11.25902901	0.765553791	0.034535886	+	219621	grey	0.203084348	FALSE	CABCOCO1
Region_2181	chr10	61781745	61821246	39501	4	16	27.59090909	4.673912124	0.787049344	0.108866016	-	107984237	grey	0.144942305	FALSE	LINC02625
Region_2182	chr10	61901684	62096944	195260	28	211	244.6969697	16.28209937	0.827133768	0.034219942	+	84159	grey	0.063015964	FALSE	ARID5B
Region_2183	chr10	62183035	62268844	85809	6	19	35.56060606	6.624264924	0.661529182	0.104797406	-	219790	grey	0.313295474	FALSE	RTKN2
Region_2184	chr10	62339553	62375166	35613	5	35	47.78787879	6.517468011	0.739676647	0.073758213	-	283045	grey	0.270973323	FALSE	LOC283045
Region_2185	chr10	62374192	62672011	297819	28	195	230.2575758	15.50911455	0.822369382	0.023186707	+	22891	grey	-0.216491357	FALSE	ZNF365
Region_2186	chr10	62811996	62819167	7171	4	11	25.60606061	5.582607022	0.005686328	0.016443191	-	1959	grey	0.270731759	FALSE	EGR2
Region_2187	chr1	62814433	62919907	105474	12	69	88.8939393	9.20723593	0.53684769	0.02562713	-	10798401	grey	0.198431287	FALSE	LOC107984012

	0						9		6	4		2				
Region_2188	chr10	63133247	63155031	21784	6	38	55.6969697	7.027352488	0.79685426	0.029023133	+	29982	grey	-0.088146477	FALSE	NRBF2
Region_2189	chr10	63167221	63521850	354629	38	288	316.8939394	18.38823101	0.81660435	0.014852009	-	221037	grey	-0.254102315	FALSE	JMJD1C
Region_2190	chr10	63521401	63625128	103727	10	55	72.1969697	8.633066479	0.789158823	0.02980307	+	221035	grey	0.028768425	FALSE	REEP3
Region_2191	chr10	63630672	63655769	25097	5	33	44.09090909	5.11927661	0.479666806	0.057820695	+	101929846	grey	0.015557511	FALSE	LOC101929846
Region_2192	chr10	64901136	65017639	116503	12	80	99.36363636	8.955679528	0.806604448	0.047199794	-	101928887	grey	0.038825732	FALSE	LINC02671
Region_2193	chr10	65570338	65781067	210729	12	69	92.57575758	11.03011347	0.710981513	0.045115946	+	101928913	grey	0.071489136	FALSE	LINC01515
Region_2194	chr10	65912457	67763637	1851180	131	997	1071.893939	37.71477115	0.842783615	0.01585705	-	29119	grey	-0.225613898	FALSE	CTNNA3
Region_2195	chr10	66079243	66118326	39083	4	22	32.75757576	5.209637832	0.84358529	0.076307752	+	105378340	grey	-0.105394117	FALSE	LOC105378340
Region_2196	chr10	66926036	67101551	175515	12	77	101.2878788	9.775582063	0.727517402	0.04178556	+	347731	grey	-0.181059478	FALSE	LRRTM3
Region_2197	chr10	67796669	67838188	41519	4	19	31.33333333	4.582016128	0.966622046	0.0304769	-	56521	grey	-0.235749354	FALSE	DNAJC12
Region_2198	chr10	67884656	67918390	33734	4	23	35.56060606	6.031019273	0.963429405	0.036082783	+	23411	grey	-0.286888712	FALSE	SIRT1
Region_2199	chr10	67921899	68075301	153402	9	47	64.03030303	8.034482793	0.867642093	0.018920183	-	26091	grey	0.064681597	FALSE	HERC4

Region_2200	chr10	68087897	68212017	124120	13	83	105.2272727	10.26539438	0.854127716	0.054121941	+	84665	grey	-0.236986877	FALSE	MYPN
Region_2201	chr10	68282660	68333049	50389	3	13	24.3030303	5.14398737	0.650671333	0.042391344	-	64081	grey	0.234512397	FALSE	PBLD
Region_2202	chr10	68331174	68343193	12019	3	18	31.60606061	5.557749373	0.644522346	0.029431167	+	3189	grey	0.126621846	FALSE	HNRNPH3
Region_2203	chr10	68341107	68407294	66187	8	41	55.24242424	7.698272745	0.837026116	0.042422686	-	55680	grey	0.029451819	FALSE	RUFY2
Region_2204	chr10	68414064	68472121	58057	7	32	49.96969697	7.509931265	0.840539382	0.018446752	-	1763	grey	0.152678142	FALSE	DNA2
Region_2205	chr10	68477998	68527523	49525	11	69	80.43939394	6.344297956	0.973819898	0.02011465	-	8034	grey	0.072815116	FALSE	SLC25A16
Region_2206	chr10	68560337	68694487	134150	14	74	102.3636364	10.84099962	0.906602909	0.021929575	+	80312	grey	-0.138127398	FALSE	TET1
Region_2207	chr10	68721012	68792377	71365	17	102	137.0909091	14.35883783	0.961328195	0.01922735	+	55749	grey	0.075351656	FALSE	CCAR1
Region_2208	chr10	68827531	68895432	67901	10	57	74	7.601619261	0.963662067	0.027368321	+	219736	grey	0.026893813	FALSE	STOX1
Region_2209	chr10	68901286	68946847	45561	7	51	65.72727273	6.844527189	0.953351696	0.02632379	+	79009	grey	-0.080078191	FALSE	DDX50
Region_2210	chr10	69123512	69174412	50900	6	33	45.18181818	6.287739207	0.96731229	0.022694962	+	9559	grey	0.120306947	FALSE	VPS26A
Region_2211	chr10	69180234	69209099	28865	4	24	32.01515152	4.909934746	0.971505259	0.039812278	+	6832	grey	0.046305007	FALSE	SUPV3L1
Region_2212	chr1	69220332	69267552	47220	4	20	33.6515151	5.41931708	0.94163636	0.03464103	+	80201	grey	0.212656986	FALSE	HKDC1

	0						5	5	6	4						
Region_2213	chr10	69269984	69401884	131900	33	242	279.6363636	15.93799173	0.668524716	0.017763922	+	3098	grey	-0.142831711	FALSE	HK1
Region_2214	chr10	69403903	69416918	13015	6	31	50.37878788	7.717642429	0.544773115	0.060923405	-	6865	grey	-0.248572399	FALSE	TACR2
Region_2215	chr10	69451465	69544257	92792	31	201	249.1818182	19.56520388	0.477693037	0.018857247	+	23555	grey	0.282365221	FALSE	TSPAN15
Region_2216	chr10	69571698	69573422	1724	6	42	57.53030303	6.617505217	0.020080582	0.018884139	-	50674	grey	0.058045958	FALSE	NEUROG3
Region_2217	chr10	69572906	69577165	4259	5	27	37.78787879	5.81646245	0.013812454	0.02215959	+	101929021	grey	-0.004571103	FALSE	LOC101929021
Region_2218	chr10	69801880	69964275	162395	30	189	223.8030303	14.37756767	0.839881714	0.03080508	+	1305	grey	-0.084214456	FALSE	COL13A1
Region_2219	chr10	70052544	70112282	59738	32	215	265.9090909	17.2615422	0.856774704	0.022336075	+	55506	grey	-0.050025509	FALSE	MACROH2A2
Region_2220	chr10	70098223	70132934	34711	16	96	124.0909091	11.10471455	0.910173788	0.017764852	-	84883	grey	-0.140848569	FALSE	AIFM2
Region_2221	chr10	70137981	70146700	8719	8	36	60.60606061	10.49963924	0.328288207	0.033007217	-	219743	grey	-0.016825509	FALSE	TYSND1
Region_2222	chr10	70147289	70170523	23234	5	22	37.34848485	6.330615292	0.951905351	0.061490464	-	56681	grey	-0.491625151	FALSE	SAR1A
Region_2223	chr10	70202835	70233911	31076	11	60	77.40909091	8.530447319	0.421920797	0.024465247	-	5464	grey	0.208548873	FALSE	PPA1
Region_2224	chr10	70247329	70284004	36675	14	98	123.9848485	11.0627475	0.733268716	0.031129083	-	64106	grey	0.006204603	FALSE	NPFFR1

Region_2225	chr10	70298970	70382650	83680	37	269	309.4545455	19.14895604	0.800530724	0.0205742	-	55222	grey	0.012207389	FALSE	LRRC20
Region_2226	chr10	70404145	70428618	24473	6	24	36.84848485	6.094745339	0.593488036	0.055582628	+	1979	grey	0.322803632	FALSE	EIF4EBP2
Region_2227	chr10	70431936	70447951	16015	4	27	38.16666667	5.015616638	0.457812025	0.032896337	-	4838	grey	-0.184804141	FALSE	NODAL
Region_2228	chr10	70478767	70668740	189973	87	618	666.6969697	28.55222998	0.791469982	0.012842721	+	27143	grey	-0.367097843	FALSE	PALD1
Region_2229	chr10	70597348	70602759	5411	5	21	37.40909091	6.665588491	0.951355805	0.039086491	-	5551	grey	0.021802949	FALSE	PRF1
Region_2230	chr10	70672506	70762441	89935	36	250	290.5909091	17.85581334	0.873221468	0.029631178	+	140766	grey	0.080138392	FALSE	ADAMTS14
Region_2231	chr10	70815905	70958701	142796	54	346	419.4242424	25.46373627	0.853376849	0.014099195	+	8879	grey	-0.193390985	FALSE	SGPL1
Region_2232	chr10	70882280	70888565	6285	5	21	36.15151515	7.149486101	0.584827345	0.024678417	-	5092	grey	-0.01893896	FALSE	PCBD1
Region_2233	chr10	70918766	70958697	39931	24	148	188.8939394	15.54559427	0.877408415	0.024109181	+	105378349	grey	-0.12495806	FALSE	LINC02622
Region_2234	chr10	70927055	71077810	150755	61	415	503.8333333	30.53451179	0.857262838	0.019354906	+	105378350	grey	-0.163464546	FALSE	LOC105378350
Region_2235	chr10	71212570	71302864	90294	44	285	320.3333333	19.89614059	0.74051971	0.020944543	+	219699	grey	0.082472781	FALSE	UNC5B
Region_2236	chr10	71319259	71381423	62164	31	204	242.2878788	16.84295347	0.835289987	0.026951482	+	55315	grey	-0.140050654	FALSE	SLC29A3
Region_2237	chr1	71396920	71815947	419027	136	961	1049.30303	42.6697405	0.82441064	0.01259844	+	64072	grey	-0.27337293	FALSE	CDH23

	0							7	7	9				1		
Region_2238	chr10	71711701	71737824	26123	9	48	65.1969697	7.708576037	0.867477589	0.038330286	-	414152	grey	-0.17195035	FALSE	C10orf105
Region_2239	chr10	71747556	71773520	25964	4	20	31.39393939	4.755171397	0.553570343	0.05787344	-	64115	grey	-0.261954897	FALSE	VSIR
Region_2240	chr10	71816298	71851251	34953	17	99	135.7121212	12.71312484	0.792680804	0.018230491	-	5660	grey	0.060273555	FALSE	PSAP
Region_2241	chr10	71964395	72013558	49163	19	110	141.4242424	12.32515952	0.721132168	0.027673015	+	9469	grey	0.498955015	FALSE	CHST3
Region_2242	chr10	72059034	72089032	29998	20	89	134.1666667	15.11044807	0.495416798	0.032505491	-	9806	grey	0.169618715	FALSE	SPOCK2
Region_2243	chr10	72096032	72217134	121102	28	208	239.6515152	16.16976516	0.86467102	0.01256329	-	51008	grey	-0.131022507	FALSE	ASCC1
Region_2244	chr10	72216000	72235860	19860	10	51	65.93939394	7.945736714	0.681111884	0.052762746	+	119504	grey	-0.056454839	FALSE	ANAPC16
Region_2245	chr10	72332830	72355149	22319	11	69	89.27272727	10.39903169	0.59875828	0.034197898	-	54788	grey	-0.161059599	FALSE	DNAJB12
Region_2246	chr10	72367340	72626131	258791	43	311	351.469697	17.68025382	0.912899617	0.013679795	-	10367	grey	0.047655333	FALSE	MICU1
Region_2247	chr10	72692143	72887694	195551	39	269	299.969697	16.34622487	0.947647608	0.015876718	+	90550	grey	-0.137603171	FALSE	MCU
Region_2248	chr10	72893584	72933036	39452	5	19	36.46969697	6.346942806	0.966719299	0.023869245	+	170392	grey	0.155527636	FALSE	OIT3
Region_2249	chr10	72934762	72954806	20044	8	52	70.42424242	9.195417933	0.896227305	0.043624536	-	84647	grey	0.135904321	FALSE	PLA2G12B

Region_2250	chr10	73007217	73096974	89757	28	192	222.8333333	14.34582044	0.83817308	0.013378753	-	5033	grey	-0.050753348	FALSE	P4HA1
Region_2251	chr10	73110375	73131828	21453	7	45	60.8030303	7.648468317	0.88980231	0.046648203	+	25961	grey	-0.325643464	FALSE	NUDT13
Region_2252	chr10	73133668	73169055	35387	11	74	90.06060606	8.441434052	0.974023811	0.01502909	-	11319	grey	-0.005523943	FALSE	ECD
Region_2253	chr10	73168119	73244504	76385	14	88	120.4242424	11.65274561	0.904489648	0.032017046	+	317662	grey	0.14897849	FALSE	FAM149B1
Region_2254	chr10	73183362	73245981	62619	13	80	111.5606061	11.21966514	0.899432947	0.034055091	-	105378358	grey	0.177926457	FALSE	LOC105378358
Region_2255	chr10	73247360	73276984	29624	12	83	100.6363636	9.508921097	0.692058794	0.030480805	+	414245	grey	-0.018324595	FALSE	DNAJC9-AS1
Region_2256	chr10	73248843	73252693	3850	3	15	24.21212121	4.645974772	0.64914422	0.032356432	-	51021	grey	0.043637258	FALSE	MRPS16
Region_2257	chr10	73253762	73358864	105102	19	94	129.3636364	12.13721089	0.891577076	0.02467232	-	118491	grey	0.105926585	FALSE	CFAP70
Region_2258	chr10	73375101	73414076	38975	13	65	91.96969697	10.05365559	0.807434024	0.02568659	-	310	grey	0.249506553	FALSE	ANXA7
Region_2259	chr10	73423579	73433561	9982	3	11	20.1969697	3.954431582	0.823248357	0.084842565	-	118490	grey	-0.018463467	FALSE	MSS51
Region_2260	chr10	73436433	73496024	59591	20	143	170.2727273	12.01351221	0.96203271	0.015951791	-	5532	grey	0.076147152	FALSE	PPP3CB
Region_2261	chr10	73495525	73520070	24545	8	31	58.22727273	7.961050287	0.973179875	0.029972547	+	101929145	grey	0.304608612	FALSE	PPP3CB-AS1
Region_2262	chr1	73497538	73626117	128579	45	301	351.378787	23.1986505	0.87373015	0.01889023	-	159195	grey	-0.06704552	FALSE	USP54

	0						9	9	3					9		
Region_2263	chr10	73644881	73663803	18922	9	53	72.22727273	8.829662341	0.906239393	0.044799193	-	79933	grey	0.136708829	FALSE	SYNPO2L
Region_2264	chr10	73653980	73675450	21470	7	30	44.71212121	7.172093679	0.961917342	0.038197	+	105378360	grey	-0.367091463	FALSE	SYNPO2L-AS1
Region_2265	chr10	73674287	73698109	23822	12	53	80.75757576	10.12697936	0.964610956	0.025784105	-	729092	grey	-0.069804007	FALSE	AGAP5
Region_2266	chr10	73674295	73730466	56171	16	72	103.0606061	11.38673829	0.842255809	0.025030866	-	113939925	grey	-0.053150324	FALSE	BMS1P4-AGAP5
Region_2267	chr10	73699151	73730487	31336	4	13	22.3030303	4.693595299	0.475762348	0.051213223	-	729096	grey	-0.058186782	FALSE	BMS1P4
Region_2268	chr10	73730562	73737790	7228	10	46	67.33333333	10.15470082	0.973851237	0.020862835	+	2749	grey	-0.047562107	FALSE	GLUD1P3
Region_2269	chr10	73744372	73772161	27789	12	62	85.06060606	10.3565346	0.757508341	0.038871429	+	9632	grey	-0.025865001	FALSE	SEC24C
Region_2270	chr10	73772276	73780251	7975	5	28	38.83333333	5.905885808	0.58766542	0.024971571	+	170384	grey	0.179897206	FALSE	FUT11
Region_2271	chr10	73785606	73801797	16191	9	41	58.31818182	7.825321996	0.964058183	0.032723277	+	23053	grey	0.139944596	FALSE	ZSWIM8
Region_2272	chr10	73796514	73811798	15284	4	13	27.10606061	5.783607572	0.946723069	0.045298125	-	8509	grey	-0.221445756	FALSE	NDST2
Region_2273	chr10	73812501	73874591	62090	28	189	224.9393939	16.94958932	0.884713493	0.022384623	-	818	grey	-0.343537883	FALSE	CAMK2G
Region_2274	chr10	73909177	73917496	8319	4	13	24.31818182	4.890049837	0.016542044	0.039804442	+	5328	grey	-0.169815533	FALSE	PLAU

Region_2275	chr10	73909969	73922777	12808	9	33	53.8030303	8.201725329	0.512288511	0.040520187	-	414236	grey	-0.037451274	FALSE	C10orf55
Region_2276	chr10	73995193	74121363	126170	43	318	355.3181818	19.67830441	0.762597973	0.019119905	+	7414	grey	-0.073762404	FALSE	VCL
Region_2277	chr10	74120255	74150842	30587	5	30	44.78787879	6.873617683	0.977351982	0.021467926	-	26985	grey	-0.017465415	FALSE	AP3M1
Region_2278	chr10	74151202	74709963	558761	72	513	570.6212121	26.89796398	0.887751137	0.010200411	+	132	grey	0.037880292	FALSE	ADK
Region_2279	chr10	74506081	74530553	24472	3	13	25.10606061	5.455071847	0.962964388	0.041217996	-	102723439	grey	-0.125494807	FALSE	LOC102723439
Region_2280	chr10	75094432	75109221	14789	5	19	34.59090909	6.051006967	0.947027964	0.053433542	-	51207	grey	0.169090275	FALSE	DUSP13B
Region_2281	chr10	75099586	75182123	82537	25	158	191.3484848	17.1208307	0.951399362	0.01668628	+	142891	grey	0.149744928	FALSE	SAMD8
Region_2282	chr10	75210154	75231448	21294	4	16	27.39393939	4.967211841	0.486713863	0.046027198	+	7417	grey	-0.229708222	FALSE	VDAC2
Region_2283	chr10	75233641	75236030	2389	3	12	22.75757576	5.637949999	0.329501321	0.098615881	-	118881	grey	0.089664326	FALSE	COMTD1
Region_2284	chr10	75243568	75373500	129932	29	191	229.030303	17.23857008	0.879970645	0.017512116	+	253264	grey	-0.176475086	FALSE	ZNF503-AS1
Region_2285	chr10	75279725	75401764	122039	38	250	295.9545455	19.61195226	0.782195172	0.019995482	-	84858	grey	-0.086831131	FALSE	ZNF503
Region_2286	chr10	75401519	75408982	7463	21	121	167.9242424	16.35030991	0.182038911	0.032422389	+	100131213	grey	0.03287307	FALSE	ZNF503-AS2
Region_2287	chr1	75431624	76560168	112854	186	1469	1571.53030	46.9012775	0.87687048	0.01522236	+	83938	grey	-0.15386618	FALSE	LRMDA

	0			4			3	6	1	7				9		
Region_2288	chr10	76869601	77638369	768768	136	1103	1194.80303	40.60929022	0.819374333	0.014029363	-	3778	grey	0.117700557	FALSE	KCNMA1
Region_2289	chr10	76888044	76980624	92580	15	121	145.0454545	11.39558864	0.83142344	0.03633847	+	101929328	grey	0.213994164	FALSE	KCNMA1-AS1
Region_2290	chr10	77350851	77376613	25762	3	11	23.98484848	4.969112053	0.919785558	0.063607607	+	101929286	grey	0.264561913	FALSE	KCNMA1-AS3
Region_2291	chr10	77953148	78029522	76374	13	96	121.0606061	10.25502322	0.877403718	0.024090586	-	11128	grey	-0.023097745	FALSE	POLR3A
Region_2292	chr10	78033760	78056813	23053	4	21	30.87878788	4.853278516	0.902936468	0.065409422	+	6229	grey	0.01905892	FALSE	RPS24
Region_2293	chr10	78179174	78542627	363453	43	283	353.5151515	21.33919646	0.783397528	0.025180602	+	105378375	grey	0.231071543	FALSE	LOC105378375
Region_2294	chr10	78248601	78551355	302754	34	231	278.8030303	17.5693962	0.783829323	0.025857336	+	100132987	grey	0.034235644	FALSE	LINC00856
Region_2295	chr10	78267310	78396465	129155	18	118	144.4090909	11.08426088	0.789782362	0.031291031	+	414243	grey	0.2214826	FALSE	LINC00595
Region_2296	chr10	78943328	79067895	124567	26	160	198.2727273	16.26278948	0.543165606	0.028618213	-	283050	grey	0.28733354	FALSE	ZMIZ1-AS1
Region_2297	chr10	79068966	79316519	247553	70	465	544.5	34.21751688	0.647553058	0.022337119	+	57178	grey	-0.029882196	FALSE	ZMIZ1
Region_2298	chr10	79347469	79355334	7865	6	40	49.54545455	5.881344216	0.723634301	0.050819115	+	10105	grey	-0.035644377	FALSE	PPIF
Region_2299	chr10	79382325	79445624	63299	21	134	170.6363636	17.11376986	0.769543489	0.026394935	-	219654	grey	-0.321749637	FALSE	ZCCHC24

Region_2300	chr10	79610939	79615455	4516	3	10	20.84848485	5.274736375	0.91153651	0.077548013	+	653509	grey	-0.161674204	FALSE	SFTPA1
Region_2301	chr10	79661394	79826594	165200	37	167	265.3939394	38.25536817	0.874242959	0.023153837	-	101060691	grey	0.37244438	FALSE	NUTM2B-AS1
Region_2302	chr10	79682997	79685436	2439	6	32	46.33333333	7.815828778	0.933840346	0.040450711	+	650623	grey	0.189108034	FALSE	BEND3P3
Region_2303	chr10	79904898	79951029	46131	10	67	82.10606061	8.714495065	0.952251391	0.036468657	+	100288974	grey	-0.229302232	FALSE	BMS1P21
Region_2304	chr10	79937467	79982614	45147	15	78	111.8181818	11.54777247	0.509920499	0.022820366	-	6441	grey	-0.046021479	FALSE	SFTPD
Region_2305	chr10	80046860	80078912	32052	9	59	81.43939394	9.713002844	0.739975563	0.019615666	-	219347	grey	0.063532568	FALSE	TMEM254-AS1
Region_2306	chr10	80078646	80092557	13911	6	30	50.18181818	9.653869835	0.329391605	0.019529399	+	80195	grey	0.113318619	FALSE	TMEM254
Region_2307	chr10	80131682	80145359	13677	3	14	22.07575758	4.956148027	0.586610144	0.077302414	+	219348	grey	-0.056707294	FALSE	PLAC9
Region_2308	chr10	80150889	80205572	54683	24	154	188.7424242	14.56477374	0.673956402	0.038442318	-	311	grey	0.107438013	FALSE	ANXA11
Region_2309	chr10	80207372	80219657	12285	3	17	25.96969697	4.683751609	0.944742309	0.046230405	+	439990	grey	0.246461366	FALSE	LINC00857
Region_2310	chr10	80407829	80437115	29286	7	54	69.53030303	8.832143562	0.884936427	0.042079429	+	84293	grey	-0.148644183	FALSE	PRXL2A
Region_2311	chr10	80454265	80533124	78859	14	103	129	12.82425586	0.672909093	0.034408214	+	81619	grey	0.005429685	FALSE	TSPAN14
Region_2312	chr1	80529592	80536026	6434	4	17	32.7424242	6.67558261	0.49881016	0.03849535	-	10192957	grey	0.062446682	FALSE	TSPAN14-AS1

	0						4	6	9	7		4				
Region_2313	chr10	80537902	80646560	108658	17	104	131.9848485	11.63217345	0.912357926	0.029286458	+	387694	grey	-0.212669618	FALSE	SH2D4B
Region_2314	chr10	81875194	82987179	1111985	144	1112	1206	44.55074894	0.794564003	0.018705438	+	10718	grey	-0.034814878	FALSE	NRG3
Region_2315	chr10	84194537	84219621	25084	6	43	57.25757576	7.684771367	0.866555248	0.05584972	+	92211	grey	-0.31408134	FALSE	CDHR1
Region_2316	chr10	84230666	84259960	29294	5	20	41.96969697	7.168241275	0.628651563	0.056683421	+	5995	grey	-0.0224	FALSE	RGR
Region_2317	chr10	84267747	84294659	26912	4	23	37.42424242	6.365920336	0.772496217	0.076314526	+	170425	grey	-0.315297409	FALSE	LINC00858
Region_2318	chr10	84328586	84518521	189935	17	87	107.6515152	10.16251396	0.881303434	0.020609027	+	54462	grey	0.029265309	FALSE	CCSER2
Region_2319	chr10	85378327	85432775	54448	7	35	53.92424242	7.768398718	0.816036875	0.064157356	-	105378403	grey	0.107742269	FALSE	LOC105378403
Region_2320	chr10	85577731	85607213	29482	8	35	54.3030303	7.684088849	0.890404686	0.051336016	+	100507470	grey	-0.279181687	FALSE	GRID1-AS1
Region_2321	chr10	85599552	86366795	767243	105	814	892.2575758	36.29846812	0.784810671	0.016088212	-	2894	grey	0.101672521	FALSE	GRID1
Region_2322	chr10	86435256	86521792	86536	16	94	117.4090909	10.91502461	0.788328702	0.024836137	-	23063	grey	0.186397675	FALSE	WAPL
Region_2323	chr10	86654518	86736072	81554	14	93	115.1212121	9.186135291	0.873177974	0.042829133	+	11155	grey	0.096357839	FALSE	LDB3
Region_2324	chr10	86756601	86932825	176224	17	97	124.9848485	11.86914866	0.834156133	0.026007609	+	657	grey	0.087789429	FALSE	BMPR1A

Region_2325	chr10	86935540	86969312	33772	5	23	35.40909091	6.196716929	0.737837366	0.046166181	-	79812	grey	-0.377926704	FALSE	MMRN2
Region_2326	chr10	86944364	86971311	26947	3	11	19.54545455	4.829976328	0.906472316	0.070695581	-	100133190	grey	-0.31394721	FALSE	ADIRF-AS1
Region_2327	chr10	86970741	87024732	53991	9	62	82.10606061	9.941565869	0.848874811	0.025791732	+	119385	grey	-0.161157377	FALSE	AGAP11
Region_2328	chr10	87094161	87191465	97304	14	80	99.65151515	9.31581356	0.827813235	0.023805568	+	54537	grey	0.005782988	FALSE	SHLD2
Region_2329	chr10	87189773	87342389	152616	8	32	52.63636364	9.466761876	0.868470517	0.04020212	-	105378410	grey	0.178778433	FALSE	LOC105378410
Region_2330	chr10	87189779	87342612	152833	8	32	52.63636364	9.466761876	0.868470517	0.04020212	-	728190	grey	0.178778433	FALSE	NUTM2A-AS1
Region_2331	chr10	87396561	87435035	38474	5	28	44.78787879	7.766823232	0.444248493	0.1075352	-	728218	grey	-0.005367773	FALSE	FAM245A
Region_2332	chr10	87504875	87553461	48586	9	58	79.31818182	8.374244565	0.830186226	0.034489134	+	9562	grey	-0.187987355	FALSE	MINPP1
Region_2333	chr10	87659613	87747705	88092	8	51	68.57575758	8.034656866	0.894768138	0.060888424	+	9060	grey	-0.294840333	FALSE	PAPSS2
Region_2334	chr10	87751512	87841361	89849	12	72	99.36363636	9.872473564	0.877863899	0.019678495	-	84896	grey	0.045542154	FALSE	ATAD1
Region_2335	chr10	88273864	88584530	310666	37	261	310.2272727	19.84462373	0.819313632	0.028713278	-	55328	grey	0.262421701	FALSE	RNLS
Region_2336	chr10	88706249	88752776	46527	13	82	102.9848485	9.58282506	0.786632887	0.062988698	+	643414	grey	0.020915049	FALSE	LIPK
Region_2337	chr1	88759982	88779626	19644	3	15	29.3484848	5.38799927	0.96106694	0.04610202	+	643418	grey	0.029073658	FALSE	LIPN

	0						5			8						
Region_2338	chr10	88819896	88851844	31948	7	42	54.53030303	6.447944686	0.749872925	0.047541686	-	118932	grey	0.15417857	FALSE	ANKRD22
Region_2339	chr10	88879734	88975153	95419	16	122	150.3939394	13.00281483	0.688582349	0.019461869	+	57559	grey	-0.090790162	FALSE	STAMBPL1
Region_2340	chr10	88935074	88991339	56265	12	61	95.83333333	11.52066148	0.634024274	0.020023157	-	59	grey	-0.042316476	FALSE	ACTA2
Region_2341	chr10	88953813	89029605	75792	13	78	104.9242424	10.82775722	0.583422299	0.022810437	+	355	grey	0.024540519	FALSE	FAS
Region_2342	chr10	88990045	88994249	4204	5	28	44.89393939	8.723317626	0.003411287	0.007304978	-	100302740	grey	-0.063796074	FALSE	FAS-AS1
Region_2343	chr10	89213569	89414557	200988	19	144	179.1969697	14.02110464	0.820747176	0.017895467	-	3988	grey	-0.156355505	FALSE	LIPA
Region_2344	chr10	89277924	89342674	64750	4	27	36.86363636	6.428030835	0.960013177	0.041708687	-	105378419	grey	0.061936748	FALSE	LOC105378419
Region_2345	chr10	89283694	89309271	25577	3	20	28.83333333	4.878787155	0.953658435	0.052778433	+	3433	grey	0.023427859	FALSE	IFIT2
Region_2346	chr10	89327307	89377473	50166	3	22	30.87878788	5.21393149	0.96517604	0.03626451	+	3437	grey	-0.092279497	FALSE	IFIT3
Region_2347	chr10	89430299	89556641	126342	12	87	105.1212121	8.82394496	0.772570865	0.029058101	-	387700	grey	-0.00933148	FALSE	SLC16A12
Region_2348	chr10	89582988	89645242	62254	11	75	105.7272727	10.19433546	0.855869654	0.03438887	-	53354	grey	-0.190271744	FALSE	PANK1
Region_2349	chr10	89701590	89774939	73349	10	59	79.3030303	8.607634709	0.950222629	0.028069352	+	9585	grey	-0.080503422	FALSE	KIF20B

Region_2350	chr10	89853595	90225443	371848	23	165	196.2878788	16.45952159	0.825851202	0.026900439	+	105378425	grey	-0.236213015	FALSE	LINC01374
Region_2351	chr10	89915489	89957373	41884	4	24	36.27272727	5.871824391	0.881168275	0.054536014	-	101926924	grey	0.093863256	FALSE	LINC01375
Region_2352	chr10	90402521	90628852	226331	12	74	93.74242424	9.632174618	0.867285701	0.047293841	-	101926942	grey	-0.142043371	FALSE	LINC02653
Region_2353	chr10	90740823	90858039	117216	17	126	150.9393939	12.8121082	0.813329403	0.031354839	-	3363	grey	-0.038176809	FALSE	HTR7
Region_2354	chr10	90871952	90908553	36601	5	25	38.77272727	5.753381493	0.960847843	0.029628217	+	10556	grey	-0.18547162	FALSE	RPP30
Region_2355	chr10	90965979	90981532	15553	4	23	35.68181818	5.807578844	0.884527745	0.063623566	+	107984252	grey	0.245566592	FALSE	LOC107984252
Region_2356	chr10	91033157	91062164	29007	3	18	27.21212121	5.127647988	0.924121712	0.063693487	+	100874184	grey	0.046835568	FALSE	LINC00502
Region_2357	chr10	91163012	91284337	121325	12	68	91.27272727	9.39811021	0.643285234	0.031845271	+	84333	grey	0.030583149	FALSE	PCGF5
Region_2358	chr10	91306958	91575243	268285	20	157	189.3636364	10.82253541	0.890863335	0.027339744	-	100188947	grey	0.05624536	FALSE	HECTD2-AS1
Region_2359	chr10	91409280	91514829	105549	4	23	36.90909091	5.411461548	0.853461955	0.07455324	+	143279	grey	0.209363156	FALSE	HECTD2
Region_2360	chr10	91782835	91798309	15474	3	11	21.25757576	4.554999303	0.627905471	0.068615505	-	100507633	grey	0.108757517	FALSE	TNKS2-DT
Region_2361	chr10	91798426	91865475	67049	4	23	33.59090909	5.446176564	0.973116733	0.032830237	+	80351	grey	0.011737906	FALSE	TNKS2
Region_2362	chr1	91923770	92031437	107667	8	43	59.3484848	6.59485212	0.85361550	0.02323528	+	9044	grey	-0.19991750	FALSE	BTAF1

	0						5	8	3	9				7		
Region_2363	chr10	92046692	92291078	244386	26	175	210.8181818	15.47693976	0.817888445	0.022196976	-	22849	grey	0.062231194	FALSE	CPEB3
Region_2364	chr10	92291167	92353964	62797	4	21	33.48484848	5.384428901	0.728039592	0.030567373	+	54708	grey	0.118454427	FALSE	MARCHF5
Region_2365	chr10	92451684	92574096	122412	19	124	151.969697	13.3173914	0.814057542	0.024493275	-	3416	grey	0.010803976	FALSE	IDE
Region_2366	chr10	92574105	92655395	81290	20	129	169.8030303	14.06383217	0.880067052	0.025401899	+	3832	grey	-0.079816517	FALSE	KIF11
Region_2367	chr10	92826831	93059493	232662	19	99	129.7272727	14.33612484	0.84032293	0.028225055	+	54536	grey	0.206329125	FALSE	EXOC6
Region_2368	chr10	93060798	93069540	8742	9	52	76.27272727	8.720524781	0.247170714	0.029845228	+	340665	grey	0.150668378	FALSE	CYP26C1
Region_2369	chr10	93063646	93077885	14239	12	68	104.0151515	11.35780643	0.350694509	0.022797129	+	1592	grey	0.177799109	FALSE	CYP26A1
Region_2370	chr10	93306429	93482334	175905	29	174	207.3181818	14.25715377	0.919154683	0.019705727	-	26509	grey	-0.235690211	FALSE	MYOF
Region_2371	chr10	93496612	93529092	32480	3	14	27.68181818	5.221727067	0.96258838	0.038201902	+	55165	grey	-0.071580905	FALSE	CEP55
Region_2372	chr10	93566665	93604480	37815	13	85	115.6060606	10.57992117	0.592258614	0.026860263	+	338557	grey	0.285783945	FALSE	FFAR4
Region_2373	chr10	93591687	93601744	10057	3	19	28.37878788	4.519241432	0.942667504	0.057206604	-	5950	grey	-0.091939966	FALSE	RBP4
Region_2374	chr10	93612537	93666010	53473	10	58	79.74242424	9.708538044	0.897350317	0.042342048	+	5146	grey	-0.025520521	FALSE	PDE6C

Region_2375	chr10	93757840	93806134	48294	7	27	48.45454545	7.365578609	0.799331622	0.053335895	+	9211	grey	0.01576565	FALSE	LGI1
Region_2376	chr10	93893973	93956062	62089	9	57	71.77272727	7.182875907	0.687739718	0.040092556	+	159371	grey	-0.319997001	FALSE	SLC35G1
Region_2377	chr10	93958191	93961540	3349	3	12	23.33333333	5.338779271	0.914608785	0.064289255	-	266971	grey	-0.140765769	FALSE	PIPSL
Region_2378	chr10	93993931	94332823	338892	68	514	572.8181818	22.56130731	0.844253561	0.019081212	+	51196	grey	0.129015983	FALSE	PLCE1
Region_2379	chr10	94333226	94362959	29733	16	104	133.8484848	12.30039702	0.971306052	0.014752048	-	64318	grey	-0.22997345	FALSE	NOC3L
Region_2380	chr10	94402541	94536332	133791	25	157	183.9242424	12.55181336	0.774061973	0.023302785	+	23232	grey	0.104596378	FALSE	TBC1D12
Region_2381	chr10	94545329	94613905	68576	15	89	113.3181818	10.39698047	0.866386144	0.025602735	+	3070	grey	0.094184426	FALSE	HELLS
Region_2382	chr10	94683729	94736190	52461	4	21	31.43939394	4.60143385	0.903513916	0.054314439	+	1562	grey	-0.265616048	FALSE	CYP2C18
Region_2383	chr10	94688154	94855547	167393	15	94	111.3181818	7.948215263	0.891440098	0.038468665	+	1557	grey	-0.144518305	FALSE	CYP2C19
Region_2384	chr10	94938658	94990091	51433	4	21	31.90909091	5.419983743	0.877438025	0.076198236	+	1559	grey	0.257697502	FALSE	CYP2C9
Region_2385	chr10	95036772	95069497	32725	6	41	52.74242424	6.09239275	0.89335064	0.049465213	-	1558	grey	-0.167565768	FALSE	CYP2C8
Region_2386	chr10	95141925	95168425	26500	6	44	59.24242424	7.012210463	0.914563141	0.048239836	-	107984257	grey	0.232902076	FALSE	LOC107984257
Region_2387	chr1	95194200	95228929	34729	11	58	81.5909090	9.07740605	0.85428331	0.05880519	+	142827	grey	0.187504459	FALSE	ACSM6

	0						9	6	3	5						
Region_2388	chr10	95237572	95291012	53440	8	41	55.89393939	6.621942053	0.733251534	0.039883319	-	9124	grey	-0.130746455	FALSE	PDLIM1
Region_2389	chr10	95311771	95561414	249643	47	363	412.3939394	21.93267937	0.850278402	0.021349911	-	10580	grey	0.119710496	FALSE	SORBS1
Region_2390	chr10	95605941	95656711	50770	9	58	81.03030303	9.306688574	0.773690443	0.028742625	-	5832	grey	0.113302	FALSE	ALDH18A1
Region_2391	chr10	95659823	95694143	34320	7	25	38.95454545	6.410164568	0.835893761	0.03177734	-	26123	grey	-0.033370537	FALSE	TCTN3
Region_2392	chr10	95711779	95877266	165487	17	115	148.3030303	13.71800579	0.839325697	0.021394411	+	953	grey	0.106813748	FALSE	ENTPD1
Region_2393	chr10	95732976	96090250	357274	34	235	287.1515152	15.79994394	0.881087442	0.018731962	-	728558	grey	-0.275153341	FALSE	ENTPD1-AS1
Region_2394	chr10	95860548	96033745	173197	16	97	134.8030303	11.25811802	0.915650956	0.033169494	+	387707	grey	-0.09443029	FALSE	CC2D2B
Region_2395	chr10	96129715	96205288	75573	14	96	118.7121212	9.005579726	0.855880914	0.021562481	+	9849	grey	-0.038679333	FALSE	ZNF518A
Region_2396	chr10	96189171	96271587	82416	11	76	96.66666667	8.73425323	0.937411922	0.028979527	-	29760	grey	-0.070072627	FALSE	BLNK
Region_2397	chr10	96304409	96338564	34155	16	115	142.8939394	11.78673151	0.85845989	0.03138417	+	1791	grey	0.126807535	FALSE	DNTT
Region_2398	chr10	96343221	96359365	16144	3	10	21.63636364	4.812280328	0.974531039	0.037671873	-	93377	grey	0.247310942	FALSE	OPALIN
Region_2399	chr10	96364608	96513926	149318	24	138	176.1060606	14.93692254	0.707161005	0.030506782	-	7093	grey	0.069537826	FALSE	TLL2

Region_2400	chr10	96518110	96587452	69342	11	69	88.22727273	10.30130007	0.604447411	0.023453715	-	56889	grey	-0.131453557	FALSE	TM9SF3
Region_2401	chr10	96593315	96720514	127199	14	90	112.9090909	10.94985391	0.911234596	0.038946909	-	118788	grey	0.207775322	FALSE	PIK3AP1
Region_2402	chr10	96832254	96995956	163702	15	111	129.9393939	11.36780716	0.877093031	0.025778898	+	84458	grey	0.034902359	FALSE	LCOR
Region_2403	chr10	96998038	97185959	187921	47	342	389.1969697	23.41940004	0.821612667	0.01956922	-	6585	grey	-0.523027136	FALSE	SLIT1
Region_2404	chr10	97065513	97102556	37043	7	53	69.04545455	7.870943289	0.828276445	0.049433544	-	105378444	grey	-0.081690123	FALSE	
Region_2405	chr10	97102756	97104355	1599	3	12	29.83333333	7.165265218	0.524079488	0.082698369	+	100505540	grey	-0.084679751	FALSE	SLIT1-AS1
Region_2406	chr10	97153046	97292637	139591	32	198	240.4242424	16.38109662	0.850430915	0.023517691	-	100533184	grey	-0.2843723	FALSE	ARHGAP19-SLIT1
Region_2407	chr10	97195907	97203721	7814	8	36	58.27272727	9.376805188	0.706049769	0.041073772	+	112268065	grey	-0.058240446	FALSE	LOC112268065
Region_2408	chr10	97222173	97292673	70500	12	67	87.68181818	10.61951627	0.897944545	0.020522458	-	84986	grey	0.06263307	FALSE	ARHGAP19
Region_2409	chr10	97319271	97321915	2644	21	135	176.7272727	21.34229852	0.009393874	0.009927677	+	10023	grey	0.149989	FALSE	FRAT1
Region_2410	chr10	97332497	97334729	2232	4	16	30.03030303	5.989656975	0.007325254	0.013764746	-	23401	grey	0.088598885	FALSE	FRAT2
Region_2411	chr10	97334564	97343204	8640	5	20	32.71212121	6.314245558	0.263359885	0.074530358	+	105378448	grey	0.139068407	FALSE	LOC105378448
Region_2412	chr1	97356358	97426076	69718	22	124	156.939393	14.1735167	0.78359339	0.02971214	-	23223	grey	0.008116889	FALSE	RRP12

	0						9	9	6	7						
Region_2413	chr1 0	97401134	97426074	24940	12	68	89.6060606 1	10.0626939 2	0.66672485	0.0361095	-	644215	grey	-0.23963625 8	FALSE	LOC644215
Region_2414	chr1 0	97435909	97446017	10108	4	27	40.3181818 2	6.37098975 7	0.49236579 7	0.01596153 4	-	51013	grey	-0.09095713	FALSE	EXOSC1
Region_2415	chr1 0	97458324	97498794	40470	8	50	64.7878787 9	6.46056713 8	0.83517592 3	0.02925834 9	-	64210	grey	0.234467649	FALSE	MMS19
Region_2416	chr1 0	97498924	97571206	72282	21	104	147.984848 5	14.2164503	0.85884885 1	0.02381944 9	+	80019	grey	0.106870484	FALSE	UBTD1
Region_2417	chr1 0	97584323	97612802	28479	6	27	40.1969697	6.64765938 4	0.95106095 9	0.03547220 4	+	112817	grey	-0.06142628 7	FALSE	HOGA1
Region_2418	chr1 0	97584374	97676434	92060	17	94	116.121212 1	10.7995942 3	0.89433180 9	0.01933208 5	+	55361	grey	-0.01439992 9	FALSE	PI4K2A
Region_2419	chr1 0	97614553	97633500	18947	6	25	42.6212121 2	6.37486688	0.96779204 5	0.03180649 1	-	118812	grey	0.121586177	FALSE	MORN4
Region_2420	chr1 0	97737121	97760907	23786	4	19	32.2727272 7	5.50117597 1	0.97480724 2	0.03093732 9	+	118813	grey	-0.2119369	FALSE	ZFYVE27
Region_2421	chr1 0	97828478	97849798	21320	4	16	30.1363636 4	5.78562240 2	0.66050577 6	0.05701862 1	-	10050556 1	grey	-0.05446775 7	FALSE	GOLGA7B-DT
Region_2422	chr1 0	97865000	98030828	165828	31	206	267.257575 8	19.2763790 1	0.76247552 7	0.02605264	-	55118	grey	-0.11670829 4	FALSE	CRTAC1
Region_2423	chr1 0	98134624	98244897	110273	15	121	151.257575 8	12.8318241 5	0.89109828 4	0.01617043 1	+	27291	grey	-0.01752021 7	FALSE	R3HCC1L
Region_2424	chr1 0	98247690	98268194	20504	13	68	88.2121212 1	11.3157686 5	0.69171264 6	0.05199152 5	-	84171	grey	-0.09729541 5	FALSE	LOXL4

Region_2425	chr10	98383565	98415182	31617	10	49	79.46969697	10.80059784	0.920787179	0.034478933	-	84795	grey	-0.104064092	FALSE	PYROXD2
Region_2426	chr10	98410939	98446963	36024	12	53	85.07575758	8.374522914	0.804814923	0.018906169	-	3257	grey	-0.128587099	FALSE	HPS1
Region_2427	chr10	98457077	99235862	778785	139	1032	1117.318182	39.01997874	0.838908534	0.016813719	-	60495	grey	0.068231201	FALSE	HPSE2
Region_2428	chr10	99329356	99394330	64974	31	191	261.6969697	22.9768912	0.528843653	0.012110814	+	26507	grey	0.187753108	FALSE	CNNM1
Region_2429	chr10	99396870	99430624	33754	8	55	73.21212121	9.219569741	0.963813821	0.021671364	-	2805	grey	0.211365901	FALSE	GOT1
Region_2430	chr10	99430917	99463038	32121	5	26	38.31818182	6.212045596	0.969660525	0.026889467	+	101927300	grey	0.030963406	FALSE	GOT1-DT
Region_2431	chr10	99526350	99531177	4827	12	71	99	11.97176165	0.172667328	0.05550069	-	101927324	grey	-0.099769868	FALSE	LINC01475
Region_2432	chr10	99532942	99536524	3582	4	22	38.60606061	7.643758214	0.014544831	0.024036952	+	159296	grey	-0.028260753	FALSE	NKX2-3
Region_2433	chr10	99610519	99659284	48765	10	72	89.95454545	8.919178676	0.667445211	0.023857664	-	81894	grey	0.105391717	FALSE	SLC25A28
Region_2434	chr10	99651595	99732127	80532	15	78	99.43939394	11.41406526	0.624250715	0.031821946	-	1355	grey	-0.072513056	FALSE	COX15
Region_2435	chr10	99659509	99711241	51732	12	58	72.78787879	8.060378215	0.699851214	0.040094632	+	57089	grey	-0.078893402	FALSE	ENTPD7
Region_2436	chr10	99702558	99756134	53576	9	52	66.95454545	7.19279943	0.757436571	0.021523843	+	51076	grey	0.061596824	FALSE	CUTC
Region_2437	chr1	99782640	99852594	69954	13	91	108.545454	9.65430445	0.90612078	0.02049955	+	1244	grey	0.172086872	FALSE	ABCC2

	0						5			7						
Region_2438	chr10	99875577	100009947	134370	20	127	156.7272727	12.58881733	0.945745709	0.023614755	-	23268	grey	-0.307721812	FALSE	DNMBP
Region_2439	chr10	100042193	100081869	39676	13	90	116.4848485	10.35856008	0.959468636	0.022034283	-	1369	grey	-0.081220845	FALSE	CPN1
Region_2440	chr10	100150094	100186033	35939	10	77	96.42424242	8.687852892	0.553158389	0.035352091	-	10613	grey	-0.104911102	FALSE	ERLIN1
Region_2441	chr10	100188300	100229596	41296	4	20	32.07575758	5.635903057	0.840066273	0.08680859	-	1147	grey	0.558038583	FALSE	CHUK
Region_2442	chr10	100232298	100267680	35382	10	59	78.27272727	9.740550379	0.882359116	0.017069948	-	55280	grey	-0.213722744	FALSE	CWF19L1
Region_2443	chr10	100288149	100330264	42115	7	31	47.95454545	6.740079475	0.934015715	0.044570815	-	9033	grey	-0.003998635	FALSE	PKD2L1
Region_2444	chr10	100373099	100454043	80944	11	60	82.16666667	9.47479788	0.866150481	0.030686266	+	90271	grey	-0.120697124	FALSE	OLMALINC
Region_2445	chr10	100463009	100483744	20735	4	19	31.09090909	6.451540717	0.831795821	0.061629435	+	7479	grey	0.020231755	FALSE	WNT8B
Region_2446	chr10	100486646	100529871	43225	4	19	32.90909091	6.146236554	0.771132195	0.042032002	-	25956	grey	0.292980303	FALSE	SEC31B
Region_2447	chr10	100529072	100559998	30926	8	30	46.89393939	6.464408564	0.586097235	0.043997374	+	55662	grey	-0.067186122	FALSE	HIF1AN
Region_2448	chr10	100735396	100829944	94548	16	81	110.3181818	11.13851128	0.240701252	0.024721795	+	5076	grey	0.080211572	FALSE	PAX2
Region_2449	chr10	100912963	100965134	52171	16	86	122.6363636	14.79151146	0.946679267	0.023634853	+	55719	grey	0.134955048	FALSE	SLF2

Region_2450	chr10	100969458	100987515	18057	5	17	31.63636364	5.960475881	0.541858831	0.05484956	-	84545	grey	-0.049541688	FALSE	MRPL43
Region_2451	chr10	100969504	100985871	16367	5	17	31.63636364	5.960475881	0.541858831	0.05484956	+	57715	grey	-0.049541688	FALSE	SEMA4G
Region_2452	chr10	100987367	100994403	7036	7	34	56.27272727	7.839830168	0.944625919	0.029379228	+	56652	grey	0.008693199	FALSE	TWNK
Region_2453	chr10	100996618	101007836	11218	7	25	44.81818182	7.464601779	0.328107841	0.036480825	+	84445	grey	0.330436738	FALSE	LZTS2
Region_2454	chr10	101007679	101032295	24616	5	20	29.31818182	5.469240113	0.739797246	0.059919644	-	79955	grey	0.17626944	FALSE	PDZD7
Region_2455	chr10	101061989	101068131	6142	4	17	30.1969697	4.989896552	0.065242827	0.048003576	+	81621	grey	-0.024132103	FALSE	KAZALD1
Region_2456	chr10	101089321	101131126	41805	9	67	82.54545455	8.421916132	0.807375149	0.044166391	-	100038246	grey	0.355394856	FALSE	TLX1NB
Region_2457	chr10	101131300	101137789	6489	7	39	55.09090909	6.667319315	0.106581385	0.054804723	+	3195	grey	0.269024334	FALSE	TLX1
Region_2458	chr10	101176306	101194147	17841	7	39	52.96969697	6.961365244	0.941601652	0.028996861	+	101927396	grey	0.063352879	FALSE	LINC01514
Region_2459	chr10	101229577	101240838	11261	4	19	30.48484848	5.418607327	0.200667343	0.070470048	+	399806	grey	0.306827946	FALSE	LBX1-AS1
Region_2460	chr10	101354033	101557321	203288	34	246	285.4242424	17.64091451	0.83045457	0.018619381	+	8945	grey	-0.111200995	FALSE	BTRC
Region_2461	chr10	101570560	101609662	39102	26	170	197.7272727	15.39484974	0.866856218	0.021282127	+	25911	grey	0.220091468	FALSE	DPCD
Region_2462	chr1	10157888	10158827	9388	7	30	50.4696969	7.74640303	0.95357530	0.03089762	-	27343	grey	-0.10932718	FALSE	POLL

	0	2	0				7		9					7		
Region_2463	chr1 0	10161066 4	10169529 5	84631	23	125	148.651515 2	11.8405982 5	0.80493783 7	0.01825099	-	6468	grey	0.015619968	FALSE	FBXW4
Region_2464	chr1 0	10170199 4	10173003 7	28043	7	41	51.4545454 5	6.10971941 5	0.83955860 9	0.03954873 7	-	10537845 6	grey	0.123529428	FALSE	LOC105378456
Region_2465	chr1 0	10177010 9	10178037 1	10262	3	14	25.6515151 5	6.24005035 5	0.54934060 7	0.07123961 3	-	2253	grey	-0.39505334 4	FALSE	FGF8
Region_2466	chr1 0	10178444 3	10181846 5	34022	14	109	131.530303	10.3421760 8	0.85683108 9	0.02702045	-	10724	grey	-0.19454651 3	FALSE	OGA
Region_2467	chr1 0	10181877 3	10182888 5	10112	4	32	45.1666666 7	7.20807951 2	0.97164277 8	0.02334919 8	+	10028950 9	grey	0.102734374	FALSE	KCNIP2-AS1
Region_2468	chr1 0	10182597 4	10184392 0	17946	9	40	67.0454545 5	9.87059630 1	0.48836596 9	0.04293317 1	-	30819	grey	0.308745764	FALSE	KCNIP2
Region_2469	chr1 0	10184559 9	10205619 3	210594	53	367	419.378787 9	18.9263878 9	0.90616511 7	0.01316534 5	-	79591	grey	0.338377804	FALSE	ARMH3
Region_2470	chr1 0	10210648 9	10212036 8	13879	7	22	34.8484848 5	5.63025460 3	0.41629475	0.03598572 6	-	8861	grey	0.088908416	FALSE	LDB1
Region_2471	chr1 0	10213299 4	10215033 3	17339	16	74	105.621212 1	13.4104037 1	0.35687483 8	0.01571847	+	23082	grey	-0.24190066 9	FALSE	PPRC1
Region_2472	chr1 0	10215217 6	10216387 1	11695	7	50	68.6969697	7.30009419 7	0.62381118 8	0.04047194 6	+	9221	grey	-0.09442209 7	FALSE	NOLC1
Region_2473	chr1 0	10223018 6	10224151 2	11326	9	37	57.0909090 9	8.66243396 7	0.16723982 7	0.02957851 7	-	5309	grey	0.151210642	FALSE	PITX3
Region_2474	chr1 0	10224537 1	10238289 9	137528	34	228	261.742424 2	17.2807679 7	0.93445553 9	0.01494808 3	+	8729	grey	-0.18243335 8	FALSE	GBF1

Region_2475	chr10	102394110	102402524	8414	9	52	70	9.341388465	0.415417805	0.025755997	+	4791	grey	-0.169295063	FALSE	NFKB2
Region_2476	chr10	102402617	102421539	18922	26	124	159.5757576	14.65926917	0.339008387	0.022137308	-	5662	grey	-0.132549854	FALSE	PSD
Region_2477	chr10	102419189	102423136	3947	10	47	73.37878788	10.69545576	0.008085618	0.01036314	+	79176	grey	-0.090639855	FALSE	FBXL15
Region_2478	chr10	102423245	102432584	9339	5	26	40.87878788	6.559819771	0.959475818	0.036610806	-	79004	grey	0.24809616	FALSE	CUEDC2
Region_2479	chr10	102449816	102461106	11290	9	58	84.45454545	10.46632963	0.50069339	0.026198403	+	100505761	grey	0.026664641	FALSE	C10orf95-AS1
Region_2480	chr10	102449837	102451543	1706	4	19	38.16666667	8.418995616	0.002310594	0.006926548	-	79946	grey	-0.082214137	FALSE	C10orf95
Region_2481	chr10	102461395	102477045	15650	15	89	111.469697	10.86592372	0.771588845	0.04366819	+	79847	grey	-0.134542499	FALSE	MFSD13A
Region_2482	chr10	102461881	102502712	40831	40	252	291.2575758	17.18703501	0.78497498	0.021894522	-	10121	grey	-0.120408382	FALSE	ACTR1A
Region_2483	chr10	102503972	102633535	129563	71	471	538.1818182	27.48365058	0.831998231	0.012041891	+	51684	grey	0.117148673	FALSE	SUFU
Region_2484	chr10	102644479	102658318	13839	7	36	52.06060606	7.083846821	0.202015712	0.036285663	+	81603	grey	0.033376237	FALSE	TRIM8
Region_2485	chr10	102673731	102714397	40666	12	68	87.22727273	8.513871856	0.893962832	0.021539752	-	403	grey	0.208967907	FALSE	ARL3
Region_2486	chr10	102714538	102743492	28954	17	106	132.5151515	13.43959984	0.58256682	0.031016532	+	118980	grey	0.116247664	FALSE	SFXN2
Region_2487	chr1	10274394	10283384	89900	67	473	519.378787	26.5977241	0.86095388	0.01184640	+	54838	grey	-0.07571418	FALSE	WBP1L

	0	8	8				9	1	7	4				2		
Region_2488	chr10	102830531	102837472	6941	4	19	32.48484848	5.384428901	0.910123091	0.057512816	-	1586	grey	0.052184396	FALSE	CYP17A1
Region_2489	chr10	102854272	102901899	47627	20	130	159.5606061	11.29210587	0.909329857	0.016725354	+	100528007	grey	0.161731482	FALSE	BORCS7-ASMT
Region_2490	chr10	102869470	102901899	32429	18	112	137.4848485	10.15759273	0.957235985	0.018688543	+	57412	grey	0.162912469	FALSE	AS3MT
Region_2491	chr10	102898649	102918335	19686	11	79	98.84848485	8.963588609	0.63452482	0.027934517	-	107984265	grey	-0.057483708	FALSE	LOC107984265
Region_2492	chr10	102918294	103090222	171928	78	590	646.8787879	28.3312902	0.890813315	0.011648599	+	54805	grey	-0.229250365	FALSE	CNNM2
Region_2493	chr10	103087185	103277605	190420	41	269	332.8181818	22.52582046	0.870496603	0.017645834	-	22978	grey	0.08301519	FALSE	NT5C2
Region_2494	chr10	103277138	103290346	13208	9	57	82.12121212	12.67767893	0.178896356	0.041798626	+	9118	grey	-0.010606534	FALSE	INA
Region_2495	chr10	103302796	103351144	48348	32	203	240.6060606	16.73768552	0.940796478	0.014844207	-	84108	grey	-0.00983471	FALSE	PCGF6
Region_2496	chr10	103367966	103389065	21099	7	39	55.65151515	7.705128011	0.809440291	0.045431091	+	6877	grey	0.133390494	FALSE	TAF5
Region_2497	chr10	103389041	103396492	7451	3	13	24.43939394	5.063075344	0.649454546	0.028048382	-	84833	grey	-0.21044212	FALSE	ATP5MK
Region_2498	chr10	103396626	103446294	49668	11	69	100.5757576	12.0141719	0.905812353	0.033302938	+	22984	grey	0.193229971	FALSE	PDCD11
Region_2499	chr10	103472804	103479240	6436	3	17	26.45454545	4.739950724	0.954258144	0.056549907	-	119395	grey	-0.237956564	FALSE	CALHM3

Region_2500	chr10	103479603	103517393	37790	13	76	92.54545455	10.1271635	0.324041091	0.025814642	-	102724341	grey	0.373018294	FALSE	NEURL1-AS1
Region_2501	chr10	103493705	103592546	98841	48	297	349.7272727	25.19129609	0.739707602	0.021857527	+	9148	grey	0.018991632	FALSE	NEURL1
Region_2502	chr10	103594027	103855584	261557	81	542	620	29.25116697	0.805532586	0.015361981	-	9644	grey	-0.052932518	FALSE	SH3PXD2A
Region_2503	chr10	103856805	103967040	110235	33	247	285.2878788	15.87331108	0.74272642	0.024887008	-	79991	grey	-0.051641629	FALSE	STN1
Region_2504	chr10	103938324	103967047	28723	11	66	92.18181818	10.68990766	0.68922298	0.029103991	-	102724351	grey	0.213995515	FALSE	LOC102724351
Region_2505	chr10	103967140	104029233	62093	19	115	153.0757576	12.35290138	0.769233549	0.024478935	+	9748	grey	0.45858408	FALSE	SLK
Region_2506	chr10	104031286	104085880	54594	7	43	56.18181818	7.474899723	0.859136059	0.048716795	-	1308	grey	-0.039019716	FALSE	COL17A1
Region_2507	chr10	104129888	104232364	102476	36	279	321.3636364	17.08587934	0.864684286	0.023216233	-	80217	grey	-0.134813889	FALSE	CFAP43
Region_2508	chr10	104235356	104267459	32103	16	81	110.4848485	11.16897134	0.592888501	0.027381189	+	9446	grey	-0.192753681	FALSE	GSTO1
Region_2509	chr10	104268873	104304950	36077	8	50	61.68181818	6.414308742	0.612068477	0.019948895	+	119391	grey	-0.036397978	FALSE	GSTO2
Region_2510	chr10	104309698	104338465	28767	24	140	175.4090909	15.10372065	0.534296879	0.027628793	-	85450	grey	0.092201462	FALSE	ITPRIP
Region_2511	chr10	104353833	104455102	101269	40	305	357.2727273	19.94495923	0.879659441	0.024206015	+	159686	grey	0.041973857	FALSE	CFAP58
Region_2512	chr1	10456693	10462479	57864	7	38	48.3181818	6.22688733	0.84995053	0.05776028	+	10537846	grey	-0.11716731	FALSE	LOC105378464

	0	1	5				2	4		8		4		8		
Region_2513	chr10	104641290	105265242	623952	109	887	966.0606061	35.32132883	0.800853802	0.022716695	+	22986	grey	0.180421175	FALSE	SORCS3
Region_2514	chr10	106573663	107164706	591043	71	496	551.469697	22.3787122	0.875776298	0.017609073	-	114815	grey	-0.061494642	FALSE	SORCS1
Region_2515	chr10	107694973	108197849	502876	34	243	283.6363636	18.71127703	0.862744371	0.0222122	-	103695365	grey	0.147034236	FALSE	LINC01435
Region_2516	chr10	108708539	108840686	132147	11	78	96.95454545	8.999883449	0.804340182	0.053192575	+	105378478	grey	0.278479364	FALSE	LINC02661
Region_2517	chr10	109864766	109923553	58787	6	38	50.43939394	6.972757512	0.967896778	0.024962709	-	7511	grey	-0.067301071	FALSE	XPNPEP1
Region_2518	chr10	109940104	110008381	68277	7	41	56.63636364	6.920729983	0.839659108	0.035432711	-	100505933	grey	0.090936132	FALSE	ADD3-AS1
Region_2519	chr10	109996368	110135565	139197	22	153	178.8333333	13.61852057	0.85668044	0.018452318	+	120	grey	0.010196636	FALSE	ADD3
Region_2520	chr10	110207605	110287365	79760	15	71	103.5757576	13.65065296	0.33464083	0.016065012	+	4601	grey	-0.276098462	FALSE	MXI1
Region_2521	chr10	110428840	110496187	67347	11	73	94.51515152	10.05254262	0.857217041	0.026518625	-	105378482	grey	0.058610947	FALSE	LOC105378482
Region_2522	chr10	110472688	110496590	23902	6	31	47.86363636	7.385820851	0.781887228	0.041520128	-	105378481	grey	0.120074282	FALSE	DUSP5-DT
Region_2523	chr10	110497907	110511533	13626	4	23	37.43939394	6.046305389	0.648268803	0.065905576	+	1847	grey	-0.216746411	FALSE	DUSP5
Region_2524	chr10	110567684	110606048	38364	4	14	28.04545455	5.550971691	0.764245382	0.097382578	+	9126	grey	0.236381326	FALSE	SMC3

Region_2525	chr10	110644336	110839468	195132	24	157	195.1666667	13.23466699	0.774234502	0.021934706	+	282996	grey	0.422036042	FALSE	RBM20
Region_2526	chr10	110898730	110919201	20471	4	23	38.74242424	5.520063004	0.475063158	0.035995456	-	92482	grey	0.014239293	FALSE	BBIP1
Region_2527	chr10	110919367	111017307	97940	5	24	43.18181818	6.63999326	0.841935862	0.072254187	+	8036	grey	-0.526798221	FALSE	SHOC2
Region_2528	chr10	111787233	111989909	202676	23	161	192.1666667	14.35118149	0.847311571	0.027455137	-	105378486	grey	0.019439832	FALSE	
Region_2529	chr10	112149865	112215377	65512	7	48	63.34848485	6.860124296	0.925573674	0.045159729	-	57678	grey	-0.118851743	FALSE	GPAM
Region_2530	chr10	112283400	112305038	21638	5	31	46.21212121	7.43280701	0.926465746	0.052194671	+	6975	grey	-0.258541447	FALSE	TECTB
Region_2531	chr10	112308181	112356574	48393	13	83	100.1666667	8.984744906	0.910487926	0.035512727	-	390003	grey	0.292338754	FALSE	GUCY2GP
Region_2532	chr10	112374116	112428379	54263	5	31	41.07575758	4.759801567	0.980368697	0.024018205	+	51703	grey	-0.332774089	FALSE	ACSL5
Region_2533	chr10	112424428	112447572	23144	8	47	64.21212121	7.562081829	0.677055638	0.04271143	-	64429	grey	0.109448019	FALSE	ZDHHC6
Region_2534	chr10	112446998	112818744	371746	57	432	479.6515152	21.1212561	0.905953167	0.011950196	+	143187	grey	-0.028610763	FALSE	VTI1A
Region_2535	chr10	112888735	112906111	17376	3	22	33.92424242	5.1569538	0.931515401	0.047528408	+	107984158	grey	-0.040072632	FALSE	LINC02935
Region_2536	chr10	112950247	113167678	217431	26	174	212.5606061	15.71195408	0.875739952	0.025426388	+	6934	grey	0.23320784	FALSE	TCF7L2
Region_2537	chr1	11355083	11358960	38765	8	45	66.4696969	8.04261667	0.85509570	0.05440099	+	3026	grey	0.065694128	FALSE	HABP2

	0	7	2				7	5	2	7						
Region_2538	chr10	113588714	113664070	75356	13	97	121.6060606	11.54373461	0.917705209	0.027046744	-	4892	grey	-0.087423152	FALSE	NRAP
Region_2539	chr10	113679162	113730907	51745	6	36	46.59090909	5.740483155	0.913998386	0.044919044	+	840	grey	-0.170380919	FALSE	CASP7
Region_2540	chr10	113751262	113783429	32167	4	24	36.59090909	5.767221095	0.94641093	0.03859104	+	79949	grey	-0.147369849	FALSE	PLEKHS1
Region_2541	chr10	113834725	113854383	19658	6	27	44.89393939	5.984926676	0.589774225	0.073807811	-	9937	grey	-0.186603146	FALSE	DCLRE1A
Region_2542	chr10	113844406	113854473	10067	4	15	26.62121212	4.620240259	0.809691981	0.084666868	+	107984271	grey	0.002560178	FALSE	
Region_2543	chr10	113854661	113917194	62533	7	45	70.62121212	8.351444189	0.804270295	0.025206225	+	374354	grey	-0.071314206	FALSE	NHLRC2
Region_2544	chr10	114120862	114174232	53370	6	27	39.87878788	7.284367142	0.802907425	0.038844903	-	55088	grey	0.076826275	FALSE	CCDC186
Region_2545	chr10	114179270	114232666	53396	11	80	99.43939394	8.932445247	0.945309077	0.028939378	+	56165	grey	-0.062119013	FALSE	TDRD1
Region_2546	chr10	114239254	114294489	55235	14	95	121.6212121	11.0045657	0.851123706	0.023613761	+	340706	grey	-0.160986869	FALSE	VWA2
Region_2547	chr10	114294824	114404756	109932	26	178	211.7727273	14.5832311	0.761092424	0.026503507	-	84632	grey	-0.216305214	FALSE	AFAP1L2
Region_2548	chr10	114431112	114768061	336949	75	577	619.469697	25.84463622	0.872446834	0.011274784	-	3983	grey	-0.018730267	FALSE	ABLIM1
Region_2549	chr10	114764788	114794344	29556	6	29	45.68181818	9.143571245	0.569124492	0.065237615	+	101927692	grey	-0.095586001	FALSE	LOC101927692

Region_2550	chr10	114821744	114899832	78088	21	136	162.2121212	11.77678963	0.750484885	0.028752742	+	57700	grey	0.144898367	FALSE	FHIP2A
Region_2551	chr10	114938195	114977676	39481	5	35	54.8030303	7.192345711	0.950853512	0.030322149	+	142940	grey	-0.025473299	FALSE	TRUB1
Region_2552	chr10	115093365	115948999	855634	93	703	767.5151515	31.14642462	0.808991976	0.018920756	+	26033	grey	-0.143969052	FALSE	ATRNL1
Region_2553	chr10	116056925	116276803	219878	72	577	639.2424242	31.10629944	0.747048138	0.024778205	-	2674	grey	0.47201651	FALSE	GFRA1
Region_2554	chr10	116324448	116380029	55581	9	68	86.16666667	9.705800539	0.904889732	0.034393135	+	374355	grey	0.105694507	FALSE	CCDC172
Region_2555	chr10	116427847	116477957	50110	9	52	74.33333333	9.1219656	0.809964596	0.048106759	+	119548	grey	0.305532119	FALSE	PNLIPRP3
Region_2556	chr10	116545931	116567855	21924	8	38	54.54545455	8.732224702	0.945944304	0.033001926	+	5406	grey	-0.185118298	FALSE	PNLIP
Region_2557	chr10	116590385	116609175	18790	6	30	40.16666667	6.306488315	0.906972086	0.058367422	+	5407	grey	-0.200041337	FALSE	PNLIPRP1
Region_2558	chr10	116620953	116645143	24190	5	25	41.1969697	6.638395768	0.727643531	0.057690538	+	5408	grey	-0.207916015	FALSE	PNLIPRP2
Region_2559	chr10	116671192	116850251	179059	42	295	342.4848485	21.57437399	0.813991876	0.018929309	-	259217	grey	0.023302507	FALSE	HSPA12A
Region_2560	chr10	116849499	116911788	62289	14	86	114.1969697	12.08841747	0.934681396	0.022857974	+	387712	grey	0.124619269	FALSE	ENO4
Region_2561	chr10	116881477	117126586	245109	27	169	199.4545455	14.10638897	0.780009213	0.026643261	-	57698	grey	-0.179340917	FALSE	SHTN1
Region_2562	chr1	11714456	11716910	24544	10	65	82.3181818	8.03675995	0.26049812	0.04236845	-	10192770	grey	-0.05123092	FALSE	MIR3663HG

	0	4	8				2		5	4		4		9		
Region_2563	chr10	117241093	117279430	38337	6	35	47.93939394	5.948183402	0.596180179	0.047165278	+	6571	grey	0.085904965	FALSE	SLC18A2
Region_2564	chr10	117277274	117375440	98166	8	44	60.15151515	8.416544923	0.692312066	0.034512421	-	118987	grey	-0.042417049	FALSE	PDZD8
Region_2565	chr10	117425194	117490419	65225	5	23	39.37878788	6.362788821	0.654239528	0.084227432	+	105378502	grey	0.184194624	FALSE	LOC105378502
Region_2566	chr10	117473215	117545068	71853	16	102	119.8787879	10.88473193	0.287605433	0.024316878	-	196047	grey	-0.225189831	FALSE	EMX2OS
Region_2567	chr10	117542445	117549546	7101	6	31	44.87878788	5.980777288	0.037547824	0.026403294	+	2018	grey	-0.159962932	FALSE	EMX2
Region_2568	chr10	118004916	118046941	42025	13	90	125.6363636	12.88247034	0.836171945	0.026144435	-	22841	grey	-0.157180727	FALSE	RAB11FIP2
Region_2569	chr10	118046279	118210158	163879	19	154	183.3939394	14.03827601	0.829177341	0.024019609	+	255082	grey	-0.239385172	FALSE	CASC2
Region_2570	chr10	118241468	118267710	26242	5	27	43.81818182	7.560848733	0.865814245	0.047786323	+	101927760	grey	-0.149521891	FALSE	LINC02944
Region_2571	chr10	118297925	118342328	44403	5	18	33.48484848	6.192408316	0.775332214	0.023859642	-	63877	grey	0.341152786	FALSE	FAM204A
Region_2572	chr10	118341437	118398468	57031	16	108	137	10.77889676	0.662087127	0.047849166	+	100506126	grey	-0.549691801	FALSE	LINC00867
Region_2573	chr10	118589997	118595648	5651	5	37	58.8030303	9.612843487	0.356471309	0.07014347	-	2834	grey	-0.107297229	FALSE	PRLHR
Region_2574	chr10	118674167	118755249	81082	19	127	155.3939394	12.98860896	0.833383116	0.019884096	-	143384	grey	-0.0692643	FALSE	CACUL1

Region_2575	chr10	119033670	119080823	47153	6	28	45.48484848	6.351843886	0.948213486	0.039898476	-	8661	grey	0.166637397	FALSE	EIF3A
Region_2576	chr10	119104086	119137984	33898	13	59	79.65151515	9.833212832	0.589143809	0.050271149	+	404636	grey	-0.141998148	FALSE	DENND10
Region_2577	chr10	119140767	119165714	24947	8	50	71.57575758	9.77762061	0.915018581	0.044201802	-	119559	grey	-0.367828817	FALSE	SFXN4
Region_2578	chr10	119207571	119459745	252174	95	645	722.6212121	33.10764037	0.853116892	0.013819832	+	2869	grey	0.33319801	FALSE	GRK5
Region_2579	chr10	119330233	119336182	5949	3	19	30	4.644268594	0.943872739	0.039700082	-	105378511	grey	0.462075748	FALSE	LOC105378511
Region_2580	chr10	119499817	119542719	42902	9	45	65.92424242	8.595724529	0.79401267	0.03811122	-	6001	grey	-0.13260095	FALSE	RGS10
Region_2581	chr10	119651380	119677819	26439	4	26	36.59090909	5.801796201	0.794389088	0.101889816	+	9531	grey	-0.022646299	FALSE	BAG3
Region_2582	chr10	119726042	119829147	103105	18	119	143.3333333	12.35043859	0.881694526	0.023420377	+	22876	grey	-0.019943769	FALSE	INPP5F
Region_2583	chr10	119793589	119815958	22369	7	31	51.51515152	7.594010645	0.918425614	0.038032646	-	105378513	grey	0.239582	FALSE	LOC105378513
Region_2584	chr10	119829404	119892556	63152	12	85	100.8181818	8.176719468	0.666376624	0.035278979	-	79892	grey	0.181904134	FALSE	MCMBP
Region_2585	chr10	119892730	119944657	51927	8	46	66.5	8.882567196	0.854074476	0.02196049	+	11196	grey	0.164473303	FALSE	SEC23IP
Region_2586	chr10	120457227	120592065	134838	29	209	253.969697	17.38785579	0.75830602	0.022381488	+	196051	grey	-0.257964721	FALSE	PLPP4
Region_2587	chr1	12060858	12082531	216737	32	241	276.848484	18.4467987	0.84229955	0.02648616	+	10537851	grey	0.204261811	FALSE	LINC02930

	0	0	7				8		2			6				
Region_2588	chr10	120759898	120851457	91559	33	249	289.1212121	17.9679331	0.793130862	0.027714738	-	283089	grey	0.03189664	FALSE	WDR11-DT
Region_2589	chr10	120851305	120909524	58219	17	102	129.3939394	13.13231925	0.719884645	0.028789755	+	55717	grey	-0.131512248	FALSE	WDR11
Region_2590	chr10	120925547	120980700	55153	37	218	288.4848485	20.155734	0.751183108	0.02179995	-	105378519	grey	0.034574393	FALSE	LOC105378519
Region_2591	chr10	121478332	121598458	120126	49	371	418.0909091	23.14152264	0.786433295	0.016496056	-	2263	grey	0.110255914	FALSE	FGFR2
Region_2592	chr10	121709393	121928801	219408	44	301	349.6060606	22.29306811	0.872347414	0.018985132	-	11101	grey	-0.072934299	FALSE	ATE1
Region_2593	chr10	121928056	121952139	24083	9	47	69.84848485	8.26157449	0.745683694	0.031228353	+	100130887	grey	-0.127167571	FALSE	ATE1-AS1
Region_2594	chr10	121957091	121975217	18126	17	101	133.8939394	16.02702409	0.177290601	0.015940767	-	54780	grey	0.060229952	FALSE	NSMCE4A
Region_2595	chr10	121989163	122254545	265382	60	406	460.2121212	24.84444848	0.79938915	0.019763138	+	10579	grey	-0.474377281	FALSE	TACC2
Region_2596	chr10	122271296	122338159	66863	7	48	60.03030303	5.974225916	0.72164558	0.064747308	+	118663	grey	-0.180863735	FALSE	BTBD16
Region_2597	chr10	122374696	122432354	57658	8	48	66.51515152	8.652687309	0.786483939	0.052083615	+	59338	grey	-0.134728899	FALSE	PLEKHA1
Region_2598	chr10	122458551	122514907	56356	15	105	126.6212121	12.32479072	0.61768645	0.031802926	+	5654	grey	0.086088103	FALSE	HTRA1
Region_2599	chr10	122560665	122643736	83071	11	83	105.9393939	11.80466503	0.845670127	0.035732742	+	1755	grey	-0.000134023	FALSE	DMBT1

Region_2600	chr10	122729429	122826448	97019	12	74	102.8939394	10.04624505	0.696837417	0.04645839	+	375940	grey	-0.164381267	FALSE	DMBT1L1
Region_2601	chr10	122832158	122845857	13699	6	43	58.84848485	7.434312185	0.937832919	0.030855393	-	50624	grey	0.099368362	FALSE	CUZD1
Region_2602	chr10	122832158	122879583	47425	10	73	97.78787879	9.906509362	0.715863511	0.032724419	-	100533195	grey	0.149568731	FALSE	FAM24B-CUZD1
Region_2603	chr10	122849078	122879641	30563	3	17	29.54545455	5.026642305	0.495415514	0.093214585	-	196792	grey	0.167011269	FALSE	FAM24B
Region_2604	chr10	122930901	122954311	23410	3	19	27.96969697	4.905161166	0.952522691	0.03958503	-	80007	grey	-0.082469345	FALSE	C10orf88
Region_2605	chr10	122954381	122997513	43132	4	19	33.98484848	6.210394325	0.88921581	0.053791335	+	118672	grey	0.236887648	FALSE	PSTK
Region_2606	chr10	123008979	123058290	49311	9	47	60.28787879	7.191373361	0.847299933	0.038156766	+	36	grey	-0.002628399	FALSE	ACADSB
Region_2607	chr10	123135970	123139423	3453	4	15	28.04545455	6.120411797	0.055467905	0.06531979	+	340784	grey	-0.376226221	FALSE	HMX3
Region_2608	chr10	123154402	123170467	16065	4	21	31.71212121	5.543576051	0.720183366	0.032431981	+	9184	grey	0.384936388	FALSE	BUB3
Region_2609	chr10	123356450	123562213	205763	56	479	520.0909091	23.85386746	0.834166027	0.02509644	+	105378531	grey	0.172101625	FALSE	LINC02641
Region_2610	chr10	123666355	123697399	31044	21	144	167.0757576	12.62392187	0.673806087	0.038386651	+	2849	grey	0.272482811	FALSE	GPR26
Region_2611	chr10	123706207	123944099	237892	98	746	815.7878788	33.93274577	0.791549367	0.016223669	-	119587	grey	0.122616707	FALSE	CPXM2
Region_2612	chr1	12400766	12409359	85930	41	286	344.530303	22.6037571	0.79018803	0.01835416	-	51363	grey	-0.22923355	FALSE	CHST15

	0	8	8					4		2				9		
Region_2613	chr10	124397303	124418976	21673	13	65	95.33333333	11.93873248	0.453333495	0.026273237	-	4942	grey	-0.016579156	FALSE	OAT
Region_2614	chr10	124445243	124450035	4792	4	18	28.3030303	5.386506494	0.14590212	0.066301769	-	390010	grey	0.13115286	FALSE	NKX1-2
Region_2615	chr10	124461823	124617888	156065	48	333	383.6515152	24.28706045	0.840798291	0.022791443	+	64077	grey	0.11665112	FALSE	LHPP
Region_2616	chr10	124617080	124791727	174647	57	427	473.1969697	23.34241415	0.778497374	0.018937705	-	9679	grey	-0.143218716	FALSE	FAM53B
Region_2617	chr10	124703625	124714217	10592	4	22	31.56060606	4.858390958	0.482736981	0.068751543	+	101927944	grey	-0.108039252	FALSE	FAM53B-AS1
Region_2618	chr10	124756254	124791887	35633	15	95	121.1818182	10.91069918	0.88791479	0.029277105	-	399818	grey	-0.285381626	FALSE	EEF1AKMT2
Region_2619	chr10	124801819	124836667	34848	15	81	116.1969697	11.52952023	0.973469899	0.019877911	+	23172	grey	-0.081089664	FALSE	ABRAXAS2
Region_2620	chr10	124917143	124988189	71046	8	51	64.62121212	7.263745911	0.940801856	0.033669859	+	54764	grey	0.041554256	FALSE	ZRANB1
Region_2621	chr10	124984317	125161170	176853	72	530	599.8484848	27.43397244	0.838620097	0.01430419	-	1488	grey	-0.013232374	FALSE	CTBP2
Region_2622	chr10	125576522	125683163	106641	28	205	245.3030303	17.2260712	0.857020716	0.027010016	-	387718	grey	0.079131154	FALSE	TEX36
Region_2623	chr10	125683229	125709677	26448	6	31	48.77272727	7.280734229	0.890461963	0.045189693	+	283038	grey	0.30191552	FALSE	LOC283038
Region_2624	chr10	125700436	125749525	49089	15	121	143.2121212	11.77417663	0.920531072	0.028267796	-	399821	grey	0.158624358	FALSE	EDRF1-DT

Region_2625	chr10	125719515	125764143	44628	11	89	114.6212121	11.29569714	0.950067062	0.023134091	+	26098	grey	0.022017172	FALSE	EDRF1
Region_2626	chr10	125725634	125752110	26476	6	37	55.40909091	7.475040052	0.973085353	0.024342209	-	101927983	grey	-0.048672285	FALSE	EDRF1-AS1
Region_2627	chr10	125753580	125775821	22241	11	73	91.78787879	10.10485816	0.901111726	0.048655843	-	118856	grey	-0.059328932	FALSE	MMP21
Region_2628	chr10	125784980	125823258	38278	19	133	162.9242424	13.40184902	0.884161191	0.025899403	-	7390	grey	0.317753386	FALSE	UROS
Region_2629	chr10	125823546	125853695	30149	14	114	142.6212121	13.76032776	0.717603424	0.022473381	+	56647	grey	-0.027683432	FALSE	BCCIP
Region_2630	chr10	125836337	125896436	60099	14	115	134.9242424	11.88316276	0.95605774	0.022126829	-	55760	grey	-0.013791148	FALSE	DHX32
Region_2631	chr10	125896564	126009592	113028	25	164	209.7424242	16.78947528	0.913551834	0.018566615	+	92565	grey	-0.092421269	FALSE	FANK1
Region_2632	chr10	126012381	126388477	376096	115	868	977.4242424	43.66553318	0.801453719	0.015597335	-	8038	grey	-0.089205672	FALSE	ADAM12
Region_2633	chr10	126413869	126421879	8010	3	15	25.83333333	5.373963824	0.877702337	0.073399007	-	101101772	grey	0.050807856	FALSE	LINC00601
Region_2634	chr10	126424997	126798708	373711	113	852	924.2121212	32.11350789	0.839697984	0.015864666	-	118611	grey	-0.121705923	FALSE	C10orf90
Region_2635	chr10	126425930	126460957	35027	13	83	108.1969697	11.84933584	0.785024778	0.038058732	+	728158	grey	-0.139767243	FALSE	LOC728158
Region_2636	chr10	126905409	127452517	547108	172	1362	1469.560606	52.53749688	0.866003679	0.013992735	+	1793	grey	0.224627708	FALSE	DOCK1
Region_2637	chr1	12700027	12702642	26146	7	47	59.8787878	6.70823868	0.75770148	0.05676155	-	10798405	grey	0.015986114	FALSE	LOC107984059

	0	7	3				8	1	4	9		9				
Region_2638	chr10	127000707	127026507	25800	7	47	59.87878788	6.708238681	0.757701484	0.056761559	-	105378552	grey	0.015986114	FALSE	LOC105378552
Region_2639	chr10	127135426	127196591	61165	12	81	106.0454545	10.4475714	0.727623568	0.040464549	-	642938	grey	-0.027767968	FALSE	INSYN2A
Region_2640	chr10	127877841	127892941	15100	11	82	103.8030303	9.751086246	0.720578507	0.041830649	-	119467	grey	0.290063242	FALSE	CLRN3
Region_2641	chr10	127907103	128085855	178752	47	359	402.7424242	22.37396193	0.817499678	0.018150587	+	5791	grey	0.058793272	FALSE	PTPRE
Region_2642	chr10	128096659	128126423	29764	10	61	92.83333333	10.30247668	0.768088524	0.016997176	-	4288	grey	0.264627785	FALSE	MKI67
Region_2643	chr10	128181032	128317726	136694	45	332	367.2575758	17.81037779	0.698808017	0.020785951	+	101927381	grey	-0.154031592	FALSE	LINC01163
Region_2644	chr10	129372194	129380753	8559	4	21	34.83333333	5.067493185	0.911525068	0.04105038	-	105378557	grey	0.226802973	FALSE	LOC105378557
Region_2645	chr10	129467190	129770983	303793	118	898	990.2272727	43.2256318	0.88406277	0.011014281	+	4255	grey	0.390697821	FALSE	MGMT
Region_2646	chr10	129701627	129717867	16240	15	101	123.5	12.52597302	0.842222614	0.032388431	+	105378560	grey	0.160521956	FALSE	LOC105378560
Region_2647	chr10	129784983	129806971	21988	11	62	92.57575758	11.2154572	0.685225526	0.055841928	+	107984280	grey	0.117624193	FALSE	LINC02666
Region_2648	chr10	129835233	129973053	137820	100	731	825.6363636	39.82679282	0.747889846	0.011168816	-	253738	grey	-0.157736325	FALSE	EBF3
Region_2649	chr10	129857638	129867384	9746	9	64	81.09090909	8.28479102	0.933336456	0.031133734	+	105378558	grey	0.197317392	FALSE	EBF3-AS1

Region_2650	chr10	130020025	130110830	90805	23	142	171.1212121	14.47599089	0.772342549	0.022531313	-	387723	grey	-0.026228866	FALSE	C10orf143
Region_2651	chr10	130136391	130184521	48130	8	39	57.09090909	6.963039285	0.727327365	0.019136401	+	10539	grey	0.165090719	FALSE	GLRX3
Region_2652	chr10	130213488	130483193	269705	82	642	693.5606061	28.16442316	0.790993779	0.018178517	+	107984002	grey	0.12980049	FALSE	LINC02646
Region_2653	chr10	131092391	131311721	219330	114	890	969.0757576	34.70593414	0.856347209	0.012772066	-	256536	grey	-0.207160101	FALSE	TCERG1L
Region_2654	chr10	131772383	131790199	17816	6	25	44.78787879	6.792564151	0.910233234	0.049685387	-	399827	grey	0.076293838	FALSE	LINC01164
Region_2655	chr10	131901008	131959834	58826	46	295	357.8484848	25.6060344	0.843402416	0.015781609	+	55844	grey	0.184014381	FALSE	PPP2R2D
Region_2656	chr10	131967684	131981967	14283	7	38	55.04545455	7.670987938	0.817441738	0.050697305	-	664	grey	-0.076171375	FALSE	BNIP3
Region_2657	chr10	132036336	132184858	148522	78	535	607.0151515	36.00106512	0.773628533	0.01853424	+	282973	grey	-0.092247766	FALSE	JAKMIP3
Region_2658	chr10	132186948	132205759	18811	23	123	158.9545455	15.07869798	0.833297191	0.027833847	+	10570	grey	0.144438138	FALSE	DPYSL4
Region_2659	chr10	132207492	132331847	124355	93	601	702.5606061	41.36149409	0.807268498	0.013698315	-	282974	grey	0.130231215	FALSE	STK32C
Region_2660	chr10	132332154	132381508	49354	35	224	276.8939394	24.44090442	0.848856363	0.017114159	+	80313	grey	-0.043673081	FALSE	LRRC27
Region_2661	chr10	132397168	132417859	20691	12	56	79.92424242	9.78902149	0.778999267	0.037385647	+	170394	grey	0.117480346	FALSE	PWWP2B
Region_2662	chr1	13244432	13244940	5081	4	11	25.1212121	5.43638780	0.28967037	0.06274014	+	170393	grey	0.218211403	FALSE	LINC02870

	0	7	8				2	4	8	9						
Region_2663	chr10	132508394	132518367	9973	8	37	50.57575758	6.157073746	0.743313386	0.052771253	-	107984282	grey	-0.037783762	FALSE	LOC107984282
Region_2664	chr10	132537787	132783480	245693	174	1266	1385.318182	59.0434414	0.881746269	0.010008483	+	3632	grey	-0.155070889	FALSE	INPP5A
Region_2665	chr10	132783179	132786147	2968	7	27	43.90909091	7.705596912	0.125899631	0.070678036	-	84504	grey	0.089739678	FALSE	NKX6-2
Region_2666	chr10	132808392	132942823	134431	119	768	864.530303	46.32169111	0.823397161	0.013978812	-	54777	grey	-0.209612415	FALSE	CFAP46
Region_2667	chr10	132943967	132965289	21322	15	107	135.3939394	11.8176905	0.784832338	0.02955532	-	101927590	grey	-0.090117762	FALSE	LINC01166
Region_2668	chr10	132965534	132976354	10820	8	51	61.42424242	7.249531775	0.889747823	0.055197333	+	399829	grey	-0.120917884	FALSE	LINC01168
Region_2669	chr10	133013653	133015790	2137	3	10	21.15151515	5.770595021	0.96625036	0.043858715	+	112268067	grey	-0.096572997	FALSE	LOC112268067
Region_2670	chr10	133083073	133087790	4717	5	26	40.21212121	6.230236819	0.651174065	0.078904194	-	100128127	grey	-0.013566637	FALSE	ADGRA1-AS1
Region_2671	chr10	133087924	133131675	43751	36	223	271.2575758	22.0539971	0.638743627	0.028108623	+	84435	grey	-0.112945112	FALSE	ADGRA1
Region_2672	chr10	133160219	133226412	66193	65	418	474.3333333	25.96348521	0.769827738	0.020832616	+	85442	grey	-0.068485686	FALSE	KNDC1
Region_2673	chr10	133230217	133231558	1341	7	21	38.71212121	7.087942419	0.057350125	0.034788515	+	8433	grey	-0.005429417	FALSE	UTF1
Region_2674	chr10	133237855	133241928	4073	3	10	17.63636364	4.270241801	0.463859191	0.101814621	+	27287	grey	0.084084977	FALSE	VENTX

Region_2675	chr10	133262420	133276868	14448	12	48	83.74242424	12.35165589	0.797484553	0.029897794	-	101	grey	0.091941649	FALSE	ADAM8
Region_2676	chr10	133278635	133318823	40188	33	233	279.5454545	22.07482508	0.6869233	0.016192965	-	10844	grey	-0.120652623	FALSE	TUBGCP2
Region_2677	chr10	133308914	133313162	4248	4	19	29.71212121	5.916769263	0.731000939	0.050737571	+	118472	grey	0.021919951	FALSE	ZNF511
Region_2678	chr10	133309410	133352529	43119	37	188	237.1818182	22.19695689	0.457649094	0.023789884	+	104326056	grey	0.158289754	FALSE	ZNF511-PRAP1
Region_2679	chr10	133324072	133336935	12863	22	78	116.5606061	15.7364142	0.22080589	0.030504658	-	50632	grey	0.243885128	FALSE	CALY
Region_2680	chr10	133347368	133352683	5315	4	20	28.90909091	4.948122481	0.736155999	0.103772783	+	118471	grey	-0.121278692	FALSE	PRAP1
Region_2681	chr10	133355158	133358025	2867	4	14	27.45454545	6.310386598	0.047738604	0.04631323	-	282969	grey	0.295943804	FALSE	FUOM
Region_2682	chr10	133362485	133373354	10869	9	49	69.84848485	9.795198862	0.741413162	0.033964682	-	1892	grey	-0.084811005	FALSE	ECHS1
Region_2683	chr10	133379261	133420271	41010	3	12	19.07575758	4.069816122	0.328375237	0.043053918	+	196743	grey	0.012992561	FALSE	PAOX
Region_2684	chr10	133453928	133523558	69630	26	177	219.9393939	35.58343555	0.866362801	0.02052132	+	619207	grey	-0.027803233	FALSE	SCART1
Region_2685	chr10	133520406	133561220	40814	26	176	219.7575758	35.11215464	0.478011339	0.046237556	+	1571	grey	-0.094251954	FALSE	CYP2E1
Region_2686	chr10	133553901	133568291	14390	6	29	43.43939394	9.351476704	0.624481471	0.078888866	-	93426	grey	-0.012884798	FALSE	SYCE1
Region_2687	chr1	167784	207383	39599	23	133	189.212121	31.0233238	0.64339379	0.02083077	-	388572	grey	-0.14025107	FALSE	

	1						2	1	9	9				9		
Region_2688	chr11	202924	207399	4475	6	27	48.12121212	10.96920886	0.166240126	0.009894892	-	51272	grey	0.12769347	FALSE	BET1L
Region_2689	chr11	207708	215113	7405	15	58	96.40909091	17.10328558	0.004882629	0.009068268	+	60626	grey	-0.233216267	FALSE	RIC8A
Region_2690	chr11	215030	236931	21901	12	66	92.40909091	12.71338153	0.560768444	0.031396369	-	23410	grey	-0.042187362	FALSE	SIRT3
Region_2691	chr11	236966	252984	16018	6	24	39.81818182	7.01626482	0.787619903	0.05695457	+	5719	grey	0.038450286	FALSE	PSMD13
Region_2692	chr11	278407	285388	6981	45	278	349.1060606	40.28556863	0.084107706	0.020008746	+	171389	grey	0.378669513	FALSE	NLRP6
Region_2693	chr11	287305	296107	8802	5	27	39.92424242	6.696291984	0.516378319	0.059544253	+	80162	grey	0.276111104	FALSE	PGGHG
Region_2694	chr11	298200	299526	1326	3	15	25.46969697	5.117750999	0.922974034	0.062907374	-	387733	grey	-0.061926255	FALSE	IFITM5
Region_2695	chr11	308408	315272	6864	6	24	37.24242424	5.833749569	0.331945073	0.086799343	+	8519	grey	-0.029412902	FALSE	IFITM1
Region_2696	chr11	319676	327537	7861	4	16	30.86363636	5.08054014	0.756782328	0.04461036	-	10410	grey	-0.053302939	FALSE	IFITM3
Region_2697	chr11	369499	382117	12618	49	161	228.469697	34.01140971	0.557165955	0.024535476	+	338707	grey	-0.156425994	FALSE	B4GALNT4
Region_2698	chr11	392614	404908	12294	13	37	65.95454545	13.00465221	0.738263693	0.118805782	+	11187	grey	-0.002784437	FALSE	PKP3
Region_2699	chr11	405716	417455	11739	25	91	130	17.8764994	0.196309416	0.019657628	-	59307	grey	0.164968366	FALSE	SIGIRR

Region_2700	chr11	417933	442011	24078	41	130	216.2272727	31.42943033	0.920544232	0.01865887	-	338440	grey	-0.11698672	FALSE	ANO9
Region_2701	chr11	694438	727727	33289	75	380	476.9393939	39.49523001	0.275328339	0.01535132	+	64787	grey	0.05116553	FALSE	EPS8L2
Region_2702	chr11	695591	705028	9437	22	102	155.8939394	22.72452338	0.176400601	0.023415404	+	283232	grey	-0.116702197	FALSE	TMEM80
Region_2703	chr11	747415	765012	17597	15	64	99.92424242	13.95962376	0.51243093	0.024043732	+	6888	grey	-0.098562743	FALSE	TALDO1
Region_2704	chr11	767220	777488	10268	10	43	62.59090909	9.82771164	0.537756461	0.038377735	-	347862	grey	-0.342191388	FALSE	GATD1
Region_2705	chr11	777574	785231	7657	18	79	112.7272727	16.12121913	0.343468915	0.026571289	+	171391	grey	0.055104966	FALSE	GATD1-DT
Region_2706	chr11	787115	790113	2998	8	25	38.16666667	6.669294354	0.656078607	0.091803016	-	51286	grey	-0.025196646	FALSE	CEND1
Region_2707	chr11	790475	798281	7806	15	53	82.53030303	13.29339882	0.518943178	0.023446015	-	79751	grey	-0.047817667	FALSE	SLC25A22
Region_2708	chr11	797511	799190	1679	10	23	51.09090909	11.52352413	0.005387861	0.014274048	+	101927423	grey	0.006154109	FALSE	PANO1
Region_2709	chr11	799179	809753	10574	19	109	151.9848485	17.56525629	0.532109166	0.018940113	-	55367	grey	-0.034074781	FALSE	PIDD1
Region_2710	chr11	809965	812880	2915	7	17	39.25757576	11.34807419	0.003543943	0.009114482	+	6181	grey	0.048805069	FALSE	RPLP2
Region_2711	chr11	818914	825573	6659	26	142	186.5454545	26.00010758	0.514479953	0.021951361	+	57104	grey	-0.106155831	FALSE	PNPLA2
Region_2712	chr1	826144	831991	5847	19	70	105.878787	17.9405129	0.09628080	0.02556401	+	283229	grey	-0.15821828	FALSE	CRACR2B

	1						9	4	2	6				9		
Region_2713	chr11	832887	838835	5948	11	37	57.37878788	10.56811627	0.360753277	0.027255518	+	977	grey	-0.024800147	FALSE	CD151
Region_2714	chr11	837356	842529	5173	5	28	41.3030303	6.404252666	0.58961972	0.049114814	-	5441	grey	-0.115997455	FALSE	POLR2L
Region_2715	chr11	842812	867116	24304	48	226	290.7424242	32.24011386	0.403878368	0.032613583	+	7106	grey	-0.292855799	FALSE	TSPAN4
Region_2716	chr11	867859	915058	47199	59	312	415.7727273	32.32612445	0.543557806	0.012809435	-	66005	grey	0.088097329	FALSE	CHID1
Region_2717	chr11	1223066	1262172	39106	60	319	397.3030303	32.7512629	0.891336614	0.01484101	+	727897	grey	-0.19028645	FALSE	MUC5B
Region_2718	chr11	1242261	1249676	7415	10	27	62.89393939	11.7710581	0.952000346	0.039337296	-	112577518	grey	-0.179343575	FALSE	MUC5B-AS1
Region_2719	chr11	1274371	1309654	35283	83	469	575.1969697	38.3655373	0.823473578	0.020552346	-	54472	grey	0.078286721	FALSE	TOLLIP
Region_2720	chr11	1298149	1309644	11495	35	209	254.3939394	20.15087613	0.749098466	0.033891381	-	105376512	grey	0.228912071	FALSE	
Region_2721	chr11	1309769	1310707	938	11	55	80.93939394	13.22803409	0.12604499	0.032002872	+	255512	grey	-0.091426663	FALSE	TOLLIP-DT
Region_2722	chr11	1350359	1364286	13927	18	106	130.3636364	11.73638178	0.875185125	0.035644233	+	105376513	grey	-0.278832933	FALSE	LINC02689
Region_2723	chr11	1389899	1462689	72790	164	871	992.3484848	54.16058528	0.792416263	0.016198612	+	9024	grey	-0.294320214	FALSE	BRSK2
Region_2724	chr11	1469457	1501247	31790	50	307	368.0909091	29.08117407	0.946149825	0.012342951	-	81532	grey	-0.083108902	FALSE	MOB2

Region_2725	chr11	1734821	1764573	29752	18	101	123	10.08578589	0.593705178	0.045506435	-	1509	grey	-0.198096182	FALSE	CTSD
Region_2726	chr11	1776930	1778388	1458	3	15	22.59090909	4.406209858	0.642491627	0.104280789	-	105376517	grey	-0.328912477	FALSE	LOC105376517
Region_2727	chr11	2129112	2158391	29279	31	185	215.9393939	16.88593296	0.406439397	0.028996242	-	3481	grey	-0.165973097	FALSE	IGF2
Region_2728	chr11	2132538	2161209	28671	42	233	273.0606061	19.22248586	0.532281089	0.027987173	-	723961	grey	-0.157255989	FALSE	INS-IGF2
Region_2729	chr11	2140501	2148666	8165	10	51	67.83333333	8.773357104	0.137708363	0.045718959	+	51214	grey	-0.032834067	FALSE	IGF2-AS
Region_2730	chr11	2159779	2161221	1442	9	40	53.78787879	7.612772974	0.882064672	0.055554123	-	3630	grey	0.033494195	FALSE	INS
Region_2731	chr11	2163929	2171815	7886	13	53	75.93939394	9.459027089	0.797047049	0.058453743	-	7054	grey	0.26682011	FALSE	TH
Region_2732	chr11	2268498	2270588	2090	10	51	71.40909091	10.08727648	0.019067073	0.017172189	-	430	grey	0.225790656	FALSE	ASCL2
Region_2733	chr11	2301997	2318204	16207	11	60	76.83333333	9.09536371	0.871864436	0.041892284	+	10077	grey	-0.220004376	FALSE	TSPAN32
Region_2734	chr11	2328749	2377992	49243	29	185	226.3636364	16.09934195	0.826685628	0.019848821	-	101927682	grey	-0.014273779	FALSE	CD81-AS1
Region_2735	chr11	2376177	2397802	21625	23	134	165.2424242	14.42653603	0.751807246	0.032964895	+	975	grey	-0.29813157	FALSE	CD81
Region_2736	chr11	2400488	2403878	3390	7	53	70.78787879	9.088479862	0.261823667	0.022496883	+	10078	grey	-0.029976344	FALSE	TSSC4
Region_2737	chr1	2404515	2444514	39999	71	414	489.757575	34.0221921	0.83717707	0.01842877	-	29850	grey	0.082149928	FALSE	TRPM5

	1						8	3	8	6						
Region_2738	chr11	2608328	2699994	91666	8	39	61.22727273	7.371035737	0.919216928	0.047029343	-	10984	grey	-0.105013843	FALSE	KCNQ1OT1
Region_2739	chr11	3087107	3166739	79632	84	529	627.1363636	36.01362407	0.65399414	0.019189439	-	114879	grey	0.220387777	FALSE	OSBPL5
Region_2740	chr11	3225030	3232417	7387	5	20	33.06060606	5.953354033	0.75446696	0.061689903	-	116534	grey	-0.237563015	FALSE	MRGPRE
Region_2741	chr11	3339261	3379222	39961	11	48	71.54545455	8.44563031	0.770183258	0.039231351	-	7748	grey	0.359060208	FALSE	ZNF195
Region_2742	chr11	3380918	3408981	28063	16	123	145.530303	13.56944497	0.848015698	0.034691304	+	650368	grey	-0.1253152	FALSE	TSSC2
Region_2743	chr11	3481520	3581211	99691	31	213	245.469697	16.84976119	0.790050642	0.023179289	-	101927708	grey	-0.373974449	FALSE	LOC101927708
Region_2744	chr11	3616679	3637672	20993	9	56	72	9.062178376	0.801416738	0.044892526	+	7221	grey	-0.527908561	FALSE	TRPC2
Region_2745	chr11	3645128	3664416	19288	16	79	106.469697	13.02922124	0.87304795	0.042133043	+	417	grey	-0.108710464	FALSE	ART1
Region_2746	chr11	3665587	3671384	5797	5	24	38	7.400623674	0.982130471	0.023800222	-	57053	grey	-0.203444105	FALSE	CHRNA10
Region_2747	chr11	3671083	3797792	126709	49	361	416.0454545	20.8905071	0.847779909	0.014164595	-	4928	grey	-0.28575975	FALSE	NUP98
Region_2748	chr11	3797724	3826371	28647	17	93	114.7272727	9.994683902	0.797335971	0.020892152	+	27315	grey	0.09055747	FALSE	PGAP2
Region_2749	chr11	3826978	3840959	13981	11	60	79.72727273	9.683523603	0.619280141	0.04193625	-	391	grey	-0.158845931	FALSE	RHOG

Region_2750	chr11	3854527	4093210	238683	35	272	301.3636364	16.8920886	0.702021616	0.016503278	+	6786	grey	-0.23852976	FALSE	STIM1
Region_2751	chr11	4094707	4138932	44225	19	127	170.3030303	15.76263322	0.765670828	0.026407388	+	6240	grey	-0.19991038	FALSE	RRM1
Region_2752	chr11	4680171	4697854	17683	4	31	41.66666667	6.177710139	0.915948127	0.048422695	-	81285	grey	0.269203113	FALSE	OR51E2
Region_2753	chr11	4704784	4992431	287647	11	49	85.13636364	9.161602755	0.847434854	0.063201973	+	56547	grey	0.276914235	FALSE	MMP26
Region_2754	chr11	5248270	5505605	257335	15	85	106.2424242	10.27328216	0.900229146	0.038968716	-	3048	grey	-0.040198911	FALSE	HBG2
Region_2755	chr11	5254392	5938619	684227	76	529	585.030303	22.94808167	0.877263267	0.014848663	-	85363	grey	-0.139876623	FALSE	TRIM5
Region_2756	chr11	5254898	5505652	250754	15	85	106.2424242	10.27328216	0.900229146	0.038968716	-	3046	grey	-0.040198911	FALSE	HBE1
Region_2757	chr11	5303444	5505652	202208	12	56	81.93939394	9.880639637	0.88337151	0.045885543	-	282763	grey	-0.048986769	FALSE	OR51B5
Region_2758	chr11	5596109	5612958	16849	5	31	49.07575758	6.617716562	0.773715603	0.03054323	+	117854	grey	-0.057394181	FALSE	TRIM6
Region_2759	chr11	5596725	5644398	47673	15	97	124.3939394	11.52639618	0.897215631	0.01798645	+	445372	grey	-0.251564128	FALSE	TRIM6-TRIM34
Region_2760	chr11	5619764	5644398	24634	9	48	70.77272727	9.549539585	0.956400575	0.027542583	+	53840	grey	-0.216475755	FALSE	TRIM34
Region_2761	chr11	5689610	5737089	47479	10	50	76.21212121	8.473515575	0.750535321	0.029915975	+	10346	grey	-0.136763453	FALSE	TRIM22
Region_2762	chr1	5938751	5951347	12596	3	21	29.9090909	4.92943208	0.84770854	0.08029916	+	390083	grey	0.192886318	FALSE	OR56A3

	1						1	7	3	3						
Region_2763	chr1 1	6211345	6234711	23366	3	11	21.7272727 3	5.34802754 3	0.96825907	0.03945794 6	-	84067	grey	0.072827832	FALSE	FHIP1B
Region_2764	chr1 1	6234765	6244479	9714	3	11	22.8030303	4.80134499 4	0.67000270 9	0.07652699	+	1262	grey	-0.06495360 4	FALSE	CNGA4
Region_2765	chr1 1	6259806	6272127	12321	3	16	25.5757575 8	4.75575960 5	0.81012737 3	0.12093547	+	887	grey	-0.09044730 8	FALSE	CCKBR
Region_2766	chr1 1	6319127	6360454	41327	4	20	31.6363636 4	5.98366073 6	0.70021977 2	0.03974680 5	+	10192782 5	grey	0.107692911	FALSE	LOC101927825
Region_2767	chr1 1	6395125	6419414	24289	17	108	127.681818 2	11.2648658 6	0.68852333 3	0.03127207 1	-	322	grey	0.150433894	FALSE	APBB1
Region_2768	chr1 1	6431049	6442617	11568	12	94	119.090909 1	12.5645744 6	0.85339590 9	0.02509312 8	-	3263	grey	-0.12252682	FALSE	HPX
Region_2769	chr1 1	6448613	6474459	25846	6	30	46.5454545 5	7.57969315	0.87117693 9	0.07204665	-	10612	grey	0.15825422	FALSE	TRIM3
Region_2770	chr1 1	6474683	6481479	6796	4	14	27.6666666 7	4.57529598 3	0.95116063 8	0.04294104 4	-	23647	grey	-0.06115407 2	FALSE	ARFIP2
Region_2771	chr1 1	6481485	6508978	27493	7	40	57.3787878 8	7.35216378 7	0.96206981 6	0.02716482 9	+	26515	grey	-0.03223701	FALSE	TIMM10B
Region_2772	chr1 1	6497260	6593758	96498	19	130	154.212121 2	11.6017441	0.95321287	0.02211163 2	+	144132	grey	-0.07371088 5	FALSE	DNHD1
Region_2773	chr1 1	6610883	6619448	8565	4	16	24.1818181 8	4.45458826 9	0.54739945 5	0.06480373 7	-	1200	grey	-0.04788611 3	FALSE	TPP1
Region_2774	chr1 1	6621330	6655809	34479	28	218	254.106060 6	21.0136430 3	0.94628747	0.01638841 2	-	8642	grey	-0.04986745 4	FALSE	DCHS1

Region_2775	chr11	6821942	6926878	104936	8	38	54.06060606	6.811480779	0.671572661	0.040292963	-	107984019	grey	0.443214331	FALSE	LOC107984019
Region_2776	chr11	6926404	7001004	74600	9	52	65.96969697	6.506729527	0.568791982	0.059312773	+	7762	grey	0.137791254	FALSE	ZNF215
Region_2777	chr11	7020479	7071526	51047	4	20	30.53030303	4.393070407	0.787708525	0.088108475	+	338323	grey	0.315394642	FALSE	NLRP14
Region_2778	chr11	7238778	7469043	230265	16	113	144.2272727	12.1690597	0.682439593	0.036236301	+	143425	grey	0.226385646	FALSE	SYT9
Region_2779	chr11	7418826	7513661	94835	14	104	129.3636364	11.1225846	0.866194682	0.037319857	-	100506258	grey	0.267517066	FALSE	SYT9-AS1
Region_2780	chr11	7485388	7511377	25989	4	24	35.42424242	6.305212998	0.970234863	0.028924877	+	283298	grey	-0.083269636	FALSE	OLFML1
Region_2781	chr11	7513298	7657127	143829	13	89	113.4242424	10.84869451	0.910902632	0.033545352	+	8495	grey	0.061486198	FALSE	PPFIBP2
Region_2782	chr11	7665100	7677222	12122	7	34	48.06060606	8.17101592	0.549869464	0.063744026	-	51700	grey	-0.236245121	FALSE	CYB5R2
Region_2783	chr11	7689437	7706477	17040	6	28	40.74242424	6.848493616	0.326706487	0.073128554	-	341277	grey	-0.187872253	FALSE	OVCH2
Region_2784	chr11	7970251	8001862	31611	3	15	27.04545455	5.545425886	0.936997156	0.050686312	+	8665	grey	0.421912126	FALSE	EIF3F
Region_2785	chr11	8019244	8106243	86999	35	229	268.1515152	17.15651249	0.650545789	0.02034757	+	7275	grey	0.108976995	FALSE	TUB
Region_2786	chr11	8106056	8169055	62999	14	95	115.0151515	10.83653709	0.841605708	0.032147459	-	79608	grey	0.093652417	FALSE	RIC3
Region_2787	chr1	8169167	8178903	9736	3	10	17.8333333	4.18268715	0.64204316	0.05730876	+	10192793	grey	0.092632003	FALSE	RIC3-DT

	1						3		3	3		6				
Region_2788	chr11	8224309	8268716	44407	13	72	89.22727273	8.589430812	0.72892825	0.042024365	-	4004	grey	0.160499706	FALSE	LMO1
Region_2789	chr11	8391868	8594289	202421	17	108	137.5	11.50685414	0.902538344	0.033481822	-	65975	grey	0.279862615	FALSE	STK33
Region_2790	chr11	8612037	8682694	70657	8	42	60.93939394	7.687910165	0.851916649	0.020151842	-	9866	grey	-0.087204848	FALSE	TRIM66
Region_2791	chr11	8682788	8689872	7084	3	11	21.39393939	4.69984873	0.358453317	0.068018942	+	6157	grey	0.21333946	FALSE	RPL27A
Region_2792	chr11	8693351	8910951	217600	53	357	400.6060606	18.84746772	0.881829921	0.017999524	-	6764	grey	0.164660324	FALSE	DENND2B
Region_2793	chr11	8768778	8810231	41453	10	41	61.95454545	7.263039877	0.838830699	0.048965479	+	102724784	grey	0.254090504	FALSE	DENND2B-AS1
Region_2794	chr11	8920076	8933006	12930	7	43	63.63636364	8.121455655	0.806761423	0.023722937	-	56673	grey	-0.024927916	FALSE	C11orf16
Region_2795	chr11	8947202	8965011	17809	8	43	61.68181818	9.333412421	0.961270847	0.026819938	-	56674	grey	0.169321665	FALSE	TMEM9B
Region_2796	chr11	8964502	8977527	13025	4	18	31.96969697	6.492527532	0.880979894	0.067279402	+	493900	grey	0.00783434	FALSE	TMEM9B-AS1
Region_2797	chr11	8965682	9005288	39606	12	69	94.75757576	10.20567053	0.912703991	0.02842081	-	56675	grey	0.033553956	FALSE	NRIP3
Region_2798	chr11	9004093	9067776	63683	18	134	161.1969697	13.81439572	0.914937534	0.03076502	+	105376541	grey	-0.284888803	FALSE	NRIP3-DT
Region_2799	chr11	9019476	9138114	118638	32	238	279.1212121	18.81857932	0.835355662	0.02564776	-	57758	grey	-0.27313566	FALSE	SCUBE2

Region_2800	chr11	9138825	9265959	127134	31	196	244.3484848	17.79411521	0.849509652	0.029364378	-	23258	grey	-0.152276164	FALSE	DENND5A
Region_2801	chr11	9280654	9314636	33982	20	136	158.0909091	12.30850522	0.958856653	0.01957452	-	440026	grey	0.017263893	FALSE	TMEM41B
Region_2802	chr11	9384652	9448127	63475	27	205	237.8030303	19.04667926	0.806744732	0.019693998	+	10527	grey	0.0354573	FALSE	IPO7
Region_2803	chr11	9459549	9460702	1153	3	16	27.36363636	5.246643949	0.047280259	0.042057569	-	644656	grey	0.165792472	FALSE	LOC644656
Region_2804	chr11	9460319	9528524	68205	35	213	247.4848485	19.81704195	0.814731497	0.011438731	+	7702	grey	0.261614277	FALSE	ZNF143
Region_2805	chr11	9573670	9593457	19787	9	48	63.8030303	8.021547322	0.42615451	0.020464352	+	7465	grey	-0.2251089	FALSE	WEE1
Region_2806	chr11	9664077	9752993	88916	22	132	170.4848485	15.03456778	0.821371937	0.039172701	+	23075	grey	0.406814616	FALSE	SWAP70
Region_2807	chr11	9758268	9811335	53067	10	61	77.74242424	9.018460735	0.773114796	0.043283989	+	283104	grey	0.036669298	FALSE	SBF2-AS1
Region_2808	chr11	9758296	9808038	49742	10	61	77.74242424	9.018460735	0.773114796	0.043283989	+	105369149	grey	0.036669298	FALSE	LOC105369149
Region_2809	chr11	9776776	10304877	528101	72	518	588.6212121	30.50587089	0.909466487	0.013624361	-	81846	grey	-0.139040517	FALSE	SBF2
Region_2810	chr11	9839143	9929263	90120	14	93	114.4393939	10.27338426	0.961489903	0.021818756	+	101928008	grey	-0.056079928	FALSE	LOC101928008
Region_2811	chr11	10308313	10410838	102525	14	93	119.8484848	11.7384869	0.876129917	0.034148736	+	100130460	grey	0.12257163	FALSE	CAND1.11
Region_2812	chr1	10317671	10507579	189908	27	205	243.484848	15.8892730	0.79538348	0.02854985	+	272	grey	0.08344214	FALSE	AMPD3

	1						5	4	3							
Region_2813	chr11	10511673	10541230	29557	5	24	40.42424242	6.08923541	0.808976074	0.073992546	-	50862	grey	0.005217806	FALSE	RNF141
Region_2814	chr11	10541258	10599939	58681	13	80	106.3939394	10.05810624	0.94280696	0.026740256	+	100129827	grey	-0.163383428	FALSE	IRAG1-AS1
Region_2815	chr11	10556966	10611689	54723	12	73	95.87878788	10.04836788	0.861995342	0.029394815	-	10894	grey	-0.101556077	FALSE	LYVE1
Region_2816	chr11	10573091	10693988	120897	22	153	189.4242424	15.14043044	0.9014377	0.01909714	-	10335	grey	-0.032205787	FALSE	IRAG1
Region_2817	chr11	10751246	10801625	50379	8	44	61.25757576	8.291442495	0.943873361	0.029372194	+	9646	grey	-0.071041192	FALSE	CTR9
Region_2818	chr11	10797050	10808940	11890	7	33	53.54545455	8.042544217	0.232221737	0.027081664	-	1982	grey	0.035243412	FALSE	EIF4G2
Region_2819	chr11	10809204	10822931	13727	3	21	30.86363636	4.550186348	0.966119041	0.034670257	+	101928053	grey	0.173692761	FALSE	LOC101928053
Region_2820	chr11	10812074	10856218	44144	5	22	33.40909091	5.329099427	0.958367516	0.035559678	-	105376547	grey	0.055838453	FALSE	
Region_2821	chr11	10849897	10858796	8899	3	21	30.93939394	5.724115754	0.00507064	0.013214226	-	58486	grey	-0.028197063	FALSE	ZBED5
Region_2822	chr11	10858179	10908972	50793	16	91	122.3181818	12.45318506	0.839171421	0.021819495	+	729013	grey	0.239997214	FALSE	ZBED5-AS1
Region_2823	chr11	11020883	11183611	162728	13	68	99.45454545	10.83751578	0.894406475	0.040980554	+	105376550	grey	-0.008499822	FALSE	LINC02752
Region_2824	chr11	11270877	11622005	351128	62	484	528.7272727	25.95768477	0.87770027	0.019555491	-	374378	grey	0.107716214	FALSE	GALNT18

Region_2825	chr11	11475177	11621758	146581	22	154	187.469697	14.77130242	0.839711631	0.025837071	-	107983976	grey	-0.063787685	FALSE	LOC107983976
Region_2826	chr11	11841423	11961887	120464	7	43	61.15151515	7.463227646	0.958928834	0.031566059	+	55031	grey	-0.18202763	FALSE	USP47
Region_2827	chr11	11956207	12009769	53562	4	28	45.75757576	7.524443462	0.946130782	0.046836436	-	27122	grey	-0.031246307	FALSE	DKK3
Region_2828	chr11	12030875	12061785	30910	4	20	33.3030303	5.828232868	0.867475889	0.057291678	+	105376554	grey	-0.012430968	FALSE	LINC02547
Region_2829	chr11	12094008	12359144	265136	66	501	550.2272727	21.84828489	0.811901086	0.018313077	+	9645	grey	0.30872118	FALSE	MICAL2
Region_2830	chr11	12377563	12535356	157793	21	130	157.1060606	12.09720729	0.838791959	0.030886724	+	55742	grey	-0.420539548	FALSE	PARVA
Region_2831	chr11	12619326	12668495	49169	4	23	31.81818182	5.212142896	0.898084079	0.063621696	+	105376556	grey	0.109775778	FALSE	LOC105376556
Region_2832	chr11	12674421	12944737	270316	57	428	492.6363636	22.51675367	0.791638347	0.018001294	+	7003	grey	-0.132713925	FALSE	TEAD1
Region_2833	chr11	12961541	12989597	28056	3	19	28.40909091	5.265204424	0.552921957	0.055716052	-	100506305	grey	-0.245337878	FALSE	LINC00958
Region_2834	chr11	13276652	13387266	110614	27	213	249.6363636	17.0028381	0.848148201	0.018880006	+	406	grey	-0.046975771	FALSE	BMAL1
Region_2835	chr11	13388008	13463297	75289	7	29	52.22727273	9.153049857	0.786309583	0.057580688	-	84280	grey	-0.214881823	FALSE	BTBD10
Region_2836	chr11	13668668	13732346	63678	7	37	53.12121212	6.769877062	0.698675028	0.018440726	+	84188	grey	0.210065375	FALSE	FAR1
Region_2837	chr1	13962723	14268133	305410	21	158	197.363636	13.9335084	0.78190597	0.02676395	+	10418	grey	-0.31010823	FALSE	SPON1

	1						4	4	2	5				4		
Region_2838	chr1<br>1	14277922	14364506	86584	12	71	90.5454545<br>5	9.07266701<br>2	0.84924106<br>9	0.03281297	-	22800	grey	0.019598253	FALSE	RRAS2
Region_2839	chr1<br>1	14451835	14500027	48192	7	36	50.9242424<br>2	6.60608252<br>6	0.69248934<br>7	0.03137763<br>6	-	1315	grey	-0.00871174<br>6	FALSE	COPB1
Region_2840	chr1<br>1	14493783	14643635	149852	31	211	239.878787<br>9	17.7092024<br>3	0.80668629<br>1	0.02022770<br>7	-	5682	grey	-0.00936299<br>2	FALSE	PSMA1
Region_2841	chr1<br>1	14643804	14872044	228240	20	146	177.984848<br>5	13.1914802<br>6	0.74727259<br>3	0.01763448<br>4	+	5140	grey	0.035578526	FALSE	PDE3B
Region_2842	chr1<br>1	14904997	15082342	177345	21	153	186.121212<br>1	14.3081635<br>1	0.63645015<br>6	0.02711010<br>7	+	797	grey	0.040866286	FALSE	CALCB
Region_2843	chr1<br>1	15112424	15247208	134784	7	45	61.4242424<br>2	7.48549490<br>9	0.90716318<br>7	0.04026309<br>6	+	387755	grey	0.073017095	FALSE	INSC
Region_2844	chr1<br>1	15552855	15705376	152521	16	114	136.893939<br>4	10.0814052<br>6	0.64731657<br>4	0.04268618<br>8	+	10537656<br>9	grey	-0.14622751<br>1	FALSE	LINC02751
Region_2845	chr1<br>1	15571813	15622447	50634	6	45	57.6515151<br>5	6.43904537<br>7	0.80660802<br>6	0.05794245<br>3	-	10537656<br>8	grey	-0.03459668<br>1	FALSE	LOC105376568
Region_2846	chr1<br>1	15966449	16739591	773142	48	362	410.121212<br>1	20.2344072<br>2	0.79074205<br>6	0.0256116	-	55553	grey	-0.12029074<br>6	FALSE	SOX6
Region_2847	chr1<br>1	16613132	16748421	135289	15	92	119.757575<br>8	10.4896441<br>3	0.82876822<br>3	0.03348335<br>7	+	10537657<br>1	grey	0.121075901	FALSE	LOC105376571
Region_2848	chr1<br>1	16777297	17014415	237118	39	261	308.818181<br>8	18.2283879<br>1	0.83526208<br>2	0.01852195<br>5	-	144100	grey	0.152624114	FALSE	PLEKHA7
Region_2849	chr1<br>1	17014304	17052976	38672	13	56	80.2121212<br>1	9.80818363<br>4	0.86595447<br>1	0.03175036<br>4	+	11226808<br>5	grey	0.110510254	FALSE	

Region_2850	chr11	17077730	17207986	130256	30	200	233.0757576	14.86069737	0.803242822	0.016415477	-	5286	grey	0.27296747	FALSE	PIK3C2A
Region_2851	chr11	17208153	17349980	141827	41	293	333.6969697	18.42487424	0.869168271	0.014988015	+	4925	grey	-0.019825405	FALSE	NUCB2
Region_2852	chr11	17351800	17377341	25541	7	34	52.72727273	7.608791382	0.808194251	0.040988151	+	374383	grey	0.111172446	FALSE	NCR3LG1
Region_2853	chr11	17365172	17389331	24159	6	27	44.5	6.650043378	0.785851575	0.047588166	-	3767	grey	-0.006016291	FALSE	KCNJ11
Region_2854	chr11	17392498	17476894	84396	29	220	252.969697	18.09459732	0.653749342	0.018930063	-	6833	grey	-0.108652519	FALSE	ABCC8
Region_2855	chr11	17493895	17544416	50521	19	137	176.3030303	14.95734417	0.73655438	0.024149363	-	10083	grey	0.145332432	FALSE	USH1C
Region_2856	chr11	17547259	17647150	99891	23	150	185.3939394	15.55820712	0.843254487	0.027022488	+	340990	grey	0.104997137	FALSE	OTOG
Region_2857	chr11	17734774	17856804	122030	50	357	403.1212121	23.26999729	0.747717258	0.020518382	+	3746	grey	-0.26791932	FALSE	KCNC1
Region_2858	chr11	17772191	17856787	84596	21	140	174.7121212	14.41021556	0.927045786	0.021926689	+	107984317	grey	-0.336016836	FALSE	LOC107984317
Region_2859	chr11	17788048	18022931	234883	27	191	224.8939394	15.58315539	0.886056024	0.018195082	-	26297	grey	0.010427723	FALSE	SERGEF
Region_2860	chr11	18017555	18046269	28714	6	33	46.42424242	7.012210463	0.813617525	0.046151122	-	7166	grey	0.146677395	FALSE	TPH1
Region_2861	chr11	18069935	18106087	36152	5	24	35.95454545	5.895061344	0.777557501	0.028808755	-	113174	grey	0.176614515	FALSE	SAAL1
Region_2862	chr1	18120955	18138488	17533	4	22	31.1666666	5.02481024	0.93567048	0.05861955	+	117195	grey	-0.03432649	FALSE	MRGPRX3

	1						7		5	1				9		
Region_2863	chr11	18412318	18452063	39745	14	88	111	10.1920029	0.937479571	0.030951779	+	3948	grey	-0.070611989	FALSE	LDHC
Region_2864	chr11	18455824	18479601	23777	5	21	35.63636364	6.257638689	0.891294699	0.062127477	+	160287	grey	0.000978496	FALSE	LDHAL6A
Region_2865	chr11	18468336	18526951	58615	11	76	96.16666667	9.977795862	0.976561219	0.017236789	-	7251	grey	-0.002397728	FALSE	TSG101
Region_2866	chr11	18529609	18588747	59138	15	108	128.1363636	10.63938455	0.909144622	0.013580231	-	55293	grey	0.340273905	FALSE	UEVLD
Region_2867	chr11	18606403	18634791	28388	9	67	81.16666667	8.630149893	0.624156194	0.032004476	-	144108	grey	0.142675555	FALSE	SPTY2D1
Region_2868	chr11	18693122	18704785	11663	8	49	66.56060606	8.572099263	0.828613978	0.031856042	+	144110	grey	-0.300885684	FALSE	TMEM86A
Region_2869	chr11	18704312	18726230	21918	13	80	106.1515152	9.788914333	0.86934018	0.031024908	-	283284	grey	0.35922088	FALSE	IGSF22
Region_2870	chr11	18706537	18740568	34031	20	124	160.6666667	13.47514331	0.850732561	0.028236049	+	105376579	grey	0.270575438	FALSE	
Region_2871	chr11	18727928	18792721	64793	30	196	239.4393939	17.25476861	0.786133967	0.033569654	-	84867	grey	0.126215101	FALSE	PTPN5
Region_2872	chr11	19117099	19176422	59323	8	41	56.46969697	7.598015322	0.827048925	0.038323102	+	54503	grey	0.124496982	FALSE	ZDHHC13
Region_2873	chr11	19182030	19210571	28541	5	21	35.27272727	5.895357899	0.773793134	0.05667586	-	8048	grey	-0.015348423	FALSE	CSRP3
Region_2874	chr11	19196775	19308358	111583	17	110	133.8333333	11.44294655	0.747626975	0.029286717	+	105376580	grey	-0.027168467	FALSE	CSRP3-AS1

Region_2875	chr11	19224063	19241620	17557	6	39	51.77272727	7.176447492	0.565220686	0.043002077	-	79733	grey	-0.094105632	FALSE	E2F8
Region_2876	chr11	19350724	20121601	770877	115	890	1001.636364	37.89241303	0.813266094	0.019659232	+	89797	grey	0.026826218	FALSE	NAV2
Region_2877	chr11	20156155	20160475	4320	5	17	32.37878788	7.015949196	0.109295032	0.054452341	-	120237	grey	-0.131034457	FALSE	DBX1
Region_2878	chr11	20363685	20383782	20097	7	41	58.22727273	8.39192919	0.556337611	0.01962667	+	10553	grey	-0.029224405	FALSE	HTATIP2
Region_2879	chr11	20387558	20509338	121780	14	88	111.5	11.33442679	0.757431879	0.02811777	+	10196	grey	0.146383384	FALSE	PRMT3
Region_2880	chr11	20599594	20659285	59691	17	101	132.030303	12.72184033	0.687240935	0.040577353	+	9152	grey	0.251504721	FALSE	SLC6A5
Region_2881	chr11	20669551	21575686	906135	75	584	644.6969697	23.21602212	0.810213506	0.023345524	+	4745	grey	0.104682443	FALSE	NELL1
Region_2882	chr11	21782659	22119899	337240	20	130	154.0151515	11.81197883	0.834087081	0.03438341	+	102723370	grey	-0.231150787	FALSE	LOC102723370
Region_2883	chr11	21799934	22283567	483633	27	170	204.8030303	15.43843326	0.81775081	0.029276318	+	203859	grey	-0.191344068	FALSE	ANO5
Region_2884	chr11	22338381	22379503	41122	6	38	48.22727273	6.188812374	0.469608611	0.040912823	+	57084	grey	0.083883205	FALSE	SLC17A6
Region_2885	chr11	22445673	22492073	46400	3	16	24.3030303	3.862919171	0.872509057	0.074038661	-	102723378	grey	-0.122598345	FALSE	LINC01495
Region_2886	chr11	22625642	22945393	319751	21	139	173.6515152	15.11951609	0.812132306	0.041434484	+	2620	grey	0.017433833	FALSE	GAS2
Region_2887	chr1	23154683	23203161	48478	5	29	40.9545454	7.14128154	0.89012497	0.07346449	+	10537659	grey	0.382704807	FALSE	LINC02718

	1						5	5	9	5		2				
Region_2888	chr11	23730588	23796970	66382	4	14	26.09090909	4.976026443	0.844254777	0.07203541	+	105376593	grey	-0.094467815	FALSE	LINC02726
Region_2889	chr11	24496970	25082638	585668	41	317	352.6969697	16.9060478	0.760930955	0.031155869	+	338645	grey	-0.074598404	FALSE	LUZP2
Region_2890	chr11	26188842	26663289	474447	43	312	344.6969697	18.5023655	0.818305651	0.030141092	+	63982	grey	0.139271451	FALSE	ANO3
Region_2891	chr11	27040725	27127809	87084	8	46	65.25757576	8.541861815	0.897405101	0.04113426	+	8424	grey	-0.136887955	FALSE	BBOX1
Region_2892	chr11	27047186	27220113	172927	13	87	108.6212121	8.307114933	0.889549974	0.032311175	-	103695435	grey	0.000462534	FALSE	BBOX1-AS1
Region_2893	chr11	27365961	27472790	106829	11	71	92.25757576	8.941365592	0.759344128	0.032088541	-	55366	grey	-0.077993524	FALSE	LGR4
Region_2894	chr11	27494418	27506769	12351	4	34	44.16666667	5.882394419	0.4907892	0.025016731	-	55327	grey	0.111608755	FALSE	LIN7C
Region_2895	chr11	27506830	27698231	191401	18	119	146.1969697	11.83504535	0.843816563	0.030453342	+	497258	grey	0.079300114	FALSE	BDNF-AS
Region_2896	chr11	27617626	27634627	17001	3	12	19.53030303	4.2831322	0.912555493	0.081093412	-	101410541	grey	0.200028559	FALSE	LINC00678
Region_2897	chr11	27654893	27722058	67165	24	165	202.3181818	15.95586132	0.325708829	0.027804201	-	627	grey	0.225442929	FALSE	BDNF
Region_2898	chr11	28108248	28527041	418793	37	288	333.8181818	18.86217957	0.871868695	0.019246151	+	196074	grey	-0.243264154	FALSE	METTL15
Region_2899	chr11	28702615	29064322	361707	15	114	137.0454545	11.97617098	0.80005234	0.038600169	+	105376603	grey	0.194014327	FALSE	LINC02742

Region_2900	chr11	29335878	29989328	653450	37	272	312.6212121	19.90729681	0.809952696	0.028814115	-	105376605	grey	0.036979523	FALSE	LINC02755
Region_2901	chr11	30044053	30322988	278935	12	86	107.2727273	10.43447755	0.820726937	0.043130178	-	105376607	grey	0.102968002	FALSE	ARL14EP-DT
Region_2902	chr11	30323104	30338223	15119	4	16	26.37878788	5.77465324	0.922312599	0.077329413	+	120534	grey	0.295065513	FALSE	ARL14EP
Region_2903	chr11	30384493	30586872	202379	24	182	215.1363636	16.0919343	0.760224272	0.034852363	-	744	grey	-0.100896296	FALSE	MPPED2
Region_2904	chr11	30584112	30632583	48471	4	18	29.93939394	5.932644469	0.690242123	0.046985387	+	105376609	grey	0.107379504	FALSE	MPPED2-AS1
Region_2905	chr11	30773913	30850559	76646	4	20	30.59090909	5.331985552	0.868210778	0.06846871	+	101928338	grey	0.112430798	FALSE	LOC101928338
Region_2906	chr11	30830369	31369810	539441	33	260	291.4090909	14.24184222	0.877327625	0.023613926	-	341019	grey	0.288225514	FALSE	DCDC1
Region_2907	chr11	31369840	31432835	62995	5	27	39.96969697	5.68390358	0.93666477	0.048116653	+	120526	grey	0.061378638	FALSE	DNAJC24
Region_2908	chr11	31432401	31509645	77244	3	17	25.28787879	4.57338505	0.982267186	0.027772158	-	196294	grey	0.284163581	FALSE	IMMP1L
Region_2909	chr11	31509755	31790324	280569	29	216	250.9242424	14.70040356	0.928324451	0.015766184	+	26610	grey	0.185214863	FALSE	ELP4
Region_2910	chr11	31784779	31817961	33182	10	56	76.43939394	9.349831406	0.130467596	0.025319379	-	5080	grey	-0.034008033	FALSE	PAX6
Region_2911	chr11	31812307	32002405	190098	67	488	536.9393939	26.10622018	0.459686739	0.017135917	+	440034	grey	-0.263049869	FALSE	PAX6-AS1
Region_2912	chr1	31812391	32105722	293331	81	604	655.242424	27.0430202	0.50237826	0.01579198	+	5954	grey	-0.22428220	FALSE	RCN1

	1						2		4	3				6		
Region_2913	chr11	31826347	31829311	2964	10	51	79.37878788	10.11589024	0.081978232	0.038720161	+	103157000	grey	-0.07373345	FALSE	PAUPAR
Region_2914	chr11	32387775	32435564	47789	23	140	175.5151515	15.14264729	0.518743945	0.040286304	-	7490	grey	0.115705649	FALSE	WT1
Region_2915	chr11	32435518	32500632	65114	30	214	252.6818182	20.86377729	0.739872124	0.021640242	+	51352	grey	-0.132452797	FALSE	WT1-AS
Region_2916	chr11	32583798	32606264	22466	4	17	25.65151515	4.130533302	0.363955026	0.058481636	+	10480	grey	0.323390365	FALSE	EIF3M
Region_2917	chr11	32602721	32794662	191941	17	121	153.8181818	13.25479198	0.911813099	0.031267581	-	493860	grey	0.206128545	FALSE	CCDC73
Region_2918	chr11	32829927	32858120	28193	5	29	38.06060606	5.383389804	0.589568414	0.036546828	+	79056	grey	-0.096713341	FALSE	PRRG4
Region_2919	chr11	32892811	32993316	100505	18	117	138.7424242	11.38867265	0.843814522	0.023339334	+	79832	grey	0.28346349	FALSE	QSER1
Region_2920	chr11	33039417	33105943	66526	22	142	176.9242424	12.46448568	0.952405446	0.016675185	+	55346	grey	0.06486143	FALSE	TCP11L1
Region_2921	chr11	33077188	33162371	85183	10	65	85.86363636	8.479634179	0.95137703	0.026444409	-	1479	grey	0.161213912	FALSE	CSTF3
Region_2922	chr11	33161657	33191598	29941	11	65	90.40909091	10.88962424	0.965309259	0.023851059	+	338739	grey	0.212855487	FALSE	CSTF3-DT
Region_2923	chr11	33256672	33357023	100351	27	162	213.530303	18.95193897	0.643107116	0.020606045	+	10114	grey	0.050604359	FALSE	HIPK3
Region_2924	chr11	33376108	33674102	297994	64	468	521.1818182	26.31636466	0.876738504	0.017871233	+	25758	grey	0.179377135	FALSE	KIAA1549L

Region_2925	chr11	33665220	33696701	31481	9	63	84.95454545	9.508590154	0.842803532	0.035491144	-	105376617	grey	-0.244138483	FALSE	LOC105376617
Region_2926	chr11	33698261	33736479	38218	4	24	34.06060606	5.697175516	0.757500691	0.071218875	-	966	grey	-0.07211524	FALSE	CD59
Region_2927	chr11	33740939	33774543	33604	4	26	34.45454545	4.611630175	0.974665059	0.030000807	-	26273	grey	-0.024594119	FALSE	FBXO3
Region_2928	chr11	33858576	33892076	33500	19	120	150.530303	12.4656077	0.306195352	0.045888059	-	4005	grey	0.180856963	FALSE	LMO2
Region_2929	chr11	34051731	34102610	50879	17	121	151.3181818	11.48665994	0.780292169	0.023325424	+	4076	grey	0.194827811	FALSE	CAPRIN1
Region_2930	chr11	34105617	34146908	41291	12	68	87.12121212	9.511960328	0.968409659	0.02421386	+	55226	grey	-0.022308707	FALSE	NAT10
Region_2931	chr11	34150987	34358010	207023	74	580	624.0909091	25.13454703	0.815367347	0.013437415	-	25841	grey	-0.186688378	FALSE	ABTB2
Region_2932	chr11	34438934	34472060	33126	6	40	51.27272727	6.254956077	0.620005733	0.047435634	+	847	grey	0.082433395	FALSE	CAT
Region_2933	chr11	34478791	34525193	46402	9	52	70.98484848	8.14954916	0.776496755	0.054235175	-	2001	grey	0.117601858	FALSE	ELF5
Region_2934	chr11	34621093	34663288	42195	11	72	100.8030303	11.67010605	0.793269958	0.047437987	+	26298	grey	0.134545123	FALSE	EHF
Region_2935	chr11	34915829	35020591	104762	16	100	125.4393939	11.21417892	0.903341088	0.03158809	+	8050	grey	0.018843413	FALSE	PDHX
Region_2936	chr11	35014075	35067192	53117	10	55	71.71212121	7.816678719	0.728300125	0.053704594	-	105376626	grey	0.031309775	FALSE	LOC105376626
Region_2937	chr1	35138882	35232402	93520	7	52	69.1212121	7.73904218	0.80855840	0.04579630	+	960	grey	-0.20410115	FALSE	CD44

	1						2	2	7	6						
Region_2938	chr11	35251205	35420063	168858	11	66	85.43939394	8.811057368	0.740087555	0.065037891	-	6506	grey	-0.067049093	FALSE	SLC1A2
Region_2939	chr11	35431823	35530300	98477	6	45	61.06060606	6.593420468	0.941967808	0.034855064	-	25891	grey	-0.276949446	FALSE	PAMR1
Region_2940	chr11	35662775	35818007	155232	16	101	125.7272727	9.595744978	0.856322139	0.028271225	+	54765	grey	0.101377315	FALSE	TRIM44
Region_2941	chr11	35943981	36232136	288155	73	562	610.1666667	25.24317628	0.83306061	0.019016044	+	143458	grey	0.038246807	FALSE	LDLRAD3
Region_2942	chr11	36272292	36289449	17157	3	15	24.22727273	4.929644876	0.971026056	0.039614433	-	29099	grey	0.354699851	FALSE	COMMD9
Region_2943	chr11	36296288	36465204	168916	30	207	250.3939394	14.22444136	0.795504914	0.021062222	+	79899	grey	-0.148455598	FALSE	PRR5L
Region_2944	chr11	36483769	36510272	26503	6	35	47.60606061	6.914597241	0.522228583	0.038349445	-	7189	grey	-0.091342667	FALSE	TRAF6
Region_2945	chr11	36510372	36593156	82784	4	12	29.1969697	5.254213538	0.896131722	0.070520994	+	5896	grey	-0.024934815	FALSE	RAG1
Region_2946	chr11	36575574	36598279	22705	3	14	29.83333333	5.682857714	0.636901995	0.059287276	-	5897	grey	0.055072478	FALSE	RAG2
Region_2947	chr11	38618264	38646384	28120	3	17	28.39393939	4.948593547	0.801021715	0.083362817	-	103312105	grey	0.337569813	FALSE	LINC02759
Region_2948	chr11	40114203	41459773	1345570	97	776	831.030303	31.27937128	0.805109611	0.018767231	-	57689	grey	0.137506764	FALSE	LRRC4C
Region_2949	chr11	41518895	41714492	195597	6	34	51.03030303	7.61571189	0.882519716	0.061620648	-	105376640	grey	0.103686288	FALSE	LINC02741

Region_2950	chr11	41714534	41857733	143199	10	57	81.60606061	11.46751772	0.847492648	0.040806244	+	102723644	grey	-0.176763401	FALSE	LINC01499
Region_2951	chr11	41993381	42163851	170470	13	87	111.9393939	12.15398483	0.818840413	0.047457897	-	101928591	grey	0.044764986	FALSE	LINC02745
Region_2952	chr11	42183292	42253721	70429	4	16	27.53030303	5.503061238	0.869764511	0.099425098	-	100507205	grey	0.218129292	FALSE	LINC02740
Region_2953	chr11	43120762	43269426	148664	7	49	68.81818182	7.677958645	0.855634025	0.053998562	-	399881	grey	0.11676798	FALSE	HNRNPKP3
Region_2954	chr11	43358920	43494933	136013	22	148	184.7272727	14.59351326	0.949351683	0.024806082	+	55761	grey	-0.273785828	FALSE	TTC17
Region_2955	chr11	43556436	43856617	300181	43	295	338.8787879	18.26178452	0.511451028	0.019480845	+	51144	grey	-0.193520038	FALSE	HSD17B12
Region_2956	chr11	43833172	43880726	47554	4	18	33.12121212	5.439217001	0.702490281	0.044534728	-	105376644	grey	0.078968871	FALSE	LOC105376644
Region_2957	chr11	43880811	43920274	39463	10	70	95.89393939	11.26619011	0.960358234	0.024157049	+	221120	grey	-0.239385607	FALSE	ALKBH3
Region_2958	chr11	44047981	44059977	11996	3	12	19.59090909	4.444451243	0.968570454	0.04403653	+	390110	grey	-0.06557566	FALSE	ACCSL
Region_2959	chr11	44095648	44251962	156314	25	165	217.030303	17.01128105	0.844167655	0.024133431	+	2132	grey	0.074427388	FALSE	EXT2
Region_2960	chr11	44260440	44310139	49699	22	155	185.8030303	14.14890014	0.599476958	0.029688938	-	60529	grey	-0.223906087	FALSE	ALX4
Region_2961	chr11	44564427	44620358	55931	19	109	141.5	13.58081226	0.787428317	0.038172544	+	3732	grey	-0.101532103	FALSE	CD82
Region_2962	chr1	44726465	44932423	205958	46	307	350.545454	18.3413629	0.79685814	0.02863869	+	90139	grey	0.062626033	FALSE	TSPAN18

	1						5	4	8	4						
Region_2963	chr1<br>1	44885903	44951306	65403	27	164	196.545454<br>5	15.6068839	0.69626295<br>9	0.04716071<br>6	-	9537	grey	0.115100488	FALSE	TP53I11
Region_2964	chr1<br>1	44973770	44978028	4258	3	12	23.8484848<br>5	5.21607699<br>4	0.96474574<br>1	0.04094186<br>2	+	221122	grey	0.019784322	FALSE	LINC02685
Region_2965	chr1<br>1	45095806	45235110	139304	34	230	274.727272<br>7	18.6238436<br>5	0.85195154<br>2	0.01685233	+	56981	grey	-0.11519348<br>2	FALSE	PRDM11
Region_2966	chr1<br>1	45240302	45286341	46039	15	114	139.196969<br>7	12.2099829<br>2	0.90860592	0.03177655<br>8	-	57586	grey	-0.11201657<br>5	FALSE	SYT13
Region_2967	chr1<br>1	45355371	45367577	12206	4	23	33.5303030<br>3	5.98001722<br>9	0.66060444<br>5	0.05755160<br>6	+	10192881<br>2	grey	-0.03935486<br>9	FALSE	LINC02696
Region_2968	chr1<br>1	45371397	45388511	17114	3	14	22.5454545<br>5	4.52406154<br>5	0.42506017<br>4	0.10504692<br>2	+	399886	grey	0.13510346	FALSE	LINC02687
Region_2969	chr1<br>1	45486867	45542487	55620	9	52	66.0303030<br>3	7.76970387<br>9	0.89059161<br>4	0.04601847<br>5	-	10537665<br>4	grey	0.071489485	FALSE	LOC105376654
Region_2970	chr1<br>1	45647689	45665622	17933	5	22	35.5	6.04152298<br>7	0.34902764	0.07672718<br>2	-	8534	grey	-0.01695208<br>2	FALSE	CHST1
Region_2971	chr1<br>1	45722279	45748029	25750	4	12	23.1060606<br>1	4.03876899<br>7	0.89222792<br>5	0.07319073<br>4	+	10050738<br>4	grey	-0.04738805<br>8	FALSE	LOC100507384
Region_2972	chr1<br>1	45804072	45813016	8944	6	21	35.8636363<br>6	6.12155426<br>1	0.63499649<br>2	0.03293492<br>3	+	55343	grey	-0.17838124<br>7	FALSE	SLC35C1
Region_2973	chr1<br>1	45847118	45883248	36130	9	59	78.1666666<br>7	8.45728331<br>2	0.85160710<br>4	0.02655866<br>1	+	1408	grey	-0.01627738<br>2	FALSE	CRY2
Region_2974	chr1<br>1	45885651	45906465	20814	8	31	46.1818181<br>8	6.55135709	0.74457283	0.05597182<br>1	+	9479	grey	-0.03507792<br>9	FALSE	MAPK8IP1

Region_2975	chr11	45909663	45918812	9149	11	83	106.3333333	11.9451739	0.410496164	0.02323375	-	9409	grey	0.012665575	FALSE	PEX16
Region_2976	chr11	45921621	45929096	7475	15	52	78.54545455	12.06033782	0.605286359	0.050778876	+	120071	grey	-0.249754258	FALSE	LARGE2
Region_2977	chr11	45929319	46121454	192135	20	120	169.6969697	14.61713399	0.951895298	0.019932072	-	51317	grey	0.182371083	FALSE	PHF21A
Region_2978	chr11	46123031	46177106	54075	22	137	166.2424242	16.08923253	0.89348172	0.021828937	+	101928894	grey	-0.425362952	FALSE	LOC101928894
Region_2979	chr11	46213150	46220438	7288	4	22	36.13636364	6.316976793	0.958549739	0.028030199	-	105376662	grey	0.002616602	FALSE	LINC02710
Region_2980	chr11	46256264	46274547	18283	8	47	71	9.976896388	0.933448353	0.040629552	-	101928919	grey	-0.045908705	FALSE	LINC02489
Region_2981	chr11	46277662	46321409	43747	22	130	169.3939394	16.80739653	0.679837906	0.038434938	+	90993	grey	-0.280033383	FALSE	CREB3L1
Region_2982	chr11	46332905	46380554	47649	37	233	289.8636364	22.25231975	0.779141787	0.02170825	+	8525	grey	-0.098726197	FALSE	DGKZ
Region_2983	chr11	46383789	46391776	7987	12	76	105.8939394	11.27574495	0.398864418	0.022870702	-	1132	grey	0.171976884	FALSE	CHRM4
Region_2984	chr11	46396414	46594125	197711	58	408	471.1060606	24.81136761	0.881522682	0.015023163	-	55626	grey	-0.180112664	FALSE	AMBRA1
Region_2985	chr11	46602861	46617909	15048	15	97	128.469697	13.94960126	0.330499003	0.012639472	-	283254	grey	-0.056582992	FALSE	HARBI1
Region_2986	chr11	46617527	46674818	57291	30	227	261.3030303	15.84829008	0.792562834	0.018328028	+	9776	grey	-0.088269667	FALSE	ATG13
Region_2987	chr1	46677080	46700619	23539	14	84	112.151515	10.6860691	0.93316574	0.02678297	-	392	grey	-0.13374325	FALSE	ARHGAP1

	1						2	8	1	6				4		
Region_2988	chr1<br>1	46701030	46705912	4882	4	14	29.6515151<br>5	5.39940858<br>4	0.73502872<br>3	0.03097827<br>6	+	79797	grey	-0.16835657<br>7	FALSE	ZNF408
Region_2989	chr1<br>1	46719196	46739506	20310	17	122	155.318181<br>8	14.0287168	0.94441019<br>2	0.02049207<br>9	+	2147	grey	0.097443514	FALSE	F2
Region_2990	chr1<br>1	46743048	46846308	103260	14	92	117.621212<br>1	11.3473346<br>9	0.96197778<br>1	0.01945439	-	9793	grey	-0.02921355<br>9	FALSE	CKAP5
Region_2991	chr1<br>1	46846410	46874421	28011	9	64	81.1515151<br>5	9.83751442<br>1	0.94093972<br>9	0.02859615<br>3	+	10050740<br>1	grey	-0.22649266<br>8	FALSE	LRP4-AS1
Region_2992	chr1<br>1	46856717	46918642	61925	25	158	191.742424<br>2	15.3786171<br>3	0.75434908<br>9	0.02122451<br>9	-	4038	grey	-0.25842516<br>1	FALSE	LRP4
Region_2993	chr1<br>1	46936689	47164385	227696	34	211	245.545454<br>5	15.6098409	0.94311271<br>9	0.01322091<br>7	+	79096	grey	0.436948816	FALSE	CSTPP1
Region_2994	chr1<br>1	47164299	47177125	12826	7	31	51.4393939<br>4	8.72155382<br>7	0.39497996<br>4	0.02918401<br>5	-	84364	grey	-0.06188658<br>3	FALSE	ARFGAP2
Region_2995	chr1<br>1	47177522	47186443	8921	9	48	69.2727272<br>7	9.46823914<br>2	0.65053632<br>8	0.02980192<br>4	-	29763	grey	-0.10663866<br>8	FALSE	PACSIN3
Region_2996	chr1<br>1	47214465	47239217	24752	15	96	117.530303	11.1579150<br>2	0.83652129<br>5	0.02103730<br>7	+	1643	grey	0.169190635	FALSE	DDB2
Region_2997	chr1<br>1	47239302	47248906	9604	5	19	36.9848484<br>8	6.95809970<br>6	0.96461625<br>6	0.03219963<br>4	-	53	grey	-0.14150154<br>6	FALSE	ACP2
Region_2998	chr1<br>1	47248300	47269033	20733	10	56	81.8030303	9.71631411<br>1	0.77672158<br>2	0.05268346<br>6	+	10062	grey	-0.12600397<br>9	FALSE	NR1H3
Region_2999	chr1<br>1	47269161	47330031	60870	45	283	327.348484<br>8	22.6046233<br>7	0.84372927<br>8	0.01989761	+	8567	grey	0.123750898	FALSE	MADD

Region_3000	chr11	47331406	47352702	21296	11	61	79.34848485	8.715137007	0.933222086	0.026207659	-	4607	grey	0.243756771	FALSE	MYBPC3
Region_3001	chr11	47354860	47409369	54509	51	307	357.5	23.85033462	0.520596112	0.030425981	-	6688	grey	-0.010797575	FALSE	SPI1
Region_3002	chr11	47407132	47416496	9364	11	60	79.8030303	10.23451129	0.63667423	0.051222173	+	91252	grey	-0.04534938	FALSE	SLC39A13
Region_3003	chr11	47418769	47426473	7704	10	50	67.34848485	7.370403233	0.590570536	0.021292731	-	5702	grey	-0.235212484	FALSE	PSMC3
Region_3004	chr11	47437764	47449143	11379	13	76	89.39393939	7.452162903	0.940560614	0.032820614	-	5913	grey	-0.036712986	FALSE	RAPSN
Region_3005	chr11	47465933	47565569	99636	29	172	206.5	16.06166003	0.776225406	0.021790446	-	10658	grey	0.056254273	FALSE	CELF1
Region_3006	chr11	47565336	47584562	19226	19	102	128.9242424	12.60806897	0.554054569	0.017321084	+	4722	grey	-0.018101452	FALSE	NDUFS3
Region_3007	chr11	47565430	47573461	8031	5	24	36.87878788	5.498293865	0.746844867	0.043469785	+	114971	grey	-0.097711945	FALSE	PTPMT1
Region_3008	chr11	47778087	47848555	70468	31	217	258.3333333	17.96948981	0.893902265	0.02167046	-	23279	grey	0.288691973	FALSE	NUP160
Region_3009	chr11	47980425	48170839	190414	54	379	430.0909091	22.19128538	0.81795071	0.020603848	+	5795	grey	-0.31691154	FALSE	PTPRJ
Region_3010	chr11	49145092	49208638	63546	4	19	31.68181818	5.640946704	0.650000684	0.059210363	-	2346	grey	0.364205261	FALSE	FOLH1
Region_3011	chr11	50258255	50298499	40244	10	57	79.68181818	11.12883862	0.865600823	0.052103452	-	441601	grey	0.068519249	FALSE	SEPTIN7P11
Region_3012	chr1	50260468	50298490	38022	10	57	79.6818181	11.1288386	0.86560082	0.05210345	-	10798433	grey	0.068519249	FALSE	

	1						8	2	3	2		0				
Region_3013	chr11	50298579	50361706	63127	10	75	94.45454545	10.3317015	0.715559733	0.06387965	+	105369150	grey	0.012435864	FALSE	LINC02750
Region_3014	chr11	57181747	57191717	9970	4	17	28.87878788	4.984638407	0.717960449	0.031270377	+	219527	grey	0.031384778	FALSE	LRRC55
Region_3015	chr11	57233577	57237250	3673	5	28	43.57575758	6.766570785	0.346511437	0.08074605	-	187	grey	0.043955044	FALSE	APLNR
Region_3016	chr11	57299638	57324952	25314	16	96	126.6515152	13.19031396	0.825331839	0.023933291	-	85456	grey	-0.33394436	FALSE	TNKS1BP1
Region_3017	chr11	57325986	57335892	9906	17	106	137.4848485	15.42848756	0.211381493	0.017206181	-	6749	grey	0.138369829	FALSE	SSRP1
Region_3018	chr11	57338352	57372396	34044	10	57	73.39393939	8.032393623	0.61361112	0.037907227	+	5024	grey	-0.087888609	FALSE	P2RX3
Region_3019	chr11	57386780	57424059	37279	10	64	89.87878788	11.87047423	0.837984377	0.032706048	-	5553	grey	0.109934923	FALSE	PRG2
Region_3020	chr11	57406954	57427580	20626	11	77	103.2575758	12.67257561	0.672077005	0.021873767	-	29015	grey	0.018890631	FALSE	SLC43A3
Region_3021	chr11	57460528	57477534	17006	8	30	49.36363636	8.058700723	0.751579249	0.04398207	+	349667	grey	-0.002022878	FALSE	RTN4RL2
Region_3022	chr11	57484534	57515780	31246	14	75	106.1060606	11.24705608	0.744612975	0.038943424	-	8501	grey	-0.044757503	FALSE	SLC43A1
Region_3023	chr11	57551656	57568284	16628	13	79	102.1060606	9.739892256	0.715621275	0.034494085	-	9246	grey	0.14843072	FALSE	UBE2L6
Region_3024	chr11	57597387	57619171	21784	5	21	36.01515152	6.615814211	0.798022441	0.070661474	+	710	grey	0.311396257	FALSE	SERPING1

Region_3025	chr11	57648188	57661865	13677	3	19	31.72727273	6.951580793	0.001683502	0.007900118	+	10978	grey	0.202654153	FALSE	CLP1
Region_3026	chr11	57667747	57701182	33435	5	25	39.24242424	6.058841235	0.957751113	0.028927425	+	25921	grey	-0.192790504	FALSE	ZDHHC5
Region_3027	chr11	57703710	57712221	8511	9	61	89.51515152	12.57254937	0.417106628	0.042465122	-	219541	grey	-0.156377966	FALSE	MED19
Region_3028	chr11	57712582	57818431	105849	21	131	166.2878788	15.08515842	0.927502082	0.018736169	+	100528016	grey	0.061070951	FALSE	TMX2-CTNND1
Region_3029	chr11	57712593	57740973	28380	9	45	70.84848485	10.10748759	0.973310186	0.020542257	+	51075	grey	-0.221819597	FALSE	TMX2
Region_3030	chr11	57743514	57752675	9161	3	14	24.33333333	4.881151609	0.972020706	0.033340579	-	643376	grey	-0.202594529	FALSE	BTBD18
Region_3031	chr11	57753243	57819546	66303	8	43	67.87878788	7.987519402	0.970143583	0.030089843	+	1500	grey	-0.067131259	FALSE	CTNND1
Region_3032	chr11	58023881	58181616	157735	8	57	76.51515152	9.834386179	0.84693492	0.053058641	+	219956	grey	-0.092244463	FALSE	OR9Q1
Region_3033	chr11	58526871	58578220	51349	3	13	25.27272727	4.840678448	0.94873765	0.052418791	-	9404	grey	-0.109669056	FALSE	LPXN
Region_3034	chr11	58640426	58731974	91548	9	49	65.22727273	7.910646097	0.795888613	0.049019998	-	10249	grey	0.0908687	FALSE	GLYAT
Region_3035	chr11	58834065	58904215	70150	4	10	23.77272727	5.252971187	0.919566362	0.054003085	-	219970	grey	0.160826774	FALSE	GLYATL2
Region_3036	chr11	58905398	59043527	138129	12	84	103.5757576	9.341488279	0.786675285	0.037750232	+	92292	grey	0.053244441	FALSE	GLYATL1
Region_3037	chr1	58933643	59058659	125016	11	80	98	9.25368949	0.77013751	0.03948260	-	283194	grey	0.056231164	FALSE	LOC283194

	1							3		1						
Region_3038	chr11	59171430	59208588	37158	3	20	31.45454545	5.519704056	0.905703955	0.066456933	+	23220	grey	-0.148450457	FALSE	DTX4
Region_3039	chr11	59208510	59212927	4417	3	18	27.1969697	5.831531523	0.896086558	0.084691005	-	219972	grey	-0.05360376	FALSE	MPEG1
Region_3040	chr11	59416969	59426413	9444	3	14	25.59090909	4.60092724	0.844147554	0.121904951	-	219981	grey	0.276084874	FALSE	OR5A2
Region_3041	chr11	59436469	59451380	14911	5	24	36.16666667	5.790133075	0.781223814	0.082867555	+	219982	grey	-0.242091364	FALSE	OR5A1
Region_3042	chr11	59545602	59558882	13280	8	51	68.74242424	8.220631842	0.64215827	0.03603678	+	102723575	grey	0.015361757	FALSE	LOC102723575
Region_3043	chr11	59560234	59566283	6049	5	32	44.90909091	6.126179064	0.009874297	0.018655718	-	105369317	grey	0.189549568	FALSE	LINC02739
Region_3044	chr11	59565923	59639861	73938	18	136	163.3333333	14.37447881	0.804418296	0.014099496	+	101927226	grey	0.004182277	FALSE	LOC101927226
Region_3045	chr11	59574398	59615774	41376	11	69	91.01515152	9.934388486	0.961443721	0.019593154	-	5007	grey	-0.160449994	FALSE	OSBP
Region_3046	chr11	59636716	59669037	32321	3	22	34.66666667	7.286094941	0.983350839	0.023975838	-	219988	grey	-0.159884862	FALSE	PATL1
Region_3047	chr11	59713456	59805882	92426	16	123	148.5909091	11.96144739	0.639672694	0.029394614	+	6809	grey	-0.186865638	FALSE	STX3
Region_3048	chr11	59806140	59810778	4638	4	26	34.92424242	6.187606984	0.008016939	0.016951492	-	54948	grey	0.053301513	FALSE	MRPL16
Region_3049	chr11	59829273	59845499	16226	3	13	23.06060606	4.120787034	0.969178703	0.035945041	-	2694	grey	0.149620073	FALSE	CBLIF

Region_3050	chr11	60185657	60318080	132423	13	68	95.33333333	9.917092212	0.94085093	0.038072485	+	51338	grey	-0.12641674	FALSE	MS4A4A
Region_3051	chr11	60200270	60243137	42867	5	23	37.62121212	7.345883518	0.952962358	0.041153761	-	643680	grey	-0.182540748	FALSE	MS4A4E
Region_3052	chr11	60327255	60396596	69341	11	70	92.5	9.287294878	0.741138282	0.033852962	+	245802	grey	0.103206281	FALSE	MS4A6E
Region_3053	chr11	60378485	60395951	17466	6	37	48.83333333	6.02196833	0.712413223	0.051173214	+	58475	grey	0.131761674	FALSE	MS4A7
Region_3054	chr11	60378530	60417756	39226	10	63	81.39393939	8.775628468	0.81304901	0.034855496	+	84689	grey	0.220069915	FALSE	MS4A14
Region_3055	chr11	60429572	60455214	25642	3	13	23.07575758	4.225601845	0.871018821	0.127024757	+	64232	grey	0.201606255	FALSE	MS4A5
Region_3056	chr11	60515392	60542721	27329	7	54	75.27272727	8.74694752	0.897122527	0.046149637	+	503497	grey	-0.094529915	FALSE	MS4A13
Region_3057	chr11	60615729	60687267	71538	8	60	77.40909091	8.236835226	0.874653622	0.043286557	+	283197	grey	0.063216514	FALSE	LINC00301
Region_3058	chr11	60699585	60715807	16222	8	51	66.03030303	7.477082312	0.760780667	0.041456379	+	83661	grey	0.056397376	FALSE	MS4A8
Region_3059	chr11	60756867	60776733	19866	22	153	174.5151515	14.14892486	0.715832811	0.029448593	+	219995	grey	-0.000521167	FALSE	MS4A15
Region_3060	chr11	60785333	60801305	15972	6	37	49.63636364	6.401048865	0.916881755	0.061590577	+	341116	grey	-0.049024848	FALSE	MS4A10
Region_3061	chr11	60842113	60851081	8968	8	43	59.12121212	7.195116189	0.899414666	0.03556925	+	79080	grey	0.074460087	FALSE	CCDC86
Region_3062	chr1	60850933	60855950	5017	3	14	25.4242424	5.07197611	0.94835952	0.04660272	-	11251	grey	-0.24700126	FALSE	PTGDR2

	1						2	6	8	5				3		
Region_3063	chr11	60867542	60875693	8151	6	34	50.04545455	6.32560564	0.592078788	0.058152045	+	22917	grey	0.087145147	FALSE	ZP1
Region_3064	chr11	60890547	60906585	16038	11	79	97.04545455	9.246091595	0.696716136	0.034565469	-	27339	grey	0.052800375	FALSE	PRPF19
Region_3065	chr11	60914158	60923443	9285	5	35	48.6969697	7.573170654	0.553056513	0.034102762	+	79073	grey	0.080359799	FALSE	TMEM109
Region_3066	chr11	60924460	60937159	12699	24	120	149.8787879	13.77457541	0.793622501	0.019666795	+	54972	grey	-0.123230491	FALSE	TMEM132A
Region_3067	chr11	60937060	60952653	15593	13	61	89.95454545	10.76951798	0.803295336	0.03834662	-	51296	grey	-0.217201483	FALSE	SLC15A3
Region_3068	chr11	60971680	61020377	48697	22	146	179.7727273	12.05601727	0.787637499	0.035479813	+	923	grey	0.149946692	FALSE	CD6
Region_3069	chr11	61102489	61127852	25363	19	150	183.7727273	14.01185811	0.903115375	0.022781074	+	921	grey	0.081059692	FALSE	CD5
Region_3070	chr11	61130257	61161615	31358	22	149	187.7272727	16.15648996	0.955108289	0.017278819	-	55048	grey	-0.137522581	FALSE	VPS37C
Region_3071	chr11	61203307	61213098	9791	4	11	37	14.6013698	0.895783892	0.070989091	+	643834	grey	0.203347028	FALSE	PGA3
Region_3072	chr11	61258286	61295316	37030	38	241	284.7272727	17.52057731	0.800515437	0.016375704	-	220001	grey	-0.038737334	FALSE	VWCE
Region_3073	chr11	61299451	61342596	43145	26	164	208.5909091	17.30004649	0.898392302	0.014958943	-	1642	grey	0.262907919	FALSE	DDB1
Region_3074	chr11	61333220	61353295	20075	16	101	135.7575758	15.23059735	0.738398416	0.040588641	+	26007	grey	0.010256727	FALSE	TKFC

Region_3075	chr11	61348754	61362283	13529	14	92	123.2727273	11.90674722	0.459849035	0.028678405	-	220002	grey	-0.029102279	FALSE	CYB561A3
Region_3076	chr11	61361964	61377890	15926	16	112	140.7727273	11.64975464	0.375255931	0.014510452	+	51524	grey	-0.092254385	FALSE	TMEM138
Region_3077	chr11	61402641	61430031	27390	14	103	122.8484848	10.4030209	0.646639821	0.027754171	-	79869	grey	0.095389401	FALSE	CPSF7
Region_3078	chr11	61429220	61485822	56602	40	242	298.3636364	18.6049095	0.852257977	0.01594593	+	54949	grey	-0.096508674	FALSE	SDHAF2
Region_3079	chr11	61430126	61490931	60805	40	238	297.8787879	19.33948941	0.912160059	0.018869955	+	220004	grey	-0.066136461	FALSE	SAXO4
Region_3080	chr11	61496440	61506649	10209	5	25	35.09090909	5.419983743	0.824812998	0.095056315	+	105369329	grey	0.320785556	FALSE	LOC105369329
Region_3081	chr11	61508749	61511018	2269	7	36	59.06060606	9.299923553	0.269443679	0.039794014	+	390205	grey	-0.182886871	FALSE	LRRC10B
Region_3082	chr11	61513714	61581148	67434	52	303	370.530303	24.21329558	0.827983129	0.023310765	-	9066	grey	0.007194325	FALSE	SYT7
Region_3083	chr11	61588442	61607550	19108	13	66	89.36363636	11.01416761	0.831343086	0.048935639	+	101927495	grey	-0.092337851	FALSE	LOC101927495
Region_3084	chr11	61615036	61639449	24413	16	82	112.2575758	12.00296195	0.869954836	0.035570126	+	113157	grey	-0.138420881	FALSE	RPLP0P2
Region_3085	chr11	61680391	61747001	66610	54	355	407.1060606	26.14523271	0.749261784	0.022138212	+	747	grey	0.076198357	FALSE	DAGLA
Region_3086	chr11	61752636	61788518	35882	27	144	176.9545455	15.58147995	0.877809033	0.029655821	+	745	grey	-0.25159177	FALSE	MYRF
Region_3087	chr1	61768501	61792802	24301	19	107	133.545454	13.3349532	0.88449961	0.03331831	-	746	grey	-0.21874820	FALSE	TMEM258

	1						5	8	6	8				6		
Region_3088	chr11	61792911	61826512	33601	13	103	123.1212121	8.491652284	0.893909221	0.0263341	+	2237	grey	0.017342196	FALSE	FEN1
Region_3089	chr11	61799627	61829318	29691	13	99	119.2121212	10.75820281	0.670442382	0.015176276	-	3992	grey	0.062457286	FALSE	FADS1
Region_3090	chr11	61816256	61867354	51098	20	123	156.1666667	12.89911545	0.703660507	0.021622591	+	9415	grey	-0.049584223	FALSE	FADS2
Region_3091	chr11	61873519	61892051	18532	19	84	112.8333333	12.78049395	0.445213214	0.032408599	-	3995	grey	-0.349912452	FALSE	FADS3
Region_3092	chr11	61897301	61920269	22968	23	122	144.5151515	13.35346964	0.665269914	0.028383672	-	5866	grey	0.229351225	FALSE	RAB3IL1
Region_3093	chr11	61950063	61965515	15452	5	23	34.21212121	5.800570666	0.887427312	0.048912301	+	7439	grey	0.063193239	FALSE	BEST1
Region_3094	chr11	61959718	61967634	7916	3	14	22.60606061	4.703121019	0.422553457	0.07547704	-	2495	grey	-0.148566102	FALSE	FTH1
Region_3095	chr11	62049863	62082184	32321	7	35	52.81818182	6.989902808	0.938586518	0.034519829	-	100507521	grey	0.087883274	FALSE	LINC02733
Region_3096	chr11	62123998	62153169	29171	18	111	143.7575758	11.12593727	0.767229776	0.017750128	+	3619	grey	0.14203441	FALSE	INCENP
Region_3097	chr11	62242239	62244812	2573	3	15	28.13636364	5.182474501	0.586744337	0.151683772	+	10647	grey	0.33743094	FALSE	SCGB1D2
Region_3098	chr11	62337448	62393412	55964	48	355	398.9242424	19.6737315	0.785602141	0.016911673	+	80150	grey	0.093372204	FALSE	ASRGL1
Region_3099	chr11	62405103	62423195	18092	12	73	90.42424242	10.66563223	0.955953291	0.022575591	+	7356	grey	0.043835339	FALSE	SCGB1A1

Region_3100	chr11	62433542	62556235	122693	106	738	808.7878788	34.14742479	0.809085379	0.011764871	-	79026	grey	-0.143798444	FALSE	AHNAK
Region_3101	chr11	62559596	62577321	17725	8	50	68.3030303	8.335225892	0.685821444	0.043322655	-	1937	grey	-0.30041928	FALSE	EEF1G
Region_3102	chr11	62559603	62591637	32034	14	97	121.530303	10.33175791	0.740055747	0.026264786	-	64852	grey	-0.236526442	FALSE	TUT1
Region_3103	chr11	62593214	62601865	8651	9	46	61.22727273	7.234097582	0.380513804	0.035280915	-	9219	grey	0.124207998	FALSE	MTA2
Region_3104	chr11	62602218	62612775	10557	20	107	132.969697	12.97568104	0.808412346	0.019541684	-	256364	grey	0.101837094	FALSE	EML3
Region_3105	chr11	62611722	62615116	3394	5	15	30.90909091	6.947555791	0.235433576	0.040432643	+	6094	grey	-0.03891193	FALSE	ROM1
Region_3106	chr11	62624826	62646726	21900	14	80	107.8484848	11.58413029	0.52481553	0.037490077	-	23193	grey	-0.078247045	FALSE	GANAB
Region_3107	chr11	62646848	62665178	18330	20	127	156.1212121	12.65946305	0.801374796	0.017936676	-	80789	grey	0.022514556	FALSE	INTS5
Region_3108	chr11	62662817	62672267	9450	8	36	53.84848485	7.882292568	0.391540838	0.037602447	-	79081	grey	0.005151052	FALSE	LBHD1
Region_3109	chr11	62689289	62709845	20556	24	154	174.8787879	12.63878664	0.733172541	0.018453514	-	26580	grey	-0.107379534	FALSE	BSCL2
Region_3110	chr11	62690275	62727384	37109	32	205	234.3181818	14.96679308	0.733380756	0.020792907	-	100534595	grey	-0.167357387	FALSE	HNRNPUL2-BSCL2
Region_3111	chr11	62712630	62727457	14827	7	38	52.16666667	7.525512164	0.836397375	0.060596229	-	221092	grey	-0.152304077	FALSE	HNRNPUL2
Region_3112	chr1	62728069	62740284	12215	7	40	55.9242424	7.43652235	0.82105659	0.02453841	+	283237	grey	-0.13538055	FALSE	TTC9C

	1						2	9	2					8		
Region_3113	chr11	62771357	62787342	15985	12	73	90.66666667	9.364964714	0.793669674	0.024505166	+	10629	grey	0.218091779	FALSE	TAF6L
Region_3114	chr11	62771629	62792006	20377	16	106	125.1363636	11.27950676	0.694809707	0.022811973	-	79064	grey	0.33594457	FALSE	TMEM223
Region_3115	chr11	62787402	62790400	2998	3	13	25.83333333	5.520823053	0.529727911	0.067742022	+	374395	grey	0.258986424	FALSE	TMEM179B
Region_3116	chr11	62792123	62806302	14179	6	43	57.56060606	6.693576227	0.637159741	0.057990662	-	10482	grey	0.069529852	FALSE	NXF1
Region_3117	chr11	62806860	62832051	25191	7	32	48.22727273	7.081493659	0.917870239	0.043101794	-	6811	grey	0.137821182	FALSE	STX5
Region_3118	chr11	62832911	62841809	8898	5	34	54.15151515	7.923174236	0.357035167	0.033066717	-	54663	grey	-0.227461044	FALSE	WDR74
Region_3119	chr11	62856004	62888880	32876	4	13	26.1969697	5.455328227	0.71436117	0.035363541	+	6520	grey	-0.095697374	FALSE	SLC3A2
Region_3120	chr11	62936385	62984967	48582	7	48	60.51515152	6.244363426	0.824850834	0.032390188	-	9356	grey	-0.24673439	FALSE	SLC22A6
Region_3121	chr11	62989154	63015841	26687	6	44	64.92424242	8.228114292	0.962141264	0.026570698	-	9376	grey	0.021800384	FALSE	SLC22A8
Region_3122	chr11	63079940	63144221	64281	6	30	47.75757576	6.631070469	0.895710632	0.061216801	-	283238	grey	-0.12817778	FALSE	SLC22A24
Region_3123	chr11	63158437	63243599	85162	5	30	47.21212121	6.808400132	0.886136603	0.060745789	-	387601	grey	0.235882504	FALSE	SLC22A25
Region_3124	chr11	63268022	63311783	43761	6	45	60.75757576	7.206963739	0.947802454	0.031894204	+	387775	grey	-0.17177717	FALSE	SLC22A10

Region_3125	chr11	63369785	63410294	40509	5	39	51.57575758	5.665171886	0.783623884	0.061278332	+	114571	grey	0.220118983	FALSE	SLC22A9
Region_3126	chr11	63461404	63491194	29790	4	18	29.66666667	5.186768175	0.508828013	0.051986676	-	117245	grey	0.218948909	FALSE	PLAAT5
Region_3127	chr11	63506052	63516774	10722	4	19	29.16666667	5.365368532	0.933888078	0.045567176	+	85329	grey	-0.144324358	FALSE	LGALS12
Region_3128	chr11	63536808	63546462	9654	3	15	28.04545455	5.842633279	0.0199929	0.028956288	+	5920	grey	-0.059094931	FALSE	PLAAT4
Region_3129	chr11	63552770	63563379	10609	4	18	26.84848485	4.265216828	0.9507809	0.056530947	-	54979	grey	0.029585213	FALSE	PLAAT2
Region_3130	chr11	63573195	63616883	43688	38	261	306.2727273	22.7816022	0.784127065	0.016564973	-	11145	grey	0.382200376	FALSE	PLAAT3
Region_3131	chr11	63616308	63658962	42654	8	47	63.16666667	7.385189614	0.901688032	0.044003208	+	107984336	grey	-0.018401008	FALSE	
Region_3132	chr11	63624087	63671921	47834	22	130	161.1818182	15.5631805	0.502079838	0.020540932	-	25923	grey	0.19508963	FALSE	ATL3
Region_3133	chr11	63681446	63759891	78445	51	345	398.1212121	23.30038966	0.903804858	0.012559064	+	10313	grey	0.182520974	FALSE	RTN3
Region_3134	chr11	63759892	63768775	8883	20	71	104.6212121	12.97780964	0.531123927	0.033039791	-	65998	grey	-0.20550321	FALSE	ZFTA
Region_3135	chr11	63813456	63827716	14260	14	77	97.46969697	10.27651497	0.678268407	0.022490681	+	144097	grey	0.071672173	FALSE	SPINDOC
Region_3136	chr11	63838928	63911020	72092	49	321	373.0757576	25.54110449	0.722144935	0.015769163	+	2011	grey	-0.036481309	FALSE	MARK2
Region_3137	chr1	63911230	63917164	5934	14	62	78.0303030	10.1420217	0.32339472	0.02719119	-	283248	grey	-0.04257199	FALSE	RCOR2

	1						3	9	7	6				1		
Region_3138	chr11	63938959	63957319	18360	32	169	200.8030303	16.24828483	0.537284596	0.029275087	+	79829	grey	-0.317097808	FALSE	NAA40
Region_3139	chr11	63974620	63988346	13726	20	129	161.8181818	12.44669754	0.556271918	0.028737404	+	1351	grey	0.142099156	FALSE	COX8A
Region_3140	chr11	63985853	64001811	15958	17	85	111.8787879	11.41593374	0.600430086	0.034508618	+	55611	grey	-0.056353635	FALSE	OTUB1
Region_3141	chr11	63998558	64166113	167555	213	1304	1468.136364	65.63884553	0.804186065	0.01112687	-	28992	grey	0.228658827	FALSE	MACROD1
Region_3142	chr11	64035931	64119173	83242	119	733	838.1666667	41.11357004	0.800506338	0.015577086	+	23769	grey	0.222639196	FALSE	FLRT1
Region_3143	chr11	64185272	64204543	19271	17	110	134.0909091	12.64391282	0.665488512	0.02055843	+	10963	grey	0.085133507	FALSE	STIP1
Region_3144	chr11	64205926	64223896	17970	19	97	123.8030303	10.88290077	0.852054249	0.031672377	+	83706	grey	-0.227106643	FALSE	FERMT3
Region_3145	chr11	64225941	64230686	4745	4	15	27.63636364	5.479012723	0.006443903	0.015238157	+	84304	grey	-0.015625706	FALSE	NUDT22
Region_3146	chr11	64234584	64239264	4680	4	17	28.36363636	5.947556353	0.31619501	0.055651985	+	7423	grey	0.214655888	FALSE	VEGFB
Region_3147	chr11	64241003	64244132	3129	4	13	21.83333333	4.248378273	0.29216293	0.055940842	+	2286	grey	-0.120996504	FALSE	FKBP2
Region_3148	chr11	64241095	64244132	3037	4	13	21.83333333	4.248378273	0.29216293	0.055940842	+	114841035	grey	-0.120996504	FALSE	LOC114841035
Region_3149	chr11	64244479	64246943	2464	5	22	36.10606061	6.276032034	0.123872218	0.065095633	-	26472	grey	-0.005419248	FALSE	PPP1R14B

Region_3150	chr11	64245838	64248221	2383	3	12	23.31818182	5.105529705	0.008496239	0.02674054	+	105369340	grey	-0.15319907	FALSE	PPP1R14B-AS1
Region_3151	chr11	64251530	64269150	17620	23	124	156.9090909	12.67429533	0.863646294	0.021813388	+	5331	grey	-0.034148305	FALSE	PLCB3
Region_3152	chr11	64269830	64284704	14874	16	112	143.7424242	13.19010189	0.664901517	0.012992582	-	572	grey	0.225081277	FALSE	BAD
Region_3153	chr11	64270062	64289500	19438	21	126	175.1666667	15.21394435	0.684080679	0.014387542	+	56834	grey	0.191745925	FALSE	GPR137
Region_3154	chr11	64291302	64300031	8729	6	25	37	6.532757693	0.304835519	0.063950031	+	50801	grey	0.084781516	FALSE	KCNK4
Region_3155	chr11	64291704	64304769	13065	13	74	96.3030303	9.979933246	0.322745189	0.038592399	+	106780802	grey	-0.029392733	FALSE	KCNK4-CATSPERZ
Region_3156	chr11	64300358	64304770	4412	7	46	59.3030303	7.084044253	0.338155057	0.041599483	+	25858	grey	-0.167223573	FALSE	CATSPERZ
Region_3157	chr11	64305497	64316743	11246	15	82	107.8333333	12.3495043	0.553677475	0.025604538	+	2101	grey	-0.155225944	FALSE	ESRRA
Region_3158	chr11	64318121	64321811	3690	5	25	45.27272727	9.707325473	0.005953056	0.015091725	+	25824	grey	0.213646979	FALSE	PRDX5
Region_3159	chr11	64325050	64340154	15104	16	86	116.8636364	11.88908532	0.824482278	0.033245948	-	102723878	grey	0.109871183	FALSE	LOC102723878
Region_3160	chr11	64340204	64357534	17330	13	70	92.18181818	9.572250103	0.798561684	0.036801451	+	283234	grey	-0.132891578	FALSE	CCDC88B
Region_3161	chr11	64359148	64372215	13067	13	65	82.13636364	9.238525296	0.518590313	0.033036347	+	8986	grey	0.102449276	FALSE	RPS6KA4
Region_3162	chr1	64555690	64572875	17185	26	159	199.5	18.4892900	0.88485784	0.02793294	+	55867	grey	0.07997544	FALSE	SLC22A11

	1							4	6	5						
Region_3163	chr1<br>1	64590641	64602353	11712	17	99	123.772727<br>3	15.2434555<br>5	0.90015016<br>7	0.02788165	+	116085	grey	0.030657236	FALSE	SLC22A12
Region_3164	chr1<br>1	64606174	64723197	117023	77	456	519.712121<br>2	25.0714457	0.70538907<br>9	0.01336072<br>9	-	9379	grey	-0.10419231<br>6	FALSE	NRXN2
Region_3165	chr1<br>1	64646399	64659681	13282	10	49	64	7.61981223<br>3	0.83476573<br>9	0.04627751<br>7	+	10798433<br>7	grey	-0.34833631<br>3	FALSE	NRXN2-AS1
Region_3166	chr1<br>1	64726911	64745456	18545	16	84	106.560606<br>1	9.75252044<br>4	0.74502949<br>4	0.03771137<br>4	-	10235	grey	-0.09991565<br>6	FALSE	RASGRP2
Region_3167	chr1<br>1	64746389	64759974	13585	9	43	71.6515151<br>5	9.32901581<br>8	0.92544999<br>7	0.04150430<br>6	-	5837	grey	0.155530808	FALSE	PYGM
Region_3168	chr1<br>1	64764606	64778786	14180	3	13	22.5303030<br>3	4.23981021<br>7	0.52082987<br>1	0.07411553	-	7536	grey	-0.14861823<br>3	FALSE	SF1
Region_3169	chr1<br>1	64784918	64803241	18323	22	123	159.530303	14.2226468<br>3	0.74532093<br>5	0.02455912<br>7	-	5871	grey	0.186044298	FALSE	MAP4K2
Region_3170	chr1<br>1	64803510	64811294	7784	17	83	111.575757<br>6	12.5625335<br>6	0.58753182<br>1	0.03221332<br>9	-	4221	grey	0.300310887	FALSE	MEN1
Region_3171	chr1<br>1	64823052	64844653	21601	13	66	85.0757575<br>8	9.09321066<br>4	0.81223910<br>6	0.03391742<br>7	-	55561	grey	0.124824451	FALSE	CDC42BPG
Region_3172	chr1<br>1	64851642	64888296	36654	54	340	404.287878<br>8	25.8681658<br>5	0.52787684<br>9	0.02039799<br>7	-	10938	grey	0.146658642	FALSE	EHD1
Region_3173	chr1<br>1	64894546	64917211	22665	28	162	202.424242<br>4	18.6630532<br>6	0.92023082<br>7	0.01750801<br>4	-	23130	grey	0.008560728	FALSE	ATG2A
Region_3174	chr1<br>1	64917553	64934475	16922	12	69	88.7878787<br>9	8.81696817<br>2	0.94444966<br>2	0.03023337<br>1	+	5526	grey	-0.19419333<br>7	FALSE	PPP2R5B

Region_3175	chr11	64937517	64972108	34591	19	107	139.4545455	13.80432688	0.899501461	0.02409912	-	283129	grey	-0.144724255	FALSE	MAJIN
Region_3176	chr11	64987945	64997018	9073	7	36	53.37878788	8.022593388	0.886264007	0.053782935	-	116071	grey	-0.046246848	FALSE	BATF2
Region_3177	chr11	65014160	65022184	8024	5	24	38.60606061	6.881142096	0.582086393	0.025102741	+	402	grey	-0.24007775	FALSE	ARL2
Region_3178	chr11	65014182	65040570	26388	16	94	117.4090909	10.30754357	0.761250228	0.025159656	+	100528018	grey	-0.274527926	FALSE	ARL2-SNX15
Region_3179	chr11	65027439	65040572	13133	10	55	72.21212121	7.36836304	0.834728956	0.034419503	+	29907	grey	-0.373068898	FALSE	SNX15
Region_3180	chr11	65040901	65044828	3927	5	23	33.59090909	5.560789299	0.267973558	0.047654592	+	29901	grey	0.028544469	FALSE	SAC3D1
Region_3181	chr11	65044818	65058553	13735	9	47	66.60606061	7.783372391	0.805895451	0.05362066	-	10004	grey	0.054605204	FALSE	NAALADL1
Region_3182	chr11	65066300	65084164	17864	16	95	115.9545455	10.71509621	0.659705012	0.023373747	-	113130	grey	0.041850669	FALSE	CDCA5
Region_3183	chr11	65089324	65111862	22538	20	107	147.6666667	15.4634391	0.732057087	0.025294036	+	738	grey	-0.114206671	FALSE	VPS51
Region_3184	chr11	65116403	65117701	1298	8	57	80.83333333	14.06313603	0.021489577	0.019036399	-	741	grey	-0.183838661	FALSE	ZNHIT2
Region_3185	chr11	65120630	65122177	1547	5	27	49.53030303	8.942929647	0.005821031	0.010792566	-	2197	grey	-0.08597732	FALSE	FAU
Region_3186	chr11	65121780	65134532	12752	26	161	216.4393939	22.30152033	0.259673296	0.019948272	-	84447	grey	0.345772977	FALSE	SYVN1
Region_3187	chr1	65122183	65127371	5188	20	123	175.712121	20.3301709	0.25721290	0.01795845	+	740	grey	0.254456858	FALSE	MRPL49

	1						2	5	4							
Region_3188	chr11	65170233	65173374	3141	4	12	24.60606061	4.939258082	0.962251532	0.040480776	+	387778	grey	0.051960347	FALSE	SPDYC
Region_3189	chr11	65180566	65212006	31440	11	60	74.51515152	7.985067649	0.602454524	0.047538594	+	823	grey	0.125992334	FALSE	CAPN1
Region_3190	chr11	65213840	65242757	28917	23	126	159.8030303	14.23129779	0.647938982	0.026598151	+	440044	grey	-0.193688934	FALSE	SLC22A20P
Region_3191	chr11	65261920	65326543	64623	65	411	476.6515152	26.23062944	0.772003324	0.014935196	+	23649	grey	0.093328576	FALSE	POLA2
Region_3192	chr11	65305345	65314737	9392	17	87	118.8030303	14.40108938	0.718423858	0.027030193	-	105369344	grey	0.216234418	FALSE	LOC105369344
Region_3193	chr11	65314866	65322417	7551	15	70	92.48484848	12.56398078	0.691980201	0.029802571	+	10435	grey	-0.017860305	FALSE	CDC42EP2
Region_3194	chr11	65333852	65354262	20410	22	136	164.2878788	13.81834361	0.862159478	0.021200718	+	5977	grey	-0.115047869	FALSE	DPF2
Region_3195	chr11	65354751	65357613	2862	5	18	31.77272727	7.029160043	0.40633668	0.058457945	+	220359	grey	0.207433075	FALSE	TIGD3
Region_3196	chr11	65375192	65383701	8509	13	55	81.56060606	9.773006447	0.374139865	0.019229826	-	283130	grey	0.024234955	FALSE	SLC25A45
Region_3197	chr11	65386621	65413525	26904	28	166	197.469697	15.43045909	0.913929498	0.019181054	+	83786	grey	-0.037906459	FALSE	FRMD8
Region_3198	chr11	65422798	65445540	22742	17	115	142.9545455	14.82869549	0.343663952	0.022809523	+	283131	grey	0.087601643	FALSE	NEAT1
Region_3199	chr11	65455257	65466720	11463	3	11	20.87878788	4.956782925	0.973894077	0.045301945	+	101927789	grey	-0.188760766	FALSE	FAUP4

Region_3200	chr11	65487241	65488714	1473	6	30	47.21212121	8.545831465	0.132854518	0.06523955	-	105369346	grey	-0.018575497	FALSE	LINC02736
Region_3201	chr11	65525077	65538704	13627	11	57	83.54545455	8.013799288	0.740822521	0.030143722	+	57410	grey	0.01640386	FALSE	SCYL1
Region_3202	chr11	65538559	65558930	20371	44	245	291.6515152	22.51187203	0.595810424	0.041065326	-	4054	grey	-0.170623724	FALSE	LTBP3
Region_3203	chr11	65568482	65570458	1976	5	27	42.33333333	7.273414886	0.002835914	0.007528215	-	254100	grey	0.249631988	FALSE	ZNRD2-DT
Region_3204	chr11	65570460	65572892	2432	3	11	22.42424242	4.896028551	0.002661718	0.012726853	+	10534	grey	0.020576625	FALSE	ZNRD2
Region_3205	chr11	65570682	65574198	3516	4	10	24.84848485	5.74922717	0.054727144	0.048588046	+	23625	grey	-0.09122729	FALSE	FAM89B
Region_3206	chr11	65576046	65592650	16604	24	85	134.5454545	20.2098086	0.822491639	0.033685827	+	254102	grey	0.131436148	FALSE	EHBP1L1
Region_3207	chr11	65597756	65615382	17626	14	78	98.59090909	9.652384756	0.826644664	0.02322721	-	4296	grey	-0.216748375	FALSE	MAP3K11
Region_3208	chr11	65615776	65637439	21663	33	179	227.5909091	21.5577773	0.896997791	0.021813088	+	399909	grey	0.053431459	FALSE	PCNX3
Region_3209	chr11	65638101	65650918	12817	19	99	128.6060606	11.21520779	0.539796775	0.026903003	+	6494	grey	-0.073999354	FALSE	SIPA1
Region_3210	chr11	65653599	65663090	9491	14	61	91.54545455	11.71873164	0.849250419	0.030536683	-	5970	grey	0.092858586	FALSE	RELA
Region_3211	chr11	65662988	65671718	8730	12	76	97.78787879	10.62003209	0.882322239	0.026534731	+	105369347	grey	0.073218566	FALSE	RELA-DT
Region_3212	chr1	65711996	65719604	7608	4	14	28.2121212	5.14262773	0.24353559	0.03340634	+	10524	grey	0.020099174	FALSE	KAT5

	1						1	8	2	9						
Region_3213	chr11	65714005	65720818	6813	3	16	24.01515152	4.287701282	0.313044587	0.029774605	-	84153	grey	0.101171541	FALSE	RNASEH2C
Region_3214	chr11	65773898	65780976	7078	18	117	144.969697	13.24441211	0.475923268	0.046153218	-	91056	grey	-0.018906766	FALSE	AP5B1
Region_3215	chr11	65787063	65797214	10151	16	96	122.530303	12.67489305	0.454756631	0.033196977	+	5017	grey	-0.109315971	FALSE	OVOL1
Region_3216	chr11	65823022	65862026	39004	30	204	236.5757576	17.09346308	0.562284244	0.019073244	-	1072	grey	0.116402561	FALSE	CFL1
Region_3217	chr11	65833834	65856896	23062	16	107	130.2727273	12.59492627	0.81233405	0.030794509	+	254122	grey	0.087994545	FALSE	SNX32
Region_3218	chr11	65857126	65867653	10527	12	58	82.63636364	10.64914061	0.229554305	0.024392943	+	80198	grey	0.111433287	FALSE	MUS81
Region_3219	chr11	65866441	65873592	7151	5	20	31.86363636	5.229756175	0.732890161	0.04963107	-	30008	grey	0.393649406	FALSE	EFEMP2
Region_3220	chr11	65879809	65883741	3932	3	15	23.56060606	4.235849884	0.940799502	0.0515036	+	1521	grey	-0.014729303	FALSE	CTSW
Region_3221	chr11	65890673	65891635	962	7	41	61.87878788	9.355401815	0.004235939	0.008049822	+	11007	grey	-0.028560065	FALSE	CCDC85B
Region_3222	chr11	65892049	65900573	8524	7	25	38.78787879	6.936004506	0.55941905	0.032311336	-	8061	grey	-0.169175783	FALSE	FOSL1
Region_3223	chr11	65945480	65959966	14486	6	28	40.31818182	5.987574547	0.954738857	0.039825816	+	254187	grey	0.210604939	FALSE	TSGA10IP
Region_3224	chr11	65961728	65980137	18409	13	48	70.3030303	8.490664008	0.863862642	0.030970299	+	9092	grey	0.104130203	FALSE	SART1

Region_3225	chr11	65996545	66002176	5631	4	19	30.06060606	4.991928217	0.448956347	0.055327267	-	84285	grey	-0.08993385	FALSE	EIF1AD
Region_3226	chr11	66002228	66004149	1921	5	16	28.89393939	5.144463156	0.769926504	0.035603991	+	8815	grey	-0.115424757	FALSE	BANF1
Region_3227	chr11	66016752	66026479	9727	20	112	156.2575758	16.0837334	0.941671036	0.023201683	-	117144	grey	-0.179081937	FALSE	CATSPER1
Region_3228	chr11	66040765	66049161	8396	10	51	77.59090909	9.887019109	0.757239052	0.037928375	-	89792	grey	0.205915798	FALSE	GAL3ST3
Region_3229	chr11	66052331	66069308	16977	20	135	166.5	12.06680125	0.820122703	0.018139117	+	10992	grey	-0.232318138	FALSE	SF3B2
Region_3230	chr11	66070272	66244744	174472	101	704	782.5	31.80142716	0.835417889	0.014812682	+	55690	grey	-0.244262897	FALSE	PACS1
Region_3231	chr11	66257294	66267860	10566	15	79	102.9242424	10.95769572	0.582493496	0.024255619	+	64837	grey	-0.016963939	FALSE	KLC2
Region_3232	chr11	66259567	66265666	6099	6	23	38.21212121	7.451412156	0.955976534	0.036337024	-	105369352	grey	-0.027684859	FALSE	KLC2-AS1
Region_3233	chr11	66268590	66277492	8902	6	31	53.8030303	7.423910844	0.627528934	0.031392399	+	81876	grey	-0.106090139	FALSE	RAB1B
Region_3234	chr11	66269832	66278525	8693	14	56	78.56060606	12.27270742	0.279550949	0.020464296	-	107984340	grey	-0.281984477	FALSE	LOC107984340
Region_3235	chr11	66284580	66289145	4565	7	34	49.03030303	8.08982209	0.279160842	0.016855048	-	10897	grey	0.054760515	FALSE	YIF1A
Region_3236	chr11	66291894	66296664	4770	7	26	47.46969697	10.12114268	0.825212415	0.089861721	+	256472	grey	-0.225833588	FALSE	TMEM151A
Region_3237	chr1	66330241	66336840	6599	13	60	84.9696969	11.2180756	0.29532176	0.03867059	-	9610	grey	0.208892363	FALSE	RIN1

	1						7	6	3	7						
Region_3238	chr11	66337333	66345125	7792	7	38	61.90909091	7.78702525	0.83418607	0.023114813	-	25855	grey	0.32917791	FALSE	BRMS1
Region_3239	chr11	66345374	66347629	2255	9	52	71.66666667	9.363321786	0.326946363	0.023278376	-	11041	grey	-0.052668292	FALSE	B4GAT1
Region_3240	chr11	66347950	66364190	16240	13	83	107.7878788	11.48445793	0.903334269	0.03325722	+	102724064	grey	-0.021771578	FALSE	B4GAT1-DT
Region_3241	chr11	66362521	66372214	9693	6	21	33.03030303	5.519112797	0.112556583	0.046064267	-	3177	grey	-0.131166669	FALSE	SLC29A2
Region_3242	chr11	66409158	66426707	17549	10	44	84.40909091	10.64096189	0.309726446	0.041343746	+	266743	grey	-0.297666431	FALSE	NPAS4
Region_3243	chr11	66435075	66466738	31663	17	112	137.1363636	12.17549413	0.767317458	0.025523618	-	65003	grey	-0.138068226	FALSE	MRPL11
Region_3244	chr11	66466327	66477337	11010	15	87	119.1969697	12.27908753	0.900460205	0.021985677	+	246330	grey	0.183025825	FALSE	PELI3
Region_3245	chr11	66473490	66480296	6806	9	53	76.66666667	11.54034173	0.535140237	0.02115451	-	101928069	grey	-0.229533872	FALSE	DPP3-DT
Region_3246	chr11	66480013	66509657	29644	16	108	133.6818182	11.94494948	0.94681682	0.025779699	+	10072	grey	0.282534659	FALSE	DPP3
Region_3247	chr11	66509079	66533613	24534	17	99	130.1212121	11.69025255	0.85389958	0.020166675	+	582	grey	0.049631877	FALSE	BBS1
Region_3248	chr11	66520637	66546235	25598	15	91	112.5909091	10.19347796	0.724346992	0.044247791	-	254359	grey	0.070054742	FALSE	ZDHHC24
Region_3249	chr11	66546395	66563334	16939	10	56	73.06060606	8.966396734	0.74489204	0.039571204	+	89	grey	-0.17639226	FALSE	ACTN3

Region_3250	chr11	66590176	66593063	2887	3	17	26.59090909	4.905184926	0.620282656	0.041454008	-	55231	grey	-0.025579025	FALSE	CCDC87
Region_3251	chr11	66593153	66606019	12866	8	45	63.46969697	7.502245196	0.843263832	0.05157585	+	9973	grey	-0.191078854	FALSE	CCS
Region_3252	chr11	66616626	66629934	13308	35	226	275.1060606	18.34802759	0.716557416	0.020522528	+	10432	grey	-0.006673505	FALSE	RBM14
Region_3253	chr11	66616626	66646469	29843	55	379	430.030303	20.28411254	0.670922819	0.015535134	+	100526737	grey	0.008061104	FALSE	RBM14-RBM4
Region_3254	chr11	66638667	66668374	29707	29	188	214.2727273	14.55973949	0.800856479	0.014060936	+	5936	grey	-0.258987277	FALSE	RBM4
Region_3255	chr11	66664998	66677887	12889	10	60	79.68181818	9.121671728	0.513355862	0.030912668	-	83759	grey	0.052278178	FALSE	RBM4B
Region_3256	chr11	66682497	66744670	62173	51	342	406.5909091	24.42191782	0.811536924	0.018705167	-	6712	grey	0.129296751	FALSE	SPTBN2
Region_3257	chr11	66744451	66843516	99065	52	379	422.7575758	21.49136543	0.889123535	0.013947833	+	79703	grey	0.254353213	FALSE	TOP6BL
Region_3258	chr11	66842835	66846552	3717	4	19	31.71212121	5.74527271	0.249685156	0.042296162	+	9986	grey	-0.016784905	FALSE	RCE1
Region_3259	chr11	66848417	66958386	109969	85	535	640.9090909	36.91058938	0.760936952	0.012989775	-	5091	grey	-0.005787159	FALSE	PC
Region_3260	chr11	66856647	66860475	3828	15	49	83.37878788	12.63422111	0.616088686	0.034428181	+	78999	grey	-0.11464774	FALSE	LRFN4
Region_3261	chr11	67006778	67050863	44085	50	344	394.3636364	20.03891319	0.70671494	0.02158653	+	91683	grey	0.212143787	FALSE	SYT12
Region_3262	chr1	67056847	67072017	15170	28	176	219.272727	19.6292916	0.74915363	0.03065580	+	29984	grey	0.098929339	FALSE	RHOD

	1						3	6		5						
Region_3263	chr11	67119263	67258082	138819	106	731	828.6212121	38.61994907	0.841113335	0.007083562	+	22992	grey	0.033777992	FALSE	KDM2A
Region_3264	chr11	67266473	67286556	20083	23	145	183.969697	16.83400377	0.244118233	0.033135365	+	156	grey	-0.206299514	FALSE	GRK2
Region_3265	chr11	67289300	67302485	13185	11	55	72.65151515	9.627672335	0.656628184	0.045757835	+	338692	grey	-0.229305018	FALSE	ANKRD13D
Region_3266	chr11	67303478	67312605	9127	7	34	52.53030303	8.055995755	0.862338933	0.043186583	+	54961	grey	-0.071716503	FALSE	SSH3
Region_3267	chr11	67317871	67392074	74203	56	358	411.530303	25.51031146	0.691656789	0.018498064	+	100130987	grey	0.101510082	FALSE	LOC100130987
Region_3268	chr11	67350772	67373584	22812	18	92	118.9242424	12.47805533	0.607807122	0.030781643	-	57804	grey	0.162315769	FALSE	POLD4
Region_3269	chr11	67364168	67374177	10009	4	21	33.13636364	5.961121122	0.664824886	0.086789081	-	23529	grey	0.22647935	FALSE	CLCF1
Region_3270	chr11	67391705	67398410	6705	13	66	88.39393939	9.854134295	0.623753462	0.03994104	+	5883	grey	-0.072294623	FALSE	RAD9A
Region_3271	chr11	67398181	67421183	23002	23	118	145.2878788	14.56419757	0.319559752	0.022312323	-	5499	grey	0.189408408	FALSE	PPP1CA
Region_3272	chr11	67403915	67410089	6174	6	28	42.10606061	6.2858297	0.28046471	0.054438558	+	374403	grey	0.156403236	FALSE	TBC1D10C
Region_3273	chr11	67415361	67425607	10246	7	27	39.07575758	7.036849396	0.393127843	0.039680521	+	57571	grey	0.216690043	FALSE	CARNS1
Region_3274	chr11	67428460	67435401	6941	9	49	65.5	7.872542352	0.714615316	0.071978776	+	6199	grey	0.296481992	FALSE	RPS6KB2

Region_3275	chr11	67435510	67443821	8311	13	61	81.06060606	10.20388883	0.497120506	0.050512182	-	57175	grey	-0.014421742	FALSE	CORO1B
Region_3276	chr11	67452406	67461752	9346	6	26	40.12121212	6.451034864	0.860362205	0.05815176	+	57010	grey	0.093320459	FALSE	CABP4
Region_3277	chr11	67461710	67469272	7562	6	24	41.63636364	7.078975075	0.875513472	0.051987428	-	80194	grey	-0.11694344	FALSE	TMEM134
Region_3278	chr11	67468174	67491154	22980	20	103	131.2121212	11.8172171	0.780426909	0.037480549	+	9049	grey	-0.140412403	FALSE	AIP
Region_3279	chr11	67491768	67506263	14495	20	92	120.6666667	13.07414752	0.739932198	0.022580903	-	9600	grey	-0.091118059	FALSE	PITPNM1
Region_3280	chr11	67506497	67508649	2152	5	25	37.06060606	5.305668342	0.515739448	0.058352879	-	10263	grey	0.080308754	FALSE	CDK2AP2
Region_3281	chr11	67605653	67612554	6901	8	39	58.37878788	8.151951445	0.608393709	0.03372526	+	4723	grey	0.197205001	FALSE	NDUFV1
Region_3282	chr11	67612652	67616257	3605	5	22	34.53030303	5.381505929	0.952486802	0.032502462	-	390213	grey	0.018870643	FALSE	DOC2GP
Region_3283	chr11	67627938	67629937	1999	5	28	41.53030303	5.746489757	0.386673052	0.027630505	-	254552	grey	-0.118702436	FALSE	NUDT8
Region_3284	chr11	67662155	67681224	19069	6	27	42.10606061	6.809786595	0.755418102	0.046770349	-	222	grey	0.159679149	FALSE	ALDH3B2
Region_3285	chr11	67735600	67784229	48629	39	276	315.3787879	18.55118918	0.843599467	0.020067306	-	105369358	grey	-0.11885858	FALSE	LOC105369358
Region_3286	chr11	67791648	67805336	13688	14	88	116.1969697	12.35029703	0.769588633	0.033116646	-	645332	grey	0.285706536	FALSE	FAM86C2P
Region_3287	chr1	67886477	67906350	19873	14	78	102.454545	11.9912555	0.88039402	0.04285439	+	10536936	grey	0.108346152	FALSE	LINC02754

	1						5	6	8	5		0				
Region_3288	chr11	67934563	67955802	21239	7	28	46.21212121	8.621110318	0.538742419	0.038435641	-	112268076	grey	-0.141772347	FALSE	LOC112268076
Region_3289	chr11	67991100	68004982	13882	13	70	88.8030303	11.31264738	0.696842115	0.041566924	-	81622	grey	0.249117704	FALSE	UNC93B1
Region_3290	chr11	68008578	68029282	20704	10	64	90.22727273	9.661653712	0.790167646	0.030283835	+	221	grey	0.218913471	FALSE	ALDH3B1
Region_3291	chr11	68030617	68036644	6027	6	32	48.57575758	7.183443798	0.664683546	0.034005613	+	4728	grey	0.156870614	FALSE	NDUFS8
Region_3292	chr11	68039025	68053694	14669	14	78	103.6969697	12.5133402	0.943237413	0.026401853	+	10312	grey	-0.041017338	FALSE	TCIRG1
Region_3293	chr11	68052859	68121444	68585	40	276	324.6363636	21.69766834	0.803362461	0.013674077	-	1119	grey	-0.086510825	FALSE	CHKA
Region_3294	chr11	68121624	68130518	8894	12	57	74.39393939	9.010897314	0.633377067	0.026427377	+	105369362	grey	0.197637433	FALSE	CHKA-DT
Region_3295	chr11	68154863	68213852	58989	33	231	280.6212121	17.57600213	0.836972235	0.017268623	-	51111	grey	-0.051658051	FALSE	KMT5B
Region_3296	chr11	68261338	68272001	10663	4	19	30.63636364	5.48743001	0.489899878	0.050039227	-	53838	grey	0.018826755	FALSE	C11orf24
Region_3297	chr11	68272100	68284884	12784	7	34	53.03030303	6.713865554	0.767414629	0.059743243	+	105369363	grey	-0.159842947	FALSE	LOC105369363
Region_3298	chr11	68312591	68449275	136684	100	645	750.5	37.52301858	0.85747608	0.009722737	+	4041	grey	0.142812803	FALSE	LRP5
Region_3299	chr11	68460731	68615334	154603	52	363	419.5909091	22.31589794	0.829875495	0.019728446	+	55291	grey	0.223218555	FALSE	PPP6R3

Region_3300	chr11	68684544	68691175	6631	7	31	44.5	5.863183698	0.46685625	0.040067121	+	51083	grey	0.122287209	FALSE	GAL
Region_3301	chr11	68707440	68751520	44080	24	187	224.5606061	12.43089054	0.854084469	0.026545355	-	9633	grey	0.094566756	FALSE	TESMIN
Region_3302	chr11	68754620	68844410	89790	95	674	739.8030303	31.1152793	0.743347242	0.014407406	-	1374	grey	-0.066281128	FALSE	CPT1A
Region_3303	chr11	68891276	68903835	12559	6	43	54.92424242	6.735235947	0.753515775	0.088385837	-	219927	grey	0.020360716	FALSE	MRPL21
Region_3304	chr11	68903863	68940602	36739	26	172	208.3939394	15.02703081	0.82736155	0.0202372	+	3508	grey	-0.129877183	FALSE	IGHMBP2
Region_3305	chr11	69004395	69013382	8987	4	20	32.74242424	4.862131872	0.944197394	0.050093399	-	116535	grey	0.01977365	FALSE	MRGPRF
Region_3306	chr11	69012283	69018447	6164	3	12	20.21212121	4.211760646	0.945804546	0.063230764	+	101928200	grey	-0.137071784	FALSE	MRGPRF-AS1
Region_3307	chr11	69048932	69136316	87384	37	237	279.7424242	19.44088523	0.822849452	0.021843563	+	219931	grey	0.140262289	FALSE	TPCN2
Region_3308	chr11	69072915	69162440	89525	20	156	182.3787879	12.86350252	0.785942521	0.036482467	+	107984345	grey	-0.054558889	FALSE	SMIM38
Region_3309	chr11	69147228	69171564	24336	9	59	73.51515152	7.365705197	0.918684717	0.034956344	-	338694	grey	0.174167822	FALSE	LOC338694
Region_3310	chr11	69294151	69341682	47531	22	135	159.469697	13.15378822	0.683144039	0.029931693	+	26579	grey	-0.326798486	FALSE	MYEOV
Region_3311	chr11	69337360	69367726	30366	12	84	99.28787879	8.310818069	0.933047839	0.032107288	+	105369370	grey	-0.000652735	FALSE	LOC105369370
Region_3312	chr1	69425678	69429621	3943	4	22	32.8484848	5.56152282	0.84132971	0.05852610	+	10272426	grey	-0.02079056	FALSE	LINC02953

	1						5	5	9	5		5		2		
Region_3313	chr11	69475567	69481545	5978	10	56	89.72727273	10.03462537	0.943946657	0.023942862	-	105379407	grey	0.032715476	FALSE	LINC02747
Region_3314	chr11	69481662	69494952	13290	9	48	67.78787879	8.640717833	0.880396461	0.036422036	+	101928292	grey	0.194917945	FALSE	LINC01488
Region_3315	chr11	69641156	69654474	13318	17	76	97.60606061	9.367503219	0.822176293	0.027259338	+	595	grey	-0.261565092	FALSE	CCND1
Region_3316	chr11	69653076	69675416	22340	13	78	103.9393939	11.25218446	0.885581278	0.019643503	-	220064	grey	-0.421104821	FALSE	LTO1
Region_3317	chr11	69698238	69704022	5784	8	46	65.77272727	10.6291261	0.403369521	0.039921669	-	9965	grey	0.049236166	FALSE	FGF19
Region_3318	chr11	69809968	69819416	9448	13	58	85.48484848	11.37371115	0.410362216	0.044876584	-	2248	grey	-0.040685078	FALSE	FGF3
Region_3319	chr11	69985876	70189530	203654	175	1154	1316.666667	53.60750043	0.761546538	0.014666455	+	55107	grey	0.057168626	FALSE	ANO1
Region_3320	chr11	70056230	70065371	9141	5	26	39.22727273	6.382175791	0.928568691	0.052967439	-	101928443	grey	-0.078879956	FALSE	LINC02753
Region_3321	chr11	70203296	70207390	4094	6	28	47.90909091	6.323891871	0.443248972	0.04169477	+	8772	grey	-7.20617E-05	FALSE	FADD
Region_3322	chr11	70467854	71252577	784723	418	3185	3437	120.1737204	0.844534181	0.010902202	-	22941	grey	0.16275225	FALSE	SHANK2
Region_3323	chr11	70626441	70635658	9217	5	27	37.90909091	5.790515515	0.83805466	0.068652452	+	100874198	grey	-0.194277515	FALSE	SHANK2-AS1
Region_3324	chr11	71405746	71423423	17677	3	15	27.90909091	4.646677134	0.87529168	0.074612513	-	399923	grey	-0.060544707	FALSE	ACTE1P

Region_3325	chr11	71428193	71452868	24675	11	64	85.3030303	8.580782996	0.270029561	0.01136565	-	1717	grey	-0.075483351	FALSE	DHCR7
Region_3326	chr11	71453109	71524107	70998	55	372	441.1060606	28.16988506	0.841973515	0.016202852	+	55191	grey	-0.216604612	FALSE	NADSYN1
Region_3327	chr11	71673885	71813859	139974	40	274	339.9393939	24.53247693	0.822593237	0.021585762	-	285407	grey	-0.068519908	FALSE	ALG1L9P
Region_3328	chr11	71787480	71804995	17515	10	81	99.92424242	11.72415076	0.846290408	0.023997796	+	55199	grey	-0.015309318	FALSE	FAM86C1P
Region_3329	chr11	71804997	71928656	123659	30	196	224.8939394	14.58991094	0.86279093	0.02447385	-	122319435	grey	-0.157107407	FALSE	XNDC1N-ZNF705EP-ALG1L9P
Region_3330	chr11	71807591	71821548	13957	4	18	28.43939394	6.079293451	0.485951741	0.046859936	-	100131539	grey	-0.072377286	FALSE	ZNF705EP
Region_3331	chr11	71865504	71928624	63120	20	125	147.3787879	11.05338814	0.94785232	0.022505822	-	100133315	grey	-0.010193511	FALSE	XNDC1N
Region_3332	chr11	71929018	71997597	68579	30	167	209.5151515	12.62140115	0.926631494	0.023302955	+	55298	grey	-0.019208295	FALSE	RNF121
Region_3333	chr11	71998613	72007315	8702	3	14	20.98484848	3.222480766	0.970192252	0.043780003	+	10068	grey	0.031942664	FALSE	IL18BP
Region_3334	chr11	72002864	72080693	77829	21	131	152.9848485	10.57354089	0.881723877	0.019834996	-	4926	grey	-0.087555313	FALSE	NUMA1
Region_3335	chr11	72080331	72110782	30451	5	30	42.43939394	6.246546832	0.390371991	0.016270278	+	220074	grey	-0.232906508	FALSE	LRTOMT
Region_3336	chr11	72080337	72096895	16558	3	14	21.6969697	4.624249451	0.324791566	0.023082535	+	120356739	grey	-0.21781922	FALSE	LRRC51
Region_3337	chr1	72114869	72139892	25023	13	82	107.045454	9.43713095	0.69471098	0.04438893	+	2352	grey	0.177899826	FALSE	FOLR3

	1						5		2	6						
Region_3338	chr11	72239077	72245664	6587	5	15	28.46969697	5.624724805	0.121617227	0.064834553	-	401	grey	0.239424023	FALSE	PHOX2A
Region_3339	chr11	72285495	72434680	149185	47	351	399.6666667	22.03796258	0.877536745	0.015632513	-	81570	grey	-0.242073658	FALSE	CLPB
Region_3340	chr11	72351347	72363555	12208	5	20	31.10606061	5.406915196	0.904103514	0.054489489	+	112268077	grey	-0.087926911	FALSE	LOC112268077
Region_3341	chr11	72530155	72535038	4883	5	16	30.28787879	8.397371661	0.922789088	0.055811713	-	105369376	grey	-0.105309551	FALSE	LOC105369376
Region_3342	chr11	72576141	72674591	98450	16	83	114.6818182	11.98866014	0.730131654	0.034428999	-	5138	grey	0.013127291	FALSE	PDE2A
Region_3343	chr11	72685069	72793599	108530	14	79	97.83333333	7.802530819	0.797467122	0.032391052	-	116985	grey	0.1092126	FALSE	ARAP1
Region_3344	chr11	72754729	72794047	39318	4	16	28.21212121	4.881724637	0.777227923	0.064987594	-	10809	grey	-0.104799881	FALSE	STARD10
Region_3345	chr11	72814406	72843674	29268	13	80	104.1363636	11.12984396	0.850303999	0.033586738	+	89849	grey	-0.119132182	FALSE	ATG16L2
Region_3346	chr11	72836745	73142318	305573	72	556	600.6060606	23.48084574	0.898747699	0.014747781	-	9873	grey	0.332174227	FALSE	FCHSD2
Region_3347	chr11	73218281	73242427	24146	6	23	46.46969697	8.394539795	0.903280186	0.047542118	+	5029	grey	0.155978006	FALSE	P2RY2
Region_3348	chr11	73264498	73305103	40605	5	35	48.09090909	6.289740786	0.70704739	0.089929621	+	5031	grey	-0.024845734	FALSE	P2RY6
Region_3349	chr11	73307235	73309361	2126	18	64	110.8181818	19.98992753	0.009324049	0.01150894	-	100287837	grey	0.032113051	FALSE	ARHGEF17-AS1

Region_3350	chr11	73308276	73369388	61112	50	260	334.4545455	31.85311569	0.491345323	0.015993662	+	9828	grey	-0.146028611	FALSE	ARHGEF17
Region_3351	chr11	73376399	73397474	21075	11	59	85.65151515	11.27487667	0.663806961	0.051132873	+	84957	grey	0.122470137	FALSE	RELT
Region_3352	chr11	73400487	73598189	197702	29	227	251.9393939	13.22454453	0.932338232	0.023721585	-	23201	grey	0.116219401	FALSE	FAM168A
Region_3353	chr11	73646178	73662819	16641	10	58	77.24242424	9.827842092	0.5892614	0.042951072	+	58473	grey	-0.163309202	FALSE	PLEKHB1
Region_3354	chr11	73675638	73761137	85499	23	150	189.3333333	15.04182204	0.864781479	0.01694391	-	5870	grey	0.312463658	FALSE	RAB6A
Region_3355	chr11	73787872	73865133	77261	19	116	135.8181818	12.0357277	0.969995509	0.019042566	+	51642	grey	0.090474771	FALSE	MRPL48
Region_3356	chr11	73876699	73931114	54415	9	52	64.75757576	6.759746482	0.864841449	0.023610522	+	80227	grey	-0.002259666	FALSE	PAAF1
Region_3357	chr11	73951026	73970366	19340	5	29	37.87878788	5.660973419	0.780027733	0.061402559	+	374407	grey	-0.021393384	FALSE	DNAJB13
Region_3358	chr11	74000277	74009085	8808	6	30	50.84848485	8.213014858	0.943111628	0.039057365	-	7352	grey	0.024435578	FALSE	UCP3
Region_3359	chr11	74012718	74171210	158492	18	112	143.6818182	12.11122695	0.844349519	0.016891253	-	26005	grey	-0.189434302	FALSE	C2CD3
Region_3360	chr11	74171267	74254703	83436	5	33	45.33333333	5.794781029	0.976345447	0.027189838	+	51400	grey	0.032091168	FALSE	PPME1
Region_3361	chr11	74247487	74311640	64153	13	87	112.3636364	10.93144563	0.800640775	0.032640244	-	283208	grey	-0.110316993	FALSE	P4HA3
Region_3362	chr1	74330316	74398433	68117	8	44	67.2272727	7.94557536	0.64117607	0.02812066	-	283209	grey	-0.07083742	FALSE	PGM2L1

	1						3	2		1				8		
Region_3363	chr11	74454841	74467729	12888	4	18	30.07575758	5.257406808	0.516791438	0.113275707	-	10008	grey	-0.296713771	FALSE	KCNE3
Region_3364	chr11	74493851	74669117	175266	30	211	265.6515152	16.80154977	0.851243393	0.029806255	+	10714	grey	0.081854545	FALSE	POLD3
Region_3365	chr11	74696429	74731426	34997	7	33	52	7.50794451	0.780008504	0.058173569	-	25884	grey	0.369926649	FALSE	CHRDL2
Region_3366	chr11	74748849	74842413	93564	12	79	107.4393939	9.606294071	0.886563202	0.017629149	+	254225	grey	0.067200593	FALSE	RNF169
Region_3367	chr11	74807739	74949200	141461	22	156	184.4090909	13.95705201	0.886908797	0.01976688	-	143570	grey	0.01077565	FALSE	XRRA1
Region_3368	chr11	74949261	74979033	29772	10	69	94.8030303	9.577569464	0.851200708	0.029746895	+	9789	grey	-0.352807859	FALSE	SPCS2
Region_3369	chr11	75070512	75096964	26452	3	18	27.45454545	4.500194246	0.930341187	0.04890114	-	341152	grey	0.218694735	FALSE	OR2AT4
Region_3370	chr11	75099172	75140148	40976	17	108	135.5	12.64333208	0.843562131	0.034345756	-	107984358	grey	0.231910422	FALSE	LOC107984358
Region_3371	chr11	75100563	75206549	105986	41	264	301.7424242	19.63537671	0.725054232	0.030359939	+	11309	grey	0.388695815	FALSE	SLCO2B1
Region_3372	chr11	75206048	75241173	35125	13	68	88.48484848	10.48257517	0.755715671	0.042968905	-	105369388	grey	0.284497155	FALSE	TPBGL-AS1
Region_3373	chr11	75240774	75243704	2930	7	23	43.5	9.322016949	0.027906867	0.036638841	+	100507050	grey	0.119237841	FALSE	TPBGL
Region_3374	chr11	75260122	75351705	91583	31	206	249.2878788	15.61731303	0.874313341	0.026895624	-	408	grey	0.077247266	FALSE	ARRB1

Region_3375	chr11	75399515	75422280	22765	11	80	104.1818182	10.37883807	0.85827344	0.023532724	+	6188	grey	-0.098049145	FALSE	RPS3
Region_3376	chr11	75422394	75433203	10809	5	18	33.42424242	5.849551215	0.465620356	0.060085862	-	283212	grey	0.264653303	FALSE	KLHL35
Region_3377	chr11	75434640	75525941	91301	39	238	283.6818182	18.44506112	0.62559833	0.020273821	-	81544	grey	0.083952436	FALSE	GDPD5
Region_3378	chr11	75583196	75594665	11469	5	11	32.72727273	6.622910015	0.682803102	0.073623561	+	105369391	grey	-0.115720844	FALSE	LOC105369391
Region_3379	chr11	75586918	75669038	82120	18	114	144.8333333	14.66506402	0.745733597	0.027167121	-	4135	grey	-0.262609719	FALSE	MAP6
Region_3380	chr11	75717838	75732958	15120	4	19	35.09090909	6.588398364	0.887441927	0.055102871	+	80168	grey	-0.076353746	FALSE	MOGAT2
Region_3381	chr11	75759512	75801535	42023	7	42	56.66666667	6.479157907	0.95398337	0.03716593	+	84649	grey	0.186712632	FALSE	DGAT2
Region_3382	chr11	75815210	76144232	329022	64	423	488.0454545	22.87183812	0.861918335	0.015061199	+	7405	grey	-0.359429466	FALSE	UVRAG
Region_3383	chr11	76186325	76210736	24411	11	62	80.89393939	8.017536142	0.777568522	0.026596376	-	7481	grey	0.092014302	FALSE	WNT11
Region_3384	chr11	76349898	76381132	31234	11	66	81.07575758	9.303945575	0.872708702	0.023493473	-	5612	grey	-0.027882758	FALSE	THAP12
Region_3385	chr11	76381303	76414619	33316	14	113	145.8787879	15.22701562	0.292549445	0.020263566	+	100506127	grey	0.172363614	FALSE	GVQW3
Region_3386	chr11	76444923	76553031	108108	20	125	154.1212121	13.44563429	0.823315573	0.016372398	+	56946	grey	0.165566855	FALSE	EMSY
Region_3387	chr1	76657524	76670747	13223	3	14	23.5909090	4.62427465	0.90192578	0.07232155	-	2615	grey	-0.11844292	FALSE	LRRC32

	1						9	5	2	4				9		
Region_3388	chr11	76694041	76719801	25760	5	23	34.03030303	5.686609635	0.864333327	0.07097116	-	390226	grey	0.027886478	FALSE	GUCY2EP
Region_3389	chr11	76782251	76798153	15902	14	88	119.469697	14.06820714	0.816881339	0.023566585	+	25987	grey	0.076456951	FALSE	TSKU
Region_3390	chr11	76860859	77026797	165938	24	177	201.5454545	14.08128252	0.908086999	0.021813228	+	55331	grey	0.033968298	FALSE	ACER3
Region_3391	chr11	77034398	77041973	7575	5	20	42.57575758	7.797555053	0.429522482	0.083174514	+	192134	grey	-0.163367848	FALSE	B3GNT6
Region_3392	chr11	77066961	77126155	59194	7	40	54.74242424	6.631650464	0.663276751	0.047120188	+	726	grey	-0.008252991	FALSE	CAPN5
Region_3393	chr11	77128246	77215241	86995	24	140	182.3181818	18.876244	0.914664597	0.025607822	+	4647	grey	-0.148487164	FALSE	MYO7A
Region_3394	chr11	77216558	77301687	85129	18	110	136.6818182	11.7462388	0.886525614	0.026555515	-	220032	grey	0.117602439	FALSE	GDPD4
Region_3395	chr11	77322017	77474635	152618	24	181	207.0454545	14.30484783	0.885903643	0.021593557	-	5058	grey	0.056841147	FALSE	PAK1
Region_3396	chr11	77514936	77637794	122858	31	220	253.1515152	18.20417739	0.80131363	0.017769078	-	1207	grey	-0.257629489	FALSE	CLNS1A
Region_3397	chr11	77589391	77610356	20965	9	45	66.28787879	8.446692847	0.624614608	0.034158883	+	282679	grey	-0.339985011	FALSE	AQP11
Region_3398	chr11	77660009	77872262	212253	46	290	340.9848485	20.37380854	0.893215551	0.013053097	-	51773	grey	0.150199848	FALSE	RSF1
Region_3399	chr11	77821109	77918432	97323	23	133	167.3030303	16.1835336	0.872914284	0.020303216	+	28971	grey	0.207531981	FALSE	AAMDC

Region_3400	chr11	77874418	77994715	120297	24	125	167.5	16.23410465	0.928574416	0.023496517	-	92105	grey	-0.306460987	FALSE	INTS4
Region_3401	chr11	78015715	78046191	30476	4	14	24.74242424	4.521097913	0.963591228	0.045590595	-	65987	grey	-0.418921031	FALSE	KCTD14
Region_3402	chr11	78016971	78079865	62894	18	95	121.0151515	12.87871521	0.675819002	0.035257477	-	100532726	grey	-0.030975194	FALSE	NDUFC2-KCTD14
Region_3403	chr11	78068297	78079862	11565	6	28	41.87878788	7.600637932	0.406474307	0.088341929	-	4718	grey	0.23067089	FALSE	NDUFC2
Region_3404	chr11	78095244	78139660	44416	8	40	56.1969697	7.575709557	0.973065552	0.025023068	-	79053	grey	-0.040504135	FALSE	ALG8
Region_3405	chr11	78139756	78175307	35551	8	46	62.42424242	6.879312591	0.937111401	0.036034099	+	100289388	grey	-0.017933161	FALSE	KCTD21-AS1
Region_3406	chr11	78188812	78225909	37097	12	87	103.1666667	9.552419481	0.745863033	0.032544184	+	57558	grey	-0.163733446	FALSE	USP35
Region_3407	chr11	78215293	78418348	203055	34	204	260.8484848	15.58622905	0.759786268	0.016468415	-	9846	grey	0.22594607	FALSE	GAB2
Region_3408	chr11	78435620	78575194	139574	24	163	200.0909091	15.65466485	0.884037622	0.028671694	-	79731	grey	0.032903415	FALSE	NARS2
Region_3409	chr11	78533176	78558565	25389	7	39	53.37878788	6.41096453	0.932367757	0.038498855	+	101928896	grey	0.2494613	FALSE	LOC101928896
Region_3410	chr11	78652829	79441030	788201	106	872	946.1363636	30.70849863	0.772847009	0.015740403	-	26011	grey	0.060553334	FALSE	TENM4
Region_3411	chr11	81821272	82718082	896810	67	496	545.3636364	25.37754087	0.812800284	0.021872831	-	101928989	grey	-0.019497382	FALSE	MIR4300HG
Region_3412	chr1	82781502	82817741	36239	6	35	52.5909090	8.63263445	0.80413946	0.08377861	+	10536941	grey	0.41521872	FALSE	LINC02734

	1						9	4	2	5		3				
Region_3413	chr11	82822936	82970584	147648	20	124	159.5151515	14.08134874	0.69254064	0.030156679	-	5547	grey	0.203108759	FALSE	PRCP
Region_3414	chr11	82899975	82958277	58302	8	45	65.95454545	8.653024048	0.44299862	0.047570915	+	220042	grey	0.32222033	FALSE	DDIAS
Region_3415	chr11	82973133	83071923	98790	15	116	139.469697	12.3577689	0.845690033	0.032888241	-	27314	grey	0.478541554	FALSE	RAB30
Region_3416	chr11	83072052	83107789	35737	4	22	34.33333333	5.321461145	0.735055584	0.027773144	+	100506233	grey	0.032278873	FALSE	RAB30-DT
Region_3417	chr11	83156988	83187451	30463	8	45	61.77272727	7.414736791	0.81713283	0.033916057	+	51585	grey	-0.175285247	FALSE	PCF11
Region_3418	chr11	83193712	83260694	66982	6	42	54.60606061	6.679195686	0.778869288	0.033787277	+	338699	grey	-0.201303786	FALSE	ANKRD42
Region_3419	chr11	83259081	83286390	27309	4	26	39.45454545	5.907444634	0.70904071	0.037579695	-	60492	grey	-0.163629692	FALSE	CCDC90B
Region_3420	chr11	83455012	85627922	2172910	140	1152	1220.393939	35.858216	0.85173301	0.012330873	-	1740	grey	-0.158130288	FALSE	DLG2
Region_3421	chr11	83643602	83725390	81788	5	35	52.77272727	7.46436757	0.814927038	0.045259791	+	101929043	grey	-0.102620171	FALSE	DLG2-AS2
Region_3422	chr11	85657742	85682908	25166	3	16	25.60606061	4.317687372	0.154505109	0.065804148	-	58487	grey	-0.157079989	FALSE	CREBZF
Region_3423	chr11	85694224	85811159	116935	12	71	89.22727273	9.233376671	0.568797347	0.046605196	-	54843	grey	0.309390297	FALSE	SYTL2
Region_3424	chr11	85855101	85920021	64920	5	26	37.5	5.583629919	0.394092011	0.0235824	+	220047	grey	-0.323720106	FALSE	CCDC83

Region_3425	chr11	85957175	86069882	112707	13	83	114.5	13.89105966	0.711045799	0.030348773	-	8301	grey	0.006509427	FALSE	PICALM
Region_3426	chr11	86302211	86345943	43732	3	21	30.89393939	4.564507852	0.9744248	0.027167931	+	51501	grey	-0.043117693	FALSE	HIKESHI
Region_3427	chr11	86374736	86423109	48373	10	63	81.63636364	9.584661406	0.916187802	0.043613267	+	60494	grey	-0.400825923	FALSE	CCDC81
Region_3428	chr11	86441108	86672636	231528	32	233	274.1212121	16.44993818	0.761997538	0.021498689	-	10873	grey	-0.143497594	FALSE	ME3
Region_3429	chr11	86727355	86765467	38112	6	27	45.24242424	8.26868161	0.914040215	0.044635772	+	102724775	grey	-0.025387999	FALSE	LOC102724775
Region_3430	chr11	86791059	86952910	161851	16	96	125.9090909	12.12845005	0.919294554	0.02438174	+	11098	grey	0.03033023	FALSE	PRSS23
Region_3431	chr11	86892214	86922104	29890	4	21	30.84848485	5.295113638	0.930206922	0.05863257	-	107984429	grey	-0.037727572	FALSE	PRSS23-AS1
Region_3432	chr11	86955616	87021909	66293	10	45	64.77272727	8.146345013	0.622771747	0.040206968	+	100506368	grey	0.32227611	FALSE	FZD4-DT
Region_3433	chr11	87037844	87328824	290980	22	129	157.6515152	13.76896446	0.891594503	0.030800936	+	65084	grey	-0.072649625	FALSE	TMEM135
Region_3434	chr11	87847259	88175443	328184	23	158	187.8030303	12.09350709	0.818913825	0.029948854	-	23682	grey	-0.370749681	FALSE	RAB38
Region_3435	chr11	88265069	88359679	94610	6	36	59.89393939	6.843590573	0.77725749	0.030056042	-	1075	grey	0.123720327	FALSE	CTSC
Region_3436	chr11	88337839	88428548	90709	5	36	49.75757576	7.051591432	0.737109793	0.033408771	+	101929174	grey	-0.069672037	FALSE	LOC101929174
Region_3437	chr1	88504576	89065982	561406	26	172	209.939393	15.9998834	0.77511516	0.03366457	-	2915	grey	0.044820749	FALSE	GRM5

	1						9	5	8	4						
Region_3438	chr11	89177875	89295759	117884	4	22	37.90909091	6.095969091	0.797677973	0.076386937	+	7299	grey	-0.02323061	FALSE	TYR
Region_3439	chr11	89324353	89589611	265258	13	96	128.3484848	10.32322613	0.675108561	0.050492057	-	50507	grey	0.268236948	FALSE	NOX4
Region_3440	chr11	89639237	89698718	59481	7	38	50.07575758	7.264901092	0.825465828	0.053713062	+	219595	grey	0.133817137	FALSE	FOLH1B
Region_3441	chr11	89766440	89808575	42135	6	11	32.89393939	10.74620332	0.656662635	0.07735026	-	57093	grey	-0.233216096	FALSE	TRIM49
Region_3442	chr11	90251204	90915052	663848	31	199	235.5909091	16.6723883	0.859466301	0.027882131	+	101929222	grey	0.089309477	FALSE	DISC1FP1
Region_3443	chr11	91794319	91872086	77767	4	23	33.01515152	5.151526807	0.838714053	0.07080928	+	105369428	grey	0.235467371	FALSE	LINC02756
Region_3444	chr11	92224818	92896473	671655	43	331	384	20.65690423	0.84208039	0.024412196	+	120114	grey	-0.273661477	FALSE	FAT3
Region_3445	chr11	92969651	92985066	15415	3	18	27.40909091	5.161923532	0.685833401	0.06145015	+	4544	grey	0.14530332	FALSE	MTNR1B
Region_3446	chr11	93144174	93197991	53817	7	46	67.04545455	8.884613871	0.752857599	0.067464041	-	120103	grey	-0.23373066	FALSE	SLC36A4
Region_3447	chr11	93329971	93438487	108516	9	55	72.53030303	8.94808909	0.755488652	0.042425863	+	159989	grey	-0.186846609	FALSE	DEUP1
Region_3448	chr11	93478472	93543391	64919	10	59	77.92424242	8.380032322	0.734384105	0.044120946	-	56935	grey	0.029131998	FALSE	SMCO4
Region_3449	chr11	93661682	93730358	68676	9	52	72.40909091	8.552061871	0.832999021	0.086467272	+	85459	grey	0.061038879	FALSE	CEP295

Region_3450	chr11	93729948	93784391	54443	11	56	81.16666667	9.922132732	0.734789925	0.053621961	-	79101	grey	0.036311772	FALSE	TAF1D
Region_3451	chr11	93741664	93814963	73299	11	58	82.95454545	9.771909219	0.932792956	0.045370963	+	9440	grey	-0.149747553	FALSE	MED17
Region_3452	chr11	93818232	93850618	32386	9	64	81.96969697	8.544849455	0.859148442	0.025008513	-	387804	grey	-0.112122799	FALSE	VSTM5
Region_3453	chr11	94021354	94114208	92854	8	51	72.36363636	8.420919671	0.798706501	0.064484921	+	341208	grey	-0.08771842	FALSE	HEPHL1
Region_3454	chr11	94128841	94181968	53127	9	70	89.89393939	9.198877503	0.517800454	0.028284078	+	24145	grey	0.430077011	FALSE	PANX1
Region_3455	chr11	94185439	94282203	96764	11	75	100.2272727	11.16146593	0.832489285	0.052723559	+	105369435	grey	0.140053182	FALSE	LOC105369435
Region_3456	chr11	94377316	94401419	24103	5	26	40.36363636	6.039960172	0.935466787	0.039356137	-	10888	grey	0.116445306	FALSE	GPR83
Region_3457	chr11	94415570	94493885	78315	13	105	128.0151515	10.42850004	0.97364069	0.015683736	-	4361	grey	0.148677001	FALSE	MRE11
Region_3458	chr11	94512461	94532123	19662	5	30	50.06060606	7.398292501	0.950361682	0.033915863	+	643037	grey	0.244186893	FALSE	C11orf97
Region_3459	chr11	94543840	94621421	77581	14	91	122.6363636	14.0182698	0.570041067	0.031555927	+	143689	grey	0.080473783	FALSE	PIWIL4
Region_3460	chr11	94543921	94549895	5974	5	14	40.22727273	10.60739286	0.032914165	0.032581923	+	2526	grey	0.033524474	FALSE	FUT4
Region_3461	chr11	94545330	94740355	195025	22	152	193.6060606	15.54534686	0.819792569	0.022168069	-	105369438	grey	-0.071526656	FALSE	PIWIL4-AS1
Region_3462	chr1	94638038	94652884	14846	4	17	27.9848484	5.65819330	0.44528038	0.08192380	+	10536943	grey	-0.03379097	FALSE	LINC02700

	1						8	8	8	3		6		2		
Region_3463	chr11	94706431	94876748	170317	25	182	214.9545455	14.56912628	0.823600538	0.024225461	+	154810	grey	-0.10138442	FALSE	AMOTL1
Region_3464	chr11	95089846	95132645	42799	4	21	34.71212121	6.499148232	0.70906849	0.041910821	+	23052	grey	0.036966788	FALSE	ENDOD1
Region_3465	chr11	95150539	95226685	76146	8	42	58.40909091	7.607642458	0.580061311	0.040579946	+	101929295	grey	0.359668926	FALSE	LNCRNA-IUR
Region_3466	chr11	95151693	95234391	82698	7	33	48.24242424	7.516260539	0.660275801	0.044056463	+	100129203	grey	0.403759668	FALSE	LOC100129203
Region_3467	chr11	95165513	95232541	67028	5	19	33.6969697	5.953588955	0.568476746	0.039466035	-	143686	grey	0.306895931	FALSE	SESN3
Region_3468	chr11	95789965	95832693	42728	6	31	43.59090909	6.295241848	0.724799688	0.034870628	+	9702	grey	-0.225644513	FALSE	CEP57
Region_3469	chr11	95821766	95925270	103504	11	60	80.24242424	8.816175005	0.882047968	0.018335971	-	8898	grey	-0.067569256	FALSE	MTMR2
Region_3470	chr11	95976598	96343195	366597	27	177	205.7727273	13.09820358	0.912594153	0.01994744	-	84441	grey	0.155724715	FALSE	MAML2
Region_3471	chr11	97878475	97959558	81083	5	22	36.6969697	7.155352374	0.876609797	0.067449705	-	105369454	grey	-0.046292433	FALSE	LINC02713
Region_3472	chr11	99020949	100358885	1337936	73	543	601.1363636	25.63764442	0.825572192	0.018040563	+	53942	grey	0.004336999	FALSE	CNTN5
Region_3473	chr11	100687288	100993941	306653	25	164	199.1212121	14.96201874	0.865545862	0.031155097	+	143872	grey	0.149873711	FALSE	ARHGAP42
Region_3474	chr11	101029624	101129813	100189	6	35	49.63636364	6.650937155	0.739103115	0.063099094	-	5241	grey	-0.019089885	FALSE	PGR

Region_3475	chr11	101129077	101209591	80514	3	14	25.8030303	4.86500754	0.818314268	0.087856226	+	101054525	grey	-0.137433342	FALSE	PGR-AS1
Region_3476	chr11	101451564	101872562	420998	35	267	293.0454545	15.95033814	0.777961387	0.028973553	-	7225	grey	0.07032531	FALSE	TRPC6
Region_3477	chr11	101890674	101916522	25848	5	28	41.75757576	6.823174551	0.521570139	0.048966522	-	253935	grey	0.359892732	FALSE	ANGPTL5
Region_3478	chr11	101915010	102001062	86052	15	107	136.6515152	13.87581451	0.794685748	0.028387962	+	57562	grey	-0.272007299	FALSE	CEP126
Region_3479	chr11	102047437	102084554	37117	10	53	75.42424242	8.859693702	0.783280272	0.036138133	+	85016	grey	-0.206526136	FALSE	CFAP300
Region_3480	chr11	102110447	102233424	122977	17	115	135.8181818	11.47560502	0.740660104	0.023591289	+	10413	grey	-0.294750048	FALSE	YAP1
Region_3481	chr11	102317450	102339403	21953	4	15	28.33333333	5.655040865	0.446954978	0.09586753	+	330	grey	0.302777107	FALSE	BIRC3
Region_3482	chr11	102347211	102378670	31459	4	16	26.40909091	5.158942267	0.897780912	0.081268229	+	329	grey	-0.346476638	FALSE	BIRC2
Region_3483	chr11	102396332	102470384	74052	4	25	36.18181818	5.922341249	0.954724773	0.053039576	-	114908	grey	0.210902627	FALSE	TMEM123
Region_3484	chr11	102467255	102498801	31546	6	36	54.06060606	8.268343314	0.853577749	0.046213263	+	102723838	grey	-0.237733453	FALSE	LOC102723838
Region_3485	chr11	102576832	102625332	48500	5	31	44.54545455	6.229413649	0.840523811	0.090414208	-	9313	grey	-0.017647367	FALSE	MMP20
Region_3486	chr11	102606916	102683922	77006	9	63	83.42424242	10.05226435	0.937680342	0.036793302	+	101928477	grey	0.013310019	FALSE	MMP20-AS1
Region_3487	chr1	10274696	10283676	89798	9	60	76.2121212	8.01251934	0.87422996	0.05483915	+	10028807	grey	0.176853932	FALSE	WTAPP1

	1	8	6				1	2	5	1		7				
Region_3488	chr11	102862736	102874982	12246	3	22	32.83333333	5.097762204	0.845680022	0.066384477	-	4321	grey	-0.152277159	FALSE	MMP12
Region_3489	chr11	102942995	102955732	12737	3	18	31.06060606	5.648359293	0.941169283	0.046703315	-	4322	grey	-0.03427916	FALSE	MMP13
Region_3490	chr11	103050686	103092194	41508	6	33	49.90909091	7.18272987	0.813732021	0.022583151	-	84259	grey	0.094725017	FALSE	DCUN1D5
Region_3491	chr11	103109410	103479863	370453	27	194	229.1212121	17.31296993	0.902132884	0.030411765	+	79659	grey	0.295054699	FALSE	DYNC2H1
Region_3492	chr11	103675994	103895271	219277	11	69	87.72727273	9.096990973	0.800652216	0.036481076	-	105369464	grey	-0.084847852	FALSE	LOC105369464
Region_3493	chr11	103907189	104164379	257190	15	102	122.7121212	11.33919703	0.924048837	0.033269317	-	80310	grey	-0.159701648	FALSE	PDGFD
Region_3494	chr11	104445868	104609498	163630	11	62	86.09090909	8.803368713	0.919353766	0.034946515	-	102723895	grey	-0.173570122	FALSE	LINC02552
Region_3495	chr11	105041326	105101431	60105	5	33	48.36363636	6.505941341	0.828671021	0.066483196	-	114769	grey	-0.137468257	FALSE	CARD16
Region_3496	chr11	105137721	105531697	393976	23	156	187.2121212	14.40140501	0.920056969	0.025623466	-	105369468	grey	-0.18991555	FALSE	LOC105369468
Region_3497	chr11	105609994	105982092	372098	16	128	153.5909091	12.41584258	0.894833641	0.025384664	+	2893	grey	0.028303787	FALSE	GRIA4
Region_3498	chr11	106051098	106077459	26361	5	31	46.07575758	6.823738218	0.777581036	0.022372149	-	143879	grey	0.099184972	FALSE	KBTBD3
Region_3499	chr11	106075501	106098699	23198	4	25	38.86363636	5.707987288	0.59237625	0.05216638	+	60496	grey	0.418005975	FALSE	AASDHPPT

Region_3500	chr11	106674019	107018476	344457	30	208	245.9545455	18.49526494	0.771054484	0.0371357	-	2977	grey	-0.085925664	FALSE	GUCY1A2
Region_3501	chr11	107326360	107457825	131465	4	16	28.78787879	4.685542909	0.958958837	0.035500876	-	143884	grey	0.164004213	FALSE	CWF19L2
Region_3502	chr11	107502727	107565742	63015	9	57	75.89393939	9.200549796	0.95474534	0.02551781	-	91801	grey	-0.128986562	FALSE	ALKBH8
Region_3503	chr11	107591091	107666779	75688	16	108	136	11.49983277	0.801216142	0.032164379	+	55531	grey	-0.301582176	FALSE	ELMOD1
Region_3504	chr11	107591745	107620470	28725	6	46	57.24242424	6.418867875	0.707189709	0.066617644	+	112267904	grey	-0.317807765	FALSE	
Region_3505	chr11	107790991	107928293	137302	42	278	328.5	19.753286	0.910557836	0.019916654	-	54733	grey	-0.24345441	FALSE	SLC35F2
Region_3506	chr11	107928448	107963482	35034	8	38	55.86363636	7.701920567	0.648242831	0.063472417	+	54734	grey	-0.008675635	FALSE	RAB39A
Region_3507	chr11	108008898	108107761	98863	23	151	181.9848485	12.83803534	0.837030549	0.017808436	+	8065	grey	-0.41756371	FALSE	CUL5
Region_3508	chr11	108116695	108147603	30908	4	16	27.34848485	4.89738525	0.728648358	0.030688351	+	38	grey	-0.184650284	FALSE	ACAT1
Region_3509	chr11	108157215	108222638	65423	4	27	37.37878788	5.414411401	0.973654506	0.029379083	-	4863	grey	0.160782113	FALSE	NPAT
Region_3510	chr11	108223044	108369102	146058	24	178	201.1818182	11.84893256	0.937342883	0.014586089	+	472	grey	0.202687484	FALSE	ATM
Region_3511	chr11	108308519	108467531	159012	31	195	237.5454545	16.38872203	0.931177634	0.017816566	-	160140	grey	0.257174203	FALSE	C11orf65
Region_3512	chr1	10847211	10849838	26272	3	14	21.1212121	3.71489787	0.65445302	0.04795173	-	143888	grey	-0.44647001	FALSE	POGLUT3

	1	2	4				2	2	5	9				3		
Region_3513	chr11	108505435	108593768	88333	12	68	97.77272727	11.4149638	0.860707382	0.031827229	-	23086	grey	-0.036421059	FALSE	EXPH5
Region_3514	chr11	108665058	108940999	275941	34	252	279.1818182	14.86545721	0.853761877	0.020676688	+	1662	grey	-0.165615903	FALSE	DDX10
Region_3515	chr11	109741625	109823893	82268	5	28	40.62121212	5.817123665	0.884416552	0.05115677	-	105369482	grey	-0.001867209	FALSE	LINC02715
Region_3516	chr11	109803905	110088184	284279	37	280	313.6515152	17.47569074	0.786637054	0.019988157	-	107984384	grey	0.074797628	FALSE	
Region_3517	chr11	109864295	110296712	432417	68	496	553.0151515	25.72546923	0.822815431	0.012616655	-	5962	grey	-0.144971976	FALSE	RDX
Region_3518	chr11	109946581	109975996	29415	6	26	45.75757576	6.447565089	0.721068096	0.049802311	+	105369484	grey	-0.111155171	FALSE	LOC105369484
Region_3519	chr11	110093392	110171841	78449	17	96	128.1363636	12.14766374	0.855365822	0.021404592	+	85463	grey	-0.307274299	FALSE	ZC3H12C
Region_3520	chr11	110355130	110407613	52483	9	50	73.36363636	8.88669942	0.917377247	0.04504858	+	105369486	grey	0.095195201	FALSE	LINC02732
Region_3521	chr11	110429948	110464884	34936	4	19	31.27272727	6.115668287	0.009205693	0.017262763	+	2230	grey	0.046070542	FALSE	FDX1
Region_3522	chr11	110577042	110713189	136147	11	67	91.68181818	9.062448457	0.884477899	0.054635426	-	57569	grey	0.053489548	FALSE	ARHGAP20
Region_3523	chr11	111091932	111179641	87709	10	65	85.68181818	9.981766594	0.93537674	0.03138988	-	105369490	grey	-0.039192291	FALSE	LINC02550
Region_3524	chr11	111245725	111286401	40676	9	76	90.62121212	7.029093719	0.86730543	0.050075384	+	341032	grey	0.110223506	FALSE	POU2AF2

Region_3525	chr11	111290787	111305498	14711	4	10	25.95454545	5.818886539	0.347487267	0.077129933	-	399948	grey	0.051743408	FALSE	COLCA1
Region_3526	chr11	111298546	111308735	10189	5	22	36.8030303	6.036350647	0.457241493	0.069184345	+	120376	grey	0.070253223	FALSE	POU2AF3
Region_3527	chr11	111352255	111449981	97726	9	59	79.71212121	9.39028164	0.878204921	0.041288086	-	5450	grey	0.082166605	FALSE	POU2AF1
Region_3528	chr11	111384737	111514367	129630	17	122	145.5	11.53623055	0.671003633	0.030146673	-	54766	grey	0.403987628	FALSE	BTG4
Region_3529	chr11	111448450	111473381	24931	4	19	34.40909091	6.409073551	0.900079305	0.057657944	+	644277	grey	0.050665826	FALSE	LOC644277
Region_3530	chr11	111510600	111513888	3288	3	16	24.53030303	4.654921951	0.008139111	0.019263595	+	728196	grey	0.478797112	FALSE	MIR34BHG
Region_3531	chr11	111514778	111537043	22265	3	14	26.09090909	4.676380167	0.652129659	0.050782291	+	399949	grey	0.122957877	FALSE	HOATZ
Region_3532	chr11	111540280	111561745	21465	3	11	20.31818182	4.325804828	0.324162986	0.042883682	+	143903	grey	-0.163531036	FALSE	LAYN
Region_3533	chr11	111602449	111730855	128406	30	195	233.6818182	15.94139178	0.880280866	0.017053732	+	23235	grey	-0.146593309	FALSE	SIK2
Region_3534	chr11	111726908	111766389	39481	16	86	110.8636364	11.6181375	0.762575426	0.022826682	-	5519	grey	0.082828764	FALSE	PPP2R1B
Region_3535	chr11	111768668	111879425	110757	25	190	218.9242424	14.59958967	0.815120961	0.035046151	-	79796	grey	0.148323961	FALSE	ALG9
Region_3536	chr11	111870295	111881243	10948	5	32	45.31818182	6.263837828	0.399372587	0.074584073	-	91893	grey	0.159382189	FALSE	FDXACB1
Region_3537	chr1	11190856	11192372	15158	5	20	35.5151515	5.78922719	0.73647421	0.05433579	-	1410	grey	0.135144344	FALSE	CRYAB

	1	4	2				2	5	6	9						
Region_3538	chr1<br>1	11191274<br>2	11192687<br>1	14129	4	16	30.2272727<br>3	4.95454866<br>2	0.93912478<br>5	0.04665627<br>1	+	10052801<br>9	grey	0.181005829	FALSE	HSPB2-C11orf52
Region_3539	chr1<br>1	11191891<br>3	11192687<br>1	7958	4	16	30.2272727<br>3	4.95454866<br>2	0.93912478<br>5	0.04665627<br>1	+	91894	grey	0.181005829	FALSE	C11orf52
Region_3540	chr1<br>1	11192714<br>4	11202265<br>3	95509	14	88	107.045454<br>5	10.3395161<br>6	0.73459490<br>6	0.02171393<br>2	+	85458	grey	0.047936716	FALSE	DIXDC1
Region_3541	chr1<br>1	11202540<br>8	11206440<br>4	38996	7	42	55.4393939<br>4	8.12996127<br>9	0.95534735<br>6	0.03397038<br>8	+	1737	grey	0.217537625	FALSE	DLAT
Region_3542	chr1<br>1	11208682<br>4	11219356<br>1	106737	19	116	143.272727<br>3	11.5619341<br>7	0.85796387<br>7	0.02595862<br>5	+	6392	grey	0.171350445	FALSE	SDHD
Region_3543	chr1<br>1	11208690<br>3	11222469<br>9	137796	21	131	159	12.9709735<br>1	0.86896459	0.02389468<br>3	+	83875	grey	0.188083428	FALSE	BCO2
Region_3544	chr1<br>1	11222636<br>7	11226995<br>5	43588	11	65	88.5757575<br>8	7.86630713<br>1	0.85584873<br>3	0.02384626<br>2	+	5805	grey	-0.10144496<br>5	FALSE	PTS
Region_3545	chr1<br>1	11224815<br>3	11226086<br>0	12707	3	14	21.4393939<br>4	3.73395557<br>6	0.96372686	0.05461700<br>1	-	349633	grey	-0.20581357<br>8	FALSE	PLET1
Region_3546	chr1<br>1	11227074<br>8	11236253<br>4	91786	13	94	111.863636<br>4	8.73170414<br>9	0.69438693<br>2	0.02543752<br>5	+	283140	grey	-0.03674611	FALSE	LINC02762
Region_3547	chr1<br>1	11239311<br>8	11262456<br>7	231449	19	128	158.712121<br>2	11.1641805<br>7	0.80337066<br>8	0.04997776<br>8	+	10192882<br>3	grey	-0.21843322<br>8	FALSE	LINC02763
Region_3548	chr1<br>1	11282280<br>6	11283512<br>4	12318	5	27	39.5303030<br>3	6.68458547<br>6	0.80554167<br>2	0.07292568	-	10536949<br>8	grey	-0.23309195<br>6	FALSE	LOC105369498
Region_3549	chr1<br>1	11295927<br>9	11296346<br>0	4181	4	15	28.9242424<br>2	6.08734022	0.21208063<br>8	0.05270131<br>7	-	10192884<br>7	grey	0.021226597	FALSE	LOC101928847

Region_3550	chr11	112961247	113278436	317189	51	341	394.5757576	20.33266459	0.797251319	0.023195082	+	4684	grey	-0.11056348	FALSE	NCAM1
Region_3551	chr11	113314579	113383544	68965	18	130	158.3484848	12.76954601	0.8361963	0.034319424	+	54970	grey	0.267698278	FALSE	TTC12
Region_3552	chr11	113387779	113400416	12637	3	11	22.89393939	4.537463839	0.879073487	0.077214066	+	255239	grey	-0.042119294	FALSE	ANKK1
Region_3553	chr11	113409605	113475691	66086	8	51	69	8.028794334	0.816240192	0.058152013	-	1813	grey	-0.105835842	FALSE	DRD2
Region_3554	chr11	113733187	113773735	40548	11	76	98.68181818	9.283830594	0.785033055	0.021537842	-	9183	grey	0.240222297	FALSE	ZW10
Region_3555	chr11	113797874	113875572	77698	23	140	179.5909091	15.16572783	0.919065545	0.015811082	-	57646	grey	-0.150520194	FALSE	USP28
Region_3556	chr11	113904796	113949079	44283	28	193	229.0606061	15.37118061	0.906214049	0.023095655	+	9177	grey	-0.076679035	FALSE	HTR3B
Region_3557	chr11	113975075	113990313	15238	7	38	55.81818182	8.260953737	0.522244694	0.06171197	+	3359	grey	0.012421791	FALSE	HTR3A
Region_3558	chr11	114059041	114256765	197724	70	508	566.1818182	31.23504397	0.740984155	0.019409765	+	7704	grey	0.130942423	FALSE	ZBTB16
Region_3559	chr11	114257787	114313536	55749	8	49	65.25757576	7.245817056	0.820564455	0.051429295	+	4837	grey	-0.108425895	FALSE	NNMT
Region_3560	chr11	114343052	114396219	53167	7	52	67.62121212	7.591017265	0.946563013	0.028240018	-	101928940	grey	0.273701853	FALSE	LOC101928940
Region_3561	chr11	114391443	114400511	9068	3	22	30.12121212	4.869102434	0.906665311	0.049709053	-	54494	grey	-0.039416039	FALSE	C11orf71
Region_3562	chr1	11440003	11444393	43902	4	10	22.9545454	4.65231726	0.44464641	0.07327988	+	10179	grey	-0.38803166	FALSE	RBM7

	1	0	2				5	6		8				9		
Region_3563	chr11	114518934	114559881	40947	3	18	28.66666667	4.971714867	0.964883438	0.040520052	-	120400	grey	0.107650326	FALSE	NXPE1
Region_3564	chr11	115169218	115504957	335739	56	415	472.9090909	24.88170615	0.723249743	0.017615442	-	23705	grey	0.403715136	FALSE	CADM1
Region_3565	chr11	115659658	115843146	183488	23	141	167.4545455	13.71824368	0.800462207	0.029973771	+	105369512	grey	-0.295073519	FALSE	LINC02698
Region_3566	chr11	115920248	115943181	22933	4	17	33.8030303	5.749166353	0.548941225	0.106360216	+	102724165	grey	0.390030529	FALSE	LINC02703
Region_3567	chr11	116639422	116658295	18873	4	15	31.90909091	5.65611248	0.881007629	0.067715835	+	101929011	grey	-0.046843524	FALSE	LINC02702
Region_3568	chr11	116748170	116772987	24817	6	29	40.75757576	5.452058485	0.813524868	0.035664342	-	84811	grey	0.336234929	FALSE	BUD13
Region_3569	chr11	116820700	116823304	2604	3	12	21.57575758	4.231968467	0.945871081	0.050129566	-	337	grey	-0.229676324	FALSE	APOA4
Region_3570	chr11	116836117	116856018	19901	4	19	33.43939394	6.276032034	0.96963556	0.035663043	+	104326055	grey	0.177793249	FALSE	APOA1-AS
Region_3571	chr11	116843402	117098437	255035	76	565	628.3939394	28.0531796	0.82698667	0.01580534	-	23387	grey	0.191550414	FALSE	SIK3
Region_3572	chr11	117144284	117176894	32610	16	74	93.09090909	7.781095973	0.67269886	0.028501218	+	5049	grey	0.216902301	FALSE	PAFAH1B2
Region_3573	chr11	117178736	117197445	18709	11	61	83.04545455	9.196038977	0.785902549	0.028541113	+	51092	grey	-0.076343062	FALSE	SIDT2
Region_3574	chr11	117204337	117232525	28188	9	34	54.27272727	10.25002132	0.430711638	0.0375848	-	9159	grey	0.11229521	FALSE	PCSK7

Region_3575	chr11	117232625	117286454	53829	12	61	85.89393939	10.56718984	0.97156089	0.016816219	+	257160	grey	-0.014604387	FALSE	RNF214
Region_3576	chr11	117285232	117316259	31027	12	60	85.71212121	8.512009889	0.874128015	0.027622761	-	23621	grey	0.234214568	FALSE	BACE1
Region_3577	chr11	117314557	117413266	98709	43	272	323.0909091	18.3443366	0.770811719	0.018988456	+	22897	grey	-0.284718681	FALSE	CEP164
Region_3578	chr11	117427772	117817525	389753	154	1109	1220.106061	46.44902211	0.812701486	0.014733062	-	57453	grey	0.13537583	FALSE	DSCAML1
Region_3579	chr11	117801730	117828698	26968	7	33	47.16666667	7.947100745	0.808613639	0.062695213	-	486	grey	0.13701508	FALSE	FXYD2
Region_3580	chr11	117820163	117876667	56504	26	164	205.969697	15.30105044	0.765163153	0.027306788	-	100533181	grey	0.095465	FALSE	FXYD6-FXYD2
Region_3581	chr11	117836976	117877486	40510	20	133	162.1818182	12.6404833	0.753401122	0.031643438	-	53826	grey	0.167019097	FALSE	FXYD6
Region_3582	chr11	117900641	117929459	28818	8	39	58.3030303	8.373896615	0.889089565	0.056041316	-	84000	grey	0.045207606	FALSE	TMPRSS13
Region_3583	chr11	117986370	118003037	16667	3	16	31.37878788	5.664369478	0.950940724	0.045173402	+	3587	grey	0.338631336	FALSE	IL10RA
Region_3584	chr11	118003634	118088299	84665	33	212	248.8030303	14.77755023	0.765691989	0.025632312	-	100526771	grey	0.264583398	FALSE	SMIM35
Region_3585	chr11	118077012	118121890	44878	7	36	50.46969697	6.636077843	0.7651574	0.054869276	+	56649	grey	0.136137635	FALSE	TMPRSS4
Region_3586	chr11	118133377	118152888	19511	5	20	34.84848485	7.630389494	0.615869811	0.092155137	-	6330	grey	-0.315810278	FALSE	SCN4B
Region_3587	chr1	11816280	11817663	13833	5	26	37.2272727	6.18135025	0.83897543	0.05635238	-	6327	grey	-0.23785719	FALSE	SCN2B

	1	6	9				3		8	3				6		
Region_3588	chr11	118193725	118225094	31369	8	46	67.27272727	8.096619341	0.848708507	0.048353601	-	120425	grey	-0.04233053	FALSE	JAML
Region_3589	chr11	118226690	118252365	25675	4	16	28.51515152	5.429952328	0.454443676	0.044342299	-	196264	grey	-0.360771429	FALSE	MPZL3
Region_3590	chr11	118304730	118316175	11445	5	32	44.57575758	7.200556829	0.874604022	0.067998779	+	916	grey	0.024329859	FALSE	CD3E
Region_3591	chr11	118359600	118399211	39611	8	54	69.51515152	7.117135172	0.969908256	0.025243593	+	9354	grey	0.187189998	FALSE	UBE4A
Region_3592	chr11	118401346	118449569	48223	41	275	318.6818182	23.46988846	0.3869162	0.015729191	+	10632	grey	0.022872762	FALSE	ATP5MG
Region_3593	chr11	118401607	118526832	125225	60	414	461.2424242	24.30198511	0.651887542	0.013078704	+	4297	grey	-0.051881805	FALSE	KMT2A
Region_3594	chr11	118510273	118531094	20821	12	67	93.57575758	8.883971056	0.400239073	0.01873943	-	101929089	grey	-0.002644883	FALSE	TTC36-AS1
Region_3595	chr11	118527472	118531197	3725	10	58	75.77272727	8.845329849	0.290516395	0.016729924	+	143941	grey	0.018716427	FALSE	TTC36
Region_3596	chr11	118531041	118547280	16239	6	57	71.36363636	6.993703462	0.795648011	0.028577386	+	84866	grey	-0.005199271	FALSE	TMEM25
Region_3597	chr11	118544528	118572970	28442	19	130	158.1060606	12.99127374	0.557069077	0.01622857	-	56912	grey	0.279786209	FALSE	IFT46
Region_3598	chr11	118572390	118603033	30643	9	55	75.06060606	9.253487971	0.536648154	0.017532345	+	372	grey	0.34456331	FALSE	ARCN1
Region_3599	chr11	118606440	118658031	51591	31	156	197.0606061	14.8467855	0.631749815	0.02674216	+	23187	grey	-0.140818241	FALSE	PHLDB1

Region_3600	chr11	118657316	118679690	22374	13	74	96.81818182	10.84138664	0.808669702	0.034609966	-	11181	grey	-0.178839542	FALSE	TREH
Region_3601	chr11	118700415	118750263	49848	25	166	209.5909091	14.59185998	0.9192313	0.018237853	+	105369519	grey	-0.077255274	FALSE	LOC105369519
Region_3602	chr11	118747763	118791164	43401	21	137	164.9090909	13.98320671	0.895065776	0.024446622	-	1656	grey	0.201903494	FALSE	DDX6
Region_3603	chr11	118883892	118897787	13895	8	43	60.22727273	8.310874164	0.673188009	0.063516991	+	643	grey	-0.219336508	FALSE	CXCR5
Region_3604	chr11	118893875	118925926	32051	29	156	189.4545455	16.5261395	0.730021312	0.029507646	-	283149	grey	-0.149662527	FALSE	BCL9L
Region_3605	chr11	118925164	118958559	33395	21	130	148.8181818	10.28203675	0.417369839	0.024983481	+	7379	grey	0.06895131	FALSE	UPK2
Region_3606	chr11	118971712	118981287	9575	8	50	63.45454545	8.44563031	0.975004663	0.027250873	+	283150	grey	0.158263678	FALSE	FOXR1
Region_3607	chr11	119084866	119093834	8968	5	30	41.34848485	5.290026681	0.394233864	0.030380954	+	3145	grey	-0.196624188	FALSE	HMBS
Region_3608	chr11	119093874	119095465	1591	3	15	29.06060606	5.927455772	0.003662695	0.011626149	-	3014	grey	0.015254703	FALSE	H2AX
Region_3609	chr11	119096025	119108331	12306	3	18	28.83333333	4.969393504	0.418206821	0.059311788	-	1798	grey	0.356968511	FALSE	DPAGT1
Region_3610	chr11	119102198	119118544	16346	6	36	49.54545455	6.818937021	0.978549199	0.020765554	+	9854	grey	-0.093237942	FALSE	C2CD2L
Region_3611	chr11	119121580	119136059	14479	4	13	26.09090909	4.741720794	0.948863689	0.059798985	+	25988	grey	-0.355544779	FALSE	HINFP
Region_3612	chr1	11916656	11918401	17448	11	69	87.3333333	9.80476074	0.83819011	0.02594571	+	79671	grey	-0.11975822	FALSE	NLRX1

	1	8	6				3	8	3	7				2		
Region_3613	chr11	119185457	119190223	4766	11	44	62.51515152	8.53453153	0.875539332	0.0499926	+	79849	grey	0.024779808	FALSE	NHERF4
Region_3614	chr11	119190250	119196769	6519	7	39	54.31818182	6.635937337	0.955059522	0.029518034	-	283152	grey	0.062163431	FALSE	CCDC153
Region_3615	chr11	119206298	119313926	107628	50	326	371.2878788	21.98374585	0.922461496	0.015129443	+	867	grey	-0.049304361	FALSE	CBL
Region_3616	chr11	119308529	119321521	12992	7	36	51.92424242	6.807937915	0.601687037	0.04740108	-	4162	grey	0.115679801	FALSE	MCAM
Region_3617	chr11	119338942	119340883	1941	4	10	22.78787879	4.78303139	0.277384458	0.089150807	-	114902	grey	-0.109422222	FALSE	C1QTNF5
Region_3618	chr11	119338942	119346705	7763	6	19	33.54545455	5.773098936	0.478282961	0.071779319	-	83552	grey	-0.188673897	FALSE	MFRP
Region_3619	chr11	119355215	119381711	26496	12	66	82.74242424	7.413730725	0.781513691	0.03129187	-	9099	grey	0.176299501	FALSE	USP2
Region_3620	chr11	119364359	119526664	162305	34	217	260.0606061	16.5537621	0.717699844	0.029577507	+	100499227	grey	-0.014676715	FALSE	USP2-AS1
Region_3621	chr11	119415476	119424985	9509	3	17	27.10606061	5.05699451	0.592225394	0.063560705	-	7070	grey	-0.260447973	FALSE	THY1
Region_3622	chr11	119608423	119659284	50861	20	125	148.2121212	11.70471983	0.855701375	0.044855157	+	105369520	grey	-0.034173868	FALSE	LOC105369520
Region_3623	chr11	119623408	119729200	105792	38	234	266.3636364	18.08070486	0.704887297	0.025735582	-	5818	grey	-0.155611955	FALSE	NECTIN1
Region_3624	chr11	119892700	119939998	47298	16	104	127.5909091	11.97301745	0.829078168	0.036104288	+	105369526	grey	-0.145821476	FALSE	LOC105369526

Region_3625	chr11	120111286	120138114	26828	6	34	47.34848485	6.918186566	0.908975605	0.048048017	-	23650	grey	-0.025230165	FALSE	TRIM29
Region_3626	chr11	120168977	120171679	2702	7	39	56.37878788	6.815740045	0.56024749	0.081642151	+	107984399	grey	-0.019372479	FALSE	LOC107984399
Region_3627	chr11	120211032	120230334	19302	6	30	40.86363636	6.208892788	0.501777438	0.054703044	+	220323	grey	-0.109476843	FALSE	OAF
Region_3628	chr11	120236640	120319945	83305	14	74	107.3030303	12.66366054	0.860756934	0.026968597	+	25833	grey	0.032047662	FALSE	POU2F3
Region_3629	chr11	120249759	120265932	16173	5	21	41.75757576	7.040674369	0.934110009	0.039599456	-	649133	grey	-0.064640032	FALSE	LOC649133
Region_3630	chr11	120336413	120489937	153524	44	312	361.1818182	22.02858918	0.802370834	0.013491554	+	23365	grey	-0.064561515	FALSE	ARHGEF12
Region_3631	chr11	120511746	120988906	477160	108	749	849.5757576	31.04887492	0.797060002	0.014986076	+	2900	grey	0.140731575	FALSE	GRIK4
Region_3632	chr11	120575799	120815438	239639	55	374	456.5151515	23.32330787	0.849563667	0.017893076	+	107984400	grey	0.358147132	FALSE	LOC107984400
Region_3633	chr11	120867933	120894797	26864	5	24	41.09090909	6.749876974	0.736478482	0.046199209	-	101929227	grey	0.281811692	FALSE	LOC101929227
Region_3634	chr11	121024072	121090776	66704	10	57	77.06060606	8.621759213	0.952461287	0.023512304	+	219899	grey	-0.009682885	FALSE	TBCEL
Region_3635	chr11	121024125	121113108	88983	12	73	93.87878788	9.699349941	0.941600384	0.027673012	+	116804918	grey	-0.022363783	FALSE	TBCEL-TECTA
Region_3636	chr11	121101243	121191490	90247	20	162	191.0454545	13.00820075	0.919534463	0.028525085	+	7007	grey	-0.017690319	FALSE	TECTA
Region_3637	chr1	12129268	12131341	20729	6	37	55.4545454	8.20540500	0.69969449	0.05774887	+	6309	grey	0.039313495	FALSE	SC5D

	1	1	0				5	7	5	5						
Region_3638	chr11	121452314	121633763	181449	42	311	352.5454545	20.68234611	0.734762072	0.020330754	+	6653	grey	0.072768486	FALSE	SORL1
Region_3639	chr11	121674261	121928976	254715	26	173	195.8787879	14.66499249	0.774342423	0.026112104	+	107984402	grey	0.232512332	FALSE	LOC107984402
Region_3640	chr11	122028325	122556721	528396	73	499	570.1212121	26.79581779	0.816268664	0.023145522	-	399959	grey	-0.023145005	FALSE	MIR100HG
Region_3641	chr11	122655722	122814473	158751	65	434	501.030303	26.04609505	0.771416132	0.018312823	+	84959	grey	0.237356317	FALSE	UBASH3B
Region_3642	chr11	122838500	122872643	34143	14	85	119.0909091	12.54128845	0.867721169	0.040253216	+	56253	grey	-0.111172275	FALSE	CRTAM
Region_3643	chr11	122882683	122963862	81179	27	189	229.8636364	12.69510671	0.872678967	0.01665265	+	79864	grey	-0.3807402	FALSE	JHY
Region_3644	chr11	122977570	122981834	4264	4	20	30.3030303	5.460261186	0.135754576	0.069656241	-	390259	grey	-0.004694325	FALSE	BSX
Region_3645	chr11	123057489	123063230	5741	4	20	31.75757576	6.739232109	0.168338112	0.050441522	-	3312	grey	-0.051543934	FALSE	HSPA8
Region_3646	chr11	123062655	123084877	22222	11	63	87.65151515	9.45351774	0.603533758	0.027812712	+	101929289	grey	-0.304197923	FALSE	
Region_3647	chr11	123069872	123195248	125376	49	349	395.7121212	18.07315489	0.784897786	0.02062753	-	79827	grey	0.191496237	FALSE	CLMP
Region_3648	chr11	123358428	123627774	269346	86	613	704.7575758	34.45936765	0.704292633	0.012102775	+	57476	grey	0.112633566	FALSE	GRAMD1B
Region_3649	chr11	123629187	123655244	26057	5	26	38.95454545	6.095223396	0.710014589	0.095791776	-	55800	grey	-0.29396419	FALSE	SCN3B

Region_3650	chr11	123723914	123741675	17761	5	27	37.16666667	5.464805812	0.652812736	0.081747677	-	7753	grey	0.037909679	FALSE	ZNF202
Region_3651	chr11	124115404	124147721	32317	5	32	46.31818182	6.737173776	0.963246701	0.029892583	+	4013	grey	0.322155407	FALSE	VWA5A
Region_3652	chr11	124622836	124635926	13090	7	43	60.04545455	9.236102771	0.953403609	0.032278517	+	84897	grey	0.155508116	FALSE	TBRG1
Region_3653	chr11	124633113	124695707	62594	17	121	151.2727273	13.01070028	0.631392606	0.027511286	-	54414	grey	-0.053519081	FALSE	SIAE
Region_3654	chr11	124739942	124747210	7268	16	78	108.8333333	12.28204861	0.197579337	0.019629103	+	4900	grey	-0.083721445	FALSE	NRGN
Region_3655	chr11	124759129	124766119	6990	4	16	26.09090909	4.263358274	0.253289595	0.037388568	+	101929340	grey	0.270780265	FALSE	ESAM-AS1
Region_3656	chr11	124766498	124800706	34208	9	60	72.60606061	8.129488181	0.434356721	0.015171173	-	79684	grey	-0.324142297	FALSE	MSANTD2
Region_3657	chr11	124800424	124834487	34063	5	29	39.74242424	5.709130625	0.627803997	0.055248758	+	100507283	grey	-0.042485309	FALSE	MSANTD2-AS1
Region_3658	chr11	124865432	124881471	16039	11	67	81.22727273	8.598381698	0.379485837	0.040317757	+	64221	grey	0.076898793	FALSE	ROBO3
Region_3659	chr11	124919205	124936412	17207	3	13	22.89393939	5.084300989	0.702040594	0.072648197	-	220296	grey	0.032615707	FALSE	HEPACAM
Region_3660	chr11	124954121	125041489	87368	21	153	184.530303	15.64531849	0.849785219	0.023134963	+	80071	grey	0.014328305	FALSE	CCDC15
Region_3661	chr11	125063302	125090516	27214	15	83	111.1818182	12.34991011	0.869183328	0.029060445	+	219855	grey	0.004746435	FALSE	SLC37A2
Region_3662	chr1	12516468	12543338	268702	83	575	646.606060	29.3843908	0.74239387	0.02356567	+	63876	grey	-0.08899402	FALSE	PKNOX2

	1	7	9				6	3	9	3				9		
Region_3663	chr11	125258626	125266798	8172	4	18	30.8030303	5.060312234	0.839214321	0.071622935	-	105369549	grey	0.005200191	FALSE	PKNOX2-AS1
Region_3664	chr11	125442881	125592510	149629	46	282	325.0757576	23.42405772	0.493531094	0.016115085	-	9638	grey	0.074910997	FALSE	FEZ1
Region_3665	chr11	125495214	125499528	4314	19	83	114.0454545	16.74186996	0.113272416	0.015326208	+	403312	grey	-0.152795773	FALSE	LOC403312
Region_3666	chr11	125569280	125584684	15404	4	14	29.56060606	6.061552956	0.261744743	0.020137256	+	9538	grey	-0.142790744	FALSE	EI24
Region_3667	chr11	125570284	125592568	22284	5	28	43.95454545	6.366817387	0.916515207	0.039073955	-	105369550	grey	0.356534415	FALSE	STT3A-AS1
Region_3668	chr11	125625163	125676255	51092	8	31	45.81818182	6.471022012	0.596745194	0.045945882	+	1111	grey	0.174797608	FALSE	CHEK1
Region_3669	chr11	125883614	125900646	17032	5	36	49.89393939	6.382979261	0.419399952	0.034785405	+	219844	grey	-0.198033268	FALSE	HYLS1
Region_3670	chr11	125893485	125903221	9736	3	18	29.04545455	4.903473865	0.649728435	0.028878907	-	83480	grey	-0.146803667	FALSE	PUS3
Region_3671	chr11	125903348	125943684	40336	19	131	158.0757576	11.78827397	0.688596312	0.042196778	+	29118	grey	0.000226407	FALSE	DDX25
Region_3672	chr11	125946044	125958647	12603	6	37	50.28787879	6.912119015	0.839043613	0.055666794	+	338667	grey	-0.027415887	FALSE	VSIG10L2
Region_3673	chr11	125955796	126063335	107539	41	281	332.3787879	18.05271354	0.794003916	0.019914478	-	50937	grey	0.139808433	FALSE	CDON
Region_3674	chr11	126202096	126211692	9596	3	20	30.34848485	5.272548426	0.64588472	0.031384745	-	84881	grey	0.23405234	FALSE	RPUSD4

Region_3675	chr11	126211724	126262984	51260	13	79	97.51515152	9.470996088	0.85997629	0.035868019	+	79607	grey	-0.180436341	FALSE	FAM118B
Region_3676	chr11	126269024	126278131	9107	5	24	35.75757576	6.358666058	0.938165572	0.054718776	+	55572	grey	-0.107553342	FALSE	FOXRED1
Region_3677	chr11	126282497	126298845	16348	13	75	98.36363636	10.28013224	0.647619323	0.022182231	+	114609	grey	-0.221197978	FALSE	TIRAP
Region_3678	chr11	126294296	126304321	10025	12	66	87.45454545	9.70357875	0.582084678	0.031969685	-	105369557	grey	0.292004143	FALSE	TIRAP-AS1
Region_3679	chr11	126304060	126350005	45945	35	235	276.8333333	18.52448138	0.804059633	0.023873747	+	28960	grey	-0.20069646	FALSE	DCPS
Region_3680	chr11	126340882	126355587	14705	7	31	46.04545455	5.671607609	0.806277599	0.060902415	-	399972	grey	-0.015500391	FALSE	GSEC
Region_3681	chr11	126355640	126440344	84704	29	169	207.969697	15.95375017	0.805871321	0.024087959	+	6484	grey	-0.066288734	FALSE	ST3GAL4
Region_3682	chr11	126423358	127003460	580102	130	1002	1094.984848	35.76740778	0.801554017	0.016531181	-	84623	grey	0.066204325	FALSE	KIRREL3
Region_3683	chr11	126652852	126682109	29257	4	27	37.10606061	5.81014706	0.707109314	0.063013704	+	101929427	grey	0.143913656	FALSE	LOC101929427
Region_3684	chr11	127021751	127116678	94927	13	89	120.7575758	10.94048319	0.87431668	0.034297159	+	101929473	grey	0.044559045	FALSE	LOC101929473
Region_3685	chr11	127021765	127139719	117954	16	114	144.2575758	11.73333267	0.873694017	0.035191784	+	105369562	grey	0.018684591	FALSE	LOC105369562
Region_3686	chr11	127271029	127337033	66004	7	45	59.10606061	7.770018885	0.901472631	0.044129232	+	101929497	grey	0.13762695	FALSE	LINC02712
Region_3687	chr1	12809575	12818367	87913	9	69	91.4545454	10.3003835	0.93745909	0.04179662	+	10536956	grey	-0.18058947	FALSE	LINC02725

	1	8	1				5	9				3		3		
Region_3688	chr1 1	12845876 1	12858755 8	128797	36	249	294.121212 1	16.0734038 8	0.72067811 4	0.02609071 9	-	2113	grey	0.329922059	FALSE	ETS1
Region_3689	chr1 1	12861434 7	12868643 3	72086	20	149	167.727272 7	11.4415510 7	0.65917282 4	0.03326734 2	-	10536956 8	grey	-0.07071380 5	FALSE	LOC105369568
Region_3690	chr1 1	12862965 3	12868692 2	57269	19	136	157.333333 3	12.1558258 7	0.61585093 7	0.03969417 9	-	10192953 8	grey	0.06215179	FALSE	LOC101929538
Region_3691	chr1 1	12868653 5	12881326 7	126732	65	416	484.984848 5	25.5351898 5	0.59343848 9	0.01554651 7	+	2313	grey	0.245513263	FALSE	FLI1
Region_3692	chr1 1	12869166 4	12869602 3	4359	10	45	65.6060606 1	9.10769545 8	0.00443014 1	0.00971728 2	-	10050739 2	grey	-0.12360309 5	FALSE	SENCR
Region_3693	chr1 1	12883631 5	12886737 3	31058	4	29	41.6212121 2	6.41815969 7	0.48527267 1	0.04350607	-	3758	grey	-0.27756076	FALSE	KCNJ1
Region_3694	chr1 1	12889135 6	12892116 3	29807	10	67	86.6363636 4	8.01275207 4	0.62160043 8	0.04536884	+	3762	grey	-0.09830373 6	FALSE	KCNJ5
Region_3695	chr1 1	12889956 5	12890604 4	6479	4	21	36.4242424 2	6.22417274 3	0.65341163 2	0.08944289 7	-	219833	grey	-0.05204986 7	FALSE	KCNJ5-AS1
Region_3696	chr1 1	12896506 0	12927932 4	314264	38	295	329.757575 8	17.6059960 1	0.92021510 6	0.02762189 2	-	9743	grey	0.118988451	FALSE	ARHGAP32
Region_3697	chr1 1	12937584 8	12945227 9	76431	8	59	76.3787878 8	7.34378890 2	0.79321628 7	0.03544858 5	+	8538	grey	-0.15434718 9	FALSE	BARX2
Region_3698	chr1 1	12981584 8	12986000 3	44155	23	145	167.924242 4	11.4573598 9	0.77560907 7	0.02015739 8	+	120224	grey	-0.08439836 2	FALSE	TMEM45B
Region_3699	chr1 1	12986363 6	12989559 0	31954	22	164	191.545454 5	13.8321607 6	0.93618183 9	0.01362097 7	-	4798	grey	-0.17563804 2	FALSE	NFRKB

Region_3700	chr11	129899706	130002835	103129	46	357	396.4242424	21.36428407	0.902903744	0.013648113	-	56980	grey	-0.000958448	FALSE	PRDM10
Region_3701	chr11	130068147	130144811	76664	40	281	320.8181818	19.48325442	0.684738356	0.014987107	+	334	grey	-0.160530015	FALSE	APLP2
Region_3702	chr11	130159782	130210362	50580	28	186	224.9393939	16.33107353	0.877454472	0.025014512	+	6768	grey	-0.168329722	FALSE	ST14
Region_3703	chr11	130226677	130314917	88240	24	155	188.5757576	13.78300024	0.938572128	0.023935323	-	29068	grey	0.091790988	FALSE	ZBTB44
Region_3704	chr11	130314993	130403657	88664	40	264	299.6818182	16.99969148	0.842332761	0.016435952	+	646383	grey	-0.037751797	FALSE	ZBTB44-DT
Region_3705	chr11	130404923	130428609	23686	4	15	26.09090909	4.754681168	0.7988446	0.092173591	-	11095	grey	-0.128252684	FALSE	ADAMTS8
Region_3706	chr11	130448645	130476645	28000	13	75	92.66666667	9.98511713	0.797371428	0.035455006	+	170689	grey	-0.101051534	FALSE	ADAMTS15
Region_3707	chr11	130672956	130717352	44396	5	31	46.56060606	6.327300525	0.92032701	0.042971386	+	283171	grey	-0.176117477	FALSE	LINC02873
Region_3708	chr11	130771402	130785533	14131	3	16	28.34848485	5.584297833	0.937913431	0.043534775	-	105369576	grey	-0.061107082	FALSE	LOC105369576
Region_3709	chr11	130866250	130916479	50229	5	16	27.03030303	5.678487602	0.967917125	0.038060846	-	399979	grey	-0.063876087	FALSE	SNX19
Region_3710	chr11	131370478	132336822	966344	197	1596	1725.924242	54.12103547	0.840889826	0.016147906	+	50863	grey	0.067774593	FALSE	NTM
Region_3711	chr11	131502735	131540867	38132	11	78	97.74242424	9.490579306	0.822067657	0.054022403	-	101929653	grey	-0.192311073	FALSE	LOC101929653
Region_3712	chr1	13162245	13166360	41156	12	74	95.1060606	8.66492272	0.87049405	0.03485421	-	10192963	grey	0.080271773	FALSE	NTM-AS1

	1	1	7				1	2	4	5		7				
Region_3713	chr11	132414977	133532501	1117524	208	1660	1828.757576	53.36379084	0.794104223	0.014484367	-	4978	grey	0.03144525	FALSE	OPCML
Region_3714	chr11	133783671	133810376	26705	29	192	241.1515152	17.04134558	0.703923991	0.026566291	-	646522	grey	-0.095741627	FALSE	LINC02743
Region_3715	chr11	133896438	133956968	60530	60	362	440.1212121	27.64528901	0.734086929	0.025687159	-	22997	grey	0.052051002	FALSE	IGSF9B
Region_3716	chr11	134008215	134030167	21952	12	68	86.95454545	10.0221782	0.818243347	0.049815141	+	107984414	grey	-0.085678343	FALSE	LINC02730
Region_3717	chr11	134032216	134067936	35720	18	121	154.6363636	12.78055779	0.839251193	0.027050901	+	100128239	grey	0.204036807	FALSE	LINC02731
Region_3718	chr11	134069071	134152001	82930	20	135	166.1212121	12.82967132	0.887009683	0.030206741	+	83700	grey	-0.400493507	FALSE	JAM3
Region_3719	chr11	134150113	134225504	75391	36	256	300.6818182	15.89306486	0.933246997	0.015161356	-	23310	grey	0.056071962	FALSE	NCAPD3
Region_3720	chr11	134224671	134247788	23117	18	132	165.2272727	13.30794494	0.8078185	0.023974875	+	112936	grey	-0.023113719	FALSE	VPS26B
Region_3721	chr11	134253548	134265855	12307	17	93	125.8787879	15.65052484	0.289117719	0.023923574	+	27034	grey	0.118716783	FALSE	ACAD8
Region_3722	chr11	134274245	134319564	45319	39	198	241.5757576	18.83210075	0.824425807	0.026077559	+	112937	grey	-0.206417244	FALSE	GLB1L3
Region_3723	chr11	134331874	134378341	46467	24	151	182.8181818	15.02808559	0.846639849	0.030594963	+	89944	grey	0.170570669	FALSE	GLB1L2
Region_3724	chr11	134378504	134412242	33738	29	175	231.6363636	17.69627198	0.821464676	0.027995831	-	27087	grey	-0.023421387	FALSE	B3GAT1

Region_3725	chr11	134412284	134505665	93381	38	279	313.7272727	20.46200641	0.753255075	0.031612725	+	283177	grey	0.165283502	FALSE	B3GAT1-DT
Region_3726	chr11	134735596	134763810	28214	15	120	144.7575758	13.82312511	0.85149437	0.034590397	+	729305	grey	-0.01760766	FALSE	LINC02714
Region_3727	chr12	164664	182399	17735	10	53	81.25757576	12.1393329	0.838899823	0.051858296	-	105369592	grey	-0.210702058	FALSE	IQSEC3-AS1
Region_3728	chr12	190077	214570	24493	16	100	123.3030303	11.05088886	0.698199184	0.043907181	-	6539	grey	0.200441205	FALSE	SLC6A12
Region_3729	chr12	220621	262873	42252	25	163	191.8030303	13.34537626	0.87900876	0.035879658	-	6540	grey	0.216797489	FALSE	SLC6A13
Region_3730	chr12	280057	389320	109263	41	316	359.4545455	22.94420109	0.918185341	0.010519242	-	5927	grey	-0.164432265	FALSE	KDM5A
Region_3731	chr12	389273	442642	53369	8	50	65.93939394	7.427348192	0.711201378	0.025837961	+	84318	grey	-0.095483856	FALSE	CCDC77
Region_3732	chr12	459939	563509	103570	52	332	388.030303	25.09855632	0.806620621	0.018619571	+	283358	grey	0.096141619	FALSE	B4GALNT3
Region_3733	chr12	564296	663589	99293	66	417	492.7878788	25.02461259	0.733393817	0.021677798	-	4815	grey	0.056682491	FALSE	NINJ2
Region_3734	chr12	630858	664196	33338	21	124	161.7121212	16.66757806	0.785631982	0.021687025	+	100049716	grey	0.103112831	FALSE	NINJ2-AS1
Region_3735	chr12	674801	684127	9326	6	18	31.33333333	4.855871413	0.894061854	0.087060923	-	105369597	grey	0.368858931	FALSE	LINC02455
Region_3736	chr12	752579	911452	158873	44	278	323.8484848	22.25118315	0.74191644	0.020357162	+	65125	grey	-0.081255856	FALSE	WNK1
Region_3737	chr1	911736	991190	79454	19	116	141.106060	10.6671729	0.67557237	0.02695241	-	5893	grey	-0.15796215	FALSE	RAD52

	2						6	2	1	2						
Region_3738	chr12	990509	1495933	505424	110	799	873.7424242	36.66493215	0.888892018	0.011971591	+	23085	grey	0.256150677	FALSE	ERC1
Region_3739	chr12	1529891	1647212	117321	95	583	660.2575758	37.44506154	0.725372024	0.012695209	+	81029	grey	-0.294836444	FALSE	WNT5B
Region_3740	chr12	1565993	1594581	28588	17	104	130.0151515	12.66915385	0.527798781	0.027657485	-	144699	grey	0.165415325	FALSE	FBXL14
Region_3741	chr12	2695765	2812902	117137	54	373	430.8333333	23.89372839	0.68384637	0.015315674	-	283440	grey	0.158944526	FALSE	ITFG2-AS1
Region_3742	chr12	2740032	2746665	6633	4	20	29.1969697	4.893385479	0.933390137	0.051473912	+	107984539	grey	-0.031892609	FALSE	LINC02371
Region_3743	chr12	2794970	2805423	10453	10	42	62	8.201313216	0.298359808	0.023124803	+	2288	grey	0.175275996	FALSE	FKBP4
Region_3744	chr12	2812622	2859791	47169	18	112	144.4848485	12.84013231	0.959247775	0.022889733	+	55846	grey	0.188678632	FALSE	ITFG2
Region_3745	chr12	2849046	2857039	7993	6	36	51.75757576	6.96598462	0.958832941	0.033131683	-	101929469	grey	0.084884954	FALSE	TEX52
Region_3746	chr12	2857680	2877174	19494	10	46	63.46969697	7.789973234	0.94250708	0.043433417	-	2305	grey	-0.129240314	FALSE	FOXM1
Region_3747	chr12	2876258	2889524	13266	5	12	23.56060606	4.785004744	0.754011435	0.052819851	+	83695	grey	-0.042487591	FALSE	RHNO1
Region_3748	chr12	2877223	2941138	63915	39	216	276.1060606	22.40956443	0.81983539	0.018025895	+	7289	grey	-0.156750937	FALSE	TULP3
Region_3749	chr12	2959330	3040676	81346	75	468	530.2424242	25.41172389	0.863530163	0.016060301	+	7004	grey	0.02139244	FALSE	TEAD4

Region_3750	chr12	3077355	3286564	209209	158	1095	1231.424242	50.54991763	0.785578016	0.018766065	+	10867	grey	-0.051796365	FALSE	TSPAN9
Region_3751	chr12	3296202	3366177	69975	54	378	420.8636364	21.59763973	0.790875896	0.020032475	-	100128253	grey	0.21216569	FALSE	LOC100128253
Region_3752	chr12	3300698	3325343	24645	20	131	171.4242424	15.56333028	0.903044746	0.027524059	+	105369606	grey	-0.227196582	FALSE	LINC02827
Region_3753	chr12	3381349	3593973	212624	58	440	496.5454545	24.48433208	0.863939454	0.020727386	+	56341	grey	0.141926674	FALSE	PRMT8
Region_3754	chr12	3606633	3764819	158186	49	342	395.9848485	23.07878975	0.942321236	0.016425782	-	84766	grey	-0.176583695	FALSE	CRACR2A
Region_3755	chr12	3791047	3873448	82401	15	115	135.4090909	11.06342174	0.867121355	0.02031792	-	57097	grey	-0.275342628	FALSE	PARP11
Region_3756	chr12	3871579	3910385	38806	11	62	85.06060606	9.122885488	0.793171235	0.035721092	+	105369609	grey	-0.119750079	FALSE	PARP11-AS1
Region_3757	chr12	3908785	3958644	49859	11	70	95.07575758	9.938751827	0.892553393	0.035056199	-	105369608	grey	-0.235241118	FALSE	LOC105369608
Region_3758	chr12	4012902	4026658	13756	4	21	32.27272727	6.05753302	0.906755739	0.06230958	+	105369611	grey	-0.122210978	FALSE	LOC105369611
Region_3759	chr12	4247981	4276252	28271	16	103	127.3484848	10.4623757	0.302571747	0.014325183	-	103752584	grey	0.02958776	FALSE	CCND2-AS1
Region_3760	chr12	4269771	4305353	35582	15	92	110.4090909	10.06895804	0.286586454	0.025041547	+	894	grey	0.193671602	FALSE	CCND2
Region_3761	chr12	4273762	4405490	131728	39	263	306.2575758	17.06396167	0.678726542	0.019800657	+	57103	grey	-0.13504237	FALSE	TIGAR
Region_3762	chr1	4428156	4445815	17659	7	39	58.7878787	8.39141530	0.91727826	0.04302062	-	2251	grey	-0.23300896	FALSE	FGF6

	2						9	5	7	2						
Region_3763	chr12	4487735	4538508	50773	10	66	90.6969697	10.41887296	0.634624725	0.048450512	-	57102	grey	-0.145233371	FALSE	C12orf4
Region_3764	chr12	4538798	4560047	21249	4	18	33.86363636	5.571593788	0.968442	0.035051502	+	10635	grey	-0.004917763	FALSE	RAD51AP1
Region_3765	chr12	4562208	4615302	53094	8	44	58.90909091	7.087860202	0.731628546	0.037589356	+	8798	grey	-0.250729509	FALSE	DYRK4
Region_3766	chr12	4604944	4774184	169240	37	261	296.0909091	19.99748236	0.832803567	0.017157487	+	4704	grey	0.052185129	FALSE	NDUFA9
Region_3767	chr12	4615508	4649051	33543	12	80	101.8181818	10.51286258	0.694695276	0.032247362	-	10566	grey	0.094592182	FALSE	AKAP3
Region_3768	chr12	4669741	4851003	181262	41	279	331.4545455	22.82588397	0.788579773	0.022294332	+	26290	grey	0.140910971	FALSE	GALNT8
Region_3769	chr12	4700417	4720102	19685	5	22	37.06060606	6.025276982	0.937055524	0.063830147	-	101929549	grey	-0.216812226	FALSE	GAU1
Region_3770	chr12	4804499	4830747	26248	10	58	81.96969697	10.37299704	0.450464179	0.033297053	-	105369614	grey	0.058669608	FALSE	
Region_3771	chr12	4809334	4851927	42593	14	92	115.4242424	12.29641675	0.560793759	0.03241649	+	3742	grey	0.15571956	FALSE	KCNA6
Region_3772	chr12	4909867	5026012	116145	38	270	309.8484848	15.5169807	0.810924031	0.024823799	+	100507560	grey	-0.087810288	FALSE	LOC100507560
Region_3773	chr12	4909901	5011745	101844	28	201	239.9090909	13.50068633	0.787999506	0.026930469	+	3736	grey	-0.082016294	FALSE	KCNA1
Region_3774	chr12	5226196	5244199	18003	3	22	31.09090909	4.891837071	0.950213331	0.040616989	-	101929584	grey	0.060998057	FALSE	LINC02443

Region_3775	chr12	5290480	5383653	93173	17	110	137.1666667	12.53374931	0.924528806	0.039084436	+	105369617	grey	-0.448189714	FALSE	LOC105369617
Region_3776	chr12	5432108	5521536	89428	15	123	148.6515152	12.48321017	0.655054383	0.036334009	+	4908	grey	0.036754207	FALSE	NTF3
Region_3777	chr12	5531869	5946232	414363	87	648	719.4545455	30.02162391	0.881148998	0.01853349	-	57101	grey	0.229339125	FALSE	ANO2
Region_3778	chr12	5948877	6124770	175893	57	432	478.7575758	20.21732738	0.789605443	0.0253172	-	7450	grey	0.262531302	FALSE	VWF
Region_3779	chr12	6199715	6238271	38556	19	130	154.0757576	12.70167839	0.562995473	0.026016084	+	928	grey	0.139445187	FALSE	CD9
Region_3780	chr12	6310436	6328506	18070	7	30	43.90909091	6.567348775	0.537559595	0.052082047	+	55200	grey	0.194061892	FALSE	PLEKHG6
Region_3781	chr12	6328757	6342114	13357	11	56	80.43939394	10.16953034	0.429127522	0.030328232	-	7132	grey	-0.058805434	FALSE	TNFRSF1A
Region_3782	chr12	6346843	6377730	30887	15	70	102.9848485	10.74242835	0.697489862	0.023040935	-	6337	grey	-0.099458268	FALSE	SCNN1A
Region_3783	chr12	6375045	6391571	16526	12	60	76.09090909	7.844110525	0.675300813	0.041644045	+	4055	grey	0.122835025	FALSE	LTBR
Region_3784	chr12	6439001	6451607	12606	4	20	30.98484848	5.13657301	0.840917437	0.081714208	-	678655	grey	-0.125680263	FALSE	CD27-AS1
Region_3785	chr12	6451690	6466517	14827	8	48	60.83333333	7.173848518	0.709930374	0.063001299	+	55080	grey	-0.093714905	FALSE	TAPBPL
Region_3786	chr12	6462237	6470677	8440	5	28	38.45454545	5.50015893	0.696409516	0.051399775	-	6843	grey	-0.015374383	FALSE	VAMP1
Region_3787	chr1	6491886	6493841	1955	5	18	34.4090909	5.95877445	0.21331794	0.04057449	-	51258	grey	-0.01395998	FALSE	MRPL51

	2						1	5	7	4						
Region_3788	chr12	6493356	6531955	38599	25	147	180.469697	17.08100132	0.790096358	0.020750584	+	9918	grey	-0.067511332	FALSE	NCAPD2
Region_3789	chr12	6534512	6538374	3862	6	20	32	6.811754546	0.134077064	0.034768187	+	2597	grey	0.148342494	FALSE	GAPDH
Region_3790	chr12	6538961	6556073	17112	13	65	89.43939394	10.89407572	0.752675497	0.040006551	-	25900	grey	-0.076504865	FALSE	IFFO1
Region_3791	chr12	6556863	6587392	30529	19	106	135.2575758	11.6266413	0.888730631	0.021574224	-	4839	grey	0.036183569	FALSE	NOP2
Region_3792	chr12	6556886	6608430	51544	38	231	271.0757576	15.46242155	0.776863545	0.015520872	-	1108	grey	0.140143259	FALSE	CHD4
Region_3793	chr12	6618835	6635960	17125	13	69	100.1212121	12.72975334	0.773471343	0.042686175	-	57121	grey	0.091437082	FALSE	LPAR5
Region_3794	chr12	6638075	6647433	9358	10	50	69.57575758	8.616728999	0.734610064	0.056662166	-	84519	grey	0.031803222	FALSE	ACRBP
Region_3795	chr12	6650301	6663142	12841	9	61	81.60606061	9.289992611	0.936482227	0.036538843	-	51147	grey	0.075792114	FALSE	ING4
Region_3796	chr12	6666477	6689572	23095	4	18	33.95454545	5.834728438	0.877566857	0.058723202	-	171017	grey	-0.292454599	FALSE	ZNF384
Region_3797	chr12	6724014	6731875	7861	9	45	67.92424242	11.29780184	0.854317615	0.023281524	+	50813	grey	-0.038686761	FALSE	COPS7A
Region_3798	chr12	6747996	6767475	19479	13	63	79.25757576	9.020166476	0.536320097	0.043629279	-	8079	grey	0.00826603	FALSE	MLF2
Region_3799	chr12	6765516	6770952	5436	9	54	70.3030303	9.255301511	0.453172739	0.052009324	+	5763	grey	-0.048581921	FALSE	PTMS

Region_3800	chr12	6772512	6778455	5943	7	26	39.1969697	6.780559761	0.57969034	0.057638556	+	3902	grey	-0.074650585	FALSE	LAG3
Region_3801	chr12	6786858	6820799	33941	25	115	172.5151515	22.99648296	0.959578544	0.02362029	+	920	grey	0.029568239	FALSE	CD4
Region_3802	chr12	6821624	6829972	8348	8	30	47.59090909	8.22187939	0.549287252	0.054980898	+	27239	grey	0.081553305	FALSE	GPR162
Region_3803	chr12	6828407	6839847	11440	8	40	54.43939394	6.209493446	0.729450716	0.025097888	+	10536	grey	-0.107626324	FALSE	P3H3
Region_3804	chr12	6839954	6847393	7439	6	26	42.83333333	5.744785819	0.816866443	0.043124858	+	2784	grey	-0.157026155	FALSE	GNB3
Region_3805	chr12	6844793	6851292	6499	4	19	36.98484848	6.249904428	0.755928878	0.056201158	-	83461	grey	0.02180822	FALSE	CDCA3
Region_3806	chr12	6852148	6866632	14484	6	22	35	5.969151467	0.945938381	0.045160126	+	8078	grey	-0.061573667	FALSE	USP5
Region_3807	chr12	6867119	6870948	3829	6	26	37.42424242	6.67731085	0.179061399	0.028454363	+	7167	grey	0.036355079	FALSE	TPI1
Region_3808	chr12	6870935	6882287	11352	15	89	109.8484848	9.893653637	0.780346216	0.028232182	-	84727	grey	-0.123846224	FALSE	SPSB2
Region_3809	chr12	6873569	6914241	40672	37	238	282.5909091	20.07599199	0.818458963	0.014932946	+	10233	grey	-0.020891657	FALSE	LRRC23
Region_3810	chr12	6875764	6889358	13594	13	77	102.030303	11.66450196	0.971572344	0.017204398	-	105369632	grey	0.054685105	FALSE	LOC105369632
Region_3811	chr12	6913745	6923698	9953	7	36	46.81818182	6.802508793	0.645434922	0.055548683	+	2026	grey	-0.246450568	FALSE	ENO2
Region_3812	chr1	6924463	6942321	17858	32	169	226.939393	25.0242213	0.31576440	0.02030575	+	1822	grey	-0.12562030	FALSE	ATN1

	2						9	7	3	3				4		
Region_3813	chr12	6942978	6946003	3025	4	19	33.87878788	5.606356699	0.357046604	0.063930091	+	113246	grey	-0.121472661	FALSE	C12orf57
Region_3814	chr12	6946468	6961316	14848	17	88	115.2878788	11.931555	0.49220626	0.036719772	+	5777	grey	-0.007817957	FALSE	PTPN6
Region_3815	chr12	6965327	6970780	5453	6	29	52.83333333	8.841481486	0.481876365	0.026001318	-	11331	grey	0.241689372	FALSE	PHB2
Region_3816	chr12	6970913	6997428	26515	13	69	92.54545455	9.779384625	0.951862729	0.024407967	+	10436	grey	0.061954553	FALSE	EMG1
Region_3817	chr12	6976185	7018477	42292	22	121	161.469697	15.72378864	0.921475569	0.019050848	-	10162	grey	-0.025750167	FALSE	LPCAT3
Region_3818	chr12	6988259	7071032	82773	58	400	452.0151515	25.59296323	0.887389083	0.015788474	+	716	grey	-0.159916763	FALSE	C1S
Region_3819	chr12	7304072	7328719	24647	6	46	64.54545455	9.444871328	0.954814396	0.028819729	+	341392	grey	-0.16804405	FALSE	ACSM4
Region_3820	chr12	7346685	7479897	133212	14	88	116.7575758	9.96618993	0.881363457	0.037678594	-	283316	grey	0.092475707	FALSE	CD163L1
Region_3821	chr12	7649400	7665908	16508	4	14	22.10606061	4.332804343	0.922070361	0.083136914	-	339	grey	0.052077603	FALSE	APOBEC1
Region_3822	chr12	7711433	7717559	6126	4	20	31.46969697	6.474137179	0.862177326	0.073636445	+	359787	grey	0.014043871	FALSE	DPPA3
Region_3823	chr12	7729383	7751605	22222	8	29	46.37878788	7.759391633	0.955121951	0.040630831	-	170482	grey	-0.115224016	FALSE	CLEC4C
Region_3824	chr12	7787794	7799146	11352	10	54	74.77272727	8.855759455	0.706752047	0.044724688	+	79923	grey	-0.067662072	FALSE	NANOG

Region_3825	chr12	7812512	7891148	78636	40	263	308.9242424	19.75566957	0.768837512	0.025578468	-	144195	grey	0.153389126	FALSE	SLC2A14
Region_3826	chr12	7919230	7936187	16957	7	40	57.04545455	8.336078802	0.94397825	0.030876689	-	6515	grey	-0.369622448	FALSE	SLC2A3
Region_3827	chr12	8032716	8055517	22801	7	33	51.83333333	6.622998004	0.61512415	0.057516387	+	55810	grey	-0.119588236	FALSE	FOXJ2
Region_3828	chr12	8056844	8066359	9515	6	36	47.51515152	5.941360694	0.937031506	0.046662145	-	719	grey	-0.078201933	FALSE	C3AR1
Region_3829	chr12	8076939	8121795	44856	9	61	86.1969697	10.362981	0.839665699	0.026904066	+	25977	grey	0.066140158	FALSE	NECAP1
Region_3830	chr12	8096207	8138607	42400	12	71	93.53030303	10.3510976	0.760658779	0.042204583	+	50856	grey	-0.211360289	FALSE	CLEC4A
Region_3831	chr12	8138140	8188537	50397	11	56	76.37878788	10.2097467	0.928032577	0.033561631	+	440077	grey	0.080090923	FALSE	ZNF705A
Region_3832	chr12	8180209	8216151	35942	10	42	79.65151515	12.19267425	0.928940681	0.032852352	+	440078	grey	0.095831326	FALSE	FAM66C
Region_3833	chr12	8221260	8227618	6358	7	31	59.84848485	18.96899691	0.878231361	0.046072663	-	55138	grey	-0.05191912	FALSE	FAM90A1
Region_3834	chr12	8232512	8242948	10436	10	57	87.51515152	19.21557255	0.572939874	0.035227272	-	653113	grey	0.194189117	FALSE	FAM86FP
Region_3835	chr12	8235415	8242564	7149	5	30	51.77272727	10.8454818	0.585844031	0.027957278	+	101927905	grey	0.012870387	FALSE	LINC02449
Region_3836	chr12	8243147	8342048	98901	26	176	211.0606061	13.28142577	0.765996384	0.025143372	+	112268090	grey	-0.061650985	FALSE	LOC112268090
Region_3837	chr1	8254527	8308855	54328	16	124	146.181818	11.7838338	0.82896416	0.03774651	-	10798717	grey	-0.07229574	FALSE	LOC107987171

	2						2	9	1			1		3		
Region_3838	chr12	8295986	8396803	100817	26	175	206.5757576	15.58604959	0.722751152	0.03054287	-	389634	grey	0.112089979	FALSE	LINC00937
Region_3839	chr12	8320263	8369597	49334	4	24	36.75757576	6.373166357	0.936124616	0.051923918	+	101927966	grey	-0.259175594	FALSE	LOC101927966
Region_3840	chr12	8637346	8662888	25542	6	37	51.62121212	7.548336855	0.919432911	0.041394117	-	8076	grey	-0.058210848	FALSE	MFAP5
Region_3841	chr12	8681600	8783095	101495	24	162	202.1212121	18.04227101	0.88553461	0.018435172	+	57494	grey	-0.394249504	FALSE	RIMKLB
Region_3842	chr12	8822621	8887001	64380	26	187	225.3333333	15.31347655	0.867973189	0.01894559	+	144568	grey	-0.051508408	FALSE	A2ML1
Region_3843	chr12	8913896	8941467	27571	8	53	67.46969697	7.348929167	0.856485993	0.04887772	+	1911	grey	0.01782783	FALSE	PHC1
Region_3844	chr12	8940361	8949761	9400	7	28	51.39393939	8.144613676	0.7102714	0.059098707	-	4074	grey	-0.035613445	FALSE	M6PR
Region_3845	chr12	8950044	9010760	60716	14	91	116.9545455	10.36478033	0.820223477	0.019435571	+	10219	grey	-0.092703437	FALSE	KLRG1
Region_3846	chr12	9067664	9116229	48565	4	20	30.53030303	5.594556962	0.9297912	0.059988989	-	2	grey	-0.111483593	FALSE	A2M
Region_3847	chr12	9148840	9208395	59555	8	55	75.81818182	9.111124377	0.927984391	0.045378057	-	5858	grey	-0.063777182	FALSE	PZP
Region_3848	chr12	9277235	9313241	36006	6	20	38.78787879	8.18350151	0.732482241	0.078224158	+	642846	grey	0.360822791	FALSE	LOC642846
Region_3849	chr12	9347056	9400387	53331	4	21	39.06060606	6.002252879	0.841733858	0.054286907	+	101930452	grey	0.095811532	FALSE	LINC02367

Region_3850	chr12	9418673	9448229	29556	10	49	77.74242424	13.86674007	0.797738341	0.034432589	-	440081	grey	0.132987193	FALSE	DDX12P
Region_3851	chr12	9448295	9633828	185533	22	141	172.1969697	14.53189563	0.790041211	0.025192822	+	408186	grey	0.304696493	FALSE	LOC408186
Region_3852	chr12	9448498	9658412	209914	22	132	165.5757576	13.79973311	0.790181436	0.025541193	+	374443	grey	0.314153905	FALSE	LOC374443
Region_3853	chr12	9594551	9607916	13365	7	36	52.5	6.656980142	0.909619304	0.045284998	-	3820	grey	0.262596537	FALSE	KLRB1
Region_3854	chr12	9664969	9699553	34584	3	17	25.45454545	4.557940892	0.914607447	0.0806938	+	29121	grey	0.040819229	FALSE	CLEC2D
Region_3855	chr12	9702182	9733299	31117	4	19	29.53030303	5.16562513	0.918166219	0.056162629	-	160365	grey	0.00011623	FALSE	CLECL1P
Region_3856	chr12	9881489	9895833	14344	5	21	38.33333333	6.205043441	0.969512567	0.029815514	+	100431172	grey	-0.231655322	FALSE	KLRF2
Region_3857	chr12	9951316	9995694	44378	4	28	39.60606061	5.471264199	0.859119557	0.103310724	+	160364	grey	-0.239267841	FALSE	CLEC12A
Region_3858	chr12	10030678	10066031	35353	8	41	68.24242424	9.104623688	0.848186955	0.054546346	+	283420	grey	0.05875604	FALSE	CLEC9A
Region_3859	chr12	10158301	10172138	13837	4	15	25.43939394	4.282805651	0.732588886	0.069812468	-	4973	grey	-0.057890044	FALSE	OLR1
Region_3860	chr12	10170542	10191804	21262	4	24	37.75757576	6.44995076	0.905076434	0.062720207	+	120939	grey	0.155404089	FALSE	TMEM52B
Region_3861	chr12	10226058	10329608	103550	23	157	182.2727273	13.02251949	0.885743964	0.021205314	+	3824	grey	-0.047306092	FALSE	KLRD1
Region_3862	chr1	10363638	10398506	34868	9	46	63.5757575	7.57132364	0.65066852	0.06996061	+	10192810	grey	-0.04609367	FALSE	KLRK1-AS1

	2						8	4	7	2		0		9		
Region_3863	chr12	10372353	10391874	19521	4	18	31.56060606	5.409759807	0.870996293	0.07732002	-	22914	grey	-0.163152053	FALSE	KLRK1
Region_3864	chr12	10372353	10410146	37793	8	45	64.39393939	7.697909382	0.507243313	0.073916418	-	100528032	grey	0.054367317	FALSE	KLRC4-KLRK1
Region_3865	chr12	10699089	10723323	24234	5	23	41.75757576	7.161995008	0.766431817	0.052977103	-	8531	grey	-0.162413837	FALSE	YBX3
Region_3866	chr12	11399381	11486708	87327	4	16	27.78787879	4.470467714	0.938152466	0.051543407	-	440084	grey	0.203521862	FALSE	LOC440084
Region_3867	chr12	11548030	11590369	42339	4	20	36.92424242	5.852837855	0.921367779	0.05021839	+	338817	grey	-0.012295598	FALSE	LINC01252
Region_3868	chr12	11649674	11895377	245703	60	460	516.9393939	25.42492949	0.71227686	0.023721302	+	2120	grey	0.293224637	FALSE	ETV6
Region_3869	chr12	12611827	12645108	33281	5	23	34.63636364	5.166865926	0.780043536	0.029100574	+	1389	grey	-0.202698246	FALSE	CREBL2
Region_3870	chr12	12660890	12696207	35317	3	11	19.96969697	4.895742882	0.981727476	0.042745752	-	2842	grey	0.201700579	FALSE	GPR19
Region_3871	chr12	12685498	12722369	36871	18	152	177.3333333	15.49325743	0.335556715	0.015486033	+	1027	grey	0.209803211	FALSE	CDKN1B
Region_3872	chr12	12725917	12814199	88282	17	118	143.5454545	12.01304652	0.812692231	0.030116739	+	81575	grey	-0.131017077	FALSE	APOLD1
Region_3873	chr12	12725929	12829981	104052	18	125	152.3484848	12.24681111	0.819599769	0.0296593	+	51202	grey	-0.161902286	FALSE	DDX47
Region_3874	chr12	12891559	12917937	26378	8	45	61.93939394	9.190092973	0.863266247	0.026056208	+	9052	grey	0.122057804	FALSE	GPRC5A

Region_3875	chr12	12927712	13040679	112967	44	344	393.3939394	22.2004011	0.837928333	0.016487314	+	100506314	grey	0.300113395	FALSE	GPRC5D-AS1
Region_3876	chr12	12940575	12952170	11595	3	22	33.04545455	6.357144541	0.91905817	0.0539055	-	55507	grey	0.035758685	FALSE	GPRC5D
Region_3877	chr12	12974870	13000265	25395	3	21	29.33333333	4.840004238	0.729796002	0.064868219	-	50865	grey	0.477705481	FALSE	HEBP1
Region_3878	chr12	13000420	13004830	4410	5	25	44.89393939	8.246451522	0.253471931	0.068756557	+	93164	grey	0.079647691	FALSE	HTR7P1
Region_3879	chr12	13044381	13142521	98140	24	181	207.0151515	13.56295001	0.707805912	0.022003735	+	57613	grey	0.161029825	FALSE	FAM234B
Region_3880	chr12	13083532	13103683	20151	7	62	76.45454545	8.105424239	0.593404648	0.031187766	-	83445	grey	0.214827106	FALSE	GSG1
Region_3881	chr12	13196723	13219941	23218	5	25	37.07575758	5.865966	0.923192319	0.046984806	+	2012	grey	0.065187519	FALSE	EMP1
Region_3882	chr12	13437942	13982002	544060	50	382	440.5909091	23.58420415	0.826553353	0.021324434	-	2904	grey	0.053585311	FALSE	GRIN2B
Region_3883	chr12	14365676	14502931	137255	23	151	175.0757576	11.77390936	0.94818223	0.019633669	+	55729	grey	-0.009513732	FALSE	ATF7IP
Region_3884	chr12	14503661	14567883	64222	9	52	66.95454545	8.232079498	0.772157292	0.047008432	-	79887	grey	0.078260462	FALSE	PLBD1
Region_3885	chr12	14530124	14543575	13451	3	15	24.06060606	4.391876374	0.706327034	0.097800983	+	101928317	grey	0.142277693	FALSE	LOC101928317
Region_3886	chr12	14567393	14906586	339193	50	353	406.3939394	20.61954173	0.80295541	0.012879708	+	144608	grey	0.083365426	FALSE	C12orf60
Region_3887	chr1	14567750	14619298	51548	5	33	44.9090909	5.70216496	0.95984175	0.02888474	+	10192829	grey	-0.14095969	FALSE	PLBD1-AS1

	2						1	7	7	7		0		8		
Region_3888	chr1<br>2	14612632	14696599	83967	11	64	88.1818181<br>8	10.3178845<br>3	0.84518640<br>8	0.03218277<br>8	-	2984	grey	-0.13055506<br>9	FALSE	GUCY2C
Region_3889	chr1<br>2	14784582	14803486	18904	3	15	24.5151515<br>2	4.42455534<br>2	0.96714382	0.04115803<br>1	-	51729	grey	0.239910515	FALSE	WBP11
Region_3890	chr1<br>2	14804650	14814182	9532	3	11	19.3030303	3.95345884<br>4	0.96843817	0.04577020<br>7	-	440087	grey	0.397718872	FALSE	SMCO3
Region_3891	chr1<br>2	14914039	14938537	24498	4	13	28.3939393<br>9	6.65612219<br>7	0.93513270<br>6	0.06774368	-	121506	grey	0.03106571	FALSE	ERP27
Region_3892	chr1<br>2	14942031	14961728	19697	6	35	49.1060606<br>1	6.56594662	0.60471236<br>9	0.04646068<br>4	-	397	grey	-0.07099992<br>5	FALSE	ARHGDIB
Region_3893	chr1<br>2	15107783	15221417	113634	10	58	76.3636363<br>6	8.96426471<br>9	0.73899736<br>2	0.05525720<br>9	-	85004	grey	0.343977656	FALSE	RERG
Region_3894	chr1<br>2	15121065	15348675	227610	16	100	126	10.8258948<br>8	0.76388855<br>6	0.03584514<br>5	-	10536967<br>3	grey	0.082455861	FALSE	LOC105369673
Region_3895	chr1<br>2	15322257	15602175	279918	22	139	170.5	13.6091032<br>9	0.86583849	0.02555506<br>8	+	5800	grey	-0.21341683<br>3	FALSE	PTPRO
Region_3896	chr1<br>2	15620134	15882329	262195	27	190	219.954545<br>5	16.0838493<br>5	0.80753388<br>2	0.02764662<br>9	-	2059	grey	0.031882222	FALSE	EPS8
Region_3897	chr1<br>2	15882387	15903478	21091	6	32	51.1212121<br>2	6.28187907<br>1	0.62696405<br>7	0.0297203	+	11171	grey	-0.08794563<br>4	FALSE	STRAP
Region_3898	chr1<br>2	15911302	16037381	126079	15	83	111.166666<br>7	12.9312779<br>2	0.88837181<br>7	0.02035516	+	51071	grey	-0.06866641<br>6	FALSE	DERA
Region_3899	chr1<br>2	16188485	16277685	89200	8	51	70.7121212<br>1	9.75510146<br>9	0.82571230<br>5	0.08572416<br>5	-	729025	grey	-0.22845748<br>6	FALSE	SLC15A5

Region_3900	chr12	16347142	16609259	262117	12	70	93.27272727	10.29793923	0.704846131	0.027935354	+	4257	grey	0.000336979	FALSE	MGST1
Region_3901	chr12	16548372	16610594	62222	3	11	19.42424242	3.746015754	0.337743247	0.051266315	-	55885	grey	-0.015605213	FALSE	LMO3
Region_3902	chr12	17509353	17590049	80696	5	23	32.5	5.949143442	0.869801541	0.075332102	-	105369678	grey	-0.018347021	FALSE	LINC02378
Region_3903	chr12	18080869	18320107	239238	19	117	140.8030303	11.09430859	0.865053132	0.046735684	-	79785	grey	0.405274073	FALSE	RERGL
Region_3904	chr12	18242961	18737878	494917	35	278	314.7272727	17.1523001	0.82628517	0.028557778	+	5288	grey	0.358608316	FALSE	PIK3C2G
Region_3905	chr12	18683169	18738100	54931	7	56	76.12121212	9.956080711	0.734910279	0.06465866	-	89869	grey	0.451895347	FALSE	PLCZ1
Region_3906	chr12	19129733	19376400	246667	40	240	290.5909091	19.4184506	0.88148821	0.014204868	+	54477	grey	0.128102147	FALSE	PLEKHA5
Region_3907	chr12	19404045	19720801	316756	79	536	588.2121212	26.45781266	0.912498761	0.013650795	+	121536	grey	0.034560507	FALSE	AEBP2
Region_3908	chr12	20014780	20098868	84088	8	45	56.89393939	5.899488343	0.832876417	0.069021198	+	100506393	grey	-0.456882684	FALSE	LINC02398
Region_3909	chr12	20368537	20688583	320046	29	186	222.9090909	17.2544106	0.892467587	0.025592481	+	5139	grey	0.153619524	FALSE	PDE3A
Region_3910	chr12	20695332	20753386	58054	3	19	29.84848485	5.195389744	0.897942355	0.067651789	+	53919	grey	0.108665708	FALSE	SLCO1C1
Region_3911	chr12	20810702	20916911	106209	5	15	28.96969697	6.258458146	0.761448369	0.072355894	+	28234	grey	0.088632515	FALSE	SLCO1B3
Region_3912	chr1	20815674	21141623	325949	21	125	148.378787	12.0317083	0.86789547	0.03069011	+	11507289	grey	0.111059665	FALSE	SLCO1B3-SLCO1B7

	2						9		3	5		6				
Region_3913	chr12	21047179	21239796	192617	8	42	60.78787879	7.271684074	0.886738677	0.068346881	+	10599	grey	-0.11005594	FALSE	SLCO1B1
Region_3914	chr12	21264600	21419594	154994	6	37	46.96969697	6.209099271	0.836566057	0.076059441	-	6579	grey	0.078350359	FALSE	SLCO1A2
Region_3915	chr12	21437615	21471250	33635	5	24	37	6.056147544	0.788710086	0.019074376	+	79912	grey	0.376490387	FALSE	PYROXD1
Region_3916	chr12	21536107	21657798	121691	23	167	190.8787879	13.13306473	0.859076887	0.024037874	-	2998	grey	0.36316795	FALSE	GYS2
Region_3917	chr12	21635342	21757857	122515	16	105	127.8939394	10.98268398	0.819846314	0.026527211	-	3945	grey	0.220387229	FALSE	LDHB
Region_3918	chr12	21654657	21757831	103174	14	85	108.4848485	9.831256942	0.807482362	0.028436601	-	102724261	grey	0.179355979	FALSE	LOC102724261
Region_3919	chr12	21662313	21766705	104392	10	63	81.10606061	8.552334432	0.845894577	0.030760421	+	105369689	grey	0.05739419	FALSE	LOC105369689
Region_3920	chr12	21797389	21942543	145154	10	58	73.87878788	8.907240186	0.855930691	0.048719079	-	10060	grey	-0.179505032	FALSE	ABCC9
Region_3921	chr12	22066419	22336200	269781	35	256	296.030303	15.78506568	0.764657619	0.021187321	-	6489	grey	-0.082692482	FALSE	ST8SIA1
Region_3922	chr12	22107159	22287194	180035	25	180	215.969697	13.84415422	0.868472514	0.023326052	-	107984497	grey	0.10047537	FALSE	SULT6B2P
Region_3923	chr12	22409237	22618868	209631	23	164	197.0757576	13.7374397	0.881412437	0.018832475	+	105369692	grey	0.044032574	FALSE	C2CD5-AS1
Region_3924	chr12	22448583	22544546	95963	9	50	66.75757576	7.736150118	0.970269411	0.025426721	-	9847	grey	0.077619398	FALSE	C2CD5

Region_3925	chr12	22699859	22888933	189074	22	164	193.530303	12.2853505	0.905755468	0.029734055	+	107984516	grey	-0.00212727	FALSE	LINC02955
Region_3926	chr12	22964577	23191587	227010	18	120	146.7424242	12.82702748	0.804339117	0.043665422	+	101928441	grey	0.315996371	FALSE	
Region_3927	chr12	23529504	24562544	1033040	92	697	752.0757576	27.36494704	0.863899475	0.016656044	-	6660	grey	-0.269710037	FALSE	SOX5
Region_3928	chr12	24566964	24584168	17204	5	30	41.13636364	5.296984232	0.930214818	0.049690106	-	144360	grey	0.063551269	FALSE	LINC00477
Region_3929	chr12	24810024	24949101	139077	15	110	129.0909091	11.3811278	0.758291262	0.020145157	-	586	grey	0.20376323	FALSE	BCAT1
Region_3930	chr12	25004342	25108335	103993	15	99	127.5757576	10.14670937	0.741274879	0.032935331	+	4033	grey	0.169918982	FALSE	IRAG2
Region_3931	chr12	25108289	25195162	86873	10	58	75.40909091	8.546663357	0.90361395	0.038573671	-	55259	grey	-0.052598039	FALSE	DNAI7
Region_3932	chr12	25205246	25250936	45690	3	20	28.6969697	5.271022954	0.947890441	0.049773576	-	3845	grey	0.155441614	FALSE	KRAS
Region_3933	chr12	25409307	25648579	239272	26	177	204.1818182	13.70559581	0.922454231	0.027817097	-	160492	grey	0.048380373	FALSE	LMNTD1
Region_3934	chr12	25936683	25959765	23082	4	13	26.75757576	4.955371929	0.459835622	0.093068405	-	100506451	grey	-0.079495013	FALSE	RASSF8-AS1
Region_3935	chr12	25958232	26079892	121660	13	72	95.54545455	10.64703905	0.806257671	0.03324951	+	11228	grey	-0.055250527	FALSE	RASSF8
Region_3936	chr12	26120030	26125037	5007	4	17	31.46969697	5.06942478	0.012474857	0.020005104	-	79365	grey	0.103229139	FALSE	BHLHE41
Region_3937	chr1	26121991	26252920	130929	26	188	228.045454	16.5607801	0.54486000	0.02359366	+	8082	grey	0.437361353	FALSE	SSPN

	2						5	9	5	6						
Region_3938	chr12	26122052	26304645	182593	35	261	302.0606061	20.12643022	0.55994577	0.020999913	+	105369705	grey	0.387568313	FALSE	LOC105369705
Region_3939	chr12	26211164	26335856	124692	24	175	207.030303	14.84377072	0.65721545	0.024717818	-	101928554	grey	0.294312083	FALSE	ITPR2-AS1
Region_3940	chr12	26335352	26833194	497842	55	436	482.8636364	21.41378877	0.858832379	0.020489514	-	3709	grey	-0.582723002	FALSE	ITPR2
Region_3941	chr12	26905181	26938326	33145	5	28	40.43939394	5.889047952	0.396139737	0.038824907	-	55726	grey	-0.080415248	FALSE	INTS13
Region_3942	chr12	26938470	26966650	28180	12	74	103.0757576	11.71496164	0.324668562	0.013589207	+	26127	grey	0.254136576	FALSE	FGFR1OP2
Region_3943	chr12	26971579	27014434	42855	16	115	140.7424242	10.63421272	0.842367639	0.032142748	-	51768	grey	0.058563206	FALSE	TM7SF3
Region_3944	chr12	27022546	27066240	43694	5	31	47.84848485	7.023570042	0.980337366	0.018555324	+	9412	grey	0.051781443	FALSE	MED21
Region_3945	chr12	27243968	27325959	81991	10	55	75.65151515	8.232192762	0.768488692	0.030205324	+	23012	grey	-0.306868613	FALSE	STK38L
Region_3946	chr12	27332836	27425289	92453	12	85	108.8030303	10.26752865	0.76898058	0.025628456	+	56938	grey	-0.03949788	FALSE	BMAL2
Region_3947	chr12	27389789	27446625	56836	11	74	95.59090909	10.00150338	0.846636321	0.034241458	-	101928646	grey	-0.099812243	FALSE	BMAL2-AS1
Region_3948	chr12	27446736	27502185	55449	5	24	40.25757576	6.320444498	0.94135459	0.051425379	+	341346	grey	0.019329874	FALSE	SMCO2
Region_3949	chr12	27523431	27695564	172133	27	209	243.4242424	16.08062437	0.856148223	0.023200143	+	8496	grey	-0.19472364	FALSE	PPFIBP1

Region_3950	chr12	27696388	27710803	14415	5	25	39.36363636	6.520114118	0.730051984	0.053406456	-	107984461	grey	-0.0167197	FALSE	MRPS35-DT
Region_3951	chr12	27710822	27756295	45473	6	38	53.75757576	7.524443462	0.884373512	0.053347669	+	60488	grey	0.134212926	FALSE	MRPS35
Region_3952	chr12	27762427	27780236	17809	7	38	56.5	7.269006916	0.588984284	0.035356552	-	100287284	grey	-0.001415145	FALSE	MANSC4
Region_3953	chr12	27780048	27803040	22992	6	33	45.96969697	6.635498235	0.487152585	0.019625352	+	57542	grey	0.062459727	FALSE	KLHL42
Region_3954	chr12	27958084	27972733	14649	8	53	68.04545455	6.893546186	0.527648454	0.061140174	-	5744	grey	0.09283802	FALSE	PTHLH
Region_3955	chr12	28133249	28573446	440197	27	191	222.3484848	17.79152125	0.9085543	0.034780881	+	55297	grey	-0.276897937	FALSE	CCDC91
Region_3956	chr12	28549063	28579943	30880	3	12	28.78787879	4.843759352	0.892306826	0.081802811	+	101928705	grey	0.354228304	FALSE	
Region_3957	chr12	29149016	29341121	192105	22	141	184.5606061	13.07508351	0.732800494	0.025058018	+	55711	grey	-0.610873659	FALSE	FAR2
Region_3958	chr12	29277955	29317848	39893	8	50	69.43939394	8.443711885	0.971241663	0.024393864	-	100506606	grey	0.022144354	FALSE	LOC100506606
Region_3959	chr12	29337352	29381189	43837	4	19	31.68181818	5.011246094	0.717056071	0.051812483	-	51290	grey	-0.19187304	FALSE	ERGIC2
Region_3960	chr12	29389294	29487488	98194	8	41	63.59090909	9.530481096	0.953231469	0.027217797	+	101055625	grey	-0.136574676	FALSE	OVCH1-AS1
Region_3961	chr12	29412474	29497686	85212	7	36	53.54545455	8.654357405	0.960291593	0.032466317	-	341350	grey	-0.011370619	FALSE	OVCH1
Region_3962	chr1	29500840	29784759	283919	44	325	379.651515	21.2678829	0.77833974	0.02277756	-	83857	grey	0.222492451	FALSE	TMTC1

	2						2	2	6							
Region_3963	chr12	30230779	30296707	65928	5	19	31.45454545	5.706088026	0.809298807	0.103297601	-	105369717	grey	-0.263427214	FALSE	LINC02386
Region_3964	chr12	30628988	30695869	66881	10	66	87.72727273	10.33968524	0.90010179	0.026935228	-	10526	grey	-0.072427548	FALSE	IPO8
Region_3965	chr12	30709552	30823302	113750	11	68	84.3030303	8.108817038	0.542388371	0.038234502	-	65981	grey	-0.037280431	FALSE	CAPRIN2
Region_3966	chr12	30755167	30879268	124101	24	149	186.3333333	11.59221666	0.570875231	0.024260255	+	100287314	grey	-0.045136831	FALSE	LINC00941
Region_3967	chr12	30926428	30996602	70174	12	78	101.4090909	10.639516	0.792303827	0.043650294	+	441631	grey	0.04579126	FALSE	TSPAN11
Region_3968	chr12	31019434	31073847	54413	9	56	74.95454545	10.09406805	0.872999061	0.033507717	-	100506660	grey	0.030058105	FALSE	DDX11-AS1
Region_3969	chr12	31073860	31104799	30939	17	83	114.5606061	13.39704765	0.894106641	0.051680188	+	1663	grey	-0.125658737	FALSE	DDX11
Region_3970	chr12	31382226	31591136	208910	45	311	362.0151515	21.95133949	0.858538482	0.016332512	-	160518	grey	0.073069298	FALSE	DENND5B
Region_3971	chr12	31589923	31615666	25743	12	66	92.74242424	9.958854747	0.69885445	0.033927961	+	100874249	grey	0.09826311	FALSE	DENND5B-AS1
Region_3972	chr12	31647160	31673114	25954	6	32	45.92424242	5.7867504	0.484389769	0.038452351	+	254013	grey	-0.368070014	FALSE	ETFBKMT
Region_3973	chr12	31671142	31729121	57979	9	55	76.10606061	8.521698617	0.968344402	0.022681473	-	196394	grey	0.222347338	FALSE	AMN1
Region_3974	chr12	31876969	31959316	82347	30	154	202.1060606	18.19224584	0.880933175	0.031599323	-	105369723	grey	-0.039301291	FALSE	LINC02422

Region_3975	chr12	31959370	31993107	33737	19	123	151.8939394	12.62857418	0.843373871	0.026970638	+	55196	grey	-0.059425704	FALSE	RESF1
Region_3976	chr12	32106847	32383633	276786	72	547	590.6666667	25.88515068	0.911800314	0.01385476	+	636	grey	0.344469536	FALSE	BICD1
Region_3977	chr12	32399558	32646050	246492	64	436	509.030303	27.76825509	0.918147862	0.013109931	+	121512	grey	0.336868981	FALSE	FGD4
Region_3978	chr12	32679200	32745650	66450	19	121	151.4242424	13.09499095	0.63367943	0.020531098	+	10059	grey	-0.080616423	FALSE	DNM1L
Region_3979	chr12	32727490	32755897	28407	8	54	75.1969697	8.188584356	0.812592275	0.05815291	-	51067	grey	0.214733721	FALSE	YARS2
Region_3980	chr12	32790745	32896777	106032	23	155	191.7575758	12.45676907	0.920860871	0.014066791	-	5318	grey	-0.156161645	FALSE	PKP2
Region_3981	chr12	33374238	33439819	65581	5	39	53.12121212	6.311199207	0.729078848	0.061727249	-	341359	grey	-0.078912071	FALSE	SYT10
Region_3982	chr12	38646822	38907430	260608	17	108	126.9545455	10.24234321	0.884062843	0.025692824	-	144402	grey	-0.04242599	FALSE	CPNE8
Region_3983	chr12	39087347	39145482	58135	5	20	31.22727273	5.007057956	0.770313861	0.069388456	-	105369730	grey	0.032899595	FALSE	LINC02406
Region_3984	chr12	39293228	39443390	150162	15	85	114.4393939	10.67726382	0.687071498	0.019276987	-	55605	grey	0.01589718	FALSE	KIF21A
Region_3985	chr12	39550033	39619803	69770	3	14	25.18181818	4.982767507	0.864503768	0.12310047	-	225	grey	0.064960372	FALSE	ABCD2
Region_3986	chr12	39626167	39908300	282133	18	136	155.8030303	11.44649049	0.923406316	0.02560488	+	283461	grey	0.134694038	FALSE	C12orf40
Region_3987	chr1	39755025	40106089	351064	29	216	252.454545	15.9433437	0.88290144	0.02243303	-	114134	grey	0.099555448	FALSE	SLC2A13

	2						5	5	4	9						
Region_3988	chr1<br>2	40196744	40369285	172541	13	72	90.7121212<br>1	9.03797995	0.96302839	0.02521281<br>5	+	120892	grey	0.08931602	FALSE	LRRK2
Region_3989	chr1<br>2	40393395	40570832	177437	13	78	102.712121<br>2	11.9289759<br>1	0.87154817<br>4	0.04948301<br>6	+	283463	grey	-0.33925437<br>6	FALSE	MUC19
Region_3990	chr1<br>2	40395853	40443847	47994	5	28	43.1060606<br>1	6.60099943	0.90270680<br>1	0.05931174<br>7	-	10536973<br>6	grey	0.270913264	FALSE	LOC105369736
Region_3991	chr1<br>2	40692439	41072415	379976	18	104	140.772727<br>3	13.3044763<br>5	0.86378122<br>1	0.04970943<br>6	+	1272	grey	0.326671315	FALSE	CNTN1
Region_3992	chr1<br>2	41188320	41574745	386425	21	159	190.409090<br>9	14.2472424	0.77870433<br>5	0.03029498<br>7	+	29951	grey	0.010200831	FALSE	PDZRN4
Region_3993	chr1<br>2	41951493	42024329	72836	8	45	62.9848484<br>8	6.99118659<br>3	0.69780311<br>6	0.05589294<br>9	-	10537824<br>7	grey	-0.10346500<br>4	FALSE	LOC105378247
Region_3994	chr1<br>2	42081845	42144874	63029	7	33	50.1515151<br>5	7.91734692<br>2	0.72735085<br>9	0.04518939<br>6	-	283464	grey	0.0208142	FALSE	GXYLT1
Region_3995	chr1<br>2	42157104	42238349	81245	8	54	69.9090909<br>1	8.60541381<br>1	0.84604233<br>1	0.01899811<br>2	-	10138	grey	-0.07444180<br>8	FALSE	YAF2
Region_3996	chr1<br>2	42238447	42459715	221268	28	192	224.151515<br>2	15.5348169	0.80957143<br>4	0.01770806<br>3	+	51535	grey	0.241673049	FALSE	PPHLN1
Region_3997	chr1<br>2	42312086	42326257	14171	3	13	23.7575757<br>6	5.54162044<br>1	0.32083201<br>6	0.02084042<br>1	-	85437	grey	-0.07483204<br>2	FALSE	ZCRB1
Region_3998	chr1<br>2	42456757	42590355	133598	26	162	195.318181<br>8	14.7148894<br>7	0.85634800<br>3	0.02783812<br>8	-	144165	grey	0.281703761	FALSE	PRICKLE1
Region_3999	chr1<br>2	42615221	42646547	31326	9	45	60.1969697	10.0616862	0.85771693<br>5	0.04995348<br>5	-	10192705<br>8	grey	0.010634361	FALSE	LINC02402

Region_4000	chr12	42646583	42686701	40118	12	73	93.75757576	9.816878097	0.903765491	0.040178132	+	105369738	grey	0.151855317	FALSE	LINC02451
Region_4001	chr12	42687195	42717120	29925	6	38	48.8030303	6.850535514	0.656619049	0.055536838	+	105369739	grey	-0.046838451	FALSE	LINC02450
Region_4002	chr12	43353866	43552203	198337	18	127	152.3030303	11.89954065	0.803222552	0.042076579	-	80070	grey	0.04915776	FALSE	ADAMTS20
Region_4003	chr12	43758944	43798307	39363	3	10	21.06060606	4.281907511	0.975280401	0.045074373	+	51135	grey	0.047635933	FALSE	IRAK4
Region_4004	chr12	43835967	44389762	553795	49	343	395.5151515	19.80617031	0.839689083	0.014968767	+	84216	grey	0.02153915	FALSE	TMEM117
Region_4005	chr12	44508275	44921848	413573	24	142	177.5606061	14.42020888	0.81521252	0.033823387	-	4753	grey	-0.111572752	FALSE	NELL2
Region_4006	chr12	45014672	45051099	36427	7	30	45.78787879	6.252421442	0.424006239	0.039848078	-	440097	grey	-0.079358032	FALSE	DBX2
Region_4007	chr12	45139782	45216047	76265	11	63	80.60606061	8.751050553	0.903582673	0.028821336	-	51054	grey	0.091593129	FALSE	PLEKHA8P1
Region_4008	chr12	45215987	45482280	266293	29	231	269.9545455	15.9020384	0.866968081	0.018447571	+	196527	grey	-0.081109246	FALSE	ANO6
Region_4009	chr12	45475520	45610590	135070	14	98	120.9090909	10.33251369	0.89555805	0.039380748	-	105369743	grey	0.140015565	FALSE	LOC105369743
Region_4010	chr12	45729706	45908040	178334	17	120	143.0454545	11.89237871	0.947880072	0.01787337	+	196528	grey	0.222528198	FALSE	ARID2
Region_4011	chr12	45919131	45992120	72989	8	47	67.01515152	8.037398018	0.575878501	0.024269989	-	9169	grey	-0.240759272	FALSE	SCAF11
Region_4012	chr1	46183063	46269951	86888	6	34	46.7878787	6.65296961	0.45533948	0.04336441	-	81539	grey	0.3274519	FALSE	SLC38A1

	2						9	2	3							
Region_4013	chr12	46358188	46372773	14585	4	28	41.1969697	6.179917094	0.261098591	0.048644446	-	54407	grey	0.263650083	FALSE	SLC38A2
Region_4014	chr12	46383433	46876784	493351	46	334	380.9242424	22.27955987	0.690241761	0.019360957	+	100288798	grey	-0.252059395	FALSE	SLC38A4-AS1
Region_4015	chr12	46764761	46832408	67647	4	23	42.37878788	6.720268199	0.708581468	0.035384607	-	55089	grey	0.001583659	FALSE	SLC38A4
Region_4016	chr12	47079603	47236662	157059	11	79	100.1666667	10.01165987	0.884775573	0.035411933	+	91523	grey	-0.017086973	FALSE	PCED1B
Region_4017	chr12	47237734	47279021	41287	3	12	24.87878788	4.925647639	0.925437047	0.062870883	-	105369746	grey	-0.026001495	FALSE	LOC105369746
Region_4018	chr12	47353754	47369935	16181	3	17	26.86363636	4.610644419	0.931745052	0.083582491	+	107984528	grey	-0.204389763	FALSE	LINC02416
Region_4019	chr12	47377638	47420192	42554	4	29	40.5	5.08769254	0.905818509	0.049019987	+	111082992	grey	-0.105293656	FALSE	LINC02156
Region_4020	chr12	47661249	47706030	44781	6	36	53.16666667	7.964504588	0.90713316	0.05173815	-	79657	grey	-0.114694354	FALSE	RPAP3
Region_4021	chr12	47706058	47742294	36236	7	44	59.57575758	8.00780505	0.77078978	0.042275252	+	105369748	grey	0.337997815	FALSE	RPAP3-DT
Region_4022	chr12	47709734	47725567	15833	4	19	32.56060606	6.327300525	0.882855919	0.060232857	-	8909	grey	0.257659876	FALSE	ENDOU
Region_4023	chr12	47734363	47771040	36677	10	48	65.45454545	7.302648241	0.813586527	0.05594112	-	10411	grey	-0.060664722	FALSE	RAPGEF3
Region_4024	chr12	47753916	47782751	28835	8	41	58.09090909	7.009284552	0.533480014	0.054409871	+	55652	grey	-0.044616506	FALSE	SLC48A1

Region_4025	chr12	47782722	47833132	50410	11	72	96.04545455	11.6347382	0.699379575	0.0243639	-	51564	grey	-0.253979416	FALSE	HDAC7
Region_4026	chr12	47841537	47943048	101511	16	94	118.4242424	11.78664251	0.724393251	0.036061737	-	7421	grey	0.151564883	FALSE	VDR
Region_4027	chr12	47972967	48011227	38260	8	46	60.13636364	7.62967156	0.784081696	0.037603301	-	1280	grey	0.167505491	FALSE	COL2A1
Region_4028	chr12	48042897	48106079	63182	12	77	93.33333333	7.106515609	0.857525478	0.032198645	-	29843	grey	-0.011173142	FALSE	SENP1
Region_4029	chr12	48105139	48146404	41265	14	95	115.7121212	10.79852576	0.819050061	0.02437526	+	5213	grey	-0.001105054	FALSE	PFKM
Region_4030	chr12	48147789	48181213	33424	16	99	125.2878788	9.42299177	0.862422823	0.036396363	-	140461	grey	0.021278555	FALSE	ASB8
Region_4031	chr12	48183644	48185926	2282	5	19	29.62121212	5.010036547	0.225806332	0.054781092	+	387856	grey	0.250848686	FALSE	CCDC184
Region_4032	chr12	48198357	48230275	31918	10	69	88.77272727	9.597748862	0.659072511	0.031230834	+	102725258	grey	0.178166791	FALSE	LOC102725258
Region_4033	chr12	48472559	48473622	1063	3	12	21.27272727	4.078255489	0.707216605	0.123779926	+	23519	grey	0.111409294	FALSE	ANP32D
Region_4034	chr12	48688458	48716998	28540	12	72	89.24242424	8.124153174	0.961433801	0.019479532	-	904	grey	0.216089562	FALSE	CCNT1
Region_4035	chr12	48727435	48765790	38355	13	59	87.93939394	10.5682817	0.956457348	0.025459737	+	255411	grey	0.219487917	FALSE	SPMIP11
Region_4036	chr12	48813794	48828941	15147	4	18	28.53030303	5.746489757	0.685826211	0.047719971	+	784	grey	-0.253229619	FALSE	CACNB3
Region_4037	chr1	48829756	48852842	23086	4	19	30.2272727	6.00460196	0.81639446	0.06977289	-	9416	grey	-0.04246779	FALSE	DDX23

	2						3	5	1	3				3		
Region_4038	chr12	48903503	48957487	53984	19	116	136.2121212	12.22546542	0.738742354	0.025183389	-	377	grey	0.14179715	FALSE	ARF3
Region_4039	chr12	48904110	48931840	27730	5	19	34.12121212	6.659903329	0.692284096	0.071704291	+	85478	grey	-0.133038187	FALSE	CCDC65
Region_4040	chr12	48978322	48982620	4298	7	44	59.84848485	7.967708722	0.013060103	0.014080708	+	7471	grey	-0.350797137	FALSE	WNT1
Region_4041	chr12	48998367	49019235	20868	4	20	31.43939394	5.597056336	0.50010154	0.025884951	+	105369758	grey	-0.111878733	FALSE	DDN-AS1
Region_4042	chr12	49002274	49024075	21801	4	19	31.98484848	5.514993338	0.496300133	0.020172051	-	5571	grey	-0.110802301	FALSE	PRKAG1
Region_4043	chr12	49012367	49060794	48427	14	74	96.68181818	8.320797097	0.733763991	0.026202796	-	8085	grey	-0.094998229	FALSE	KMT2D
Region_4044	chr12	49064676	49070025	5349	6	30	49.84848485	7.567628272	0.790380221	0.028752166	-	121268	grey	-0.079209688	FALSE	RHEBL1
Region_4045	chr12	49086656	49094801	8145	5	32	45.40909091	6.459286154	0.347605277	0.054779814	-	50846	grey	-0.069966917	FALSE	DHH
Region_4046	chr12	49090208	49093312	3104	3	17	27.40909091	4.826138041	0.094852755	0.076259905	+	105369759	grey	-0.171285964	FALSE	LOC105369759
Region_4047	chr12	49097136	49110900	13764	10	57	78.8030303	10.28848452	0.097737986	0.009697631	-	55716	grey	0.269330904	FALSE	LMBR1L
Region_4048	chr12	49127782	49131397	3615	4	22	32.27272727	5.827112901	0.041410201	0.031557434	-	10376	grey	-0.088467637	FALSE	TUBA1B
Region_4049	chr12	49130978	49150076	19098	7	30	43.03030303	5.989656975	0.69797038	0.023317935	+	105369760	grey	-0.0896906	FALSE	TUBA1B-AS1

Region_4050	chr12	49184686	49189080	4394	4	19	30.28787879	5.134394284	0.678572012	0.049461403	-	7846	grey	-0.331264638	FALSE	TUBA1A
Region_4051	chr12	49208546	49274600	66054	53	386	435.3636364	25.04496655	0.822298148	0.011896337	+	84790	grey	0.299733523	FALSE	TUBA1C
Region_4052	chr12	49292631	49324576	31945	17	94	124.1818182	14.16486345	0.376660764	0.036384078	-	101927267	grey	0.185671634	FALSE	TROAP-AS1
Region_4053	chr12	49295147	49298686	3539	9	37	66.53030303	10.52355555	0.114615496	0.050847745	+	5630	grey	0.160762202	FALSE	PRPH
Region_4054	chr12	49346888	49353924	7036	4	19	32.87878788	5.630999779	0.895813636	0.063288841	+	79962	grey	0.035455002	FALSE	DNAJC22
Region_4055	chr12	49366584	49527425	160841	83	564	619.2878788	25.6975097	0.88057489	0.012661082	+	65244	grey	-0.369316741	FALSE	SPATS2
Region_4056	chr12	49539030	49558337	19307	10	50	69.40909091	8.564645168	0.597386647	0.036194324	+	23416	grey	-0.149049195	FALSE	KCNH3
Region_4057	chr12	49556544	49568145	11601	4	18	32.12121212	6.3452348	0.712852624	0.041019996	-	10445	grey	-0.046299398	FALSE	MCRS1
Region_4058	chr12	49568372	49644666	76294	37	260	310.1666667	22.35970506	0.771318045	0.017731183	+	25766	grey	0.011073743	FALSE	PRPF40B
Region_4059	chr12	49582885	49605639	22754	7	53	68.39393939	8.323976099	0.96932764	0.024279739	-	84070	grey	0.096259827	FALSE	FAM186B
Region_4060	chr12	49636499	49708165	71666	38	247	276.0909091	17.1793501	0.786603123	0.017953228	-	91010	grey	0.228883421	FALSE	FMNL3
Region_4061	chr12	49707725	49764934	57209	31	211	250.1515152	16.43752033	0.792938521	0.017238176	+	7009	grey	-0.079461385	FALSE	TMBIM6
Region_4062	chr1	49791146	49828750	37604	17	116	144.863636	11.6696666	0.91248253	0.02175057	-	57701	grey	0.064249711	FALSE	NCKAP5L

	2						4	1	3	8						
Region_4063	chr12	49827913	49841143	13230	4	19	31.04545455	4.919136316	0.741678786	0.037297762	+	100286844	grey	0.077698329	FALSE	BCDIN3D-AS1
Region_4064	chr12	49836043	49843106	7063	3	17	29.5	5.539369584	0.119321144	0.080710794	-	144233	grey	-0.143345726	FALSE	BCDIN3D
Region_4065	chr12	49866896	49904217	37321	7	32	49.40909091	7.266312731	0.906962188	0.044540512	-	23017	grey	0.020565072	FALSE	FAIM2
Region_4066	chr12	49900311	49932764	32453	17	111	139.7878788	10.61858335	0.90314896	0.022877332	+	101927292	grey	-0.036086115	FALSE	LINC02395
Region_4067	chr12	49950737	49958878	8141	7	34	55.5	7.151653924	0.657738402	0.070388048	+	359	grey	-0.167749785	FALSE	AQP2
Region_4068	chr12	49951512	49962924	11412	17	68	98.3030303	11.43943207	0.222705997	0.034986577	-	101927318	grey	-0.160935017	FALSE	AQP5-AS1
Region_4069	chr12	49961872	49965682	3810	5	21	31.90909091	5.560097599	0.356384739	0.077263964	+	362	grey	-0.180915651	FALSE	AQP5
Region_4070	chr12	49967194	49977139	9945	6	28	44.09090909	6.722470408	0.719941771	0.087187364	+	363	grey	0.0718293	FALSE	AQP6
Region_4071	chr12	49976923	50033136	56213	12	71	86.36363636	8.839833559	0.862864402	0.030221316	-	29127	grey	0.189071666	FALSE	RACGAP1
Region_4072	chr12	50057548	50083611	26063	9	40	51.87878788	7.31177165	0.718360043	0.058729957	+	41	grey	-0.09410076	FALSE	ASIC1
Region_4073	chr12	50085200	50100707	15507	9	53	74.03030303	8.866479133	0.932467237	0.031447699	+	6602	grey	-0.008892725	FALSE	SMARCD1
Region_4074	chr12	50103982	50111313	7331	6	19	32.10606061	4.845707975	0.946352726	0.038414736	+	2819	grey	-0.087365371	FALSE	GPD1

Region_4075	chr12	50112082	50165618	53536	24	122	178.8636364	17.53404281	0.867027912	0.023378121	+	84987	grey	-0.056119868	FALSE	COX14
Region_4076	chr12	50129289	50167533	38244	13	61	95.24242424	13.1465826	0.935274776	0.035845849	-	91012	grey	0.096061339	FALSE	CERS5
Region_4077	chr12	50175788	50283520	107732	50	336	380.3030303	20.17307398	0.911987014	0.017796657	-	51474	grey	0.123997319	FALSE	LIMA1
Region_4078	chr12	50326230	50396622	70392	21	120	149.3636364	11.90040253	0.91864801	0.020850016	-	121006	grey	0.005506742	FALSE	FAM186A
Region_4079	chr12	50392383	50480004	87621	30	203	237.5606061	15.30020491	0.958549096	0.013529559	+	113251	grey	0.271618881	FALSE	LARP4
Region_4080	chr12	50504985	50748657	243672	74	526	574.8787879	24.51844841	0.863479824	0.009314004	+	57609	grey	-0.053960015	FALSE	DIP2B
Region_4081	chr12	50763710	50821162	57452	21	130	159.8484848	12.95939508	0.800907168	0.02484356	+	466	grey	0.342740627	FALSE	ATF1
Region_4082	chr12	50842920	50887884	44964	6	40	54.15151515	6.96638616	0.977963925	0.02067357	+	283471	grey	-0.143164922	FALSE	TMPRSS12
Region_4083	chr12	50923472	50970700	47228	15	86	117.8333333	12.23938577	0.695061028	0.023583721	+	25840	grey	0.212190306	FALSE	TMT1A
Region_4084	chr12	50952538	50970607	18069	4	18	32.93939394	6.387195816	0.954346227	0.037359133	+	613227	grey	0.059455164	FALSE	HIGD1C
Region_4085	chr12	50979401	51028566	49165	20	121	157.3787879	15.49170016	0.691482813	0.021740902	-	4891	grey	0.040372271	FALSE	SLC11A2
Region_4086	chr12	51047962	51060424	12462	3	21	30.10606061	5.120483114	0.646920265	0.033309266	+	25875	grey	-0.208030504	FALSE	LETMD1
Region_4087	chr1	51061205	51083664	22459	5	23	33.5	5.72242552	0.96309264	0.03347254	-	81566	grey	-0.04081542	FALSE	CSRNP2

	2							1	4	1				6		
Region_4088	chr1<br>2	51093656	51173135	79479	17	77	105.469697	11.7985914<br>4	0.96488687<br>5	0.02295085<br>7	-	7024	grey	0.1504825	FALSE	TFCP2
Region_4089	chr1<br>2	51186936	51218062	31126	7	46	63.2272727<br>3	7.59715625<br>9	0.95053818<br>7	0.03088093<br>4	-	5463	grey	0.129648409	FALSE	POU6F1
Region_4090	chr1<br>2	51238886	51271362	32476	6	23	33.8030303	4.45566086<br>6	0.66673621<br>1	0.05490851<br>9	+	10272398<br>3	grey	0.018632767	FALSE	
Region_4091	chr1<br>2	51244558	51270415	25857	6	23	33.8030303	4.45566086<br>6	0.66673621<br>1	0.05490851<br>9	-	57228	grey	0.018632767	FALSE	SMAGP
Region_4092	chr1<br>2	51281038	51324668	43630	12	71	89.9090909<br>1	8.89896670<br>4	0.78315572<br>3	0.02563396<br>6	-	51411	grey	-0.21064003<br>3	FALSE	BIN2
Region_4093	chr1<br>2	51328442	51346679	18237	3	16	25.9393939<br>4	4.70965877<br>5	0.63930206<br>8	0.03561041	-	1990	grey	-0.13280253<br>4	FALSE	CELA1
Region_4094	chr1<br>2	51351247	51392867	41620	11	56	70.3333333<br>3	8.53649781<br>1	0.51527802<br>1	0.03505778<br>3	-	11226	grey	-0.15983526<br>2	FALSE	GALNT6
Region_4095	chr1<br>2	51391317	51515763	124446	51	348	395.257575<br>8	22.0616692<br>3	0.73892168<br>1	0.01488478<br>1	+	9498	grey	-0.11245906<br>2	FALSE	SLC4A8
Region_4096	chr1<br>2	51590266	51812864	222598	44	300	347.151515<br>2	17.0963265<br>7	0.74118994<br>6	0.02324881<br>3	+	6334	grey	0.121795727	FALSE	SCN8A
Region_4097	chr1<br>2	51817899	51848718	30819	14	55	75.7424242<br>4	12.0464621<br>1	0.31172476<br>9	0.03347120<br>3	-	401720	grey	-0.07785774<br>3	FALSE	FIGNL2
Region_4098	chr1<br>2	51906908	51923361	16453	7	34	54.6363636<br>4	8.17244218	0.91734036<br>5	0.04972430<br>3	+	94	grey	-0.23954827	FALSE	ACVRL1
Region_4099	chr1<br>2	51951699	51997078	45379	6	34	46.2575757<br>6	6.95990850<br>1	0.63349939<br>6	0.03083280<br>6	+	91	grey	-0.30716694<br>2	FALSE	ACVR1B

Region_4100	chr12	52022832	52059507	36675	15	101	118.5454545	9.230687645	0.709412037	0.017883113	+	3164	grey	-0.054813555	FALSE	NR4A1
Region_4101	chr12	52164115	52223813	59698	33	215	250.9393939	15.5888311	0.727873986	0.033029759	+	283404	grey	0.05258881	FALSE	LINC00592
Region_4102	chr12	52168996	52192014	23018	4	17	26.96969697	5.077763581	0.907536579	0.067560178	-	144501	grey	-0.02483998	FALSE	KRT80
Region_4103	chr12	52205392	52213583	8191	8	46	63.81818182	8.355392714	0.8171432	0.055285537	-	283403	grey	-0.162318774	FALSE	LINC02874
Region_4104	chr12	52233243	52252186	18943	16	91	114.4090909	9.939783735	0.63630698	0.038432457	+	3855	grey	0.009920463	FALSE	KRT7
Region_4105	chr12	52249300	52309163	59863	33	183	224.530303	15.08358221	0.492009096	0.027235145	+	3892	grey	0.052986361	FALSE	KRT86
Region_4106	chr12	52250466	52258867	8401	15	74	96.15151515	10.746366	0.337534811	0.040748454	-	85349	grey	0.039310541	FALSE	KRT87P
Region_4107	chr12	52314301	52321398	7097	3	12	26.34848485	5.562214347	0.889848844	0.076307053	-	3889	grey	-0.08648332	FALSE	KRT83
Region_4108	chr12	52360006	52367481	7475	4	20	30.10606061	5.386960861	0.928550889	0.054616143	-	3891	grey	-0.028236083	FALSE	KRT85
Region_4109	chr12	52393931	52406335	12404	11	67	85.78787879	10.19579876	0.883311074	0.044020793	-	3888	grey	0.218114926	FALSE	KRT82
Region_4110	chr12	52424070	52434371	10301	3	14	26.34848485	5.070252381	0.810170971	0.089389096	-	9119	grey	0.098310707	FALSE	KRT75
Region_4111	chr12	52468516	52473805	5289	5	28	46.86363636	8.127609855	0.933921286	0.041101615	-	286887	grey	0.125189992	FALSE	KRT6C
Region_4112	chr1	52487176	52493257	6081	4	20	30.2272727	4.73869652	0.90655615	0.06715406	-	3853	grey	0.15252786	FALSE	KRT6A

	2						3	5	4	5						
Region_4113	chr12	52565782	52573843	8061	7	35	54.31818182	7.198630406	0.797593671	0.06778678	-	121391	grey	-0.0043182	FALSE	KRT74
Region_4114	chr12	52585589	52601538	15949	6	26	44.39393939	6.577066889	0.832136894	0.056619344	-	140807	grey	0.09509839	FALSE	KRT72
Region_4115	chr12	52601467	52615305	13838	3	15	23.39393939	4.003378527	0.761183573	0.073087372	+	100127967	grey	0.029111788	FALSE	KRT73-AS1
Region_4116	chr12	52644558	52652211	7653	4	22	34.07575758	5.553406733	0.804997365	0.111219742	-	3849	grey	0.23447771	FALSE	KRT2
Region_4117	chr12	52689626	52703524	13898	7	40	54.03030303	7.283215049	0.772638644	0.04606741	-	374454	grey	0.082413652	FALSE	KRT77
Region_4118	chr12	52768155	52777345	9190	6	35	49.39393939	6.002252879	0.778929002	0.070499893	-	51350	grey	-0.068328266	FALSE	KRT76
Region_4119	chr12	52821408	52834311	12903	3	13	20.98484848	4.605990829	0.952146489	0.063965276	-	338785	grey	-0.025504036	FALSE	KRT79
Region_4120	chr12	52837804	52849092	11288	4	19	30.39393939	4.777765129	0.915464066	0.047835464	-	196374	grey	-0.118877158	FALSE	KRT78
Region_4121	chr12	52897187	52949954	52767	16	85	118.3787879	12.06108192	0.935184462	0.031907567	-	3856	grey	0.21879845	FALSE	KRT8
Region_4122	chr12	53006282	53042215	35933	10	43	70.34848485	8.95715	0.865318341	0.024411811	+	1975	grey	-0.056148456	FALSE	EIF4B
Region_4123	chr12	53012884	53054450	41566	11	52	75.10606061	10.00788733	0.839019241	0.039245821	-	283335	grey	0.148887958	FALSE	TNS2-AS1
Region_4124	chr12	53046969	53064379	17410	7	34	49.65151515	7.267403354	0.863899577	0.055920116	+	23371	grey	0.235330478	FALSE	TNS2

Region_4125	chr12	53064316	53079404	15088	10	53	68.01515152	8.077494707	0.881899877	0.040184197	-	84926	grey	0.176010143	FALSE	SPRYD3
Region_4126	chr12	53103486	53124535	21049	5	18	28.89393939	6.018251184	0.872415108	0.071120839	+	8435	grey	0.224951083	FALSE	SOAT2
Region_4127	chr12	53157663	53180925	23262	7	44	56.12121212	6.28922192	0.826408706	0.028816483	-	51380	grey	0.027982543	FALSE	CSAD
Region_4128	chr12	53180704	53195142	14438	7	50	63.31818182	6.229357519	0.697302258	0.038304862	+	283337	grey	0.095028614	FALSE	ZNF740
Region_4129	chr12	53191323	53207282	15959	6	24	41.27272727	7.527478799	0.79276301	0.071517125	-	3695	grey	-0.129768701	FALSE	ITGB7
Region_4130	chr12	53210567	53232980	22413	8	43	58.75757576	8.712609087	0.335579731	0.044998075	-	5916	grey	-0.226333642	FALSE	RARG
Region_4131	chr12	53268299	53293638	25339	13	87	117.2878788	10.63776314	0.769597799	0.032257467	+	9700	grey	0.136157232	FALSE	ESPL1
Region_4132	chr12	53295291	53299452	4161	7	30	49.16666667	6.962721248	0.806144763	0.032394242	+	5204	grey	-0.163443903	FALSE	PFDN5
Region_4133	chr12	53295492	53307177	11685	12	63	90	10.66554481	0.632973989	0.020561614	+	60314	grey	-0.160070841	FALSE	MYG1
Region_4134	chr12	53298655	53300375	1720	4	17	31.33333333	6.094974811	0.216126361	0.03882731	-	112268097	grey	-0.063114536	FALSE	MYG1-AS1
Region_4135	chr12	53307456	53324864	17408	7	39	54.62121212	7.120425984	0.686977742	0.028405104	-	8086	grey	-0.152439453	FALSE	AAAS
Region_4136	chr12	53326575	53345315	18740	9	60	82.60606061	9.899306561	0.724892772	0.040461704	-	121340	grey	0.041586018	FALSE	SP7
Region_4137	chr1	53380176	53416446	36270	24	141	172.939393	16.0296636	0.90770943	0.02209576	+	6667	grey	-0.16532438	FALSE	SP1

	2						9	4	5	2				6		
Region_4138	chr12	53441741	53481162	39421	11	53	75.87878788	8.61575507	0.616079691	0.024622423	+	5094	grey	-0.230664962	FALSE	PCBP2
Region_4139	chr12	53479669	53500063	20394	15	82	105.1212121	12.19901024	0.86995342	0.032620057	-	7786	grey	0.165203624	FALSE	MAP3K12
Region_4140	chr12	53500921	53506431	5510	3	14	24.43939394	5.072182925	0.003262863	0.01199266	+	6895	grey	-0.069245878	FALSE	TARBP2
Region_4141	chr12	53506691	53626363	119672	52	367	422.1666667	23.28127893	0.837109967	0.009432647	-	114108587	grey	-0.189113381	FALSE	ATF7-NPFF
Region_4142	chr12	53507856	53626410	118554	51	358	412.7272727	23.05147781	0.834435315	0.009733322	-	11016	grey	-0.187488022	FALSE	ATF7
Region_4143	chr12	53632726	53677408	44682	17	106	124.969697	11.55451253	0.94548521	0.029877731	-	517	grey	0.117466679	FALSE	ATP5MC2
Region_4144	chr12	53708517	53727745	19228	13	95	116.1818182	11.22073505	0.882494518	0.029231342	-	57658	grey	-0.108063357	FALSE	CALCOCO1
Region_4145	chr12	53739611	53898976	159365	51	360	404.1363636	22.42861247	0.657819526	0.020383422	+	107984525	grey	-0.028654505	FALSE	LOC107984525
Region_4146	chr12	53746337	53757227	10890	11	64	92.1969697	9.829656379	0.17930423	0.047581358	-	102216268	grey	-0.284954569	FALSE	CISTR
Region_4147	chr12	53935328	53939643	4315	6	23	45.59090909	8.5933378	0.051835287	0.039377129	-	100874366	grey	0.176451218	FALSE	HOXC13-AS
Region_4148	chr12	53938831	53946544	7713	5	11	24.15151515	5.192427694	0.061545138	0.068756415	+	3229	grey	0.168303484	FALSE	HOXC13
Region_4149	chr12	53962308	53974956	12648	7	35	49.54545455	7.22001879	0.08493656	0.038469865	-	100124700	grey	0.264427066	FALSE	HOTAIR

Region_4150	chr12	53973126	53977643	4517	4	16	28.09090909	5.493288976	0.117865741	0.073205622	+	3227	grey	0.23494802	FALSE	HOXC11
Region_4151	chr12	53985845	54035361	49516	34	189	238.6818182	19.80921238	0.156015795	0.029983372	+	3222	grey	0.060918496	FALSE	HOXC5
Region_4152	chr12	53990624	54030823	40199	29	161	205.1666667	18.50620132	0.146492662	0.034034942	+	3223	grey	-0.023643156	FALSE	HOXC6
Region_4153	chr12	53994895	54003337	8442	6	20	35.95454545	7.252441113	0.034913665	0.040075778	+	3225	grey	-0.222707104	FALSE	HOXC9
Region_4154	chr12	54016931	54056030	39099	13	70	97.18181818	11.8748721	0.268760803	0.057381203	+	3221	grey	0.352263787	FALSE	HOXC4
Region_4155	chr12	54058254	54122235	63981	25	168	200.530303	15.16191554	0.676386595	0.027937696	+	440101	grey	-0.077648635	FALSE	FLJ12825
Region_4156	chr12	54085132	54125992	40860	11	66	89.3030303	11.07869709	0.694172719	0.041325693	-	112488744	grey	-0.038233133	FALSE	FAM242C
Region_4157	chr12	54121277	54189008	67731	21	114	141.2878788	13.84059264	0.675582764	0.032499692	-	23583	grey	0.289135523	FALSE	SMUG1
Region_4158	chr12	54126019	54142493	16474	9	46	60.21212121	7.349547661	0.578378498	0.058904002	+	400043	grey	0.16908163	FALSE	LINC02381
Region_4159	chr12	54126191	54147485	21294	12	58	76.40909091	8.595127909	0.659036641	0.050557689	+	105369776	grey	0.195343053	FALSE	SMUG1-AS1
Region_4160	chr12	54230942	54280133	49191	14	88	110.4090909	10.40558618	0.886442842	0.021017356	-	23468	grey	0.059764334	FALSE	CBX5
Region_4161	chr12	54262615	54279063	16448	3	15	23.63636364	4.273843032	0.934363777	0.049969954	+	112935960	grey	0.076695667	FALSE	SCAT2
Region_4162	chr1	54301202	54351846	50644	23	135	169.954545	14.1197426	0.77085501	0.03449459	+	22818	grey	-0.22226438	FALSE	COPZ1

	2						5	6	9	5				3		
Region_4163	chr12	54353661	54497688	144027	82	546	593.3636364	23.14810932	0.686473999	0.017828983	+	102724050	grey	0.024873139	FALSE	GPR84-AS1
Region_4164	chr12	54369133	54391298	22165	21	107	134.8030303	11.61990295	0.61753427	0.047869493	-	25946	grey	-0.139606158	FALSE	ZNF385A
Region_4165	chr12	54395261	54419266	24005	14	86	109.4090909	11.29462401	0.554899516	0.040621096	-	3678	grey	0.104635952	FALSE	ITGA5
Region_4166	chr12	54455950	54473602	17652	5	26	41.04545455	5.808301269	0.856868	0.042307092	-	121355	grey	0.041608001	FALSE	GTSF1
Region_4167	chr12	54497752	54548243	50491	17	115	147.7121212	12.26221859	0.94096444	0.023500116	+	3071	grey	0.151208738	FALSE	NCKAP1L
Region_4168	chr12	54549601	54579239	29638	3	12	19.66666667	3.943186269	0.45611057	0.086139182	+	5153	grey	0.028897473	FALSE	PDE1B
Region_4169	chr12	54575387	54588659	13272	3	13	23.01515152	4.622661319	0.854437699	0.094496889	-	5502	grey	0.168407295	FALSE	PPP1R1A
Region_4170	chr12	54630811	54634895	4084	3	20	31.81818182	5.632075969	0.799040175	0.061239019	-	90070	grey	-0.038518532	FALSE	LACRT
Region_4171	chr12	54830518	54896008	65490	3	17	26.81818182	5.420499809	0.954767202	0.043935042	+	118430	grey	0.024378312	FALSE	MUCL1
Region_4172	chr12	54948015	54984762	36747	4	21	32.01515152	6.170631246	0.90803913	0.063243685	-	9840	grey	-0.007793257	FALSE	TESPA1
Region_4173	chr12	55684568	55716404	31836	3	10	21.45454545	5.4805441	0.979431362	0.035777778	-	3679	grey	0.170202201	FALSE	ITGA7
Region_4174	chr12	55716036	55724703	8667	3	13	20.86363636	4.447596978	0.923911456	0.062140656	+	100528022	grey	-0.055316769	FALSE	BLOC1S1-RDH5

Region_4175	chr12	55743122	55757264	14142	4	20	29.5	4.871423738	0.188086219	0.064617525	+	10220	grey	-0.129286412	FALSE	GDF11
Region_4176	chr12	55752463	55817724	65261	10	57	72.81818182	8.868108967	0.948537815	0.031323346	-	84324	grey	-0.077471023	FALSE	SARNP
Region_4177	chr12	55757275	55830824	73549	10	58	74.63636364	9.22477661	0.971664063	0.024299655	-	85406	grey	-0.086183753	FALSE	DNAJC14
Region_4178	chr12	55927319	55954027	26708	4	16	26.07575758	4.814435364	0.582913688	0.063809582	+	1606	grey	0.111814222	FALSE	DGKA
Region_4179	chr12	55954105	55973317	19212	4	21	30.43939394	4.74950617	0.931739223	0.049127867	-	6490	grey	-0.136515982	FALSE	PMEL
Region_4180	chr12	55973913	55996683	22770	4	16	25.06060606	5.440245435	0.689841606	0.105997194	+	5869	grey	-0.202288611	FALSE	RAB5B
Region_4181	chr12	55997180	56006641	9461	4	15	27.74242424	5.084851124	0.641670976	0.083037731	+	6821	grey	0.145614687	FALSE	SUOX
Region_4182	chr12	56007659	56038435	30776	4	29	37.40909091	4.547111586	0.917983331	0.044447945	+	64375	grey	-0.009903392	FALSE	IKZF4
Region_4183	chr12	56029649	56041806	12157	9	57	78.39393939	9.502839718	0.718536481	0.024318151	-	105369780	grey	0.145193784	FALSE	
Region_4184	chr12	56041351	56044697	3346	10	51	76.04545455	11.47898658	0.10342768	0.016892987	+	6231	grey	0.186310447	FALSE	RPS26
Region_4185	chr12	56076799	56103505	26706	16	76	107.2727273	14.31142143	0.776961339	0.022965292	+	2065	grey	-0.114503121	FALSE	ERBB3
Region_4186	chr12	56101331	56113910	12579	7	38	60.60606061	8.0534346	0.83476761	0.023461943	+	5036	grey	-0.077860939	FALSE	PA2G4
Region_4187	chr1	56118250	56144674	26424	16	85	111.924242	12.5186760	0.57350943	0.02797872	+	23344	grey	0.189374533	FALSE	ESYT1

	2						4	2	1	2						
Region_4188	chr1<br>2	56118260	56127514	9254	4	16	32.6060606<br>1	7.41490969<br>5	0.90655766<br>9	0.05304289<br>1	+	84872	grey	-0.15797994<br>3	FALSE	ZC3H10
Region_4189	chr1<br>2	56152256	56159647	7391	6	22	37.5757575<br>8	7.70825852	0.15453224<br>6	0.03020223<br>6	+	140465	grey	0.277533437	FALSE	MYL6B
Region_4190	chr1<br>2	56158346	56163008	4662	4	17	28.4090909<br>1	4.83887231<br>8	0.90759980<br>8	0.05748400<br>8	+	4637	grey	0.011549513	FALSE	MYL6
Region_4191	chr1<br>2	56162359	56189567	27208	8	50	65.6212121<br>2	7.05748953<br>3	0.96105998<br>1	0.02347621<br>6	-	6601	grey	0.015524347	FALSE	SMARCC2
Region_4192	chr1<br>2	56202179	56221933	19754	16	66	92.1212121<br>2	12.1484600<br>5	0.86578004<br>8	0.03082831<br>2	-	10193	grey	-0.11822410<br>1	FALSE	RNF41
Region_4193	chr1<br>2	56222015	56229854	7839	12	65	87.9848484<br>8	9.38655736<br>7	0.38902525<br>6	0.04187794	+	79035	grey	-0.21767518<br>7	FALSE	NABP2
Region_4194	chr1<br>2	56230049	56237846	7797	5	18	29.4090909<br>1	4.85791114<br>6	0.92260271<br>3	0.05501623<br>3	+	283375	grey	0.135344172	FALSE	SLC39A5
Region_4195	chr1<br>2	56237807	56258384	20577	9	44	62.1212121<br>2	9.77046593<br>9	0.60628158<br>6	0.03698304<br>1	-	283373	grey	-0.05516704	FALSE	ANKRD52
Region_4196	chr1<br>2	56266890	56270966	4076	3	17	26.8939393<br>9	4.79143077<br>4	0.64812460<br>3	0.05459919<br>6	+	93058	grey	-0.13294339<br>3	FALSE	COQ10A
Region_4197	chr1<br>2	56271699	56300391	28692	7	38	56.1969697	8.76745677<br>9	0.59432042<br>6	0.04499593<br>8	-	1431	grey	-0.16267052<br>4	FALSE	CS
Region_4198	chr1<br>2	56285916	56316119	30203	18	112	142.727272<br>7	13.4112988<br>6	0.71435017<br>1	0.02119837<br>3	-	10330	grey	0.027229165	FALSE	CNPY2
Region_4199	chr1<br>2	56300075	56317992	17917	14	81	108.393939<br>4	12.6599049<br>6	0.75975545<br>8	0.02238463<br>7	+	11226809<br>8	grey	0.140150243	FALSE	CNPY2-AS1

Region_4200	chr12	56316223	56334053	17830	8	51	70.86363636	8.015151735	0.691497714	0.033761691	-	9924	grey	-0.296823645	FALSE	PAN2
Region_4201	chr12	56341597	56360203	18606	5	25	36.24242424	6.478510289	0.884654119	0.061134362	-	6773	grey	-0.095176799	FALSE	STAT2
Region_4202	chr12	56360568	56362857	2289	4	33	44.28787879	5.64804977	0.256263974	0.026120192	-	319	grey	-0.002636865	FALSE	APOF
Region_4203	chr12	56416363	56449426	33063	19	109	131.2272727	11.13110051	0.877336627	0.030885338	-	8914	grey	-0.199213056	FALSE	TIMELESS
Region_4204	chr12	56449502	56488170	38668	12	74	102.1515152	10.27804594	0.641445013	0.015360245	-	4284	grey	0.070186675	FALSE	MIP
Region_4205	chr12	56468578	56479708	11130	4	20	33.77272727	5.299623948	0.715498653	0.037597516	+	283377	grey	-0.006209112	FALSE	SPRYD4
Region_4206	chr12	56470944	56488414	17470	5	21	37.18181818	5.788341309	0.768026519	0.031626455	-	27165	grey	-0.119745831	FALSE	GLS2
Region_4207	chr12	56521820	56596193	74373	37	242	285.3636364	17.62571231	0.858187537	0.017711208	+	5939	grey	0.096164802	FALSE	RBMS2
Region_4208	chr12	56595596	56636816	41220	17	106	129.8030303	12.35029703	0.748570102	0.022875518	-	11176	grey	0.037171222	FALSE	BAZ2A
Region_4209	chr12	56638175	56645984	7809	3	13	24.25757576	4.694712594	0.97444905	0.032251832	-	506	grey	-0.476684337	FALSE	ATP5F1B
Region_4210	chr12	56663341	56688408	25067	15	98	126.4545455	12.0934589	0.635219012	0.032707842	-	10728	grey	0.025507432	FALSE	PTGES3
Region_4211	chr12	56712305	56731628	19323	13	84	104.9242424	11.72415076	0.67218202	0.016834997	-	4666	grey	0.229209174	FALSE	NACA
Region_4212	chr1	56714612	56752374	37762	17	109	130.212121	12.2957342	0.74160075	0.01503792	-	5557	grey	0.210295073	FALSE	PRIM1

	2						2	8	7	3						
Region_4213	chr12	56752161	56787790	35629	10	59	72.10606061	8.774313539	0.826202812	0.037310023	+	8630	grey	0.269532836	FALSE	HSD17B6
Region_4214	chr12	56923133	56934408	11275	3	17	27.18181818	5.392042829	0.933142612	0.048455845	-	121214	grey	-0.137740191	FALSE	SDR9C7
Region_4215	chr12	56951431	56959374	7943	3	16	27.57575758	5.912335491	0.878569915	0.075772369	-	8608	grey	0.27839203	FALSE	RDH16
Region_4216	chr12	56998836	57006546	7710	4	24	33.06060606	5.417574785	0.68016115	0.046757772	-	9880	grey	-0.153994188	FALSE	ZBTB39
Region_4217	chr12	57010000	57028883	18883	5	19	28.71212121	4.926428419	0.921366275	0.059890797	-	6866	grey	-0.029813182	FALSE	TAC3
Region_4218	chr12	57028517	57051198	22681	7	35	56.22727273	7.793084616	0.691570825	0.052912449	-	4640	grey	0.040850681	FALSE	MYO1A
Region_4219	chr12	57055643	57088063	32420	26	161	203	15.6027611	0.766784989	0.021786355	-	23306	grey	0.176153115	FALSE	NEMP1
Region_4220	chr12	57089043	57095476	6433	4	21	30.84848485	4.959039684	0.755786806	0.082292901	+	4665	grey	0.043040336	FALSE	NAB2
Region_4221	chr12	57095408	57132139	36731	19	116	140.9393939	12.5977761	0.810426664	0.029017172	-	6778	grey	-0.38821411	FALSE	STAT6
Region_4222	chr12	57128483	57213361	84878	50	315	365.4242424	20.92849409	0.888295346	0.024600082	+	4035	grey	-0.071432247	FALSE	LRP1
Region_4223	chr12	57144620	57147619	2999	6	24	41.28787879	7.578847389	0.936543829	0.054955118	-	105751187	grey	-0.37265533	FALSE	LRP1-AS
Region_4224	chr12	57249609	57431043	181434	43	279	312.0454545	18.66748042	0.947539808	0.01599314	-	22864	grey	0.16909669	FALSE	R3HDM2

Region_4225	chr12	57434784	57452062	17278	4	19	33	6.556265353	0.957630074	0.047134188	+	3626	grey	0.031886248	FALSE	INHBC
Region_4226	chr12	57459785	57472268	12483	7	31	53.57575758	7.194144213	0.822197657	0.033859926	+	2735	grey	0.004697038	FALSE	GLI1
Region_4227	chr12	57472264	57488814	16550	21	115	159.5151515	15.1599091	0.692056709	0.022054116	-	64333	grey	0.050312681	FALSE	ARHGAP9
Region_4228	chr12	57487294	57517569	30275	14	84	102.7727273	10.60884312	0.593196949	0.020308472	+	4141	grey	0.141292863	FALSE	MARS1
Region_4229	chr12	57516588	57521737	5149	6	32	50.31818182	6.843658695	0.447947838	0.041471865	-	1649	grey	0.038305622	FALSE	DDIT3
Region_4230	chr12	57520710	57530148	9438	7	41	57.6969697	7.803292593	0.675640428	0.053936064	+	114785	grey	0.030022335	FALSE	MBD6
Region_4231	chr12	57529633	57547224	17591	3	11	20.24242424	4.260951884	0.505330808	0.0696653	-	10540	grey	0.147808549	FALSE	DCTN2
Region_4232	chr12	57546026	57586633	40607	23	133	168.030303	14.0690273	0.742412327	0.023868838	+	3798	grey	-0.019242881	FALSE	KIF5A
Region_4233	chr12	57591174	57603418	12244	15	85	111.4545455	11.51344608	0.51450227	0.026317536	+	79837	grey	0.182601906	FALSE	PIP4K2C
Region_4234	chr12	57610180	57619379	9199	14	64	84.13636364	9.151521712	0.288278336	0.039151107	+	115557	grey	-0.084046189	FALSE	ARHGEF25
Region_4235	chr12	57615280	57626151	10871	14	92	116.030303	12.29880507	0.293092732	0.037980267	+	65012	grey	0.090946608	FALSE	SLC26A10P
Region_4236	chr12	57623409	57633239	9830	30	139	179.5454545	21.07801028	0.118185378	0.021521037	-	2583	grey	-0.182271636	FALSE	B4GALNT1
Region_4237	chr1	57693841	57721557	27716	7	35	53.0151515	7.38291671	0.65104537	0.04912487	+	10956	grey	0.123828396	FALSE	OS9

	2						2	4	1	3						
Region_4238	chr12	57719093	57728356	9263	4	10	23.78787879	5.771322076	0.941459272	0.05179382	+	100130776	grey	-0.040101533	FALSE	AGAP2-AS1
Region_4239	chr12	57723761	57742157	18396	10	35	56.10606061	8.579275185	0.904411246	0.045675139	-	116986	grey	0.138475282	FALSE	AGAP2
Region_4240	chr12	57738013	57750219	12206	6	36	52	7.481258635	0.532150708	0.047816299	+	6302	grey	0.134630263	FALSE	TSPAN31
Region_4241	chr12	57747727	57756013	8286	6	23	37.71212121	7.267980677	0.454677155	0.037292016	-	1019	grey	0.07820304	FALSE	CDK4
Region_4242	chr12	57755103	57760411	5308	6	17	34.16666667	6.869292503	0.342205879	0.03026778	+	92979	grey	0.05034205	FALSE	MARCHF9
Region_4243	chr12	57768471	57772119	3648	3	12	22.66666667	4.258325179	0.337096027	0.062344342	-	4234	grey	0.049574113	FALSE	METTL1
Region_4244	chr12	57771492	57782541	11049	9	49	68.68181818	8.076859807	0.392428699	0.033193946	+	25895	grey	-0.222629717	FALSE	EEF1AKMT3
Region_4245	chr12	57772660	57808071	35411	13	72	97.60606061	10.94866172	0.616710993	0.026000006	+	10102	grey	-0.047037749	FALSE	TSFM
Region_4246	chr12	57797376	57818734	21358	10	71	98.25757576	9.440193293	0.904377189	0.030995103	-	10677	grey	0.129597246	FALSE	AVIL
Region_4247	chr12	57819927	57846729	26802	16	105	135.2272727	12.55853428	0.527298191	0.02520997	-	10106	grey	0.027260762	FALSE	CTDSP2
Region_4248	chr12	57869834	57896482	26648	14	86	116.9393939	11.3542656	0.918590642	0.029384059	-	283387	grey	0.099851608	FALSE	LOC283387
Region_4249	chr12	58544124	58813060	268936	21	156	187.5	15.21158465	0.871003329	0.032826665	+	100506869	grey	-0.374003984	FALSE	LOC100506869

Region_4250	chr12	58565959	58781747	215788	17	123	146.8484848	12.44092802	0.882650417	0.030617422	-	101927653	grey	-0.371767722	FALSE	LINC02388
Region_4251	chr12	58920639	59130879	210240	10	56	81.92424242	9.43367227	0.855374045	0.041615623	+	105369791	grey	-0.027165092	FALSE	LRIG3-DT
Region_4252	chr12	59596029	59789855	193826	9	61	75.5	8.14531484	0.742434601	0.023859189	+	9194	grey	0.161563101	FALSE	SLC16A7
Region_4253	chr12	61668302	61702771	34469	3	21	30.92424242	5.030095904	0.67644499	0.076676126	-	105369793	grey	0.20449716	FALSE	LOC105369793
Region_4254	chr12	61708273	62279150	570877	24	175	206.7575758	14.46487587	0.852132921	0.026015805	-	338811	grey	0.047559114	FALSE	TAFA2
Region_4255	chr12	62260338	62417431	157093	5	26	42.43939394	6.610315454	0.831239209	0.041507449	+	9958	grey	0.218698569	FALSE	USP15
Region_4256	chr12	62466817	62600476	133659	13	90	109.3030303	8.957969717	0.83905768	0.027113825	+	23041	grey	-0.556185306	FALSE	MON2
Region_4257	chr12	62643994	62935150	291156	43	336	382.0757576	19.27158978	0.735735157	0.014455709	-	57460	grey	-0.060220237	FALSE	PPM1H
Region_4258	chr12	63142759	63151201	8442	3	11	21.72727273	5.163616665	0.429752793	0.091172892	-	552	grey	0.122787024	FALSE	AVPR1A
Region_4259	chr12	63558913	63668939	110026	9	54	69.6969697	9.802620838	0.961438643	0.031091273	-	283417	grey	0.215287299	FALSE	DPY19L2
Region_4260	chr12	63779833	63809792	29959	6	30	45.62121212	6.996186107	0.955650068	0.036605834	+	10329	grey	-0.170374041	FALSE	RXYLT1
Region_4261	chr12	63843761	64162217	318456	31	197	236.4090909	16.81846721	0.913796408	0.016048096	+	57522	grey	-0.24245847	FALSE	SRGAP1
Region_4262	chr1	64186316	64222296	35980	4	19	31.2272727	4.09049726	0.71916573	0.04149874	-	144577	grey	0.120912172	FALSE	KICS2

	2						3		5	8						
Region_4263	chr12	64264762	64390758	125996	17	108	136.4393939	12.09466352	0.889765126	0.024034961	-	115749	grey	0.128628827	FALSE	C12orf56
Region_4264	chr12	64404392	64451125	46733	6	26	48.46969697	6.526206807	0.932604303	0.03812444	+	11260	grey	0.08304471	FALSE	XPOT
Region_4265	chr12	64452090	64507329	55239	7	39	55.75757576	7.118903559	0.974480705	0.023555225	+	29110	grey	0.085028116	FALSE	TBK1
Region_4266	chr12	64507001	64697564	190563	47	309	371.0606061	22.5871841	0.788069762	0.018728865	+	283349	grey	-0.065173334	FALSE	RASSF3
Region_4267	chr12	64507166	64533638	26472	4	16	32.83333333	5.771503826	0.969768174	0.03423254	-	105369803	grey	-0.055224353	FALSE	LOC105369803
Region_4268	chr12	64713445	64759431	45986	8	43	61.03030303	8.802044687	0.687156932	0.035910368	-	2799	grey	-0.055466812	FALSE	GNS
Region_4269	chr12	64759484	64977509	218025	38	282	313.9242424	15.40959801	0.785602482	0.020220681	+	23329	grey	-0.035329412	FALSE	TBC1D30
Region_4270	chr12	64883394	64990593	107199	12	85	109.530303	9.793592406	0.84521758	0.02659849	+	400046	grey	0.105594677	FALSE	LINC02389
Region_4271	chr12	64883703	64990646	106943	12	85	109.530303	9.793592406	0.84521758	0.02659849	+	107984522	grey	0.105594677	FALSE	LOC107984522
Region_4272	chr12	64920845	64982524	61679	7	50	61.42424242	6.253763436	0.764595133	0.041245007	-	109729131	grey	0.107927612	FALSE	LINC02231
Region_4273	chr12	65050626	65121305	70679	8	44	66.34848485	7.632909364	0.934281604	0.029306239	-	11197	grey	-0.041614753	FALSE	WIF1
Region_4274	chr12	65169583	65248355	78772	7	33	49.87878788	7.313875436	0.934332406	0.032224974	+	23592	grey	-0.082978134	FALSE	LEMD3

Region_4275	chr12	65278643	65491430	212787	14	87	110.3030303	10.25965916	0.749998252	0.039106938	+	253827	grey	0.21360323	FALSE	MSRB3
Region_4276	chr12	65466820	65642372	175552	20	151	176.3181818	13.10567592	0.871173571	0.026666663	-	100507065	grey	0.049203895	FALSE	MSRB3-AS1
Region_4277	chr12	65499887	65558689	58802	9	57	74.34848485	8.396871989	0.8711682	0.045782027	-	105369187	grey	0.041468606	FALSE	LOC105369187
Region_4278	chr12	65589111	65614169	25058	4	28	40.77272727	5.683431939	0.895108842	0.044512334	+	105369807	grey	0.012239369	FALSE	LINC02454
Region_4279	chr12	65758020	65826974	68954	12	61	79.6969697	8.106919546	0.573995289	0.0289602	-	204010	grey	0.124399651	FALSE	RPSAP52
Region_4280	chr12	65824460	65966291	141831	20	139	165.1515152	12.53946683	0.723873704	0.024490406	+	8091	grey	-0.174225047	FALSE	HMGA2
Region_4281	chr12	66116555	66130750	14195	4	13	32.96969697	7.007621558	0.983421045	0.019501831	-	84298	grey	-0.092475801	FALSE	LLPH
Region_4282	chr12	66123917	66170027	46110	6	38	52.04545455	7.424350411	0.800828172	0.035829566	-	51643	grey	0.316760606	FALSE	TMBIM4
Region_4283	chr12	66189195	66254622	65427	14	99	119.9242424	12.01577246	0.835708187	0.034187851	+	11213	grey	-0.052563491	FALSE	IRAK3
Region_4284	chr12	66302493	66343643	41150	5	30	43.15151515	6.414508613	0.956210254	0.034094807	+	92797	grey	0.207468425	FALSE	HELB
Region_4285	chr12	66347431	67069162	721731	65	486	554.2727273	21.20199225	0.854466443	0.021474808	-	23426	grey	0.004436964	FALSE	GRIP1
Region_4286	chr12	66950754	67096410	145656	16	126	149.1060606	9.333362471	0.787213312	0.043290632	-	102724421	grey	0.162213675	FALSE	LOC102724421
Region_4287	chr1	67269358	67319953	50595	3	18	27.8636363	5.47971465	0.96809356	0.03623642	+	55832	grey	0.157603227	FALSE	CAND1

	2						6	7		5						
Region_4288	chr12	67443105	67590771	147666	16	131	156.1060606	11.28256489	0.873120999	0.040083219	+	105369812	grey	0.241721916	FALSE	LOC105369812
Region_4289	chr12	67519829	67567126	47297	3	19	26.36363636	5.012711115	0.887346334	0.083065619	+	100507175	grey	0.005103157	FALSE	LINC02408
Region_4290	chr12	67571197	67589987	18790	3	16	25.72727273	4.959979698	0.922283181	0.059530705	-	105369815	grey	0.429102161	FALSE	LINC02442
Region_4291	chr12	67648338	67665406	17068	10	44	63.22727273	9.022698649	0.587945579	0.024819785	+	8445	grey	0.12540528	FALSE	DYRK2
Region_4292	chr12	67929235	67970017	40782	4	23	33.37878788	4.979234268	0.935804116	0.046147943	+	101927922	grey	-0.089546899	FALSE	LINC01479
Region_4293	chr12	67989445	68234686	245241	29	205	253.2272727	18.83678517	0.888657611	0.024560494	+	100885789	grey	0.026653695	FALSE	IFNG-AS1
Region_4294	chr12	68294566	68332381	37815	5	29	42.87878788	6.986834105	0.389699868	0.021275658	-	56890	grey	0.19016926	FALSE	MDM1
Region_4295	chr12	68332889	68451679	118790	11	82	102.0757576	9.021871895	0.820910269	0.022409525	-	100507195	grey	0.029182513	FALSE	LINC02384
Region_4296	chr12	68610855	68671901	61046	9	50	68.24242424	8.467241138	0.850857865	0.03684563	+	5908	grey	-0.095558873	FALSE	RAP1B
Region_4297	chr12	68686951	68745809	58858	10	46	70.1969697	8.81470746	0.904652152	0.027209627	+	57122	grey	-0.096855169	FALSE	NUP107
Region_4298	chr12	68746125	68781468	35343	11	74	97.51515152	10.89706019	0.799997533	0.01513025	+	55508	grey	0.302298014	FALSE	SLC35E3
Region_4299	chr12	68746125	68793954	47829	16	108	135.2272727	12.30859044	0.784401885	0.019851359	+	105369820	grey	0.072594429	FALSE	

Region_4300	chr12	68808177	68845544	37367	6	30	46.65151515	6.125589263	0.946174067	0.035475759	+	4193	grey	-0.183479826	FALSE	MDM2
Region_4301	chr12	68842197	68971570	129373	23	153	182.3787879	13.57006338	0.72024365	0.029212751	-	1368	grey	0.038240162	FALSE	CPM
Region_4302	chr12	69239569	69274358	34789	13	65	99.77272727	11.39472949	0.412778014	0.024839619	+	11052	grey	-0.203917459	FALSE	CPSF6
Region_4303	chr12	69359710	69390870	31160	5	27	42.63636364	6.393834443	0.767098974	0.025683651	+	8089	grey	-0.054524439	FALSE	YEATS4
Region_4304	chr12	69470349	69579793	109444	20	134	162.7272727	12.85965121	0.846446872	0.019673365	+	10818	grey	0.229331599	FALSE	FRS2
Region_4305	chr12	69643360	69699476	56116	5	31	44.81818182	6.226269635	0.838375269	0.07311587	-	144453	grey	-0.088901255	FALSE	BEST3
Region_4306	chr12	69713633	69738574	24941	5	26	36.63636364	6.200834593	0.929533928	0.050774193	-	101928002	grey	-0.206002214	FALSE	LOC101928002
Region_4307	chr12	69738860	69823204	84344	14	91	115.2878788	10.80991731	0.81312052	0.033513906	+	117177	grey	0.052710982	FALSE	RAB3IP
Region_4308	chr12	69801669	69959097	157428	20	102	141.4545455	14.20420503	0.869427742	0.029820018	+	196446	grey	0.152931562	FALSE	MYRFL
Region_4309	chr12	69901918	70243405	341487	38	268	299	17.27782038	0.878845781	0.025695066	-	101928062	grey	-0.060707741	FALSE	PRANCR
Region_4310	chr12	70243002	70354993	111991	15	100	124.9848485	11.77284022	0.901096749	0.033482647	+	4848	grey	-0.113836642	FALSE	CNOT2
Region_4311	chr12	70366290	70434292	68002	8	40	56.6969697	6.656332316	0.814663543	0.044079895	+	27345	grey	-0.463293679	FALSE	KCNMB4
Region_4312	chr1	70468080	70543040	74960	10	52	75.0757575	9.40427170	0.88317419	0.05069447	+	10536982	grey	-0.21510337	FALSE	

	2						8	7	8	6		7		2		
Region_4313	chr12	70515870	70637440	121570	13	71	94.93939394	9.191766864	0.794885408	0.037062058	-	5787	grey	-0.050125456	FALSE	PTPRB
Region_4314	chr12	70638073	70920738	282665	30	191	235.6969697	17.99911938	0.854631936	0.022486994	-	5801	grey	0.371574956	FALSE	PTPRR
Region_4315	chr12	71125085	71441898	316813	16	127	146.530303	10.41628857	0.829697505	0.029858226	-	7103	grey	0.326233974	FALSE	TSPAN8
Region_4316	chr12	71157748	71315756	158008	6	44	61.10606061	8.845909594	0.832424058	0.066240842	-	105369832	grey	0.343777842	FALSE	LOC105369832
Region_4317	chr12	71439798	71586310	146512	13	77	108.9393939	10.98373453	0.763608805	0.029609514	+	8549	grey	-0.118207712	FALSE	LGR5
Region_4318	chr12	71609599	71667725	58126	6	30	40.93939394	6.081191148	0.651771412	0.021493227	-	196441	grey	0.022611354	FALSE	ZFC3H1
Region_4319	chr12	71674204	71705047	30843	3	22	29.34848485	4.747444411	0.649931893	0.027243484	+	55266	grey	-0.281392934	FALSE	TMEM19
Region_4320	chr12	71754863	71800286	45423	4	21	35.40909091	6.549168527	0.979826371	0.026696463	+	23011	grey	0.154543285	FALSE	RAB21
Region_4321	chr12	71839707	71927248	87541	10	49	73.15151515	8.43297999	0.794749863	0.045522071	+	64786	grey	-0.225784372	FALSE	TBC1D15
Region_4322	chr12	71938845	72186618	247773	20	152	183.469697	13.07871988	0.841728297	0.029617636	+	121278	grey	-0.278407721	FALSE	TPH2
Region_4323	chr12	72031299	72670758	639459	57	446	499.8636364	21.32667983	0.790037653	0.021243127	+	29953	grey	0.172961768	FALSE	TRHDE
Region_4324	chr12	72249964	72276954	26990	5	22	37.98484848	6.059937609	0.022654063	0.029137103	-	283392	grey	0.230316038	FALSE	TRHDE-AS1

Region_4325	chr12	73159190	73208317	49127	3	13	26.5	4.595147943	0.832370057	0.078563386	+	101928137	grey	-0.095608375	FALSE	LINC02444
Region_4326	chr12	73820315	74402600	582285	29	183	213.8787879	15.44271314	0.831090192	0.033848481	-	100507377	grey	0.134614993	FALSE	LINC02882
Region_4327	chr12	74199572	74402532	202960	10	47	65.51515152	9.037090111	0.832183154	0.070935087	-	107987178	grey	0.212203762	FALSE	LOC107987178
Region_4328	chr12	75040077	75209839	169762	9	44	63.54545455	8.925644675	0.876972359	0.049208241	-	3747	grey	-0.203220007	FALSE	KCNC2
Region_4329	chr12	75275979	75390928	114949	8	50	73.84848485	8.071302296	0.830072463	0.05295921	-	84698	grey	0.24429074	FALSE	CAPS2
Region_4330	chr12	75391089	75432688	41599	4	20	29.51515152	5.069493752	0.965165727	0.040197738	+	144321	grey	-0.044042136	FALSE	GLIPR1L2
Region_4331	chr12	75563202	75984015	420813	38	291	339.5	18.66485797	0.886738012	0.020255353	-	105369844	grey	0.096209083	FALSE	LOC105369844
Region_4332	chr12	75649195	75691937	42742	3	19	28.83333333	4.90394922	0.947884799	0.051576513	+	107984533	grey	-0.07045129	FALSE	LOC107984533
Region_4333	chr12	76036585	76084735	48150	7	40	54.07575758	6.390643656	0.677885764	0.02681704	-	4673	grey	0.224031778	FALSE	NAP1L1
Region_4334	chr12	76351797	76559809	208012	29	222	252.3484848	16.35988755	0.917034883	0.019900067	-	114882	grey	-0.116526013	FALSE	OSBPL8
Region_4335	chr12	76559870	76615567	55697	8	52	69.10606061	8.58644511	0.802455425	0.050354284	+	105369850	grey	-0.299034814	FALSE	LOC105369850
Region_4336	chr12	76764103	76853696	89593	11	77	96.96969697	10.18893774	0.974069538	0.021191506	+	23390	grey	-0.047867722	FALSE	ZDHHC17
Region_4337	chr1	76858709	76879023	20314	5	28	42.2575757	6.48622352	0.73489067	0.05732590	-	1466	grey	0.101031918	FALSE	CSRP2

	2						6	2	8	9						
Region_4338	chr12	77021248	77065569	44321	8	44	67.75757576	9.716062207	0.971392989	0.019625143	-	144455	grey	0.126084822	FALSE	E2F7
Region_4339	chr12	77129178	77163638	34460	6	37	52.92424242	6.327742594	0.753571395	0.04025115	+	105369853	grey	0.169376215	FALSE	LOC105369853
Region_4340	chr12	77219603	77317234	97631	14	77	109.4090909	10.70869851	0.823844783	0.045662133	-	105369854	grey	-0.201070844	FALSE	LINC02464
Region_4341	chr12	77324641	78213010	888369	53	398	442.1666667	20.85302659	0.789180421	0.024828724	+	89795	grey	-0.174546816	FALSE	NAV3
Region_4342	chr12	78863993	79452008	588015	33	242	282.7121212	17.33716786	0.85655211	0.025294519	+	6857	grey	-0.095090182	FALSE	SYT1
Region_4343	chr12	78960258	79045644	85386	5	25	42.10606061	7.279325387	0.882638375	0.061471082	-	105369863	grey	-0.019066365	FALSE	LOC105369863
Region_4344	chr12	79574979	79690964	115985	10	54	77.36363636	7.981972696	0.84015128	0.039664301	-	5074	grey	-0.149075085	FALSE	PAWR
Region_4345	chr12	79690144	79778451	88307	12	74	92.53030303	8.010031589	0.834832043	0.038662887	+	105369864	grey	0.059759209	FALSE	PPP1R12A-AS2
Region_4346	chr12	79773563	79935460	161897	23	159	205.1060606	14.1488013	0.851912416	0.015838522	-	4659	grey	-0.135673603	FALSE	PPP1R12A
Region_4347	chr12	80099537	80380880	281343	20	122	160.0151515	15.05731861	0.902221823	0.030172566	+	283310	grey	0.210611611	FALSE	OTOGL
Region_4348	chr12	80405994	80680273	274279	14	80	112.0909091	12.29599969	0.837229987	0.036747607	+	374462	grey	-0.076350113	FALSE	PTPRQ
Region_4349	chr12	80716912	80719671	2759	3	14	26.51515152	6.939834678	0.437626073	0.145147277	+	4617	grey	-0.041253186	FALSE	MYF5

Region_4350	chr12	80792520	80937934	145414	9	51	69.77272727	8.178387004	0.914265448	0.051595205	-	8825	grey	0.300812542	FALSE	LIN7A
Region_4351	chr12	80937815	81261210	323395	21	136	162.7575758	11.07604568	0.836572803	0.025777512	+	79611	grey	-0.116812911	FALSE	ACSS3
Region_4352	chr12	81094371	81125863	31492	3	18	27.8030303	4.293894402	0.952619716	0.046335214	-	107984535	grey	0.021633406	FALSE	LOC107984535
Region_4353	chr12	81257975	81759553	501578	32	248	283.3636364	16.97113963	0.860962946	0.026761535	-	8499	grey	0.213611841	FALSE	PPFIA2
Region_4354	chr12	82223681	82358805	135124	9	62	83.04545455	9.123818057	0.677575436	0.043646702	-	29080	grey	0.039133355	FALSE	CCDC59
Region_4355	chr12	82358528	82479239	120711	3	13	24.48484848	4.890312006	0.96389663	0.039676085	+	84190	grey	-0.019466972	FALSE	METTL25
Region_4356	chr12	82686880	83134870	447990	48	347	394.8181818	23.15759327	0.912461082	0.016478593	+	160335	grey	-0.087048363	FALSE	TMTC2
Region_4357	chr12	83888705	84186576	297871	17	105	134.1363636	10.8894958	0.823610889	0.041136842	-	107984536	grey	-0.147653583	FALSE	LOC107984536
Region_4358	chr12	85036314	85264457	228143	5	34	48.22727273	6.432598361	0.753500322	0.086329485	+	84125	grey	0.138235329	FALSE	LRRIQ1
Region_4359	chr12	85280220	85301784	21564	6	38	57.31818182	7.662413942	0.392447826	0.046799228	+	8092	grey	0.08268473	FALSE	ALX1
Region_4360	chr12	85955666	86838998	883332	47	351	404.5909091	22.49921522	0.788764985	0.026328908	-	25834	grey	0.063958391	FALSE	MGAT4C
Region_4361	chr12	88049016	88142099	93083	10	60	81.34848485	8.752129278	0.962680625	0.021042578	-	80184	grey	0.024269565	FALSE	CEP290
Region_4362	chr1	88142296	88199887	57591	7	59	77.5	8.20928180	0.91735880	0.02806612	+	160418	grey	-0.08052187	FALSE	TMTC3

	2							1	7	1				2		
Region_4363	chr12	88492793	88580851	88058	6	38	57.40909091	7.85831705	0.934417785	0.041655058	-	4254	grey	-0.078863441	FALSE	KITLG
Region_4364	chr12	88781256	88847391	66135	4	18	37.24242424	6.709489505	0.786159394	0.096076123	-	105369887	grey	0.140070282	FALSE	LOC105369887
Region_4365	chr12	89010681	89309566	298885	19	134	169.6363636	12.39433663	0.899623797	0.029058483	-	728084	grey	-0.280132116	FALSE	LINC02458
Region_4366	chr12	89347235	89352501	5266	4	26	40.06060606	7.519981154	0.028196752	0.026446727	-	1848	grey	0.087579827	FALSE	DUSP6
Region_4367	chr12	89419718	89526047	106329	9	55	68.71212121	7.527927801	0.850585992	0.022587361	-	282809	grey	0.121410652	FALSE	POC1B
Region_4368	chr12	89524594	89548005	23411	3	19	28.72727273	4.70856978	0.318630752	0.024579249	+	109729146	grey	0.223725409	FALSE	POC1B-AS1
Region_4369	chr12	89588049	89709300	121251	16	103	121.969697	11.32997345	0.892362503	0.023879266	-	490	grey	-0.245798062	FALSE	ATP2B1
Region_4370	chr12	89712048	90100763	388715	38	246	285.0909091	16.77702392	0.92455726	0.016557949	+	105369890	grey	0.039786491	FALSE	LOC105369890
Region_4371	chr12	90576402	90889917	313515	18	114	146.8787879	11.46568815	0.847909725	0.030784111	+	102724834	grey	0.131729521	FALSE	LINC02822
Region_4372	chr12	90617759	90808748	190989	8	50	69.57575758	8.586322946	0.88351414	0.040478246	-	105369895	grey	0.381171207	FALSE	LOC105369895
Region_4373	chr12	90905622	90955176	49554	7	31	42.37878788	5.882869921	0.929438656	0.048374314	-	196477	grey	0.356253877	FALSE	CCER1
Region_4374	chr12	91140484	91183217	42733	6	33	44.27272727	5.573413412	0.94769776	0.041588752	-	1634	grey	-0.053709027	FALSE	DCN

Region_4375	chr12	91276241	91634227	357986	12	78	106.6515152	9.673902123	0.801740967	0.036157677	-	105369896	grey	0.069732286	FALSE	LOC105369896
Region_4376	chr12	91327045	91368607	41562	3	13	21.5151515 2	3.927192868	0.882493713	0.0775076	+	105369897	grey	-0.168810467	FALSE	LINC02823
Region_4377	chr12	91984976	92142914	157938	10	65	83.0151515 2	8.635334253	0.8812702	0.041110592	-	256021	grey	0.387133993	FALSE	LINC01619
Region_4378	chr12	92145573	92226557	80984	3	15	25.5151515 2	4.500401432	0.86011075	0.066947267	+	101928617	grey	0.192449021	FALSE	BTG1-DT
Region_4379	chr12	92247697	92363832	116135	9	50	67.5303030 3	8.092198897	0.706173197	0.045532292	-	101930023	grey	0.128372788	FALSE	LINC02391
Region_4380	chr12	92421531	92492091	70560	7	52	65.6818181 8	8.017594289	0.943454476	0.035679776	+	100507616	grey	-0.166565172	FALSE	LINC02397
Region_4381	chr12	92702843	92772455	69612	4	32	43.8181818 2	6.164187116	0.783053366	0.097198372	+	440107	grey	0.309349421	FALSE	PLEKHG7
Region_4382	chr12	92770637	92929331	158694	20	127	165.8030303	14.47567689	0.789485357	0.013664034	-	8411	grey	0.303304977	FALSE	EEA1
Region_4383	chr12	92999218	93019820	20602	3	10	21.2727272 7	4.572645942	0.961301325	0.043387814	+	105369906	grey	0.256330108	FALSE	LINC02413
Region_4384	chr12	93003390	93377753	374363	43	319	358.4090909	19.51329115	0.943360184	0.015842621	-	643339	grey	-0.084601995	FALSE	LOC643339
Region_4385	chr12	93377883	93408146	30263	3	20	29.7575757 6	4.930472301	0.687104303	0.057739682	+	11163	grey	0.2963302	FALSE	NUDT4
Region_4386	chr12	93405684	93441947	36263	7	32	49.3484848 5	6.505600958	0.762474772	0.077905468	-	7334	grey	0.1777963	FALSE	UBE2N
Region_4387	chr1	93467514	93516214	48700	8	40	59.8939393	8.31149118	0.97618762	0.02237183	+	28977	grey	0.060646594	FALSE	MRPL42

	2						9	9	7							
Region_4388	chr12	93677375	93894840	217465	40	262	315.3333333	20.82922011	0.830844218	0.021126375	+	8738	grey	0.23735097	FALSE	CRADD
Region_4389	chr12	93707791	93737823	30032	7	50	64.09090909	6.733903366	0.953265103	0.030292747	-	101928731	grey	-0.121882712	FALSE	CRADD-AS1
Region_4390	chr12	93894965	93943603	48638	6	24	42.74242424	7.201090956	0.906578556	0.053480783	-	105369911	grey	-0.190934663	FALSE	LOC105369911
Region_4391	chr12	94148577	94307675	159098	17	119	143.9393939	10.59154785	0.798008454	0.032214101	+	10154	grey	0.523675703	FALSE	PLXNC1
Region_4392	chr12	94277758	94459988	182230	17	108	130.9090909	9.536532627	0.935799489	0.029281658	-	51134	grey	-0.191418014	FALSE	CEP83
Region_4393	chr12	94567122	94650557	83435	18	111	141.030303	12.15283404	0.627321777	0.023660797	-	57458	grey	-0.250392159	FALSE	TMCC3
Region_4394	chr12	94895297	95003708	108411	32	209	264.0454545	16.22669388	0.887344375	0.0235136	-	105369915	grey	-0.250341164	FALSE	LOC105369915
Region_4395	chr12	94971328	95003748	32420	7	46	61.53030303	7.436334285	0.788773378	0.028251911	-	55967	grey	-0.350526262	FALSE	NDUFA12
Region_4396	chr12	95020229	95073628	53399	14	80	108.6363636	11.67592706	0.964268385	0.017076062	-	7181	grey	-0.096236663	FALSE	NR2C1
Region_4397	chr12	95076749	95217482	140733	27	189	228.1363636	16.5342408	0.859264694	0.021836067	-	55785	grey	0.115424688	FALSE	FGD6
Region_4398	chr12	95217746	95302799	85053	13	63	87.87878788	9.376755469	0.956293477	0.024601113	+	55591	grey	-0.228231958	FALSE	VEZT
Region_4399	chr12	95311312	95442482	131170	22	153	185.3333333	14.02232652	0.899195357	0.029155846	-	105369917	grey	0.164340784	FALSE	LOC105369917

Region_4400	chr12	95345853	95442482	96629	16	107	131.4090909	11.38687135	0.883079389	0.03913209	-	107984545	grey	0.113498519	FALSE	LOC107984545
Region_4401	chr12	95473520	95515839	42319	8	40	58.53030303	8.333841476	0.719500132	0.029942682	+	10988	grey	0.003813646	FALSE	METAP2
Region_4402	chr12	95516560	95551476	34916	9	45	69.59090909	8.30009689	0.812697271	0.03753508	-	84101	grey	-0.030375764	FALSE	USP44
Region_4403	chr12	95551582	95673999	122417	30	191	218	16.37634316	0.910572248	0.025268168	+	100132594	grey	0.136557664	FALSE	PGAM1P5
Region_4404	chr12	95657807	95791189	133382	19	147	167.5454545	13.03035724	0.912322686	0.026070053	-	59277	grey	-0.042767236	FALSE	NTN4
Region_4405	chr12	95791733	95858839	67106	10	63	84.98484848	8.468025697	0.803118895	0.044420032	-	105369921	grey	0.060413471	FALSE	SNRPF-DT
Region_4406	chr12	95858952	95903828	44876	7	38	56.36363636	8.644493781	0.559357252	0.019341103	+	6636	grey	-0.132773564	FALSE	SNRPF
Region_4407	chr12	95867048	95942635	75587	18	106	131.4545455	13.50803956	0.524231723	0.026670715	-	120935	grey	0.062616299	FALSE	CCDC38
Region_4408	chr12	95943331	95968720	25389	13	82	106.6515152	13.85584289	0.72258845	0.060984907	+	144193	grey	-0.321661042	FALSE	AMDHD1
Region_4409	chr12	95996460	96011711	15251	4	17	33.53030303	5.847339163	0.765967112	0.044148849	+	102723340	grey	0.038346004	FALSE	LOC102723340
Region_4410	chr12	96000753	96043520	42767	9	48	66.43939394	8.44735514	0.590437687	0.037968	-	4048	grey	0.008036028	FALSE	LTA4H
Region_4411	chr12	96194375	96269824	75449	28	172	206.2727273	18.22386048	0.739051482	0.023823361	+	2004	grey	0.168460714	FALSE	ELK3
Region_4412	chr1	96278261	96400480	122219	18	108	130.818181	9.18512022	0.86650423	0.02525861	-	5128	grey	-0.30175742	FALSE	CDK17

	2						8	6	7					7		
Region_4413	chr12	96489571	96875555	385984	47	327	378.3484848	20.30649189	0.879782266	0.023797016	+	144535	grey	0.022813369	FALSE	CFAP54
Region_4414	chr12	96907224	96953780	46556	10	47	70.24242424	8.24259221	0.637613971	0.048998358	+	121441	grey	-0.042774423	FALSE	NEDD1
Region_4415	chr12	97430884	97598415	167531	17	133	158.8181818	10.16314471	0.853564392	0.030092379	+	196475	grey	0.091847848	FALSE	RMST
Region_4416	chr12	97644345	97756517	112172	15	89	112.9090909	11.19715249	0.83380294	0.07427713	-	643711	grey	-0.363562742	FALSE	PAFAH1B2P2
Region_4417	chr12	98515579	98550351	34772	6	39	55.90909091	7.169867015	0.779721042	0.024198143	+	7112	grey	-0.038309674	FALSE	TMPO
Region_4418	chr12	98593591	98606379	12788	3	11	23.98484848	4.439308415	0.651725589	0.030347754	+	5250	grey	-0.251525035	FALSE	SLC25A3
Region_4419	chr12	98613405	98645113	31708	10	43	67.09090909	11.10748503	0.406671416	0.031409581	-	121457	grey	-0.155485781	FALSE	IKBIP
Region_4420	chr12	98645290	98735433	90143	12	68	88.54545455	9.153699241	0.598121498	0.024731601	+	317	grey	0.209075443	FALSE	APAF1
Region_4421	chr12	98726457	99984936	1258479	115	838	934.4848485	40.12106388	0.764062797	0.014471105	-	56899	grey	0.263187113	FALSE	ANKS1B
Region_4422	chr12	100028455	100142874	114419	11	64	78.46969697	7.940586487	0.880964762	0.021660695	-	23074	grey	0.037115675	FALSE	BLTP3B
Region_4423	chr12	100156357	100173659	17302	8	44	59.37878788	7.601143945	0.841539618	0.033005552	-	55592	grey	0.04283072	FALSE	GOLGA2P5
Region_4424	chr12	100199122	100241865	42743	10	44	69.21212121	9.160979376	0.907018597	0.034922223	+	64431	grey	-0.006722761	FALSE	ACTR6

Region_4425	chr12	100203669	100267079	63410	13	72	95.42424242	10.61212747	0.913816421	0.028829401	-	120863	grey	0.050311477	FALSE	DEPDC4
Region_4426	chr12	100267140	100341715	74575	6	30	45.8030303	6.519130893	0.961890607	0.027940661	+	55681	grey	0.021878396	FALSE	SCYL2
Region_4427	chr12	100357074	100422055	64981	12	85	110.3484848	10.25894345	0.895745251	0.035688758	+	246213	grey	0.097501911	FALSE	SLC17A8
Region_4428	chr12	100473708	100564414	90706	21	153	175.9545455	12.51390835	0.877330889	0.026538666	+	9971	grey	0.063433445	FALSE	NR1H4
Region_4429	chr12	100573683	100628288	54605	7	44	57.57575758	6.791534563	0.912035529	0.040749091	+	283431	grey	0.303917054	FALSE	GAS2L3
Region_4430	chr12	101280105	101386618	106513	9	50	73.37878788	8.978204637	0.829426828	0.036991854	+	27340	grey	0.222898538	FALSE	UTP20
Region_4431	chr12	101568353	101686028	117675	14	86	111.9848485	11.22290573	0.943175208	0.03026184	+	4604	grey	-0.160205202	FALSE	MYBPC1
Region_4432	chr12	101696947	101744140	47193	10	63	83.83333333	8.608731405	0.586548814	0.017165948	+	56994	grey	-0.071573685	FALSE	CHPT1
Region_4433	chr12	101745499	101830959	85460	20	124	151.4242424	13.27236644	0.836366883	0.024744486	-	79158	grey	0.200210153	FALSE	GNPTAB
Region_4434	chr12	101877580	102012130	134550	14	87	104.9242424	8.87574154	0.679611554	0.047364817	+	55332	grey	0.121839179	FALSE	DRAM1
Region_4435	chr12	102012840	102062149	49309	9	53	71.33333333	7.313492974	0.839746749	0.027751003	-	51019	grey	0.119050826	FALSE	WASHC3
Region_4436	chr12	102073103	102120120	47017	7	38	52.65151515	8.280639931	0.812141706	0.032300354	-	79023	grey	-0.060077044	FALSE	NUP37
Region_4437	chr1	10212018	10219752	77337	4	21	35.3787878	6.68353925	0.97845994	0.03663909	+	55010	grey	0.068264726	FALSE	PARPBP

	2	3	0				8	5	4	6						
Region_4438	chr12	102304355	102463491	159136	14	92	112.7121212	10.33703596	0.886700422	0.032065676	+	105369942	grey	0.143487883	FALSE	LINC02456
Region_4439	chr12	102395874	102481744	85870	7	37	60.27272727	9.006912652	0.907543997	0.034837925	-	3479	grey	-0.046211363	FALSE	IGF1
Region_4440	chr12	102836889	102958410	121521	10	51	73.68181818	9.04205404	0.645071339	0.037552756	-	5053	grey	-0.034816423	FALSE	PAH
Region_4441	chr12	102957674	102960513	2839	4	16	28.22727273	4.882607924	0.075265496	0.05005819	+	429	grey	0.099922955	FALSE	ASCL1
Region_4442	chr12	103079634	103495978	416344	34	223	259.3181818	17.93506157	0.79240598	0.023325077	-	374470	grey	0.107980669	FALSE	C12orf42
Region_4443	chr12	103547751	103578947	31196	3	18	31.48484848	5.358652024	0.40651213	0.062434685	+	101929084	grey	0.194749909	FALSE	LINC02401
Region_4444	chr12	103587273	103766719	179446	9	51	79.51515152	8.218632535	0.941359127	0.036702099	+	55576	grey	0.418480373	FALSE	STAB2
Region_4445	chr12	103746315	103841234	94919	7	33	53.8030303	7.547225057	0.942311288	0.038283111	-	51559	grey	0.068685856	FALSE	NT5DC3
Region_4446	chr12	103843749	103930211	86462	10	64	85.39393939	10.25652331	0.935541421	0.042862433	-	253724	grey	-0.409963114	FALSE	TTC41P
Region_4447	chr12	103950194	103965708	15514	4	33	44.07575758	5.985394033	0.274383115	0.050811589	-	728568	grey	-0.137151329	FALSE	UQCC6
Region_4448	chr12	103965822	103988874	23052	3	10	18.12121212	4.592687001	0.967845062	0.042688305	+	6996	grey	-0.059925514	FALSE	TDG
Region_4449	chr12	103988987	104064183	75196	6	28	42.8030303	6.476513062	0.776168805	0.047393459	-	83468	grey	0.373414388	FALSE	GLT8D2

Region_4450	chr12	104064531	104106524	41993	5	18	27.31818182	4.915154246	0.75685281	0.046784328	+	29915	grey	-0.048078543	FALSE	HCFC2
Region_4451	chr12	104215779	104350307	134528	18	110	131.0151515	10.25294318	0.859530017	0.018259221	+	7296	grey	0.001320495	FALSE	TXNRD1
Region_4452	chr12	104456948	104762014	305066	108	851	931.5606061	35.50910123	0.719803659	0.013310678	+	50515	grey	0.094312879	FALSE	CHST11
Region_4453	chr12	104801801	104958744	156943	33	234	269.7878788	15.8361953	0.874956365	0.017643807	-	84102	grey	-0.024687526	FALSE	SLC41A2
Region_4454	chr12	104986316	105074197	87881	17	118	145.7272727	13.14949014	0.92829185	0.024432312	+	121053	grey	-0.069651642	FALSE	NOPCHAP1
Region_4455	chr12	105019784	105107643	87859	27	146	185.6363636	13.73892434	0.866400084	0.022032626	-	160428	grey	-0.265983016	FALSE	ALDH1L2
Region_4456	chr12	105102472	105107179	4707	3	11	18.57575758	3.863281212	0.832000505	0.097131791	-	414300	grey	0.087896353	FALSE	LOC414300
Region_4457	chr12	105107324	105169130	61806	23	147	180.5151515	16.84338941	0.738496525	0.023241221	+	23325	grey	-0.187109666	FALSE	WASHC4
Region_4458	chr12	105173297	105236203	62906	15	125	150.9090909	12.47980186	0.949954587	0.025357269	-	55198	grey	-0.349456806	FALSE	APPL2
Region_4459	chr12	105235290	105396097	160807	25	169	195.0151515	14.07069232	0.902741077	0.030171905	+	387882	grey	-0.049540512	FALSE	C12orf75
Region_4460	chr12	105704203	105744062	39859	9	40	54.1969697	7.164484408	0.909822693	0.046639315	+	101929110	grey	-0.087693981	FALSE	CASC18
Region_4461	chr12	106063345	106138954	75609	17	124	158.5	14.74214263	0.837074228	0.02712257	-	9891	grey	-0.135226975	FALSE	NUAK1
Region_4462	chr1	10623788	10630427	66398	8	49	65.9848484	8.35278382	0.61549755	0.0402834	-	10970	grey	-0.20900517	FALSE	CKAP4

	2	1	9				8	8	8					6		
Region_4463	chr12	106301929	106347003	45074	4	21	36.22727273	6.121325785	0.950584054	0.035473412	+	255394	grey	-0.027036318	FALSE	TCP11L2
Region_4464	chr12	106357748	106510198	152450	27	183	218.5	15.2691242	0.887309263	0.027392342	+	55703	grey	-0.228640104	FALSE	POLR3B
Region_4465	chr12	106495958	106774926	278968	58	423	485.7424242	24.62975724	0.800566343	0.017423254	-	100287944	grey	0.080379618	FALSE	LOC100287944
Region_4466	chr12	106583004	106762803	179799	37	267	313.1212121	18.81040232	0.826057372	0.024398283	+	5992	grey	0.225800127	FALSE	RFX4
Region_4467	chr12	106774621	106889316	114695	13	94	113.8939394	10.67582285	0.966267435	0.018141132	+	55188	grey	0.052819411	FALSE	RIC8B
Region_4468	chr12	106977277	106987160	9883	10	58	82.96969697	9.139886714	0.941489754	0.027512252	-	80298	grey	-0.18644062	FALSE	MTERF2
Region_4469	chr12	106991364	107093549	102185	11	62	83.37878788	8.701485552	0.955495593	0.03211917	-	1407	grey	-0.335073247	FALSE	CRY1
Region_4470	chr12	107318421	107659642	341221	58	455	499.5909091	24.17244284	0.790349257	0.019257208	+	121551	grey	-0.138512137	FALSE	ABTB3
Region_4471	chr12	107685799	107713162	27363	5	31	44.21212121	6.173180583	0.75422753	0.028769974	+	11137	grey	0.110772604	FALSE	PWP1
Region_4472	chr12	107732871	107761272	28401	5	28	38.83333333	5.874543081	0.765452205	0.039631601	-	11108	grey	-0.269668089	FALSE	PRDM4
Region_4473	chr12	107736555	107759968	23413	3	18	27.13636364	4.777789523	0.960340525	0.036286506	+	101929162	grey	-0.191611776	FALSE	PRDM4-AS1
Region_4474	chr12	108129288	108250537	121249	26	186	219.3484848	16.06572311	0.657548978	0.030421995	+	9671	grey	-0.209284002	FALSE	WSCD2

Region_4475	chr12	108288044	108339317	51273	9	52	69.98484848	8.326957934	0.61043577	0.05504682	-	1240	grey	-0.039201291	FALSE	CMKLR1
Region_4476	chr12	108434130	108492585	58455	6	35	48.06060606	6.775246321	0.762264106	0.067247516	-	102723562	grey	0.094324914	FALSE	LINC01498
Region_4477	chr12	108515277	108525832	10555	7	34	55.74242424	9.404271707	0.691978391	0.034655425	+	11153	grey	-0.093102993	FALSE	FICD
Region_4478	chr12	108522214	108561173	38959	7	57	75.09090909	9.411494237	0.732158421	0.033441786	-	9733	grey	0.225671979	FALSE	SART3
Region_4479	chr12	108589851	108598320	8469	5	22	31.21212121	4.589335972	0.749103718	0.0834356	-	338773	grey	0.104554134	FALSE	TMEM119
Region_4480	chr12	108628668	108642349	13681	4	18	29.78787879	5.573329764	0.486115859	0.087735837	+	105369968	grey	-0.160224299	FALSE	LOC105369968
Region_4481	chr12	108645109	108731526	86417	17	119	147.8787879	13.13774966	0.730737767	0.03336123	-	23603	grey	-0.285528699	FALSE	CORO1C
Region_4482	chr12	108778191	108857590	79399	24	158	188.469697	11.71090182	0.803742019	0.025992937	-	54434	grey	0.038749383	FALSE	SSH1
Region_4483	chr12	108858932	108901043	42111	7	50	64.68181818	7.634252955	0.743745991	0.046177738	+	1610	grey	-0.010006537	FALSE	DAO
Region_4484	chr12	108907741	109021068	113327	29	190	238.0757576	14.09889314	0.862277467	0.020876403	-	55530	grey	-0.146963255	FALSE	SVOP
Region_4485	chr12	109023089	109088023	64934	18	105	128.4090909	11.01324695	0.814375431	0.025548871	+	84749	grey	-0.095986279	FALSE	USP30
Region_4486	chr12	109097597	109126725	29128	12	74	101.5606061	11.1908325	0.575031677	0.03704944	+	7374	grey	-0.134335646	FALSE	UNG
Region_4487	chr1	10911121	10926822	157008	87	639	699.075757	25.2131288	0.84468194	0.01186309	+	32	grey	-0.10474939	FALSE	ACACB

	2	8	6				6	2	8	1				2		
Region_4488	chr12	109277978	109309284	31306	15	109	132.1212121	11.29535664	0.766829727	0.038834302	-	121643	grey	-0.048553274	FALSE	FOXN4
Region_4489	chr12	109347900	109455523	107623	13	82	104.2575758	8.38920663	0.894426281	0.024656728	+	283446	grey	0.175984183	FALSE	MYO1H
Region_4490	chr12	109448655	109477359	28704	8	53	72.3030303	7.922644657	0.639446361	0.04695071	-	83892	grey	0.108614649	FALSE	KCTD10
Region_4491	chr12	109477402	109536705	59303	24	158	193.8484848	16.59864909	0.885367901	0.01620631	+	89910	grey	-0.052644311	FALSE	UBE3B
Region_4492	chr12	109553715	109573580	19865	10	75	99.01515152	10.17310545	0.819994491	0.030102185	-	326625	grey	-0.180279233	FALSE	MMAB
Region_4493	chr12	109573255	109598125	24870	16	93	123.4393939	10.77765322	0.750172302	0.026834635	+	4598	grey	0.157876394	FALSE	MVK
Region_4494	chr12	109713825	109770495	56670	39	249	284.5909091	18.36214942	0.704173831	0.025009949	+	84915	grey	0.107698337	FALSE	FAM222A
Region_4495	chr12	109734166	109773508	39342	12	68	94.42424242	9.486931264	0.84348826	0.051891124	-	84983	grey	-0.067769626	FALSE	FAM222A-AS1
Region_4496	chr12	109783087	109833406	50319	22	133	164.7272727	12.03398451	0.767004675	0.024176745	-	59341	grey	0.15517069	FALSE	TRPV4
Region_4497	chr12	109850945	109880541	29596	18	115	142.3181818	11.48532052	0.664106067	0.027922698	-	51228	grey	0.071457916	FALSE	GLTP
Region_4498	chr12	109880676	109953269	72593	31	206	232.1060606	13.49088443	0.88952606	0.01861231	+	84260	grey	-0.197483561	FALSE	TCHP
Region_4499	chr12	109929804	109996389	66585	28	196	234.5757576	15.13941428	0.787891139	0.014374682	-	9815	grey	-0.03976969	FALSE	GIT2

Region_4500	chr12	109999186	110039763	40577	27	195	221.1666667	12.33205122	0.771791389	0.015950749	+	88455	grey	0.140361196	FALSE	ANKRD13A
Region_4501	chr12	110027028	110073686	46658	17	99	133.4242424	10.17093991	0.855038263	0.017169548	-	400073	grey	0.072824962	FALSE	C12orf76
Region_4502	chr12	110124335	110218793	94458	26	154	193.1212121	17.08577019	0.892852116	0.013323686	+	28981	grey	0.1496833	FALSE	IFT81
Region_4503	chr12	110280756	110351093	70337	41	290	332.2727273	21.09213886	0.76322732	0.015123474	+	488	grey	0.392162246	FALSE	ATP2A2
Region_4504	chr12	110372900	110445548	72648	39	229	265.1515152	18.03350934	0.827325014	0.018341248	-	51434	grey	0.315175206	FALSE	ANAPC7
Region_4505	chr12	110434823	110450422	15599	6	28	43.89393939	6.295612118	0.816622638	0.038272047	-	10094	grey	-0.027960259	FALSE	ARPC3
Region_4506	chr12	110452486	110469268	16782	9	48	66.46969697	8.956681557	0.334348647	0.023812022	-	51184	grey	0.255697918	FALSE	GPN3
Region_4507	chr12	110468415	110490385	21970	11	61	76.40909091	7.932362676	0.477389301	0.02741914	+	29902	grey	0.003653887	FALSE	FAM216A
Region_4508	chr12	110491083	110502111	11028	4	27	39.59090909	5.454644521	0.005236749	0.013357306	-	51699	grey	0.019385558	FALSE	VPS29
Region_4509	chr12	110501655	110533556	31901	12	75	112.0454545	11.41716901	0.714670688	0.018823716	+	144715	grey	-0.244275279	FALSE	RAD9B
Region_4510	chr12	110533245	110583318	50073	16	84	116	11.30690735	0.718990343	0.037494155	-	160760	grey	0.051889863	FALSE	PPTC7
Region_4511	chr12	110614027	110663431	49404	9	63	79.21212121	8.843102747	0.847461542	0.026513179	+	79600	grey	0.237244578	FALSE	TCTN1
Region_4512	chr1	11062784	11070495	77109	16	98	117.439393	10.5701012	0.80103983	0.02754923	-	84329	grey	0.082841267	FALSE	HVCN1

	2	1	0				9	1	3	6						
Region_4513	chr12	110719680	110742939	23259	11	65	91.46969697	10.7520584	0.798670022	0.039095329	-	5501	grey	0.079764167	FALSE	PPP1CC
Region_4514	chr12	110831779	110907535	75756	41	285	328.7121212	23.06630253	0.770197525	0.018056926	+	160762	grey	-0.063276831	FALSE	CCDC63
Region_4515	chr12	110910819	110921443	10624	6	44	56.66666667	6.540602968	0.943515101	0.031251463	-	4633	grey	0.181330934	FALSE	MYL2
Region_4516	chr12	110934590	110959093	24503	7	39	55.98484848	8.265763354	0.937106274	0.031958853	+	100131138	grey	0.11170156	FALSE	LINC01405
Region_4517	chr12	110951683	110957820	6137	3	17	26.90909091	4.656599197	0.938425808	0.046421535	-	105369980	grey	0.181397333	FALSE	LINC01404
Region_4518	chr12	111034165	111350554	316389	120	932	1016.878788	42.76429681	0.776406603	0.012808268	+	23316	grey	-0.020478615	FALSE	CUX2
Region_4519	chr12	111360651	111369121	8470	6	26	41.68181818	6.836911338	0.792030478	0.035091586	-	144717	grey	0.404974709	FALSE	PHETA1
Region_4520	chr12	111369261	111403310	34049	10	52	70.96969697	8.148562303	0.60534319	0.041802637	+	105369984	grey	0.151317375	FALSE	LINC02356
Region_4521	chr12	111405923	111451623	45700	20	137	168.5606061	16.07590532	0.69102236	0.026703364	+	10019	grey	0.174686478	FALSE	SH2B3
Region_4522	chr12	111443485	111599676	156191	56	370	421.5454545	21.48572465	0.868211223	0.011861956	-	6311	grey	0.182055313	FALSE	ATXN2
Region_4523	chr12	111642146	111685956	43810	10	84	106.7121212	10.06099116	0.815953077	0.029542687	-	8315	grey	-0.007515757	FALSE	BRAP
Region_4524	chr12	111686053	111757107	71054	30	187	239.4242424	15.79879314	0.976984936	0.011758189	+	80724	grey	0.25847974	FALSE	ACAD10

Region_4525	chr12	111753890	111817532	63642	39	261	323.3181818	19.52683289	0.754138952	0.018441046	+	217	grey	0.052476765	FALSE	ALDH2
Region_4526	chr12	111839758	111842902	3144	6	25	37.66666667	6.162749969	0.211066066	0.051649148	-	51275	grey	-0.168763001	FALSE	MAPKAPK5-AS1
Region_4527	chr12	111842228	111902222	59994	36	237	277.4090909	19.06747313	0.840707464	0.014173669	+	8550	grey	-0.223573159	FALSE	MAPKAPK5
Region_4528	chr12	111931282	112013166	81884	19	117	146.9090909	12.81192626	0.874234367	0.014572272	-	89894	grey	0.154957009	FALSE	TMEM116
Region_4529	chr12	112026689	112108796	82107	40	287	325.6818182	18.42586734	0.944212729	0.013631458	-	80018	grey	-0.10320704	FALSE	NAA25
Region_4530	chr12	112125538	112153604	28066	11	69	90.81818182	8.945835457	0.793725506	0.016529785	+	10906	grey	0.11622146	FALSE	TRAFD1
Region_4531	chr12	112160188	112382439	222251	71	486	540.3484848	26.14956533	0.905018945	0.010422505	-	283450	grey	-0.146873685	FALSE	HECTD4
Region_4532	chr12	112405189	112418838	13649	5	32	47.12121212	6.510382599	0.542166303	0.054303241	-	6128	grey	-0.004013436	FALSE	RPL6
Region_4533	chr12	112418907	112509918	91011	21	159	189.8181818	13.7123292	0.949707726	0.015355635	+	5781	grey	0.014697745	FALSE	PTPN11
Region_4534	chr12	112570380	112898881	328501	75	543	602.9545455	25.43191926	0.812967691	0.01927255	+	22895	grey	0.125226079	FALSE	RPH3A
Region_4535	chr12	112905856	112933219	27363	9	56	78.22727273	9.309706191	0.89332642	0.046497705	+	4938	grey	0.044487913	FALSE	OAS1
Region_4536	chr12	112938051	112976460	38409	12	78	104.9545455	10.75522316	0.882367164	0.024430398	+	4940	grey	0.291730314	FALSE	OAS3
Region_4537	chr1	11297839	11301172	33328	6	40	53.0303030	6.56323020	0.90747949	0.03961480	+	4939	grey	-0.18852966	FALSE	OAS2

	2	5	3				3	1	8	4				2		
Region_4538	chr12	113056730	113098028	41298	10	46	65.39393939	8.868056396	0.665429774	0.069832854	+	1840	grey	0.323778689	FALSE	DTX1
Region_4539	chr12	113098819	113136239	37420	11	71	89.10606061	10.05083814	0.856361889	0.031680458	-	8437	grey	0.056833529	FALSE	RASAL1
Region_4540	chr12	113157173	113185479	28306	9	57	74.33333333	7.690820369	0.723329418	0.055231067	-	79039	grey	-0.021990976	FALSE	DDX54
Region_4541	chr12	113185526	113192368	6842	4	18	34.45454545	6.117268918	0.48545147	0.025978213	+	84934	grey	-0.003874942	FALSE	RITA1
Region_4542	chr12	113195441	113221094	25653	4	21	34.68181818	4.905755147	0.886143064	0.056887329	-	115811	grey	-0.046596607	FALSE	IQCD
Region_4543	chr12	113221050	113298585	77535	19	124	153.8939394	13.04681368	0.846506025	0.023892076	+	53373	grey	-0.015546438	FALSE	TPCN1
Region_4544	chr12	113298759	113359493	60734	26	191	220.9545455	17.17460721	0.662750238	0.023895135	-	80024	grey	0.160553946	FALSE	SLC8B1
Region_4545	chr12	113334672	113359466	24794	11	62	81.96969697	9.769607026	0.59932158	0.034856511	-	105369989	grey	-0.045135474	FALSE	LOC105369989
Region_4546	chr12	113358566	113391629	33063	11	65	93.31818182	11.25393482	0.880126175	0.03194429	+	196463	grey	0.089775309	FALSE	PLBD2
Region_4547	chr12	113392445	113426301	33856	8	47	66.63636364	9.470011557	0.898654518	0.049754016	-	10993	grey	0.046593464	FALSE	SDS
Region_4548	chr12	113422380	113438276	15896	3	16	25.09090909	4.256135021	0.910785686	0.069925974	+	113675	grey	0.066233489	FALSE	SDSL
Region_4549	chr12	113462033	113471871	9838	12	68	91.93939394	9.868175403	0.039144889	0.029295315	-	64211	grey	0.342427183	FALSE	LHX5

Region_4550	chr12	113471981	113480636	8655	6	35	47.89393939	6.476297109	0.040520075	0.036268658	+	104355219	grey	-0.023236097	FALSE	LHX5-AS1
Region_4551	chr12	113583886	113773726	189840	78	564	613.6666667	22.43783435	0.752104644	0.016996164	-	100506465	grey	0.231459999	FALSE	LINC01234
Region_4552	chr12	113816738	113966325	149587	87	616	684.9393939	30.75755256	0.914789482	0.010876468	-	9904	grey	-0.111681529	FALSE	RBM19
Region_4553	chr12	114077133	114113489	36356	6	52	66.06060606	7.394132368	0.791077944	0.064120569	+	105369995	grey	-0.072940161	FALSE	LOC105369995
Region_4554	chr12	114353911	114408442	54531	19	120	163.8030303	14.68355406	0.366132065	0.034315231	-	6910	grey	-0.379776295	FALSE	TBX5
Region_4555	chr12	114408131	114412832	4701	3	19	27.04545455	4.511291196	0.021239616	0.030319954	+	255480	grey	-0.170411616	FALSE	TBX5-AS1
Region_4556	chr12	114670255	114684175	13920	3	13	22.15151515	4.247143562	0.170733195	0.073303767	-	6926	grey	0.117940724	FALSE	TBX3
Region_4557	chr12	114682292	114767989	85697	19	101	131.6363636	12.06091764	0.505261246	0.032405589	+	107984437	grey	-0.07191318	FALSE	
Region_4558	chr12	115582061	115639734	57673	8	40	57.90909091	7.592966933	0.713152487	0.045891399	-	105370002	grey	0.175554887	FALSE	LOC105370002
Region_4559	chr12	115810359	115886137	75778	10	69	90.75757576	9.262099124	0.862204698	0.05143782	-	105370004	grey	-0.134885989	FALSE	LINC02463
Region_4560	chr12	115958576	116277693	319117	46	345	390.1363636	18.2123258	0.897669116	0.015064422	-	23389	grey	-0.159556167	FALSE	MED13L
Region_4561	chr12	116482073	116521047	38974	14	89	106.6212121	9.147955049	0.903996295	0.02772367	+	107984439	grey	0.003976148	FALSE	LINC02457
Region_4562	chr1	11671017	11673807	27899	13	84	106.878787	10.1610344	0.86396538	0.02758328	-	79794	grey	-0.14609000	FALSE	SPRING1

	2	1	0				9		8	6				8		
Region_4563	chr12	116738178	116853631	115453	37	255	299.7272727	17.3733177	0.783073711	0.017488422	+	84900	grey	-0.094232485	FALSE	RNFT2
Region_4564	chr12	116856144	116881441	25297	14	81	107.3030303	11.2002331	0.476065703	0.03150931	-	8739	grey	-0.035071067	FALSE	HRK
Region_4565	chr12	116910950	117031148	120198	43	297	350.8333333	20.37161172	0.884656585	0.016128346	+	26259	grey	-0.03547238	FALSE	FBXW8
Region_4566	chr12	117038923	117099479	60556	40	251	301.7424242	19.50330197	0.548386627	0.023578293	-	54997	grey	0.04539751	FALSE	TESC
Region_4567	chr12	117099481	117142091	42610	19	102	124.7878788	10.24246838	0.589115127	0.032100197	+	101928244	grey	-0.121003259	FALSE	TESC-AS1
Region_4568	chr12	117141991	117190470	48479	32	220	251.1363636	17.59710323	0.912754914	0.017289036	-	23014	grey	0.073517234	FALSE	FBXO21
Region_4569	chr12	117208142	117452170	244028	110	777	881.7878788	39.17829664	0.794062317	0.014300392	-	4842	grey	-0.100084713	FALSE	NOS1
Region_4570	chr12	117453012	117968990	515978	142	1060	1164.151515	40.38592383	0.7964864	0.01480567	-	283455	grey	0.205179681	FALSE	KSR2
Region_4571	chr12	118013588	118033130	19542	6	36	49.12121212	7.336977268	0.502677506	0.022411028	+	5985	grey	-0.135488535	FALSE	RFC5
Region_4572	chr12	118032687	118062430	29743	10	56	79.07575758	9.325417056	0.853735723	0.027603924	-	55884	grey	0.283548289	FALSE	WSB2
Region_4573	chr12	118063593	118104011	40418	16	102	127.530303	10.23901993	0.828816982	0.026679887	-	54621	grey	0.181895894	FALSE	VSIG10
Region_4574	chr12	118095622	118136026	40404	18	118	142.9848485	12.88110414	0.821494768	0.029344565	-	101928274	grey	0.54238676	FALSE	LOC101928274

Region_4575	chr12	118136124	118145584	9460	4	14	28.31818182	4.864815882	0.72431737	0.033671882	+	5037	grey	-0.196962365	FALSE	PEBP1
Region_4576	chr12	118149801	118372907	223106	64	428	492.3030303	27.16896969	0.878811007	0.014346992	-	51347	grey	-0.020516257	FALSE	TAOK3
Region_4577	chr12	118376555	118418033	41478	14	84	106.7121212	11.21642361	0.729826911	0.030948427	+	64426	grey	-0.242090398	FALSE	SUDS3
Region_4578	chr12	118782926	118833536	50610	8	44	62.93939394	8.715819019	0.644531779	0.05892314	-	105370016	grey	0.010695517	FALSE	LINC02439
Region_4579	chr12	118981541	119163051	181510	31	201	258.8787879	19.75660758	0.770610335	0.02643412	+	84530	grey	-0.341024654	FALSE	SRRM4
Region_4580	chr12	119031039	119116961	85922	12	80	105.5454545	10.45750642	0.826229779	0.042756716	-	105370022	grey	-0.355905033	FALSE	LOC105370022
Region_4581	chr12	119179590	119224855	45265	9	47	73.90909091	11.15447792	0.91201829	0.046630871	+	105370026	grey	-0.007569205	FALSE	LOC105370026
Region_4582	chr12	119225834	119259017	33183	7	44	61.07575758	7.089126248	0.807845791	0.053888175	-	105370025	grey	0.011770738	FALSE	LOC105370025
Region_4583	chr12	119283825	119303380	19555	3	20	27.66666667	5.130102213	0.721689463	0.089987955	-	144742	grey	0.008560479	FALSE	LINC00934
Region_4584	chr12	119334712	119541040	206328	27	194	222.3787879	14.58214414	0.846319067	0.023835281	+	160777	grey	0.106093771	FALSE	CCDC60
Region_4585	chr12	119361247	119668079	306832	67	477	538.9545455	28.03263583	0.739510369	0.02382736	-	105370027	grey	-0.238526724	FALSE	LOC105370027
Region_4586	chr12	119593774	119643075	49301	19	125	149.3030303	13.24325047	0.513736678	0.055801569	+	387890	grey	-0.351687678	FALSE	TMEM233
Region_4587	chr1	11966786	11968162	13760	9	53	82.1212121	11.8950735	0.7827581	0.03197641	+	5564	grey	-0.16692519	FALSE	PRKAB1

	2	4	4				2	1						5		
Region_4588	chr12	119685791	119877320	191529	60	424	477.5454545	25.60780039	0.957113581	0.01275179	-	11113	grey	0.147271975	FALSE	CIT
Region_4589	chr12	119989236	120094494	105258	35	216	247.0909091	15.04631544	0.789033854	0.014641332	+	92558	grey	-0.127452974	FALSE	BICDL1
Region_4590	chr12	120095099	120117502	22403	12	61	82.87878788	9.929776745	0.825346309	0.034478895	-	11021	grey	-0.015351905	FALSE	RAB35
Region_4591	chr12	120127202	120194715	67513	29	201	226.3636364	11.90686468	0.905883859	0.018901721	-	10985	grey	-0.053690333	FALSE	GCN1
Region_4592	chr12	120196699	120201235	4536	3	17	25.89393939	4.065346188	0.290556518	0.048552321	-	6175	grey	-0.101168341	FALSE	RPLP0
Region_4593	chr12	120201274	120213233	11959	5	22	37.90909091	6.671932652	0.777812953	0.035360537	+	100506649	grey	0.00502349	FALSE	PXN-AS1
Region_4594	chr12	120210439	120265771	55332	20	121	145.0757576	10.53245622	0.514964656	0.037797635	-	5829	grey	0.090740907	FALSE	PXN
Region_4595	chr12	120302316	120313249	10933	4	18	28.04545455	4.675408063	0.665329424	0.060982942	+	23409	grey	0.157577189	FALSE	SIRT4
Region_4596	chr12	120322115	120327779	5664	5	24	35.66666667	6.450601244	0.798328151	0.072009716	-	5319	grey	0.146470131	FALSE	PLA2G1B
Region_4597	chr12	120341330	120369164	27834	18	119	140.8030303	10.90126273	0.77805118	0.03036532	-	4440	grey	0.064247693	FALSE	MSI1
Region_4598	chr12	120438198	120463749	25551	12	87	122.9545455	12.71151122	0.482169534	0.01508574	+	283459	grey	-0.13681766	FALSE	GATC
Region_4599	chr12	120469850	120498493	28643	10	59	78.71212121	7.420895968	0.761651354	0.025129283	+	8655	grey	0.031286489	FALSE	DYNLL1

Region_4600	chr12	120488079	120495954	7875	3	12	22.92424242	4.804839247	0.9760458	0.042573127	-	100506668	grey	-0.019306841	FALSE	NRAV
Region_4601	chr12	120503279	120534434	31155	18	124	149.2424242	12.26007982	0.732586091	0.031331997	-	84274	grey	-0.280063802	FALSE	COQ5
Region_4602	chr12	120534356	120577588	43232	38	198	244.3181818	20.65925689	0.774063978	0.021546757	+	9921	grey	0.027495138	FALSE	RNF10
Region_4603	chr12	120628830	120641591	12761	12	69	87.01515152	9.384918219	0.554994202	0.023241361	-	101928300	grey	-0.091662007	FALSE	CABP1-DT
Region_4604	chr12	120640626	120667324	26698	29	178	216.1666667	15.101282	0.568387083	0.022180933	+	9478	grey	-0.307479766	FALSE	CABP1
Region_4605	chr12	120687149	120701859	14710	5	23	36.01515152	5.674483844	0.48996756	0.059358969	+	9761	grey	0.10631803	FALSE	MLEC
Region_4606	chr12	120710458	120723640	13182	10	62	82.6969697	9.380781822	0.811122903	0.026720665	+	84747	grey	0.040758459	FALSE	UNC119B
Region_4607	chr12	120725774	120740008	14234	19	108	142.3333333	13.32243144	0.850866975	0.027790103	+	35	grey	-0.106181499	FALSE	ACADS
Region_4608	chr12	120762510	120904358	141848	65	446	506.5	27.98199971	0.923823074	0.012115932	-	121665	grey	-0.295149881	FALSE	SPPL3
Region_4609	chr12	120904702	120907937	3235	4	19	32.13636364	5.423272823	0.934470869	0.036967825	+	105500240	grey	-0.092506568	FALSE	XLOC_009911
Region_4610	chr12	120978543	121002512	23969	10	48	68.22727273	7.897020771	0.951235535	0.028333573	+	6927	grey	-0.04711287	FALSE	HNF1A
Region_4611	chr12	121000486	121016502	16016	7	34	51.28787879	5.814478352	0.801527236	0.04215335	-	64897	grey	0.023410206	FALSE	C12orf43
Region_4612	chr1	12101776	12103924	21483	16	102	126.045454	10.6228082	0.93724324	0.02539467	-	8638	grey	0.154492298	FALSE	OASL

	2	3	6				5	9	9	8						
Region_4613	chr12	121132819	121188032	55213	19	132	154.3484848	11.09745976	0.902977087	0.021579755	+	5027	grey	-0.175618976	FALSE	P2RX7
Region_4614	chr12	121210065	121234106	24041	21	118	148.3030303	11.41115473	0.709038635	0.020187954	+	5025	grey	-0.082822129	FALSE	P2RX4
Region_4615	chr12	121237675	121298308	60633	19	106	138.1212121	12.58755814	0.754082133	0.022225682	-	10645	grey	0.18420006	FALSE	CAMKK2
Region_4616	chr12	121308245	121399896	91651	53	379	419.3939394	21.41234639	0.87171757	0.012359692	-	51433	grey	-0.00891823	FALSE	ANAPC5
Region_4617	chr12	121400083	121430623	30540	15	93	117.8636364	11.40429928	0.905984267	0.025143743	+	80196	grey	-0.292074936	FALSE	RNF34
Region_4618	chr12	121429096	121581023	151927	99	643	736.8030303	36.19911588	0.764149682	0.013021847	-	84678	grey	0.12537224	FALSE	KDM2B
Region_4619	chr12	121580792	121593504	12712	10	63	82.34848485	8.062243292	0.65483725	0.040275841	+	101928346	grey	0.012117713	FALSE	KDM2B-DT
Region_4620	chr12	121626509	121643218	16709	13	73	97.3030303	10.52757507	0.571519798	0.033829378	+	84876	grey	-0.236984329	FALSE	ORAI1
Region_4621	chr12	121648742	121672631	23889	22	141	173.469697	14.19124291	0.827251706	0.026892946	-	283385	grey	0.068708542	FALSE	MORN3
Region_4622	chr12	121687187	121689380	2193	3	15	27.18181818	5.640264835	0.280665757	0.108099303	+	105370034	grey	-0.087220125	FALSE	LOC105370034
Region_4623	chr12	121712752	121782068	69316	56	349	417.8333333	27.28853427	0.891899088	0.011743044	+	144404	grey	-0.086041531	FALSE	TMEM120B
Region_4624	chr12	121777754	121800677	22923	23	132	159.5909091	13.52601741	0.241724841	0.023131976	-	54509	grey	-0.001525804	FALSE	RHOF

Region_4625	chr12	121780952	121803906	22954	22	120	150.7878788	12.91214712	0.189478339	0.023196411	-	338799	grey	-0.049064637	FALSE	LINC01089
Region_4626	chr12	121804009	121832656	28647	26	145	179.1363636	15.81711475	0.577055877	0.021110456	+	23067	grey	0.262127419	FALSE	SETD1B
Region_4627	chr12	121839527	121863596	24069	7	28	44.07575758	6.409873648	0.932998677	0.043650735	-	3242	grey	0.130722738	FALSE	HPD
Region_4628	chr12	121888732	121921470	32738	30	162	204.7272727	14.24785593	0.835416538	0.021891264	+	5715	grey	-0.242152493	FALSE	PSMD9
Region_4629	chr12	121915333	122003927	88594	66	451	530.3484848	25.53458736	0.87349458	0.01286919	+	144406	grey	-0.045697318	FALSE	CFAP251
Region_4630	chr12	122019422	122062044	42622	44	273	323.2121212	23.71660577	0.79543673	0.01961448	+	605	grey	0.010722643	FALSE	BCL7A
Region_4631	chr12	122167738	122203471	35733	35	229	272.6515152	17.42808734	0.661238098	0.020554163	+	254050	grey	-0.080525415	FALSE	LRRC43
Region_4632	chr12	122203681	122208952	5271	7	27	46.46969697	7.309045391	0.40470062	0.051698194	+	79369	grey	0.12790031	FALSE	B3GNT4
Region_4633	chr12	122207662	122227534	19872	21	115	163.2878788	15.04226369	0.758064141	0.017035841	-	56616	grey	-0.159694234	FALSE	DIABLO
Region_4634	chr12	122207779	122266494	58715	69	434	537.2878788	34.18536131	0.832645903	0.010503433	-	65082	grey	-0.146250382	FALSE	VPS33A
Region_4635	chr12	122271432	122422669	151237	76	567	627.8787879	26.15252019	0.867606104	0.012797038	-	6249	grey	0.373825801	FALSE	CLIP1
Region_4636	chr12	122471599	122501073	29474	22	143	170.6363636	12.18780773	0.839322971	0.018118116	-	55596	grey	0.183026972	FALSE	ZCCHC8
Region_4637	chr1	12250345	12252700	23546	10	75	97.0909090	11.4121760	0.94794569	0.02665472	-	65117	grey	0.167984244	FALSE	RSRC2

	2	4	0				9	5	7	1						
Region_4638	chr12	122527246	122626396	99150	21	134	170.1515152	13.98377348	0.929027445	0.012765635	+	9735	grey	0.070396004	FALSE	KNTC1
Region_4639	chr12	122752824	122771064	18240	7	29	44.1969697	7.405236602	0.5541219	0.029675694	+	8562	grey	-0.207888524	FALSE	DENR
Region_4640	chr12	122774526	122827528	53002	27	171	195.8939394	12.16062851	0.858304636	0.023091716	+	84660	grey	0.110887759	FALSE	CCDC62
Region_4641	chr12	122834453	122862961	28508	23	153	185.2727273	16.03606076	0.89437751	0.020553647	+	9026	grey	-0.150524307	FALSE	HIP1R
Region_4642	chr12	122865330	122896127	30797	16	108	131.5454545	10.54686511	0.700459002	0.038699176	-	79720	grey	-0.170496487	FALSE	VPS37B
Region_4643	chr12	122920951	122981649	60698	44	291	337.7575758	19.42485371	0.838877814	0.01717424	-	23457	grey	-0.01803575	FALSE	ABCB9
Region_4644	chr12	122974580	122982907	8327	7	38	51.48484848	6.663892197	0.503322615	0.04990998	+	79676	grey	-0.040134266	FALSE	OGFOD2
Region_4645	chr12	122983480	123151090	167610	75	512	590.0151515	28.3698065	0.796785838	0.013651145	-	57605	grey	-0.16359887	FALSE	PITPNM2
Region_4646	chr12	123152320	123244014	91694	41	275	322.6666667	23.45754485	0.793934145	0.019053923	-	10198	grey	0.11743763	FALSE	MPHOSPH9
Region_4647	chr12	123233385	123258079	24694	19	120	156.6666667	16.71326818	0.705618374	0.022312867	+	91574	grey	-0.069441271	FALSE	MTRFR
Region_4648	chr12	123250112	123272334	22222	23	94	139.969697	19.60295872	0.477715747	0.032125791	-	8099	grey	-0.055673274	FALSE	CDK2AP1
Region_4649	chr12	123289109	123364847	75738	48	355	405.8484848	22.77666986	0.924470198	0.014062216	-	55206	grey	0.323962233	FALSE	SBNO1

Region_4650	chr12	123384132	123409353	25221	16	92	117.0757576	11.4291269	0.615984898	0.036374067	+	387893	grey	0.018411402	FALSE	KMT5A
Region_4651	chr12	123415039	123436684	21645	18	105	135.0606061	11.6141542<br>1	0.694864741	0.044584414	-	196383	grey	-0.483360861	FALSE	RILPL2
Region_4652	chr12	123458088	123473154	15066	25	147	194.9393939	18.04599149	0.785592409	0.020238243	+	11066	grey	0.263540575	FALSE	SNRNP35
Region_4653	chr12	123470054	123533719	63665	32	191	240.5606061	18.11937237	0.908922393	0.027079223	-	353116	grey	0.315739992	FALSE	RILPL1
Region_4654	chr12	123575891	123585115	9224	5	21	32.8030303	5.449685092	0.569118659	0.028315554	-	101927415	grey	0.380465865	FALSE	TMED2-DT
Region_4655	chr12	123584533	123598582	14049	4	12	24.16666667	4.866157332	0.486886178	0.022768345	+	10959	grey	-0.093348513	FALSE	TMED2
Region_4656	chr12	123602077	123620943	18866	5	24	37.13636364	5.732193468	0.957790394	0.033838759	+	57696	grey	-0.01419367	FALSE	DDX55
Region_4657	chr12	123620406	123633766	13360	11	82	102.030303	10.51002408	0.762107931	0.029953645	-	1967	grey	-0.221630326	FALSE	EIF2B1
Region_4658	chr12	123633739	123662604	28865	19	119	138.8636364	11.83981076	0.88055604	0.024462278	+	2967	grey	0.065556937	FALSE	GTF2H3
Region_4659	chr12	123671110	123708399	37289	21	139	162.1060606	11.95134854	0.956524454	0.021020827	+	79867	grey	0.166288456	FALSE	TCTN2
Region_4660	chr12	123712353	123761755	49402	39	251	295.3787879	17.71115698	0.837240733	0.015209966	+	23545	grey	0.206630063	FALSE	ATP6V0A2
Region_4661	chr12	123713408	123723169	9761	10	59	75.57575758	8.489016625	0.845700497	0.030153407	-	105370042	grey	0.213995441	FALSE	LOC105370042
Region_4662	chr1	12397184	12408859	116749	60	406	465.045454	25.5375631	0.71950297	0.01753476	+	144348	grey	-0.19481621	FALSE	ZNF664

	2	5	4				5	7	8	4				4		
Region_4663	chr12	123973219	124316022	342803	193	1375	1511.075758	55.750629	0.837195203	0.010759412	+	100533183	grey	-0.008915925	FALSE	ZNF664-RFLNA
Region_4664	chr12	124289164	124316024	26860	16	94	113.6212121	9.652046657	0.768382582	0.03177207	+	144347	grey	-0.370421039	FALSE	RFLNA
Region_4665	chr12	124324415	124567612	243197	197	1351	1476.772727	67.60263888	0.771192582	0.01142474	-	9612	grey	-0.147703718	FALSE	NCOR2
Region_4666	chr12	124776856	124882668	105812	123	869	953.0909091	35.74857814	0.80484918	0.014073676	-	949	grey	-0.090219509	FALSE	SCARB1
Region_4667	chr12	124911646	124917368	5722	12	79	100.030303	9.843341673	0.081132497	0.011857158	-	7316	grey	0.253845192	FALSE	UBC
Region_4668	chr12	124946825	124989131	42306	59	373	430.4242424	22.42598809	0.949322274	0.010946388	-	57647	grey	-0.017203047	FALSE	DHX37
Region_4669	chr12	124993645	125031231	37586	35	248	285.3333333	15.76298813	0.752362657	0.024990412	+	140707	grey	-0.024073813	FALSE	BRI3BP
Region_4670	chr12	125065434	125143333	77899	57	413	479.2575758	29.92005276	0.941427286	0.010924727	+	65985	grey	-0.102705334	FALSE	AACS
Region_4671	chr12	125186386	125662377	475991	199	1597	1727.772727	53.22312842	0.794268138	0.014162798	+	114795	grey	-0.424310927	FALSE	TMEM132B
Region_4672	chr12	125958688	125983665	24977	6	42	53.31818182	6.19965034	0.865825278	0.062012574	-	400084	grey	0.043278436	FALSE	LINC00939
Region_4673	chr12	125983702	126043485	59783	8	42	64.21212121	8.855272487	0.875613849	0.046195667	+	105370057	grey	0.217903262	FALSE	LINC02826
Region_4674	chr12	126094112	126177632	83520	7	43	59.72727273	7.51520603	0.792468175	0.061816039	+	101927464	grey	0.012074155	FALSE	LINC02359

Region_4675	chr12	126095970	126171797	75827	7	43	59.72727273	7.51520603	0.792468175	0.061816039	+	107984446	grey	0.012074155	FALSE	
Region_4676	chr12	126191151	126434227	243076	32	249	273.5909091	15.78117405	0.832036006	0.026918018	+	105370059	grey	0.003885219	FALSE	LINC02350
Region_4677	chr12	126191281	126406953	215672	27	203	231.2727273	15.57665458	0.82622003	0.026302617	+	105379613	grey	-0.028713348	FALSE	LOC105379613
Region_4678	chr12	126438837	126472790	33953	8	58	78.96969697	8.087920141	0.839136217	0.043941546	+	100128554	grey	0.183570628	FALSE	LINC02347
Region_4679	chr12	126610034	126690322	80288	11	60	86.83333333	8.948714275	0.799217854	0.049157588	-	100996671	grey	-0.154875738	FALSE	LOC100996671
Region_4680	chr12	126688341	126720333	31992	4	16	27.48484848	5.589116964	0.928732876	0.060021949	+	105370060	grey	-0.210221047	FALSE	LINC02824
Region_4681	chr12	126723412	126749027	25615	6	31	51.43939394	7.465054563	0.361711671	0.057655639	+	100507206	grey	-0.137084843	FALSE	LINC00943
Region_4682	chr12	126729787	126772519	42732	3	16	29.83333333	5.266974999	0.65402605	0.091703687	-	387895	grey	-0.229468869	FALSE	LINC00944
Region_4683	chr12	126915199	127060565	145366	17	104	134.2878788	13.10756112	0.840463874	0.029274005	-	101927592	grey	0.160486676	FALSE	LINC02405
Region_4684	chr12	127130005	127207706	77701	11	67	88.45454545	9.813933295	0.70644026	0.040301024	+	107984449	grey	0.13737515	FALSE	LOC107984449
Region_11143	chr2	223052190	223198399	146209	33	246	297.8484848	23.88771324	0.83471074	0.043000805	+	23704	blue	0.571227472	FALSE	KCNE4
Region_4686	chr12	127914707	127960028	45321	12	70	93.1969697	9.812424929	0.913156659	0.033032947	+	100862680	grey	0.002459653	FALSE	LINC00507
Region_4687	chr1	12793368	12798385	50165	11	61	90.2424242	10.3909140	0.92203861	0.03337240	-	10447271	grey	0.003976774	FALSE	LINC00508

	2	9	4				4	9	4	4		8				
Region_4688	chr12	128086621	128118656	32035	6	23	41.25757576	6.587425331	0.881506016	0.058098757	-	100996679	grey	0.543465767	FALSE	LINC02369
Region_4689	chr12	128267170	128707911	440741	105	774	872.8636364	38.56019724	0.793129177	0.015509414	+	92293	grey	-0.167920415	FALSE	TMEM132C
Region_4690	chr12	128793194	128823958	30764	13	83	111.3030303	11.6869022	0.833292857	0.044805543	-	121260	grey	0.154974458	FALSE	SLC15A4
Region_4691	chr12	128853427	128984968	131541	58	409	477.5	27.54534723	0.798292866	0.017308827	+	144423	grey	-0.2517648	FALSE	GLT1D1
Region_4692	chr12	129071725	129904025	832300	236	1888	2029.560606	57.31374618	0.854790497	0.013279119	-	121256	grey	0.054020486	FALSE	TMEM132D
Region_4693	chr12	129208601	129212662	4061	3	13	27.56060606	5.580539781	0.885636733	0.070859685	+	101927735	grey	0.220634845	FALSE	TMEM132D-AS2
Region_4694	chr12	130032928	130045057	12129	4	18	28.09090909	5.037481888	0.687663216	0.064664786	-	100190940	grey	0.100194268	FALSE	LINC02418
Region_4695	chr12	130144315	130162256	17941	8	47	62.6969697	8.205007283	0.693830104	0.056552375	-	440119	grey	-0.104918075	FALSE	FZD10-AS1
Region_4696	chr12	130396133	130768228	372095	197	1452	1623.015152	61.98051615	0.836656971	0.011446607	-	23504	grey	0.21866479	FALSE	RIMBP2
Region_4697	chr12	130789600	130839266	49666	25	166	200.4090909	17.43734037	0.778500515	0.020353571	-	2054	grey	-0.001178616	FALSE	STX2
Region_4698	chr12	130872037	130877678	5641	3	11	18.34848485	3.681523197	0.608946703	0.051855865	+	5901	grey	-0.000554912	FALSE	RAN
Region_4699	chr12	130953907	131141469	187562	121	931	1028.348485	48.44838409	0.858877926	0.013082049	+	283383	grey	0.099518012	FALSE	ADGRD1

Region_4700	chr12	131165011	131232940	67929	26	159	187.969697	13.89960674	0.79141838	0.032104251	+	116437	grey	0.058744555	FALSE	LINC01257
Region_4701	chr12	131347470	131367555	20085	21	135	182.0757576	15.70135771	0.859925134	0.031365359	+	338797	grey	-0.051904696	FALSE	LINC02370
Region_4702	chr12	131447337	131529894	82557	50	343	388.2727273	22.29766835	0.8588662	0.030727077	-	101929974	grey	0.152382006	FALSE	LOC101929974
Region_4703	chr12	131639396	131661823	22427	6	28	42.1969697	7.205168438	0.677824824	0.0841314	-	105370085	grey	-0.460947684	FALSE	LINC02414
Region_4704	chr12	131711081	131799738	88657	69	485	557.2272727	30.10228484	0.904730543	0.0128981	+	6433	grey	-0.133181572	FALSE	SFSWAP
Region_4705	chr12	131828393	131851783	23390	29	159	195.5606061	17.42423492	0.693910965	0.028844603	+	4326	grey	0.15823771	FALSE	MMP17
Region_4706	chr12	131894622	131923150	28528	24	143	176.8030303	15.00073813	0.873764156	0.01717559	+	8408	grey	-0.14845945	FALSE	ULK1
Region_4707	chr12	131924736	131929351	4615	5	17	28.22727273	6.027617094	0.749766635	0.045557079	-	107984455	grey	0.230138631	FALSE	PUS1-AS1
Region_4708	chr12	131929200	131945896	16696	15	107	130.3333333	12.28048274	0.656928805	0.015027188	+	80324	grey	0.362580263	FALSE	PUS1
Region_4709	chr12	131949942	132080460	130518	85	631	704.9090909	31.56713348	0.851240221	0.01009979	+	57634	grey	-0.155211475	FALSE	EP400
Region_4710	chr12	132084275	132131639	47364	44	305	345.0606061	18.78778555	0.91991553	0.014018	+	347918	grey	0.031828756	FALSE	EP400P1
Region_4711	chr12	132489551	132585188	95637	109	638	744.7575758	38.51595498	0.802311002	0.011309058	+	57666	grey	0.261118757	FALSE	FBRSL1
Region_4712	chr1	13260315	13261058	7432	14	75	96.1060606	11.5467328	0.47452457	0.08404770	-	10050705	grey	-0.20216944	FALSE	LRCOL1

	2	0	2				1	6		3		5		8		
Region_4713	chr12	132618776	132622388	3612	12	45	67.68181818	10.68306941	0.275481876	0.061046156	+	22953	grey	0.003599443	FALSE	P2RX2
Region_4714	chr12	132623753	132687376	63623	94	681	774.0151515	40.72968769	0.81800457	0.01546889	-	5426	grey	0.090168077	FALSE	POLE
Region_4715	chr12	132687587	132714912	27325	35	217	251.8787879	18.04312369	0.74201301	0.01633255	+	5827	grey	0.286021644	FALSE	PXMP2
Region_4716	chr12	132768902	132829143	60241	32	217	256.5909091	15.26884941	0.822014362	0.025824246	-	2802	grey	-0.343090526	FALSE	GOLGA3
Region_4717	chr12	132822187	132956304	134117	232	1722	1844.545455	57.30087442	0.85376194	0.008517603	-	55743	grey	0.125863752	FALSE	CHFR
Region_4718	chr12	132918306	132956306	38000	48	340	379.8333333	25.31316674	0.905048733	0.019444854	-	100289635	grey	-0.071380373	FALSE	ZNF605
Region_4719	chr12	132986365	133027102	40737	40	281	345.3787879	23.60297579	0.731365807	0.02084547	+	7574	grey	-0.15532983	FALSE	ZNF26
Region_4720	chr12	133030389	133037222	6833	5	21	37.09090909	6.627976307	0.552280178	0.102651145	-	101928597	grey	-0.096674607	FALSE	ZNF84-DT
Region_4721	chr12	133037292	133063304	26012	11	70	88.63636364	8.967696495	0.708956125	0.027528951	+	7637	grey	-0.078718593	FALSE	ZNF84
Region_4722	chr12	133080474	133107526	27052	16	115	136.969697	11.54918538	0.795214729	0.013104607	+	7699	grey	0.193058119	FALSE	ZNF140
Region_4723	chr12	133104779	133130274	25495	18	123	152.9848485	13.41467901	0.765940309	0.027902893	-	101060200	grey	0.118078623	FALSE	ZNF891
Region_4724	chr12	133130575	133159465	28890	30	213	256.0606061	19.36760085	0.855155078	0.017046332	+	7556	grey	0.109029465	FALSE	ZNF10

Region_4725	chr12	133130984	133214832	83848	61	423	481.4242424	28.72846919	0.909218596	0.020042837	+	10795	grey	0.248414548	FALSE	ZNF268
Region_4726	chr12	133218312	133235877	17565	12	63	89.24242424	12.3824449	0.818103523	0.052705662	-	647589	grey	-0.186096507	FALSE	ANHX
Region_4727	chr13	19173772	19181824	8052	3	11	23.37878788	4.245414364	0.854348135	0.094141301	-	7278	grey	-0.007382588	FALSE	TUBA3C
Region_4728	chr13	19262797	19344973	82176	8	53	69.93939394	7.61350781	0.634731131	0.061268999	-	100101938	grey	0.05478308	FALSE	ANKRD26P3
Region_4729	chr13	19422876	19561625	138749	20	131	164.6060606	13.63578864	0.867085937	0.038387179	-	93492	grey	-0.044995915	FALSE	TPTE2
Region_4730	chr13	19561328	19588603	27275	4	18	35.22727273	5.989209411	0.731577078	0.0425357	+	100874136	grey	0.055484818	FALSE	TPTE2-AS1
Region_4731	chr13	19633659	19673441	39782	11	57	82.53030303	9.315363153	0.763239577	0.043314296	+	54737	grey	0.062096087	FALSE	MPHOSPH8
Region_4732	chr13	19674752	19783019	108267	41	249	298.2272727	20.22473387	0.869982902	0.013344379	-	55269	grey	0.09619346	FALSE	PSPC1
Region_4733	chr13	19823482	19863649	40167	11	62	89.45454545	10.85028439	0.798793924	0.016152136	-	9205	grey	-0.267226417	FALSE	ZMYM5
Region_4734	chr13	19958677	20091829	133152	19	129	156.5909091	12.45790114	0.84372327	0.022699313	+	7750	grey	0.122552921	FALSE	ZMYM2
Region_4735	chr13	20138255	20161052	22797	8	48	62.81818182	8.412278738	0.777502715	0.050268012	-	2700	grey	-0.078053887	FALSE	GJA3
Region_4736	chr13	20187470	20192938	5468	5	32	52.03030303	6.91924784	0.527992988	0.041850472	-	2706	grey	0.092935608	FALSE	GJB2
Region_4737	chr1	20221962	20232365	10403	7	36	48.5454545	6.70518012	0.77454959	0.04946169	-	10804	grey	-0.01444506	FALSE	GJB6

	3						5	9	3	2				6		
Region_4738	chr13	20403666	20525873	122207	56	398	448.2575758	24.53337482	0.830268498	0.016908559	-	51084	grey	0.372117343	FALSE	CRYL1
Region_4739	chr13	20567138	20691444	124306	18	113	133.8484848	11.62258071	0.890812851	0.031960777	+	8100	grey	-0.12963076	FALSE	IFT88
Region_4740	chr13	20702127	20723098	20971	23	135	167.6363636	17.66668866	0.613923153	0.019277133	+	53342	grey	-0.163064493	FALSE	IL17D
Region_4741	chr13	20728731	20773961	45230	23	125	159.2121212	14.55865077	0.918159121	0.017561419	-	221143	grey	-0.18474478	FALSE	EEF1AKMT1
Region_4742	chr13	20777329	20903048	125719	27	187	226.2121212	13.9686629	0.926943241	0.017484029	-	64328	grey	0.220947772	FALSE	XPO4
Region_4743	chr13	20938564	20949428	10864	5	22	35.13636364	5.691792643	0.94894206	0.042765427	-	101930748	grey	-0.118456763	FALSE	LINC00367
Region_4744	chr13	20973036	21061586	88550	52	350	395.0151515	21.04975236	0.794371934	0.018688121	-	26524	grey	-0.154064135	FALSE	LATS2
Region_4745	chr13	21140119	21149097	8978	10	70	96.07575758	10.90421317	0.387119089	0.014442859	+	10284	grey	0.007434966	FALSE	SAP18
Region_4746	chr13	21260015	21293429	33414	12	75	105.6818182	13.17475335	0.913015496	0.040854497	+	105370105	grey	0.083875685	FALSE	LOC105370105
Region_4747	chr13	21283487	21298091	14604	7	38	61.81818182	9.601136296	0.405919639	0.024320893	-	101928764	grey	0.037168727	FALSE	LOC101928764
Region_4748	chr13	21298115	21353228	55113	19	127	155.1666667	12.02380118	0.673554216	0.028421721	+	650794	grey	-0.013221281	FALSE	MIPEPP3
Region_4749	chr13	21302862	21362139	59277	16	93	113.7424242	10.40164278	0.83993148	0.035994079	-	100652865	grey	-0.048277119	FALSE	LINC00539

Region_4750	chr13	21348784	21459370	110586	27	159	200.0757576	17.18345412	0.817234552	0.016091627	-	253832	grey	0.16529697	FALSE	ZDHHC20
Region_4751	chr13	21492691	21604181	111490	33	217	248.7121212	15.97282803	0.906429856	0.014206812	-	221154	grey	0.147577139	FALSE	MICU2
Region_4752	chr13	21671073	21704498	33425	16	91	121.5	11.5867962	0.390394122	0.024158753	+	2254	grey	-0.261040107	FALSE	FGF9
Region_4753	chr13	22040972	22276526	235554	52	423	469.1060606	23.80893742	0.862018547	0.019320237	+	100506622	grey	-0.142919924	FALSE	LINC00540
Region_4754	chr13	22859050	22916369	57319	6	34	46.71212121	6.292278911	0.847702735	0.062389804	-	100996930	grey	-0.324472348	FALSE	LINC00621
Region_4755	chr13	23180979	23325162	144183	25	178	206.3030303	14.79393815	0.858730108	0.034450744	+	6445	grey	-0.121161277	FALSE	SGCG
Region_4756	chr13	23288689	23433763	145074	36	260	302.3787879	18.68423041	0.847020835	0.016878368	-	26278	grey	0.227777655	FALSE	SACS
Region_4757	chr13	23418971	23428869	9898	4	20	30.06060606	5.031833395	0.971631755	0.034359516	+	100506680	grey	0.188109163	FALSE	SACS-AS1
Region_4758	chr13	23465776	23487712	21936	4	17	29.83333333	5.109819606	0.952955855	0.042802674	+	100506697	grey	-0.002985674	FALSE	LINC00327
Region_4759	chr13	23570370	23676104	105734	22	142	170.0151515	13.57315498	0.775576933	0.024504287	+	55504	grey	0.303850776	FALSE	TNFRSF19
Region_4760	chr13	23715152	23735387	20235	7	37	53.40909091	8.099339522	0.951558728	0.032482196	+	105370113	grey	0.082273991	FALSE	LOC105370113
Region_4761	chr13	23730189	23889400	159211	28	171	209.469697	14.58262369	0.939874039	0.018608357	-	4285	grey	-0.029616343	FALSE	MIPEP
Region_4762	chr1	23954493	23970188	15695	4	19	35.0909090	5.79582680	0.87966939	0.04877312	+	10537011	grey	0.005046967	FALSE	LOC105370115

	3						9	8		4		5				
Region_4763	chr13	23979700	24321031	341331	97	768	873.3333333	34.81879834	0.830372731	0.013908104	+	221178	grey	0.104904266	FALSE	SPATA13
Region_4764	chr13	24307166	24322535	15369	9	40	54.15151515	6.975214194	0.793755717	0.061618076	+	338872	grey	0.308609604	FALSE	C1QTNF9
Region_4765	chr13	24408157	24597676	189519	38	290	334.6666667	20.36084729	0.785660562	0.019126917	+	374491	grey	0.182480506	FALSE	TPTE2P6
Region_4766	chr13	24420931	24512778	91847	15	89	117.530303	10.90972706	0.81114081	0.047939696	-	143	grey	-0.020710769	FALSE	PARP4
Region_4767	chr13	24517794	24530461	12667	6	69	88.57575758	9.480442388	0.930844623	0.035261997	+	105370117	grey	0.123322103	FALSE	LOC105370117
Region_4768	chr13	24539139	24541720	2581	3	11	21.6969697	4.478906793	0.963167094	0.046442198	-	105370295	grey	0.064385318	FALSE	LOC105370295
Region_4769	chr13	24680408	24712472	32064	12	71	90.8030303	10.55858338	0.78300475	0.031431497	+	479	grey	0.027808139	FALSE	ATP12A
Region_4770	chr13	24723101	24738690	15589	5	29	42.84848485	7.998542991	0.6400854	0.050840237	-	105370118	grey	0.175816198	FALSE	LOC105370118
Region_4771	chr13	24747658	24879921	132263	29	214	243.5454545	15.83878571	0.864787075	0.02438283	+	56163	grey	0.09341782	FALSE	RNF17
Region_4772	chr13	24882279	24922889	40610	14	77	104.0454545	9.703975106	0.947243062	0.026686054	-	55835	grey	-0.168774649	FALSE	CENPJ
Region_4773	chr13	24924673	24968520	43847	6	38	56.43939394	8.114808473	0.540084193	0.063163368	-	646405	grey	0.082007662	FALSE	TPTE2P1
Region_4774	chr13	25161679	25172288	10609	7	35	50.92424242	7.041220624	0.294128217	0.06135653	-	219287	grey	-0.00731251	FALSE	AMER2

Region_4775	chr13	25168969	25190643	21674	7	34	48.93939394	6.567703703	0.364302303	0.053928748	+	102723318	grey	0.070950056	FALSE	LINC01053
Region_4776	chr13	25246222	25287488	41266	12	74	99.59090909	9.941331396	0.764266442	0.02531115	-	9107	grey	-0.312071615	FALSE	MTMR6
Region_4777	chr13	25301524	25365390	63866	12	78	101.030303	10.26865237	0.822192101	0.018225657	+	9818	grey	0.055764554	FALSE	NUP58
Region_4778	chr13	25371974	26025851	653877	161	1259	1360.5	48.3293986	0.793044394	0.013221991	+	51761	grey	0.10311603	FALSE	ATP8A2
Region_4779	chr13	26044597	26052016	7419	5	31	44.16666667	6.227935411	0.397804283	0.035580137	-	387914	grey	0.106678762	FALSE	SHISA2
Region_4780	chr13	26094724	26222314	127590	17	114	133.969697	10.74740712	0.850664859	0.0332021	-	6049	grey	0.364339855	FALSE	RNF6
Region_4781	chr13	26254104	26420982	166878	24	155	186.2878788	14.2750618	0.929867336	0.01566387	+	1024	grey	-0.148401913	FALSE	CDK8
Region_4782	chr13	26557683	26688948	131265	30	218	248.6515152	15.14391721	0.770305722	0.028094241	+	10810	grey	0.024481319	FALSE	WASF3
Region_4783	chr13	26685640	26698804	13164	5	30	44.12121212	6.215403079	0.752497659	0.050399736	-	107984597	grey	-0.422661692	FALSE	LOC107984597
Region_4784	chr13	26755200	26760786	5586	9	54	71.95454545	10.06659643	0.107113942	0.016391101	-	2835	grey	-0.001478595	FALSE	GPR12
Region_4785	chr13	27066156	27171811	105655	23	150	183.469697	11.45455193	0.914271926	0.027750929	-	219333	grey	0.281105909	FALSE	USP12
Region_4786	chr13	27162855	27169135	6280	3	16	25.39393939	4.328363656	0.764450831	0.10147762	+	100874070	grey	0.194661693	FALSE	USP12-AS1
Region_4787	chr1	27178263	27251288	73025	16	106	132.636363	12.1764417	0.86852247	0.02690815	-	10537012	grey	-0.00818129	FALSE	LINC02340

	3						6	7	6	3		5		6		
Region_4788	chr13	27435643	27450591	14948	7	34	53.39393939	8.23291478	0.418338077	0.034851679	-	219402	grey	-0.217132964	FALSE	MTIF3
Region_4789	chr13	27545913	27620529	74616	20	128	161.6212121	13.64130058	0.721394821	0.02482365	-	222484	grey	0.059322391	FALSE	LNX2
Region_4790	chr13	27620742	27667415	46673	12	76	99.3030303	9.617243643	0.581472194	0.030988198	+	51082	grey	-0.109472095	FALSE	POLR1D
Region_4791	chr13	27621875	27744237	122362	21	151	180.2121212	12.75631385	0.794748429	0.027948706	+	105370129	grey	-0.08632069	FALSE	LOC105370129
Region_4792	chr13	27792483	27794768	2285	3	11	18.21212121	3.971107776	0.155466496	0.069829206	+	219409	grey	-0.147917022	FALSE	GSX1
Region_4793	chr13	27819376	27917298	97922	29	190	240	17.27782038	0.736198447	0.02561522	-	100861550	grey	-0.391024336	FALSE	PLUT
Region_4794	chr13	27920000	27926313	6313	9	49	66.93939394	8.417708053	0.036540816	0.031205238	+	3651	grey	0.3048827	FALSE	PDX1
Region_4795	chr13	27960918	27969315	8397	5	20	30.51515152	4.661602314	0.171323575	0.051913832	-	1045	grey	-0.047334076	FALSE	CDX2
Region_4796	chr13	27977717	27988693	10976	4	17	32.10606061	6.570631132	0.029769625	0.039038346	-	646625	grey	-0.272916685	FALSE	URAD
Region_4797	chr13	28003274	28100592	97318	35	202	241.9393939	18.21316412	0.84675715	0.023249551	-	2322	grey	0.03773803	FALSE	FLT3
Region_4798	chr13	28138506	28295335	156829	25	195	222.6363636	14.91476951	0.946794857	0.017175517	+	255967	grey	-0.062467556	FALSE	PAN3
Region_4799	chr13	28300346	28495145	194799	81	590	654.1060606	34.50401483	0.596097722	0.011129641	-	2321	grey	0.256491421	FALSE	FLT1

Region_4800	chr13	28659065	28678959	19894	7	47	69.93939394	8.501364212	0.943597907	0.03124717	+	51371	grey	-0.092153017	FALSE	POMP
Region_4801	chr13	28700064	28718970	18906	11	72	92.21212121	10.36638822	0.690448677	0.024644555	-	283537	grey	0.061975716	FALSE	SLC46A3
Region_4802	chr13	28820339	29505947	685608	108	856	946.2878788	32.66132276	0.833978668	0.017442357	+	23281	grey	-0.263355485	FALSE	MTUS2
Region_4803	chr13	29509414	29595688	86274	20	142	168.2727273	13.49478014	0.923733298	0.02167553	-	6541	grey	0.12168963	FALSE	SLC7A1
Region_4804	chr13	29764371	29850617	86246	6	25	42.65151515	6.500869585	0.81590345	0.035090214	-	5412	grey	0.018600546	FALSE	UBL3
Region_4805	chr13	30151886	30159621	7735	5	22	34	6.017921952	0.947582294	0.057860301	-	102157400	grey	0.041512578	FALSE	LINC00384
Region_4806	chr13	30153744	30160592	6848	5	20	32.56060606	6.167230491	0.943884713	0.059020877	+	105370141	grey	-0.086953773	FALSE	LINC00385
Region_4807	chr13	30202630	30307551	104921	12	61	87.62121212	9.988863263	0.861645155	0.026531117	-	84056	grey	0.164666108	FALSE	KATNAL1
Region_4808	chr13	30340267	30377145	36878	10	50	70.28787879	8.517430366	0.818986512	0.044905495	-	100188949	grey	-0.157151449	FALSE	LINC00426
Region_4809	chr13	30422488	30429758	7270	3	17	30.18181818	5.610180556	0.647296754	0.023399412	+	171222	grey	0.154050519	FALSE	UBE2L5
Region_4810	chr13	30456704	30617597	160893	33	225	267.7424242	17.70207342	0.85864891	0.015240828	-	3146	grey	0.091221885	FALSE	HMGB1
Region_4811	chr13	30617693	30660770	43077	8	53	75.31818182	8.762084562	0.951641859	0.032094908	+	10208	grey	0.107374384	FALSE	USPL1
Region_4812	chr1	30713478	30764426	50948	7	48	63.0151515	8.03165357	0.66778081	0.04333146	+	241	grey	0.282116195	FALSE	ALOX5AP

	3						2	9	9	2						
Region_4813	chr13	30863369	30883424	20055	4	27	39.66666667	6.150255363	0.882515352	0.064256731	+	440132	grey	0.070069769	FALSE	LINC00545
Region_4814	chr13	30881933	30933846	51913	9	49	67.33333333	8.788687367	0.838418594	0.050765136	-	100507064	grey	0.095276211	FALSE	TEX26-AS1
Region_4815	chr13	30932656	30975500	42844	12	81	102.3333333	10.02663121	0.873606722	0.042090993	+	122046	grey	0.001482124	FALSE	TEX26
Region_4816	chr13	30973289	30977623	4334	3	14	27.86363636	4.94550725	0.940400512	0.049944151	-	105370145	grey	0.017356469	FALSE	LOC105370145
Region_4817	chr13	31037049	31056366	19317	5	21	34.04545455	5.470007223	0.37849578	0.063231185	-	102723392	grey	0.101560967	FALSE	LOC102723392
Region_4818	chr13	31199975	31332276	132301	25	154	185.7272727	13.31459056	0.902629566	0.021399658	+	145173	grey	-0.072428907	FALSE	B3GLCT
Region_4819	chr13	31739526	31803389	63863	22	164	206.4545455	14.82843611	0.912948512	0.022883922	+	122042	grey	0.079381719	FALSE	RXFP2
Region_4820	chr13	31796368	31846539	50171	12	65	84.33333333	8.87751409	0.75916817	0.042120222	-	105370152	grey	0.18172316	FALSE	LOC105370152
Region_4821	chr13	31846713	32299125	452412	63	483	527.7424242	21.44424657	0.911029027	0.013150322	+	10129	grey	-0.050557428	FALSE	FRY
Region_4822	chr13	31846841	31991931	145090	22	147	179.7121212	13.72562472	0.885277661	0.025501278	+	196549	grey	-0.092568192	FALSE	EEF1DP3
Region_4823	chr13	32315086	32400268	85182	16	102	128.5454545	10.84602986	0.922213467	0.018752081	+	675	grey	0.077102168	FALSE	BRCA2
Region_4824	chr13	32400723	32432954	32231	13	99	122	10.36710775	0.511887545	0.028079563	-	90634	grey	-0.149070168	FALSE	N4BP2L1

Region_4825	chr13	32432417	32538885	106468	16	113	138.9242424	13.1269665	0.954661149	0.023133423	-	10443	grey	0.010519435	FALSE	N4BP2L2
Region_4826	chr13	32586452	32778019	191567	13	67	92.46969697	10.98000938	0.798666723	0.029610847	+	23047	grey	0.060609464	FALSE	PDS5B
Region_4827	chr13	32877431	32911650	34219	6	37	50.09090909	6.602394141	0.952338529	0.032806273	-	100874167	grey	-0.03436124	FALSE	LINC00423
Region_4828	chr13	33016423	33066143	49720	13	69	85.51515152	8.438451233	0.675294303	0.032365338	+	9365	grey	0.103686229	FALSE	KL
Region_4829	chr13	33103137	33676820	573683	72	576	630.8181818	27.85727862	0.853018785	0.01199793	-	90627	grey	0.039410732	FALSE	STARD13
Region_4830	chr13	33180401	33281584	101183	11	83	104.2272727	10.85682409	0.84312272	0.04220936	+	100874241	grey	0.000718741	FALSE	STARD13-AS
Region_4831	chr13	33679474	33687175	7701	3	11	19.28787879	4.436787304	0.943974502	0.080486762	+	107984551	grey	-0.125152876	FALSE	LOC107984551
Region_4832	chr13	33818069	33966558	148489	18	110	150.7121212	13.44477611	0.914154809	0.021009082	+	5983	grey	0.063793054	FALSE	RFC3
Region_4833	chr13	34347986	34616216	268230	23	162	188.3181818	12.48156683	0.811721853	0.034049907	+	105370158	grey	-0.066003076	FALSE	LINC02343
Region_4834	chr13	34435450	34640803	205353	14	93	118.6515152	10.10939004	0.845819661	0.035095796	-	100874179	grey	0.165120879	FALSE	LINC00457
Region_4835	chr13	34624514	34696612	72098	6	39	57.60606061	7.450098167	0.808182107	0.060911759	-	105370159	grey	-0.069561117	FALSE	LOC105370159
Region_4836	chr13	34942270	35673022	730752	59	418	499.7424242	21.69105495	0.786350674	0.01569339	+	26960	grey	-0.017786971	FALSE	NBEA
Region_4837	chr1	35473789	35476689	2900	3	17	34.8636363	6.12406693	0.09460673	0.0561429	-	4081	grey	-0.35600792	FALSE	MAB21L1

	3						6	3	3					9		
Region_4838	chr13	35768652	36131382	362730	41	351	394.6363636	20.03891319	0.853084413	0.023933528	-	9201	grey	0.050546952	FALSE	DCLK1
Region_4839	chr13	36168217	36214588	46371	6	31	45.06060606	6.449517067	0.946519652	0.040458087	-	54937	grey	-0.011877582	FALSE	SOHLH2
Region_4840	chr13	36168794	36297842	129048	18	126	156.0909091	13.74686232	0.884353221	0.024318538	-	100526761	grey	-0.017718211	FALSE	CCDC169-SOHLH2
Region_4841	chr13	36222008	36297840	75832	11	75	100.2727273	10.98046581	0.845648364	0.030560446	-	728591	grey	-0.005786192	FALSE	CCDC169
Region_4842	chr13	36301638	36370180	68542	11	54	74.24242424	9.305185661	0.597629786	0.035061696	-	23111	grey	0.177266014	FALSE	SPART
Region_4843	chr13	36431520	36442870	11350	3	14	24.51515152	4.192456318	0.327468456	0.034101727	+	8900	grey	0.090319292	FALSE	CCNA1
Region_4844	chr13	36674020	36697839	23819	3	10	22	4.832581728	0.412931639	0.08768856	+	400120	grey	-0.101604704	FALSE	SERTM1
Region_4845	chr13	36844831	36920765	75934	20	121	151.0151515	13.51977745	0.530857911	0.024204126	-	4093	grey	0.121397122	FALSE	SMAD9
Region_4846	chr13	36949738	37000763	51025	9	67	83.63636364	8.238066173	0.862194309	0.024541772	-	29880	grey	-0.014714629	FALSE	ALG5
Region_4847	chr13	36998816	37025881	27065	8	41	59.98484848	7.378747912	0.847523306	0.030544113	+	11340	grey	0.14576229	FALSE	EXOSC8
Region_4848	chr13	37009312	37059713	50401	5	23	42.57575758	6.610156768	0.966191304	0.041314105	-	55578	grey	0.63591437	FALSE	SUPT20H
Region_4849	chr13	37562583	37598844	36261	6	36	51.34848485	7.5945171	0.806414157	0.059615059	-	10631	grey	0.107229953	FALSE	POSTN

Region_4850	chr13	37632063	37869802	237739	21	146	174.4848485	12.97481872	0.849666528	0.028037899	-	7223	grey	0.015141413	FALSE	TRPC4
Region_4851	chr13	37934298	38066444	132146	8	34	63.46969697	9.537326987	0.798859645	0.051502205	+	101929077	grey	-0.026660914	FALSE	LINC02334
Region_4852	chr13	38050817	38350259	299442	16	104	125.8787879	12.94189993	0.771705842	0.048207012	-	100874188	grey	0.033428308	FALSE	LINC00571
Region_4853	chr13	38567715	38622671	54956	10	62	83.71212121	9.549490766	0.868937335	0.042732296	+	100874147	grey	-0.029112866	FALSE	LINC00366
Region_4854	chr13	38687077	38887131	200054	13	86	105.7878788	10.71808702	0.8793223	0.035266177	+	341640	grey	-0.28905721	FALSE	FREM2
Region_4855	chr13	38965925	38990859	24934	3	17	25.96969697	4.693595299	0.926035593	0.047802622	-	161003	grey	-0.025716527	FALSE	STOML3
Region_4856	chr13	39209116	39603528	394412	39	287	325.0909091	14.88802025	0.882241948	0.016145503	-	10186	grey	0.010701502	FALSE	LHFPL6
Region_4857	chr13	39655627	39791665	136038	14	81	105.6212121	10.92881182	0.765404219	0.044533001	+	57511	grey	-0.194269099	FALSE	COG6
Region_4858	chr13	40172505	40195361	22856	5	17	29.75757576	5.244955396	0.930712612	0.062464337	+	100874127	grey	-0.078615582	FALSE	LINC00332
Region_4859	chr13	40194509	40350313	155804	9	43	58.01515152	7.214285879	0.899770088	0.041577625	-	400123	grey	0.145786641	FALSE	LINC00548
Region_4860	chr13	40198074	40535807	337733	40	274	315.9242424	18.52010185	0.889103002	0.019460753	-	646982	grey	-0.190540525	FALSE	LINC00598
Region_4861	chr13	40469955	40666641	196686	42	304	345.2424242	19.80680583	0.768975348	0.015985798	-	2308	grey	-0.150439359	FALSE	FOXO1
Region_4862	chr1	40729128	40771190	42062	9	56	79.0454545	9.11369522	0.86925258	0.02035233	-	10240	grey	0.125476125	FALSE	MRPS31

	3						5		8	6						
Region_4863	chr1<br>3	40789412	40812460	23048	9	49	66.1060606<br>1	7.65632727<br>4	0.81138019<br>8	0.04183445<br>1	+	10166	grey	0.142761122	FALSE	SLC25A15
Region_4864	chr1<br>3	40812309	40921749	109440	5	25	36.7121212<br>1	6.88982561<br>2	0.93063006<br>7	0.06429151<br>5	-	10061666<br>8	grey	0.235955694	FALSE	TPTE2P5
Region_4865	chr1<br>3	40931924	41061440	129516	24	169	205.015151<br>5	15.3717947<br>7	0.71077363<br>5	0.02157214	-	1997	grey	-0.09768739<br>1	FALSE	ELF1
Region_4866	chr1<br>3	41061509	41084006	22497	9	61	78.7727272<br>7	7.86383240<br>8	0.64982800<br>3	0.01817726<br>9	+	11193	grey	-0.03575778<br>6	FALSE	WBP4
Region_4867	chr1<br>3	41127569	41132802	5233	4	20	36.9545454<br>5	7.40152129<br>5	0.56136563<br>5	0.06746979<br>8	-	89890	grey	0.097425572	FALSE	KBTBD6
Region_4868	chr1<br>3	41132928	41236686	103758	18	141	164.590909<br>1	10.8471581<br>1	0.80880394<br>2	0.01438065<br>7	+	10192914<br>0	grey	0.093865706	FALSE	KBTBD6-DT
Region_4869	chr1<br>3	41189834	41194569	4735	5	33	46.7727272<br>7	6.47550521<br>8	0.38616211<br>2	0.02398186<br>7	-	84078	grey	0.147729738	FALSE	KBTBD7
Region_4870	chr1<br>3	41216369	41263577	47208	19	125	150.742424<br>2	12.0898057<br>6	0.97343001<br>5	0.01651426<br>7	-	9617	grey	0.114186774	FALSE	MTRF1
Region_4871	chr1<br>3	41311267	41377030	65763	16	112	142.560606<br>1	13.2793984<br>9	0.86154581<br>1	0.02730003<br>8	+	79612	grey	0.214283462	FALSE	NAA16
Region_4872	chr1<br>3	41517168	41590462	73294	16	113	137.984848<br>5	11.9711093<br>6	0.94751275	0.02586366<br>7	+	10537017<br>4	grey	-0.14422966	FALSE	LOC105370174
Region_4873	chr1<br>3	41566835	41961120	394285	46	323	370.378787<br>9	18.8514558<br>9	0.91988403<br>8	0.01570103<br>6	-	23078	grey	-0.08466944<br>9	FALSE	VWA8
Region_4874	chr1<br>3	41955808	41981565	25757	4	18	30.5	5.51152638<br>4	0.56313053<br>5	0.06424011<br>6	+	10050724<br>0	grey	-0.11404232<br>8	FALSE	VWA8-AS1

Region_4875	chr13	42040036	42256578	216542	25	184	224.2272727	18.71798432	0.941742435	0.020198676	+	160851	grey	0.000500078	FALSE	DGKH
Region_4876	chr13	42339188	42485956	146768	13	76	96.93939394	9.154870562	0.912831046	0.038604311	+	105370177	grey	-0.24697652	FALSE	LINC02341
Region_4877	chr13	42562736	42608013	45277	6	40	57.48484848	7.64046399	0.655211658	0.030927734	+	8600	grey	0.055673769	FALSE	TNFSF11
Region_4878	chr13	42886388	42992271	105883	10	61	83.15151515	8.868687223	0.759168916	0.026311564	-	94240	grey	-0.106850528	FALSE	EPSTI1
Region_4879	chr13	43023203	43114213	91010	9	58	81.53030303	9.386110355	0.976774407	0.020679956	+	29103	grey	0.037647877	FALSE	DNAJC15
Region_4880	chr13	43213130	43786972	573842	57	443	489.7424242	22.19376947	0.81349148	0.018099233	-	55068	grey	0.054251965	FALSE	ENOX1
Region_4881	chr13	43823909	43879740	55831	4	29	41.59090909	6.164357282	0.631202252	0.051753067	-	160857	grey	-0.359480762	FALSE	CCDC122
Region_4882	chr13	43879284	44028526	149242	16	112	135.8030303	12.21124286	0.888220199	0.02129242	+	144811	grey	0.025250074	FALSE	LACC1
Region_4883	chr13	43908669	44026841	118172	15	105	127.5909091	11.76955299	0.947434879	0.022711915	+	107984576	grey	0.025250074	FALSE	
Region_4884	chr13	43908727	44030461	121734	15	105	127.5909091	11.76955299	0.947434879	0.022711915	+	121838	grey	0.025250074	FALSE	NRAD1
Region_4885	chr13	44094822	44147765	52943	5	27	38.93939394	5.582607022	0.916569488	0.044608866	-	105370183	grey	0.297685796	FALSE	LINC00390
Region_4886	chr13	44110451	44240517	130066	11	71	96.93939394	9.153189924	0.825751437	0.034099245	+	101929212	grey	0.261852159	FALSE	SMIM2-AS1
Region_4887	chr1	44373665	44397714	24049	6	30	44.6060606	7.02934243	0.77907682	0.03811296	+	387923	grey	-0.15887059	FALSE	SERP2

	3						1	1	9	7						
Region_4888	chr13	44399540	44405984	6444	3	10	18.25757576	4.329898226	0.844788606	0.106297866	-	400128	grey	-0.028780116	FALSE	TUSC8
Region_4889	chr13	44432143	44577147	145004	23	154	194.2575758	14.65218346	0.684803558	0.017767433	-	8848	grey	0.076439507	FALSE	TSC22D1
Region_4890	chr13	44575893	44580432	4539	4	18	30	5.922577402	0.004312375	0.013705453	+	641467	grey	0.171521188	FALSE	TSC22D1-AS1
Region_4891	chr13	44651812	44701016	49204	6	28	41.13636364	5.86747584	0.94466738	0.037789822	+	106144541	grey	-0.006046818	FALSE	LINC00407
Region_4892	chr13	44939249	44989471	50222	15	112	133.3333333	9.308942489	0.94059356	0.025128619	-	26747	grey	0.051952715	FALSE	NUFIP1
Region_4893	chr13	44982077	45083125	101048	27	170	218.2272727	14.48585034	0.757992771	0.015147003	-	105370188	grey	-0.035850733	FALSE	LOC105370188
Region_4894	chr13	44989538	45037669	48131	14	83	110.7878788	10.014628	0.792860513	0.019995068	+	55425	grey	0.218187264	FALSE	GPALPP1
Region_4895	chr13	45120510	45284893	164383	36	234	265.0151515	15.56474559	0.923316162	0.011399498	+	2963	grey	-0.149314043	FALSE	GTF2F2
Region_4896	chr13	45341345	45417975	76630	15	94	125.1515152	11.4706883	0.748388212	0.041116066	+	100190939	grey	-0.163165656	FALSE	TPT1-AS1
Region_4897	chr13	45393316	45418455	25139	8	58	76.04545455	7.275738013	0.367414152	0.056896272	-	253512	grey	-0.009125638	FALSE	SLC25A30
Region_4898	chr13	45418162	45420371	2209	5	19	29.36363636	5.128011651	0.355735931	0.062712548	+	100874259	grey	0.346911268	FALSE	SLC25A30-AS1
Region_4899	chr13	45464898	45536701	71803	9	38	61.25757576	8.188755153	0.643046165	0.032481134	+	83548	grey	-0.028788292	FALSE	COG3

Region_4900	chr13	45534522	45615739	81217	13	80	107.3030303	11.6420586<br>3	0.91118260<br>1	0.04524258<br>5	-	220081	grey	0.131305168	FALSE	ERICH6B
Region_4901	chr13	45680182	45701187	21005	4	19	32.3030303	5.84668136<br>5	0.92264114<br>8	0.05795163<br>2	-	10375258<br>1	grey	-0.25690235<br>8	FALSE	LINC01055
Region_4902	chr13	45777242	45851753	74511	11	72	95.2727272<br>7	8.9503682	0.93953148<br>8	0.02435307<br>9	-	283514	grey	-0.22891796<br>4	FALSE	SIAH3
Region_4903	chr13	45954465	46052759	98294	25	129	179.803030<br>3	15.8028426<br>8	0.73718444	0.01802064<br>6	-	23091	grey	0.011121963	FALSE	ZC3H13
Region_4904	chr13	46052497	46161379	108882	19	151	181.348484<br>8	13.7745500<br>3	0.58334784<br>5	0.02303694<br>8	+	10050989<br>4	grey	-0.03953894<br>4	FALSE	CPB2-AS1
Region_4905	chr13	46053186	46105033	51847	3	11	22.7878787<br>9	4.52859343<br>6	0.95455040<br>2	0.06665119	-	1361	grey	-0.07810452<br>5	FALSE	CPB2
Region_4906	chr13	46125920	46211871	85951	16	132	152.787878<br>8	11.8575067<br>4	0.73274043<br>4	0.03124438	-	3936	grey	-0.23662445<br>1	FALSE	LCP1
Region_4907	chr13	46334681	46438190	103509	19	124	159.287878<br>8	14.2362107<br>7	0.79365883<br>4	0.02215839<br>8	-	80183	grey	-0.20157413	FALSE	RUBCNL
Region_4908	chr13	46455131	46515958	60827	14	72	98.4545454<br>5	12.8460491<br>6	0.87939196<br>8	0.03424821<br>6	-	10192934<br>4	grey	0.138255977	FALSE	LINC01198
Region_4909	chr13	46553168	46753040	199872	32	211	250	16.6520962	0.79938277<br>5	0.02379631<br>2	+	23143	grey	0.166899332	FALSE	LRCH1
Region_4910	chr13	46771256	46797420	26164	3	14	26.5	5.00538171<br>9	0.33421704<br>6	0.01584400<br>5	-	2098	grey	-0.37813752	FALSE	ESD
Region_4911	chr13	46831546	46897076	65530	7	37	55.9393939<br>4	7.90497173<br>2	0.66382755<br>5	0.03728357<br>4	-	3356	grey	0.14470679	FALSE	HTR2A
Region_4912	chr1	47745736	48037968	292232	25	164	197.681818	14.4341039	0.65822102	0.01924776	-	8803	grey	0.106196157	FALSE	SUCLA2

	3						2	4		1						
Region_4913	chr13	47746438	47935821	189383	7	37	53.51515152	6.39288649	0.931986463	0.049459952	-	107984570	grey	0.031413275	FALSE	LINC00444
Region_4914	chr13	48037726	48047221	9495	4	22	39.92424242	7.365847606	0.005231017	0.011154451	+	55270	grey	0.056443242	FALSE	NUDT15
Region_4915	chr13	48053323	48095131	41808	5	22	34.53030303	5.735770881	0.394875049	0.027110688	-	29079	grey	-0.295403133	FALSE	MED4
Region_4916	chr13	48232612	48270357	37745	3	19	28.83333333	4.963197894	0.631632433	0.035980476	+	9445	grey	0.012629325	FALSE	ITM2B
Region_4917	chr13	48303744	48599436	295692	36	247	302.8636364	21.22544653	0.801930215	0.024021159	+	5925	grey	-0.098504781	FALSE	RB1
Region_4918	chr13	48389567	48444704	55137	3	18	29.59090909	5.380812843	0.749864601	0.13076886	-	10161	grey	-0.21716403	FALSE	LPAR6
Region_4919	chr13	48488963	48533256	44293	4	24	37.95454545	6.239900932	0.97665461	0.033374006	-	1102	grey	-0.161064975	FALSE	RCBTB2
Region_4920	chr13	48576974	48590587	13613	4	18	29.78787879	6.947086055	0.953974456	0.046147166	-	100129597	grey	-0.206879784	FALSE	LINC00462
Region_4921	chr13	48653711	48711226	57515	4	17	26.98484848	4.362400272	0.801022648	0.080607907	+	57105	grey	-0.21493729	FALSE	CYSLTR2
Region_4922	chr13	48975912	49209779	233867	21	137	169.8787879	12.94189993	0.916377794	0.024047776	+	22862	grey	-0.190006172	FALSE	FNDC3A
Region_4923	chr13	49220338	49222377	2039	6	28	43.8030303	8.057905238	0.213375366	0.052705112	+	2862	grey	-0.066470134	FALSE	MLNR
Region_4924	chr13	49308650	49444064	135414	11	58	86.15151515	9.412336298	0.879667452	0.035009093	-	81617	grey	-0.222199671	FALSE	CAB39L

Region_4925	chr13	49444274	49495003	50729	4	15	31.07575758	6.419467042	0.730649159	0.027821964	+	83852	grey	0.187479889	FALSE	SETDB2
Region_4926	chr13	49495610	49528981	33371	3	14	24.86363636	4.5905802	0.652406094	0.026676633	+	51131	grey	-0.466927248	FALSE	PHF11
Region_4927	chr13	49531946	49585558	53612	14	82	116.8636364	12.45284813	0.788124523	0.036148593	-	55213	grey	-0.289162361	FALSE	RCBTB1
Region_4928	chr13	49660674	49691486	30812	9	52	73.13636364	7.791289739	0.646605701	0.035413831	-	84650	grey	0.11525136	FALSE	EBPL
Region_4929	chr13	49699320	49792682	93362	16	92	115.0454545	10.35884136	0.855595601	0.014327584	-	3839	grey	0.086129948	FALSE	KPNA3
Region_4930	chr13	49956733	50125720	168987	28	150	191.3484848	16.56362319	0.650051946	0.019015364	-	8847	grey	0.051984345	FALSE	DLEU2
Region_4931	chr13	49995888	50018467	22579	4	18	31.40909091	5.406484064	0.962324292	0.033451282	+	10206	grey	-0.13921331	FALSE	TRIM13
Region_4932	chr13	49996813	50020922	24109	4	18	31.40909091	5.406484064	0.962324292	0.033451282	+	283518	grey	-0.13921331	FALSE	KCNRG
Region_4933	chr13	50082169	50654079	571910	103	785	869.2272727	33.48928437	0.785754441	0.010838363	+	10301	grey	0.374240669	FALSE	DLEU1
Region_4934	chr13	50390198	50906856	516658	64	488	555.7121212	25.64717117	0.861227646	0.014004556	+	107984567	grey	0.16410017	FALSE	
Region_4935	chr13	50519364	50843613	324249	39	295	341.7121212	20.88265025	0.867518611	0.016795618	-	107984568	grey	0.200248448	FALSE	LOC107984568
Region_4936	chr13	50695648	50843939	148291	19	127	157.9090909	12.39941348	0.836546231	0.020828711	-	220107	grey	0.079513436	FALSE	DLEU7
Region_4937	chr1	50807855	50849905	42050	4	27	40.5	6.14754485	0.76860845	0.04297162	+	10087407	grey	0.113629407	FALSE	DLEU7-AS1

	3							1	9	1		4				
Region_4938	chr13	50862172	50910764	48592	5	33	46.31818182	6.700537501	0.663470532	0.055384771	-	100874255	grey	-0.172784021	FALSE	RNASEH2B-AS1
Region_4939	chr13	50909747	51024120	114373	9	66	88.33333333	9.927945536	0.751672259	0.033466103	+	79621	grey	-0.195272168	FALSE	RNASEH2B
Region_4940	chr13	50994511	51080862	86351	7	34	49.6969697	7.323239504	0.946324522	0.0370596	-	2974	grey	0.363905282	FALSE	GUCY1B2
Region_4941	chr13	51082119	51200252	118133	13	81	102.5909091	9.990729969	0.905427725	0.031799495	-	647166	grey	0.039946263	FALSE	C13orf42
Region_4942	chr13	51222334	51284239	61905	13	78	105.1969697	9.625638356	0.941430194	0.031485628	+	220108	grey	0.266534671	FALSE	FAM124A
Region_4943	chr13	51335773	51364735	28962	5	27	38.6969697	5.694720084	0.929478707	0.049992594	+	647174	grey	0.124162623	FALSE	SERPINE3
Region_4944	chr13	51354077	51454264	100187	15	101	122.8030303	10.59204302	0.846008155	0.016381715	-	26512	grey	0.147050334	FALSE	INTS6
Region_4945	chr13	51452370	51549875	97505	9	54	77.72727273	8.157196831	0.830410745	0.035636517	+	100507398	grey	0.167044514	FALSE	INTS6-AS1
Region_4946	chr13	51584455	51767709	183254	27	224	251.3333333	17.37844229	0.849794995	0.017832667	+	115825	grey	0.052429463	FALSE	WDFY2
Region_4947	chr13	51763951	51845159	81208	31	222	267.8181818	16.01144695	0.793001874	0.026272023	-	79758	grey	-0.070332114	FALSE	DHRS12
Region_4948	chr13	51804297	51813832	9535	6	35	54.12121212	7.231374274	0.790312302	0.041041268	+	107984561	grey	-0.134012703	FALSE	LOC107984561
Region_4949	chr13	51813347	51845177	31830	11	64	87.43939394	8.999365427	0.77501324	0.036405436	-	283521	grey	0.242898401	FALSE	TMEM272

Region_4950	chr13	51930436	52012125	81689	8	40	55.39393939	6.669975869	0.88836203	0.046746922	-	540	grey	-0.092749429	FALSE	ATP7B
Region_4951	chr13	52012391	52079232	66841	7	28	46.27272727	7.612834213	0.984280129	0.019569069	+	440138	grey	-0.092124075	FALSE	ALG11
Region_4952	chr13	52024691	52033600	8909	3	12	26.03030303	4.9674934	0.97872664	0.032768674	+	9724	grey	-0.200891847	FALSE	UTP14C
Region_4953	chr13	52033611	52129092	95481	9	39	56.1969697	7.971525666	0.976009766	0.020203356	-	341676	grey	-0.15246958	FALSE	NEK5
Region_4954	chr13	52132639	52159861	27222	11	54	71.86363636	8.964693763	0.495514047	0.029659124	-	4752	grey	0.293410998	FALSE	NEK3
Region_4955	chr13	52167709	52291557	123848	22	133	170.2272727	14.9293675	0.710800145	0.022929098	-	100887750	grey	-0.027131797	FALSE	MRPS31P5
Region_4956	chr13	52219344	52334051	114707	14	82	114.4848485	16.51036264	0.947345374	0.022754052	-	644623	grey	-0.033989546	FALSE	TPTE2P2
Region_4957	chr13	52334295	52341896	7601	3	12	24.83333333	4.984848839	0.949831243	0.040642697	+	103191607	grey	0.166822128	FALSE	LINC02333
Region_4958	chr13	52377167	52416373	39206	30	188	235.4090909	18.84509916	0.895339729	0.013986252	-	55901	grey	-0.159285098	FALSE	THSD1
Region_4959	chr13	52412602	52450634	38032	14	82	103.1363636	9.78992632	0.90177965	0.016060161	-	51028	grey	-0.061574148	FALSE	VPS36
Region_4960	chr13	52455429	52476628	21199	4	17	32.28787879	5.932349778	0.971441487	0.029267224	+	26586	grey	-0.129700851	FALSE	CKAP2
Region_4961	chr13	52488993	52587095	98102	14	69	111.8484848	17.55540015	0.782238921	0.036584688	+	220115	grey	-0.052527758	FALSE	TPTE2P3
Region_4962	chr1	52600042	52643773	43731	13	61	96.4696969	11.7056458	0.78951937	0.02533837	+	144983	grey	0.058335428	FALSE	HNRNPA1L2

	3						7	4	3	5						
Region_4963	chr13	52652709	52700909	48200	8	37	57.37878788	7.951323357	0.831632656	0.028064413	+	10910	grey	0.053023127	FALSE	SUGT1
Region_4964	chr13	52703264	52739820	36556	10	63	81.8030303	8.925709964	0.525411515	0.028756625	-	11061	grey	-0.055990862	FALSE	CNMD
Region_4965	chr13	52842889	52848641	5752	10	71	94.06060606	11.44334377	0.138154404	0.04286213	-	5100	grey	-0.292640995	FALSE	PCDH8
Region_4966	chr13	53028813	53052057	23244	8	43	62.03030303	6.985632942	0.938540896	0.030968817	+	10562	grey	-0.19070519	FALSE	OLFM4
Region_4967	chr13	53099397	53151914	52517	7	38	52.24242424	6.523473841	0.886452869	0.070048747	-	102723875	grey	-0.142455442	FALSE	LINC01065
Region_4968	chr13	53815419	53939960	124541	7	39	55.5	6.930978397	0.830566012	0.058222577	+	100861552	grey	-0.253748055	FALSE	LINC00558
Region_4969	chr13	57631744	57729311	97567	17	126	153.969697	13.32893867	0.676002532	0.033190376	+	27253	grey	0.106116681	FALSE	PCDH17
Region_4970	chr13	58165827	58209483	43656	9	59	76.06060606	8.005534216	0.842085218	0.044149294	+	101926897	grey	0.161827959	FALSE	LINC02338
Region_4971	chr13	59121879	59230636	108757	11	70	95.15151515	9.340140708	0.826542404	0.057646986	-	105370224	grey	0.179463302	FALSE	LOC105370224
Region_4972	chr13	59665583	60163928	498345	37	253	306.3181818	16.75264304	0.895329792	0.016378277	-	81624	grey	0.111779666	FALSE	DIAPH3
Region_4973	chr13	60396457	60573878	177421	11	81	104.2424242	9.863780842	0.76436062	0.030162673	+	81550	grey	-0.180804986	FALSE	TDRD3
Region_4974	chr13	60618991	61176758	557767	39	257	296.5909091	16.92788075	0.81321861	0.03133412	+	101926930	grey	-0.013750156	FALSE	LINC00378

Region_4975	chr13	60916981	60945955	28974	3	13	20.56060606	4.141034107 8	0.704987311	0.138585166	+	103456507	grey	-0.049902758	FALSE	LINC01442
Region_4976	chr13	61409685	61427849	18164	5	30	42.75757576	6.285662822	0.5232663	0.06012354	-	64881	grey	0.000278532	FALSE	PCDH20
Region_4977	chr13	62539351	62574905	35554	4	21	30.86363636	5.426108858	0.830749953	0.082737785	-	105370232	grey	-0.025970114	FALSE	LOC105370232
Region_4978	chr13	62672285	62796714	124429	7	39	60.57575758	7.704265764	0.853232656	0.056351121	-	100874176	grey	-0.042741739	FALSE	LINC00448
Region_4979	chr13	63183096	63328129	145033	12	72	92.28787879	9.243318005	0.77054865	0.044793695	-	104355293	grey	-0.112295388	FALSE	LINC00376
Region_4980	chr13	63667682	63738018	70336	3	27	37.77272727	5.919447621	0.881760535	0.061566364	-	100874157	grey	-0.027144556	FALSE	LINC00395
Region_4981	chr13	63851197	64076044	224847	15	118	143.3030303	10.75170068	0.828558707	0.043215725	-	144766	grey	0.071350257	FALSE	LINC00355
Region_4982	chr13	65866070	65922731	56661	3	12	25.10606061	5.02953978	0.781539415	0.096587823	+	105370242	grey	-0.09133506	FALSE	LINC01052
Region_4983	chr13	65866073	65915107	49034	3	12	25.10606061	5.02953978	0.781539415	0.096587823	+	105370243	grey	-0.09133506	FALSE	LOC105370243
Region_4984	chr13	66302834	67230445	927611	64	493	541.9545455	22.55006861	0.86012148	0.015284017	-	5101	grey	-0.037106853	FALSE	PCDH9
Region_4985	chr13	66825169	66915031	89862	4	26	42.92424242	6.617716562	0.875212901	0.050570049	+	100874064	grey	0.019063677	FALSE	PCDH9-AS2
Region_4986	chr13	67326177	67379994	53817	4	29	43.68181818	6.86161921	0.726451379	0.094813564	+	100874145	grey	0.100856864	FALSE	LINC00364
Region_4987	chr1	67577624	67787804	210180	9	55	75.0606060	9.88530967	0.91688216	0.03750206	-	10537024	grey	-0.03002871	FALSE	LOC105370248

	3						6	2	9			8		9		
Region_4988	chr13	69221866	69322190	100324	5	35	49.13636364	6.651515417	0.859800135	0.056090549	+	103689913	grey	0.060029821	FALSE	LINC00383
Region_4989	chr13	69700594	70108493	407899	27	169	206.1515152	15.37554744	0.812939956	0.029494586	-	57626	grey	0.16140099	FALSE	KLHL1
Region_4990	chr13	70107213	70171738	64525	5	22	35.03030303	5.47151982	0.423977836	0.070728564	+	6315	grey	0.285303665	FALSE	ATXN8OS
Region_4991	chr13	71015042	71168417	153375	9	68	84.78787879	9.135754192	0.820128099	0.047736459	+	100885781	grey	-0.003162894	FALSE	LINC00348
Region_4992	chr13	71437966	71867204	429238	31	219	264.0909091	15.82862769	0.831391852	0.019009001	-	1602	grey	-0.304506369	FALSE	DACH1
Region_4993	chr13	72782133	73016461	234328	22	147	177.8333333	13.88589027	0.906175786	0.023277866	+	10464	grey	0.116379926	FALSE	PIBF1
Region_4994	chr13	73533759	73661891	128132	13	75	100.2424242	9.116444324	0.906385411	0.031847913	-	100874156	grey	0.120671165	FALSE	LINC00393
Region_4995	chr13	73536426	73661867	125441	13	75	100.2424242	9.116444324	0.906385411	0.031847913	-	107984024	grey	0.120671165	FALSE	
Region_4996	chr13	73564244	73588070	23826	3	11	21.27272727	4.160419559	0.960457696	0.042268921	+	100874155	grey	-0.094853247	FALSE	LINC00392
Region_4997	chr13	73686089	74133929	447840	45	346	387.2424242	19.77649001	0.918990936	0.015555941	-	11278	grey	-0.182402147	FALSE	KLF12
Region_4998	chr13	74231401	74259976	28575	4	25	40.28787879	6.253036558	0.875869632	0.070076636	+	100507612	grey	-0.009437612	FALSE	LINC00402
Region_4999	chr13	74288070	74408810	120740	15	104	126.8181818	11.08138993	0.878757029	0.045293666	+	105370259	grey	0.134725402	FALSE	LOC105370259

Region_5000	chr13	74419158	74444735	25577	5	31	42.72727273	5.584443928	0.863302248	0.059315628	+	100874151	grey	0.166231184	FALSE	LINC00381
Region_5001	chr13	74434538	74557099	122561	12	68	90.28787879	8.637601433	0.786003854	0.06566962	+	338864	grey	-0.06754465	FALSE	LINC00347
Region_5002	chr13	75283503	75482169	198666	26	189	212	11.93056837	0.779290139	0.023292682	-	9882	grey	-0.122291327	FALSE	TBC1D4
Region_5003	chr13	75549773	75859870	310097	26	179	210.8484848	14.93394937	0.864098179	0.023659086	+	4008	grey	0.33936125	FALSE	LMO7
Region_5004	chr13	76552807	76846524	293717	15	81	108.7424242	9.982000117	0.883011512	0.032299137	-	105370264	grey	-0.163719063	FALSE	LOC105370264
Region_5005	chr13	76990660	77129589	138929	11	73	92.34848485	8.970880112	0.870117679	0.022242258	+	1203	grey	0.057012737	FALSE	CLN5
Region_5006	chr13	76992598	77027195	34597	3	20	29.74242424	5.02703646	0.654397045	0.022996858	-	26224	grey	-0.169365637	FALSE	FBXL3
Region_5007	chr13	77008602	77129717	121115	8	47	59.39393939	7.611486841	0.971246186	0.028747176	+	100874212	grey	0.103636343	FALSE	MYCBP2-AS1
Region_5008	chr13	77042474	77327094	284620	25	150	183.5606061	13.38900673	0.85149069	0.019901617	-	23077	grey	0.189634514	FALSE	MYCBP2
Region_5009	chr13	77535674	77645263	109589	11	64	81.62121212	8.834835163	0.773137272	0.046535901	+	8796	grey	-0.119351837	FALSE	SCEL
Region_5010	chr13	77697687	77764242	66555	7	45	64.96969697	7.61975105	0.834407769	0.030956036	+	122060	grey	-0.282772272	FALSE	SLAIN1
Region_5011	chr13	77895481	77975529	80048	8	49	66.60606061	7.869862256	0.948307519	0.040438447	-	1910	grey	0.111296339	FALSE	EDNRB
Region_5012	chr1	77919689	78617328	697639	53	368	421.772727	25.7065064	0.76772930	0.02183406	+	10087422	grey	-0.02941754	FALSE	OBI1-AS1

	3						3	8	5	1		2		7		
Region_5013	chr13	77997909	78053604	55695	6	32	50.43939394	6.266219049	0.912815031	0.051445361	+	100874175	grey	-0.077452193	FALSE	LINC00446
Region_5014	chr13	78598362	78603552	5190	3	13	26.66666667	5.754819608	0.028043131	0.037756637	-	5457	grey	0.148716622	FALSE	POU4F1
Region_5015	chr13	78614289	78659155	44866	3	16	25.21212121	4.411576211	0.956376529	0.043096799	-	79596	grey	-0.157123684	FALSE	OBI1
Region_5016	chr13	79311824	79406477	94653	10	51	69.03030303	8.019149575	0.827920967	0.044496044	-	64062	grey	0.173455398	FALSE	RBM26
Region_5017	chr13	79481155	79556077	74922	7	36	53.93939394	6.333983527	0.853057895	0.059270274	+	54602	grey	-0.032371489	FALSE	NDFIP2
Region_5018	chr13	79872586	79918042	45456	6	34	54.87878788	8.628245479	0.873839454	0.054066921	+	101927195	grey	0.217063901	FALSE	LINC00382
Region_5019	chr13	80335976	80341126	5150	3	22	30.66666667	4.043354789	0.101376517	0.055176336	-	10253	grey	0.04525143	FALSE	SPRY2
Region_5020	chr13	80697177	81017737	320560	28	185	218.4090909	15.94121632	0.92354779	0.023997108	-	105370278	grey	0.190416581	FALSE	LOC105370278
Region_5021	chr13	81250438	81302407	51969	4	19	33.45454545	5.762429558	0.746916625	0.113745286	+	105370281	grey	0.125905838	FALSE	LOC105370281
Region_5022	chr13	84086802	84563236	476434	18	116	142.5757576	11.48652804	0.83394466	0.039236165	+	100874128	grey	0.074841436	FALSE	LINC00333
Region_5023	chr13	84867514	84902424	34910	6	28	43.77272727	7.345946982	0.872411745	0.055900541	-	105370290	grey	-0.08054698	FALSE	LOC105370290
Region_5024	chr13	85065087	85148121	83034	4	17	29.27272727	5.845564932	0.926881368	0.05954113	-	104355140	grey	-0.056415696	FALSE	LINC00375

Region_5025	chr13	85363601	85544570	180969	16	105	128.8333333	11.38363648	0.898836035	0.031153953	+	100874137	grey	-0.152653625	FALSE	LINC00351
Region_5026	chr13	86402982	86462734	59752	5	28	44.54545455	6.214577944	0.870821762	0.061401654	-	105370299	grey	0.538754345	FALSE	LOC105370299
Region_5027	chr13	87427200	87671640	244440	14	94	124.469697	10.52647899	0.827735307	0.03960058	-	642345	grey	-0.22946102	FALSE	MIR4500HG
Region_5028	chr13	88142867	88236082	93215	5	26	39.74242424	6.42665269	0.829647772	0.068970476	+	105370306	grey	-0.183487279	FALSE	LINC00373
Region_5029	chr13	88494789	88607814	113025	8	49	64.21212121	7.284367142	0.821374408	0.056117248	+	100874168	grey	0.11911001	FALSE	LINC00433
Region_5030	chr13	89181324	89280240	98916	5	29	39.43939394	5.508395796	0.78553264	0.085927382	+	100874172	grey	-0.063250197	FALSE	LINC00440
Region_5031	chr13	89402398	89818453	416055	23	137	168.1515152	14.43525857	0.846952006	0.03867655	+	107984621	grey	0.086643601	FALSE	LOC107984621
Region_5032	chr13	91347820	91354579	6759	4	15	23.51515152	4.239892685	0.234248107	0.029081678	+	407975	grey	-0.002210218	FALSE	MIR17HG
Region_5033	chr13	91398621	92867237	1468616	132	1034	1103.5	36.0167696	0.840250398	0.014255556	+	2262	grey	0.229154986	FALSE	GPC5
Region_5034	chr13	92610646	92677725	67079	6	46	60.45454545	7.901668408	0.858082215	0.055793509	-	105370315	grey	-0.213358291	FALSE	LOC105370315
Region_5035	chr13	93032972	93057926	24954	6	33	46.59090909	6.292797526	0.761085447	0.096496181	+	105370317	grey	0.155461074	FALSE	LOC105370317
Region_5036	chr13	93226807	94408020	1181213	101	773	867.6818182	32.26818347	0.845823439	0.017549896	+	10082	grey	-0.105636925	FALSE	GPC6
Region_5037	chr1	94154193	94187991	33798	3	26	35.8636363	5.53001979	0.83750327	0.07748910	-	10087397	grey	0.027788223	FALSE	GPC6-AS1

	3						6		8	5		2				
Region_5038	chr13	94436811	94479682	42871	6	35	50.51515152	7.457040915	0.920857881	0.045147137	-	1638	grey	0.016808272	FALSE	DCT
Region_5039	chr13	94601857	94634661	32804	6	33	45.3030303	6.923693316	0.486035426	0.025302347	+	160897	grey	-0.400165319	FALSE	GPR180
Region_5040	chr13	94698725	94708489	9764	3	14	26.56060606	4.713740269	0.059604971	0.048833141	-	101927248	grey	-0.001987322	FALSE	LINC00391
Region_5041	chr13	94703454	94803430	99976	12	84	102.1515152	9.028574168	0.64584837	0.033977783	+	101927284	grey	-0.060331549	FALSE	LOC101927284
Region_5042	chr13	95019835	95301475	281640	64	465	527.3484848	25.65180601	0.890873504	0.014096431	-	10257	grey	-0.024173548	FALSE	ABCC4
Region_5043	chr13	95433604	95579759	146155	27	179	205.9848485	15.65738206	0.834106597	0.037154964	+	9071	grey	0.23120642	FALSE	CLDN10
Region_5044	chr13	95479444	95533910	54466	13	80	99.74242424	9.741758815	0.731821957	0.06213874	-	100874194	grey	0.217253595	FALSE	CLDN10-AS1
Region_5045	chr13	95578202	95644706	66504	19	110	143.8030303	12.12907466	0.788177869	0.02772964	-	22873	grey	-0.276370203	FALSE	DZIP1
Region_5046	chr13	95648771	95676973	28202	6	36	52.07575758	6.296056412	0.784890555	0.034846138	-	100289274	grey	-0.008505716	FALSE	DNAJC3-DT
Region_5047	chr13	95677139	95794988	117849	19	127	161.2575758	14.2346388	0.917226834	0.026700201	+	5611	grey	0.165596569	FALSE	DNAJC3
Region_5048	chr13	95801580	96053482	251902	25	173	197.9848485	14.25319659	0.868352071	0.028581532	-	55757	grey	-0.201449343	FALSE	UGGT2
Region_5049	chr13	96090107	96839562	749455	84	671	736.8030303	28.99507634	0.791365655	0.015227391	+	266722	grey	-0.139837837	FALSE	HS6ST3

Region_5050	chr13	96941281	96984172	42891	5	25	36.84848485	5.463845995	0.644021268	0.05590544	-	100887754	grey	-0.078480408	FALSE	LINC00359
Region_5051	chr13	97221434	97394120	172686	28	177	227.1363636	17.51824224	0.899616553	0.024084003	+	10150	grey	-0.246954954	FALSE	MBNL2
Region_5052	chr13	97953658	98024296	70638	22	129	163.7575758	13.05616342	0.801241934	0.022473694	+	3843	grey	0.312411742	FALSE	IPO5
Region_5053	chr13	98142562	98455176	312614	73	524	587.0757576	28.95052044	0.931557256	0.014527294	+	10160	grey	-0.312950676	FALSE	FARP1
Region_5054	chr13	98328191	98377918	49727	15	101	132.030303	12.37238824	0.954074928	0.021811969	+	105370327	grey	-0.143457214	FALSE	LOC105370327
Region_5055	chr13	98445185	98577940	132755	31	203	239.1363636	18.38886483	0.814883083	0.027396566	-	8428	grey	0.242207651	FALSE	STK24
Region_5056	chr13	98683801	98752672	68871	28	210	244.3181818	16.19511711	0.874342937	0.025449674	-	6564	grey	0.087833226	FALSE	SLC15A1
Region_5057	chr13	98793429	99086625	293196	82	612	665.1666667	24.2281358	0.822254564	0.01507564	-	23348	grey	0.184121359	FALSE	DOCK9
Region_5058	chr13	99181223	99200757	19534	12	66	85.96969697	10.03527578	0.861506258	0.047333571	-	100289373	grey	-0.086270806	FALSE	UBAC2-AS1
Region_5059	chr13	99200774	99386504	185730	53	379	444.8787879	21.70325403	0.715805405	0.019297912	+	337867	grey	0.142898107	FALSE	UBAC2
Region_5060	chr13	99486962	99499306	12344	3	17	26.40909091	4.388717257	0.57704505	0.061744246	-	102725509	grey	0.05880818	FALSE	LINC01232
Region_5061	chr13	99498956	99564048	65092	16	126	148.530303	10.69179369	0.69794499	0.026823231	+	9375	grey	0.281373752	FALSE	TM9SF2
Region_5062	chr1	99606669	99909459	302790	77	567	624.727272	26.6224575	0.86954260	0.01307971	+	171425	grey	-0.02958854	FALSE	CLYBL

	3						7	3	4	5				1		
Region_5063	chr13	99841879	99957167	115288	25	177	205.4090909	13.54874649	0.744910732	0.026196539	-	101927437	grey	-0.009164236	FALSE	CLYBL-AS3
Region_5064	chr13	99962964	99971767	8803	5	19	28.28787879	5.694167465	0.048988682	0.078652862	-	85416	grey	0.14647151	FALSE	ZIC5
Region_5065	chr13	99981784	99986765	4981	3	16	29.42424242	5.288770705	0.023180273	0.026204589	+	7546	grey	-0.044931621	FALSE	ZIC2
Region_5066	chr13	100053074	100088972	35898	12	60	91.42424242	10.67860637	0.874228259	0.028796381	-	105370333	grey	0.193724956	FALSE	PCCA-DT
Region_5067	chr13	100089015	100530437	441422	120	916	994.1363636	33.0835243	0.883716156	0.009168402	+	5095	grey	0.021874496	FALSE	PCCA
Region_5068	chr13	100338982	100364959	25977	5	25	37.46969697	5.657451715	0.862163796	0.052571604	-	105370335	grey	0.086810792	FALSE	LOC105370335
Region_5069	chr13	100464440	100480285	15845	4	22	33.12121212	5.498293865	0.869821419	0.052005477	-	100885777	grey	-0.12996693	FALSE	PCCA-AS1
Region_5070	chr13	100530164	100589528	59364	23	174	201.3636364	14.18193473	0.862851846	0.02015062	-	87769	grey	0.029276418	FALSE	GGACT
Region_5071	chr13	100603625	100675093	71468	63	470	521.6666667	25.8613661	0.818893543	0.019530844	-	84899	grey	0.084780666	FALSE	TMTC4
Region_5072	chr13	100708325	101059286	350961	50	368	405.8636364	18.41143989	0.836745958	0.021968319	+	100885778	grey	0.187860379	FALSE	NALCN-AS1
Region_5073	chr13	101053776	101416508	362732	95	706	763.7121212	33.72456998	0.813165854	0.016136006	-	259232	grey	0.130295834	FALSE	NALCN
Region_5074	chr13	101452593	101720856	268263	39	286	327.7575758	17.39589749	0.887537685	0.022764122	+	9358	grey	-0.171148525	FALSE	ITGBL1

Region_5075	chr13	101710804	102402457	691653	74	565	629.5454545	26.26092846	0.78691343	0.019304006	-	2259	grey	-0.079189332	FALSE	FGF14
Region_5076	chr13	102292327	102394519	102192	6	46	64.28787879	7.564624454	0.843752259	0.049382338	-	283480	grey	0.509430637	FALSE	FGF14-IT1
Region_5077	chr13	102596958	102679958	83000	13	67	89.83333333	10.20495104	0.8527609	0.032741399	+	7174	grey	-0.222146602	FALSE	TPP2
Region_5078	chr13	102765888	102773811	7923	7	46	63.09090909	8.502899547	0.008801453	0.012497005	-	93081	grey	0.051422939	FALSE	TEX30
Region_5079	chr13	102784281	102798976	14695	3	16	25.77272727	4.495452247	0.649393319	0.032719489	-	79070	grey	0.216119993	FALSE	POGLUT2
Region_5080	chr13	102799110	102875994	76884	12	71	94.48484848	9.39761414	0.846308563	0.030799572	+	100533467	grey	0.013403949	FALSE	BIVM-ERCC5
Region_5081	chr13	102799119	102841533	42414	9	48	70.63636364	8.03192929	0.821546297	0.03484461	+	54841	grey	0.043146823	FALSE	BIVM
Region_5082	chr13	102845831	102875995	30164	3	17	23.84848485	4.286804168	0.920371034	0.059240675	+	2073	grey	-0.053267558	FALSE	ERCC5
Region_5083	chr13	103043998	103066417	22419	8	57	75.83333333	8.34189304	0.818217443	0.033688281	-	6555	grey	0.119489429	FALSE	SLC10A2
Region_5084	chr13	103343660	103396450	52790	8	52	65.48484848	6.254210703	0.847031336	0.058253437	+	105370339	grey	0.077255949	FALSE	LOC105370339
Region_5085	chr13	103420825	103428298	7473	3	13	23.31818182	5.39560816	0.869937823	0.08127082	+	104326190	grey	-0.200571365	FALSE	LINC01309
Region_5086	chr13	105459055	105505681	46626	5	29	44.43939394	6.104547581	0.891797508	0.058451883	-	282706	grey	0.099778091	FALSE	DAOA-AS1
Region_5087	chr1	10570269	10579753	94840	9	52	74.5757575	8.93405000	0.74156022	0.04528763	+	144920	grey	-0.04364907	FALSE	LINC00343

	3	9	9				8	1	9	9				5		
Region_5088	chr13	106232131	106371082	138951	9	62	82.1969697	8.625935306	0.87585419	0.03918086	-	107984626	grey	0.203283944	FALSE	LOC107984626
Region_5089	chr13	106489745	106535662	45917	10	77	95.74242424	9.79530595	0.621548107	0.030966701	-	1948	grey	0.278863615	FALSE	EFNB2
Region_5090	chr13	106541673	106568137	26464	6	31	42.56060606	5.828652801	0.463157085	0.063138267	-	55082	grey	-0.104680834	FALSE	ARGLU1
Region_5091	chr13	106568208	106716679	148471	21	168	195.1666667	14.60725145	0.874507944	0.028726794	+	105370348	grey	0.097815055	FALSE	
Region_5092	chr13	106568261	106631659	63398	9	79	93.93939394	9.408472103	0.854147852	0.048041586	+	283483	grey	-0.039467773	FALSE	ARGLU1-DT
Region_5093	chr13	107163510	107867496	703986	129	1000	1124.515152	43.71700687	0.819883943	0.012923554	-	728215	grey	0.056996914	FALSE	NALF1
Region_5094	chr13	107788342	107835451	47109	11	63	83.6969697	8.662864506	0.875895828	0.039702224	-	100874375	grey	0.117732763	FALSE	NALF1-IT1
Region_5095	chr13	108335354	108448316	112962	16	102	130.0606061	11.84369847	0.93286054	0.028079197	+	105370355	grey	0.04997097	FALSE	LOC105370355
Region_5096	chr13	108596152	109208005	611853	75	521	593.6212121	25.91213862	0.841706788	0.018242103	+	23026	grey	-0.185648273	FALSE	MYO16
Region_5097	chr13	109163902	109201483	37581	8	44	57.57575758	8.726723953	0.944144729	0.041273243	-	100885782	grey	0.16054967	FALSE	MYO16-AS1
Region_5098	chr13	109478739	109731700	252961	27	219	248.4090909	14.16167881	0.870877953	0.021726005	-	107984602	grey	0.470989676	FALSE	LOC107984602
Region_5099	chr13	109752695	109786583	33888	15	84	103.6515152	10.05445546	0.756091568	0.02756993	-	8660	grey	-0.23175545	FALSE	IRS2

Region_5100	chr13	109785831	109808252	22421	8	42	65.37878788	8.424807967	0.672506268	0.047770811	+	105370360	grey	-0.039228919	FALSE	LOC105370360
Region_5101	chr13	109988097	110129136	141039	38	293	341.2272727	20.21255928	0.871801212	0.023600409	-	101927646	grey	0.037371044	FALSE	LINC03082
Region_5102	chr13	110148963	110307157	158194	40	304	342.469697	20.21135987	0.750632261	0.023504078	-	1282	grey	0.25777337	FALSE	COL4A1
Region_5103	chr13	110305812	110513209	207397	123	857	977.0454545	46.38979723	0.770091656	0.015534739	+	1284	grey	-0.104706226	FALSE	COL4A2
Region_5104	chr13	110456396	110463287	6891	6	32	46.1969697	7.023520259	0.879945276	0.08101509	-	100129836	grey	0.037521027	FALSE	COL4A2-AS2
Region_5105	chr13	110523066	110561722	38656	11	85	108.2575758	11.25824225	0.727847966	0.033542484	-	55647	grey	0.071246494	FALSE	RAB20
Region_5106	chr13	110615545	110639996	24451	15	113	134.030303	11.96529558	0.882388006	0.024602979	+	55739	grey	-0.240693965	FALSE	NAXD
Region_5107	chr13	110641412	110713603	72191	27	150	189.2424242	15.34030449	0.701294105	0.022053977	-	79587	grey	-0.104232866	FALSE	CARS2
Region_5108	chr13	110712736	110723339	10603	11	43	63.31818182	10.81332382	0.004791957	0.009936844	+	3621	grey	0.044660466	FALSE	ING1
Region_5109	chr13	110878540	110915069	36529	20	136	159.1060606	12.99719353	0.753129781	0.016314393	-	55608	grey	0.133782674	FALSE	ANKRD10
Region_5110	chr13	110945478	110971603	26125	13	81	105.2575758	10.15769599	0.909196407	0.025594192	-	105370364	grey	-0.166260408	FALSE	LOC105370364
Region_5111	chr13	110965704	110990579	24875	8	62	79.98484848	9.370152982	0.84341995	0.043128391	+	104355135	grey	0.003320021	FALSE	LINC00431
Region_5112	chr1	11109583	11110309	7259	4	13	23.2121212	4.82785237	0.71688344	0.07392668	+	10192780	grey	0.021679812	FALSE	LINC00368

	3	8	7				1		3	2		2				
Region_5113	chr13	111113812	111115678	1866	5	15	28.72727273	6.299073081	0.009713313	0.021532025	-	100874238	grey	-0.126660977	FALSE	ARHGEF7-AS2
Region_5114	chr13	111114559	111305737	191178	128	1051	1139.212121	42.19606805	0.873547414	0.008867479	+	8874	grey	-0.129727497	FALSE	ARHGEF7
Region_5115	chr13	111316184	111344249	28065	20	123	153	14.90379404	0.899771305	0.031977382	+	121793	grey	-0.14418795	FALSE	TEX29
Region_5116	chr13	111588291	111672608	84317	28	181	214.0909091	16.93037986	0.848196496	0.029793001	+	102724489	grey	-0.170553462	FALSE	LOC102724489
Region_5117	chr13	111596009	111642079	46070	18	113	142.530303	13.83331508	0.863602866	0.032735346	+	105370369	grey	-0.276999054	FALSE	LINC02337
Region_5118	chr13	111865136	111901264	36128	21	141	175.7424242	16.38227053	0.688970651	0.030247407	+	101928616	grey	-0.04759882	FALSE	LINC00354
Region_5119	chr13	112056845	112108015	51170	41	284	324.0151515	19.15643489	0.162367921	0.022080718	+	100505996	grey	0.064011	FALSE	SOX1-OT
Region_5120	chr13	112067149	112071706	4557	6	35	53.07575758	7.271251307	0.117861423	0.046668959	+	6656	grey	0.254077992	FALSE	SOX1
Region_5121	chr13	112647044	112684497	37453	26	190	219.7575758	12.01545237	0.919443733	0.024849675	+	400165	grey	-0.119251761	FALSE	ATP11AUN
Region_5122	chr13	112690038	112887168	197130	244	1741	1946.863636	89.45319126	0.854219254	0.007985482	+	23250	grey	0.193771354	FALSE	ATP11A
Region_5123	chr13	112745449	112754693	9244	5	24	38.06060606	6.314522426	0.846656678	0.064261251	-	100874205	grey	-0.14028405	FALSE	ATP11A-AS1
Region_5124	chr13	112894378	113099742	205364	135	911	1030.893939	51.05024035	0.820315567	0.012158109	+	23263	grey	0.042776893	FALSE	MCF2L

Region_5125	chr13	113105788	113120685	14897	15	73	93.87878788	12.00963094	0.460467347	0.038725869	+	2155	grey	0.112414544	FALSE	F7
Region_5126	chr13	113122799	113149529	26730	10	48	67.87878788	9.429112286	0.90399975	0.047712756	+	2159	grey	0.215614798	FALSE	F10
Region_5127	chr13	113149432	113154002	4570	7	26	37.10606061	6.52834951	0.573781791	0.052617393	-	102724474	grey	-0.0238131	FALSE	LOC102724474
Region_5128	chr13	113158648	113172386	13738	6	22	40.15151515	6.766984158	0.883752048	0.059897497	+	8858	grey	-0.254969673	FALSE	PROZ
Region_5129	chr13	113177536	113208715	31179	17	120	151.5909091	13.48158547	0.70753725	0.023751201	-	55795	grey	0.151926365	FALSE	PCID2
Region_5130	chr13	113208193	113267108	58915	46	327	373.2878788	20.59556722	0.810634638	0.011388755	+	8451	grey	-0.068507828	FALSE	CUL4A
Region_5131	chr13	113297239	113323672	26433	22	132	156.8636364	11.36917068	0.825652463	0.02273756	+	3916	grey	-0.175110836	FALSE	LAMP1
Region_5132	chr13	113324163	113364148	39985	45	288	339.2424242	18.64160822	0.895620946	0.015656475	-	79774	grey	-0.021043676	FALSE	GRTP1
Region_5133	chr13	113351645	113361868	10223	10	61	77.37878788	8.470337658	0.867315726	0.046107337	+	100874068	grey	0.124836585	FALSE	GRTP1-AS1
Region_5134	chr13	113399611	113453488	53877	78	535	608.6060606	32.61612569	0.894275329	0.01492478	-	113622	grey	-0.38856308	FALSE	ADPRHL1
Region_5135	chr13	113455819	113490951	35132	35	259	313.969697	18.4657435	0.820946085	0.017770471	-	55208	grey	-0.047215884	FALSE	DCUN1D2
Region_5136	chr13	113491021	113554590	63569	91	647	724.6969697	35.56747421	0.72601117	0.030022573	+	55002	grey	-0.185222206	FALSE	TMCO3
Region_5137	chr1	11358472	11364147	56752	81	598	658.893939	36.4801858	0.90062908	0.00940674	+	7027	grey	0.010914563	FALSE	TFDP1

	3	1	3				4	9	4	9						
Region_5138	chr13	113648804	113658198	9394	32	205	246.1515152	19.72912133	0.916331726	0.024095199	-	496	grey	0.189227371	FALSE	ATP4B
Region_5139	chr13	113759226	113816995	57769	81	485	552.4848485	32.55917331	0.86404972	0.019930453	+	348013	grey	0.023841433	FALSE	TMEM255B
Region_5140	chr13	113815630	113845744	30114	26	133	174.6060606	13.64706651	0.86649381	0.021702224	+	650669	grey	-0.091310071	FALSE	GAS6-AS1
Region_5141	chr13	113820549	113864076	43527	64	313	397.1818182	35.01951504	0.38387549	0.01587611	-	2621	grey	0.03602226	FALSE	GAS6
Region_5142	chr13	113864112	113866834	2722	6	20	51.98484848	13.64437605	0.011565211	0.015373906	+	100506394	grey	0.067991025	FALSE	GAS6-DT
Region_5143	chr13	113878489	113883651	5162	4	15	26.15151515	5.257207285	0.97363211	0.029519339	-	100874178	grey	-0.021798121	FALSE	LINC00454
Region_5144	chr13	113888306	113923512	35206	17	110	149.2575758	13.94197934	0.869231843	0.026623262	+	643365	grey	0.263336475	FALSE	LINC00452
Region_5145	chr13	113923052	113928991	5939	4	20	37.39393939	7.723846766	0.597658438	0.082349213	-	100861555	grey	-0.156049796	FALSE	SWINGN
Region_5146	chr13	114154691	114223085	68394	58	422	484.0757576	26.93028641	0.842189698	0.020195136	+	101929355	grey	-0.099302881	FALSE	CFAP97D2
Region_5147	chr13	114234887	114272723	37836	28	173	224.8636364	17.75550737	0.908590253	0.017823649	+	8881	grey	0.137354933	FALSE	CDC16
Region_5148	chr13	114281601	114305817	24216	32	210	251.8484848	16.81688019	0.849175407	0.015885323	+	65110	grey	-0.064536534	FALSE	UPF3A
Region_5149	chr13	114314482	114337626	23144	9	44	62.68181818	8.34848506	0.61159955	0.025597517	+	283489	grey	-0.172124479	FALSE	CHAMP1

Region_5150	chr14	19062316	19131167	68851	4	17	34.07575758	12.60928914	0.49560982	0.082817379	+	503638	grey	-0.081963707	FALSE	DUXAP9
Region_5151	chr14	19268853	19337688	68835	4	17	57.98484848	21.96885377	0.727254938	0.088603127	-	115801414	grey	-0.188369639	FALSE	LINC01297-DUXAP10-NBEAP6
Region_5152	chr14	19284653	19337730	53077	4	17	57.98484848	21.96885377	0.727254938	0.088603127	-	503639	grey	-0.188369639	FALSE	DUXAP10
Region_5153	chr14	19719015	19830253	111238	14	95	159.7272727	47.77818796	0.761011402	0.036201408	+	390429	grey	-0.049341481	FALSE	OR4N2
Region_5154	chr14	20425852	20436166	10314	3	17	27.98484848	5.106534047	0.965384383	0.035053762	-	123103	grey	0.013348085	FALSE	KLHL33
Region_5155	chr14	20684177	20698971	14794	4	18	27.98484848	5.166437321	0.392133551	0.092224203	+	283	grey	-0.088724416	FALSE	ANG
Region_5156	chr14	20684560	20701216	16656	3	11	21.39393939	4.593904952	0.522844735	0.122965604	+	6038	grey	-0.088724416	FALSE	RNASE4
Region_5157	chr14	20870278	20936255	65977	19	126	149.6666667	11.88319218	0.690174008	0.02665563	-	100507513	grey	-0.016486877	FALSE	LOC100507513
Region_5158	chr14	21016763	21070872	54109	19	122	143.530303	11.99130415	0.554098338	0.025247343	-	57447	grey	-0.097643218	FALSE	NDRG2
Region_5159	chr14	21024109	21036276	12167	3	17	31.98484848	5.842792863	0.84541196	0.059875972	+	122664	grey	0.017887607	FALSE	TPPP2
Region_5160	chr14	21070273	21090248	19975	14	77	102.1969697	12.31911547	0.764272597	0.025325349	+	55701	grey	0.044783867	FALSE	ARHGEF40
Region_5161	chr14	21090077	21104722	14645	18	82	114.3484848	13.56408428	0.81655277	0.032492967	-	51222	grey	0.063245869	FALSE	ZNF219
Region_5162	chr1	21209136	21269494	60358	23	145	175.712121	13.3506152	0.72192343	0.02498279	-	3183	grey	0.030538925	FALSE	HNRNPC

	4						2	6	2	5						
Region_5163	chr14	21280083	21351301	71218	18	110	133.6515152	11.41994534	0.933218946	0.030777853	+	57096	grey	0.07920874	FALSE	RPGRIP1
Region_5164	chr14	21351476	21384019	32543	6	32	50.78787879	8.232235235	0.924837215	0.032540475	-	11198	grey	-0.011524277	FALSE	SUPT16H
Region_5165	chr14	21385194	21456126	70932	20	146	172.9393939	13.1650806	0.816138842	0.021895174	-	57680	grey	-0.233678081	FALSE	CHD8
Region_5166	chr14	21459020	21476960	17940	7	33	49.3030303	6.843232922	0.818243417	0.031121787	-	84932	grey	0.082676605	FALSE	RAB2B
Region_5167	chr14	21476597	21499175	22578	12	59	83.57575758	10.42638754	0.733723079	0.021028878	+	9878	grey	-0.024749627	FALSE	TOX4
Region_5168	chr14	21521080	21537216	16136	9	44	61.16666667	9.72322095	0.532090339	0.056834595	-	6297	grey	0.443206115	FALSE	SALL2
Region_5169	chr14	21568520	21580076	11556	3	10	19.81818182	3.790307846	0.911464441	0.079640315	-	26533	grey	0.453951036	FALSE	OR10G3
Region_5170	chr14	21653835	21667642	13807	7	62	80.3030303	9.352860027	0.822824996	0.036696761	+	26686	grey	0.135014109	FALSE	OR4E2
Region_5171	chr14	22197446	22484462	287016	27	149	210.9090909	21.88974278	0.750328474	0.028902369	-	105370401	grey	-0.074971853	FALSE	TRD-AS1
Region_5172	chr14	22564907	22589224	24317	3	10	18.93939394	4.374326455	0.938352697	0.082811218	-	1603	grey	0.354880088	FALSE	DAD1
Region_5173	chr14	22701476	22766562	65086	9	42	61	7.597570462	0.93374684	0.036280206	-	105370404	grey	0.06543062	FALSE	OXA1L-DT
Region_5174	chr14	22773222	22829820	56598	10	49	70.42424242	9.480442388	0.819934043	0.075025145	-	9056	grey	0.143696358	FALSE	SLC7A7

Region_5175	chr14	22871740	22881713	9973	3	10	18.8030303	4.188701642	0.002609428	0.014559688	+	26020	grey	-0.118973196	FALSE	LRP10
Region_5176	chr14	22893204	22919182	25978	5	27	42.22727273	6.126350286	0.951735146	0.039345735	-	55147	grey	-0.001200635	FALSE	RBM23
Region_5177	chr14	22929607	22956436	26829	7	45	57.48484848	7.49818937	0.876512747	0.038171115	+	101926933	grey	0.127944713	FALSE	PRMT5-DT
Region_5178	chr14	22946228	22982258	36030	10	48	71.21212121	8.829173934	0.77689292	0.045509065	-	54930	grey	0.154158059	FALSE	HAUS4
Region_5179	chr14	22985894	23010166	24272	6	38	55.12121212	7.037296578	0.451405575	0.044239179	-	60686	grey	0.481288453	FALSE	C14orf93
Region_5180	chr14	23016543	23035230	18687	8	36	53.06060606	6.954530981	0.870675248	0.033317593	-	5693	grey	0.082643551	FALSE	PSMB5
Region_5181	chr14	23047062	23057538	10476	5	22	34.25757576	6.135171256	0.939431625	0.048268206	-	64403	grey	0.119009115	FALSE	CDH24
Region_5182	chr14	23057702	23058661	959	5	16	30.77272727	6.943276669	0.001768989	0.006312832	+	105370705	grey	0.02044183	FALSE	LOC105370705
Region_5183	chr14	23058564	23095614	37050	14	76	97.63636364	8.818924343	0.769038531	0.033771341	-	22985	grey	-0.209441288	FALSE	ACIN1
Region_5184	chr14	23095505	23100456	4951	6	23	41.18181818	9.346178307	0.488126267	0.050619053	+	55017	grey	0.177195051	FALSE	C14orf119
Region_5185	chr14	23125295	23183674	58379	8	47	63.03030303	7.724027839	0.702692379	0.032001762	-	23428	grey	-0.121035535	FALSE	SLC7A8
Region_5186	chr14	23185316	23273477	88161	27	165	202.3030303	13.37847835	0.912554282	0.019174797	+	100507650	grey	0.024930669	FALSE	RNF212B
Region_5187	chr1	23272422	23299447	27025	8	55	71.9545454	8.44061940	0.74745440	0.04174077	-	57594	grey	-0.03627561	FALSE	HOMEZ

	4						5	5	7	9				8		
Region_5188	chr14	23295652	23302859	7207	8	44	58.40909091	8.392262504	0.315164029	0.028234213	-	90673	grey	-0.114961128	FALSE	PPP1R3E
Region_5189	chr14	23298790	23326175	27385	25	145	179.9393939	16.02198373	0.364402588	0.023057827	+	100529063	grey	-0.063568596	FALSE	BCL2L2-PABPN1
Region_5190	chr14	23302296	23311751	9455	9	44	63.81818182	9.634727398	0.34116708	0.059742456	+	599	grey	-0.096006395	FALSE	BCL2L2
Region_5191	chr14	23321457	23326163	4706	4	10	20.75757576	5.326452438	0.494410924	0.083753526	+	8106	grey	0.216615429	FALSE	PABPN1
Region_5192	chr14	23346314	23352912	6598	5	18	29.39393939	5.264916649	0.832567548	0.057050686	-	51310	grey	0.188061849	FALSE	SLC22A17
Region_5193	chr14	23356403	23365752	9349	5	25	40.77272727	6.569850612	0.517343477	0.09484843	-	10278	grey	-0.165733451	FALSE	EFS
Region_5194	chr14	23376773	23379772	2999	3	10	21.92424242	4.651916416	0.875032824	0.075959302	+	116173	grey	-0.126131878	FALSE	CMTM5
Region_5195	chr14	23381982	23408273	26291	14	89	114	10.24019231	0.869810542	0.036445877	-	4624	grey	0.107337907	FALSE	MYH6
Region_5196	chr14	23412740	23435660	22920	18	88	129.8636364	12.56232017	0.915685676	0.031356075	-	4625	grey	-0.179196182	FALSE	MYH7
Region_5197	chr14	23469679	23509862	40183	19	122	161.5909091	12.10718448	0.867300846	0.018172307	+	25983	grey	-0.047133885	FALSE	NGDN
Region_5198	chr14	23511760	23560778	49018	26	148	176.7878788	12.97039843	0.760823414	0.024544863	+	79178	grey	-0.082922573	FALSE	THTPA
Region_5199	chr14	23520857	23556192	35335	20	111	133.2272727	10.59723542	0.835500043	0.027225944	-	85446	grey	-0.100718614	FALSE	ZFHX2

Region_5200	chr14	23559565	23568070	8505	13	81	103.2272727	9.37229215	0.49678832	0.036974065	-	8906	grey	0.054938892	FALSE	AP1G2
Region_5201	chr14	23561097	23568073	6976	12	76	97.24242424	9.011207733	0.523272502	0.032792822	+	102724814	grey	0.032765323	FALSE	AP1G2-AS1
Region_5202	chr14	23568038	23578790	10752	6	21	36.84848485	5.823671652	0.510139944	0.05202388	-	84502	grey	0.115276505	FALSE	JPH4
Region_5203	chr14	24399003	24419283	20280	7	29	44.78787879	7.03292291	0.939068435	0.038772834	+	57523	grey	0.005218381	FALSE	NYNRIN
Region_5204	chr14	24429286	24441843	12557	4	19	29.86363636	5.389123987	0.478200643	0.117024266	+	23351	grey	0.028084374	FALSE	KHNYN
Region_5205	chr14	24443010	24503991	60981	11	45	64.59090909	10.08880152	0.81617182	0.034916967	+	101927045	grey	0.020974138	FALSE	LOC101927045
Region_5206	chr14	24595723	24634492	38769	10	70	90.90909091	10.60149086	0.821219768	0.035056719	+	107984667	grey	0.084571137	FALSE	
Region_5207	chr14	24809454	25050147	240693	14	92	110.6212121	8.964485744	0.811312911	0.03590263	-	29091	grey	0.299358412	FALSE	STXBP6
Region_5208	chr14	25647304	26143305	496001	37	285	332.7878788	18.44452402	0.845180871	0.021310636	-	107984700	grey	0.025356085	FALSE	LINC02306
Region_5209	chr14	26443090	26598033	154943	7	47	59.90909091	7.460103676	0.699419868	0.054287978	-	4857	grey	0.019796546	FALSE	NOVA1
Region_5210	chr14	26598369	27295516	697147	35	267	302.5151515	17.68723349	0.823470908	0.02719565	+	102725045	grey	0.405550968	FALSE	NOVA1-DT
Region_5211	chr14	26843452	27160420	316968	10	69	87.28787879	8.862653104	0.860692509	0.042947781	+	105370420	grey	0.353625501	FALSE	LOC105370420
Region_5212	chr1	26873092	26918594	45502	3	18	29.9545454	5.98313480	0.91909618	0.05158488	+	10192708	grey	0.181883249	FALSE	MIR4307HG

	4						5	5	3	4		1				
Region_5213	chr14	27259142	27845286	586144	26	195	234.1212121	14.82878194	0.805429418	0.0306535	-	728755	grey	-0.112149864	FALSE	MIR3171HG
Region_5214	chr14	28592223	28613623	21400	4	20	31.93939394	5.471264199	0.850152932	0.067729395	-	105370422	grey	0.147995488	FALSE	LINC02300
Region_5215	chr14	28726675	28765292	38617	3	13	25.57575758	4.537232657	0.610637693	0.072364567	-	103695363	grey	-0.248811622	FALSE	FOXG1-AS1
Region_5216	chr14	28765640	28823817	58177	8	58	76.81818182	9.80095614	0.168298842	0.032808664	+	387978	grey	-0.001230316	FALSE	LINC01551
Region_5217	chr14	28766787	28770277	3490	3	18	33.31818182	5.455157308	0.084991743	0.052782153	+	2290	grey	0.054502911	FALSE	FOXG1
Region_5218	chr14	28829881	28968400	138519	7	43	56.83333333	6.715271535	0.841138137	0.046593109	+	102724917	grey	0.037229452	FALSE	LINC02327
Region_5219	chr14	28875404	29064993	189589	9	60	78.89393939	8.80756456	0.818866615	0.050230218	-	105370425	grey	-0.014152366	FALSE	LINC02326
Region_5220	chr14	28936889	29170064	233175	10	67	85.18181818	9.413425913	0.806928667	0.051673734	+	107984685	grey	-0.023223113	FALSE	LOC107984685
Region_5221	chr14	29210986	29392048	181062	17	119	145.6060606	12.19946883	0.743615959	0.03926293	-	102724934	grey	0.325157366	FALSE	LOC102724934
Region_5222	chr14	29576479	30191898	615419	41	330	373.4848485	17.67069928	0.781929441	0.021908776	-	5587	grey	0.207516917	FALSE	PRKD1
Region_5223	chr14	30437992	30573629	135637	8	40	61.13636364	7.671899501	0.925193287	0.032731478	-	105370431	grey	0.06053433	FALSE	G2E3-AS1
Region_5224	chr14	30559158	30620064	60906	4	19	28.93939394	4.889167895	0.971362639	0.036703004	+	55632	grey	-0.124264244	FALSE	G2E3

Region_5225	chr14	30622291	30737694	115403	12	70	93.18181818	9.159961523	0.856707439	0.027106616	+	23256	grey	0.246478665	FALSE	SCFD1
Region_5226	chr14	30874514	30895065	20551	3	15	26.96969697	4.803747566	0.91565234	0.058399829	+	1690	grey	0.023055257	FALSE	COCH
Region_5227	chr14	30893799	31026401	132602	17	114	134.8636364	10.31486808	0.845836461	0.034529254	-	29966	grey	-0.01411514	FALSE	STRN3
Region_5228	chr14	31025106	31130996	105890	23	153	190.4090909	13.29890368	0.885430888	0.022520845	+	11154	grey	-0.02255595	FALSE	AP4S1
Region_5229	chr14	31100117	31207804	107687	16	104	136.9848485	12.55448731	0.883771724	0.021052471	-	25831	grey	0.117706074	FALSE	HECTD1
Region_5230	chr14	31248628	31302739	54111	10	57	78.93939394	8.911321732	0.91720669	0.030443752	+	105370436	grey	0.02917929	FALSE	
Region_5231	chr14	31291788	31457441	165653	29	169	202.9242424	16.21519763	0.855291573	0.017229068	-	25938	grey	-0.192733235	FALSE	HEATR5A
Region_5232	chr14	31420286	31452883	32597	9	30	46.10606061	6.773543074	0.86247635	0.030604478	+	101927124	grey	-0.022055372	FALSE	HEATR5A-DT
Region_5233	chr14	31427507	31457506	29999	9	33	54.81818182	7.873830249	0.698133561	0.048151901	-	112487	grey	-0.241402301	FALSE	DTD2
Region_5234	chr14	31489956	31562197	72241	17	95	127.1969697	11.68196466	0.858808979	0.034018192	+	105370438	grey	0.260684082	FALSE	LOC105370438
Region_5235	chr14	31561385	31861224	299839	39	285	336.0909091	18.46804081	0.944863705	0.015329786	+	80224	grey	0.236219285	FALSE	NUBPL
Region_5236	chr14	32076114	32159728	83614	4	23	33.63636364	5.548766637	0.737689598	0.020497457	+	394	grey	-0.191680455	FALSE	ARHGAP5
Region_5237	chr1	32329298	32837684	508386	52	381	427.651515	21.3940987	0.85545278	0.02521344	+	9472	grey	-0.01122578	FALSE	AKAP6

	4						2			2				4		
Region_5238	chr14	32934396	33820863	886467	101	779	860.8484848	30.35697483	0.835556548	0.019531869	+	64067	grey	-0.148648724	FALSE	NPAS3
Region_5239	chr14	33924227	33951074	26847	3	17	26.22727273	4.498873208	0.610697619	0.100752335	-	112399	grey	0.072310921	FALSE	EGLN3
Region_5240	chr14	33929099	34462774	533675	79	565	630.4848485	23.01721257	0.863489716	0.016915829	-	102724945	grey	-0.291310104	FALSE	LOC102724945
Region_5241	chr14	34432788	34462240	29452	13	67	98.31818182	11.11777384	0.959644908	0.022962779	-	171546	grey	-0.150277477	FALSE	SPTSSA
Region_5242	chr14	34515938	34539704	23766	8	35	52.96969697	6.914799506	0.965887254	0.027748891	-	55837	grey	0.046297561	FALSE	EAPP
Region_5243	chr14	34561093	34630160	69067	18	111	133.9848485	11.51653316	0.875205491	0.027172802	-	58533	grey	-0.213982848	FALSE	SNX6
Region_5244	chr14	34752731	34875647	122916	25	159	189.6212121	14.52823792	0.7939564	0.022764131	-	11177	grey	-0.180738336	FALSE	BAZ1A
Region_5245	chr14	34911580	34982912	71332	33	212	255.4545455	19.61724659	0.943745341	0.01587663	-	100506157	grey	0.098390955	FALSE	SRP54-AS1
Region_5246	chr14	34981957	35029686	47729	22	155	195.5757576	15.17088366	0.963704613	0.017013694	+	6729	grey	-0.279334395	FALSE	SRP54
Region_5247	chr14	35045117	35114363	69246	30	183	216.3787879	15.6812138	0.89385327	0.018686146	+	283635	grey	0.259069107	FALSE	FAM177A1
Region_5248	chr14	35081081	35116630	35549	13	72	94.89393939	8.710963539	0.972833186	0.018844967	+	101927178	grey	0.294895466	FALSE	LOC101927178
Region_5249	chr14	35085467	35122517	37050	14	80	104.1666667	9.347288103	0.942339373	0.021129864	-	55012	grey	0.248555969	FALSE	PPP2R3C

Region_5250	chr14	35121846	35317474	195628	44	299	346.1969697	22.0839097	0.831066558	0.015467108	+	9692	grey	0.030668222	FALSE	PRORP
Region_5251	chr14	35278633	35317493	38860	5	25	44.77272727	7.406432661	0.392559808	0.02234652	+	5687	grey	3.26332E-05	FALSE	PSMA6
Region_5252	chr14	35401079	35404749	3670	6	31	49.34848485	6.669084643	0.122355236	0.026613749	-	4792	grey	-0.262114374	FALSE	NFKBIA
Region_5253	chr14	35534164	35537054	2890	3	10	20.89393939	4.893099656	0.04057055	0.044120374	+	84684	grey	-0.106535744	FALSE	INSM2
Region_5254	chr14	35538352	35809304	270952	39	285	330.0909091	17.78394027	0.886030351	0.016200915	-	253959	grey	0.099849236	FALSE	RALGAPA1
Region_5255	chr14	35826338	35932325	105987	22	151	177.3484848	13.38137905	0.887931205	0.018474277	+	84312	grey	0.170819549	FALSE	BRMS1L
Region_5256	chr14	36061026	36067190	6164	3	14	23.96969697	4.516532697	0.909061582	0.070245946	-	105370452	grey	0.03592607	FALSE	LOC105370452
Region_5257	chr14	36070427	36165288	94861	10	68	88.51515152	8.473020394	0.80865325	0.046542573	+	101101773	grey	0.067909304	FALSE	LINC00609
Region_5258	chr14	36136108	36176468	40360	3	11	24.65151515	5.512203034	0.652291351	0.099762085	-	100886964	grey	-0.169383098	FALSE	PTCSC3
Region_5259	chr14	36473207	36521149	47942	16	95	115.6818182	10.44275102	0.243980097	0.029196688	-	253970	grey	-0.089658411	FALSE	SFTA3
Region_5260	chr14	36516392	36521149	4757	5	20	32.31818182	5.248176506	0.047369848	0.040231584	-	7080	grey	0.174053209	FALSE	NKX2-1
Region_5261	chr14	36647083	36658801	11718	6	39	55.15151515	8.541739013	0.068882126	0.037397316	-	105370455	grey	-0.173205802	FALSE	LOC105370455
Region_5262	chr1	36657568	36679362	21794	7	58	80.2272727	9.61856450	0.06001060	0.04052142	+	5083	grey	0.029560323	FALSE	PAX9

	4						3	9	1	3						
Region_5263	chr14	36677921	37172606	494685	58	431	486.4393939	26.37605487	0.789776096	0.01879919	-	89874	grey	0.054701061	FALSE	SLC25A21
Region_5264	chr14	37197894	37579125	381231	27	190	222.2727273	13.37799048	0.880416776	0.023928505	+	145282	grey	0.07924248	FALSE	MIPOL1
Region_5265	chr14	37595629	38041442	445813	36	251	290.5454545	18.39078517	0.761128645	0.022450461	+	319089	grey	-0.1052084	FALSE	TTC6
Region_5266	chr14	38003113	38194281	191168	21	158	186.3636364	13.32855392	0.850537095	0.035290868	-	105370456	grey	-0.229292018	FALSE	LOC105370456
Region_5267	chr14	38256202	38371338	115136	5	24	36.34848485	6.220295378	0.936165691	0.036832876	+	112268136	grey	-0.038982486	FALSE	LOC112268136
Region_5268	chr14	38738155	38948273	210118	19	130	151.5606061	11.93202386	0.907428666	0.033402247	-	283547	grey	0.208945801	FALSE	LINC00639
Region_5269	chr14	39031919	39109646	77727	11	65	86.33333333	9.69747522	0.9743121	0.02069068	-	10484	grey	-0.114447871	FALSE	SEC23A
Region_5270	chr14	39230231	39388513	158282	18	102	144.0151515	13.56635252	0.741471771	0.024306165	+	4253	grey	0.046548972	FALSE	MIA2
Region_5271	chr14	39397684	39432433	34749	9	54	71.78787879	9.091864752	0.935947006	0.049006842	-	254170	grey	0.409306102	FALSE	FBXO33
Region_5272	chr14	39432599	39513780	81181	6	32	46.68181818	5.865568609	0.898129934	0.060610737	+	112267899	grey	0.080542181	FALSE	LOC112267899
Region_5273	chr14	39432607	40200727	768120	62	453	502.4393939	21.1188059	0.822913385	0.024651994	+	105370461	grey	0.226902428	FALSE	LOC105370461
Region_5274	chr14	39432607	40390547	957940	70	503	557.8181818	22.90375118	0.827494467	0.023479299	+	105370462	grey	0.318261113	FALSE	LOC105370462

Region_5275	chr14	40267283	40348331	81048	4	17	28.31818182	5.786347568	0.871912436	0.080375169	-	105370463	grey	0.381741007	FALSE	LOC105370463
Region_5276	chr14	40630006	40905513	275507	12	74	91.42424242	8.634564897	0.843535663	0.038926884	+	105370467	grey	0.139502689	FALSE	LOC105370467
Region_5277	chr14	40954693	41149309	194616	9	53	68.3030303	8.754725664	0.727181711	0.063990761	+	644919	grey	0.05721895	FALSE	LINC02315
Region_5278	chr14	41606876	41904549	297673	28	191	230.4393939	16.03949816	0.692337561	0.036566335	+	145581	grey	-0.170456892	FALSE	LRFN5
Region_5279	chr14	43990539	44386064	395525	12	76	98.37878788	9.449078346	0.840283034	0.043636982	-	105370471	grey	0.018044779	FALSE	LINC02307
Region_5280	chr14	44444747	44480626	35879	4	15	28.66666667	6.145250407	0.91164584	0.053594371	-	107984027	grey	-0.100869518	FALSE	LINC02277
Region_5281	chr14	44924324	45042308	117984	13	95	115.3939394	11.11738595	0.893584661	0.015776624	-	54813	grey	-0.043386457	FALSE	KLHL28
Region_5282	chr14	44962190	45074431	112241	14	101	120.9848485	9.942128582	0.870253652	0.026475598	+	23116	grey	-0.158315685	FALSE	TOGARAM1
Region_5283	chr14	45084107	45116282	32175	9	45	68.33333333	8.120881599	0.843618423	0.027478713	+	55015	grey	-0.133000023	FALSE	PRPF39
Region_5284	chr14	45135930	45200890	64960	4	19	28.72727273	4.737884807	0.793793815	0.057453982	+	57697	grey	0.098315019	FALSE	FANCM
Region_5285	chr14	45203190	45253540	50350	5	25	41.71212121	7.593043682	0.940149609	0.042575502	-	55320	grey	-0.335928328	FALSE	MIS18BP1
Region_5286	chr14	45891087	46501903	610816	43	282	337.5909091	19.416866	0.825144855	0.022575734	+	100506412	grey	0.514809508	FALSE	LINC00871
Region_5287	chr1	46839629	47675605	835976	49	375	430.742424	21.0357282	0.86000489	0.02151513	-	161357	grey	0.1473624	FALSE	MDGA2

	4						2	9	6							
Region_5288	chr14	47764954	47795302	30348	3	11	22.60606061	5.149693905	0.738342108	0.105727913	-	100506433	grey	0.107670141	FALSE	LINC00648
Region_5289	chr14	48194174	48228216	34042	5	27	45.24242424	6.759746482	0.884695417	0.072846412	+	101927483	grey	0.150422645	FALSE	LOC101927483
Region_5290	chr14	48233743	48260643	26900	3	18	30.45454545	5.014384902	0.662482148	0.057556401	-	105370482	grey	0.138805115	FALSE	LOC105370482
Region_5291	chr14	48394815	49077505	682690	36	276	309.1818182	16.87137684	0.834712163	0.032860491	-	105378178	grey	0.573183965	FALSE	LOC105378178
Region_5292	chr14	49570984	49598690	27706	14	88	117.7424242	11.81564882	0.543896853	0.024786785	-	6235	grey	0.06395707	FALSE	RPS29
Region_5293	chr14	49598761	49614672	15911	4	20	31.57575758	4.794421778	0.716752303	0.03598403	+	122769	grey	-0.134166208	FALSE	LRR1
Region_5294	chr14	49693105	49753150	60045	13	72	100.0909091	9.869498113	0.801131952	0.031139811	+	122773	grey	0.132387431	FALSE	KLHDC1
Region_5295	chr14	49768130	49786385	18255	4	17	27.34848485	5.055058165	0.004640152	0.013154832	+	23588	grey	0.071577463	FALSE	KLHDC2
Region_5296	chr14	49782083	49852821	70738	7	34	46.3030303	7.268413639	0.976276057	0.032074579	-	9147	grey	0.049023906	FALSE	NEMF
Region_5297	chr14	49893082	49897054	3972	3	23	33.31818182	4.820338612	0.002509382	0.008203764	+	382	grey	0.217254735	FALSE	ARF6
Region_5298	chr14	49927571	49961960	34389	4	25	39.21212121	6.601899847	0.943413638	0.042488483	-	100506446	grey	-0.191589978	FALSE	LOC100506446
Region_5299	chr14	49960544	50105102	144558	40	282	343.3484848	20.48451004	0.849457212	0.017887608	-	196913	grey	0.131975581	FALSE	LINC01599

Region_5300	chr14	49981673	50007520	25847	3	14	25.45454545	4.855391351	0.877893485	0.070764199	-	283551	grey	0.217738602	FALSE	LINC01588
Region_5301	chr14	50117130	50231578	114448	26	179	212.1666667	15.88863487	0.974372576	0.012532024	-	6655	grey	0.303381335	FALSE	SOS2
Region_5302	chr14	50237563	50312229	74666	18	124	156.7575758	15.50092861	0.909005579	0.019096334	-	79944	grey	0.166479681	FALSE	L2HGDH
Region_5303	chr14	50312324	50335558	23234	10	52	73.34848485	9.586035395	0.923167222	0.031025846	+	27109	grey	-0.035010919	FALSE	DMAC2L
Region_5304	chr14	50326265	50416461	90196	29	167	206.3333333	15.49822157	0.766252415	0.01899215	-	8814	grey	-0.187258271	FALSE	CDKL1
Region_5305	chr14	50418501	50561126	142625	20	129	160.3484848	13.24618078	0.746560542	0.026593924	-	11183	grey	0.103338314	FALSE	MAP4K5
Region_5306	chr14	50532509	50634017	101508	18	118	145.2575758	12.68713535	0.810186784	0.031139544	+	51062	grey	-0.080476488	FALSE	ATL1
Region_5307	chr14	50632058	50668306	36248	8	33	53.62121212	8.068485992	0.252663733	0.018601592	-	60485	grey	-0.04967789	FALSE	SAV1
Region_5308	chr14	50719763	50831162	111399	20	138	156.5909091	10.20705227	0.729776393	0.023485035	-	51199	grey	-0.106014654	FALSE	NIN
Region_5309	chr14	50857891	50944483	86592	21	115	144.530303	13.9120531	0.903108972	0.020125158	-	5836	grey	0.103243239	FALSE	PYGL
Region_5310	chr14	50975262	51096061	120799	19	128	160.5	14.51285902	0.657463368	0.025090625	-	114088	grey	-0.391733234	FALSE	TRIM9
Region_5311	chr14	51240162	51257655	17493	4	21	32.77272727	5.830412189	0.708322633	0.044294203	+	81542	grey	-0.029812783	FALSE	TMX1
Region_5312	chr1	51333068	51365557	32489	10	66	84.4545454	10.0967349	0.94929300	0.02844006	+	283553	grey	-0.13828454	FALSE	LINC00640

	4						5	2	8	2				4		
Region_5313	chr14	51379509	51599972	220463	49	395	447.2121212	21.71105011	0.900626506	0.020179403	-	105370496	grey	0.110175266	FALSE	LINC02310
Region_5314	chr14	51454512	51651233	196721	39	304	354.5909091	18.35293083	0.885307228	0.022528317	-	100874185	grey	0.043094622	FALSE	FRMD6-AS2
Region_5315	chr14	51489100	51730727	241627	39	310	356.4848485	17.9093808	0.858029981	0.024164276	+	122786	grey	-0.017508889	FALSE	FRMD6
Region_5316	chr14	51765276	51825422	60146	9	53	67.03030303	7.834773959	0.927925078	0.033924559	+	101927598	grey	0.097975338	FALSE	LOC101927598
Region_5317	chr14	51826195	51979342	153147	13	72	94.45454545	9.359786023	0.794864542	0.024424311	+	54331	grey	-0.055846084	FALSE	GNG2
Region_5318	chr14	51989514	52010694	21180	3	21	34.45454545	6.251601193	0.97372676	0.028408019	+	51637	grey	-0.051637979	FALSE	RTRAF
Region_5319	chr14	52004809	52069059	64250	13	90	112.0757576	11.66890754	0.916468822	0.028440045	-	22795	grey	0.017238438	FALSE	NID2
Region_5320	chr14	52314305	52328598	14293	4	20	34.03030303	7.860259727	0.006495311	0.01521713	+	5732	grey	0.186618391	FALSE	PTGER2
Region_5321	chr14	52430596	52552522	121926	22	158	186.6363636	13.47891386	0.865502137	0.026313917	-	57544	grey	0.272257894	FALSE	TXNDC16
Region_5322	chr14	52552836	52637713	84877	8	40	52.8030303	6.337239625	0.781908105	0.039451994	+	283554	grey	-0.235971042	FALSE	GPR137C
Region_5323	chr14	52639915	52695900	55985	7	33	55.5	6.469454858	0.409798635	0.028884319	-	30001	grey	0.039516343	FALSE	ERO1A
Region_5324	chr14	52707178	52728590	21412	4	28	39.1969697	5.85522698	0.487899131	0.02063095	+	5706	grey	0.00148033	FALSE	PSMC6

Region_5325	chr14	52730166	52774989	44823	11	75	96.96969697	9.783769443	0.745855993	0.039231682	+	6815	grey	0.48101156	FALSE	STYX
Region_5326	chr14	52775193	52791668	16475	4	24	34.60606061	5.397659857	0.464752914	0.036540078	-	64841	grey	-0.034330414	FALSE	GNPNAT1
Region_5327	chr14	52791756	52930185	138429	25	168	205.1060606	16.97249249	0.882544997	0.024094321	+	105370500	grey	-0.254703392	FALSE	LOC105370500
Region_5328	chr14	52857268	52952435	95167	16	104	130.6515152	12.93354901	0.753161626	0.027379739	-	10979	grey	-0.055805806	FALSE	FERMT2
Region_5329	chr14	53036745	53153323	116578	20	134	156.469697	12.68702511	0.91543443	0.017273603	-	80821	grey	0.024803895	FALSE	DDHD1
Region_5330	chr14	53169010	53406231	237221	22	164	187	12.91748963	0.856243539	0.03759301	+	105370502	grey	-0.026945394	FALSE	LOC105370502
Region_5331	chr14	53169034	53575250	406216	40	290	327.8636364	17.32839942	0.865077461	0.024557506	+	112268134	grey	-0.20578545	FALSE	
Region_5332	chr14	53217655	53786106	568451	57	425	475.8939394	24.77413589	0.837119934	0.023221692	+	105370504	grey	-0.094292538	FALSE	LOC105370504
Region_5333	chr14	53685139	53850882	165743	11	99	122.3939394	10.83850513	0.883907221	0.039338399	-	105370503	grey	0.044250165	FALSE	LINC02331
Region_5334	chr14	54396849	54420218	23369	6	40	54.18181818	7.159521022	0.654508331	0.015493296	+	1033	grey	0.221408049	FALSE	CDKN3
Region_5335	chr14	54566117	54793315	227198	33	194	236.0606061	15.39718135	0.791094572	0.021917809	+	23034	grey	0.318135667	FALSE	SAMD4A
Region_5336	chr14	54684026	54729324	45298	7	36	55.5	7.847096474	0.770858338	0.06399773	-	729451	grey	0.006567682	FALSE	SAMD4A-AS1
Region_5337	chr1	54842008	54902826	60818	19	123	166.181818	14.2244741	0.86219726	0.02702232	-	2643	grey	0.036100078	FALSE	GCH1

	4						2	3	9							
Region_5338	chr14	54938949	55027105	88156	19	118	142.6818182	14.34965461	0.932638656	0.018930176	-	11169	grey	-0.275261402	FALSE	WDHD1
Region_5339	chr14	55027230	55049489	22259	15	92	111.7272727	11.6876601	0.972220287	0.016317037	+	122809	grey	0.026326738	FALSE	SOCS4
Region_5340	chr14	55051647	55070194	18547	3	13	20.65151515	4.589259784	0.968015031	0.052230382	+	93487	grey	-0.025193318	FALSE	MAPK1IP1L
Region_5341	chr14	55124110	55145423	21313	7	40	59.59090909	8.66287796	0.729322664	0.026085029	+	3958	grey	0.150533233	FALSE	LGALS3
Region_5342	chr14	55148112	55191608	43496	11	63	85.51515152	9.364815369	0.965027978	0.021047052	-	9787	grey	-0.082133798	FALSE	DLGAP5
Region_5343	chr14	55262222	55272075	9853	5	14	23.15151515	5.491930932	0.004242424	0.013904995	-	105370509	grey	-0.183643629	FALSE	FBXO34-AS1
Region_5344	chr14	55271344	55370053	98709	44	309	356.2878788	25.96907341	0.825985919	0.013664641	+	55030	grey	0.045396001	FALSE	FBXO34
Region_5345	chr14	55366391	55411830	45439	25	180	225.8787879	20.36702852	0.890567767	0.016446627	-	22863	grey	0.092261587	FALSE	ATG14
Region_5346	chr14	55414210	55456726	42516	8	30	50.77272727	9.261658689	0.825713423	0.03941471	-	387332	grey	-0.216342493	FALSE	TBPL2
Region_5347	chr14	55496786	55580888	84102	16	99	127.5909091	10.94458387	0.629285353	0.022542824	-	100129075	grey	-0.016514113	FALSE	KTN1-AS1
Region_5348	chr14	55559072	55701526	142454	25	150	187.8181818	15.07408279	0.707209808	0.018122876	+	3895	grey	0.062767093	FALSE	KTN1
Region_5349	chr14	55896443	55962970	66527	9	44	70.27272727	8.890003815	0.936204475	0.036875512	+	102723670	grey	0.033001582	FALSE	LOC102723670

Region_5350	chr14	56117814	56301524	183710	33	222	264.1363636	17.891026	0.569634649	0.02798459	+	57161	grey	0.030723389	FALSE	PELI2
Region_5351	chr14	56310880	56427032	116152	10	71	90.62121212	8.439404192	0.795428179	0.062534112	+	102723699	grey	0.22561249	FALSE	LINC02284
Region_5352	chr14	56488354	56650606	162252	25	178	207.7878788	12.76957339	0.851153994	0.024771101	+	54916	grey	-0.041523033	FALSE	TMEM260
Region_5353	chr14	56514331	56551309	36978	3	17	26.39393939	5.137730082	0.801872013	0.074879917	+	101927690	grey	0.188052907	FALSE	LOC101927690
Region_5354	chr14	56799905	56816693	16788	6	18	37.54545455	5.865628219	0.051856492	0.042333861	-	5015	grey	0.235886443	FALSE	OTX2
Region_5355	chr14	56813183	57152177	338994	36	258	296.469697	17.0530572	0.760417309	0.030109931	+	100309464	grey	0.272236642	FALSE	OTX2-AS1
Region_5356	chr14	57200507	57268905	68398	7	34	53.06060606	6.759332666	0.827711921	0.030241603	-	10640	grey	-0.078792864	FALSE	EXOC5
Region_5357	chr14	57268924	57298742	29818	13	78	109.6060606	11.4083354	0.974811795	0.016171777	+	55745	grey	0.104245113	FALSE	AP5M1
Region_5358	chr14	57390544	57415906	25362	4	21	35.90909091	5.606938758	0.949190115	0.045055443	+	122830	grey	0.044555299	FALSE	NAA30
Region_5359	chr14	57469300	57493867	24567	3	16	24.83333333	4.919610158	0.567113638	0.108908522	-	55195	grey	0.007335263	FALSE	CCDC198
Region_5360	chr14	57563920	57982194	418274	37	277	311.1969697	17.77051984	0.759932427	0.028513371	-	341880	grey	-0.209201223	FALSE	SLC35F4
Region_5361	chr14	57999735	58298139	298404	26	158	190.9848485	13.06256239	0.748456372	0.024785835	-	145407	grey	-0.125075409	FALSE	ARMH4
Region_5362	chr1	58200080	58235636	35556	5	14	28.2575757	5.19559164	0.78334208	0.02220579	+	55860	grey	0.072701614	FALSE	ACTR10

	4						6		7	8						
Region_5363	chr14	58298504	58373887	75383	9	63	83.22727273	8.791246441	0.864466706	0.019484881	+	5926	grey	-0.025846726	FALSE	ARID4A
Region_5364	chr14	58370023	58395641	25618	4	14	25.83333333	4.802510027	0.965224758	0.047212315	-	105370522	grey	-0.11245468	FALSE	TOMM20L-DT
Region_5365	chr14	58408495	58427531	19036	6	33	48.07575758	7.494224661	0.938461408	0.035142281	-	26520	grey	-0.001185563	FALSE	TIMM9
Region_5366	chr14	58427385	58551297	123912	18	112	135.5151515	13.61361584	0.951887402	0.028216911	+	9786	grey	0.242035997	FALSE	KIAA0586
Region_5367	chr14	58633967	58648321	14354	5	23	34.24242424	5.513788607	0.296948531	0.051001602	+	51339	grey	-0.304795304	FALSE	DACT1
Region_5368	chr14	58646631	59184247	537616	74	518	591.1363636	28.49609224	0.902502164	0.019874133	+	102723742	grey	-0.111646654	FALSE	LINC01500
Region_5369	chr14	58828185	59184247	356062	46	323	365.1060606	20.03775574	0.899830768	0.024930937	+	107984642	grey	-0.133271414	FALSE	LOC107984642
Region_5370	chr14	59188646	59371405	182759	17	107	128.1969697	9.200701808	0.824641141	0.028402247	+	23002	grey	-0.244932584	FALSE	DAAM1
Region_5371	chr14	59429022	59475898	46876	6	32	45.12121212	5.679965199	0.904574968	0.041670503	-	64582	grey	0.193782343	FALSE	GPR135
Region_5372	chr14	59479274	59576812	97538	9	67	90.86363636	10.6653809	0.581197844	0.044926931	-	729665	grey	0.029613558	FALSE	CCDC175
Region_5373	chr14	59484443	59505410	20967	5	39	54.43939394	7.407125027	0.583013443	0.018635187	+	51528	grey	-0.056327627	FALSE	JKAMP
Region_5374	chr14	59595976	59870776	274800	27	160	207.6515152	16.0992044	0.845185514	0.024683515	-	6252	grey	-0.463251914	FALSE	RTN1

Region_5375	chr14	59919713	60063559	143846	9	49	63.6969697	7.698091066	0.840808863	0.039798154	+	341883	grey	-0.108122397	FALSE	LRRC9
Region_5376	chr14	59969116	60091783	122667	8	45	67.75757576	7.870217681	0.918748291	0.037143369	-	105370524	grey	-0.108835531	FALSE	PCNX4-DT
Region_5377	chr14	60245752	60299087	53335	5	26	38.01515152	6.749341676	0.868448905	0.054923025	+	5494	grey	0.207307401	FALSE	PPM1A
Region_5378	chr14	60396469	60515543	119074	12	69	97.83333333	12.93008815	0.541201675	0.026491005	-	317761	grey	-0.111556555	FALSE	C14orf39
Region_5379	chr14	60509146	60512850	3704	5	22	45.03030303	9.390616752	0.04246135	0.034985538	+	4990	grey	-0.00092526	FALSE	SIX6
Region_5380	chr14	60643421	60658259	14838	8	36	57.98484848	9.662763459	0.042554215	0.032346685	-	6495	grey	0.146232282	FALSE	SIX1
Region_5381	chr14	60709539	60724351	14812	15	108	139.6515152	16.13166253	0.374190438	0.01813824	-	51804	grey	-0.105629479	FALSE	SIX4
Region_5382	chr14	60734742	60982585	247843	33	233	267.6666667	14.78079142	0.87616879	0.013016701	+	4331	grey	-0.187204394	FALSE	MNAT1
Region_5383	chr14	60981114	61114316	133202	17	113	144.0757576	11.13872055	0.813146679	0.023366107	+	145389	grey	-0.13986743	FALSE	SLC38A6
Region_5384	chr14	61187559	61657964	470405	69	526	580.8333333	23.35846632	0.637135456	0.017997149	+	5583	grey	0.186742257	FALSE	PRKCH
Region_5385	chr14	61277370	61281338	3968	3	18	28.87878788	5.736563301	0.157055493	0.063078607	-	161291	grey	0.264778612	FALSE	TMEM30B
Region_5386	chr14	61556273	61570653	14380	3	17	25.90909091	4.440122218	0.103421451	0.076649507	-	101927780	grey	-0.091578883	FALSE	LINC01303
Region_5387	chr1	61570405	61658696	88291	14	83	112.560606	12.1644232	0.94028218	0.02220585	+	400221	grey	0.198752722	FALSE	LINC03033

	4						1	7	4	1						
Region_5388	chr14	61680723	61695892	15169	3	10	19.54545455	4.413900484	0.6631949	0.084150961	-	100750246	grey	0.216236483	FALSE	HIF1A-AS1
Region_5389	chr14	61695513	61748259	52746	9	66	82.87878788	8.363869461	0.833648821	0.036896054	+	3091	grey	0.002731311	FALSE	HIF1A
Region_5390	chr14	61707298	61751163	43865	6	39	57.75757576	7.807413846	0.777400414	0.045716788	-	105370526	grey	0.056975773	FALSE	HIF1A-AS3
Region_5391	chr14	61734138	61796428	62290	17	100	136.0151515	12.5324102	0.799148935	0.017588372	+	6617	grey	0.087290329	FALSE	SNAPC1
Region_5392	chr14	61811974	62128421	316447	35	249	302.6969697	21.16162688	0.865616071	0.02574612	+	83851	grey	0.034950006	FALSE	SYT16
Region_5393	chr14	62699464	63102037	402573	29	202	247.6363636	18.00823432	0.807029233	0.026090471	-	27133	grey	0.394323821	FALSE	KCNH5
Region_5394	chr14	63122888	63178190	55302	3	15	22.89393939	4.56113612	0.960116974	0.036772222	-	105370531	grey	-0.091777655	FALSE	LOC105370531
Region_5395	chr14	63204114	63293508	89394	12	76	93.54545455	8.432869423	0.884986966	0.036786155	+	57381	grey	0.062682686	FALSE	RHOJ
Region_5396	chr14	63371364	63543377	172013	21	139	166.6969697	11.51316263	0.920644752	0.019407163	-	5529	grey	-0.152341003	FALSE	PPP2R5E
Region_5397	chr14	63597039	63641871	44832	6	32	52.84848485	7.56966095	0.64521879	0.03384155	-	112840	grey	-0.087628149	FALSE	WDR89
Region_5398	chr14	63642035	63665593	23558	3	18	27.10606061	5.552903019	0.645107946	0.056711222	+	105370532	grey	0.095900388	FALSE	LOC105370532
Region_5399	chr14	63761899	64226433	464534	108	884	938.7575758	31.62086001	0.887384171	0.009777482	+	23224	grey	-0.019194881	FALSE	SYNE2

Region_5400	chr14	64084232	64338639	254407	71	571	617.6969697	27.6572259	0.884202062	0.014051762	-	2100	grey	-0.064194219	FALSE	ESR2
Region_5401	chr14	64341673	64387986	46313	8	47	64.96969697	8.607634709	0.902126372	0.064018579	-	441687	grey	-0.500304144	FALSE	TEX21P
Region_5402	chr14	64388031	64463457	75426	18	113	137.8636364	11.90977151	0.909893096	0.018381754	+	4522	grey	-0.106299442	FALSE	MTHFD1
Region_5403	chr14	64449106	64505213	56107	23	131	165.1060606	16.21787123	0.214296316	0.009435499	-	7597	grey	-0.025728132	FALSE	ZBTB25
Region_5404	chr14	64503725	64533690	29965	23	124	157.3333333	15.5051687	0.08628353	0.007348016	+	22890	grey	0.172515214	FALSE	ZBTB1
Region_5405	chr14	64535905	64546173	10268	3	24	35.10606061	5.069148883	0.131085171	0.062641814	+	3306	grey	-0.167663216	FALSE	HSPA2
Region_5406	chr14	64549920	64589381	39461	8	44	65.59090909	8.691246299	0.884624969	0.032288483	+	145376	grey	0.091824511	FALSE	PPP1R36
Region_5407	chr14	64704102	64750249	46147	11	76	92.68181818	9.067539894	0.861142517	0.032553923	+	26030	grey	0.014349736	FALSE	PLEKHG3
Region_5408	chr14	64746283	64879907	133624	47	313	361.5909091	23.19016852	0.85582522	0.018108497	-	6710	grey	-0.125623661	FALSE	SPTB
Region_5409	chr14	64914361	64935368	21007	8	50	70.28787879	10.01347577	0.6472945	0.05114681	+	91612	grey	0.192572773	FALSE	CHURC1
Region_5410	chr14	64914443	65062652	148209	57	378	438.1666667	26.02518504	0.617180786	0.017458418	+	100529261	grey	0.291696167	FALSE	CHURC1-FNTB
Region_5411	chr14	64945814	64973226	27412	26	127	169.0151515	16.79422284	0.491239319	0.025609871	-	376267	grey	0.060353685	FALSE	RAB15
Region_5412	chr1	64986895	65062650	75755	21	148	177.166666	12.3942708	0.73182625	0.02189370	+	2342	grey	0.39969544	FALSE	FNTB

	4						7		8	7						
Region_5413	chr14	65006174	65102695	96521	28	225	265.7575758	18.56469818	0.786766511	0.017252497	-	4149	grey	0.235673278	FALSE	MAX
Region_5414	chr14	65197254	65222443	25189	10	64	83.45454545	10.1529561	0.856259922	0.047753784	-	100128233	grey	0.253172819	FALSE	LINC02324
Region_5415	chr14	65410592	65744121	333529	55	377	433.030303	24.24234674	0.858671695	0.014004812	+	2530	grey	0.137437623	FALSE	FUT8
Region_5416	chr14	65411170	65412690	1520	3	17	25.39393939	4.967211841	0.006862369	0.019986647	-	645431	grey	-0.00918552	FALSE	FUT8-AS1
Region_5417	chr14	66411704	66498677	86973	7	50	66.46969697	7.475538976	0.80150112	0.031924696	+	440184	grey	-0.253577415	FALSE	CCDC196
Region_5418	chr14	66507407	67181803	674396	55	406	450.9242424	20.87347275	0.880998902	0.015870025	+	10243	grey	-0.111523115	FALSE	GPHN
Region_5419	chr14	67189393	67228550	39157	7	33	45.22727273	6.304566	0.955953814	0.037961943	+	161142	grey	-0.019311546	FALSE	GARIN2
Region_5420	chr14	67240713	67336061	95348	15	95	122.3333333	10.752221	0.8604543	0.039082069	+	64398	grey	-0.120175613	FALSE	PALS1
Region_5421	chr14	67294371	67360265	65894	6	41	52.51515152	6.87971919	0.797278378	0.054254567	-	51382	grey	-0.103180795	FALSE	ATP6V1D
Region_5422	chr14	67447084	67533739	86655	8	36	54	8.562889519	0.546042635	0.043570851	-	161145	grey	0.238900154	FALSE	TMEM229B
Region_5423	chr14	67533290	67589612	56322	10	55	70.83333333	8.386038458	0.6577376	0.038825766	+	57475	grey	0.19934418	FALSE	PLEKHH1
Region_5424	chr14	67581955	67600286	18331	4	21	31.51515152	5.201308783	0.75341183	0.067011833	-	5283	grey	0.052755163	FALSE	PIGH

Region_5425	chr14	67619920	67651708	31788	6	35	48.09090909	5.80643482	0.91355757	0.052811396	+	384	grey	0.182381941	FALSE	ARG2
Region_5426	chr14	67647085	67674820	27735	6	27	42.77272727	6.836093024	0.78645079	0.049698593	-	10490	grey	-0.036705435	FALSE	VTI1B
Region_5427	chr14	67659820	67695793	35973	7	51	69.03030303	8.882081698	0.451193311	0.03661331	-	51109	grey	-0.161719446	FALSE	RDH11
Region_5428	chr14	67701886	67734451	32565	7	50	62.53030303	7.664543133	0.86401267	0.055695777	+	145226	grey	-0.008120079	FALSE	RDH12
Region_5429	chr14	67727374	67816590	89216	15	85	111.2727273	11.09614688	0.903251308	0.018773968	-	23503	grey	0.069978914	FALSE	ZFYVE26
Region_5430	chr14	67819779	68730218	910439	118	926	1012.378788	44.41751739	0.881550483	0.009603417	+	5890	grey	-0.057866575	FALSE	RAD51B
Region_5431	chr14	68787660	68796253	8593	5	24	33.34848485	4.934937999	0.166019536	0.051271632	-	677	grey	0.090994979	FALSE	ZFP36L1
Region_5432	chr14	68874128	68979440	105312	23	174	203.3787879	14.53353168	0.76473072	0.040722932	-	87	grey	0.421142005	FALSE	ACTN1
Region_5433	chr14	69050881	69153150	102269	18	129	156.6363636	12.98239798	0.744417868	0.02510456	-	8816	grey	-0.04674575	FALSE	DCAF5
Region_5434	chr14	69183017	69260567	77550	30	177	238.9242424	21.8690583	0.436294454	0.015532707	-	105370550	grey	-0.250590149	FALSE	GALNT16-AS1
Region_5435	chr14	69191498	69244020	52522	6	37	54.6969697	6.750429495	0.978885055	0.019940542	+	55218	grey	-0.178710906	FALSE	EXD2
Region_5436	chr14	69259277	69357033	97756	67	448	519.6818182	31.88882567	0.547546777	0.011514206	+	57452	grey	-0.238093925	FALSE	GALNT16
Region_5437	chr1	69380128	69398299	18171	8	53	65.1060606	6.78035349	0.83972356	0.02325747	-	2079	grey	0.01558778	FALSE	ERH

	4						1	2	7	6						
Region_5438	chr14	69398015	69462390	64375	14	91	112.1060606	10.34501558	0.903339052	0.018965838	+	55334	grey	0.233708422	FALSE	SLC39A9
Region_5439	chr14	69484692	69531551	46859	10	48	67.15151515	7.967708722	0.732879739	0.041399179	+	400224	grey	0.272538173	FALSE	PLEKHD1
Region_5440	chr14	69569799	69574871	5072	4	15	30.28787879	6.024986822	0.39437292	0.148699578	-	56936	grey	0.069151337	FALSE	CCDC177
Region_5441	chr14	69611596	69715144	103548	20	141	166.5454545	12.46062329	0.639056319	0.02547502	+	9766	grey	0.243350766	FALSE	SUSD6
Region_5442	chr14	69726900	69772005	45105	19	120	147.5606061	12.01169787	0.507067182	0.016013905	+	6430	grey	-0.285543512	FALSE	SRSF5
Region_5443	chr14	69775416	69797241	21825	3	13	23.45454545	5.075375903	0.968146019	0.03833892	-	6554	grey	-0.029651372	FALSE	SLC10A1
Region_5444	chr14	69854131	70032366	178235	34	266	294.0757576	15.59517737	0.817613542	0.023305441	+	64093	grey	-0.117030314	FALSE	SMOC1
Region_5445	chr14	70044215	70189070	144855	24	143	175.6666667	14.25410422	0.614613226	0.03990396	-	6547	grey	0.036687739	FALSE	SLC8A3
Region_5446	chr14	70187123	70230187	43064	9	42	60.78787879	8.776744011	0.27631613	0.034450567	+	646548	grey	-0.061817803	FALSE	LOC646548
Region_5447	chr14	70255629	70343388	87759	12	82	102.5151515	10.71051593	0.861376343	0.023323091	+	101928046	grey	-0.199602221	FALSE	LOC101928046
Region_5448	chr14	70325081	70359700	34619	8	38	60.25757576	9.954219215	0.964756632	0.034991118	-	51241	grey	0.004051105	FALSE	COX16
Region_5449	chr14	70326064	70417074	91010	12	65	90.96969697	11.60499852	0.964711771	0.028054847	-	100529257	grey	-0.036362896	FALSE	SYNJ2BP-COX16

Region_5450	chr14	70359582	70417090	57508	4	21	30.71212121	4.982323065	0.964401526	0.042473044	-	55333	grey	-0.062095734	FALSE	SYNJ2BP
Region_5451	chr14	70417107	70460427	43320	4	24	34.40909091	5.406484064	0.843512721	0.05536452	+	8747	grey	-0.006779582	FALSE	ADAM21
Region_5452	chr14	70453541	70483775	30234	5	19	29.86363636	4.914300525	0.947020673	0.044606441	-	317760	grey	-0.187568114	FALSE	ADAM20P1
Region_5453	chr14	70581257	70641204	59947	8	59	70.83333333	7.494784511	0.920907311	0.033754483	-	10001	grey	0.060457511	FALSE	MED6
Region_5454	chr14	70608798	70641298	32500	4	27	39.42424242	4.980147066	0.877209085	0.051970718	-	101928075	grey	0.175438058	FALSE	TTC9-DT
Region_5455	chr14	70641916	70675366	33450	4	17	28.63636364	5.707313429	0.627590754	0.074479947	+	23508	grey	0.143215828	FALSE	TTC9
Region_5456	chr14	70722526	70809534	87008	25	177	211.9090909	13.95677643	0.875543515	0.020853914	-	4293	grey	-0.328103455	FALSE	MAP3K9
Region_5457	chr14	70907405	71115382	207977	25	195	224.9848485	14.77054493	0.856697045	0.027581933	+	22990	grey	-0.461678502	FALSE	PCNX1
Region_5458	chr14	71179053	71243841	64788	11	68	86.28787879	9.388643142	0.942794232	0.026604898	-	107984695	grey	0.219389568	FALSE	
Region_5459	chr14	71292729	71321882	29153	11	48	67.22727273	8.573295667	0.333123932	0.037802904	-	102724015	grey	0.026333486	FALSE	SIPA1L1-AS1
Region_5460	chr14	71320449	71741229	420780	64	475	532.4393939	24.73187217	0.827326132	0.0124798	+	26037	grey	-0.019799776	FALSE	SIPA1L1
Region_5461	chr14	71848606	72566530	717924	92	686	744.1666667	29.2088413	0.82162101	0.015704123	+	9628	grey	-0.08237102	FALSE	RGS6
Region_5462	chr1	72609034	72894101	285067	51	371	417.984848	25.0393489	0.89752453	0.01878981	-	8110	grey	-0.22311927	FALSE	DPF3

	4						5	9	9					1		
Region_5463	chr14	72926377	72959703	33326	13	70	103.9545455	10.06201053	0.928294613	0.038141424	+	26094	grey	-0.034907158	FALSE	DCAF4
Region_5464	chr14	72969451	73027131	57680	14	91	112.9393939	11.75503661	0.96152641	0.024936124	-	53349	grey	0.220842367	FALSE	ZFYVE1
Region_5465	chr14	73058532	73123899	65367	20	118	151.8787879	13.21248262	0.909569069	0.023610281	+	58517	grey	-0.130639013	FALSE	RBM25
Region_5466	chr14	73136418	73223691	87273	25	159	194.030303	14.07886544	0.947413642	0.015047589	+	5663	grey	0.305645759	FALSE	PSEN1
Region_5467	chr14	73237497	73274640	37143	21	110	138.4848485	12.95345802	0.749198865	0.022479332	+	89932	grey	0.069118178	FALSE	PAPLN
Region_5468	chr14	73275107	73458617	183510	56	390	435	22.70750469	0.8899656	0.015427869	-	8650	grey	-0.000841088	FALSE	NUMB
Region_5469	chr14	73366897	73463642	96745	36	223	265.5606061	16.34919785	0.790815616	0.019863438	-	101928143	grey	-0.025585566	FALSE	LOC101928143
Region_5470	chr14	73478484	73558947	80463	37	202	259.6363636	25.6218875	0.733965203	0.017247041	-	399671	grey	0.044812186	FALSE	HEATR4
Region_5471	chr14	73490933	73493394	2461	5	29	41.0454545	6.097746919	0.012215762	0.017993019	+	79697	grey	-0.07148178	FALSE	RIOX1
Region_5472	chr14	73567620	73575658	8038	8	16	53.1818181 8	10.11154571	0.403674421	0.043534599	+	10965	grey	-0.173786902	FALSE	ACOT2
Region_5473	chr14	73610945	73619888	8943	6	32	48.9545454 5	6.82851897	0.344634945	0.043211329	+	641372	grey	0.248989533	FALSE	ACOT6
Region_5474	chr14	73644875	73703732	58857	30	179	212.3939394	16.51654535	0.949153394	0.027915695	+	83544	grey	0.261815717	FALSE	DNAL1

Region_5475	chr14	73711783	73714384	2601	10	65	93.71212121	14.06114685	0.062994846	0.036196358	-	9240	grey	0.24094576	FALSE	PNMA1
Region_5476	chr14	73715122	73790285	75163	41	223	291.4545455	18.33800746	0.526388743	0.01731851	-	91748	grey	0.111056709	FALSE	MIDEAS
Region_5477	chr14	73822559	73830135	7576	3	13	26.1969697	5.899725409	0.970507577	0.037847844	-	100506498	grey	0.194436474	FALSE	LINC02274
Region_5478	chr14	73851831	73885726	33895	13	69	92.40909091	9.310006647	0.944990275	0.023917661	+	145482	grey	-0.005322098	FALSE	PTGR2
Region_5479	chr14	73851971	73932521	80550	32	210	243.1515152	17.98567158	0.857267704	0.017967565	+	57862	grey	0.070676611	FALSE	ZNF410
Region_5480	chr14	73913067	73950414	37347	22	131	179.469697	15.33444559	0.834259543	0.025971692	-	145483	grey	-0.050597312	FALSE	FAM161B
Region_5481	chr14	73949926	73963670	13744	9	59	77.96969697	10.37151379	0.742442055	0.023421002	+	51004	grey	0.177576943	FALSE	COQ6
Region_5482	chr14	73958010	74019399	61389	27	175	209.3484848	14.88467712	0.940882762	0.021894129	-	957	grey	0.414380994	FALSE	ENTPD5
Region_5483	chr14	74019349	74082863	63514	28	163	211.5909091	20.99375532	0.933733977	0.017204215	+	80127	grey	0.068672925	FALSE	BBOF1
Region_5484	chr14	74056847	74084492	27645	13	78	98.45454545	10.22091644	0.961819996	0.020997878	-	4329	grey	0.020032638	FALSE	ALDH6A1
Region_5485	chr14	74084956	74201493	116537	39	264	300.1363636	17.90907494	0.963579605	0.011858195	+	91750	grey	0.212479895	FALSE	LIN52
Region_5486	chr14	74239449	74262738	23289	28	174	207.7727273	18.78444156	0.469918018	0.023494205	+	338917	grey	-0.405280632	FALSE	VSX2
Region_5487	chr1	74285269	74303055	17786	6	30	45.0151515	5.62820486	0.5356276	0.04660882	-	5826	grey	-0.10268771	FALSE	ABCD4

	4						2	3		9				8		
Region_5488	chr14	74303069	74360008	56939	31	178	217.5909091	14.89551019	0.919069464	0.018997983	+	55237	grey	0.157417474	FALSE	VRTN
Region_5489	chr14	74405894	74474864	68970	21	150	179.6060606	15.01576483	0.790002112	0.02485008	-	646658	grey	0.012080964	FALSE	SYNDIG1L
Region_5490	chr14	74476192	74494177	17985	3	12	22.62121212	5.131397025	0.316084785	0.034701696	-	10577	grey	-0.06773027	FALSE	NPC2
Region_5491	chr14	74498183	74612378	114195	43	290	337.8181818	19.87569764	0.837635265	0.022680181	-	4053	grey	-0.042917597	FALSE	LTBP2
Region_5492	chr14	74653437	74713117	59680	20	112	137.8636364	12.32369371	0.795739001	0.027358331	-	9870	grey	-0.174787214	FALSE	AREL1
Region_5493	chr14	74713144	74738620	25476	10	50	79.62121212	8.834835163	0.812322236	0.03041927	+	51077	grey	0.0265321	FALSE	FCF1
Region_5494	chr14	74763316	74859435	96119	19	104	142.2272727	12.59767433	0.92618946	0.02502959	+	56252	grey	-0.325130317	FALSE	YLPM1
Region_5495	chr14	74852871	74876154	23283	4	19	30.07575758	5.030095904	0.488201717	0.044899892	-	283571	grey	-0.050888081	FALSE	PROX2
Region_5496	chr14	74881891	74903743	21852	13	84	108.2575758	12.71015416	0.418705218	0.034265866	+	1743	grey	-0.140345806	FALSE	DLST
Region_5497	chr14	74903951	74923302	19351	6	26	41.78787879	7.019785558	0.686305074	0.0527135	-	83694	grey	0.185326186	FALSE	RPS6KL1
Region_5498	chr14	75002921	75012366	9445	6	36	56.83333333	7.355967401	0.803096062	0.032453723	+	8892	grey	-0.052276014	FALSE	EIF2B2
Region_5499	chr14	75013769	75051532	37763	11	65	85.83333333	10.5153124	0.79148779	0.019174098	-	27030	grey	-0.167595164	FALSE	MLH3

Region_5500	chr14	75053237	75069483	16246	5	19	32.3030303	6.196698121	0.573056312	0.073251234	-	97	grey	0.291167993	FALSE	ACYP1
Region_5501	chr14	75064109	75119502	55393	21	134	159.6666667	12.45587082	0.914417307	0.018487746	+	79696	grey	0.066054092	FALSE	ZC2HC1C
Region_5502	chr14	75079353	75127344	47991	10	60	76.48484848	8.873889832	0.968808385	0.02389426	-	91754	grey	-0.055008699	FALSE	NEK9
Region_5503	chr14	75131469	75176612	45143	5	26	38.46969697	4.810948082	0.976405287	0.038595446	-	10972	grey	0.004799831	FALSE	TMED10
Region_5504	chr14	75278826	75282230	3404	3	15	25.07575758	5.378906397	0.287963838	0.047415931	+	2353	grey	-0.319613118	FALSE	FOS
Region_5505	chr14	75427716	75474111	46395	26	162	188.5151515	14.52132906	0.900265857	0.022509439	+	122953	grey	-0.077838551	FALSE	JDP2
Region_5506	chr14	75522455	75546993	24538	7	38	53.86363636	6.788874074	0.926786857	0.06647263	+	10538	grey	-0.151417097	FALSE	BATF
Region_5507	chr14	75574888	75579588	4700	3	13	20.78787879	4.662502304	0.119250706	0.069091104	-	102724153	grey	0.219325443	FALSE	FLVCR2-AS1
Region_5508	chr14	75578620	75663164	84544	17	117	138.2575758	10.06793938	0.636824028	0.030208121	+	55640	grey	0.070132998	FALSE	FLVCR2
Region_5509	chr14	75579584	75955079	375495	67	469	525.2878788	24.61504553	0.876752429	0.010007371	+	23093	grey	0.114943635	FALSE	TTLL5
Region_5510	chr14	75902136	76084585	182449	40	281	313.8181818	18.6489093	0.86498144	0.015941986	+	112752	grey	0.073457696	FALSE	IFT43
Region_5511	chr14	75958097	75983011	24914	4	12	24.15151515	6.16252302	0.494731636	0.049526236	-	7043	grey	0.220962106	FALSE	TGFB3
Region_5512	chr1	76151916	76254342	102426	13	69	99.6363636	10.8452561	0.81875068	0.03695212	+	55668	grey	0.108118559	FALSE	GPATCH2L

	4						4	3	2	1						
Region_5513	chr14	76280943	76310066	29123	4	21	33.12121212	5.193235694	0.932782001	0.045730992	-	105370575	grey	-0.046240275	FALSE	LOC105370575
Region_5514	chr14	76310712	76501837	191125	51	369	411.1363636	18.75828954	0.841841797	0.020143733	+	2103	grey	0.022382755	FALSE	ESRRB
Region_5515	chr14	76710658	76761388	50730	12	45	66.62121212	9.814847703	0.902406939	0.036502428	-	105370577	grey	0.10051098	FALSE	LOC105370577
Region_5516	chr14	76761468	76783015	21547	8	41	60.34848485	7.800379526	0.814721151	0.029975281	+	22846	grey	0.03302419	FALSE	VASH1
Region_5517	chr14	76786009	76826246	40237	12	73	96.15151515	9.628870729	0.773220418	0.022332045	-	23357	grey	0.214836034	FALSE	ANGEL1
Region_5518	chr14	76826372	76870304	43932	14	85	111.1060606	11.48527993	0.785104931	0.030670828	+	145497	grey	-0.114413767	FALSE	LRRC74A
Region_5519	chr14	77024543	77028708	4165	14	61	87.90909091	11.50481808	0.406393034	0.035919141	-	64207	grey	-0.19252535	FALSE	IRF2BPL
Region_5520	chr14	77031562	77038743	7181	4	18	26.84848485	4.830169369	0.440943107	0.068492804	-	105370579	grey	0.21120847	FALSE	LOC105370579
Region_5521	chr14	77041064	77069503	28439	12	75	100.530303	9.820261154	0.865618171	0.025679475	+	283575	grey	0.378781114	FALSE	LINC02288
Region_5522	chr14	77098126	77117287	19161	4	25	35.71212121	5.734551559	0.969467617	0.039790614	+	85457	grey	0.07313036	FALSE	CIPC
Region_5523	chr14	77098311	77259495	161184	69	400	495.1969697	31.04697171	0.700430643	0.016553403	+	57156	grey	-0.16386058	FALSE	TMEM63C
Region_5524	chr14	77131270	77142734	11464	12	74	96.86363636	11.60488805	0.124765337	0.02469212	-	283576	grey	-0.259023545	FALSE	ZDHHC22

Region_5525	chr14	77274956	77320883	45927	9	60	81.93939394	8.285072374	0.863802682	0.017423472	-	29954	grey	-0.055873886	FALSE	POMT2
Region_5526	chr14	77320996	77331597	10601	3	17	27.15151515	5.435615949	0.539116233	0.070376771	+	2954	grey	-0.018777714	FALSE	GSTZ1
Region_5527	chr14	77335029	77377094	42065	13	75	99.24242424	11.42879037	0.774785646	0.031587248	-	283578	grey	-0.262884764	FALSE	TMED8
Region_5528	chr14	77376689	77392163	15474	15	92	115.6818182	11.85574718	0.673134569	0.027732238	+	161394	grey	-0.387928599	FALSE	SAMD15
Region_5529	chr14	77394021	77423523	29502	11	52	73.95454545	10.63004714	0.936627749	0.0296833	-	122945	grey	0.100771588	FALSE	NOXRED1
Region_5530	chr14	77426675	77457952	31277	8	48	63.45454545	7.027816858	0.815968544	0.038488551	-	63894	grey	-0.269032293	FALSE	VIPAS39
Region_5531	chr14	77457870	77469472	11602	5	33	45.25757576	6.580415245	0.585884614	0.022396596	+	10598	grey	-0.22822203	FALSE	AHSA1
Region_5532	chr14	77474394	77498816	24422	28	175	217.6666667	14.91239374	0.868105357	0.023078714	-	145501	grey	0.09746691	FALSE	ISM2
Region_5533	chr14	77505997	77616637	110640	29	207	246.7727273	18.33180964	0.715804042	0.033048901	-	9517	grey	-0.281521405	FALSE	SPTLC2
Region_5534	chr14	77672404	77708023	35619	12	48	79.37878788	9.082129793	0.884683473	0.030173776	-	8846	grey	0.063783469	FALSE	ALKBH1
Region_5535	chr14	77708071	77761104	53033	22	131	158.530303	12.29286184	0.840726897	0.024502705	+	81892	grey	-0.07925369	FALSE	SLIRP
Region_5536	chr14	77717599	77761207	43608	19	119	144.030303	11.91117084	0.827009113	0.0262978	-	22938	grey	-0.080295083	FALSE	SNW1
Region_5537	chr1	77760834	77769742	8908	3	13	23.8939393	5.27148727	0.62237360	0.04922888	+	283579	grey	-0.02660957	FALSE	C14orf178

	4						9	5	9	3				6		
Region_5538	chr14	77800109	77935014	134905	31	201	226.7272727	12.25817837	0.860905937	0.026079636	+	57143	grey	0.113717595	FALSE	ADCK1
Region_5539	chr14	78170373	79868291	1697918	172	1400	1474.393939	46.41646882	0.825324919	0.017770268	+	9369	grey	-0.258363379	FALSE	NRXN3
Region_5540	chr14	79611418	79633311	21893	3	16	29.65151515	5.22565395	0.812419629	0.116460607	-	105370583	grey	-0.03787646	FALSE	LOC105370583
Region_5541	chr14	79893080	79974169	81089	4	18	31.24242424	5.732661098	0.794576336	0.085768687	-	112267892	grey	-0.085357766	FALSE	LOC112267892
Region_5542	chr14	80197526	80387757	190231	26	196	235.2272727	15.40608816	0.782200311	0.030098722	-	1734	grey	0.243265244	FALSE	DIO2
Region_5543	chr14	80211419	80455469	244050	29	222	261.7121212	16.92041566	0.791930319	0.029363722	+	100628307	grey	0.249455788	FALSE	DIO2-AS1
Region_5544	chr14	80476983	80959517	482534	34	258	299.2878788	17.55673454	0.926955294	0.014618848	-	145508	grey	-0.220536482	FALSE	CEP128
Region_5545	chr14	80954989	81146306	191317	9	45	66.48484848	8.700561298	0.847583199	0.039940741	+	7253	grey	-0.020464771	FALSE	TSHR
Region_5546	chr14	81012099	81170429	158330	9	46	67.28787879	9.080435692	0.774186553	0.045818437	-	101928462	grey	0.093721264	FALSE	LOC101928462
Region_5547	chr14	81175452	81221377	45925	3	17	27.22727273	5.420177274	0.967419533	0.033801388	-	2957	grey	0.319386443	FALSE	GTF2A1
Region_5548	chr14	81260652	81436465	175813	22	159	191.0757576	13.58541141	0.789760213	0.017870103	-	85439	grey	-0.152054595	FALSE	STON2
Region_5549	chr14	81471547	81533853	62306	8	48	65.74242424	9.384620161	0.851390667	0.020515923	-	6400	grey	0.113061636	FALSE	SEL1L

Region_5550	chr14	81741002	82030349	289347	23	146	179.6818182	12.42969037	0.809336062	0.03858809	+	107984704	grey	-0.007649019	FALSE	LOC107984704
Region_5551	chr14	82642600	82746664	104064	8	51	76.01515152	9.242864065	0.884905228	0.046527889	+	101928599	grey	0.000940505	FALSE	LINC02301
Region_5552	chr14	85516531	85654428	137897	17	107	141.9545455	10.99150976	0.668202026	0.028319206	+	23768	grey	0.041618764	FALSE	FLRT2
Region_5553	chr14	85517967	85531585	13618	4	23	33.36363636	5.290445273	0.365471755	0.055072803	-	100506731	grey	-0.064983457	FALSE	FLRT2-AS1
Region_5554	chr14	85911438	86161539	250101	25	171	203.5151515	12.24272773	0.912229798	0.028621974	+	101928767	grey	0.289835411	FALSE	LINC02328
Region_5555	chr14	86332376	86401285	68909	9	52	74.59090909	9.504617943	0.865151931	0.049110025	+	105370607	grey	0.129366719	FALSE	LINC02309
Region_5556	chr14	87353070	87573022	219952	14	87	122.7878788	12.33446098	0.838945401	0.041238113	-	105370610	grey	0.066050728	FALSE	LINC02296
Region_5557	chr14	87837820	87993665	155845	11	58	76.56060606	7.82919347	0.864120574	0.03182717	-	2581	grey	-0.065587302	FALSE	GALC
Region_5558	chr14	88024519	88098025	73506	5	23	33.22727273	5.568580691	0.947224846	0.04808466	+	283587	grey	-0.352094241	FALSE	HISLA
Region_5559	chr14	88180103	88326907	146804	27	187	234.8030303	15.2267171	0.76134177	0.025070197	-	54207	grey	-0.024332326	FALSE	KCNK10
Region_5560	chr14	88384924	88470350	85426	9	60	79.03030303	7.834773959	0.935497288	0.029591502	+	55812	grey	0.086347922	FALSE	SPATA7
Region_5561	chr14	88465778	88555007	89229	12	76	102.9242424	11.18283108	0.934832008	0.025416147	-	11099	grey	0.254812018	FALSE	PTPN21
Region_5562	chr1	88562970	88627596	64626	6	38	50.8636363	7.14010636	0.95403270	0.02911397	+	79882	grey	0.024125268	FALSE	ZC3H14

	4						6	3	4	9						
Region_5563	chr14	88612431	88792953	180522	25	160	196.9393939	14.83227125	0.682789568	0.023277518	-	161436	grey	-0.204360674	FALSE	EML5
Region_5564	chr14	88824153	88881078	56925	6	31	45.46969697	7.230745673	0.801195752	0.030065652	+	123016	grey	-0.290803858	FALSE	TTC8
Region_5565	chr14	89124871	89954659	829788	181	1345	1456.318182	50.03911058	0.846377794	0.009568742	-	1112	grey	0.24915691	FALSE	FOXN3
Region_5566	chr14	89628921	89642671	13750	4	16	29.6969697	4.930188628	0.82339258	0.105865794	-	105370616	grey	-0.440163895	FALSE	LOC105370616
Region_5567	chr14	89794669	89954777	160108	38	272	321.6515152	20.04110578	0.886864763	0.017386581	-	90141	grey	0.305302759	FALSE	EFCAB11
Region_5568	chr14	89954939	90044764	89825	13	92	111.7121212	10.51411528	0.638836612	0.025067734	+	55775	grey	0.232859132	FALSE	TDP1
Region_5569	chr14	90061994	90185853	123859	38	280	327.4393939	18.45754279	0.83061999	0.019437989	+	56659	grey	0.046980844	FALSE	KCNK13
Region_5570	chr14	90256527	90275429	18902	3	10	19.22727273	4.626995884	0.779553653	0.144270906	+	5700	grey	-0.169787905	FALSE	PSMC1
Region_5571	chr14	90267860	90331969	64109	16	120	148.1515152	12.30664914	0.897562855	0.024169941	-	55051	grey	0.197675136	FALSE	NRDE2
Region_5572	chr14	90396502	90408268	11766	6	33	49.84848485	7.821552913	0.330636595	0.019584105	+	801	grey	-0.318847756	FALSE	CALM1
Region_5573	chr14	90453571	90490352	36781	9	43	62.07575758	7.706761479	0.937354296	0.033275949	+	400238	grey	-0.088354806	FALSE	LINC00642
Region_5574	chr14	90524564	90816479	291915	89	688	766.7575758	33.40707461	0.887954143	0.012987611	-	145567	grey	-0.195278478	FALSE	TTC7B

Region_5575	chr14	90820064	90828202	8138	3	16	27.1969697	5.344212389	0.856315577	0.084436864	-	105370623	grey	0.097604898	FALSE	LINC02321
Region_5576	chr14	90847861	91060641	212780	58	418	472.0454545	23.82721897	0.866269326	0.009653607	-	9252	grey	-0.052799188	FALSE	RPS6KA5
Region_5577	chr14	91060333	91225632	165299	43	298	343.8636364	20.67245389	0.924318917	0.014988454	+	80017	grey	0.022044893	FALSE	DGLUCY
Region_5578	chr14	91232532	91253925	21393	15	89	115.7878788	10.41229325	0.549068502	0.031874728	-	8111	grey	-0.416775946	FALSE	GPR68
Region_5579	chr14	91271323	91417820	146497	61	425	477.5454545	23.89025023	0.80477771	0.01697946	-	440193	grey	-0.167998841	FALSE	CCDC88C
Region_5580	chr14	91378641	91417844	39203	7	28	44.42424242	7.321329442	0.36207127	0.039178772	-	105370624	grey	0.016986472	FALSE	LOC105370624
Region_5581	chr14	91457508	91510554	53046	7	44	59.28787879	7.46636592	0.553199675	0.023831155	-	55671	grey	-0.152968674	FALSE	PPP4R3A
Region_5582	chr14	91869411	91947987	78576	24	168	211.8484848	17.08732542	0.889287225	0.021699788	-	10516	grey	-0.068139155	FALSE	FBLN5
Region_5583	chr14	91965991	92040896	74905	19	114	137.8333333	10.28005288	0.831153939	0.018811578	-	9321	grey	0.199544889	FALSE	TRIP11
Region_5584	chr14	92049611	92106621	57010	11	56	74.12121212	7.786606156	0.779155833	0.037966409	-	4287	grey	0.038212715	FALSE	ATXN3
Region_5585	chr14	92121969	92172145	50176	4	23	34.72727273	5.931780001	0.971645036	0.027267897	+	53981	grey	0.095222421	FALSE	CPSF2
Region_5586	chr14	92322581	92501481	178900	57	409	457.9545455	21.28374737	0.806434517	0.018422552	+	123041	grey	0.157763198	FALSE	SLC24A4
Region_5587	chr1	92513781	92688994	175213	70	470	515.393939	23.9620718	0.79427174	0.01723580	+	79890	grey	0.266975806	FALSE	RIN3

	4						4	2	3	8						
Region_5588	chr14	92703807	92748679	44872	16	104	122.7878788	10.90026838	0.903971534	0.019483751	-	5641	grey	0.241989807	FALSE	LGMN
Region_5589	chr14	92794305	92839947	45642	9	57	77.5	7.805816372	0.97017278	0.02069141	+	9950	grey	-0.148489021	FALSE	GOLGA5
Region_5590	chr14	94462717	94479689	16972	7	37	52.93939394	6.584080539	0.758083818	0.069106892	-	327657	grey	0.124574843	FALSE	SERPINA9
Region_5591	chr14	94487274	94517844	30570	6	37	51.46969697	7.93477196	0.865459502	0.063783958	-	145264	grey	-0.20915137	FALSE	SERPINA12
Region_5592	chr14	94561442	94593118	31676	6	41	56.89393939	6.124675911	0.838706615	0.075748285	+	5104	grey	-0.436443182	FALSE	SERPINA5
Region_5593	chr14	94592058	94624646	32588	6	25	41.87878788	6.439099678	0.859168959	0.061551812	+	12	grey	-0.200714292	FALSE	SERPINA3
Region_5594	chr14	94640725	94646994	6269	4	16	27.65151515	5.133577023	0.899923004	0.076263121	+	388007	grey	-0.247575731	FALSE	SERPINA13P
Region_5595	chr14	95086228	95158010	71782	29	190	232.3787879	18.6430149	0.559487296	0.016569982	-	23405	grey	0.216972909	FALSE	DICER1
Region_5596	chr14	95157645	95181475	23830	5	35	50.53030303	6.91758005	0.600312478	0.045328553	+	400242	grey	-0.256419252	FALSE	DICER1-AS1
Region_5597	chr14	95181940	95319908	137968	54	386	438.3333333	25.20276745	0.827692503	0.015479653	-	79789	grey	0.067343978	FALSE	CLMN
Region_5598	chr14	95319483	95335757	16274	5	30	45.01515152	7.345312318	0.769020469	0.027308468	+	101929080	grey	0.068894651	FALSE	LINC02292
Region_5599	chr14	95407266	95516650	109384	38	296	335.1666667	16.94296617	0.842852006	0.021077981	-	161176	grey	-0.179579817	FALSE	SYNE3

Region_5600	chr14	95521958	95534889	12931	6	58	71.34848485	7.030685324	0.199510685	0.028691314	-	283596	grey	-0.240129702	FALSE	SNHG10
Region_5601	chr14	95522136	95534043	11907	4	37	49.07575758	6.381006928	0.298921675	0.043525382	-	112268122	grey	-0.231935992	FALSE	
Region_5602	chr14	95533503	95544724	11221	15	94	113.1363636	12.98624878	0.32253126	0.020886934	+	51218	grey	0.124608724	FALSE	GLRX5
Region_5603	chr14	95650498	95679833	29335	9	62	78.57575758	8.31321581	0.710686997	0.057050106	+	27004	grey	0.155563994	FALSE	TCL6
Region_5604	chr14	95663256	95692630	29374	10	62	76.12121212	7.798451822	0.886253203	0.038192106	+	9623	grey	-0.013815376	FALSE	TCL1B
Region_5605	chr14	95711747	95757656	45909	7	39	58	7.470969456	0.809533964	0.063376934	+	107984703	grey	0.176266777	FALSE	LOC107984703
Region_5606	chr14	95866587	95925663	59076	18	119	151.8030303	14.42990452	0.542778992	0.028913571	+	100507043	grey	0.115721723	FALSE	TUNAR
Region_5607	chr14	96039362	96093971	54609	12	80	109.9848485	10.87621601	0.593800303	0.040442307	+	56967	grey	0.02485675	FALSE	C14orf132
Region_5608	chr14	96204679	96244166	39487	13	89	113.9090909	11.80837678	0.796811089	0.043343384	+	624	grey	0.037009344	FALSE	BDKRB2
Region_5609	chr14	96204844	96268967	64123	17	120	147.6515152	13.68827957	0.824344196	0.035570747	+	623	grey	0.113615047	FALSE	BDKRB1
Region_5610	chr14	96279195	96363341	84146	19	139	162.5757576	13.5760656	0.903638661	0.016494805	-	55102	grey	-0.114383088	FALSE	ATG2B
Region_5611	chr14	96363452	96387288	23836	7	45	63.36363636	9.859999007	0.716834303	0.02154425	+	51527	grey	0.251627247	FALSE	GSKIP
Region_5612	chr1	96392128	96489427	97299	20	128	157.833333	14.1220867	0.79422719	0.02943679	+	122481	grey	-0.21122986	FALSE	AK7

	4						3	2	4	8				2		
Region_5613	chr14	96501433	96567116	65683	21	148	180.7878788	13.57318074	0.849337267	0.017378799	+	10914	grey	0.051413402	FALSE	PAPOLA
Region_5614	chr14	96738021	96793084	55063	14	92	111.3030303	8.768772736	0.618660308	0.039744519	+	105370646	grey	0.182960487	FALSE	LINC02299
Region_5615	chr14	96797304	96954846	157542	32	236	266.5	16.31540094	0.929172656	0.020069575	+	7443	grey	0.082913086	FALSE	VRK1
Region_5616	chr14	96932483	96946074	13591	7	45	60.54545455	7.500116549	0.965822431	0.023472424	+	145249	grey	0.038366321	FALSE	LINC00618
Region_5617	chr14	97427071	97582146	155075	29	218	249.2575758	16.35501392	0.893152362	0.02469756	+	101929241	grey	-0.292196888	FALSE	LINC02325
Region_5618	chr14	97632647	97686658	54011	13	90	110.9242424	8.762350591	0.873527587	0.032884237	-	100129345	grey	0.16269771	FALSE	LINC02291
Region_5619	chr14	97662638	97688600	25962	5	26	40.83333333	6.108212211	0.902898151	0.064276147	+	105370650	grey	0.202278185	FALSE	LOC105370650
Region_5620	chr14	97749264	97763989	14725	3	15	26.57575758	4.742802177	0.950709193	0.050058993	-	100132612	grey	-0.049068102	FALSE	LINC02312
Region_5621	chr14	97924637	97978124	53487	3	20	29.03030303	4.857887154	0.932689648	0.052855625	-	388011	grey	0.027938105	FALSE	LINC01550
Region_5622	chr14	98068161	98093182	25021	4	20	32.93939394	5.801535043	0.8024931	0.088758578	-	105370654	grey	0.055746657	FALSE	LOC105370654
Region_5623	chr14	98068240	98205143	136903	21	142	179.4545455	14.59734625	0.769051869	0.037761331	-	105370655	grey	0.270037415	FALSE	LOC105370655
Region_5624	chr14	98135986	98163151	27165	5	18	31.96969697	6.351183286	0.746983222	0.074773267	+	105370652	grey	0.035059061	FALSE	LINC02295

Region_5625	chr14	99169287	99272197	102910	32	207	246.8484848	16.64862427	0.574662134	0.038029696	-	64919	grey	0.013713967	FALSE	BCL11B
Region_5626	chr14	99397748	99480889	83141	30	225	260.7121212	16.15669916	0.89620816	0.015874724	-	84193	grey	-0.201685721	FALSE	SETD3
Region_5627	chr14	99481169	99535044	53875	25	163	202.8181818	13.2710316	0.798113833	0.033286304	+	8812	grey	-0.45037374	FALSE	CCNK
Region_5628	chr14	99500190	99604207	104017	53	337	409.6060606	28.59041194	0.898940972	0.022380751	-	317762	grey	-0.35614348	FALSE	CCDC85C
Region_5629	chr14	99604517	99680569	76052	54	335	387	22.31246633	0.70834677	0.021646469	+	84439	grey	-0.072858392	FALSE	HHIPL1
Region_5630	chr14	99684298	99727301	43003	26	157	192.3181818	13.23882297	0.727103367	0.030908574	+	10858	grey	-0.400867558	FALSE	CYP46A1
Region_5631	chr14	99737693	99942060	204367	84	669	722.8787879	27.0091239	0.828460265	0.013221978	+	2009	grey	0.091343872	FALSE	EML1
Region_5632	chr14	99971449	100144236	172787	99	775	839.3636364	32.10494156	0.847270665	0.012895118	+	51466	grey	0.35901258	FALSE	EVL
Region_5633	chr14	100143957	100159645	15688	24	132	178.7121212	16.15574692	0.763442428	0.027770215	-	123099	grey	0.022511852	FALSE	DEGS2
Region_5634	chr14	100239144	100282788	43644	24	144	173.8939394	17.02679227	0.538188903	0.022363316	+	7528	grey	-0.418745906	FALSE	YY1
Region_5635	chr14	100279959	100306547	26588	15	81	111.8939394	11.47455887	0.696391532	0.028896175	-	123096	grey	0.177925435	FALSE	SLC25A29
Region_5636	chr14	100333790	100376805	43015	17	87	116.8636364	11.6867925	0.594253523	0.039713498	-	7453	grey	0.28179853	FALSE	WARS1
Region_5637	chr1	10037641	10053030	153885	56	453	502.863636	25.3522181	0.91168541	0.01310266	+	79446	grey	0.08376676	FALSE	WDR25

	4	8	3				4	5	6	3						
Region_5638	chr14	100537147	100587417	50270	51	263	328.4848485	27.29792465	0.638544404	0.01799741	-	57596	grey	0.02965877	FALSE	BEGAIN
Region_5639	chr14	100657250	100672779	15529	10	59	79.51515152	11.04848395	0.825678664	0.044837502	+	283601	grey	0.195795818	FALSE	LINC00523
Region_5640	chr14	100725705	100738224	12519	18	99	129.0909091	10.68820669	0.437671368	0.033497336	+	8788	grey	0.314718803	FALSE	DLK1
Region_5641	chr14	100779410	100861031	81621	63	427	488.9545455	27.32788478	0.791227977	0.020574964	+	55384	grey	0.304823861	FALSE	MEG3
Region_5642	chr14	100836269	101038859	202590	106	755	847.0909091	42.90875015	0.822194233	0.012383661	+	79104	grey	0.08784453	FALSE	MEG8
Region_5643	chr14	100879753	100903722	23969	18	105	130.8939394	10.54386995	0.93525856	0.030328395	-	388015	grey	-0.001641708	FALSE	RTL1
Region_5644	chr14	101042908	101056244	13336	9	63	85.93939394	10.3505909	0.90432278	0.032649825	+	378881	grey	0.046565034	FALSE	MIR381HG
Region_5645	chr14	101055035	101072937	17902	9	56	76.39393939	9.618697797	0.937614578	0.038312379	+	100507257	grey	0.116576279	FALSE	MEG9
Region_5646	chr14	101071978	101077910	5932	12	69	84.81818182	9.048432233	0.586275044	0.068712201	-	105370670	grey	0.147284497	FALSE	LOC105370670
Region_5647	chr14	101120840	101123545	2705	3	12	21.6969697	5.397918964	0.344172774	0.058392244	+	101929422	grey	-0.048324524	FALSE	LINC02285
Region_5648	chr14	101552221	101620355	68134	35	207	252.7272727	17.16306002	0.729847236	0.020597246	-	64150	grey	-0.3358084	FALSE	DIO3OS
Region_5649	chr14	101634454	101731108	96654	27	182	217.6515152	17.84419097	0.893006806	0.022614448	-	105370676	grey	-0.150287724	FALSE	LINC02320

Region_5650	chr14	101761709	101927989	166280	80	536	659.6363636	38.94241759	0.86938097	0.011149305	+	5527	grey	-0.100596178	FALSE	PPP2R5C
Region_5651	chr14	101761862	101836701	74839	22	151	180.9242424	12.74762193	0.668529208	0.022065811	+	107984659	grey	-0.316254627	FALSE	
Region_5652	chr14	101964573	102056443	91870	90	705	787.030303	35.34575144	0.914050457	0.009069937	+	1778	grey	0.063567886	FALSE	DYNC1H1
Region_5653	chr14	102036315	102066228	29913	25	166	204.3484848	16.02545324	0.909192937	0.016632418	-	107984661	grey	0.045120901	FALSE	LOC107984661
Region_5654	chr14	102080742	102139699	58957	30	182	214.2121212	15.6160816	0.56941867	0.012466032	-	3320	grey	-0.236769299	FALSE	HSP90AA1
Region_5655	chr14	102139503	102224847	85344	27	181	218.1969697	16.64490654	0.835764779	0.022236297	+	91833	grey	-0.134220177	FALSE	WDR20
Region_5656	chr14	102224500	102305190	80690	51	326	375.8333333	24.36175018	0.860137288	0.017732411	-	5891	grey	-0.293263348	FALSE	MOK
Region_5657	chr14	102317377	102342702	25325	12	80	97.92424242	8.841006915	0.720164694	0.022415308	+	55778	grey	0.124913089	FALSE	ZNF839
Region_5658	chr14	102341102	102362916	21814	16	114	135.1818182	11.31222496	0.864527284	0.028616646	-	51550	grey	-0.14286529	FALSE	CINP
Region_5659	chr14	102362941	102502477	139536	90	632	705	33.77755286	0.92757338	0.009392387	+	9895	grey	-0.164792904	FALSE	TECPR2
Region_5660	chr14	102501767	102509799	8032	19	93	130.0606061	14.66956954	0.831889624	0.034979473	-	122416	grey	-0.333574197	FALSE	ANKRD9
Region_5661	chr14	102545254	102555826	10572	16	73	102.7272727	11.55394765	0.649081444	0.034730894	+	105370681	grey	0.257849911	FALSE	LINC02323
Region_5662	chr1	10259264	10273056	137912	71	422	474.560606	25.7150831	0.83763711	0.01239111	+	23186	grey	0.112999811	FALSE	RCOR1

	4	9	1				1	5		4						
Region_5663	chr14	102777449	102911500	134051	85	636	726.0454545	36.23021766	0.871937356	0.019247422	+	7187	grey	0.370663098	FALSE	TRAF3
Region_5664	chr14	102922663	102933596	10933	20	111	143.4393939	16.04429329	0.328527543	0.030807961	+	81693	grey	-0.122107323	FALSE	AMN
Region_5665	chr14	102932380	103057549	125169	129	921	1022.439394	42.6723217	0.86893302	0.012302356	-	9578	grey	-0.179084228	FALSE	CDC42BPB
Region_5666	chr14	103084210	103090027	5817	5	18	25.46969697	4.794932251	0.747912584	0.079979365	+	107984640	grey	-0.054290345	FALSE	LBHD2
Region_5667	chr14	103094725	103110559	15834	20	110	142.5454545	16.11896334	0.526176854	0.035966879	+	91828	grey	0.018437768	FALSE	EXOC3L4
Region_5668	chr14	103121476	103137439	15963	9	46	72.06060606	8.580620002	0.537526342	0.025162348	+	7127	grey	-0.104673317	FALSE	TNFAIP2
Region_5669	chr14	103333544	103345025	11481	9	46	65.22727273	8.731543972	0.345473093	0.029482248	+	1983	grey	0.062471321	FALSE	EIF5
Region_5670	chr14	103385377	103503831	118454	70	480	562.3030303	26.88606714	0.859286888	0.011671966	+	4140	grey	0.093574906	FALSE	MARK3
Region_5671	chr14	103529196	103537073	7877	5	24	37.8030303	5.844707526	0.713014322	0.054408987	+	115708	grey	-0.157900838	FALSE	TRMT61A
Region_5672	chr14	103556545	103562657	6112	7	39	55.83333333	8.941834837	0.710502645	0.041031862	-	9529	grey	-0.039150363	FALSE	BAG5
Region_5673	chr14	103561896	103714249	152353	143	1046	1139.030303	40.54246385	0.846618404	0.012121191	+	3831	grey	0.019825764	FALSE	KLC1
Region_5674	chr14	103562960	103607523	44563	67	461	525.0606061	24.73420477	0.850442815	0.01585119	+	84334	grey	0.063985882	FALSE	COA8

Region_5675	chr14	103697609	103715504	17895	20	126	157.1515152	14.42566348	0.94093575	0.027851597	-	7517	grey	-0.176006205	FALSE	XRCC3
Region_5676	chr14	103715730	103733668	17938	9	35	59.83333333	8.601580058	0.732362341	0.044753918	+	79038	grey	-0.047611725	FALSE	ZFYVE21
Region_5677	chr14	103733195	103847575	114380	65	443	525.8787879	30.51028334	0.903741863	0.01557962	-	23368	grey	0.043929823	FALSE	PPP1R13B
Region_5678	chr14	103847721	103858049	10328	9	44	63.68181818	7.23487088	0.887637481	0.031924262	+	145216	grey	-0.021236366	FALSE	PPP1R13B-DT
Region_5679	chr14	103854366	103880111	25745	21	89	123.2121212	13.0943501	0.733470669	0.023137979	-	105370690	grey	-0.137003565	FALSE	
Region_5680	chr14	103873148	103880381	7233	4	13	23.21212121	5.121643821	0.731331169	0.034496018	+	105370689	grey	-0.009352055	FALSE	LOC105370689
Region_5681	chr14	103912288	103928269	15981	12	60	83.56060606	9.606294071	0.830877389	0.032463316	-	9556	grey	-0.096861548	FALSE	ATP5MJ
Region_5682	chr14	103928456	104052667	124211	61	435	515.7878788	26.66516896	0.888782696	0.014028438	+	122402	grey	0.078023027	FALSE	TDRD9
Region_5683	chr14	104085686	104115582	29896	38	204	253.8030303	22.23525938	0.81455058	0.028821906	+	374569	grey	-0.310029821	FALSE	ASPG
Region_5684	chr14	104134783	104137898	3115	9	36	54.01515152	8.487986849	0.016182405	0.021634418	-	105370708	grey	0.41789028	FALSE	KIF26A-DT
Region_5685	chr14	104136454	104180894	44440	76	424	503.8636364	35.91180923	0.817105879	0.014769257	+	26153	grey	-0.342607483	FALSE	KIF26A
Region_5686	chr14	104223584	104288069	64485	43	273	310.5	22.50282034	0.885921279	0.023106737	+	145195	grey	-0.008387192	FALSE	LINC02691
Region_5687	chr1	10466112	10466555	4438	6	19	41.0151515	7.84168824	0.88569882	0.07078957	-	10192963	grey	-0.02165647	FALSE	LINC02280

	4	0	8				2	9	1	8		4		8		
Region_5688	chr14	104681146	104722535	41389	63	323	396.1212121	32.10009113	0.770930516	0.023116475	+	64423	grey	-0.425345764	FALSE	INF2
Region_5689	chr14	104724229	104747325	23096	32	186	228.3333333	18.5665815	0.768542353	0.023126239	+	122622	grey	-0.150303641	FALSE	ADSS1
Region_5690	chr14	104753147	104768494	15347	10	65	87.96969697	9.33310023	0.855829101	0.038203817	+	10572	grey	0.147424244	FALSE	SIVA1
Region_5691	chr14	104769349	104795751	26402	31	170	212.6060606	20.4178495	0.890439421	0.02313616	-	207	grey	-0.120900513	FALSE	AKT1
Region_5692	chr14	104800596	104804712	4116	11	62	86.77272727	12.38708441	0.074786187	0.025455294	+	100128927	grey	-0.258579014	FALSE	ZBTB42
Region_5693	chr14	104821201	104823718	2517	4	17	29.45454545	5.002097462	0.733365391	0.091661358	+	196872	grey	0.132255931	FALSE	LINC00638
Region_5694	chr14	104865268	104896747	31479	30	141	187.030303	17.98800429	0.814923015	0.024511455	+	283638	grey	0.176141353	FALSE	CEP170B
Region_5695	chr14	104924713	104937761	13048	11	54	74.24242424	8.959843072	0.736520667	0.045412355	+	122618	grey	-0.131080859	FALSE	PLD4
Region_5696	chr14	104937244	104978374	41130	31	176	214.2424242	15.81242025	0.755286211	0.02817716	-	113146	grey	0.202012562	FALSE	AHNAK2
Region_5697	chr14	104985775	105010482	24707	16	105	129.2878788	13.38973792	0.858955584	0.025689287	+	122616	grey	-0.129583403	FALSE	CLBA1
Region_5698	chr14	105009573	105021083	11510	21	143	173.3939394	14.94901982	0.773986089	0.028598599	-	55038	grey	0.013437033	FALSE	CDCA4
Region_5699	chr14	105049395	105065445	16050	24	153	186.4393939	16.44864155	0.704749666	0.027929754	-	29933	grey	0.215359411	FALSE	GPR132

Region_5700	chr14	105140982	105181194	40212	69	382	448.6212121	31.99940632	0.650206608	0.014747287	-	3714	grey	-0.005571018	FALSE	JAG2
Region_5701	chr14	105172938	105181323	8385	12	53	72.89393939	10.09208187	0.510646668	0.04974353	-	256281	grey	-0.167002585	FALSE	NUDT14
Region_5702	chr14	105209286	105315589	106303	166	1155	1260.909091	54.11319398	0.786691214	0.008592116	-	2972	grey	-0.160201282	FALSE	BRF1
Region_5703	chr14	105248533	105251093	2560	11	44	68.07575758	12.46201688	0.397269438	0.055114415	+	90135	grey	0.090708683	FALSE	BTBD6
Region_5704	chr14	105300563	105398147	97584	134	890	987.1212121	49.62738548	0.736229672	0.011069695	+	23241	grey	-0.119279336	FALSE	PACS2
Region_5705	chr14	105398538	105413859	15321	23	111	133.3939394	12.25483112	0.456154972	0.03435446	+	647310	grey	0.088213317	FALSE	TEX22
Region_5706	chr14	105398579	105470729	72150	108	655	739.6515152	37.00789622	0.719212783	0.012755592	+	9112	grey	0.23601824	FALSE	MTA1
Region_5707	chr14	105472962	105480162	7200	10	25	46.90909091	8.734947092	0.179405681	0.08441292	+	1397	grey	-0.050384104	FALSE	CRIP2
Region_5708	chr14	105486317	105491659	5342	32	160	194.9545455	15.96383588	0.083886542	0.014924004	+	1396	grey	0.07006284	FALSE	CRIP1
Region_5709	chr14	105487199	105499575	12376	29	153	187.6666667	16.07897177	0.209392291	0.015669518	+	283643	grey	0.171063373	FALSE	TEDC1
Region_5710	chr14	105526583	105530202	3619	11	37	57.13636364	8.699931677	0.070508552	0.057514087	+	80757	grey	0.196271871	FALSE	TMEM121
Region_5711	chr14	106482439	106521073	38634	5	29	44.71212121	6.588486815	0.86794996	0.059302912	+	112268139	grey	-0.041852621	FALSE	
Region_5712	chr1	20657638	20756183	98545	6	15	56.7424242	25.2234997	0.82471409	0.08406131	-	606	grey	0.196401273	FALSE	NBEAP1

	5						4	9	6	7						
Region_5713	chr15	22664359	22686213	21854	11	61	78.09090909	8.784124268	0.523195011	0.019912249	+	339005	grey	-0.004656844	FALSE	WHAMMP3
Region_5714	chr15	22757835	22778741	20906	9	45	58.98484848	6.96693824	0.811239591	0.043699191	+	283683	grey	0.114936375	FALSE	LOC283683
Region_5715	chr15	22838644	22869362	30718	14	94	116.3484848	11.11408313	0.907040533	0.024177614	+	81614	grey	-0.057193291	FALSE	NIPA2
Region_5716	chr15	23685400	23687305	1905	7	46	67.96969697	11.68163542	0.50105652	0.085438704	-	4692	grey	-0.123834841	FALSE	NDN
Region_5717	chr15	23912411	24090821	178410	25	175	211.469697	14.87095227	0.785175436	0.038124289	+	105370731	grey	-0.241859622	FALSE	PWRN4
Region_5718	chr15	24101827	24447967	346140	23	144	184.3181818	17.35436907	0.746354012	0.04160364	+	101928840	grey	-0.209950848	FALSE	PWRN3
Region_5719	chr15	24126593	24454028	327435	20	119	163.0151515	15.19563948	0.753031212	0.045192331	+	105370733	grey	-0.129357941	FALSE	LOC105370733
Region_5720	chr15	24145363	24912924	767561	80	540	631.3333333	33.72984678	0.758395866	0.019881458	+	791114	grey	-0.102615911	FALSE	PWRN1
Region_5721	chr15	24823637	24978583	154946	20	120	140.0757576	10.83201893	0.74051028	0.043159284	+	6638	grey	0.165088679	FALSE	SNRPN
Region_5722	chr15	24955034	25420336	465302	73	594	657.0757576	23.31279649	0.866294055	0.01928163	+	104472715	grey	0.076574269	FALSE	SNHG14
Region_5723	chr15	25070147	25122561	52414	11	98	118.4090909	10.33288592	0.871105783	0.037017802	+	3653	grey	0.088256958	FALSE	IPW
Region_5724	chr15	25333728	25439051	105323	12	72	93.98484848	10.05598547	0.736896776	0.023333787	-	7337	grey	0.205644923	FALSE	UBE3A

Region_5725	chr15	25456271	25578813	122542	17	117	140.8636364	12.80491055	0.887986872	0.033526499	-	105370737	grey	0.083600812	FALSE	LINC02250
Region_5726	chr15	25677273	25865184	187911	47	341	388.0757576	21.55730153	0.805810312	0.017451866	-	57194	grey	0.098805132	FALSE	ATP10A
Region_5727	chr15	25865295	25877211	11916	4	17	28.84848485	5.002283861	0.912845619	0.053891003	+	105370738	grey	0.061830386	FALSE	ATP10A-DT
Region_5728	chr15	25902119	26053123	151004	50	362	411.6818182	22.84877313	0.86922181	0.019325257	+	100128714	grey	0.184482383	FALSE	LINC02346
Region_5729	chr15	26115726	26134001	18275	7	49	65.40909091	7.783567054	0.653535851	0.056087444	+	503519	grey	0.024575844	FALSE	LINC00929
Region_5730	chr15	26395037	26455219	60182	8	54	69.84848485	7.686636604	0.823481584	0.050878857	+	107984780	grey	-0.05512406	FALSE	LINC02248
Region_5731	chr15	26403480	26477840	74360	13	84	112.5151515	10.70476878	0.856101017	0.03613622	-	105370740	grey	-0.089010937	FALSE	LOC105370740
Region_5732	chr15	26543546	26939539	395993	56	406	441.6515152	18.54349766	0.654001542	0.021242762	-	2562	grey	-0.144103395	FALSE	GABRB3
Region_5733	chr15	26749935	26790300	40365	5	21	34.15151515	6.95533536	0.759649247	0.105409858	-	112268151	grey	0.015493316	FALSE	LOC112268151
Region_5734	chr15	26866911	26949208	82297	21	132	161.4393939	13.57954207	0.540315357	0.035005233	+	2558	grey	-0.160316755	FALSE	GABRA5
Region_5735	chr15	26971181	27541984	570803	125	965	1055.80303	42.07912682	0.816433099	0.01503791	+	2567	grey	-0.091243743	FALSE	GABRG3
Region_5736	chr15	27038978	27161319	122341	30	226	253.7575758	17.06463785	0.810361495	0.022664384	-	101928869	grey	0.08408053	FALSE	GABRG3-AS1
Region_5737	chr1	28841832	28848675	6843	5	22	35.8333333	6.92727792	0.94058775	0.04549770	-	728310	grey	-0.09219028	FALSE	GOLGA6L7

	5						3	8		2				3		
Region_5738	chr15	30427080	30470946	43866	4	20	45.24242424	9.457696265	0.961648284	0.038693099	-	105376704	grey	-0.090907262	FALSE	LOC105376704
Region_5739	chr15	30470779	30507623	36844	8	33	67.3030303	13.43241739	0.713437468	0.035939793	+	100288203	grey	0.041529742	FALSE	WHAMMP4
Region_5740	chr15	30626129	30773002	146873	27	154	192.5454545	14.09002027	0.897197844	0.020697515	+	100288637	grey	0.195735537	FALSE	LOC100288637
Region_5741	chr15	30815271	30844153	28882	11	66	85.33333333	10.65014746	0.870982186	0.042327328	+	390561	grey	0.008712633	FALSE	HERC2P10
Region_5742	chr15	30992171	31042302	50131	13	84	108.530303	11.15239843	0.673036367	0.03758187	+	105370752	grey	-0.343009694	FALSE	LOC105370752
Region_5743	chr15	31202934	31230886	27952	8	45	65.15151515	9.016638337	0.831595248	0.071696675	-	283710	grey	-0.057398777	FALSE	LINC03034
Region_5744	chr15	31215622	31224445	8823	3	10	21.42424242	5.917537445	0.714055522	0.165845894	+	102725022	grey	-0.108865744	FALSE	LINC02352
Region_5745	chr15	31392843	31404729	11886	7	27	49.65151515	8.132541327	0.64880267	0.04546122	+	105370939	grey	0.084025136	FALSE	LOC105370939
Region_5746	chr15	31475398	31870789	395391	91	658	719.6818182	26.93305609	0.845547312	0.017871406	-	161725	grey	0.109813864	FALSE	OTUD7A
Region_5747	chr15	32536714	32575643	38929	7	29	57.36363636	11.0323111	0.462204778	0.051666873	+	100996255	grey	-0.144458699	FALSE	LINC02256
Region_5748	chr15	32585524	32615158	29634	13	51	83.62121212	14.68202999	0.896382014	0.038378136	-	101928042	grey	-0.06274434	FALSE	ARHGAP11A-DT
Region_5749	chr15	32765544	33194714	429170	81	609	671.9393939	31.28867025	0.888177997	0.014527299	-	342184	grey	-0.078954911	FALSE	FMN1

Region_5750	chr15	33293568	33310659	17091	6	35	48.51515152	7.095485941	0.601304669	0.052916845	-	101928134	grey	0.351625481	FALSE	RYR3-DT
Region_5751	chr15	33310962	33866121	555159	99	741	830	40.87767869	0.844327056	0.013813251	+	6263	grey	-0.122472011	FALSE	RYR3
Region_5752	chr15	33851785	34075155	223370	42	288	334.1060606	20.00702091	0.900285187	0.018840817	-	57099	grey	-0.133228701	FALSE	AVEN
Region_5753	chr15	33968497	34067458	98961	20	143	172.4090909	14.2083153	0.892103984	0.021406967	+	1133	grey	-0.094701367	FALSE	CHRM5
Region_5754	chr15	34102083	34108686	6603	3	18	31.48484848	5.18056256	0.94764585	0.037455768	+	161779	grey	-0.196211484	FALSE	PGBD4
Region_5755	chr15	34140674	34210096	69422	15	97	125.6363636	10.89902799	0.894764523	0.022784229	-	79768	grey	-0.003744137	FALSE	KATNBL1
Region_5756	chr15	34229784	34338060	108276	23	137	182.7121212	14.32347739	0.805272659	0.032774491	-	9990	grey	0.384017135	FALSE	SLC12A6
Region_5757	chr15	34343315	34357737	14422	7	37	54.06060606	8.318429563	0.952680925	0.026482937	+	256646	grey	0.029748231	FALSE	NUTM1
Region_5758	chr15	34378935	34437808	58873	5	17	38.54545455	10.27196606	0.906086891	0.076394911	-	23015	grey	0.109505708	FALSE	GOLGA8A
Region_5759	chr15	34525095	34588503	63408	8	30	64.33333333	17.94378687	0.578898929	0.067729808	-	440270	grey	-0.23031387	FALSE	GOLGA8B
Region_5760	chr15	34651135	34671095	19960	5	22	30.25757576	4.881079975	0.915881078	0.051152239	-	105370762	grey	0.019597703	FALSE	LINC02252
Region_5761	chr15	34755084	34813505	58421	16	89	114.4090909	11.85810631	0.764373185	0.048186057	+	101928174	grey	0.052764562	FALSE	GJD2-DT
Region_5762	chr1	34851782	34969742	117960	19	133	165.606060	13.7167823	0.9284794	0.01213758	-	9716	grey	0.18495308	FALSE	AQR

	5						6			8						
Region_5763	chr15	34993646	35011191	17545	3	17	31.04545455	4.80522734	0.962966745	0.034520752	+	105370764	grey	-0.085980591	FALSE	LOC105370764
Region_5764	chr15	35217345	35546193	328848	43	288	336.7424242	21.09269695	0.923619975	0.015764262	-	89978	grey	-0.100441562	FALSE	DPH6
Region_5765	chr15	35546154	35860199	314045	24	164	199.5	13.54451351	0.808323618	0.0259742	+	100507466	grey	0.071157171	FALSE	DPH6-DT
Region_5766	chr15	35919892	36049537	129645	8	39	53.6969697	7.223827447	0.87344727	0.0539822	+	100507480	grey	0.058393172	FALSE	LINC02853
Region_5767	chr15	36579626	36810248	230622	19	131	160.6212121	13.67059185	0.892768427	0.0216523	+	84529	grey	0.153022641	FALSE	CDIN1
Region_5768	chr15	36865628	36887052	21424	7	32	53.57575758	7.213364959	0.364874844	0.057570062	-	145845	grey	-0.026023313	FALSE	LOC145845
Region_5769	chr15	36889204	37101299	212095	26	174	210.8939394	14.78578193	0.720527095	0.027485454	-	4212	grey	-0.204390556	FALSE	MEIS2
Region_5770	chr15	37364020	37490864	126844	12	62	92.92424242	11.97858423	0.870321084	0.048235996	-	105370772	grey	0.075175808	FALSE	LOC105370772
Region_5771	chr15	37921939	38040987	119048	9	49	66.68181818	9.22231256	0.84666852	0.055675605	+	145942	grey	-0.02820448	FALSE	TMCO5A
Region_5772	chr15	38252836	38357249	104413	9	59	78.27272727	7.892636207	0.817321408	0.03076375	+	161742	grey	-0.060063662	FALSE	SPRED1
Region_5773	chr15	38488103	38565575	77472	9	55	76.89393939	10.07835272	0.811097962	0.030278855	-	10125	grey	-0.040567401	FALSE	RASGRP1
Region_5774	chr15	38628005	39058327	430322	34	282	317.9848485	17.26044159	0.799442078	0.019883831	+	105370775	grey	-0.116843679	FALSE	LOC105370775

Region_5775	chr15	38628060	39180499	552439	49	409	452.3030303	23.31851011	0.817821965	0.017609622	+	105370780	grey	-0.240390897	FALSE	LOC105370780
Region_5776	chr15	38696598	38733321	36723	3	22	33.15151515	5.054297255	0.935947303	0.048156606	+	400359	grey	-0.2125809	FALSE	LINC02694
Region_5777	chr15	38865322	39118713	253391	19	142	163.9393939	12.16031223	0.792731539	0.029900312	-	105370777	grey	-0.155702294	FALSE	LOC105370777
Region_5778	chr15	39581079	39599466	18387	4	28	41.37878788	5.953060367	0.934713838	0.041620019	+	7057	grey	-0.276088256	FALSE	THBS1
Region_5779	chr15	39588357	39801333	212976	23	148	174.2121212	11.19960872	0.835114023	0.02936302	-	161835	grey	-0.118344787	FALSE	FSIP1
Region_5780	chr15	39799008	39920266	121258	11	71	92.24242424	9.138356368	0.857170477	0.031316059	-	11245	grey	0.29140544	FALSE	GPR176
Region_5781	chr15	39934115	40035591	101476	17	124	158.4242424	14.07459316	0.913722968	0.01592131	+	440275	grey	0.019645627	FALSE	EIF2AK4
Region_5782	chr15	40039242	40080614	41372	20	117	150.8030303	15.38652727	0.752728825	0.039366525	+	100131089	grey	0.095294084	FALSE	SRP14-DT
Region_5783	chr15	40075204	40078704	3500	4	19	32.34848485	4.878500477	0.681232839	0.065138762	-	105370787	grey	0.226021107	FALSE	LOC105370787
Region_5784	chr15	40087890	40108892	21002	7	38	59.98484848	8.138214554	0.784912792	0.032310166	-	90427	grey	-0.014197271	FALSE	BMF
Region_5785	chr15	40161023	40221123	60100	11	63	81.74242424	8.765861415	0.949856337	0.025991202	+	701	grey	-0.218204189	FALSE	BUB1B
Region_5786	chr15	40217428	40276396	58968	22	139	173.9393939	14.84574924	0.880459531	0.02637909	+	106821730	grey	0.33933244	FALSE	BUB1B-PAK6
Region_5787	chr1	40218500	40277487	58987	22	139	173.939393	14.8457492	0.88045953	0.02637909	+	56924	grey	0.33933244	FALSE	PAK6

	5						9	4	1							
Region_5788	chr15	40278176	40307935	29759	16	102	135.1363636	12.76399547	0.521299884	0.032126831	-	5330	grey	0.056258282	FALSE	PLCB2
Region_5789	chr15	40278372	40283064	4692	8	46	66.24242424	10.56126538	0.224621398	0.022771329	-	100131244	grey	0.102723	FALSE	ANKRD63
Region_5790	chr15	40323692	40326715	3023	5	16	28.13636364	6.111493242	0.001551227	0.006569841	+	100505573	grey	-0.150077098	FALSE	INAFM2
Region_5791	chr15	40331452	40340939	9487	3	11	20.62121212	4.153831045	0.901824906	0.095461741	-	388115	grey	0.143199982	FALSE	CCDC9B
Region_5792	chr15	40351033	40356434	5401	3	19	29.1969697	4.648307209	0.968953234	0.037505482	+	644844	grey	-0.015563322	FALSE	PHGR1
Region_5793	chr15	40358219	40378621	20402	13	70	94.16666667	10.46838954	0.58029711	0.029406805	+	85455	grey	-0.286786741	FALSE	DISP2
Region_5794	chr15	40382721	40394288	11567	3	16	28.36363636	6.050140147	0.654847601	0.027444043	+	90417	grey	-0.151387972	FALSE	KNSTRN
Region_5795	chr15	40405485	40435947	30462	14	93	120.0454545	11.64531179	0.653126627	0.024941302	+	3712	grey	0.070280768	FALSE	IVD
Region_5796	chr15	40439721	40468236	28515	12	58	79.24242424	9.537070355	0.721257858	0.024190064	+	22893	grey	0.230447767	FALSE	BAHD1
Region_5797	chr15	40528683	40565054	36371	21	139	163.8484848	12.37024025	0.813323688	0.02444021	-	90416	grey	0.005957879	FALSE	CCDC32
Region_5798	chr15	40569299	40574949	5650	8	46	70.03030303	9.768032157	0.731214518	0.022232251	+	27079	grey	0.056092441	FALSE	RPUSD2
Region_5799	chr15	40594020	40664342	70322	41	243	284.4848485	19.04185061	0.878459971	0.009988383	+	57082	grey	0.213767224	FALSE	KNL1

Region_5800	chr15	40686183	40695107	8924	4	26	37.86363636	5.532801109	0.948182388	0.039531579	-	100505648	grey	0.014444731	FALSE	RAD51-AS1
Region_5801	chr15	40694774	40732340	37566	24	150	198.5	15.30736374	0.8201789	0.016879194	+	5888	grey	-0.264339542	FALSE	RAD51
Region_5802	chr15	40735884	40755851	19967	7	42	56.77272727	7.435456542	0.891947858	0.056397979	-	55177	grey	0.056266545	FALSE	RMDN3
Region_5803	chr15	40764068	40767708	3640	4	18	30.43939394	6.547175052	0.005657768	0.01288992	+	2644	grey	0.056697667	FALSE	GCHFR
Region_5804	chr15	40765161	40807478	42317	24	140	170.2272727	12.22330115	0.873955695	0.021354681	-	55192	grey	-0.193194737	FALSE	DNAJC17
Region_5805	chr15	40807086	40815084	7998	5	27	40.36363636	6.156013604	0.581867485	0.034542507	+	84936	grey	0.355681189	FALSE	ZFYVE19
Region_5806	chr15	40815451	40828709	13258	8	41	59.15151515	7.807772113	0.956722182	0.029048871	-	54866	grey	0.004348424	FALSE	PPP1R14D
Region_5807	chr15	40844018	40858207	14189	7	25	36.24242424	5.059828535	0.529120402	0.039572998	+	6692	grey	0.175626288	FALSE	SPINT1
Region_5808	chr15	40872214	40874234	2020	5	13	28.72727273	5.848196184	0.258428189	0.060084178	-	171177	grey	-0.125740056	FALSE	RHOV
Region_5809	chr15	40894450	40903975	9525	5	20	31.71212121	6.103631079	0.226981831	0.039925937	+	57617	grey	-0.124822855	FALSE	VPS18
Region_5810	chr15	40906811	40910337	3526	5	29	41.86363636	5.637474513	0.642931809	0.057259134	+	105370943	grey	-0.158546183	FALSE	LOC105370943
Region_5811	chr15	40929340	40939073	9733	10	74	91.09090909	10.29970466	0.596266104	0.038161459	+	54567	grey	0.225744728	FALSE	DLL4
Region_5812	chr1	40978880	41116280	137400	64	448	486.666666	19.9894844	0.96634077	0.01145621	-	54617	grey	-0.03054781	FALSE	INO80

	5						7	2		6						
Region_5813	chr15	41182725	41230757	48032	33	191	257.4393939	22.75632036	0.895724394	0.027826668	-	161829	grey	0.013099927	FALSE	EXD1
Region_5814	chr15	41230839	41281887	51048	33	209	242.7575758	16.30862028	0.875147181	0.015577966	+	11261	grey	-0.054634263	FALSE	CHP1
Region_5815	chr15	41283990	41309737	25747	7	40	55.37878788	7.395976352	0.624663525	0.039226116	+	729082	grey	-0.161879467	FALSE	OIP5-AS1
Region_5816	chr15	41284007	41381050	97043	34	212	252.1666667	19.82739622	0.844067055	0.015885822	+	51203	grey	-0.043702254	FALSE	NUSAP1
Region_5817	chr15	41309273	41332591	23318	8	36	54.93939394	9.225180904	0.978640528	0.02100334	-	11339	grey	-0.049385011	FALSE	OIP5
Region_5818	chr15	41387218	41409403	22185	23	136	168.6515152	14.38213895	0.824884744	0.02069122	-	51103	grey	0.059551444	FALSE	NDUFAF1
Region_5819	chr15	41408408	41483563	75155	40	293	339.4090909	18.26385861	0.900437115	0.013192067	+	23168	grey	-0.379496765	FALSE	RTF1
Region_5820	chr15	41493393	41503551	10158	10	46	64.98484848	9.892487319	0.320576889	0.03436699	+	3706	grey	-0.07698291	FALSE	ITPKA
Region_5821	chr15	41503637	41513887	10250	10	50	80.92424242	10.11215659	0.209162351	0.037831013	-	4058	grey	0.300225899	FALSE	LTK
Region_5822	chr15	41517176	41544269	27093	12	67	87.83333333	9.330814678	0.640912978	0.026975778	-	26015	grey	-0.148524994	FALSE	RPAP1
Region_5823	chr15	41557675	41583589	25914	18	118	142.3181818	11.45044049	0.861743855	0.033037874	+	7301	grey	-0.140094594	FALSE	TYRO3
Region_5824	chr15	41609457	41630908	21451	16	95	122.8636364	12.55864564	0.725805608	0.017672502	-	105370791	grey	-0.030008982	FALSE	LOC105370791

Region_5825	chr15	41621134	41773081	151947	62	403	467.8787879	25.65541787	0.77304653	0.014173649	+	23269	grey	-0.256029324	FALSE	MGA
Region_5826	chr15	41774434	41827855	53421	35	233	274.7575758	17.74005516	0.877185229	0.014534335	+	23005	grey	0.152276377	FALSE	MAPKBP1
Region_5827	chr15	41828092	41837581	9489	8	39	57.33333333	7.754568546	0.920304146	0.042266958	+	100137047	grey	0.111929065	FALSE	JMJD7
Region_5828	chr15	41828095	41848155	20060	19	99	131.2878788	10.63052955	0.941359381	0.024382515	+	8681	grey	-0.00884701	FALSE	JMJD7-PLA2G4B
Region_5829	chr15	41836785	41848147	11362	14	73	101.4393939	9.666960047	0.960663171	0.022982805	+	100137049	grey	-0.095258988	FALSE	PLA2G4B
Region_5830	chr15	41848146	41894053	45907	36	225	289.5757576	22.94143756	0.894304391	0.025311298	-	51332	grey	-0.03733337	FALSE	SPTBN5
Region_5831	chr15	41895933	41972557	76624	37	261	300.8939394	17.23692708	0.854561246	0.021714267	-	30844	grey	0.057960986	FALSE	EHD4
Region_5832	chr15	41972763	41999094	26331	10	67	85.83333333	8.829292738	0.884665813	0.036608578	+	101928388	grey	-0.39488084	FALSE	PLA2G4E-AS1
Region_5833	chr15	41981394	42051190	69796	24	156	184.6363636	15.15624914	0.90754955	0.021908213	-	123745	grey	-0.068951131	FALSE	PLA2G4E
Region_5834	chr15	42067009	42094562	27553	7	42	58.43939394	7.950091993	0.707270874	0.062689749	-	283748	grey	0.405883105	FALSE	PLA2G4D
Region_5835	chr15	42139034	42156636	17602	17	98	124.6363636	10.14454968	0.916364937	0.027022768	-	255189	grey	-0.013427581	FALSE	PLA2G4F
Region_5836	chr15	42158701	42208307	49606	21	129	162.9242424	14.21084159	0.861267831	0.016061832	-	23339	grey	0.147241192	FALSE	VPS39
Region_5837	chr1	42208413	42226990	18577	3	15	27.0606060	5.08959357	0.97058215	0.03427784	+	10537079	grey	-0.12031654	FALSE	VPS39-DT

	5						6		4	7		5		1		
Region_5838	chr15	42210447	42273584	63137	17	117	137.1363636	11.60223634	0.923162161	0.023189035	-	25963	grey	0.043824952	FALSE	TMEM87A
Region_5839	chr15	42273201	42353666	80465	16	101	125.8333333	10.67359551	0.75778938	0.01848552	+	2595	grey	-0.150383567	FALSE	GANC
Region_5840	chr15	42348103	42412949	64846	22	154	186.3333333	13.839001	0.92701914	0.016378531	+	825	grey	-0.031248354	FALSE	CAPN3
Region_5841	chr15	42412823	42491141	78318	20	126	154.8333333	13.23234189	0.955679916	0.020776129	-	64397	grey	-0.009864837	FALSE	ZNF106
Region_5842	chr15	42491233	42545356	54123	9	50	71.25757576	9.182442454	0.60900527	0.036621023	+	8773	grey	0.146674177	FALSE	SNAP23
Region_5843	chr15	42497394	42548802	51408	6	37	48.90909091	6.539177254	0.910509668	0.054106144	-	255252	grey	0.160663632	FALSE	LRRC57
Region_5844	chr15	42548828	42569994	21166	5	30	44.33333333	7.115169782	0.97184568	0.026268586	+	55142	grey	-0.073210001	FALSE	HAUS2
Region_5845	chr15	42575606	42720998	145392	21	154	180.530303	14.20424606	0.901680196	0.018418099	+	57519	grey	-0.018455886	FALSE	STARD9
Region_5846	chr15	42723544	42737128	13584	4	19	28.96969697	4.9674934	0.788281689	0.07351221	-	146059	grey	0.022449069	FALSE	CDAN1
Region_5847	chr15	42738730	42920809	182079	42	297	330.530303	17.08370316	0.973280027	0.008115401	-	146057	grey	-0.021613822	FALSE	TTBK2
Region_5848	chr15	42942897	43106113	163216	39	277	313.5454545	21.36581152	0.908207173	0.01445357	-	197131	grey	0.094978035	FALSE	UBR1
Region_5849	chr15	43123279	43185144	61865	8	48	68.13636364	8.765116812	0.821077642	0.02524871	+	80021	grey	0.156986189	FALSE	TMEM62

Region_5850	chr15	43179793	43221018	41225	8	47	65.18181818	6.85657257	0.763464263	0.045893731	-	2038	grey	-0.039315779	FALSE	EPB42
Region_5851	chr15	43232590	43266928	34338	8	50	67.37878788	9.008995763	0.872321696	0.042688206	-	9333	grey	-0.132975581	FALSE	TGM5
Region_5852	chr15	43276271	43302255	25984	7	34	50.24242424	7.499867909	0.844844956	0.051578403	-	116179	grey	-0.047008982	FALSE	TGM7
Region_5853	chr15	43330672	43354569	23897	5	25	39.95454545	6.354724029	0.967704807	0.036447978	+	161823	grey	-0.155056383	FALSE	ADAL
Region_5854	chr15	43358172	43371043	12871	8	47	62.72727273	7.341233295	0.939742458	0.038522147	-	146050	grey	-0.286328695	FALSE	ZSCAN29
Region_5855	chr15	43369221	43409771	40550	11	75	94.60606061	8.933736901	0.954285585	0.025424845	+	27229	grey	-0.035985026	FALSE	TUBGCP4
Region_5856	chr15	43403061	43510728	107667	24	176	203.1060606	13.72491142	0.842454751	0.014340928	-	7158	grey	0.16427276	FALSE	TP53BP1
Region_5857	chr15	43510958	43531620	20662	6	41	52.87878788	7.03292291	0.784377602	0.039122352	+	4130	grey	0.014127869	FALSE	MAP1A
Region_5858	chr15	43533462	43590208	56746	7	30	50.03030303	8.197503742	0.938070005	0.033223812	-	9677	grey	0.000249445	FALSE	PPIP5K1
Region_5859	chr15	43599563	43632283	32720	3	19	29.84848485	4.912284207	0.759312346	0.07214129	-	161497	grey	-0.055652401	FALSE	STRC
Region_5860	chr15	43628503	43648877	20374	4	16	30.83333333	5.603799078	0.977462757	0.028335124	-	117155	grey	-0.123446373	FALSE	CATSPER2
Region_5861	chr15	43726918	43747094	20176	10	49	74.62121212	8.610843167	0.772333001	0.020831366	-	440278	grey	0.187196881	FALSE	CATSPER2P1
Region_5862	chr1	43746394	43773279	26885	4	24	34.2424242	5.58035181	0.94683318	0.04387971	+	2923	grey	0.16957462	FALSE	PDIA3

	5						4	2	8	2						
Region_5863	chr15	43772605	43799133	26528	8	46	64.90909091	7.474525501	0.650762143	0.027357714	-	80237	grey	0.161689581	FALSE	ELL3
Region_5864	chr15	43777087	43799007	21920	7	43	59.74242424	7.17326362	0.613206965	0.028366687	+	10169	grey	-0.025581483	FALSE	SERF2
Region_5865	chr15	43792354	43804427	12073	4	21	31.40909091	5.241776777	0.411415103	0.077238178	+	25764	grey	-0.073175636	FALSE	HYPK
Region_5866	chr15	43804492	43824690	20198	7	42	58.57575758	7.49576415	0.699892701	0.02100506	-	4236	grey	-5.93245E-05	FALSE	MFAP1
Region_5867	chr15	43826980	43868412	41432	4	19	27.5	5.186520992	0.957879246	0.039474246	+	79968	grey	-0.110979849	FALSE	WDR76
Region_5868	chr15	43870761	44195271	324510	79	593	646.5	26.54647594	0.895107895	0.014802564	-	84978	grey	-0.184646886	FALSE	FRMD5
Region_5869	chr15	44288719	44415758	127039	13	76	97.5	9.307151923	0.878880018	0.03239654	+	113201	grey	0.285115481	FALSE	GOLM2
Region_5870	chr15	44427622	44529038	101416	29	189	223.469697	15.76775648	0.907645957	0.019209534	+	51496	grey	-0.275409967	FALSE	CTDSPL2
Region_5871	chr15	44527257	44537046	9789	5	18	28.33333333	5.24868726	0.96917799	0.038629962	-	645212	grey	-0.155902431	FALSE	EIF3J-DT
Region_5872	chr15	44537125	44563029	25904	15	102	123.2727273	11.10169142	0.521763567	0.014552298	+	8669	grey	0.011070366	FALSE	EIF3J
Region_5873	chr15	44554818	44663688	108870	26	197	230.9090909	16.09942882	0.886569288	0.011982071	-	80208	grey	-0.201557357	FALSE	SPG11
Region_5874	chr15	44665732	44711323	45591	9	61	81.72727273	8.126964529	0.764431669	0.039160067	-	197135	grey	0.236218454	FALSE	PATL2

Region_5875	chr15	45023147	45077185	54038	10	51	70.37878788	7.588990308	0.960676958	0.028383698	+	6652	grey	0.619472026	FALSE	SORD
Region_5876	chr15	45092650	45114172	21522	8	39	58.60606061	7.406605758	0.502172645	0.037779993	-	50506	grey	0.158758731	FALSE	DUOX2
Region_5877	chr15	45117366	45129938	12572	6	29	39.5	6.162541933	0.461494415	0.045824897	-	90527	grey	-0.175041953	FALSE	DUOXA1
Region_5878	chr15	45129933	45165576	35643	14	82	103.530303	11.26085076	0.715373685	0.036871761	+	53905	grey	-0.296256789	FALSE	DUOX1
Region_5879	chr15	45167214	45201175	33961	6	27	36.62121212	5.077143811	0.448944696	0.076981153	-	90525	grey	0.014440688	FALSE	SHF
Region_5880	chr15	45235930	45279251	43321	4	20	31.48484848	5.358652024	0.7702173	0.115300809	-	101928414	grey	-0.183008033	FALSE	SLC28A2-AS1
Region_5881	chr15	45252234	45277846	25612	3	12	21.48484848	4.507233244	0.718772438	0.150967805	+	9153	grey	-0.137819186	FALSE	SLC28A2
Region_5882	chr15	45361124	45402327	41203	10	70	94.92424242	9.985082113	0.413073913	0.031382045	-	2628	grey	-0.107805194	FALSE	GATM
Region_5883	chr15	45402336	45421415	19079	14	99	124.1515152	13.71719014	0.436999544	0.018759245	+	79029	grey	0.172657825	FALSE	AFG2B
Region_5884	chr15	45430579	45448761	18182	6	30	43.90909091	5.612423781	0.809833643	0.049998856	+	84419	grey	0.169888895	FALSE	C15orf48
Region_5885	chr15	45430652	45441808	11156	4	15	28.27272727	4.390708562	0.943983211	0.050971386	-	107987223	grey	0.01887127	FALSE	LOC107987223
Region_5886	chr15	45448427	45513767	65340	27	195	226.8787879	13.84809362	0.723297058	0.026179763	+	105376714	grey	0.054289963	FALSE	SLC30A4-AS1
Region_5887	chr1	45479606	45522755	43149	16	106	127.257575	10.8163844	0.83576133	0.02199015	-	7782	grey	0.098912217	FALSE	SLC30A4

	5						8	5	5	5						
Region_5888	chr15	45511136	45556730	45594	7	42	56.68181818	7.686469813	0.677759993	0.030050729	+	283651	grey	-0.014779368	FALSE	HMGN2P46
Region_5889	chr15	45587214	45615945	28731	3	11	24.62121212	5.199896897	0.643356943	0.033812103	+	26258	grey	-0.096692022	FALSE	BLOC1S6
Region_5890	chr15	45587351	45691281	103930	22	166	195.1363636	15.36518178	0.81755884	0.019139038	+	58472	grey	-0.106056945	FALSE	SQOR
Region_5891	chr15	45705078	45931069	225991	37	243	292	19.83237447	0.81727342	0.025985875	+	105370802	grey	0.122289311	FALSE	LOC105370802
Region_5892	chr15	47184101	47774228	590127	37	282	316.2121212	16.76857423	0.805397518	0.024281813	+	80031	grey	-0.0779562	FALSE	SEMA6D
Region_5893	chr15	47774219	47846266	72047	5	31	45.56060606	5.80219796	0.664403389	0.059358689	-	101928442	grey	0.393716416	FALSE	LINC01491
Region_5894	chr15	47812729	48006060	193331	18	128	155.8939394	12.62370028	0.816940697	0.034155267	+	107984136	grey	0.179339843	FALSE	
Region_5895	chr15	47812729	48050507	237778	26	195	231.2424242	14.39237042	0.839150727	0.031048684	+	105370805	grey	0.222235261	FALSE	LOC105370805
Region_5896	chr15	47956521	47988044	31523	4	22	32.81818182	5.479778465	0.795112373	0.110084163	-	102724553	grey	0.100710616	FALSE	LOC102724553
Region_5897	chr15	48178122	48203758	25636	6	29	43.06060606	6.444744521	0.767095093	0.0470823	+	399697	grey	-0.027082554	FALSE	CTXN2
Region_5898	chr15	48178438	48304078	125640	17	101	136.4393939	12.80400032	0.861990707	0.029245386	+	6557	grey	-0.023016912	FALSE	SLC12A1
Region_5899	chr15	48312353	48331856	19503	3	19	30.48484848	5.50032845	0.964513346	0.033535152	-	107984755	grey	-0.061256851	FALSE	LOC107984755

Region_5900	chr15	48408313	48645721	237408	29	228	260.2878788	17.16236056	0.77551043	0.018661839	-	2200	grey	-0.08494141	FALSE	FBN1
Region_5901	chr15	48645951	48652016	6065	3	15	24.60606061	4.526431054	0.327579692	0.020107097	+	105370809	grey	-0.207168015	FALSE	FBN1-DT
Region_5902	chr15	48712928	48811146	98218	7	51	67.90909091	8.053145153	0.757927186	0.041795562	-	22995	grey	-0.036128998	FALSE	CEP152
Region_5903	chr15	48823741	48963919	140178	11	80	102.5757576	10.34640124	0.92497817	0.033207193	-	399694	grey	-0.059447372	FALSE	SHC4
Region_5904	chr15	48988476	49046447	57971	9	58	77.5	8.489359856	0.87555412	0.022436456	-	9728	grey	0.084518775	FALSE	SECISBP2L
Region_5905	chr15	49106068	49155661	49593	5	17	32.25757576	5.79472069	0.96859304	0.034150005	-	9318	grey	0.042392125	FALSE	COPS2
Region_5906	chr15	49155656	49367869	212213	27	168	197.3030303	13.72024859	0.820171884	0.017129908	+	2585	grey	-0.06360707	FALSE	GALK2
Region_5907	chr15	49326962	49620929	293967	21	133	161.1818182	12.93642334	0.820787245	0.031091376	-	196951	grey	-0.222542616	FALSE	FAM227B
Region_5908	chr15	49423237	49488775	65538	3	18	26.93939394	4.660402056	0.889575751	0.068574622	+	2252	grey	0.065494742	FALSE	FGF7
Region_5909	chr15	49858238	50182817	324579	33	242	280.3030303	18.26385223	0.861158059	0.023582753	-	79895	grey	-0.087943942	FALSE	ATP8B4
Region_5910	chr15	50182196	50236385	54189	3	17	25.90909091	5.152228115	0.890539568	0.080282747	+	11001	grey	0.206048578	FALSE	SLC27A2
Region_5911	chr15	50241947	50265965	24018	4	19	31.3030303	5.533032822	0.717904975	0.098061951	-	3067	grey	0.658836137	TRUE	HDC
Region_5912	chr1	50275389	50355408	80019	10	54	75.4696969	8.51644508	0.74285534	0.04195521	-	2553	grey	-0.24259915	FALSE	GABPB1

	5						7	2	8	1				3		
Region_5913	chr15	50424380	50514421	90041	25	155	191.8333333	15.73881369	0.965318926	0.021661829	+	9101	grey	-0.269388725	FALSE	USP8
Region_5914	chr15	50494018	50546708	52690	17	95	123.8181818	11.27408068	0.933202986	0.0286202	-	373509	grey	-0.065960168	FALSE	USP50
Region_5915	chr15	50552473	50686797	134324	32	201	255.1666667	19.37213171	0.814255062	0.02205431	-	54822	grey	-0.082953445	FALSE	TRPM7
Region_5916	chr15	50702266	50765948	63682	19	106	128.6212121	10.19617598	0.856220568	0.036287381	-	84888	grey	-0.237612372	FALSE	SPPL2A
Region_5917	chr15	50908672	51005895	97223	18	102	128.5757576	11.44627666	0.89449396	0.026574824	+	23431	grey	-0.347833457	FALSE	AP4E1
Region_5918	chr15	51037488	51293912	256424	47	378	414.6363636	20.49888279	0.734803423	0.018389271	+	109729174	grey	0.11224528	FALSE	MIR4713HG
Region_5919	chr15	51056598	51105276	48678	17	108	139.9545455	11.97488631	0.558890944	0.022843501	-	388121	grey	-0.078722223	FALSE	TNFAIP8L3
Region_5920	chr15	51208057	51338601	130544	13	95	116.6818182	11.03025084	0.894634657	0.032941615	-	1588	grey	0.113023665	FALSE	CYP19A1
Region_5921	chr15	51341655	51408005	66350	20	112	146.8030303	14.26800584	0.77752119	0.028362863	+	342035	grey	0.170737224	FALSE	GLDN
Region_5922	chr15	51447711	51622833	175122	20	130	160.0757576	14.24544257	0.895157766	0.020487957	-	23312	grey	0.011795852	FALSE	DMXL2
Region_5923	chr15	51681492	51721026	39534	10	61	88.39393939	11.04379922	0.807307885	0.024492732	+	29106	grey	-0.139896573	FALSE	SCG3
Region_5924	chr15	51723011	51751585	28574	9	57	83.77272727	8.639436332	0.653058165	0.031576963	-	256586	grey	0.114616604	FALSE	LYSMD2

Region_5925	chr15	51751597	51816363	64766	6	29	43.16666667	6.831112835	0.617050356	0.077667209	+	29767	grey	0.436936428	FALSE	TMOD2
Region_5926	chr15	51829628	51931308	101680	16	108	128.1818182	9.565819103	0.854632675	0.01404482	+	29766	grey	-0.138447008	FALSE	TMOD3
Region_5927	chr15	51919073	51930627	11554	3	19	30.21212121	5.240420275	0.978038857	0.030413017	-	100422556	grey	-0.167392268	FALSE	LOC100422556
Region_5928	chr15	51921494	51947295	25801	4	24	36.33333333	4.965522153	0.938296503	0.070776453	+	112268148	grey	0.31764378	FALSE	LOC112268148
Region_5929	chr15	51938025	51971778	33753	5	23	40.33333333	6.627061847	0.953417761	0.039518751	-	123169	grey	-0.124127285	FALSE	LEO1
Region_5930	chr15	51952106	52067375	115269	24	167	198.030303	14.88620394	0.905342563	0.017577341	+	5597	grey	0.074222208	FALSE	MAPK6
Region_5931	chr15	52056675	52100523	43848	8	52	71.92424242	9.369854454	0.660785058	0.043309677	-	112268150	grey	-0.220950649	FALSE	LOC112268150
Region_5932	chr15	52115100	52191392	76292	23	135	178.030303	15.88029228	0.710817683	0.025655646	-	10681	grey	-0.087164123	FALSE	GNB5
Region_5933	chr15	52179999	52206915	26916	9	56	74.51515152	8.711164232	0.681709214	0.029462632	+	100129973	grey	-0.102223793	FALSE	CERNA1
Region_5934	chr15	52192322	52295804	103482	44	335	372.5151515	17.73327374	0.801868812	0.019438779	-	55930	grey	0.093913504	FALSE	MYO5C
Region_5935	chr15	52307281	52529132	221851	48	322	387.1212121	23.56563022	0.861188082	0.013342638	-	4644	grey	-0.2460152	FALSE	MYO5A
Region_5936	chr15	52547045	52569883	22838	9	58	76.66666667	7.302967433	0.493493336	0.04279173	-	10776	grey	-0.061835206	FALSE	ARPP19
Region_5937	chr1	52581317	52709817	128500	16	102	128.772727	11.1380090	0.96306522	0.02462011	-	56204	grey	0.453285324	FALSE	ATOSA

	5						3	1	6	6						
Region_5938	chr15	52679474	52690077	10603	3	11	22.87878788	5.193235694	0.960059049	0.045652359	+	105370821	grey	0.101372571	FALSE	LOC105370821
Region_5939	chr15	52755053	52791078	36025	8	42	59.87878788	8.473515575	0.483816921	0.032106543	-	3175	grey	-0.068581096	FALSE	ONECUT1
Region_5940	chr15	52800161	52805972	5811	3	17	27	4.939635614	0.082172478	0.048698104	-	101928499	grey	-0.489298394	FALSE	LOC101928499
Region_5941	chr15	53035170	53130344	95174	18	131	157.2272727	12.91248109	0.853927585	0.030251945	+	105370822	grey	0.013812176	FALSE	LINC02490
Region_5942	chr15	53513741	53762878	249137	17	107	134.030303	11.30822668	0.794812034	0.028429496	-	256764	grey	-0.039008834	FALSE	WDR72
Region_5943	chr15	53978201	54628707	650506	39	292	322.7575758	17.33299265	0.852323839	0.025683621	+	440279	grey	-0.003137679	FALSE	UNC13C
Region_5944	chr15	55202966	55319113	116147	14	97	118.3939394	11.0563296	0.843408462	0.032860868	-	5873	grey	0.026177287	FALSE	RAB27A
Region_5945	chr15	55289888	55348466	58578	6	40	54.36363636	7.529708062	0.739288842	0.042810784	-	101928527	grey	0.088875247	FALSE	PIGBOS1
Region_5946	chr15	55318960	55355648	36688	3	17	29.78787879	5.25800533	0.535931801	0.06964993	+	9488	grey	0.012254037	FALSE	PIGB
Region_5947	chr15	55340032	55408510	68478	12	73	93.81818182	10.38624696	0.799186896	0.04185956	-	9236	grey	0.036332304	FALSE	CCPG1
Region_5948	chr15	55355248	55498360	143112	29	181	216.1363636	15.6518727	0.894439878	0.020483512	-	100533483	grey	0.187939379	FALSE	DNAAF4-CCPG1
Region_5949	chr15	55410525	55508234	97709	20	130	151.9242424	10.00816683	0.952972314	0.019102825	-	161582	grey	0.255930388	FALSE	DNAAF4

Region_5950	chr15	55538884	55588947	50063	18	74	100.1666667	13.65800226	0.50381607	0.034583645	-	26108	grey	-0.224013439	FALSE	PYGO1
Region_5951	chr15	55611544	55743152	131608	28	186	217.6666667	14.6248822	0.881453544	0.016520848	-	283659	grey	0.116401415	FALSE	PRTG
Region_5952	chr15	55826922	55993660	166738	31	214	248.4090909	17.61815259	0.868896742	0.031878735	-	4734	grey	-0.166891747	FALSE	NEDD4
Region_5953	chr15	56087280	56245082	157802	17	113	137.1515152	11.31131826	0.918828171	0.020605122	-	64864	grey	0.163531454	FALSE	RFX7
Region_5954	chr15	56243971	56445997	202026	26	166	214.7121212	17.6101065	0.862605897	0.015988339	+	374618	grey	0.09478261	FALSE	TEX9
Region_5955	chr15	56421544	56465137	43593	5	30	46.10606061	6.899562501	0.740472259	0.038768902	-	55329	grey	0.19668889	FALSE	MNS1
Region_5956	chr15	56542952	56629592	86640	6	42	62.51515152	8.175465029	0.955023735	0.029540365	-	105370832	grey	0.087537626	FALSE	LOC105370832
Region_5957	chr15	56630176	56918571	288395	25	172	208.2878788	14.53564865	0.808149688	0.013529928	-	54816	grey	0.069684685	FALSE	ZNF280D
Region_5958	chr15	56795460	56918499	123039	13	69	106.1818182	11.61684333	0.747306115	0.017524157	-	145783	grey	0.111662234	FALSE	TCF12-DT
Region_5959	chr15	56918623	57299281	380658	49	370	416.5454545	23.58759897	0.812237219	0.019974821	+	6938	grey	0.07544516	FALSE	TCF12
Region_5960	chr15	57375967	57550717	174750	25	177	212.4242424	15.81436602	0.858821683	0.023885167	+	84952	grey	-0.19783967	FALSE	CGNL1
Region_5961	chr15	57591904	57685364	93460	18	125	146.4545455	10.82473211	0.934137859	0.020758561	+	100820829	grey	-0.072656933	FALSE	MYZAP
Region_5962	chr1	57591908	57714745	122837	22	161	182.075757	10.8561370	0.88928607	0.01855111	+	145781	grey	-0.08149832	FALSE	GCOM1

	5						6	2	2	6				5		
Region_5963	chr15	57706695	57717557	10862	4	25	35.62121212	5.593306856	0.68796782	0.045833092	+	81488	grey	-0.027704054	FALSE	POLR2M
Region_5964	chr15	57706712	57782762	76050	18	143	169.969697	13.01829446	0.836209224	0.02787732	+	105370834	grey	0.379308288	FALSE	LOC105370834
Region_5965	chr15	57953424	58473913	520489	68	490	550.2424242	25.57105371	0.828730307	0.014711468	-	8854	grey	0.013862526	FALSE	ALDH1A2
Region_5966	chr15	58138169	58185911	47742	7	40	55.51515152	6.741929476	0.923384834	0.053892076	+	366	grey	0.090366844	FALSE	AQP9
Region_5967	chr15	58410569	58569844	159275	27	184	217.9393939	14.88817681	0.940221212	0.019433529	+	3990	grey	-0.060049499	FALSE	LIPC
Region_5968	chr15	58434890	58498735	63845	9	43	67.39393939	7.951543223	0.949052022	0.028498202	-	101928694	grey	-0.183214033	FALSE	LIPC-AS1
Region_5969	chr15	58588809	58749791	160982	28	189	230.5454545	16.5893217	0.732358834	0.02089708	-	102	grey	0.143632445	FALSE	ADAM10
Region_5970	chr15	58771192	58861900	90708	26	159	197.9242424	12.58975237	0.840784823	0.019210731	+	54629	grey	-0.037624309	FALSE	MINDY2
Region_5971	chr15	58865175	59097419	232244	56	393	473.8333333	27.23266327	0.801135737	0.012918344	+	54778	grey	-0.0133829	FALSE	RNF111
Region_5972	chr15	58879050	58933679	54629	6	35	47.10606061	6.122163489	0.807860075	0.022598421	-	79811	grey	-0.11066253	FALSE	SLTM
Region_5973	chr15	59105126	59125045	19919	6	33	47.81818182	6.563088135	0.660926808	0.038768406	+	9133	grey	0.217591809	FALSE	CCNB2
Region_5974	chr15	59132434	59372871	240437	72	540	593.7272727	20.68782284	0.855086045	0.015822727	-	4643	grey	0.143796342	FALSE	MYO1E

Region_5975	chr15	59372693	59523555	150862	34	221	264.969697	17.08437856	0.805591009	0.020557866	+	145773	grey	-0.237561666	FALSE	FAM81A
Region_5976	chr15	59594875	59640239	45364	6	31	46.16666667	5.71525175	0.911069122	0.036673783	+	9245	grey	-0.258200228	FALSE	GCNT3
Region_5977	chr15	59638062	59657541	19479	7	29	46.09090909	8.548013305	0.548751158	0.023129536	-	2958	grey	-0.139282762	FALSE	GTF2A2
Region_5978	chr15	59659146	59689339	30193	11	74	98.36363636	10.0546757	0.696669579	0.021067591	-	663	grey	0.089427825	FALSE	BNIP2
Region_5979	chr15	60004234	60061730	57496	19	111	144.6515152	14.90120535	0.31574167	0.027441187	+	27023	grey	0.227890198	FALSE	FOXB1
Region_5980	chr15	60347134	60402883	55749	10	56	75.09090909	8.641095502	0.862277944	0.037829854	-	302	grey	0.09081081	FALSE	ANXA2
Region_5981	chr15	60419609	60479160	59551	10	67	89.5	9.15885448	0.678480933	0.043872758	-	79664	grey	-0.301047662	FALSE	ICE2
Region_5982	chr15	60479152	60630637	151485	32	189	233	19.67896182	0.654521782	0.01862533	+	101928784	grey	0.127492982	FALSE	RORA-AS1
Region_5983	chr15	60488284	61229302	741018	141	1080	1163.863636	42.92637216	0.793098867	0.013333712	-	6095	grey	-0.188975099	FALSE	RORA
Region_5984	chr15	60634503	60640915	6412	3	12	21.8030303	5.011432152	0.944705659	0.063271042	+	105370846	grey	0.128306442	FALSE	LOC105370846
Region_5985	chr15	61038826	61229233	190407	41	322	355.1212121	18.63207751	0.777152948	0.025864071	-	107984805	grey	0.066677767	FALSE	LOC107984805
Region_5986	chr15	61298791	61715203	416412	56	445	500.6666667	25.71460724	0.830014048	0.019885296	-	107984782	grey	0.099999069	FALSE	LOC107984782
Region_5987	chr1	61852389	62060473	208084	28	197	232.454545	15.2942239	0.86665540	0.01701118	-	54832	grey	0.024144253	FALSE	VPS13C

	5						5	7	1	6						
Region_5988	chr15	62193542	62222911	29369	4	20	29.95454545	5.106351454	0.920249731	0.051846368	-	107984784	grey	-0.229435615	FALSE	LOC107984784
Region_5989	chr15	62227896	62270680	42784	5	20	36.06060606	6.743381457	0.840818288	0.081024181	-	255180	grey	0.218184223	FALSE	GOLGA2P11
Region_5990	chr15	62390526	62844631	454105	115	843	920.0454545	38.86364004	0.861246502	0.012105975	+	83660	grey	0.255081069	FALSE	TLN2
Region_5991	chr15	62827675	62884034	56359	13	92	117.9848485	11.00837782	0.939948641	0.030072095	-	105370854	grey	-0.120139195	FALSE	LOC105370854
Region_5992	chr15	63042632	63071915	29283	12	67	94.87878788	11.93510034	0.799563896	0.024177332	+	7168	grey	0.10193288	FALSE	TPM1
Region_5993	chr15	63121833	63142061	20228	4	13	23.15151515	5.18353139	0.972682042	0.042725231	+	114294	grey	-0.065391273	FALSE	LACTB
Region_5994	chr15	63125872	63158021	32149	6	30	46.01515152	6.878041815	0.817682049	0.021658473	-	51065	grey	-0.022668737	FALSE	RPS27L
Region_5995	chr15	63189560	63267776	78216	13	78	104.030303	10.50123758	0.761909559	0.028160619	+	51762	grey	0.089160477	FALSE	RAB8B
Region_5996	chr15	63276018	63309126	33108	12	52	78.86363636	10.22047171	0.643089746	0.020940901	+	83464	grey	-0.138080524	FALSE	APH1B
Region_5997	chr15	63321378	63381846	60468	23	168	197.6212121	13.88830737	0.933849109	0.020239651	-	771	grey	-0.370630959	FALSE	CA12
Region_5998	chr15	63390136	63438320	48184	16	106	126.0757576	9.539086565	0.879548434	0.026475989	+	102723344	grey	0.058104848	FALSE	LINC02568
Region_5999	chr15	63504511	63594640	90129	14	87	108.2424242	9.690694356	0.749855261	0.029341262	+	9960	grey	0.048011442	FALSE	USP3

Region_6000	chr15	63552912	63601589	48677	9	57	74.92424242	8.220631842	0.644312795	0.021397768	-	100130855	grey	0.150033025	FALSE	USP3-AS1
Region_6001	chr15	63597353	63602428	5075	3	16	27.60606061	4.87340913	0.011438926	0.019919374	+	283807	grey	0.019768705	FALSE	FBXL22
Region_6002	chr15	63608618	63833948	225330	44	304	341.0909091	17.83835735	0.912243947	0.01340067	-	8925	grey	-0.118872446	FALSE	HERC1
Region_6003	chr15	63907036	64072033	164997	30	203	237.4848485	16.48238447	0.758058029	0.026287099	-	23604	grey	0.134756035	FALSE	DAPK2
Region_6004	chr15	64072565	64093857	21292	5	22	35.18181818	5.377757871	0.964828926	0.031849875	-	84191	grey	0.05803369	FALSE	CIAO2A
Region_6005	chr15	64094123	64146090	51967	10	71	90.42424242	8.859693702	0.882797255	0.015912722	+	6642	grey	0.224350458	FALSE	SNX1
Region_6006	chr15	64151715	64157481	5766	4	17	28.62121212	5.229399588	0.603099626	0.071354387	+	79856	grey	0.066947157	FALSE	SNX22
Region_6007	chr15	64155740	64163134	7394	4	20	30.51515152	4.684647345	0.885169535	0.07488664	-	5479	grey	-0.10045305	FALSE	PPIB
Region_6008	chr15	64165525	64381420	215895	47	289	337.3181818	20.18389235	0.922991205	0.012779836	-	53944	grey	0.060414088	FALSE	CSNK1G1
Region_6009	chr15	64251545	64387687	136142	34	216	245.3636364	17.04982415	0.877751018	0.014631687	-	9768	grey	0.104641877	FALSE	PCLAF
Region_6010	chr15	64387748	64455303	67555	28	169	218.3484848	20.6147762	0.819210684	0.022547656	+	9325	grey	0.01722684	FALSE	TRIP4
Region_6011	chr15	64460578	64686068	225490	59	415	463.4242424	21.51068472	0.900807016	0.011506878	+	23060	grey	0.294437751	FALSE	ZNF609
Region_6012	chr1	64687573	64703281	15708	3	12	23.4393939	4.68427414	0.77240342	0.08479455	-	4947	grey	-0.10951714	FALSE	OAZ2

	5						4	3	2	9				8		
Region_6013	chr15	64739891	64775589	35698	19	110	136.6818182	11.87130877	0.827508494	0.027768499	-	348093	grey	0.250505484	FALSE	RBPMS2
Region_6014	chr15	64815632	64825668	10036	3	13	25.21212121	4.708470768	0.33027898	0.026707059	-	80119	grey	0.101069614	FALSE	PIF1
Region_6015	chr15	64841883	64930920	89037	38	275	309.1666667	18.08746556	0.791108622	0.035190338	+	80301	grey	-0.005814429	FALSE	PLEKHO2
Region_6016	chr15	64911902	64958691	46789	12	78	98.37878788	8.68732968	0.870175921	0.041320659	+	348094	grey	0.134983719	FALSE	ANKDD1A
Region_6017	chr15	64963022	64990310	27288	10	54	74.63636364	8.612399583	0.802991846	0.030407319	-	51324	grey	-0.217197302	FALSE	SPG21
Region_6018	chr15	65001512	65029639	28127	4	23	34.27272727	5.049129261	0.248785511	0.019423902	-	123263	grey	-0.047472821	FALSE	MTFMT
Region_6019	chr15	65045387	65053397	8010	7	32	46.6969697	6.268283281	0.836543105	0.057022031	+	123264	grey	-0.013260385	FALSE	SLC51B
Region_6020	chr15	65053337	65076690	23353	29	165	199.6363636	16.41259963	0.417883126	0.036536981	-	51285	grey	-0.163221847	FALSE	RASL12
Region_6021	chr15	65076746	65079948	3202	7	38	61.83333333	9.642188282	0.635665438	0.079505895	+	390594	grey	0.042040731	FALSE	KBTBD13
Region_6022	chr15	65092760	65115200	22440	10	43	63.75757576	9.62058787	0.945360001	0.031103795	-	390595	grey	0.268610071	FALSE	UBAP1L
Region_6023	chr15	65117379	65133808	16429	23	116	161.2121212	16.0456226	0.458146087	0.019318557	-	10081	grey	0.128057233	FALSE	PDCD7
Region_6024	chr15	65148219	65185342	37123	10	56	73.31818182	7.019005169	0.875457455	0.033560085	-	10845	grey	-0.085430944	FALSE	CLPX

Region_6025	chr15	65194760	65211473	16713	14	95	127.7575758	15.80755477	0.92374842	0.028725656	-	8483	grey	-0.298085154	FALSE	CILP
Region_6026	chr15	65234460	65300618	66158	34	234	273.8030303	17.46928707	0.730563931	0.016704227	-	54956	grey	0.170237266	FALSE	PARP16
Region_6027	chr15	65327127	65378002	50875	23	137	175.6666667	15.55470083	0.704230221	0.028619387	-	9543	grey	-0.229886772	FALSE	IGDCC3
Region_6028	chr15	65381484	65422947	41463	18	76	107.3333333	12.74644244	0.568834171	0.027292584	-	57722	grey	0.120654879	FALSE	IGDCC4
Region_6029	chr15	65442463	65517704	75241	31	207	243.8484848	15.52887378	0.90398814	0.013611842	-	54878	grey	0.074294494	FALSE	DPP8
Region_6030	chr15	65530418	65578349	47931	18	115	141.8181818	13.42547417	0.765013156	0.024902786	+	51495	grey	-0.152006308	FALSE	HACD3
Region_6031	chr15	65578753	65611289	32536	7	38	53	6.921538291	0.954583232	0.027900573	-	81556	grey	0.070399806	FALSE	INTS14
Region_6032	chr15	65611366	65660995	49629	18	132	153.8333333	11.23673007	0.895427732	0.023787919	+	9187	grey	0.177762802	FALSE	SLC24A1
Region_6033	chr15	65659123	65792293	133170	33	236	269.1363636	17.495394	0.882445847	0.024928224	-	10260	grey	0.32233322	FALSE	DENND4A
Region_6034	chr15	65726054	65891989	165935	54	375	420.0909091	25.42846816	0.793926134	0.021010667	+	8766	grey	0.092001212	FALSE	RAB11A
Region_6035	chr15	65792447	65877819	85372	35	235	272.5606061	19.45418981	0.776128691	0.021886573	+	105370866	grey	-0.038978661	FALSE	LOC105370866
Region_6036	chr15	66293217	66333898	40681	9	52	66.96969697	7.189671431	0.753099077	0.024675792	+	115752	grey	-0.297323244	FALSE	DIS3L
Region_6037	chr1	66336191	66386746	50555	18	107	135.772727	11.8305932	0.78323287	0.02548271	-	54962	grey	-0.26181548	FALSE	TIPIN

	5						3	7	6	1				9		
Region_6038	chr15	66386837	66491656	104819	38	254	292.2727273	17.18455967	0.820498514	0.018813244	+	5604	grey	0.113783748	FALSE	MAP2K1
Region_6039	chr15	66488658	66497780	9122	3	15	27.34848485	5.38799927	0.49158203	0.079284076	-	10302	grey	0.267106489	FALSE	SNAPC5
Region_6040	chr15	66498015	66524532	26517	10	66	84.66666667	9.244263641	0.843947708	0.029522048	-	6124	grey	-0.05102147	FALSE	RPL4
Region_6041	chr15	66504959	66550130	45171	19	114	152.5454545	14.4947543	0.918642482	0.017383587	+	55055	grey	0.019980651	FALSE	ZWILCH
Region_6042	chr15	66547179	66565998	18819	5	29	38.78787879	4.672390762	0.951373374	0.049393599	-	197021	grey	-0.107081316	FALSE	LCTL
Region_6043	chr15	66582190	66685794	103604	61	372	430.6515152	25.34530296	0.845366157	0.021163866	+	102723165	grey	0.095896878	FALSE	LINC01169
Region_6044	chr15	66702236	66782849	80613	30	214	249.6515152	15.80310819	0.754790074	0.020528227	+	4091	grey	-0.075647009	FALSE	SMAD6
Region_6045	chr15	66931537	66956518	24981	8	45	64.53030303	8.532687731	0.856084024	0.053270689	-	102723481	grey	0.364796243	FALSE	SMASR
Region_6046	chr15	66984103	67065268	81165	34	231	271.030303	18.6489593	0.775510047	0.017259434	-	102723493	grey	0.183578191	FALSE	SMAD3-DT
Region_6047	chr15	67063763	67195173	131410	60	443	489.8030303	23.34373228	0.66972518	0.017511701	+	4088	grey	-0.16323337	FALSE	SMAD3
Region_6048	chr15	67200667	67255195	54528	18	96	153.1212121	13.53913433	0.663240332	0.023364286	-	79719	grey	0.100518941	FALSE	AAGAB
Region_6049	chr15	67254786	67502260	247474	54	389	451.6212121	23.85840613	0.931368653	0.014647357	+	64799	grey	-0.067047924	FALSE	IQCH

Region_6050	chr15	67290636	67521844	231208	55	399	457.9242424	23.3806696	0.891191501	0.014747045	-	100506686	grey	0.049899944	FALSE	IQCH-AS1
Region_6051	chr15	67521131	67530146	9015	4	27	40.12121212	6.924905223	0.24625639	0.015647647	+	145853	grey	-0.114000134	FALSE	C15orf61
Region_6052	chr15	67542703	67807117	264414	35	268	312.9393939	17.71907168	0.870068143	0.016302014	+	5607	grey	-0.139885049	FALSE	MAP2K5
Region_6053	chr15	67819704	67873866	54162	41	279	325.6969697	22.27034572	0.114564592	0.013224028	+	390598	grey	-0.261022047	FALSE	SKOR1
Region_6054	chr15	67834310	67838879	4569	13	73	102.8181818	12.41699715	0.021102555	0.019276067	-	101929076	grey	0.087910733	FALSE	SKOR1-AS1
Region_6055	chr15	68054309	68193847	139538	37	280	316.5	14.93395718	0.874069007	0.011621368	+	8554	grey	0.43696431	FALSE	PIAS1
Region_6056	chr15	68184032	68229718	45686	8	35	56.22727273	8.286774365	0.580198224	0.046838792	-	91860	grey	0.099866878	FALSE	CALML4
Region_6057	chr15	68206992	68257211	50219	10	42	64.01515152	9.032407336	0.663074591	0.045035179	-	54982	grey	0.042512931	FALSE	CLN6
Region_6058	chr15	68277745	68295862	18117	10	49	69.75757576	8.490828728	0.348905068	0.028632695	+	10116	grey	-0.031249775	FALSE	FEM1B
Region_6059	chr15	68296532	68432163	135631	32	198	230.3939394	14.61914318	0.865832923	0.028404454	-	22801	grey	-0.194529277	FALSE	ITGA11
Region_6060	chr15	68578993	68727806	148813	43	277	307.8181818	17.19115076	0.81716459	0.022626101	+	10391	grey	-0.37764107	FALSE	CORO2B
Region_6061	chr15	68778535	68820897	42362	16	103	132.8939394	12.64561802	0.741670242	0.02968554	-	8125	grey	-0.26536405	FALSE	ANP32A
Region_6062	chr1	68818221	68946811	128590	30	223	259.090909	18.5221092	0.77843853	0.02529829	+	246777	grey	-0.11210656	FALSE	SPESP1

	5						1	6	1	4				3		
Region_6063	chr15	68930504	69062762	132258	31	231	271.8939394	20.40673251	0.931528735	0.015023509	+	79400	grey	-0.366055802	FALSE	NOX5
Region_6064	chr15	69072926	69099987	27061	11	58	78	9.033271833	0.578642848	0.03462619	+	283673	grey	-0.309910375	FALSE	EWSAT1
Region_6065	chr15	69160584	69272217	111633	14	85	114.2575758	11.88445734	0.738974553	0.019374862	+	26035	grey	-0.209121896	FALSE	GLCE
Region_6066	chr15	69278328	69298795	20467	4	17	30.09090909	5.419983743	0.901060955	0.077937999	-	102723548	grey	0.152631031	FALSE	PAQR5-DT
Region_6067	chr15	69278675	69407780	129105	34	212	252.3939394	17.39219877	0.924929788	0.023544709	+	54852	grey	0.007436697	FALSE	PAQR5
Region_6068	chr15	69396904	69415029	18125	7	38	56.95454545	8.004675207	0.386965713	0.038489795	-	145694	grey	0.243534723	FALSE	KIF23-AS1
Region_6069	chr15	69414246	69448427	34181	6	38	53.40909091	8.875163743	0.364773432	0.032092528	+	9493	grey	0.125163826	FALSE	KIF23
Region_6070	chr15	69452814	69456205	3391	11	78	98.22727273	11.99075985	0.053553819	0.024025755	+	6176	grey	0.085106451	FALSE	RPLP1
Region_6071	chr15	69462921	69695750	232829	45	311	363.9242424	21.25327892	0.873813239	0.019636167	+	145837	grey	0.260183562	FALSE	DRAIC
Region_6072	chr15	69570785	69840828	270043	62	463	520.8333333	23.49964539	0.812527614	0.01778134	+	104472713	grey	0.159020979	FALSE	PCAT29
Region_6073	chr15	69823478	69843120	19642	5	33	46.66666667	6.107582509	0.872807218	0.05328303	+	414926	grey	-0.177293316	FALSE	LINC00593
Region_6074	chr15	70047790	70098176	50386	11	54	80.1969697	9.784162551	0.576520272	0.033778619	-	7090	grey	-0.006297268	FALSE	TLE3

Region_6075	chr15	70570958	70586606	15648	4	20	34.48484848	5.364390923	0.944180737	0.037753237	-	101929151	grey	-0.004901625	FALSE	LINC02204
Region_6076	chr15	70654554	70778868	124314	23	139	173.7575758	14.26891253	0.771026316	0.026523978	-	55075	grey	-0.28245892	FALSE	UACA
Region_6077	chr15	70829130	70854157	25027	6	33	48.5	7.642492545	0.917605217	0.047137178	-	55323	grey	0.203429099	FALSE	LARP6
Region_6078	chr15	70853239	71053658	200419	22	132	160.2121212	13.00356774	0.807769323	0.029964132	+	54839	grey	0.141076116	FALSE	LRRC49
Region_6079	chr15	71096952	71783383	686431	108	794	884.3484848	40.8725947	0.826681871	0.014356945	+	79875	grey	-0.120328202	FALSE	THSD4
Region_6080	chr15	71147650	71189064	41414	7	35	55.6969697	7.350499087	0.923193434	0.042866752	-	101929196	grey	-0.102957423	FALSE	THSD4-AS1
Region_6081	chr15	71792638	71818259	25621	5	20	34.03030303	6.798121227	0.751021464	0.040253858	+	10002	grey	0.067735707	FALSE	NR2E3
Region_6082	chr15	71822291	72118577	296286	37	246	296.1363636	19.09964669	0.930891312	0.01444494	-	4649	grey	0.10118592	FALSE	MYO9A
Region_6083	chr15	71972206	72040265	68059	10	56	76	8.246211251	0.964348147	0.017772514	+	107984714	grey	-0.009641202	FALSE	LOC107984714
Region_6084	chr15	72114258	72143692	29434	3	15	22.68181818	4.239590296	0.342816891	0.059091376	+	123228	grey	0.044396736	FALSE	SENP8
Region_6085	chr15	72159806	72197787	37981	4	17	27.21212121	4.42202582	0.94280303	0.040683024	-	196996	grey	0.075578648	FALSE	GRAMD2A
Region_6086	chr15	72199029	72231819	32790	9	52	69.25757576	8.856654354	0.450319087	0.046473011	-	5315	grey	0.090737537	FALSE	PKM
Region_6087	chr1	72241181	72272999	31818	6	27	43.2121212	7.32858502	0.48577487	0.02807424	-	56965	grey	0.114006801	FALSE	PARP6

	5						1	8	2	4						
Region_6088	chr15	72266746	72375981	109235	23	149	190.4545455	12.6602732	0.733188204	0.017700432	-	60677	grey	-0.050563587	FALSE	CELF6
Region_6089	chr15	72278867	72351794	72927	16	101	127.9393939	10.48937747	0.867691203	0.025161566	+	105370887	grey	-0.078318424	FALSE	LOC105370887
Region_6090	chr15	72340924	72376420	35496	9	56	77.18181818	8.666308044	0.703676778	0.030501977	-	3073	grey	-0.10560967	FALSE	HEXA
Region_6091	chr15	72407778	72474258	66480	16	85	109.2878788	11.78756209	0.90380697	0.037846133	-	105370888	grey	0.252211052	FALSE	TMEM202-AS1
Region_6092	chr15	72474330	72602987	128657	38	237	289.6212121	20.38005447	0.844865157	0.01410707	+	25820	grey	0.461671846	FALSE	ARIH1
Region_6093	chr15	72608481	72636962	28481	9	36	60.57575758	9.016328105	0.888953911	0.042566771	-	102723640	grey	-0.422445416	FALSE	LINC02259
Region_6094	chr15	72654697	72669599	14902	4	27	45.78787879	9.394786036	0.805831003	0.060226321	+	55889	grey	-0.210145278	FALSE	GOLGA6B
Region_6095	chr15	72686179	72738475	52296	11	72	90.03030303	10.29558486	0.776448195	0.024277698	+	585	grey	-0.008753118	FALSE	BBS4
Region_6096	chr15	72751294	72785846	34552	13	87	111.7575758	9.949194952	0.648685409	0.02755402	-	83440	grey	0.0308996	FALSE	ADPGK
Region_6097	chr15	72782835	72798199	15364	13	71	99.96969697	10.87547657	0.322868201	0.029348229	+	100287559	grey	-0.079186549	FALSE	ADPGK-AS1
Region_6098	chr15	73051710	73305205	253495	36	263	301.8636364	17.1741186	0.800450954	0.028495092	+	4756	grey	-0.289761965	FALSE	NEO1
Region_6099	chr15	73319859	73368958	49099	18	107	141.6818182	14.45434094	0.793787432	0.032377756	-	10021	grey	-0.135128445	FALSE	HCN4

Region_6100	chr15	73443164	73560013	116849	10	52	83.1969697	13.21912332	0.919073641	0.035875645	+	283677	grey	0.11928683	FALSE	REC114
Region_6101	chr15	73560014	73634134	74120	13	90	108.4393939	9.377762219	0.82279736	0.030748087	-	27020	grey	-0.238567001	FALSE	NPTN
Region_6102	chr15	73683966	73714514	30548	3	11	23.12121212	4.53198938	0.747799799	0.109802647	+	80381	grey	-0.116201201	FALSE	CD276
Region_6103	chr15	73735458	73753351	17893	4	23	36.89393939	5.087325995	0.793081793	0.070530674	-	388135	grey	-0.018706686	FALSE	INSYN1
Region_6104	chr15	73752317	73770613	18296	4	20	28.07575758	4.896814054	0.873776919	0.070244195	+	101929221	grey	-0.008067133	FALSE	INSYN1-AS1
Region_6105	chr15	73908071	73928248	20177	7	20	42.96969697	7.8504673	0.405905326	0.040412233	-	100287616	grey	-0.07095489	FALSE	LOXL1-AS1
Region_6106	chr15	73925989	73952137	26148	7	37	51	7.129570059	0.419670496	0.041536282	+	4016	grey	-0.016977354	FALSE	LOXL1
Region_6107	chr15	73978926	73994622	15696	6	31	43.59090909	6.981243603	0.350431624	0.038375683	-	9399	grey	-0.009853968	FALSE	STOML1
Region_6108	chr15	73994673	74047827	53154	23	143	184.2272727	13.29290783	0.819673774	0.018516059	+	5371	grey	0.011501749	FALSE	PML
Region_6109	chr15	74100311	74138540	38229	14	93	114.0909091	10.96810379	0.372527813	0.031922789	+	57611	grey	-0.161376755	FALSE	ISLR2
Region_6110	chr15	74126373	74129341	2968	7	36	51.43939394	7.469175195	0.301657488	0.043155695	-	283731	grey	-0.038038718	FALSE	LOC283731
Region_6111	chr15	74541206	74598131	56925	21	121	151.6818182	11.86612385	0.847645206	0.019849125	+	10620	grey	0.111709083	FALSE	ARID3B
Region_6112	chr1	74608372	74645414	37042	15	109	133.681818	11.0190870	0.59325531	0.02444610	+	1198	grey	-0.08631626	FALSE	CLK3

	5						2	6	4	5				5		
Region_6113	chr15	74630558	74696292	65734	24	199	227.969697	14.851024	0.853530143	0.019187929	-	80153	grey	-0.154701211	FALSE	EDC3
Region_6114	chr15	74748845	74756607	7762	4	16	26.3030303	4.275042769	0.877363987	0.070136211	+	1544	grey	-0.111514044	FALSE	CYP1A2
Region_6115	chr15	74782080	74803197	21117	9	44	65.10606061	9.111495339	0.538036689	0.063444858	+	1445	grey	0.187847991	FALSE	CSK
Region_6116	chr15	74812716	74831233	18517	10	62	81.16666667	7.999198678	0.642841851	0.040986921	+	79748	grey	-0.11102831	FALSE	LMAN1L
Region_6117	chr15	74821074	74831802	10728	7	41	53.31818182	6.69824108	0.519242944	0.051807756	+	594855	grey	-0.125098638	FALSE	CPLX3
Region_6118	chr15	74836118	74843346	7228	9	42	62.83333333	9.450410383	0.511404538	0.029718857	-	25989	grey	0.341634552	FALSE	ULK3
Region_6119	chr15	74843730	74873365	29635	13	72	88.90909091	9.396770759	0.664064895	0.050110654	-	10066	grey	-0.008025697	FALSE	SCAMP2
Region_6120	chr15	74890005	74902219	12214	3	12	19.89393939	4.382232616	0.979727778	0.031490884	+	4351	grey	-0.101823849	FALSE	MPI
Region_6121	chr15	74899992	74906883	6891	8	30	51	9.7491617	0.107699636	0.022661977	-	57184	grey	-0.144577893	FALSE	FAM219B
Region_6122	chr15	74919791	74938083	18292	11	50	70.62121212	8.227094359	0.801600759	0.03020525	-	9377	grey	-0.018589472	FALSE	COX5A
Region_6123	chr15	74957219	75021495	64276	30	185	224.7878788	16.7318353	0.747753945	0.019633363	+	192683	grey	-0.090144655	FALSE	SCAMP5
Region_6124	chr15	75023586	75117462	93876	39	218	272.0909091	19.06969184	0.823451737	0.015960765	+	60490	grey	0.107424067	FALSE	PPCDC

Region_6125	chr15	75195643	75212169	16526	19	120	138.8030303	11.29495421	0.586573753	0.028696056	+	56905	grey	0.056974362	FALSE	C15orf39
Region_6126	chr15	75226401	75234834	8433	9	53	71.86363636	10.82289079	0.812302773	0.056543984	-	105376731	grey	-0.082322956	FALSE	LOC105376731
Region_6127	chr15	75336020	75343224	7204	3	12	19.07575758	3.591916592	0.468486998	0.079244716	+	54939	grey	0.22909673	FALSE	COMMD4
Region_6128	chr15	75346955	75357115	10160	5	21	37.07575758	6.269119938	0.468375915	0.048809674	+	79661	grey	0.051172976	FALSE	NEIL1
Region_6129	chr15	75355792	75368612	12820	3	11	19.68181818	4.046495505	0.946549068	0.061194922	-	4123	grey	-0.239788092	FALSE	MAN2C1
Region_6130	chr15	75369379	75455842	86463	33	219	257.2727273	15.82650693	0.652713096	0.008262215	-	25942	grey	0.252065164	FALSE	SIN3A
Region_6131	chr15	75463251	75579315	116064	44	301	353.3939394	21.51913552	0.888761049	0.016823316	-	5780	grey	0.112813213	FALSE	PTPN9
Region_6132	chr15	75527150	75626469	99319	36	229	272.4848485	21.08535213	0.757544549	0.020297744	-	10073	grey	0.285931347	FALSE	SNUPN
Region_6133	chr15	75639093	75640224	1131	11	99	141.0454545	15.78649064	0.0234202	0.015033148	-	55272	grey	-0.061417671	FALSE	IMP3
Region_6134	chr15	75647912	75662301	14389	9	53	73.57575758	8.663025952	0.718323042	0.028011548	+	257364	grey	0.277668706	FALSE	SNX33
Region_6135	chr15	75674322	75712848	38526	20	112	141.0757576	12.44224888	0.786606823	0.024740892	-	1464	grey	0.159708041	FALSE	CSPG4
Region_6136	chr15	75727670	75738629	10959	4	10	22.75757576	5.979607927	0.366468794	0.123633476	-	100128285	grey	-0.20466541	FALSE	DNM1P35
Region_6137	chr1	75843307	75901078	57771	19	93	132.166666	12.5423385	0.75205318	0.02813378	+	92912	grey	0.168060321	FALSE	UBE2Q2

	5						7	5	3	8						
Region_6138	chr15	75903876	75942511	38635	18	147	178.1515152	15.22876067	0.763444368	0.016469763	+	26263	grey	-0.124287635	FALSE	FBXO22
Region_6139	chr15	75935969	76059795	123826	33	235	279.3030303	18.65473312	0.871797624	0.018864422	-	145957	grey	-0.104299455	FALSE	NRG4
Region_6140	chr15	76059958	76229121	169163	37	267	306.3787879	19.53754976	0.873896401	0.023604299	+	123591	grey	0.091643546	FALSE	TMEM266
Region_6141	chr15	76174891	76181486	6595	4	20	31.89393939	5.150440722	0.716706008	0.078634677	-	101929439	grey	0.100896234	FALSE	LOC101929439
Region_6142	chr15	76188555	76311730	123175	19	118	156.7121212	12.00738895	0.770211987	0.019452111	-	2108	grey	0.105606343	FALSE	ETFA
Region_6143	chr15	76336773	76342475	5702	6	37	54.86363636	7.575032599	0.019601674	0.021006001	+	64843	grey	-0.006436556	FALSE	ISL2
Region_6144	chr15	76347904	76905444	557540	63	470	520.4090909	22.70816679	0.924069204	0.01283159	-	49855	grey	0.013659944	FALSE	SCAPER
Region_6145	chr15	76993359	77037475	44116	8	47	62.40909091	6.686328814	0.834957945	0.026460004	+	9051	grey	0.292190449	FALSE	PSTPIP1
Region_6146	chr15	77041404	77083984	42580	6	34	48.5	7.730558742	0.578320237	0.044291905	-	10099	grey	-0.019170208	FALSE	TSPAN3
Region_6147	chr15	77100656	77420144	319488	45	333	380.7878788	23.70687349	0.918180283	0.010886207	-	79834	grey	0.302765809	FALSE	PEAK1
Region_6148	chr15	77420412	77485603	65191	8	55	79.92424242	9.550369475	0.925329066	0.025004894	+	10363	grey	0.03705474	FALSE	HMG20A
Region_6149	chr15	77568970	77608888	39918	9	42	58.98484848	7.132789775	0.837040417	0.058303622	+	105370906	grey	0.164221897	FALSE	LOC105370906

Region_6150	chr15	77613027	77820900	207873	64	380	449.8939394	28.39725273	0.724913551	0.023563354	-	84894	grey	-0.109928344	FALSE	LINGO1
Region_6151	chr15	77914217	77926846	12629	4	19	34.31818182	6.682144038	0.816355781	0.060662568	-	645752	grey	-0.176323341	FALSE	GOLGA6FP
Region_6152	chr15	77984036	78077724	93688	28	183	214.2121212	13.85475775	0.852457343	0.027053409	-	23102	grey	-0.399280304	FALSE	TBC1D2B
Region_6153	chr15	78077808	78104370	26562	7	46	63.48484848	7.277451845	0.890662883	0.052957807	+	646892	grey	0.11706184	FALSE	SH2D7
Region_6154	chr15	78104606	78131535	26929	11	54	74.59090909	8.30009689	0.699472984	0.050829792	-	10518	grey	-0.149382601	FALSE	CIB2
Region_6155	chr15	78131498	78171945	40447	16	91	113.7575758	10.28675116	0.709103773	0.021798679	+	3419	grey	0.246398507	FALSE	IDH3A
Region_6156	chr15	78167468	78245688	78220	41	301	341.0909091	20.27786005	0.877533005	0.016375689	-	23205	grey	0.060818056	FALSE	ACSBG1
Region_6157	chr15	78250502	78264156	13654	6	40	52.86363636	7.168062422	0.89535633	0.064626214	-	105370911	grey	0.12305722	FALSE	DNAJA4-DT
Region_6158	chr15	78264086	78282196	18110	12	67	94.3030303	11.70005877	0.713354135	0.021307429	+	55466	grey	-0.030449415	FALSE	DNAJA4
Region_6159	chr15	78277835	78299703	21868	9	58	81.59090909	9.46243955	0.961157625	0.023849091	-	80349	grey	-0.126835067	FALSE	SKIC8
Region_6160	chr15	78437431	78501453	64022	20	109	150.8484848	12.66034685	0.620958484	0.022169609	+	3658	grey	0.079859065	FALSE	IREB2
Region_6161	chr15	78507564	78537372	29808	11	69	88.81818182	9.80252572	0.75898259	0.038692459	+	123688	grey	-0.139770465	FALSE	HYKK
Region_6162	chr1	78565520	78595269	29749	9	54	73.4090909	8.90631111	0.72671763	0.04173239	+	1138	grey	-0.09188486	FALSE	CHRNA5

	5						1	2	1	8						
Region_6163	chr15	78593052	78621295	28243	7	38	53.01515152	7.65303846	0.780868738	0.046336506	-	1136	grey	0.133700006	FALSE	CHRNA3
Region_6164	chr15	78624111	78720286	96175	25	154	190.1818182	14.61601765	0.86452499	0.022488073	-	1143	grey	-0.045247372	FALSE	CHRNB4
Region_6165	chr15	78660644	78729770	69126	18	108	138.9545455	11.46155019	0.889369922	0.026403585	+	112268142	grey	-0.228555716	FALSE	
Region_6166	chr15	78686918	78727754	40836	8	47	63.1969697	8.071258975	0.917444721	0.041063266	-	105370913	grey	-0.221978675	FALSE	LOC105370913
Region_6167	chr15	78759206	78811464	52258	24	122	158.969697	15.0342887	0.797425268	0.039132757	-	11173	grey	-0.118587485	FALSE	ADAMTS7
Region_6168	chr15	78810487	78898139	87652	38	234	274.030303	20.89973846	0.754675059	0.022170606	+	10933	grey	0.005977938	FALSE	MORF4L1
Region_6169	chr15	78921058	78949574	28516	12	69	91.39393939	11.68019861	0.840373604	0.037149352	-	1512	grey	-0.257988491	FALSE	CTSH
Region_6170	chr15	78959906	79090780	130874	41	271	312.6212121	18.683407	0.754274715	0.024139105	-	5923	grey	-0.084419471	FALSE	RASGRF1
Region_6171	chr15	78978889	78985926	7037	3	11	17.48484848	3.915422855	0.658055889	0.095773471	+	100129540	grey	-0.272696706	FALSE	LOC100129540
Region_6172	chr15	79123939	79283964	160025	41	326	365.1363636	17.91766328	0.76918272	0.02784596	-	729911	grey	0.379729696	FALSE	ANKRD34C-AS1
Region_6173	chr15	79282722	79298239	15517	5	36	51.36363636	6.26009674	0.690126228	0.043479797	+	390616	grey	-0.028694112	FALSE	ANKRD34C
Region_6174	chr15	79311112	79427432	116320	20	134	179.4393939	14.96984537	0.863961803	0.029286058	+	23423	grey	0.119085061	FALSE	TMED3

Region_6175	chr15	79432336	79472304	39968	8	52	78.83333333	11.04199443	0.699900582	0.050529658	+	23251	grey	0.41376134	FALSE	MINAR1
Region_6176	chr15	79843547	79897379	53832	3	20	32.31818182	5.174372027	0.64356935	0.03440043	-	10588	grey	0.120998099	FALSE	MTHFS
Region_6177	chr15	79845150	79923754	78604	7	44	60.68181818	8.069237102	0.436486413	0.032464135	-	100528021	grey	0.191003859	FALSE	ST20-MTHFS
Region_6178	chr15	79898840	79923702	24862	4	15	28.36363636	6.055223717	0.279601497	0.048809882	-	400410	grey	0.223241277	FALSE	ST20
Region_6179	chr15	79922771	79926993	4222	5	25	35.81818182	4.748794473	0.572062399	0.031417705	+	283687	grey	-0.143112294	FALSE	ST20-AS1
Region_6180	chr15	80059568	80138393	78825	12	88	105.530303	8.132025383	0.821915168	0.03201668	+	54469	grey	0.062469651	FALSE	ZFAND6
Region_6181	chr15	80152490	80186946	34456	9	54	70.62121212	7.709664567	0.845545382	0.027899075	+	2184	grey	0.111468677	FALSE	FAH
Region_6182	chr15	80195481	80252213	56732	31	230	266.4242424	17.06553937	0.77575296	0.019239135	-	100996492	grey	0.255650478	FALSE	CTXND1
Region_6183	chr15	80263068	80341805	78737	18	120	143.7424242	13.03641126	0.892951951	0.032924067	-	283688	grey	0.11673897	FALSE	LINC00927
Region_6184	chr15	80344853	80404214	59361	6	31	47.13636364	7.137951362	0.942268003	0.034913187	-	101929560	grey	-0.457476838	FALSE	ARNT2-DT
Region_6185	chr15	80404350	80597933	193583	39	288	334.5454545	18.909649	0.806971112	0.024268565	+	9915	grey	0.14425541	FALSE	ARNT2
Region_6186	chr15	80679684	80755621	75937	23	166	200.0454545	14.67329528	0.744243927	0.02219999	+	58489	grey	-0.022950838	FALSE	ABHD17C
Region_6187	chr1	80779343	80951776	172433	36	291	327.469697	18.5025733	0.80809751	0.01966763	+	57214	grey	-0.04684898	FALSE	CEMIP

	5							7	5	1				7		
Region_6188	chr15	80896190	80948867	52677	12	97	118.8030303	10.25253394	0.840068121	0.024934519	-	107984749	grey	-0.065421999	FALSE	
Region_6189	chr15	80946289	80989828	43539	17	105	134.0909091	11.93537376	0.735208539	0.037484407	-	23184	grey	0.016996334	FALSE	MESD
Region_6190	chr15	81000923	81005788	4865	3	14	23.10606061	4.598089195	0.658433177	0.020462408	+	59274	grey	0.183014101	FALSE	TLNRD1
Region_6191	chr15	81007033	81149179	142146	29	213	243.7575758	14.96771973	0.755306944	0.025008679	+	161502	grey	0.18383291	FALSE	CFAP161
Region_6192	chr15	81182600	81314058	131458	35	271	310.4090909	15.44317352	0.694147726	0.022430922	+	3603	grey	-0.171572563	FALSE	IL16
Region_6193	chr15	81309053	81324183	15130	4	24	38.87878788	6.301440987	0.564756082	0.067740671	-	80765	grey	-0.091228659	FALSE	STARD5
Region_6194	chr15	81324338	81516976	192638	24	173	202.9242424	14.29503476	0.908876597	0.020028409	+	101929655	grey	-0.046149475	FALSE	TMC3-AS1
Region_6195	chr15	81331088	81374213	43125	8	47	66.53030303	7.380264112	0.953240461	0.032757459	-	342125	grey	0.003068568	FALSE	TMC3
Region_6196	chr15	81554003	81696780	142777	29	228	263.2727273	14.72365491	0.906584549	0.023415412	+	105370921	grey	0.092023888	FALSE	LOC105370921
Region_6197	chr15	81633426	81871125	237699	31	211	250.8636364	18.70901581	0.891009443	0.028322532	-	102723985	grey	-0.069072673	FALSE	LOC102723985
Region_6198	chr15	81816761	81995666	178905	23	152	187.5909091	13.81635293	0.91513098	0.023570712	-	102724001	grey	-0.212008376	FALSE	LINC01418
Region_6199	chr15	81915347	82013579	98232	15	88	111.5	10.30944302	0.936468061	0.02833752	-	105370922	grey	0.018575186	FALSE	LOC105370922

Region_6200	chr15	82041778	82046018	4240	9	44	60.12121212	8.799501999	0.517978379	0.040575869	-	84206	grey	-0.129561912	FALSE	MEX3B
Region_6201	chr15	82130206	82262773	132567	21	143	169.7575758	13.65741343	0.776185245	0.022157595	-	79631	grey	-0.097393407	FALSE	EFL1
Region_6202	chr15	82262810	82284930	22120	3	14	26.51515152	5.06038133	0.944550175	0.059333112	+	283726	grey	-0.251690056	FALSE	SAXO2
Region_6203	chr15	82647770	82692820	45050	27	133	190.4848485	25.92963403	0.369452428	0.022784929	+	283692	grey	-0.182363777	FALSE	CPEB1-AS1
Region_6204	chr15	82659281	82710112	50831	11	55	73.39393939	9.306538294	0.708666685	0.03212031	-	8120	grey	-0.193445911	FALSE	AP3B2
Region_6205	chr15	82725873	82738904	13031	10	54	73.40909091	9.883906535	0.969208344	0.02263551	-	283693	grey	0.196807519	FALSE	ACTG1P17
Region_6206	chr15	82755362	82806070	50708	22	131	162.6060606	16.21953123	0.91620539	0.02289149	-	123722	grey	-0.221127048	FALSE	FSD2
Region_6207	chr15	82809628	82836108	26480	14	86	110.3333333	10.73217066	0.422218333	0.016962705	+	123720	grey	-0.207033208	FALSE	WHAMM
Region_6208	chr15	82836946	82986153	149207	32	221	262.8939394	16.40649851	0.908178102	0.017164761	-	9455	grey	0.152279295	FALSE	HOMER2
Region_6209	chr15	82988441	83011641	23200	5	18	35.90909091	6.121154423	0.707190867	0.062694546	-	123207	grey	0.082569963	FALSE	C15orf40
Region_6210	chr15	83016423	83067252	50829	7	39	52.40909091	6.813961398	0.543186945	0.036862963	-	53339	grey	0.323522001	FALSE	BTBD1
Region_6211	chr15	83107572	83144854	37282	12	76	104.2424242	10.24178561	0.803092648	0.020205516	+	53346	grey	0.114161412	FALSE	TM6SF1
Region_6212	chr1	83112738	83207823	95085	20	131	170.015151	13.6376091	0.93619542	0.02207361	-	50810	grey	0.286818687	FALSE	HDGFL3

	5						5	1	6	4						
Region_6213	chr15	83255884	83284664	28780	5	31	47.33333333	7.262831263	0.727219597	0.036962063	-	646	grey	0.059304614	FALSE	BNC1
Region_6214	chr15	83447228	83618743	171515	34	213	257.3484848	15.29252449	0.802427958	0.025318521	+	6457	grey	0.114489219	FALSE	SH3GL3
Region_6215	chr15	83654088	84039842	385754	74	562	633.3333333	28.68895973	0.797119037	0.015703762	+	57188	grey	0.195178068	FALSE	ADAMTSL3
Region_6216	chr15	84080168	84165291	85123	17	113	140.6060606	10.13733205	0.884356953	0.029502906	+	648809	grey	0.064619765	FALSE	EFL1P1
Region_6217	chr15	84526781	84571216	44435	8	40	62.98484848	8.406027918	0.604896376	0.024369537	-	388165	grey	-0.165168768	FALSE	UBE2Q2P1
Region_6218	chr15	84570649	84580175	9526	5	30	48.25757576	6.737519758	0.584375778	0.029981627	+	100506874	grey	-0.031958727	FALSE	LINC00933
Region_6219	chr15	84597809	84633988	36179	7	42	52.12121212	6.3452348	0.818574433	0.032569938	-	105370947	grey	-0.143876463	FALSE	LOC105370947
Region_6220	chr15	84600986	84623717	22731	6	32	41.62121212	5.185082602	0.953348973	0.037754664	+	54993	grey	-0.140893346	FALSE	ZSCAN2
Region_6221	chr15	84631451	84647478	16027	3	18	30.40909091	5.05957515	0.961754277	0.036771324	+	54581	grey	0.098950536	FALSE	SCAND2P
Region_6222	chr15	84639285	84654343	15058	4	25	40.33333333	6.926722687	0.977196911	0.024935579	-	84942	grey	0.160350553	FALSE	WDR73
Region_6223	chr15	84669538	84716460	46922	8	44	58.37878788	6.82250834	0.724762598	0.024209799	-	23478	grey	0.025390697	FALSE	SEC11A
Region_6224	chr15	84748592	84806445	57853	19	92	123.5909091	15.1576102	0.461147421	0.025985599	+	9640	grey	0.067673358	FALSE	ZNF592

Region_6225	chr15	84817356	84873479	56123	20	138	162.3181818	12.97590559	0.926000626	0.021731888	+	57538	grey	0.092105669	FALSE	ALPK3
Region_6226	chr15	84884654	84945798	61144	21	120	150.9848485	12.84881608	0.908011889	0.0360336	+	9154	grey	0.360735138	FALSE	SLC28A1
Region_6227	chr15	84980440	85139145	158705	21	119	161.9090909	16.9911536	0.427876944	0.015377581	+	5151	grey	0.168717395	FALSE	PDE8A
Region_6228	chr15	85180200	85234795	54595	10	24	62.27272727	15.68590298	0.847169647	0.043586014	-	101929479	grey	-0.057318587	FALSE	LOC101929479
Region_6229	chr15	85380571	85749358	368787	91	685	768.1969697	35.85578794	0.818235523	0.014294824	+	11214	grey	-0.004676897	FALSE	AKAP13
Region_6230	chr15	85759326	85794925	35599	20	141	171	13.83195854	0.707671879	0.020350744	-	64410	grey	0.194255049	FALSE	KLHL25
Region_6231	chr15	86078809	86116747	37938	6	34	49.45454545	6.716642518	0.728833174	0.055787986	-	101929701	grey	-0.057033404	FALSE	LINC01584
Region_6232	chr15	86079871	87049169	969298	108	802	900.1515152	30.04419433	0.818796307	0.017092819	+	123624	grey	0.04237333	FALSE	AGBL1
Region_6233	chr15	86295649	86317173	21524	7	40	57.66666667	7.372158297	0.839641651	0.043704773	-	727915	grey	0.003096402	FALSE	AGBL1-AS1
Region_6234	chr15	86932880	86946331	13451	4	23	35.53030303	4.94343293	0.799434776	0.083928827	+	105370954	grey	0.020293814	FALSE	LOC105370954
Region_6235	chr15	86938797	86988426	49629	5	26	40.24242424	5.580351812	0.820013813	0.085647005	-	102724452	grey	0.109077829	FALSE	LOC102724452
Region_6236	chr15	87325829	87703825	377996	28	216	244.5606061	15.74814156	0.846304677	0.033464752	-	105370956	grey	-0.217247439	FALSE	
Region_6237	chr1	87432058	87703852	271794	19	137	161.348484	12.3132104	0.87391883	0.03522267	-	10272446	grey	0.140344558	FALSE	LOC102724465

	5						8	5	9	1		5				
Region_6238	chr15	87859751	88256791	397040	44	328	363.5454545	19.01835698	0.812563648	0.025355031	-	4916	grey	-0.09828527	FALSE	NTRK3
Region_6239	chr15	88252730	88271066	18336	3	12	22.89393939	4.936921457	0.818193142	0.071698162	+	283738	grey	-0.012761195	FALSE	NTRK3-AS1
Region_6240	chr15	88459477	88511521	52044	13	79	111.1666667	11.20931389	0.818530786	0.022521169	-	26589	grey	0.165663411	FALSE	MRPL46
Region_6241	chr15	88459501	88546681	87180	17	111	146	13.34627577	0.84503425	0.020181781	-	55070	grey	0.196446874	FALSE	DET1
Region_6242	chr15	88585566	88605110	19544	5	13	31.06060606	8.5392826	0.202382319	0.060286356	-	101929743	grey	-0.158774283	FALSE	LINC01586
Region_6243	chr15	88621337	88632281	10944	3	16	26.36363636	5.368387134	0.647650855	0.054211147	+	64782	grey	0.256225051	FALSE	AEN
Region_6244	chr15	88803436	88875353	71917	19	129	161.6818182	13.03387196	0.847196349	0.02781146	+	176	grey	-0.073244119	FALSE	ACAN
Region_6245	chr15	88877294	88895597	18303	6	32	50	7.251525039	0.891758098	0.039752013	-	145864	grey	0.05926091	FALSE	HAPLN3
Region_6246	chr15	88898683	88913468	14785	6	25	41.03030303	7.662033667	0.528174358	0.066810701	-	4240	grey	0.070447808	FALSE	MFGE8
Region_6247	chr15	89041223	89082819	41596	11	58	84.66666667	10.54270923	0.854994838	0.036076272	+	102724566	grey	0.088165827	FALSE	CARMAL
Region_6248	chr15	89087459	89202355	114896	43	327	373.8030303	18.19655056	0.812345792	0.017070355	+	11057	grey	0.090697117	FALSE	ABHD2
Region_6249	chr15	89243945	89317261	73316	10	64	84.5	8.518306079	0.950179896	0.028225266	+	55215	grey	-0.009834487	FALSE	FANCI

Region_6250	chr15	89316305	89334861	18556	7	34	48.86363636	6.95454774	0.65938839	0.041033494	-	5428	grey	-0.101420449	FALSE	POLG
Region_6251	chr15	89361579	89424983	63404	34	224	284.7727273	20.15157597	0.24253534	0.025715789	+	254559	grey	0.123932095	FALSE	MIR9-3HG
Region_6252	chr15	89471398	89496589	25191	6	19	42.3030303	6.456885882	0.880511433	0.051475208	-	51458	grey	-0.254427599	FALSE	RHCG
Region_6253	chr15	89504909	89524049	19140	4	23	37.56060606	6.58700069	0.819809249	0.081684285	-	283761	grey	-0.048202803	FALSE	LINC00928
Region_6254	chr15	89575469	89631056	55587	15	96	121.6060606	10.28199141	0.846408913	0.016186166	+	90381	grey	0.012204546	FALSE	TICRR
Region_6255	chr15	89627977	89663086	35109	18	103	129.530303	14.52554217	0.933450062	0.027848544	-	374654	grey	-0.0791476	FALSE	KIF7
Region_6256	chr15	89690811	89743638	52827	12	68	92.12121212	9.427480537	0.858812262	0.029703726	+	56964	grey	-0.308897598	FALSE	WDR93
Region_6257	chr15	89749875	89751249	1374	5	24	42.60606061	7.920525989	0.017119965	0.034946474	-	55897	grey	0.144430953	FALSE	MESP1
Region_6258	chr15	89760591	89778754	18163	15	81	115.7878788	12.5961107	0.616972086	0.037888422	+	145873	grey	0.039234074	FALSE	MESP2
Region_6259	chr15	89784895	89815401	30506	8	37	49.31818182	7.262462161	0.936831954	0.029958144	-	290	grey	0.229250106	FALSE	ANPEP
Region_6260	chr15	89830599	89894028	63429	19	130	159.7727273	13.27901231	0.909438724	0.023127577	-	10239	grey	0.056626757	FALSE	AP3S2
Region_6261	chr15	89834308	89912882	78574	35	256	295.8787879	18.46953013	0.824880421	0.018340278	-	100526783	grey	-0.012913804	FALSE	ARPIN-AP3S2
Region_6262	chr1	89895006	89912952	17946	15	88	116	10.1980390	0.83769426	0.02678420	-	348110	grey	-0.07731321	FALSE	ARPIN

	5							3	7	5				5		
Region_6263	chr15	90001324	90082191	80867	36	229	276.0606061	19.09238977	0.807703462	0.029023239	+	374655	grey	-0.115745637	FALSE	ZNF710
Region_6264	chr15	90074515	90082207	7692	6	30	46.3030303	6.939230055	0.902658939	0.05088193	-	109729181	grey	0.010036234	FALSE	ZNF710-AS1
Region_6265	chr15	90083045	90102477	19432	7	35	46.53030303	6.71672928	0.828530376	0.046756254	-	3418	grey	-0.003652937	FALSE	IDH2
Region_6266	chr15	90102649	90138016	35367	11	62	83.27272727	10.31137602	0.823728021	0.036224652	+	105370966	grey	0.060855099	FALSE	IDH2-DT
Region_6267	chr15	90160604	90229679	69075	48	301	360.9242424	25.86135258	0.762770849	0.021194756	+	10509	grey	0.199934915	FALSE	SEMA4B
Region_6268	chr15	90229975	90234047	4072	6	14	35.03030303	8.125874527	0.006483423	0.022254662	-	10519	grey	0.123960118	FALSE	CIB1
Region_6269	chr15	90233808	90245811	12003	9	36	56.27272727	9.164388359	0.429644298	0.039585057	+	390637	grey	-0.044516168	FALSE	GDPGP1
Region_6270	chr15	90234200	90272208	38008	14	79	101.8030303	10.16815496	0.721923479	0.033890002	+	440307	grey	0.108499323	FALSE	TTLL13
Region_6271	chr15	90265659	90314499	48840	22	119	151.4090909	13.31393402	0.87921289	0.02578221	+	51335	grey	0.002556959	FALSE	NGRN
Region_6272	chr15	90352284	90369146	16862	11	81	110.8787879	11.09471829	0.762046548	0.02510662	+	342132	grey	-0.0346932	FALSE	ZNF774
Region_6273	chr15	90388242	90502239	113997	29	188	210.1666667	15.87798024	0.843909006	0.023505168	+	8826	grey	-0.160062919	FALSE	IQGAP1
Region_6274	chr15	90529923	90645345	115422	48	310	354.1515152	19.19955322	0.877861583	0.020082127	+	64784	grey	0.086711229	FALSE	CRTC3

Region_6275	chr15	90620007	90717141	97134	42	266	312.3787879	21.05433641	0.808489593	0.019452388	-	101926895	grey	0.017140562	FALSE	CRTC3-AS1
Region_6276	chr15	90717346	90816166	98820	47	335	390.5151515	24.78538181	0.822344153	0.013618693	+	641	grey	0.075785586	FALSE	BLM
Region_6277	chr15	90839641	90853608	13967	6	28	40.27272727	6.625232546	0.919810787	0.076999841	+	105370969	grey	-0.254134221	FALSE	LOC105370969
Region_6278	chr15	90868588	90883564	14976	12	60	75.42424242	8.880506926	0.721723524	0.053558101	+	5045	grey	-0.038078815	FALSE	FURIN
Region_6279	chr15	90883695	90895776	12081	10	45	70.3030303	9.131466639	0.918789202	0.041092129	+	2242	grey	-0.022599733	FALSE	FES
Region_6280	chr15	90902218	90922584	20366	22	87	159.6969697	16.76899125	0.331321079	0.021797559	+	4122	grey	-0.062444336	FALSE	MAN2A2
Region_6281	chr15	90929968	90935196	5228	14	92	118.5151515	13.42356415	0.302651393	0.040833409	-	374659	grey	0.016607585	FALSE	HDDC3
Region_6282	chr15	90930180	90954093	23913	30	205	239.4848485	19.00626814	0.629014093	0.022936143	+	55898	grey	0.01007456	FALSE	UNC45A
Region_6283	chr15	90954881	90963125	8244	3	17	27.42424242	4.451656929	0.653366532	0.042217487	+	91433	grey	-0.063326575	FALSE	RCCD1
Region_6284	chr15	90966040	91022566	56526	16	103	132.0757576	10.83485914	0.663394922	0.020280553	-	9055	grey	-0.381457888	FALSE	PRC1
Region_6285	chr15	90966340	90988625	22285	4	15	28.25757576	3.939194006	0.972207012	0.033857645	+	100507118	grey	0.140232155	FALSE	PRC1-AS1
Region_6286	chr15	90974600	91022603	48003	14	91	117	10.61493868	0.617718221	0.023411631	-	26276	grey	-0.434000179	FALSE	VPS33B
Region_6287	chr1	91022619	91036611	13992	6	28	41	6.46410205	0.76056185	0.05700870	+	10192691	grey	0.185412427	FALSE	VPS33B-DT

	5							6		8		1				
Region_6288	chr15	91099950	91302565	202615	46	339	401.7121212	20.70136759	0.837863119	0.02062167	+	9899	grey	0.151131221	FALSE	SV2B
Region_6289	chr15	91408708	91653389	244681	76	552	605.8939394	28.44110178	0.910157499	0.017343378	+	101926928	grey	-0.073762127	FALSE	CRAT37
Region_6290	chr15	91643017	91649114	6097	3	14	23.83333333	5.371100255	0.929741352	0.074876171	-	105370973	grey	-0.071452516	FALSE	LOC105370973
Region_6291	chr15	91853708	92172435	318727	54	411	469.9545455	25.54418905	0.843339891	0.019658471	+	28232	grey	-0.220418023	FALSE	SLCO3A1
Region_6292	chr15	92393881	92468728	74847	37	267	314.4242424	20.09443904	0.806507253	0.025625789	+	8128	grey	0.127153054	FALSE	ST8SIA2
Region_6293	chr15	92471654	92501117	29463	4	24	40.24242424	6.688820998	0.869493149	0.053162053	+	145858	grey	0.116667993	FALSE	C15orf32
Region_6294	chr15	92617448	92809798	192350	50	356	407.969697	20.13567037	0.805740926	0.018961514	-	400451	grey	0.26697821	FALSE	FAM174B
Region_6295	chr15	92782055	92809861	27806	12	81	106.1515152	9.741651138	0.802877712	0.020232066	-	101926994	grey	0.175010525	FALSE	LOC101926994
Region_6296	chr15	92819540	92899701	80161	30	168	226.1363636	18.49148358	0.798336679	0.023540296	+	100507217	grey	0.170536978	FALSE	CHASERR
Region_6297	chr15	92883413	93027996	144583	31	228	262.7424242	17.46672494	0.801362097	0.020989007	+	1106	grey	-0.156832929	FALSE	CHD2
Region_6298	chr15	93035271	93089211	53940	11	72	93.68181818	10.44569708	0.749937006	0.030599945	-	56963	grey	-0.088536071	FALSE	RGMA
Region_6299	chr15	93230034	93569483	339449	131	952	1066.121212	43.41942676	0.859204505	0.014169474	+	105370982	grey	-0.008979236	FALSE	LOC105370982

Region_6300	chr15	93589867	93760886	171019	42	303	353.2121212	18.71035513	0.850142492	0.02753469	+	107983974	grey	0.181394445	FALSE	LOC107983974
Region_6301	chr15	93718542	94038412	319870	61	465	528.6515152	25.22483052	0.879253793	0.018701007	-	283682	grey	-0.006016051	FALSE	LINC01579
Region_6302	chr15	93835490	93878822	43332	10	71	94.96969697	9.818302684	0.736467287	0.029390723	+	101927153	grey	-0.10047514	FALSE	LINC02207
Region_6303	chr15	93894431	93984150	89719	14	106	133.7575758	12.55885909	0.877626581	0.032735458	+	101927129	grey	-0.033006848	FALSE	LINC01580
Region_6304	chr15	93905405	94107938	202533	27	215	252.0757576	15.99933711	0.884218349	0.025545125	-	101927112	grey	0.293596248	FALSE	LINC01581
Region_6305	chr15	94231538	94483952	252414	38	289	327.9848485	16.77222285	0.84318916	0.013704659	+	55784	grey	-0.16146002	FALSE	MCTP2
Region_6306	chr15	94841059	95169864	328805	36	227	263.3181818	17.19584842	0.876340257	0.025000207	+	105370991	grey	-0.089910612	FALSE	LOC105370991
Region_6307	chr15	94855474	94856912	1438	5	21	30.84848485	6.787414653	0.921631666	0.055296268	+	440311	grey	-0.125123452	FALSE	LOC440311
Region_6308	chr15	94971837	95018114	46277	6	27	41.66666667	6.514677708	0.91816738	0.044360967	-	105370989	grey	0.078113852	FALSE	LOC105370989
Region_6309	chr15	95166065	95327100	161035	20	151	181.7121212	13.46764231	0.857517508	0.030951499	-	400456	grey	-0.068365789	FALSE	LETR1
Region_6310	chr15	95195011	95327129	132118	18	140	169.3181818	13.01260819	0.852451868	0.032175881	-	105370994	grey	-0.087374956	FALSE	
Region_6311	chr15	95326528	95507865	181337	19	124	150.8030303	11.30040121	0.879434569	0.032233903	+	145820	grey	0.181909148	FALSE	LINC00924
Region_6312	chr1	95463587	95493843	30256	3	11	18	3.70446715	0.94287117	0.06175931	-	10537099	grey	0.0334782	FALSE	LOC105370993

	5							8	2	9		3				
Region_6313	chr15	95990582	96300990	310408	50	362	417.6212121	22.80874674	0.823380689	0.025983152	+	112268156	grey	-0.092261794	FALSE	LOC112268156
Region_6314	chr15	96080713	96135469	54756	12	69	94.46969697	10.87583022	0.801101133	0.057008372	-	105369212	grey	0.247319023	FALSE	LOC105369212
Region_6315	chr15	96127369	96327361	199992	32	226	267.9090909	17.60483087	0.75352385	0.030967337	-	644192	grey	-0.290973531	FALSE	NR2F2-AS1
Region_6316	chr15	96325938	96340263	14325	5	24	32.77272727	4.932764725	0.044447434	0.044126384	+	7026	grey	0.31398701	FALSE	NR2F2
Region_6317	chr15	96354237	96405235	50998	13	92	112.3030303	10.11163792	0.264447565	0.036377728	+	101927263	grey	0.114342086	FALSE	LOC101927263
Region_6318	chr15	97215812	97432094	216282	33	234	283.1363636	16.78267088	0.816865181	0.03268725	+	107984764	grey	0.225470263	FALSE	LINC02253
Region_6319	chr15	97272020	97319321	47301	7	42	59.09090909	6.541529515	0.780848398	0.066823043	-	105371006	grey	-0.208865412	FALSE	LOC105371006
Region_6320	chr15	97339693	97523442	183749	30	219	261.2727273	14.51211213	0.862003786	0.027348058	-	101927286	grey	0.20188326	FALSE	LINC02254
Region_6321	chr15	97629206	97875650	246444	29	215	257.6060606	15.77035075	0.835598414	0.030940419	-	91948	grey	0.014380283	FALSE	LINC00923
Region_6322	chr15	97960703	97973833	13130	6	24	43.28787879	6.382102744	0.321805547	0.026542836	+	91947	grey	-0.057502983	FALSE	ARRDC4
Region_6323	chr15	98003038	98087384	84346	8	52	69.62121212	7.383485005	0.841389481	0.056098095	+	105371008	grey	0.06999503	FALSE	LINC02251
Region_6324	chr15	98111729	98131707	19978	4	18	34.1969697	6.392831796	0.899775787	0.06761209	-	105371010	grey	0.044680772	FALSE	LOC105371010

Region_6325	chr15	98323902	98421021	97119	20	139	169.4545455	13.79652333	0.727164745	0.036766419	-	105371011	grey	0.170300641	FALSE	LINC02351
Region_6326	chr15	98437162	98520386	83224	24	174	216.1212121	16.17644551	0.804224074	0.028434241	-	283777	grey	0.117883256	FALSE	FAM169BP
Region_6327	chr15	98648539	98964530	315991	80	585	632.9090909	21.2108298	0.754519452	0.01387826	+	3480	grey	-0.393128664	FALSE	IGF1R
Region_6328	chr15	98954149	99105824	151675	70	500	566.1363636	28.08606902	0.685082507	0.015463155	-	105371016	grey	-0.119617478	FALSE	SYNM-AS1
Region_6329	chr15	98968229	99007792	39563	12	74	93.56060606	10.94479685	0.855365257	0.042282984	-	145814	grey	0.135705443	FALSE	PGPEP1L
Region_6330	chr15	99014526	99031054	16528	6	35	49.56060606	6.043760388	0.825986054	0.048065126	+	104564224	grey	-0.174256856	FALSE	LUNAR1
Region_6331	chr15	99098217	99135593	37376	26	163	206.3030303	18.75097511	0.475919956	0.021225151	+	23336	grey	-0.03900107	FALSE	SYNM
Region_6332	chr15	99136323	99251223	114900	37	272	315.7121212	16.90586166	0.862984404	0.025697682	-	64927	grey	-0.314981746	FALSE	TTC23
Region_6333	chr15	99251362	99390729	139367	25	168	203.9242424	14.53622595	0.867474996	0.016317364	+	123355	grey	0.017378216	FALSE	LRRC28
Region_6334	chr15	99565417	99716488	151071	52	367	426.8181818	21.633243	0.863134821	0.01848524	+	4205	grey	-0.001601854	FALSE	MEF2A
Region_6335	chr15	99790156	99806927	16771	10	39	77.21212121	15.83813815	0.941070581	0.038450085	-	196968	grey	0.104996545	FALSE	DNM1P46
Region_6336	chr15	99805793	99916818	111025	23	164	198.9242424	17.0359896	0.873046869	0.030713085	+	400464	grey	0.322481232	FALSE	LOC400464
Region_6337	chr1	99971437	10034200	370568	141	1114	1206.80303	42.2130930	0.82613299	0.01560214	-	170691	grey	0.171645816	FALSE	ADAMTS17

	5		5					1	9	8						
Region_6338	chr15	99976481	99980774	4293	4	13	23.93939394	5.618318357	0.944122748	0.077225172	+	105371022	grey	0.259363736	FALSE	LOC105371022
Region_6339	chr15	100344047	100350725	6678	3	18	32.46969697	4.814144854	0.894419337	0.057500652	-	388182	grey	-0.082395471	FALSE	SPATA41
Region_6340	chr15	100372936	100437914	64978	11	67	82.21212121	7.966304329	0.860913206	0.038822056	+	102723320	grey	-0.130527228	FALSE	CERS3-AS1
Region_6341	chr15	100400395	100544995	144600	34	229	266.3030303	19.03193244	0.878462284	0.026787778	-	204219	grey	-0.252195447	FALSE	CERS3
Region_6342	chr15	100559369	100603230	43861	6	44	61.8030303	8.16789154	0.285393651	0.048384982	-	55180	grey	-0.140127179	FALSE	LINS1
Region_6343	chr15	100602589	100651701	49112	16	141	161.7575758	11.65010479	0.831209574	0.03764109	+	140460	grey	0.179397964	FALSE	ASB7
Region_6344	chr15	100716249	100828497	112248	29	206	242.4242424	15.60676442	0.892913822	0.019444861	+	105371024	grey	-0.102468059	FALSE	LOC105371024
Region_6345	chr15	100849561	100885329	35768	29	192	225.2424242	17.38351173	0.454425821	0.023912645	+	105369201	grey	0.055149063	FALSE	GCAWKR
Region_6346	chr15	100877714	100916626	38912	25	175	208.4242424	15.49298661	0.411317738	0.024142587	+	220	grey	0.12605133	FALSE	ALDH1A3
Region_6347	chr15	100892343	100919391	27048	11	80	102.6969697	10.90514303	0.490388293	0.021884846	-	101927751	grey	0.028940055	FALSE	ALDH1A3-AS1
Region_6348	chr15	100919327	101078257	158930	43	301	355.8030303	19.62903634	0.836933316	0.014905852	+	79705	grey	-0.013864811	FALSE	LRRK1
Region_6349	chr15	101043716	101086066	42350	19	120	152.8787879	12.5630902	0.942194132	0.027874813	-	105371026	grey	-0.068476336	FALSE	LOC105371026

Region_6350	chr15	101175727	101252048	76321	35	227	285.6363636	21.14974913	0.613563672	0.020830187	-	22856	grey	-0.077581521	FALSE	CHSY1
Region_6351	chr15	101270817	101277500	6683	3	16	27.40909091	4.927090803	0.002829578	0.011879033	-	55829	grey	0.059748354	FALSE	SELENOS
Region_6352	chr15	101281510	101295282	13772	3	15	24.53030303	5.315347002	0.730864679	0.078329249	-	6627	grey	0.014417835	FALSE	SNRPA1
Region_6353	chr15	101289784	101525202	235418	133	971	1076.969697	43.9828532	0.82460718	0.012866726	-	5046	grey	-0.09866005	FALSE	PCSK6
Region_6354	chr15	101602786	101614531	11745	7	31	48.65151515	7.469924156	0.910388789	0.053263961	-	101927505	grey	0.052232673	FALSE	LINC02348
Region_6355	chr15	101632977	101652391	19414	10	69	91.56060606	11.05111033	0.636806644	0.024209037	-	80213	grey	-0.37495237	FALSE	TM2D3
Region_6356	chr15	101653596	101724473	70877	27	168	202.1818182	12.83733628	0.914649197	0.020509898	-	123283	grey	-0.032260753	FALSE	TARS3
Region_6357	chr15	101737099	101746870	9771	4	21	35.60606061	6.55832715	0.732215939	0.025958116	-	100128108	grey	0.049393004	FALSE	UBE2Q2P13
Region_6358	chr15	101772807	101794302	21495	6	46	60.12121212	7.606707873	0.91363159	0.035692171	+	105371030	grey	0.061885037	FALSE	
Region_6359	chr15	101812202	101820197	7995	5	28	41.53030303	6.445558273	0.921153492	0.045461936	+	390649	grey	-0.210057704	FALSE	OR4F15
Region_6360	chr16	58059	76355	18296	19	110	135.4848485	15.32844758	0.749496253	0.027916463	-	64285	grey	-0.080116226	FALSE	RHBDF1
Region_6361	chr16	77007	85851	8844	4	19	28.68181818	4.961882679	0.540868694	0.063673669	+	4350	grey	0.206691049	FALSE	MPG
Region_6362	chr1	84271	138677	54406	63	413	491.545454	25.1317646	0.88150289	0.01325009	-	8131	grey	-0.14227571	FALSE	NPRL3

	6						5	5	3					3		
Region_6363	chr16	142728	154503	11775	5	27	41.13636364	5.250308016	0.798766911	0.064352989	+	3050	grey	0.104687133	FALSE	HBZ
Region_6364	chr16	153892	166764	12872	7	28	42.18181818	5.961179777	0.538952534	0.050783292	+	3042	grey	-0.054590952	FALSE	HBM
Region_6365	chr16	188969	229463	40494	44	295	340.5	22.85691277	0.893312965	0.013249022	-	55692	grey	0.100715635	FALSE	LUC7L
Region_6366	chr16	234521	282084	47563	60	445	502.3939394	28.11672308	0.777056282	0.016296992	+	83986	grey	-0.056207475	FALSE	FAM234A
Region_6367	chr16	268301	275980	7679	10	52	76.96969697	9.119665475	0.663629328	0.027165024	-	8786	grey	-0.240946836	FALSE	RGS11
Region_6368	chr16	268727	283010	14283	14	75	104.0151515	11.18308121	0.637441212	0.035315135	+	398	grey	-0.094387164	FALSE	ARHGDIG
Region_6369	chr16	283164	287215	4051	11	32	61.21212121	10.12463128	0.921414927	0.042334266	+	64714	grey	0.164978319	FALSE	PDIA2
Region_6370	chr16	287440	352723	65283	129	911	1010.318182	46.00139098	0.783875942	0.012098266	-	8312	grey	0.204136157	FALSE	AXIN1
Region_6371	chr16	366969	371289	4320	6	25	43.5	7.19241053	0.797493827	0.05141305	-	10573	grey	0.186499762	FALSE	MRPL28
Region_6372	chr16	370788	387113	16325	29	147	180.5	13.29574831	0.682008901	0.023988261	-	58986	grey	0.19195501	FALSE	PGAP6
Region_6373	chr16	382097	392960	10863	21	128	158.4393939	12.73170336	0.674517805	0.021702236	+	100134368	grey	-0.019666256	FALSE	LOC100134368
Region_6374	chr16	396725	410367	13642	48	233	309.530303	26.5950948	0.529239098	0.023884377	+	4833	grey	-0.185437014	FALSE	NME4

Region_6375	chr16	560394	565529	5135	12	44	64.36363636	9.537999086	0.726892212	0.048805852	+	146325	grey	0.113429494	FALSE	PRR35
Region_6376	chr16	566995	584109	17114	50	253	304.4848485	20.51734158	0.718249798	0.018875549	+	9091	grey	-0.222468932	FALSE	PIGQ
Region_6377	chr16	567005	569495	2490	4	15	29.60606061	5.670107279	0.695798814	0.046889343	+	283948	grey	-0.100777556	FALSE	NHLRC4
Region_6378	chr16	589357	629272	39915	132	851	971.8030303	56.4981197	0.800161852	0.011956123	+	57799	grey	0.064658668	FALSE	RAB40C
Region_6379	chr16	630985	634117	3132	10	50	73.13636364	9.514177864	0.960058925	0.026516731	+	117166	grey	0.014567984	FALSE	WFIKKN1
Region_6380	chr16	634427	636366	1939	4	14	23.68181818	5.014315172	0.375761143	0.099161637	-	84326	grey	0.127015932	FALSE	METTL26
Region_6381	chr16	636817	638620	1803	3	11	20.03030303	4.502886927	0.614689295	0.05551469	+	100287175	grey	0.272957588	FALSE	LOC100287175
Region_6382	chr16	636903	648474	11571	37	186	235.3333333	18.46687957	0.638617466	0.030823621	+	84331	grey	0.101895445	FALSE	MCRIP2
Region_6383	chr16	649311	667833	18522	53	305	363.3333333	28.68359667	0.69215994	0.018130812	+	197335	grey	-0.401217792	FALSE	WDR90
Region_6384	chr16	668100	674174	6074	15	77	99.90909091	12.07760454	0.688224888	0.031861504	+	89941	grey	0.198120015	FALSE	RHOT2
Region_6385	chr16	675671	678268	2597	14	62	83.48484848	10.73060673	0.460679596	0.053831513	+	9028	grey	-0.186855785	FALSE	RHBDL1
Region_6386	chr16	678504	679777	1273	4	17	28.57575758	5.335110427	0.466799704	0.037764326	-	105371184	grey	0.055119652	FALSE	STUB1-DT
Region_6387	chr1	680224	682801	2577	12	67	83.2121212	10.9706113	0.36393188	0.03601695	+	10273	grey	0.106885424	FALSE	STUB1

	6						1		6	7						
Region_6388	chr16	681670	684528	2858	28	153	205.530303	26.05037705	0.297524106	0.02593034	-	339123	grey	0.05368841	FALSE	JMJD8
Region_6389	chr16	684622	690444	5822	31	182	239.3787879	22.67005151	0.784283876	0.018072298	-	84219	grey	0.172428325	FALSE	WDR24
Region_6390	chr16	692498	705808	13310	37	212	254	21.90609329	0.485011218	0.028684346	-	146330	grey	0.021059953	FALSE	FBXL16
Region_6391	chr16	715118	719655	4537	21	88	124.9393939	15.995075	0.515288809	0.036665334	+	79006	grey	0.206694281	FALSE	METRN
Region_6392	chr16	720581	722590	2009	13	64	91.25757576	14.69412296	0.227960383	0.029363141	+	65990	grey	0.197759613	FALSE	ANTKMT
Region_6393	chr16	722582	726954	4372	13	75	109.1212121	11.16107956	0.67064478	0.03864952	-	124093	grey	0.166257351	FALSE	CCDC78
Region_6394	chr16	726936	735353	8417	56	338	399.6515152	31.82185626	0.58019786	0.016482922	+	84264	grey	-0.1715357	FALSE	HAGHL
Region_6395	chr16	729760	741329	11569	58	372	472.3030303	33.8185064	0.816257794	0.022065172	-	64428	grey	-0.055973209	FALSE	CIAO3
Region_6396	chr16	760734	768865	8131	27	117	165.8484848	20.68462262	0.909510548	0.023684918	+	10232	grey	0.222269574	FALSE	MSLN
Region_6397	chr16	784974	788397	3423	9	42	68.53030303	9.505010334	0.658287646	0.030809171	-	113000	grey	0.09266232	FALSE	RPUSD1
Region_6398	chr16	788046	798074	10028	43	219	285.6969697	26.66196928	0.628692835	0.013500082	+	63922	grey	-0.284689836	FALSE	CHTF18
Region_6399	chr16	798041	800734	2693	7	28	43.66666667	7.746628713	0.344648937	0.06497331	-	51764	grey	0.158890635	FALSE	GNG13

Region_6400	chr16	853634	981318	127684	298	1853	2064.212121	112.0062519	0.901665901	0.010150886	-	64788	grey	-0.372281987	FALSE	LMF1
Region_6401	chr16	921033	934495	13462	27	163	204.7878788	18.01240184	0.958463721	0.016626822	+	101929387	grey	-0.003432184	FALSE	LMF1-AS1
Region_6402	chr16	975761	981596	5835	14	68	95.89393939	11.62110651	0.363094277	0.049870678	-	115804232	grey	-0.413265339	FALSE	CEROX1
Region_6403	chr16	981770	986979	5209	20	106	134.3181818	13.55916845	0.683213558	0.027034831	+	30812	grey	0.207234737	FALSE	SOX8
Region_6404	chr16	991151	1000926	9775	11	59	78.87878788	8.792505815	0.850191136	0.036591788	-	107984898	grey	-0.112307405	FALSE	
Region_6405	chr16	1064087	1078707	14620	23	114	136.7878788	14.31461346	0.558162218	0.043555997	-	146336	grey	-0.196003696	FALSE	SSTR5-AS1
Region_6406	chr16	1072747	1081454	8707	23	114	143.8787879	15.29556512	0.786978469	0.039312009	+	6755	grey	0.130694589	FALSE	SSTR5
Region_6407	chr16	1088226	1096244	8018	32	161	201.1363636	21.1259129	0.629981666	0.04252012	-	390664	grey	-0.18578843	FALSE	C1QTNF8
Region_6408	chr16	1153106	1221771	68665	228	1311	1488.787879	90.02828874	0.805096376	0.011701833	+	8912	grey	-0.221980823	FALSE	CACNA1H
Region_6409	chr16	1221651	1225268	3617	17	67	100.9545455	12.93942315	0.826360067	0.044805392	-	25823	grey	0.333650038	FALSE	TPSG1
Region_6410	chr16	1256059	1259008	2949	9	43	55.74242424	7.168972904	0.820149344	0.058859829	+	23430	grey	0.223636431	FALSE	TPSD1
Region_6411	chr16	1308880	1327018	18138	31	174	218.4242424	16.89065341	0.591482566	0.029279468	+	7329	grey	-0.1868069	FALSE	UBE2I
Region_6412	chr1	1333638	1349441	15803	22	129	164.363636	17.4176182	0.81629538	0.03392974	+	8938	grey	0.005652643	FALSE	BAIAP3

	6						4	8	3	4						
Region_6413	chr16	1349240	1351878	2638	11	60	89.72727273	11.36600255	0.312636831	0.02079772	-	115939	grey	0.276688433	FALSE	TSR3
Region_6414	chr16	1351931	1365737	13806	25	172	205.8787879	19.66137576	0.897491335	0.019979435	+	84572	grey	-0.137893615	FALSE	GNPTG
Region_6415	chr16	1363205	1414751	51546	129	843	933.1515152	49.10498719	0.885047513	0.008544347	-	64718	grey	0.030550051	FALSE	UNKL
Region_6416	chr16	1408834	1412248	3414	6	36	51.40909091	7.433763459	0.952942809	0.041711823	+	101929440	grey	0.026522155	FALSE	LOC101929440
Region_6417	chr16	1419752	1420747	995	6	47	61.83333333	8.157268271	0.042660509	0.026646734	-	283951	grey	-0.346852815	FALSE	UQCC4
Region_6418	chr16	1434383	1444556	10173	21	84	123.8636364	18.32349174	0.935927289	0.028948658	-	645811	grey	0.1975102	FALSE	CCDC154
Region_6419	chr16	1444934	1475077	30143	81	460	560.6666667	40.65060633	0.795399932	0.016652208	-	1186	grey	-0.112689241	FALSE	CLCN7
Region_6420	chr16	1485886	1488981	3095	14	72	95.83333333	11.22165946	0.662997934	0.045824911	-	390667	grey	0.209276778	FALSE	PTX4
Region_6421	chr16	1493344	1510457	17113	76	429	507.6515152	34.03504638	0.850154505	0.010288905	+	9894	grey	-0.079611941	FALSE	TELO2
Region_6422	chr16	1510427	1612072	101645	236	1674	1850.454545	83.33937694	0.839260711	0.007190774	-	9742	grey	0.265336284	FALSE	IFT140
Region_6423	chr16	1528688	1555580	26892	76	550	616.2424242	36.59132331	0.776845587	0.015692667	+	79652	grey	0.40809859	FALSE	TMEM204
Region_6424	chr16	1580527	1610328	29801	47	335	386.0151515	24.76131749	0.890049393	0.01337001	+	105371046	grey	-0.043110809	FALSE	LOC105371046

Region_6425	chr16	1612337	1686715	74378	100	728	799.4393939	37.51558806	0.783036618	0.008580807	+	57585	grey	0.105162321	FALSE	CRAMP1
Region_6426	chr16	1678256	1702280	24024	12	83	98.66666667	8.849742862	0.803374921	0.020266709	+	90861	grey	-0.029016685	FALSE	JPT2
Region_6427	chr16	1706166	1770351	64185	108	716	841.0909091	53.44378132	0.880719027	0.010327441	+	23162	grey	0.019384349	FALSE	MAPK8IP3
Region_6428	chr16	1771890	1773150	1260	4	19	33.24242424	6.206846359	0.04890092	0.043822657	-	65993	grey	0.069784395	FALSE	MRPS34
Region_6429	chr16	1772810	1781708	8898	16	85	116.0757576	14.65638281	0.66214991	0.027877723	+	197342	grey	0.061993154	FALSE	EME2
Region_6430	chr16	1776712	1793700	16988	75	450	519.3333333	36.27643157	0.755204758	0.012863405	-	90864	grey	0.114026404	FALSE	SPSB3
Region_6431	chr16	1782932	1789186	6254	37	224	276.8333333	22.13704402	0.831317325	0.01731705	+	10101	grey	0.178301961	FALSE	NUBP2
Region_6432	chr16	1790413	1794971	4558	13	66	91.84848485	11.2759	0.952417875	0.020946322	-	3483	grey	0.084223797	FALSE	IGFALS
Region_6433	chr16	1795620	1827157	31537	81	503	589.2727273	35.8022541	0.825376617	0.011497702	-	3029	grey	0.365731381	FALSE	HAGH
Region_6434	chr16	1826967	1840207	13240	19	119	165.2878788	18.83512688	0.553778233	0.03593306	+	81889	grey	0.046889344	FALSE	FAHD1
Region_6435	chr16	1833986	1884294	50308	48	288	348.1515152	21.78019031	0.827318745	0.02330364	-	254528	grey	-0.182989303	FALSE	MEIOB
Region_6436	chr16	1938229	1943199	4970	9	50	71.03030303	7.957521255	0.43220498	0.064525888	-	51734	grey	-0.083868975	FALSE	MSRB1
Region_6437	chr1	1943974	1957606	13632	12	67	97.5303030	10.8134100	0.84538952	0.0408303	-	6123	grey	0.207642352	FALSE	RPL3L

	6						3	5								
Region_6438	chr16	1959538	1961975	2437	8	45	62.06060606	7.715875319	0.325404428	0.060195678	+	4716	grey	-0.101583884	FALSE	NDUFB10
Region_6439	chr16	1962058	1964841	2783	8	49	63.06060606	7.928291673	0.340671478	0.02819184	-	6187	grey	0.036784813	FALSE	RPS2
Region_6440	chr16	1964895	1965509	614	3	12	20.3030303	4.413161075	0.008257357	0.024228134	+	735301	grey	0.069082038	FALSE	SNHG9
Region_6441	chr16	1966823	1968975	2152	17	105	132.1363636	16.71745874	0.958366733	0.02063181	+	146310	grey	-0.052448966	FALSE	RNF151
Region_6442	chr16	1972053	1982929	10876	25	140	169.8030303	14.97713097	0.408069665	0.036898578	+	10607	grey	-0.169804944	FALSE	TBL3
Region_6443	chr16	1978917	1984192	5275	19	109	144.0606061	15.2506028	0.148560082	0.037781896	-	124056	grey	-0.096737893	FALSE	NOXO1
Region_6444	chr16	1984193	1987749	3556	9	41	61.25757576	9.41898346	0.396104471	0.029812337	+	2671	grey	0.121474321	FALSE	GFER
Region_6445	chr16	1989660	1994275	4615	16	102	123.5454545	11.34111895	0.302546371	0.030356029	+	9143	grey	-0.008535994	FALSE	SYNGR3
Region_6446	chr16	1997654	2009821	12167	31	174	218.8333333	20.67892523	0.529971083	0.028102481	-	90850	grey	-0.222135817	FALSE	ZNF598
Region_6447	chr16	2009926	2020755	10829	29	159	193.6818182	16.35204912	0.68025032	0.037993416	+	283869	grey	-0.03503872	FALSE	NPW
Region_6448	chr16	2025356	2039026	13670	36	186	229.1515152	16.78024702	0.590884137	0.025566648	+	9351	grey	-0.165596495	FALSE	NHERF2
Region_6449	chr16	2039815	2047866	8051	16	83	117.1363636	13.44834718	0.833953855	0.030149888	-	4913	grey	-0.097198665	FALSE	NTHL1

Region_6450	chr16	2047967	2089491	41524	118	751	848.6666667	47.88201311	0.902366339	0.00904827	+	7249	grey	0.172806096	FALSE	TSC2
Region_6451	chr16	2091436	2095433	3997	10	40	62	10.56554633	0.938746729	0.037782632	+	105371049	grey	0.281415106	FALSE	PKD1-AS1
Region_6452	chr16	2140803	2154165	13362	35	183	226.8939394	16.33413489	0.595959188	0.02289284	+	25837	grey	-0.015481597	FALSE	RAB26
Region_6453	chr16	2155698	2178129	22431	68	344	442.9242424	31.9081907	0.832506595	0.013028764	+	84231	grey	0.061574366	FALSE	TRAF7
Region_6454	chr16	2177180	2196605	19425	39	193	244.8939394	21.37729118	0.845306477	0.02145473	-	57524	grey	-0.262926948	FALSE	CASKIN1
Region_6455	chr16	2204596	2209453	4857	23	106	144.2272727	17.87201978	0.237262922	0.03051149	+	64223	grey	-0.006500514	FALSE	MLST8
Region_6456	chr16	2209253	2210905	1652	5	12	24.72727273	5.070413288	0.934709541	0.070612501	-	283870	grey	-0.037892005	FALSE	BRICD5
Region_6457	chr16	2211593	2214840	3247	29	119	221.9848485	33.91958473	0.091418033	0.010700737	-	283871	grey	0.020119905	FALSE	PGP
Region_6458	chr16	2223580	2235742	12162	54	347	401.4090909	24.99598569	0.87288709	0.014210906	+	1877	grey	-0.092537583	FALSE	E4F1
Region_6459	chr16	2235816	2238711	2895	11	57	76.45454545	8.485611021	0.167088398	0.052483348	+	1775	grey	-0.196482379	FALSE	DNASE1L2
Region_6460	chr16	2239402	2252300	12898	33	197	256.7878788	21.43793557	0.801478972	0.015456579	-	1632	grey	0.41261324	FALSE	ECI1
Region_6461	chr16	2253116	2268397	15281	25	134	169.530303	16.52524381	0.745897909	0.022442339	-	10921	grey	-0.033571275	FALSE	RNPS1
Region_6462	chr1	2268155	2273418	5263	8	47	61.2727272	7.63100044	0.37414561	0.02425539	+	10666060	grey	-0.02744868	FALSE	MIR3677HG

	6						7	8	4			6				
Region_6463	chr16	2275881	2340746	64865	116	782	886.7575758	47.89766675	0.897449045	0.01301929	-	21	grey	0.153359958	FALSE	ABCA3
Region_6464	chr16	2339150	2426699	87549	44	313	357.9545455	21.94989526	0.783522767	0.015321048	+	650655	grey	0.060802377	FALSE	ABCA17P
Region_6465	chr16	2429394	2458854	29460	13	72	98.16666667	11.67849218	0.823356318	0.017819995	+	899	grey	-0.120010971	FALSE	CCNF
Region_6466	chr16	2460086	2464963	4877	3	14	24.37878788	5.092272129	0.961091761	0.047295947	+	80178	grey	0.032002679	FALSE	TEDC2
Region_6467	chr16	2464950	2468213	3263	5	28	41.66666667	7.890273142	0.206337844	0.024860383	-	729652	grey	0.192383519	FALSE	TEDC2-AS1
Region_6468	chr16	2471297	2474145	2848	4	21	35.03030303	7.61975105	0.2556797	0.037817185	+	4917	grey	-0.126609814	FALSE	NTN3
Region_6469	chr16	2475051	2520218	45167	70	434	508.6212121	32.66414885	0.628216303	0.019529398	+	57465	grey	-0.172938715	FALSE	TBC1D24
Region_6470	chr16	2513952	2520218	6266	15	67	93.24242424	10.37313187	0.40309345	0.029630293	+	527	grey	0.012222889	FALSE	ATP6V0C
Region_6471	chr16	2513965	2531422	17457	47	270	332.1666667	21.60881824	0.66374979	0.017503524	+	51005	grey	0.000869895	FALSE	AMDHD2
Region_6472	chr16	2530035	2531408	1373	4	29	39.59090909	5.337753122	0.947486973	0.034056775	-	752014	grey	-0.074013941	FALSE	CEMP1
Region_6473	chr16	2537979	2603188	65209	51	331	410.0606061	36.29616584	0.926300807	0.013361451	+	5170	grey	-0.001965228	FALSE	PDPK1
Region_6474	chr16	2603336	2630494	27158	13	65	109.969697	14.29359157	0.795512136	0.031498449	+	652276	grey	-0.211757648	FALSE	LOC652276

Region_6475	chr16	2660348	2682379	22031	34	200	267.2272727	27.52665587	0.702536261	0.027049691	-	100507321	grey	0.127535622	FALSE	ERVK13-1
Region_6476	chr16	2682523	2709030	26507	36	263	292.7727273	15.53734504	0.731714784	0.018193084	+	54442	grey	-0.166926464	FALSE	KCTD5
Region_6477	chr16	2712419	2720551	8132	15	59	89.42424242	13.08441305	0.822285109	0.035199651	-	83886	grey	-0.059069245	FALSE	PRSS27
Region_6478	chr16	2737076	2752600	15524	23	157	191.0909091	16.14618533	0.602188328	0.026434406	-	100128788	grey	-0.17910054	FALSE	SRRM2-AS1
Region_6479	chr16	2752626	2771412	18786	30	181	215.9090909	15.57683415	0.834343423	0.015663143	+	23524	grey	0.250671533	FALSE	SRRM2
Region_6480	chr16	2771414	2777289	5875	4	14	25.95454545	5.324635974	0.472291965	0.052744225	-	6923	grey	-0.075056189	FALSE	ELOB
Region_6481	chr16	2783953	2787948	3995	5	14	25.43939394	4.877353595	0.255368144	0.081447367	-	260429	grey	0.15773604	FALSE	PRSS33
Region_6482	chr16	2798470	2805304	6834	18	72	106.3181818	16.37367408	0.940066217	0.028810854	+	360226	grey	-0.039071629	FALSE	PRSS41
Region_6483	chr16	2817180	2826304	9124	3	11	18.03030303	3.679338139	0.849347734	0.118388806	+	10942	grey	0.021301725	FALSE	PRSS21
Region_6484	chr16	2883213	2899382	16169	8	27	45.3030303	7.575201844	0.497979476	0.025106065	+	114984	grey	0.016216093	FALSE	FLYWCH2
Region_6485	chr16	2911931	2951208	39277	55	309	368.6212121	25.25059948	0.880754091	0.020996383	+	84256	grey	0.047893919	FALSE	FLYWCH1
Region_6486	chr16	2939714	2954276	14562	16	94	127.6515152	12.38049635	0.930444755	0.032274579	-	105371055	grey	0.071475751	FALSE	LOC105371055
Region_6487	chr1	2964216	2968383	4167	9	35	48.7878787	7.39545630	0.22476670	0.06162195	+	79412	grey	0.208892701	FALSE	KREMEN2

	6						9	1	5	7						
Region_6488	chr16	2968102	2980479	12377	32	175	230.1363636	20.18673316	0.506380845	0.025731951	-	9088	grey	-0.168596107	FALSE	PKMYT1
Region_6489	chr16	2969270	2973484	4214	13	67	87.12121212	10.59973171	0.363571181	0.02539463	+	124222	grey	-0.088492986	FALSE	PAQR4
Region_6490	chr16	2988256	3002016	13760	6	18	30.13636364	5.648379927	0.931756878	0.06361619	+	283875	grey	-0.081408292	FALSE	GREP1
Region_6491	chr16	3012923	3014505	1582	7	31	50.46969697	8.139589287	0.95845221	0.026392241	+	9080	grey	-0.031733501	FALSE	CLDN9
Region_6492	chr16	3014712	3020071	5359	34	211	261.3787879	24.1869419	0.686585472	0.028481151	-	9074	grey	-0.064800721	FALSE	CLDN6
Region_6493	chr16	3018445	3022383	3938	23	136	167.7878788	16.51341194	0.534255468	0.036976577	+	51330	grey	-0.159114522	FALSE	TNFRSF12A
Region_6494	chr16	3022625	3024286	1661	6	25	42.75757576	7.744100695	0.144034714	0.0612649	-	54985	grey	-0.037771658	FALSE	HCFC1R1
Region_6495	chr16	3024027	3027755	3728	7	43	61.15151515	8.877356544	0.282777474	0.012689764	+	79228	grey	-0.151951727	FALSE	THOC6
Region_6496	chr16	3027682	3036944	9262	17	91	116.9090909	14.28364021	0.38371423	0.033450604	-	146439	grey	0.122866247	FALSE	BICDL2
Region_6497	chr16	3032481	3039133	6652	6	22	36.36363636	6.925174507	0.216122336	0.04951374	+	100128770	grey	0.100459955	FALSE	LOC100128770
Region_6498	chr16	3037400	3059370	21970	23	113	149.530303	11.04148777	0.604259585	0.042919362	-	100507419	grey	0.132609355	FALSE	MMP25-AS1
Region_6499	chr16	3046062	3060726	14664	21	105	132.9848485	12.50291341	0.574040875	0.045196412	+	64386	grey	0.127350768	FALSE	MMP25

Region_6500	chr16	3065297	3082192	16895	24	143	174.3636364	13.20330537	0.770902669	0.029685087	+	9235	grey	-0.073232787	FALSE	IL32
Region_6501	chr16	3076911	3087112	10201	11	70	85.81818182	10.05968204	0.792062985	0.02801078	-	105371058	grey	-0.362034878	FALSE	
Region_6502	chr16	3088890	3099295	10405	24	142	185.3787879	17.54007732	0.845486167	0.025423571	-	84891	grey	0.163336387	FALSE	ZSCAN10
Region_6503	chr16	3100696	3143734	43038	57	387	453.4545455	29.5474491	0.822539872	0.016024797	-	81854	grey	-0.113748848	FALSE	ZNF205-AS1
Region_6504	chr16	3112560	3120517	7957	12	76	101.8939394	10.70604256	0.882638917	0.0454825	+	7755	grey	-0.03051562	FALSE	ZNF205
Region_6505	chr16	3116456	3134892	18436	15	106	135.7424242	12.11522958	0.756680749	0.028213548	-	100507458	grey	0.003488759	FALSE	ZNF213-AS1
Region_6506	chr16	3129777	3142804	13027	23	150	187.0606061	15.20615137	0.866799673	0.02100992	+	7760	grey	0.01094681	FALSE	ZNF213
Region_6507	chr16	3144015	3149963	5948	10	48	66.12121212	8.495274979	0.780544203	0.042291695	+	197350	grey	0.029594084	FALSE	CASP16P
Region_6508	chr16	3188204	3224779	36575	47	320	374.8333333	31.17572198	0.642999996	0.021893283	+	4992	grey	-0.151462778	FALSE	OR1F1
Region_6509	chr16	3222325	3236221	13896	7	47	65.10606061	7.043008072	0.697097465	0.025318948	-	7752	grey	0.066021221	FALSE	ZNF200
Region_6510	chr16	3242027	3256633	14606	15	91	118.969697	10.37151379	0.726095836	0.052930752	-	4210	grey	-0.253857733	FALSE	MEFV
Region_6511	chr16	3263743	3267567	3824	3	17	28	5.721753362	0.288769285	0.053923507	+	283876	grey	-0.065673763	FALSE	LINC00921
Region_6512	chr1	3263800	3301401	37601	20	113	154	14.0153761	0.76379725	0.01966726	+	10127	grey	-0.01483999	FALSE	ZNF263

	6							7		3				6		
Region_6513	chr16	3298808	3305729	6921	13	80	101.9545455	9.867478547	0.637179301	0.028858592	-	91151	grey	0.072785198	FALSE	TIGD7
Region_6514	chr16	3305406	3318852	13446	9	45	64.5454545 5	9.636759459	0.97313252	0.028479016	+	7627	grey	0.077813323	FALSE	ZNF75A
Region_6515	chr16	3365099	3384706	19607	8	47	66.60606061	9.289992611	0.780667456	0.045910633	+	105371059	grey	0.142656214	FALSE	LOC105371059
Region_6516	chr16	3365112	3486953	121841	50	341	398.4393939	23.07988054	0.839339328	0.01923702	+	79903	grey	0.096236235	FALSE	NAA60
Region_6517	chr16	3382081	3401065	18984	5	27	40.21212121	6.070141683	0.663873861	0.076635694	-	54925	grey	0.145881736	FALSE	ZSCAN32
Region_6518	chr16	3432414	3443504	11090	8	32	53.40909091	9.050248229	0.763090677	0.073114113	-	146434	grey	-0.19498936	FALSE	ZNF597
Region_6519	chr16	3458071	3539048	80977	61	374	453.5757576	29.14476367	0.869236272	0.013539587	+	23059	grey	0.096578806	FALSE	CLUAP1
Region_6520	chr16	3539033	3577403	38370	30	182	232.2121212	15.53706749	0.880349382	0.023021376	-	197358	grey	-0.098054502	FALSE	NLRC3
Region_6521	chr16	3581181	3611606	30425	18	109	139.7878788	11.64277941	0.780429164	0.025514477	-	84464	grey	-0.022708446	FALSE	SLX4
Region_6522	chr16	3611728	3680143	68415	69	465	550.6060606	29.24584325	0.884405441	0.014096493	+	1773	grey	0.126870316	FALSE	DNASE1
Region_6523	chr16	3651639	3717553	65914	62	447	523.2878788	28.33033989	0.858303816	0.019010382	-	10131	grey	0.563313426	FALSE	TRAP1
Region_6524	chr16	3725054	3880713	155659	100	798	865.6515152	36.49045921	0.903766018	0.011003196	-	1387	grey	0.156013958	FALSE	CREBBP

Region_6525	chr16	3931536	3950586	19050	9	58	80.63636364	8.648052451	0.946115217	0.033560789	-	102724927	grey	-0.132436407	FALSE	LINC02861
Region_6526	chr16	3953387	4116442	163055	141	1061	1148.5	47.24315172	0.845238896	0.012311757	-	115	grey	0.169975085	FALSE	ADCY9
Region_6527	chr16	4180117	4183515	3398	5	26	41.15151515	7.304882296	0.770529786	0.029487506	-	105371062	grey	-0.04894073	FALSE	LOC105371062
Region_6528	chr16	4189374	4242080	52706	31	191	236.1969697	18.78970233	0.908690178	0.020090031	-	6345	grey	0.15460596	FALSE	SRL
Region_6529	chr16	4243943	4253817	9874	6	43	56.04545455	8.016198635	0.673819903	0.051044015	-	100507501	grey	0.111478406	FALSE	LINC01569
Region_6530	chr16	4257186	4273075	15889	16	84	104.8787879	11.7872358	0.718483742	0.025116823	-	7023	grey	-0.273926839	FALSE	TFAP4
Region_6531	chr16	4556340	4600758	44418	35	214	251.0454545	17.98839953	0.884063386	0.023111692	+	342346	grey	0.061240057	FALSE	C16orf96
Region_6532	chr16	4608883	4615027	6144	10	44	63.89393939	9.831079114	0.37930067	0.028432138	-	124402	grey	-0.252192043	FALSE	UBALD1
Region_6533	chr16	4616493	4690974	74481	113	715	876.8636364	51.90876208	0.735116578	0.022869236	+	23295	grey	0.239854587	FALSE	MGRN1
Region_6534	chr16	4693562	4695859	2297	4	14	31.54545455	11.2416558	0.004055435	0.012185034	+	84309	grey	-0.082088873	FALSE	NUDT16L1
Region_6535	chr16	4696510	4734378	37868	51	338	386.3787879	22.26675007	0.798317384	0.01578968	-	124401	grey	0.29312152	FALSE	ANKS3
Region_6536	chr16	4730115	4767216	37101	56	346	410.0757576	28.59333468	0.701767958	0.016886349	-	26048	grey	0.007304033	FALSE	ZNF500
Region_6537	chr1	4734344	4749396	15052	28	162	197.5	18.9194649	0.71475774	0.02769522	+	146562	grey	-0.09796194	FALSE	DNAAF8

	6							5	1	5				6		
Region_6538	chr16	4777606	4801423	23817	34	226	267.4545455	19.33604193	0.878163628	0.017541115	-	124404	grey	-0.036941512	FALSE	SEPTIN12
Region_6539	chr16	4788397	4796491	8094	8	49	66.21212121	7.968235306	0.891838761	0.033297992	+	440335	grey	0.240513445	FALSE	SMIM22
Region_6540	chr16	4796968	4802880	5912	14	62	93.12121212	12.08370187	0.320865462	0.028390371	-	79641	grey	0.091161568	FALSE	ROGDI
Region_6541	chr16	4803203	4847288	44085	35	253	302.0757576	22.14866595	0.774080911	0.015755078	-	84656	grey	-0.060340298	FALSE	GLYR1
Region_6542	chr16	4846665	4882401	35736	37	236	284.7878788	22.59783186	0.44042375	0.012050808	+	29855	grey	0.384731172	FALSE	UBN1
Region_6543	chr16	4882507	4960741	78234	59	401	454.9090909	25.41818082	0.847464735	0.015189214	-	5493	grey	-0.145020836	FALSE	PPL
Region_6544	chr16	4958330	5042250	83920	65	424	493.6363636	30.86179434	0.813066194	0.020671563	+	9717	grey	-0.114150824	FALSE	SEC14L5
Region_6545	chr16	5024844	5034141	9297	13	80	104.8333333	11.57262464	0.6683722	0.031613525	-	51172	grey	-0.034264742	FALSE	NAGPA
Region_6546	chr16	5033702	5043999	10297	8	48	67.89393939	7.965558144	0.798186561	0.037395779	+	100507589	grey	0.074874811	FALSE	NAGPA-AS1
Region_6547	chr16	5033923	5087379	53456	38	265	316.5	18.87387286	0.870772263	0.025403029	+	56052	grey	-0.202888712	FALSE	ALG1
Region_6548	chr16	5044122	5066110	21988	15	101	126.7424242	11.47212086	0.859306359	0.046402758	-	146556	grey	-0.131803401	FALSE	C16orf89
Region_6549	chr16	5084284	5097795	13511	9	52	74.81818182	8.678725697	0.953980984	0.027077525	-	196483	grey	0.136936973	FALSE	EEF2KMT

Region_6550	chr16	5107672	5235822	128150	52	334	388.2121212	20.92147601	0.827915985	0.019949061	+	105371067	grey	-0.250374265	FALSE	LOC105371067
Region_6551	chr16	5239802	7713340	2473538	535	4215	4473.893939	120.0344987	0.826177614	0.010430314	+	54715	grey	-0.157392349	FALSE	RBFOX1
Region_6552	chr16	5596856	5616373	19517	4	17	33.25757576	6.531133972	0.676939415	0.056581467	-	101926950	grey	-0.114382698	FALSE	LINC01570
Region_6553	chr16	7878799	8112756	233957	27	175	210.6060606	16.45775832	0.833409965	0.035453399	-	105371070	grey	9.8621E-05	FALSE	LOC105371070
Region_6554	chr16	8526549	8590511	63962	19	142	167.6212121	11.90832308	0.791643881	0.031935724	-	283953	grey	-0.046485923	FALSE	TMEM114
Region_6555	chr16	8621683	8649654	27971	3	13	21.28787879	4.57338505	0.325853594	0.02233178	+	79091	grey	-0.095274394	FALSE	METTL22
Region_6556	chr16	8674596	8784575	109979	39	243	279.8333333	16.41161252	0.918919107	0.017910276	+	18	grey	-0.245585105	FALSE	ABAT
Region_6557	chr16	8780384	8797642	17258	12	87	106.3333333	9.185830783	0.786873888	0.023096655	-	25880	grey	-0.036503935	FALSE	TMEM186
Region_6558	chr16	8788823	8862124	73301	34	221	257.5909091	18.47241375	0.795049506	0.034765514	+	5373	grey	0.338281065	FALSE	PMM2
Region_6559	chr16	8797842	8860417	62575	27	165	198.8030303	17.39515379	0.824881226	0.042462798	+	100130283	grey	0.383944195	FALSE	LOC100130283
Region_6560	chr16	8852942	8869012	16070	9	45	63.03030303	8.859535839	0.683520608	0.038317279	-	23589	grey	0.105055579	FALSE	CARHSP1
Region_6561	chr16	8892097	8975328	83231	77	483	568.1363636	28.9316895	0.562628833	0.015523712	-	7874	grey	0.120850478	FALSE	USP7
Region_6562	chr1	8962706	8966990	4284	25	96	145.772727	18.6587125	0.12177509	0.01236463	+	10537107	grey	0.068243002	FALSE	USP7-AS1

	6						3	1	9	4		4				
Region_6563	chr16	9091644	9121635	29991	19	113	146.3030303	14.22453968	0.703215551	0.021862141	+	29035	grey	-0.079849524	FALSE	HAPSTR1
Region_6564	chr16	9355588	9408093	52505	10	50	70.07575758	8.75532472	0.910341307	0.036452977	+	101927009	grey	-0.123144397	FALSE	LINC02177
Region_6565	chr16	9365540	9614899	249359	35	225	277.2878788	19.02934184	0.876430213	0.022959853	+	101927026	grey	0.114684403	FALSE	LOC101927026
Region_6566	chr16	9441294	9444985	3691	3	11	22.75757576	5.443586505	0.934042859	0.05339622	-	104355218	grey	0.090019045	FALSE	LINC01177
Region_6567	chr16	9753404	10182928	429524	58	439	499.3787879	24.84893754	0.840793712	0.020844573	-	2903	grey	0.200835906	FALSE	GRIN2A
Region_6568	chr16	10214784	10227581	12797	4	14	25.15151515	4.996129038	0.822313009	0.102343894	-	107984900	grey	-0.148880021	FALSE	LOC107984900
Region_6569	chr16	10326434	10483638	157204	63	458	514.2424242	25.39416081	0.875342052	0.010891887	+	80063	grey	0.091471268	FALSE	ATF7IP2
Region_6570	chr16	10514842	10528202	13360	6	35	52.78787879	7.054764133	0.933123627	0.03863487	-	106144584	grey	0.143382514	FALSE	LINC01290
Region_6571	chr16	10528422	10580632	52210	19	124	149.1969697	12.14301914	0.515164243	0.030939079	-	2013	grey	0.197710056	FALSE	EMP2
Region_6572	chr16	10627501	10694930	67429	21	144	171.0757576	12.14060017	0.866084777	0.037724492	-	146279	grey	0.220988947	FALSE	TEKT5
Region_6573	chr16	10743786	10769351	25565	10	53	75.90909091	8.329239087	0.653734664	0.030906715	+	4682	grey	-0.099034746	FALSE	NUBP1
Region_6574	chr16	10760919	10818794	57875	22	139	174.6060606	13.66171381	0.918639407	0.024495476	-	780776	grey	-0.071470972	FALSE	TVP23A

Region_6575	chr16	10864914	10888752	23838	13	82	113.8939394	10.91805673	0.33754293	0.043371688	-	105371080	grey	0.288489144	FALSE	
Region_6576	chr16	10866222	10943021	76799	45	304	349.6969697	18.31936319	0.497694066	0.019425121	+	4261	grey	0.00102891	FALSE	CIITA
Region_6577	chr16	10928891	10942468	13577	12	56	85.06060606	9.223513077	0.4292854	0.035638483	-	28955	grey	-0.058313452	FALSE	DEXI
Region_6578	chr16	10944539	11182186	237647	100	800	856.8333333	29.06734003	0.901387086	0.012798707	+	23274	grey	0.023471264	FALSE	CLEC16A
Region_6579	chr16	11196177	11224969	28792	13	75	101.9545455	9.92809815	0.865505064	0.032810166	+	107984859	grey	0.221498582	FALSE	LOC107984859
Region_6580	chr16	11249619	11381662	132043	71	461	513.2424242	25.45708945	0.76481996	0.017046151	+	105371082	grey	0.295114436	FALSE	LOC105371082
Region_6581	chr16	11249649	11351760	102111	62	405	443.1969697	21.15017345	0.745257748	0.018844766	+	116028	grey	0.262289233	FALSE	RMI2
Region_6582	chr16	11254417	11256204	1787	4	16	29.01515152	6.410310023	0.019350546	0.025214936	-	8651	grey	-0.172799316	FALSE	SOCS1
Region_6583	chr16	11359845	11527247	167402	91	620	689.3939394	34.2685677	0.838413879	0.017249677	-	400499	grey	0.247670494	FALSE	LOC400499
Region_6584	chr16	11465260	11473174	7914	4	23	35.03030303	5.997357616	0.89061192	0.08172633	+	101927131	grey	0.44919545	FALSE	LOC101927131
Region_6585	chr16	11547722	11636381	88659	48	340	394.6969697	24.73799301	0.731784502	0.02693493	-	9516	grey	-0.087052444	FALSE	LITAF
Region_6586	chr16	11668455	11679152	10697	8	32	49.09090909	6.441995087	0.874655089	0.068160548	+	8303	grey	-0.08593349	FALSE	SNN
Region_6587	chr1	11679083	11742857	63774	40	271	320.484848	21.9280238	0.68434906	0.01983255	-	51061	grey	0.189942641	FALSE	TXNDC11

	6						5	2	1	4						
Region_6588	chr16	11750586	11797258	46672	13	86	108.1666667	10.63026642	0.665581256	0.034519089	-	29066	grey	-0.070478831	FALSE	ZC3H7A
Region_6589	chr16	11819829	11828845	9016	3	17	26.51515152	4.906016477	0.917529878	0.052773366	-	400500	grey	-0.024674418	FALSE	BCAR4
Region_6590	chr16	11833850	11851580	17730	12	78	100.3636364	10.83532168	0.658059571	0.03802756	-	26156	grey	-0.035830635	FALSE	RSL1D1
Region_6591	chr16	11851622	11907054	55432	28	176	217.6818182	18.0751926	0.863751131	0.020537446	+	105371084	grey	-0.081607227	FALSE	RSL1D1-DT
Region_6592	chr16	11868128	11916082	47954	36	229	268.1363636	19.58169189	0.725922111	0.017760447	-	2935	grey	0.066314804	FALSE	GSPT1
Region_6593	chr16	11927259	11976643	49384	17	97	119.4242424	9.735044713	0.832617473	0.029863216	-	729978	grey	-0.190577473	FALSE	NPIPB2
Region_6594	chr16	11976734	12574287	597553	253	1978	2119.621212	69.24466437	0.894554939	0.00926639	+	92017	grey	0.025582489	FALSE	SNX29
Region_6595	chr16	12086746	12090302	3556	3	14	22.24242424	4.325454555	0.935028218	0.05156288	-	642696	grey	-0.015013745	FALSE	LOC642696
Region_6596	chr16	12366982	12372582	5600	5	29	44.12121212	7.10907362	0.868353866	0.057065035	-	101927227	grey	-0.057439465	FALSE	LOC101927227
Region_6597	chr16	12659799	12803887	144088	56	437	503.1212121	26.22771004	0.906193177	0.014615218	-	55313	grey	0.170461667	FALSE	CPPED1
Region_6598	chr16	12745873	12757835	11962	5	32	43.28787879	6.001961606	0.96598344	0.027790931	+	105371090	grey	0.18095435	FALSE	LOC105371090
Region_6599	chr16	12901598	13563388	661790	115	929	1003.909091	37.34031736	0.82397147	0.017077429	+	729993	grey	-0.251444436	FALSE	SHISA9

Region_6600	chr16	13246232	13316729	70497	11	64	84.72727273	8.215455331	0.844033952	0.05014535	+	107984137	grey	-0.421500235	FALSE	LOC107984137
Region_6601	chr16	13920138	13952348	32210	6	45	61.75757576	7.166289902	0.664377446	0.065966704	+	2072	grey	0.284642013	FALSE	ERCC4
Region_6602	chr16	14001198	14016056	14858	7	48	61.63636364	6.819962471	0.733595685	0.043129969	-	101927311	grey	0.031263488	FALSE	LINC02185
Region_6603	chr16	14071319	14266773	195454	35	265	306.5909091	17.08438538	0.949145499	0.013516818	+	57496	grey	0.465245633	FALSE	MRTFB
Region_6604	chr16	14301389	14331067	29678	14	94	114.1363636	9.944285353	0.872389766	0.030803039	+	100129781	grey	0.08197341	FALSE	MIR193BHG
Region_6605	chr16	14363109	14370266	7157	3	11	22.25757576	4.393388761	0.884508085	0.102775428	-	105447648	grey	-0.221309917	FALSE	LINC02130
Region_6606	chr16	14911551	14952060	40509	12	46	89.46969697	22.32778173	0.950742668	0.029271431	+	109951030	grey	-0.140628562	FALSE	PKD1P3-NPIPA1
Region_6607	chr16	17933189	18151595	218406	35	244	288.2575758	20.09692713	0.83056073	0.025655799	-	107984893	grey	0.121586847	FALSE	LOC107984893
Region_6608	chr16	18358086	18401940	43854	8	42	63.66666667	11.86634975	0.967403906	0.039360234	-	110006322	grey	-0.005543533	FALSE	PKD1P5-LOC105376752
Region_6609	chr16	18417325	18562211	144886	16	57	103.8333333	14.18296197	0.698302709	0.030580793	-	283820	grey	0.314630506	FALSE	NOMO2
Region_6610	chr16	18781295	18790383	9088	5	34	47.48484848	6.036408571	0.537822823	0.056672099	-	6210	grey	-0.045847729	FALSE	RPS15A
Region_6611	chr16	18788063	18801572	13509	15	99	124.0454545	11.35230484	0.460759718	0.030572274	-	23204	grey	-0.008127143	FALSE	ARL6IP1
Region_6612	chr1	18804860	18926408	121548	30	203	238.636363	15.4667249	0.86143104	0.02243250	-	23049	grey	-0.12158788	FALSE	SMG1

	6						6	4	1	5				3		
Region_6613	chr16	18926863	18937043	10180	7	37	51.68181818	7.728387414	0.938066783	0.051871931	+	107984844	grey	0.109687792	FALSE	SMG1-DT
Region_6614	chr16	18983934	19063942	80008	36	215	275.9242424	19.32579831	0.882714104	0.01264896	+	79905	grey	0.238079615	FALSE	TMC7
Region_6615	chr16	19062144	19067691	5547	3	10	18.5	4.095964243	0.968610192	0.049553656	-	102723385	grey	-0.053111131	FALSE	COQ7-DT
Region_6616	chr16	19067614	19080095	12481	8	39	59.43939394	8.167720307	0.462011731	0.035246894	+	10229	grey	-0.121186159	FALSE	COQ7
Region_6617	chr16	19113932	19173578	59646	29	198	241.0151515	16.40215035	0.818550447	0.019994212	+	162073	grey	0.217328746	FALSE	ITPRIPL2
Region_6618	chr16	19167971	19268332	100361	27	190	229.0909091	16.14713813	0.747741603	0.023010074	+	51760	grey	0.210051584	FALSE	SYT17
Region_6619	chr16	19343647	19401693	58046	10	52	67.53030303	9.817127415	0.800749884	0.048758794	-	105371114	grey	-0.066134985	FALSE	LOC105371114
Region_6620	chr16	19410496	19499113	88617	20	127	154.5606061	13.56026865	0.789617908	0.021038564	+	79838	grey	-0.221182099	FALSE	TMC5
Region_6621	chr16	19523811	19553408	29597	7	33	50.03030303	8.197503742	0.977817906	0.021490409	+	9738	grey	-0.086895982	FALSE	CCP110
Region_6622	chr16	19555240	19706793	151553	56	420	465.9545455	25.03562497	0.920469598	0.012533514	+	57020	grey	-0.335393078	FALSE	VPS35L
Region_6623	chr16	19701937	19718235	16298	8	48	64.24242424	7.97314841	0.784595061	0.040541355	-	400506	grey	-0.078368689	FALSE	KNOP1
Region_6624	chr16	19716456	19858467	142011	56	393	449.7272727	28.07930826	0.822535586	0.013396895	+	124152	grey	0.100756675	FALSE	IQCK

Region_6625	chr16	19856691	19886167	29476	25	172	201.4242424	14.04942989	0.590592788	0.019260446	-	51704	grey	-0.001418626	FALSE	GPRC5B
Region_6626	chr16	20028239	20073890	45651	10	82	99.06060606	7.910808162	0.777284803	0.039711826	-	124274	grey	0.068618333	FALSE	GPR139
Region_6627	chr16	20309574	20327808	18234	5	30	42.34848485	5.458916275	0.908345749	0.051229101	-	2813	grey	0.144941999	FALSE	GP2
Region_6628	chr16	20333051	20356301	23250	7	49	72.90909091	10.01341757	0.651538702	0.074703214	-	7369	grey	-0.27300328	FALSE	UMOD
Region_6629	chr16	20359175	20404737	45562	11	75	92.63636364	9.04286606	0.840407364	0.048887473	-	204474	grey	0.233878915	FALSE	PDILT
Region_6630	chr16	20409534	20441336	31802	4	24	40.5	6.421717958	0.864104275	0.071460178	+	54988	grey	0.220861954	FALSE	ACSM5
Region_6631	chr16	20536226	20576427	40201	3	14	28.37878788	5.377346076	0.866695132	0.075629707	-	348158	grey	0.030069027	FALSE	ACSM2B
Region_6632	chr16	20610243	20797581	187338	19	157	186.4090909	13.94602482	0.878588463	0.022089182	+	6296	grey	-0.173577438	FALSE	ACSM3
Region_6633	chr16	20623235	20698890	75655	8	61	81.33333333	9.498717862	0.913443033	0.035523196	-	116285	grey	0.030614331	FALSE	ACSM1
Region_6634	chr16	20674510	20755608	81098	6	40	52.42424242	7.075549679	0.785508998	0.041372248	+	105379461	grey	-0.230037523	FALSE	
Region_6635	chr16	20702816	20742084	39268	3	15	25.53030303	5.318240591	0.900072704	0.076398389	-	55623	grey	-0.160165779	FALSE	THUMPD1
Region_6636	chr16	20780193	20900349	120156	16	127	157.3636364	13.10623617	0.852918513	0.038465409	-	112479	grey	-0.105059621	FALSE	ERI2
Region_6637	chr1	20806429	20849665	43236	7	41	66.5454545	8.08451857	0.86924600	0.03243403	+	81691	grey	-0.09928790	FALSE	REXO5

	6						5	3	2	3				6		
Region_6638	chr16	20854925	20900358	45433	4	23	38.34848485	5.990999465	0.801378918	0.100889178	-	123879	grey	-0.153480426	FALSE	DCUN1D3
Region_6639	chr16	20933111	21159441	226330	59	398	454.7272727	24.16389261	0.885258817	0.016792619	-	55567	grey	-0.038786183	FALSE	DNAH3
Region_6640	chr16	21116439	21130108	13669	4	21	30.90909091	5.524009895	0.95935164	0.04262134	+	107984888	grey	-0.021774693	FALSE	LOC107984888
Region_6641	chr16	21158377	21180616	22239	15	95	128.1969697	11.69117971	0.777273345	0.021165963	+	57146	grey	0.057769118	FALSE	LDAF1
Region_6642	chr16	21238874	21303083	64209	20	132	161.469697	13.8821631	0.607636849	0.031226949	-	1428	grey	0.187398245	FALSE	CRYM
Region_6643	chr16	21300849	21339037	38188	20	114	158.5	15.40754061	0.910929045	0.015238419	+	400508	grey	-0.038682376	FALSE	CRYM-AS1
Region_6644	chr16	21347139	21386064	38925	26	170	213.3181818	18.38574623	0.913261848	0.028356398	+	100652781	grey	-0.300348346	FALSE	SNX29P1
Region_6645	chr16	21519830	21520365	535	3	27	48.21212121	9.404606321	0.006471325	0.012753332	-	387254	grey	0.13224204	FALSE	SLC7A5P2
Region_6646	chr16	21796106	21820444	24338	3	11	20.34848485	3.939904037	0.978762771	0.031025394	-	730092	grey	0.13031133	FALSE	RRN3P1
Region_6647	chr16	22092538	22156964	64426	12	66	84.12121212	8.896870931	0.882882683	0.037759403	+	146177	grey	0.172903724	FALSE	VWA3A
Region_6648	chr16	22162492	22192208	29716	12	76	105.9090909	11.61395351	0.77044618	0.028777633	+	100288072	grey	-0.03327622	FALSE	SDR42E2
Region_6649	chr16	22345936	22431596	85660	29	203	232.7727273	15.6910513	0.814833019	0.019158579	-	1039	grey	0.313793143	FALSE	CDR2

Region_6650	chr16	22349372	22437715	88343	37	260	296.1818182	19.4279015	0.638922579	0.01550689	-	100131998	grey	0.31023955	FALSE	RRN3P3
Region_6651	chr16	22382436	22407598	25162	10	55	75.13636364	9.880651432	0.964250255	0.026842817	+	105371130	grey	0.26935725	FALSE	MFSD13B
Region_6652	chr16	22545698	22576865	31167	5	36	60.09090909	10.41408406	0.918929669	0.050635907	+	653786	grey	0.159847485	FALSE	OTOAP1
Region_6653	chr16	22814162	22916338	102176	22	154	187.1969697	17.9839413	0.855045915	0.036124408	+	9956	grey	-0.04886626	FALSE	HS3ST2
Region_6654	chr16	23017723	23026285	8562	3	10	21.93939394	4.324807823	0.91887847	0.065420085	+	112268176	grey	-0.190237131	FALSE	LOC112268176
Region_6655	chr16	23061406	23149452	88046	26	161	200.969697	16.63358021	0.670620317	0.018820436	-	57478	grey	0.108332936	FALSE	USP31
Region_6656	chr16	23182745	23216883	34138	10	53	75.24242424	7.874126288	0.662236766	0.043145487	+	6340	grey	-0.058319246	FALSE	SCNN1G
Region_6657	chr16	23278231	23381294	103063	41	292	345.6212121	19.07052914	0.850286608	0.020909886	+	6338	grey	0.00250371	FALSE	SCNN1B
Region_6658	chr16	23388493	23453189	64696	31	229	255	14.8903686	0.861235589	0.013487068	-	91949	grey	-0.276692776	FALSE	COG7
Region_6659	chr16	23463542	23521995	58453	30	194	227.1666667	14.96003222	0.781406963	0.019717159	-	23062	grey	0.059266257	FALSE	GGA2
Region_6660	chr16	23520754	23557731	36977	17	109	136.0909091	13.68292285	0.953700203	0.019569073	-	124454	grey	-0.100337878	FALSE	EARS2
Region_6661	chr16	23557721	23574389	16668	9	55	72.18181818	8.628947865	0.92034107	0.031696443	+	56061	grey	0.088754133	FALSE	UBFD1
Region_6662	chr1	23581014	23596316	15302	16	87	121.227272	12.4946701	0.91319757	0.01592214	-	4706	grey	-0.01701353	FALSE	NDUFAB1

	6						7	9	7	4						
Region_6663	chr16	23603160	23641321	38161	13	83	104.8030303	9.69094692	0.952275708	0.022553089	-	79728	grey	0.22537509	FALSE	PALB2
Region_6664	chr16	23641466	23677472	36006	4	13	28.15151515	5.71164108	0.715941464	0.050954241	+	84516	grey	0.025853841	FALSE	DCTN5
Region_6665	chr16	23677656	23690367	12711	9	56	81.77272727	11.57822287	0.758816583	0.019951433	+	5347	grey	-0.135920435	FALSE	PLK1
Region_6666	chr16	23690310	23713226	22916	12	77	99.90909091	11.15033945	0.902920269	0.028450886	-	10595	grey	-0.044678213	FALSE	ERN2
Region_6667	chr16	23835983	24220611	384628	75	548	612.469697	26.93766005	0.802704126	0.013264739	+	5579	grey	0.084471723	FALSE	PRKCB
Region_6668	chr16	24256335	24362412	106077	20	168	191.2878788	12.47861574	0.88505855	0.026407576	+	10368	grey	0.077716054	FALSE	CACNG3
Region_6669	chr16	24472798	24495177	22379	5	19	29.46969697	4.431425181	0.880395617	0.065316764	-	105371143	grey	0.138722488	FALSE	LOC105371143
Region_6670	chr16	24537693	24572863	35170	3	15	27.07575758	4.965451737	0.588522177	0.098340671	+	5930	grey	-0.297681757	FALSE	RBBP6
Region_6671	chr16	24610209	24827632	217423	70	499	561.2424242	25.58188097	0.879742896	0.01330909	+	27327	grey	0.043616901	FALSE	TNRC6A
Region_6672	chr16	24661422	24671062	9640	5	34	45.75757576	6.177030917	0.872778183	0.082116201	-	400511	grey	0.15789067	FALSE	LINC01567
Region_6673	chr16	25066937	25088618	21681	11	74	93.92424242	9.646538807	0.778867091	0.028960661	+	283887	grey	0.124024471	FALSE	LINC02175
Region_6674	chr16	25085168	25111555	26387	8	53	65.43939394	6.766725803	0.843035341	0.027038439	-	102723510	grey	0.082673423	FALSE	LCMT1-AS1

Region_6675	chr16	25111731	25178231	66500	21	124	160.530303	14.10970173	0.799252652	0.027285748	+	51451	grey	0.161029044	FALSE	LCMT1
Region_6676	chr16	25215731	25228932	13201	8	51	66.07575758	9.492200209	0.880082246	0.045558808	+	343	grey	0.043241925	FALSE	AQP8
Region_6677	chr16	25236001	25257845	21844	8	28	51.21212121	7.190838512	0.791638473	0.0424671	-	342357	grey	-0.1336847	FALSE	ZKSCAN2
Region_6678	chr16	25257952	25261066	3114	4	12	22.21212121	4.53198938	0.037029795	0.03861616	+	112577464	grey	-0.369589459	FALSE	ZKSCAN2-DT
Region_6679	chr16	25691959	26137685	445726	86	655	742.1969697	27.25109437	0.810847733	0.01474744	+	9951	grey	0.184843827	FALSE	HS3ST4
Region_6680	chr16	26584755	26595456	10701	3	18	26.87878788	4.941239806	0.857401115	0.068500686	-	105371152	grey	-0.028258655	FALSE	LINC02195
Region_6681	chr16	27158451	27176587	18136	4	20	30.65151515	5.390853862	0.760463232	0.111263754	-	105371156	grey	-0.063662786	FALSE	LINC02129
Region_6682	chr16	27203508	27221768	18260	14	92	121.3484848	12.41400255	0.80574807	0.030758606	+	79831	grey	0.210742188	FALSE	KDM8
Region_6683	chr16	27224994	27268772	43778	25	172	220.3939394	15.83266224	0.543271176	0.027696143	-	197370	grey	0.022173092	FALSE	NSMCE1
Region_6684	chr16	27268205	27290492	22287	13	82	108.7121212	11.34191023	0.515329769	0.018443931	+	400512	grey	0.209468946	FALSE	NSMCE1-DT
Region_6685	chr16	27313668	27364778	51110	20	116	148.8939394	12.30775714	0.738237043	0.027195847	+	3566	grey	0.068852373	FALSE	IL4R
Region_6686	chr16	27402174	27452042	49868	15	103	132.6818182	12.99130963	0.864585379	0.035726728	+	50615	grey	0.133308613	FALSE	IL21R
Region_6687	chr1	27447669	27453393	5724	3	14	24.0606060	5.17948256	0.95873401	0.04506608	-	283888	grey	0.199120719	FALSE	IL21R-AS1

	6						6	4	3	2						
Region_6688	chr16	27460613	27549913	89300	50	410	446.7424242	18.3624287	0.931634214	0.012790283	-	2975	grey	-0.153434155	FALSE	GTF3C1
Region_6689	chr16	27550133	27780344	230211	85	629	686.8181818	31.17538925	0.890243059	0.009477016	+	23247	grey	-0.036245891	FALSE	KATNIP
Region_6690	chr16	27678940	27718806	39866	14	100	122.4393939	11.21417892	0.845947569	0.022858638	-	100128079	grey	-0.208510036	FALSE	LOC100128079
Region_6691	chr16	27787528	28063714	276186	80	560	632.1818182	34.3698925	0.799839121	0.018592528	-	146395	grey	-0.152566065	FALSE	GSG1L
Region_6692	chr16	28097976	28211965	113989	44	292	331.3333333	17.91632975	0.861006955	0.022453504	-	23214	grey	0.116165824	FALSE	XPO6
Region_6693	chr16	28258686	28323849	65163	44	279	327.3939394	20.91071039	0.776571365	0.020843659	+	388228	grey	-0.06763638	FALSE	SBK1
Region_6694	chr16	28456329	28482634	26305	9	46	66.75757576	8.994118773	0.967906555	0.026191989	-	440350	grey	-0.094223521	FALSE	NPIPB7
Region_6695	chr16	28456371	28495567	39196	16	83	111.5151515	12.25905307	0.849051509	0.016882476	-	1201	grey	-0.084421393	FALSE	CLN3
Region_6696	chr16	28499362	28512051	12689	9	44	62.5	8.47121824	0.734494845	0.057079349	-	246778	grey	-0.044298865	FALSE	IL27
Region_6697	chr16	28532708	28620133	87425	45	278	342.7878788	22.54466688	0.875075903	0.018489229	-	26471	grey	-0.105741041	FALSE	NUPR1
Region_6698	chr16	28553915	28591790	37875	18	113	139.1060606	13.26200912	0.893346576	0.019233723	+	112869	grey	-0.183171735	FALSE	SGF29
Region_6699	chr16	28591943	28623363	31420	23	128	165.6060606	15.93822574	0.815578922	0.027573966	-	6799	grey	0.151210475	FALSE	SULT1A2

Region_6700	chr16	28605196	28623625	18429	17	92	128.6515152	16.31751529	0.672590841	0.024827765	-	6817	grey	-0.049407585	FALSE	SULT1A1
Region_6701	chr16	28637654	28658744	21090	5	20	32.27272727	6.065147483	0.942717629	0.046337576	+	728734	grey	-0.144663374	FALSE	NPIPB8
Region_6702	chr16	28688558	28735727	47169	8	18	55.15151515	14.57314962	0.917340467	0.056283477	+	8663	grey	0.22981835	FALSE	EIF3C
Region_6703	chr16	28822999	28837237	14238	23	132	176.7424242	15.48032061	0.865657337	0.02063602	+	11273	grey	0.108097616	FALSE	ATXN2L
Region_6704	chr16	28842411	28846348	3937	4	24	35.12121212	5.458980326	0.490168102	0.018072021	-	7284	grey	-0.022021835	FALSE	TUFM
Region_6705	chr16	28846600	28874212	27612	24	119	149.0606061	14.49018637	0.882594817	0.02549091	+	25970	grey	-0.124046978	FALSE	SH2B1
Region_6706	chr16	28878405	28904466	26061	33	165	192.9848485	14.49296108	0.625497659	0.024571521	+	487	grey	0.05014203	FALSE	ATP2A1
Region_6707	chr16	28878957	28879938	981	6	13	28.72727273	6.254956077	0.066443293	0.037714915	-	100289092	grey	-0.090625264	FALSE	ATP2A1-AS1
Region_6708	chr16	28904421	28936526	32105	36	189	236.0909091	18.84326223	0.714859792	0.018217115	-	79874	grey	0.035846816	FALSE	RABEP2
Region_6709	chr16	28931965	28939342	7377	14	63	90.07575758	12.7234618	0.417094362	0.049867546	+	930	grey	-0.199714587	FALSE	CD19
Region_6710	chr16	28950807	28967092	16285	14	84	111.0757576	10.80215165	0.914210751	0.026552122	+	84901	grey	-0.101636187	FALSE	NFATC2IP
Region_6711	chr16	28974221	28990775	16554	11	61	77.12121212	8.921047113	0.719977535	0.06075266	+	83985	grey	0.222989161	FALSE	SPNS1
Region_6712	chr1	28984826	28990784	5958	6	25	38.8787878	7.23137427	0.84531925	0.10198834	+	27040	grey	0.270937459	FALSE	LAT

	6						8	4		2						
Region_6713	chr16	29053732	29108108	54376	25	154	187.2878788	14.11247691	0.777405106	0.024996152	-	101928215	grey	-0.021547169	FALSE	LOC101928215
Region_6714	chr16	29074976	29116718	41742	21	142	168.5606061	14.93486246	0.816591345	0.027432082	+	653390	grey	-0.125359401	FALSE	RRN3P2
Region_6715	chr16	29114717	29217860	103143	93	680	771.2878788	51.69370298	0.856859481	0.016571953	+	107984832	grey	0.240079642	FALSE	
Region_6716	chr16	29139379	29372110	232731	204	1504	1675.863636	81.30472337	0.886881242	0.011786098	+	440352	grey	0.101371818	FALSE	SNX29P2
Region_6717	chr16	29381310	29406711	25401	7	26	40.72727273	7.729156496	0.900250387	0.052433562	-	728888	grey	0.386315518	FALSE	NPIPB11
Region_6718	chr16	29662979	29670876	7897	9	27	52.04545455	7.768638764	0.128704731	0.024704596	+	6693	grey	0.00288638	FALSE	SPN
Region_6719	chr16	29663279	29698699	35420	30	167	198.0606061	12.98387022	0.607081575	0.024613004	+	23475	grey	0.009372469	FALSE	QPRT
Region_6720	chr16	29790727	29805385	14658	8	36	52.77272727	6.639729963	0.605207477	0.024800824	+	3835	grey	-0.096757875	FALSE	KIF22
Region_6721	chr16	29806106	29812227	6121	4	10	26.98484848	5.595306891	0.010051817	0.020973933	+	4150	grey	-0.023814497	FALSE	MAZ
Region_6722	chr16	29808636	29820366	11730	22	105	131.7727273	13.51101597	0.249476357	0.014782891	-	112268170	grey	-0.336323987	FALSE	MVP-DT
Region_6723	chr16	29811547	29820092	8545	21	102	125.5909091	12.72789459	0.261249721	0.015509168	+	112476	grey	-0.338762327	FALSE	PRRT2
Region_6724	chr16	29816152	29830627	14475	27	136	169.8030303	14.54247857	0.438200028	0.023834147	+	79447	grey	-0.373890774	FALSE	PAGR1

Region_6725	chr16	29820394	29848039	27645	19	108	144.969697	12.9792375	0.867824166	0.034784589	+	9961	grey	-0.196081777	FALSE	MVP
Region_6726	chr16	29858357	29863414	5057	5	31	46.21212121	7.733076115	0.387971095	0.025176062	-	10423	grey	0.233912761	FALSE	CDIPT
Region_6727	chr16	29871159	29899550	28391	23	133	169.7272727	13.2078595	0.918218512	0.021985623	-	26470	grey	-0.041530597	FALSE	SEZ6L2
Region_6728	chr16	29900375	29919864	19489	19	113	143.5454545	13.16660323	0.888254941	0.035194868	+	253982	grey	-0.048102222	FALSE	ASPHD1
Region_6729	chr16	29905012	29926236	21224	18	113	132.5151515	11.40612848	0.804399644	0.031830186	-	253980	grey	-0.271294086	FALSE	KCTD13
Region_6730	chr16	29940885	29973050	32165	18	126	158.7575758	13.56699684	0.9469709	0.022139253	+	124446	grey	-0.23458564	FALSE	TMEM219
Region_6731	chr16	29973868	29992261	18393	18	105	127.6969697	10.80024173	0.79444681	0.033248638	+	9344	grey	-0.275178607	FALSE	TAOK2
Region_6732	chr16	29992330	29996074	3744	3	14	24.84848485	5.174619791	0.332269484	0.020415283	-	8479	grey	-0.028480824	FALSE	HIRIP3
Region_6733	chr16	29995715	30005793	10078	24	155	187.5606061	14.84911682	0.714564472	0.025177044	+	283899	grey	-0.044882834	FALSE	INO80E
Region_6734	chr16	30005514	30023270	17756	34	191	225.6363636	17.14340991	0.707778757	0.024560905	-	8448	grey	0.172227997	FALSE	DOC2A
Region_6735	chr16	30023198	30027736	4538	5	18	25.31818182	5.057086698	0.82704767	0.083799777	+	146378	grey	0.12171096	FALSE	C16orf92
Region_6736	chr16	30024427	30052978	28551	29	164	205.2878788	16.29984913	0.823838954	0.024740575	-	83723	grey	-0.123384561	FALSE	TLCD3B
Region_6737	chr1	30053123	30070414	17291	20	100	124.378787	12.2484098	0.33638167	0.01936829	+	11269475	grey	0.124254665	FALSE	LOC112694756

	6						9	2	6	8		6				
Region_6738	chr16	30064274	30070457	6183	10	35	55.75757576	7.992420652	0.101715727	0.010884832	+	226	grey	-0.186091364	FALSE	ALDOA
Region_6739	chr16	30075978	30085376	9398	17	109	134.0606061	13.12763238	0.53793294	0.022880637	+	5531	grey	0.037757985	FALSE	PPP4C
Region_6740	chr16	30092314	30096915	4601	6	25	36.31818182	7.099640491	0.326890416	0.043673606	-	83719	grey	0.121722602	FALSE	YPEL3
Region_6741	chr16	30096430	30105193	8763	12	70	90.89393939	9.658999451	0.819220326	0.02520685	+	101928595	grey	0.151113501	FALSE	YPEL3-DT
Region_6742	chr16	30104810	30113537	8727	5	23	37.89393939	6.488163869	0.960880489	0.038246393	-	79153	grey	0.153614335	FALSE	GDPD3
Region_6743	chr16	30114105	30123506	9401	5	25	35.92424242	6.288721543	0.947984565	0.056117691	-	5595	grey	-0.349593426	FALSE	MAPK3
Region_6744	chr16	30182827	30189076	6249	7	21	37.66666667	7.533174493	0.222288726	0.044715341	+	11151	grey	0.019547946	FALSE	CORO1A
Region_6745	chr16	30204316	30207182	2866	4	27	48.78787879	10.01616409	0.021139783	0.036914954	-	613038	grey	-0.182765645	FALSE	LOC613038
Region_6746	chr16	30267553	30335395	67842	13	74	95.40909091	9.860530915	0.951050915	0.026434995	-	595101	grey	0.242395701	FALSE	SMG1P5
Region_6747	chr16	30350773	30355308	4535	6	14	29.8030303	6.154972216	0.319517688	0.051280339	-	10421	grey	-0.172703421	FALSE	CD2BP2
Region_6748	chr16	30357102	30370494	13392	11	65	80.04545455	8.404086585	0.610830913	0.026030801	-	26000	grey	0.016119495	FALSE	TBC1D10B
Region_6749	chr16	30370934	30377991	7057	7	30	44.45454545	6.142366916	0.781198113	0.045670824	+	29895	grey	0.144001442	FALSE	MYL11

Region_6750	chr16	30378135	30382815	4680	7	28	39.27272727	5.620143591	0.317937661	0.063260413	-	1731	grey	0.193881185	FALSE	SEPTIN1
Region_6751	chr16	30378312	30400108	21796	38	219	251.4090909	17.03026923	0.62569007	0.025483113	+	197407	grey	0.140282823	FALSE	ZNF48
Region_6752	chr16	30407414	30431108	23694	46	319	401.0151515	34.66698382	0.363797756	0.0137301	+	51333	grey	0.095000133	FALSE	ZNF771
Region_6753	chr16	30423615	30430030	6415	12	77	104.4242424	11.62234003	0.597436577	0.025232429	-	79077	grey	0.101279892	FALSE	DCTPP1
Region_6754	chr16	30472658	30523567	50909	19	119	149.9090909	13.89048072	0.930674774	0.026983411	+	3683	grey	-0.032991505	FALSE	ITGAL
Region_6755	chr16	30525923	30535347	9424	16	98	122.469697	12.07313572	0.389863534	0.025017961	-	65988	grey	-0.084925978	FALSE	ZNF747
Region_6756	chr16	30534752	30537149	2397	5	27	43.25757576	7.342836606	0.00525948	0.012904014	+	107984875	grey	0.125796004	FALSE	ZNF747-DT
Region_6757	chr16	30553764	30558374	4610	6	27	40.33333333	6.219901887	0.425923909	0.048048608	-	92595	grey	0.079449338	FALSE	ZNF764
Region_6758	chr16	30569672	30572734	3062	13	76	102.4242424	13.77853471	0.697448848	0.027777839	-	146542	grey	-0.448865581	FALSE	ZNF688
Region_6759	chr16	30573740	30585769	12029	19	102	141.8636364	16.64921931	0.920962503	0.014853055	-	146540	grey	-0.18721427	FALSE	ZNF785
Region_6760	chr16	30602558	30624012	21454	11	79	98.09090909	9.151254261	0.837185622	0.036390663	-	115509	grey	-0.381355291	FALSE	ZNF689
Region_6761	chr16	30650717	30656413	5696	3	12	20.42424242	3.906840573	0.018811492	0.031620821	+	78994	grey	0.091352198	FALSE	PRR14
Region_6762	chr1	30658431	30670810	12379	25	131	157.318181	12.8950307	0.92855749	0.01667639	+	64319	grey	-0.35308225	FALSE	FBRS

	6						8	6	1	6				3		
Region_6763	chr16	30697704	30699058	1354	5	24	35.84848485	5.904997686	0.045465241	0.031209143	-	730183	grey	0.050593126	FALSE	LOC730183
Region_6764	chr16	30698209	30745169	46960	31	212	246.2121212	16.89653142	0.796663218	0.018631476	+	10847	grey	-0.047839736	FALSE	SRCAP
Region_6765	chr16	30740667	30761176	20509	13	87	104.3030303	8.945939684	0.498595709	0.027499738	+	5261	grey	0.06954672	FALSE	PHKG2
Region_6766	chr16	30757423	30762221	4798	3	14	27.03030303	4.920818246	0.030070533	0.036536545	-	90835	grey	-0.396424959	FALSE	CFAP119
Region_6767	chr16	30761745	30776307	14562	16	97	130.0454545	13.40084018	0.31286709	0.019762945	+	9810	grey	-0.366773848	FALSE	RNF40
Region_6768	chr16	30778456	30787205	8749	17	100	129.6363636	13.18581565	0.511034708	0.027932954	-	23361	grey	0.3594738	FALSE	ZNF629
Region_6769	chr16	30833626	30894302	60676	24	149	174.4242424	12.12506699	0.844192805	0.017498634	-	9274	grey	-0.22024547	FALSE	BCL7C
Region_6770	chr16	30875222	30895220	19998	10	53	70.3030303	8.212503969	0.960908193	0.026572987	+	101928736	grey	-0.118952751	FALSE	MIR762HG
Region_6771	chr16	30896614	30903547	6933	13	62	83.60606061	10.98793574	0.407453804	0.029020381	+	1489	grey	-0.137057413	FALSE	CTF1
Region_6772	chr16	30919319	30923269	3950	6	44	57.04545455	8.716795138	0.326173696	0.014807793	-	283932	grey	0.305818627	FALSE	FBXL19-AS1
Region_6773	chr16	30923055	30948783	25728	34	205	245.030303	15.46208235	0.782588622	0.031782082	+	54620	grey	-0.360883289	FALSE	FBXL19
Region_6774	chr16	30948386	30956511	8125	3	10	19.60606061	4.613954712	0.626059649	0.067862234	+	93129	grey	-0.168219337	FALSE	ORAI3

Region_6775	chr16	30957754	30984664	26910	37	197	250.030303	17.83597893	0.869183351	0.013593623	+	9739	grey	0.168924951	FALSE	SETD1A
Region_6776	chr16	30985207	30989147	3940	4	18	31.01515152	5.307447378	0.93572136	0.050978402	+	80270	grey	-0.090159618	FALSE	HSD3B7
Region_6777	chr16	30989256	31010638	21382	18	99	122.5909091	12.02302569	0.763177962	0.035617698	-	112755	grey	0.055322607	FALSE	STX1B
Region_6778	chr16	31032889	31040168	7279	5	27	42.48484848	5.876268895	0.389428571	0.02245085	+	6810	grey	-0.137823807	FALSE	STX4
Region_6779	chr16	31039080	31049868	10788	10	61	85.3030303	9.952146575	0.931263484	0.022438825	+	101928762	grey	0.0037575	FALSE	LOC101928762
Region_6780	chr16	31060843	31074240	13397	23	129	157.2575758	15.50613834	0.394368205	0.016591677	-	79759	grey	0.048864686	FALSE	ZNF668
Region_6781	chr16	31074422	31084196	9774	6	28	43.65151515	7.715104913	0.680924796	0.045047696	+	9726	grey	0.278603485	FALSE	ZNF646
Region_6782	chr16	31083437	31094602	11165	13	71	90.48484848	10.32583381	0.882515586	0.025704793	-	339105	grey	0.130911568	FALSE	PRSS53
Region_6783	chr16	31086760	31095980	9220	12	73	93.75757576	10.31363639	0.733408696	0.033421486	-	79001	grey	0.10911313	FALSE	VKORC1
Region_6784	chr16	31106107	31117892	11785	45	233	297.6060606	25.24213741	0.474622388	0.02435608	+	10295	grey	-0.145331565	FALSE	BCKDK
Region_6785	chr16	31115754	31131393	15639	39	201	268.5151515	25.81546491	0.620659501	0.025966503	+	84148	grey	-0.171741971	FALSE	KAT8
Region_6786	chr16	31138926	31150083	11157	15	77	100.6363636	11.70882161	0.544555916	0.044422981	-	146547	grey	0.095420267	FALSE	PRSS36
Region_6787	chr1	31180138	31191605	11467	15	76	102.242424	11.8659961	0.46746727	0.02574900	+	2521	grey	0.049982806	FALSE	FUS

	6						2	6	9	4						
Region_6788	chr16	31214091	31231537	17446	22	123	155.7424242	13.86341129	0.728988398	0.021314911	+	493829	grey	-0.279367498	FALSE	TRIM72
Region_6789	chr16	31259967	31332892	72925	16	102	122.6969697	11.47702682	0.770220511	0.035837668	+	3684	grey	-0.007820927	FALSE	ITGAM
Region_6790	chr16	31355134	31382999	27865	19	100	121.7575758	10.60052336	0.86280962	0.033311202	+	3687	grey	0.179800106	FALSE	ITGAX
Region_6791	chr16	31393335	31426505	33170	21	130	152.3787879	13.87944263	0.907550235	0.032770754	+	3681	grey	-0.270347839	FALSE	ITGAD
Region_6792	chr16	31432593	31443160	10567	6	32	51.07575758	7.569799522	0.787161512	0.056176589	-	283933	grey	-0.075107337	FALSE	ZNF843
Region_6793	chr16	31458080	31467166	9086	9	53	73.46969697	9.910708578	0.328470948	0.02258005	+	79798	grey	-0.131409233	FALSE	ARMC5
Region_6794	chr16	31471585	31477960	6375	16	71	104.6363636	11.79522245	0.500595035	0.04392094	+	7041	grey	-0.109058926	FALSE	TGFB1I1
Region_6795	chr16	31483002	31490769	7767	34	237	290.2878788	28.01854614	0.336321241	0.029366211	+	6524	grey	0.181901953	FALSE	SLC5A2
Region_6796	chr16	31489471	31509309	19838	10	80	97.09090909	8.785875505	0.952034419	0.021340088	-	64755	grey	0.135037369	FALSE	RUSF1
Region_6797	chr16	31568386	31569475	1089	4	16	30.40909091	7.05140962	0.018975143	0.03559114	-	440359	grey	-0.19215746	FALSE	YBX3P1
Region_6798	chr16	31713229	31793707	80478	8	46	58.83333333	7.41222137	0.843627705	0.025209545	+	124411	grey	0.0693138	FALSE	KRBOX5
Region_6799	chr16	31713247	31794869	81622	8	46	58.83333333	7.41222137	0.843627705	0.025209545	+	107983990	grey	0.0693138	FALSE	LOC107983990

Region_6800	chr16	31873807	31917357	43550	8	42	59.8030303	8.023465004	0.512044507	0.020330402	+	10308	grey	0.060563051	FALSE	ZNF267
Region_6801	chr16	32152111	32188107	35996	4	24	36.34848485	8.254588326	0.847459476	0.082245839	-	100289574	grey	0.161880071	FALSE	HERC2P4
Region_6802	chr16	35207884	35284146	76262	15	87	114.8939394	20.27133542	0.705207145	0.0525228	+	105371200	grey	0.013123387	FALSE	LOC105371200
Region_6803	chr16	46469341	46569097	99756	7	45	61.78787879	8.301093814	0.659035383	0.066403584	-	124149	grey	-0.14276668	FALSE	ANKRD26P1
Region_6804	chr16	46578591	46621379	42788	8	58	74.18181818	7.986351995	0.978514926	0.02202185	-	79801	grey	0.111165279	FALSE	SHCBP1
Region_6805	chr16	46656132	46689518	33386	11	70	90.84848485	9.892098516	0.758573608	0.038400504	-	55737	grey	0.056353158	FALSE	VPS35
Region_6806	chr16	46689643	46698394	8751	3	19	30.27272727	4.98164463	0.009864698	0.018742869	+	23594	grey	0.053193795	FALSE	ORC6
Region_6807	chr16	46702282	46790407	88125	25	151	188.8484848	12.58844699	0.871172348	0.021861686	-	91807	grey	-0.067646556	FALSE	MYLK3
Region_6808	chr16	46796603	46831180	34577	3	17	25.22727273	4.359941618	0.659721038	0.015927894	-	388272	grey	0.184792066	FALSE	C16orf87
Region_6809	chr16	46884362	46931289	46927	26	182	215.3030303	15.60174517	0.871691803	0.031322032	+	84706	grey	0.09104299	FALSE	GPT2
Region_6810	chr16	46955362	46973674	18312	6	41	55.25757576	6.988585432	0.680960307	0.033377755	-	10294	grey	-0.048702246	FALSE	DNAJA2
Region_6811	chr16	47077703	47143945	66242	15	98	124.7121212	10.09152752	0.836911687	0.016367028	-	81831	grey	-0.09314273	FALSE	NETO2
Region_6812	chr1	47154387	47464149	309762	46	351	396.181818	22.0250969	0.94222891	0.00924549	-	81533	grey	-0.02148549	FALSE	ITFG1

	6						2	4	6	2				3		
Region_6813	chr16	47461123	47701523	240400	13	91	108.1363636	8.594965187	0.888284727	0.016387546	+	5257	grey	-0.017767737	FALSE	PHKB
Region_6814	chr16	47724568	47908431	183863	17	118	148.1515152	13.11164183	0.888475645	0.029978007	+	101927132	grey	0.205714175	FALSE	LINC02133
Region_6815	chr16	47849307	47887130	37823	4	26	40.21212121	5.709681795	0.872722572	0.081190071	-	100507534	grey	0.249490056	FALSE	LINC02192
Region_6816	chr16	48080882	48156018	75136	22	161	187.530303	15.21357663	0.870211677	0.024599573	-	94160	grey	-0.047886481	FALSE	ABCC12
Region_6817	chr16	48164952	48247568	82616	20	139	170.3030303	14.07449378	0.884267055	0.026134341	-	85320	grey	0.123727084	FALSE	ABCC11
Region_6818	chr16	48244300	48363122	118822	22	151	176.1060606	12.20734809	0.872504528	0.014229449	+	83752	grey	-0.107121044	FALSE	LONP2
Region_6819	chr16	48356364	48448402	92038	36	259	314.3181818	18.1253406	0.853113152	0.018354783	-	6477	grey	-0.356654794	FALSE	SIAH1
Region_6820	chr16	48538726	48620148	81422	11	69	89.87878788	9.852005109	0.924057764	0.035849624	-	9683	grey	0.150707225	FALSE	N4BP1
Region_6821	chr16	48623415	48746318	122903	17	102	133.6363636	10.718261	0.807284083	0.044612749	+	105371240	grey	0.033498891	FALSE	LOC105371240
Region_6822	chr16	49072543	49275579	203036	17	126	156.530303	12.40622635	0.709294537	0.046105533	-	105371241	grey	-0.085048074	FALSE	LOC105371241
Region_6823	chr16	49277917	49281838	3921	4	14	23.53030303	4.791722661	0.068489831	0.070327535	-	869	grey	-0.120222573	FALSE	CBLN1
Region_6824	chr16	49282021	49350504	68483	16	114	135.469697	12.33908075	0.654622327	0.028885863	+	102724859	grey	-0.074348934	FALSE	LOC102724859

Region_6825	chr16	49373804	49399431	25627	4	19	28.33333333	4.333530568	0.894366692	0.06334106	+	123970	grey	-0.054558854	FALSE	C16orf78
Region_6826	chr16	49487524	49857919	370395	169	1258	1389.772727	53.09057441	0.854631072	0.013489978	-	23090	grey	-0.127125113	FALSE	ZNF423
Region_6827	chr16	50024410	50037088	12678	4	15	28.06060606	5.689068567	0.973053546	0.040542284	+	255919	grey	0.356883947	FALSE	CNEP1R1
Region_6828	chr16	50065967	50107272	41305	12	71	91.74242424	10.07099507	0.630606309	0.029039867	+	55027	grey	0.180246602	FALSE	HEATR3
Region_6829	chr16	50152911	50235310	82399	30	200	239.7424242	15.93091876	0.781911518	0.01962798	+	64282	grey	0.176122459	FALSE	TENT4B
Region_6830	chr16	50246137	50318135	71998	33	215	258.1969697	17.81807181	0.778991708	0.020197026	+	113	grey	-0.062097144	FALSE	ADCY7
Region_6831	chr16	50313487	50368988	55501	13	79	105.4090909	10.41445338	0.874444859	0.025900042	-	29117	grey	0.00725297	FALSE	BRD7
Region_6832	chr16	50548396	50649249	100853	42	259	314.030303	17.84115355	0.897664783	0.021051866	+	85407	grey	0.087809051	FALSE	NKD1
Region_6833	chr16	50664903	50671639	6736	5	21	37.57575758	5.686855576	0.496976209	0.068243794	+	101927272	grey	0.107965265	FALSE	LOC101927272
Region_6834	chr16	50666300	50681353	15053	9	52	69.54545455	8.505530903	0.717337936	0.044146404	-	124460	grey	-0.186915731	FALSE	SNX20
Region_6835	chr16	50693588	50733077	39489	19	120	146.4242424	14.0000666	0.772217443	0.025404182	+	64127	grey	0.094400842	FALSE	NOD2
Region_6836	chr16	50727417	50742815	15398	7	35	50.59090909	7.036120594	0.681456422	0.035272974	-	102724907	grey	0.326184709	FALSE	CYLD-AS1
Region_6837	chr1	50742050	50801935	59885	14	78	103.272727	10.2740082	0.87517576	0.03000017	+	1540	grey	-0.09816804	FALSE	CYLD

	6						3	1	5	9				3		
Region_6838	chr1 6	50783859	50803338	19479	7	34	51.4242424 2	7.71224921	0.9603151	0.03578407 3	-	10537125 1	grey	-0.14109263 9	FALSE	CYLD-AS2
Region_6839	chr1 6	50840017	50901063	61046	14	100	122.893939 4	13.5682080 8	0.88682497 4	0.02741385 9	+	10272494 0	grey	0.030256408	FALSE	LINC02128
Region_6840	chr1 6	51017544	51035784	18240	7	48	64.8787878 8	8.75744105 8	0.78699549 5	0.03675435 7	+	10192733 4	grey	-0.10307116 6	FALSE	LINC02127
Region_6841	chr1 6	51135982	51152334	16352	13	83	111.666666 7	11.8870755 1	0.46131906 3	0.02689819 4	-	6299	grey	0.110244989	FALSE	SALL1
Region_6842	chr1 6	51150770	51356666	205896	42	321	371.984848 5	19.8436135 2	0.64816086 6	0.02316875	+	10798488 6	grey	-0.25083285 7	FALSE	LOC107984886
Region_6843	chr1 6	51246855	51442519	195664	20	147	180.954545 5	11.8677738 4	0.84978207 8	0.02888262 5	+	10537125 6	grey	-0.08791048 7	FALSE	LOC105371256
Region_6844	chr1 6	51355483	51525173	169690	18	112	143.787878 8	11.4576345 4	0.82848775 3	0.03476088 4	-	10272332 3	grey	-0.14137381 6	FALSE	LOC102723323
Region_6845	chr1 6	52005487	52078474	72987	5	28	40.9090909 1	6.04273823	0.50176373 1	0.07901131 6	-	388276	grey	0.272597777	FALSE	LINC02911
Region_6846	chr1 6	52238081	52269574	31493	3	16	24.8030303	4.59504648 7	0.80484260 4	0.08683093 3	+	10537126 1	grey	0.095134804	FALSE	LOC105371261
Region_6847	chr1 6	52258564	52280299	21735	5	36	49.1363636 4	6.24102152 3	0.80794935 6	0.06710745 7	+	283854	grey	0.185283307	FALSE	CASC22
Region_6848	chr1 6	52271566	52280638	9072	3	21	32.3030303	4.98912570 4	0.83582129	0.06630703 5	+	10798396 1	grey	0.240740041	FALSE	LOC107983961
Region_6849	chr1 6	52296987	52383740	86753	6	38	58.5151515 2	7.44465202 8	0.58912159	0.05315778 4	-	10798490 1	grey	0.128190022	FALSE	LOC107984901

Region_6850	chr16	52436417	52547802	111385	11	85	107.3787879	8.867859253	0.65549791	0.04721197	-	27324	grey	-0.023584627	FALSE	TOX3
Region_6851	chr16	52552084	52652132	100048	7	42	56.10606061	7.567397252	0.81079319	0.073684546	-	643714	grey	0.052062418	FALSE	CASC16
Region_6852	chr16	53035690	53052897	17207	3	17	30.04545455	4.865678285	0.892235109	0.060132352	-	105371267	grey	0.143534651	FALSE	CHD9NB
Region_6853	chr16	53054991	53329150	274159	46	349	383.1363636	19.22409255	0.795519697	0.013978955	+	80205	grey	0.324605321	FALSE	CHD9
Region_6854	chr16	53373479	53385142	11663	5	21	34.72727273	5.51235104	0.907476589	0.067767481	+	102723373	grey	0.001451705	FALSE	LOC102723373
Region_6855	chr16	53433977	53491648	57671	14	101	129.9090909	10.89350868	0.80437348	0.022885766	+	5934	grey	0.107817627	FALSE	RBL2
Region_6856	chr16	53491040	53504411	13371	4	15	31.51515152	6.114905935	0.611359172	0.055301947	-	64400	grey	0.116576917	FALSE	AKTIP
Region_6857	chr16	53598153	53703938	105785	14	94	119.1212121	10.24847479	0.961025516	0.020872037	-	23322	grey	0.036309823	FALSE	RPGRIP1L
Region_6858	chr16	53701692	54153448	451756	66	468	541.2272727	28.353155	0.926565943	0.013893355	+	79068	grey	0.175064646	FALSE	FTO
Region_6859	chr16	53826361	54040726	214365	28	191	240.4848485	17.72633193	0.914270344	0.020271037	+	100505692	grey	0.082621165	FALSE	FTO-IT1
Region_6860	chr16	54245544	54270879	25335	6	40	54.56060606	6.353990358	0.935259411	0.031498178	-	100996338	grey	0.077951076	FALSE	LINC02169
Region_6861	chr16	54283304	54286787	3483	6	16	36.59090909	7.187547507	0.017519114	0.030071077	-	79191	grey	0.169053178	FALSE	IRX3
Region_6862	chr1	54542808	54559100	16292	5	32	50.4242424	8.09949781	0.86941265	0.05204372	-	10537127	grey	-0.15175784	FALSE	LINC02183

	6						2	2	8	1		3		8		
Region_6863	chr16	54845189	54874168	28979	10	60	88.33333333	9.91398908	0.888596454	0.036353471	-	101927480	grey	0.008576156	FALSE	LOC101927480
Region_6864	chr16	54847316	54854991	7675	4	23	35.22727273	5.80661547	0.804859848	0.079395373	+	105371276	grey	0.151612818	FALSE	LOC105371276
Region_6865	chr16	54851640	54928843	77203	25	176	212.0151515	15.97256535	0.835092132	0.031412255	-	105371275	grey	-0.209072654	FALSE	LOC105371275
Region_6866	chr16	54916492	54929189	12697	5	31	45.65151515	6.808348776	0.590828885	0.07412375	-	643911	grey	-0.184456297	FALSE	CRNDE
Region_6867	chr16	54930865	54934485	3620	6	26	44.46969697	6.755555428	0.051225814	0.038611449	+	10265	grey	0.172857871	FALSE	IRX5
Region_6868	chr16	55389700	55506691	116991	22	152	175.2727273	12.84169346	0.701067688	0.028707702	+	4313	grey	0.02023434	FALSE	MMP2
Region_6869	chr16	55426797	55462297	35500	4	20	28.63636364	4.662902245	0.896893311	0.059815081	-	107984884	grey	0.277932311	FALSE	MMP2-AS1
Region_6870	chr16	55509072	55586666	77594	4	26	39.40909091	6.565591596	0.698627443	0.061403906	+	54947	grey	-0.243264071	FALSE	LPCAT2
Region_6871	chr16	55655988	55707645	51657	13	104	126.469697	12.49642373	0.801977802	0.039912116	+	6530	grey	-0.205407186	FALSE	SLC6A2
Region_6872	chr16	55728115	55750111	21996	5	44	59.87878788	7.39961569	0.842870179	0.048400751	+	390732	grey	0.068256552	FALSE	CES1P2
Region_6873	chr16	55991744	56076647	84903	10	62	86.5	9.395989324	0.860442088	0.052975121	-	105371281	grey	-0.050828179	FALSE	LOC105371281
Region_6874	chr16	56092987	56191118	98131	11	57	86.81818182	10.2129768	0.776277681	0.03718921	-	283856	grey	0.231426785	FALSE	GNAO1-DT

Region_6875	chr16	56191390	56357444	166054	43	307	360.6818182	21.69874799	0.779969008	0.019997716	+	2775	grey	0.166977559	FALSE	GNAO1
Region_6876	chr16	56361452	56425545	64093	24	132	189.7424242	15.57444713	0.734045975	0.024887895	-	267	grey	-0.111909682	FALSE	AMFR
Region_6877	chr16	56429133	56501497	72364	20	128	150.8787879	11.83412946	0.736786763	0.019557918	-	11051	grey	0.002854602	FALSE	NUDT21
Region_6878	chr16	56451521	56479104	27583	8	39	55.18181818	8.223622804	0.949334488	0.031257796	+	55239	grey	0.049702451	FALSE	OGFOD1
Region_6879	chr16	56465640	56582667	117027	22	133	162.7878788	12.23301353	0.889493762	0.024360579	-	583	grey	-0.232706068	FALSE	BBS2
Region_6880	chr16	56676132	56677763	1631	3	14	24.53030303	5.038985542	0.89705959	0.068451335	+	644314	grey	-0.122224169	FALSE	MT1IP
Region_6881	chr16	56708450	56730010	21560	6	42	54.43939394	6.732535899	0.909653873	0.045447356	-	105371286	grey	0.031346213	FALSE	NUP93-DT
Region_6882	chr16	56730118	56850286	120168	25	177	213.469697	16.35680964	0.854967642	0.031766	+	9688	grey	-0.117979893	FALSE	NUP93
Region_6883	chr16	56865207	56915850	50643	27	151	186.5151515	12.22512221	0.852224693	0.029545313	+	6559	grey	-0.062413575	FALSE	SLC12A3
Region_6884	chr16	56932142	56944864	12722	5	36	49.24242424	6.937214263	0.563714981	0.031980796	+	9709	grey	0.138569231	FALSE	HERPUD1
Region_6885	chr16	56961923	56983845	21922	14	81	112.1818182	13.37265763	0.757014698	0.02805299	+	1071	grey	-0.165765621	FALSE	CETP
Region_6886	chr16	56989485	57083531	94046	32	210	249.7424242	17.1547801	0.695523865	0.033506939	+	84166	grey	0.171074886	FALSE	NLRC5
Region_6887	chr1	57092583	57148369	55786	30	207	234.151515	15.1751238	0.78045561	0.02288794	+	221184	grey	-0.13504757	FALSE	CPNE2

	6						2		5	2				5		
Region_6888	chr16	57152466	57186116	33650	16	105	124.5606061	9.81541768	0.893057455	0.028196384	-	80011	grey	-0.006446238	FALSE	PSME3IP1
Region_6889	chr16	57186137	57240469	54332	11	68	83.83333333	8.563937508	0.977240342	0.019079172	+	89970	grey	-0.18968693	FALSE	RSPRY1
Region_6890	chr16	57249039	57284672	35633	18	115	141.6515152	13.45933182	0.732790773	0.025805474	-	51090	grey	0.224790227	FALSE	PLLP
Region_6891	chr16	57372477	57385044	12567	6	35	53.10606061	7.815426142	0.926985897	0.046074389	+	6376	grey	0.00216705	FALSE	CX3CL1
Region_6892	chr16	57404767	57416063	11296	5	20	32.6969697	6.404252666	0.942788583	0.04422434	+	6361	grey	0.087531182	FALSE	CCL17
Region_6893	chr16	57428187	57447420	19233	7	44	59.48484848	6.957546925	0.964555891	0.034360769	-	57019	grey	-0.230307801	FALSE	CIAPIN1
Region_6894	chr16	57447425	57461270	13845	5	25	38.40909091	6.406672658	0.559662192	0.036148804	+	57017	grey	-0.023378267	FALSE	COQ9
Region_6895	chr16	57471922	57487327	15405	13	73	98.6969697	11.96143765	0.738786688	0.035772262	-	55715	grey	0.312804734	FALSE	DOK4
Region_6896	chr16	57512181	57536571	24390	16	92	118.0606061	10.19483849	0.78313195	0.038212014	-	92922	grey	-0.060982582	FALSE	CCDC102A
Region_6897	chr16	57542643	57591681	49038	22	138	168.6666667	14.38517756	0.755785844	0.02666692	+	221188	grey	0.050101218	FALSE	ADGRG5
Region_6898	chr16	57610652	57665580	54928	28	203	233.8787879	17.61512912	0.809940673	0.028180529	+	9289	grey	-0.063169005	FALSE	ADGRG1
Region_6899	chr16	57668277	57689378	21101	11	71	95.75757576	9.543520728	0.626405274	0.047011336	+	222487	grey	-0.122765563	FALSE	ADGRG3

Region_6900	chr16	57694793	57731805	37012	10	65	80	7.619812233	0.940197358	0.028335619	+	84229	grey	-0.166421383	FALSE	DRC7
Region_6901	chr16	57735739	57757244	21505	7	33	50.98484848	6.632072247	0.808337498	0.045840868	+	10300	grey	-0.20252878	FALSE	KATNB1
Region_6902	chr16	57758217	57863053	104836	60	374	438.8181818	25.22569453	0.830506657	0.015750988	-	3801	grey	0.104785232	FALSE	KIFC3
Region_6903	chr16	57798186	57816946	18760	11	56	76.04545455	8.744988575	0.595930237	0.03038286	+	388282	grey	-0.030925051	FALSE	LOC388282
Region_6904	chr16	57882340	57971128	88788	39	301	341.0454545	18.55671707	0.890768794	0.018696917	-	1258	grey	0.063633843	FALSE	CNGB1
Region_6905	chr16	57976435	57988116	11681	6	29	42.54545455	6.363796202	0.446110681	0.050064886	+	374739	grey	0.006088397	FALSE	SPMIP8
Region_6906	chr16	57994676	58000672	5996	6	19	37.18181818	7.295750272	0.758941708	0.066729239	-	57567	grey	-0.157411833	FALSE	ZNF319
Region_6907	chr16	57999546	58021618	22072	10	59	78.33333333	7.640647039	0.773217741	0.034346046	+	79650	grey	-0.027418493	FALSE	USB1
Region_6908	chr16	58025754	58046901	21147	29	168	216.3787879	16.57316195	0.658674071	0.018745968	+	4324	grey	0.418661241	FALSE	MMP15
Region_6909	chr16	58113592	58129381	15789	10	74	96.90909091	9.789676309	0.539448922	0.038322311	-	29105	grey	0.017671151	FALSE	CFAP20
Region_6910	chr16	58129519	58163246	33727	12	68	87.43939394	8.783076013	0.931770225	0.037524822	+	101927556	grey	-0.212910406	FALSE	LOC101927556
Region_6911	chr16	58157907	58198106	40199	8	40	61.33333333	7.905856317	0.975659614	0.023721772	-	1459	grey	-0.028161628	FALSE	CSNK2A2
Region_6912	chr1	58197112	58283836	86724	32	213	254.636363	16.4378890	0.73055254	0.02616366	+	29070	grey	-0.10924336	FALSE	CCDC113

	6						6	3		9				6		
Region_6913	chr16	58279997	58295047	15050	8	49	70.56060606	9.306951559	0.910794015	0.048216505	-	221191	grey	0.247276118	FALSE	PRSS54
Region_6914	chr16	58392394	58406147	13753	4	31	44.07575758	6.852984989	0.919251409	0.04280829	+	64785	grey	0.213961464	FALSE	GINS3
Region_6915	chr16	58421326	58462470	41144	21	144	178.2272727	14.62431637	0.836564962	0.043211816	+	105371295	grey	-0.228111923	FALSE	LINC02137
Region_6916	chr16	58462846	58513628	50782	28	190	226.7121212	15.84517898	0.566251152	0.027139524	+	65009	grey	-0.271091347	FALSE	NDRG4
Region_6917	chr16	58515479	58523842	8363	11	55	77.59090909	10.71444356	0.598038256	0.038234534	+	79918	grey	-0.239756209	FALSE	SETD6
Region_6918	chr16	58519951	58629885	109934	39	258	321	20.48113579	0.937711721	0.016072619	-	23019	grey	-0.032747015	FALSE	CNOT1
Region_6919	chr16	58665109	58684770	19661	6	37	53.07575758	6.629330181	0.954590951	0.041409731	-	55238	grey	0.257340378	FALSE	SLC38A7
Region_6920	chr16	58707131	58734342	27211	13	77	113.0909091	11.05472718	0.826275162	0.013960777	-	2806	grey	-0.362214903	FALSE	GOT2
Region_6921	chr16	58733910	59199395	465485	62	435	487.8636364	28.42203174	0.895190009	0.016253072	+	107984867	grey	-0.368549898	FALSE	LOC107984867
Region_6922	chr16	59855353	60053973	198620	13	69	92.68181818	9.155341595	0.814836839	0.046530436	+	101927580	grey	0.015417836	FALSE	LINC02141
Region_6923	chr16	60346478	60499364	152886	20	139	164.8030303	13.79768063	0.844254928	0.036080269	+	101927605	grey	0.223034806	FALSE	LOC101927605
Region_6924	chr16	61647242	62037035	389793	36	268	310.5757576	18.02396747	0.75488451	0.019781474	-	1006	grey	0.245275109	FALSE	CDH8

Region_6925	chr16	63056786	63129654	72868	3	11	19.1969697	4.214333406	0.622137683	0.094715478	-	105371307	grey	0.121673282	FALSE	LOC105371307
Region_6926	chr16	63314264	63618046	303782	16	105	128.1060606	10.09817773	0.836730917	0.042115991	-	105371308	grey	0.19646338	FALSE	LOC105371308
Region_6927	chr16	64364781	64600247	235466	15	104	132.5	10.06020339	0.797972204	0.043887831	+	105371310	grey	-0.058260016	FALSE	LOC105371310
Region_6928	chr16	64943753	65126112	182359	23	170	202.3787879	14.33743368	0.792400064	0.028031269	-	1009	grey	-0.255764466	FALSE	CDH11
Region_6929	chr16	65141751	65176713	34962	5	30	40.13636364	5.39768145	0.897743698	0.047150147	-	101927650	grey	-0.06370172	FALSE	LINC02126
Region_6930	chr16	65284496	65576345	291849	28	211	252.1060606	14.80034177	0.863745287	0.030599686	-	283867	grey	-0.009863835	FALSE	LINC00922
Region_6931	chr16	66366622	66404784	38162	8	56	70.10606061	8.664922722	0.90751378	0.041271433	+	1003	grey	0.061016583	FALSE	CDH5
Region_6932	chr16	66427295	66493529	66234	20	134	152.5151515	9.977270205	0.893775881	0.02259007	+	146227	grey	-0.006649289	FALSE	BEAN1
Region_6933	chr16	66469796	66517312	47516	17	115	140.4090909	13.22349572	0.902903406	0.022001756	-	101927726	grey	0.132867657	FALSE	BEAN1-AS1
Region_6934	chr16	66481175	66552544	71369	28	196	227.1060606	15.27806753	0.8147832	0.021991457	-	7084	grey	0.098938537	FALSE	TK2
Region_6935	chr16	66552563	66570086	17523	3	10	21.56060606	6.397424453	0.359494369	0.050347406	+	51192	grey	-0.059001638	FALSE	CKLF
Region_6936	chr16	66552613	66579135	26522	4	17	30.07575758	6.986383693	0.514621609	0.039287436	+	100529251	grey	-0.081789058	FALSE	CKLF-CMTM1
Region_6937	chr1	66579448	66588275	8827	6	28	38.7575757	5.82054876	0.25686488	0.06416373	+	146225	grey	0.033800756	FALSE	CMTM2

	6						6	1	2	4						
Region_6938	chr16	66603874	66613892	10018	4	18	28.27272727	5.652897023	0.958626716	0.039022298	+	123920	grey	0.047212183	FALSE	CMTM3
Region_6939	chr16	66614750	66696743	81993	29	194	229.6060606	17.65993186	0.825698432	0.017189258	-	146223	grey	0.078770198	FALSE	CMTM4
Region_6940	chr16	66720893	66751609	30716	16	106	133.6666667	11.06808764	0.836426405	0.027235939	-	1783	grey	-0.247676682	FALSE	DYNC1LI2
Region_6941	chr16	66754640	66801620	46980	14	81	104.8181818	9.703146343	0.964199267	0.019196865	-	283847	grey	-0.106089088	FALSE	TERB1
Region_6942	chr16	66802875	66873256	70381	25	155	181.6515152	13.74771861	0.805810517	0.025410096	-	8883	grey	0.24704346	FALSE	NAE1
Region_6943	chr16	66878589	66895754	17165	9	48	72.48484848	10.42075211	0.863982757	0.027980481	+	57546	grey	-0.191917572	FALSE	PDP2
Region_6944	chr16	66899984	66905266	5282	6	30	40.78787879	6.350082135	0.911061638	0.051973972	-	107984821	grey	-0.102996156	FALSE	LOC107984821
Region_6945	chr16	66908122	66918917	10795	4	18	28.78787879	5.970479047	0.932967785	0.051988217	-	1014	grey	0.00537254	FALSE	CDH16
Region_6946	chr16	66921679	66925536	3857	3	16	27.51515152	5.421445803	0.570359324	0.113494498	-	6236	grey	-0.22704253	FALSE	RRAD
Region_6947	chr16	66932065	66934423	2358	4	19	32.87878788	6.333100229	0.420843508	0.052261955	-	51647	grey	-0.239753466	FALSE	CIAO2B
Region_6948	chr16	66934444	66963256	28812	26	193	218.6515152	15.30057055	0.50867401	0.026446134	+	8824	grey	-0.020770058	FALSE	CES2
Region_6949	chr16	66961245	66975149	13904	6	41	53.98484848	6.898143394	0.792758252	0.058233019	+	23491	grey	0.027596363	FALSE	CES3

Region_6950	chr16	66988589	67010417	21828	9	43	61.84848485	8.793778263	0.959915603	0.025867999	+	283848	grey	-0.26753205	FALSE	CES4A
Region_6951	chr16	67028984	67101058	72074	38	274	314.7121212	17.12916106	0.747072542	0.014896585	+	865	grey	0.080534334	FALSE	CBFB
Region_6952	chr16	67109941	67148544	38603	17	94	127.8939394	14.04950455	0.740098123	0.030005092	+	80262	grey	-0.005970916	FALSE	PHAF1
Region_6953	chr16	67148104	67150998	2894	5	23	39.06060606	7.558752009	0.469587188	0.094601137	-	84752	grey	-0.063853841	FALSE	B3GNT9
Region_6954	chr16	67154185	67159909	5724	10	42	60.33333333	9.167389249	0.147513254	0.036807818	-	8717	grey	0.154295122	FALSE	TRADD
Region_6955	chr16	67159932	67164570	4638	8	39	54.31818182	7.742309516	0.100588236	0.031959738	+	55336	grey	0.006457584	FALSE	FBXL8
Region_6956	chr16	67163385	67169945	6560	7	30	42.75757576	6.899410468	0.053856368	0.031116076	+	3299	grey	-0.068098046	FALSE	HSF4
Region_6957	chr16	67175599	67184040	8441	14	55	94.93939394	13.74031551	0.143365432	0.025625834	-	653319	grey	-0.149239121	FALSE	MATCAP1
Region_6958	chr16	67184379	67190185	5806	25	173	205.2121212	18.8161266	0.442317783	0.02115261	-	283849	grey	0.109604453	FALSE	EXOC3L1
Region_6959	chr16	67192155	67198918	6763	7	33	48.36363636	7.165379079	0.631336191	0.048395247	+	1874	grey	0.286080449	FALSE	E2F4
Region_6960	chr16	67199111	67204029	4918	16	55	88.60606061	12.03057488	0.824857919	0.090710924	+	79767	grey	0.162432833	FALSE	ELMO3
Region_6961	chr16	67207139	67227048	19909	23	132	173.3030303	13.57142892	0.878361833	0.015615959	-	26231	grey	0.151446792	FALSE	FBXL9P
Region_6962	chr1	67229387	67247481	18094	15	78	98.8939393	9.66536845	0.85413651	0.03117539	-	29109	grey	-0.31696765	FALSE	FHOD1

	6						9	2	8	2				1		
Region_6963	chr1 6	67237683	67272191	34508	17	98	118.727272 7	11.4791388 8	0.64003351 7	0.02890410 1	+	6553	grey	-0.0992914	FALSE	SLC9A5
Region_6964	chr1 6	67277510	67289499	11989	14	68	95.8484848 5	11.9489200 3	0.76856934	0.02819789 4	+	25894	grey	0.058346948	FALSE	PLEKHG4
Region_6965	chr1 6	67289428	67326760	37332	9	56	73.8787878 8	8.37306147 8	0.88494787 6	0.03535782 3	-	146212	grey	0.009659052	FALSE	KCTD19
Region_6966	chr1 6	67326798	67385204	58406	21	137	172.893939 4	14.6886966 4	0.92230704 1	0.02609524 7	+	55282	grey	0.004201871	FALSE	LRRC36
Region_6967	chr1 6	67394152	67416833	22681	10	51	72.6363636 4	9.45537919 9	0.36215243	0.02406053 1	-	29800	grey	-0.04702388 1	FALSE	ZDHHC1
Region_6968	chr1 6	67430652	67437553	6901	10	49	68.6515151 5	8.63533425 3	0.40457861 8	0.02352685 1	+	3291	grey	0.278113654	FALSE	HSD11B2
Region_6969	chr1 6	67438014	67481181	43167	20	129	153.136363 6	13.8385041	0.7842358	0.03055482 5	-	9114	grey	0.216309737	FALSE	ATP6V0D1
Region_6970	chr1 6	67481314	67505977	24663	7	30	46.9545454 5	7.31370014 4	0.65065093	0.04940030 4	+	10192783 7	grey	0.117465385	FALSE	ATP6V0D1-DT
Region_6971	chr1 6	67517862	67528768	10906	8	38	55.3333333 3	7.84922011 5	0.72863792 6	0.05645816 5	-	10050594 2	grey	0.095412622	FALSE	LOC100505942
Region_6972	chr1 6	67518418	67546788	28370	14	67	92.5151515 2	11.2417387 4	0.68277406 5	0.0406413	+	79567	grey	0.047144218	FALSE	RIPOR1
Region_6973	chr1 6	67561411	67562309	898	4	12	25.7878787 9	5.42221967 5	0.09361325 8	0.05807975 9	-	10798481 3	grey	-0.22649449 1	FALSE	CTCF-DT
Region_6974	chr1 6	67562467	67639177	76710	62	341	410.227272 7	34.2101179 2	0.45624528 5	0.01300696 1	+	10664	grey	0.02187699	FALSE	CTCF

Region_6975	chr16	67644988	67657569	12581	9	23	39.07575758	7.080440243	0.94929013	0.047071902	+	146206	grey	-0.02956972	FALSE	CARMIL2
Region_6976	chr16	67660946	67662774	1828	8	36	53.71212121	7.814710293	0.492530232	0.029321827	+	50855	grey	-0.127124448	FALSE	PARD6A
Region_6977	chr16	67662945	67667265	4320	3	17	26.07575758	4.743613054	0.670373944	0.031460824	-	84080	grey	-0.040373066	FALSE	ENKD1
Region_6978	chr16	67666814	67668757	1943	3	16	27.10606061	4.60143385	0.394729036	0.076034141	+	388284	grey	-0.005728532	FALSE	C16orf86
Region_6979	chr16	67674531	67719339	44808	40	279	313.8939394	18.60531668	0.958845921	0.014278086	-	81577	grey	0.382956302	FALSE	GFOD2
Region_6980	chr16	67723070	67806560	83490	34	238	278.3939394	17.05906372	0.935318489	0.015881697	-	57610	grey	-0.164992363	FALSE	RANBP10
Region_6981	chr16	67806765	67832148	25383	13	68	95.22727273	11.14215206	0.725712373	0.025071721	+	55815	grey	0.040429742	FALSE	TSNAXIP1
Region_6982	chr16	67828157	67847811	19654	17	91	121.5	12.87125599	0.347108843	0.013628016	-	80152	grey	-0.0155324	FALSE	CENPT
Region_6983	chr16	67842320	67844195	1875	5	22	37.95454545	7.816022632	0.006819124	0.014165791	+	57215	grey	-0.066881218	FALSE	THAP11
Region_6984	chr16	67846882	67872567	25685	18	109	143.1515152	12.40873442	0.798606733	0.02055241	+	10204	grey	0.109186529	FALSE	NUTF2
Region_6985	chr16	67873052	67884499	11447	17	102	129.3181818	11.58667549	0.708251427	0.023385064	+	23644	grey	-0.211885277	FALSE	EDC4
Region_6986	chr16	67893254	67929676	36422	27	212	243.1212121	18.89038433	0.819631374	0.016053853	+	5681	grey	0.198825619	FALSE	PSKH1
Region_6987	chr1	67929574	67936017	6443	6	33	45.3636363	7.45991619	0.70712984	0.05906950	-	1506	grey	-0.35833379	FALSE	CTRL

	6						6	6	1	6				2		
Region_6988	chr16	67930925	67936864	5939	8	42	57.87878788	7.70517339	0.291784171	0.035077271	-	5699	grey	-0.278436576	FALSE	PSMB10
Region_6989	chr16	67939750	67944131	4381	6	39	59.16666667	8.803117164	0.563523945	0.042353114	-	3931	grey	-0.158423627	FALSE	LCAT
Region_6990	chr16	67943474	67969601	26127	19	107	133.7424242	10.86888375	0.568760874	0.02886808	-	6560	grey	0.25115365	FALSE	SLC12A4
Region_6991	chr16	67975663	67980549	4886	5	12	26.5	5.759941239	0.89868866	0.072078123	-	64180	grey	0.195696958	FALSE	DPEP3
Region_6992	chr16	67987394	68000586	13192	11	54	75.15151515	8.702329351	0.695562893	0.024413977	-	64174	grey	-0.110291653	FALSE	DPEP2
Region_6993	chr16	67987746	68079320	91574	66	454	528.1969697	24.57028831	0.80380238	0.009423941	+	54920	grey	0.075992933	FALSE	DUS2
Region_6994	chr16	68013436	68015911	2475	4	15	29.12121212	6.40556285	0.964299845	0.038389884	-	100131303	grey	-0.124084839	FALSE	DPEP2NB
Region_6995	chr16	68020916	68023232	2316	6	42	56.74242424	7.43445328	0.162330101	0.01362906	-	55794	grey	-0.150393765	FALSE	DDX28
Region_6996	chr16	68077340	68229259	151919	30	215	260.2878788	17.43891771	0.797439099	0.013301074	+	4775	grey	0.267552868	FALSE	NFATC3
Region_6997	chr16	68229033	68238102	9069	9	49	67.75757576	8.98727409	0.136757259	0.024899516	-	80004	grey	0.00140203	FALSE	ESRP2
Region_6998	chr16	68245304	68261058	15754	5	28	44.3030303	7.485308065	0.09849605	0.051957433	+	23659	grey	-0.067672673	FALSE	PLA2G15
Region_6999	chr16	68264516	68301823	37307	20	153	178.0757576	14.14464901	0.623473174	0.022219956	+	9057	grey	0.063664168	FALSE	SLC7A6

Region_7000	chr16	68284503	68310946	26443	7	46	62.60606061	7.707895628	0.520701677	0.024554312	-	84138	grey	0.044767294	FALSE	SLC7A6OS
Region_7001	chr16	68310951	68360852	49901	29	211	244.2575758	16.04446764	0.753633473	0.019140583	+	54496	grey	0.265253436	FALSE	PRMT7
Region_7002	chr16	68358327	68448508	90181	44	313	358.8484848	21.71581659	0.819736485	0.021470284	-	55512	grey	0.126683874	FALSE	SMPD3
Region_7003	chr16	68530090	68576072	45982	15	85	105.5151515	11.55886929	0.913868603	0.016061239	+	146198	grey	0.171585923	FALSE	ZFP90
Region_7004	chr16	68644993	68727468	82475	48	296	354.2727273	20.75167868	0.862656002	0.013567384	+	1001	grey	-0.186751002	FALSE	CDH3
Region_7005	chr16	68737292	68835537	98245	45	291	371.030303	25.4159064	0.818757591	0.018627707	+	999	grey	0.03352607	FALSE	CDH1
Region_7006	chr16	68843531	69085182	241651	65	436	494.3181818	22.81777923	0.93537757	0.014887417	+	79613	grey	-0.058484056	FALSE	TANGO6
Region_7007	chr16	69105653	69118719	13066	16	89	113.6060606	10.58282904	0.509682281	0.034391663	+	3038	grey	0.019388441	FALSE	HAS3
Region_7008	chr16	69118010	69132588	14578	9	48	66.18181818	7.767603513	0.745341974	0.024085622	-	113455421	grey	-0.101474993	FALSE	DERPC
Region_7009	chr16	69118010	69132588	14578	9	48	66.18181818	7.767603513	0.745341974	0.024085622	-	54921	grey	-0.101474993	FALSE	CHTF8
Region_7010	chr16	69187147	69309052	121905	49	336	399.4848485	21.45065351	0.924425311	0.011552375	+	6645	grey	-0.088992751	FALSE	SNTB2
Region_7011	chr16	69299682	69326939	27257	9	60	78.78787879	8.518784947	0.969230409	0.021514478	+	27183	grey	0.190182703	FALSE	VPS4A
Region_7012	chr1	69321991	69356306	34315	25	164	197.893939	15.0549034	0.69896414	0.02080751	-	84342	grey	-0.14328225	FALSE	COG8

	6						4	1	7	7				5		
Region_7013	chr16	69326913	69330588	3675	4	10	22.21212121	5.781975046	0.004878917	0.019414875	-	64146	grey	-0.123932112	FALSE	PDF
Region_7014	chr16	69335276	69351786	16510	13	90	112.3484848	9.340552486	0.80760969	0.033700453	-	146456	grey	-0.294999754	FALSE	TMED6
Region_7015	chr16	69355567	69408571	53004	27	190	224.7424242	17.2236962	0.787963401	0.01961714	-	7014	grey	-0.302797871	FALSE	TERF2
Region_7016	chr16	69398402	69466264	67862	18	126	150.7575758	12.49499667	0.854809245	0.017738619	+	80777	grey	-0.257818163	FALSE	CYB5B
Region_7017	chr16	69565094	69704666	139572	33	198	243.0151515	16.02161273	0.901004579	0.024085605	+	10725	grey	0.078006957	FALSE	NFAT5
Region_7018	chr16	69706996	69726668	19672	8	40	53.42424242	6.002718887	0.939936469	0.029783207	-	1728	grey	-0.180913353	FALSE	NQO1
Region_7019	chr16	69741871	69754926	13055	9	57	82.83333333	9.219683514	0.969424445	0.01676137	-	28987	grey	-0.311100708	FALSE	NOB1
Region_7020	chr16	69762328	69941741	179413	88	636	730.9393939	34.18406232	0.908964619	0.00944685	+	11060	grey	0.149069323	FALSE	WWP2
Region_7021	chr16	69976297	70065948	89651	35	213	263.3181818	20.06003228	0.940115549	0.015564478	-	283970	grey	-0.062945381	FALSE	PDXDC2P-NPIPB14P
Region_7022	chr16	70114332	70162537	48205	30	161	284.6666667	72.14650337	0.879065985	0.018858513	+	55066	grey	0.056569354	FALSE	PDPR
Region_7023	chr16	70138337	70173483	35146	25	146	273.2575758	71.02227399	0.895405877	0.020885772	-	400541	grey	0.149428275	FALSE	LOC400541
Region_7024	chr16	70173322	70187361	14039	7	12	38.45454545	11.36144874	0.960343403	0.036094519	+	283971	grey	-0.21035851	FALSE	CLEC18C

Region_7025	chr16	70219574	70246612	27038	16	109	135.3181818	10.77198516	0.945624336	0.026732172	-	100506060	grey	-0.154747642	FALSE	SMG1P7
Region_7026	chr16	70246778	70251940	5162	4	28	40.07575758	6.542901276	0.244313015	0.022409639	-	118460	grey	-0.069221988	FALSE	EXOSC6
Region_7027	chr16	70251983	70289707	37724	29	188	227.8484848	18.67638775	0.746129863	0.016338483	-	16	grey	0.120610151	FALSE	AARS1
Region_7028	chr16	70289663	70366260	76597	50	304	363.5909091	20.60244741	0.915559916	0.009049082	+	11269	grey	-0.034962534	FALSE	DDX19B
Region_7029	chr16	70299224	70373383	74159	45	282	332.4242424	20.15330521	0.926484909	0.009054684	+	55308	grey	-0.139908032	FALSE	DDX19A
Region_7030	chr16	70315442	70346806	31364	23	136	156.2575758	13.82896693	0.934966436	0.012562767	-	100506083	grey	-0.173576626	FALSE	DDX19A-DT
Region_7031	chr16	70375977	70439237	63260	44	291	334.1666667	20.25497723	0.616001187	0.016744504	-	6483	grey	-0.01070825	FALSE	ST3GAL2
Region_7032	chr16	70454595	70480274	25679	14	80	106.2272727	11.81367585	0.901570398	0.016918558	+	197258	grey	0.125083822	FALSE	FCSK
Region_7033	chr16	70480568	70523560	42992	24	152	190.6515152	14.64922411	0.933717655	0.014090384	-	25839	grey	0.020199497	FALSE	COG4
Region_7034	chr16	70523791	70577668	53877	17	113	138.5151515	12.36899651	0.743409035	0.030057921	+	23450	grey	-0.124768408	FALSE	SF3B3
Region_7035	chr16	70579895	70660682	80787	37	232	279.5606061	19.39954701	0.859553204	0.028390747	+	146433	grey	-0.050462842	FALSE	IL34
Region_7036	chr16	70661204	70686053	24849	16	69	91.33333333	10.86325257	0.732201388	0.036068548	-	92154	grey	-0.041472686	FALSE	MTSS2
Region_7037	chr1	70687439	70801160	113721	75	535	593	28.5506297	0.81858218	0.01727522	-	55697	grey	-0.24542169	FALSE	VAC14

	6							9	1	7				9		
Region_7038	chr16	70755035	70773251	18216	9	57	80.25757576	9.684739155	0.737364521	0.030328125	+	100130894	grey	-0.020074592	FALSE	VAC14-AS1
Region_7039	chr16	70802084	71230722	428638	50	309	352.3636364	21.72955192	0.842219342	0.028064832	-	54768	grey	0.025281674	FALSE	HYDIN
Region_7040	chr16	71366933	71426464	59531	20	117	155.469697	12.71972387	0.746645562	0.032780974	-	105371332	grey	0.080155063	FALSE	LOC105371332
Region_7041	chr16	71429980	71442054	12074	7	40	54.96969697	7.144593861	0.780449056	0.055157991	-	102723796	grey	-0.029659401	FALSE	TLE7
Region_7042	chr16	71447597	71489311	41714	17	134	169.6515152	13.44904049	0.74690363	0.026705205	-	7571	grey	-0.18675798	FALSE	ZNF23
Region_7043	chr16	71464555	71565089	100534	29	227	272.1212121	18.21201222	0.832683165	0.019411488	-	7567	grey	-0.097793301	FALSE	ZNF19
Region_7044	chr16	71525233	71538748	13515	7	48	65.5	8.211155639	0.93797689	0.032803481	+	10164	grey	0.116632556	FALSE	CHST4
Region_7045	chr16	71564952	71578187	13235	5	23	37.83333333	5.478863816	0.89452243	0.048389225	+	100132529	grey	-0.01483466	FALSE	TAT-AS1
Region_7046	chr16	71565660	71577092	11432	5	23	37.83333333	5.478863816	0.89452243	0.048389225	-	6898	grey	-0.01483466	FALSE	TAT
Region_7047	chr16	71626161	71641965	15804	8	57	74.51515152	8.860009419	0.834992381	0.039193599	+	91862	grey	-0.100292292	FALSE	MARVELD3
Region_7048	chr16	71637835	71724701	86866	21	125	155.6818182	14.25931177	0.938960651	0.02588692	-	23035	grey	-0.015554266	FALSE	PHLPP2
Region_7049	chr16	71729000	71809201	80201	26	177	209.9242424	14.91959023	0.787480766	0.009261864	-	164	grey	-0.12426624	FALSE	AP1G1

Region_7050	chr16	71808439	71885268	76829	43	273	317.5	19.03135065	0.736181066	0.014469882	+	342371	grey	0.224276599	FALSE	ATXN1L
Region_7051	chr16	71845996	71931199	85203	51	353	396.0454545	25.15027562	0.728581507	0.024244497	+	9798	grey	0.191471191	FALSE	IST1
Region_7052	chr16	71859680	71895336	35656	23	121	160.6515152	15.58844979	0.659432945	0.017818707	-	55565	grey	0.223292491	FALSE	ZNF821
Region_7053	chr16	71929538	72000402	70864	29	181	218.1363636	16.00181371	0.837620793	0.026485099	-	342372	grey	0.111158743	FALSE	PKD1L3
Region_7054	chr16	72008588	72027664	19076	11	63	91.3030303	9.876109008	0.695413332	0.019857014	+	1723	grey	-0.118279085	FALSE	DHODH
Region_7055	chr16	72044289	72094431	50142	19	128	151.6363636	11.51538952	0.64507961	0.034126859	-	54957	grey	0.044432603	FALSE	TXNL4B
Region_7056	chr16	72054505	72061055	6550	5	31	40.84848485	5.743872787	0.808084	0.06244525	+	3240	grey	0.035705229	FALSE	HP
Region_7057	chr16	72093613	72112912	19299	9	62	84.54545455	9.895114285	0.643610591	0.020149821	+	9785	grey	0.324096057	FALSE	DHX38
Region_7058	chr16	72112157	72176878	64721	14	103	128.5606061	11.58796297	0.924378135	0.025205715	-	83449	grey	0.126083074	FALSE	PMFBP1
Region_7059	chr16	72236281	72665014	428733	51	366	409.7727273	21.11929155	0.927028448	0.013915048	-	101927957	grey	0.100513628	FALSE	LINC01572
Region_7060	chr16	72665123	72828293	163170	16	115	141.4848485	11.82597197	0.930022886	0.02351844	+	101927978	grey	0.149576518	FALSE	ZFHX3-AS1
Region_7061	chr16	72782885	73891871	1108986	333	2543	2719.439394	70.77703426	0.826654035	0.008885453	-	463	grey	0.123017402	FALSE	ZFHX3
Region_7062	chr1	73092349	73099337	6988	9	54	70.1212121	9.71203084	0.92737127	0.03495539	+	283902	grey	0.16060882	FALSE	HCCAT5

	6						2	4	5	9						
Region_7063	chr16	73386771	73504954	118183	23	147	179.969697	13.27226106	0.866666359	0.023820413	+	100506172	grey	0.217150137	FALSE	LOHAN2
Region_7064	chr16	73943078	74296762	353684	42	322	364.5606061	17.5447812	0.889514383	0.019101917	-	101928035	grey	-0.029268236	FALSE	PSMD7-DT
Region_7065	chr16	74296814	74306288	9474	8	61	83.96969697	11.30958708	0.125904825	0.017750826	+	5713	grey	-0.164167019	FALSE	PSMD7
Region_7066	chr16	74310100	74368240	58140	29	206	262.530303	29.36571507	0.84045281	0.021135011	-	283922	grey	-0.354050984	FALSE	PDPR2P
Region_7067	chr16	74376306	74392115	15809	7	23	52.22727273	15.14930357	0.928695932	0.050638378	+	440348	grey	0.012613304	FALSE	NPIPB15
Region_7068	chr16	74408631	74421478	12847	5	24	47.24242424	12.87222485	0.959164424	0.032766067	-	497190	grey	-0.014857952	FALSE	CLEC18B
Region_7069	chr16	74422120	74441937	19817	8	44	69.71212121	8.71032129	0.882134329	0.062188935	+	107984827	grey	-0.492664436	FALSE	LOC107984827
Region_7070	chr16	74447427	74607144	159717	60	409	459.7575758	25.47098537	0.883880251	0.021953681	-	2734	grey	-0.36876951	FALSE	GLG1
Region_7071	chr16	74621399	74666877	45478	24	129	174.8030303	16.63566114	0.890929733	0.020342293	-	55159	grey	-0.16014547	FALSE	RFWD3
Region_7072	chr16	74671855	74700960	29105	23	147	181.7878788	15.72603442	0.713146166	0.017517819	-	197259	grey	-0.17451349	FALSE	MLKL
Region_7073	chr16	74712955	74774831	61876	33	208	242.0454545	14.4397228	0.793207315	0.023155831	-	79152	grey	0.272238196	FALSE	FA2H
Region_7074	chr16	74871362	75000173	128811	64	438	490.030303	19.15440877	0.860164144	0.010594321	-	79726	grey	-0.232358356	FALSE	WDR59

Region_7075	chr16	74999024	75110994	111970	47	332	374.530303	16.62733588	0.694027772	0.016949842	+	84937	grey	0.255071353	FALSE	ZNRF1
Region_7076	chr16	75111860	75116780	4920	7	38	49.24242424	6.787002524	0.711353743	0.027441692	-	197257	grey	0.134108866	FALSE	LDHD
Region_7077	chr16	75119558	75172236	52678	44	282	322.4848485	23.74754557	0.616730214	0.011597189	+	162239	grey	0.140178786	FALSE	ZFP1
Region_7078	chr16	75228181	75268053	39872	45	245	286.0454545	19.49471867	0.611419514	0.021453623	-	9564	grey	0.292825969	FALSE	BCAR1
Region_7079	chr16	75293698	75464552	170854	57	370	432.7424242	25.15509467	0.884821115	0.01637061	-	10428	grey	-0.236990216	FALSE	CFDP1
Region_7080	chr16	75412684	75465497	52813	21	114	162.030303	15.72452244	0.63572825	0.036729804	-	124491	grey	-0.126988789	FALSE	TMEM170A
Region_7081	chr16	75472052	75495445	23393	11	73	96.01515152	10.74529224	0.865033752	0.026020276	-	4166	grey	0.067080786	FALSE	CHST6
Region_7082	chr16	75528530	75545428	16898	18	90	134.5151515	17.93255949	0.95539733	0.020808711	-	23563	grey	-0.041475241	FALSE	CHST5
Region_7083	chr16	75536741	75608445	71704	40	299	353.4090909	22.35102976	0.796159675	0.014702376	-	79583	grey	0.027779467	FALSE	TMEM231
Region_7084	chr16	75566375	75577881	11506	4	17	33.16666667	6.047461854	0.493230091	0.030937035	+	11345	grey	0.15637336	FALSE	GABARAPL2
Region_7085	chr16	75596868	75623300	26432	14	99	128.8636364	11.0019388	0.74316758	0.030924598	-	23536	grey	0.178209671	FALSE	ADAT1
Region_7086	chr16	75627474	75648643	21169	10	71	98.1969697	12.56272839	0.356417416	0.022356088	-	3735	grey	-0.131704962	FALSE	KARS1
Region_7087	chr1	75647773	75657432	9659	7	35	61.6969697	9.67784098	0.41546670	0.02165705	+	54386	grey	-0.06702976	FALSE	TERF2IP

	6							1	3	4				7		
Region_7088	chr16	75647791	75761872	114081	29	205	249.6212121	18.12670376	0.789783385	0.022968119	+	105371348	grey	0.030774587	FALSE	LOC105371348
Region_7089	chr16	75660227	75677009	16782	5	26	44.03030303	6.729886722	0.964070088	0.03661652	-	112268164	grey	-0.010398356	FALSE	CPHXL2
Region_7090	chr16	75693893	75701461	7568	3	16	27.96969697	5.640430144	0.902876141	0.061662076	-	100033411	grey	0.029408083	FALSE	DUXB
Region_7091	chr16	76107283	76147779	40496	5	27	40.86363636	5.95337361	0.852018594	0.076171256	-	105371349	grey	-0.135895565	FALSE	LOC105371349
Region_7092	chr16	76277278	76819624	542346	41	291	352.7424242	21.22430436	0.850387449	0.031354362	+	85445	grey	0.028316876	FALSE	CNTNAP4
Region_7093	chr16	77190835	77202398	11563	3	12	20.1969697	4.26513485	0.983328905	0.031178501	+	22879	grey	0.05179737	FALSE	MON1B
Region_7094	chr16	77199408	77213215	13807	4	14	23.60606061	5.543891407	0.957580353	0.046331082	+	100130958	grey	0.038994209	FALSE	SYCE1L
Region_7095	chr16	77247813	77435034	187221	28	169	208.7878788	17.77683467	0.755053242	0.023776903	-	170692	grey	0.113537244	FALSE	ADAMTS18
Region_7096	chr16	77722492	77742260	19768	10	65	83.51515152	9.683042154	0.851675354	0.024487033	+	283927	grey	-0.009240195	FALSE	NUDT7
Region_7097	chr16	77788564	77980107	191543	51	351	389.0757576	19.34887045	0.802448061	0.021023442	+	57687	grey	-0.115684008	FALSE	VAT1L
Region_7098	chr16	78022515	78066761	44246	7	35	50.31818182	7.181512785	0.822223285	0.04495259	+	107984897	grey	0.26709671	FALSE	LOC107984897
Region_7099	chr16	78022546	78032101	9555	3	10	23.92424242	5.304020554	0.941211636	0.050245946	+	10143	grey	0.264220744	FALSE	CLEC3A

Region_7100	chr16	78099400	79212667	1113267	283	2220	2353.681818	59.97798547	0.895749667	0.008700188	+	51741	grey	0.081963535	FALSE	WWOX
Region_7101	chr16	79090050	79101754	11704	3	20	30.12121212	4.57926817	0.550275787	0.084735719	+	107984806	grey	0.126317545	FALSE	LOC107984806
Region_7102	chr16	79208493	79600737	392244	98	749	829.8030303	33.47937124	0.810486068	0.015929596	-	4094	grey	-0.131540081	FALSE	MAF
Region_7103	chr16	79676048	79807923	131875	26	172	205.5606061	16.46546856	0.857555435	0.025727084	+	105371356	grey	-0.233280509	FALSE	LOC105371356
Region_7104	chr16	79676050	79809716	133666	26	172	205.5606061	16.46546856	0.857555435	0.025727084	+	101928248	grey	-0.233280509	FALSE	LINC01229
Region_7105	chr16	79715220	79770651	55431	7	46	58.06060606	7.425276556	0.721609786	0.062313322	-	102467146	grey	-0.041796043	FALSE	MAFTRR
Region_7106	chr16	79798050	79827150	29100	6	33	45.53030303	7.986950312	0.909105687	0.052884618	-	104266962	grey	-0.021445638	FALSE	LINC01228
Region_7107	chr16	80065836	80167577	101741	9	70	87.93939394	9.226848429	0.922766328	0.032545355	+	105371357	grey	-0.126095947	FALSE	LOC105371357
Region_7108	chr16	80154979	80563354	408375	50	371	417.1363636	19.76001118	0.845835181	0.017315639	-	102724084	grey	-0.20211069	FALSE	DYNLRB2-AS1
Region_7109	chr16	80540734	80550811	10077	4	13	25.25757576	4.999417215	0.677059238	0.069168586	+	83657	grey	-0.071907059	FALSE	DYNLRB2
Region_7110	chr16	80553256	80572808	19552	8	47	64.96969697	7.589404957	0.808057315	0.042819265	-	101928276	grey	-0.009893187	FALSE	LINC01227
Region_7111	chr16	80597907	80804598	206691	34	259	298.7575758	17.34097913	0.94720086	0.015144897	-	124359	grey	0.252763683	FALSE	CDYL2
Region_7112	chr1	80826074	81006894	180820	39	260	303.727272	18.2078135	0.87020976	0.01961370	-	10099642	grey	0.189666263	FALSE	ARLNC1

	6						7	8	8	4		5				
Region_7113	chr16	80966448	81020270	53822	17	130	163.6515152	13.47418321	0.678725756	0.021795933	-	56942	grey	0.052700878	FALSE	CMC2
Region_7114	chr16	81006552	81033114	26562	14	101	129.7424242	12.10252435	0.683802534	0.024132379	+	55839	grey	0.070023167	FALSE	CENPN
Region_7115	chr16	81016792	81096284	79492	29	178	213.4393939	16.25387886	0.803464832	0.014571772	-	107984858	grey	0.108299883	FALSE	CENPN-AS1
Region_7116	chr16	81035842	81047350	11508	5	22	36.45454545	5.00824495	0.775864716	0.03380636	+	23300	grey	0.052191597	FALSE	ATMIN
Region_7117	chr16	81053497	81096296	42799	19	107	135.3636364	14.03143324	0.77303529	0.019562207	-	123775	grey	0.088257774	FALSE	C16orf46
Region_7118	chr16	81081945	81096395	14450	10	38	69.03030303	10.74597556	0.786571537	0.031864598	-	2653	grey	0.095892899	FALSE	GCSH
Region_7119	chr16	81100875	81220370	119495	63	398	445.5909091	23.48222062	0.868417825	0.018869409	-	114780	grey	0.048765228	FALSE	PKD1L2
Region_7120	chr16	81238689	81291142	52453	29	199	232.4242424	16.73877176	0.892844789	0.020813613	+	53630	grey	-0.009026899	FALSE	BCO1
Region_7121	chr16	81314944	81390809	75865	23	133	157.8333333	13.7824675	0.752996138	0.016402159	+	8139	grey	0.220522433	FALSE	GAN
Region_7122	chr16	81444808	81711762	266954	197	1456	1598.636364	63.64749953	0.823379091	0.013218664	+	80790	grey	0.03133642	FALSE	CMIP
Region_7123	chr16	81739033	81962685	223652	85	597	653.8787879	27.65919803	0.741069866	0.01663458	+	5336	grey	-0.128497286	FALSE	PLCG2
Region_7124	chr16	81988855	82011481	22626	4	19	32.24242424	5.585862942	0.66544122	0.06864917	-	93517	grey	0.084983966	FALSE	SDR42E1

Region_7125	chr16	82035004	82098534	63530	19	108	144.469697	14.86577866	0.90039833	0.034079346	+	3294	grey	0.206844322	FALSE	HSD17B2
Region_7126	chr16	82044336	82139631	95295	22	145	173.1818182	15.01169707	0.898567795	0.033115119	-	105371363	grey	0.205667814	FALSE	HSD17B2-AS1
Region_7127	chr16	82147798	82170224	22426	7	33	49.3030303	6.483042251	0.933515378	0.090379013	-	10200	grey	-0.051956873	FALSE	MPHOSPH6
Region_7128	chr16	82491738	82575518	83780	13	95	113.4242424	9.575707415	0.843756855	0.042232983	-	101928392	grey	-0.015241639	FALSE	LOC101928392
Region_7129	chr16	82626965	83800640	1173675	224	1819	1942.924242	64.51340006	0.839893428	0.01471809	+	1012	grey	-0.072824974	FALSE	CDH13
Region_7130	chr16	82773319	82829638	56319	9	55	74.04545455	8.486064265	0.864365238	0.051900532	+	101928446	grey	-0.376281218	FALSE	LOC101928446
Region_7131	chr16	83687779	83772949	85170	19	139	166.7272727	12.50420908	0.779709059	0.038713041	-	105371366	grey	-0.070030674	FALSE	LOC105371366
Region_7132	chr16	83719311	83819737	100426	24	152	196.4848485	16.12856274	0.806619567	0.037586	+	3281	grey	0.060549219	FALSE	HSBP1
Region_7133	chr16	83789967	83807834	17867	3	12	20.07575758	4.308526809	0.766853993	0.080487377	-	102724163	grey	0.233303129	FALSE	CDH13-AS2
Region_7134	chr16	83808101	83951155	143054	79	545	616.6363636	33.34003662	0.898995954	0.015472691	+	23417	grey	0.310072178	FALSE	MLYCD
Region_7135	chr16	83908132	83957650	49518	50	308	369.1666667	29.52317647	0.865427404	0.015845622	+	105371371	grey	0.177142315	FALSE	
Region_7136	chr16	83949067	83966332	17265	22	138	167.7878788	15.33273537	0.759075428	0.024501564	+	29948	grey	0.448787477	FALSE	OSGIN1
Region_7137	chr1	83968244	84002776	34532	42	245	289.272727	19.4576822	0.72871403	0.02499077	+	54550	grey	-0.02792250	FALSE	NECAB2

	6						3	5	5	4				2		
Region_7138	chr16	84009667	84042795	33128	31	217	249.3484848	17.49944388	0.802212334	0.028185746	-	146167	grey	-0.282402415	FALSE	SLC38A8
Region_7139	chr16	84053761	84116942	63181	54	390	434.3939394	20.87905007	0.814756715	0.014124049	-	8720	grey	0.222747503	FALSE	MBTPS1
Region_7140	chr16	84122141	84145192	23051	10	53	72.28787879	8.402866089	0.8380234	0.031556282	-	83693	grey	-0.083891223	FALSE	HSDL1
Region_7141	chr16	84145287	84178767	33480	22	166	197.7424242	13.51843255	0.811683134	0.03320844	+	123872	grey	0.18107966	FALSE	DNAAF1
Region_7142	chr16	84177847	84187070	9223	13	67	93.83333333	11.37552478	0.890547487	0.044048058	-	9013	grey	0.29372223	FALSE	TAF1C
Region_7143	chr16	84191138	84197168	6030	14	66	89.09090909	10.52137352	0.822065814	0.037429367	+	161931	grey	0.025105547	FALSE	ADAD2
Region_7144	chr16	84192558	84197053	4495	8	36	50.13636364	7.755665648	0.70643283	0.063107888	-	654780	grey	0.023906661	FALSE	LOC654780
Region_7145	chr16	84218657	84240012	21355	19	124	150.469697	15.56645278	0.798652627	0.029053107	-	93107	grey	0.040693415	FALSE	KCNG4
Region_7146	chr16	84294846	84329844	34998	16	120	143.3030303	11.34490017	0.89978148	0.035339946	+	58189	grey	-0.06221349	FALSE	WFDC1
Region_7147	chr16	84368527	84464187	95660	54	369	410.2878788	24.63441317	0.877863886	0.019157715	+	9914	grey	0.025214609	FALSE	ATP2C2
Region_7148	chr16	84459259	84467361	8102	3	18	28.53030303	5.075490721	0.901799892	0.082074671	-	105371374	grey	0.03280719	FALSE	ATP2C2-AS1
Region_7149	chr16	84476355	84554033	77678	66	452	519.2727273	30.24949864	0.787674569	0.01491696	-	57707	grey	0.143206317	FALSE	MEAK7

Region_7150	chr1<br>6	84565596	84618078	52482	40	267	317.318181<br>8	20.0070442<br>1	0.64555428<br>4	0.02226632	-	23406	grey	0.08721388	FALSE	COTL1
Region_7151	chr1<br>6	84648511	84667686	19175	21	143	167.030303	11.8073897<br>3	0.88948278<br>2	0.01895833<br>2	+	79786	grey	0.065448426	FALSE	KLHL36
Region_7152	chr1<br>6	84699986	84779922	79936	52	347	391.787878<br>8	21.7676649<br>1	0.76159031<br>6	0.01987190<br>5	+	9100	grey	-0.14680987<br>5	FALSE	USP10
Region_7153	chr1<br>6	84819985	84920768	100783	78	516	577.121212<br>1	30.5274228<br>1	0.82583645<br>9	0.01666761<br>9	+	83716	grey	0.079405179	FALSE	CRISPLD2
Region_7154	chr1<br>6	84917377	84924808	7431	5	14	29.2575757<br>6	6.61771656<br>2	0.95260179<br>2	0.04275176<br>8	-	10192847<br>4	grey	-0.12568255<br>5	FALSE	LOC101928474
Region_7155	chr1<br>6	84928353	84939603	11250	8	32	51.0757575<br>8	8.35244893<br>8	0.91146094<br>3	0.04577417<br>4	+	10537137<br>9	grey	-0.03551880<br>1	FALSE	LINC02176
Region_7156	chr1<br>6	84974175	85011535	37360	35	240	278.878787<br>9	16.9274607<br>6	0.87103639<br>6	0.01910483<br>6	-	55625	grey	-0.31694306<br>4	FALSE	ZDHHC7
Region_7157	chr1<br>6	85027782	85094230	66448	45	334	388.893939<br>4	22.3118656<br>2	0.78252809<br>9	0.02168016<br>4	+	9764	grey	-0.25859380<br>7	FALSE	KIAA0513
Region_7158	chr1<br>6	85098358	85112472	14114	17	108	136.409090<br>9	12.1806621<br>9	0.84109836<br>5	0.02876910<br>9	-	339145	grey	-0.06199080<br>9	FALSE	CIBAR2
Region_7159	chr1<br>6	85137150	85149443	12293	16	108	127.712121<br>2	10.4348014<br>6	0.71044960<br>2	0.03364820<br>7	+	400548	grey	-0.09395783	FALSE	LINC02139
Region_7160	chr1<br>6	85169525	85676204	506679	676	4570	4943.78787<br>9	171.201052<br>8	0.77781608<br>4	0.01084128<br>9	+	23199	grey	-0.27023564<br>5	FALSE	GSE1
Region_7161	chr1<br>6	85227203	85309176	81973	102	675	775.469697	35.8433427<br>8	0.73419174	0.02013283<br>9	+	10798483<br>0	grey	-0.19081806<br>6	FALSE	LOC107984830
Region_7162	chr1	85282958	85285963	3005	8	37	56.9090909	8.71908123	0.89796523	0.04417218	+	197196	grey	0.27634888	FALSE	LINC00311

	6						1	7	7	4						
Region_7163	chr16	85676198	85690073	13875	29	190	242.1818182	18.87277982	0.709299567	0.016387455	-	51659	grey	-0.015659965	FALSE	GINS2
Region_7164	chr16	85690084	85751129	61045	57	399	454.3484848	28.95327399	0.848441115	0.017625527	-	404550	grey	0.382985081	FALSE	C16orf74
Region_7165	chr16	85771758	85799608	27850	32	233	271.030303	17.40996151	0.88949678	0.014410779	-	10328	grey	0.055183183	FALSE	EMC8
Region_7166	chr16	85792415	85792933	518	4	28	41.06060606	6.869954177	0.977370572	0.02731051	+	101928557	grey	0.188567597	FALSE	LOC101928557
Region_7167	chr16	85798633	85807068	8435	9	51	74.93939394	10.20087295	0.31707201	0.028509788	+	1327	grey	0.108208363	FALSE	COX4I1
Region_7168	chr16	85899116	85922606	23490	9	54	77.1969697	8.994390897	0.678210779	0.047599387	+	3394	grey	0.054496485	FALSE	IRF8
Region_7169	chr16	85924984	85948824	23840	26	176	229.030303	16.86869627	0.655755422	0.03288182	+	105371388	grey	-0.136899537	FALSE	LOC105371388
Region_7170	chr16	86221423	86301303	79880	24	165	202	16.90880517	0.766078238	0.030215622	-	101154687	grey	-0.038197767	FALSE	LINC01081
Region_7171	chr16	86286427	86293389	6962	3	12	22.51515152	5.475097257	0.300937638	0.132058393	+	146513	grey	-0.0674868	FALSE	LINC02135
Region_7172	chr16	86331632	86349688	18056	11	55	81.25757576	11.15390322	0.678141502	0.041676174	-	732275	grey	-0.069041986	FALSE	LINC00917
Region_7173	chr16	86474529	86509099	34570	20	113	151.4848485	17.57641989	0.132953277	0.024245969	-	400550	grey	-0.012083201	FALSE	FENDRR
Region_7174	chr16	86510527	86515422	4895	6	39	54.3030303	8.866479133	0.065349656	0.044522546	+	2294	grey	-0.007336445	FALSE	FOXF1

Region_7175	chr16	86530186	86555235	25049	56	386	454.9242424	31.18101523	0.918088075	0.012940919	-	64779	grey	0.312187511	FALSE	MTHFSD
Region_7176	chr16	86565145	86567847	2702	14	85	127.6363636	13.4709218	0.086203999	0.030616008	-	103752587	grey	-0.070962734	FALSE	FOXC2-AS1
Region_7177	chr16	86566829	86569728	2899	5	25	37.68181818	6.288350868	0.047930344	0.037369656	+	2303	grey	-0.025533943	FALSE	FOXC2
Region_7178	chr16	86578549	86583478	4929	6	13	37.72727273	8.142094712	0.313537666	0.046723779	+	2300	grey	-0.002689958	FALSE	FOXL1
Region_7179	chr16	86710122	86742083	31961	19	139	167.1818182	12.59292731	0.681437447	0.034269495	+	102724344	grey	-0.001726006	FALSE	LINC02188
Region_7180	chr16	87057784	87070105	12321	13	73	95.34848485	12.27942923	0.797245271	0.040621239	+	440390	grey	-0.012329258	FALSE	LINC02181
Region_7181	chr16	87212122	87226430	14308	8	55	75.40909091	8.772294275	0.897465336	0.040501682	+	101928708	grey	-0.222626815	FALSE	LOC101928708
Region_7182	chr16	87262759	87292438	29679	18	128	160.1060606	14.30989844	0.862639854	0.026342146	-	101928682	grey	-0.212266301	FALSE	LOC101928682
Region_7183	chr16	87302801	87334273	31472	19	116	144	13.5510431	0.745269526	0.022058158	-	100506581	grey	-0.155609378	FALSE	C16orf95
Region_7184	chr16	87326987	87392142	65155	65	480	538.5909091	30.0921545	0.780386387	0.014918442	-	79791	grey	-0.09292522	FALSE	FBXO31
Region_7185	chr16	87383953	87404779	20826	29	158	205.3333333	20.59897931	0.384218866	0.01628815	+	81631	grey	0.334214125	FALSE	MAP1LC3B
Region_7186	chr16	87406246	87493024	86778	102	752	827.1818182	42.73276858	0.863725033	0.012129718	-	23174	grey	0.36352182	FALSE	ZCCHC14
Region_7187	chr1	87492555	87516940	24385	24	176	204.121212	13.9532352	0.76453946	0.02853648	+	10192873	grey	0.358459596	FALSE	ZCCHC14-DT

	6						1	5	7	7		7				
Region_7188	chr16	87601835	87698156	96321	159	1067	1159.121212	52.78243156	0.788945712	0.01590548	+	57338	grey	-0.055822988	FALSE	JPH3
Region_7189	chr16	87696485	87765992	69507	118	803	940.5	48.84740762	0.8896409	0.015038486	-	54758	grey	0.207190845	FALSE	KLHDC4
Region_7190	chr16	87773304	87779374	6070	7	35	53	6.823037898	0.629759106	0.061950476	-	102724467	grey	0.186529475	FALSE	LOC102724467
Region_7191	chr16	87830023	87869507	39484	71	492	557.8939394	29.43003635	0.689256608	0.020614006	-	8140	grey	0.122224929	FALSE	SLC7A5
Region_7192	chr16	87881546	87936580	55034	83	584	677.0606061	37.48102239	0.871664352	0.014542112	-	763	grey	0.152800693	FALSE	CA5A
Region_7193	chr16	87949244	88077318	128074	175	1191	1320.106061	54.51637123	0.891469742	0.00917728	+	54971	grey	0.061924828	FALSE	BANP
Region_7194	chr16	88088041	88100985	12944	20	104	130.6212121	11.85523598	0.714649126	0.040151362	-	400553	grey	0.028793731	FALSE	LOC400553
Region_7195	chr16	88177313	88186929	9616	15	76	97.56060606	9.190511475	0.875080065	0.048772614	-	105371401	grey	-0.504340583	FALSE	LOC105371401
Region_7196	chr16	88186605	88195217	8612	11	55	79.24242424	9.160215997	0.646547962	0.062902757	+	101928880	grey	-0.09784618	FALSE	LINC02182
Region_7197	chr16	88382959	88440753	57794	12	49	74.45454545	8.561419397	0.732914127	0.046245649	+	84627	grey	0.024625138	FALSE	ZNF469
Region_7198	chr16	88453280	88537031	83751	169	912	1078.090909	69.68472458	0.68530533	0.013879366	+	161882	grey	0.098534341	FALSE	ZFPM1
Region_7199	chr16	88512960	88531053	18093	24	142	175.6363636	16.11462438	0.831242935	0.023956845	-	100128882	grey	0.084197086	FALSE	ZFPM1-AS1

Region_7200	chr16	88570403	88631964	61561	82	543	607.4090909	32.55101429	0.885580138	0.015740839	+	124245	grey	0.231148017	FALSE	ZC3H18
Region_7201	chr16	88643275	88651083	7808	16	75	103.7121212	10.9076757	0.763599392	0.047815951	-	1535	grey	-0.136559327	FALSE	CYBA
Region_7202	chr16	88651935	88663161	11226	21	121	161.4545455	15.2680479	0.614210049	0.029030009	-	4597	grey	-0.063203452	FALSE	MVD
Region_7203	chr16	88663298	88687282	23984	29	155	184.6666667	16.2028804	0.657303888	0.027003257	+	197187	grey	0.152618023	FALSE	SNAI3-AS1
Region_7204	chr16	88677688	88686507	8819	11	38	62.51515152	9.969557392	0.51428062	0.061961066	-	333929	grey	0.180460778	FALSE	SNAI3
Region_7205	chr16	88696499	88706408	9909	34	208	245.5	20.48432797	0.296048931	0.019659892	-	115992	grey	0.096030818	FALSE	RNF166
Region_7206	chr16	88706483	88715396	8913	18	86	120.8787879	15.83036532	0.626290328	0.027619161	+	348180	grey	-0.136969588	FALSE	CTU2
Region_7207	chr16	88715338	88785220	69882	159	974	1083.651515	65.47800392	0.811065875	0.013041523	-	9780	grey	-0.113233364	FALSE	PIEZO1
Region_7208	chr16	88731180	88741425	10245	7	28	46.57575758	6.850178226	0.85903014	0.055435567	+	100289580	grey	-0.205348086	FALSE	LOC100289580
Region_7209	chr16	88742767	88745748	2981	6	25	41.09090909	6.674238125	0.776352653	0.075980656	+	339059	grey	0.007539661	FALSE	LOC339059
Region_7210	chr16	88803789	88809258	5469	27	150	195.3939394	17.99305745	0.660883925	0.025427832	+	81620	grey	-0.100134701	FALSE	CDT1
Region_7211	chr16	88813734	88856947	43213	103	607	717.530303	45.51963894	0.881572998	0.01021282	-	2588	grey	0.02683307	FALSE	GALNS
Region_7212	chr1	88856220	88862686	6466	11	82	109.060606	10.6984956	0.41607472	0.02140307	+	51693	grey	-0.12603266	FALSE	TRAPPC2L

	6						1	4	2	4				9		
Region_7213	chr16	88874858	88977207	102349	300	1870	2065.19697	104.8151809	0.790369241	0.010584031	-	863	grey	-0.307201577	FALSE	CBFA2T3
Region_7214	chr16	88883272	88887136	3864	15	87	110.6212121	12.25468846	0.960493604	0.020715048	+	101927793	grey	0.116141395	FALSE	LOC101927793
Region_7215	chr16	88936779	88937756	977	4	26	41.16666667	7.203809533	0.31680012	0.06985576	-	107984871	grey	-0.196698655	FALSE	LOC107984871
Region_7216	chr16	88939789	88951524	11735	30	196	234.9545455	22.50020979	0.444898635	0.027358861	+	100129697	grey	-0.136943807	FALSE	LOC100129697
Region_7217	chr16	89088375	89164121	75746	123	917	999.8030303	47.5534419	0.833469764	0.011396264	+	197322	grey	0.106870106	FALSE	ACSF3
Region_7218	chr16	89159146	89163675	4529	18	94	134.6212121	14.87461974	0.871042807	0.031860306	+	283860	grey	0.168620397	FALSE	LINC00304
Region_7219	chr16	89166383	89169147	2764	10	32	48.8030303	7.848997382	0.567731548	0.056677901	+	400558	grey	0.158185651	FALSE	LINC02138
Region_7220	chr16	89171748	89195492	23744	35	171	211.1969697	17.25039103	0.708581285	0.026880711	+	1013	grey	0.04158554	FALSE	CDH15
Region_7221	chr16	89195761	89201651	5890	10	39	61.31818182	9.674962279	0.73816809	0.037956274	-	146429	grey	0.19313036	FALSE	SLC22A31
Region_7222	chr16	89217703	89237141	19438	28	202	231.5151515	17.40578368	0.794020341	0.023137138	+	197320	grey	0.047394908	FALSE	ZNF778
Region_7223	chr16	89267630	89490561	222931	276	1959	2191.121212	86.38242612	0.786156419	0.009138474	-	29123	grey	-0.276056404	FALSE	ANKRD11
Region_7224	chr16	89321133	89325110	3977	9	43	62.72727273	8.466305078	0.767294227	0.041272307	+	100287036	grey	0.00656728	FALSE	LOC100287036

Region_7225	chr16	89430988	89453678	22690	24	180	229.4545455	19.57170785	0.794169162	0.02229249	+	101927817	grey	-0.093839958	FALSE	LOC101927817
Region_7226	chr16	89490719	89499285	8566	21	120	167.9848485	13.99944221	0.654464958	0.022381933	+	101930112	grey	0.165743648	FALSE	LOC101930112
Region_7227	chr16	89490917	89557766	66849	114	824	900.8333333	35.3521116	0.777447505	0.009108825	+	6687	grey	-0.148698937	FALSE	SPG7
Region_7228	chr16	89492017	89504460	12443	20	102	142.6666667	13.32358618	0.90983513	0.028106419	-	101927863	grey	0.162675252	FALSE	LOC101927863
Region_7229	chr16	89560677	89563043	2366	3	20	33.43939394	5.841835296	0.012958962	0.025338701	+	606500	grey	-0.117434066	FALSE	SNORD68
Region_7230	chr16	89560677	89564542	3865	5	37	51.37878788	6.871938818	0.254635949	0.052709234	+	6137	grey	0.064040251	FALSE	RPL13
Region_7231	chr16	89575758	89597246	21488	50	263	335.5606061	27.10215932	0.842753571	0.019002743	+	27132	grey	0.120040582	FALSE	CPNE7
Region_7232	chr16	89613308	89638456	25148	49	269	321.7727273	20.61500234	0.857636323	0.021732079	+	1800	grey	0.179927572	FALSE	DPEP1
Region_7233	chr16	89644237	89657738	13501	32	171	216.9393939	19.57225571	0.723466877	0.024057608	-	5119	grey	0.232636086	FALSE	CHMP1A
Region_7234	chr16	89657740	89671272	13532	19	110	143.8636364	11.52774094	0.724684402	0.025144003	+	124045	grey	0.117358719	FALSE	SPATA33
Region_7235	chr16	89680737	89696354	15617	32	180	234.4393939	19.14891344	0.679847645	0.015565471	+	8558	grey	-0.1942713	FALSE	CDK10
Region_7236	chr16	89682620	89686569	3949	7	34	52.46969697	7.48787676	0.788992489	0.036586964	-	105371416	grey	-0.07376488	FALSE	LINC02166
Region_7237	chr1	89696357	89701705	5348	13	65	86.4090909	9.22867983	0.43284876	0.02463966	-	124044	grey	-0.04532648	FALSE	SPATA2L

	6						1	3	2							
Region_7238	chr16	89707134	89720898	13764	21	109	141.5454545	14.45223626	0.789485765	0.02891968	-	9605	grey	-0.127099656	FALSE	VPS9D1
Region_7239	chr16	89711856	89718165	6309	12	59	83.74242424	11.98628781	0.789297738	0.029869425	+	100128881	grey	-0.179769091	FALSE	VPS9D1-AS1
Region_7240	chr16	89720400	89740925	20525	44	292	336.3181818	23.79796959	0.656064498	0.021371184	+	92822	grey	-0.077783199	FALSE	ZNF276
Region_7241	chr16	89726683	89816977	90294	141	1031	1155.666667	57.58400775	0.870161316	0.009762837	-	2175	grey	0.028158698	FALSE	FANCA
Region_7242	chr16	89818179	89871319	53140	57	337	419.1515152	28.15301182	0.821629468	0.024624419	+	84501	grey	-0.039766423	FALSE	SPIRE2
Region_7243	chr16	89873570	89918233	44663	101	651	765.5909091	45.07804421	0.888006976	0.011199351	+	22980	grey	-0.068540099	FALSE	TCF25
Region_7244	chr16	89918862	89920972	2110	5	21	37.93939394	7.625622371	0.951285509	0.044793063	+	4157	grey	-0.178523573	FALSE	MC1R
Region_7245	chr16	89919165	89938761	19596	31	160	200.0757576	16.32111479	0.578407828	0.026023274	+	10381	grey	-0.172678839	FALSE	TUBB3
Region_7246	chr16	89948697	89968060	19363	25	167	194.1818182	15.41119382	0.789880467	0.031000235	+	54849	grey	0.117875862	FALSE	DEF8
Region_7247	chr16	89969791	89972534	2743	4	25	38.98484848	6.383855657	0.464526123	0.05185319	-	92806	grey	-0.449173123	FALSE	CENPBD1P
Region_7248	chr16	89972581	90002161	29580	61	397	463.9848485	25.81263401	0.835107496	0.014461491	+	172	grey	-0.143934061	FALSE	AFG3L1P
Region_7249	chr16	90004871	90019890	15019	20	104	128.969697	11.59306118	0.779802363	0.024346175	-	79007	grey	0.145194745	FALSE	DBNDD1

Region_7250	chr16	90019629	90044975	25346	28	169	222.5909091	18.98013315	0.780126987	0.0182275	+	2622	grey	0.199536368	FALSE	GAS8
Region_7251	chr16	90028908	90029901	993	3	18	31.04545455	5.289850422	0.793743573	0.113909024	-	750	grey	0.237266906	FALSE	GAS8-AS1
Region_7252	chr16	90039761	90047773	8012	5	22	35.16666667	6.391300177	0.751134926	0.047024515	-	100130015	grey	-0.441371143	FALSE	URAHP
Region_7253	chr16	90056566	90092072	35506	30	208	248.3030303	17.66262433	0.697347885	0.028292189	-	11105	grey	-0.03886484	FALSE	PRDM7
Region_7254	chr16	90102271	90177606	75335	15	47	92.16666667	22.85579094	0.845520963	0.056771079	+	100996541	grey	-0.005845438	FALSE	FAM157C
Region_7255	chr16	90106457	90178344	71887	12	30	72.83333333	22.68688766	0.892452673	0.040207981	+	105376781	grey	-0.224156815	FALSE	LOC105376781
Region_7256	chr17	732412	742968	10556	17	115	139.2727273	12.53493022	0.844727223	0.024949648	+	79850	grey	-0.287579284	FALSE	TLCD3A
Region_7257	chr17	744421	753999	9578	13	84	111.969697	13.16635537	0.516925037	0.029613166	-	50628	grey	-0.110145399	FALSE	GEMIN4
Region_7258	chr17	759323	782294	22971	30	196	236.8181818	14.51991639	0.828186947	0.020130734	-	51031	grey	-0.046967478	FALSE	GLOD4
Region_7259	chr17	782353	792509	10156	12	70	100.9848485	10.97618741	0.758486339	0.0305959	+	55178	grey	0.088346108	FALSE	MRM3
Region_7260	chr17	799310	979776	180466	270	1840	1980.848485	70.60425841	0.873645964	0.008423825	-	64359	grey	0.089534124	FALSE	NXN
Region_7261	chr17	909632	911212	1580	4	18	28.36363636	4.741425828	0.917394217	0.045915982	+	101927727	grey	0.050390931	FALSE	LOC101927727
Region_7262	chr1	1270444	1271815	1371	3	13	23.7727272	5.77206923	0.44510065	0.15381212	+	727857	grey	-0.01314139	FALSE	BHLHA9

	7						7	2	1					2		
Region_7263	chr17	1279662	1300978	21316	15	84	110.7575758	12.108561	0.799483268	0.028785312	+	286753	grey	0.080435186	FALSE	TRARG1
Region_7264	chr17	1420689	1463162	42473	43	281	329.8636364	22.92991755	0.898271611	0.014995498	-	1398	grey	-0.2170245	FALSE	CRK
Region_7265	chr17	1464186	1492686	28500	54	300	339.7878788	22.6005549	0.759078201	0.016069501	-	4641	grey	0.102430444	FALSE	MYO1C
Region_7266	chr17	1494577	1516742	22165	29	171	220.5909091	15.83761566	0.841136997	0.019458849	-	51763	grey	-0.429338269	FALSE	INPP5K
Region_7267	chr17	1516877	1518101	1224	5	25	39	7.179029289	0.033722936	0.029774085	+	100306951	grey	-0.0335047	FALSE	PITPNA-AS1
Region_7268	chr17	1517718	1562792	45074	46	259	338.7121212	25.02783998	0.914090781	0.017838515	-	5306	grey	-0.065989706	FALSE	PITPNA
Region_7269	chr17	1779485	1830634	51149	59	406	455	27.46914353	0.868744039	0.019242278	-	114826	grey	-0.465040774	FALSE	SMYD4
Region_7270	chr17	1829702	1900082	70380	56	390	443.6060606	21.408035	0.94742158	0.010751339	+	6117	grey	-0.165098603	FALSE	RPA1
Region_7271	chr17	1934677	2025334	90657	106	673	751.0909091	35.20564958	0.849741597	0.015968192	-	146760	grey	-0.003718141	FALSE	RTN4RL1
Region_7272	chr17	2019952	2023664	3712	3	11	23.72727273	4.70530128	0.096205396	0.059832264	-	105371486	grey	0.38740941	FALSE	LOC105371486
Region_7273	chr17	2030137	2043898	13761	9	44	65.86363636	7.643773461	0.634445184	0.028351684	+	1801	grey	0.052387016	FALSE	DPH1
Region_7274	chr17	2054154	2063241	9087	16	95	124.2121212	13.62747807	0.033189878	0.025059476	+	3090	grey	-0.352305992	FALSE	HIC1

Region_7275	chr17	2059843	2303785	243942	131	890	982.4242424	41.29520762	0.818600677	0.011414145	-	23293	grey	0.366047879	FALSE	SMG6
Region_7276	chr17	2303383	2325260	21877	29	126	166.6969697	17.70959731	0.594663448	0.022717075	+	63826	grey	0.183100446	FALSE	SRR
Region_7277	chr17	2322396	2336657	14261	6	26	44.84848485	6.855076556	0.734138079	0.050655214	-	55720	grey	0.093755394	FALSE	TSR1
Region_7278	chr17	2337498	2381058	43560	38	253	294.5606061	19.38923475	0.877370847	0.020863901	+	9905	grey	0.188799794	FALSE	SGSM2
Region_7279	chr17	2384073	2401104	17031	24	106	134.7727273	13.22910015	0.271750014	0.017878107	-	4335	grey	0.247727949	FALSE	MNT
Region_7280	chr17	2405562	2416085	10523	9	62	88.22727273	10.63636065	0.873154805	0.053526938	-	284009	grey	0.353206834	FALSE	LOC284009
Region_7281	chr17	2416049	2511891	95842	48	331	372.2727273	21.86078009	0.920097952	0.013393294	-	79066	grey	0.207103482	FALSE	METTL16
Region_7282	chr17	2593210	2685615	92405	78	551	622.2121212	32.04012636	0.853738185	0.011473303	+	5048	grey	-0.052571916	FALSE	PAFAH1B1
Region_7283	chr17	2689386	2712663	23277	43	227	263.2121212	20.38363986	0.788107213	0.019762247	-	23277	grey	0.317262085	FALSE	CLUH
Region_7284	chr17	2712012	2713607	1595	7	33	49.75757576	6.390041786	0.772707791	0.041597051	+	105371592	grey	0.21724233	FALSE	LOC105371592
Region_7285	chr17	2720801	2749652	28851	27	142	173.2878788	13.08171378	0.691062711	0.021619131	-	101928991	grey	0.001629884	FALSE	CCDC92B
Region_7286	chr17	2755705	3037741	282036	249	1681	1874.393939	62.99056798	0.798274574	0.011967316	+	23108	grey	0.067473773	FALSE	RAP1GAP2
Region_7287	chr1	3134969	3177031	42062	7	41	56.6515151	7.60261579	0.81474886	0.05588668	-	10028872	grey	0.137323378	FALSE	LOC100288728

	7						5	3	7	2		8				
Region_7288	chr1<br>7	3277199	3386317	109118	20	111	139.545454<br>5	12.3701648<br>7	0.83187168<br>9	0.04520407<br>1	-	4995	grey	0.099556383	FALSE	OR3A2
Region_7289	chr1<br>7	3411370	3424070	12700	6	21	37.8787878<br>8	8.09466139<br>6	0.85543641<br>8	0.07849070<br>3	+	8392	grey	-0.20459239<br>4	FALSE	OR3A3
Region_7290	chr1<br>7	3440019	3513852	73833	38	228	278.909090<br>9	19.7513211	0.90766783<br>2	0.02268474<br>1	-	84690	grey	0.049910951	FALSE	SPATA22
Region_7291	chr1<br>7	3472374	3503405	31031	20	125	160.681818<br>2	14.0681408<br>7	0.88718649<br>8	0.03445401<br>3	+	443	grey	-0.03406541<br>9	FALSE	ASPA
Region_7292	chr1<br>7	3510502	3557812	47310	36	191	244.772727<br>3	17.0707085<br>6	0.84134548<br>1	0.01720259<br>6	-	162514	grey	0.125730495	FALSE	TRPV3
Region_7293	chr1<br>7	3565444	3609411	43967	58	392	436.106060<br>6	28.7220787<br>7	0.93270227<br>4	0.01754116<br>6	-	7442	grey	0.120626193	FALSE	TRPV1
Region_7294	chr1<br>7	3585149	3636250	51101	69	449	510.696969<br>7	32.5018199<br>2	0.90845113<br>7	0.01716592<br>4	-	23729	grey	0.11384579	FALSE	SHPK
Region_7295	chr1<br>7	3636459	3663103	26644	32	184	219.469697	17.4789717<br>2	0.78825699<br>7	0.0165311	+	1497	grey	0.077256573	FALSE	CTNS
Region_7296	chr1<br>7	3655621	3658092	2471	3	13	18.6818181<br>8	4.42774153<br>5	0.95160152<br>7	0.05628503<br>9	-	10537149<br>2	grey	-0.14530124<br>7	FALSE	CTNS-AS1
Region_7297	chr1<br>7	3662895	3668679	5784	10	44	75.0303030<br>3	11.1396308<br>4	0.38623612<br>7	0.01635031<br>4	-	30851	grey	0.04381453	FALSE	TAX1BP3
Region_7298	chr1<br>7	3663063	3696240	33177	38	212	282.424242<br>4	25.9970771<br>2	0.58459032<br>3	0.02358844<br>9	-	10053397<br>0	grey	0.126599166	FALSE	P2RX5-TAX1BP3
Region_7299	chr1<br>7	3668812	3669668	856	3	20	27.1363636<br>4	4.31771436<br>6	0.00733531<br>3	0.01769742<br>4	+	83460	grey	0.072839627	FALSE	EMC6

Region_7300	chr17	3673227	3696216	22989	22	111	155.4848485	17.30383899	0.790583926	0.037715306	-	5026	grey	0.116441813	FALSE	P2RX5
Region_7301	chr17	3714628	3803382	88754	90	626	694.7121212	35.67320268	0.737814964	0.016367807	-	3682	grey	0.025993385	FALSE	ITGAE
Region_7302	chr17	3723903	3726699	2796	6	27	44.53030303	7.459056853	0.15509651	0.020635726	+	83903	grey	-0.044128127	FALSE	HASPIN
Region_7303	chr17	3802158	3846246	44088	34	254	305.7121212	19.14219275	0.826425378	0.02085005	-	55421	grey	0.088399776	FALSE	NCBP3
Region_7304	chr17	3860315	3894891	34576	47	263	309.969697	20.63378089	0.881365738	0.018745951	-	84254	grey	-0.149749451	FALSE	CAMKK1
Region_7305	chr17	3896592	3916476	19884	25	151	183.4545455	15.71102682	0.88399389	0.031390977	-	5023	grey	0.214689835	FALSE	P2RX1
Region_7306	chr17	3923870	3964464	40594	21	136	168.0757576	17.72812681	0.865561156	0.019249027	-	489	grey	-0.018359968	FALSE	ATP2A3
Region_7307	chr17	3977103	3981899	4796	3	10	22.93939394	5.206146627	0.955611656	0.052121953	+	105371496	grey	0.032674052	FALSE	LINC01975
Region_7308	chr17	4004445	4143030	138585	60	441	496.7121212	23.51808806	0.882633075	0.0104185	-	23140	grey	-0.026679734	FALSE	ZZEF1
Region_7309	chr17	4143168	4187310	44142	34	208	264.4545455	19.7983541	0.851212194	0.016404816	+	124936	grey	0.217864431	FALSE	CYB5D2
Region_7310	chr17	4163821	4263995	100174	48	335	394.030303	20.01535541	0.910248888	0.013286864	-	51479	grey	-0.003721337	FALSE	ANKFY1
Region_7311	chr17	4269259	4366675	97416	49	321	362	20.40135742	0.90978655	0.018496847	-	7326	grey	-0.044383557	FALSE	UBE2G1
Region_7312	chr1	4433940	4488208	54268	51	327	365.636363	22.3328914	0.85641343	0.02285633	+	201305	grey	0.097083448	FALSE	SPNS3

	7						6	8	9	7						
Region_7313	chr17	4497394	4499674	2280	7	32	47.46969697	8.659756076	0.056408794	0.040106935	-	107985053	grey	-0.237526033	FALSE	SPNS2-AS1
Region_7314	chr17	4498881	4539035	40154	35	225	264.8484848	18.19065054	0.708547807	0.022364541	+	124976	grey	0.059780882	FALSE	SPNS2
Region_7315	chr17	4538897	4555386	16489	29	173	214.1212121	18.09761153	0.75995394	0.024272078	-	10514	grey	0.027093417	FALSE	MYBBP1A
Region_7316	chr17	4556927	4560818	3891	10	56	77.62121212	10.68970051	0.958452428	0.025057522	-	124975	grey	-0.021745247	FALSE	GGT6
Region_7317	chr17	4583999	4608319	24320	29	157	198.6060606	15.0505597	0.687034244	0.029355212	+	342527	grey	-0.092003953	FALSE	SMTNL2
Region_7318	chr17	4610449	4617650	7201	12	52	83.87878788	10.26047705	0.959994085	0.025546577	+	400568	grey	-0.298618274	FALSE	LINC01996
Region_7319	chr17	4630919	4642294	11375	14	73	103.1969697	11.49343585	0.556500688	0.04327831	-	246	grey	0.112339045	FALSE	ALOX15
Region_7320	chr17	4669774	4704337	34563	30	184	226.6060606	18.98977765	0.797267779	0.02006199	-	27043	grey	-0.260875787	FALSE	PELP1
Region_7321	chr17	4704230	4705529	1299	3	13	20.3030303	4.246155535	0.328940504	0.021949415	+	101559451	grey	-0.018263815	FALSE	PELP1-DT
Region_7322	chr17	4710596	4721499	10903	9	42	57.93939394	7.621586324	0.624053337	0.034330799	+	409	grey	-0.005198738	FALSE	ARRB2
Region_7323	chr17	4731428	4733608	2180	4	15	26.34848485	5.523355792	0.696187342	0.049172411	+	400569	grey	-0.054352641	FALSE	MED11
Region_7324	chr17	4733533	4739928	6395	10	48	67.39393939	8.466910776	0.69206011	0.03816377	-	58191	grey	-0.164922084	FALSE	CXCL16

Region_7325	chr17	4739833	4746119	6286	5	13	24.28787879	5.22351238	0.430394538	0.083525884	+	84225	grey	0.260667074	FALSE	ZMYND15
Region_7326	chr17	4771884	4783213	11329	6	22	33.95454545	5.578618021	0.840670875	0.074645182	+	9032	grey	-0.200399123	FALSE	TM4SF5
Region_7327	chr17	4785285	4786433	1148	4	25	37.09090909	5.973601601	0.094736163	0.058740509	-	284013	grey	-0.021691297	FALSE	VMO1
Region_7328	chr17	4788964	4790589	1625	5	19	29.21212121	4.789460068	0.019781473	0.024644235	+	388323	grey	0.107078664	FALSE	GLTPD2
Region_7329	chr17	4801159	4807013	5854	17	91	123.3939394	13.18143075	0.928307611	0.028588077	-	119139905	grey	-0.086433436	FALSE	C17orf114
Region_7330	chr17	4807152	4823434	16282	8	39	52.34848485	8.444374412	0.759519992	0.03148515	+	5338	grey	-0.010253685	FALSE	PLD2
Region_7331	chr17	4833340	4898061	64721	57	355	411.7878788	23.31886998	0.908263893	0.014450266	+	50488	grey	-0.146363107	FALSE	MINK1
Region_7332	chr17	4897771	4934438	36667	53	388	436.530303	25.40334302	0.524914312	0.04204096	-	1145	grey	-0.108418944	FALSE	CHRNE
Region_7333	chr17	4899418	4902934	3516	24	145	190.8636364	20.69179426	0.311958232	0.091209183	+	100130311	grey	-0.070883213	FALSE	C17orf107
Region_7334	chr17	4932277	4935023	2746	3	19	28.98484848	4.724705686	0.081198916	0.071310839	+	2811	grey	-0.038637277	FALSE	GP1BA
Region_7335	chr17	4937130	4940053	2923	8	40	65.92424242	10.93380166	0.262381143	0.025222288	-	8402	grey	-0.186390929	FALSE	SLC25A11
Region_7336	chr17	4940008	4945222	5214	3	21	30.31818182	4.781885984	0.34659686	0.061182228	+	26001	grey	0.052307262	FALSE	RNF167
Region_7337	chr1	4948092	4957131	9039	12	49	69.6969697	8.46162322	0.49409349	0.02917165	+	2027	grey	0.277051815	FALSE	ENO3

	7							5	5	5						
Region_7338	chr17	4959226	4967817	8591	8	42	55.28787879	6.166323307	0.486220019	0.028151507	-	9552	grey	-0.055290131	FALSE	SPAG7
Region_7339	chr17	4967992	4987675	19683	14	65	86.51515152	10.08310457	0.653888619	0.037468431	-	23125	grey	0.03862524	FALSE	CAMTA2
Region_7340	chr17	4997950	5028401	30451	29	218	247.9545455	15.91751026	0.900805595	0.021004847	+	10749	grey	0.131527421	FALSE	KIF1C
Region_7341	chr17	5032600	5052009	19409	5	26	40.81818182	6.131655796	0.937083161	0.036282036	-	55065	grey	-0.026013054	FALSE	SLC52A1
Region_7342	chr17	5078467	5096374	17907	12	81	101.7121212	9.867998241	0.967271772	0.021861674	+	124961	grey	0.090057979	FALSE	ZFP3
Region_7343	chr17	5097223	5114395	17172	12	77	94.45454545	9.287181933	0.642772297	0.024853549	+	101928000	grey	-0.042007716	FALSE	ZNF232-AS1
Region_7344	chr17	5105269	5123162	17893	16	101	125.3030303	12.94957047	0.502249659	0.032560854	-	7775	grey	0.112856022	FALSE	ZNF232
Region_7345	chr17	5116032	5175034	59002	35	254	293.5909091	17.28047121	0.738486237	0.021987206	+	9098	grey	0.246639732	FALSE	USP6
Region_7346	chr17	5179535	5191868	12333	5	32	46.72727273	7.024233776	0.778474163	0.026006568	-	84622	grey	-0.166743178	FALSE	ZNF594
Region_7347	chr17	5192007	5248182	56175	26	165	206.0151515	18.86754839	0.944144279	0.03102129	+	100130950	grey	-0.007032275	FALSE	ZNF594-DT
Region_7348	chr17	5208920	5234860	25940	17	105	141.5151515	15.36253427	0.933824664	0.039315576	-	388325	grey	-0.007177894	FALSE	SCIMP
Region_7349	chr17	5282265	5386340	104075	35	222	257.9545455	15.7484376	0.968408416	0.012377639	+	9135	grey	0.18160527	FALSE	RABEP1

Region_7350	chr17	5359668	5420164	60496	26	165	210.3636364	13.14491579	0.924171691	0.02127148	-	4927	grey	-0.066891038	FALSE	NUP88
Region_7351	chr17	5432777	5448830	16053	10	61	87.22727273	8.902541477	0.764850891	0.02588947	-	708	grey	-0.08626039	FALSE	C1QBP
Region_7352	chr17	5440917	5468982	28065	16	103	132.9393939	13.33806443	0.747314011	0.021648964	-	56919	grey	0.214578898	FALSE	DHX33
Region_7353	chr17	5499415	5619424	120009	21	130	162.4393939	13.17938809	0.822994703	0.024791143	-	22861	grey	0.110495344	FALSE	NLRP1
Region_7354	chr17	5499427	5517887	18460	3	12	21.10606061	4.417199906	0.628301338	0.091856763	-	728392	grey	0.133877084	FALSE	LOC728392
Region_7355	chr17	5772234	5930696	158462	45	317	355.4242424	21.05162929	0.823244202	0.024907719	+	339166	grey	0.185244963	FALSE	LOC339166
Region_7356	chr17	5772926	6124427	351501	117	904	967.5151515	34.05481664	0.789881753	0.017035649	+	23302	grey	0.050406121	FALSE	WSCD1
Region_7357	chr17	6163893	6190541	26648	5	32	44.54545455	6.258979563	0.864931848	0.070589527	-	105371508	grey	-0.221887693	FALSE	LOC105371508
Region_7358	chr17	6393693	6435199	41506	14	80	106.4090909	12.55140479	0.845034406	0.03990731	-	23746	grey	0.310697107	FALSE	AIPL1
Region_7359	chr17	6451263	6556555	105292	50	317	382.8333333	20.80723494	0.817310369	0.019608921	-	83394	grey	0.056669063	FALSE	PITPNM3
Region_7360	chr17	6578147	6640711	62564	10	64	81.31818182	8.970308443	0.903342253	0.039004045	-	9851	grey	0.212556598	FALSE	KIAA0753
Region_7361	chr17	6657034	6693577	36543	10	59	84.21212121	10.81951961	0.830804718	0.026713712	+	100652883	grey	0.106031465	FALSE	ALOX15P1
Region_7362	chr1	6684719	6713377	28658	7	35	49.1212121	7.97209585	0.78641868	0.04928481	-	284111	grey	-0.09670643	FALSE	SLC13A5

	7						2	7	3	5				4		
Region_7363	chr17	6755447	6775647	20200	7	29	45.40909091	6.96359163	0.64178265	0.04732079	+	54739	grey	0.293298412	FALSE	XAF1
Region_7364	chr17	6789133	6831761	42628	9	56	74.42424242	9.318402977	0.861387598	0.051583326	-	83659	grey	-0.052836135	FALSE	TEKT1
Region_7365	chr17	6853861	6902192	48331	18	109	135.6212121	11.40818215	0.882152718	0.040075342	+	245	grey	-0.213851544	FALSE	ALOX12P2
Region_7366	chr17	6875232	7012474	137242	75	481	543.530303	27.68294098	0.817007928	0.018205904	-	100506713	grey	-0.172043802	FALSE	ALOX12-AS1
Region_7367	chr17	6985494	7019630	34136	25	140	166.8030303	12.0680375	0.692907188	0.038683482	-	100506755	grey	-0.018729358	FALSE	MIR497HG
Region_7368	chr17	6996049	7010754	14705	11	61	77.06060606	7.797196316	0.763708139	0.053324188	+	239	grey	0.226788383	FALSE	ALOX12
Region_7369	chr17	7023050	7030290	7240	4	13	25.65151515	5.501027664	0.268421278	0.037418486	+	255877	grey	0.271885164	FALSE	BCL6B
Region_7370	chr17	7036015	7040117	4102	4	13	22.1969697	5.221905626	0.171325402	0.063215978	+	201232	grey	0.153300334	FALSE	SLC16A13
Region_7371	chr17	7041621	7044092	2471	7	26	42.34848485	7.555929764	0.152644614	0.035594789	-	162515	grey	-0.093646865	FALSE	SLC16A11
Region_7372	chr17	7101322	7115700	14378	16	93	119.8333333	14.39506682	0.87502574	0.028479763	-	433	grey	0.102224014	FALSE	ASGR2
Region_7373	chr17	7137348	7144767	7419	19	112	143.1969697	12.49272982	0.874187062	0.027282834	+	105371512	grey	0.035385287	FALSE	LOC105371512
Region_7374	chr17	7173431	7179564	6133	6	18	32.24242424	5.775440326	0.52875239	0.069986821	-	432	grey	-0.009938028	FALSE	ASGR1

Region_7375	chr17	7187187	7219841	32654	25	123	156.8333333	13.89364363	0.799751423	0.035706155	-	1742	grey	-0.107742939	FALSE	DLG4
Region_7376	chr17	7217125	7225266	8141	11	57	86.27272727	13.01306498	0.302766957	0.038148047	+	37	grey	-0.136910705	FALSE	ACADVL
Region_7377	chr17	7225342	7234517	9175	13	73	96.78787879	9.468189903	0.739826237	0.021764147	-	1856	grey	-0.02936463	FALSE	DVL2
Region_7378	chr17	7240008	7244635	4627	8	27	44.31818182	7.694471724	0.162479511	0.036458649	-	11337	grey	0.116210663	FALSE	GABARAP
Region_7379	chr17	7243591	7252491	8900	10	63	81.72727273	10.21092244	0.407786693	0.025455502	-	23399	grey	-0.319599032	FALSE	CTDNEP1
Region_7380	chr17	7548508	7557890	9382	14	55	79.07575758	10.65300334	0.794494591	0.044305009	+	8742	grey	-0.084558345	FALSE	TNFSF12
Region_7381	chr17	7549099	7561601	12502	16	67	96.84848485	11.50684402	0.721507429	0.044176885	+	407977	grey	-0.095833998	FALSE	TNFSF12-TNFSF13
Region_7382	chr17	7561919	7571969	10050	6	35	47.6969697	7.779597967	0.797997163	0.033447276	+	26168	grey	0.102412828	FALSE	SENP3
Region_7383	chr17	7563287	7578715	15428	10	50	71.37878788	8.796441853	0.71682331	0.042146864	+	100533955	grey	0.153890875	FALSE	SENP3-EIF4A1
Region_7384	chr17	7572825	7582024	9199	6	26	40.03030303	6.440185609	0.546654846	0.065978099	+	1973	grey	0.043772625	FALSE	EIF4A1
Region_7385	chr17	7581964	7584098	2134	5	34	50.42424242	7.144789615	0.100808972	0.05950873	-	100996842	grey	0.052884375	FALSE	MPDU1-AS1
Region_7386	chr17	7583666	7592789	9123	26	154	194.2121212	18.44786012	0.672615889	0.027248368	+	9526	grey	0.001621937	FALSE	MPDU1
Region_7387	chr1	7588178	7590094	1916	13	65	92.6666666	11.4305035	0.62573610	0.04533335	-	6665	grey	-0.01549403	FALSE	SOX15

	7						7		1	6				4		
Region_7388	chr1<br>7	7591230	7614897	23667	16	102	123.227272<br>7	10.4687012<br>7	0.95909055<br>3	0.02220224<br>4	-	9513	grey	0.134722513	FALSE	FXR2
Region_7389	chr1<br>7	7614064	7633382	19318	13	65	84.9848484<br>8	9.37015298<br>2	0.81887135<br>4	0.01925790<br>7	+	6462	grey	0.131963263	FALSE	SHBG
Region_7390	chr1<br>7	7646627	7657770	11143	9	35	49.6363636<br>4	7.30149903<br>2	0.54026902<br>8	0.04484916<br>1	+	482	grey	-0.13516632<br>3	FALSE	ATP1B2
Region_7391	chr1<br>7	7661779	7687538	25759	20	118	143.030303	13.2629845<br>8	0.89086872<br>7	0.02200473<br>7	-	7157	grey	0.145076917	FALSE	TP53
Region_7392	chr1<br>7	7686071	7703502	17431	21	110	138.106060<br>6	13.4851813<br>7	0.84080703<br>2	0.01727878<br>5	+	55135	grey	0.135125691	FALSE	WRAP53
Region_7393	chr1<br>7	7705202	7711372	6170	5	18	36.1212121<br>2	6.33552900<br>2	0.70164202<br>2	0.06775865<br>2	+	1949	grey	0.084588179	FALSE	EFNB3
Region_7394	chr1<br>7	7717744	7833742	115998	93	636	711.621212<br>1	32.1390082<br>4	0.90295577<br>7	0.01285829<br>3	+	146754	grey	0.144789082	FALSE	DNAH2
Region_7395	chr1<br>7	7834217	7854796	20579	28	138	177.045454<br>5	16.3551279<br>4	0.18316909<br>4	0.01454952<br>1	+	23135	grey	0.110488495	FALSE	KDM6B
Region_7396	chr1<br>7	7855066	7856099	1033	5	15	41.4393939<br>4	11.1301790<br>5	0.90879576<br>8	0.05952478	+	92162	grey	0.165057845	FALSE	TMEM88
Region_7397	chr1<br>7	7856685	7885238	28553	7	40	62.4090909<br>1	8.01532622<br>8	0.29551605<br>7	0.05061552<br>4	-	84316	grey	-0.03655013<br>6	FALSE	NAA38
Region_7398	chr1<br>7	7884796	7912760	27964	22	118	150.954545<br>5	11.8962590<br>3	0.68842943<br>8	0.02053967	+	1107	grey	0.083154422	FALSE	CHD3
Region_7399	chr1<br>7	7913339	7916289	2950	8	33	47.7878787<br>9	8.27138747<br>8	0.00304683<br>7	0.00909683<br>7	-	284023	grey	-0.09973192	FALSE	RNF227

Region_7400	chr17	7932101	7949920	17819	7	29	43.46969697	6.942000813	0.713138971	0.038047367	+	116840	grey	-0.057842889	FALSE	CNTROB
Region_7401	chr17	8002615	8020342	17727	17	96	129.2424242	12.3861717	0.648320123	0.029391917	+	3000	grey	0.166266177	FALSE	GUCY2D
Region_7402	chr17	8039034	8049134	10100	6	30	47.33333333	7.060907184	0.458636647	0.064226324	+	247	grey	-0.101794841	FALSE	ALOX15B
Region_7403	chr17	8072636	8087716	15080	32	181	239.1969697	24.40883806	0.278485712	0.017966368	-	242	grey	-0.059571876	FALSE	ALOX12B
Region_7404	chr17	8079482	8081565	2083	15	58	99.60606061	15.56512748	0.135755911	0.020732689	+	107985075	grey	0.001654155	FALSE	LOC107985075
Region_7405	chr17	8095900	8119047	23147	29	198	233.3787879	19.39846556	0.63599688	0.029302878	-	59344	grey	0.086918448	FALSE	ALOXE3
Region_7406	chr17	8120592	8124106	3514	8	27	42.48484848	7.239299635	0.008438943	0.02050297	-	84667	grey	-0.016958269	FALSE	HES7
Region_7407	chr17	8140472	8156506	16034	27	147	175.2727273	15.36383912	0.398836774	0.017826342	-	5187	grey	-0.106640312	FALSE	PER1
Region_7408	chr17	8150816	8163546	12730	27	141	173.8636364	14.94285151	0.191897127	0.016154745	-	6844	grey	0.064597389	FALSE	VAMP2
Region_7409	chr17	8172457	8176399	3942	3	17	26.87878788	5.178402342	0.636243255	0.038798836	-	84314	grey	0.09122911	FALSE	TMEM107
Region_7410	chr17	8188345	8190180	1835	3	13	23.18181818	4.973496188	0.002188552	0.010353614	-	54785	grey	-0.013412156	FALSE	BORCS6
Region_7411	chr17	8204733	8210600	5867	5	21	32.89393939	5.075215154	0.783249552	0.027061073	-	9212	grey	0.057281776	FALSE	AURKB
Region_7412	chr1	8220624	8224043	3419	7	37	60.5606060	8.59360905	0.00285510	0.00631094	-	284029	grey	-0.30343419	FALSE	LINC00324

	7						6	3	5	3				7		
Region_7413	chr1<br>7	8224815	8248058	23243	13	77	97.6363636<br>4	9.78167260<br>1	0.80922852<br>6	0.03060195<br>4	-	80169	grey	0.105003776	FALSE	CTC1
Region_7414	chr1<br>7	8247618	8270491	22873	11	67	89.3333333<br>3	10.3065824<br>1	0.70454879<br>5	0.01900107<br>6	+	5198	grey	-0.00828096<br>3	FALSE	PFAS
Region_7415	chr1<br>7	8287763	8295400	7637	12	70	95.8030303	10.9322665<br>6	0.40354128<br>2	0.02010645<br>6	-	399512	grey	0.066992882	FALSE	SLC25A35
Region_7416	chr1<br>7	8288654	8290092	1438	7	36	51.8181818<br>2	7.80908561<br>8	0.27902124<br>3	0.02719871<br>4	+	29098	grey	0.157086679	FALSE	RANGRF
Region_7417	chr1<br>7	8310241	8322514	12273	11	49	73.8787878<br>8	9.76416549<br>1	0.75158969<br>8	0.04574479	+	22899	grey	0.044676385	FALSE	ARHGEF15
Region_7418	chr1<br>7	8356902	8383187	26285	20	125	156	13.6156019<br>1	0.76838891<br>8	0.03136403<br>8	-	124751	grey	0.163284812	FALSE	KRBA2
Region_7419	chr1<br>7	8377516	8383213	5697	5	30	46.3787878<br>8	6.95870269	0.58719600<br>6	0.06224248<br>9	-	6154	grey	0.064257257	FALSE	RPL26
Region_7420	chr1<br>7	8390702	8397827	7125	3	12	22.5757575<br>8	4.65439612<br>2	0.93313918<br>1	0.05667264<br>3	-	643904	grey	0.054885371	FALSE	RNF222
Region_7421	chr1<br>7	8413131	8490411	77280	28	174	210.924242<br>4	15.4255638	0.89676623<br>9	0.01337586<br>7	+	81565	grey	-0.09292703	FALSE	NDEL1
Region_7422	chr1<br>7	8474207	8631376	157169	52	339	382.621212<br>1	19.4989271<br>1	0.86837249<br>1	0.01343537<br>8	-	4628	grey	-0.12742292<br>2	FALSE	MYH10
Region_7423	chr1<br>7	8729935	8744836	14901	19	99	127.727272<br>7	12.8357019<br>6	0.81460392	0.04626932<br>8	-	146849	grey	0.177215024	FALSE	CCDC42
Region_7424	chr1<br>7	8747524	8758559	11035	3	17	26.1060606<br>1	4.96178872<br>2	0.90521917<br>6	0.06825672<br>4	-	388333	grey	0.016198226	FALSE	SPDYE4

Region_7425	chr17	8802722	8867677	64955	16	98	129.3787879	12.40442248	0.884073313	0.027858547	-	146850	grey	-0.254607357	FALSE	PIK3R6
Region_7426	chr17	8878911	8965712	86801	31	174	214.1666667	16.79430612	0.906020373	0.029087602	-	23533	grey	-0.250004975	FALSE	PIK3R5
Region_7427	chr17	9021510	9244000	222490	130	889	1003.393939	42.74355688	0.824892145	0.012617939	+	9423	grey	-0.255047409	FALSE	NTN1
Region_7428	chr17	9171068	9179118	8050	8	37	54.53030303	6.986183501	0.89370597	0.050911232	-	101928266	grey	-0.121087617	FALSE	LOC101928266
Region_7429	chr17	9250471	9576591	326120	121	842	931.530303	38.62212188	0.913970459	0.010225845	-	9482	grey	0.188709274	FALSE	STX8
Region_7430	chr17	9576627	9643447	66820	20	141	168.030303	12.71216219	0.837214363	0.038383136	+	146845	grey	-0.074753889	FALSE	CFAP52
Region_7431	chr17	9644698	9729687	84989	39	258	311.9848485	16.44898166	0.839569557	0.021512024	+	124739	grey	-0.12669882	FALSE	USP43
Region_7432	chr17	9771434	9791592	20158	12	83	106.3333333	10.11371245	0.87616928	0.037242094	-	201140	grey	0.093390356	FALSE	DHRS7C
Region_7433	chr17	9791227	9822079	30852	13	76	95.1969697	10.48841074	0.707514103	0.062345707	-	644070	grey	-0.136156288	FALSE	GSG1L2
Region_7434	chr17	9822206	9892099	69893	28	203	244.6363636	15.06767948	0.890996966	0.028551673	+	9340	grey	-0.117852499	FALSE	GLP2R
Region_7435	chr17	9910606	10198606	288000	145	1087	1163.060606	35.02301608	0.834746253	0.012486551	-	8522	grey	-0.129055479	FALSE	GAS7
Region_7436	chr17	10300865	10373130	72265	16	99	129.1363636	11.15331805	0.75997297	0.049899704	-	8735	grey	0.397980733	FALSE	MYH13
Region_7437	chr1	10320392	10341458	21066	5	29	44.9848484	7.51305003	0.76049107	0.06977445	+	10798500	grey	0.326168939	FALSE	LOC107985004

	7						8		7	3		4				
Region_7438	chr17	10383132	10623886	240754	37	243	291.5757576	19.16973625	0.860089769	0.026317023	+	100128560	grey	-0.04462275	FALSE	MYHAS
Region_7439	chr17	10390322	10421950	31628	4	21	32.37878788	5.185082602	0.93887342	0.060075849	-	4626	grey	0.144913044	FALSE	MYH8
Region_7440	chr17	10492307	10518542	26235	4	15	24.22727273	4.938998511	0.890014791	0.071422282	-	4619	grey	0.013501788	FALSE	MYH1
Region_7441	chr17	10521148	10549700	28552	8	37	62.01515152	7.614747684	0.867953663	0.048225193	-	4620	grey	0.097679273	FALSE	MYH2
Region_7442	chr17	10628526	10657309	28783	15	94	116.5	11.0832514	0.920972349	0.025699205	-	4621	grey	-0.031249607	FALSE	MYH3
Region_7443	chr17	10672474	10698375	25901	9	60	75.10606061	9.655813375	0.831018772	0.029073153	-	6341	grey	-0.148693055	FALSE	SCO1
Region_7444	chr17	10699015	10730023	31008	9	50	68.56060606	10.07529925	0.773728977	0.036219263	-	388335	grey	-0.196696014	FALSE	TMEM220
Region_7445	chr17	10729777	10880895	151118	52	374	408.8636364	19.83073478	0.86095311	0.017362283	+	101101775	grey	-0.282276939	FALSE	TMEM220-AS1
Region_7446	chr17	10794906	10838094	43188	8	37	55.84848485	7.94644076	0.83401395	0.057943523	-	100289255	grey	-0.294851163	FALSE	TMEM238L
Region_7447	chr17	11241213	11564063	322850	66	494	550.8636364	24.18192991	0.846868559	0.019892793	+	388336	grey	0.086404956	FALSE	SHISA6
Region_7448	chr17	11598470	11969748	371278	91	628	708.5909091	34.41286757	0.863413088	0.016228692	+	1770	grey	0.157270518	FALSE	DNAH9
Region_7449	chr17	11953889	12021508	67619	14	81	100.8484848	9.130241254	0.800553043	0.028990278	-	7566	grey	-0.010437455	FALSE	ZNF18

Region_7450	chr17	12020829	12143830	123001	13	105	128	10.96147098	0.901957405	0.022066772	+	6416	grey	0.09633767	FALSE	MAP2K4
Region_7451	chr17	12549764	12642854	93090	23	174	206.9393939	13.79395496	0.772319437	0.033895221	+	284034	grey	-0.085823754	FALSE	LINC00670
Region_7452	chr17	12596743	12632959	36216	9	46	62.53030303	8.335687313	0.830443915	0.05254338	-	105371540	grey	-0.218604102	FALSE	LOC105371540
Region_7453	chr17	12665890	12768949	103059	21	131	169.9545455	14.60709187	0.856216316	0.030612783	+	93649	grey	0.023515704	FALSE	MYOCD
Region_7454	chr17	12671862	12706135	34273	7	42	58.93939394	7.105531513	0.870814546	0.050062066	-	101928418	grey	-0.059514148	FALSE	MYOCD-AS1
Region_7455	chr17	12760140	12790242	30102	4	14	23.18181818	4.657199855	0.627382713	0.070107051	-	100128006	grey	0.055976065	FALSE	ARHGAP44-AS1
Region_7456	chr17	12789498	12991643	202145	62	475	533.7727273	28.69725018	0.869665847	0.017693203	+	9912	grey	0.02026334	FALSE	ARHGAP44
Region_7457	chr17	12991612	13018065	26453	15	100	126.3787879	12.97188101	0.646441046	0.032585394	-	60528	grey	0.002640969	FALSE	ELAC2
Region_7458	chr17	13494032	13601929	107897	19	132	165.0757576	14.99262445	0.821589522	0.020312633	-	9955	grey	0.009963206	FALSE	HS3ST3A1
Region_7459	chr17	13755574	14069492	313918	48	363	397.6515152	19.90476744	0.859995919	0.024874258	-	100874058	grey	-0.279717393	FALSE	COX10-DT
Region_7460	chr17	13776373	14069489	293116	48	363	397.6515152	19.90476744	0.859995919	0.024874258	-	107985014	grey	-0.279717393	FALSE	LOC107985014
Region_7461	chr17	13777307	14069495	292188	47	354	390.0757576	19.2132251	0.858899972	0.025108584	-	100506974	grey	-0.268816626	FALSE	LOC100506974
Region_7462	chr1	14069490	14231736	162246	33	213	262.348484	18.2347870	0.80848524	0.02100547	+	1352	grey	-0.28453906	FALSE	COX10

	7						8	6	5	1				8		
Region_7463	chr17	14301081	14349404	48323	27	154	189.6969697	16.59283413	0.69629746	0.027675013	+	9953	grey	-0.275165649	FALSE	HS3ST3B1
Region_7464	chr17	14374139	14526498	152359	26	172	202.0606061	13.4013533	0.924101789	0.025923094	+	107985080	grey	0.051212101	FALSE	LOC107985080
Region_7465	chr17	14834557	14957216	122659	15	96	126.7727273	11.48483342	0.89466205	0.039443332	+	107984977	grey	-0.087636706	FALSE	LINC02096
Region_7466	chr17	15229773	15272292	42519	8	56	78.65151515	8.158982637	0.551731381	0.037809481	-	5376	grey	-0.005385361	FALSE	PMP22
Region_7467	chr17	15303811	15341632	37821	7	52	69.96969697	8.030652233	0.885635786	0.038464907	-	64518	grey	-0.030755679	FALSE	TEKT3
Region_7468	chr17	15436015	15503608	67593	16	102	135.7727273	11.87861097	0.81500024	0.023132157	-	284040	grey	-0.212000667	FALSE	CDRT4
Region_7469	chr17	15436021	15563561	127540	40	295	340.9545455	22.58211124	0.844282627	0.019900435	-	100533496	grey	-0.366322599	FALSE	TVP23C-CDRT4
Region_7470	chr17	15502264	15563595	61331	26	179	217.9393939	17.6616741	0.825302603	0.026923138	-	201158	grey	-0.353793989	FALSE	TVP23C
Region_7471	chr17	15565484	15651653	86169	25	133	174.6818182	18.21593477	0.796065672	0.027518148	-	374286	grey	-0.109858299	FALSE	FBXW10B
Region_7472	chr17	15627960	15684311	56351	9	48	66.66666667	7.712793235	0.869575809	0.024143194	-	10626	grey	0.217754999	FALSE	TRIM16
Region_7473	chr17	15699552	15746162	46610	19	109	134.8030303	12.53821199	0.904407595	0.021137437	+	353149	grey	0.175637402	FALSE	TBC1D26
Region_7474	chr17	15699577	15720787	21210	6	23	36.5	6.271424817	0.963524565	0.033186423	+	57335	grey	0.144078774	FALSE	ZNF286A

Region_7475	chr17	15699577	15744778	45201	18	96	118.4090909	11.71189701	0.955040019	0.022030575	+	119086082	grey	0.206220951	FALSE	ZNF286A-TBC1D26
Region_7476	chr17	15735023	15743799	8776	5	22	38.8030303	6.750999235	0.930645343	0.046525203	-	105371560	grey	0.117570001	FALSE	TBC1D26-AS1
Region_7477	chr17	15806241	15818077	11836	3	12	23.40909091	4.829324758	0.952467384	0.044126004	-	101928567	grey	0.263340791	FALSE	LINC02087
Region_7478	chr17	15806390	15847996	41606	11	66	89.81818182	10.05968204	0.833113197	0.03475463	-	105371557	grey	0.067554536	FALSE	LOC105371557
Region_7479	chr17	15945130	15975746	30616	8	57	74.72727273	10.99306838	0.43128054	0.036015707	+	136	grey	-0.120126796	FALSE	ADORA2B
Region_7480	chr17	15976560	15999717	23157	11	64	85.78787879	8.982915668	0.677751103	0.037177262	-	125150	grey	0.17067411	FALSE	ZSWIM7
Region_7481	chr17	15999784	16045015	45231	12	51	75.24242424	8.279837617	0.890630219	0.016989874	+	54902	grey	-0.028503301	FALSE	TTC19
Region_7482	chr17	16029157	16218185	189028	58	402	459.7878788	28.14004273	0.715239838	0.01378238	-	9611	grey	-0.283909607	FALSE	NCOR1
Region_7483	chr17	16217191	16348801	131610	59	345	414.1969697	21.54652904	0.871501239	0.010335728	+	9487	grey	-0.150477798	FALSE	PIGL
Region_7484	chr17	16342534	16353656	11122	8	43	55.92424242	6.613065404	0.250136357	0.025945276	-	201161	grey	-0.061842893	FALSE	CENPV
Region_7485	chr17	16415571	16437003	21432	12	72	93.43939394	10.72040296	0.828994879	0.030899892	+	51393	grey	-0.155865328	FALSE	TRPV2
Region_7486	chr17	16438767	16480249	41482	30	213	256.0757576	17.78530993	0.547131424	0.01693658	+	125144	grey	0.055937361	FALSE	SNHG29
Region_7487	chr1	16441577	16492193	50616	25	147	180.651515	13.7533128	0.77578112	0.03068080	-	388341	grey	-0.12826080	FALSE	LRRC75A

	7						2		9	7				5		
Region_7488	chr17	16546954	16569204	22250	5	39	52.74242424	7.498329262	0.961823744	0.024846081	-	57336	grey	0.033043109	FALSE	ZNF287
Region_7489	chr17	16689537	16804453	114916	29	170	246.9545455	41.85852797	0.889780736	0.018779722	+	9720	grey	-0.277456463	FALSE	CCDC144A
Region_7490	chr17	16786489	16816540	30051	10	30	87.53030303	28.54158623	0.919878891	0.041140371	+	162632	grey	-0.29697586	FALSE	USP32P1
Region_7491	chr17	16922915	16934839	11924	8	46	65.42424242	7.682268507	0.935328062	0.032309974	-	96597	grey	-0.315689747	FALSE	TBC1D27P
Region_7492	chr17	16929816	16972118	42302	23	131	172.5606061	13.54323991	0.79188177	0.040266022	-	23495	grey	0.205231294	FALSE	TNFRSF13B
Region_7493	chr17	16975999	16981353	5354	4	11	28.21212121	6.801617797	0.948197454	0.049623159	-	284191	grey	-0.049612076	FALSE	LOC284191
Region_7494	chr17	17042457	17217679	175222	133	987	1068.424242	42.49997669	0.814805606	0.013905516	+	23164	grey	-0.118909641	FALSE	MPRIP
Region_7495	chr17	17200995	17206333	5338	13	84	105.1969697	10.43104788	0.493897312	0.053269629	-	201164	grey	-0.120161968	FALSE	PLD6
Region_7496	chr17	17202649	17237188	34539	48	282	331.5151515	21.59433147	0.740450099	0.030650714	-	201163	grey	-0.326146955	FALSE	FLCN
Region_7497	chr17	17246616	17281273	34657	29	187	220.6212121	15.83935231	0.722713893	0.021007021	-	8533	grey	-0.028973231	FALSE	COPS3
Region_7498	chr17	17303335	17347663	44328	23	145	169.7272727	13.64894524	0.864285926	0.015961851	+	56953	grey	0.073878067	FALSE	NT5M
Region_7499	chr17	17476994	17493221	16227	17	101	123.4545455	12.96288408	0.784955363	0.019907097	+	55090	grey	0.085390316	FALSE	MED9

Region_7500	chr17	17494437	17496395	1958	24	146	195.1363636	21.37423781	0.19080312	0.030792279	-	51655	grey	0.092654158	FALSE	RASD1
Region_7501	chr17	17505563	17591708	86145	68	451	520.6212121	33.20878714	0.91767024	0.013190757	-	10400	grey	0.202510169	FALSE	PEMT
Region_7502	chr17	17681458	17811453	129995	100	627	700.2575758	37.19111168	0.643419083	0.020233837	+	10743	grey	-0.029976315	FALSE	RAI1
Region_7503	chr17	17759154	17770821	11667	6	29	42.06060606	6.258234668	0.882710361	0.04280266	-	100861516	grey	-0.189096939	FALSE	RAI1-AS1
Region_7504	chr17	17810399	17837002	26603	22	126	155.6969697	14.71364589	0.791520282	0.023507573	-	6720	grey	0.242139302	FALSE	SREBF1
Region_7505	chr17	17843511	17972422	128911	43	324	367.5454545	21.52221165	0.904304892	0.018752622	-	146691	grey	-0.247771517	FALSE	TOM1L2
Region_7506	chr17	17972813	18016889	44076	26	186	219.4393939	13.82542673	0.904443882	0.030964669	+	83450	grey	-0.142076058	FALSE	DRC3
Region_7507	chr17	17977409	18039168	61759	34	237	271.530303	15.09377834	0.919077334	0.023434422	-	107984989	grey	-0.112108122	FALSE	LOC107984989
Region_7508	chr17	18015059	18039209	24150	9	40	58.25757576	7.034662763	0.965326951	0.029546913	-	91647	grey	0.120802294	FALSE	ATPAF2
Region_7509	chr17	18039408	18068405	28997	16	81	116	11.8178483	0.668018692	0.033031385	+	79018	grey	0.259921174	FALSE	GID4
Region_7510	chr17	18087892	18107970	20078	15	91	114.9242424	12.8018155	0.489767757	0.025544991	+	1819	grey	0.030706885	FALSE	DRG2
Region_7511	chr17	18108756	18179802	71046	57	338	419.1363636	24.07481056	0.826166239	0.019521034	+	51168	grey	-0.15439923	FALSE	MYO15A
Region_7512	chr1	18183078	18209954	26876	20	125	158.515151	15.9530488	0.72571308	0.02494386	+	54890	grey	-0.18794686	FALSE	ALKBH5

	7						5	3	1	8				4		
Region_7513	chr17	18225635	18244875	19240	23	129	162.4848485	16.60142943	0.900858596	0.024342725	+	3996	grey	-0.094864248	FALSE	LLGL1
Region_7514	chr17	18517382	18625653	108271	13	67	84.1969697	8.826916348	0.751686824	0.049811464	-	284047	grey	0.316054505	FALSE	CCDC144BP
Region_7515	chr17	18634247	18661950	27703	11	56	85.43939394	10.35096248	0.725800666	0.045382756	-	254272	grey	-0.260858278	FALSE	TBC1D28
Region_7516	chr17	18658429	18682241	23812	14	91	122.1666667	12.62303552	0.95624414	0.019089197	-	729288	grey	0.02756199	FALSE	ZNF286B
Region_7517	chr17	18667629	18682314	14685	9	46	76.36363636	11.92623013	0.969138438	0.01884226	-	2310	grey	0.010929828	FALSE	FOXO3B
Region_7518	chr17	18697998	18736118	38120	7	36	57.10606061	8.581068228	0.730457873	0.047228863	+	147166	grey	-0.355590767	FALSE	TRIM16L
Region_7519	chr17	18744026	18779349	35323	22	139	170.5757576	13.32442593	0.88610227	0.036025977	+	10517	grey	0.165216634	FALSE	FBXW10
Region_7520	chr17	18781111	18806714	25603	6	43	58.34848485	7.156476194	0.804426298	0.027015352	+	51030	grey	0.017720276	FALSE	TVP23B
Region_7521	chr17	18840085	18931287	91202	46	299	354.1212121	20.41682199	0.828589953	0.02707049	+	5636	grey	-0.02290012	FALSE	PRPSAP2
Region_7522	chr17	18950345	19022595	72250	72	486	550.4242424	25.04031249	0.805274522	0.016256679	+	125206	grey	0.063894377	FALSE	SLC5A10
Region_7523	chr17	18968789	19004764	35975	41	261	309.3939394	19.74208997	0.750901731	0.02392356	-	644815	grey	0.027522805	FALSE	FAM83G
Region_7524	chr17	19020656	19047011	26355	5	20	34.09090909	6.713310023	0.809261832	0.085089704	-	10750	grey	0.297020193	FALSE	GRAP

Region_7525	chr17	19215615	19336715	121100	46	340	394.469697	25.20269808	0.844543518	0.016820939	+	22905	grey	-0.07034102	FALSE	EPN2
Region_7526	chr17	19237088	19274388	37300	22	131	171.4090909	18.19042628	0.880086675	0.025754743	+	100874309	grey	-0.111101336	FALSE	EPN2-IT1
Region_7527	chr17	19296596	19306261	9665	3	14	26.15151515	5.622051157	0.734663395	0.072488541	-	100874018	grey	-0.102907951	FALSE	EPN2-AS1
Region_7528	chr17	19334695	19378193	43498	19	141	164.3787879	13.65595407	0.800050251	0.019537937	-	27077	grey	-0.140353228	FALSE	B9D1
Region_7529	chr17	19362760	19370236	7476	4	20	32.96969697	7.310623875	0.699887902	0.046813403	+	105371573	grey	-0.052514295	FALSE	LOC105371573
Region_7530	chr17	19377721	19383544	5823	8	45	67.25757576	10.45180972	0.244019652	0.036285289	+	5598	grey	0.045734938	FALSE	MAPK7
Region_7531	chr17	19411125	19417276	6151	5	17	28.3030303	4.80054387	0.857566112	0.082819243	+	7732	grey	0.208659426	FALSE	RNF112
Region_7532	chr17	19417769	19461020	43251	20	123	158.4090909	12.55263046	0.885749381	0.024640718	-	105371574	grey	-0.147181589	FALSE	LOC105371574
Region_7533	chr17	19495385	19579034	83649	26	157	186.7424242	14.86483781	0.819414284	0.026380129	+	55244	grey	-0.052319794	FALSE	SLC47A1
Region_7534	chr17	19560111	19597922	37811	11	52	76.42424242	9.580526091	0.831788217	0.031827549	-	105371578	grey	0.409214817	FALSE	LOC105371578
Region_7535	chr17	19615789	19633825	18036	3	14	25.31818182	5.613607344	0.966367116	0.033140979	+	112451549	grey	-0.125922919	FALSE	SLC47A1P2
Region_7536	chr17	19648136	19685760	37624	21	146	190.3484848	17.99785665	0.509879082	0.012190527	+	224	grey	-0.032026567	FALSE	ALDH3A2
Region_7537	chr1	19678288	19718979	40691	12	78	99.3333333	10.4562580	0.90556590	0.02279342	-	146802	grey	0.035074656	FALSE	SLC47A2

	7						3	9	5	6						
Region_7538	chr17	19737984	19748943	10959	5	21	36.21212121	5.307513257	0.748994339	0.072153594	-	218	grey	0.019543229	FALSE	ALDH3A1
Region_7539	chr17	19770830	19867936	97106	28	186	223.5151515	16.2312542	0.888661412	0.015334308	-	9706	grey	0.048656783	FALSE	ULK2
Region_7540	chr17	19904302	19978343	74041	26	170	207.3939394	15.16562793	0.831449058	0.018896765	-	11216	grey	-0.209690137	FALSE	AKAP10
Region_7541	chr17	20008865	20319026	310161	57	419	482.6666667	23.81036189	0.859560417	0.01075026	+	92521	grey	0.102214219	FALSE	SPECC1
Region_7542	chr17	20321150	20403557	82407	21	105	131.6060606	10.67257995	0.90900337	0.029279613	+	348254	grey	-0.172093926	FALSE	CCDC144CP
Region_7543	chr17	20408311	20447224	38913	14	72	101.1818182	11.10912181	0.802911134	0.038485425	+	347716	grey	0.014345759	FALSE	USP32P3
Region_7544	chr17	20436337	20447249	10912	5	30	42.90909091	6.840439196	0.910459513	0.061795392	+	339256	grey	0.196863213	FALSE	NOS2P3
Region_7545	chr17	20449395	20467539	18144	7	27	43.5	8.74114937	0.750909898	0.055630145	-	284194	grey	0.036565101	FALSE	LGALS9B
Region_7546	chr17	20612912	20633234	20322	3	15	24.27272727	5.079507713	0.908372579	0.071824984	-	105371582	grey	0.135264593	FALSE	LINC02088
Region_7547	chr17	20716481	20737127	20646	4	20	33.18181818	6.258309162	0.944232649	0.041379841	+	100287072	grey	-0.132071005	FALSE	LOC100287072
Region_7548	chr17	20836447	20896162	59715	18	89	126.0606061	13.07008185	0.65222461	0.040237703	-	339184	grey	-0.111769024	FALSE	CCDC144NL
Region_7549	chr17	20868433	20933416	64983	20	127	163.1060606	13.45091222	0.8880797	0.03168301	+	440416	grey	-0.11730259	FALSE	CCDC144NL-AS1

Region_7550	chr17	20868497	21002276	133779	41	283	323.7575758	17.1114542	0.907234074	0.019168972	+	339260	grey	-0.068779847	FALSE	LOC339260
Region_7551	chr17	20999596	21043760	44164	36	255	291.8939394	17.50967099	0.72277073	0.017596066	-	23326	grey	-0.250148479	FALSE	USP22
Region_7552	chr17	21075362	21092351	16989	6	28	46.24242424	6.983630544	0.862083776	0.067748025	+	101060544	grey	0.056486671	FALSE	LINC01563
Region_7553	chr17	21123364	21193265	69901	35	251	285.0909091	16.55640919	0.878604968	0.017106438	+	25979	grey	-0.186160634	FALSE	DHRS7B
Region_7554	chr17	21197954	21214176	16222	20	142	171.5454545	13.73281507	0.765204728	0.023533424	-	8834	grey	0.117241323	FALSE	TMEM11
Region_7555	chr17	21214322	21230035	15713	20	121	160.7878788	14.74972245	0.480889858	0.027445276	+	107984969	grey	0.101064645	FALSE	TMEM11-DT
Region_7556	chr17	21238870	21253410	14540	11	64	85.16666667	9.181223872	0.850188632	0.029054037	-	256302	grey	-0.036702409	FALSE	NATD1
Region_7557	chr17	21284672	21315232	30560	16	141	169.3636364	14.53762901	0.775056642	0.031541283	+	5606	grey	0.015998364	FALSE	MAP2K3
Region_7558	chr17	21376357	21419870	43513	25	180	225.1666667	20.16057335	0.781208994	0.031449553	+	3768	grey	0.184281342	FALSE	KCNJ12
Region_7559	chr17	21428263	21574517	146254	29	181	239.6212121	18.08932125	0.71897912	0.027528892	-	339263	grey	-0.3823183	FALSE	LINC02693
Region_7560	chr17	21692523	21704612	12089	8	31	67.86363636	15.73031015	0.864048156	0.100292515	+	100134444	grey	0.191286089	FALSE	KCNJ18
Region_7561	chr17	22090743	22205156	114413	18	118	144.5606061	12.7474025	0.790052085	0.04660051	+	23666	grey	0.288391605	FALSE	UBBP4
Region_7562	chr1	22233926	22266392	32466	4	27	37.0606060	6.28032039	0.69575975	0.18064896	-	10537159	grey	-0.40440208	FALSE	LINC02002

	7						6	3	2	8		5		9		
Region_7563	chr17	27294076	27315926	21850	3	12	19.63636364	4.288217717	0.950456768	0.059733041	+	26118	grey	0.167600046	FALSE	WSB1
Region_7564	chr17	27417690	27431623	13933	4	17	25.90909091	5.028311455	0.956616652	0.049662307	+	440419	grey	0.01771719	FALSE	TBC1D3P5
Region_7565	chr17	27456448	27626438	169990	70	574	625.6666667	26.00078894	0.850658186	0.016324438	+	8844	grey	0.276295152	FALSE	KSR1
Region_7566	chr17	27623364	27649560	26196	5	20	34.33333333	5.876506899	0.790912141	0.043661764	+	3965	grey	0.002858325	FALSE	LGALS9
Region_7567	chr17	27756766	27800529	43763	14	90	118.8333333	11.33080666	0.852008221	0.036589394	-	4843	grey	-0.083669209	FALSE	NOS2
Region_7568	chr17	27798806	27893383	94577	33	247	293.0757576	19.50487955	0.618327532	0.018213087	-	201229	grey	-0.275591246	FALSE	LYRM9
Region_7569	chr17	27928924	27991787	62863	28	207	240.7424242	18.11358233	0.860423211	0.026318058	-	105371708	grey	-0.062339037	FALSE	LINC01992
Region_7570	chr17	28042677	28196381	153704	18	119	143.7272727	11.8354688	0.917196813	0.023202235	+	51701	grey	-0.019827657	FALSE	NLK
Region_7571	chr17	28275986	28317783	41797	5	17	30.95454545	5.333296911	0.930176266	0.04938214	-	284085	grey	0.059082836	FALSE	KRT18P55
Region_7572	chr17	28335602	28347009	11407	6	34	47.56060606	6.164735412	0.957421961	0.03504724	+	7126	grey	-0.173760952	FALSE	TNFAIP1
Region_7573	chr17	28346633	28357527	10894	6	17	35.8030303	6.497856918	0.641974362	0.027839724	-	26073	grey	0.306766721	FALSE	POLDIP2
Region_7574	chr17	28357642	28381697	24055	10	68	85.87878788	9.347175882	0.560610993	0.024942663	+	147007	grey	-0.096766702	FALSE	TMEM199

Region_7575	chr17	28364356	28404049	39693	11	72	95.40909091	9.601245549	0.577090033	0.028119997	+	23098	grey	-0.078896077	FALSE	SARM1
Region_7576	chr17	28367284	28373091	5807	8	52	72.5	8.341278264	0.45861917	0.027633877	-	7448	grey	-0.169233435	FALSE	VTN
Region_7577	chr17	28455752	28614197	158445	46	307	355.7424242	22.73276903	0.82993175	0.015347562	-	124923	grey	-0.004102318	FALSE	RSKR
Region_7578	chr17	28473293	28497781	24488	5	21	34.31818182	7.522599982	0.885904185	0.057387547	+	9058	grey	0.061970397	FALSE	SLC13A2
Region_7579	chr17	28506243	28538900	32657	4	16	28.60606061	5.716047015	0.881411018	0.069846925	+	8456	grey	0.14030587	FALSE	FOXN1
Region_7580	chr17	28553383	28571794	18411	12	59	95.81818182	11.14092814	0.733807533	0.019205443	-	94005	grey	-0.076496589	FALSE	PIGS
Region_7581	chr17	28577565	28599025	21460	7	48	67.96969697	9.314950261	0.939394368	0.028342701	-	10615	grey	0.088159106	FALSE	SPAG5
Region_7582	chr17	28598790	28617377	18587	5	30	40.24242424	5.001724644	0.764490028	0.032734542	+	100506436	grey	-0.16926769	FALSE	SPAG5-AS1
Region_7583	chr17	28614446	28645454	31008	4	18	30.74242424	5.594806949	0.976606567	0.029944329	-	9703	grey	0.123336247	FALSE	BLTP2
Region_7584	chr17	28648346	28662189	13843	6	30	48.81818182	8.133329512	0.490216577	0.02365747	-	6388	grey	-0.049107996	FALSE	SDF2
Region_7585	chr17	28662198	28702679	40481	9	51	69.04545455	8.533070181	0.834119805	0.035214878	+	6830	grey	0.120963966	FALSE	SUPT6H
Region_7586	chr17	28703197	28711888	8691	10	37	58.56060606	8.067272512	0.841538531	0.038914403	-	147011	grey	-0.34475376	FALSE	PROCA1
Region_7587	chr1	28714281	28718429	4148	4	15	24.0606060	4.27831306	0.5180908	0.05214163	-	83871	grey	0.037913249	FALSE	RAB34

	7						6	8		4						
Region_7588	chr17	28725897	28743455	17558	7	31	48.37878788	6.487301564	0.180263003	0.028976556	+	284086	grey	-0.036725073	FALSE	NEK8
Region_7589	chr17	28755980	28855232	99252	38	242	282.4242424	17.72530621	0.922447005	0.017072635	-	55731	grey	-0.17061317	FALSE	FAM222B
Region_7590	chr17	28879335	28897733	18398	9	55	76.90909091	9.137795177	0.952649346	0.028403623	-	2319	grey	-0.116756941	FALSE	FLOT2
Region_7591	chr17	28897781	28903079	5298	8	39	55.53030303	7.175213097	0.366288361	0.018227018	-	147015	grey	0.044281382	FALSE	DHRS13
Region_7592	chr17	28905250	28951771	46521	12	77	95.78787879	8.145815635	0.583520302	0.033301676	-	57649	grey	0.177243962	FALSE	PHF12
Region_7593	chr17	28926275	28944749	18474	5	25	43.89393939	7.760112584	0.821618305	0.052642262	+	101927018	grey	0.146604572	FALSE	LOC101927018
Region_7594	chr17	28950513	29057216	106703	69	470	523.4242424	22.02170999	0.683096987	0.016639734	+	51268	grey	-0.042537582	FALSE	PIPOX
Region_7595	chr17	28954901	29006440	51539	28	173	202.6515152	15.38879955	0.669877245	0.02696565	-	124925	grey	-0.127630483	FALSE	SEZ6
Region_7596	chr17	29071122	29180398	109276	63	366	438.6212121	28.36724284	0.889786317	0.016205204	-	399687	grey	-0.004427762	FALSE	MYO18A
Region_7597	chr17	29255839	29294148	38309	9	54	73.33333333	9.534283618	0.67692561	0.030514482	-	57532	grey	-0.102819041	FALSE	NUFIP2
Region_7598	chr17	29390363	29551903	161540	38	235	269.6969697	16.44475104	0.899998496	0.012856258	+	57551	grey	-0.185256027	FALSE	TAOK1
Region_7599	chr17	29560547	29567037	6490	3	14	27.53030303	5.020633417	0.277543626	0.047147025	-	116236	grey	-0.290646277	FALSE	ABHD15

Region_7600	chr17	29566052	29573157	7105	4	17	29.63636364	5.584694368	0.068054094	0.066728296	+	90313	grey	0.061195586	FALSE	TP53I13
Region_7601	chr17	29573475	29594054	20579	8	40	62.59090909	9.275239616	0.376105423	0.023016758	-	28964	grey	-0.03071715	FALSE	GIT1
Region_7602	chr17	29589769	29614761	24992	13	75	100.1212121	11.10580604	0.587137533	0.021947166	+	124930	grey	-0.078932121	FALSE	ANKRD13B
Region_7603	chr17	29625938	29930276	304338	75	538	599.8939394	26.40928088	0.86608915	0.011625411	-	85464	grey	-0.163353638	FALSE	SSH2
Region_7604	chr17	29929200	30108452	179252	27	179	211.2121212	14.67338265	0.794439101	0.020043905	+	374786	grey	0.100864328	FALSE	EFCAB5
Region_7605	chr17	30115521	30186475	70954	20	131	160.2575758	12.58486344	0.882838351	0.040688864	+	84081	grey	-0.515945095	FALSE	NSRP1
Region_7606	chr17	30194319	30236002	41683	16	92	122.469697	10.54837895	0.955981673	0.024561022	-	6532	grey	0.172539396	FALSE	SLC6A4
Region_7607	chr17	30248203	30292056	43853	13	81	105.9545455	11.59765469	0.786208514	0.027495378	-	642	grey	-0.084987238	FALSE	BLMH
Region_7608	chr17	30316333	30334059	17726	9	56	75.25757576	7.71872968	0.892097229	0.049175805	-	388364	grey	0.250501501	FALSE	TMIGD1
Region_7609	chr17	30378927	30469989	91062	27	195	229.969697	14.68748258	0.897156877	0.024912738	+	1362	grey	0.09050972	FALSE	CPD
Region_7610	chr17	30477362	30527592	50230	13	77	96.45454545	9.092992768	0.918068791	0.018021322	+	9527	grey	-0.166445229	FALSE	GOSR1
Region_7611	chr17	30573471	30577000	3529	4	22	37.90909091	6.270142368	0.85686118	0.062658406	-	101927093	grey	0.170507847	FALSE	LOC101927093
Region_7612	chr1	30576485	30672789	96304	47	279	332.666666	23.8922796	0.81201298	0.02236349	+	10713351	grey	0.308928734	FALSE	SMURF2P1-LRRC37BP1

	7						7	2	2	8		5				
Region_7613	chr17	30646161	30665672	19511	10	42	71.07575758	10.36608465	0.930860247	0.037277498	+	105371723	grey	-0.045859627	FALSE	LOC105371723
Region_7614	chr17	30709299	30790908	81609	34	199	236.3636364	16.77143759	0.8475298	0.021158041	+	440423	grey	0.201351357	FALSE	SUZ12P1
Region_7615	chr17	30772363	30824692	52329	31	190	237.6060606	18.73776835	0.722405898	0.027089999	-	51379	grey	-0.08662596	FALSE	CRLF3
Region_7616	chr17	30831966	30895869	63903	22	135	172.9090909	16.54711434	0.920898401	0.018040304	+	79915	grey	-0.031512377	FALSE	ATAD5
Region_7617	chr17	30898986	30906238	7252	3	16	23.96969697	4.60424452	0.969806666	0.03917232	-	79736	grey	-0.287980019	FALSE	TEFM
Region_7618	chr17	30921680	30959322	37642	9	35	59.01515152	8.083206569	0.851564247	0.036420735	+	55803	grey	0.021815136	FALSE	ADAP2
Region_7619	chr17	30968785	30999911	31126	9	46	65.53030303	7.843114956	0.772553619	0.035358191	+	84282	grey	-0.101226979	FALSE	RNF135
Region_7620	chr17	31008497	31045937	37440	7	49	63.53030303	7.264708575	0.710042789	0.024689749	+	107984974	grey	-0.115612983	FALSE	LOC107984974
Region_7621	chr17	31090787	31095450	4663	6	25	41.60606061	7.448032859	0.488968937	0.02214675	-	105371722	grey	0.117569772	FALSE	MIR4733HG
Region_7622	chr17	31094927	31381887	286960	55	377	437.5909091	22.7683838	0.894826448	0.009391317	+	4763	grey	-0.148921008	FALSE	NF1
Region_7623	chr17	31391675	31538211	146536	37	224	255.030303	18.23603339	0.813379423	0.016755031	+	84440	grey	-0.087681039	FALSE	RAB11FIP4
Region_7624	chr17	31860904	31901708	40804	12	77	98.87878788	8.86221912	0.824547632	0.028827303	-	55813	grey	-0.013287829	FALSE	UTP6

Region_7625	chr17	31937007	32001038	64031	18	110	134.0454545	12.58012085	0.91380283	0.021971824	+	23512	grey	0.349384263	FALSE	SUZ12
Region_7626	chr17	32007383	32053504	46121	12	71	99.42424242	11.52663885	0.797942849	0.036298031	+	114659	grey	-0.058934125	FALSE	LRRC37B
Region_7627	chr17	32142454	32253374	110920	31	203	239.6969697	15.08026698	0.873500468	0.016267981	+	55288	grey	-0.065510288	FALSE	RHOT1
Region_7628	chr17	32258615	32265404	6789	3	13	22.43939394	4.971081875	0.818479353	0.076731972	-	105371731	grey	-0.288649803	FALSE	LOC105371731
Region_7629	chr17	32265832	32324659	58827	18	103	135.1212121	15.57662465	0.808486684	0.02887828	+	162494	grey	0.146049944	FALSE	RHBDL3
Region_7630	chr17	32324431	32381885	57454	13	72	98.12121212	9.242901894	0.774761724	0.041922531	+	7756	grey	-0.27240876	FALSE	ZNF207
Region_7631	chr17	32324565	32350023	25458	6	36	50.25757576	6.271573489	0.575030399	0.066245965	-	64149	grey	-0.333426836	FALSE	C17orf75
Region_7632	chr17	32444379	32483319	38940	6	26	38.5	6.919871053	0.791747303	0.033740125	+	5717	grey	0.339051334	FALSE	PSMD11
Region_7633	chr17	32486993	32491253	4260	4	10	25.03030303	5.343492655	0.705284635	0.052862644	+	8851	grey	0.089555135	FALSE	CDK5R1
Region_7634	chr17	32492522	32877177	384655	60	442	508.6212121	21.05945076	0.832088847	0.018162992	-	4642	grey	-0.193493979	FALSE	MYO1D
Region_7635	chr17	32509954	32523424	13470	4	14	27.12121212	4.629388235	0.784548417	0.096601571	-	105371734	grey	-0.100827547	FALSE	LOC105371734
Region_7636	chr17	32876759	32906584	29825	5	19	37.16666667	6.103172777	0.591000479	0.049913072	+	102724715	grey	0.226656749	FALSE	MYO1D-DT
Region_7637	chr1	32970376	32997877	27501	4	20	29.2272727	5.17923503	0.76869513	0.07891179	+	124912	grey	-0.11793882	FALSE	SPACA3

	7						3	3	7					5		
Region_7638	chr17	33013087	34157294	1144207	197	1564	1671.621212	48.06590512	0.824646883	0.013285697	-	40	grey	-0.000831049	FALSE	ASIC2
Region_7639	chr17	33111858	33131743	19885	3	14	23.77272727	4.498873208	0.930250149	0.059243432	+	105371737	grey	-0.198098536	FALSE	LOC105371737
Region_7640	chr17	33565764	33624122	58358	13	92	113.9545455	11.15269104	0.848971316	0.041526764	+	147004	grey	0.152950178	FALSE	LOC147004
Region_7641	chr17	33931132	34156000	224868	43	286	343.2575758	20.91102808	0.853617274	0.024087978	-	107985038	grey	-0.066636105	FALSE	LOC107985038
Region_7642	chr17	33955024	34071562	116538	24	159	194.8484848	16.63290753	0.846367718	0.03191579	-	107987247	grey	-0.136328782	FALSE	LOC107987247
Region_7643	chr17	34155566	34165736	10170	7	45	64.6969697	8.386747232	0.694314888	0.048729505	+	105371735	grey	-0.046090893	FALSE	LOC105371735
Region_7644	chr17	34157181	34195937	38756	7	41	54.92424242	7.153935132	0.93862526	0.033678635	-	101927239	grey	-0.049826722	FALSE	LINC01989
Region_7645	chr17	34479272	34482148	2876	3	10	22.07575758	4.622056173	0.915772273	0.072240954	+	105371739	grey	-0.005984389	FALSE	LOC105371739
Region_7646	chr17	34579487	34639318	59831	37	227	274.2878788	19.04308084	0.417342331	0.020588449	+	124842	grey	0.295459717	FALSE	TMEM132E
Region_7647	chr17	34927859	34981078	53219	18	123	158.0757576	13.20409098	0.763321941	0.019802388	-	10693	grey	0.241792354	FALSE	CCT6B
Region_7648	chr17	34980512	35009743	29231	13	79	98.84848485	10.81770973	0.735574799	0.029393479	+	3980	grey	0.008382481	FALSE	LIG3
Region_7649	chr17	35005990	35089319	83329	23	158	189	14.28824153	0.756669275	0.013511994	-	117584	grey	0.11049028	FALSE	RFFL

Region_7650	chr17	35011349	35121522	110173	34	229	272.9242424	19.52931572	0.73835514	0.011809017	-	100529207	grey	-0.156956701	FALSE	RAD51L3-RFFL
Region_7651	chr17	35026496	35121522	95026	30	195	239.3030303	18.85325493	0.706976009	0.013077953	-	5892	grey	-0.141104303	FALSE	RAD51D
Region_7652	chr17	35121615	35130732	9117	3	10	23.37878788	4.991577989	0.917295674	0.053892053	+	54752	grey	-0.228342697	FALSE	FNDC8
Region_7653	chr17	35147817	35189345	41528	18	133	156.9090909	11.70105488	0.878119134	0.026569869	+	146862	grey	-0.004021862	FALSE	UNC45B
Region_7654	chr17	35243071	35273655	30584	6	39	52.84848485	7.656555611	0.468892249	0.038013547	+	162394	grey	-0.075235375	FALSE	SLFN5
Region_7655	chr17	35406684	35433283	26599	6	30	49.74242424	8.084763649	0.774057202	0.047973094	-	55106	grey	-0.104225417	FALSE	SLFN12
Region_7656	chr17	35464249	35537678	73429	12	82	97.51515152	8.825212875	0.579809055	0.044102884	-	100506736	grey	0.146447416	FALSE	SLFN12L
Region_7657	chr17	35578046	35726413	148367	36	256	290.2727273	16.09734374	0.808781666	0.020415303	+	163	grey	0.082374199	FALSE	AP2B1
Region_7658	chr17	35731639	35743521	11882	6	26	41.63636364	6.269919307	0.588059674	0.071250185	+	91608	grey	-0.129111614	FALSE	RASL10B
Region_7659	chr17	35744511	35753239	8728	9	53	73.54545455	10.29141811	0.546526601	0.050446468	-	246176	grey	-0.263106384	FALSE	GAS2L2
Region_7660	chr17	35760887	35765079	4192	5	24	39.93939394	6.428012704	0.587391412	0.035269762	+	146853	grey	0.204302046	FALSE	C17orf50
Region_7661	chr17	36116854	36149080	32226	5	23	37.96969697	9.236998675	0.757599849	0.038311226	-	101927369	grey	0.006679553	FALSE	LOC101927369
Region_7662	chr1	37642947	37684252	41305	18	130	164.530303	14.1489001	0.71061608	0.02958369	+	10537175	grey	0.172051859	FALSE	LOC105371756

	7							4	8	7		6				
Region_7663	chr17	38078262	38124770	46508	4	11	24.04545455	5.765765859	0.938204248	0.063040945	+	101929950	grey	-0.095968192	FALSE	LOC101929950
Region_7664	chr17	38869859	38921770	51911	15	92	111.6666667	10.31553471	0.737223879	0.04437707	+	3927	grey	-0.100516286	FALSE	LASP1
Region_7665	chr17	38936432	38967403	30971	6	29	44.01515152	7.085179384	0.951658344	0.033829857	-	494188	grey	0.100359688	FALSE	FBXO47
Region_7666	chr17	39027277	39053215	25938	5	29	41.03030303	5.502362281	0.757801797	0.038712192	+	342666	grey	0.136642414	FALSE	LRRC37A11P
Region_7667	chr17	39057019	39112239	55220	13	79	97.25757576	8.538258886	0.727043804	0.034978692	+	100131347	grey	0.166834378	FALSE	RDM1P5
Region_7668	chr17	39063313	39154394	91081	21	131	160.7272727	12.67650213	0.822221884	0.023384959	-	57125	grey	0.1279461	FALSE	PLXDC1
Region_7669	chr17	39156894	39167065	10171	5	22	30.63636364	4.963362271	0.222674834	0.032945285	-	390790	grey	-0.029208341	FALSE	ARL5C
Region_7670	chr17	39173453	39197703	24250	5	23	36.68181818	5.438209804	0.641563426	0.071647175	-	782	grey	-0.008878836	FALSE	CACNB1
Region_7671	chr17	39200283	39204840	4557	5	20	34.46969697	5.925744866	0.945288567	0.042607405	+	6143	grey	-0.225730147	FALSE	RPL19
Region_7672	chr17	39210541	39225945	15404	7	42	60.33333333	9.417387084	0.561592861	0.039491916	-	342667	grey	0.061324108	FALSE	STAC2
Region_7673	chr17	39238466	39250818	12352	4	15	23.98484848	4.862707142	0.685858067	0.092470461	+	101929578	grey	-0.07401352	FALSE	LOC101929578
Region_7674	chr17	39252663	39402523	149860	29	172	219.030303	15.36025811	0.875206471	0.019676109	-	84961	grey	-0.033003282	FALSE	FBXL20

Region_7675	chr17	39404285	39451272	46987	7	41	55.1969697	6.832545864	0.970063087	0.023052091	-	5469	grey	-0.180191009	FALSE	MED1
Region_7676	chr17	39461486	39564907	103421	28	159	195.0151515	17.89068724	0.877266789	0.019866542	+	51755	grey	-0.246981207	FALSE	CDK12
Region_7677	chr17	39603768	39607920	4152	5	24	36.72727273	6.340457286	0.018481116	0.035390527	-	4761	grey	0.06339909	FALSE	NEUROD2
Region_7678	chr17	39637090	39664201	27111	10	54	68.78787879	7.035110084	0.852068831	0.032069066	+	10948	grey	0.151914086	FALSE	STARD3
Region_7679	chr17	39671122	39696797	25675	15	86	110.3181818	11.20049325	0.829631654	0.037174508	-	93210	grey	0.102472427	FALSE	PGAP3
Region_7680	chr17	39687914	39728662	40748	21	147	182.1818182	12.95187434	0.91130255	0.018202418	+	2064	grey	0.219896894	FALSE	ERBB2
Region_7681	chr17	39737927	39747291	9364	9	47	62.33333333	8.321119254	0.613998824	0.052166093	+	2886	grey	0.163785528	FALSE	GRB7
Region_7682	chr17	39757718	39864312	106594	28	192	225.7575758	14.15090169	0.852772823	0.037210748	-	22806	grey	0.123108455	FALSE	IKZF3
Region_7683	chr17	39868202	39877896	9694	3	14	25.6969697	4.094911277	0.938514964	0.055333264	+	124626	grey	-0.253634989	FALSE	ZPBP2
Region_7684	chr17	39904595	39919854	15259	5	25	34.77272727	4.663427114	0.828775854	0.074402255	-	55876	grey	-0.022968129	FALSE	GSDMB
Region_7685	chr17	39921041	39927601	6560	5	20	35.95454545	7.006440593	0.386176596	0.025754944	-	94103	grey	0.036904577	FALSE	ORMDL3
Region_7686	chr17	39927732	39944999	17267	9	60	85.22727273	10.72566362	0.943144874	0.032472763	+	100505591	grey	0.22880562	FALSE	LRRC3C
Region_7687	chr1	39927844	39939601	11757	8	54	78.3030303	10.6250353	0.96048152	0.02881573	+	11226818	grey	0.103543928	FALSE	

	7							1	2	3		7				
Region_7688	chr17	39953263	39977768	24505	18	82	118.1212121	12.41279136	0.866516161	0.034173681	+	284110	grey	0.173794218	FALSE	GSDMA
Region_7689	chr17	39980807	39997959	17152	16	90	108	10.10102813	0.776091868	0.033867076	+	5709	grey	-0.228725937	FALSE	PSMD3
Region_7690	chr17	40019097	40061215	42118	32	178	209.9242424	15.0315674	0.8760897	0.021004129	-	9862	grey	-0.189285751	FALSE	MED24
Region_7691	chr17	40058290	40093867	35577	18	86	105.5	8.705877767	0.639682329	0.034491209	+	7067	grey	0.050781463	FALSE	THRA
Region_7692	chr17	40092793	40100589	7796	6	32	47.24242424	6.363503162	0.462155738	0.040254974	-	9572	grey	-0.009602402	FALSE	NR1D1
Region_7693	chr17	40121971	40136917	14946	9	41	62.12121212	9.110459165	0.582919455	0.02786676	+	339287	grey	-0.200764284	FALSE	MSL1
Region_7694	chr17	40140318	40172171	31853	16	96	116.8787879	10.15346118	0.830282888	0.025685599	+	22794	grey	0.158982268	FALSE	CASC3
Region_7695	chr17	40177010	40195656	18646	9	39	58.60606061	7.747711896	0.540989804	0.023029837	+	51195	grey	-0.029247206	FALSE	RAPGEFL1
Region_7696	chr17	40219304	40284136	64832	36	200	248.7727273	17.23930701	0.947127884	0.015023357	+	147179	grey	-0.336726691	FALSE	WIPF2
Region_7697	chr17	40287879	40304657	16778	5	29	45.40909091	6.32693211	0.962671259	0.03384178	+	990	grey	-0.056549698	FALSE	CDC6
Region_7698	chr17	40309180	40357643	48463	34	204	234.8787879	17.75431922	0.597997648	0.022159322	+	5914	grey	0.025484499	FALSE	RARA
Region_7699	chr17	40360652	40364737	4085	9	45	63.39393939	9.119512112	0.161473568	0.040976972	-	125111	grey	-0.307957986	FALSE	GJD3

Region_7700	chr17	40388525	40417896	29371	11	76	94.98484848	9.115485428	0.708519896	0.018696335	-	7153	grey	0.076535221	FALSE	TOP2A
Region_7701	chr17	40443450	40457725	14275	8	36	50.34848485	6.383855657	0.704802977	0.036870166	+	3487	grey	-0.035758106	FALSE	IGFBP4
Region_7702	chr17	40475828	40501623	25795	7	41	57.07575758	7.17755177	0.747278078	0.046495905	-	84951	grey	-0.158302939	FALSE	TNS4
Region_7703	chr17	40553769	40565472	11703	6	34	46.36363636	6.726838013	0.518715056	0.070948682	-	1236	grey	-0.155203328	FALSE	CCR7
Region_7704	chr17	40624962	40665135	40173	13	98	127.6515152	12.04797133	0.693891829	0.033157656	-	6605	grey	0.080291534	FALSE	SMARCE1
Region_7705	chr17	40628797	40665181	36384	12	81	114.4848485	11.54821654	0.670114404	0.036232391	-	125113	grey	0.053589306	FALSE	KRT222
Region_7706	chr17	40697991	40703752	5761	4	16	26.22727273	5.634496645	0.918748497	0.055761587	-	192666	grey	-0.289523759	FALSE	KRT24
Region_7707	chr17	40748021	40755331	7310	4	26	42.8030303	8.111188279	0.727616262	0.131192196	-	147183	grey	0.162529726	FALSE	KRT25
Region_7708	chr17	40818117	40822614	4497	9	48	66.87878788	10.13829777	0.627637091	0.037682437	-	3858	grey	0.04397823	FALSE	KRT10
Region_7709	chr17	40819101	40836270	17169	17	105	135.030303	12.81821984	0.793410463	0.026973921	+	147184	grey	-0.015227396	FALSE	KRT10-AS1
Region_7710	chr17	41436154	41440983	4829	7	44	66.90909091	9.710782703	0.940996214	0.030255894	-	8687	grey	-0.049527573	FALSE	KRT38
Region_7711	chr17	41459513	41467386	7873	4	16	25.77272727	5.479459419	0.882136914	0.056138879	-	3882	grey	-0.096741678	FALSE	KRT32
Region_7712	chr1	41476710	41481151	4441	3	20	32.8333333	5.69097351	0.90934513	0.05408830	-	3886	grey	0.183746861	FALSE	KRT35

	7						3		9	1						
Region_7713	chr17	41486136	41492546	6410	3	12	22.27272727	4.763791168	0.952956962	0.065511157	-	8689	grey	-0.045956922	FALSE	KRT36
Region_7714	chr17	41513745	41522529	8784	3	16	28.81818182	5.504988202	0.944671102	0.05000574	-	3866	grey	0.057365761	FALSE	KRT15
Region_7715	chr17	41549606	41554495	4889	3	15	24.74242424	4.766261577	0.811942152	0.089131472	-	147093	grey	0.304507926	FALSE	LINC00974
Region_7716	chr17	41565836	41572059	6223	3	12	24.09090909	5.049683228	0.878928273	0.067936253	-	3857	grey	0.103474434	FALSE	KRT9
Region_7717	chr17	41619442	41624842	5400	7	34	48.3030303	6.961365244	0.869059524	0.05418876	-	3872	grey	0.261409895	FALSE	KRT17
Region_7718	chr17	41626327	41640199	13872	8	48	62.12121212	6.376676616	0.780488095	0.051706477	-	284116	grey	-0.311956428	FALSE	KRT42P
Region_7719	chr17	41712331	41715969	3638	3	13	20.77272727	4.234639044	0.902727947	0.080763232	+	2520	grey	-0.20620683	FALSE	GAST
Region_7720	chr17	41717742	41734644	16902	8	34	50.10606061	6.607987679	0.824691617	0.049989209	-	9001	grey	0.193934649	FALSE	HAP1
Region_7721	chr17	41754604	41786931	32327	19	113	134.469697	9.809288686	0.636299104	0.023510696	-	3728	grey	0.077402348	FALSE	JUP
Region_7722	chr17	41801947	41812603	10656	4	24	35.39393939	5.718737859	0.71159804	0.041338601	-	10609	grey	0.257427637	FALSE	P3H4
Region_7723	chr17	41812680	41823213	10533	6	26	39.78787879	6.291667631	0.950589097	0.043330756	+	60681	grey	0.136653219	FALSE	FKBP10
Region_7724	chr17	41825057	41836260	11203	8	34	54.09090909	8.63397096	0.602151941	0.029222055	-	115024	grey	-0.053319481	FALSE	NT5C3B

Region_7725	chr17	41835685	41848384	12699	14	79	104.3787879	13.54055464	0.571691409	0.025595174	+	317719	grey	0.171592182	FALSE	KLHL10
Region_7726	chr17	41848518	41865423	16905	11	70	88.86363636	10.38917538	0.802098367	0.024765983	-	55175	grey	0.101587741	FALSE	KLHL11
Region_7727	chr17	41866917	41930542	63625	42	270	303.4848485	15.67285004	0.805290198	0.010793198	-	47	grey	0.067061182	FALSE	ACLY
Region_7728	chr17	41930617	41966503	35886	13	92	114.0909091	10.10974743	0.897578059	0.034964734	+	83538	grey	0.049386669	FALSE	ODAD4
Region_7729	chr17	41966763	41977740	10977	5	16	27.5	4.858774774	0.393041016	0.024907154	+	1267	grey	-0.070429091	FALSE	CNP
Region_7730	chr17	41976421	42021376	44955	24	161	201.6969697	18.13960367	0.786298965	0.020810736	-	7266	grey	0.002548544	FALSE	DNAJC7
Region_7731	chr17	42011382	42025644	14262	12	82	107.0909091	11.01988032	0.55267158	0.022242675	+	28511	grey	0.241654218	FALSE	NKIRAS2
Region_7732	chr17	42025576	42098479	72903	51	329	381.0909091	22.46414392	0.719420866	0.016981831	-	201181	grey	-0.041751443	FALSE	ZNF385C
Region_7733	chr17	42038232	42050601	12369	7	40	59.60606061	9.69358041	0.577297049	0.027661496	-	110806298	grey	0.021423899	FALSE	C17orf113
Region_7734	chr17	42101404	42112714	11310	11	50	72.90909091	8.974711948	0.743545423	0.030588128	-	79132	grey	0.095431565	FALSE	DHX58
Region_7735	chr17	42113111	42125866	12755	15	76	101.530303	10.09069594	0.86214267	0.033452699	-	2648	grey	-0.131040973	FALSE	KAT2A
Region_7736	chr17	42119674	42155044	35370	34	212	242.0757576	16.81053754	0.839920253	0.029158721	-	5878	grey	-0.055251177	FALSE	RAB5C
Region_7737	chr1	42154600	42155972	1372	4	24	35.4848484	5.89978467	0.03861124	0.04265394	+	10537178	grey	-0.26607576	FALSE	RAB5C-AS1

	7						8	5	1	5		1		6		
Region_7738	chr17	42156891	42181142	24251	17	102	134.969697	12.85776592	0.852535386	0.028966702	-	23415	grey	0.373513705	FALSE	KCNH4
Region_7739	chr17	42184060	42185452	1392	3	10	27.42424242	5.314886513	0.171545675	0.0577944	-	3060	grey	0.031211345	FALSE	HCRT
Region_7740	chr17	42199176	42288633	89457	78	477	538.1666667	27.57121316	0.656765972	0.011775161	-	6777	grey	-0.031120175	FALSE	STAT5B
Region_7741	chr17	42287547	42311943	24396	29	155	197.3787879	16.10901816	0.621659376	0.019724605	+	6776	grey	-0.105418729	FALSE	STAT5A
Region_7742	chr17	42313324	42388540	75216	37	207	237.9545455	14.22936489	0.748295208	0.024225711	-	6774	grey	0.173811735	FALSE	STAT3
Region_7743	chr17	42402449	42423256	20807	25	117	150.1969697	17.35353628	0.482479932	0.0208147	-	284119	grey	0.065217459	FALSE	CAVIN1
Region_7744	chr17	42458844	42522582	63738	17	101	129.8181818	10.30296312	0.801630772	0.034543761	+	535	grey	-0.18943081	FALSE	ATP6V0A1
Region_7745	chr17	42536241	42562062	25821	11	52	73.93939394	9.415010581	0.846526369	0.037890141	+	4669	grey	-0.083651343	FALSE	NAGLU
Region_7746	chr17	42579513	42610623	31110	17	90	115.3181818	11.70425181	0.846409988	0.022837757	-	162427	grey	0.027859703	FALSE	RETREG3
Region_7747	chr17	42609641	42615238	5597	4	20	29.36363636	4.894695288	0.974088871	0.038169782	+	7283	grey	0.11039262	FALSE	TUBG1
Region_7748	chr17	42659284	42667006	7722	6	29	41.86363636	5.758969896	0.648549816	0.040973324	+	27175	grey	-0.155772537	FALSE	TUBG2
Region_7749	chr17	42678889	42683917	5028	8	38	59.39393939	9.27507626	0.085163115	0.032678438	-	2826	grey	-0.229347541	FALSE	CCR10

Region_7750	chr17	42679963	42699993	20030	22	127	164.5606061	12.54546046	0.747983781	0.023007717	+	8506	grey	-0.095764783	FALSE	CNTNAP1
Region_7751	chr17	42700275	42745049	44774	28	160	206.8030303	20.77440711	0.940902668	0.016314051	-	2145	grey	0.078275491	FALSE	EZH1
Region_7752	chr17	42773449	42779599	6150	4	12	26.8030303	6.026147377	0.431447141	0.10717869	+	84313	grey	0.267843404	FALSE	VPS25
Region_7753	chr17	42780610	42797066	16456	12	58	76.90909091	7.980220303	0.55156551	0.044907111	+	65266	grey	0.06067233	FALSE	WNK4
Region_7754	chr17	42795147	42798704	3557	3	16	26.60606061	4.735719547	0.635730845	0.046326345	-	28958	grey	0.007368907	FALSE	COA3
Region_7755	chr17	42810134	42833350	23216	13	65	89.16666667	10.20645849	0.603666397	0.027958526	-	8678	grey	0.090889339	FALSE	BECN1
Region_7756	chr17	42824385	42833026	8641	9	43	59.90909091	7.443587375	0.764440168	0.037415242	+	107985007	grey	0.054208848	FALSE	
Region_7757	chr17	42824426	42843760	19334	21	137	163.5454545	14.93182643	0.757053	0.020965611	+	10197	grey	-0.003920182	FALSE	PSME3
Region_7758	chr17	42844580	42850707	6127	3	16	26.51515152	4.801417817	0.735815041	0.084439099	+	314	grey	0.104461821	FALSE	AOC2
Region_7759	chr17	42851184	42858130	6946	9	54	68.37878788	7.962923992	0.919785674	0.039146331	+	8639	grey	0.082341942	FALSE	AOC3
Region_7760	chr17	42874670	42898704	24034	8	47	60.10606061	6.575312307	0.707020745	0.045372663	-	388387	grey	-0.071767442	FALSE	LINC00671
Region_7761	chr17	42900797	42914438	13641	12	77	98.10606061	9.871682559	0.879902574	0.022494525	+	2538	grey	0.100042031	FALSE	G6PC1
Region_7762	chr1	42950526	42964498	13972	5	26	39.3181818	5.59163961	0.76969810	0.03478035	-	80755	grey	-0.2166731	FALSE	AARSD1

	7						2	3	5	3						
Region_7763	chr17	42950526	42980528	30002	20	120	148.9393939	12.74227221	0.859757343	0.024397603	-	100885850	grey	-0.003865059	FALSE	PTGES3L-AARSD1
Region_7764	chr17	42968088	42980433	12345	15	86	109.6212121	12.19301837	0.889808117	0.030885576	-	100885848	grey	0.111318067	FALSE	PTGES3L
Region_7765	chr17	42980565	42995142	14577	6	34	48.03030303	7.202498919	0.664543383	0.035531936	+	146923	grey	0.082593156	FALSE	RUNDC1
Region_7766	chr17	42998273	43002959	4686	5	28	39.36363636	5.887761397	0.595566764	0.045645665	+	6155	grey	-0.230743495	FALSE	RPL27
Region_7767	chr17	43006740	43014456	7716	9	60	75.53030303	7.145132172	0.954805449	0.026156111	+	3430	grey	-0.125525103	FALSE	IFI35
Region_7768	chr17	43014607	43025123	10516	13	69	93.48484848	10.797781	0.118201742	0.027024924	-	10493	grey	-0.023030064	FALSE	VAT1
Region_7769	chr17	43044295	43170245	125950	84	538	640.7272727	34.2931011	0.89329334	0.016200382	-	672	grey	-0.034206623	FALSE	BRCA1
Region_7770	chr17	43125551	43153671	28120	35	207	256.3484848	18.29038623	0.871780771	0.019783549	+	10230	grey	0.072628761	FALSE	NBR2
Region_7771	chr17	43148368	43171037	22669	8	58	73.37878788	8.045572412	0.746453854	0.025665197	-	101929767	grey	0.075839809	FALSE	LOC101929767
Region_7772	chr17	43170481	43211689	41208	34	237	275.1818182	17.05277698	0.914836797	0.016659085	+	4077	grey	-0.020264081	FALSE	NBR1
Region_7773	chr17	43211835	43220041	8206	7	28	44.48484848	8.751689814	0.261070284	0.050724716	+	113277	grey	0.023823965	FALSE	TMEM106A
Region_7774	chr17	43216941	43305397	88456	27	285	493.6515152	104.8580715	0.604876054	0.025174441	-	100874261	grey	0.062210644	FALSE	CCDC200

Region_7775	chr17	43338741	43389226	50485	38	268	344.0606061	28.7417567	0.779311792	0.013549085	-	100130581	grey	-0.019449976	FALSE	LINC00910
Region_7776	chr17	43444707	43451200	6493	5	28	43	6.600699264	0.759167811	0.043306461	+	106660605	grey	0.358221558	FALSE	MIR2117HG
Region_7777	chr17	43483865	43590741	106876	78	522	610.1666667	29.22305904	0.830131378	0.013755442	+	1659	grey	-0.117945697	FALSE	DHX8
Region_7778	chr17	43527844	43579620	51776	39	234	272.1060606	17.45687327	0.798972278	0.021412462	-	2118	grey	-0.034366358	FALSE	ETV4
Region_7779	chr17	43640389	43661922	21533	17	100	118.0606061	10.84418141	0.779655525	0.036082082	-	4222	grey	0.251810094	FALSE	MEOX1
Region_7780	chr17	43679341	43706709	27368	19	120	146.7121212	12.52783381	0.91369456	0.025425421	+	105371786	grey	-0.071092015	FALSE	LINC02594
Region_7781	chr17	43766125	43778977	12852	3	13	22.42424242	4.706985336	0.581363158	0.077129835	-	1845	grey	0.041331603	FALSE	DUSP3
Region_7782	chr17	43800799	43833170	32371	8	42	55.95454545	6.893546186	0.945158556	0.028674646	-	4356	grey	0.088848869	FALSE	MPP3
Region_7783	chr17	43847148	43863639	16491	8	38	56	8.313103651	0.744139499	0.048822769	+	146894	grey	-0.139726165	FALSE	CD300LG
Region_7784	chr17	43875357	43909711	34354	24	149	181.8333333	14.62830064	0.570730585	0.023721941	-	4355	grey	0.048968644	FALSE	MPP2
Region_7785	chr17	43914433	43923001	8568	3	11	17.87878788	4.255368199	0.353922579	0.062198797	-	105371787	grey	-0.042452769	FALSE	LOC105371787
Region_7786	chr17	43952733	44004469	51736	38	252	287.5757576	18.24032777	0.591630373	0.022989403	-	5697	grey	0.052316223	FALSE	PYY
Region_7787	chr1	44004622	44009068	4446	6	25	42.8181818	8.04584764	0.02701897	0.03029010	+	162417	grey	-0.21939983	FALSE	NAGS

	7						2	6	2	4				3		
Region_7788	chr17	44011188	44023151	11963	5	27	41.8030303	6.276254878	0.774807872	0.036932038	-	84336	grey	-0.168692543	FALSE	TMEM101
Region_7789	chr17	44034328	44067619	33291	23	121	175.3636364	15.89933367	0.818800065	0.018540105	-	124801	grey	0.284533536	FALSE	LSM12
Region_7790	chr17	44076746	44123702	46956	18	89	111.8181818	10.93323668	0.730206775	0.030917512	-	10014	grey	0.180181899	FALSE	HDAC5
Region_7791	chr17	44141906	44162476	20570	4	21	33.43939394	5.491612592	0.928665306	0.046115842	+	78995	grey	-0.028872016	FALSE	HROB
Region_7792	chr17	44175968	44186723	10755	6	26	43.03030303	7.759797176	0.953898231	0.032750128	-	339201	grey	0.177410005	FALSE	ASB16-AS1
Region_7793	chr17	44191805	44200113	8308	4	11	23.72727273	4.277114248	0.968311315	0.046649302	-	56970	grey	-0.070380479	FALSE	ATXN7L3
Region_7794	chr17	44198537	44223707	25170	14	71	93.06060606	11.12845112	0.14595001	0.015427444	+	101926967	grey	0.178319305	FALSE	ATXN7L3-AS1
Region_7795	chr17	44205033	44221626	16593	11	53	74.74242424	9.93565546	0.10216485	0.014051523	-	7343	grey	0.135796908	FALSE	UBTF
Region_7796	chr17	44248390	44268141	19751	5	24	37.63636364	5.537665055	0.932278728	0.061449124	-	6521	grey	0.048256281	FALSE	SLC4A1
Region_7797	chr17	44299574	44315315	15741	6	33	47.15151515	7.678626528	0.748467115	0.049981018	-	101926996	grey	-0.054669044	FALSE	RUNDC3A-AS1
Region_7798	chr17	44308413	44318670	10257	7	38	58.21212121	10.17918714	0.272270665	0.029882314	+	10900	grey	-0.16904699	FALSE	RUNDC3A
Region_7799	chr17	44319625	44324870	5245	5	21	35.86363636	5.391977983	0.776032402	0.026458789	-	51629	grey	-0.071547878	FALSE	SLC25A39

Region_7800	chr17	44345246	44353106	7860	9	58	76.72727273	8.319662446	0.630934965	0.043332835	+	2896	grey	-0.161053748	FALSE	GRN
Region_7801	chr17	44372180	44389649	17469	5	26	39.18181818	5.662043912	0.675914028	0.046093693	-	3674	grey	0.104694411	FALSE	ITGA2B
Region_7802	chr17	44395281	44503430	108149	30	196	233.5606061	16.18653646	0.944980058	0.010228498	-	23131	grey	0.085145067	FALSE	GPATCH8
Region_7803	chr17	44656404	44690308	33904	14	85	116.4090909	13.65957232	0.548964745	0.033241808	+	284071	grey	-0.123755933	FALSE	MEIOC
Region_7804	chr17	44708608	44752264	43656	10	57	71.54545455	8.218519085	0.732151405	0.036237445	+	80174	grey	-0.348400897	FALSE	DBF4B
Region_7805	chr17	44758988	44781846	22858	11	52	74.83333333	9.083515642	0.738465866	0.035833808	+	4185	grey	0.003779466	FALSE	ADAM11
Region_7806	chr17	44794747	44830816	36069	7	44	58.28787879	6.287387011	0.563316974	0.023463241	-	10052	grey	0.048339191	FALSE	GJC1
Region_7807	chr17	44846353	44850476	4123	11	70	91.27272727	11.78988544	0.950399604	0.027423039	+	51751	grey	-0.122647025	FALSE	HIGD1B
Region_7808	chr17	44849948	44899445	49497	31	214	252.7878788	16.28733831	0.812894097	0.02339646	-	9343	grey	0.090253495	FALSE	EFTUD2
Region_7809	chr17	44899142	44905390	6248	8	48	63.54545455	7.129962387	0.101874845	0.032773825	+	388389	grey	-0.044513827	FALSE	CCDC103
Region_7810	chr17	44903159	44915951	12792	8	45	63.71212121	6.706518417	0.710012329	0.06556994	-	2670	grey	0.045189793	FALSE	GFAP
Region_7811	chr17	44924709	44947773	23064	8	40	57.92424242	7.388786943	0.850805476	0.024320349	-	146909	grey	0.023244302	FALSE	KIF18B
Region_7812	chr1	44957992	45109016	151024	76	496	569.712121	27.3972290	0.73431538	0.01440453	+	4836	grey	0.024664331	FALSE	NMT1

	7						2	3	7	7						
Region_7813	chr17	44959693	44968303	8610	10	49	66.59090909	9.509472642	0.320614341	0.038984637	-	10882	grey	-0.120108875	FALSE	C1QL1
Region_7814	chr17	45023338	45061131	37793	27	176	208.8333333	14.04123963	0.873933723	0.019366875	-	79877	grey	-0.08885848	FALSE	DCAKD
Region_7815	chr17	45108959	45133354	24395	8	39	55.53030303	7.847037064	0.640591364	0.045374046	-	113026	grey	0.045434824	FALSE	PLCD3
Region_7816	chr17	45132600	45144181	11581	9	52	70.13636364	8.202975752	0.676738226	0.043911411	+	79777	grey	-0.209235389	FALSE	ACBD4
Region_7817	chr17	45190931	45222222	31291	13	75	95.95454545	10.97329881	0.891957162	0.033447426	-	339192	grey	-0.107727129	FALSE	FMNL1-DT
Region_7818	chr17	45221885	45247319	25434	10	40	63.62121212	8.333002324	0.42872624	0.038247335	+	752	grey	0.37931379	FALSE	FMNL1
Region_7819	chr17	45247916	45269824	21908	10	52	77.81818182	9.933485082	0.682339803	0.016858722	+	100133991	grey	-0.200784269	FALSE	MAP3K14-AS1
Region_7820	chr17	45254393	45262094	7701	3	16	25.74242424	4.382870875	0.652024603	0.036643971	-	124783	grey	-0.199970249	FALSE	SPATA32
Region_7821	chr17	45263119	45317029	53910	13	67	88.86363636	9.100949001	0.807237067	0.031331897	-	9020	grey	-0.086172998	FALSE	MAP3K14
Region_7822	chr17	45506741	45584705	77964	23	109	162.1212121	34.44215447	0.814910331	0.023884144	-	55073	grey	-0.040862448	FALSE	LRRC37A4P
Region_7823	chr17	46274784	46361797	87013	3	23	48.87878788	10.81525298	0.888575605	0.071406963	-	100506084	grey	0.036677661	FALSE	ARL17B
Region_7824	chr17	46923075	47049932	126857	51	316	361.1818182	22.9587651	0.79639905	0.022397147	+	9570	grey	-0.133454252	FALSE	GOSR2

Region_7825	chr17	46983287	47100291	117004	39	230	276.9545455	18.00720524	0.884663913	0.018824306	-	101927060	grey	0.109024293	FALSE	LOC101927060
Region_7826	chr17	47117703	47189422	71719	18	102	131.2575758	11.65571581	0.868641628	0.016213999	-	996	grey	-0.141414681	FALSE	CDC27
Region_7827	chr17	47200446	47223679	23233	10	61	83.13636364	8.719361945	0.778289858	0.043030092	+	4635	grey	-0.264239645	FALSE	MYL4
Region_7828	chr17	47253827	47344292	90465	32	219	252.4545455	16.46271481	0.748846797	0.021677623	+	3690	grey	0.166240174	FALSE	ITGB3
Region_7829	chr17	47303460	47323613	20153	11	67	84.10606061	8.579275185	0.856479401	0.026426001	-	102724508	grey	-0.051812476	FALSE	EFCAB13-DT
Region_7830	chr17	47323290	47441312	118022	25	166	191.6515152	13.00827243	0.824589357	0.026164536	+	124989	grey	-0.487658098	FALSE	EFCAB13
Region_7831	chr17	47450568	47492493	41925	21	135	165.3181818	16.50655729	0.878964812	0.030537007	-	653479	grey	-0.075170392	FALSE	MRPL45P2
Region_7832	chr17	47522942	47624665	101723	39	233	272.6818182	17.18421377	0.960933915	0.014910126	+	9520	grey	0.073680775	FALSE	NPEPPS
Region_7833	chr17	47603860	47649449	45589	22	147	171.1060606	12.06537167	0.861361191	0.017930564	-	112268184	grey	-0.101947616	FALSE	KPNB1-DT
Region_7834	chr17	47649476	47685505	36029	9	33	64.36363636	8.590936841	0.745499373	0.023865673	+	3837	grey	0.005801959	FALSE	KPNB1
Region_7835	chr17	47694161	47712052	17891	4	17	32.48484848	5.367258072	0.412096353	0.067912082	+	9755	grey	-0.012729117	FALSE	TBKBP1
Region_7836	chr17	47733236	47746122	12886	6	29	49.13636364	7.65383034	0.446861184	0.048666109	+	30009	grey	0.043696563	FALSE	TBX21
Region_7837	chr1	47807372	47821834	14462	3	17	24.3030303	4.62757519	0.78409036	0.13190660	-	114881	grey	-0.08217838	FALSE	OSBPL7

	7							8	2	7				3		
Region_7838	chr17	47823272	47831541	8269	8	41	56.57575758	8.051871462	0.864730249	0.031091642	-	124995	grey	-0.088975064	FALSE	MRPL10
Region_7839	chr17	47837692	47841289	3597	12	51	80.1969697	12.27908753	0.154656322	0.023969738	-	90507	grey	-0.163890872	FALSE	SCRN2
Region_7840	chr17	47844908	47855874	10966	10	44	66.13636364	9.967464555	0.276986481	0.071157158	-	80320	grey	0.104698662	FALSE	SP6
Region_7841	chr17	47896150	47928957	32807	15	78	107.8484848	12.96176914	0.62475249	0.028607721	+	6668	grey	-0.097652394	FALSE	SP2
Region_7842	chr17	47897330	47941410	44080	16	85	112.9545455	11.05292418	0.874604499	0.025882649	-	100506325	grey	0.073765458	FALSE	SP2-AS1
Region_7843	chr17	47945424	47981781	36357	14	93	115.6515152	10.14888008	0.773098706	0.033140528	+	80279	grey	-0.338082611	FALSE	CDK5RAP3
Region_7844	chr17	48026167	48038030	11863	10	52	73.36363636	8.86242954	0.387092192	0.03865862	-	51226	grey	-0.032952483	FALSE	COPZ2
Region_7845	chr17	48046876	48048098	1222	4	22	37.8030303	7.780092341	0.216818104	0.091512632	-	116435298	grey	-0.228503926	FALSE	NFE2L1-DT
Region_7846	chr17	48048329	48061545	13216	16	90	121.4090909	13.30006047	0.642936431	0.026807676	+	4779	grey	-0.253496638	FALSE	NFE2L1
Region_7847	chr17	48070052	48101478	31426	16	86	115.969697	10.94738423	0.811423312	0.025546422	-	10951	grey	-0.113402276	FALSE	CBX1
Region_7848	chr17	48103357	48123601	20244	5	23	38.78787879	6.369434588	0.350377127	0.056897401	+	29916	grey	0.053462598	FALSE	SNX11
Region_7849	chr17	48133442	48430275	296833	93	678	749.3636364	28.46192402	0.87419165	0.015981648	-	8631	grey	-0.010388467	FALSE	SKAP1

Region_7850	chr17	48294335	48307829	13494	3	11	20.40909091	4.696896759	0.952238488	0.050883123	+	105371807	grey	-0.159874505	FALSE	SKAP1-AS2
Region_7851	chr17	48528526	48531011	2485	3	11	18.81818182	3.802465155	0.96168157	0.050067219	-	3211	grey	0.156548001	FALSE	HOXB1
Region_7852	chr17	48542655	48545109	2454	4	15	28.8030303	5.162646003	0.007656926	0.018819211	-	3212	grey	0.039157834	FALSE	HOXB2
Region_7853	chr17	48543551	48551250	7699	10	36	55.95454545	7.732910325	0.56737871	0.05917837	+	100874362	grey	-0.058909827	FALSE	HOXB-AS1
Region_7854	chr17	48548870	48590241	41371	32	173	214.7878788	15.76414154	0.413031954	0.028914647	-	3213	grey	-0.119749874	FALSE	HOXB3
Region_7855	chr17	48549630	48606414	56784	47	270	320.1363636	18.17765862	0.431136321	0.026443743	+	404266	grey	-0.230069005	FALSE	HOXB-AS3
Region_7856	chr17	48573982	48604992	31010	33	192	234.5606061	16.43647799	0.293244432	0.028599147	-	3216	grey	-0.250529557	FALSE	HOXB6
Region_7857	chr17	48579630	48582259	2629	4	14	25.59090909	4.634244682	0.124736406	0.088143408	-	406902	grey	-0.044504406	FALSE	MIR10A
Region_7858	chr17	48591257	48593779	2522	3	12	20.21212121	4.865941767	0.293245405	0.046875735	-	3215	grey	-0.429606146	FALSE	HOXB5
Region_7859	chr17	48607232	48633572	26340	42	261	304.2424242	22.07543752	0.197841399	0.01742735	-	3217	grey	0.126446206	FALSE	HOXB7
Region_7860	chr17	48612346	48615292	2946	8	39	59.6969697	9.863639049	0.019286782	0.025071629	-	3218	grey	0.010680217	FALSE	HOXB8
Region_7861	chr17	48621156	48626358	5202	9	51	68.36363636	9.5862664	0.183827981	0.031332737	-	3219	grey	-0.067999684	FALSE	HOXB9
Region_7862	chr1	48628675	48634932	6257	6	25	37.3484848	6.01662421	0.42349479	0.07814821	+	10087435	grey	-0.00228888	FALSE	HOXB-AS4

	7						5	1	2	6		1		4		
Region_7863	chr17	48635923	48647023	11100	21	117	141.9242424	11.27326395	0.197339986	0.024001343	-	101060400	grey	0.000836479	FALSE	LOC101060400
Region_7864	chr17	48646923	48707346	60423	25	183	221.0151515	14.82460784	0.773149301	0.019910118	+	105371809	grey	-0.102680298	FALSE	LINC02086
Region_7865	chr17	48762234	48817229	54995	27	155	213.2272727	21.64027261	0.849118916	0.029913012	-	284076	grey	-0.104675778	FALSE	TTLL6
Region_7866	chr17	48831018	48865245	34227	5	33	44.31818182	6.19965034	0.979272671	0.018954575	+	10241	grey	0.165117603	FALSE	CALCOCO2
Region_7867	chr17	48874860	48908983	34123	18	97	131.0151515	13.26591053	0.775609326	0.023899707	-	105371814	grey	-0.182049921	FALSE	LOC105371814
Region_7868	chr17	48908407	48929056	20649	11	75	92.34848485	9.729404163	0.786905659	0.01930345	+	65264	grey	0.035339612	FALSE	UBE2Z
Region_7869	chr17	48929316	48944842	15526	22	127	174.2575758	18.4976594	0.551833359	0.029702336	-	11267	grey	-0.325372771	FALSE	SNF8
Region_7870	chr17	48958554	48968596	10042	6	28	43.39393939	8.102950627	0.823468238	0.068674212	-	2695	grey	0.131046587	FALSE	GIP
Region_7871	chr17	48997385	49056145	58760	22	145	168.469697	11.73714642	0.81010506	0.019610972	+	10642	grey	-0.022852204	FALSE	IGF2BP1
Region_7872	chr17	49132460	49176840	44380	13	79	97.18181818	9.956689426	0.874378383	0.043305126	+	124872	grey	-0.134955945	FALSE	B4GALNT2
Region_7873	chr17	49202791	49210574	7783	3	10	22.77272727	4.783932899	0.897159883	0.054253488	-	2793	grey	0.091943345	FALSE	GNGT2
Region_7874	chr17	49210411	49223225	12814	13	70	90.96969697	8.703614982	0.717185084	0.038978676	+	51225	grey	-0.110893999	FALSE	ABI3

Region_7875	chr17	49223366	49230787	7421	8	41	54.62121212	7.517702496	0.398049836	0.046222025	-	162466	grey	-0.082269844	FALSE	PHOSPHO1
Region_7876	chr17	49230853	49318011	87158	33	184	222.8636364	20.64415459	0.763822739	0.018380488	+	124871	grey	0.086360973	FALSE	FLJ40194
Region_7877	chr17	49289206	49362473	73267	18	98	123.4545455	11.07343574	0.800980882	0.019192979	-	22834	grey	0.266239553	FALSE	ZNF652
Region_7878	chr17	49361150	49476988	115838	32	216	257.6363636	18.60160156	0.80432149	0.017847631	+	102724596	grey	-0.183761766	FALSE	ZNF652-AS1
Region_7879	chr17	49404049	49414905	10856	3	16	26.54545455	4.743195347	0.956570171	0.049608866	-	5245	grey	0.169478559	FALSE	PHB1
Region_7880	chr17	49495293	49515008	19715	6	27	41.07575758	6.622364454	0.315711636	0.038015493	+	4804	grey	0.225237607	FALSE	NGFR
Region_7881	chr17	49505657	49574064	68407	11	59	76.10606061	8.481888012	0.796892915	0.037329596	-	100288866	grey	0.059844719	FALSE	NGFR-AS1
Region_7882	chr17	49598884	49678163	79279	10	64	84.1969697	9.862469182	0.925508996	0.026089571	-	8405	grey	0.087188611	FALSE	SPOP
Region_7883	chr17	49710332	49789180	78848	10	64	82.60606061	8.577033355	0.685300353	0.037974978	-	81558	grey	-0.161320905	FALSE	FAM117A
Region_7884	chr17	49788648	49835026	46378	13	95	117.8333333	10.58675999	0.886533463	0.018153125	+	11143	grey	-0.4954084	FALSE	KAT7
Region_7885	chr17	50055968	50090481	34513	6	29	44.46969697	6.132435072	0.847308807	0.066489005	+	3675	grey	-0.316210078	FALSE	ITGA3
Region_7886	chr17	50094737	50112152	17415	3	10	20.22727273	4.270814927	0.580223221	0.105861086	+	5164	grey	0.077939619	FALSE	PDK2
Region_7887	chr1	50110040	50130160	20120	4	12	23.9545454	4.99055051	0.48851474	0.03617629	-	201191	grey	-0.02374530	FALSE	SAMD14

	7						5	1	7	6				3		
Region_7888	chr17	50184101	50201632	17531	5	22	34.04545455	5.668894393	0.761068879	0.050182706	-	1277	grey	0.273397794	FALSE	COL1A1
Region_7889	chr17	50346126	50363138	17012	3	12	21.54545455	4.232409091	0.374130723	0.050234753	+	64132	grey	0.046381579	FALSE	XYLT2
Region_7890	chr17	50375059	50397523	22464	3	11	21.01515152	4.862707142	0.770342768	0.081152114	-	55379	grey	-0.008707566	FALSE	LRRC59
Region_7891	chr17	50426158	50474845	48687	9	48	69.5	9.459630827	0.841241729	0.045987165	+	80221	grey	-0.272082027	FALSE	ACSF2
Region_7892	chr17	50508384	50531501	23117	4	18	30.78787879	5.234545463	0.695352289	0.054380223	+	84073	grey	-0.15484554	FALSE	MYCBPAP
Region_7893	chr17	50532682	50543750	11068	3	16	26.62121212	4.429846851	0.653344248	0.026543466	+	55040	grey	-0.334748493	FALSE	EPN3
Region_7894	chr17	50560715	50627474	66759	13	76	97.24242424	9.12487826	0.706962393	0.048559222	+	8913	grey	0.080557362	FALSE	CACNA1G
Region_7895	chr17	50634777	50692253	57476	12	76	98.65151515	9.304246217	0.889107901	0.042046956	+	8714	grey	-0.098574244	FALSE	ABCC3
Region_7896	chr17	50693198	50707914	14716	3	17	26.89393939	4.571243857	0.967281106	0.041799223	-	91369	grey	0.120214883	FALSE	ANKRD40
Region_7897	chr17	50719565	50756219	36654	7	44	60.09090909	6.501425341	0.974742613	0.020836433	+	51747	grey	-0.327367524	FALSE	LUC7L3
Region_7898	chr17	50834650	50842353	7703	3	14	23.31818182	4.83308817	0.910792855	0.060773576	+	124857	grey	0.109826848	FALSE	WFIKKN2
Region_7899	chr17	50866679	50910774	44095	9	58	80.56060606	9.089518542	0.74373038	0.030019276	+	400604	grey	0.11769591	FALSE	TOB1-AS1

Region_7900	chr17	50962174	51120868	158694	19	112	146.1515152	13.27605412	0.859316791	0.018778008	-	9043	grey	0.36149932	FALSE	SPAG9
Region_7901	chr17	51153590	51171744	18154	5	20	31.96969697	5.412150708	0.37397044	0.037024677	+	654364	grey	-0.276148007	FALSE	NME1-NME2
Region_7902	chr17	51165435	51171744	6309	4	12	22.03030303	4.444425019	0.468269728	0.049165212	+	4831	grey	-0.297550938	FALSE	NME2
Region_7903	chr17	51177425	51260163	82738	10	50	68.12121212	7.502851051	0.754386075	0.039987214	-	54799	grey	0.258270565	FALSE	MBTD1
Region_7904	chr17	51260546	51297936	37390	9	51	78.74242424	10.14557214	0.613360089	0.027126892	+	51096	grey	0.124280761	FALSE	UTP18
Region_7905	chr17	51336646	51521401	184755	17	116	138.3787879	11.06590766	0.908636364	0.030033451	+	440446	grey	0.007661075	FALSE	LINC02072
Region_7906	chr17	51336737	51445802	109065	11	78	93.71212121	8.347759076	0.895024648	0.035564074	+	105371828	grey	0.008241939	FALSE	LINC02073
Region_7907	chr17	51630313	52160017	529704	41	317	355.7575758	22.40677137	0.777296302	0.022092658	-	56934	grey	0.308760177	FALSE	CA10
Region_7908	chr17	52390515	52535701	145186	4	19	30.07575758	5.889522917	0.915000761	0.042828505	+	105371830	grey	-0.263021643	FALSE	LINC01982
Region_7909	chr17	52862121	52899588	37467	4	14	27.62121212	6.536913295	0.929037929	0.060501378	+	105371831	grey	-0.046475434	FALSE	LINC02089
Region_7910	chr17	53276760	54066825	790065	86	637	712.6515152	31.75845977	0.844595231	0.018110159	-	107985001	grey	0.032247826	FALSE	LOC107985001
Region_7911	chr17	54899387	54961956	62569	15	114	137.1666667	12.473459	0.920636453	0.023750689	+	10040	grey	-0.444159846	FALSE	TOM1L1
Region_7912	chr1	54968727	55173632	204905	41	293	334.151515	19.1256914	0.83191292	0.02276202	+	252983	grey	-0.08300446	FALSE	STXBP4

	7						2	8	2	7				9		
Region_7913	chr17	55264960	55325187	60227	9	59	78.09090909	8.770101785	0.620880722	0.040006245	+	3131	grey	-0.182808186	FALSE	HLF
Region_7914	chr17	55392622	55421924	29302	5	27	42.06060606	6.31208557	0.751635611	0.042444816	-	23531	grey	-0.063205086	FALSE	MMD
Region_7915	chr17	55448433	55511444	63011	23	133	169.5606061	15.7783969	0.899489792	0.027190463	-	101927367	grey	0.412382987	FALSE	SMIM36
Region_7916	chr17	55526964	55561091	34127	7	35	51.51515152	6.906501785	0.925914794	0.04646071	-	105371833	grey	-0.038415066	FALSE	
Region_7917	chr17	55612948	55642230	29282	4	15	28.65151515	5.954939577	0.732371257	0.063678681	+	101927389	grey	0.158738998	FALSE	LOC101927389
Region_7918	chr17	55751051	55842830	91779	14	113	134	10.62218288	0.873227926	0.037457177	+	58488	grey	-0.33653681	FALSE	PCTP
Region_7919	chr17	55882301	56517016	634715	133	1006	1112.272727	41.77689662	0.897544845	0.012402921	+	162282	grey	-0.159516629	FALSE	ANKFN1
Region_7920	chr17	56593699	56595611	1912	3	14	21.46969697	4.199705839	0.010628471	0.023995674	+	9241	grey	-0.287859896	FALSE	NOG
Region_7921	chr17	56791913	56838773	46860	39	213	258.7727273	28.23407404	0.215195047	0.009360183	-	339210	grey	0.224604194	FALSE	C17orf67
Region_7922	chr17	56834107	56869567	35460	18	108	137.8787879	13.32841401	0.533622117	0.017841422	+	8526	grey	0.303144747	FALSE	DGKE
Region_7923	chr17	56836387	56914080	77693	19	137	164.7727273	12.35101423	0.909958576	0.023692276	-	7706	grey	-0.103333802	FALSE	TRIM25
Region_7924	chr17	56938199	56961050	22851	9	49	71.75757576	10.90386044	0.378235902	0.029438012	-	8161	grey	-0.143924126	FALSE	COIL

Region_7925	chr17	56978129	57006768	28639	4	21	35.03030303	5.914700583	0.606788575	0.063940579	+	59342	grey	-0.25673643	FALSE	SCPEP1
Region_7926	chr17	57085092	57121346	36254	16	105	125.7272727	10.7109512	0.687796372	0.029880437	+	8165	grey	-0.038155538	FALSE	AKAP1
Region_7927	chr17	57255851	57684689	428838	177	1378	1474.515152	47.96833649	0.818965324	0.011660819	+	124540	grey	-0.030315441	FALSE	MSI2
Region_7928	chr17	57834781	57850056	15275	4	13	24.83333333	4.609633169	0.856609521	0.081808302	-	51649	grey	-0.280474791	FALSE	MRPS23
Region_7929	chr17	57861243	57955412	94169	39	233	284.469697	19.50165255	0.679430608	0.01948829	-	404093	grey	0.171757642	FALSE	CUEDC1
Region_7930	chr17	57912333	57922581	10248	7	40	56.66666667	7.672795766	0.860099929	0.041684056	+	107985065	grey	0.148517502	FALSE	LOC107985065
Region_7931	chr17	57971547	57988259	16712	3	11	23.48484848	5.372987779	0.340083634	0.061030095	-	7716	grey	0.02001175	FALSE	VEZF1
Region_7932	chr17	57989038	58007346	18308	6	43	58.62121212	7.470485828	0.381065465	0.056975372	-	6426	grey	0.280689604	FALSE	SRSF1
Region_7933	chr17	58083419	58095542	12123	10	52	71.81818182	9.200182424	0.576109306	0.02678576	+	140735	grey	-0.141975209	FALSE	DYNLL2
Region_7934	chr17	58148384	58159585	11201	6	21	41.72727273	7.935403542	0.660520666	0.034224653	+	26689	grey	-0.173719457	FALSE	OR4D1
Region_7935	chr17	58192726	58205174	12448	21	132	170.1666667	16.70889522	0.857140222	0.028579097	+	8288	grey	0.087281974	FALSE	EPX
Region_7936	chr17	58205441	58219605	14164	13	73	92.57575758	8.74785355	0.635676302	0.039073824	-	54903	grey	0.089754971	FALSE	MKS1
Region_7937	chr1	58218548	58268518	49970	20	120	150.636363	13.4079613	0.61253561	0.03409792	+	4025	grey	0.211445617	FALSE	LPO

	7						6	1	6	4						
Region_7938	chr17	58219375	58233479	14104	7	44	63.93939394	8.964680762	0.802985986	0.036805633	+	105371841	grey	0.227311718	FALSE	LOC105371841
Region_7939	chr17	58269855	58280935	11080	6	30	43.51515152	6.293445734	0.272710626	0.087388717	-	4353	grey	-0.106388089	FALSE	MPO
Region_7940	chr17	58301231	58328795	27564	9	40	58.09090909	7.548259652	0.593861001	0.052933907	-	9256	grey	0.002089464	FALSE	TSPOAP1
Region_7941	chr17	58324472	58415766	91294	23	128	162.4848485	13.77873772	0.408890029	0.02671369	+	100506779	grey	-0.13472593	FALSE	TSPOAP1-AS1
Region_7942	chr17	58345175	58353093	7918	4	18	31.24242424	5.569313191	0.329052839	0.053177891	-	6827	grey	0.065251489	FALSE	SUPT4H1
Region_7943	chr17	58345206	58417595	72389	14	75	96.84848485	9.343434427	0.656999402	0.039107913	-	54894	grey	-0.029674593	FALSE	RNF43
Region_7944	chr17	58420167	58488408	68241	18	121	149.8030303	14.70344773	0.707517007	0.030730424	-	124535	grey	-0.025373219	FALSE	HSF5
Region_7945	chr17	58489529	58517905	28376	9	59	76.8030303	8.103597866	0.581071482	0.034311916	-	9110	grey	-0.023313881	FALSE	MTMR4
Region_7946	chr17	58519837	58557799	37962	6	30	42.04545455	5.69866872	0.727570217	0.036704639	+	101927688	grey	-0.019283212	FALSE	SEPTIN4-AS1
Region_7947	chr17	58520250	58544368	24118	6	30	42.04545455	5.69866872	0.727570217	0.036704639	-	5414	grey	-0.019283212	FALSE	SEPTIN4
Region_7948	chr17	58556678	58692055	135377	60	400	464.7878788	26.10246976	0.896531836	0.012634847	-	56155	grey	0.193723465	FALSE	TEX14
Region_7949	chr17	58660424	58692045	31621	24	149	179.7424242	13.38691739	0.906008603	0.02175235	-	645545	grey	0.011862179	FALSE	IGBP1C

Region_7950	chr17	58692573	58735611	43038	20	123	155.8181818	11.78774995	0.969948584	0.014190657	+	5889	grey	-0.106054433	FALSE	RAD51C
Region_7951	chr17	58755854	58985179	229325	49	314	358.2727273	22.73766476	0.860061785	0.017677526	+	22843	grey	0.130246732	FALSE	PPM1E
Region_7952	chr17	58982638	59106921	124283	25	174	203.6515152	14.02579209	0.925192207	0.023208831	-	4591	grey	0.196536534	FALSE	TRIM37
Region_7953	chr17	59109857	59155260	45403	12	68	88.93939394	9.203475577	0.725406594	0.04072614	-	348235	grey	0.142933377	FALSE	SKA2
Region_7954	chr17	59155732	59206709	50977	14	87	112.9545455	10.64740029	0.949831062	0.020674176	+	55771	grey	-0.140857845	FALSE	PRR11
Region_7955	chr17	59197566	59215239	17673	6	29	41.86363636	5.360500446	0.781148642	0.039522705	+	55181	grey	-0.190854447	FALSE	SMG8
Region_7956	chr17	59220467	59275970	55503	14	74	97.63636364	9.066652955	0.877328539	0.026157851	+	284161	grey	0.110127135	FALSE	GDPD1
Region_7957	chr17	59331655	59401729	70074	23	157	188.1666667	14.88063619	0.748198975	0.027586231	+	388403	grey	-0.314429554	FALSE	YPEL2
Region_7958	chr17	59422088	59526946	104858	63	461	519.5909091	28.5850834	0.875378097	0.015440742	-	101927728	grey	-0.062240326	FALSE	LINC01476
Region_7959	chr17	59565558	59608345	42787	8	34	53.72727273	7.711221503	0.826697414	0.04045122	+	79665	grey	-0.026538998	FALSE	DHX40
Region_7960	chr17	59619684	59696956	77272	9	48	68.77272727	8.893896705	0.968617953	0.026734684	+	1213	grey	-0.027576892	FALSE	CLTC
Region_7961	chr17	59674636	59707626	32990	5	31	42.07575758	6.877635117	0.980381264	0.023730862	-	51651	grey	-0.297991195	FALSE	PTRH2
Region_7962	chr1	59707648	59842255	134607	23	144	171.575757	13.6054973	0.90796098	0.02884812	+	81671	grey	-0.34117420	FALSE	VMP1

	7						6	1	3	1				7		
Region_7963	chr17	59859479	59892945	33466	8	47	64.71212121	7.191373361	0.956606843	0.028997276	-	51174	grey	-0.025869318	FALSE	TUBD1
Region_7964	chr17	59893046	59950574	57528	16	95	117.7424242	10.64433487	0.97164898	0.019368257	+	6198	grey	0.020022252	FALSE	RPS6KB1
Region_7965	chr17	59940908	59996972	56064	15	87	107.3181818	10.37476093	0.83722726	0.027808652	+	101927755	grey	-0.024522767	FALSE	RNFT1-DT
Region_7966	chr17	59952240	59964761	12521	6	28	39.16666667	6.335694567	0.659084693	0.045405811	-	51136	grey	-0.207961338	FALSE	RNFT1
Region_7967	chr17	59962321	60018975	56654	20	118	154.2272727	12.61597943	0.854232452	0.023938573	-	653645	grey	-0.299245019	FALSE	TBC1D3P1-DHX40P1
Region_7968	chr17	60041008	60078922	37914	4	13	25.6969697	5.056510493	0.975452441	0.028751174	-	63897	grey	0.050816698	FALSE	HEATR6
Region_7969	chr17	60083562	60091885	8323	3	14	31.06060606	6.146615799	0.937159631	0.060324218	-	645638	grey	-0.073434173	FALSE	WFDC21P
Region_7970	chr17	60122642	60135743	13101	4	23	32.77272727	5.609868926	0.480563203	0.028825709	-	100996660	grey	0.003966791	FALSE	LOC100996660
Region_7971	chr17	60149942	60170899	20957	15	81	107.2575758	12.6604297	0.468509803	0.032041867	+	762	grey	-0.044280319	FALSE	CA4
Region_7972	chr17	60177327	60422470	245143	60	451	510.6212121	24.01074449	0.939387761	0.007920586	-	84669	grey	0.178661645	FALSE	USP32
Region_7973	chr17	60392429	60431426	38997	7	35	54.13636364	6.646887909	0.921170468	0.042422903	+	124773	grey	0.174676939	FALSE	CHCT1
Region_7974	chr17	60443158	60526242	83084	21	139	168.6969697	14.32906642	0.97591385	0.01345765	-	10513	grey	0.170322341	FALSE	APPBP2

Region_7975	chr17	60526293	60550798	24505	10	59	81.63636364	9.33749491	0.872132792	0.024277155	+	120017341	grey	-0.038033011	FALSE	APPBP2-DT
Region_7976	chr17	60564547	60586623	22076	10	56	74.03030303	8.406901372	0.94765391	0.034481617	+	388406	grey	-0.235605765	FALSE	LINC01999
Region_7977	chr17	60600193	60666280	66087	18	122	153.4393939	13.76297015	0.865938679	0.011049067	+	8493	grey	0.129030874	FALSE	PPM1D
Region_7978	chr17	60677453	61392838	715385	140	1083	1162.469697	42.04511119	0.945473466	0.011257966	+	54828	grey	-0.292385976	FALSE	BCAS3
Region_7979	chr17	61361668	61400243	38575	14	98	126.1515152	13.58873111	0.728573303	0.031056209	-	101927855	grey	-0.033334165	FALSE	LOC101927855
Region_7980	chr17	61393455	61411555	18100	13	60	93.09090909	11.35676995	0.090612348	0.039390408	-	103689912	grey	-0.06361248	FALSE	TBX2-AS1
Region_7981	chr17	61399843	61409466	9623	8	34	57.72727273	7.999125826	0.069248295	0.040988348	+	6909	grey	0.112378568	FALSE	TBX2
Region_7982	chr17	61452404	61485110	32706	12	76	103.7575758	11.46105191	0.477732047	0.043671423	+	9496	grey	-0.001184907	FALSE	TBX4
Region_7983	chr17	61679139	61863559	184420	43	311	363.5	24.19933248	0.959537485	0.011606469	-	83990	grey	0.083245869	FALSE	BRIP1
Region_7984	chr17	61865367	61928016	62649	25	150	184.7727273	15.73999849	0.929762909	0.015573453	-	57508	grey	0.020227854	FALSE	INTS2
Region_7985	chr17	61942605	62065278	122673	35	249	305.0757576	20.88526209	0.939319922	0.013730906	-	9969	grey	-0.369353612	FALSE	MED13
Region_7986	chr17	62264705	62275655	10950	3	10	19.09090909	5.214557353	0.920654142	0.059928386	-	440452	grey	0.000166494	FALSE	TBC1D3P2
Region_7987	chr1	62343941	62358249	14308	4	17	35.5303030	6.29827741	0.92716279	0.05822058	+	10537193	grey	-0.10679639	FALSE	LOC105371936

	7						3	3	4	9		6		2		
Region_7988	chr17	62355864	62416480	60616	12	64	89.63636364	9.194708116	0.927290077	0.031544266	+	146779	grey	-0.061624962	FALSE	EFCAB3
Region_7989	chr17	62458658	62615481	156823	37	224	271.7424242	17.86643661	0.892417605	0.013821843	+	11011	grey	-0.098088462	FALSE	TLK2
Region_7990	chr17	62627670	62693597	65927	34	196	230.7575758	16.42424156	0.89598823	0.015782904	+	9902	grey	-0.14268132	FALSE	MRC2
Region_7991	chr17	62699244	62754973	55729	27	193	226.6818182	15.83682086	0.789368859	0.023460563	+	105371855	grey	-0.270579144	FALSE	LOC105371855
Region_7992	chr17	62701314	62808344	107030	50	369	421.3333333	23.97712585	0.855841114	0.016907621	-	162333	grey	-0.265457199	FALSE	MARCHF10
Region_7993	chr17	62808500	62836371	27871	11	75	93.96969697	9.519162369	0.921879158	0.036110933	+	101927877	grey	-0.073870337	FALSE	MARCHF10-DT
Region_7994	chr17	62966235	63427703	461468	86	625	726.5151515	30.58518617	0.930189171	0.014884305	+	26115	grey	0.175218985	FALSE	TANC2
Region_7995	chr17	63193931	63339053	145122	25	197	220.1060606	12.19347718	0.950603326	0.019882026	-	105371857	grey	-0.001758335	FALSE	
Region_7996	chr17	63381231	63414312	33081	3	18	27.13636364	5.273697765	0.963501314	0.038536268	-	105371856	grey	-0.210108414	FALSE	LOC105371856
Region_7997	chr17	63432304	63446354	14050	10	59	78.63636364	9.271355993	0.566752662	0.033983758	-	1534	grey	0.386503126	FALSE	CYB561
Region_7998	chr17	63477061	63521848	44787	14	75	92.34848485	10.62579217	0.888794468	0.037109948	+	1636	grey	0.143265928	FALSE	ACE
Region_7999	chr17	63523334	63548977	25643	19	115	153.6515152	13.46504583	0.952040964	0.023024583	+	81033	grey	0.171553248	FALSE	KCNH6

Region_8000	chr17	63550222	63608347	58125	34	272	307.6666667	18.60383197	0.915985436	0.01325315	+	10238	grey	-0.01530487	FALSE	DCAF7
Region_8001	chr17	63600882	63608365	7483	5	38	53.25757576	7.17755177	0.978642604	0.018055217	+	51204	grey	-0.067483876	FALSE	TACO1
Region_8002	chr17	63622415	63696305	73890	14	80	101.4545455	8.532619434	0.556141759	0.034531124	+	4215	grey	-0.287212631	FALSE	MAP3K3
Region_8003	chr17	63682336	63752097	69761	19	106	141.0909091	12.01886629	0.660785393	0.036140455	-	92335	grey	0.023373583	FALSE	STRADA
Region_8004	chr17	63695888	63701172	5284	5	16	29.90909091	6.671932652	0.171862817	0.049504495	-	80774	grey	-0.040258712	FALSE	LIMD2
Region_8005	chr17	63700847	63702670	1823	3	11	21.46969697	4.801344994	0.033342702	0.040807941	+	729683	grey	-0.059586421	FALSE	LOC729683
Region_8006	chr17	63745255	63776351	31096	5	18	34.36363636	6.095739656	0.370948227	0.033508358	-	57003	grey	-0.030006029	FALSE	CCDC47
Region_8007	chr17	63773603	63819317	45714	10	68	85.33333333	8.931075107	0.705892405	0.034543943	+	11325	grey	-0.16678979	FALSE	DDX42
Region_8008	chr17	63832081	63842698	10617	4	21	34.83333333	5.655720952	0.697225539	0.049821956	-	6603	grey	-0.143049121	FALSE	SMARCD2
Region_8009	chr17	63849292	63864379	15087	5	35	46.45454545	6.15987467	0.875056656	0.043139808	+	146771	grey	-0.186491832	FALSE	TCAM1P
Region_8010	chr17	63917200	63931385	14185	6	44	57.51515152	7.325912753	0.933229356	0.037634179	-	2688	grey	0.04036185	FALSE	GH1
Region_8011	chr17	63928738	63932336	3598	3	19	29.83333333	5.501281902	0.651989219	0.022650512	-	974	grey	0.144354133	FALSE	CD79B
Region_8012	chr1	63938554	63972918	34364	10	50	67.3333333	7.61442625	0.78251117	0.04362867	-	6329	grey	-0.01001616	FALSE	SCN4A

	7						3	4	6	7				6		
Region_8013	chr17	63998351	64004305	5954	3	10	21.03030303	4.11739162	0.631066801	0.086752178	+	92340	grey	-0.04676165	FALSE	PRR29
Region_8014	chr17	64039080	64130144	91064	18	130	153.4393939	10.74477159	0.763545727	0.02328276	-	2081	grey	-0.209232745	FALSE	ERN1
Region_8015	chr17	64147227	64263260	116033	26	186	215.8333333	13.60834962	0.925686903	0.016952739	-	55852	grey	0.111282901	FALSE	TEX2
Region_8016	chr17	64319415	64413776	94361	35	225	265.1969697	18.73804825	0.734526057	0.024486478	-	5175	grey	0.107030496	FALSE	PECAM1
Region_8017	chr17	64449037	64468643	19606	5	27	38.36363636	5.35691175	0.955072419	0.038480036	+	284021	grey	0.084693109	FALSE	MILR1
Region_8018	chr17	64477785	64497054	19269	5	26	38.6969697	5.938064168	0.945932166	0.041870606	-	11232	grey	0.351207243	FALSE	POLG2
Region_8019	chr17	64498254	64507712	9458	4	24	34.31818182	5.338277138	0.003754348	0.011954488	-	1655	grey	-0.075894095	FALSE	DDX5
Region_8020	chr17	64506865	64542461	35596	8	48	73.24242424	8.346460523	0.589040103	0.027553681	+	90799	grey	-0.033372317	FALSE	CEP95
Region_8021	chr17	64542282	64662307	120025	31	227	259.6515152	16.83173964	0.907106007	0.01699207	-	64750	grey	0.030686752	FALSE	SMURF2
Region_8022	chr17	64749663	64781707	32044	26	167	200.2121212	15.78559728	0.305142561	0.023216572	-	109286553	grey	0.113180411	FALSE	ARHGAP27P1-BPTFP1-KPNA2P3
Region_8023	chr17	64779259	64837207	57948	31	190	223.6363636	15.60338855	0.685548322	0.021195901	-	440456	grey	-0.250607693	FALSE	PLEKHM1P1
Region_8024	chr17	64892729	64910180	17451	5	33	63.39393939	14.59491881	0.764409001	0.050435731	+	105376844	grey	0.19214328	FALSE	LOC105376844

Region_8025	chr17	64966550	64975630	9080	4	27	34.48484848	4.661602314	0.441012954	0.052927728	-	201283	grey	0.053324489	FALSE	AMZ2P1
Region_8026	chr17	65009289	65056740	47451	15	104	137.6212121	12.63787367	0.643771993	0.027567273	-	10672	grey	0.337134145	FALSE	GNA13
Region_8027	chr17	65100812	65119585	18773	3	14	25.53030303	5.254213538	0.659767695	0.077479242	+	100507002	grey	-0.15084691	FALSE	LOC100507002
Region_8028	chr17	65100986	65227703	126717	24	152	197.2424242	12.66620046	0.763106481	0.028701652	+	8787	grey	0.329309478	FALSE	RGS9
Region_8029	chr17	65528563	65641033	112470	40	250	282.1212121	16.50316068	0.820053187	0.022853358	-	8313	grey	0.122836467	FALSE	AXIN2
Region_8030	chr17	65635537	66192133	556596	113	849	940.0151515	30.95281393	0.858588914	0.018857816	-	201134	grey	0.154514112	FALSE	CEP112
Region_8031	chr17	66212033	66256525	44492	12	74	95.65151515	9.330664787	0.884193786	0.028685277	-	350	grey	0.212819893	FALSE	APOH
Region_8032	chr17	66302613	66810743	508130	133	1024	1094.121212	35.65756588	0.864484871	0.01191288	+	5578	grey	-0.348142979	FALSE	PRKCA
Region_8033	chr17	66835117	66894751	59634	7	29	46.24242424	6.584505369	0.852497243	0.068295524	+	27091	grey	-0.087999467	FALSE	CACNG5
Region_8034	chr17	66964707	67033398	68691	15	79	99.04545455	9.342099613	0.759294722	0.031354702	+	27092	grey	0.216183484	FALSE	CACNG4
Region_8035	chr17	67044554	67056797	12243	3	10	19.40909091	4.607610002	0.954064791	0.048983674	+	786	grey	0.324289535	FALSE	CACNG1
Region_8036	chr17	67070444	67245989	175545	24	151	187.6212121	13.92370989	0.922012739	0.02063871	-	9931	grey	-0.256632861	FALSE	HELZ
Region_8037	chr1	67244282	67273503	29221	6	30	47.2424242	7.86239463	0.88443498	0.0630709	+	10192802	grey	0.08561193	FALSE	HELZ-AS1

	7						4	6	8			1				
Region_8038	chr17	67337916	67366605	28689	8	43	64.78787879	9.076622807	0.506947608	0.043538422	-	5718	grey	-0.007217912	FALSE	PSMD12
Region_8039	chr17	67377281	67697256	319975	74	526	591.5454545	28.30013961	0.840758701	0.014530006	+	26207	grey	-0.116480147	FALSE	PITPNC1
Region_8040	chr17	67717931	67744531	26600	4	19	28.1969697	4.694414675	0.778896048	0.069754716	+	25926	grey	-0.208468974	FALSE	NOL11
Region_8041	chr17	67825503	67984378	158875	19	111	133.1363636	11.79554853	0.900846835	0.020441716	+	2186	grey	0.258127203	FALSE	BPTF
Region_8042	chr17	68035636	68047364	11728	4	18	31.46969697	5.309555094	0.250495895	0.024931873	+	3838	grey	0.01720765	FALSE	KPNA2
Region_8043	chr17	68101871	68161227	59356	15	93	115.030303	10.00917994	0.877963974	0.03329529	+	100499466	grey	-0.237327355	FALSE	LINC00674
Region_8044	chr17	68205481	68257712	52231	9	43	70.81818182	11.60226648	0.686655963	0.030599125	+	51321	grey	0.226399385	FALSE	AMZ2
Region_8045	chr17	68259182	68493670	234488	57	418	462.469697	21.30671234	0.857560719	0.013525494	+	22901	grey	0.284736022	FALSE	ARSG
Region_8046	chr17	68267026	68291267	24241	8	43	59.36363636	8.163024258	0.971258949	0.032687849	-	9120	grey	-0.108899631	FALSE	SLC16A6
Region_8047	chr17	68413623	68551319	137696	29	175	216.2727273	16.02934372	0.870648566	0.020037015	+	5573	grey	-0.16287375	FALSE	PRKAR1A
Region_8048	chr17	68420948	68457513	36565	7	40	54.03030303	7.153201966	0.966307329	0.027160801	-	55062	grey	0.077555877	FALSE	WIPI1
Region_8049	chr17	68535113	68601367	66254	18	95	122.3333333	11.07086729	0.769608483	0.03238116	-	54757	grey	-0.086944028	FALSE	FAM20A

Region_8050	chr17	68557516	68582577	25061	3	11	18.42424242	4.374965867	0.89233458	0.088034649	+	105371873	grey	-0.167794653	FALSE	
Region_8051	chr17	68628103	68679606	51503	5	26	40.42424242	6.219227273	0.903965559	0.042516952	+	101928104	grey	0.15543102	FALSE	LINC01482
Region_8052	chr17	68867289	68955392	88103	23	180	208.4848485	12.30414867	0.882871382	0.02856056	-	10351	grey	-0.049214301	FALSE	ABCA8
Region_8053	chr17	68944531	69034926	90395	8	40	56.65151515	7.742971849	0.833750097	0.039582005	+	104355297	grey	-0.035048885	FALSE	ABCA9-AS1
Region_8054	chr17	68974488	69060949	86461	6	29	43.5	7.692653838	0.907349103	0.050387294	-	10350	grey	-0.072063309	FALSE	ABCA9
Region_8055	chr17	69078702	69141895	63193	4	23	33.15151515	5.435615949	0.892897962	0.060517995	-	23460	grey	0.11494145	FALSE	ABCA6
Region_8056	chr17	69147214	69244846	97632	8	48	62.98484848	8.192853216	0.950427069	0.029241199	-	10349	grey	0.075447035	FALSE	ABCA10
Region_8057	chr17	69244311	69327244	82933	12	81	104.1818182	10.24606355	0.795838453	0.024108461	-	23461	grey	-0.215599715	FALSE	ABCA5
Region_8058	chr17	69414697	69553865	139168	15	112	138.2575758	11.93612565	0.823413924	0.021257279	+	5608	grey	0.08509629	FALSE	MAP2K6
Region_8059	chr17	69577251	69903000	325749	31	218	263.1060606	16.12273005	0.823982799	0.029837354	+	101928122	grey	0.11342525	FALSE	LINC01483
Region_8060	chr17	69961568	69994845	33277	5	24	35.6969697	5.62950933	0.872626252	0.078070638	+	102723487	grey	-0.196820204	FALSE	LINC01497
Region_8061	chr17	70017324	70128859	111535	25	177	218.6212121	16.81552868	0.856739594	0.031527707	-	112267896	grey	0.215110593	FALSE	LOC112267896
Region_8062	chr1	70053429	70135608	82179	23	181	213.606060	16.0938390	0.85276799	0.03279036	+	3773	grey	0.215167439	FALSE	KCNJ16

	7						6	4	7	6						
Region_8063	chr17	70166961	70169404	2443	4	21	32.6969697	5.775198157	0.04058279	0.029801448	-	400617	grey	-0.145697096	FALSE	KCNJ2-AS1
Region_8064	chr17	70168673	70180044	11371	9	53	76.1969697	8.054085818	0.494499284	0.029781361	+	3759	grey	-0.366234288	FALSE	KCNJ2
Region_8065	chr17	71097774	71202203	104429	26	193	233.3181818	15.55057168	0.803513572	0.044919243	-	101928165	grey	-0.010926214	FALSE	CASC17
Region_8066	chr17	71829870	71871750	41880	10	47	65.54545455	7.636313686	0.887494235	0.039807356	-	107985015	grey	-0.060130688	FALSE	LOC107985015
Region_8067	chr17	72021851	72220948	199097	70	500	550	27.2803113	0.707564947	0.020099109	-	102723517	grey	0.232081156	FALSE	LOC102723517
Region_8068	chr17	72040714	72173583	132869	42	292	337.469697	22.99044597	0.635041996	0.024728677	-	400618	grey	0.048732903	FALSE	SOX9-AS1
Region_8069	chr17	72072333	72103035	30702	4	23	37.92424242	6.224790651	0.728573589	0.061162384	+	101928205	grey	0.219522429	FALSE	LINC02097
Region_8070	chr17	72121020	72126416	5396	6	19	34.65151515	6.034496791	0.209786374	0.063891219	+	6662	grey	0.070942167	FALSE	SOX9
Region_8071	chr17	72290091	72618438	328347	134	1031	1111.363636	34.53989282	0.855866437	0.015249677	-	100499467	grey	0.021424242	FALSE	LINC00673
Region_8072	chr17	72342692	72355136	12444	12	78	100.5	10.10445446	0.803125776	0.043814821	+	146795	grey	0.188102357	FALSE	LINC02003
Region_8073	chr17	72428267	72640472	212205	77	599	644.4090909	24.28293308	0.895233183	0.017276097	-	400619	grey	0.126076352	FALSE	LINC00511
Region_8074	chr17	72645949	73092712	446763	250	1923	2051.378788	56.32536391	0.905022032	0.006872554	-	201266	grey	0.277684092	FALSE	SLC39A11

Region_8075	chr17	73164977	73196593	31616	32	210	250.6818182	17.47979853	0.580328257	0.018296988	+	6752	grey	-0.152534443	FALSE	SSTR2
Region_8076	chr17	73193055	73208507	15452	15	81	106.4393939	11.21829383	0.646043452	0.029176963	+	9382	grey	0.078203137	FALSE	COG1
Region_8077	chr17	73207353	73236753	29400	15	87	112.3484848	11.99037104	0.608580269	0.02214553	-	84923	grey	-0.063753359	FALSE	VCF1
Region_8078	chr17	73232233	73248959	16726	13	80	99.31818182	9.684498463	0.7586563	0.060266413	+	55028	grey	-0.044639488	FALSE	C17orf80
Region_8079	chr17	73248449	73262352	13903	14	85	114.0909091	8.97813973	0.829575955	0.02802584	-	642843	grey	0.168899189	FALSE	CPSF4L
Region_8080	chr17	73283624	73312005	28381	26	118	159.6060606	16.77441164	0.546949969	0.02603695	-	23580	grey	-0.026873121	FALSE	CDC42EP4
Region_8081	chr17	73334384	73644445	310061	225	1579	1721.045455	57.90162262	0.846662684	0.012969959	-	54549	grey	-0.014561885	FALSE	SDK2
Region_8082	chr17	73513469	73521407	7938	7	22	41.53030303	7.750374079	0.928443998	0.052572424	+	101928251	grey	-0.161952886	FALSE	LOC101928251
Region_8083	chr17	73737854	73756932	19078	20	152	174.6666667	13.15392113	0.792205144	0.056819297	+	100134391	grey	-0.099836793	FALSE	LOC100134391
Region_8084	chr17	73749270	73828520	79250	47	335	380.3787879	19.55722582	0.883302162	0.026374102	-	283982	grey	0.047504587	FALSE	LINC00469
Region_8085	chr17	73786675	73800966	14291	3	13	19.90909091	4.131407928	0.845582518	0.104095465	+	400620	grey	0.084584054	FALSE	LINC02092
Region_8086	chr17	73912190	73931436	19246	6	30	50.18181818	8.368271747	0.652668883	0.066100636	+	107985081	grey	0.116157655	FALSE	LOC107985081
Region_8087	chr1	73926421	73949906	23485	11	52	79.7878787	10.7051607	0.86782145	0.04829423	-	10537188	grey	0.098501059	FALSE	LOC105371888

	7						9	3	3	1		8				
Region_8088	chr17	74043452	74154212	110760	68	460	525.1969697	27.11696585	0.779329962	0.019754886	-	102723567	grey	-0.085799861	FALSE	LINC02074
Region_8089	chr17	74213571	74262020	48449	37	245	291.3333333	19.71437065	0.927592347	0.017096477	+	94015	grey	0.069155589	FALSE	TTYH2
Region_8090	chr17	74274234	74314884	40650	32	212	249.4090909	16.19969351	0.692001558	0.030087434	+	64446	grey	0.071339148	FALSE	DNAI2
Region_8091	chr17	74326210	74355820	29610	33	205	254.9848485	20.44165613	0.601232544	0.023929807	+	124602	grey	0.124208588	FALSE	KIF19
Region_8092	chr17	74356416	74361868	5452	18	110	159.2121212	17.62036859	0.324277516	0.035913129	-	388419	grey	-0.148228483	FALSE	BTBD17
Region_8093	chr17	74367458	74372600	5142	6	37	48.66666667	5.957111673	0.934664338	0.039222229	+	350383	grey	0.001655614	FALSE	GPR142
Region_8094	chr17	74424851	74450580	25729	30	180	209.4393939	14.81384881	0.552138845	0.014690603	+	55890	grey	0.201807333	FALSE	GPRC5C
Region_8095	chr17	74466399	74484794	18395	18	107	139.1515152	14.12607238	0.755709777	0.021872026	+	11314	grey	-0.164554914	FALSE	CD300A
Region_8096	chr17	74521174	74531475	10301	17	117	150.030303	14.21480236	0.793135629	0.0317439	-	124599	grey	0.165373981	FALSE	CD300LB
Region_8097	chr17	74541073	74546115	5042	7	31	47.37878788	7.122586288	0.831843291	0.06496412	-	10871	grey	-0.010869971	FALSE	CD300C
Region_8098	chr17	74560701	74567343	6642	6	43	62.42424242	8.210800778	0.751768805	0.048683953	-	100130520	grey	-0.029356391	FALSE	CD300H
Region_8099	chr17	74579365	74592283	12918	11	62	102.3787879	11.40548472	0.786412123	0.0266805	-	100131439	grey	-0.092995005	FALSE	CD300LD

Region_8100	chr17	74584679	74594209	9530	4	23	37.77272727	5.994344654	0.732670008	0.054716342	+	146723	grey	-0.062375244	FALSE	CD300LD-AS1
Region_8101	chr17	74599820	74607246	7426	7	55	66.48484848	6.910955459	0.91799616	0.036827713	+	101928343	grey	0.024401782	FALSE	LOC101928343
Region_8102	chr17	74609885	74623738	13853	7	32	49.74242424	8.046615354	0.949869767	0.034706622	-	342510	grey	-0.097823847	FALSE	CD300E
Region_8103	chr17	74670077	74677624	7547	6	32	57.66666667	10.83773086	0.484047595	0.037690915	-	105371894	grey	0.073714418	FALSE	LOC105371894
Region_8104	chr17	74670578	74747335	76757	47	323	370.530303	21.8816535	0.792807722	0.016013226	+	326624	grey	-0.047419768	FALSE	RAB37
Region_8105	chr17	74694311	74712978	18667	11	64	79.92424242	9.071189568	0.820605653	0.041830117	-	146722	grey	-0.058836941	FALSE	CD300LF
Region_8106	chr17	74748628	74769353	20725	14	67	92.93939394	9.520631504	0.519786004	0.03612468	+	9368	grey	0.031471244	FALSE	NHERF1
Region_8107	chr17	74770529	74776367	5838	3	11	18.27272727	4.2482411	0.332314907	0.036768921	-	26151	grey	0.144722354	FALSE	NAT9
Region_8108	chr17	74776499	74839753	63254	45	316	356.1818182	18.5811394	0.822429109	0.01912851	+	54868	grey	-0.157002233	FALSE	TMEM104
Region_8109	chr17	74842023	74861504	19481	24	119	153.2424242	11.05936513	0.404113686	0.030257852	-	2905	grey	0.015361966	FALSE	GRIN2C
Region_8110	chr17	74862497	74873031	10534	18	94	132.3030303	16.25562123	0.826943767	0.03042999	-	2232	grey	-0.165054341	FALSE	FDXR
Region_8111	chr17	74877302	74893781	16479	12	56	88.63636364	11.02812679	0.67118231	0.033899524	-	283985	grey	-0.254146586	FALSE	FADS6
Region_8112	chr1	74916083	74923256	7173	6	32	47.6212121	7.18281100	0.74139977	0.08924874	-	124590	grey	-0.24769438	FALSE	USH1G

	7						2	2	9	8				2		
Region_8113	chr17	74922950	74933912	10962	14	65	87.51515152	10.67742756	0.445048383	0.031912699	+	92736	grey	0.173161517	FALSE	OTOP2
Region_8114	chr17	74935898	74949992	14094	11	66	84.48484848	8.790278589	0.716732488	0.033816834	+	347741	grey	-0.184580983	FALSE	OTOP3
Region_8115	chr17	74950742	74973166	22424	12	61	77.27272727	10.08966791	0.807225926	0.040267498	-	283987	grey	0.204798995	FALSE	HID1
Region_8116	chr17	74987632	75005800	18168	22	103	130.7575758	12.35881573	0.775292296	0.017490406	+	30850	grey	0.044261737	FALSE	CDR2L
Region_8117	chr17	75012670	75021261	8591	5	28	43.74242424	6.162693233	0.966701951	0.028256001	+	3396	grey	0.014861985	FALSE	MRPL58
Region_8118	chr17	75032575	75065889	33314	32	215	259.1818182	17.98282008	0.47646182	0.012875041	+	23510	grey	-0.187936735	FALSE	KCTD2
Region_8119	chr17	75038863	75046985	8122	10	68	87.74242424	11.64250912	0.222079827	0.020709374	-	10476	grey	-0.000830726	FALSE	ATP5PD
Region_8120	chr17	75087727	75106162	18435	12	47	68.21212121	9.206210532	0.873633521	0.029018917	+	9121	grey	0.066652252	FALSE	SLC16A5
Region_8121	chr17	75109952	75130272	20320	19	119	145.0757576	12.29422705	0.846392176	0.028098066	+	79637	grey	-0.00072024	FALSE	ARMC7
Region_8122	chr17	75130225	75131757	1532	4	22	30.71212121	4.719077979	0.303535469	0.04192219	-	30833	grey	-0.038698914	FALSE	NT5C
Region_8123	chr17	75135248	75168281	33033	15	97	116.8939394	8.884089128	0.761485119	0.018854208	-	51155	grey	0.071350323	FALSE	JPT1
Region_8124	chr17	75165586	75182959	17373	6	31	43.74242424	6.448161589	0.616730641	0.04257283	-	6613	grey	0.06176438	FALSE	SUMO2

Region_8125	chr17	75205659	75235758	30099	25	180	211.0909091	16.50895074	0.864412117	0.017541776	+	79902	grey	-0.001193417	FALSE	NUP85
Region_8126	chr17	75236599	75262363	25764	24	149	197.8333333	15.39888441	0.782819794	0.02614502	-	23163	grey	-0.257053781	FALSE	GGA3
Region_8127	chr17	75261674	75266376	4702	4	19	28.68181818	5.616347269	0.654939388	0.07170668	+	51081	grey	0.053245906	FALSE	MRPS7
Region_8128	chr17	75266228	75271231	5003	6	37	49.92424242	7.279709644	0.165593869	0.062990458	-	57409	grey	-0.026099259	FALSE	MIF4GD
Region_8129	chr17	75272981	75289510	16529	17	113	133.5606061	10.35690596	0.904155833	0.028945276	-	60386	grey	-0.114109861	FALSE	SLC25A19
Region_8130	chr17	75318076	75405709	87633	57	346	402.8333333	20.45000157	0.755357297	0.028342474	-	2885	grey	0.094048321	FALSE	GRB2
Region_8131	chr17	75441159	75500452	59293	55	349	397.4242424	24.49681964	0.772995964	0.015506672	+	9772	grey	0.055403132	FALSE	TMEM94
Region_8132	chr17	75500261	75515537	15276	10	53	70.40909091	8.443435817	0.941904938	0.032791845	-	57513	grey	-0.151031891	FALSE	CASKIN2
Region_8133	chr17	75515944	75524735	8791	7	24	39.21212121	7.863284011	0.570894296	0.052341212	+	283989	grey	-0.05644542	FALSE	TSEN54
Region_8134	chr17	75525080	75575209	50129	49	289	346.0606061	25.50889968	0.816995439	0.015847883	+	3993	grey	-0.343198142	FALSE	LLGL2
Region_8135	chr17	75587800	75626849	39049	31	153	188.5454545	14.10966043	0.747366834	0.024428491	+	80022	grey	0.176206291	FALSE	MYO15B
Region_8136	chr17	75626845	75667189	40344	32	229	259.2727273	14.9084854	0.887993142	0.015587141	-	9400	grey	0.008668361	FALSE	RECQL5
Region_8137	chr1	75633434	75641404	7970	10	56	75.3636363	8.31798119	0.86817042	0.02488088	+	643008	grey	0.040045923	FALSE	SMIM5

	7						6	7	7	1						
Region_8138	chr17	75667251	75708062	40811	23	164	185.7575758	12.32890363	0.788433284	0.02516113	+	29115	grey	-0.093482897	FALSE	SAP30BP
Region_8139	chr17	75721328	75757818	36490	26	154	188.4090909	14.65603291	0.838126084	0.019567785	+	3691	grey	0.085615482	FALSE	ITGB4
Region_8140	chr17	75751594	75765236	13642	14	70	93.62121212	11.07702425	0.709663507	0.031845133	-	2584	grey	0.065154023	FALSE	GALK1
Region_8141	chr17	75776434	75785486	9052	8	57	78.15151515	8.474835917	0.296367314	0.033857998	-	3021	grey	-0.139633223	FALSE	H3-3B
Region_8142	chr17	75784806	75825799	40993	25	152	179.0909091	14.71966479	0.942303771	0.014686152	+	85451	grey	-0.177182169	FALSE	UNK
Region_8143	chr17	75827225	75844785	17560	15	69	99.3030303	12.24615443	0.567627196	0.034907543	-	201294	grey	0.244250318	FALSE	UNC13D
Region_8144	chr17	75874164	75878581	4417	7	26	39.65151515	6.148151503	0.286234481	0.036716541	-	91107	grey	-0.059964889	FALSE	TRIM47
Region_8145	chr17	75880335	75896951	16616	19	94	121	12.27254597	0.848165212	0.028043753	-	201292	grey	-0.224920084	FALSE	TRIM65
Region_8146	chr17	75898644	75909817	11173	12	71	91.75757576	10.57873136	0.811247209	0.03534551	-	64978	grey	-0.250630258	FALSE	MRPL38
Region_8147	chr17	75898951	75941140	42189	26	150	191.3333333	17.70383125	0.851555523	0.018790131	-	85302	grey	-0.04836415	FALSE	FBF1
Region_8148	chr17	75941507	75979177	37670	31	204	239.3939394	16.46710361	0.875583669	0.010198084	-	51	grey	0.204723025	FALSE	ACOX1
Region_8149	chr17	75979231	76005999	26768	14	64	93.54545455	10.44425763	0.917445007	0.050207052	+	100529145	grey	0.076309413	FALSE	TEN1-CDK3

Region_8150	chr17	75979240	76000586	21346	13	62	88.65151515	10.32620628	0.936408998	0.035767027	+	100134934	grey	0.036007151	FALSE	TEN1
Region_8151	chr17	76006845	76027306	20461	12	66	90.42424242	11.21820033	0.789512753	0.025080172	-	2125	grey	-0.221176852	FALSE	EVPL
Region_8152	chr17	76038775	76072517	33742	13	89	108.8181818	11.10912181	0.732354094	0.037576229	-	6730	grey	-0.019334107	FALSE	SRP68
Region_8153	chr17	76071961	76079778	7817	9	46	66.6969697	8.64686639	0.036788995	0.026695654	+	8811	grey	0.050198	FALSE	GALR2
Region_8154	chr17	76081016	76121576	40560	39	260	294.8030303	19.22195835	0.872326683	0.016338388	-	23265	grey	-0.077883556	FALSE	EXOC7
Region_8155	chr17	76136333	76141245	4912	9	35	54.31818182	7.553214478	0.549803554	0.039326157	-	2302	grey	0.0455361	FALSE	FOXJ1
Region_8156	chr17	76138432	76154643	16211	22	113	137.3484848	13.01063757	0.514408613	0.036918624	+	100507218	grey	-0.01681013	FALSE	RNF157-AS1
Region_8157	chr17	76142465	76240493	98028	78	546	615.2575758	34.61005217	0.870441113	0.013557858	-	114804	grey	0.039367788	FALSE	RNF157
Region_8158	chr17	76265348	76271298	5950	15	62	86.93939394	13.1041729	0.116947631	0.025839558	+	283991	grey	0.197052219	FALSE	UBALD2
Region_8159	chr17	76274049	76308276	34227	35	227	274.2272727	20.13935715	0.878271221	0.01866746	-	84074	grey	-0.243449912	FALSE	QRICH2
Region_8160	chr17	76309478	76384521	75043	88	598	666.3030303	29.46077951	0.819902758	0.014600568	-	5635	grey	0.166250522	FALSE	PRPSAP1
Region_8161	chr17	76376584	76387860	11276	21	119	152.1666667	18.07725586	0.432235109	0.023650726	+	8877	grey	-0.193584509	FALSE	SPHK1
Region_8162	chr1	76389456	76453152	63696	56	420	458.121212	21.7987403	0.86791751	0.01457670	-	63893	grey	-0.11068071	FALSE	UBE2O

	7						1	7	6	2				2		
Region_8163	chr17	76453351	76470117	16766	18	104	133.9090909	12.21314207	0.83871342	0.034318973	+	15	grey	0.034918373	FALSE	AANAT
Region_8164	chr17	76470891	76501790	30899	50	300	342.1060606	18.23869832	0.652767886	0.022701455	-	79651	grey	0.004441222	FALSE	RHBDF2
Region_8165	chr17	76527356	76557683	30327	33	206	243.530303	17.55976143	0.611441937	0.028949666	-	114757	grey	0.237144069	FALSE	CYGB
Region_8166	chr17	76527586	76553578	25992	27	154	190.8787879	15.01949006	0.638083441	0.03365246	+	768206	grey	0.22999206	FALSE	PRCD
Region_8167	chr17	76557727	76670985	113258	64	383	463.5151515	25.69240679	0.67902812	0.018892463	+	100507246	grey	-0.249381681	FALSE	SNHG16
Region_8168	chr17	76557769	76560433	2664	3	17	29.42424242	5.156207927	0.586119574	0.06891996	+	677850	grey	-0.081562032	FALSE	SNORD1C
Region_8169	chr17	76563710	76586956	23246	18	75	110.6212121	13.57119705	0.457865507	0.034370742	-	10610	grey	-0.274363569	FALSE	ST6GALNAC2
Region_8170	chr17	76617959	76643786	25827	14	81	105.2424242	11.38157838	0.778852646	0.061264485	-	55808	grey	-0.036177731	FALSE	ST6GALNAC1
Region_8171	chr17	76672551	76711004	38453	20	101	128.5606061	12.23630008	0.891025783	0.032850646	-	439921	grey	-0.066521152	FALSE	MXRA7
Region_8172	chr17	76712832	76726799	13967	10	62	80.09090909	7.851951786	0.503564603	0.032191169	-	23210	grey	0.634050817	FALSE	JMJD6
Region_8173	chr17	76726830	76738522	11692	7	30	39.40909091	6.063706084	0.414419123	0.037223194	+	124512	grey	-0.287701318	FALSE	METTL23
Region_8174	chr17	76735865	76781449	45584	18	105	126.5	9.369754943	0.958481155	0.020934979	+	79157	grey	-0.023418784	FALSE	MFSD11

Region_8175	chr17	76799044	76807102	8058	4	21	33.04545455	5.13938583	0.946081717	0.039557675	+	101928514	grey	0.159845438	FALSE	LINC02080
Region_8176	chr17	76849840	76861836	11996	9	57	76.60606061	8.299914342	0.92426523	0.041740043	+	283994	grey	0.435907441	FALSE	LINC00868
Region_8177	chr17	76868404	76950393	81989	85	535	613.5757576	34.73019551	0.764606398	0.017107466	+	146664	grey	-0.106227702	FALSE	MGAT5B
Region_8178	chr17	76950474	76969156	18682	17	100	125.3030303	12.08300739	0.873626154	0.026464939	-	105371899	grey	-0.121307321	FALSE	LOC105371899
Region_8179	chr17	77086716	77099902	13186	19	134	160.8484848	11.09849945	0.68941587	0.02887733	+	654434	grey	-0.054596539	FALSE	SNHG20
Region_8180	chr17	77088749	77217101	128352	120	846	932.8030303	41.02015253	0.833170403	0.012775287	+	6397	grey	-0.094861546	FALSE	SEC14L1
Region_8181	chr17	77516887	77536601	19714	14	82	97.8030303	10.22247854	0.858024121	0.024206988	+	400622	grey	-0.030247902	FALSE	LOC400622
Region_8182	chr17	77590532	77680687	90155	59	421	464.0757576	22.5840724	0.811955139	0.017269057	+	107985079	grey	0.129521436	FALSE	LOC107985079
Region_8183	chr17	77877330	77884119	6789	3	21	29.5	5.171668381	0.844318953	0.063052686	-	400624	grey	-0.000379106	FALSE	LINC01973
Region_8184	chr17	77958703	78108822	150119	101	788	863.2272727	38.03876381	0.877313324	0.009536499	+	57690	grey	-0.158967171	FALSE	TNRC6C
Region_8185	chr17	78107397	78132407	25010	27	148	184.8484848	18.89097662	0.5391997	0.027429616	-	11322	grey	0.022447739	FALSE	TMC6
Region_8186	chr17	78130770	78142968	12198	22	125	157.6212121	15.77219086	0.353496019	0.031562679	+	147138	grey	0.282350772	FALSE	TMC8
Region_8187	chr1	78146385	78166297	19912	27	154	191.696969	14.9707095	0.78262894	0.03375863	+	10014151	grey	-0.13517525	FALSE	C17orf99

	7						7	5	6	4		5				
Region_8188	chr17	78168581	78173527	4946	7	27	44.06060606	7.232147862	0.35299019	0.03920515	+	9144	grey	0.005873645	FALSE	SYNGR2
Region_8189	chr17	78174091	78187233	13142	16	117	138.6515152	10.925612	0.673101151	0.026792061	-	7083	grey	0.025723531	FALSE	TK1
Region_8190	chr17	78187358	78207702	20344	24	153	179.1969697	13.44186308	0.839929193	0.024945806	+	125061	grey	-0.141614899	FALSE	AFMID
Region_8191	chr17	78214186	78225636	11450	16	88	124.5909091	12.85059385	0.910058284	0.018067267	+	332	grey	-0.091289151	FALSE	BIRC5
Region_8192	chr17	78231310	78240987	9677	22	100	133.3030303	12.61375281	0.84149389	0.034403921	+	283999	grey	0.211027741	FALSE	TMEM235
Region_8193	chr17	78261349	78278492	17143	18	103	129.2575758	13.13399653	0.71536228	0.034492459	-	100996291	grey	0.022783378	FALSE	LINC01993
Region_8194	chr17	78315729	78347798	32069	34	197	230.2121212	16.37494818	0.801214672	0.023892317	+	105371912	grey	0.149232466	FALSE	LOC105371912
Region_8195	chr17	78356778	78360077	3299	6	16	33.36363636	5.336770454	0.433783024	0.071273095	-	9021	grey	0.045663461	FALSE	SOCS3
Region_8196	chr17	78360376	78373911	13535	14	80	107.7272727	10.56051493	0.644447676	0.038109721	+	101928674	grey	0.097504094	FALSE	SOCS3-DT
Region_8197	chr17	78378649	78425114	46465	56	348	403.0454545	24.28322106	0.760760103	0.0176724	+	9489	grey	0.103835848	FALSE	PGS1
Region_8198	chr17	78423697	78577396	153699	150	1016	1114.060606	51.03690333	0.887129296	0.011373817	-	8632	grey	0.216882236	FALSE	DNAH17
Region_8199	chr17	78484882	78503056	18174	19	102	134.5151515	13.35001288	0.817342235	0.027969934	+	100996295	grey	0.209307661	FALSE	DNAH17-AS1

Region_8200	chr17	78605233	78632155	26922	23	155	187.4545455	15.28416155	0.941836526	0.019627196	-	101928710	grey	-0.305882621	FALSE	SCAT1
Region_8201	chr17	78674048	78782297	108249	56	391	440.030303	22.84460529	0.788955199	0.019744562	-	9267	grey	0.439071274	FALSE	CYTH1
Region_8202	chr17	78787381	78841441	54060	70	470	553.7121212	29.28836217	0.875835179	0.009965966	-	57602	grey	0.112935715	FALSE	USP36
Region_8203	chr17	78852977	78925387	72410	101	673	756.6969697	40.21326597	0.820306994	0.014422068	-	7077	grey	0.186430152	FALSE	TIMP2
Region_8204	chr17	78870910	78903217	32307	49	303	363	29.11409915	0.820856584	0.022544376	-	100653515	grey	-0.050251403	FALSE	CEP295NL
Region_8205	chr17	78971238	78979947	8709	17	94	121.6969697	11.89695462	0.862086056	0.031342508	-	3959	grey	-0.001290034	FALSE	LGALS3BP
Region_8206	chr17	78991716	79009867	18151	34	220	254.5454545	15.41645656	0.764694389	0.018204176	-	124583	grey	-0.027278145	FALSE	CANT1
Region_8207	chr17	79018131	79027673	9542	21	122	158.5	16.41036258	0.463400814	0.023501833	-	100507410	grey	-0.127384657	FALSE	C1QTNF1-AS1
Region_8208	chr17	79022814	79049788	26974	57	378	445.6060606	28.83527689	0.711207988	0.014861687	+	114897	grey	-0.091350376	FALSE	C1QTNF1
Region_8209	chr17	79074824	79088599	13775	33	180	216.0909091	17.51898072	0.776442531	0.023398744	+	64772	grey	0.082452722	FALSE	ENGASE
Region_8210	chr17	79089345	79611051	521706	763	5369	5769.166667	188.9430181	0.746610029	0.012714889	-	146713	grey	0.187235094	FALSE	RBFOX3
Region_8211	chr17	79707266	79712317	5051	4	11	16.09090909	3.436130528	0.748630105	0.107920415	+	100652929	grey	-0.054030716	FALSE	LINC02078
Region_8212	chr1	79778148	79787983	9835	13	58	78.4696969	9.29221294	0.35521116	0.04037202	+	84733	grey	0.135971435	FALSE	CBX2

	7						7	7	6	9						
Region_8213	chr17	79792132	79801683	9551	25	149	175.3333333	14.82963077	0.1174019	0.028272449	-	57332	grey	0.031559967	FALSE	CBX8
Region_8214	chr17	79819083	79827020	7937	5	28	45.24242424	6.177030917	0.679393042	0.055540868	+	102723961	grey	0.248584128	FALSE	
Region_8215	chr17	79822634	79827704	5070	9	46	85.92424242	22.18752984	0.798457303	0.037587172	+	105371919	grey	0.243295527	FALSE	LINC01977
Region_8216	chr17	79833156	79839440	6284	8	25	41.60606061	7.255509923	0.326589869	0.041053791	-	8535	grey	0.327063131	FALSE	CBX4
Region_8217	chr17	79915252	79926725	11473	16	79	120.5	14.80774226	0.800735065	0.043497806	-	101928766	grey	-0.10667719	FALSE	LINC01979
Region_8218	chr17	79919310	79926018	6708	8	47	60.06060606	8.108644557	0.71432035	0.051183631	+	101928738	grey	-0.146455452	FALSE	LINC01978
Region_8219	chr17	79932343	80035872	103529	228	1551	1734.757576	75.3286258	0.843984484	0.01013047	-	125058	grey	0.210612163	FALSE	TBC1D16
Region_8220	chr17	80036632	80100613	63981	96	641	734.1969697	36.96660435	0.83404391	0.017621005	+	55036	grey	-0.07673095	FALSE	CCDC40
Region_8221	chr17	80101556	80119881	18325	21	110	141.7272727	14.49089417	0.849986654	0.023475193	+	2548	grey	0.013517929	FALSE	GAA
Region_8222	chr17	80134369	80147151	12782	12	66	87.66666667	8.711324784	0.56190459	0.017777323	-	9775	grey	0.032000552	FALSE	EIF4A3
Region_8223	chr17	80169992	80209331	39339	80	569	625.1818182	25.41405371	0.896348432	0.016880458	+	79092	grey	0.020138992	FALSE	CARD14
Region_8224	chr17	80201589	80220923	19334	41	256	311.0454545	25.24491225	0.889094076	0.017043531	-	6448	grey	0.021604097	FALSE	SGSH

Region_8225	chr17	80219699	80253500	33801	31	186	222.969697	16.86048607	0.880134289	0.022676526	+	284129	grey	0.111148142	FALSE	SLC26A11
Region_8226	chr17	80260852	80398794	137942	174	1262	1378.409091	54.14822315	0.912883541	0.007606965	+	57674	grey	-0.13139861	FALSE	RNF213
Region_8227	chr17	80351828	80415168	63340	52	367	418.469697	21.04516732	0.875310271	0.013725355	-	100294362	grey	0.024427511	FALSE	RNF213-AS1
Region_8228	chr17	80415165	80438086	22921	29	186	226.969697	16.77082604	0.819686188	0.016146843	+	284131	grey	-0.091214278	FALSE	ENDOV
Region_8229	chr17	80466834	80477843	11009	43	185	282.6363636	30.06947666	0.139425436	0.013580751	-	4884	grey	-0.018134905	FALSE	NPTX1
Region_8230	chr17	80544819	80966371	421552	585	4260	4671.075758	164.1889447	0.849641438	0.006987375	+	57521	grey	0.222428858	FALSE	RPTOR
Region_8231	chr17	80801640	80805632	3992	8	44	64.18181818	8.01711456	0.905457046	0.03060146	-	101928855	grey	0.234167654	FALSE	LOC101928855
Region_8232	chr17	80966239	80971213	4974	10	45	63.06060606	9.535163728	0.791808596	0.067135603	-	400627	grey	0.222584434	FALSE	LOC400627
Region_8233	chr17	80991598	81009517	17919	34	207	246.7575758	17.14379063	0.653270775	0.027917622	+	79643	grey	-0.285951241	FALSE	CHMP6
Region_8234	chr17	81003335	81005132	1797	3	13	22.96969697	4.332239417	0.010111087	0.022068184	+	100129503	grey	-0.522887914	FALSE	
Region_8235	chr17	81029130	81034881	5751	14	90	110.6818182	10.51908023	0.545243271	0.058768964	-	440465	grey	0.020152501	FALSE	BAIAP2-DT
Region_8236	chr17	81035122	81117434	82312	176	1132	1289.378788	66.73801019	0.862072144	0.010469793	+	10458	grey	-0.192974902	FALSE	BAIAP2
Region_8237	chr1	81110487	81166221	55734	89	570	643.242424	38.7794793	0.82732804	0.01816573	-	9625	grey	-0.03656708	FALSE	AATK

	7						2	5	3	4						
Region_8238	chr1<br>7	81165507	81183166	17659	10	48	69	8.92877802	0.65724176<br>1	0.03172996	+	388428	grey	-0.15432469<br>6	FALSE	PVALEF
Region_8239	chr1<br>8	47221	73545	26324	14	74	98.3484848<br>5	11.5352202<br>1	0.80458233<br>3	0.05813990<br>2	-	260334	grey	-0.09551104<br>2	FALSE	TUBB8B
Region_8240	chr1<br>8	109065	122340	13275	9	15	39.6969697	12.2461544<br>3	0.19029317<br>9	0.06683989<br>8	+	727758	grey	0.288552022	FALSE	ROCK1P1
Region_8241	chr1<br>8	158383	214629	56246	20	85	123.863636<br>4	14.0130558<br>5	0.64300768	0.01183612<br>2	+	9097	grey	-0.05970564<br>8	FALSE	USP14
Region_8242	chr1<br>8	214520	268050	53530	8	44	70.4848484<br>8	10.9196258<br>4	0.61718741<br>1	0.01663976<br>9	-	9984	grey	-0.13830515<br>5	FALSE	THOC1
Region_8243	chr1<br>8	316737	500722	183985	48	287	343.454545<br>5	28.3430533<br>2	0.39296372<br>3	0.01507848<br>1	-	81035	grey	0.138724237	FALSE	COLEC12
Region_8244	chr1<br>8	508568	515294	6726	10	33	58.8787878<br>8	13.6838086<br>8	0.98061454<br>4	0.02187339<br>6	-	10537685<br>4	grey	0.296083231	FALSE	LINC01925
Region_8245	chr1<br>8	596988	650334	53346	13	65	87.1363636<br>4	10.8157810<br>1	0.90716563<br>9	0.01802917<br>3	+	27098	grey	-0.31040496<br>1	FALSE	CLUL1
Region_8246	chr1<br>8	623743	642518	18775	7	25	45.3787878<br>8	7.62741039<br>1	0.97900578<br>9	0.02224879<br>9	-	10537195<br>2	grey	-0.17505400<br>8	FALSE	LOC105371952
Region_8247	chr1<br>8	641320	658350	17030	20	80	123.257575<br>8	21.8866384<br>3	0.12760798<br>9	0.02148129<br>1	-	494514	grey	-0.03810545<br>6	FALSE	TYMSOS
Region_8248	chr1<br>8	657653	673578	15925	15	83	113.469697	16.1352168<br>1	0.20020870<br>2	0.00726564<br>4	+	7298	grey	-0.03824087<br>1	FALSE	TYMS
Region_8249	chr1<br>8	670318	712662	42344	28	213	276.075757<br>6	31.8884163<br>2	0.29236878<br>3	0.01296836<br>7	-	55556	grey	-0.00596248<br>2	FALSE	ENOSF1

Region_8250	chr18	721588	812546	90958	495	3435	3836.348485	142.9063698	0.835521037	0.011281376	-	7525	grey	0.105116942	FALSE	YES1
Region_8251	chr18	904871	912172	7301	300	1876	2287.530303	189.2151579	0.065192054	0.022935138	+	116	grey	-0.250248643	FALSE	ADCYAP1
Region_8252	chr18	926083	927993	1910	12	68	102.5606061	14.90805542	0.888178173	0.04107986	-	388456	grey	0.283142161	FALSE	LINC01904
Region_8253	chr18	976589	1310018	333429	1917	16242	16989.31818	330.5738674	0.846357598	0.014086205	+	105371953	grey	0.255991445	FALSE	LOC105371953
Region_8254	chr18	1254383	1407244	152861	874	6808	7241.227273	225.352428	0.86081534	0.017439621	-	56651	grey	0.078203237	FALSE	LINC00470
Region_8255	chr18	1269528	1408344	138816	801	6171	6601.439394	218.4095045	0.86305081	0.017722812	-	105371954	grey	0.089451449	FALSE	LOC105371954
Region_8256	chr18	1883704	2240847	357143	1672	11269	13484.60606	495.5118528	0.864751165	0.015402373	-	105371958	grey	0.160480818	FALSE	LOC105371958
Region_8257	chr18	2034144	2489426	455282	824	6059	6539.106061	192.3220959	0.863109697	0.013189874	-	105371956	grey	-0.227432961	FALSE	LOC105371956
Region_8258	chr18	2537525	2571509	33984	10	51	73.25757576	10.37053608	0.295033719	0.024246762	-	64863	grey	0.011484292	FALSE	METTL4
Region_8259	chr18	2652170	2655395	3225	11	43	82.5	15.78436422	0.003486906	0.00706637	-	645158	grey	-0.089101307	FALSE	CBX3P2
Region_8260	chr18	2655726	2805017	149291	62	328	400.7878788	34.97934122	0.466367209	0.010959134	+	23347	grey	-0.004489618	FALSE	SMCHD1
Region_8261	chr18	2847006	2916003	68997	98	540	645.969697	48.12546733	0.405274123	0.013315313	+	84034	grey	0.026010567	FALSE	EMILIN2
Region_8262	chr1	2885296	3013144	127848	108	636	730.787878	41.8004472	0.51259449	0.01160685	-	9663	grey	-0.15394179	FALSE	LPIN2

	8						8	4	3					2		
Region_8263	chr18	2967018	2985410	18392	5	21	36.72727273	5.353777831	0.775399617	0.049767072	+	105371963	grey	0.10138348	FALSE	LOC105371963
Region_8264	chr18	3066807	3219968	153161	59	412	479.7424242	27.58556512	0.900865073	0.011461013	-	8736	grey	0.04570318	FALSE	MYOM1
Region_8265	chr18	3190397	3247277	56880	30	204	246.0151515	17.94135097	0.845280093	0.018069538	+	101927044	grey	0.088320814	FALSE	LOC101927044
Region_8266	chr18	3247481	3256236	8755	9	48	73.33333333	11.38735241	0.11170986	0.010820972	+	10627	grey	0.115190069	FALSE	MYL12A
Region_8267	chr18	3255436	3261850	6414	9	47	65.62121212	8.368007118	0.312813881	0.021552693	-	104968399	grey	-0.178773709	FALSE	MYL12-AS1
Region_8268	chr18	3261479	3278461	16982	25	126	171.0151515	20.47173844	0.06237158	0.01059252	+	103910	grey	0.021658715	FALSE	MYL12B
Region_8269	chr18	3347696	3383532	35836	23	143	171.5	13.46705382	0.930726944	0.023285551	-	105371964	grey	-0.156949669	FALSE	LINC01895
Region_8270	chr18	3411608	3459978	48370	81	500	584.0606061	36.91273651	0.297895027	0.009459731	+	7050	grey	0.319217653	FALSE	TGIF1
Region_8271	chr18	3466250	3487045	20795	7	36	54.13636364	9.601099879	0.946803125	0.065019465	+	100505592	grey	0.102437378	FALSE	GAPLINC
Region_8272	chr18	3496032	4455307	959275	207	1351	1493.439394	70.554099	0.741496495	0.00940407	-	9229	grey	-0.219798439	FALSE	DLGAP1
Region_8273	chr18	3603738	3610103	6365	3	14	22.72727273	4.58608415	0.482556184	0.098151149	+	84777	grey	0.010845366	FALSE	DLGAP1-AS2
Region_8274	chr18	3878180	3897069	18889	8	35	58.21212121	9.696177124	0.860353144	0.026054497	+	201477	grey	-0.214793984	FALSE	DLGAP1-AS3

Region_8275	chr18	3962353	4024150	61797	12	53	89.1969697	16.63658591	0.915060485	0.045340681	+	101410534	grey	-0.236028541	FALSE	DLGAP1-AS4
Region_8276	chr18	4264602	4295405	30803	4	14	23.83333333	5.774168827	0.76310863	0.086427258	+	284215	grey	-0.21229709	FALSE	DLGAP1-AS5
Region_8277	chr18	5142911	5197503	54592	28	172	220.2575758	20.80703328	0.278448927	0.033740094	-	642597	grey	0.140239496	FALSE	AKAIN1
Region_8278	chr18	5236724	5238598	1874	23	148	211.3636364	25.64769377	0.004419452	0.006420735	-	147525	grey	0.109703652	FALSE	LINC00526
Region_8279	chr18	5237844	5290608	52764	26	179	229.8333333	23.71145996	0.099123933	0.015560019	+	339290	grey	0.54767686	FALSE	LINC00667
Region_8280	chr18	5289019	5297053	8034	53	244	313.0757576	31.06584105	0.021521081	0.004385045	-	7541	grey	0.03938666	FALSE	ZBTB14
Region_8281	chr18	5392381	5630700	238319	151	850	1023.833333	68.55061764	0.382755238	0.010172658	-	23136	grey	-0.121352727	FALSE	EPB41L3
Region_8282	chr18	5721812	5909122	187310	99	500	602.6212121	48.72363671	0.197312395	0.016173358	+	645355	grey	-0.221767026	FALSE	MIR3976HG
Region_8283	chr18	5882072	5895955	13883	78	344	447.8333333	46.62900495	0.068047516	0.016368214	-	645369	grey	-0.084242599	FALSE	TMEM200C
Region_8284	chr18	5895745	6108382	212637	40	280	324.8787879	19.73635081	0.682123044	0.019559098	+	121725015	grey	0.090462017	FALSE	LOC121725015
Region_8285	chr18	5954706	6415237	460531	122	876	980.3333333	41.67911609	0.676470021	0.009756919	-	91133	grey	0.247028471	FALSE	L3MBTL4
Region_8286	chr18	6511416	6590653	79237	8	50	72.03030303	8.195626783	0.911408467	0.045297484	+	100130480	grey	-0.2157245	FALSE	LINC01387
Region_8287	chr1	6728821	6729862	1041	12	42	69.6060606	17.7286198	0.01046821	0.01983361	-	10192716	grey	0.128862769	FALSE	ARHGAP28-AS1

	8						1	8	9	4		8				
Region_8288	chr18	6729716	6915716	186000	63	370	463.1969697	31.53177625	0.634665191	0.011424956	+	79822	grey	0.127540006	FALSE	ARHGAP28
Region_8289	chr18	6919496	6929966	10470	8	33	56.48484848	8.973880773	0.784986478	0.057608117	-	400643	grey	-0.071902706	FALSE	LINC00668
Region_8290	chr18	6941742	7117797	176055	114	754	844.8787879	42.75890016	0.710525539	0.011684506	-	284217	grey	-0.112648617	FALSE	LAMA1
Region_8291	chr18	6954677	6957419	2742	5	29	44.24242424	6.231583589	0.943281208	0.034109078	+	101927188	grey	0.157595895	FALSE	LOC101927188
Region_8292	chr18	7231089	7232044	955	3	19	31.72727273	6.374336658	0.92673084	0.055384835	+	339291	grey	0.085641567	FALSE	LRRC30
Region_8293	chr18	7455844	7461135	5291	5	26	35.6969697	6.260915875	0.945156465	0.046831515	-	112577592	grey	-0.109814471	FALSE	LOC112577592
Region_8294	chr18	7566782	8406861	840079	223	1429	1625.106061	75.95527607	0.607856591	0.007338411	+	5797	grey	0.216631943	FALSE	PTPRM
Region_8295	chr18	8609437	8639383	29946	27	138	184.6818182	19.19708238	0.295787782	0.016041616	+	201475	grey	-0.049617401	FALSE	RAB12
Region_8296	chr18	8695856	8707699	11843	62	249	315.0151515	33.91640965	0.091666844	0.013149438	-	100287082	grey	-0.019012673	FALSE	GACAT2
Region_8297	chr18	8705556	8832778	127222	110	653	738.2727273	42.25874346	0.587559416	0.010747878	+	23255	grey	-0.242582513	FALSE	MTCL1
Region_8298	chr18	9102630	9182524	79894	74	413	530.1818182	42.23119849	0.269466702	0.010089436	+	4729	grey	0.032319036	FALSE	NDUFV2
Region_8299	chr18	9102736	9285985	183249	91	554	662.2727273	46.68274115	0.400701681	0.009503772	+	23253	grey	0.124392069	FALSE	ANKRD12

Region_8300	chr18	9121179	9136645	15466	15	90	122.8484848	14.88958585	0.274371897	0.021091583	-	101927275	grey	-0.014597574	FALSE	NDUFV2-AS1
Region_8301	chr18	9310522	9334445	23923	12	70	93.24242424	10.52478482	0.733864432	0.02151343	-	102724397	grey	-0.006307998	FALSE	TWSG1-DT
Region_8302	chr18	9334767	9402420	67653	35	196	233.4090909	19.24815857	0.556508059	0.018579275	+	57045	grey	0.126545008	FALSE	TWSG1
Region_8303	chr18	9475009	9538114	63105	49	268	333.6969697	27.71834699	0.594273746	0.014513801	+	10928	grey	0.359969106	FALSE	RALBP1
Region_8304	chr18	9546791	9615240	68449	47	240	307.3030303	27.10831249	0.508037282	0.016477808	-	9989	grey	0.036709461	FALSE	PPP4R1
Region_8305	chr18	9708275	9862551	154276	71	485	555.7121212	33.36684912	0.656185937	0.011493728	+	11031	grey	0.170077246	FALSE	RAB31
Region_8306	chr18	9914016	9960021	46005	29	141	181.7878788	18.79731172	0.337354807	0.016873212	+	9218	grey	-0.046469292	FALSE	VAPA
Region_8307	chr18	10085838	10144406	58568	22	114	160.6666667	18.06853051	0.69442543	0.019361403	+	105371984	grey	-0.141238141	FALSE	LOC105371984
Region_8308	chr18	10321380	10372387	51007	13	92	113.5606061	11.62375391	0.949007876	0.035661345	-	105371985	grey	0.202045543	FALSE	LOC105371985
Region_8309	chr18	10323132	10372386	49254	13	92	113.5606061	11.62375391	0.949007876	0.035661345	-	105371986	grey	0.202045543	FALSE	LOC105371986
Region_8310	chr18	10405133	10414370	9237	4	20	34.98484848	5.606294331	0.824951436	0.09969141	-	101927350	grey	0.194504459	FALSE	LINC01254
Region_8311	chr18	10406558	10422360	15802	6	39	54.93939394	7.204440485	0.878582741	0.067398728	+	105371988	grey	0.204479642	FALSE	LOC105371988
Region_8312	chr1	10454635	10489949	35314	43	246	317.5	27.4832816	0.40533608	0.01303646	+	147495	grey	-0.02197487	FALSE	APCDD1

	8							3	3	3						
Region_8313	chr18	10525905	10552764	26859	16	111	141.969697	16.1197153	0.235582423	0.044298336	+	8774	grey	-0.12186755	FALSE	NAPG
Region_8314	chr18	10594590	10604798	10208	4	13	28.78787879	6.026901619	0.96776703	0.032887876	+	105371990	grey	0.038230985	FALSE	
Region_8315	chr18	10666483	11149569	483086	199	1404	1537.075758	65.57445812	0.706148602	0.011099612	-	63895	grey	-0.11305491	FALSE	PIEZO2
Region_8316	chr18	11447756	11489369	41613	5	21	33.63636364	5.973367466	0.968123552	0.032447382	-	107985173	grey	0.2165524	FALSE	LOC107985173
Region_8317	chr18	11486205	11523087	36882	8	53	67.63636364	7.43098785	0.8399987	0.048939116	+	101927433	grey	-0.029820032	FALSE	LINC01255
Region_8318	chr18	11689264	11885685	196421	156	1056	1175.636364	56.09240628	0.531369137	0.006823942	+	2774	grey	0.021800409	FALSE	GNAL
Region_8319	chr18	11851413	11854444	3031	12	93	122.5909091	12.550179	0.084629738	0.006484919	+	57132	grey	0.424165354	FALSE	CHMP1B
Region_8320	chr18	11882622	11908366	25744	12	66	98.28787879	15.28575521	0.637692002	0.015415307	-	65258	grey	0.187876469	FALSE	MPPE1
Region_8321	chr18	11910634	11914344	3710	3	14	23.81818182	4.663801985	0.982292932	0.025223857	-	101927511	grey	0.163020659	FALSE	LOC101927511
Region_8322	chr18	11981025	12030877	49852	50	355	421.969697	25.67966552	0.623964801	0.01209645	+	3613	grey	0.1896848	FALSE	IMPA2
Region_8323	chr18	12093843	12129764	35921	7	49	61.98484848	7.45549343	0.953596431	0.032260289	+	342850	grey	0.225213409	FALSE	ANKRD62
Region_8324	chr18	12200779	12223539	22760	12	42	70.34848485	12.29069995	0.957477271	0.030151664	+	497259	grey	0.065589887	FALSE	C18orf61

Region_8325	chr18	12254361	12277595	23234	35	152	214.030303	23.40083476	0.474379185	0.023969589	+	1149	grey	-0.051372031	FALSE	CIDEA
Region_8326	chr18	12307669	12337225	29556	57	349	441.4545455	38.01585977	0.433316874	0.007232483	+	84617	grey	-0.042167145	FALSE	TUBB6
Region_8327	chr18	12310967	12344320	33353	25	156	205.4848485	19.74704841	0.97618605	0.009502497	+	107985154	grey	-0.019937268	FALSE	LOC107985154
Region_8328	chr18	12328944	12377227	48283	38	221	270.7272727	24.63989601	0.588229634	0.012401094	-	10939	grey	0.016843544	FALSE	AFG3L2
Region_8329	chr18	12407896	12432238	24342	46	252	302.1515152	26.25569091	0.389502838	0.014451379	+	10650	grey	0.001079898	FALSE	PRELID3A
Region_8330	chr18	12432897	12447798	14901	21	101	147.0757576	18.31124968	0.953519299	0.016250234	+	105371998	grey	-0.156590794	FALSE	LOC105371998
Region_8331	chr18	12446512	12658107	211595	105	513	620.8484848	55.82561927	0.487819065	0.012099465	-	56907	grey	-0.026895461	FALSE	SPIRE1
Region_8332	chr18	12658043	12728945	70902	71	452	526.7121212	32.2427166	0.529625523	0.01201534	+	56984	grey	0.002395335	FALSE	PSMG2
Region_8333	chr18	12661833	12702777	40944	36	230	288.3030303	22.49031908	0.582093761	0.011934471	-	79959	grey	0.449967523	FALSE	CEP76
Region_8334	chr18	12739490	12776140	36650	21	134	166.1212121	15.34076034	0.958442737	0.016324845	-	100996324	grey	-0.121422271	FALSE	LINC01882
Region_8335	chr18	12785478	12929643	144165	131	773	917.969697	53.18124878	0.499595735	0.019191784	-	5771	grey	0.098422108	FALSE	PTPN2
Region_8336	chr18	12947133	12987536	40403	45	237	317.2575758	33.01948637	0.247953799	0.009046742	+	81929	grey	0.076123884	FALSE	SEH1L
Region_8337	chr1	12991362	13125052	133690	34	227	262.893939	19.5346861	0.80330238	0.01232496	+	55125	grey	-0.12445789	FALSE	CEP192

	8						4	4	6	1						
Region_8338	chr18	13217498	13652755	435257	330	2338	2602.227273	122.5673812	0.749842945	0.007720124	+	753	grey	0.142351036	FALSE	LDLRAD4
Region_8339	chr18	13239543	13242076	2533	6	26	44.5	8.378084966	0.980397443	0.022098733	+	147276	grey	-0.14100275	FALSE	C18orf15
Region_8340	chr18	13419421	13427480	8059	5	28	41.33333333	5.638694157	0.774488766	0.036127296	-	100288122	grey	-0.018143249	FALSE	LDLRAD4-AS1
Region_8341	chr18	13663347	13726663	63316	40	253	332.3636364	34.02968675	0.2986476	0.009279092	-	125228	grey	0.337378215	FALSE	FAM210A
Region_8342	chr18	13726660	13764556	37896	19	118	157.0909091	17.54618282	0.413700625	0.012770624	+	8731	grey	-0.071711736	FALSE	RNMT
Region_8343	chr18	13824149	13827323	3174	16	88	144.1060606	19.74384328	0.542527051	0.015159106	+	4161	grey	0.189353287	FALSE	MC5R
Region_8344	chr18	13882044	13915707	33663	8	54	72.72727273	9.412831593	0.901627846	0.034126827	-	4158	grey	-0.113041289	FALSE	MC2R
Region_8345	chr18	14057457	14132490	75033	12	62	83.60606061	12.13498285	0.461860392	0.034260908	-	162655	grey	-0.202111165	FALSE	ZNF519
Region_8346	chr18	14179097	14244811	65714	12	54	82.25757576	12.01321145	0.484396221	0.062944622	+	440482	grey	-0.064332732	FALSE	ANKRD20A5P
Region_8347	chr18	14225224	14342524	117300	11	74	102.1969697	12.48410645	0.933074163	0.030374918	+	284233	grey	-0.006172312	FALSE	CYP4F35P
Region_8348	chr18	14404551	14430923	26372	4	22	36.92424242	5.575525104	0.654919106	0.052504552	-	105372003	grey	-0.037450289	FALSE	LOC105372003
Region_8349	chr18	14477955	14499278	21323	27	135	171.0151515	15.80797503	0.907529831	0.028216391	-	440224	grey	-0.301284981	FALSE	CXADRP3

Region_8350	chr18	14507339	14543600	36261	5	10	19.57575758	4.513435008	0.898447462	0.084484329	-	388468	grey	0.037930799	FALSE	POTEC
Region_8351	chr18	14728272	14854667	126395	26	153	188.8939394	17.87747081	0.594508008	0.039118241	+	374860	grey	-0.156011824	FALSE	ANKRD30B
Region_8352	chr18	14877611	14884052	6441	3	14	23.42424242	5.920136709	0.940405148	0.057532386	+	105372009	grey	-0.081142146	FALSE	LINC01906
Region_8353	chr18	14946267	14973739	27472	4	25	42.43939394	6.814303481	0.925169108	0.050180248	+	400644	grey	-0.19013278	FALSE	LINC01443
Region_8354	chr18	20946906	21111813	164907	32	222	265.8030303	20.12745518	0.929655511	0.01598132	-	6093	grey	-0.178454969	FALSE	ROCK1
Region_8355	chr18	21242232	21526112	283880	71	461	511.8333333	23.09550961	0.662647643	0.018289835	+	80000	grey	-0.002930412	FALSE	GREB1L
Region_8356	chr18	21380286	21451017	70731	18	116	144.9545455	11.88073012	0.889214564	0.030341136	-	101927521	grey	-0.060754955	FALSE	LOC101927521
Region_8357	chr18	21529284	21600884	71600	23	152	174.0454545	14.1925569	0.761416863	0.014509005	-	114799	grey	-0.035696701	FALSE	ESCO1
Region_8358	chr18	21612314	21633520	21206	7	37	59.18181818	7.967065073	0.968834048	0.026735357	+	6632	grey	-0.056031769	FALSE	SNRPD1
Region_8359	chr18	21650901	21704780	53879	10	62	79.57575758	9.913565851	0.872533992	0.024784368	-	171586	grey	0.017221336	FALSE	ABHD3
Region_8360	chr18	21704957	21870953	165996	45	327	365.2424242	21.13841607	0.916122229	0.016137488	+	57534	grey	0.125774147	FALSE	MIB1
Region_8361	chr18	21981121	22051159	70038	11	52	71.71212121	9.580051632	0.922710014	0.040884793	+	102724208	grey	0.019000133	FALSE	LINC01900
Region_8362	chr1	22169589	22202528	32939	11	58	83.2121212	10.8351495	0.49963329	0.04140413	+	2627	grey	0.023888956	FALSE	GATA6

	8						1	7	1	1						
Region_8363	chr1<br>8	22723491	22907721	184230	21	127	153.787878<br>8	13.0708308<br>9	0.83072113	0.03250738	-	10192757<br>1	grey	-0.02064702<br>1	FALSE	LOC101927571
Region_8364	chr1<br>8	22798261	23026488	228227	28	179	213.272727<br>3	14.8391845<br>7	0.79731288<br>1	0.02383570<br>6	+	5932	grey	-0.31922346<br>4	FALSE	RBBP8
Region_8365	chr1<br>8	23134564	23260470	125906	26	190	223.742424<br>2	15.9115928<br>8	0.70031523<br>1	0.01862587<br>7	+	91768	grey	0.0574557	FALSE	CABLES1
Region_8366	chr1<br>8	23197755	23437961	240206	29	187	225.727272<br>7	16.2002331	0.85092960<br>4	0.02142945<br>6	-	85019	grey	-0.25604624<br>1	FALSE	TMEM241
Region_8367	chr1<br>8	23453287	23486603	33316	5	20	32.3030303	5.69472008<br>4	0.77459421<br>1	0.02890177<br>5	+	8780	grey	0.093848413	FALSE	RIOK3
Region_8368	chr1<br>8	23503470	23531822	28352	8	46	59.8787878<br>8	6.38391042<br>8	0.45851333<br>7	0.02738485<br>8	+	29919	grey	0.300503091	FALSE	RMC1
Region_8369	chr1<br>8	23506184	23586506	80322	15	102	122.515151<br>5	10.3837779<br>2	0.93745503<br>9	0.02324000<br>7	-	4864	grey	0.389680957	FALSE	NPC1
Region_8370	chr1<br>8	23598926	23662911	63985	14	82	108.772727<br>3	11.1075794<br>7	0.81841883<br>1	0.02205241<br>8	-	147463	grey	0.02442287	FALSE	ANKRD29
Region_8371	chr1<br>8	23689453	23956222	266769	31	224	256.848484<br>8	15.5090469<br>1	0.84371019<br>4	0.01963286<br>2	+	3909	grey	0.22679445	FALSE	LAMA3
Region_8372	chr1<br>8	23992773	24135610	142837	42	291	337.333333<br>3	20.3812382<br>3	0.86672459<br>8	0.01983541<br>3	+	125488	grey	0.096587526	FALSE	TTC39C
Region_8373	chr1<br>8	23994213	24015339	21126	7	29	45.3484848<br>5	7.43689849<br>4	0.73242961<br>3	0.06025925<br>9	-	10272424<br>6	grey	-0.04992280<br>4	FALSE	TTC39C-AS1
Region_8374	chr1<br>8	24138987	24161600	22613	6	32	47.5	6.28857327<br>6	0.81482489<br>5	0.03784212<br>4	+	26256	grey	0.068259246	FALSE	CABYR

Region_8375	chr18	24162045	24397880	235835	34	224	262.7121212	13.34139325	0.906689311	0.023104674	-	114876	grey	-0.308976447	FALSE	OSBPL1A
Region_8376	chr18	24426634	24453531	26897	4	20	31	5.53867521	0.509610707	0.049675913	+	55364	grey	-0.098741744	FALSE	IMPACT
Region_8377	chr18	24460637	24479961	19324	3	13	23.93939394	4.48888812	0.759578105	0.093250661	+	59340	grey	0.134923041	FALSE	HRH4
Region_8378	chr18	24534551	24708542	173991	12	85	106.2424242	10.14519304	0.880471373	0.028513511	+	729950	grey	0.052383333	FALSE	LINC01915
Region_8379	chr18	24725762	24986980	261218	24	168	213.3484848	15.4973868	0.850787888	0.026304782	+	105372028	grey	-0.259728246	FALSE	LOC105372028
Region_8380	chr18	24932771	24987919	55148	7	45	61.84848485	7.608178644	0.904795091	0.048132707	-	105372030	grey	-0.156595652	FALSE	LINC01894
Region_8381	chr18	25061924	25352190	290266	32	226	258.6969697	14.5157418	0.825487743	0.025974572	-	25925	grey	0.493011374	FALSE	ZNF521
Region_8382	chr18	26016253	26091217	74964	7	46	58.36363636	6.858204214	0.689343506	0.023173457	-	6760	grey	-0.190394983	FALSE	SS18
Region_8383	chr18	26133852	26193355	59503	4	12	24.46969697	5.418542799	0.927143994	0.079832119	+	143471	grey	-0.057450548	FALSE	PSMA8
Region_8384	chr18	26226445	26391685	165240	16	114	138.5757576	11.54131123	0.943804837	0.028809514	+	6875	grey	-0.235523549	FALSE	TAF4B
Region_8385	chr18	26454910	26657401	202491	25	172	212.0757576	13.86563056	0.818499267	0.024729862	-	284252	grey	0.13327756	FALSE	KCTD1
Region_8386	chr18	26655737	26689668	33931	4	22	34.74242424	5.897354312	0.310983744	0.100940773	+	102725227	grey	-0.028577052	FALSE	LOC102725227
Region_8387	chr1	26655742	26998768	343026	29	191	233.136363	14.4880708	0.77113219	0.02799059	+	147429	grey	-0.16078813	FALSE	AQP4-AS1

	8						6	1	3	3				1		
Region_8388	chr18	26865307	27190698	325391	19	129	150.3030303	11.38956296	0.740768336	0.039716617	+	107985143	grey	-0.289126829	FALSE	
Region_8389	chr18	26906481	27185308	278827	15	100	122.8939394	10.46184097	0.700016308	0.042642718	-	83539	grey	-0.274981624	FALSE	CHST9
Region_8390	chr18	27335968	27392867	56899	5	24	39.71212121	6.527492513	0.900624462	0.047920686	-	105372038	grey	0.218006496	FALSE	LOC105372038
Region_8391	chr18	27336379	27595164	258785	22	144	181.3939394	16.69350752	0.821088526	0.032994242	-	107985126	grey	0.026941161	FALSE	LOC107985126
Region_8392	chr18	27375310	27401910	26600	4	21	34.40909091	6.697823464	0.895948378	0.056838852	+	105372041	grey	0.035039743	FALSE	LOC105372041
Region_8393	chr18	27785821	27795637	9816	4	18	28.53030303	5.265912725	0.898814443	0.081843113	+	105372042	grey	0.193825844	FALSE	LOC105372042
Region_8394	chr18	27932879	28177946	245067	16	115	140.0606061	10.14946575	0.868536486	0.033295005	-	1000	grey	-0.008305172	FALSE	CDH2
Region_8395	chr18	29629842	29650440	20598	3	10	17.59090909	3.90257222	0.663458266	0.145468184	+	105372045	grey	0.048321064	FALSE	LOC105372045
Region_8396	chr18	30290161	30414073	123912	10	47	74.54545455	10.66436455	0.844031344	0.050944639	+	105372046	grey	-0.162087454	FALSE	LOC105372046
Region_8397	chr18	30989365	31042815	53450	7	37	56.34848485	7.904219758	0.631653587	0.050979185	-	1825	grey	0.098395249	FALSE	DSC3
Region_8398	chr18	31058840	31102522	43682	3	14	26.54545455	4.883825166	0.78313088	0.138485706	-	1824	grey	0.004777114	FALSE	DSC2
Region_8399	chr18	31101540	31163414	61874	4	19	33.95454545	6.984448259	0.802268053	0.106793561	+	101927698	grey	0.415837484	FALSE	DSCAS

Region_8400	chr18	31129236	31162856	33620	4	19	33.95454545	6.984448259	0.802268053	0.106793561	-	1823	grey	0.415837484	FALSE	DSC1
Region_8401	chr18	31318160	31359246	41086	4	16	25.92424242	5.054504788	0.834872608	0.084432184	+	1828	grey	0.133780493	FALSE	DSG1
Region_8402	chr18	31343368	31426988	83620	11	73	86.90909091	7.924115622	0.789810841	0.047341722	-	101927718	grey	-0.025600309	FALSE	DSG1-AS1
Region_8403	chr18	31376777	31414912	38135	3	13	21.53030303	4.308202184	0.611128322	0.087824741	+	147409	grey	-0.032937401	FALSE	DSG4
Region_8404	chr18	31622246	31685836	63590	11	69	95.24242424	9.456069449	0.652621435	0.03767186	-	9331	grey	0.280067387	FALSE	B4GALT6
Region_8405	chr18	31829197	31943136	113939	10	70	87.43939394	9.326766754	0.872925954	0.01615425	-	22878	grey	0.08380368	FALSE	TRAPPC8
Region_8406	chr18	32018825	32111779	92954	17	107	134.7878788	12.66432318	0.810771117	0.034955436	+	54941	grey	0.070623843	FALSE	RNF125
Region_8407	chr18	32091874	32131561	39687	5	25	37.03030303	5.791159568	0.901067215	0.042064831	+	51444	grey	0.206327787	FALSE	RNF138
Region_8408	chr18	32124877	32470882	346005	43	326	368.3787879	18.90442777	0.859515491	0.020177462	-	64762	grey	-0.016708021	FALSE	GAREM1
Region_8409	chr18	32470288	32745567	275279	24	149	186.9242424	15.26313871	0.786617067	0.026374878	+	112268208	grey	0.324213484	FALSE	LOC112268208
Region_8410	chr18	32672673	32773023	100350	6	36	49.18181818	7.439828557	0.571090796	0.04338762	-	57565	grey	0.247857252	FALSE	KLHL14
Region_8411	chr18	32833454	32938316	104862	12	89	106.969697	9.18857099	0.738106331	0.042213081	+	105372058	grey	0.022813619	FALSE	LOC105372058
Region_8412	chr1	32937402	33441101	503699	46	327	387.712121	18.9832032	0.84307953	0.02045318	-	374864	grey	0.437944056	FALSE	CCDC178

	8						2	2	1	3						
Region_8413	chr18	33578219	33751195	172976	12	81	101.8484848	10.47963949	0.801768238	0.042465535	+	80816	grey	-0.165823323	FALSE	ASXL3
Region_8414	chr18	33851100	34224952	373852	32	219	256.1666667	17.6509606	0.686768853	0.026430504	-	8715	grey	-0.113869062	FALSE	NOL4
Region_8415	chr18	34493291	34891844	398553	40	304	343.5909091	21.28269595	0.778048458	0.01963075	+	1837	grey	0.141449528	FALSE	DTNA
Region_8416	chr18	34976928	35143470	166542	31	245	275	15.02920234	0.764730245	0.021505854	+	10982	grey	0.015556281	FALSE	MAPRE2
Region_8417	chr18	35241030	35267133	26103	4	23	35.5	5.19245014	0.975694785	0.02772967	+	84307	grey	0.136933971	FALSE	ZNF397
Region_8418	chr18	35251058	35290245	39187	7	36	49.60606061	7.302009814	0.582702981	0.04718931	-	100101467	grey	0.110269724	FALSE	ZSCAN30
Region_8419	chr18	35290276	35328390	38114	7	33	52.28787879	7.026904673	0.828888518	0.043655278	+	10778	grey	-0.311336875	FALSE	ZNF271P
Region_8420	chr18	35332227	35345482	13255	3	21	30.77272727	4.819177889	0.901109985	0.064551626	-	7572	grey	-0.162792123	FALSE	ZNF24
Region_8421	chr18	35366694	35377337	10643	5	18	36.95454545	6.767414728	0.31872891	0.088954814	-	252884	grey	0.014784561	FALSE	ZNF396
Region_8422	chr18	35443871	35497979	54108	4	30	41.46969697	6.003126613	0.504935495	0.042425768	-	125476	grey	-0.230167851	FALSE	INO80C
Region_8423	chr18	35581117	35711834	130717	13	77	94.18181818	8.514077195	0.963921102	0.027545999	+	2589	grey	0.018390961	FALSE	GALNT1
Region_8424	chr18	35972625	35979286	6661	4	25	36.03030303	5.227058879	0.006415523	0.013427996	+	83608	grey	-0.193913132	FALSE	C18orf21

Region_8425	chr18	35984387	36067576	83189	11	67	88.6969697	9.788485696	0.956494987	0.030603747	-	55197	grey	-0.126565582	FALSE	RPRD1A
Region_8426	chr18	36129444	36180557	51113	6	39	57.16666667	7.316121994	0.785427056	0.037110978	+	55250	grey	-0.009016148	FALSE	ELP2
Region_8427	chr18	36187497	36272157	84660	5	26	41.01515152	5.731136078	0.934602794	0.039685996	+	55034	grey	0.056414912	FALSE	MOCOS
Region_8428	chr18	36297713	36780220	482507	40	308	348.7575758	19.97156487	0.734046105	0.019930059	+	80206	grey	0.164105661	FALSE	FHOD3
Region_8429	chr18	36777647	36829216	51569	8	47	62.5	7.632420727	0.726177363	0.025955256	-	25941	grey	-0.529718429	FALSE	TPGS2
Region_8430	chr18	36829106	37231518	402412	25	172	201.2272727	13.49734499	0.894955889	0.021791885	+	57536	grey	0.013350855	FALSE	KIAA1328
Region_8431	chr18	37243040	37565827	322787	45	336	377.2272727	23.03361788	0.7843133	0.023044436	-	56853	grey	0.054951183	FALSE	CELF4
Region_8432	chr18	37565281	37762131	196850	16	117	137.2575758	10.71348626	0.741950564	0.036767184	+	105372072	grey	-0.074552756	FALSE	
Region_8433	chr18	37567723	37638931	71208	6	32	44.54545455	5.338342636	0.729674631	0.074513942	+	107985118	grey	-0.234891349	FALSE	LOC107985118
Region_8434	chr18	39206917	39800322	593405	45	342	396.5	20.81659841	0.896682852	0.022750259	-	647946	grey	0.012546168	FALSE	MIR924HG
Region_8435	chr18	39275634	39340622	64988	5	16	33.40909091	6.591316602	0.956527597	0.043170836	-	112268214	grey	0.056064082	FALSE	
Region_8436	chr18	39841174	39924840	83666	9	44	59.22727273	8.406582502	0.813619293	0.047356276	-	105372080	grey	0.304084328	FALSE	LINC01901
Region_8437	chr1	39841688	39896298	54610	8	38	52.9090909	8.05696501	0.79514252	0.05266823	+	10537207	grey	0.28810212	FALSE	LINC01902

	8						1	9	8	2		9				
Region_8438	chr18	41341319	41507550	166231	8	39	56.31818182	7.471297057	0.779839953	0.061255384	+	105372083	grey	-0.052017203	FALSE	LOC105372083
Region_8439	chr18	41480271	41632185	151914	8	54	69.92424242	8.494904547	0.860678297	0.051091279	-	641516	grey	-0.20468582	FALSE	KC6
Region_8440	chr18	41955234	42087830	132596	7	45	62.95454545	7.650906066	0.753797823	0.031913017	+	5289	grey	-0.23738388	FALSE	PIK3C3
Region_8441	chr18	42159283	42691422	532139	38	286	325.3333333	16.65517553	0.845271986	0.023926434	+	284260	grey	-0.105596175	FALSE	LINC00907
Region_8442	chr18	42743227	43115691	372464	32	199	238.1363636	17.03470337	0.782117592	0.029396531	-	6014	grey	0.111249411	FALSE	RIT2
Region_8443	chr18	43267892	43277499	9607	3	16	24.53030303	4.40705622	0.285671666	0.076161722	-	6860	grey	-0.053244853	FALSE	SYT4
Region_8444	chr18	44280768	44579723	298955	36	277	319.530303	17.90592484	0.913306384	0.017230923	-	101927921	grey	-0.032185135	FALSE	LINC01478
Region_8445	chr18	44680173	45068510	388337	52	358	409.6969697	23.84176665	0.857500834	0.014839625	+	26040	grey	0.212037028	FALSE	SETBP1
Region_8446	chr18	45212995	45683688	470693	70	557	615.0909091	29.38169355	0.877460587	0.015056684	+	8170	grey	-0.019233292	FALSE	SLC14A2
Region_8447	chr18	45669367	45747215	77848	19	148	175.1363636	13.85072768	0.827806441	0.029476524	-	105372093	grey	0.298037816	FALSE	LOC105372093
Region_8448	chr18	45687025	45725019	37994	9	67	84.48484848	9.62567468	0.798926474	0.043671424	+	105372092	grey	0.317396925	FALSE	LOC105372092
Region_8449	chr18	45724127	45752520	28393	6	37	50.51515152	6.603594416	0.929473633	0.037918697	+	6563	grey	-0.206171854	FALSE	SLC14A1

Region_8450	chr18	45800581	45967329	166748	38	282	318.5909091	18.57208598	0.863947025	0.018093203	-	57724	grey	0.05140019	FALSE	EPG5
Region_8451	chr18	45825675	45844094	18419	9	61	73.31818182	8.740989366	0.582146382	0.043572636	+	284266	grey	0.001935363	FALSE	SIGLEC15
Region_8452	chr18	45983536	46072272	88736	30	169	211.5	23.57590034	0.825174266	0.028504487	-	9050	grey	-0.001094229	FALSE	PSTPIP2
Region_8453	chr18	46080248	46104334	24086	10	47	71.74242424	9.862610992	0.690343846	0.03376345	-	498	grey	0.110504275	FALSE	ATP5F1A
Region_8454	chr18	46104378	46128333	23955	11	75	91.04545455	8.853390169	0.973536495	0.019055498	+	115106	grey	-0.194807409	FALSE	HAUS1
Region_8455	chr18	46173553	46266992	93439	30	210	248.3030303	15.33118461	0.811956438	0.015475723	+	147339	grey	0.125363424	FALSE	ARK2N
Region_8456	chr18	46326809	46463140	136331	58	432	478.5606061	24.83801602	0.813952548	0.016283225	+	494470	grey	0.075934434	FALSE	ARK2C
Region_8457	chr18	46476972	46657220	180248	32	251	289.9090909	20.31954517	0.844826367	0.023003033	-	125336	grey	0.165726348	FALSE	LOXHD1
Region_8458	chr18	46667821	46759257	91436	11	70	86	9.802668397	0.551823933	0.046642158	-	29906	grey	0.082266829	FALSE	ST8SIA5
Region_8459	chr18	46756933	46802449	45516	9	48	70.04545455	8.690280722	0.581269755	0.060037368	+	105372097	grey	-0.026847617	FALSE	ST8SIA5-DT
Region_8460	chr18	46803218	46920160	116942	20	141	170.1363636	13.37838252	0.932652064	0.021202941	-	9063	grey	0.003325324	FALSE	PIAS2
Region_8461	chr18	46917492	47103478	185986	24	132	202.3484848	28.11815314	0.844993767	0.039346309	+	83473	grey	0.336018872	FALSE	KATNAL2
Region_8462	chr1	47107408	47176345	68937	12	84	108.333333	12.4990256	0.40199140	0.02535017	-	84064	grey	0.063622366	FALSE	HDHD2

	8						3		7	6						
Region_8463	chr18	47134656	47176364	41708	10	64	87.83333333	11.7821029	0.380667782	0.019715177	-	51124	grey	0.058808857	FALSE	IER3IP1
Region_8464	chr18	47206169	47251660	45491	7	40	54.16666667	7.701315239	0.610432867	0.051666078	-	652991	grey	-0.036860211	FALSE	SKOR2
Region_8465	chr18	47285725	47594550	308825	37	263	306.530303	16.0645043	0.769315892	0.030837357	+	110175910	grey	-0.07831931	FALSE	MIR4527HG
Region_8466	chr18	47808957	47931146	122189	28	195	222.3333333	15.1010273	0.782339658	0.025395007	-	4087	grey	-0.146258418	FALSE	SMAD2
Region_8467	chr18	48026672	48410752	384080	87	625	693.5151515	32.19471644	0.725978979	0.013735499	-	201501	grey	0.007238107	FALSE	ZBTB7C
Region_8468	chr18	48919853	48950965	31112	13	57	79.51515152	9.537363648	0.540305541	0.044449748	-	4092	grey	0.270599634	FALSE	SMAD7
Region_8469	chr18	49023703	49048474	24771	4	15	27.3030303	4.472031708	0.894726845	0.091708546	+	100129878	grey	0.033437753	FALSE	DYM-AS1
Region_8470	chr18	49036387	49461347	424960	43	328	369.3636364	18.99525152	0.932055986	0.015350431	-	54808	grey	0.283024007	FALSE	DYM
Region_8471	chr18	49481681	49492479	10798	4	30	42.5	6.594286571	0.250506591	0.042152769	-	100526842	grey	-0.256537113	FALSE	RPL17-C18orf32
Region_8472	chr18	49560699	49599185	38486	7	44	64.34848485	8.497044598	0.933370231	0.038065779	+	9388	grey	-0.05381996	FALSE	LIPG
Region_8473	chr18	49782164	49813953	31789	4	20	29.72727273	5.723219789	0.490359206	0.033342567	-	10449	grey	-0.073092873	FALSE	ACAA2
Region_8474	chr18	49795793	50195164	399371	44	330	392	20.79718916	0.73609876	0.026410021	-	4645	grey	-0.331517848	FALSE	MYO5B

Region_8475	chr18	50266882	50281774	14892	4	17	35.81818182	7.597386374	0.97629476	0.025242314	-	4152	grey	0.086229745	FALSE	MBD1
Region_8476	chr18	50560087	50731826	171739	17	100	128.4848485	12.14938102	0.860824139	0.035153646	+	5596	grey	-0.002254935	FALSE	MAPK4
Region_8477	chr18	50795120	50825402	30282	7	47	63.95454545	6.969012331	0.634083551	0.034608566	-	83876	grey	-0.061261165	FALSE	MRO
Region_8478	chr18	50879080	50954257	75177	9	46	60.39393939	6.878905968	0.857087423	0.020212994	+	4200	grey	0.1954597	FALSE	ME2
Region_8479	chr18	50967991	51047111	79120	5	23	45.98484848	6.232649577	0.722477941	0.046792113	+	55520	grey	-0.065209007	FALSE	ELAC1
Region_8480	chr18	50968019	51085045	117026	6	26	51.34848485	6.806088732	0.762198228	0.041722151	+	4089	grey	-0.061375075	FALSE	SMAD4
Region_8481	chr18	51174550	51218333	43783	7	37	51.57575758	6.954933182	0.593813284	0.049947184	-	51320	grey	0.243933499	FALSE	MEX3C
Region_8482	chr18	51346275	51643939	297664	20	139	167.3181818	12.48526404	0.902022783	0.027995907	+	100287225	grey	-0.165224024	FALSE	LINC01630
Region_8483	chr18	52340197	53535903	1195706	117	932	1026.80303	35.75911705	0.807938007	0.017402985	+	1630	grey	-0.260130259	FALSE	DCC
Region_8484	chr18	53480825	53581107	100282	7	24	40.13636364	6.213846483	0.945955298	0.038174861	-	101928167	grey	-0.256852156	FALSE	LINC01917
Region_8485	chr18	54151606	54224669	73063	8	49	66.65151515	7.596542582	0.953503087	0.029980707	-	8932	grey	0.010078969	FALSE	MBD2
Region_8486	chr18	54269517	54321266	51749	7	33	50.75757576	7.499867909	0.949080894	0.03567924	+	11201	grey	0.035487031	FALSE	POLI
Region_8487	chr1	54324358	54357964	33606	5	26	38.4545454	6.73722567	0.88933199	0.05454444	-	147323	grey	0.047425161	FALSE	STARD6

	8						5	5	8							
Region_8488	chr18	54357906	54385218	27312	3	11	21.86363636	4.860789311	0.96735774	0.04284937	+	162681	grey	-0.179115924	FALSE	C18orf54
Region_8489	chr18	54717860	54895516	177656	14	102	124.530303	9.703638805	0.772477806	0.02996131	+	5874	grey	-0.414596425	FALSE	RAB27B
Region_8490	chr18	54901509	54959461	57952	9	44	83.16666667	9.662908199	0.942990445	0.027190364	-	80323	grey	0.159026246	FALSE	CCDC68
Region_8491	chr18	55222185	55664787	442602	77	594	666.5606061	27.88279248	0.801716413	0.017550329	-	6925	grey	-0.001198126	FALSE	TCF4
Region_8492	chr18	55452533	55482940	30407	5	35	50.53030303	7.142978687	0.913464738	0.052614467	+	105372127	grey	0.18159626	FALSE	TCF4-AS1
Region_8493	chr18	55589904	55839184	249280	32	229	264.2878788	16.9911879	0.805213418	0.027464971	+	105372130	grey	-0.069418304	FALSE	LOC105372130
Region_8494	chr18	55881688	55998219	116531	21	162	202.7878788	12.99054704	0.87222248	0.028746588	-	101927273	grey	0.035822884	FALSE	LINC01416
Region_8495	chr18	55893038	56026082	133044	18	142	176.0757576	12.58730815	0.89783496	0.030283555	+	107985164	grey	-0.066079153	FALSE	LINC03092
Region_8496	chr18	56003613	56191262	187649	18	109	143.3030303	11.88142661	0.908367816	0.030786756	-	642484	grey	0.062460525	FALSE	LINC03069
Region_8497	chr18	56031830	56135295	103465	9	42	67.06060606	8.26648244	0.931160218	0.041150652	+	102724698	grey	0.110569484	FALSE	LINC01905
Region_8498	chr18	56597209	56651593	54384	13	69	94.22727273	10.23537674	0.50887225	0.025556614	-	9352	grey	-0.069978358	FALSE	TXNL1
Region_8499	chr18	56651343	57029811	378468	55	390	446.6818182	22.52120989	0.885328432	0.015468678	+	23335	grey	0.622347153	FALSE	WDR7

Region_8500	chr18	57054558	57072119	17561	13	77	99.74242424	10.9745308	0.908708243	0.039020192	-	100885779	grey	-0.119483957	FALSE	LINC-ROR
Region_8501	chr18	57352557	57371731	19174	4	23	34.04545455	4.900335367	0.707789211	0.041029554	+	51046	grey	-0.056875786	FALSE	ST8SIA3
Region_8502	chr18	57435374	57491298	55924	14	102	127.0606061	11.03961926	0.40270766	0.029051673	+	9480	grey	-0.000328031	FALSE	ONECUT2
Region_8503	chr18	57544377	57586732	42355	9	52	71.90909091	8.857378103	0.76764058	0.034132274	-	2235	grey	-0.108201355	FALSE	FECH
Region_8504	chr18	57600656	57622213	21557	5	20	32.65151515	5.696132086	0.899056297	0.083092079	-	4677	grey	-0.173165127	FALSE	NARS1
Region_8505	chr18	57630280	57669296	39016	21	129	154.1515152	13.86000602	0.920193226	0.020378209	+	100505549	grey	0.14608996	FALSE	ATP8B1-AS1
Region_8506	chr18	57639455	57738603	99148	33	221	263.0151515	16.85457475	0.932086225	0.016241575	+	105372141	grey	0.107364739	FALSE	LOC105372141
Region_8507	chr18	57646426	57803315	156889	36	249	289.7575758	17.21453823	0.908383335	0.01375392	-	5205	grey	0.223913725	FALSE	ATP8B1
Region_8508	chr18	58044226	58401540	357314	51	385	454.030303	22.79606558	0.880709258	0.016828451	+	23327	grey	0.113576891	FALSE	NEDD4L
Region_8509	chr18	58481247	58629091	147844	20	132	172.8484848	13.08815369	0.877782567	0.029957368	-	115701	grey	-0.147833382	FALSE	ALPK2
Region_8510	chr18	58671465	58754477	83012	9	57	74.42424242	8.474505851	0.820510731	0.036275027	+	10892	grey	-0.012745268	FALSE	MALT1
Region_8511	chr18	58672613	58752730	80117	9	57	74.42424242	8.474505851	0.820510731	0.036275027	-	105372146	grey	-0.012745268	FALSE	LOC105372146
Region_8512	chr1	58808468	58834364	25896	5	28	43.2878787	6.05809097	0.90978274	0.05066874	+	10537214	grey	-0.04596963	FALSE	LINC01926

	8						9		5	6		7		3		
Region_8513	chr18	58862600	58986480	123880	23	155	199.1515152	15.45638272	0.875934028	0.022050794	+	55205	grey	0.051214392	FALSE	ZNF532
Region_8514	chr18	58996734	59069338	72604	10	59	85.71212121	10.18560848	0.932222617	0.032674305	+	390858	grey	0.10911773	FALSE	OACYLP
Region_8515	chr18	59220158	59230774	10616	7	27	50.43939394	8.58644511	0.309592643	0.049127818	+	2922	grey	-0.046499759	FALSE	GRP
Region_8516	chr18	59267035	59273454	6419	7	25	42.95454545	9.264376467	0.038006624	0.031763796	-	30062	grey	0.223297546	FALSE	RAX
Region_8517	chr18	59275156	59318649	43493	7	32	48.8030303	7.106269598	0.920375911	0.0507914	-	339302	grey	-0.003650677	FALSE	CPLX4
Region_8518	chr18	59430939	59697662	266723	39	282	325.2727273	17.89675128	0.785171864	0.02695609	-	147372	grey	-0.009582198	FALSE	CCBE1
Region_8519	chr18	60611773	60654623	42850	6	30	46.98484848	7.24407965	0.882312911	0.050801112	+	105372156	grey	6.22998E-05	FALSE	LOC105372156
Region_8520	chr18	61333430	61555779	222349	26	187	214.9393939	13.44032828	0.76553889	0.027148281	+	28316	grey	-0.276279342	FALSE	CDH20
Region_8521	chr18	61748082	61758464	10382	5	24	34.46969697	5.446861332	0.911391231	0.056989521	+	100996669	grey	-0.070447716	FALSE	LINC01544
Region_8522	chr18	61808067	61894247	86180	14	109	128.5757576	11.27565193	0.857499641	0.033956471	-	220441	grey	0.13902413	FALSE	RNF152
Region_8523	chr18	61905255	62187118	281863	15	96	125.1969697	11.00939417	0.900322839	0.037346792	-	23556	grey	-0.315695611	FALSE	PIGN
Region_8524	chr18	62187255	62310249	122994	5	18	36.45454545	7.003695328	0.921413144	0.064986479	+	57614	grey	-0.239760778	FALSE	RELCH

Region_8525	chr18	62325287	62391288	66001	12	95	110.1060606	9.366271383	0.853088484	0.031574135	+	8792	grey	-0.190026486	FALSE	TNFRSF11A
Region_8526	chr18	62523025	62587709	64684	14	79	98.60606061	11.41238031	0.477733043	0.020099351	+	54877	grey	-0.145104105	FALSE	ZCCHC2
Region_8527	chr18	62715541	62980433	264892	30	208	249.7424242	15.64147407	0.949437315	0.014990193	+	23239	grey	-0.032706926	FALSE	PHLPP1
Region_8528	chr18	63123346	63320128	196782	32	225	261.9848485	16.96012738	0.633693572	0.026597006	-	596	grey	0.169346326	FALSE	BCL2
Region_8529	chr18	63327726	63367228	39502	8	47	67.12121212	7.699181078	0.825855919	0.023190547	-	2531	grey	0.108028369	FALSE	KDSR
Region_8530	chr18	63389190	63422483	33293	10	73	92.96969697	8.880349433	0.869781579	0.063556013	-	9525	grey	0.224155532	FALSE	VPS4B
Region_8531	chr18	63647579	63726432	78853	6	34	49.54545455	7.512413969	0.929719648	0.045432273	+	89778	grey	0.0421992	FALSE	SERPINB11
Region_8532	chr18	63871692	63917490	45798	4	26	37.33333333	5.619562899	0.745685781	0.045931058	+	5055	grey	0.088532809	FALSE	SERPINB2
Region_8533	chr18	63949357	64019779	70422	3	12	23.33333333	4.421393214	0.621816287	0.057180177	+	5271	grey	0.048356613	FALSE	SERPINB8
Region_8534	chr18	64041555	64423601	382046	24	191	233.469697	14.95039973	0.859984995	0.028976754	+	284294	grey	-0.055471208	FALSE	LINC01924
Region_8535	chr18	64181314	64260055	78741	6	42	60.28787879	8.669602336	0.878637273	0.058906633	-	400654	grey	0.101932963	FALSE	LINC01538
Region_8536	chr18	64872264	64992828	120564	7	44	57.90909091	6.3844216	0.835353924	0.049686683	-	101927404	grey	0.014069861	FALSE	LOC101927404
Region_8537	chr1	65423958	65448167	24209	3	16	28.5	5.39301542	0.90984813	0.07052750	+	10798512	grey	0.025019433	FALSE	LINC01916

	8							6	3	4		8				
Region_15067	chr5	140786136	141012347	226211	149	1204	1461.227273	103.1686887	0.508765118	0.029331001	+	56147	blue	0.928483497	FALSE	PCDHA1
Region_8539	chr18	66501083	66604138	103055	6	35	53.87878788	8.037441521	0.901368675	0.038446282	-	28513	grey	0.083029523	FALSE	CDH19
Region_8540	chr18	67516546	67899619	383073	37	286	323.3939394	16.47644359	0.841098046	0.019261069	+	643542	grey	-0.139546672	FALSE	DSEL-AS1
Region_8541	chr18	67672221	67767051	94830	7	30	46.68181818	7.819421759	0.916135683	0.04431364	-	105372173	grey	-0.403708596	FALSE	LOC105372173
Region_8542	chr18	67808505	67814822	6317	3	14	22.10606061	4.624778707	0.877554038	0.095524893	-	101927430	grey	-0.082650439	FALSE	LINC01903
Region_8543	chr18	68673688	68715108	41420	9	48	70.22727273	10.12809566	0.959044719	0.041596413	-	54495	grey	0.080402573	FALSE	TMX3
Region_8544	chr18	68715209	69055189	339980	31	236	267.5606061	16.16180568	0.81539292	0.024545912	+	79839	grey	0.122042728	FALSE	CCDC102B
Region_8545	chr18	69400888	69849087	448199	40	301	342.9242424	20.24033339	0.840416634	0.02195757	+	220164	grey	0.369133375	FALSE	DOK6
Region_8546	chr18	69831158	69961803	130645	23	181	218.0454545	16.96823441	0.8870279	0.030262019	-	10666	grey	0.268075961	FALSE	CD226
Region_8547	chr18	70003031	70205726	202695	17	102	122.1363636	10.18277031	0.882751657	0.025393897	-	25914	grey	-0.230786721	FALSE	RTTN
Region_8548	chr18	70289045	70330199	41154	8	38	57.1969697	7.782069523	0.941777257	0.035779586	+	9306	grey	0.032798792	FALSE	SOCS6
Region_8549	chr18	70464917	70555465	90548	18	112	148.0606061	14.15070402	0.843376534	0.032102335	+	105376872	grey	-0.266094804	FALSE	LOC105376872

Region_8550	chr18	71137829	71208366	70537	6	34	49.59090909	6.867935022	0.809675966	0.068365006	+	105372188	grey	0.024818879	FALSE	LOC105372188
Region_8551	chr18	71519962	71578956	58994	6	30	45.36363636	7.013473547	0.751980497	0.066369299	-	100505776	grey	-0.191472481	FALSE	LINC01541
Region_8552	chr18	72536680	72638521	101841	14	80	104.8030303	10.32132922	0.510944909	0.03396767	-	147381	grey	0.075606698	FALSE	CBLN2
Region_8553	chr18	72742314	72867987	125673	47	321	373.5909091	23.50972124	0.655240122	0.025059753	-	81832	grey	-0.147681304	FALSE	NETO1
Region_8554	chr18	72868388	72881404	13016	8	36	55.34848485	7.100362773	0.453960994	0.055036107	+	100505797	grey	-0.053209313	FALSE	NETO1-DT
Region_8555	chr18	73151241	73264480	113239	16	111	135.7272727	11.20788933	0.903467968	0.024715884	-	400655	grey	0.114642592	FALSE	LINC02864
Region_8556	chr18	73324941	73349879	24938	5	18	30.16666667	5.551391602	0.910572134	0.061750207	+	100505817	grey	-0.200543202	FALSE	LINC02582
Region_8557	chr18	74073353	74147843	74490	34	320	352.1060606	15.5307425	0.818420486	0.01941883	-	201456	grey	0.060221751	FALSE	FBXO15
Region_8558	chr18	74148523	74160531	12008	7	36	51.8030303	8.482052903	0.827611626	0.025303549	+	29090	grey	-0.16780209	FALSE	TIMM21
Region_8559	chr18	74250846	74291973	41127	27	187	250.5	23.48174414	0.482751467	0.02329349	-	1528	grey	0.094173217	FALSE	CYB5A
Region_8560	chr18	74315839	74359189	43350	8	49	63.15151515	7.179548784	0.903081758	0.038132016	+	644041	grey	0.023522329	FALSE	C18orf63
Region_8561	chr18	74409448	74413209	3761	7	39	55.62121212	6.151335444	0.942126559	0.041215126	+	101927606	grey	-0.053292261	FALSE	LINC01922
Region_8562	chr1	74434775	74457944	23169	19	124	151.530303	12.5749686	0.53343934	0.02936791	-	125704	grey	0.188835348	FALSE	DIPK1C

	8							6	2	8						
Region_8563	chr18	74495816	74523454	27638	32	219	261.2424242	18.74284323	0.718996754	0.015949259	+	55748	grey	-0.076326501	FALSE	CNDP2
Region_8564	chr18	74534500	74587212	52712	29	206	245.8484848	16.88444202	0.87414117	0.026204643	+	84735	grey	0.018753697	FALSE	CNDP1
Region_8565	chr18	74590317	74598885	8568	3	10	19.43939394	3.742186888	0.001346801	0.007920582	-	400657	grey	0.077610951	FALSE	ZNF407-AS1
Region_8566	chr18	74597870	75065671	467801	126	1038	1114.393939	35.48125346	0.90165339	0.007601617	+	55628	grey	-0.07033087	FALSE	ZNF407
Region_8567	chr18	75195113	75209139	14026	5	24	37.25757576	6.102485259	0.516101568	0.116435624	-	284273	grey	0.022520258	FALSE	PTGR3
Region_8568	chr18	75210755	75289950	79195	25	155	204.3333333	17.39702303	0.842996709	0.017236208	+	10194	grey	-0.397965542	FALSE	TSHZ1
Region_8569	chr18	75409476	75436957	27481	3	19	26.75757576	4.813636419	0.953586894	0.046669695	-	284274	grey	0.135309972	FALSE	SMIM21
Region_8570	chr18	75455787	75468835	13048	6	30	49.24242424	7.801500065	0.470387254	0.029002082	+	107985177	grey	-0.403166599	FALSE	LOC107985177
Region_8571	chr18	75696044	75712851	16807	6	32	46.65151515	7.132789775	0.895349867	0.045415015	-	100505853	grey	0.147900709	FALSE	LINC01898
Region_8572	chr18	76122968	76145259	22291	3	18	28.84848485	5.572576877	0.901777405	0.06557867	+	339298	grey	-0.192692619	FALSE	LOC339298
Region_8573	chr18	76209422	76221718	12296	8	62	77.78787879	8.952034838	0.684797082	0.037814861	+	105372207	grey	0.273517356	FALSE	LINC01893
Region_8574	chr18	76232927	76260114	27187	9	39	57.10606061	7.075697928	0.850959882	0.052428031	+	105372209	grey	0.180291376	FALSE	LOC105372209

Region_8575	chr18	76357682	76495242	137560	129	1034	1138.893939	47.99747711	0.755067792	0.011639547	-	9658	grey	0.111631561	FALSE	ZNF516
Region_8576	chr18	76491629	76493920	2291	3	23	35.45454545	6.018386746	0.005644671	0.012220518	+	101927989	grey	-0.115299982	FALSE	ZNF516-AS1
Region_8577	chr18	76495521	76498088	2567	6	36	52.16666667	7.126512435	0.017632989	0.021419683	+	400658	grey	-0.13971493	FALSE	ZNF516-DT
Region_8578	chr18	76528652	76670063	141411	92	679	739.8030303	32.1641079	0.792586377	0.014379844	+	284276	grey	0.25774469	FALSE	LINC00908
Region_8579	chr18	76528753	76625940	97187	75	543	599.5151515	26.545892	0.784083072	0.015910635	+	400660	grey	0.393965817	FALSE	LINC00683
Region_8580	chr18	76622136	76639030	16894	10	47	67.45454545	8.23161227	0.749712286	0.066989996	-	101927651	grey	-0.184837479	FALSE	LINC01927
Region_8581	chr18	76686058	76693636	7578	9	70	87.09090909	9.015759318	0.890053694	0.043097134	+	400661	grey	-0.046399822	FALSE	LINC01879
Region_8582	chr18	76794732	76822295	27563	20	142	169.4242424	11.62498715	0.701121486	0.029233596	-	100131655	grey	-0.034138214	FALSE	ZNF236-DT
Region_8583	chr18	76822557	76972901	150344	54	409	453.6818182	22.29809174	0.874681399	0.016428503	+	7776	grey	0.245145025	FALSE	ZNF236
Region_8584	chr18	76978827	77133683	154856	101	721	801.1363636	31.76882185	0.712878279	0.016519861	-	4155	grey	-0.354927913	FALSE	MBP
Region_8585	chr18	77249848	77277900	28052	23	149	176.6060606	15.70681262	0.352787721	0.029537536	+	2587	grey	0.243217551	FALSE	GALR1
Region_8586	chr18	77421619	77561044	139425	84	645	708.1969697	28.9632232	0.849249636	0.018326703	+	107985172	grey	0.171667017	FALSE	LOC107985172
Region_8587	chr1	77970637	77993731	23094	9	62	84.5151515	9.66236541	0.88346778	0.05184304	-	10192771	grey	-0.04146981	FALSE	LINC01029

	8						2	2	7	7		5		9		
Region_8588	chr18	77971347	77993722	22375	9	62	84.51515152	9.662365412	0.883467787	0.051843047	-	105372218	grey	-0.041469819	FALSE	
Region_8589	chr19	281040	291403	10363	6	17	34.13636364	7.283039018	0.360445434	0.079520652	-	8612	grey	0.111497212	FALSE	PLPP2
Region_8590	chr19	305573	344815	39242	26	148	182.0757576	14.73489903	0.89467367	0.020535614	-	54531	grey	-0.173242481	FALSE	MIER2
Region_8591	chr19	361747	376026	14279	17	85	121.6515152	12.16108854	0.928879456	0.027241691	-	51298	grey	0.059790222	FALSE	SPMAP2
Region_8592	chr19	405445	409147	3702	13	50	70.16666667	12.34078084	0.540308737	0.041096988	-	126567	grey	0.093933588	FALSE	C2CD4C
Region_8593	chr19	416589	461033	44444	42	227	280.7878788	22.00805108	0.913373405	0.026631339	-	25759	grey	0.234433101	FALSE	SHC2
Region_8594	chr19	463346	474983	11637	6	24	39.87878788	7.701179034	0.904758647	0.053466211	-	284451	grey	-0.016405308	FALSE	CIMAP1D
Region_8595	chr19	489176	505343	16167	29	183	233.1666667	26.32786376	0.512509251	0.024266656	+	8174	grey	-0.266022766	FALSE	MADCAM1
Region_8596	chr19	490046	507833	17787	36	256	305.3181818	28.91481339	0.482841355	0.018642163	-	105372232	grey	-0.250649097	FALSE	MADCAM1-AS1
Region_8597	chr19	507497	519654	12157	43	239	343.8787879	43.81658104	0.460751111	0.070775324	+	91978	grey	0.0911303	FALSE	TPGS1
Region_8598	chr19	531760	542092	10332	21	71	106.1363636	16.91870732	0.422605548	0.055738179	+	997	grey	0.300792313	FALSE	CDC34
Region_8599	chr19	544034	549924	5890	5	16	30.06060606	5.346109406	0.933308106	0.041370154	+	3004	grey	0.038554483	FALSE	GZMM

Region_8600	chr19	562360	572228	9868	18	89	127.1969697	15.84173621	0.41233108	0.029049801	-	105372233	grey	-0.139797802	FALSE	BSG-AS1
Region_8601	chr19	571277	583494	12217	39	212	281.0151515	27.35380296	0.449363939	0.014826689	+	682	grey	-0.140763374	FALSE	BSG
Region_8602	chr19	589881	617159	27278	66	327	409.1060606	38.63881221	0.483664887	0.029813428	+	610	grey	-0.305828802	FALSE	HCN2
Region_8603	chr19	617221	633537	16316	46	231	329.1363636	35.07349925	0.850963986	0.010833706	-	5442	grey	-0.087143314	FALSE	POLRMT
Region_8604	chr19	639879	644373	4494	15	47	90.36363636	17.67017162	0.411071237	0.029301121	+	27006	grey	-0.078423734	FALSE	FGF22
Region_8605	chr19	647526	663233	15707	32	149	200.8030303	25.3736506	0.538754492	0.016802515	-	55658	grey	-0.177923356	FALSE	RNF126
Region_8606	chr19	676392	683392	7000	19	68	100.0606061	16.31787956	0.183365504	0.021447309	+	10272	grey	-0.019451692	FALSE	FSTL3
Region_8607	chr19	685546	695498	9952	6	20	35.18181818	6.025354355	0.864567478	0.063885413	-	400668	grey	-0.261425944	FALSE	PRSS57
Region_8608	chr19	708935	748329	39394	34	140	183.7727273	17.45746079	0.802357803	0.028595292	+	5064	grey	-0.082885877	FALSE	PALM
Region_8609	chr19	751112	764318	13206	12	56	86.33333333	11.3981555	0.832417153	0.023280154	+	126353	grey	0.044787617	FALSE	MISP
Region_8610	chr19	797075	812327	15252	55	270	341.1969697	32.49341694	0.795193362	0.01570044	+	5725	grey	0.300713968	FALSE	PTBP1
Region_8611	chr19	812488	821955	9467	40	144	210.6969697	31.41580675	0.874577152	0.021389424	-	79948	grey	0.012515279	FALSE	PLPPR3
Region_8612	chr1	896503	913245	16742	24	115	153.303030	15.5335564	0.90525072	0.03546534	-	91300	grey	-0.08564396	FALSE	R3HDM4

	9						3	3	8	3				1		
Region_8613	chr19	917287	921005	3718	28	179	240.8636364	27.50434168	0.007702897	0.006421174	+	84634	grey	-0.192332539	FALSE	KISS1R
Region_8614	chr19	925781	975939	50158	92	418	524.3333333	45.56156725	0.595803507	0.017655912	+	1820	grey	0.086428156	FALSE	ARID3A
Region_8615	chr19	984332	998438	14106	39	186	245.2121212	27.4414146	0.557971455	0.018887097	+	57418	grey	0.121514155	FALSE	WDR18
Region_8616	chr19	1000419	1009732	9313	67	270	347.7272727	37.26394234	0.674822554	0.031719368	+	116444	grey	-0.188932356	FALSE	GRIN3B
Region_8617	chr19	1009648	1021179	11531	54	266	319.2878788	28.57258781	0.710426128	0.019077437	-	91304	grey	0.004699075	FALSE	TMEM259
Region_8618	chr19	1026586	1039068	12482	24	110	140.2121212	13.512974	0.396430278	0.024069525	+	1265	grey	-0.154574883	FALSE	CNN2
Region_8619	chr19	1039997	1065572	25575	89	464	556.8333333	43.88747266	0.701952403	0.014856095	+	10347	grey	-0.004398987	FALSE	ABCA7
Region_8620	chr19	1065923	1086628	20705	93	414	517.0606061	41.63573864	0.675270501	0.02004474	+	23526	grey	0.191742	FALSE	ARHGAP45
Region_8621	chr19	1086574	1095380	8806	33	174	203.8787879	17.92592892	0.49254076	0.022718647	-	5434	grey	-0.091371149	FALSE	POLR2E
Region_8622	chr19	1103994	1106791	2797	30	136	191.1363636	29.16208622	0.187988205	0.016944123	+	2879	grey	0.090039467	FALSE	GPX4
Region_8623	chr19	1163451	1165333	1882	6	18	36.43939394	7.436146206	0.932197457	0.048268626	+	102723798	grey	-0.178664967	FALSE	LOC102723798
Region_8624	chr19	1177558	1228431	50873	129	687	786.6666667	53.65827295	0.591283317	0.01274445	+	6794	grey	-0.009326201	FALSE	STK11

Region_8625	chr19	1228287	1238027	9740	146	437	667.9545455	95.6542542 9	0.790644545	0.012338334	-	255057	grey	0.22996404	FALSE	CBARP
Region_8626	chr19	1241746	1244825	3079	21	104	139	21.43254176	0.138652859	0.020654043	+	513	grey	-0.204204483	FALSE	ATP5F1D
Region_8627	chr19	1245890	1249559	3669	30	97	140.7424242	26.1476933	0.122954649	0.014535746	-	102723811	grey	0.039891254	FALSE	LOC102723811
Region_8628	chr19	1248553	1259143	10590	82	307	443.5454545	52.42230348	0.293246109	0.010163671	+	90007	grey	-0.151327118	FALSE	MIDN
Region_8629	chr19	1259384	1274880	15496	97	459	566.3181818	48.17696835	0.164226587	0.011548631	+	1153	grey	-0.046133488	FALSE	CIRBP
Region_8630	chr19	1261166	1271029	9863	73	335	420.6666667	39.60859788	0.137312879	0.012902813	+	107985303	grey	-0.042871253	FALSE	LOC107985303
Region_8631	chr19	1267471	1270260	2789	38	147	203.4242424	29.27800992	0.162812117	0.016465271	-	148046	grey	-0.159722565	FALSE	CIRBP-AS1
Region_8632	chr19	1275530	1279244	3714	30	152	234.3030303	29.84883438	0.162176188	0.017765586	+	55009	grey	0.053773983	FALSE	FAM174C
Region_8633	chr19	1285873	1301431	15558	61	260	335.2878788	32.35988264	0.406787698	0.019895868	+	1943	grey	0.028903548	FALSE	EFNA2
Region_8634	chr19	1354711	1378431	23720	89	498	574.6212121	45.13663535	0.663168885	0.01695439	+	84939	grey	-0.205874623	FALSE	PWWP3A
Region_8635	chr19	1383527	1395589	12062	55	368	442.6666667	41.19609143	0.639712222	0.008160237	+	374291	grey	0.169959574	FALSE	NDUFS7
Region_8636	chr19	1397026	1401570	4544	15	54	82.03030303	14.6686161	0.331132697	0.022263738	-	2593	grey	-0.108232649	FALSE	GAMT
Region_8637	chr1	1407569	1435687	28118	120	714	815.833333	49.2115006	0.69685208	0.01061948	+	26528	grey	-0.00263323	FALSE	DAZAP1

	9						3	4	2							
Region_8638	chr19	1438358	1440495	2137	30	176	217.7727273	21.92911339	0.251000681	0.011708579	+	6209	grey	0.222871893	FALSE	RPS15
Region_8639	chr19	1446302	1473244	26942	223	1182	1453.69697	108.9402052	0.202807232	0.019522749	+	10297	grey	-0.186813581	FALSE	APC2
Region_8640	chr19	1461143	1479219	18076	160	835	1048.80303	83.89356255	0.188888737	0.023054088	-	148223	grey	-0.168336114	FALSE	C19orf25
Region_8641	chr19	1481428	1490752	9324	47	200	278.2424242	29.14265212	0.897773905	0.016572132	-	54760	grey	0.169067775	FALSE	PCSK4
Region_8642	chr19	1491166	1497927	6761	38	231	318.6515152	40.87485306	0.569310664	0.021074043	+	92840	grey	0.048671369	FALSE	REEP6
Region_8643	chr19	1505022	1513604	8582	34	132	186.030303	24.16225263	0.223539992	0.019406361	-	339366	grey	-0.029002617	FALSE	ADAMTSL5
Region_8644	chr19	1508023	1536046	28023	99	435	547.7878788	50.47299816	0.378514617	0.020509275	+	126520	grey	0.22574284	FALSE	PLK5
Region_8645	chr19	1554672	1568325	13653	68	227	355.0757576	46.3386566	0.685655862	0.015484246	-	399664	grey	-0.084003165	FALSE	MEX3D
Region_8646	chr19	1573596	1605445	31849	102	558	660.969697	53.91102918	0.565059419	0.009761246	-	53615	grey	-0.10266403	FALSE	MBD3
Region_8647	chr19	1597169	1605473	8304	24	112	156.5454545	16.99065971	0.451034023	0.016571362	-	10975	grey	-0.209514199	FALSE	UQCR11
Region_8648	chr19	1609291	1652615	43324	109	497	632.7272727	46.20740715	0.716693235	0.013226931	-	6929	grey	0.089606311	FALSE	TCF3
Region_8649	chr19	1753506	1780988	27482	150	672	833.5757576	76.83610141	0.076539573	0.015714566	+	390874	grey	-0.120696698	FALSE	ONECUT3

Region_8650	chr19	1782075	1812276	30201	47	258	317.8181818	22.743323	0.706343777	0.033525102	-	148229	grey	-0.242390326	FALSE	ATP8B3
Region_8651	chr19	1815248	1848483	33235	92	502	592	39.11246113	0.773979547	0.013026173	-	57455	grey	0.072232874	FALSE	REXO1
Region_8652	chr19	1822089	1824542	2453	10	37	56.72727273	10.27251067	0.949286951	0.034553012	+	100288123	grey	0.278919188	FALSE	LOC100288123
Region_8653	chr19	1852399	1863579	11180	60	199	276.2272727	33.41478045	0.134263092	0.012544352	-	83855	grey	0.384364769	FALSE	KLF16
Region_8654	chr19	1876810	1885496	8686	40	186	273.4545455	34.11978078	0.537951892	0.026791321	-	81926	grey	0.145482864	FALSE	ABHD17A
Region_8655	chr19	1905372	1926013	20641	101	576	698.2575758	50.20643863	0.746122269	0.009005146	+	113178	grey	-0.1314118	FALSE	SCAMP4
Region_8656	chr19	1905399	1913447	8048	66	325	430.969697	40.89911404	0.652315247	0.010460133	+	113179	grey	-0.011250937	FALSE	ADAT3
Region_8657	chr19	1941172	1981338	40166	132	665	792.4090909	57.35958837	0.825126867	0.014088809	+	1455	grey	0.222406654	FALSE	CSNK1G2
Region_8658	chr19	1952531	1954586	2055	8	27	40.77272727	5.723891776	0.959844317	0.038234285	-	255193	grey	-0.159193792	FALSE	CSNK1G2-AS1
Region_8659	chr19	1985438	2015714	30276	65	354	423.2575758	28.25015213	0.863217498	0.01388182	-	55643	grey	-0.025799987	FALSE	BTBD2
Region_8660	chr19	1993101	2034881	41780	64	335	394.0606061	26.87399589	0.835391607	0.018508326	-	107985278	grey	-0.221879963	FALSE	LOC107985278
Region_8661	chr19	2037465	2051244	13779	57	242	331.8333333	41.29242185	0.357440397	0.018932675	-	2872	grey	-0.026561023	FALSE	MKNK2
Region_8662	chr1	2071036	2096673	25637	65	377	448.166666	31.8489624	0.61907504	0.03038016	-	126308	grey	-0.11256394	FALSE	MOB3A

	9						7	7	3	3				4		
Region_8663	chr19	2096429	2099593	3164	18	88	118.8484848	16.07888479	0.201960683	0.022682644	+	113177	grey	-0.072827203	FALSE	IZUMO4
Region_8664	chr19	2100988	2164468	63480	164	1005	1121.69697	55.01271733	0.719330183	0.009843762	-	8943	grey	0.210146799	FALSE	AP3D1
Region_8665	chr19	2163933	2232578	68645	245	1468	1684.545455	98.23569488	0.814391138	0.008060188	+	84444	grey	-0.03200866	FALSE	DOT1L
Region_8666	chr19	2230084	2237704	7620	50	256	331.2424242	29.87670078	0.377759785	0.015906871	-	55111	grey	0.049600036	FALSE	PLEKHJ1
Region_8667	chr19	2236824	2248655	11831	26	136	173.5	18.83062154	0.525340684	0.023934881	+	8175	grey	-0.078510047	FALSE	SF3A2
Region_8668	chr19	2249309	2252073	2764	57	287	398.8333333	53.12151044	0.105959997	0.032902815	+	268	grey	0.17328238	FALSE	AMH
Region_8669	chr19	2252252	2269759	17507	61	328	395.030303	33.40703275	0.296930602	0.014568037	-	126306	grey	0.02963068	FALSE	JSRP1
Region_8670	chr19	2269509	2273490	3981	37	153	204.6818182	25.07046015	0.109680461	0.018817949	+	4946	grey	0.305959581	FALSE	OAZ1
Region_8671	chr19	2269525	2355095	85570	308	1770	2045.924242	122.2132198	0.498825041	0.012971975	+	56928	grey	-0.029347132	FALSE	SPPL2B
Region_8672	chr19	2274631	2282175	7544	29	149	179.2575758	16.77113877	0.905685958	0.021285632	-	374872	grey	0.015333195	FALSE	PEAK3
Region_8673	chr19	2287164	2308154	20990	110	670	833.1363636	67.69385527	0.39966157	0.026322949	-	645191	grey	-0.02982229	FALSE	LINGO3
Region_8674	chr19	2321520	2328611	7091	19	91	130.8787879	16.28355957	0.498063457	0.028609083	-	51690	grey	-0.181839103	FALSE	LSM7

Region_8675	chr19	2360238	2426261	66023	102	581	723.5151515	48.05229281	0.863260773	0.016822341	+	360200	grey	-0.120877015	FALSE	TMPRSS9
Region_8676	chr19	2425625	2427586	1961	14	93	122.1363636	17.64773802	0.030667047	0.016310302	-	26517	grey	-0.120697128	FALSE	TIMM13
Region_8677	chr19	2427638	2456959	29321	89	497	581.3484848	44.96784917	0.610670319	0.014815725	-	84823	grey	-0.116950934	FALSE	LMNB2
Region_8678	chr19	2458935	2462185	3250	7	33	50.60606061	8.406735007	0.326614198	0.045844747	-	101928602	grey	-0.12044063	FALSE	LINC01775
Region_8679	chr19	2476127	2478259	2132	13	82	107.6818182	13.27711634	0.022620037	0.015052055	+	4616	grey	0.144317682	FALSE	GADD45B
Region_8680	chr19	2511219	2702694	191475	322	2013	2268.106061	96.05048813	0.802663014	0.012747704	-	2788	grey	0.060794736	FALSE	GNG7
Region_8681	chr19	2714567	2721372	6805	19	78	119.8636364	14.09952139	0.739228599	0.044363458	-	148252	grey	0.20414377	FALSE	DIRAS1
Region_8682	chr19	2717769	2740048	22279	64	372	429.6666667	29.76644127	0.702250076	0.016188671	-	29985	grey	0.131925414	FALSE	SLC39A3
Region_8683	chr19	2754715	2783362	28647	78	455	532.4545455	39.18209238	0.778008236	0.014863502	-	6449	grey	0.127924978	FALSE	SGTA
Region_8684	chr19	2785503	2815807	30304	89	503	591.030303	38.41432813	0.845985429	0.00986446	+	7064	grey	-0.15735561	FALSE	THOP1
Region_8685	chr19	2819868	2836735	16867	32	155	224.3181818	24.63278559	0.545233883	0.021449267	+	115196	grey	0.299625209	FALSE	ZNF554
Region_8686	chr19	2841437	2860471	19034	26	111	149.9090909	14.85180877	0.3891433	0.021986416	+	148254	grey	0.041477826	FALSE	ZNF555
Region_8687	chr1	2867335	2883445	16110	21	117	152.560606	14.8148873	0.89893587	0.03247804	+	80032	grey	0.007587285	FALSE	ZNF556

	9						1		3	8						
Region_8688	chr19	2900928	2918473	17545	23	113	145.530303	16.53548138	0.783479992	0.029003898	+	126295	grey	0.296547122	FALSE	ZNF57
Region_8689	chr19	2908371	2926847	18476	16	75	106.8181818	14.51249763	0.969960916	0.02103198	-	101928631	grey	-0.164232371	FALSE	
Region_8690	chr19	2933218	2944971	11753	19	87	111.3484848	11.75845676	0.662114645	0.021292872	-	58492	grey	0.125920081	FALSE	ZNF77
Region_8691	chr19	2977538	2995179	17641	26	134	159.8636364	15.21222824	0.759910644	0.026278561	+	79816	grey	0.139549436	FALSE	TLE6
Region_8692	chr19	2997639	3047635	49996	85	438	527.469697	34.71653106	0.737310174	0.013718367	-	7089	grey	0.040078955	FALSE	TLE2
Region_8693	chr19	3052910	3063107	10197	28	90	136.6666667	18.8967505	0.254943733	0.024099766	-	166	grey	-0.293397138	FALSE	TLE5
Region_8694	chr19	3094362	3123999	29637	96	556	671.6666667	45.62736471	0.799998214	0.012861963	+	2767	grey	0.009273865	FALSE	GNA11
Region_8695	chr19	3136033	3163749	27716	32	154	211.2727273	17.34673142	0.637632521	0.024104307	+	2769	grey	0.015668189	FALSE	GNA15
Region_8696	chr19	3141576	3155175	13599	9	30	47.96969697	7.799348488	0.776021973	0.046285938	-	100996351	grey	-0.304493833	FALSE	GNA15-DT
Region_8697	chr19	3178769	3180332	1563	17	115	141.7575758	14.82104596	0.004203854	0.005645722	+	8698	grey	-0.034273657	FALSE	S1PR4
Region_8698	chr19	3185563	3209575	24012	106	608	692.5151515	49.47858664	0.640606516	0.014504823	+	56926	grey	0.077850732	FALSE	NCLN
Region_8699	chr19	3224661	3297076	72415	98	536	628.8333333	36.16142583	0.615276308	0.016305727	+	60680	grey	-0.105514613	FALSE	CELF5

Region_8700	chr19	3296765	3306887	10122	10	59	81.1969697	9.760548057	0.725420377	0.042372503	-	105372244	grey	-0.089420501	FALSE	LOC105372244
Region_8701	chr19	3314403	3469217	154814	256	1500	1690.181818	77.83452054	0.737853365	0.008364545	+	4782	grey	-0.262121913	FALSE	NFIC
Region_8702	chr19	3473986	3480525	6539	7	20	42.21212121	9.247893983	0.574525591	0.09768624	-	284422	grey	-0.38555862	FALSE	SMIM24
Region_8703	chr19	3482110	3483441	1331	13	38	73.42424242	16.44390053	0.956103951	0.034734588	-	122405565	grey	0.114971509	FALSE	SMIM44
Region_8704	chr19	3490821	3500674	9853	23	125	175.6666667	19.36160621	0.671965723	0.013849814	-	83475	grey	-0.403889602	FALSE	DOHH
Region_8705	chr19	3506311	3538334	32023	68	379	453.969697	32.23565618	0.790294072	0.017276561	+	51343	grey	0.271940683	FALSE	FZR1
Region_8706	chr19	3538261	3573851	35590	127	722	845.9393939	54.16381954	0.498080007	0.009881162	-	126321	grey	-0.158707847	FALSE	MFSD12
Region_8707	chr19	3539171	3544030	4859	10	41	73.5	10.98985546	0.883543009	0.038994899	+	100128569	grey	0.116861152	FALSE	TEKTIP1
Region_8708	chr19	3572777	3579088	6311	40	160	210.8939394	24.17381214	0.43516667	0.035300725	+	10362	grey	-0.036608238	FALSE	HMG20B
Region_8709	chr19	3585478	3593541	8063	15	61	94.66666667	13.20761241	0.542102702	0.039082277	+	126326	grey	0.352779634	FALSE	GIPC3
Region_8710	chr19	3594507	3606875	12368	41	183	253.3030303	27.53513543	0.828986609	0.013141172	-	6915	grey	0.130798511	FALSE	TBXA2R
Region_8711	chr19	3607247	3613930	6683	23	98	138.4393939	17.61129776	0.911629787	0.020816548	+	404665	grey	-0.062317571	FALSE	CACTIN-AS1
Region_8712	chr1	3610645	3626815	16170	61	293	376.590909	34.9487436	0.72342705	0.01086544	-	58509	grey	-0.20281455	FALSE	CACTIN

	9						1	9	7	2				3		
Region_8713	chr19	3630183	3700468	70285	176	1053	1225.893939	77.69350607	0.827409504	0.010110465	-	23396	grey	0.099522579	FALSE	PIP5K1C
Region_8714	chr19	3708362	3750813	42451	88	561	625.9848485	35.86362735	0.841674093	0.014076992	+	27134	grey	-0.216195018	FALSE	TJP3
Region_8715	chr19	3750772	3761692	10920	25	108	153.530303	16.32008644	0.770788106	0.022761613	-	9546	grey	-0.149937498	FALSE	APBA3
Region_8716	chr19	3762682	3768575	5893	16	86	116.3484848	13.74883763	0.781145957	0.018624129	+	116541	grey	0.201277663	FALSE	MRPL54
Region_8717	chr19	3769089	3772228	3139	10	35	55.77272727	10.38015185	0.91882556	0.041950876	-	84839	grey	-0.094604415	FALSE	RAX2
Region_8718	chr19	3777970	3802129	24159	53	277	332.530303	24.30778267	0.53044976	0.020054351	-	4145	grey	-0.08220768	FALSE	MATK
Region_8719	chr19	3804024	3869038	65014	117	725	819.2272727	41.9357734	0.779147892	0.012225532	-	23217	grey	-0.076402419	FALSE	ZFR2
Region_8720	chr19	3879864	3928082	48218	70	436	503.2878788	29.9398192	0.87059687	0.011983259	+	85300	grey	0.266582901	FALSE	ATCAY
Region_8721	chr19	3933069	3942416	9347	19	81	107.6969697	11.9601514	0.594976783	0.037361492	+	27231	grey	0.262269432	FALSE	NMRK2
Region_8722	chr19	3958453	3971123	12670	60	287	369	40.45168058	0.762970734	0.014672115	-	1613	grey	-0.16687526	FALSE	DAPK3
Region_8723	chr19	3976056	3985463	9407	46	279	344.1818182	34.55292889	0.628695672	0.017529109	-	1938	grey	-0.070014335	FALSE	EEF2
Region_8724	chr19	4007736	4039386	31650	72	337	403.3181818	26.93876766	0.884716103	0.013733965	+	51588	grey	-0.055005021	FALSE	PIAS4

Region_8725	chr19	4043303	4066899	23596	125	523	654.8939394	62.70741063	0.686961136	0.015123931	-	51341	grey	0.378687299	FALSE	ZBTB7A
Region_8726	chr19	4090321	4124122	33801	72	407	477.3333333	33.1501634	0.825678698	0.016737878	-	5605	grey	0.04685492	FALSE	MAP2K2
Region_8727	chr19	4153631	4173054	19423	18	95	118.9090909	13.64013002	0.918201035	0.022935526	+	84699	grey	-0.418189155	FALSE	CREB3L3
Region_8728	chr19	4174109	4182566	8457	20	93	126.1666667	13.7366252	0.792009781	0.021635622	-	51548	grey	-0.039040451	FALSE	SIRT6
Region_8729	chr19	4182689	4224814	42125	75	426	531.7575758	40.73443473	0.788572388	0.014668618	+	170961	grey	-0.102854878	FALSE	ANKRD24
Region_8730	chr19	4229523	4237528	8005	9	44	66.07575758	10.53099543	0.908931896	0.059720312	+	10148	grey	0.179979934	FALSE	EBI3
Region_8731	chr19	4247080	4269088	22008	40	227	286.2272727	20.27715308	0.887293937	0.020448278	+	55702	grey	-0.136702976	FALSE	YJU2
Region_8732	chr19	4279266	4290722	11456	34	169	218.8636364	21.30574958	0.509925729	0.018769599	+	56961	grey	0.065096388	FALSE	SHD
Region_8733	chr19	4292227	4302431	10204	14	65	84.36363636	9.226444208	0.921245381	0.039739493	-	126259	grey	-0.365004613	FALSE	TMIGD2
Region_8734	chr19	4304598	4323843	19245	39	208	270.7272727	17.87696881	0.808945094	0.016472258	+	79187	grey	-0.155928665	FALSE	FSD1
Region_8735	chr19	4324043	4342786	18743	33	178	230.0909091	19.70531148	0.498357846	0.019233793	-	55620	grey	-0.194878824	FALSE	STAP2
Region_8736	chr19	4343527	4360086	16559	22	117	164.8939394	14.89669947	0.870280039	0.021017019	+	84954	grey	0.024354034	FALSE	MPND
Region_8737	chr1	4360370	4400547	40177	69	423	495.272727	33.5024892	0.70166613	0.01825012	-	6455	grey	0.370937647	FALSE	SH3GL1

	9						3	1		3						
Region_8738	chr19	4402640	4448217	45577	88	508	569.2727273	33.8960154	0.700974432	0.015854585	+	10036	grey	-0.127781686	FALSE	CHAF1A
Region_8739	chr19	4444999	4471279	26280	56	314	370.6363636	22.29842103	0.848714502	0.016977523	-	80700	grey	0.060341858	FALSE	UBXN6
Region_8740	chr19	4472297	4502208	29911	67	347	411.1060606	26.74444804	0.892533562	0.011992777	+	84717	grey	-0.308383058	FALSE	HDGFL2
Region_8741	chr19	4502192	4518486	16294	41	197	254.0151515	21.9898525	0.925370276	0.017998213	-	729359	grey	-0.039051516	FALSE	PLIN4
Region_8742	chr19	4522531	4540067	17536	36	201	245.8484848	18.42510196	0.757639188	0.021683372	-	440503	grey	-0.167921757	FALSE	PLIN5
Region_8743	chr19	4536402	4540036	3634	7	35	50.93939394	6.961164332	0.944524268	0.032206863	-	116844	grey	0.168564286	FALSE	LRG1
Region_8744	chr19	4542593	4581776	39183	83	422	493.0606061	33.2597603	0.674478828	0.022075409	-	10501	grey	0.027286387	FALSE	SEMA6B
Region_8745	chr19	4639516	4655568	16052	32	182	227.1969697	20.84168877	0.766404326	0.020561273	+	126282	grey	0.139974579	FALSE	TNFAIP8L1
Region_8746	chr19	4641374	4670362	28988	53	294	373.3484848	30.63858423	0.739882742	0.016028389	-	56005	grey	0.180017438	FALSE	MYDGF
Region_8747	chr19	4674341	4724673	50332	105	654	726.5	38.12983891	0.778731958	0.011189788	-	91039	grey	0.155850019	FALSE	DPP9
Region_8748	chr19	4679282	4685948	6666	17	67	94.40909091	11.88402583	0.958973386	0.030239014	+	100131094	grey	-0.325465725	FALSE	DPP9-AS1
Region_8749	chr19	4769031	4785077	16046	17	99	120.1363636	11.18499869	0.658914047	0.063856053	+	284424	grey	-0.314679711	FALSE	MIR7-3HG

Region_8750	chr19	4791734	4801273	9539	36	224	273.8333333	26.16255888	0.556570328	0.02105868	+	55527	grey	0.151133524	FALSE	FEM1A
Region_8751	chr19	4815932	4831712	15780	29	166	195.6515152	15.01434435	0.929887256	0.017910637	-	148022	grey	0.110524726	FALSE	TICAM1
Region_8752	chr19	4838341	4867694	29353	46	276	317.3484848	19.18612674	0.91511574	0.014158939	-	10226	grey	0.011717367	FALSE	PLIN3
Region_8753	chr19	4890437	4902896	12459	18	85	119.5151515	13.64860368	0.951609022	0.026355554	-	645432	grey	-0.120746829	FALSE	ARRDC5
Region_8754	chr19	4903080	4962154	59074	132	759	901.4848485	52.14070975	0.661121713	0.011235004	+	29128	grey	0.066497417	FALSE	UHRF1
Region_8755	chr19	4969113	5153598	184485	400	2487	2782.045455	130.0807361	0.878255153	0.005451398	+	23030	grey	0.247298157	FALSE	KDM4B
Region_8756	chr19	5158495	5340812	182317	273	1613	1814.151515	94.62301597	0.768967343	0.01101272	-	5802	grey	-0.062398527	FALSE	PTPRS
Region_8757	chr19	5455417	5456856	1439	18	68	126.1818182	22.09205154	0.939494217	0.030319448	+	148066	grey	-0.251425825	FALSE	ZNRF4
Region_8758	chr19	5558167	5578349	20182	39	190	228.7424242	19.7642339	0.561084615	0.027030224	-	257000	grey	0.223248485	FALSE	TINCR
Region_8759	chr19	5586999	5624046	37047	103	696	804.8787879	66.46815667	0.553592954	0.007225289	-	9667	grey	0.045966675	FALSE	SAFB2
Region_8760	chr19	5623035	5668478	45443	79	490	584.2727273	44.32660096	0.705519841	0.012344739	+	6294	grey	-0.242914797	FALSE	SAFB
Region_8761	chr19	5674947	5691875	16928	66	410	480.2121212	33.39070122	0.32994092	0.011181877	+	25873	grey	0.068489545	FALSE	RPL36
Region_8762	chr1	5678421	5680516	2095	10	67	90.9848484	10.7667471	0.29329274	0.01526002	-	125988	grey	0.080929587	FALSE	MICOS13

	9						8	3	1	7						
Region_8763	chr19	5680604	5690327	9723	38	220	260.0909091	21.71828529	0.41885385	0.016815486	+	374875	grey	0.135871677	FALSE	HSD11B1L
Region_8764	chr19	5691834	5720572	28738	103	685	782.3181818	52.81874932	0.662129747	0.011292961	-	9361	grey	-0.015725846	FALSE	LONP1
Region_8765	chr19	5720637	5778734	58097	71	468	527.5	30.80671755	0.909664584	0.017264372	+	257062	grey	-0.261965604	FALSE	CATSPERD
Region_8766	chr19	5782960	5784746	1786	8	29	42.92424242	7.488063539	0.952314296	0.036554791	-	163154	grey	0.159810559	FALSE	PRR22
Region_8767	chr19	5784005	5791225	7220	33	175	224.7272727	22.32111484	0.732342137	0.015515253	-	56931	grey	0.036695612	FALSE	DUS3L
Region_8768	chr19	5805067	5828324	23257	77	483	578.8787879	52.23571285	0.44919982	0.011392031	+	4902	grey	-0.05530672	FALSE	NRTN
Region_8769	chr19	5830408	5839717	9309	13	71	100.7727273	11.81628011	0.889428977	0.024914393	-	2528	grey	-0.021989535	FALSE	FUT6
Region_8770	chr19	5842888	5851471	8583	8	41	61.89393939	7.730317513	0.959038619	0.035051496	-	2525	grey	0.041054937	FALSE	FUT3
Region_8771	chr19	5847467	5858239	10772	8	44	58.86363636	7.820852528	0.950967848	0.032191677	+	101928844	grey	-0.011938981	FALSE	LOC101928844
Region_8772	chr19	5865826	5903787	37961	68	414	464.9848485	28.4515742	0.742794194	0.015706457	-	2527	grey	0.178512326	FALSE	FUT5
Region_8773	chr19	5867615	5904006	36391	68	409	461.1666667	29.79498323	0.659000978	0.015352929	-	126328	grey	0.16077177	FALSE	NDUFA11
Region_8774	chr19	5904841	5916211	11370	21	121	163.6515152	23.63927138	0.408816019	0.01209663	+	828	grey	0.044751107	FALSE	CAPS

Region_8775	chr19	5904872	5910853	5981	14	63	109.6363636	22.25145552	0.13174021	0.010614947	+	400673	grey	0.022223419	FALSE	VMAC
Region_8776	chr19	5916139	5978142	62003	84	602	678.1666667	33.99890647	0.793958118	0.01174269	-	8498	grey	0.05586557	FALSE	RANBP3
Region_8777	chr19	5978333	6020363	42030	87	572	656.3787879	36.39939355	0.801474853	0.013437578	+	100128568	grey	-0.02115718	FALSE	RANBP3-DT
Region_8778	chr19	5993164	6199529	206365	215	1405	1605.651515	75.74785956	0.77401134	0.008675365	-	5990	grey	-0.00208652	FALSE	RFX2
Region_8779	chr19	6135247	6193094	57847	35	230	276.9090909	19.88639061	0.94196135	0.016147063	+	81616	grey	0.359928146	FALSE	ACSBG2
Region_8780	chr19	6175873	6199572	23699	19	121	152.8484848	12.96770239	0.646080626	0.024684314	-	105372255	grey	0.209709377	FALSE	LOC105372255
Region_8781	chr19	6210381	6279975	69594	128	785	875.7121212	43.11015056	0.827450646	0.016045216	-	4298	grey	-0.085169697	FALSE	MLLT1
Region_8782	chr19	6306142	6362149	56007	55	363	412.530303	29.50317829	0.77943097	0.013137494	-	125981	grey	-0.152554078	FALSE	ACER1
Region_8783	chr19	6361531	6370242	8711	16	88	118.9848485	14.59556563	0.461493801	0.01996704	+	8192	grey	0.058083094	FALSE	CLPP
Region_8784	chr19	6372794	6375250	2456	17	38	70.77272727	12.22204245	0.234326111	0.024548517	+	84266	grey	0.225844791	FALSE	ALKBH7
Region_8785	chr19	6375148	6375933	785	5	12	31.66666667	7.702813339	0.969078556	0.03601576	-	5623	grey	-0.054959609	FALSE	PSPN
Region_8786	chr19	6379572	6393981	14409	41	241	300.1060606	25.46498579	0.592994366	0.012837184	-	2962	grey	-0.223805538	FALSE	GTF2F1
Region_8787	chr1	6392921	6392983	62	3	13	31.4848484	8.46757148	0.00187207	0.00667200	-	10246547	grey	-0.00750371	FALSE	MIR6790

	9						8	8	6	8		3				
Region_8788	chr19	6393794	6412404	18610	26	155	178.2878788	13.23487834	0.919273319	0.023346905	+	390877	grey	-0.089840313	FALSE	LOC390877
Region_8789	chr19	6413102	6424811	11709	37	118	173.3636364	23.63285611	0.644226924	0.023938547	-	8570	grey	-0.041893079	FALSE	KHSRP
Region_8790	chr19	6426037	6433779	7742	11	48	68.51515152	9.848455442	0.959928252	0.030728995	-	284427	grey	0.037415514	FALSE	SLC25A41
Region_8791	chr19	6436079	6465203	29124	40	205	248.469697	18.57394345	0.513753039	0.018521152	-	79085	grey	-0.143472715	FALSE	SLC25A23
Region_8792	chr19	6463777	6467221	3444	14	59	79.98484848	11.93507104	0.417470252	0.029784899	+	92359	grey	0.014517707	FALSE	CRB3
Region_8793	chr19	6467207	6482557	15350	33	137	183.6818182	17.07554174	0.927835293	0.024837156	-	79958	grey	-0.07791628	FALSE	DENND1C
Region_8794	chr19	6494319	6502848	8529	29	127	165.6818182	18.78228223	0.598157964	0.022260364	-	10382	grey	-0.112246718	FALSE	TUBB4A
Region_8795	chr19	6531026	6535924	4898	22	95	143.9393939	26.78798743	0.169641975	0.019802413	+	8744	grey	-0.160060747	FALSE	TNFSF9
Region_8796	chr19	6583183	6604103	20920	34	194	250.5606061	17.0773163	0.593161107	0.01795101	-	970	grey	-0.229610701	FALSE	CD70
Region_8797	chr19	6661253	6670588	9335	9	41	62.12121212	8.801250176	0.529731375	0.03386427	-	8740	grey	-0.105477652	FALSE	TNFSF14
Region_8798	chr19	6677704	6730562	52858	53	307	362.3484848	23.14469032	0.740587244	0.029145119	-	718	grey	-0.114428442	FALSE	C3
Region_8799	chr19	6729914	6737580	7666	13	57	81.96969697	12.37860399	0.736780434	0.026142046	-	56927	grey	0.01436764	FALSE	GPR108

Region_8800	chr19	6737925	6751530	13605	37	176	218.5151515	21.84859962	0.419242626	0.014505702	+	9322	grey	-0.044893376	FALSE	TRIP10
Region_8801	chr19	6752160	6767463	15303	24	125	174.3181818	20.19227506	0.661176894	0.024290224	-	10045	grey	-0.077337083	FALSE	SH2D3A
Region_8802	chr19	6772708	6857366	84658	60	369	443.2575758	30.30831854	0.923646854	0.015451461	+	7409	grey	0.312536795	FALSE	VAV1
Region_8803	chr19	6887566	6940459	52893	17	96	124.6060606	15.31904675	0.892282545	0.030108065	+	2015	grey	-0.10125576	FALSE	ADGRE1
Region_8804	chr19	6952500	6997872	45372	25	162	195.1818182	25.06171404	0.894974726	0.034690353	-	326342	grey	-0.318738566	FALSE	ADGRE4P
Region_8805	chr19	7056207	7069665	13458	3	11	19.01515152	4.126807014	0.641761353	0.059223705	-	653657	grey	0.02819907	FALSE	MBD3L3
Region_8806	chr19	7069703	7087968	18265	19	118	155.8030303	19.35038835	0.620167954	0.013199462	+	79230	grey	-0.059030251	FALSE	ZNF557
Region_8807	chr19	7112255	7294414	182159	197	1203	1312.909091	60.57080466	0.749343446	0.007619256	-	3643	grey	0.107216241	FALSE	INSR
Region_8808	chr19	7348937	7472485	123548	65	389	456.2272727	31.18816119	0.953077633	0.0123499	+	23370	grey	0.066401214	FALSE	ARHGEF18
Region_8809	chr19	7476875	7497449	20574	30	186	222.1212121	14.49829184	0.724616454	0.025441075	-	92960	grey	0.084244265	FALSE	PEX11G
Region_8810	chr19	7492976	7508450	15474	16	75	99.33333333	11.22131671	0.595490595	0.040133157	+	374877	grey	-0.05665338	FALSE	SAXO5
Region_8811	chr19	7507052	7521025	13973	18	99	131.9545455	9.680164999	0.758934467	0.034290839	+	140467	grey	-0.136501959	FALSE	ZNF358
Region_8812	chr1	7522624	7534009	11385	19	107	134.106060	14.3023707	0.77281587	0.02861239	+	57192	grey	0.097788519	FALSE	MCOLN1

	9						6	4	5	2						
Region_8813	chr19	7533595	7561764	28169	42	224	282.030303	24.35188806	0.7055426	0.013068825	+	10908	grey	-0.041291682	FALSE	PNPLA6
Region_8814	chr19	7595863	7618304	22441	34	172	208.4393939	16.41681014	0.875660889	0.029692414	+	57662	grey	-0.178858774	FALSE	CAMSAP3
Region_8815	chr19	7619525	7629545	10020	29	144	185.7727273	17.90126378	0.932974844	0.017423523	-	56949	grey	-0.129904376	FALSE	XAB2
Region_8816	chr19	7629788	7639807	10019	13	65	87.74242424	9.412447742	0.532192477	0.037889266	+	100131801	grey	-0.028823185	FALSE	PET100
Region_8817	chr19	7629796	7647873	18077	21	113	147.8181818	15.05059068	0.697933301	0.026646938	+	6813	grey	-0.06689464	FALSE	STXBP2
Region_8818	chr19	7631611	7636990	5379	8	42	55.92424242	7.577924632	0.749384356	0.057896912	-	126006	grey	-0.056903599	FALSE	PCP2
Region_8819	chr19	7678501	7687703	9202	24	139	178.5909091	18.10905194	0.801473105	0.029640312	+	126003	grey	-0.045859531	FALSE	TRAPPC5
Region_8820	chr19	7688758	7702146	13388	16	56	136.969697	27.87608277	0.78602043	0.040318606	-	2208	grey	-0.148896993	FALSE	FCER2
Region_8821	chr19	7728957	7745662	16705	13	46	69.46969697	9.178938596	0.684316264	0.049514931	-	339390	grey	-0.171796567	FALSE	CLEC4G
Region_8822	chr19	7728958	7747564	18606	18	80	104.5606061	11.56803126	0.748669951	0.037403063	-	30835	grey	-0.230004679	FALSE	CD209
Region_8823	chr19	7830218	7864976	34758	48	246	293.1666667	20.06955853	0.743445512	0.028658316	+	115704	grey	0.259368785	FALSE	EVI5L
Region_8824	chr19	7868719	7874390	5671	6	18	30.77272727	6.088565461	0.118133263	0.057431956	-	80164	grey	0.060196526	FALSE	PRR36

Region_8825	chr19	7888505	7902021	13516	7	36	53.54545455	8.088323622	0.915979625	0.036809052	+	80131	grey	0.050913485	FALSE	LRRC8E
Region_8826	chr19	7903843	7914478	10635	16	67	87.21212121	11.22019491	0.724336526	0.035880924	+	5609	grey	-0.325242605	FALSE	MAP2K7
Region_8827	chr19	7914830	7919097	4267	11	52	68.37878788	7.928071162	0.296922735	0.05435955	+	100507588	grey	-0.030435908	FALSE	TGFBR3L
Region_8828	chr19	7920338	7923250	2912	4	10	18.03030303	4.723003272	0.23575487	0.047264243	+	6618	grey	0.332311667	FALSE	SNAPC2
Region_8829	chr19	7926718	7943667	16949	31	175	213	17.23234883	0.949201249	0.019525	-	10469	grey	-0.092455474	FALSE	TIMM44
Region_8830	chr19	7958573	8005659	47086	84	582	634.6060606	33.33276456	0.926638152	0.011263657	-	1994	grey	-0.025402939	FALSE	ELAVL1
Region_8831	chr19	8052318	8062660	10342	7	37	54.22727273	7.873608213	0.812305916	0.092395932	+	6370	grey	-0.265143476	FALSE	CCL25
Region_8832	chr19	8065402	8149592	84190	56	367	421.0909091	24.6462525	0.891413291	0.018907197	-	84467	grey	0.140625156	FALSE	FBN3
Region_8833	chr19	8206736	8262421	55685	45	272	316.1969697	21.3384591	0.77549158	0.018206037	+	79603	grey	-0.336309913	FALSE	CERS4
Region_8834	chr19	8302127	8308358	6231	4	23	32.56060606	5.288969037	0.283662024	0.043207369	-	51293	grey	-0.239667486	FALSE	CD320
Region_8835	chr19	8308283	8321379	13096	16	101	122.8333333	10.28304554	0.786488613	0.024630765	-	4701	grey	0.3334059	FALSE	NDUFA7
Region_8836	chr19	8321158	8323340	2182	3	12	22.01515152	5.070252381	0.285171156	0.054507674	+	6234	grey	0.154155567	FALSE	RPS28
Region_8837	chr1	8322584	8343262	20678	23	138	168.151515	14.7525668	0.58875660	0.02485561	-	256949	grey	0.273218258	FALSE	KANK3

	9						2	4	9	6						
Region_8838	chr19	8363299	8374370	11071	13	66	94.86363636	11.2999474	0.88533108	0.035207518	+	51129	grey	0.229955139	FALSE	ANGPTL4
Region_8839	chr19	8374372	8390685	16313	11	74	100.2727273	9.332400886	0.726438013	0.022949455	-	100507567	grey	0.085427837	FALSE	RAB11B-AS1
Region_8840	chr19	8389981	8404434	14453	35	179	249.0454545	28.49422712	0.895178219	0.015673523	+	9230	grey	0.347230828	FALSE	RAB11B
Region_8841	chr19	8413270	8439017	25747	27	151	181.2575758	16.43852006	0.860357916	0.016868812	+	51257	grey	0.217146254	FALSE	MARCHF2
Region_8842	chr19	8444767	8489114	44347	34	230	268.6969697	19.79197172	0.721274619	0.018276567	+	4670	grey	-0.004547655	FALSE	HNRNPM
Region_8843	chr19	8490056	8502640	12584	14	67	93.04545455	11.10846083	0.648536747	0.05797918	-	84106	grey	-0.077822805	FALSE	PRAM1
Region_8844	chr19	8509678	8514167	4489	10	38	62.15151515	8.658019714	0.875179324	0.02559516	-	84330	grey	-0.141141334	FALSE	ZNF414
Region_8845	chr19	8520778	8577577	56799	57	380	441.5454545	22.50550826	0.730935374	0.014275716	-	4542	grey	0.153643326	FALSE	MYO1F
Region_8846	chr19	8580240	8610735	30495	41	219	270.7575758	20.25078202	0.677827997	0.019816009	-	81794	grey	-0.072585439	FALSE	ADAMTS10
Region_8847	chr19	8630633	8681151	50518	31	198	236.1515152	16.35965245	0.653101916	0.028045138	+	105372267	grey	-0.174444627	FALSE	NFILZ
Region_8848	chr19	8806170	8832314	26144	11	72	87.33333333	9.210918827	0.767047698	0.042888325	-	148156	grey	0.173403	FALSE	ZNF558
Region_8849	chr19	8832377	8843340	10963	6	33	49.40909091	7.755845979	0.628387443	0.050220149	+	85509	grey	-0.194579692	FALSE	MBD3L1

Region_8850	chr19	8848844	8981342	132498	27	201	252.469697	15.82813434	0.740624213	0.035320473	-	94025	grey	-0.347038057	FALSE	MUC16
Region_8851	chr19	9087061	9095669	8608	3	16	23.90909091	4.033426765	0.894280912	0.066450121	+	125963	grey	-0.198125672	FALSE	OR1M1
Region_8852	chr19	9140397	9163424	23027	15	103	132.3787879	12.83236911	0.723756202	0.011839003	+	57693	grey	0.080930073	FALSE	ZNF317
Region_8853	chr19	9178979	9188818	9839	6	36	51.53030303	9.165520166	0.931939698	0.034145967	+	162998	grey	0.112657898	FALSE	OR7D2
Region_8854	chr19	9291140	9309838	18698	10	63	79.21212121	8.544031028	0.950481033	0.024722165	-	374879	grey	-0.064623751	FALSE	ZNF699
Region_8855	chr19	9323772	9371752	47980	9	52	72.8030303	7.971525666	0.704504	0.05089888	+	84527	grey	0.371529732	FALSE	ZNF559
Region_8856	chr19	9324174	9382617	58443	10	54	80.53030303	9.824959889	0.821474247	0.048887443	+	100529215	grey	0.337590864	FALSE	ZNF559-ZNF177
Region_8857	chr19	9363013	9382617	19604	5	22	35.83333333	6.763211193	0.763293966	0.039331927	+	7730	grey	0.059736854	FALSE	ZNF177
Region_8858	chr19	9383581	9407080	23499	4	20	31.25757576	5.410276848	0.944581874	0.048259917	-	112268250	grey	-0.077048536	FALSE	LOC112268250
Region_8859	chr19	9412429	9435573	23144	8	34	52.03030303	8.573608336	0.733201789	0.023699225	-	10781	grey	-0.102319966	FALSE	ZNF266
Region_8860	chr19	9435590	9462529	26939	7	34	52.98484848	7.380832607	0.851885865	0.057999592	+	105372269	grey	0.168836897	FALSE	LOC105372269
Region_8861	chr19	9466355	9498616	32261	5	35	45.28787879	6.39173782	0.724155693	0.047411204	-	147741	grey	-0.156705585	FALSE	ZNF560
Region_8862	chr1	9560329	9584504	24175	7	40	56.9848484	7.78063162	0.83397736	0.02253710	-	7675	grey	-0.03537098	FALSE	ZNF121

	9						8	2	8	7				1		
Region_8863	chr19	9604680	9621236	16556	3	11	26.10606061	5.544585128	0.929242641	0.0980906	-	93134	grey	-0.419120364	FALSE	ZNF561
Region_8864	chr19	9621249	9674662	53413	10	59	78.54545455	9.091300691	0.841158126	0.02866318	+	284385	grey	0.160942614	FALSE	ZNF561-AS1
Region_8865	chr19	9641807	9675100	33293	4	16	30.86363636	5.596389945	0.969326791	0.031699385	-	54811	grey	0.212610119	FALSE	ZNF562
Region_8866	chr19	9689924	9700776	10852	4	21	33.10606061	6.280932777	0.914832609	0.059823058	-	729648	grey	-0.114978915	FALSE	ZNF812P
Region_8867	chr19	9751993	9793097	41104	17	79	116.8030303	12.06931226	0.628204719	0.028881056	-	162993	grey	-0.410469794	FALSE	ZNF846
Region_8868	chr19	9787408	9793180	5772	7	24	39.48484848	7.884244121	0.748160396	0.055894105	-	100505555	grey	-0.223714488	FALSE	LOC100505555
Region_8869	chr19	9810267	9827816	17549	10	58	74.43939394	9.101358796	0.472838821	0.03392825	-	54850	grey	0.226041858	FALSE	FBXL12
Region_8870	chr19	9835257	9849689	14432	12	80	97.95454545	8.32684596	0.963945364	0.022413369	+	5300	grey	-0.00167476	FALSE	PIN1
Region_8871	chr19	9853718	9936515	82797	61	370	454.7878788	25.46038561	0.747418827	0.015260546	-	93145	grey	-0.235174871	FALSE	OLFM2
Region_8872	chr19	9959561	10010504	50943	26	138	176.9545455	14.91765275	0.682884238	0.032164397	-	50509	grey	0.079848077	FALSE	COL5A3
Region_8873	chr19	10013249	10022279	9030	6	33	50.71212121	6.39414432	0.853115021	0.059313542	+	50700	grey	0.295109385	FALSE	RDH8
Region_8874	chr19	10037803	10074770	36967	24	158	181.030303	12.14143534	0.773760354	0.037526492	+	388503	grey	-0.106247247	FALSE	C3P1

Region_8875	chr19	10092338	10102678	10340	4	21	32.34848485	4.744202695	0.72625374	0.057400615	-	83854	grey	-0.146005322	FALSE	ANGPTL6
Region_8876	chr19	10106223	10114780	8557	10	41	77.98484848	12.50168287	0.834217941	0.033272574	+	692312	grey	-0.230016071	FALSE	PPAN-P2RY11
Region_8877	chr19	10106362	10112012	5650	7	22	50.8030303	9.428630208	0.933811418	0.040952806	+	56342	grey	-0.164103512	FALSE	PPAN
Region_8878	chr19	10111693	10115372	3679	4	16	33.18181818	7.358739631	0.89739536	0.064341791	+	5032	grey	-0.100435205	FALSE	P2RY11
Region_8879	chr19	10115014	10119918	4904	11	39	65.66666667	11.28693368	0.794796436	0.047183163	-	8666	grey	-0.104633044	FALSE	EIF3G
Region_8880	chr19	10133342	10231286	97944	106	709	804.2424242	39.88370039	0.737301117	0.011976906	-	1786	grey	0.145033727	FALSE	DNMT1
Region_8881	chr19	10221433	10231331	9898	31	146	188.7878788	18.06783385	0.416311478	0.018523983	-	9294	grey	0.176608588	FALSE	S1PR2
Region_8882	chr19	10251901	10260055	8154	7	45	57.3030303	6.265828442	0.433716129	0.038153792	+	51073	grey	-0.003229288	FALSE	MRPL4
Region_8883	chr19	10252268	10285108	32840	43	294	328.7878788	19.35777733	0.58280614	0.018019708	-	105372272	grey	-0.211386112	FALSE	LIMASI
Region_8884	chr19	10271093	10286615	15522	17	95	132.4393939	13.22250853	0.84342855	0.030535553	+	3383	grey	-0.305160327	FALSE	ICAM1
Region_8885	chr19	10289952	10296778	6826	11	61	76.81818182	10.46592874	0.160246963	0.051820511	+	7087	grey	-0.408688708	FALSE	ICAM5
Region_8886	chr19	10304803	10309880	5077	7	28	44.25757576	5.701040677	0.538082642	0.071430126	-	100125288	grey	0.16964373	FALSE	ZGLP1
Region_8887	chr1	10305427	10316015	10588	9	40	55.2272727	6.76369369	0.57971575	0.04924021	-	112812	grey	0.371931639	FALSE	FDX2

	9						3	9	7	3						
Region_8888	chr19	10315471	10333638	18167	19	90	118.2272727	12.3010887	0.632623506	0.029027053	-	125950	grey	-0.241493835	FALSE	RAVER1
Region_8889	chr19	10333776	10339661	5885	17	75	106.6363636	10.55481861	0.735557152	0.038663252	-	3385	grey	-0.010306648	FALSE	ICAM3
Region_8890	chr19	10350533	10380608	30075	43	263	307.0757576	21.637795	0.860267636	0.014461447	-	7297	grey	-0.137305424	FALSE	TYK2
Region_8891	chr19	10391133	10420121	28988	58	302	367.4545455	27.91811202	0.467384483	0.01656722	-	11140	grey	-0.055656027	FALSE	CDC37
Region_8892	chr19	10416773	10469630	52857	58	353	401.2272727	22.63135704	0.576318263	0.015306819	+	5141	grey	-0.216177681	FALSE	PDE4A
Region_8893	chr19	10486125	10503558	17433	39	199	248.1060606	22.29531085	0.838972293	0.01565682	-	9817	grey	0.075551018	FALSE	KEAP1
Region_8894	chr19	10512742	10517931	5189	39	267	352.6060606	38.08245257	0.132492108	0.026087782	-	53637	grey	0.119958724	FALSE	S1PR5
Region_8895	chr19	10543895	10553418	9523	12	65	85.86363636	10.11151113	0.671417038	0.041260951	+	84971	grey	0.039325787	FALSE	ATG4D
Region_8896	chr19	10553085	10566031	12946	13	60	88.34848485	11.11823509	0.763768053	0.029882928	-	65095	grey	0.237660004	FALSE	KRI1
Region_8897	chr19	10572671	10587315	14644	15	55	91.25757576	10.34974635	0.838766941	0.024030618	-	10053	grey	0.332838863	FALSE	AP1M2
Region_8898	chr19	10602457	10644557	42100	50	289	348.9848485	22.33417004	0.79825919	0.017253683	+	57153	grey	0.16390001	FALSE	SLC44A2
Region_8899	chr19	10654304	10692400	38096	65	394	460.0606061	28.55756873	0.663528143	0.013854409	+	3609	grey	-0.089199556	FALSE	ILF3

Region_8900	chr19	10701430	10713437	12007	11	51	76.8030303	10.84324632	0.892591893	0.032327074	+	81890	grey	0.204520656	FALSE	QTRT1
Region_8901	chr19	10718055	10833488	115433	145	954	1088.621212	49.24937021	0.802884345	0.010268015	+	1785	grey	0.13796934	FALSE	DNM2
Region_8902	chr19	10832067	10836318	4251	7	37	54.81818182	7.456353184	0.812886821	0.032038831	-	11018	grey	-0.186362912	FALSE	TMED1
Region_8903	chr19	10836575	10869790	33215	26	165	195.3636364	13.72211731	0.9195836	0.019788672	+	255809	grey	0.123366301	FALSE	C19orf38
Region_8904	chr19	10871553	10923075	51522	48	298	341.7575758	16.9015799	0.86228356	0.012292243	+	10498	grey	-0.259187941	FALSE	CARM1
Region_8905	chr19	10922185	10928681	6496	11	55	70.28787879	9.015823955	0.44003114	0.016764303	-	78992	grey	-0.098519375	FALSE	YIPF2
Region_8906	chr19	10928811	10930254	1443	6	28	39.60606061	5.766958371	0.007520734	0.014121778	+	90580	grey	0.003596607	FALSE	TIMM29
Region_8907	chr19	10960932	11079426	118494	146	1041	1141.924242	41.9598015	0.889633577	0.011655974	+	6597	grey	0.144394927	FALSE	SMARCA4
Region_8908	chr19	11089462	11133820	44358	42	291	329.6666667	20.490398	0.816339555	0.013500292	+	3949	grey	-0.014574449	FALSE	LDLR
Region_8909	chr19	11131520	11155807	24287	33	197	240.2575758	18.85190103	0.806271774	0.018914855	-	147841	grey	-0.126609419	FALSE	SPC24
Region_8910	chr19	11164270	11197791	33521	40	256	301.5757576	19.0295807	0.832031363	0.022808534	-	25959	grey	-0.023245474	FALSE	KANK2
Region_8911	chr19	11199295	11262524	63229	60	342	391.4393939	20.60256055	0.841666072	0.014082463	-	57572	grey	0.048073557	FALSE	DOCK6
Region_8912	chr1	11203628	11216168	12540	8	30	47.8636363	6.65613970	0.71915192	0.03609710	+	10537227	grey	-0.21398923	FALSE	DOCK6-AS1

	9						6	7	7	3		3		7		
Region_8913	chr19	11237450	11241943	4493	5	25	41	6.246229632	0.852393873	0.056963115	+	55908	grey	0.070842149	FALSE	ANGPTL8
Region_8914	chr19	11296139	11326996	30857	25	166	194.3181818	13.67778557	0.836088924	0.025183141	+	26526	grey	-0.115738729	FALSE	TSPAN16
Region_8915	chr19	11300583	11324441	23858	22	144	173.4393939	13.07390682	0.829205038	0.026430024	-	105372274	grey	0.008279847	FALSE	HSPC102
Region_8916	chr19	11322068	11346270	24202	15	92	113.1818182	10.40696378	0.680636105	0.026045516	-	9545	grey	0.003816072	FALSE	RAB3D
Region_8917	chr19	11342776	11346518	3742	5	16	33.36363636	6.93405501	0.399366652	0.037955205	-	374882	grey	0.124134317	FALSE	TMEM205
Region_8918	chr19	11345301	11354944	9643	8	47	63.04545455	8.96906104	0.548839233	0.029276685	+	126075	grey	-0.110805168	FALSE	CCDC159
Region_8919	chr19	11355386	11365698	10312	10	38	54.98484848	7.991326884	0.894301791	0.052924753	+	64748	grey	0.168994109	FALSE	PLPPR2
Region_8920	chr19	11377207	11384342	7135	16	85	113.5757576	12.07420723	0.168176221	0.028256964	-	2057	grey	-0.10831867	FALSE	EPOR
Region_8921	chr19	11384341	11419328	34987	34	229	258.8030303	15.78823291	0.836282167	0.020687222	-	57139	grey	0.295282441	FALSE	RGL3
Region_8922	chr19	11420604	11435782	15178	21	91	123.9545455	13.41919614	0.560981944	0.026224523	-	115948	grey	0.022205676	FALSE	ODAD3
Region_8923	chr19	11435284	11450968	15684	12	78	98.90909091	9.455527114	0.952247081	0.026910901	+	5589	grey	-0.123992847	FALSE	PRKCSH
Region_8924	chr19	11451326	11481046	29720	35	176	218.3636364	19.93345573	0.797071246	0.023953093	-	1995	grey	-0.042022489	FALSE	ELAVL3

Region_8925	chr19	11466240	11505839	39599	47	306	354.4545455	24.03598701	0.747926304	0.015308109	-	115950	grey	0.06518699	FALSE	ZNF653
Region_8926	chr19	11505929	11529172	23243	18	126	155.8939394	12.49015463	0.635921173	0.019958647	-	51295	grey	-0.036868277	FALSE	ECSIT
Region_8927	chr19	11538767	11550323	11556	9	44	63.39393939	7.491284758	0.912063785	0.04488487	+	1264	grey	0.319237436	FALSE	CNN1
Region_8928	chr19	11551147	11559230	8083	6	27	41.51515152	6.259128532	0.574183326	0.04299387	-	84337	grey	-0.104970494	FALSE	ELOF1
Region_8929	chr19	11559374	11619161	59787	55	373	430.1818182	26.70978449	0.606103364	0.022579676	+	199692	grey	-0.191246184	FALSE	ZNF627
Region_8930	chr19	11639776	11686569	46793	33	195	231.2878788	15.48474699	0.747656372	0.018273844	+	401898	grey	-0.137565348	FALSE	ZNF833P
Region_8931	chr19	11721265	11739009	17744	21	118	159.1666667	17.84426935	0.466685197	0.012589098	-	55552	grey	-0.08862579	FALSE	ZNF823
Region_8932	chr19	11767000	11784078	17078	6	29	44.81818182	6.894611253	0.654394549	0.033041839	+	126068	grey	0.295306112	FALSE	ZNF441
Region_8933	chr19	11814273	11835216	20943	12	63	88.1969697	9.714730602	0.454324893	0.026150328	+	126070	grey	0.245703806	FALSE	ZNF440
Region_8934	chr19	11848726	11883750	35024	13	79	106.2272727	12.03558244	0.448868939	0.015704495	+	90594	grey	-0.087658261	FALSE	ZNF439
Region_8935	chr19	11887782	11914329	26547	9	60	74.86363636	8.015151735	0.95274775	0.034095069	+	7620	grey	0.092807183	FALSE	ZNF69
Region_8936	chr19	11925068	11950773	25705	18	98	146.9393939	14.64647901	0.371681482	0.019142021	+	90592	grey	0.001461076	FALSE	ZNF700
Region_8937	chr1	11925075	11980617	55542	27	186	225.984848	16.7410067	0.52128668	0.01611282	+	284390	grey	-0.02623073	FALSE	ZNF763

	9						5			4						
Region_8938	chr19	11987617	12046275	58658	30	210	239.469697	14.34971959	0.644036183	0.028625192	+	101928464	grey	-0.22621606	FALSE	ZNF433-AS1
Region_8939	chr19	11987641	12081565	93924	48	309	358.7727273	21.16077318	0.681396739	0.022449531	+	284391	grey	-0.116338062	FALSE	ZNF844
Region_8940	chr19	12014715	12035741	21026	10	64	82.46969697	9.109960224	0.726117185	0.045035143	-	163059	grey	-0.198915662	FALSE	ZNF433
Region_8941	chr19	12017888	12052967	35079	25	155	192.5	15.06115738	0.564018005	0.034613753	-	729747	grey	-0.209235516	FALSE	ZNF878
Region_8942	chr19	12075327	12137235	61908	22	134	158.8181818	13.75042275	0.911557853	0.020090983	+	388507	grey	0.031016958	FALSE	ZNF788P
Region_8943	chr19	12092843	12140355	47512	15	88	108.1818182	11.25359305	0.855293925	0.023158829	-	7568	grey	0.036609891	FALSE	ZNF20
Region_8944	chr19	12132117	12156731	24614	17	125	154.0909091	14.06548123	0.58716841	0.01956965	-	100529855	grey	-0.31878599	FALSE	ZNF625-ZNF20
Region_8945	chr19	12140217	12156734	16517	15	99	135.1969697	13.37646578	0.535938365	0.021050917	-	90589	grey	-0.332401366	FALSE	ZNF625
Region_8946	chr19	12163064	12189871	26807	14	69	93.18181818	11.41242116	0.622111312	0.01890854	+	7695	grey	-0.010723705	FALSE	ZNF136
Region_8947	chr19	12195015	12237767	42752	20	109	146.8181818	15.058766	0.876809862	0.021615899	+	100289333	grey	0.182472362	FALSE	LOC100289333
Region_8948	chr19	12224686	12294883	70197	27	175	210.4090909	15.05066037	0.917399568	0.020647308	-	51710	grey	-0.153271324	FALSE	ZNF44
Region_8949	chr19	12345944	12365905	19961	9	39	55.63636364	7.677412151	0.637266522	0.025624419	-	79973	grey	0.101235172	FALSE	ZNF442

Region_8950	chr19	12379189	12441082	61893	10	48	77.03030303	10.1677767	0.955889248	0.033244617	-	90576	grey	-0.058487009	FALSE	ZNF799
Region_8951	chr19	12433103	12551474	118371	51	310	360.1060606	23.97314445	0.770900621	0.017776947	-	163051	grey	-0.677001158	FALSE	ZNF709
Region_8952	chr19	12525373	12580975	55602	29	183	216.6060606	18.34446367	0.572460273	0.010917898	-	163050	grey	-0.024201669	FALSE	ZNF564
Region_8953	chr19	12576100	12610813	34713	15	89	115.469697	11.39800212	0.870810495	0.021915171	-	57474	grey	0.216439846	FALSE	ZNF490
Region_8954	chr19	12583485	12640098	56613	29	171	207.969697	13.57936183	0.78332211	0.018929697	-	105372279	grey	0.076403597	FALSE	LOC105372279
Region_8955	chr19	12610918	12633840	22922	18	101	127.9393939	12.18558895	0.761440988	0.021903964	+	163049	grey	0.013668606	FALSE	ZNF791
Region_8956	chr19	12643831	12669397	25566	25	142	175.1363636	13.90615399	0.701195397	0.020852432	-	4125	grey	-0.138520412	FALSE	MAN2B1
Region_8957	chr19	12666802	12675832	9030	13	77	96.95454545	11.11261489	0.535925007	0.02492992	+	84292	grey	-0.332859943	FALSE	WDR83
Region_8958	chr19	12668071	12681834	13763	16	94	125.6969697	12.68914702	0.60864177	0.024664218	-	51398	grey	-0.161352101	FALSE	WDR83OS
Region_8959	chr19	12675717	12681880	6163	5	23	38.63636364	6.379381122	0.622556682	0.051319485	-	1725	grey	0.038423183	FALSE	DHPS
Region_8960	chr19	12688053	12696631	8578	5	18	34.87878788	7.10907362	0.69428149	0.088667931	-	84261	grey	-0.013841186	FALSE	FBXW9
Region_8961	chr19	12699201	12724011	24810	22	126	153.0606061	13.7111732	0.563704894	0.033643716	-	30000	grey	-0.172108718	FALSE	TNPO2
Region_8962	chr1	12730640	12734684	4044	4	19	36.0454545	7.18637989	0.32753451	0.06980127	-	79002	grey	0.087318371	FALSE	TRIR

	9						5	1	2	6						
Region_8963	chr19	12737139	12748323	11184	10	37	57.51515152	7.823519618	0.679134989	0.024866187	+	439	grey	0.349986816	FALSE	GET3
Region_8964	chr19	12751702	12758458	6756	12	67	89.65151515	10.66192712	0.655818841	0.050921026	+	54831	grey	0.119215751	FALSE	BEST2
Region_8965	chr19	12763003	12792582	29579	48	276	317.9848485	22.86380574	0.330984055	0.019664252	-	29911	grey	-0.210596929	FALSE	HOOK2
Region_8966	chr19	12791486	12793315	1829	19	120	161.5606061	20.28575005	0.003654375	0.005758583	+	3726	grey	0.043859189	FALSE	JUNB
Region_8967	chr19	12796820	12801800	4980	5	24	38.96969697	6.297666716	0.223170527	0.036622058	-	7001	grey	0.221606174	FALSE	PRDX2
Region_8968	chr19	12802031	12806790	4759	6	28	41.18181818	5.930129304	0.590770925	0.048315796	+	111644133	grey	0.116109823	FALSE	THSD8
Region_8969	chr19	12802063	12813640	11577	16	105	125.3484848	10.75101773	0.762018415	0.024358373	+	10535	grey	0.029098681	FALSE	RNASEH2A
Region_8970	chr19	12825478	12835428	9950	18	92	125.0454545	14.8483947	0.236616749	0.031344421	-	83546	grey	0.076283658	FALSE	RTBDN
Region_8971	chr19	12825711	12832983	7272	13	72	98.98484848	12.07475743	0.246817722	0.030298227	+	105372281	grey	0.190713006	FALSE	LOC105372281
Region_8972	chr19	12833951	12874952	41001	54	322	387.5151515	24.57969295	0.671405781	0.018322374	+	22983	grey	0.168105867	FALSE	MAST1
Region_8973	chr19	12875209	12881595	6386	13	89	110.6818182	8.507298389	0.880972489	0.021380715	-	1777	grey	0.068677336	FALSE	DNASE2
Region_8974	chr19	12884422	12887201	2779	10	40	66.40909091	9.987649716	0.171948695	0.027990147	-	10661	grey	-0.099742933	FALSE	KLF1

Region_8975	chr19	12891160	12914207	23047	40	253	288.3636364	17.66930094	0.722759993	0.017341685	+	2639	grey	-0.273747308	FALSE	GCDH
Region_8976	chr19	12898786	12919293	20507	38	241	272.7878788	17.23459415	0.774055266	0.022001346	-	256126	grey	-0.216272855	FALSE	SYCE2
Region_8977	chr19	12922479	12934037	11558	12	89	110.9090909	10.25029421	0.623337277	0.020962257	-	2193	grey	0.019547232	FALSE	FARSA
Region_8978	chr19	12938578	12944489	5911	11	71	92.83333333	10.40204615	0.078778807	0.024806247	+	811	grey	0.007508591	FALSE	CALR
Region_8979	chr19	12945855	12953642	7787	17	113	138.6060606	12.12356737	0.530714952	0.034012119	+	5886	grey	0.054255511	FALSE	RAD23A
Region_8980	chr19	12953119	12957223	4104	3	11	20.28787879	5.357281605	0.458319048	0.10554731	-	90480	grey	0.045257979	FALSE	GADD45GIP1
Region_8981	chr19	12965159	12974760	9601	10	60	80.18181818	8.430547187	0.783796647	0.031309739	+	199699	grey	0.066922204	FALSE	DAND5
Region_8982	chr19	12995475	13098796	103321	131	777	903.7272727	50.81930144	0.594584884	0.01438768	+	4784	grey	0.025918284	FALSE	NFIX
Region_8983	chr19	13104902	13117567	12665	28	169	214.2272727	17.36999664	0.759550002	0.019271686	-	55621	grey	0.029899	FALSE	TRMT1
Region_8984	chr19	13116862	13141147	24285	46	307	364.1666667	26.89957582	0.777646479	0.020573602	+	112939	grey	-0.014727478	FALSE	NACC1
Region_8985	chr19	13144058	13150383	6325	3	11	19.96969697	4.670594418	0.598170769	0.055831007	-	8677	grey	0.229780833	FALSE	STX10
Region_8986	chr19	13150411	13154911	4500	8	33	57.8030303	7.82150821	0.005553138	0.012660297	+	9592	grey	0.196606422	FALSE	IER2
Region_8987	chr1	13206442	13633025	426583	219	1537	1646.27272	55.6473913	0.83961690	0.01492946	-	773	grey	0.069731546	FALSE	CACNA1A

	9						7	8	8	4						
Region_8988	chr19	13518797	13624489	105692	30	197	233.7575758	13.68667022	0.802164179	0.024328122	-	107985287	grey	0.027255141	FALSE	LOC107985287
Region_8989	chr19	13731760	13763296	31536	33	207	248.8333333	18.94005388	0.786363649	0.020725133	+	81576	grey	-0.256269559	FALSE	YJU2B
Region_8990	chr19	13764522	13774282	9760	7	27	45.37878788	7.159211713	0.833507086	0.028781828	+	84245	grey	-0.240019416	FALSE	MRI1
Region_8991	chr19	13774456	13778773	4317	3	16	23.27272727	4.240992099	0.728802138	0.093252344	+	28974	grey	0.015246154	FALSE	C19orf53
Region_8992	chr19	14305458	14370196	64738	67	405	464.6666667	26.249884	0.880743312	0.016949043	-	105372288	grey	-0.049748013	FALSE	LINC01841
Region_8993	chr19	14333743	14344074	10331	8	35	46.42424242	5.867932688	0.929623607	0.057484063	+	101928845	grey	0.065646876	FALSE	LINC01842
Region_8994	chr19	14380501	14408725	28224	22	132	157.2727273	14.28452143	0.788098609	0.030181849	+	976	grey	-0.024151445	FALSE	ADGRE5
Region_8995	chr19	14408798	14419383	10585	17	91	112.9242424	12.17982014	0.761597846	0.037500815	-	10212	grey	-0.053017768	FALSE	DDX39A
Region_8996	chr19	14433053	14471867	38814	22	147	179.6666667	14.67843351	0.911353497	0.023623629	+	5585	grey	-0.127406691	FALSE	PKN1
Region_8997	chr19	14472466	14475354	2888	12	60	91.65151515	16.04943558	0.255419248	0.050715911	-	5731	grey	0.312604738	FALSE	PTGER1
Region_8998	chr19	14477760	14496149	18389	17	90	108.1060606	10.4087667	0.908854931	0.026665966	-	10755	grey	0.348301083	FALSE	GIPC1
Region_8999	chr19	14514769	14560262	45493	62	422	470.469697	24.85788511	0.54293914	0.012331965	-	3337	grey	0.044112559	FALSE	DNAJB1

Region_9000	chr19	14517085	14565980	48895	68	441	503.3030303	25.76220828	0.59142458	0.011867064	+	9524	grey	0.081034858	FALSE	TECR
Region_9001	chr19	14527726	14560391	32665	45	306	351.3030303	20.48787236	0.603476506	0.014248956	-	112268253	grey	0.125955215	FALSE	
Region_9002	chr19	14529543	14532528	2985	12	68	102.1818182	15.46997995	0.010196498	0.009883824	+	693224	grey	-0.045180751	FALSE	MIR639
Region_9003	chr19	14566078	14572066	5988	5	35	47.93939394	6.324260462	0.734901626	0.04262727	-	4713	grey	-0.011087214	FALSE	NDUFB7
Region_9004	chr19	14583084	14612035	28951	18	105	130.3030303	12.46900119	0.880996368	0.031051356	+	388512	grey	-0.075627316	FALSE	CLEC17A
Region_9005	chr19	14619117	14690027	70910	48	296	352.0606061	23.80360592	0.861612684	0.01971539	-	84658	grey	-0.050613582	FALSE	ADGRE3
Region_9006	chr19	14689801	14733746	43945	16	95	123.8181818	11.31494463	0.959541994	0.029717751	+	84449	grey	-0.238609702	FALSE	ZNF333
Region_9007	chr19	14732392	14778560	46168	18	88	119.2424242	12.34262234	0.804332503	0.052422131	-	30817	grey	0.109460275	FALSE	ADGRE2
Region_9008	chr19	14789260	14835376	46116	10	72	89.83333333	9.329165736	0.837745718	0.03081288	-	26664	grey	-0.01095279	FALSE	OR7C1
Region_9009	chr19	14809815	14835197	25382	7	50	69.68181818	8.510914424	0.782144025	0.037782782	-	26659	grey	-0.204443665	FALSE	OR7A5
Region_9010	chr19	14840466	14848922	8456	3	15	25.57575758	4.791211846	0.936548918	0.063138507	-	390892	grey	0.000529511	FALSE	OR7A10
Region_9011	chr19	14878203	14886132	7929	6	23	36.12121212	5.650092311	0.765119631	0.046877976	-	26333	grey	0.132162247	FALSE	OR7A17
Region_9012	chr1	14950033	15022990	72957	17	84	107.863636	11.8527976	0.70972382	0.03639939	-	6511	grey	0.102247447	FALSE	SLC1A6

	9						4	1	6	4						
Region_9013	chr19	15049480	15058293	8813	3	11	21.27272727	4.565912006	0.737947855	0.092318738	+	23581	grey	0.261205632	FALSE	CASP14
Region_9014	chr19	15082211	15092970	10759	8	56	72.01515152	8.848280883	0.803392149	0.075244606	+	126370	grey	-0.187438369	FALSE	OR1I1
Region_9015	chr19	15107401	15114985	7584	4	12	21.16666667	4.602953344	0.72654822	0.058769467	+	85360	grey	0.116338555	FALSE	SYDE1
Region_9016	chr19	15114984	15125786	10802	9	39	58.92424242	8.979139253	0.74983326	0.034943894	-	10994	grey	-0.314888437	FALSE	ILVBL
Region_9017	chr19	15159038	15200995	41957	27	178	207.0606061	15.22536223	0.751233425	0.01751895	-	4854	grey	-0.237933443	FALSE	NOTCH3
Region_9018	chr19	15226919	15233435	6516	10	54	78.31818182	10.17411359	0.391013116	0.027924352	-	79852	grey	-0.104766788	FALSE	EPHX3
Region_9019	chr19	15235519	15332545	97026	69	520	568.3939394	25.98572317	0.739929166	0.01760602	-	23476	grey	0.108439105	FALSE	BRD4
Region_9020	chr19	15353385	15379815	26430	35	244	281.469697	16.89535184	0.849139279	0.019053262	-	10270	grey	-0.200086262	FALSE	AKAP8
Region_9021	chr19	15380050	15419141	39091	28	180	227.4545455	21.12321495	0.695968569	0.014359475	-	26993	grey	-0.228013611	FALSE	AKAP8L
Region_9022	chr19	15419978	15449956	29978	29	157	204.0454545	17.44656177	0.842600056	0.019056947	-	58525	grey	-0.022955899	FALSE	WIZ
Region_9023	chr19	15451624	15464544	12920	14	60	85.78787879	12.16996954	0.976258949	0.016559093	-	64926	grey	-0.075203286	FALSE	RASAL3
Region_9024	chr19	15468645	15498956	30311	26	178	212.6515152	16.20493033	0.866638095	0.020659742	-	114770	grey	0.016573781	FALSE	PGLYRP2

Region_9025	chr19	15508525	15552317	43792	31	207	244.2575758	17.99000629	0.713975026	0.023777627	+	126410	grey	0.148548201	FALSE	CYP4F22
Region_9026	chr19	15615218	15630639	15421	4	23	37.37878788	5.66708486	0.479029622	0.066285776	+	11283	grey	-0.259502797	FALSE	CYP4F8
Region_9027	chr19	15640897	15662825	21928	5	21	34.51515152	6.317843898	0.794133098	0.101852303	+	4051	grey	-0.178195863	FALSE	CYP4F3
Region_9028	chr19	15804549	15815664	11115	5	23	33.48484848	5.265979124	0.867464455	0.055322468	-	26539	grey	-0.089507473	FALSE	OR10H1
Region_9029	chr19	15846262	15864924	18662	6	23	36.04545455	6.629611471	0.802211781	0.094018339	-	102724279	grey	-0.349091754	FALSE	CLEC4OP
Region_9030	chr19	15912367	15934867	22500	10	55	79.06060606	9.016017862	0.856430232	0.05444613	-	57834	grey	-0.165958986	FALSE	CYP4F11
Region_9031	chr19	16015287	16027462	12175	3	14	24.12121212	4.698658242	0.92054584	0.088333016	+	126536	grey	0.162568607	FALSE	LINC00661
Region_9032	chr19	16031170	16042142	10972	5	35	48.18181818	6.355879388	0.772113183	0.049828893	+	148231	grey	-0.024845539	FALSE	LINC00905
Region_9033	chr19	16067021	16103002	35981	21	142	164.5	11.52021634	0.51703837	0.025435922	+	7171	grey	-0.145319484	FALSE	TPM4
Region_9034	chr19	16111889	16134234	22345	19	104	129.1363636	13.82626972	0.833745542	0.042696945	+	4218	grey	-0.260680953	FALSE	RAB8A
Region_9035	chr19	16134028	16158575	24547	15	116	140.4393939	11.60123175	0.557559181	0.047696907	+	84941	grey	0.247065462	FALSE	HSH2D
Region_9036	chr19	16185380	16192046	6666	14	100	125.2424242	13.10438635	0.887759724	0.026803105	+	26017	grey	0.320780806	FALSE	FAM32A
Region_9037	chr1	16197854	16245906	48052	37	231	273.575757	20.3470357	0.82697437	0.01736237	+	8907	grey	0.054750475	FALSE	AP1M1

	9						6	7	8	2						
Region_9038	chr19	16324826	16328685	3859	3	10	23.33333333	4.896885457	0.00838945	0.019018075	+	10365	grey	-0.103050749	FALSE	KLF2
Region_9039	chr19	16355239	16472085	116846	107	830	920.8636364	34.77969627	0.793885163	0.017002233	-	58513	grey	0.214839102	FALSE	EPS15L1
Region_9040	chr19	16479061	16496167	17106	18	113	140.4242424	12.21356195	0.929171721	0.025872074	-	125972	grey	0.092179166	FALSE	CALR3
Region_9041	chr19	16479067	16629062	149995	149	1054	1154.772727	47.12235969	0.71349627	0.00880139	-	9441	grey	-0.035939035	FALSE	MED26
Region_9042	chr19	16496394	16521352	24958	18	115	139.9090909	11.14895962	0.834856526	0.020400197	+	84167	grey	0.313458008	FALSE	C19orf44
Region_9043	chr19	16517894	16542437	24543	38	211	263.6212121	22.10169861	0.804967111	0.017625942	-	10523	grey	-0.075755397	FALSE	CHERP
Region_9044	chr19	16549837	16572415	22578	22	140	169.530303	13.34076425	0.585846556	0.018906911	-	79939	grey	0.077467393	FALSE	SLC35E1
Region_9045	chr19	16577642	16660442	82800	81	608	665.5151515	29.11105654	0.756777362	0.01382024	-	79086	grey	-0.137715942	FALSE	SMIM7
Region_9046	chr19	16661139	16690023	28884	28	173	211.1363636	16.10340276	0.81073943	0.020209397	+	79041	grey	0.267354924	FALSE	TMEM38A
Region_9047	chr19	16719847	16817963	98116	45	313	355.8787879	22.02202754	0.821620128	0.019996272	+	284434	grey	-0.319319058	FALSE	NWD1
Region_9048	chr19	16829398	16880353	50955	41	248	319.6666667	23.88905983	0.936346905	0.013715419	+	23309	grey	0.29317025	FALSE	SIN3B
Region_9049	chr19	16888999	16892606	3607	3	13	26.92424242	5.987963841	0.886276569	0.069997277	+	9002	grey	0.118686869	FALSE	F2RL3

Region_9050	chr19	16892951	17026815	133864	77	526	594.1515152	27.73625366	0.893378445	0.016681224	-	27151	grey	0.319833845	FALSE	CPAMD8
Region_9051	chr19	17049729	17075625	25896	17	113	141.3484848	12.97867176	0.793281102	0.017075386	-	93323	grey	-0.137076675	FALSE	HAUS8
Region_9052	chr19	17075777	17214537	138760	116	793	871.1212121	41.79529423	0.879432724	0.014333484	+	4650	grey	-0.230436539	FALSE	MYO9B
Region_9053	chr19	17215346	17219829	4483	5	24	34.62121212	5.249952825	0.968600779	0.038889977	+	55850	grey	-0.167876631	FALSE	USE1
Region_9054	chr19	17231883	17245919	14036	27	154	186.4848485	14.63109694	0.654762891	0.024094348	-	2063	grey	-0.125540948	FALSE	NR2F6
Region_9055	chr19	17235940	17282786	46846	50	293	330.8030303	18.11690218	0.67333253	0.018451013	-	83878	grey	-0.195272624	FALSE	USHBP1
Region_9056	chr19	17267376	17282966	15590	11	58	78.33333333	9.152273082	0.476948426	0.04434223	+	29086	grey	-0.426918713	FALSE	BABAM1
Region_9057	chr19	17281645	17287646	6001	10	43	62.56060606	9.86076731	0.597427989	0.035799812	+	126549	grey	-0.373404118	FALSE	ANKLE1
Region_9058	chr19	17292131	17310236	18105	40	248	291.7727273	19.81358932	0.462762629	0.014321758	-	79575	grey	0.107340445	FALSE	ABHD8
Region_9059	chr19	17292609	17306843	14234	31	190	234.3636364	17.2373801	0.495873537	0.016176961	+	64981	grey	0.178653825	FALSE	MRPL34
Region_9060	chr19	17309518	17323298	13780	25	133	165.5	14.97921637	0.928988795	0.022470851	+	79016	grey	-0.303243645	FALSE	DDA1
Region_9061	chr19	17323223	17334855	11632	26	137	159.2424242	12.18203041	0.654122946	0.027511302	-	57719	grey	-0.114675638	FALSE	ANO8
Region_9062	chr1	17334920	17342731	7811	22	140	175.818181	17.5594893	0.29058549	0.02094586	+	84705	grey	-0.05839362	FALSE	GTPBP3

	9						8		3	4				1		
Region_9063	chr19	17351450	17377342	25892	37	208	271.1515152	20.77442394	0.850853128	0.026823406	-	83483	grey	0.090520094	FALSE	PLVAP
Region_9064	chr19	17387196	17394196	7000	13	56	85.31818182	12.4482425	0.594848735	0.068542722	-	110806280	grey	0.144532377	FALSE	CCDC194
Region_9065	chr19	17405686	17420130	14444	15	87	115.2121212	12.1129494	0.67208084	0.023962997	+	105221694	grey	-0.093297099	FALSE	BISPR
Region_9066	chr19	17405722	17433724	28002	35	217	256.4242424	19.13841132	0.657913783	0.014252631	+	93343	grey	0.105400218	FALSE	MVB12A
Region_9067	chr19	17435509	17448668	13159	28	163	197.3181818	17.46923369	0.865334911	0.018564177	-	100130519	grey	0.120569088	FALSE	TMEM221
Region_9068	chr19	17436611	17460926	24315	44	229	285.1060606	22.89180491	0.737846678	0.019374536	-	115861	grey	-0.073738375	FALSE	NXNL1
Region_9069	chr19	17468769	17506168	37399	47	260	304.030303	17.90914652	0.780374122	0.020574004	+	376497	grey	-0.222978145	FALSE	SLC27A1
Region_9070	chr19	17488990	17511889	22899	26	150	183	15.18501285	0.804866402	0.023536079	-	100507551	grey	-0.046189421	FALSE	PGLS-DT
Region_9071	chr19	17511636	17521288	9652	15	75	103.3030303	13.28268937	0.433779502	0.01800734	+	25796	grey	-0.201692936	FALSE	PGLS
Region_9072	chr19	17523301	17553839	30538	27	136	183.6969697	16.70188354	0.697910455	0.03654371	+	199786	grey	-0.005800806	FALSE	NIBAN3
Region_9073	chr19	17555649	17583162	27513	31	173	234.3333333	19.40050224	0.921709352	0.017908473	+	79709	grey	-0.22419551	FALSE	COLGALT1
Region_9074	chr19	17601336	17688365	87029	76	450	518.8333333	27.51312041	0.692344302	0.020142686	-	23025	grey	0.018950789	FALSE	UNC13A

Region_9075	chr19	17719242	17734513	15271	14	77	100.4545455	10.37479463	0.772679089	0.020607594	+	55201	grey	0.186008406	FALSE	MAP1S
Region_9076	chr19	17747718	17788568	40850	33	189	223.3333333	14.74014229	0.770047618	0.023372902	+	23149	grey	-0.124423007	FALSE	FCHO1
Region_9077	chr19	17794828	17813576	18748	8	45	61.46969697	7.92895317	0.697904824	0.047299831	+	10331	grey	0.294316111	FALSE	B3GNT3
Region_9078	chr19	17816512	17821574	5062	4	14	24.65151515	4.846862345	0.964180128	0.04707148	-	3640	grey	0.134964275	FALSE	INSL3
Region_9079	chr19	17824780	17848071	23291	33	170	211.3636364	17.38136612	0.927594957	0.018033528	-	3718	grey	0.223206701	FALSE	JAK3
Region_9080	chr19	17859910	17864153	4243	8	50	66.6969697	6.149345674	0.575948163	0.037863261	+	6142	grey	0.177227482	FALSE	RPL18A
Region_9081	chr19	17871945	17895174	23229	26	166	197.3181818	14.82841253	0.732691028	0.020855467	+	6528	grey	-0.192570552	FALSE	SLC5A5
Region_9082	chr19	17933015	17943991	10976	11	57	88.3030303	11.1119751	0.821262674	0.030197136	+	115098	grey	-0.052944423	FALSE	CCDC124
Region_9083	chr19	17951293	18000085	48792	52	326	369.8939394	21.12317633	0.700766196	0.019220512	+	3780	grey	0.061477216	FALSE	KCNN1
Region_9084	chr19	18001132	18014102	12970	12	83	105.9090909	11.08668956	0.458038228	0.027310958	+	27106	grey	-0.086497475	FALSE	ARRDC2
Region_9085	chr19	18058995	18098944	39949	41	249	307.3636364	19.91337885	0.879762905	0.016715362	-	3594	grey	-0.259808796	FALSE	IL12RB1
Region_9086	chr19	18097778	18151692	53914	75	455	512.9393939	30.16920426	0.638319473	0.014077785	+	23031	grey	0.443665474	FALSE	MAST3
Region_9087	chr1	18153158	18178117	24959	27	138	182.227272	15.6606272	0.72134455	0.02466179	+	5296	grey	-0.02393004	FALSE	PIK3R2

	9						7	1		3				2		
Region_9088	chr19	18173162	18178117	4955	5	21	33	5.477225575	0.234599684	0.041075848	+	10437	grey	-0.061323364	FALSE	IFI30
Region_9089	chr19	18193218	18196948	3730	3	14	24.95454545	4.981293679	0.207289035	0.081202669	+	84769	grey	-0.118144382	FALSE	MPV17L2
Region_9090	chr19	18196784	18204042	7258	7	37	46.66666667	6.217427945	0.523851273	0.067326244	-	5864	grey	-0.172799771	FALSE	RAB3A
Region_9091	chr19	18204730	18220480	15750	23	150	183.6212121	12.63909095	0.727450916	0.030000668	+	102725254	grey	-0.272559565	FALSE	LOC102725254
Region_9092	chr19	18207961	18255419	47458	63	411	475.4545455	26.47098953	0.627264947	0.022165546	-	5143	grey	-0.343084695	FALSE	PDE4C
Region_9093	chr19	18257098	18274500	17402	24	151	189.7121212	14.00853053	0.933774734	0.026634122	-	80726	grey	-0.276577152	FALSE	IQCN
Region_9094	chr19	18279694	18281622	1928	19	88	128.3333333	18.18057572	0.013213768	0.013753155	-	3727	grey	0.104669999	FALSE	JUND
Region_9095	chr19	18306236	18323112	16876	33	220	262.6818182	20.69571423	0.765738159	0.020791842	-	25804	grey	-0.04987358	FALSE	LSM4
Region_9096	chr19	18340598	18369950	29352	29	188	227.7121212	17.19102194	0.933356808	0.017510269	+	54858	grey	0.078496867	FALSE	PGPEP1
Region_9097	chr19	18374731	18389176	14445	18	115	154.1212121	16.85094396	0.610293461	0.026500593	+	9518	grey	-0.218560941	FALSE	GDF15
Region_9098	chr19	18391137	18397622	6485	10	60	83.81818182	9.805664126	0.56671647	0.041772602	-	126364	grey	-0.110892607	FALSE	LRRC25
Region_9099	chr19	18418864	18434562	15698	28	135	160.6363636	15.18261796	0.637170362	0.028342819	+	170463	grey	0.043114836	FALSE	SSBP4

Region_9100	chr19	18434388	18438167	3779	5	17	26.63636364	5.284626073	0.537381049	0.081328698	-	51477	grey	0.043475996	FALSE	ISYNA1
Region_9101	chr19	18442663	18522116	79453	70	463	521.2878788	27.03033347	0.779769151	0.015313664	-	8178	grey	-0.013703464	FALSE	ELL
Region_9102	chr19	18531751	18544077	12326	16	91	120.5909091	13.04899321	0.966357824	0.021833936	-	23770	grey	0.208968057	FALSE	FKBP8
Region_9103	chr19	18557762	18569387	11625	15	94	122.8333333	14.29102282	0.901329241	0.014902808	+	79036	grey	-0.112011994	FALSE	KXD1
Region_9104	chr19	18571730	18577550	5820	6	32	48.07575758	8.77813824	0.646556107	0.023020392	+	7311	grey	-0.0201625	FALSE	UBA52
Region_9105	chr19	18572220	18607741	35521	40	236	271.8484848	18.05141582	0.708265564	0.022633372	-	9244	grey	-0.148501418	FALSE	CRLF1
Region_9106	chr19	18588685	18592337	3652	9	37	50.75757576	7.595668008	0.448359053	0.067347556	+	55049	grey	-0.034108256	FALSE	REX1BD
Region_9107	chr19	18607430	18621039	13609	7	30	47.68181818	7.106138389	0.043891684	0.04313262	+	25789	grey	-0.005882562	FALSE	TMEM59L
Region_9108	chr19	18637025	18671721	34696	57	342	392.3636364	29.18779321	0.832435935	0.01791242	+	55295	grey	0.123677931	FALSE	KLHL26
Region_9109	chr19	18683678	18782333	98655	101	688	764.1363636	33.88527539	0.907712421	0.011995789	+	23373	grey	-0.111074286	FALSE	CRTC1
Region_9110	chr19	18782773	18791305	8532	19	80	108.8939394	12.61882451	0.11970286	0.029031322	-	1311	grey	0.191821938	FALSE	COMP
Region_9111	chr19	18831959	18868230	36271	58	391	449.9545455	29.58452393	0.747657566	0.012804798	+	5976	grey	0.210702386	FALSE	UPF1
Region_9112	chr1	18868545	18896158	27613	55	292	375.621212	30.0741469	0.70494438	0.02045512	-	2657	grey	0.385897571	FALSE	GDF1

	9						1	1		2						
Region_9113	chr19	18877909	18896727	18818	44	210	276.7878788	25.25349338	0.655314633	0.020714689	-	10715	grey	-0.113526054	FALSE	CERS1
Region_9114	chr19	18899514	18919387	19873	21	126	153.3939394	12.97082974	0.966094718	0.016970057	-	11316	grey	0.274641725	FALSE	COPE
Region_9115	chr19	18907013	18941261	34248	26	142	185.5	15.71011433	0.753795281	0.022909081	-	9454	grey	0.045453161	FALSE	HOMER3
Region_9116	chr19	18919705	18929189	9484	10	59	79.18181818	8.448776135	0.705322071	0.048566753	+	54555	grey	-0.003201527	FALSE	DDX49
Region_9117	chr19	18940235	18947452	7217	9	40	59.37878788	8.187730317	0.671097642	0.051456467	+	102724360	grey	0.104455998	FALSE	HOMER3-AS1
Region_9118	chr19	18990888	19034023	43135	43	245	290.3787879	19.32297568	0.687577731	0.013348183	-	10147	grey	-0.110262892	FALSE	SUGP2
Region_9119	chr19	19033575	19060311	26736	24	144	180.1060606	13.29792209	0.891033731	0.019834756	+	93436	grey	-0.13637792	FALSE	ARMC6
Region_9120	chr19	19063994	19113030	49036	36	261	289.6060606	19.96988409	0.887473816	0.01395343	+	284439	grey	0.146898879	FALSE	SLC25A42
Region_9121	chr19	19119169	19138513	19344	18	107	136.6666667	11.90259614	0.787598204	0.022162762	-	54929	grey	-0.161770698	FALSE	TMEM161A
Region_9122	chr19	19145567	19170289	24722	16	74	95.13636364	11.13675324	0.597105567	0.075966308	-	100271849	grey	-0.116785845	FALSE	MEF2B
Region_9123	chr19	19145567	19192158	46591	33	177	215.530303	17.90850223	0.708221058	0.047788631	-	4207	grey	-0.104722881	FALSE	BORCS8-MEF2B
Region_9124	chr19	19176903	19192591	15688	10	40	65.13636364	9.418290493	0.929677729	0.040061091	-	729991	grey	0.105411485	FALSE	BORCS8

Region_9125	chr19	19192229	19201869	9640	4	17	25.60606061	4.676878604	0.738873858	0.030731248	+	8625	grey	0.163473983	FALSE	RFXANK
Region_9126	chr19	19211958	19252233	40275	39	267	311.4090909	19.96456651	0.723274047	0.025720331	+	1463	grey	-0.142552179	FALSE	NCAN
Region_9127	chr19	19254748	19273369	18621	20	111	135.9090909	11.53686702	0.668528752	0.040319944	-	404037	grey	-0.17687926	FALSE	HAPLN4
Region_9128	chr19	19264364	19273391	9027	5	20	32.07575758	5.242755019	0.753430492	0.04380347	-	53345	grey	0.09848694	FALSE	TM6SF2
Region_9129	chr19	19276018	19320509	44491	37	266	300.8484848	18.95682741	0.916677924	0.011357201	-	57794	grey	-0.228792571	FALSE	SUGP1
Region_9130	chr19	19320829	19358754	37925	25	173	199.8181818	13.13118319	0.839485871	0.029438233	+	23383	grey	-0.220699401	FALSE	MAU2
Region_9131	chr19	19385826	19508932	123106	92	652	729.3787879	33.54526914	0.789584182	0.013506635	+	54815	grey	0.062630155	FALSE	GATAD2A
Region_9132	chr19	19514219	19515548	1329	3	13	22.15151515	4.554769011	0.8865298	0.089883826	-	83983	grey	-0.014713037	FALSE	TSSK6
Region_9133	chr19	19515736	19529054	13318	27	200	256.2878788	24.70363732	0.330329883	0.018451042	+	51079	grey	-0.216017851	FALSE	NDUFA13
Region_9134	chr19	19516227	19537581	21354	26	184	236.8181818	24.44202503	0.425519768	0.019645762	+	374887	grey	-0.166386264	FALSE	YJEFN3
Region_9135	chr19	19538248	19546659	8411	11	60	83.31818182	10.70608611	0.735509707	0.060038522	+	148113	grey	0.076254905	FALSE	CILP2
Region_9136	chr19	19561707	19618693	56986	41	269	309.8939394	18.52493437	0.930944496	0.016968354	-	80714	grey	0.052788584	FALSE	PBX4
Region_9137	chr1	19623655	19628930	5275	15	94	124.590909	15.9325281	0.74917842	0.02336906	-	9170	grey	-0.13332818	FALSE	LPAR2

	9						1	9	1	9				3		
Region_9138	chr19	19629476	19643657	14181	21	126	163.1515152	16.77291078	0.894889352	0.020933502	-	51291	grey	0.054069206	FALSE	GMIP
Region_9139	chr19	19645198	19663676	18478	48	326	377.2727273	22.58150738	0.811954427	0.019256222	-	57130	grey	0.101544218	FALSE	ATP13A1
Region_9140	chr19	19668796	19683506	14710	13	82	106.1969697	8.534490561	0.827652156	0.027650507	+	94039	grey	0.040922203	FALSE	ZNF101
Region_9141	chr19	19710472	19733112	22640	12	70	91.46969697	10.21645686	0.651736979	0.021038883	-	7561	grey	0.059791384	FALSE	ZNF14
Region_9142	chr19	19749637	19754986	5349	5	24	44.57575758	7.585534018	0.936344743	0.035518512	-	101060187	grey	-0.030616287	FALSE	LOC101060187
Region_9143	chr19	19757366	19776413	19047	24	157	184.0757576	11.70445097	0.838251831	0.02341023	-	284440	grey	-0.255339544	FALSE	LINC00663
Region_9144	chr19	19761038	19821751	60713	42	284	331.4848485	19.94163979	0.808900183	0.014743562	-	440515	grey	-0.037559205	FALSE	ZNF506
Region_9145	chr19	19776561	19836196	59635	23	165	197.0454545	15.5449045	0.804171915	0.01241508	+	7608	grey	0.282594468	FALSE	ZNF56P
Region_9146	chr19	19776586	19816005	39419	20	135	164.1212121	14.23485987	0.879423221	0.013868826	+	112267872	grey	0.27919121	FALSE	
Region_9147	chr19	19865882	19894674	28792	10	65	83.46969697	9.157123662	0.768408255	0.025774895	+	56242	grey	-0.129119426	FALSE	ZNF253
Region_9148	chr19	19900913	19935575	34662	7	40	57.45454545	7.034381102	0.975029219	0.022008547	+	81931	grey	0.327837784	FALSE	ZNF93
Region_9149	chr19	19997058	20039505	42447	14	81	104.7575758	11.38968576	0.952101685	0.028660539	-	91120	grey	-0.24400482	FALSE	ZNF682

Region_9150	chr19	20077994	20127076	49082	11	52	71.1969697	7.678580993	0.972726646	0.021333122	+	7643	grey	0.138931451	FALSE	ZNF90
Region_9151	chr19	20122569	20321305	198736	54	374	435.6212121	27.0092663	0.873159036	0.017811982	-	105372310	grey	-0.202841942	FALSE	LOC105372310
Region_9152	chr19	20167214	20200488	33274	9	55	68.92424242	7.342836606	0.8659434	0.047408542	+	90649	grey	-0.168771573	FALSE	ZNF486
Region_9153	chr19	20340262	20424986	84724	17	99	135.5151515	12.57988923	0.560471572	0.03581114	-	664701	grey	0.0533962	FALSE	ZNF826P
Region_9154	chr19	20432552	20528615	96063	7	18	45.63636364	8.183729381	0.918923778	0.047404991	+	105372317	grey	0.101927342	FALSE	LOC105372317
Region_9155	chr19	20472986	20571095	98109	5	14	30.06060606	5.804186256	0.906095037	0.063518769	+	105372316	grey	0.073724519	FALSE	LOC105372316
Region_9156	chr19	20553792	20661596	107804	19	120	154.8939394	14.37425989	0.941951317	0.021147831	-	199777	grey	0.168579703	FALSE	ZNF626
Region_9157	chr19	20611559	20615389	3830	3	15	24.45454545	4.177528965	0.956724149	0.040994227	+	105372318	grey	0.241246326	FALSE	LOC105372318
Region_9158	chr19	21082159	21125270	43111	4	13	24.25757576	4.971644539	0.953928763	0.074628927	+	148206	grey	0.239074656	FALSE	ZNF714
Region_9159	chr19	21142024	21196053	54029	7	39	53.98484848	6.524492139	0.961089505	0.037317197	+	170959	grey	0.24228546	FALSE	ZNF431
Region_9160	chr19	21291160	21329425	38265	4	16	24.37878788	4.349235659	0.966862084	0.032404148	-	7562	grey	0.117540657	FALSE	ZNF708
Region_9161	chr19	21358930	21388582	29652	3	13	20.98484848	4.753921213	0.500041821	0.126410374	+	148203	grey	-0.010144785	FALSE	ZNF738
Region_9162	chr1	21405159	21503238	98079	15	95	123.454545	9.65589786	0.73200959	0.02316251	+	400680	grey	0.046413547	FALSE	LINC00664

	9						5	8	3	8						
Region_9163	chr19	21444241	21463908	19667	5	29	46.74242424	6.56168507	0.963248517	0.027045077	-	105372321	grey	0.11042539	FALSE	LOC105372321
Region_9164	chr19	21496682	21555397	58715	12	47	67.13636364	9.645862188	0.950023535	0.032168226	+	353088	grey	-0.078023648	FALSE	ZNF429
Region_9165	chr19	21569738	21594628	24890	9	46	67.45454545	8.765635381	0.851009776	0.065629054	-	101929007	grey	-0.009321317	FALSE	LOC101929007
Region_9166	chr19	21932958	22010949	77991	5	23	36.3030303	5.815019537	0.293514518	0.067422038	-	7757	grey	0.055650859	FALSE	ZNF208
Region_9167	chr19	22017898	22052370	34472	6	38	55.07575758	6.823738218	0.883647103	0.054922746	-	112268248	grey	0.109909447	FALSE	LOC112268248
Region_9168	chr19	22052430	22091480	39050	5	30	43.3030303	7.350499087	0.834857616	0.076937571	+	113835	grey	0.098338413	FALSE	ZNF257
Region_9169	chr19	22286441	22317176	30735	7	36	53.56060606	6.315131494	0.534177288	0.070926166	+	100287226	grey	-0.28966811	FALSE	ZNF729
Region_9170	chr19	22391019	22422357	31338	4	18	28.34848485	5.313241584	0.903803518	0.067823267	-	148198	grey	0.042345595	FALSE	ZNF98
Region_9171	chr19	22403434	22532485	129051	19	124	154.1969697	13.37531561	0.804913169	0.030427274	-	105376917	grey	0.004683215	FALSE	LOC105376917
Region_9172	chr19	22615551	22623980	8429	7	35	50.68181818	8.573948181	0.390038485	0.07889448	-	374890	grey	0.087185246	FALSE	LINC01785
Region_9173	chr19	22634324	22667671	33347	3	10	20.66666667	5.559007475	0.95904044	0.044744839	+	57615	grey	-0.421932877	FALSE	ZNF492
Region_9174	chr19	22752183	22784151	31968	14	77	107.8636364	10.22197687	0.812570464	0.038893536	-	7652	grey	-0.028180322	FALSE	ZNF99

Region_9175	chr19	22832291	22858667	26376	8	50	68.63636364	8.910903198	0.918945025	0.035809105	+	646864	grey	-0.125719088	FALSE	ZNF723
Region_9176	chr19	22974883	23003176	28293	3	10	18.36363636	4.086135521	0.854033607	0.08320247	-	388523	grey	0.047783203	FALSE	ZNF728
Region_9177	chr19	23015059	23031818	16759	3	19	31.93939394	5.563282893	0.897582572	0.057411155	-	101929164	grey	0.022575544	FALSE	LINC01859
Region_9178	chr19	23046958	23063705	16747	10	53	88.10606061	11.18670748	0.896485472	0.046096515	-	101929144	grey	0.009129187	FALSE	LINC01858
Region_9179	chr19	23075201	23147221	72020	18	136	162.0909091	11.93666269	0.824274971	0.025867662	+	100129543	grey	-0.031472521	FALSE	ZNF730
Region_9180	chr19	23221599	23274221	52622	19	116	142.4242424	13.44282549	0.839721439	0.031265646	-	440519	grey	0.037849937	FALSE	ZNF724
Region_9181	chr19	23257255	23274251	16996	10	51	73.72727273	10.06065521	0.885058944	0.048047519	-	100132815	grey	-0.087324054	FALSE	IPO5P1
Region_9182	chr19	23304991	23395471	90480	24	170	198.6212121	13.46764231	0.955988149	0.01516177	-	7644	grey	0.032767693	FALSE	ZNF91
Region_9183	chr19	23525631	23687197	161566	33	209	247.4393939	16.91165168	0.86420813	0.021076119	-	105372337	grey	-0.338064466	FALSE	LOC105372337
Region_9184	chr19	23534180	23687220	153040	32	207	244	16.87784164	0.861129663	0.021449086	-	171392	grey	-0.321653339	FALSE	ZNF675
Region_9185	chr19	23762944	23871162	108218	14	87	109.8030303	10.30641279	0.85192806	0.031649795	+	388524	grey	-0.159540873	FALSE	RPSA2
Region_9186	chr19	23914876	23945159	30283	7	41	58.31818182	8.447410329	0.825835014	0.039813779	+	730087	grey	0.046157765	FALSE	ZNF726
Region_9187	chr1	23919081	23958035	38954	7	41	56.0909090	8.03976133	0.82680431	0.04210217	-	10050585	grey	0.127277955	FALSE	LOC100505851

	9						9		6			1				
Region_9188	chr1 9	24033401	24129968	96567	9	57	74.9848484 8	9.25617037 3	0.93801341	0.04040400 8	+	9534	grey	-0.26715043 7	FALSE	ZNF254
Region_9189	chr1 9	27681072	27793703	112631	7	49	62.9090909 1	7.19343134 7	0.90419016 2	0.04162496 7	-	10537234 5	grey	-0.25788132 7	FALSE	LOC105372345
Region_9190	chr1 9	27681072	27794005	112933	8	53	72.4696969 7	7.84507625 5	0.79193143 6	0.03669319 7	-	148189	grey	-0.28907839 8	FALSE	LINC00662
Region_9191	chr1 9	27793431	27984984	191553	14	90	113.560606 1	9.72724767 4	0.71881333 5	0.03736224 6	+	10192715 1	grey	0.192830959	FALSE	LINC02987
Region_9192	chr1 9	28294093	28386178	92085	12	82	110.212121 2	9.34717588 2	0.88000453 3	0.03805755 5	-	10798526 9	grey	-0.14650072 6	FALSE	
Region_9193	chr1 9	28435388	28727777	292389	41	321	361.439393 9	18.8770343 1	0.83177926	0.02240043 8	-	10042058 7	grey	0.263287171	FALSE	LOC100420587
Region_9194	chr1 9	28606688	28615229	8541	3	20	28.1818181 8	3.76587548 7	0.88003428	0.07957559 7	+	10272490 8	grey	-0.11453578 7	FALSE	LOC102724908
Region_9195	chr1 9	28883532	28970874	87342	18	116	148.939393 9	11.6788813 8	0.8354143	0.03214238 6	+	148145	grey	0.011902723	FALSE	LINC00906
Region_9196	chr1 9	29287011	29525752	238741	52	376	421.242424 2	23.8579518 1	0.76869879 4	0.02356342 8	-	284395	grey	-0.03682827 2	FALSE	VSTM2B-DT
Region_9197	chr1 9	29525994	29564551	38557	13	79	102.181818 2	9.55294411 5	0.72509522 4	0.03266445 3	+	342865	grey	0.114831239	FALSE	VSTM2B
Region_9198	chr1 9	29606283	29617237	10954	5	30	43.3030303	5.57458435	0.62034873 7	0.04378753 1	+	10775	grey	0.1264652	FALSE	POP4
Region_9199	chr1 9	29665459	29675477	10018	4	22	32.5757575 8	5.48020383 1	0.63513385 6	0.05872893 1	+	79156	grey	-0.01889044 8	FALSE	PLEKHF1

Region_9200	chr19	29698937	29715789	16852	12	70	94.93939394	11.89166326	0.906434172	0.027381555	-	83636	grey	0.324320925	FALSE	C19orf12
Region_9201	chr19	29811991	29824312	12321	4	19	28.3030303	5.510743903	0.506414727	0.052480965	+	898	grey	0.194327582	FALSE	CCNE1
Region_9202	chr19	29923644	30016612	92968	19	133	155.5757576	13.27236644	0.732015332	0.025681779	+	8725	grey	-0.026314005	FALSE	URI1
Region_9203	chr19	30078133	30085893	7760	6	25	45.62121212	7.293337637	0.718297908	0.048974943	+	107985271	grey	-0.042543051	FALSE	LOC107985271
Region_9204	chr19	30228290	30713538	485248	188	1503	1580.045455	49.29235604	0.772100489	0.010574288	+	9745	grey	0.112184996	FALSE	ZNF536
Region_9205	chr19	31100304	31147981	47677	14	90	122.5757576	9.769750184	0.902264962	0.033949317	+	105372358	grey	0.0976718	FALSE	LOC105372358
Region_9206	chr19	31149979	31349436	199457	59	460	516.530303	24.85974175	0.824014899	0.016038079	-	57616	grey	-0.181707726	FALSE	TSHZ3
Region_9207	chr19	31167457	31225143	57686	20	129	165.0909091	14.75828557	0.831548355	0.025955727	+	107985312	grey	-0.026361773	FALSE	LINC01791
Region_9208	chr19	31348881	31417794	68913	18	122	152.6969697	12.62716205	0.624490376	0.0302164	+	107985311	grey	-0.114576571	FALSE	TSHZ3-AS1
Region_9209	chr19	31520155	31554803	34648	4	20	30.51515152	5.224917884	0.918836912	0.051638338	+	105372360	grey	0.092512124	FALSE	LINC02841
Region_9210	chr19	31797666	32072113	274447	50	351	402.469697	24.32107013	0.860890233	0.025395768	-	105372363	grey	0.272178724	FALSE	LINC01837
Region_9211	chr19	32025808	32049055	23247	4	15	25.43939394	4.614788229	0.682830445	0.070017816	+	100996665	grey	-0.195574896	FALSE	LINC01533
Region_9212	chr1	32025898	32073809	47911	11	75	96.1515151	8.70939797	0.79842176	0.03701502	+	10537689	grey	0.005419508	FALSE	LOC105376899

	9						5	3	9	3		9				
Region_9213	chr19	32206057	32222484	16427	3	19	32.03030303	5.566298859	0.846088385	0.092607566	+	107985270	grey	-0.243965558	FALSE	
Region_9214	chr19	32345594	32387667	42073	5	34	47.3030303	6.397041857	0.964353127	0.02830248	+	22847	grey	0.11027883	FALSE	ZNF507
Region_9215	chr19	32390050	32405560	15510	5	24	42.98484848	8.407857907	0.360819304	0.030110368	-	400684	grey	-0.093580757	FALSE	DPY19L3-DT
Region_9216	chr19	32405543	32485895	80352	25	183	232.2121212	16.85459549	0.929505407	0.017132412	+	147991	grey	-0.286453732	FALSE	DPY19L3
Region_9217	chr19	32581190	32587453	6263	3	16	27.5	4.595147943	0.4344474	0.053466343	+	9141	grey	-0.141974336	FALSE	PDCD5
Region_9218	chr19	32597006	32676597	79591	37	257	301.2272727	19.76850288	0.781252193	0.016816653	-	84079	grey	-0.309778613	FALSE	ANKRD27
Region_9219	chr19	32691821	32713792	21971	4	21	30.92424242	4.753332778	0.660157043	0.080876749	+	390916	grey	0.105891961	FALSE	NUDT19
Region_9220	chr19	32718298	32719595	1297	3	13	24.78787879	5.234545463	0.944838032	0.054210976	-	105372368	grey	0.126307648	FALSE	
Region_9221	chr19	32719753	32829580	109827	40	289	330.4545455	19.36625282	0.860450581	0.024524154	+	91646	grey	-0.113283727	FALSE	TDRD12
Region_9222	chr19	32830509	32869767	39258	19	111	139.2575758	12.87849801	0.940432914	0.025312635	-	11136	grey	0.198490626	FALSE	SLC7A9
Region_9223	chr19	32875925	32971991	96066	21	140	177.8030303	14.14563776	0.86634227	0.018837215	-	84902	grey	0.061586622	FALSE	CEP89
Region_9224	chr19	32972209	32978229	6020	3	12	24.10606061	3.922827818	0.660032862	0.043076468	+	91442	grey	0.111739342	FALSE	FAAP24

Region_9225	chr19	32978592	33064888	86296	42	291	334.0757576	22.31405946	0.754045162	0.019424668	-	85415	grey	0.107531767	FALSE	RHPN2
Region_9226	chr19	33080899	33130542	49643	33	184	242.4090909	18.98580626	0.880546885	0.01971804	+	55094	grey	-0.074681895	FALSE	GPATCH1
Region_9227	chr19	33132090	33175799	43709	32	226	262.2878788	19.10599193	0.847186576	0.024608488	+	126248	grey	-0.229534479	FALSE	WDR88
Region_9228	chr19	33177603	33208864	31261	21	135	176.3939394	15.28385653	0.76616066	0.02184845	+	4037	grey	-0.10392581	FALSE	LRP3
Region_9229	chr19	33208664	33225850	17186	15	83	102.8484848	11.12203969	0.747682601	0.035852781	-	56301	grey	-0.148952	FALSE	SLC7A10
Region_9230	chr19	33299934	33302534	2600	8	42	64.78787879	8.90378511	0.072630592	0.032436728	-	1050	grey	-0.190406277	FALSE	CEBPA
Region_9231	chr19	33302857	33305054	2197	5	31	44.04545455	6.986650608	0.036512725	0.030515118	+	80054	grey	0.351870815	FALSE	CEBPA-DT
Region_9232	chr19	33373685	33382686	9001	7	25	40.77272727	7.435456542	0.281193848	0.031586114	+	1054	grey	0.053191669	FALSE	CEBPG
Region_9233	chr19	33386950	33521823	134873	80	589	648.3939394	31.86407613	0.887549142	0.016526363	-	5184	grey	0.091175346	FALSE	PEPD
Region_9234	chr19	33621953	33773509	151556	136	1058	1153.727273	50.40895691	0.715318501	0.011599932	+	64377	grey	0.270233162	FALSE	CHST8
Region_9235	chr19	33795933	33815763	19830	11	57	77.92424242	9.103356281	0.700258604	0.04431723	+	79047	grey	-0.226708649	FALSE	KCTD15
Region_9236	chr19	33997289	34066283	68994	11	72	93.72727273	9.31094551	0.718673948	0.041622026	-	105372371	grey	0.326244258	FALSE	LOC105372371
Region_9237	chr1	34396315	34426168	29853	15	94	116.636363	11.4228332	0.71950821	0.02128916	+	84306	grey	-0.08997935	FALSE	PDCD2L

	9						6	2	4	9				3		
Region_9238	chr19	34428352	34471251	42899	16	99	116.7878788	10.72526156	0.806077857	0.016270483	+	10054	grey	0.047803339	FALSE	UBA2
Region_9239	chr19	34481758	34512304	30546	7	31	49.74242424	7.093465262	0.813395902	0.034012751	+	126374	grey	-0.123321077	FALSE	WTIP
Region_9240	chr19	34590644	34677159	86515	21	154	174.9242424	11.18833267	0.822764044	0.037630751	-	284402	grey	0.532765294	FALSE	SCGB2B2
Region_9241	chr19	34758073	34773229	15156	6	33	54.04545455	7.718971271	0.470247485	0.037356766	-	148103	grey	-0.073638239	FALSE	ZNF599
Region_9242	chr19	34788527	34832869	44342	6	37	54.65151515	6.995586354	0.910514661	0.048144251	-	400685	grey	-0.373152146	FALSE	LINC01801
Region_9243	chr19	34891605	34905139	13534	12	82	103.7575758	12.36877036	0.876799202	0.032132145	-	105372377	grey	0.098969326	FALSE	
Region_9244	chr19	34891713	34905107	13394	12	82	103.7575758	12.36877036	0.876799202	0.032132145	-	100652909	grey	0.098969326	FALSE	LINC00904
Region_9245	chr19	34926903	34945170	18267	3	18	32.40909091	5.449000678	0.651168118	0.029080206	+	90075	grey	-0.010434654	FALSE	ZNF30
Region_9246	chr19	34956354	34964229	7875	6	34	54.96969697	8.093624646	0.529711125	0.032620518	-	126375	grey	-0.048248981	FALSE	ZNF792
Region_9247	chr19	34994784	35026471	31687	19	110	137.8939394	11.32747346	0.804761212	0.028635154	+	57655	grey	-0.051621136	FALSE	GRAMD1A
Region_9248	chr19	35030470	35040449	9979	7	30	47.03030303	7.166094735	0.828752274	0.055838295	+	6324	grey	-0.038592728	FALSE	SCN1B
Region_9249	chr19	35040506	35066573	26067	7	36	47.98484848	7.104694925	0.68041369	0.03615008	+	3249	grey	-0.096880288	FALSE	HPN

Region_9250	chr19	35042902	35106307	63405	22	133	162.7424242	12.77053171	0.850000046	0.0236981	-	100128675	grey	0.076633941	FALSE	HPN-AS1
Region_9251	chr19	35124513	35142451	17938	7	37	51.66666667	6.891094216	0.690098627	0.064919993	-	163175	grey	-0.027297375	FALSE	LGI4
Region_9252	chr19	35154730	35169881	15151	7	34	51.5	6.712216188	0.613445456	0.04628255	+	53827	grey	0.085851954	FALSE	FXYD5
Region_9253	chr19	35224800	35228739	3939	7	25	42.6969697	8.08982209	0.728446483	0.094395861	-	148109	grey	0.083348279	FALSE	FAM187B
Region_9254	chr19	35248330	35267964	19634	4	17	32.22727273	6.025064199	0.708458992	0.03848055	+	51599	grey	0.098830683	FALSE	LSR
Region_9255	chr19	35268962	35279821	10859	5	16	25.92424242	4.871615136	0.86352089	0.081389062	+	7392	grey	-0.272249199	FALSE	USF2
Region_9256	chr19	35292125	35313807	21682	5	18	31.72727273	5.310498903	0.580928381	0.089189922	+	4099	grey	0.081445512	FALSE	MAG
Region_9257	chr19	35319261	35347361	28100	10	60	82.09090909	9.377103494	0.710985628	0.04041912	+	933	grey	0.011486095	FALSE	CD22
Region_9258	chr19	35347902	35353864	5962	3	10	18.65151515	5.353625441	0.949067333	0.055914934	+	2864	grey	0.086725543	FALSE	FFAR1
Region_9259	chr19	35399511	35419385	19874	6	36	51.16666667	6.696918541	0.941890154	0.043673774	+	100128682	grey	0.181001972	FALSE	LINC01531
Region_9260	chr19	35424062	35434642	10580	5	32	44.86363636	6.226213477	0.90633657	0.069026437	-	101927522	grey	-0.076223206	FALSE	LOC101927522
Region_9261	chr19	35487324	35495558	8234	7	37	54.81818182	7.264050768	0.777941845	0.043048535	-	388533	grey	-0.046011029	FALSE	KRTDAP
Region_9262	chr1	35497220	35513658	16438	15	74	92.9545454	10.1016858	0.69721053	0.03375251	-	93099	grey	-0.06787757	FALSE	DMKN

	9						5		4	5				9		
Region_9263	chr19	35523367	35528351	4984	8	37	49.84848485	7.591984486	0.783179709	0.082463783	-	374897	grey	0.061808859	FALSE	SBSN
Region_9264	chr19	35533455	35545319	11864	10	63	79.22727273	7.783207673	0.564398759	0.03173153	+	26330	grey	-0.045897971	FALSE	GAPDHS
Region_9265	chr19	35540738	35546029	5291	7	34	50.77272727	8.116072287	0.148568827	0.015261645	-	100506469	grey	0.005646364	FALSE	TMEM147-AS1
Region_9266	chr19	35550031	35563658	13627	16	86	113.4242424	11.21682885	0.623056056	0.034340501	-	495	grey	0.030113537	FALSE	ATP4A
Region_9267	chr19	35596581	35601596	5015	4	16	25.63636364	4.484627979	0.76444492	0.076379286	+	102723617	grey	-0.077862001	FALSE	LINC01766
Region_9268	chr19	35612735	35625355	12620	11	70	92.37878788	10.34298744	0.799454632	0.029142816	+	23354	grey	0.084289658	FALSE	HAUS5
Region_9269	chr19	35629030	35637686	8656	14	57	92.87878788	14.08492391	0.853643708	0.031708036	+	79171	grey	0.165064806	FALSE	RBM42
Region_9270	chr19	35641745	35644871	3126	4	21	32.74242424	6.879871658	0.205406644	0.079002742	+	2116	grey	-0.127981889	FALSE	ETV2
Region_9271	chr19	35648323	35658782	10459	4	31	44.60606061	5.707966869	0.310328855	0.056766434	+	1340	grey	0.07802565	FALSE	COX6B1
Region_9272	chr19	35666517	35678481	11964	6	23	42.09090909	8.831285772	0.944698038	0.037245137	+	11045	grey	-0.19515649	FALSE	UPK1A
Region_9273	chr19	35704558	35717038	12480	8	39	51.89393939	6.861551266	0.669146958	0.050466271	+	27033	grey	-0.293320119	FALSE	ZBTB32
Region_9274	chr19	35717973	35738880	20907	17	84	109.2727273	12.01095071	0.750137596	0.034061046	+	9757	grey	-0.112759978	FALSE	KMT2B

Region_9275	chr19	35738801	35745432	6631	16	82	111.7727273	12.32358022	0.509137826	0.022528052	-	79713	grey	-0.047438763	FALSE	IGFLR1
Region_9276	chr19	35745678	35754519	8841	8	36	59.12121212	7.999067545	0.601871191	0.032462506	+	55957	grey	0.164444418	FALSE	LIN37
Region_9277	chr19	35754566	35758079	3513	5	30	41.21212121	6.566851843	0.251164545	0.042538061	-	126393	grey	0.207850029	FALSE	HSPB6
Region_9278	chr19	35758143	35771166	13023	25	144	183.5454545	15.19127464	0.701873753	0.030563763	+	148137	grey	-0.289002256	FALSE	PROSER3
Region_9279	chr19	35774532	35788822	14290	18	70	98.75757576	10.60052336	0.853707032	0.023999559	+	115703	grey	-0.253275695	FALSE	ARHGAP33
Region_9280	chr19	35785697	35797935	12238	18	100	129.5757576	12.74369902	0.858282189	0.027364015	-	644050	grey	-0.042367617	FALSE	LINC01529
Region_9281	chr19	35799988	35813299	13311	4	20	34.3030303	5.694720084	0.694927276	0.060016236	-	58510	grey	0.038743329	FALSE	PRODH2
Region_9282	chr19	35825372	35852504	27132	15	80	102.5454545	9.413871628	0.729601032	0.04289327	-	4868	grey	0.026874225	FALSE	NPHS1
Region_9283	chr19	35851651	35869287	17636	12	65	84.1969697	9.796733674	0.740849297	0.039926172	-	107985317	grey	0.216175957	FALSE	LOC107985317
Region_9284	chr19	35855861	35867136	11275	7	34	47.65151515	7.492544857	0.768741451	0.049684641	+	84063	grey	0.331642629	FALSE	KIRREL2
Region_9285	chr19	35867899	35879792	11893	7	28	39.48484848	6.388071634	0.741025552	0.050600881	+	333	grey	-0.092380696	FALSE	APLP1
Region_9286	chr19	35887653	35902303	14650	9	47	59.1969697	6.404853201	0.689919219	0.028512107	-	84807	grey	0.016431594	FALSE	NFKBID
Region_9287	chr1	35902529	35904377	1848	3	16	24.8787878	5.20802679	0.07829283	0.05535864	+	10870	grey	-0.20369062	FALSE	HCST

	9						8	8		8				7		
Region_9288	chr19	35935550	35946624	11074	10	45	62.33333333	8.586811593	0.783148715	0.026466339	+	79414	grey	0.048041833	FALSE	LRFN3
Region_9289	chr19	35947115	35964063	16948	14	81	105.7727273	10.85965781	0.689889433	0.035547519	+	105372383	grey	0.074288518	FALSE	LOC105372383
Region_9290	chr19	36003307	36008813	5506	4	18	32.60606061	6.836280563	0.98217539	0.022593608	-	163183	grey	-0.139997176	FALSE	SYNE4
Region_9291	chr19	36008638	36014239	5601	5	14	27.06060606	5.812132636	0.398006012	0.041091278	-	84964	grey	-0.146241292	FALSE	ALKBH6
Region_9292	chr19	36014493	36045972	31479	30	206	230.9090909	16.53037044	0.88479736	0.01759198	+	101927572	grey	-0.100935418	FALSE	LOC101927572
Region_9293	chr19	36014660	36033343	18683	20	120	145.3333333	11.36571543	0.913551074	0.022392502	-	25999	grey	-0.151760062	FALSE	CLIP3
Region_9294	chr19	36034984	36054739	19755	21	122	169.2878788	13.48932929	0.770135182	0.023810751	-	199745	grey	-0.091279347	FALSE	THAP8
Region_9295	chr19	36054649	36105108	50459	36	228	267.1666667	16.89568296	0.890811443	0.018560953	+	284403	grey	0.004963278	FALSE	WDR62
Region_9296	chr19	36115439	36125947	10508	8	31	45.8030303	8.16035387	0.944717444	0.030808178	+	1155	grey	0.173213153	FALSE	TBCB
Region_9297	chr19	36139953	36150353	10400	11	51	79.09090909	10.42294403	0.794345268	0.02278288	+	826	grey	0.12233114	FALSE	CAPNS1
Region_9298	chr19	36182276	36246257	63981	69	478	559.1363636	33.31891879	0.772677991	0.012089093	-	147929	grey	-0.131903005	FALSE	ZNF565
Region_9299	chr19	36214602	36238771	24169	26	148	194.6212121	19.45943722	0.711665144	0.01772943	+	7705	grey	-0.083361428	FALSE	ZNF146

Region_9300	chr19	36257662	36321276	63614	42	241	410.9848485	83.50375255	0.896339642	0.02520066	+	100134317	grey	0.058589767	FALSE	LOC100134317
Region_9301	chr19	36262260	36331770	69510	42	255	419.3484848	83.35992256	0.876284656	0.024087676	-	100506930	grey	0.061905432	FALSE	LINC00665
Region_9302	chr19	36334453	36379201	44748	38	199	278.6212121	31.04625093	0.904409343	0.021013501	-	57677	grey	-0.344097499	FALSE	ZFP14
Region_9303	chr19	36383120	36418644	35524	13	82	104.3939394	9.063670148	0.943056751	0.034639537	-	284406	grey	-0.177865822	FALSE	ZFP82
Region_9304	chr19	36421536	36422534	998	3	20	29.33333333	5.030165415	0.088362168	0.063012445	+	644189	grey	-0.140648895	FALSE	LOC644189
Region_9305	chr19	36445119	36489902	44783	23	131	163.4090909	13.7067948	0.968879598	0.016179328	-	84924	grey	-0.026006351	FALSE	ZNF566
Region_9306	chr19	36510687	36528271	17584	8	44	66.06060606	8.414051961	0.965752826	0.019789818	-	339324	grey	0.114183757	FALSE	ZNF260
Region_9307	chr19	36534774	36605276	70502	39	272	303.0909091	15.67921434	0.747881071	0.012972754	-	57711	grey	-0.058421275	FALSE	ZNF529
Region_9308	chr19	36573070	36594712	21642	11	62	80.09090909	8.331085944	0.714957448	0.02919602	+	101927599	grey	0.037518387	FALSE	ZNF529-AS1
Region_9309	chr19	36604817	36634114	29297	22	144	191.7121212	15.93908129	0.569139457	0.015016102	+	84911	grey	0.267631556	FALSE	ZNF382
Region_9310	chr19	36636618	36666853	30235	11	71	92.06060606	9.21683875	0.875867099	0.019444328	-	92283	grey	0.219315633	FALSE	ZNF461
Region_9311	chr19	36682636	36687468	4832	5	27	38.53030303	6.246770728	0.289928342	0.049467184	-	101927621	grey	-0.116282504	FALSE	ZNF567-DT
Region_9312	chr1	36687612	36727701	40089	17	102	137.5	13.1163433	0.94565783	0.02125100	+	163081	grey	0.219985931	FALSE	ZNF567

	9								6	6						
Region_9313	chr19	36714383	36772825	58442	59	435	492.8787879	23.77491577	0.763004081	0.012882166	-	342892	grey	-0.006506458	FALSE	ZNF850
Region_9314	chr19	36773100	36777078	3978	20	113	145.1363636	14.63703035	0.765968563	0.061990555	+	728485	grey	-0.127219849	FALSE	LOC728485
Region_9315	chr19	36797501	36831596	34095	24	172	215.1969697	17.0006513	0.727507058	0.011858352	+	284408	grey	-0.025682742	FALSE	ZNF790-AS1
Region_9316	chr19	36817428	36850787	33359	14	89	109.7121212	10.14474499	0.760478178	0.017939671	-	388536	grey	-0.042037874	FALSE	ZNF790
Region_9317	chr19	36850361	36913029	62668	24	164	189.5	12.21757876	0.919801532	0.01698005	+	25850	grey	0.116651906	FALSE	ZNF345
Region_9318	chr19	36888124	36916291	28167	10	56	77.43939394	9.67014245	0.951423173	0.030540896	-	374899	grey	0.036681939	FALSE	ZNF829
Region_9319	chr19	36916329	36998700	82371	35	227	267.7272727	19.69423614	0.77816814	0.031110477	+	374900	grey	-0.063729203	FALSE	ZNF568
Region_9320	chr19	37007857	37130368	122511	36	256	287.9090909	15.99492927	0.830807237	0.022339064	+	147923	grey	0.035083811	FALSE	ZNF420
Region_9321	chr19	37093019	37207254	114235	36	246	289.4393939	18.78225741	0.965975972	0.013179138	-	199704	grey	0.198001016	FALSE	ZNF585A
Region_9322	chr19	37156331	37218153	61822	23	142	187.469697	15.82910629	0.8876814	0.012289417	-	92285	grey	0.145293938	FALSE	ZNF585B
Region_9323	chr19	37217926	37248740	30814	9	67	93.28787879	10.10371623	0.658364428	0.01562583	+	163087	grey	0.134426131	FALSE	ZNF383
Region_9324	chr19	37235507	37304395	68888	40	294	493.1060606	89.28780239	0.872775089	0.025614437	-	284412	grey	-0.151808488	FALSE	LOC284412

Region_9325	chr19	37251867	37265547	13680	8	40	60.95454545	8.447907011	0.875481815	0.067684902	+	101927667	grey	-0.148483449	FALSE	LINC01535
Region_9326	chr19	37312837	37369365	56528	18	125	149.3181818	12.41978458	0.836050821	0.020582019	+	284459	grey	0.212426363	FALSE	ZNF875
Region_9327	chr19	37411155	37469275	58120	11	83	108.0606061	10.8314056	0.845246224	0.037883402	-	148266	grey	0.137547662	FALSE	ZNF569
Region_9328	chr19	37467585	37488652	21067	8	47	61.3030303	7.123028088	0.81317094	0.030329349	+	148268	grey	0.359953461	FALSE	ZNF570
Region_9329	chr19	37497159	37507122	9963	5	25	39.8030303	5.904938473	0.772787146	0.038345208	-	101927720	grey	-0.037951273	FALSE	ZNF793-AS1
Region_9330	chr19	37506939	37548762	41823	13	93	114.1212121	10.06366679	0.862075447	0.025316601	+	390927	grey	-0.21005255	FALSE	ZNF793
Region_9331	chr19	37548914	37587348	38434	11	66	88.84848485	9.331901322	0.609988158	0.036200906	+	100507433	grey	-0.171152301	FALSE	ZNF571-AS1
Region_9332	chr19	37551406	37614179	62773	5	29	46.10606061	6.825582619	0.587203835	0.028905849	+	163255	grey	-0.02874405	FALSE	ZNF540
Region_9333	chr19	37613749	37656261	42512	8	35	61.3030303	9.253639113	0.705510257	0.037700832	-	22835	grey	0.112409664	FALSE	ZFP30
Region_9334	chr19	37647813	37692337	44524	10	52	76.71212121	9.29146035	0.776272988	0.019700518	-	163115	grey	-0.23423365	FALSE	ZNF781
Region_9335	chr19	37689865	37719761	29896	11	79	97.53030303	9.47095917	0.795943321	0.019424847	-	84775	grey	0.002924288	FALSE	ZNF607
Region_9336	chr19	37738287	37817300	79013	118	831	926.7878788	40.17060355	0.838513099	0.009209531	-	126231	grey	0.117242658	FALSE	ZNF573
Region_9337	chr1	37792570	37817292	24722	14	102	128	11.2660142	0.94272808	0.02423897	-	10537239	grey	0.044611246	FALSE	LOC105372394

	9							4	4	5		4				
Region_9338	chr19	37817332	37832160	14828	6	34	49.93939394	6.813739034	0.912769775	0.045852869	+	644554	grey	-0.143526556	FALSE	LOC644554
Region_9339	chr19	37823722	37855215	31493	8	41	58.71212121	6.715688066	0.79700446	0.054430657	-	100631378	grey	-0.238193008	FALSE	WDR87BP
Region_9340	chr19	37884823	37906677	21854	6	29	43.8030303	6.404853201	0.932905039	0.04419312	-	83889	grey	0.120050918	FALSE	WDR87
Region_9341	chr19	37907208	38208369	301161	128	901	993.0909091	39.17523836	0.896348426	0.01042842	+	23094	grey	-0.242761977	FALSE	SIPA1L3
Region_9342	chr19	38211006	38229714	18708	9	45	57.60606061	6.772975231	0.363698311	0.040384376	-	8193	grey	0.060867519	FALSE	DPF1
Region_9343	chr19	38244035	38292615	48580	35	239	287.1212121	18.28199211	0.732700898	0.019211	+	10653	grey	-0.176286067	FALSE	SPINT2
Region_9344	chr19	38251237	38256532	5295	7	35	50.22727273	7.408509566	0.421769242	0.05680696	-	94274	grey	-0.177742534	FALSE	PPP1R14A
Region_9345	chr19	38289151	38305006	15855	10	56	77.3030303	9.072307309	0.934117412	0.044000063	+	64073	grey	-0.315156774	FALSE	C19orf33
Region_9346	chr19	38303558	38317273	13715	7	19	35.92424242	6.127643809	0.939955085	0.04817302	-	90522	grey	-0.384297277	FALSE	YIF1B
Region_9347	chr19	38335775	38370943	35168	15	80	108.4242424	11.41262541	0.926320693	0.032806913	+	57828	grey	0.007404327	FALSE	CATSPERG
Region_9348	chr19	38374563	38383850	9287	5	30	42.10606061	5.804848869	0.770981872	0.029320013	+	5714	grey	0.087625747	FALSE	PSMD8
Region_9349	chr19	38384267	38388082	3815	4	11	23.78787879	5.205071939	0.159098977	0.074503331	-	199720	grey	-0.108423584	FALSE	GGN

Region_9350	chr19	38388421	38399587	11166	9	49	67.8030303	9.781017246	0.244772246	0.047118862	+	399473	grey	0.192219965	FALSE	SPRED3
Region_9351	chr19	38403093	38409088	5995	3	11	21.13636364	5.417596298	0.332667786	0.017718923	+	147965	grey	-0.026599147	FALSE	FAM98C
Region_9352	chr19	38409051	38426305	17254	16	83	113.7878788	11.67313174	0.780819096	0.024950817	-	115727	grey	-0.193753525	FALSE	RASGRP4
Region_9353	chr19	38433691	38595273	161582	94	596	674.1818182	34.0081027	0.875207842	0.012408088	+	6261	grey	-0.164469738	FALSE	RYR1
Region_9354	chr19	38526954	38528555	1601	5	26	35.89393939	4.441513214	0.729571464	0.082736234	-	107985290	grey	0.227697836	FALSE	
Region_9355	chr19	38587641	38618882	31241	23	136	170.3484848	15.07161614	0.900294831	0.019919718	-	11184	grey	0.107234236	FALSE	MAP4K1
Region_9356	chr19	38730187	38769904	39717	37	204	245.469697	17.94368944	0.825675163	0.026167334	-	147968	grey	-0.02244728	FALSE	CAPN12
Region_9357	chr19	38990714	39032785	42071	28	196	239.7878788	18.22636733	0.930821685	0.024010196	-	126433	grey	-0.013691045	FALSE	FBXO27
Region_9358	chr19	39083913	39111493	27580	18	113	137.969697	13.12070554	0.879608465	0.025664859	+	390928	grey	-0.06223092	FALSE	ACP7
Region_9359	chr19	39125770	39182816	57046	44	247	288.6212121	16.12238306	0.843116468	0.018812509	+	10298	grey	-0.235840391	FALSE	PAK4
Region_9360	chr19	39246314	39248856	2542	3	10	18.74242424	4.442142955	0.715976476	0.131660225	-	101180976	grey	0.057281339	FALSE	IFNL4
Region_9361	chr19	39306566	39320863	14297	20	93	127.8787879	13.9609512	0.887085167	0.03184879	-	57622	grey	-0.018014912	FALSE	LRFN1
Region_9362	chr1	39328353	39342372	14019	25	151	186.651515	16.1097127	0.49608744	0.02914835	-	9535	grey	0.236648638	FALSE	GMFG

	9						2	2	2	2						
Region_9363	chr19	39342421	39385710	43289	40	221	262.0606061	18.54796592	0.66994032	0.024507787	+	55095	grey	0.178583694	FALSE	SAMD4B
Region_9364	chr19	39385629	39391154	5525	10	41	72.18181818	13.71345111	0.704022169	0.04638471	-	54623	grey	0.022780479	FALSE	PAF1
Region_9365	chr19	39391303	39400641	9338	6	27	42.13636364	6.355824376	0.83761189	0.054715577	+	55588	grey	-0.266366458	FALSE	MED29
Region_9366	chr19	39412669	39428415	15746	12	59	81.43939394	9.609496566	0.787979236	0.037073987	+	64857	grey	0.142094739	FALSE	PLEKHG2
Region_9367	chr19	39436156	39476670	40514	44	289	329.9848485	18.92291442	0.710651702	0.015154981	+	6829	grey	-0.187721692	FALSE	SUPT5H
Region_9368	chr19	39480412	39493779	13367	9	50	65.34848485	7.7330309	0.749709611	0.02463045	+	92609	grey	0.069963412	FALSE	TIMM50
Region_9369	chr19	39498895	39508481	9586	15	88	115.1060606	15.66390146	0.375241957	0.043615758	+	10683	grey	0.287839356	FALSE	DLL3
Region_9370	chr19	39515139	39520675	5536	5	14	25.40909091	5.314645289	0.400891806	0.059680023	+	348303	grey	0.253364618	FALSE	SELENOV
Region_9371	chr19	39538707	39540161	1454	5	20	36.77272727	6.681306769	0.150727729	0.037037793	-	163126	grey	0.004353277	FALSE	EID2
Region_9372	chr19	39655913	39660647	4734	4	24	35.8030303	5.180495067	0.927092775	0.04801299	+	148003	grey	-0.168977195	FALSE	LGALS16
Region_9373	chr19	39679372	39694577	15205	7	53	71.25757576	7.692775044	0.907917605	0.045531485	+	400696	grey	-0.256882899	FALSE	LGALS17A
Region_9374	chr19	40191426	40215575	24149	5	21	33.86363636	4.841761802	0.764380666	0.043717777	+	4294	grey	0.134172538	FALSE	MAP3K10

Region_9375	chr19	40222208	40226697	4489	4	16	25.01515152	5.20795966	0.265145639	0.052983598	-	79935	grey	0.238853331	FALSE	CCNP
Region_9376	chr19	40230317	40285536	55219	19	111	146.1818182	16.09004383	0.849055416	0.022448301	-	208	grey	-0.078791455	FALSE	AKT2
Region_9377	chr19	40319536	40348527	28991	22	147	183.6363636	16.11748822	0.823153253	0.017506442	-	126526	grey	-0.088583031	FALSE	C19orf47
Region_9378	chr19	40348456	40389472	41016	25	160	191.3787879	14.54622885	0.831064447	0.025351171	+	23646	grey	-0.214284537	FALSE	PLD3
Region_9379	chr19	40379271	40390181	10910	15	94	121.3484848	12.0070395	0.922307746	0.021693576	-	147746	grey	-0.122712912	FALSE	HIPK4
Region_9380	chr19	40393766	40414793	21027	24	152	179.7878788	14.2143104	0.660990577	0.026205541	-	57716	grey	0.027571496	FALSE	PRX
Region_9381	chr19	40421589	40425992	4403	4	11	18.83333333	3.601637804	0.85335598	0.10315042	-	29950	grey	-0.090458813	FALSE	SERTAD1
Region_9382	chr19	40440844	40444335	3491	3	10	22.48484848	4.598469392	0.03350757	0.039976413	-	29946	grey	0.024902866	FALSE	SERTAD3
Region_9383	chr19	40447765	40465764	17999	7	41	56.22727273	7.248969061	0.653983393	0.038523715	-	645	grey	0.259027879	FALSE	BLVRB
Region_9384	chr19	40466241	40576464	110223	42	253	305.8787879	21.89029652	0.731225841	0.021701769	+	57731	grey	0.075723227	FALSE	SPTBN4
Region_9385	chr19	40592883	40629818	36935	34	189	228.1363636	20.48405486	0.430333631	0.021064897	+	8425	grey	-0.029768354	FALSE	LTBP4
Region_9386	chr19	40665905	40690972	25067	16	124	145.9090909	10.73703479	0.937244539	0.022197463	-	9253	grey	-0.060849888	FALSE	NUMBL
Region_9387	chr1	40691514	40725784	34270	22	141	173.151515	16.8406490	0.83424106	0.02375918	-	79934	grey	-0.10646782	FALSE	COQ8B

	9						2	1	8	3				1		
Region_9388	chr19	40717112	40740860	23748	30	189	223.6969697	17.58843125	0.93592269	0.015680546	+	80271	grey	-0.012246958	FALSE	ITPKC
Region_9389	chr19	40740856	40751553	10697	11	74	100.5	11.93733639	0.530305748	0.02378226	-	284325	grey	0.245043723	FALSE	ACTMAP
Region_9390	chr19	40750637	40765389	14752	12	75	96.16666667	10.8579405	0.68318041	0.036338778	+	6626	grey	0.036175283	FALSE	SNRPA
Region_9391	chr19	40771648	40796943	25295	20	85	112.1212121	11.48847603	0.757653626	0.030194668	+	100529262	grey	-0.022277341	FALSE	MIA-RAB4B
Region_9392	chr19	40778216	40808418	30202	20	88	112.8333333	9.651756848	0.700519969	0.031600181	+	100529264	grey	0.035635118	FALSE	RAB4B-EGLN2
Region_9393	chr19	40778247	40796942	18695	16	66	87.27272727	9.505537584	0.767397954	0.035282162	+	53916	grey	-0.03710342	FALSE	RAB4B
Region_9394	chr19	40801297	40912357	111060	47	288	359.7878788	28.19193292	0.819050401	0.020397602	+	22952	grey	0.009849306	FALSE	CYP2G1P
Region_9395	chr19	40808474	40812100	3626	4	20	30.95454545	5.690563898	0.921356168	0.053987561	-	171523	grey	0.157315836	FALSE	CYP2T1P
Region_9396	chr19	40875439	40882752	7313	7	21	41.81818182	10.32384706	0.465147799	0.082284815	-	1549	grey	0.129838603	FALSE	CYP2A7
Region_9397	chr19	40924219	40950676	26457	17	105	128.7878788	10.09876634	0.820087289	0.041241385	+	1556	grey	-0.00674617	FALSE	CYP2B7P
Region_9398	chr19	40991282	41018398	27116	11	70	91.77272727	9.42793795	0.80337709	0.03538101	+	1555	grey	-0.1081278	FALSE	CYP2B6
Region_9399	chr19	41088451	41096195	7744	6	30	41.48484848	5.280566474	0.693946982	0.07846161	+	1553	grey	0.10101182	FALSE	CYP2A13

Region_9400	chr19	41114432	41128381	13949	11	70	89.57575758	8.69670248	0.636272906	0.026400897	+	1572	grey	-0.018598385	FALSE	CYP2F1
Region_9401	chr19	41193210	41207539	14329	13	72	91.63636364	9.795817575	0.81568893	0.027887729	+	29785	grey	-0.24118478	FALSE	CYP2S1
Region_9402	chr19	41219223	41261766	42543	37	235	279.9848485	20.39343214	0.801813687	0.019189812	+	558	grey	0.014811954	FALSE	AXL
Region_9403	chr19	41262496	41307787	45291	35	257	293.1666667	16.89750399	0.774064069	0.016171499	+	11100	grey	0.2044362	FALSE	HNRNPUL1
Region_9404	chr19	41301587	41353922	52335	38	250	280.4090909	16.59284116	0.679354789	0.021172453	-	7040	grey	-0.129414519	FALSE	TGFB1
Region_9405	chr19	41310172	41324873	14701	10	61	74.60606061	7.053376252	0.808340039	0.033579919	+	90324	grey	-0.066119904	FALSE	CCDC97
Region_9406	chr19	41350853	41425002	74149	46	272	327.9848485	22.73424555	0.678979338	0.015717855	+	593	grey	-0.123197167	FALSE	BCKDHA
Region_9407	chr19	41350911	41384083	33172	25	117	171.1363636	16.43812301	0.559414073	0.019945957	+	641649	grey	-0.331608985	FALSE	TMEM91
Region_9408	chr19	41354417	41364165	9748	7	32	50	7.988453205	0.915072578	0.054432987	-	80776	grey	-0.10660097	FALSE	B9D2
Region_9409	chr19	41386371	41397362	10991	4	12	24.07575758	5.863342722	0.785668372	0.078483524	-	56915	grey	0.107840462	FALSE	EXOSC5
Region_9410	chr19	41431318	41440717	9399	5	28	43.46969697	5.370058579	0.900745065	0.043415696	-	55101	grey	-0.194721593	FALSE	DMAC2
Region_9411	chr19	41708585	41756737	48152	19	94	138.4090909	14.29575222	0.84368796	0.025060019	+	1048	grey	-0.156493247	FALSE	CEACAM5
Region_9412	chr1	41750977	41772211	21234	12	61	80.8939393	9.82011873	0.56916011	0.06985166	+	4680	grey	0.166400668	FALSE	CEACAM6

	9						9	3	4	5						
Region_9413	chr19	41757898	41786893	28995	15	93	114.1818182	9.185120226	0.645147948	0.047435103	-	112268252	grey	0.234961365	FALSE	LOC112268252
Region_9414	chr19	41796587	41811554	14967	5	20	34.01515152	5.453276853	0.657335976	0.05638648	+	1084	grey	0.078871655	FALSE	CEACAM3
Region_9415	chr19	41837074	41844697	7623	6	33	44.42424242	5.349509269	0.903679475	0.043259392	-	147719	grey	-0.384452318	FALSE	LYPD4
Region_9416	chr19	41844743	41852333	7590	6	28	41.62121212	5.835607282	0.888871356	0.072997783	+	63946	grey	-0.076953078	FALSE	DMRTC2
Region_9417	chr19	41860255	41872925	12670	12	67	90.18181818	10.83002819	0.668802406	0.038008044	+	6223	grey	-0.213086395	FALSE	RPS19
Region_9418	chr19	41883173	41930150	46977	37	208	256.9545455	18.62127139	0.668832861	0.017713716	+	9138	grey	0.319377606	FALSE	ARHGEF1
Region_9419	chr19	41907704	41928449	20745	14	81	105.4090909	11.31231769	0.582357629	0.037451775	-	390937	grey	0.051075748	FALSE	ERFL
Region_9420	chr19	41956879	41997497	40618	15	87	110.9545455	10.80944291	0.85674014	0.031263197	-	478	grey	-0.031894855	FALSE	ATP1A3
Region_9421	chr19	41998321	42070206	71885	27	169	199.6666667	15.78054628	0.81502982	0.02668074	-	2901	grey	-0.04870995	FALSE	GRIK5
Region_9422	chr19	42086110	42196585	110475	23	145	170.7272727	12.34945711	0.783779641	0.025393791	-	5452	grey	-0.037929759	FALSE	POU2F2
Region_9423	chr19	42198598	42232149	33551	14	75	99.25757576	10.01124077	0.5337278	0.020684195	-	162989	grey	0.198331867	FALSE	DEDD2
Region_9424	chr19	42220312	42228201	7889	6	29	44.12121212	7.416229918	0.64981101	0.027025721	+	116115	grey	0.196568364	FALSE	ZNF526

Region_9425	chr19	42226225	42255132	28907	13	57	77.95454545	9.820593462	0.513103039	0.040982416	-	2931	grey	0.099065011	FALSE	GSK3A
Region_9426	chr19	42247569	42255128	7559	8	26	42.24242424	6.996835782	0.420326403	0.057809606	-	2077	grey	0.070709593	FALSE	ERF
Region_9427	chr19	42268537	42295797	27260	16	70	98.90909091	10.51259651	0.526922163	0.026893896	+	23152	grey	0.046249371	FALSE	CIC
Region_9428	chr19	42302098	42310814	8716	8	37	53.1969697	7.164484408	0.959609558	0.03785328	+	284338	grey	0.135722086	FALSE	PRR19
Region_9429	chr19	42313309	42325064	11755	9	44	61.60606061	8.041964528	0.536076103	0.05169558	+	284339	grey	0.193098111	FALSE	TMEM145
Region_9430	chr19	42325609	42378769	53160	28	166	196.6818182	15.23069683	0.880785513	0.023151772	+	1954	grey	0.079265073	FALSE	MEGF8
Region_9431	chr19	42387019	42390297	3278	7	32	44.43939394	6.996985698	0.408952027	0.030388222	-	84518	grey	0.046360428	FALSE	CNFN
Region_9432	chr19	42397114	42408452	11338	6	29	42.6969697	7.144593861	0.484765837	0.047741736	+	101930071	grey	-0.188108326	FALSE	LOC101930071
Region_9433	chr19	42397150	42652367	255217	110	794	894.530303	41.90032851	0.842205063	0.012592547	+	100996307	grey	-0.120135256	FALSE	LIPE-AS1
Region_9434	chr19	42401514	42427388	25874	27	183	227	18.7485384	0.684965788	0.018240645	-	3991	grey	-0.168775915	FALSE	LIPE
Region_9435	chr19	42428278	42442946	14668	11	66	81.24242424	8.571976894	0.933458996	0.034854246	-	284340	grey	-0.115696082	FALSE	CXCL17
Region_9436	chr19	42507304	42561234	53930	16	113	131.9393939	11.4083354	0.945774691	0.031612692	-	634	grey	-0.097567509	FALSE	CEACAM1
Region_9437	chr1	42580243	42595055	14812	4	22	34.2575757	7.21389813	0.95763105	0.04009708	-	1088	grey	-0.20501333	FALSE	CEACAM8

	9						6	8	3					5		
Region_9438	chr19	42721638	42740481	18843	3	18	26.75757576	4.832774664	0.69789601	0.102399987	-	5671	grey	0.270406592	FALSE	PSG3
Region_9439	chr19	42752686	42855691	103005	33	195	280.3484848	44.96476994	0.844941892	0.033059272	-	440533	grey	0.083717147	FALSE	PSG8
Region_9440	chr19	42866464	42879822	13358	7	31	51.37878788	7.621356939	0.846559048	0.066226469	-	5669	grey	0.084705096	FALSE	PSG1
Region_9441	chr19	42902086	42919563	17477	7	25	52.09090909	9.816355697	0.742123233	0.067498592	-	5675	grey	0.038126103	FALSE	PSG6
Region_9442	chr19	42977463	43171515	194052	25	160	212.1969697	19.05475487	0.852996614	0.026635611	+	107985349	grey	-0.150453923	FALSE	
Region_9443	chr19	43007656	43026474	18818	3	15	31.04545455	5.816242028	0.876819004	0.056609099	-	5680	grey	-0.105987944	FALSE	PSG11
Region_9444	chr19	43064209	43083045	18836	3	17	27.8030303	5.227794643	0.707001384	0.077550769	-	5670	grey	-0.066243761	FALSE	PSG2
Region_9445	chr19	43166256	43186536	20280	3	14	22.40909091	4.328713693	0.879824759	0.082930991	-	5673	grey	0.360195207	FALSE	PSG5
Region_9446	chr19	43192702	43207299	14597	4	17	33.51515152	7.648514032	0.787343266	0.138832694	-	5672	grey	0.044088101	FALSE	PSG4
Region_9447	chr19	43211791	43260746	48955	7	37	49.51515152	6.682335898	0.896131466	0.052491354	-	284344	grey	0.00406628	FALSE	LOC284344
Region_9448	chr19	43211791	43269530	57739	7	37	49.51515152	6.682335898	0.896131466	0.052491354	-	5678	grey	0.00406628	FALSE	PSG9
Region_9449	chr19	43401496	43418597	17101	3	16	23.75757576	4.271769967	0.974365526	0.034012064	+	83639	grey	0.176128321	FALSE	TEX101

Region_9450	chr19	43474954	43504935	29981	7	38	56.65151515	7.991326884	0.689598895	0.024363047	-	653583	grey	-0.076942877	FALSE	PHLDB3
Region_9451	chr19	43543311	43593036	49725	10	46	78.12121212	9.518427717	0.924605075	0.034191852	-	7515	grey	-0.12263226	FALSE	XRCC1
Region_9452	chr19	43584367	43596134	11767	4	18	34.87878788	6.217877826	0.938590631	0.039789656	-	126298	grey	-0.099421753	FALSE	IRGQ
Region_9453	chr19	43596392	43614497	18105	5	25	39.89393939	5.812794344	0.768188016	0.041633502	+	79177	grey	0.091461376	FALSE	ZNF576
Region_9454	chr19	43622368	43639850	17482	8	39	59.56060606	9.593473403	0.481992345	0.046414515	-	199731	grey	0.250355408	FALSE	CADM4
Region_9455	chr19	43646095	43670547	24452	5	36	50.18181818	5.790998561	0.962011421	0.031700055	-	5329	grey	0.112931555	FALSE	PLAUR
Region_9456	chr19	43766533	43780976	14443	6	30	43.04545455	6.902467381	0.527660024	0.093278103	-	3783	grey	0.215915246	FALSE	KCNN4
Region_9457	chr19	43785874	43827199	41325	8	56	74.33333333	7.732714387	0.454342529	0.043936621	-	284348	grey	-0.094813179	FALSE	LYPD5
Region_15068	chr5	140794852	141012347	217495	131	1011	1247.742424	89.60190771	0.522639666	0.028552113	+	56146	blue	0.921066016	FALSE	PCDHA2
Region_9459	chr19	43901866	43927767	25901	7	35	46.45454545	6.689099786	0.966861163	0.03069149	+	118827818	grey	0.036231205	FALSE	ZNF45-AS1
Region_9460	chr19	43912624	43935282	22658	3	16	21.6969697	3.941767261	0.975038149	0.034827307	-	7596	grey	-0.01369656	FALSE	ZNF45
Region_9461	chr19	43951223	43967709	16486	6	27	44.66666667	8.171871706	0.649941852	0.066891905	+	7638	grey	-0.085254556	FALSE	ZNF221
Region_9462	chr1	43967862	43998326	30464	11	83	105.772727	9.61056382	0.87382854	0.02177669	+	7711	grey	0.066726321	FALSE	ZNF155

	9						3	6	3	1						
Region_9463	chr1 9	44025342	44033110	7768	5	32	52	7.28539107 2	0.72754885 7	0.03741419	+	7673	grey	0.076037116	FALSE	ZNF222
Region_9464	chr1 9	44025354	44089613	64259	30	214	247.227272 7	14.2221879 2	0.81963284 6	0.01585594	+	342909	grey	-0.04552787 6	FALSE	ZNF284
Region_9465	chr1 9	44051367	44067999	16632	6	29	40.2727272 7	6.02697897 2	0.97023719 1	0.02936407 6	+	7766	grey	-0.22449626 2	FALSE	ZNF223
Region_9466	chr1 9	44094361	44109886	15525	6	29	42.0606060 6	5.74021920 7	0.80883896 5	0.03042256 7	+	7767	grey	-0.02645214	FALSE	ZNF224
Region_9467	chr1 9	44103007	44113183	10176	3	14	21.6363636 4	4.20489225 6	0.65319227 6	0.02403851 4	-	10037922 4	grey	0.178077704	FALSE	ZNF225-AS1
Region_9468	chr1 9	44112181	44134822	22641	11	71	89.9696969 7	8.98026845 1	0.7030109	0.01910749 9	+	7768	grey	-0.10291658 4	FALSE	ZNF225
Region_9469	chr1 9	44141554	44160313	18759	11	58	83.3787878 8	8.82438082 6	0.88089316 7	0.02068122 9	+	10780	grey	-0.04811771 2	FALSE	ZNF234
Region_9470	chr1 9	44165073	44178381	13308	7	44	63.8939393 9	6.72796411 7	0.56738443 4	0.01839536 1	+	7769	grey	0.096452055	FALSE	ZNF226
Region_9471	chr1 9	44207547	44237268	29721	13	81	108.666666 7	13.7374651 5	0.52222647 8	0.01811540 5	+	7770	grey	-0.11715272 6	FALSE	ZNF227
Region_9472	chr1 9	44228729	44305046	76317	20	138	170.575757 6	14.1661140 7	0.72566220 9	0.02501445 1	-	9310	grey	-0.07580830 7	FALSE	ZNF235
Region_9473	chr1 9	44259880	44275317	15437	10	61	89.2424242 4	11.5639500 9	0.5076936	0.02997116 6	+	353355	grey	-0.21674985 4	FALSE	ZNF233
Region_9474	chr1 9	44326555	44401608	75053	13	74	96.9242424 2	11.1041372 8	0.84233859 9	0.03612321 1	-	7771	grey	0.159345406	FALSE	ZNF112

Region_9475	chr19	44392439	44448578	56139	13	63	92.28787879	10.73422299	0.847804063	0.029979331	-	7772	grey	0.1997465	FALSE	ZNF229
Region_9476	chr19	44474428	44500524	26096	6	26	38.92424242	7.008369954	0.86081836	0.092993579	-	7733	grey	-0.08810802	FALSE	ZNF180
Region_9477	chr19	44501677	44529788	28111	4	17	30.51515152	5.580853048	0.854459504	0.065744877	-	125931	grey	-0.356238551	FALSE	CEACAM20
Region_9478	chr19	44537058	44620821	83763	34	228	269.5757576	16.52881922	0.875042791	0.023454958	-	388550	grey	0.029759784	FALSE	CEACAM22P
Region_9479	chr19	44613563	44636781	23218	11	63	85.96969697	9.68260883	0.752079037	0.046665579	+	147710	grey	0.256091941	FALSE	IGSF23
Region_9480	chr19	44631573	44725217	93644	33	234	266.3636364	15.73299948	0.806245705	0.024425397	-	107985305	grey	0.337556744	FALSE	CEACAM16-AS1
Region_9481	chr19	44643798	44666162	22364	13	76	98.03030303	9.225332509	0.78728924	0.033653094	+	5817	grey	0.271864343	FALSE	PVR
Region_9482	chr19	44699151	44710718	11567	5	23	37.90909091	5.787858041	0.904942295	0.044450854	+	388551	grey	-0.137721687	FALSE	CEACAM16
Region_9483	chr19	44777869	44800652	22783	7	41	53.87878788	6.395948601	0.835708133	0.035249742	+	23624	grey	-0.012915724	FALSE	CBLC
Region_9484	chr19	44809071	44821421	12350	10	48	67.54545455	9.746292105	0.855232709	0.026212547	+	4059	grey	-0.069612584	FALSE	BCAM
Region_9485	chr19	44846175	44889223	43048	9	51	68.87878788	8.136366892	0.791947177	0.048014631	+	5819	grey	0.088939073	FALSE	NECTIN2
Region_9486	chr19	44890569	44903689	13120	6	25	39.13636364	6.447149313	0.156523142	0.027751669	+	10452	grey	-0.190498634	FALSE	TOMM40
Region_9487	chr1	44914247	44919349	5102	3	11	26.0909090	6.01210899	0.97279980	0.03395788	+	341	grey	-0.04627161	FALSE	APOC1

	9						9	3	4	3						
Region_9488	chr19	44954585	44993341	38756	20	107	135.8030303	13.09870187	0.893308179	0.023252323	+	1209	grey	0.288398796	FALSE	CLPTM1
Region_9489	chr19	45001461	45038192	36731	19	116	142.3939394	12.34862657	0.930120672	0.030349021	+	5971	grey	-0.127791137	FALSE	RELB
Region_9490	chr19	45039045	45070956	31911	19	103	127.3636364	12.76971943	0.809875789	0.017032028	+	11129	grey	-0.13251836	FALSE	CLASRP
Region_9491	chr19	45076510	45092635	16125	16	87	120.8333333	10.66782846	0.442395547	0.015035243	-	105372419	grey	0.220580045	FALSE	GEMIN7-AS1
Region_9492	chr19	45079195	45091518	12323	11	51	71.51515152	7.932876914	0.460825219	0.020096608	+	79760	grey	0.223491443	FALSE	GEMIN7
Region_9493	chr19	45079288	45305284	225996	166	1113	1196.348485	45.6038097	0.775491788	0.010791151	+	57787	grey	0.133419929	FALSE	MARK4
Region_9494	chr19	45093174	45148077	54903	31	201	232.030303	16.13307133	0.837714162	0.021568026	+	284352	grey	-0.071147133	FALSE	PPP1R37
Region_9495	chr19	45149744	45160982	11238	19	119	145.5	13.02335181	0.622779294	0.028084481	-	284353	grey	0.070126184	FALSE	NKPD1
Region_9496	chr19	45162928	45178237	15309	8	43	63.04545455	7.833717691	0.794542282	0.042610668	-	79090	grey	0.073842889	FALSE	TRAPPC6A
Region_9497	chr19	45178784	45216933	38149	26	149	197.7121212	15.38607277	0.706638316	0.017308413	+	388552	grey	0.069180005	FALSE	BLOC1S3
Region_9498	chr19	45212370	45245407	33037	25	142	176.2727273	14.68389537	0.803962709	0.032291005	-	90332	grey	0.036026947	FALSE	EXOC3L2
Region_9499	chr19	45306413	45322875	16462	20	103	128.2575758	11.52296784	0.753921439	0.040097035	-	1158	grey	0.130925503	FALSE	CKM

Region_9500	chr19	45333434	45351520	18086	25	131	167.1363636	15.24051924	0.750594242	0.030997347	+	147700	grey	-0.067627464	FALSE	KLC3
Region_9501	chr19	45349837	45370918	21081	23	125	160.9090909	13.84388482	0.854599767	0.023191359	-	2068	grey	0.229295082	FALSE	ERCC2
Region_9502	chr19	45379638	45406349	26711	41	249	290.3636364	18.19013796	0.678179	0.019115425	-	10848	grey	0.249916915	FALSE	PPP1R13L
Region_9503	chr19	45406644	45410737	4093	6	25	39.07575758	6.526421109	0.32684527	0.028858101	+	10849	grey	-0.076391641	FALSE	POLR1G
Region_9504	chr19	45407334	45478828	71494	65	402	450.8181818	25.07766956	0.584323603	0.015906308	-	2067	grey	0.312110375	FALSE	ERCC1
Region_9505	chr19	45467995	45475179	7184	9	34	46.75757576	6.295445499	0.483354018	0.064597715	+	2354	grey	0.16843248	FALSE	FOSB
Region_9506	chr19	45485294	45497055	11761	18	89	126.8181818	14.35796117	0.606572301	0.025464603	-	6253	grey	-0.042686127	FALSE	RTN2
Region_9507	chr19	45488777	45502513	13736	21	125	160.5909091	16.23574146	0.470636559	0.025539393	+	147699	grey	-0.076500951	FALSE	PPM1N
Region_9508	chr19	45506579	45526983	20404	20	97	125.3333333	11.41164497	0.453683811	0.026103866	+	7408	grey	0.187755414	FALSE	VASP
Region_9509	chr19	45527767	45602212	74445	84	604	677.5454545	36.01460465	0.708401166	0.014111448	-	80207	grey	-0.083578673	FALSE	OPA3
Region_9510	chr19	45589764	45602212	12448	20	111	139.0151515	11.43744515	0.542364693	0.035629437	-	2828	grey	-0.123522676	FALSE	GPR4
Region_9511	chr19	45606994	45645602	38608	35	202	245.6363636	17.97574123	0.64960713	0.016294279	-	24139	grey	0.006798547	FALSE	EML2
Region_9512	chr1	45641494	45642840	1346	4	21	34.5303030	6.04144582	0.01335805	0.02459897	+	10028717	grey	-0.16737482	FALSE	EML2-AS1

	9						3		4	9		7		9		
Region_9513	chr19	45668221	45683722	15501	42	242	287.6363636	22.05631698	0.485770463	0.020638766	+	2696	grey	0.165187153	FALSE	GIPR
Region_9514	chr19	45687460	45692316	4856	8	51	65.09090909	8.685491479	0.607786933	0.017612684	-	6633	grey	0.386187764	FALSE	SNRPD2
Region_9515	chr19	45692403	45703989	11586	12	67	85.51515152	8.309850363	0.641296053	0.029892495	+	54814	grey	-0.014001202	FALSE	QPCTL
Region_9516	chr19	45710629	45730896	20267	17	91	121.2272727	12.83240544	0.610207391	0.025178534	-	23403	grey	0.001805617	FALSE	FBXO46
Region_9517	chr19	45733439	45764541	31102	25	152	185.469697	16.07886305	0.934001206	0.017631556	+	388553	grey	0.017938852	FALSE	MEIOSIN
Region_9518	chr19	45764785	45769252	4467	12	59	96.28787879	13.79717379	0.18104384	0.027909672	-	147912	grey	-0.010061979	FALSE	SIX5
Region_9519	chr19	45767796	45772504	4708	20	94	139.2272727	16.09280341	0.18877192	0.024342342	+	109729182	grey	-0.051176965	FALSE	DM1-AS
Region_9520	chr19	45769709	45785713	16004	27	133	167.1515152	15.69148953	0.644647571	0.028012919	-	1760	grey	0.10021756	FALSE	DMPK
Region_9521	chr19	45779437	45792845	13408	25	132	167.4393939	14.79930226	0.700294565	0.029479135	-	1762	grey	0.077077344	FALSE	DMWD
Region_9522	chr19	45795713	45815308	19595	20	103	139.1212121	13.59922526	0.924709755	0.021710759	-	81492	grey	0.107763567	FALSE	RSPH6A
Region_9523	chr19	45815410	45863194	47784	52	317	373.8030303	21.64626079	0.901414779	0.015165229	-	8189	grey	0.06705308	FALSE	SYMPK
Region_9524	chr19	45863989	45873797	9808	9	38	62.04545455	8.207748343	0.720298346	0.060264677	+	3171	grey	0.08150213	FALSE	FOXA3

Region_9525	chr19	45883608	45886141	2533	18	112	143.2272727	18.10379939	0.338733971	0.018817171	-	26145	grey	-0.09198414	FALSE	IRF2BP1
Region_9526	chr19	45890023	45902613	12590	22	131	154.2727273	12.72858135	0.683027966	0.019922304	-	339344	grey	0.059446866	FALSE	MYPOP
Region_9527	chr19	45933734	45974044	40310	41	224	262.8939394	18.12616366	0.631243278	0.0291514	-	4858	grey	0.060859217	FALSE	NOVA2
Region_9528	chr19	45995461	46018616	23155	33	182	217.530303	16.11804502	0.597548714	0.018412055	+	729440	grey	-0.02268278	FALSE	CCDC61
Region_9529	chr19	46019153	46023053	3900	8	36	58.46969697	8.718185586	0.526781057	0.050968809	-	8993	grey	-0.008348433	FALSE	PGLYRP1
Region_9530	chr19	46039182	46077118	37936	10	65	82.60606061	8.55727992	0.839831427	0.049552237	-	444882	grey	-0.006157995	FALSE	IGFL4
Region_9531	chr19	46101122	46214838	113716	51	375	425.5909091	22.96752039	0.83170349	0.015350292	+	147920	grey	-0.336158287	FALSE	IGFL2
Region_9532	chr19	46163893	46180647	16754	10	69	86.87878788	8.991474854	0.783739491	0.03759851	-	105372424	grey	-0.166057994	FALSE	LOC105372424
Region_9533	chr19	46189029	46203160	14131	8	46	62.51515152	7.708621396	0.786893708	0.04828648	-	645553	grey	-0.300158466	FALSE	IGFL2-AS1
Region_9534	chr19	46210242	46214837	4595	4	17	31.21212121	5.542882204	0.884666023	0.055758988	+	93429	grey	-0.263093537	FALSE	LOC93429
Region_9535	chr19	46297042	46343433	46391	29	163	196.5454545	14.36468085	0.834336008	0.025769036	+	64344	grey	-0.088029751	FALSE	HIF3A
Region_9536	chr19	46347087	46392981	45894	40	274	312.969697	21.49774206	0.830659848	0.01796007	+	5536	grey	-0.140489739	FALSE	PPP5C
Region_9537	chr1	46393359	46404276	10917	14	84	107.696969	9.73964096	0.82652344	0.03303699	-	10537242	grey	0.016650035	FALSE	LOC105372426

	9						7	2	3	2		6				
Region_9538	chr19	46424697	46428936	4239	5	28	41.90909091	6.858408142	0.229273288	0.041748777	-	110806277	grey	-0.049411621	FALSE	PNMA8C
Region_9539	chr19	46466491	46471563	5072	5	27	41.3030303	5.493967872	0.904981582	0.057413701	-	55228	grey	-0.047574746	FALSE	PNMA8A
Region_9540	chr19	46480788	46601200	120412	70	461	530	27.09186651	0.716983706	0.015347971	-	100506012	grey	-0.010850259	FALSE	PPP5D1P
Region_9541	chr19	46491191	46495867	4676	9	37	59.51515152	8.732331479	0.197757526	0.059268933	-	57469	grey	-0.110708128	FALSE	PNMA8B
Region_9542	chr19	46601074	46610782	9708	5	30	41.39393939	6.050756565	0.819067392	0.049325671	+	808	grey	-0.133464385	FALSE	CALM3
Region_9543	chr19	46633953	46636541	2588	4	15	26.46969697	4.605079794	0.060245216	0.051781405	-	94235	grey	-0.030281284	FALSE	GNG8
Region_9544	chr19	46647551	46661182	13631	6	18	30.13636364	6.009956774	0.419964526	0.075412594	-	147906	grey	-0.029715819	FALSE	DACT3
Region_9545	chr19	46660364	46678991	18627	9	51	68.3030303	8.032567742	0.542479039	0.032685693	+	100506068	grey	0.082239046	FALSE	DACT3-AS1
Region_9546	chr19	46674275	46717127	42852	22	126	154.5	10.68464031	0.664979831	0.019074907	-	25865	grey	-0.119057219	FALSE	PRKD2
Region_9547	chr19	46719511	46746994	27483	13	72	94.10606061	10.30179788	0.794778575	0.040621752	-	29888	grey	-0.176742274	FALSE	STRN4
Region_9548	chr19	46746046	46776988	30942	13	77	108.3484848	11.74405568	0.767297401	0.05393899	+	79147	grey	0.251604626	FALSE	FKRP
Region_9549	chr19	46774883	46788594	13711	15	79	98.71212121	7.826513423	0.326684437	0.017024283	-	6510	grey	0.275862509	FALSE	SLC1A5

Region_9550	chr19	46838136	46850846	12710	14	64	84.45454545	9.267282098	0.562873676	0.040929185	-	1175	grey	0.026070145	FALSE	AP2S1
Region_9551	chr19	46860997	47005077	144080	110	777	871.5757576	39.63761252	0.911458303	0.010029271	+	2909	grey	-0.019876914	FALSE	ARHGAP35
Region_9552	chr19	47019837	47045775	25938	27	149	179.8030303	13.51719099	0.479884062	0.021937957	+	4861	grey	0.141649626	FALSE	NPAS1
Region_9553	chr19	47045909	47048624	2715	5	13	31.42424242	8.253783479	0.284665678	0.061051029	-	54958	grey	-0.16595804	FALSE	TMEM160
Region_9554	chr19	47064187	47113776	49589	37	243	285.4848485	17.73500877	0.79373433	0.018803866	-	23211	grey	0.234050689	FALSE	ZC3H4
Region_9555	chr19	47113274	47210636	97362	66	457	521	27.66391707	0.846135273	0.009778263	+	10055	grey	-0.073664899	FALSE	SAE1
Region_9556	chr19	47220824	47232766	11942	23	128	154.9545455	12.45722752	0.310576977	0.015977116	-	27113	grey	-0.258267569	FALSE	BBC3
Region_9557	chr19	47256523	47273701	17178	13	68	83.22727273	9.129488071	0.808299326	0.026847825	+	26093	grey	-0.003402925	FALSE	CCDC9
Region_9558	chr19	47290023	47307754	17731	14	86	114.6818182	11.07201474	0.806672339	0.029576562	+	105372427	grey	-0.139791419	FALSE	
Region_9559	chr19	47297075	47322066	24991	13	79	104.3636364	10.2471555	0.752612986	0.037785205	+	728	grey	-0.036649534	FALSE	C5AR1
Region_9560	chr19	47332175	47347329	15154	14	76	107.530303	10.69179369	0.787659758	0.039811401	+	27202	grey	0.056857109	FALSE	C5AR2
Region_9561	chr19	47349315	47382704	33389	36	227	277.1666667	17.92942003	0.839115457	0.017441838	+	9704	grey	0.059441579	FALSE	DHX34
Region_9562	chr1	47403124	47419527	16403	9	38	58.9393939	9.13131347	0.87754803	0.05285789	-	56917	grey	-0.03953623	FALSE	MEIS3

	9						4	5		4						
Region_9563	chr19	47428017	47471893	43876	25	157	195.2272727	16.31025676	0.507774637	0.032290895	-	6543	grey	0.127838366	FALSE	SLC8A2
Region_9564	chr19	47475150	47484265	9115	4	20	30.96969697	5.120005099	0.754350577	0.051570616	-	11133	grey	-0.284380722	FALSE	KPTN
Region_9565	chr19	47484282	47502399	18117	22	119	151.5606061	12.57241032	0.729542562	0.029929102	+	100505681	grey	-0.230004747	FALSE	NAPA-AS1
Region_9566	chr19	47487637	47515091	27454	30	159	203.6363636	16.81266589	0.686093353	0.022412791	-	8775	grey	-0.219448142	FALSE	NAPA
Region_9567	chr19	47520690	47573148	52458	49	295	349.4848485	21.279273	0.803182391	0.022501794	-	84215	grey	-0.224290319	FALSE	ZNF541
Region_9568	chr19	47608196	47703277	95081	66	424	482.0909091	27.76091734	0.874712154	0.012334187	+	29998	grey	-0.083799055	FALSE	BICRA
Region_9569	chr19	47713422	47743134	29712	38	193	242.1060606	15.21449591	0.886362839	0.020711475	+	30846	grey	-0.144063197	FALSE	EHD2
Region_9570	chr19	47745546	47757058	11512	17	93	119.8484848	14.23996805	0.8873973	0.025953557	+	29997	grey	0.051509252	FALSE	NOP53
Region_9571	chr19	47757036	47768840	11804	14	94	117.6060606	10.42759473	0.921111386	0.030383824	-	106144593	grey	-0.117026513	FALSE	NOP53-AS1
Region_9572	chr19	47778677	47784686	6009	6	22	31.56060606	5.384104205	0.499667012	0.065158374	+	6415	grey	-0.108501442	FALSE	SELENOW
Region_9573	chr19	47801232	47819051	17819	12	78	95.54545455	9.796674189	0.862020232	0.038977471	-	284355	grey	0.176122839	FALSE	TPRX1
Region_9574	chr19	47819779	47843330	23551	20	139	160.8181818	13.98560699	0.841858968	0.041553178	+	1406	grey	-0.067428176	FALSE	CRX

Region_9575	chr19	47870467	47886315	15848	17	100	125.1969697	13.00321818	0.918435671	0.033797253	-	6822	grey	0.325719337	FALSE	SULT2A1
Region_9576	chr19	47968046	47992170	24124	13	86	105.4090909	9.617255762	0.876640966	0.047395945	-	100131137	grey	0.049454926	FALSE	BSPH1
Region_9577	chr19	47994632	48025154	30522	12	56	75.15151515	8.832183151	0.88917054	0.039067557	+	64100	grey	0.261869874	FALSE	ELSPBP1
Region_9578	chr19	48029383	48044079	14696	11	42	64.46969697	10.37188463	0.787280202	0.067259362	-	56344	grey	-0.203109202	FALSE	CABP5
Region_9579	chr19	48047843	48110817	62974	46	306	346.7575758	20.78835518	0.891249311	0.019664138	-	8605	grey	0.371815896	FALSE	PLA2G4C
Region_9580	chr19	48115445	48170603	55158	47	311	364.969697	27.77490273	0.847412007	0.014834213	-	3978	grey	-0.076456065	FALSE	LIG1
Region_9581	chr19	48170680	48197620	26940	25	172	203.1212121	15.60129694	0.856271594	0.037141251	+	374920	grey	0.095053086	FALSE	ZSWIM9
Region_9582	chr19	48203146	48255946	52800	43	323	373.7878788	22.65155125	0.817159324	0.013290119	-	22900	grey	0.023181411	FALSE	CARD8
Region_9583	chr19	48262900	48271283	8383	8	36	56.63636364	7.139567674	0.40743859	0.057052991	-	105372429	grey	-0.023807872	FALSE	ZNF114-AS1
Region_9584	chr19	48270081	48287608	17527	17	97	122.1969697	13.08695145	0.847310945	0.021823531	+	163071	grey	0.074576019	FALSE	ZNF114
Region_9585	chr19	48296457	48321971	25514	21	113	151.4848485	12.26658052	0.842138312	0.025831835	-	93233	grey	-0.255326287	FALSE	ODAD1
Region_9586	chr19	48321509	48330553	9044	6	28	41.10606061	6.691277417	0.325544223	0.028839169	+	2014	grey	0.236089861	FALSE	EMP3
Region_9587	chr1	48332356	48364059	31703	28	150	183.469697	14.7128616	0.67818266	0.02325647	-	55260	grey	-0.06555648	FALSE	TMEM143

	9							6	8	3				5		
Region_9588	chr19	48364367	48376377	12010	10	47	69.87878788	8.457159282	0.833610571	0.038350953	+	23546	grey	0.216438235	FALSE	SYNGR4
Region_9589	chr19	48382575	48391551	8976	5	12	27.40909091	6.171839952	0.92226526	0.073104542	-	10945	grey	-0.075966601	FALSE	KDELR1
Region_9590	chr19	48393668	48444931	51263	47	241	284.7121212	19.35479678	0.675685447	0.02225182	+	2906	grey	-0.360263693	FALSE	GRIN2D
Region_9591	chr19	48445841	48457022	11181	11	47	66.40909091	8.240569944	0.931265398	0.038223988	+	83743	grey	0.062315361	FALSE	GRWD1
Region_9592	chr19	48455574	48466980	11406	5	21	32.95454545	5.539874528	0.952480146	0.036891188	+	3770	grey	-0.154808792	FALSE	KCNJ14
Region_9593	chr19	48465837	48482314	16477	5	21	33.39393939	6.28276957	0.328552079	0.042169605	+	9266	grey	0.153525404	FALSE	CYTH2
Region_9594	chr19	48485271	48513935	28664	17	84	114.1212121	12.21917176	0.605366642	0.031398753	-	114783	grey	-0.128673095	FALSE	LMTK3
Region_9595	chr19	48552172	48599425	47253	32	193	222.2121212	16.67011619	0.802333852	0.025852756	+	6820	grey	-0.078442129	FALSE	SULT2B1
Region_9596	chr19	48599961	48615076	15115	15	60	86.45454545	10.96172617	0.928686152	0.030722351	-	54854	grey	-0.108225434	FALSE	FAM83E
Region_9597	chr19	48615328	48619184	3856	3	14	23.51515152	4.61516705	0.897814559	0.060029933	-	6141	grey	-0.070149625	FALSE	RPL18
Region_9598	chr19	48619291	48630717	11426	20	127	163.3484848	16.10971272	0.586538394	0.025356966	+	56848	grey	0.141550205	FALSE	SPHK2
Region_9599	chr19	48630030	48637379	7349	11	43	66.12121212	9.908062221	0.287859325	0.042419954	-	1628	grey	0.10125964	FALSE	DBP

Region_9600	chr19	48637946	48646187	8241	7	23	41.28787879	6.593155312	0.579797553	0.079252335	-	770	grey	-0.159649768	FALSE	CA11
Region_9601	chr19	48638071	48682245	44174	44	258	290.8787879	18.01410998	0.814335613	0.023559483	+	653677	grey	-0.311607505	FALSE	SEC1P
Region_9602	chr19	48661407	48673081	11674	12	52	75.37878788	9.293115982	0.918725196	0.040580162	-	126147	grey	-0.340087943	FALSE	NTN5
Region_9603	chr19	48695971	48705951	9980	8	42	62.48484848	8.707631355	0.549652172	0.064995976	+	2524	grey	-0.004296578	FALSE	FUT2
Region_9604	chr19	48720585	48740610	20025	18	82	104.7272727	11.2366782	0.943048181	0.029295683	-	54922	grey	-0.169852594	FALSE	RASIP1
Region_9605	chr19	48740852	48746909	6057	6	27	38.75757576	6.394855158	0.463506377	0.068421346	-	284359	grey	0.062779294	FALSE	IZUMO1
Region_9606	chr19	48748011	48755390	7379	5	22	36.37878788	6.311808595	0.393004302	0.035660733	-	2523	grey	-0.153352476	FALSE	FUT1
Region_9607	chr19	48795062	48811029	15967	14	62	91.37878788	11.88375122	0.592501815	0.025791167	-	587	grey	-0.072056682	FALSE	BCAT2
Region_9608	chr19	48813018	48836510	23492	22	126	156.530303	14.94113547	0.909379307	0.02066203	-	51171	grey	-0.018915561	FALSE	HSD17B14
Region_9609	chr19	48837097	48868617	31520	34	172	224.7878788	17.01900761	0.829502764	0.025312139	-	57664	grey	-0.194939946	FALSE	PLEKHA4
Region_9610	chr19	48872421	48876058	3637	8	36	52.21212121	8.449879656	0.176488447	0.05823812	+	23645	grey	0.13646756	FALSE	PPP1R15A
Region_9611	chr19	48880967	48898744	17777	27	125	158.5606061	16.49347544	0.738683461	0.02329125	-	7288	grey	-0.106531766	FALSE	TULP2
Region_9612	chr1	48900312	48923372	23060	26	153	198.954545	13.1960738	0.88005807	0.02446439	+	4924	grey	0.308399739	FALSE	NUCB1

	9						5			2						
Region_9613	chr19	48910930	48918891	7961	11	47	69.48484848	9.011518141	0.870493889	0.040796746	-	100874085	grey	0.120343606	FALSE	NUCB1-AS1
Region_9614	chr19	48933699	48944969	11270	15	63	97	12.42330317	0.927666486	0.028796501	+	27294	grey	0.100310981	FALSE	DHDH
Region_9615	chr19	48954815	48961798	6983	6	21	34.28787879	6.024986822	0.806753919	0.026930271	+	581	grey	-0.088315354	FALSE	BAX
Region_9616	chr19	48965309	48966879	1570	8	49	74.46969697	10.50013875	0.00202767	0.005150568	+	2512	grey	-0.171414815	FALSE	FTL
Region_9617	chr19	48968130	48993310	25180	35	215	259.4545455	19.62900665	0.87126013	0.013322526	-	2997	grey	-0.083465168	FALSE	GYS1
Region_9618	chr19	48993562	49015970	22408	33	231	260.6060606	15.79764227	0.821530922	0.012096913	+	10856	grey	-0.117747584	FALSE	RUVBL2
Region_9619	chr19	49022869	49036895	14026	11	53	77.48484848	11.92830174	0.93509142	0.032723923	-	1082	grey	-0.013335628	FALSE	CGB3
Region_9620	chr19	49054274	49065076	10802	16	79	109.7272727	12.72737263	0.436062977	0.046764631	-	94027	grey	0.054042219	FALSE	CGB7
Region_9621	chr19	49057858	49065076	7218	9	43	60.87878788	9.471439094	0.58238616	0.08023878	-	4909	grey	0.053048001	FALSE	NTF4
Region_9622	chr19	49067397	49072699	5302	12	81	108.6666667	13.97727093	0.365857143	0.028518788	-	3743	grey	-0.156021621	FALSE	KCNA7
Region_9623	chr19	49085419	49108605	23186	31	183	222.9393939	17.84366191	0.932525169	0.024128806	+	6625	grey	0.129928677	FALSE	SNRNP70
Region_9624	chr19	49114324	49118460	4136	8	40	55.65151515	8.511516947	0.372246766	0.024103796	+	64130	grey	0.04448431	FALSE	LIN7B

Region_9625	chr19	49119544	49151026	31482	51	297	343.2727273	23.79046057	0.692331162	0.029008864	+	8541	grey	-0.036852077	FALSE	PPFIA3
Region_9626	chr19	49157741	49211836	54095	54	339	393.7878788	22.32316158	0.80264953	0.022367786	+	54795	grey	-0.177188268	FALSE	TRPM4
Region_9627	chr19	49289638	49340303	50665	34	217	256.8333333	18.00049857	0.893863116	0.025356383	-	28968	grey	-0.077779063	FALSE	SLC6A16
Region_9628	chr19	49335171	49340606	5435	7	15	31.16666667	7.139453401	0.956951745	0.037272157	+	951	grey	0.057137563	FALSE	CD37
Region_9629	chr19	49340595	49362457	21862	11	56	80.13636364	9.961288708	0.80670258	0.055942477	-	8463	grey	-0.272702967	FALSE	TEAD2
Region_9630	chr19	49361783	49375116	13333	15	92	111.530303	10.33920053	0.695352481	0.034870133	+	27120	grey	-0.172236761	FALSE	DKKL1
Region_9631	chr19	49368558	49388091	19533	10	39	66.39393939	10.59445252	0.951494675	0.031580567	-	101928295	grey	0.34095373	FALSE	LOC101928295
Region_9632	chr19	49388219	49417990	29771	23	127	152	13.3554944	0.894414637	0.030174725	+	147872	grey	0.178607011	FALSE	KASH5
Region_9633	chr19	49423749	49428987	5238	4	16	25.77272727	4.436892379	0.370183026	0.064467788	+	100507003	grey	-0.076112007	FALSE	GFY
Region_9634	chr19	49429401	49442360	12959	20	103	141.1818182	14.50295364	0.453651266	0.05362448	-	57030	grey	-0.009274166	FALSE	SLC17A7
Region_9635	chr19	49446298	49453497	7199	6	27	41.95454545	7.384873976	0.632882324	0.039928655	-	55011	grey	0.028883457	FALSE	PIH1D1
Region_9636	chr19	49453225	49471050	17825	32	178	208.6969697	17.16523293	0.809510369	0.021670224	+	126133	grey	-0.066838563	FALSE	ALDH16A1
Region_9637	chr1	49462752	49487637	24885	30	171	199.757575	13.4599812	0.73447772	0.02152141	+	2323	grey	-0.08487982	FALSE	FLT3LG

	9						8	6	8	2				6		
Region_9638	chr19	49487510	49493057	5547	8	52	71.22727273	9.416508343	0.434009209	0.044347473	+	23521	grey	-0.38795264	FALSE	RPL13A
Region_9639	chr19	49496365	49499708	3343	8	50	70.83333333	9.192946014	0.121205833	0.016310521	+	6205	grey	0.082356056	FALSE	RPS11
Region_9640	chr19	49506816	49526428	19612	31	179	214.4393939	18.57718103	0.893095244	0.020106969	+	2217	grey	-0.062500989	FALSE	FCGRT
Region_9641	chr19	49528003	49546962	18959	22	97	156.5757576	17.13032453	0.775590752	0.032787961	+	57333	grey	-0.265374027	FALSE	RCN3
Region_9642	chr19	49555468	49590262	34794	43	245	287.969697	18.96796465	0.803626066	0.025918674	-	51070	grey	0.209818726	FALSE	NOSIP
Region_9643	chr19	49580646	49591004	10358	11	53	73.31818182	8.726897574	0.851436246	0.044555894	+	5639	grey	0.105988225	FALSE	PRRG2
Region_9644	chr19	49591182	49626439	35257	57	308	364.7121212	25.41517727	0.912857756	0.015815054	+	57479	grey	0.285567841	FALSE	PRR12
Region_9645	chr19	49635292	49640143	4851	9	30	46.37878788	7.505413742	0.861704998	0.06256509	-	6237	grey	-0.149561741	FALSE	RRAS
Region_9646	chr19	49642209	49658642	16433	38	184	232.1818182	19.82764899	0.957039287	0.0166467	+	58506	grey	-0.036448965	FALSE	SCAF1
Region_9647	chr19	49659569	49665875	6306	9	40	57.57575758	8.318765822	0.627079007	0.042231995	-	3661	grey	0.00916663	FALSE	IRF3
Region_9648	chr19	49665142	49673916	8774	9	55	73.81818182	8.483303223	0.753429038	0.028613551	+	83596	grey	-0.17830742	FALSE	BCL2L12
Region_9649	chr19	49675786	49689029	13243	18	95	115.3787879	11.08257836	0.687650025	0.032717133	+	3276	grey	-0.153789522	FALSE	PRMT1

Region_9650	chr19	49690898	49713731	22833	23	125	156.6666667	15.5912533	0.886002564	0.02889918	+	126129	grey	-0.007510066	FALSE	CPT1C
Region_9651	chr19	49739757	49763306	23549	9	45	59.86363636	7.00963373	0.926992881	0.046460354	-	60385	grey	-0.193423732	FALSE	TSKS
Region_9652	chr19	49767001	49807114	40113	18	121	150.2727273	11.97631696	0.957188936	0.020860834	+	160	grey	-0.128504575	FALSE	AP2A1
Region_9653	chr19	49806866	49817376	10510	6	33	44.24242424	6.246378904	0.17975091	0.026587263	-	80199	grey	0.161403005	FALSE	FUZ
Region_9654	chr19	49808933	49809738	805	3	14	23.37878788	4.509017125	0.026443058	0.045233777	+	105372435	grey	0.191813465	FALSE	LOC105372435
Region_9655	chr19	49818282	49840383	22101	13	79	102.7272727	10.22597816	0.837405048	0.022564242	+	81857	grey	0.031811829	FALSE	MED25
Region_9656	chr19	49838639	49851676	13037	15	103	137.0454545	12.1824992	0.65707453	0.029148596	-	100506033	grey	-0.038647237	FALSE	PTOV1-AS1
Region_9657	chr19	49850735	49860744	10009	12	45	61.68181818	8.666751837	0.796654033	0.053144497	+	53635	grey	-0.235754496	FALSE	PTOV1
Region_9658	chr19	49860099	49878351	18252	30	162	194.3181818	16.30588293	0.392900774	0.02219241	-	11284	grey	-0.029551857	FALSE	PNKP
Region_9659	chr19	49869033	49878459	9426	8	45	64.15151515	8.350481192	0.47700212	0.023700318	-	84335	grey	-0.234425359	FALSE	AKT1S1
Region_9660	chr19	49877666	49888750	11084	8	38	57.75757576	8.190164094	0.966905827	0.02601889	+	79735	grey	-0.265404459	FALSE	TBC1D17
Region_9661	chr19	49889654	49929539	39885	42	257	299.8484848	19.29307761	0.708848136	0.02839011	-	259307	grey	0.094186206	FALSE	IL4I1
Region_9662	chr1	49906825	49958391	51566	58	345	397.803030	21.2792565	0.71744183	0.01988031	-	23636	grey	0.005046413	FALSE	NUP62

	9						3	6	2	1						
Region_9663	chr19	49928702	49933935	5233	9	34	52.07575758	8.608244002	0.103076678	0.023473216	+	22809	grey	-0.102321331	FALSE	ATF5
Region_9664	chr19	49948985	49961172	12187	13	75	95.39393939	9.169982448	0.845453232	0.037837257	-	114132	grey	-0.061555999	FALSE	SIGLEC11
Region_9665	chr19	49969600	49975819	6219	5	26	41.27272727	7.592598531	0.783462426	0.08559217	+	400709	grey	-0.12198363	FALSE	SIGLEC16
Region_9666	chr19	49976468	50025946	49478	50	374	419.5757576	19.82621466	0.858137674	0.01352808	-	51231	grey	0.093522648	FALSE	VRK3
Region_9667	chr19	50025714	50053414	27700	33	266	313.8484848	19.7431526	0.682871886	0.01572263	+	25888	grey	0.180287224	FALSE	ZNF473
Region_9668	chr19	50050589	50075902	25313	28	201	239.2121212	15.13682738	0.778100273	0.023879008	+	400710	grey	-0.009232019	FALSE	ZNF473CR
Region_9669	chr19	50095898	50148302	52404	32	282	408.3787879	53.00297964	0.827435778	0.033325864	-	105372436	grey	-0.035753693	FALSE	LOC105372436
Region_9670	chr19	50152548	50163281	10733	11	56	79.51515152	9.692570389	0.912175916	0.039399758	-	126123	grey	0.168800566	FALSE	IZUMO2
Region_9671	chr19	50188186	50310542	122356	106	693	791.0151515	40.37954258	0.834435679	0.018006304	+	79784	grey	-0.076698744	FALSE	MYH14
Region_9672	chr19	50311937	50333515	21578	27	118	148.9545455	13.74765078	0.402231799	0.032341988	-	3748	grey	-0.074098204	FALSE	KCNC3
Region_9673	chr19	50329653	50383388	53735	72	523	585.4393939	25.86540832	0.60990849	0.01544441	+	7376	grey	-0.089897778	FALSE	NR1H2
Region_9674	chr19	50329722	50371440	41718	50	354	395.8181818	21.46977058	0.567669388	0.019726697	+	105372437	grey	-0.17664008	FALSE	LOC105372437

Region_9675	chr19	50333796	50344806	11010	7	40	62	10.64388593	0.439925114	0.038131788	-	256236	grey	-0.295359775	FALSE	NAPSB
Region_9676	chr19	50358477	50369331	10854	16	92	117.8636364	12.15779125	0.655702917	0.027434926	-	9476	grey	0.127363702	FALSE	NAPSA
Region_9677	chr19	50384204	50418018	33814	45	267	307.9090909	20.09571502	0.849636446	0.019163676	+	5424	grey	0.040764298	FALSE	POLD1
Region_9678	chr19	50415799	50431314	15515	13	90	110.6060606	11.08134786	0.669347706	0.029115237	+	6689	grey	-0.096587313	FALSE	SPIB
Region_9679	chr19	50432892	50466321	33429	33	198	239.7727273	16.17956494	0.793268108	0.032243268	+	4606	grey	0.02308985	FALSE	MYBPC2
Region_9680	chr19	50466785	50476848	10063	9	45	62.27272727	8.626516285	0.824124212	0.037584137	-	112703	grey	0.124672076	FALSE	GARIN5A
Region_9681	chr19	50476400	50490871	14471	14	65	88.12121212	10.43148363	0.885448181	0.026398584	+	284361	grey	0.118190933	FALSE	EMC10
Region_9682	chr19	50505998	50511220	5222	8	27	53.8030303	8.69875269	0.859524395	0.02347089	-	126119	grey	0.007408539	FALSE	JOSD2
Region_9683	chr19	50516892	50568435	51543	43	234	261.0606061	15.2506028	0.784125362	0.033224257	-	94030	grey	-0.072514586	FALSE	LRRC4B
Region_9684	chr19	50621977	50639881	17904	20	114	143.5909091	11.66451196	0.801705753	0.041081998	-	84258	grey	0.357366793	FALSE	SYT3
Region_9685	chr19	50649445	50659310	9865	3	12	22.22727273	4.643590966	0.103690097	0.093416225	+	342918	grey	-0.167609981	FALSE	C19orf81
Region_9686	chr19	50659255	50719802	60547	46	243	296.1515152	20.96681805	0.599133851	0.020207154	-	50944	grey	0.149772079	FALSE	SHANK1
Region_9687	chr1	50723364	50725708	2344	5	13	27.8333333	5.93965379	0.00881094	0.02149391	+	6320	grey	0.52675586	FALSE	CLEC11A

	9						3	3	5	1						
Region_9688	chr19	50776141	50786160	10019	12	59	82.13636364	8.680457917	0.618023429	0.054740706	-	105372440	grey	-0.143694419	FALSE	SMIM47
Region_9689	chr19	50797687	50804929	7242	9	44	59.53030303	9.123460371	0.509304144	0.038206699	-	84798	grey	-0.010493085	FALSE	C19orf48P
Region_9690	chr19	50808874	50818880	10006	6	30	47.86363636	7.253405277	0.558585052	0.050348541	+	284365	grey	0.022155203	FALSE	LINC01869
Region_9691	chr19	50825289	50837213	11924	17	77	102.0757576	10.68184744	0.725983627	0.052843814	-	55554	grey	0.171645758	FALSE	KLK15
Region_9692	chr19	50830530	50851089	20559	17	91	110.3787879	8.810422415	0.743754827	0.046569977	+	105372441	grey	0.126752828	FALSE	LOC105372441
Region_9693	chr19	50861568	50880567	18999	9	60	75.43939394	7.084389747	0.807546859	0.071224318	+	3817	grey	-0.033167526	FALSE	KLK2
Region_9694	chr19	50882096	50896398	14302	5	28	44.27272727	5.562361022	0.776865395	0.084268653	-	606293	grey	-0.184321368	FALSE	KLKP1
Region_9695	chr19	50906351	50911395	5044	3	14	23.15151515	4.297557175	0.652504489	0.126707308	-	9622	grey	-0.133061281	FALSE	KLK4
Region_9696	chr19	50943303	50953093	9790	8	29	43.18181818	6.670045764	0.882465686	0.064328911	-	25818	grey	-0.147574266	FALSE	KLK5
Region_9697	chr19	50958631	50969673	11042	8	34	56.72727273	7.343328641	0.622050969	0.059923401	-	5653	grey	0.300744544	FALSE	KLK6
Region_9698	chr19	50968193	51012129	43936	30	174	207.5454545	13.88544542	0.708679454	0.036900062	+	105372442	grey	0.079867164	FALSE	LOC105372442
Region_9699	chr19	50976468	50984099	7631	4	13	25.06060606	4.914276808	0.265927665	0.129937255	-	5650	grey	0.327831764	FALSE	KLK7

Region_9700	chr19	50996018	51009634	13616	7	21	31.90909091	6.406072294	0.766820549	0.088511173	-	284366	grey	-0.029940755	FALSE	KLK9
Region_9701	chr19	51012739	51020175	7436	13	48	67.59090909	9.989189961	0.295745499	0.06086838	-	5655	grey	0.026803067	FALSE	KLK10
Region_9702	chr19	51022216	51028039	5823	4	12	23.04545455	5.178154759	0.807698741	0.094222597	-	11012	grey	0.002537361	FALSE	KLK11
Region_9703	chr19	51029092	51035230	6138	5	31	43.16666667	6.871531759	0.821354221	0.090690185	-	43849	grey	-0.089307182	FALSE	KLK12
Region_9704	chr19	51055626	51065114	9488	6	17	29.90909091	5.521224147	0.673191993	0.062814374	-	26085	grey	-0.157123599	FALSE	KLK13
Region_9705	chr19	51077495	51084245	6750	5	18	32	6.043432545	0.874087672	0.073031784	-	43847	grey	0.186992027	FALSE	KLK14
Region_9706	chr19	51097606	51108409	10803	18	50	95.13636364	15.44507526	0.745336763	0.058110237	-	90353	grey	-0.084262243	FALSE	CTU1
Region_9707	chr19	51124906	51138634	13728	8	54	69.8030303	7.811667205	0.595832204	0.05455803	+	27180	grey	-0.024803536	FALSE	SIGLEC9
Region_9708	chr19	51142299	51153526	11227	6	23	38.39393939	6.263149338	0.777458491	0.074878818	+	27036	grey	-0.023496773	FALSE	SIGLEC7
Region_9709	chr19	51146475	51181966	35491	11	63	86.48484848	9.466121656	0.812285843	0.039729463	-	101928517	grey	0.082054017	FALSE	LOC101928517
Region_9710	chr19	51211080	51243860	32780	11	74	100.7424242	9.212550981	0.729689416	0.046443404	+	945	grey	0.133016134	FALSE	CD33
Region_9711	chr19	51246348	51269330	22982	8	55	77.31818182	8.272585106	0.91287912	0.035165941	+	284369	grey	-0.151103432	FALSE	SIGLECL1
Region_9712	chr1	51251231	51271179	19948	7	50	69.9848484	7.49254485	0.90017506	0.03863197	-	10798532	grey	-0.21929990	FALSE	LOC107985327

	9						8	7	9	7		7		9		
Region_9713	chr19	51271266	51281234	9968	3	12	20.46969697	4.493481422	0.628388825	0.057768651	+	105372444	grey	0.132392237	FALSE	LINC01872
Region_9714	chr19	51311644	51330891	19247	8	31	49.84848485	6.60825224	0.736156801	0.063066317	+	402665	grey	0.040153842	FALSE	IGLON5
Region_9715	chr19	51331536	51342139	10603	19	85	121.7575758	13.60323559	0.31232116	0.015809947	-	147645	grey	0.058910095	FALSE	VSIG10L
Region_9716	chr19	51345169	51368099	22930	7	32	47.18181818	6.818321677	0.698728294	0.043885008	-	2109	grey	-0.1212101	FALSE	ETFB
Region_9717	chr19	51388289	51390591	2302	3	10	23.28787879	4.793473613	0.966276106	0.036967029	-	147646	grey	-0.039315133	FALSE	C19orf84
Region_9718	chr19	51394488	51403650	9162	4	17	24.87878788	4.925647639	0.826548595	0.086600032	+	105372446	grey	0.121518604	FALSE	LIM2-AS1
Region_9719	chr19	51410020	51417803	7783	5	27	42.54545455	6.10720084	0.88167233	0.060205723	-	89790	grey	-0.09939903	FALSE	SIGLEC10
Region_9720	chr19	51450847	51458456	7609	6	22	37.84848485	8.427505196	0.919466317	0.052446399	-	27181	grey	0.087539152	FALSE	SIGLEC8
Region_9721	chr19	51478539	51491301	12762	3	17	28.18181818	5.417660838	0.923672017	0.051576889	+	729767	grey	0.036767772	FALSE	CEACAM18
Region_9722	chr19	51517819	51531856	14037	13	73	96.53030303	8.149034293	0.873390879	0.044063446	-	946	grey	-0.020811239	FALSE	SIGLEC6
Region_9723	chr19	51571283	51592510	21227	3	14	23.46969697	5.084576064	0.970315401	0.043825488	+	7728	grey	-0.166630943	FALSE	ZNF175
Region_9724	chr19	51610960	51645545	34585	15	45	96.10606061	19.48105888	0.69284214	0.047242612	-	8778	grey	-0.067677605	FALSE	SIGLEC5

Region_9725	chr19	51689128	51712387	23259	16	95	124.2272727	14.33639312	0.681975113	0.02509541	+	147650	grey	0.317697268	FALSE	SPACA6
Region_9726	chr19	51713112	51723991	10879	9	46	63.92424242	10.27215895	0.737945668	0.046633311	-	3036	grey	-0.27299162	FALSE	HAS1
Region_9727	chr19	51745172	51804115	58943	20	125	154.8636364	12.17549413	0.747303469	0.037486384	-	2357	grey	0.225000211	FALSE	FPR1
Region_9728	chr19	51752026	51770531	18505	5	27	39.93939394	6.196472416	0.641723092	0.078296133	+	2358	grey	0.264698103	FALSE	FPR2
Region_9729	chr19	51795157	51826190	31033	18	116	143.2272727	11.77976812	0.885422184	0.030478185	+	2359	grey	-0.351975414	FALSE	FPR3
Region_9730	chr19	51804816	51890950	86134	39	299	339.8939394	18.92993461	0.861877862	0.017077053	-	84765	grey	-0.246415881	FALSE	ZNF577
Region_9731	chr19	51888025	51900463	12438	6	34	54.93939394	7.761599335	0.980836204	0.019887719	+	101928571	grey	-0.073575248	FALSE	ZNF649-AS1
Region_9732	chr19	51889235	51905040	15805	8	58	77.54545455	9.333449883	0.767123259	0.022557474	-	65251	grey	-0.149898854	FALSE	ZNF649
Region_9733	chr19	51927147	51946621	19474	5	27	38.53030303	5.964561231	0.776116367	0.036045116	+	79898	grey	-0.216890341	FALSE	ZNF613
Region_9734	chr19	51949116	51981367	32251	15	98	120.8787879	10.38862566	0.91697851	0.021334287	+	101669766	grey	0.124366233	FALSE	ZNF350-AS1
Region_9735	chr19	51964340	51986856	22516	14	86	120.5909091	13.66520261	0.707798265	0.014790594	-	59348	grey	0.093491456	FALSE	ZNF350
Region_9736	chr19	52031378	52064528	33150	5	21	30.81818182	5.110800301	0.975712755	0.03077051	-	9668	grey	-0.038373983	FALSE	ZNF432
Region_9737	chr1	52048810	52095765	46955	18	109	137.075757	13.8567513	0.65690748	0.01548662	-	284371	grey	0.319560496	FALSE	ZNF841

	9						6	2	2	9						
Region_9738	chr19	52113091	52139938	26847	6	28	44.98484848	6.907058651	0.822628849	0.044311622	-	90317	grey	-0.00877809	FALSE	ZNF616
Region_9739	chr19	52130548	52171643	41095	17	115	143.0909091	12.65121128	0.797256008	0.026232628	-	162962	grey	-0.086300903	FALSE	ZNF836
Region_9740	chr19	52190048	52229518	39470	27	200	237.5909091	14.70319408	0.921568047	0.018552398	+	5518	grey	-0.136832085	FALSE	PPP2R1A
Region_9741	chr19	52269587	52296046	26459	5	17	37.43939394	7.405047733	0.428439994	0.043581065	+	90321	grey	0.277333944	FALSE	ZNF766
Region_9742	chr19	52336243	52367778	31535	4	22	31.3030303	5.235881226	0.699287224	0.045646984	+	162963	grey	0.006453927	FALSE	ZNF610
Region_9743	chr19	52369917	52385795	15878	4	18	24.78787879	4.141776473	0.978918167	0.029845641	+	400713	grey	0.202546339	FALSE	ZNF880
Region_9744	chr19	52397849	52418403	20554	5	16	31.31818182	6.139804785	0.590852449	0.034191777	+	84436	grey	0.018917544	FALSE	ZNF528
Region_9745	chr19	52429148	52452315	23167	8	47	61.27272727	7.826081551	0.80789509	0.042253209	+	147658	grey	-0.131694544	FALSE	ZNF534
Region_9746	chr19	52453553	52516882	63329	17	110	138.4545455	11.81678313	0.937391319	0.022287162	+	147660	grey	0.089721781	FALSE	ZNF578
Region_9747	chr19	52527652	52574130	46478	19	132	158.9848485	11.2174211	0.763616033	0.021938496	+	388558	grey	0.119517041	FALSE	ZNF808
Region_9748	chr19	52570273	52633353	63080	24	150	185.5	13.16901959	0.820931098	0.01255696	+	55762	grey	-0.243361341	FALSE	ZNF701
Region_9749	chr19	52594060	52690504	96444	37	244	277.530303	14.78795736	0.812240394	0.016028249	-	55769	grey	0.197599704	FALSE	ZNF83

Region_9750	chr19	52650447	52690496	40049	8	50	71.21212121	8.006757053	0.65805474	0.047376921	-	122539214	grey	0.340677269	FALSE	LOC122539214
Region_9751	chr19	52702813	52735073	32260	9	51	67.01515152	8.884718816	0.798239443	0.046405432	-	81856	grey	-0.015612498	FALSE	ZNF611
Region_9752	chr19	52749942	52786807	36865	7	32	45.04545455	7.100231455	0.926738725	0.048443224	-	162966	grey	0.253744613	FALSE	ZNF600
Region_9753	chr19	52797408	52857600	60192	24	147	180.1515152	14.56681415	0.932168806	0.016737077	-	7576	grey	0.104331103	FALSE	ZNF28
Region_9754	chr19	52838010	52857649	19639	13	74	106.4090909	12.75445898	0.893413894	0.024315513	-	90333	grey	0.079165236	FALSE	ZNF468
Region_9755	chr19	52860851	52897693	36842	20	148	179.2121212	10.66195992	0.85934718	0.021545798	-	162967	grey	-0.177508732	FALSE	ZNF320
Region_9756	chr19	52904415	52923470	19055	6	45	62.59090909	9.119678255	0.811794459	0.02036717	-	388559	grey	0.008173595	FALSE	ZNF888
Region_9757	chr19	52927135	52942601	15466	7	39	53.09090909	6.616360312	0.8198506	0.041318134	-	399669	grey	0.158158825	FALSE	ZNF321P
Region_9758	chr19	52928475	52962823	34348	17	107	131.6212121	10.60879918	0.896798167	0.030047565	-	100529240	grey	0.037063799	FALSE	ZNF816-ZNF321P
Region_9759	chr19	52949379	52962911	13532	9	54	73.28787879	8.251368467	0.947989516	0.040251217	-	125893	grey	-0.104939059	FALSE	ZNF816
Region_9760	chr19	52956041	53037898	81857	41	255	298.8636364	24.34741267	0.877710709	0.019711545	-	79986	grey	-0.171806763	FALSE	ZNF702P
Region_9761	chr19	53044740	53051680	6940	6	38	51.77272727	7.611318403	0.879484245	0.062643337	+	100271846	grey	-0.269848955	FALSE	ERVV-2
Region_9762	chr1	53066606	53103434	36828	26	183	217.272727	14.4738974	0.84207524	0.03166655	-	90338	grey	-0.22756957	FALSE	ZNF160

	9						3	1	5	4				5		
Region_9763	chr19	53107879	53133077	25198	8	46	59.57575758	8.002039367	0.815663814	0.042759874	-	55786	grey	-0.233547717	FALSE	ZNF415
Region_9764	chr19	53124072	53159075	35003	14	77	100.5454545	9.620781702	0.830613514	0.023021239	-	84671	grey	-0.214075513	FALSE	ZNF347
Region_9765	chr19	53162424	53193386	30962	9	55	74.12121212	8.507942265	0.574869916	0.050919417	-	79788	grey	-0.156882605	FALSE	ZNF665
Region_9766	chr19	53235381	53254898	19517	6	33	54.92424242	9.62418525	0.875615869	0.073634439	-	342926	grey	-0.23472126	FALSE	ZNF677
Region_9767	chr19	53277462	53284947	7485	6	21	36.72727273	6.188190874	0.899396555	0.061746185	+	646508	grey	0.011276327	FALSE	FAM90A27P
Region_9768	chr19	53289601	53291426	1825	4	27	40.90909091	6.70872514	0.805910465	0.074386215	-	112401	grey	0.257745479	FALSE	BIRC8
Region_9769	chr19	53333749	53356906	23157	4	12	25.01515152	5.669058867	0.976406515	0.031582679	+	91664	grey	0.117852601	FALSE	ZNF845
Region_9770	chr19	53365693	53392217	26524	4	24	36.31818182	6.64983306	0.925928596	0.052536415	+	170958	grey	-0.277314695	FALSE	ZNF525
Region_9771	chr19	53389793	53430413	40620	20	124	149.2575758	14.21408901	0.830956733	0.037405655	+	91661	grey	0.257994725	FALSE	ZNF765
Region_9772	chr19	53431984	53444670	12686	8	45	68.66666667	8.851481116	0.72163631	0.074037404	+	147804	grey	0.341921209	FALSE	TPM3P9
Region_9773	chr19	53431984	53461862	29878	17	88	140.9545455	14.72040114	0.83607802	0.044465727	+	388561	grey	0.203562355	FALSE	ZNF761
Region_9774	chr19	53467733	53496255	28522	12	63	81.51515152	10.21347891	0.723397218	0.031225071	+	126017	grey	-0.02025818	FALSE	ZNF813

Region_9775	chr19	53503819	53580269	76450	50	333	382.5151515	22.80704508	0.822888304	0.020119684	+	55422	grey	0.122039438	FALSE	ZNF331
Region_9776	chr19	53599628	53608451	8823	14	68	91.95454545	13.01056591	0.953706263	0.031019976	-	284379	grey	0.161748011	FALSE	LOC284379
Region_9777	chr19	53631999	53637014	5015	6	32	52.6969697	8.557443358	0.935506833	0.046872505	+	503834	grey	0.128785884	FALSE	DPRX
Region_9778	chr19	53793741	53824403	30662	25	159	192.7575758	16.16459627	0.845621299	0.032953244	-	91662	grey	0.244173737	FALSE	NLRP12
Region_9779	chr19	53854581	53869107	14526	6	19	32.86363636	5.499332445	0.485533884	0.045748039	-	105372457	grey	0.037752461	FALSE	MYADM-AS1
Region_9780	chr19	53864763	53876435	11672	12	38	57.3030303	8.566427668	0.14763492	0.027924593	+	91663	grey	0.013981033	FALSE	MYADM
Region_9781	chr19	53879190	53907652	28462	27	139	171	15.31615537	0.499832262	0.025136217	+	5582	grey	-0.001923828	FALSE	PRKCG
Region_9782	chr19	53909278	53943950	34672	20	126	151.6818182	13.37639608	0.78225915	0.03293006	+	59284	grey	0.054414455	FALSE	CACNG7
Region_9783	chr19	53962937	53990215	27278	25	142	176.0606061	15.87729757	0.485327065	0.018397144	+	59283	grey	0.178645674	FALSE	CACNG8
Region_9784	chr19	53991637	54012666	21029	13	67	90.48484848	10.7763879	0.667705258	0.048679885	+	59285	grey	-0.16606542	FALSE	CACNG6
Region_9785	chr19	55006193	55048086	41893	38	222	263.3939394	22.74668448	0.92757092	0.016972529	+	107985325	grey	0.25976016	FALSE	GP6-AS1
Region_9786	chr19	55072020	55087923	15903	14	73	92.25757576	9.262514372	0.609251805	0.041435326	+	54869	grey	-0.271445653	FALSE	EPS8L1
Region_9787	chr1	55090914	55117637	26723	9	47	60.9696969	7.90514865	0.62480678	0.03301920	-	54776	grey	0.116169909	FALSE	PPP1R12C

	9						7	7	2	5						
Region_9788	chr19	55132698	55149206	16508	14	73	94.12121212	9.7436609	0.535168656	0.051275671	-	7138	grey	-0.275111055	FALSE	TNNT1
Region_9789	chr19	55151767	55160671	8904	3	10	25.65151515	4.572467518	0.831650011	0.092310164	-	7137	grey	-0.140753566	FALSE	TNNI3
Region_9790	chr19	55158661	55166722	8061	3	11	16.84848485	3.783536905	0.273304473	0.095209341	-	352909	grey	0.237661462	FALSE	DNAAF3
Region_9791	chr19	55158939	55177540	18601	14	62	83.84848485	10.34072222	0.571046211	0.051571433	+	101930593	grey	0.136296117	FALSE	DNAAF3-AS1
Region_9792	chr19	55171196	55180289	9093	12	44	65.72727273	7.880932109	0.58523665	0.052573425	-	6861	grey	0.232266784	FALSE	SYT5
Region_9793	chr19	55181247	55209506	28259	9	50	69.34848485	7.201479387	0.803630319	0.044690234	-	5794	grey	0.16573826	FALSE	PTPRH
Region_9794	chr19	55229779	55259017	29238	18	111	133.9848485	10.60840367	0.49164323	0.022971355	-	22870	grey	-0.079917358	FALSE	PPP6R1
Region_9795	chr19	55262223	55280381	18158	9	63	76.96969697	7.308519153	0.950025478	0.032445749	-	23640	grey	0.057956322	FALSE	HSPBP1
Region_9796	chr19	55282072	55312562	30490	13	86	112.6363636	9.68525662	0.842116014	0.032456254	+	84446	grey	0.090142466	FALSE	BRSK1
Region_9797	chr19	55312801	55334048	21247	15	74	102.3636364	10.28162867	0.788434831	0.035404487	-	284417	grey	-0.243976338	FALSE	TMEM150B
Region_9798	chr19	55339853	55348121	8268	7	31	47.43939394	7.815426142	0.81802927	0.029628877	+	84787	grey	0.121412266	FALSE	KMT5C
Region_9799	chr19	55349306	55354719	5413	3	10	20.68181818	4.826717601	0.020543844	0.039831515	-	125965	grey	-0.037964012	FALSE	COX6B2

Region_9800	chr19	55352301	55363260	10959	8	40	58.28787879	7.964855789	0.548875103	0.053485165	-	284418	grey	0.017389858	FALSE	GARIN5B
Region_9801	chr19	55379244	55384292	5048	10	51	73.34848485	11.25165618	0.292846888	0.012715305	-	388564	grey	-0.290607069	FALSE	TMEM238
Region_9802	chr19	55385932	55403250	17318	16	94	119.2272727	10.46576169	0.693202103	0.027918388	+	6158	grey	0.088515233	FALSE	RPL28
Region_9803	chr19	55399745	55407788	8043	9	49	66.93939394	8.312879327	0.909705298	0.047613742	-	27338	grey	0.231486744	FALSE	UBE2S
Region_9804	chr19	55428740	55443300	14560	16	86	112.9393939	12.02545862	0.413091482	0.036409006	-	729956	grey	0.25582156	FALSE	SHISA7
Region_9805	chr19	55452985	55462343	9358	14	86	110.8333333	10.8593573	0.608101491	0.029615172	-	79763	grey	-0.149094117	FALSE	ISOC2
Region_9806	chr19	55476617	55484487	7870	11	43	60.63636364	8.473735646	0.857028427	0.040181001	+	89887	grey	-0.134552643	FALSE	ZNF628
Region_9807	chr19	55488404	55519099	30695	29	165	200.5454545	16.02131447	0.75957406	0.035761451	+	284297	grey	-0.027030527	FALSE	SSC5D
Region_9808	chr19	55528611	55537133	8522	7	28	46.12121212	7.610751811	0.904649055	0.05161902	-	646643	grey	-0.074686328	FALSE	SBK2
Region_9809	chr19	55540656	55545543	4887	9	26	46.59090909	7.625821061	0.892390206	0.061966884	-	100130827	grey	0.109706198	FALSE	SBK3
Region_9810	chr19	55591376	55601970	10594	39	173	224.6212121	24.98785253	0.91148562	0.016177364	-	84922	grey	0.053145901	FALSE	FIZ1
Region_9811	chr19	55605647	55617269	11622	20	112	137.530303	11.04845231	0.745202164	0.024714796	+	100507290	grey	0.113958951	FALSE	ZNF865
Region_9812	chr1	55620741	55624566	3825	5	20	28.9545454	4.76019333	0.63339585	0.05811558	-	147808	grey	-0.14416590	FALSE	ZNF784

	9						5	2	1	9				5		
Region_9813	chr19	55635016	55642818	7802	7	31	43.8030303	6.149971099	0.326424153	0.057614796	+	107983998	grey	0.013591578	FALSE	LOC107983998
Region_9814	chr19	55635459	55645623	10164	15	72	96.48484848	11.34527001	0.300943475	0.039912956	+	51545	grey	-0.086194768	FALSE	ZNF581
Region_9815	chr19	55640985	55643470	2485	8	31	51.01515152	8.718666843	0.056800403	0.042829948	+	51157	grey	-0.26532119	FALSE	ZNF580
Region_9816	chr19	55641062	55653161	12099	22	96	125.8939394	13.74395396	0.327342018	0.02554615	+	29903	grey	-0.057601993	FALSE	CCDC106
Region_9817	chr19	55654146	55674716	20570	26	141	178.0606061	15.39718135	0.556930334	0.024899503	+	11338	grey	0.144932588	FALSE	U2AF2
Region_15069	chr5	140801028	141012347	211319	127	974	1211.5	88.19249052	0.517648036	0.028623854	+	56145	blue	0.926819962	FALSE	PCDHA3
Region_9819	chr19	55708438	55738402	29964	24	158	185.0454545	13.74429316	0.896537522	0.024262711	-	338321	grey	-0.082501163	FALSE	NLRP9
Region_9820	chr19	55769118	55773420	4302	5	37	58.27272727	16.93748274	0.82602229	0.060267695	+	729974	grey	0.028466368	FALSE	RFPL4AL1
Region_9821	chr19	55785397	55836800	51403	38	265	296.4090909	17.35420789	0.845909669	0.021143438	-	204801	grey	0.103444316	FALSE	NLRP11
Region_9822	chr19	55836540	55881855	45315	50	282	356.4242424	39.27381614	0.937090147	0.01702911	+	147945	grey	0.069984207	FALSE	NLRP4
Region_9823	chr19	55891699	55932336	40637	19	110	141.2424242	13.02667158	0.813020488	0.038959323	-	126204	grey	-0.025842611	FALSE	NLRP13
Region_9824	chr19	55947832	55988629	40797	35	278	321.0151515	20.78571434	0.879805982	0.024381357	+	126205	grey	-0.358152054	FALSE	NLRP8

Region_9825	chr19	55999726	56061810	62084	51	401	447.4393939	21.66821762	0.817275414	0.025460343	+	126206	grey	-0.401253297	FALSE	NLRP5
Region_9826	chr19	56066684	56078801	12117	11	61	85.74242424	9.892204555	0.815663806	0.028656555	+	101928886	grey	-0.040837018	FALSE	LINC01864
Region_9827	chr19	56087366	56121295	33929	78	424	517.1060606	38.12131598	0.748971833	0.021211132	-	126208	grey	-0.212951092	FALSE	ZNF787
Region_9828	chr19	56132599	56160893	28294	56	323	381.8787879	24.91432406	0.887500851	0.013917916	+	55311	grey	-0.125013193	FALSE	ZNF444
Region_9829	chr19	56176008	56185775	9767	6	27	43.92424242	6.261753517	0.836658494	0.067024475	+	85569	grey	-0.069197865	FALSE	GALP
Region_9830	chr19	56189686	56197815	8129	11	60	78.18181818	11.29316892	0.915731299	0.039009244	-	342933	grey	0.152467843	FALSE	ZSCAN5B
Region_9831	chr19	56202273	56209461	7188	11	46	72.93939394	9.714190711	0.873189934	0.040347681	+	649137	grey	-0.147180978	FALSE	ZSCAN5C
Region_9832	chr19	56219670	56368383	148713	65	468	517.530303	27.0209148	0.799467115	0.017104681	-	79149	grey	0.214764774	FALSE	ZSCAN5A
Region_9833	chr19	56272748	56310454	37706	15	84	111.0909091	11.46194677	0.925448048	0.03727817	+	100506374	grey	0.150922455	FALSE	EDDM13
Region_9834	chr19	56314703	56365316	50613	17	107	140.4393939	12.53564614	0.735445663	0.027746677	+	112268243	grey	0.234997941	FALSE	ZSCAN5A-AS1
Region_9835	chr19	56368099	56379828	11729	4	26	42.36363636	5.908628277	0.47382815	0.028841045	+	147947	grey	0.097616356	FALSE	ZNF542P
Region_9836	chr19	56375846	56393538	17692	3	13	26.86363636	5.317276236	0.629543857	0.044375416	-	147948	grey	0.203797296	FALSE	ZNF582
Region_9837	chr1	56393656	56407060	13404	7	56	73.7575757	8.04039916	0.62391387	0.05855368	+	386758	grey	0.086022694	FALSE	ZNF582-DT

	9						6		4	1						
Region_9838	chr19	56404014	56427364	23350	5	26	38.71212121	5.981420339	0.743310803	0.064160025	+	147949	grey	-0.100923701	FALSE	ZNF583
Region_9839	chr19	56439325	56478065	38740	17	113	147.9393939	14.98602301	0.604069269	0.025637834	-	63934	grey	-0.008727822	FALSE	ZNF667
Region_9840	chr19	56477567	56504362	26795	10	75	92.84848485	9.285625656	0.478475826	0.017451357	+	100128252	grey	0.026022995	FALSE	ZNF667-AS1
Region_9841	chr19	56536156	56538575	2419	7	41	57.43939394	7.579585513	0.106416173	0.031157835	-	101928982	grey	0.179202806	FALSE	ZFP28-DT
Region_9842	chr19	56538948	56556808	17860	10	38	59.39393939	9.248498902	0.394665101	0.033935847	+	140612	grey	0.115636175	FALSE	ZFP28
Region_9843	chr19	56545566	56567441	21875	3	10	20.21212121	4.089442887	0.625109683	0.052805538	-	112268241	grey	0.063413759	FALSE	ZNF470-DT
Region_9844	chr19	56567468	56588911	21443	5	25	37.06060606	6.179068359	0.596526042	0.056371169	+	388566	grey	-0.146456671	FALSE	ZNF470
Region_9845	chr19	56595300	56626481	31181	34	239	276.2424242	19.50073814	0.882059754	0.022361482	+	58491	grey	-0.072500113	FALSE	ZNF71
Region_9846	chr19	56595313	56823395	228082	123	894	989.5606061	37.70457176	0.775769722	0.013319599	+	101929059	grey	-0.403767574	FALSE	ZIM2-AS1
Region_9847	chr19	56595335	56610543	15208	12	81	105.5606061	11.29755425	0.827800924	0.030811007	+	114818278	grey	0.068131588	FALSE	ZNF71-SMIM17
Region_9848	chr19	56643159	56657247	14088	9	51	69.51515152	7.388644977	0.727014801	0.054282383	+	147670	grey	-0.096531913	FALSE	SMIM17
Region_9849	chr19	56661980	56671783	9803	18	134	164.6666667	15.82338419	0.694326465	0.028970664	-	90485	grey	-0.145291799	FALSE	ZNF835

Region_9850	chr19	56669941	56763968	94027	42	323	362.3030303	21.42821267	0.690671421	0.026666694	+	105372473	grey	-0.365272272	FALSE	LOC105372473
Region_9851	chr19	56774547	56840731	66184	19	114	149.5757576	13.05380653	0.682776529	0.051254278	-	23619	grey	-0.238678574	FALSE	ZIM2
Region_9852	chr19	56810077	56840728	30651	15	95	114.969697	11.84641419	0.666074974	0.056985983	-	5178	grey	-0.256907283	FALSE	PEG3
Region_9853	chr19	57119138	57131926	12788	4	21	35.36363636	6.367311626	0.83298232	0.070310592	+	57663	grey	-0.154514153	FALSE	USP29
Region_9854	chr19	57134096	57145202	11106	3	18	30.37878788	5.832970352	0.820766951	0.072779382	-	114026	grey	0.112141719	FALSE	ZIM3
Region_9855	chr19	57191500	57222846	31346	16	112	136.3333333	12.2616768	0.749681024	0.031661825	+	9422	grey	0.059396627	FALSE	ZNF264
Region_9856	chr19	57240632	57262728	22096	6	31	42.92424242	6.084812382	0.963743962	0.037174096	+	390980	grey	0.038069597	FALSE	ZNF805
Region_9857	chr19	57267211	57280334	13123	5	29	40.51515152	6.303216332	0.546962075	0.045868649	-	105372476	grey	0.103215996	FALSE	ZNF460-AS1
Region_9858	chr19	57320472	57330770	10298	3	17	25.72727273	4.408193262	0.65260944	0.021715606	+	125919	grey	0.010195566	FALSE	ZNF543
Region_9859	chr19	57351271	57359898	8627	8	46	65.72727273	10.13530836	0.084428256	0.036315444	+	57343	grey	0.005268459	FALSE	ZNF304
Region_9860	chr19	57363477	57398584	35107	6	42	53.24242424	5.802017172	0.846536669	0.039418659	+	284306	grey	-0.067491529	FALSE	ZNF547
Region_9861	chr19	57389850	57438457	48607	18	132	156.7727273	11.72872274	0.578032151	0.018202511	+	147694	grey	0.24993861	FALSE	ZNF548
Region_9862	chr1	57390008	57421934	31926	14	100	117.863636	9.63628776	0.60868234	0.02090458	+	7565	grey	0.272431046	FALSE	ZNF17

	9						4	9	5	8						
Region_9863	chr19	57435325	57447101	11776	4	24	36.25757576	6.870921125	0.481744658	0.023953696	+	388567	grey	-0.02659018	FALSE	ZNF749
Region_9864	chr19	57437970	57477570	39600	14	77	109.9242424	10.31997407	0.901673874	0.018844608	-	400720	grey	-0.180582298	FALSE	ZNF772
Region_9865	chr19	57487711	57505381	17670	12	55	79.95454545	9.394649571	0.335490343	0.035465689	+	79744	grey	0.008241017	FALSE	ZNF419
Region_9866	chr19	57487718	57518404	30686	16	85	108.5909091	11.38552019	0.546999175	0.021715071	+	374928	grey	-0.152714521	FALSE	ZNF773
Region_9867	chr19	57527325	57557542	30217	12	86	104.8030303	10.28100518	0.723837125	0.015098541	+	256051	grey	-0.129978731	FALSE	ZNF549
Region_9868	chr19	57535257	57559863	24606	6	40	53.81818182	6.722886493	0.961278614	0.025489447	-	162972	grey	-0.183813571	FALSE	ZNF550
Region_9869	chr19	57578456	57593890	15434	12	69	93.01515152	9.993062862	0.383718711	0.036233168	+	284307	grey	0.311424576	FALSE	ZIK1
Region_9870	chr19	57599885	57612722	12837	7	39	57.78787879	8.181621339	0.444404471	0.033008646	+	348327	grey	0.144024472	FALSE	ZNF530
Region_9871	chr19	57614233	57624724	10491	6	32	51.40909091	7.468863105	0.369940187	0.05346181	+	7693	grey	-0.184644331	FALSE	ZNF134
Region_9872	chr19	57630395	57644041	13646	10	46	67.27272727	9.468239142	0.680258247	0.037144665	+	10520	grey	-0.131005167	FALSE	ZNF211
Region_9873	chr19	57668935	57679152	10217	3	13	24	4.94586074	0.927422206	0.04826331	+	201516	grey	-0.086217106	FALSE	ZSCAN4
Region_9874	chr19	57681969	57747072	65103	21	137	162.6666667	13.18779545	0.718238376	0.020338225	+	90233	grey	-0.280436863	FALSE	ZNF551

Region_9875	chr1<br>9	57682045	57758148	76103	23	143	167.484848<br>5	13.4476105<br>1	0.74144422<br>4	0.01846106<br>5	+	284309	grey	-0.29472573<br>6	FALSE	ZNF776
Region_9876	chr1<br>9	57696275	57709204	12929	7	34	50.6818181<br>8	7.13207077<br>7	0.78073905<br>5	0.03617993<br>8	-	7710	grey	-0.32818800<br>5	FALSE	ZNF154
Region_9877	chr1<br>9	57769655	57819939	50284	21	102	129.060606<br>1	14.8612855<br>7	0.95360887<br>5	0.02409821<br>2	+	54807	grey	0.206457845	FALSE	ZNF586
Region_9878	chr1<br>9	57819719	57865117	45398	18	109	142.818181<br>8	15.2859000<br>8	0.79586590<br>5	0.01910340<br>3	+	84914	grey	-0.01441866<br>5	FALSE	ZNF587
Region_9879	chr1<br>9	57819721	57858641	38920	16	91	124.015151<br>5	14.8795082<br>9	0.77207754<br>9	0.02146817<br>2	+	10029351<br>6	grey	0.05643779	FALSE	ZNF587B
Region_9880	chr1<br>9	57850315	57916591	66276	33	241	274.257575<br>8	14.7766037<br>6	0.90928808<br>3	0.01665485	-	730051	grey	-0.14048380<br>1	FALSE	ZNF814
Region_9881	chr1<br>9	57900296	57916610	16314	6	32	44.3787878<br>8	5.95047548<br>6	0.95561523<br>7	0.04806060<br>1	-	147687	grey	0.155005216	FALSE	ZNF417
Region_9882	chr1<br>9	57921882	57935402	13520	4	23	40.8636363<br>6	7.20872625<br>8	0.48725355<br>2	0.03244307<br>4	-	147686	grey	0.359117174	FALSE	ZNF418
Region_9883	chr1<br>9	57958437	57974534	16097	12	72	94.5757575<br>8	10.6930036<br>2	0.92438852<br>3	0.03614244<br>3	-	147685	grey	-0.04975564<br>9	FALSE	C19orf18
Region_9884	chr1<br>9	57977076	58003349	26273	9	62	79.0454545<br>5	7.67900598<br>2	0.86364936<br>8	0.01959775<br>5	-	80095	grey	-0.04426018<br>2	FALSE	ZNF606
Region_9885	chr1<br>9	58002061	58011232	9171	8	44	62.7272727<br>3	9.70574049<br>7	0.38303909<br>8	0.07833208<br>1	+	10012839<br>8	grey	-0.19627745<br>4	FALSE	ZNF606-AS1
Region_9886	chr1<br>9	58034025	58054631	20606	13	70	87.2727272<br>7	8.89519395<br>7	0.77038403<br>6	0.04492359<br>3	+	284312	grey	-0.27419408<br>4	FALSE	ZSCAN1
Region_9887	chr1	58059239	58086310	27071	9	48	61.9545454	6.21024418	0.95210465	0.03113688	+	7694	grey	0.172807286	FALSE	ZNF135

	9						5	7	7	1						
Region_9888	chr19	58083838	58118427	34589	28	178	217.1818182	19.49114915	0.828981053	0.018681988	-	65982	grey	-0.178800456	FALSE	ZSCAN18
Region_9889	chr19	58126248	58155110	28862	9	50	72.06060606	8.657373536	0.861428493	0.023438448	-	79673	grey	-0.058996115	FALSE	ZNF329
Region_9890	chr19	58183029	58213562	30533	8	50	64.90909091	7.601067279	0.814840615	0.025459724	+	10782	grey	0.186506441	FALSE	ZNF274
Region_9891	chr19	58228594	58286807	58213	39	267	320.8636364	25.92289535	0.800307963	0.015286696	+	27300	grey	-0.201711274	FALSE	ZNF544
Region_9892	chr19	58228914	58315197	86283	55	379	445.5151515	30.58216797	0.830940195	0.013168411	+	108903150	grey	-0.12679237	FALSE	ZNF8-ERVK3-1
Region_9893	chr19	58257270	58278830	21560	18	108	138.3484848	15.23608311	0.808132812	0.026828157	-	105372482	grey	-0.238467453	FALSE	ZNF8-DT
Region_9894	chr19	58278955	58302791	23836	19	134	160.6666667	12.8258553	0.868207033	0.015498202	+	7554	grey	0.017953372	FALSE	ZNF8
Region_9895	chr19	58305374	58316812	11438	5	23	41.51515152	6.234500595	0.962679593	0.039181059	+	105372481	grey	0.275819522	FALSE	ERVK3-1
Region_9896	chr19	58309727	58327252	17525	13	93	113.1666667	10.84234339	0.840862209	0.043318828	-	105372480	grey	0.073756999	FALSE	LOC105372480
Region_9897	chr19	58326994	58342332	15338	20	145	194.3787879	18.61576268	0.65139401	0.019185149	+	342945	grey	-0.028840595	FALSE	ZSCAN22
Region_9898	chr19	58345178	58362751	17573	17	106	126.8636364	10.44529539	0.622795483	0.028780868	-	1	grey	-0.172310057	FALSE	A1BG
Region_9899	chr19	58347718	58355455	7737	6	27	40.21212121	6.067606679	0.659615773	0.03966215	+	503538	grey	-0.08636143	FALSE	A1BG-AS1

Region_9900	chr19	58354357	58362848	8491	10	66	87.71212121	9.501036626	0.565785594	0.041928198	-	162968	grey	-0.195564425	FALSE	ZNF497
Region_9901	chr19	58362569	58367620	5051	4	24	39.63636364	6.432217858	0.173677528	0.046570073	+	105372483	grey	-0.30565347	FALSE	ZNF497-AS1
Region_9902	chr19	58367618	58381030	13412	14	90	120.469697	12.20242056	0.742634262	0.025615765	-	116412	grey	-0.30621754	FALSE	ZNF837
Region_9903	chr19	58386400	58394806	8406	9	52	71.25757576	9.013341575	0.317233421	0.027097506	+	6193	grey	0.098904883	FALSE	RPS5
Region_9904	chr19	58401504	58418327	16823	11	48	71.8030303	7.760292812	0.617460648	0.032367533	+	201514	grey	-0.096359603	FALSE	ZNF584
Region_9905	chr19	58467056	58475436	8380	9	46	63.06060606	8.12380886	0.752402692	0.026818893	+	25799	grey	0.147774556	FALSE	ZNF324
Region_9906	chr19	58474017	58481230	7213	11	67	96.87878788	13.69167647	0.439234062	0.02217582	+	55663	grey	0.079204337	FALSE	ZNF446
Region_9907	chr19	58479512	58511992	32480	21	136	173.0909091	13.88715762	0.707735559	0.029152455	-	10998	grey	-0.303975552	FALSE	SLC27A5
Region_9908	chr19	58513530	58538911	25381	46	215	298.7272727	29.6100531	0.337773282	0.018977407	-	84878	grey	0.094587467	FALSE	ZBTB45
Region_9909	chr19	58544064	58550722	6658	18	90	136.969697	18.6679237	0.114701642	0.009356636	+	10155	grey	0.087006827	FALSE	TRIM28
Region_9910	chr19	58551452	58555105	3653	4	17	30.09090909	5.496088883	0.559353231	0.09786132	-	27243	grey	-0.006390929	FALSE	CHMP2A
Region_9911	chr19	58555712	58558954	3242	7	19	37.53030303	7.390679571	0.526108761	0.04341608	-	9040	grey	0.006722032	FALSE	UBE2M
Region_9912	chr1	58559129	58574797	15668	21	115	140.318181	13.4840750	0.37173023	0.02974537	+	10013169	grey	0.066140424	FALSE	MZF1-AS1

	9						8	4	4	5		1				
Region_9913	chr19	58561932	58573575	11643	18	89	115.2424242	11.63953555	0.397180692	0.033979769	-	7593	grey	0.107336439	FALSE	MZF1
Region_9914	chr19	58573503	58605223	31720	10	37	78.16666667	16.84461414	0.862109666	0.070213231	+	65996	grey	-0.335588967	FALSE	CENPBD2P
Region_9915	chr19	58582596	58601316	18720	7	19	49.15151515	14.78798101	0.80834085	0.102637753	+	107987272	grey	-0.329461395	FALSE	LOC107987272
Region_9916	chr2	38814	46870	8056	21	91	148.2272727	30.61412216	0.085293838	0.027274568	-	642273	grey	-0.081862131	FALSE	FAM110C
Region_9917	chr2	218136	264866	46730	28	107	182.7878788	22.98395102	0.367958056	0.03011262	-	26751	grey	-0.302667472	FALSE	SH3YL1
Region_9918	chr2	264869	278283	13414	10	37	64.56060606	13.22832484	0.264972818	0.022723444	+	52	grey	-0.043401196	FALSE	ACP1
Region_9919	chr2	279558	288851	9293	22	99	139.7727273	20.46670501	0.104670019	0.01696523	-	285016	grey	0.179325549	FALSE	ALKAL2
Region_9920	chr2	286419	301515	15096	28	109	163.0757576	24.86041686	0.054945717	0.013886356	+	101927262	grey	0.029476877	FALSE	LOC101927262
Region_9921	chr2	317912	342118	24206	4	13	28.22727273	6.1987479	0.927004115	0.060932022	+	105373350	grey	0.151996756	FALSE	LINC01865
Region_9922	chr2	490944	503905	12961	23	108	152.5606061	24.23546593	0.929246974	0.034631973	-	727944	grey	-0.044576624	FALSE	LINC01874
Region_9923	chr2	558153	578145	19992	5	30	44.72727273	6.482035422	0.936495709	0.04144376	+	105373352	grey	0.089825588	FALSE	LOC105373352
Region_9924	chr2	663877	677406	13529	8	36	54.37878788	8.144399022	0.615613942	0.02809989	-	129787	grey	0.175008701	FALSE	TMEM18

Region_9925	chr2	677186	697371	20185	10	56	76.5	9.81247244	0.478840247	0.02159872	+	105373354	grey	0.035663363	FALSE	TMEM18-DT
Region_9926	chr2	1374223	1543711	169488	78	564	626	29.45922867	0.859146404	0.017901296	+	7173	grey	-0.142909198	FALSE	TPO
Region_9927	chr2	1631887	1744852	112965	79	547	630.2121212	35.34294191	0.858902922	0.014069074	-	7837	grey	0.203240242	FALSE	PXDN
Region_9928	chr2	1789113	2331664	542551	231	1844	1978.818182	59.99946387	0.834654892	0.012036672	-	23040	grey	0.176451586	FALSE	MYT1L
Region_9929	chr2	2641989	2696442	54453	29	210	250.4393939	16.32282855	0.847680509	0.02519488	-	107985839	grey	0.187976237	FALSE	LOC107985839
Region_9930	chr2	2707368	2840512	133144	78	537	619.4393939	38.15197496	0.862814046	0.019391437	-	105373390	grey	0.362008753	FALSE	LOC105373390
Region_9931	chr2	2895048	3126026	230978	83	585	667.6212121	35.34392131	0.835793911	0.022699609	-	101927554	grey	-0.252828662	FALSE	LINC01250
Region_9932	chr2	3131581	3145780	14199	7	50	65.87878788	7.917876855	0.963071745	0.020242555	-	107985840	grey	-0.025931711	FALSE	LOC107985840
Region_9933	chr2	3188925	3377830	188905	129	948	1061.424242	49.32843168	0.915669522	0.010756817	-	7260	grey	-0.174466092	FALSE	EIPR1
Region_9934	chr2	3594832	3644644	49812	53	365	418.6666667	20.5009068	0.911814263	0.016169918	+	78989	grey	0.187740707	FALSE	COLEC11
Region_9935	chr2	3658200	3702671	44471	21	160	192.5454545	17.10257004	0.901855623	0.028370156	+	55821	grey	0.013982648	FALSE	ALLC
Region_9936	chr2	3702585	3704153	1568	6	20	40.13636364	7.00304629	0.316549056	0.087783726	-	105373393	grey	0.149698135	FALSE	LOC105373393
Region_9937	chr2	3703575	3848008	144433	60	420	494.924242	24.6927648	0.75685138	0.02586950	+	728597	grey	0.057741352	FALSE	DCDC2C

							4	1		2						
Region_9938	chr2	3957652	3974124	16472	10	61	88.2121212<br>1	10.0007925<br>1	0.82950064<br>6	0.04374381<br>9	-	10050596<br>4	grey	0.191846548	FALSE	LINC01304
Region_9939	chr2	5618327	5691118	72791	17	99	124.954545<br>5	12.6727227<br>6	0.74970296<br>3	0.03839434<br>3	-	10272381<br>8	grey	0.172341218	FALSE	LINC01248
Region_9940	chr2	5692384	5701385	9001	8	32	49.1363636<br>4	7.03373489<br>5	0.03670344<br>4	0.03394976<br>3	+	6664	grey	-0.00177003<br>8	FALSE	SOX11
Region_9941	chr2	5932543	6003510	70967	10	65	82.2272727<br>3	9.15136888<br>4	0.90904343<br>3	0.04359297<br>7	+	150622	grey	0.128846324	FALSE	SILC1
Region_9942	chr2	5932721	6003508	70787	10	65	82.2272727<br>3	9.15136888<br>4	0.90904343<br>3	0.04359297<br>7	+	400940	grey	0.128846324	FALSE	LOC400940
Region_9943	chr2	6258348	6341788	83440	6	39	53.7272727<br>3	8.52966850<br>2	0.89311717<br>5	0.04990987<br>4	-	10537340<br>1	grey	0.068220212	FALSE	LOC105373401
Region_9944	chr2	6495651	6511839	16188	4	22	33.1969697	5.27175258<br>3	0.82728899<br>4	0.08040406<br>8	+	10798584<br>4	grey	0.198270907	FALSE	LINC01824
Region_9945	chr2	6728177	6770311	42134	6	35	58.0454545<br>5	7.44090941<br>1	0.95111437<br>1	0.03201961<br>1	-	400941	grey	-0.11558275<br>1	FALSE	LINC00487
Region_9946	chr2	6840570	6866635	26065	3	16	25.8939393<br>9	4.14405519<br>7	0.77305588<br>6	0.0848933	-	129607	grey	-0.02887070<br>9	FALSE	CMPK2
Region_9947	chr2	6865557	6898239	32682	3	16	24.8636363<br>6	4.66372701<br>3	0.94446916<br>7	0.05393401<br>1	+	91543	grey	0.103680866	FALSE	RSAD2
Region_9948	chr2	6911754	6918734	6980	8	32	60.8636363<br>6	8.87847243<br>2	0.34895428<br>3	0.03392597<br>6	-	386597	grey	-0.07963582<br>7	FALSE	GRASLND
Region_9949	chr2	6917412	7068286	150874	35	266	307.818181<br>8	21.2116869<br>8	0.72252324<br>9	0.02012567<br>2	+	9781	grey	0.191840083	FALSE	RNF144A

Region_9950	chr2	7045329	7077916	32587	7	41	58.0303030<br>3	7.56504043<br>8	0.82099850<br>9	0.02677475	-	10192945<br>2	grey	-0.06325726<br>1	FALSE	LOC101929452
Region_9951	chr2	7383227	7429427	46200	7	44	63.6363636<br>4	8.42822431<br>1	0.82453791<br>8	0.04357040<br>6	+	10537340<br>6	grey	0.148278277	FALSE	LOC105373406
Region_9952	chr2	7421246	7450544	29298	9	63	89.1666666<br>7	9.57654720<br>7	0.40397682<br>2	0.03337563<br>1	+	10050627<br>4	grey	-0.03379696<br>2	FALSE	LOC100506274
Region_9953	chr2	7922425	8278186	355761	63	505	549.666666<br>7	26.1159151<br>8	0.89249823<br>8	0.01572327<br>2	-	339788	grey	0.019831452	FALSE	LINC00298
Region_9954	chr2	7988683	8383819	395136	81	642	699.666666<br>7	29.7612723<br>7	0.87849539<br>4	0.01590564<br>9	-	339789	grey	0.315333923	FALSE	LINC00299
Region_9955	chr2	8461703	8592903	131200	44	323	366.075757<br>6	18.6797386	0.78383481<br>9	0.01923214	-	10192956<br>7	grey	0.050642292	FALSE	LINC01814
Region_9956	chr2	8600892	8622942	22050	5	30	40.6060606<br>1	5.90665540<br>6	0.94472718<br>5	0.03103260<br>3	-	10537341<br>1	grey	-0.09143696<br>3	FALSE	LOC105373411
Region_9957	chr2	8666636	8681864	15228	10	55	76.9242424<br>2	8.46769536<br>5	0.57661548<br>3	0.02187522<br>7	-	10050629<br>9	grey	-0.03781808<br>3	FALSE	ID2-AS1
Region_9958	chr2	8678845	8684461	5616	5	34	45.3939393<br>9	6.43279766<br>4	0.18947254<br>8	0.02379796<br>3	+	3398	grey	0.043872097	FALSE	ID2
Region_9959	chr2	8721081	8837630	116549	35	249	277.469697	15.2267171	0.88775941<br>7	0.01575946<br>9	-	57498	grey	-0.19037479<br>5	FALSE	KIDINS220
Region_9960	chr2	8852690	9003709	151019	28	214	247.772727<br>3	15.0126054<br>3	0.85486580<br>5	0.01882420<br>7	-	129642	grey	-0.06647553<br>6	FALSE	MBOAT2
Region_9961	chr2	9110457	9116947	6490	3	14	24.8484848<br>5	5.30092132<br>6	0.85144424	0.07684294<br>9	+	10537341<br>6	grey	0.16117582	FALSE	LOC105373416
Region_9962	chr2	9206765	9405683	198918	87	623	724.606060	35.9790019	0.90747612	0.01024937	+	8853	grey	0.049539546	FALSE	ASAP2

							6	1	6	1						
Region_9963	chr2	9403475	9423528	20053	9	55	72.93939394	8.840255457	0.817540421	0.032684274	-	9270	grey	0.02385062	FALSE	ITGB1BP1
Region_9964	chr2	9423651	9473101	49450	24	155	181.0909091	13.77481232	0.891909633	0.015541599	+	51692	grey	0.36319645	FALSE	CPSF3
Region_9965	chr2	9473658	9512413	38755	19	116	140	11.21537406	0.606296632	0.025679686	+	285148	grey	-0.087665775	FALSE	IAH1
Region_9966	chr2	9488486	9556732	68246	26	160	199.4393939	14.65724162	0.757966742	0.01674553	-	6868	grey	0.058471375	FALSE	ADAM17
Region_9967	chr2	9583967	9630997	47030	18	110	135.9090909	13.16957715	0.623804889	0.02086295	-	10971	grey	-0.018681939	FALSE	YWHAQ
Region_9968	chr2	9638707	9712920	74213	18	118	150.3333333	13.16794866	0.898428136	0.028865179	+	105373418	grey	-0.32727567	FALSE	LOC105373418
Region_9969	chr2	9757496	9770341	12845	5	30	41.39393939	5.686363683	0.710411374	0.050768428	-	100996549	grey	0.228640467	FALSE	LOC100996549
Region_9970	chr2	9843443	9934416	90973	25	163	209.9090909	16.23739963	0.940745573	0.028530997	+	9014	grey	0.094155832	FALSE	TAF1B
Region_9971	chr2	9939088	9950817	11729	15	91	114	10.51299562	0.840510152	0.045917331	-	105373422	grey	-0.157530231	FALSE	LOC105373422
Region_9972	chr2	9951693	10002277	50584	25	159	196.2727273	17.08939883	0.631896372	0.019997737	+	29841	grey	0.300314207	FALSE	GRHL1
Region_9973	chr2	10042849	10054836	11987	5	23	34.98484848	5.520569715	0.924794489	0.045291044	+	8462	grey	-0.015007178	FALSE	KLF11
Region_9974	chr2	10056473	10080944	24471	8	42	59.18181818	6.773044063	0.835833657	0.040549931	-	192668	grey	0.008897026	FALSE	CYS1

Region_9975	chr2	10120698	10211725	91027	87	592	670.6212121	29.95266273	0.905155449	0.013517315	+	6241	grey	0.134596919	FALSE	RRM2
Region_9976	chr2	10302889	10427617	124728	109	801	893.1515152	41.62441681	0.706276158	0.012189258	+	3241	grey	-0.126921001	FALSE	HPCAL1
Region_9977	chr2	10439968	10448327	8359	8	59	79.43939394	9.321816904	0.819323918	0.040804754	-	4953	grey	-0.118559664	FALSE	ODC1
Region_9978	chr2	10448682	10457693	9011	5	24	40.34848485	6.296500675	0.900324581	0.050244501	+	101929715	grey	0.085545196	FALSE	ODC1-DT
Region_9979	chr2	10562347	10689987	127640	39	261	297.6515152	16.36740892	0.91414709	0.01426234	-	79954	grey	0.314274037	FALSE	NOL10
Region_9980	chr2	10721100	10785110	64010	26	145	177.8787879	13.85697842	0.79358082	0.024042582	+	245973	grey	0.313815321	FALSE	ATP6V1C2
Region_9981	chr2	10783391	10837977	54586	19	111	139.4848485	14.01893491	0.707548466	0.032205214	-	10130	grey	0.104912248	FALSE	PDIA6
Region_9982	chr2	10844224	10885152	40928	22	151	182.8636364	14.33112413	0.812763665	0.02725027	+	101929733	grey	-0.289480651	FALSE	LINC01954
Region_9983	chr2	10911934	10914225	2291	9	46	65.31818182	9.947800825	0.360632235	0.053294383	+	3754	grey	0.178248268	FALSE	KCNF1
Region_9984	chr2	11105317	11132821	27504	12	64	86.71212121	10.56083498	0.788254535	0.033742908	-	285150	grey	-0.264406009	FALSE	FLJ33534
Region_9985	chr2	11133128	11151317	18189	9	49	67.63636364	7.697424871	0.730422774	0.063134838	+	130813	grey	0.148342718	FALSE	C2orf50
Region_9986	chr2	11155198	11178870	23672	11	71	90.72727273	10.02695667	0.850097356	0.026336522	+	130814	grey	-0.143677076	FALSE	SLC66A3
Region_9987	chr2	11179759	11348330	168571	31	195	228.287878	15.1949031	0.90805568	0.02518415	-	9475	grey	-0.02713017	FALSE	ROCK2

							8	4	4	1				3		
Region_9988	chr2	11372612	11403175	30563	8	42	60.2121212<br>1	8.19665063<br>2	0.94924383<br>3	0.03460830<br>9	+	10087405<br>5	grey	0.231999223	FALSE	LINC00570
Region_9989	chr2	11405508	11427186	21678	5	32	47.9545454<br>5	6.24975524	0.90808299<br>7	0.04901627<br>9	+	10537342<br>9	grey	0.074873254	FALSE	LOC105373429
Region_9990	chr2	11444375	11466177	21802	4	24	37.5606060<br>6	4.94626133<br>2	0.95415150<br>4	0.03605734<br>8	-	1876	grey	0.144633777	FALSE	E2F6
Region_9991	chr2	11482341	11642788	160447	105	736	840.909090<br>9	36.8713886<br>5	0.80224206<br>1	0.01426605<br>7	+	9687	grey	-0.16015561<br>5	FALSE	GREB1
Region_9992	chr2	11677595	11827409	149814	64	488	538.090909<br>1	21.2875747<br>6	0.87143684<br>2	0.01261006<br>8	+	23175	grey	0.100105102	FALSE	LPIN1
Region_9993	chr2	11833926	11866284	32358	16	92	115.151515<br>2	10.9576	0.75932781<br>2	0.04473883<br>2	+	10537343<br>0	grey	-0.07552481<br>2	FALSE	LOC105373430
Region_9994	chr2	11865885	12702913	837028	132	1008	1100.18181<br>8	37.2380736<br>1	0.85129539<br>8	0.01150895<br>1	+	10050645<br>7	grey	-0.14927530<br>3	FALSE	MIR3681HG
Region_9995	chr2	12716910	12742734	25824	8	48	66.6363636<br>4	8.64627329<br>9	0.42827953<br>4	0.04424079<br>7	+	28951	grey	-0.19112313<br>9	FALSE	TRIB2
Region_9996	chr2	12780593	13007029	226436	17	98	122.651515<br>2	12.1027554<br>7	0.76899970<br>9	0.03258415<br>1	-	10050647<br>4	grey	0.094074699	FALSE	LOC100506474
Region_9997	chr2	13000953	13331683	330730	34	241	282.833333<br>3	18.6914899<br>1	0.83525018	0.02624929<br>2	+	10537343<br>6	grey	-0.23530153<br>6	FALSE	LOC105373436
Region_9998	chr2	13710531	14400963	690432	48	345	407.136363<br>6	22.6076136<br>7	0.83775150<br>5	0.02523206<br>1	-	10049917<br>1	grey	0.313153723	FALSE	LINC00276
Region_9999	chr2	13723048	13758152	35104	3	12	21.0757575<br>8	4.78558928<br>5	0.80334177<br>6	0.09871603<br>7	+	10537343<br>8	grey	0.26698582	FALSE	LOC105373438

Region_10000	chr2	14632700	14650814	18114	7	36	54.31818182	7.787878334	0.348581563	0.047778019	+	151354	grey	-0.331335088	FALSE	LRATD1
Region_10001	chr2	15166916	15561340	394424	48	361	436.0606061	25.54204906	0.90950144	0.012854498	-	51594	grey	0.220267771	FALSE	NBAS
Region_10002	chr2	15591178	15634346	43168	7	33	49.74242424	6.421863152	0.701723621	0.022706699	+	1653	grey	0.11490782	FALSE	DDX1
Region_10003	chr2	15690782	15744339	53557	8	46	61.71212121	8.580416256	0.881080662	0.047323782	+	101926966	grey	-0.134346473	FALSE	LINC01804
Region_10004	chr2	15918350	15942249	23899	11	67	85.03030303	10.28063107	0.338281934	0.020144103	-	10408	grey	-0.007250948	FALSE	MYCNOS
Region_10005	chr2	15920399	15936017	15618	3	15	25.63636364	4.498329138	0.919877591	0.051119606	+	103752554	grey	-0.005767112	FALSE	MYCNUT
Region_10006	chr2	15940550	15947007	6457	7	34	54.1969697	8.463895624	0.12458241	0.021404185	+	4613	grey	-0.039073588	FALSE	MYCN
Region_10007	chr2	16013928	16087201	73273	26	188	213.5454545	13.13970122	0.852227137	0.02526966	+	104797537	grey	0.11184523	FALSE	GACAT3
Region_10008	chr2	16354256	16432702	78446	14	84	108.5909091	10.24916848	0.900390842	0.037771609	+	107985855	grey	-0.029862653	FALSE	LOC107985855
Region_10009	chr2	16452112	16478623	26511	4	36	49.33333333	6.99963369	0.923655567	0.045545113	-	105373444	grey	-0.179552697	FALSE	LOC105373444
Region_10010	chr2	16549459	16666331	116872	14	105	126.8030303	10.92100263	0.895863247	0.037002	-	81553	grey	0.045906734	FALSE	CYRIA
Region_10011	chr2	17539126	17657018	117892	4	26	36.09090909	4.54288039	0.640465226	0.049481038	+	7447	grey	0.147305549	FALSE	VSNL1
Region_1001	chr2	17663812	17800195	136383	14	102	121.333333	9.50195661	0.84003689	0.01992868	-	79677	grey	0.086221159	FALSE	SMC6

2							3	4	8	9						
Region_10013	chr2	17741679	17800242	58563	9	64	80.72727273	8.529668502	0.865144802	0.024609548	-	105373449	grey	0.166980127	FALSE	LOC105373449
Region_10014	chr2	17753858	17788946	35088	8	58	74.71212121	7.968717977	0.854564369	0.022299007	+	348654	grey	0.376324498	FALSE	GEN1
Region_10015	chr2	17877847	18361616	483769	40	294	338.6818182	16.62913022	0.795161342	0.02744583	+	3790	grey	0.027457821	FALSE	KCNS3
Region_10016	chr2	18403967	18504484	100517	7	40	55.04545455	7.47391735	0.897731024	0.041188233	+	105373454	grey	0.037077259	FALSE	LOC105373454
Region_10017	chr2	18555545	18589564	34019	3	16	25.75757576	5.026271308	0.941712159	0.058320547	-	100526794	grey	-0.002132431	FALSE	NT5C1B-RDH14
Region_10018	chr2	18562871	18589572	26701	3	16	25.75757576	5.026271308	0.941712159	0.058320547	-	93034	grey	-0.002132431	FALSE	NT5C1B
Region_10019	chr2	18986451	19348067	361616	26	178	214.469697	14.53401284	0.817622895	0.030542461	-	400945	grey	-0.338306929	FALSE	LINC01376
Region_10020	chr2	19044866	19346748	301882	18	121	147.3181818	11.77240462	0.835536801	0.0400252	-	105373455	grey	-0.28650412	FALSE	
Region_10021	chr2	19351485	19358623	7138	4	24	37.71212121	5.991699776	0.057580355	0.04051097	-	130497	grey	-0.231310876	FALSE	OSR1
Region_10022	chr2	19910263	19990105	79842	12	96	113.6818182	9.910614498	0.729306128	0.023314552	-	57539	grey	0.311215951	FALSE	WDR35
Region_10023	chr2	19990209	20004795	14586	3	13	23.01515152	5.024531893	0.319036267	0.04178585	+	101928222	grey	-0.043830253	FALSE	WDR35-DT
Region_10024	chr2	19992052	20012668	20616	5	26	38.12121212	5.327765159	0.933645046	0.044656674	-	4148	grey	0.103950973	FALSE	MATN3

Region_10025	chr2	20200797	20225433	24636	14	81	108.0606061	10.66825454	0.836273305	0.037614401	-	6382	grey	-0.241808554	FALSE	SDC1
Region_10026	chr2	20248691	20352234	103543	19	102	143.4848485	12.77647167	0.965260852	0.019591706	-	23369	grey	-0.078771988	FALSE	PUM2
Region_10027	chr2	20447074	20449440	2366	3	16	29.28787879	5.202854694	0.036431589	0.032249424	+	388	grey	-0.155688186	FALSE	RHOB
Region_10028	chr2	20560448	20651130	90682	47	311	360	21.80119968	0.730953041	0.020044492	-	64342	grey	0.170730934	FALSE	HS1BP3
Region_10029	chr2	20667144	20679243	12099	7	40	58.39393939	7.657834174	0.493440268	0.088963129	+	151449	grey	-0.013777356	FALSE	GDF7
Region_10030	chr2	20684014	20823130	139116	24	172	199.4242424	12.6601259	0.742301425	0.018522132	-	60526	grey	-0.173552733	FALSE	LDAH
Region_10031	chr2	21001429	21044073	42644	7	54	66.33333333	6.794417014	0.921453291	0.032442578	-	338	grey	0.493284955	FALSE	APOB
Region_10032	chr2	21221169	21264086	42917	7	35	47.77272727	6.46837384	0.828526131	0.058000105	+	105374317	grey	0.189841266	FALSE	LOC105374317
Region_10033	chr2	21221175	21970959	749784	81	645	713.2878788	25.5504118	0.812211123	0.021060306	+	105374320	grey	0.161023658	FALSE	LOC105374320
Region_10034	chr2	21317660	21561313	243653	21	162	188.3636364	12.6838921	0.825000612	0.033732936	+	105374318	grey	0.226345235	FALSE	LOC105374318
Region_10035	chr2	21607465	21649572	42107	3	13	22.96969697	5.146977297	0.858022624	0.095421504	-	101929230	grey	-0.057037453	FALSE	LOC101929230
Region_10036	chr2	21933336	22536322	602986	42	339	391.2727273	19.19418618	0.818973853	0.025525091	-	105374322	grey	-0.030749354	FALSE	LINC01830
Region_1003	chr2	22508129	22548800	40671	5	26	49.1666666	7.50299085	0.72777540	0.08336834	+	10272336	grey	0.109441774	FALSE	LINC01884

7							7	7	7	5		2				
Region_10038	chr2	23018125	23199056	180931	19	142	173.4393939	12.31650398	0.843575932	0.036046864	-	107985792	grey	0.328436559	FALSE	LOC107985792
Region_10039	chr2	23357516	23381299	23783	3	16	27.95454545	4.972019612	0.912061363	0.054081391	-	105374323	grey	0.119330188	FALSE	LINC02923
Region_10040	chr2	23385179	23708611	323432	131	969	1073.272727	49.64165294	0.80949295	0.012018215	+	114818	grey	-0.016698745	FALSE	KLHL29
Region_10041	chr2	23507043	23524344	17301	9	49	75.40909091	7.866144149	0.852313678	0.053397792	-	105374325	grey	0.195241989	FALSE	LOC105374325
Region_10042	chr2	23748664	23927123	178459	56	403	451.8181818	22.5442533	0.869853725	0.016336691	-	54454	grey	0.437121018	FALSE	ATAD2B
Region_10043	chr2	23927285	24004909	77624	18	100	134.8939394	11.77236502	0.86598319	0.016970828	+	165324	grey	0.06590841	FALSE	UBXN2A
Region_10044	chr2	24010081	24026775	16694	3	14	23.74242424	4.946826818	0.721509837	0.090146261	+	388931	grey	0.333685142	FALSE	MFSD2B
Region_10045	chr2	24024153	24063681	39528	8	42	58.75757576	8.650585762	0.853627904	0.03462171	+	2281	grey	0.197469012	FALSE	FKBP1B
Region_10046	chr2	24029347	24049575	20228	5	23	37.86363636	6.511259747	0.966429585	0.035068371	-	80304	grey	0.166682964	FALSE	WDCP
Region_10047	chr2	24067586	24076373	8787	3	17	30.60606061	5.019588668	0.426654112	0.07110647	-	51639	grey	0.202342221	FALSE	SF3B6
Region_10048	chr2	24076526	24190436	113910	38	263	296.1363636	18.77632418	0.798739556	0.020604477	+	375190	grey	0.229566897	FALSE	FAM228B
Region_10049	chr2	24114809	24123464	8655	3	17	25.65151515	3.971019727	0.652449552	0.029598528	-	375189	grey	-0.110919773	FALSE	PFN4

Region_10050	chr2	24172798	24200849	28051	13	69	100.0151515	11.05718343	0.861517486	0.048555169	+	653140	grey	0.000906534	FALSE	FAM228A
Region_10051	chr2	24202864	24360536	157672	36	250	285.1515152	16.43564833	0.885362585	0.014127565	-	50618	grey	0.074522144	FALSE	ITSN2
Region_10052	chr2	24402995	24413292	10297	3	19	28.22727273	5.062062379	0.948951451	0.05191178	+	105374329	grey	-0.043373503	FALSE	LOC105374329
Region_10053	chr2	24491254	24770702	279448	52	333	407.8030303	23.98410993	0.940835488	0.01299906	+	8648	grey	-0.184708016	FALSE	NCOA1
Region_10054	chr2	24793136	24822376	29240	4	14	27.25757576	5.327174474	0.979699961	0.024524198	+	79172	grey	0.193143719	FALSE	CENPO
Region_10055	chr2	24819169	24920237	101068	23	146	175.9090909	12.82512831	0.869096832	0.025620788	-	109	grey	-0.147241119	FALSE	ADCY3
Region_10056	chr2	24943636	24972094	28458	3	15	25.86363636	4.529854349	0.629652258	0.038536017	-	51277	grey	0.023531838	FALSE	DNAJC27
Region_10057	chr2	24971442	25039716	68274	22	140	162.9242424	12.34916454	0.835292275	0.021358252	+	729723	grey	-0.213085369	FALSE	DNAJC27-AS1
Region_10058	chr2	25042076	25159135	117059	74	511	575.7575758	29.3126702	0.857411346	0.015303705	+	22979	grey	-0.034907663	FALSE	EFR3B
Region_10059	chr2	25160853	25168903	8050	7	31	49.51515152	6.968594217	0.202575644	0.066201519	-	5443	grey	0.103600827	FALSE	POMC
Region_10060	chr2	25227855	25342590	114735	76	475	530.3939394	23.64342731	0.711430708	0.017993953	-	1788	grey	-0.510104265	FALSE	DNMT3A
Region_10061	chr2	25377198	25673647	296449	111	818	869.5	29.40865911	0.924456587	0.011269169	-	1838	grey	0.019003917	FALSE	DTNB
Region_1006	chr2	25733753	25878487	144734	36	234	259.575757	15.7011127	0.81991139	0.01561943	-	55252	grey	-0.14155205	FALSE	ASXL2

2							6	5	9	8				7		
Region_10063	chr2	25926598	25982749	56151	20	115	144.7424242	12.06687852	0.880179955	0.016765859	-	3797	grey	0.20689091	FALSE	KIF3C
Region_10064	chr2	26034084	26137454	103370	19	127	147.9848485	11.14035274	0.912157876	0.021483538	+	10890	grey	-0.236273894	FALSE	RAB10
Region_10065	chr2	26173088	26189663	16575	17	83	108.1666667	10.59111868	0.505887665	0.024573517	+	150946	grey	0.028490893	FALSE	GAREM2
Region_10066	chr2	26190635	26244672	54037	16	120	144.2121212	9.699349941	0.65979529	0.025553488	-	3030	grey	-0.095413568	FALSE	HADHA
Region_10067	chr2	26243170	26290465	47295	24	169	196.8484848	13.69474062	0.739854969	0.021100215	+	3032	grey	-0.099492251	FALSE	HADHB
Region_10068	chr2	26308173	26346817	38644	13	83	111.6515152	10.40191169	0.647300385	0.019903951	-	165082	grey	0.235846994	FALSE	ADGRF3
Region_10069	chr2	26308547	26395885	87338	26	211	240.6818182	15.55749544	0.79407193	0.014696092	+	85465	grey	0.079643021	FALSE	SELENOI
Region_10070	chr2	26401920	26456711	54791	23	154	182.2727273	13.10495556	0.899622803	0.021178828	+	92749	grey	-0.104973414	FALSE	DRC1
Region_10071	chr2	26457203	26558756	101553	49	303	357.030303	21.97828978	0.850656736	0.022714149	-	9381	grey	-0.181737131	FALSE	OTOF
Region_10072	chr2	26562585	26579532	16947	10	64	87.8030303	8.872116558	0.570655116	0.048808427	+	339778	grey	0.335586383	FALSE	CIMIP2C
Region_10073	chr2	26581205	26641366	60161	22	153	182.4545455	12.66513302	0.856836293	0.026103626	-	130106	grey	-0.025494832	FALSE	CIB4
Region_10074	chr2	26692722	26733420	40698	22	122	154.6666667	15.34559153	0.843562941	0.034369633	+	3777	grey	0.366049646	FALSE	KCNK3

Region_10075	chr2	26764284	26781231	16947	4	27	37.34848485	5.130579287	0.789624791	0.045375268	+	54978	grey	-0.069475003	FALSE	SLC35F6
Region_10076	chr2	26764289	26801067	36778	19	144	179.0454545	16.67834323	0.414917806	0.016367351	+	1058	grey	0.11053603	FALSE	CENPA
Region_10077	chr2	26847747	26950351	102604	38	255	294.1818182	16.31507947	0.724575804	0.02155319	+	56896	grey	0.111556323	FALSE	DPYSL5
Region_10078	chr2	26970637	27027219	56582	17	102	131.2424242	11.96669816	0.841562794	0.026160115	+	22924	grey	-0.012410479	FALSE	MAPRE3
Region_10079	chr2	26984776	27014612	29836	9	45	63.90909091	7.246701685	0.732706849	0.044502969	-	100129724	grey	0.008256674	FALSE	MAPRE3-AS1
Region_10080	chr2	27032910	27041694	8784	8	50	66.84848485	8.171700556	0.688936456	0.039499056	+	54867	grey	0.140726632	FALSE	TMEM214
Region_10081	chr2	27042364	27070622	28258	20	130	157.8181818	13.40253603	0.633149183	0.018718807	+	60509	grey	0.187127868	FALSE	AGBL5
Region_10082	chr2	27070472	27071654	1182	9	56	75.45454545	9.325204585	0.070386222	0.029515806	-	100128731	grey	-0.065844212	FALSE	OST4
Region_10083	chr2	27086747	27100762	14015	8	42	54.24242424	6.382814923	0.930938418	0.046409319	+	3795	grey	-0.113036055	FALSE	KHK
Region_10084	chr2	27098889	27119128	20239	9	45	60.86363636	8.714067078	0.933017661	0.038584728	-	10669	grey	-0.162455224	FALSE	CGREF1
Region_10085	chr2	27123789	27130812	7023	4	17	27.54545455	4.644870846	0.72329578	0.033526248	+	84696	grey	0.120966745	FALSE	ABHD1
Region_10086	chr2	27149004	27156974	7970	6	32	52.57575758	8.331701472	0.97173143	0.027671095	+	150921	grey	-0.129905523	FALSE	TCF23
Region_1008	chr2	27199587	27212958	13371	5	19	33.5303030	6.24923305	0.97724680	0.03057086	-	8884	grey	0.008912111	FALSE	SLC5A6

7							3	3	6	8						
Region_10088	chr2	27217369	27243943	26574	6	35	48.28787879	6.604812119	0.828865638	0.038103303	+	790	grey	0.114400182	FALSE	CAD
Region_10089	chr2	27253684	27275817	22133	11	67	89.5	9.508295973	0.568240983	0.03576073	-	7781	grey	-0.015839287	FALSE	SLC30A3
Region_10090	chr2	27282429	27307439	25010	18	116	142.030303	14.0876047	0.836606626	0.02290995	+	57159	grey	0.191885421	FALSE	TRIM54
Region_10091	chr2	27309492	27325680	16188	8	52	70.57575758	8.212674269	0.568099304	0.035155689	-	4358	grey	-0.242790958	FALSE	MPV17
Region_10092	chr2	27325849	27356999	31150	24	137	159.0909091	12.20432114	0.803235237	0.017609929	-	2976	grey	-0.167743701	FALSE	GTF3C2
Region_10093	chr2	27335520	27342599	7079	3	17	24.87878788	4.337724152	0.983683698	0.024847212	+	100505624	grey	0.058767124	FALSE	GTF3C2-AS1
Region_10094	chr2	27356246	27367622	11376	16	99	133.1060606	12.24761049	0.290023699	0.023088247	+	105374363	grey	-0.110258691	FALSE	GTF3C2-AS2
Region_10095	chr2	27364352	27370338	5986	7	41	52.22727273	6.576871958	0.52225387	0.043619082	-	8890	grey	0.092343906	FALSE	EIF2B4
Region_10096	chr2	27370496	27377535	7039	4	20	36.13636364	6.623701878	0.004827716	0.011170938	+	9784	grey	-0.001246281	FALSE	SNX17
Region_10097	chr2	27381195	27409591	28396	12	73	93.10606061	9.435006482	0.947275256	0.026413281	-	5496	grey	0.042273098	FALSE	PPM1G
Region_10098	chr2	27427790	27442259	14469	10	51	71.66666667	8.402075446	0.587237422	0.018899261	+	29959	grey	0.116162375	FALSE	NRBP1
Region_10099	chr2	27442366	27446481	4115	6	26	41.40909091	6.300127649	0.783708002	0.088052875	+	200634	grey	-0.154615759	FALSE	KRTCAP3

Region_10100	chr2	27444377	27489805	45428	23	153	181.1212121	13.64552917	0.881449139	0.019955335	-	26160	grey	-0.187193038	FALSE	IFT172
Region_10101	chr2	27496839	27523684	26845	8	50	66.90909091	8.26247732	0.945165739	0.029077976	+	2646	grey	-0.219583281	FALSE	GCKR
Region_10102	chr2	27537386	27582722	45336	13	81	104	9.905709309	0.95418407	0.022637456	+	84226	grey	0.00107158	FALSE	SPATA31H1
Region_10103	chr2	27582969	27635174	52205	20	142	165.0909091	10.81555471	0.854758163	0.021341955	+	84450	grey	-0.068321397	FALSE	ZNF512
Region_10104	chr2	27625638	27629012	3374	3	11	23.15151515	4.880865069	0.32268932	0.029067157	-	79635	grey	-0.056762101	FALSE	CCDC121
Region_10105	chr2	27628247	27651511	23264	7	40	66.16666667	8.538750284	0.690386131	0.025446577	+	11321	grey	0.089447186	FALSE	GPN1
Region_10106	chr2	27650809	27663840	13031	5	31	49.77272727	8.54993559	0.775034347	0.02471249	-	9913	grey	0.275629848	FALSE	SUPT7L
Region_10107	chr2	27663426	27695366	31940	15	105	132	11.88146585	0.670275198	0.026971663	+	22950	grey	-0.414537738	FALSE	SLC4A1AP
Region_10108	chr2	27771739	28338901	567162	75	565	628.3787879	30.54216009	0.884871185	0.011489084	+	9577	grey	-0.10449569	FALSE	BABAM2
Region_10109	chr2	27781379	27890681	109302	19	126	150.8484848	12.94751823	0.896653799	0.026422681	-	64080	grey	-0.097819034	FALSE	RBKS
Region_10110	chr2	28384409	28394680	10271	9	46	62.54545455	7.658442939	0.186222345	0.027621803	-	403150	grey	-0.100044903	FALSE	FOSL2-AS1
Region_10111	chr2	28392448	28417317	24869	8	56	72.72727273	7.347517539	0.537322196	0.031494003	+	2355	grey	0.072640171	FALSE	FOSL2
Region_1011	chr2	28457145	28644142	186997	58	439	485.696969	23.0977652	0.83187771	0.01672928	+	151056	grey	-0.03737673	FALSE	PLB1

2							7	6	9	2				7		
Region_10113	chr2	28708990	28751722	42732	5	29	42.43939394	6.231751915	0.767976701	0.04303986	-	112267877	grey	0.064133145	FALSE	PPP1CB-DT
Region_10114	chr2	28751640	28802940	51300	15	84	127.469697	15.34848496	0.81969046	0.024297191	+	5500	grey	0.18773076	FALSE	PPP1CB
Region_10115	chr2	28782517	28850611	68094	20	133	164.6212121	14.41982092	0.78179251	0.018054693	+	245711	grey	-0.186665769	FALSE	SPDYA
Region_10116	chr2	28894667	28948219	53552	19	120	158.7727273	16.01133777	0.763641281	0.030861277	+	23160	grey	0.101018989	FALSE	WDR43
Region_10117	chr2	28956611	29052230	95619	42	291	337.3030303	25.32373146	0.933928506	0.015176863	+	165186	grey	0.129947061	FALSE	TOGARAM2
Region_10118	chr2	29061695	29074523	12828	5	38	46.54545455	5.992769536	0.934532488	0.043443074	-	388939	grey	0.192915306	FALSE	PCARE
Region_10119	chr2	29097705	29199777	102072	16	110	133.3787879	12.06873284	0.700025788	0.022386412	+	79745	grey	0.157437391	FALSE	CLIP4
Region_10120	chr2	29192774	29921586	728812	118	920	1006.060606	35.32002212	0.831774275	0.01929015	-	238	grey	-0.176442422	FALSE	ALK
Region_10121	chr2	29319554	29352214	32660	6	41	55.87878788	7.422450666	0.952124855	0.029357973	+	101929386	grey	0.200999071	FALSE	LOC101929386
Region_10122	chr2	29841187	30146606	305419	39	288	327.1666667	18.91648311	0.813110686	0.021116555	-	101929418	grey	0.166495792	FALSE	
Region_10123	chr2	30231534	30323730	92196	34	257	292.9090909	17.97667486	0.853215527	0.022985369	+	81606	grey	0.281546495	FALSE	LBH
Region_10124	chr2	30447226	30644225	196999	37	246	283.5	17.34558246	0.891729605	0.015005203	+	253558	grey	0.105100918	FALSE	LCLAT1

Region_10125	chr2	30722771	30820542	97771	23	156	183.8181818	14.20607572	0.881975953	0.024616287	-	92291	grey	0.226315461	FALSE	CAPN13
Region_10126	chr2	30910467	31155202	244735	41	300	346.8787879	18.34164253	0.851372382	0.02201465	-	79623	grey	0.358067044	FALSE	GALNT14
Region_10127	chr2	31173056	31233858	60802	9	50	71.90909091	8.569583551	0.78683831	0.049925556	-	440854	grey	0.10745009	FALSE	CAPN14
Region_10128	chr2	31234152	31269451	35299	6	35	50.65151515	8.612142455	0.968689581	0.023382828	+	30845	grey	-0.134936952	FALSE	EHD3
Region_10129	chr2	31334321	31414742	80421	12	85	108.2727273	10.7539552	0.896139207	0.047737945	-	7498	grey	-0.242593837	FALSE	XDH
Region_10130	chr2	31522480	31580938	58458	5	30	41.68181818	5.826092742	0.4975285	0.0615574	-	6716	grey	-0.01032923	FALSE	SRD5A2
Region_10131	chr2	31865060	32023757	158697	20	118	139.8333333	11.33216434	0.866331259	0.018166418	-	51072	grey	-0.232738558	FALSE	MEMO1
Region_10132	chr2	32011886	32039805	27919	8	49	69.60606061	8.11243829	0.978874867	0.018051136	-	84661	grey	-0.248890529	FALSE	DPY30
Region_10133	chr2	32063556	32157637	94081	12	61	83.72727273	8.578229071	0.693403033	0.036325877	+	6683	grey	0.394918524	FALSE	SPAST
Region_10134	chr2	32165841	32224379	58538	13	68	93.83333333	11.17632571	0.93517302	0.037323823	+	55676	grey	0.104929065	FALSE	SLC30A6
Region_10135	chr2	32224453	32265732	41279	16	105	127.4848485	10.90552778	0.864936795	0.027609083	-	58484	grey	-0.270972771	FALSE	NLRC4
Region_10136	chr2	32277904	32316594	38690	20	124	153.469697	11.84414129	0.814265336	0.021534333	+	84272	grey	0.097519875	FALSE	YIPF4
Region_1013	chr2	32357023	32619571	262548	60	462	500.878787	21.7216766	0.91461733	0.01128964	+	57448	grey	0.026155107	FALSE	BIRC6

7							9	3		3						
Region_10138	chr2	32557273	32574818	17545	4	24	36.1969697	5.775864096	0.970771055	0.039269295	-	103752586	grey	0.013484161	FALSE	BIRC6-AS2
Region_10139	chr2	32628032	32821051	193019	38	254	302.7121212	19.24079412	0.940034626	0.009206891	+	55622	grey	-0.116093988	FALSE	TTC27
Region_10140	chr2	32825359	32946149	120790	30	219	252.7878788	17.1970616	0.898280804	0.020542063	+	285045	grey	0.189351381	FALSE	LINC00486
Region_10141	chr2	32946953	33399509	452556	67	483	557.9393939	26.11859272	0.88588747	0.015464049	+	4052	grey	0.270884854	FALSE	LTBP1
Region_10142	chr2	33436324	33564750	128426	17	113	140.2727273	10.67786417	0.828004226	0.035479625	+	25780	grey	-0.110854	FALSE	RASGRP3
Region_10143	chr2	33532744	33571283	38539	9	51	68.28787879	7.513608478	0.88107183	0.052305831	-	105374454	grey	0.104698828	FALSE	RASGRP3-AS1
Region_10144	chr2	33552522	33599347	46825	9	61	75.93939394	8.121914871	0.834014458	0.056300926	-	25940	grey	0.291466429	FALSE	FAM98A
Region_10145	chr2	33599442	33657460	58018	7	52	67.31818182	8.445588909	0.551168137	0.023496851	+	105374455	grey	-0.032918807	FALSE	LOC105374455
Region_10146	chr2	33706886	34297753	590867	91	678	753.0606061	31.12104348	0.860857816	0.018168914	+	104355287	grey	0.043765576	FALSE	LINC01317
Region_10147	chr2	34189813	34738231	548418	57	435	488.3939394	23.272221	0.854535838	0.024747002	+	104355288	grey	-0.015978135	FALSE	LINC01320
Region_10148	chr2	34700359	34822726	122367	12	64	82.87878788	9.320804111	0.85846125	0.04503795	-	105374458	grey	-0.049496421	FALSE	LOC105374458
Region_10149	chr2	34799850	35174909	375059	25	175	203.4545455	12.39128952	0.874523048	0.029017903	+	107985808	grey	-0.032241237	FALSE	LOC107985808

Region_10150	chr2	36551427	36646087	94660	15	112	136.1818182	12.89592552	0.863901271	0.028538268	-	9637	grey	0.155776322	FALSE	FEZ2
Region_10151	chr2	36696690	36814794	118104	22	157	188.2878788	15.2272682	0.931509306	0.024735371	+	5212	grey	-0.067655208	FALSE	VIT
Region_10152	chr2	36837698	36966536	128838	16	91	115.1212121	10.35747987	0.90101404	0.020125923	-	6801	grey	-0.113414039	FALSE	STRN
Region_10153	chr2	36968383	37084372	115989	24	157	197.5151515	16.25209328	0.942062887	0.023405767	-	54497	grey	0.211853543	FALSE	HEATR5B
Region_10154	chr2	37084518	37099244	14726	3	18	29.04545455	5.237238903	0.644592368	0.034925816	+	253635	grey	0.046158788	FALSE	GPATCH11
Region_10155	chr2	37099210	37157522	58312	14	65	85.63636364	9.468386856	0.76682822	0.030365223	-	5610	grey	0.048510727	FALSE	EIF2AK2
Region_10156	chr2	37167820	37196598	28778	9	55	75.04545455	8.545681443	0.706826022	0.031025513	-	391365	grey	-0.262280598	FALSE	SULT6B1
Region_10157	chr2	37196488	37216193	19705	7	50	70.40909091	9.830842006	0.451893235	0.042618297	+	100505876	grey	0.10142537	FALSE	CEBPZOS
Region_10158	chr2	37231631	37253403	21772	4	19	34.39393939	6.333983527	0.735057892	0.021030406	+	55471	grey	0.394960763	FALSE	NDUFAF7
Region_10159	chr2	37250502	37324833	74331	14	105	125.0909091	9.391857804	0.959403636	0.018252014	-	23683	grey	-0.196866438	FALSE	PRKD3
Region_10160	chr2	37342827	37373322	30495	8	42	60.92424242	8.226244317	0.923025817	0.042176193	+	25797	grey	-0.077808258	FALSE	QPCT
Region_10161	chr2	37466781	37646698	179917	28	208	248.7575758	17.48851105	0.816488881	0.03677952	+	105374464	grey	-0.041505418	FALSE	LINC03063
Region_1016	chr2	37641882	37738468	96586	7	53	74.6060606	8.91649946	0.35189035	0.05580401	-	10602	grey	0.09269278	FALSE	CDC42EP3

2							1	5	1	6						
Region_10163	chr2	37820498	37876274	55776	5	18	37.33333333	6.66179309	0.696087904	0.052501658	-	101929559	grey	0.023259361	FALSE	PIRAT1
Region_10164	chr2	37923187	38067142	143955	20	117	142.969697	12.71458242	0.631217385	0.023493685	+	151393	grey	0.148469161	FALSE	RMDN2
Region_10165	chr2	37949911	38039327	89416	9	56	68.96969697	9.398804665	0.856465265	0.028327075	-	101410544	grey	-0.111264168	FALSE	RMDN2-AS1
Region_10166	chr2	38035888	38067041	31153	4	16	29.72727273	5.313395132	0.71691689	0.042373529	-	107985872	grey	-0.029979313	FALSE	
Region_10167	chr2	38067509	38109902	42393	9	38	59.36363636	8.064425896	0.599812336	0.052757605	-	1545	grey	-0.191047525	FALSE	CYP1B1
Region_10168	chr2	38073447	38231651	158204	27	175	210.5	16.60884843	0.81847653	0.023527729	+	285154	grey	-0.35235155	FALSE	CYP1B1-AS1
Region_10169	chr2	38203363	38239590	36227	6	33	52.59090909	8.75649609	0.899639037	0.044722368	-	102723739	grey	-0.15095684	FALSE	LOC102723739
Region_10170	chr2	38293954	38377285	83331	26	187	223.2727273	16.32039352	0.866820108	0.028705717	-	64225	grey	-0.291164736	FALSE	ATL2
Region_10171	chr2	38406527	38515934	109407	12	79	97.90909091	8.916238035	0.955020425	0.026356342	-	101929596	grey	-0.110079871	FALSE	LINC02613
Region_10172	chr2	38561969	38603586	41617	13	82	103.6969697	9.171812499	0.371133585	0.025924533	-	92906	grey	0.058667718	FALSE	HNRNPLL
Region_10173	chr2	38666081	38741237	75156	14	84	110.2121212	11.62426527	0.900250908	0.034186016	+	130589	grey	-0.151941192	FALSE	GALM
Region_10174	chr2	38743599	38751494	7895	4	27	39.63636364	6.039960172	0.224926152	0.025569299	-	6432	grey	-0.15481053	FALSE	SRSF7

Region_10175	chr2	38751534	38785002	33468	11	59	73.10606061	7.110401455	0.890759731	0.028968389	+	79833	grey	0.098815975	FALSE	GEMIN6
Region_10176	chr2	38797729	38875934	78205	16	112	133.1212121	10.7924691	0.889948091	0.026143179	-	90957	grey	0.256146096	FALSE	DHX57
Region_10177	chr2	38875991	38975454	99463	20	123	147.530303	12.98664367	0.794865506	0.024813947	+	100271715	grey	-0.194670691	FALSE	ARHGEF33
Region_10178	chr2	38962206	39124345	162139	29	215	247.5454545	16.51403302	0.738189494	0.019573026	-	6654	grey	-0.212901557	FALSE	SOS1
Region_10179	chr2	39168045	39243983	75938	15	109	133.030303	10.1329162	0.962036495	0.019092059	-	344387	grey	0.065494768	FALSE	CDKL4
Region_10180	chr2	39249266	39437301	188035	33	246	282.2272727	17.49531406	0.925368916	0.01533961	-	8491	grey	0.036449103	FALSE	MAP4K3
Region_10181	chr2	39436530	39631127	194597	29	190	230.7727273	16.06984319	0.872447044	0.016503031	+	728730	grey	0.129231303	FALSE	MAP4K3-DT
Region_10182	chr2	39518639	39717963	199324	31	238	278.0757576	18.02162001	0.748596371	0.026530998	+	130733	grey	0.013862812	FALSE	TMEM178A
Region_10183	chr2	39736060	39779267	43207	3	14	26.43939394	5.381246032	0.934286594	0.053247375	-	80745	grey	0.057126086	FALSE	THUMPD2
Region_10184	chr2	39786453	40255209	468756	48	358	403.4090909	16.53711655	0.781134203	0.021364739	+	100128590	grey	0.091153054	FALSE	SLC8A1-AS1
Region_10185	chr2	40097270	40611053	513783	40	298	345.8636364	17.66603553	0.764554759	0.025305809	-	6546	grey	-0.007193344	FALSE	SLC8A1
Region_10186	chr2	41860155	41894050	33895	5	29	45.81818182	6.757125221	0.826034436	0.082587297	+	388942	grey	0.086160933	FALSE	LINC01913
Region_1018	chr2	42048021	42058517	10496	4	10	20.2121212	4.07058926	0.44689249	0.05944342	+	91461	grey	0.01726974	FALSE	PKDCC

7							1	6	1	1						
Region_10188	chr2	42143238	42170301	27063	8	40	54.68181818	7.253983714	0.834536227	0.03152611	-	102723824	grey	0.023121209	FALSE	EML4-AS1
Region_10189	chr2	42169353	42332548	163195	32	220	271.469697	17.41636692	0.923154446	0.019164975	+	27436	grey	-0.00445647	FALSE	EML4
Region_10190	chr2	42333546	42388507	54961	13	95	115.9090909	10.80132082	0.512769902	0.019550009	-	9167	grey	0.316327589	FALSE	COX7A2L
Region_10191	chr2	42365670	42425088	59418	11	58	80.8030303	8.997811182	0.934125361	0.036773564	-	105374537	grey	0.063229636	FALSE	
Region_10192	chr2	42442017	42493982	51965	14	86	113.5454545	10.24647304	0.735916283	0.033245116	-	170850	grey	0.258045348	FALSE	KCNG3
Region_10193	chr2	42494569	42756947	262378	82	587	646.8636364	28.13696521	0.918943813	0.010637995	+	57504	grey	-0.051747468	FALSE	MTA3
Region_10194	chr2	42767089	42792593	25504	8	37	53.57575758	7.26225353	0.938618065	0.03579343	-	23498	grey	-0.216949347	FALSE	HAAO
Region_10195	chr2	43027823	43040662	12839	9	57	73.95454545	7.471858626	0.92214742	0.032854397	-	102723854	grey	-0.09781704	FALSE	LINC01819
Region_10196	chr2	43092530	43157585	65055	20	113	147.1060606	13.1957205	0.624650996	0.032020599	-	100506047	grey	-0.084746671	FALSE	LINC02580
Region_10197	chr2	43222402	43226606	4204	15	75	100.3181818	14.27117492	0.004257412	0.010046108	-	678	grey	-0.040599713	FALSE	ZFP36L2
Region_10198	chr2	43227210	43228855	1645	3	10	20.53030303	4.611756538	0.01038329	0.028770522	+	100129726	grey	-0.196275921	FALSE	LINC01126
Region_10199	chr2	43230851	43596038	365187	98	736	802.1969697	35.90466852	0.92551473	0.01062784	-	63892	grey	-0.076342357	FALSE	THADA

Region_10200	chr2	43637260	43767987	130727	14	85	108.4545455	9.810797534	0.934232103	0.028930893	+	130271	grey	-0.053033055	FALSE	PLEKHH2
Region_10201	chr2	43774039	43810010	35971	9	54	78.42424242	9.376009658	0.751270069	0.019840061	+	51626	grey	-0.14000003	FALSE	DYNC2LI1
Region_10202	chr2	43812472	43838865	26393	10	68	84.1969697	7.680584307	0.810147154	0.048799298	-	64240	grey	0.389954424	FALSE	ABCG5
Region_10203	chr2	43831942	43882988	51046	15	101	124.6818182	12.04882259	0.890336595	0.041594494	+	64241	grey	0.171418437	FALSE	ABCG8
Region_10204	chr2	43886224	43996226	110002	24	190	225.6515152	13.80244396	0.875547362	0.020358164	-	10128	grey	0.162939729	FALSE	LRPPRC
Region_10205	chr2	44167969	44244384	76415	16	91	117.1515152	12.05658763	0.893625036	0.030346178	+	5495	grey	0.061293487	FALSE	PPM1B
Region_10206	chr2	44168851	44321494	152643	34	226	275.030303	18.34118501	0.892542466	0.019252837	+	6519	grey	0.115918391	FALSE	SLC3A1
Region_10207	chr2	44316281	44361862	45581	6	43	54.13636364	6.114010049	0.751147605	0.032995291	-	9581	grey	-0.294388023	FALSE	PREPL
Region_10208	chr2	44361947	44772592	410645	51	333	388.969697	24.96395537	0.890015303	0.015101435	+	79823	grey	-0.123918141	FALSE	CAMKMT
Region_10209	chr2	44921077	44939199	18122	10	52	72.59090909	9.253651708	0.075834403	0.043309522	-	107985879	grey	-0.097022161	FALSE	LINC01833
Region_10210	chr2	44941702	44946071	4369	3	11	22.28787879	5.074112735	0.17098839	0.08496994	+	6496	grey	0.073731059	FALSE	SIX3
Region_10211	chr2	45164816	45323397	158581	78	517	608.9393939	30.44939246	0.580454363	0.017213476	-	400952	grey	-0.120112511	FALSE	LINC01121
Region_1021	chr2	45169616	45211673	42057	38	253	313.257575	21.6064880	0.56011291	0.03025893	+	10537457	grey	-0.20522453	FALSE	LOC105374576

2							8	6	9	3		6				
Region_10213	chr2	45388680	45612165	223485	36	230	267.9090909	16.18235247	0.770666162	0.024637235	-	55133	grey	0.081288376	FALSE	SRBD1
Region_10214	chr2	45651345	46187990	536645	118	882	984.8787879	42.84085582	0.876999093	0.013545943	+	5581	grey	0.34220326	FALSE	PRKCE
Region_10215	chr2	46293667	46386697	93030	14	86	112.1969697	9.501772623	0.682018957	0.031779978	+	2034	grey	-0.214361655	FALSE	EPAS1
Region_10216	chr2	46429190	46501278	72088	16	86	122.2424242	11.43955433	0.670478698	0.024576824	+	101805491	grey	-0.125643627	FALSE	LINC02583
Region_10217	chr2	46490750	46542577	51827	15	83	105.1212121	10.71377999	0.692630207	0.023756582	-	90423	grey	-0.128650437	FALSE	ATP6V1E2
Region_10218	chr2	46541806	46584688	42882	7	40	56.31818182	7.15360929	0.810050623	0.030721143	+	23433	grey	0.036246968	FALSE	RHOQ
Region_10219	chr2	46580937	46617055	36118	7	32	49.16666667	8.567529638	0.427136335	0.018542973	-	5281	grey	-0.08516345	FALSE	PIGF
Region_10220	chr2	46616416	46630176	13760	6	32	44.42424242	6.707196149	0.338409737	0.021386947	+	9419	grey	-0.086657464	FALSE	CRIPT
Region_10221	chr2	46698940	46858210	159270	29	193	234.5	16.06166003	0.762491678	0.021449142	+	388948	grey	0.419276199	FALSE	LINC01118
Region_10222	chr2	46698952	46763129	64177	11	60	77.62121212	8.641162941	0.680210381	0.033052324	+	9655	grey	-0.008756238	FALSE	SOCS5
Region_10223	chr2	46827864	46859009	31145	9	60	79.3030303	9.080782238	0.712109797	0.033060944	+	100134259	grey	0.437507058	FALSE	LINC01119
Region_10224	chr2	46901870	46941855	39985	10	63	80.63636364	8.286479003	0.373061174	0.029273768	-	90411	grey	0.379295054	FALSE	MCFD2

Region_10225	chr2	46915869	47076137	160268	37	262	301.8939394	17.56055789	0.838949297	0.018928691	+	57217	grey	0.111965667	FALSE	TTC7A
Region_10226	chr2	47065941	47176511	110570	35	234	270.2727273	18.67416599	0.821530097	0.018094422	-	285051	grey	0.020465369	FALSE	STPG4
Region_10227	chr2	47160084	47176921	16837	9	50	66.87878788	9.003444044	0.54049481	0.019990373	-	805	grey	0.223439106	FALSE	CALM2
Region_10228	chr2	47192405	47345074	152669	56	388	451.8333333	22.04860598	0.875660051	0.015266112	-	101927043	grey	-0.02167589	FALSE	EPCAM-DT
Region_10229	chr2	47345158	47387601	42443	15	72	101.6969697	11.26597286	0.841392837	0.022902689	+	4072	grey	0.028716406	FALSE	EPCAM
Region_10230	chr2	47403067	47663146	260079	93	677	761.7878788	31.89140957	0.725743103	0.010281084	+	4436	grey	-0.124102217	FALSE	MSH2
Region_10231	chr2	47509290	47570985	61695	19	137	163.6666667	13.17262118	0.77499906	0.024535059	-	56660	grey	-0.013439046	FALSE	KCNK12
Region_10232	chr2	47695530	47757793	62263	3	13	21.93939394	4.556917946	0.937245821	0.062685363	+	440864	grey	-0.241777408	FALSE	
Region_10233	chr2	47695819	47810063	114244	25	171	198.4545455	13.8477233	0.855211256	0.02367577	+	2956	grey	-0.3374712	FALSE	MSH6
Region_10234	chr2	47789316	47906498	117182	24	161	195.4545455	16.12850493	0.887537909	0.025163733	-	80204	grey	-0.081479937	FALSE	FBXO11
Region_10235	chr2	47924181	48314206	390025	58	435	480.6060606	18.74433559	0.837429239	0.021430781	-	105374591	grey	0.157489247	FALSE	LOC105374591
Region_10236	chr2	48006039	48314224	308185	46	338	377.4242424	16.48501474	0.808070194	0.025282388	-	105374593	grey	0.201138774	FALSE	LOC105374593
Region_1023	chr2	48314637	48379295	64658	11	61	85.7121212	8.77192225	0.96342317	0.02456265	+	3344	grey	0.238549927	FALSE	FOXN2

7							1	5	2	1						
Region_10238	chr2	48440598	48515391	74793	11	56	81.06060606	9.764738427	0.965209921	0.032585656	+	129285	grey	0.105402215	FALSE	PPP1R21
Region_10239	chr2	48529546	48598513	68967	13	61	82.6969697	9.372578164	0.873342787	0.034806818	+	11037	grey	-0.118478829	FALSE	STON1
Region_10240	chr2	48529925	48776517	246592	49	326	364.3333333	20.90957332	0.905599596	0.020316035	+	286749	grey	0.146891848	FALSE	STON1-GTF2A1L
Region_10241	chr2	48617798	48733148	115350	23	161	189.0757576	12.99504119	0.924105817	0.031038564	+	11036	grey	0.075827635	FALSE	GTF2A1L
Region_10242	chr2	48632291	48755730	123439	26	187	214.1666667	13.24512489	0.914387118	0.027535769	-	3973	grey	0.153844735	FALSE	LHCGR
Region_10243	chr2	48962157	49154527	192370	15	102	131.7121212	11.31474892	0.80226928	0.053133523	-	2492	grey	0.189774386	FALSE	FSHR
Region_10244	chr2	49202126	49451896	249770	18	119	147.530303	12.79088581	0.857873843	0.027674563	-	107985810	grey	-0.147485282	FALSE	LOC107985810
Region_10245	chr2	49918503	51082864	1164361	93	679	775.7575758	37.2554841	0.823708902	0.018869514	-	9378	grey	0.057066197	FALSE	NRXN1
Region_10246	chr2	51032601	52407917	1375316	107	757	825.4090909	35.40055509	0.851341454	0.018980985	+	730100	grey	-0.10390345	FALSE	NRXN1-DT
Region_10247	chr2	51977787	52022435	44648	7	24	42.90909091	8.395469974	0.935250413	0.038383605	-	105374597	grey	0.086337782	FALSE	LOC105374597
Region_10248	chr2	52722671	52961452	238781	15	76	105.0757576	9.865730284	0.824591515	0.043661454	-	105369165	grey	0.380373557	FALSE	LOC105369165
Region_10249	chr2	53532672	53860160	327488	36	254	295.2727273	16.34770787	0.907662678	0.018727625	-	100302652	grey	0.030334637	FALSE	GPR75-ASB3

Region_10250	chr2	53581204	53606459	25255	4	25	36.72727273	6.420029845	0.826963734	0.096977552	-	105374601	grey	-0.135399982	FALSE	
Region_10251	chr2	53669980	53787009	117029	14	90	111.5454545	10.09063819	0.931756121	0.030046479	-	51130	grey	0.312731579	FALSE	ASB3
Region_10252	chr2	53767804	53775196	7392	3	11	24.78787879	4.808694534	0.969075313	0.033178302	+	494143	grey	0.18670428	FALSE	CHAC2
Region_10253	chr2	53787009	53833038	46029	10	76	92.31818182	9.671781462	0.876144828	0.017829254	+	27248	grey	-0.04090475	FALSE	ERLEC1
Region_10254	chr2	53864069	53970993	106924	19	144	169.3484848	12.69947597	0.842131419	0.02430267	-	23198	grey	0.227844554	FALSE	PSME4
Region_10255	chr2	53970838	54305300	334462	51	372	414.3333333	23.83942006	0.872953632	0.016641433	+	98	grey	-0.126633281	FALSE	ACYP2
Region_10256	chr2	54456317	54671446	215129	40	304	349.5606061	19.56773544	0.600140873	0.014645986	+	6711	grey	-0.115667978	FALSE	SPTBN1
Region_10257	chr2	54516048	54540697	24649	6	36	51.37878788	7.06620375	0.8262952	0.045346197	-	105374640	grey	-0.322178745	FALSE	SPTBN1-AS1
Region_10258	chr2	54723499	54972025	248526	46	341	394.7121212	20.59257906	0.889740867	0.012845253	+	400954	grey	-0.057931923	FALSE	EML6
Region_10259	chr2	54972187	55112621	140434	21	133	156	10.45576764	0.791676548	0.019405998	-	57142	grey	-0.059903735	FALSE	RTN4
Region_10260	chr2	55172547	55232563	60016	10	59	82.75757576	9.36615939	0.576022829	0.024817105	-	130162	grey	0.400915705	FALSE	CLHC1
Region_10261	chr2	55287842	55419895	132053	28	202	232.3484848	15.22497182	0.927139743	0.016031073	-	55704	grey	0.142460106	FALSE	CCDC88A
Region_1026	chr2	55547292	55618880	71588	11	62	78.4393939	8.97882772	0.79320894	0.02389699	-	57223	grey	0.061857767	FALSE	PPP4R3B

2							4	5	6							
Region_10263	chr2	55634061	55693863	59802	16	107	133.6969697	11.3760473	0.902654602	0.026229923	-	87178	grey	-0.301778779	FALSE	PNPT1
Region_10264	chr2	55865967	55924139	58172	12	89	110.4393939	11.62110651	0.553975718	0.037577512	-	2202	grey	-0.140125418	FALSE	EFEMP1
Region_10265	chr2	55952158	56181652	229494	23	180	204.2878788	14.52823792	0.861372444	0.039300032	+	105374690	grey	-0.120749417	FALSE	LOC105374690
Region_10266	chr2	55963191	56047326	84135	10	68	94.28787879	9.742476626	0.768974169	0.067843406	-	104355290	grey	-0.007564159	FALSE	MIR217HG
Region_10267	chr2	56147630	56386172	238542	20	141	169.2878788	12.89694675	0.869179962	0.037320611	+	114800	grey	0.058532294	FALSE	CCDC85A
Region_10268	chr2	57289648	57380132	90484	5	26	41.15151515	5.974928192	0.83459437	0.066107442	-	105377632	grey	-0.063550248	FALSE	
Region_10269	chr2	57907629	58159920	252291	15	105	123.6666667	10.99696928	0.896574645	0.020023338	+	7444	grey	0.233416748	FALSE	VRK2
Region_10270	chr2	58159243	58241410	82167	9	53	69.36363636	9.773805437	0.862942515	0.023494579	-	55120	grey	0.246260219	FALSE	FANCL
Region_10271	chr2	58427799	59277688	849889	75	554	614.3030303	31.66846026	0.853928534	0.019318247	+	400955	grey	0.012198907	FALSE	LINC01122
Region_10272	chr2	59217708	59279400	61692	3	18	28.43939394	5.602551034	0.9618459	0.033073687	+	101927285	grey	0.007271943	FALSE	LINC01793
Region_10273	chr2	59238703	59733396	494693	32	247	285.2575758	15.06734687	0.877293039	0.022647989	-	105374754	grey	-0.088756331	FALSE	LOC105374754
Region_10274	chr2	60340166	60439828	99662	17	103	127.3939394	11.32849204	0.724726215	0.036340767	-	106660609	grey	0.066453179	FALSE	MIR4432HG

Region_10275	chr2	60450520	60554467	103947	32	208	254.2121212	15.26939136	0.627314181	0.026376244	-	53335	grey	0.041145313	FALSE	BCL11A
Region_10276	chr2	60756253	60802086	45833	20	131	174.8181818	16.81108525	0.934661227	0.019878302	+	64895	grey	-0.160695368	FALSE	PAPOLG
Region_10277	chr2	60823069	60881317	58248	14	88	111.8787879	11.97499337	0.94097076	0.032784524	-	400957	grey	-0.216357291	FALSE	REL-DT
Region_10278	chr2	60881491	60931612	50121	7	32	49.8030303	7.658519031	0.69892538	0.039909714	+	5966	grey	-0.102012124	FALSE	REL
Region_10279	chr2	60940222	61018259	78037	19	113	137.4545455	12.71967805	0.901259897	0.022496197	-	150962	grey	-0.061801267	FALSE	PUS10
Region_10280	chr2	61017225	61051990	34765	9	69	91.10606061	10.4161543	0.758360401	0.016136422	+	5194	grey	0.075539506	FALSE	PEX13
Region_10281	chr2	61065871	61138034	72163	21	144	176.3787879	13.34024005	0.872037698	0.013186454	+	84542	grey	-0.069547121	FALSE	SANBR
Region_10282	chr2	61115787	61164828	49041	9	54	77.10606061	9.18716293	0.868123334	0.02233115	+	339804	grey	-0.328823907	FALSE	C2orf74
Region_10283	chr2	61151433	61162105	10672	3	12	20.6969697	4.332239417	0.970894075	0.045551243	-	105374758	grey	-0.124220468	FALSE	C2orf74-AS1
Region_10284	chr2	61177418	61191203	13785	6	21	38.09090909	6.774902265	0.670246359	0.041429249	+	130872	grey	-0.13387797	FALSE	AHSA2P
Region_10285	chr2	61187463	61471087	283624	71	487	536.7424242	25.5615761	0.894653371	0.008915631	-	9736	grey	0.111102734	FALSE	USP34
Region_10286	chr2	61476032	61538741	62709	20	115	143.5909091	12.00381641	0.853625328	0.023839628	-	7514	grey	-0.008613677	FALSE	XPO1
Region_1028	chr2	61728808	61764201	35393	11	70	91.5454545	10.1073953	0.72596526	0.03958906	-	10798576	grey	-0.30554044	FALSE	LOC107985767

7							5	4	5	2		7				
Region_10288	chr2	61824848	61854143	29295	8	39	55.1969697	7.073721025	0.800908717	0.059977657	-	84140	grey	-0.261026475	FALSE	FAM161A
Region_10289	chr2	61868085	61888671	20586	4	23	32.63636364	5.965635858	0.976684867	0.033656484	-	10575	grey	-0.055515684	FALSE	CCT4
Region_10290	chr2	61888724	62147247	258523	68	501	558.7878788	26.32373316	0.928898578	0.010295207	+	150684	grey	0.173355032	FALSE	COMMD1
Region_10291	chr2	62069447	62146881	77434	18	122	159.7272727	14.06309459	0.950020179	0.019549387	-	107985768	grey	-0.003856011	FALSE	
Region_10292	chr2	62196115	62224731	28616	6	32	54.46969697	8.177303857	0.629395635	0.045946707	+	10678	grey	0.258251036	FALSE	B3GNT2
Region_10293	chr2	62296284	62464073	167789	35	247	288.8484848	19.8983899	0.673441888	0.020530898	+	105374764	grey	-0.06357054	FALSE	LOC105374764
Region_10294	chr2	62673851	63046487	372636	35	231	268.530303	19.12969476	0.933520916	0.011956143	+	23301	grey	-0.131552258	FALSE	EHBP1
Region_10295	chr2	62957326	63048640	91314	12	63	82.66666667	9.089864742	0.891519796	0.018581065	-	100132215	grey	0.093934412	FALSE	EHBP1-AS1
Region_10296	chr2	63050057	63057836	7779	3	16	27.65151515	5.897828608	0.041246118	0.043167472	+	5013	grey	-0.376672602	FALSE	OTX1
Region_10297	chr2	63106879	63197037	90158	4	17	28.60606061	5.004240626	0.888756642	0.079807883	+	107985769	grey	0.148222572	FALSE	LOC107985769
Region_10298	chr2	63119559	63827843	708284	54	409	452.0909091	20.52894937	0.891768687	0.011433143	-	51057	grey	-0.183427454	FALSE	WDPCP
Region_10299	chr2	63840952	63891562	50610	5	30	40.53030303	6.295834269	0.575444927	0.043589768	+	7360	grey	-0.302669356	FALSE	UGP2

Region_10300	chr2	63892146	64019428	127282	10	75	95.21212121	8.757441058	0.9753046	0.018314675	-	51542	grey	-0.080736452	FALSE	VPS54
Region_10301	chr2	64092652	64144420	51768	5	31	44.16666667	5.674730311	0.496884592	0.043204087	-	57162	grey	-0.051394792	FALSE	PELI1
Region_10302	chr2	64143239	64257610	114371	18	132	159.2575758	13.9992424	0.734144961	0.021251023	+	100507006	grey	0.067327257	FALSE	LOC100507006
Region_10303	chr2	64185078	64205485	20407	3	20	29.72727273	4.408193262	0.974572544	0.033470531	-	150992	grey	0.140233277	FALSE	LINC00309
Region_10304	chr2	64395220	64453897	58677	9	50	67.48484848	7.965777618	0.84015581	0.032034099	-	105374771	grey	0.119533496	FALSE	LGALSL-DT
Region_10305	chr2	64486353	64500904	14551	3	15	25.65151515	5.13956725	0.841774359	0.130715338	+	105374774	grey	0.25464164	FALSE	LINC01805
Region_10306	chr2	64524299	64593005	68706	7	34	51.36363636	6.902923268	0.684810774	0.032119862	+	54812	grey	0.221190709	FALSE	AFTPH
Region_10307	chr2	64606975	64616556	9581	6	36	46.04545455	6.417360632	0.441965265	0.041729843	+	339807	grey	0.015259776	FALSE	LINC02579
Region_10308	chr2	64631621	64751005	119384	14	91	114.3636364	10.39472701	0.828918713	0.029155485	-	9792	grey	-0.261144832	FALSE	SERTAD2
Region_10309	chr2	64846130	64863626	17496	8	40	53.12121212	6.286775256	0.554300362	0.067787509	-	101927438	grey	0.107280573	FALSE	LINC01800
Region_10310	chr2	64901840	65056168	154328	38	291	333.3636364	19.09300022	0.740531254	0.022177816	-	400958	grey	0.037979328	FALSE	LINC02245
Region_10311	chr2	64953253	65056226	102973	25	193	227.3484848	15.80700178	0.718888562	0.021270905	-	107984063	grey	0.233807409	FALSE	
Region_1031	chr2	64988477	65023865	35388	12	85	106.590909	10.9586316	0.66917005	0.02606206	+	6509	grey	0.071900131	FALSE	SLC1A4

2							1	8	7							
Region_10313	chr2	65030720	65053017	22297	4	21	34.16666667	5.445063631	0.714081701	0.052383126	+	102724233	grey	-0.130612826	FALSE	LINC02576
Region_10314	chr2	65070696	65130331	59635	21	136	159.8636364	12.35735392	0.880069386	0.021498974	-	5861	grey	-0.045159702	FALSE	RAB1A
Region_10315	chr2	65227788	65271253	43465	13	74	91.1969697	8.727004415	0.790622859	0.029715249	+	10097	grey	0.012477957	FALSE	ACTR2
Region_10316	chr2	65310851	65432637	121786	36	242	279.2575758	15.79124451	0.831080142	0.019073024	-	200734	grey	-0.137629842	FALSE	SPRED2
Region_10317	chr2	65436711	66200373	763662	126	917	1013.909091	38.15463568	0.892975444	0.013615956	+	105369168	grey	-0.182550503	FALSE	LINC02934
Region_10318	chr2	65439838	65456571	16733	4	18	26.68181818	4.933898726	0.715128598	0.076289957	-	105374780	grey	-0.031111189	FALSE	LOC105374780
Region_10319	chr2	66433452	66573869	140417	24	153	183.0909091	12.39941348	0.484635543	0.023678721	+	4211	grey	-0.121412731	FALSE	MEIS1
Region_10320	chr2	66574030	66731223	157193	18	126	153.6212121	12.57074155	0.698902747	0.032976353	+	100507073	grey	0.112156521	FALSE	LINC01798
Region_10321	chr2	66904436	66971462	67026	4	30	41.89393939	7.080045171	0.919609024	0.042405694	+	101060019	grey	0.310529456	FALSE	LINC01799
Region_10322	chr2	67086446	67311439	224993	17	104	130.9393939	10.99773234	0.809569273	0.030249114	+	102800447	grey	-0.061251967	FALSE	LINC01828
Region_10323	chr2	67087013	67257966	170953	12	67	91.93939394	8.514022439	0.757028609	0.039547716	+	101927661	grey	-0.119885018	FALSE	LOC101927661
Region_10324	chr2	67123357	67397980	274623	20	131	157.1818182	12.24356546	0.825378429	0.022923937	-	644838	grey	0.009074345	FALSE	LINC01829

Region_10325	chr2	67562067	67620545	58478	3	15	26.37878788	5.134394284	0.817402666	0.088549181	+	102724373	grey	0.127829191	FALSE	LINC02831
Region_10326	chr2	67565604	67684077	118473	4	21	32.93939394	6.038030213	0.854019712	0.066778934	-	105374786	grey	0.081463209	FALSE	LOC105374786
Region_10327	chr2	67796054	67825562	29508	5	28	39.36363636	5.562612457	0.916785158	0.052546348	-	101927701	grey	-0.045233661	FALSE	LINC01812
Region_10328	chr2	68047277	68110948	63671	5	45	61.89393939	7.159993098	0.934638144	0.037385979	-	105374789	grey	-0.17900562	FALSE	LOC105374789
Region_10329	chr2	68122936	68261230	138294	14	83	115.3939394	11.12153668	0.642706874	0.020885096	-	116143	grey	0.014659207	FALSE	DNAAF10
Region_10330	chr2	68178857	68256237	77380	8	45	60.53030303	8.41650338	0.404705148	0.0274105	-	5534	grey	0.053533711	FALSE	PPP3R1
Region_10331	chr2	68251603	68300006	48403	7	30	47.74242424	7.288158164	0.6434503	0.033169039	+	107985892	grey	0.037928701	FALSE	LOC107985892
Region_10332	chr2	68251603	68346035	94432	14	68	94.25757576	9.633771697	0.62611789	0.035187283	+	102724389	grey	0.225647993	FALSE	LOC102724389
Region_10333	chr2	68284171	68320051	35880	10	51	69.96969697	8.296374921	0.682457483	0.048605516	-	25927	grey	0.277037458	FALSE	CNRIP1
Region_10334	chr2	68467572	68630724	163152	13	85	106.5909091	10.94177214	0.962489781	0.026001312	+	200558	grey	0.123330692	FALSE	APLF
Region_10335	chr2	68586854	68658251	71397	5	23	34.84848485	6.119935707	0.428238916	0.044328276	+	10887	grey	-0.123282366	FALSE	PROKR1
Region_10336	chr2	68679601	68826833	147232	11	67	92.03030303	9.857398154	0.851402713	0.035975886	+	9938	grey	0.319921996	FALSE	ARHGAP25
Region_1033	chr2	69013176	69249327	236151	26	204	236.030303	14.5633572	0.79862386	0.02792177	+	84168	grey	0.021188085	FALSE	ANTXR1

7								8	8	7						
Region_10338	chr2	69319780	69387250	67470	12	72	89.77272727	9.962692646	0.899104415	0.035422065	-	2673	grey	0.130486696	FALSE	GFPT1
Region_10339	chr2	69396113	69437628	41515	10	55	71.22727273	7.745560425	0.861139999	0.037696051	-	27247	grey	-0.264053498	FALSE	NFU1
Region_10340	chr2	69457997	69674349	216352	38	301	332.6969697	17.94947584	0.87257072	0.01604548	-	22848	grey	0.135719718	FALSE	AAK1
Region_10341	chr2	69644425	69827112	182687	33	218	252.6515152	15.28246096	0.910962188	0.018285572	+	307	grey	-0.326308261	FALSE	ANXA4
Region_10342	chr2	69829660	69881384	51724	6	30	43.18181818	6.263223773	0.800882535	0.035665882	+	64395	grey	-0.088730027	FALSE	GMCL1
Region_10343	chr2	69893956	69905575	11619	3	18	28.06060606	4.056822508	0.612784447	0.049522691	+	11017	grey	0.10378708	FALSE	SNRNP27
Region_10344	chr2	69897688	69942945	45257	9	64	79.16666667	8.262209303	0.468522333	0.023464352	+	4084	grey	-0.201390641	FALSE	MXD1
Region_10345	chr2	69960089	70102909	142820	43	266	320.469697	22.34018091	0.850660916	0.017188902	-	151516	grey	-0.081791695	FALSE	ASPRV1
Region_10346	chr2	69962263	70089206	126943	38	232	290.4242424	21.97345294	0.845035235	0.017504246	-	400960	grey	-0.055068149	FALSE	PCBP1-AS1
Region_10347	chr2	70149885	70248615	98730	26	176	210.3787879	15.16348362	0.783745424	0.017500938	-	54980	grey	0.024204626	FALSE	C2orf42
Region_10348	chr2	70209444	70248660	39216	8	54	73.65151515	9.229538574	0.980239664	0.017604494	-	7072	grey	-0.285856838	FALSE	TIA1
Region_10349	chr2	70257386	70281185	23799	8	43	60.07575758	7.504481955	0.969738625	0.028637517	+	51449	grey	-0.124706816	FALSE	PCYOX1

Region_10350	chr2	70281362	70293740	12378	4	21	32.34848485	6.205437874	0.519386984	0.061178719	-	6637	grey	0.046622996	FALSE	SNRPG
Region_10351	chr2	70295975	70302090	6115	7	31	44.40909091	6.684027512	0.465887396	0.053867934	-	84908	grey	-0.033870986	FALSE	FAM136A
Region_10352	chr2	70447284	70554193	106909	39	268	305.9545455	17.45361485	0.859774976	0.021269997	-	7039	grey	-0.004937327	FALSE	TGFA
Region_10353	chr2	70607618	70768225	160607	16	110	135.8030303	11.91794987	0.907227697	0.028578329	-	119	grey	0.085592614	FALSE	ADD2
Region_10354	chr2	70777310	70790643	13333	6	35	48.71212121	7.509512227	0.628810228	0.030326857	-	344018	grey	-0.134232441	FALSE	FIGLA
Region_10355	chr2	70887871	70889959	2088	3	11	21.98484848	4.984568261	0.093569001	0.071715003	+	104355141	grey	0.020815272	FALSE	LINC01143
Region_10356	chr2	70900576	70933446	32870	13	88	108.3787879	11.18070474	0.438141921	0.033444402	+	25806	grey	-0.105265607	FALSE	VAX2
Region_10357	chr2	70900798	70965431	64633	17	101	128.6060606	11.62077554	0.500045095	0.028942068	+	525	grey	0.136353119	FALSE	ATP6V1B1
Region_10358	chr2	70939318	70995336	56018	28	142	180.7424242	18.80041784	0.268724115	0.016274088	-	113419	grey	0.353002949	FALSE	TEX261
Region_10359	chr2	70941817	70994749	52932	18	76	100.8939394	10.98828577	0.416862041	0.026506674	-	101927750	grey	0.266254964	FALSE	ATP6V1B1-AS1
Region_10360	chr2	70994510	71002754	8244	20	93	144.4393939	18.75848837	0.006140744	0.007601311	+	105374796	grey	0.153918505	FALSE	LOC105374796
Region_10361	chr2	71109684	71130239	20555	15	76	111.3484848	14.61452641	0.965067854	0.020532883	-	84693	grey	0.229006281	FALSE	MCEE
Region_1036	chr2	71130310	71150101	19791	7	39	54.8787878	7.51923717	0.71364155	0.06267107	+	10199	grey	-0.46027620	FALSE	MPHOSPH10

2							8	8	7	3				3		
Region_10363	chr2	71182738	71227103	44365	14	81	104.9090909	11.51951825	0.891966288	0.035008398	-	400961	grey	-0.008104279	FALSE	PAIP2B
Region_10364	chr2	71276561	71435069	158508	39	272	318.3181818	19.30576565	0.779434374	0.015850337	+	27332	grey	-0.210010981	FALSE	ZNF638
Region_10365	chr2	71453561	71686763	233202	68	460	512.7121212	26.74653976	0.707663329	0.023791069	+	8291	grey	-0.173990593	FALSE	DYSF
Region_10366	chr2	72129238	72147862	18624	6	29	49.90909091	7.326930878	0.805279542	0.037813735	-	56603	grey	-0.043375545	FALSE	CYP26B1
Region_10367	chr2	72175984	72826041	650057	60	462	508.6060606	23.38630683	0.929311945	0.012464798	-	23233	grey	0.00510001	FALSE	EXOC6B
Region_10368	chr2	72916260	72934891	18631	10	51	76.54545455	8.160282458	0.590152642	0.031678549	+	2016	grey	0.124298764	FALSE	EMX1
Region_10369	chr2	72942036	73075619	133583	24	146	171.3939394	13.44032828	0.879893908	0.026511042	-	94097	grey	-0.342316259	FALSE	SFXN5
Region_10370	chr2	73073382	73156721	83339	20	135	164.0606061	11.90459354	0.808737754	0.046741546	-	26056	grey	-0.193714411	FALSE	RAB11FIP5
Region_10371	chr2	73202574	73212513	9939	5	20	33.53030303	5.849969617	0.662914712	0.083437888	+	344022	grey	0.036521737	FALSE	NOTO
Region_10372	chr2	73214222	73227221	12999	8	37	55.1969697	8.407358859	0.512019781	0.043220484	+	10322	grey	-0.303783243	FALSE	SMYD5
Region_10373	chr2	73233420	73253021	19601	9	55	76.46969697	9.535713756	0.318612086	0.023150439	+	10574	grey	0.056302328	FALSE	CCT7
Region_10374	chr2	73254682	73284478	29796	13	61	92.96969697	11.12442869	0.566746937	0.065993522	-	150726	grey	0.283734678	FALSE	FBXO41

Region_10375	chr2	73352610	73385677	33067	3	21	31.37878788	4.954454566	0.625078004	0.039169323	-	105374804	grey	0.170198575	FALSE	LOC105374804
Region_10376	chr2	73385758	73625166	239408	62	469	512.2575758	24.13571283	0.934240656	0.011973824	+	7840	grey	-0.278460675	FALSE	ALMS1
Region_10377	chr2	73641083	73697509	56426	4	19	33.54545455	5.84987	0.864291978	0.053409503	+	112268418	grey	-0.157199565	FALSE	LOC112268418
Region_10378	chr2	73644919	73685576	40657	3	15	27	5.212411224	0.842489257	0.062811891	+	200420	grey	-0.041899201	FALSE	ALMS1P1
Region_10379	chr2	73761782	73780132	18350	5	19	33.93939394	5.529998714	0.972475958	0.033014313	-	8446	grey	0.000745729	FALSE	DUSP11
Region_10380	chr2	73828916	73873659	44743	11	55	72.62121212	8.658948509	0.682204805	0.029776313	+	10617	grey	-0.228401954	FALSE	STAMBP
Region_10381	chr2	73834422	73856965	22543	5	18	28.42424242	5.435101318	0.921990385	0.048350483	-	112268419	grey	-0.100954511	FALSE	LOC112268419
Region_10382	chr2	73892314	73919865	27551	6	34	55.3030303	7.773663025	0.605920351	0.039638026	+	72	grey	0.107348802	FALSE	ACTG2
Region_10383	chr2	73926826	73958961	32135	10	55	81.51515152	9.755854139	0.831633901	0.031669734	+	1716	grey	-0.059997924	FALSE	DGUOK
Region_10384	chr2	73947322	73982173	34851	11	75	94.1969697	10.13633175	0.90182274	0.034487849	-	100874048	grey	-0.207827583	FALSE	DGUOK-AS1
Region_10385	chr2	73984910	74135640	150730	41	253	299.5	18.31959858	0.708931088	0.018651928	+	200424	grey	0.103040773	FALSE	TET3
Region_10386	chr2	74135400	74147912	12512	10	51	68	9.33974139	0.663406793	0.023387848	-	388962	grey	0.114543453	FALSE	BOLA3
Region_1038	chr2	74152528	74178898	26370	8	42	57.6212121	8.25136846	0.92580895	0.03981792	-	55233	grey	0.277065692	FALSE	MOB1A

7							2	7	3	5						
Region_10388	chr2	74186172	74217565	31393	8	45	62.68181818	8.723371068	0.953249399	0.025452861	+	10797	grey	0.106545603	FALSE	MTHFD2
Region_10389	chr2	74211604	74363377	151773	36	243	287.4090909	19.12467377	0.928799599	0.020860271	-	57835	grey	-0.125047367	FALSE	SLC4A5
Region_10390	chr2	74361154	74392087	30933	8	52	64.45454545	6.363796202	0.898021476	0.03340346	-	1639	grey	-0.192930435	FALSE	DCTN1
Region_10391	chr2	74393836	74421662	27826	12	70	84.36363636	8.558641811	0.701209813	0.033822572	-	388963	grey	0.306487785	FALSE	C2orf81
Region_10392	chr2	74421678	74425755	4077	3	14	23.78787879	4.753994762	0.621770631	0.048066557	+	84058	grey	0.258404229	FALSE	WDR54
Region_10393	chr2	74425835	74442422	16587	8	42	53.78787879	6.778961008	0.794871426	0.059532916	-	6242	grey	-0.108432484	FALSE	RTKN
Region_10394	chr2	74455088	74460884	5796	4	17	31.98484848	6.22524	0.000541126	0.004396125	+	100532735	grey	0.091877341	FALSE	INO80B-WBP1
Region_10395	chr2	74461057	74465410	4353	4	16	30.90909091	6.206020087	0.478841718	0.035203659	-	7841	grey	-0.045426905	FALSE	MOGS
Region_10396	chr2	74471982	74472687	705	5	26	43.04545455	8.064035673	0.006739145	0.014042621	-	116540	grey	0.216173477	FALSE	MRPL53
Region_10397	chr2	74471986	74483408	11422	12	77	104.9090909	14.87354624	0.089666574	0.010086049	-	84865	grey	0.047882606	FALSE	CCDC142
Region_10398	chr2	74497517	74503316	5799	4	22	33.66666667	6.200082712	0.260777049	0.042318277	-	85474	grey	0.04779109	FALSE	LBX2
Region_10399	chr2	74513463	74517148	3685	5	22	35.22727273	7.46436757	0.094996519	0.056558939	+	3196	grey	-0.270158438	FALSE	TLX2

Region_10400	chr2	74532258	74555690	23432	9	43	65.74242424	8.359813406	0.328520443	0.029465528	-	84695	grey	-0.053430969	FALSE	LOXL3
Region_10401	chr2	74549026	74557551	8525	3	10	18.74242424	4.521097913	0.01356846	0.029981337	+	1796	grey	-0.123244403	FALSE	DOK1
Region_10402	chr2	74557883	74648338	90455	22	146	178.3181818	14.07032785	0.773668964	0.030968295	-	130951	grey	-0.383101137	FALSE	M1AP
Region_10403	chr2	74654228	74683853	29625	16	79	111.8181818	11.96778894	0.788260923	0.018705906	+	10505	grey	-0.01522542	FALSE	SEMA4F
Region_10404	chr2	74715269	74805979	90710	34	226	260.1363636	14.53365999	0.844378926	0.03185825	+	102724497	grey	-0.19464671	FALSE	LOC102724497
Region_10405	chr2	74723873	74778780	54907	21	138	163.469697	12.47301049	0.881100897	0.038758175	-	102724482	grey	-0.196634065	FALSE	LOC102724482
Region_10406	chr2	74834127	74893359	59232	30	217	254.6666667	20.442289	0.630882824	0.025069456	+	3099	grey	0.055211146	FALSE	HK2
Region_10407	chr2	74918148	74938418	20270	3	13	22.31818182	3.717563681	0.576320474	0.086315526	+	102724515	grey	0.018885555	FALSE	LINC01291
Region_10408	chr2	75046463	75199520	153057	28	212	242.6515152	18.0967421	0.89662848	0.02131159	-	6869	grey	-0.140252954	FALSE	TACR1
Region_10409	chr2	75154366	75189949	35583	7	45	65.06060606	7.699907668	0.895046855	0.04275071	+	105374811	grey	0.172809387	FALSE	LOC105374811
Region_10410	chr2	75469302	75569722	100420	10	52	73.45454545	8.809403749	0.716006161	0.043750221	-	84141	grey	-0.118177785	FALSE	EVA1A
Region_10411	chr2	75646783	75690851	44068	8	52	71.39393939	9.314800114	0.855585581	0.020160315	+	9801	grey	0.040376754	FALSE	MRPL19
Region_1041	chr2	75652000	75710985	58985	6	33	51.4242424	8.79171044	0.97775993	0.02484686	-	6936	grey	0.031487175	FALSE	GCFC2

2							2	2	6	7						
Region_10413	chr2	76747685	77593196	845511	71	525	571.2121212	24.63175883	0.794980807	0.019797918	-	80059	grey	0.071198338	FALSE	LRRTM4
Region_10414	chr2	77652025	78290469	638444	35	230	284.1060606	16.2140763	0.840345323	0.027542204	-	101927967	grey	-0.014617288	FALSE	LOC101927967
Region_10415	chr2	78088729	78127806	39077	3	14	23.33333333	4.442221666	0.910008538	0.061928305	+	101927948	grey	0.203170353	FALSE	LOC101927948
Region_10416	chr2	78880713	78931146	50433	3	12	23.10606061	4.61145326	0.95517407	0.040861174	-	105374822	grey	-0.000347438	FALSE	LOC105374822
Region_10417	chr2	79185231	80648861	1463630	144	1172	1248.272727	33.65962907	0.819901951	0.015347288	+	1496	grey	0.036610051	FALSE	CTNNA2
Region_10418	chr2	79269905	79292878	22973	3	10	20.48484848	3.997085185	0.890900416	0.077269906	-	105374823	grey	-0.142319017	FALSE	LOC105374823
Region_10419	chr2	79281464	79340171	58707	4	15	29.53030303	5.090623951	0.89798858	0.06476551	+	107985902	grey	-0.145101004	FALSE	
Region_10420	chr2	84516455	84819589	303134	25	192	226.6212121	13.49617057	0.90401798	0.019416955	+	1768	grey	0.184446887	FALSE	DNAH6
Region_10421	chr2	84821650	84881975	60325	10	76	95.25757576	8.391040288	0.814543594	0.026521027	-	129293	grey	-0.025834317	FALSE	TRABD2A
Region_10422	chr2	84870589	84907008	36419	6	26	48.13636364	7.259765547	0.595979959	0.029351687	-	105374836	grey	-0.211487162	FALSE	LOC105374836
Region_10423	chr2	84926019	84966685	40666	8	45	70.89393939	8.615065137	0.783395931	0.042164752	+	105374838	grey	0.186453724	FALSE	
Region_10424	chr2	84971093	85059472	88379	12	71	99.68181818	9.705128051	0.957883277	0.029694847	+	56888	grey	-0.195299202	FALSE	KCMF1

Region_10425	chr2	85133392	85310387	176995	65	459	510.1363636	23.4369113	0.764293211	0.019225742	+	83439	grey	0.198015431	FALSE	TCF7L1
Region_10426	chr2	85213623	85302494	88871	22	158	190.1212121	13.71076517	0.851165009	0.028248279	+	102724579	grey	0.199796183	FALSE	LOC102724579
Region_10427	chr2	85318027	85328251	10224	7	42	62.04545455	10.28281886	0.031755988	0.026598924	-	10618	grey	-0.195825281	FALSE	TGOLN2
Region_10428	chr2	85341955	85354531	12576	4	20	30.21212121	5.039887519	0.73238721	0.025122918	-	54884	grey	0.179760414	FALSE	RETSAT
Region_10429	chr2	85354394	85391752	37358	23	143	174.1212121	13.56070698	0.872396631	0.02954468	+	84173	grey	0.065134351	FALSE	ELMOD3
Region_10430	chr2	85394753	85418432	23679	16	98	114.8030303	8.804229223	0.7332527	0.040775961	-	822	grey	-0.094598569	FALSE	CAPG
Region_10431	chr2	85418504	85437029	18525	9	48	64.54545455	8.029142723	0.64620479	0.040530785	+	284948	grey	-0.091191607	FALSE	SH2D6
Region_10432	chr2	85539168	85545281	6113	8	57	79.59090909	11.20161702	0.223902743	0.034939589	+	4144	grey	-0.091538826	FALSE	MAT2A
Region_10433	chr2	85544720	85561532	16812	13	75	107.6212121	13.42416323	0.800018607	0.029391813	-	2677	grey	0.025304262	FALSE	GGCX
Region_10434	chr2	85561562	85582031	20469	19	121	150.7121212	12.47368325	0.55814289	0.019149405	+	8673	grey	0.26131533	FALSE	VAMP8
Region_10435	chr2	85598547	85603196	4649	4	16	29.54545455	5.438274099	0.724057229	0.069299273	-	129303	grey	-0.476224673	FALSE	TMEM150A
Region_10436	chr2	85602856	85649283	46427	25	162	197.2121212	15.21994153	0.819991928	0.015488019	+	10713	grey	-0.014671469	FALSE	USP39
Region_1043	chr2	85605254	85612066	6812	7	33	50.8939393	6.54012182	0.72443895	0.04119505	-	388969	grey	0.077937139	FALSE	C2orf68

7							9	4	9	4						
Region_10438	chr2	85657314	85668741	11427	6	36	51.1969697	7.805756647	0.840193275	0.047039345	-	6439	grey	0.019665271	FALSE	SFTPB
Region_10439	chr2	85685175	85698852	13677	5	25	39.60606061	6.416034692	0.887015384	0.045997487	+	10578	grey	0.079034254	FALSE	GNLY
Region_10440	chr2	85751344	85791383	40039	23	147	184.3484848	17.74823278	0.491097048	0.029563874	+	84913	grey	0.174229295	FALSE	ATOH8
Region_10441	chr2	85815130	85825391	10261	6	33	44.04545455	6.26941739	0.886638384	0.05531244	+	284950	grey	0.347262478	FALSE	LOC284950
Region_10442	chr2	85837120	85905199	68079	29	209	258.469697	15.68950624	0.681897462	0.016706911	-	8869	grey	-0.206051595	FALSE	ST3GAL5
Region_10443	chr2	86020216	86106155	85939	26	192	220.3484848	14.30061045	0.848188238	0.021316396	-	25885	grey	-0.297700017	FALSE	POLR1A
Region_10444	chr2	86106223	86142157	35934	6	44	59.86363636	7.064290184	0.776897223	0.032062762	+	55037	grey	0.004692322	FALSE	PTCD3
Region_10445	chr2	86143932	86195472	51540	15	93	119.1969697	12.19359188	0.944237014	0.026148006	-	10989	grey	0.034581166	FALSE	IMMT
Region_10446	chr2	86199355	86213790	14435	11	70	89.74242424	9.142143506	0.968423778	0.027304996	+	51318	grey	0.123761245	FALSE	MRPL35
Region_10447	chr2	86213993	86338083	124090	36	253	297.2272727	18.20802482	0.797176929	0.027405568	-	65055	grey	0.110049197	FALSE	REEP1
Region_10448	chr2	86440647	86492716	52069	9	58	73.95454545	7.923541978	0.543803146	0.017628096	+	55818	grey	0.030276056	FALSE	KDM3A
Region_10449	chr2	86503430	86563479	60049	11	58	80.21212121	10.02537573	0.613692089	0.020985729	-	51652	grey	-0.071134189	FALSE	CHMP3

Region_10450	chr2	86505668	86721122	215454	32	212	256.6363636	18.31320361	0.696600376	0.012194655	-	100526767	grey	-0.127566152	FALSE	RNF103-CHMP3
Region_10451	chr2	86510424	86623866	113442	18	111	139.969697	12.32504604	0.641463305	0.015203415	-	7844	grey	-0.010309373	FALSE	RNF103
Region_10452	chr2	86562070	86618766	56696	9	55	74.27272727	8.790808932	0.533497336	0.01795505	+	105374845	grey	0.11427113	FALSE	CHMP3-AS1
Region_10453	chr2	86720291	86778041	57750	11	59	77.21212121	10.33070874	0.709547205	0.036986295	+	64795	grey	-0.060786007	FALSE	RMND5A
Region_10454	chr2	86784610	86808396	23786	17	116	165.9393939	19.91665618	0.197703434	0.029419972	-	925	grey	-0.251178027	FALSE	CD8A
Region_10455	chr2	86815339	86861924	46585	12	78	102.2424242	10.14064268	0.773237292	0.035422613	-	926	grey	0.06810211	FALSE	CD8B
Region_10456	chr2	86861825	86912978	51153	7	27	35.71212121	5.459684836	0.826075848	0.041903273	+	100286979	grey	-0.082476894	FALSE	ANAPC1P1
Region_10457	chr2	86907953	87013976	106023	10	36	54.62121212	9.509129462	0.960477954	0.034685952	+	400966	grey	-0.134418564	FALSE	RGPD1
Region_10458	chr2	87030675	87076429	45754	7	28	43.15151515	6.810043317	0.715172619	0.056873502	-	285074	grey	-0.287142532	FALSE	ANAPC1P2
Region_10459	chr2	87075653	87118267	42614	10	40	52.28787879	6.39414432	0.664647133	0.041743076	+	107985772	grey	-0.027239651	FALSE	
Region_10460	chr2	87125198	87196629	71431	10	36	58.36363636	9.828743348	0.945752199	0.03595278	+	102724642	grey	0.007255997	FALSE	LOC102724642
Region_10461	chr2	87311460	87348728	37268	3	13	25.66666667	6.776278383	0.861268485	0.099948837	-	105374848	grey	0.006644282	FALSE	LOC105374848
Region_1046	chr2	87454781	87636740	181959	28	168	205.969697	27.5507753	0.61191647	0.03160036	+	112597	grey	0.07251008	FALSE	CYTOR

2								3		2						
Region_10463	chr2	87455476	87767359	311883	42	259	302.2121212	37.11002981	0.700303844	0.025603202	+	730268	grey	0.085310221	FALSE	ANAPC1P4
Region_10464	chr2	87755960	87992858	236898	18	103	140.3181818	13.78534237	0.892343207	0.028994359	-	729857	grey	-0.131247491	FALSE	RGPD2
Region_10465	chr2	88027205	88064252	37047	8	33	48.33333333	7.277644026	0.747250164	0.031139445	-	51315	grey	-0.045595289	FALSE	KRCC1
Region_10466	chr2	88067825	88113384	45559	17	110	140.5757576	12.10729037	0.943829514	0.025767948	+	150572	grey	0.162494258	FALSE	SMYD1
Region_10467	chr2	88170295	88186636	16341	3	14	24.95454545	5.559280026	0.862894115	0.072369952	+	55258	grey	0.022372342	FALSE	THNSL2
Region_10468	chr2	88538720	88575610	36890	5	32	46.66666667	6.308317668	0.756235285	0.046964257	+	101928371	grey	0.115525997	FALSE	LOC101928371
Region_10469	chr2	88556741	88691476	134735	17	130	154.2121212	11.77286992	0.882466112	0.022853351	-	9451	grey	-0.198568032	FALSE	EIF2AK3
Region_10470	chr2	88691673	88750929	59256	7	40	59.57575758	7.365325427	0.901421138	0.04926152	+	22934	grey	0.124170664	FALSE	RPIA
Region_10471	chr2	88765807	88806612	40805	5	24	43.84848485	7.961914003	0.917156506	0.045479144	+	645784	grey	0.223554739	FALSE	ANKRD36BP2
Region_10472	chr2	88811186	88861563	50377	11	73	94.24242424	10.02774705	0.878582453	0.04627858	-	105374855	grey	-0.248261232	FALSE	MALLP2
Region_10473	chr2	91587019	91660038	73019	12	148	177.1666667	13.41707676	0.865412917	0.03532087	-	654342	grey	0.064927273	FALSE	LSP1P4
Region_10474	chr2	91686102	91714745	28643	7	35	48.40909091	6.644783432	0.819655134	0.059359715	-	107985798	grey	-0.046267102	FALSE	KMT2CP5

Region_10475	chr2	91775944	91781169	5225	5	25	36.45454545	6.023497114	0.888443849	0.064052677	+	645367	grey	-0.121030856	FALSE	GGT8P
Region_10476	chr2	95297327	95386077	88750	14	81	105.0606061	11.36239247	0.488118836	0.027250121	+	30818	grey	-0.015580058	FALSE	KCNIP3
Region_10477	chr2	95402708	95416616	13908	6	43	58.77272727	7.846205265	0.487903917	0.027605905	+	51011	grey	-0.288059637	FALSE	FAHD2A
Region_10478	chr2	95660588	95668727	8139	4	12	22.10606061	6.154744981	0.674048016	0.065061591	+	101926959	grey	0.045904542	FALSE	LOC101926959
Region_10479	chr2	95807052	95833963	26911	7	45	61.18181818	6.852083548	0.970356258	0.022917205	-	150759	grey	-0.0346993	FALSE	LINC00342
Region_10480	chr2	95836919	95991831	154912	25	167	202.7727273	16.13290517	0.930777921	0.018033347	-	400986	grey	-0.11402761	FALSE	ANKRD36C
Region_10481	chr2	96010526	96023398	12872	12	57	85.37878788	10.73995438	0.883012225	0.040372405	+	729234	grey	-0.04530695	FALSE	FAHD2CP
Region_10482	chr2	96112876	96116571	3695	5	14	25.63636364	5.864197404	0.499722432	0.082310147	-	151	grey	0.024643373	FALSE	ADRA2B
Region_10483	chr2	96122818	96138502	15684	6	28	38.83333333	6.482125324	0.961821832	0.03548634	-	431705	grey	-0.042256369	FALSE	ASTL
Region_10484	chr2	96184859	96208827	23968	5	18	35.74242424	6.431438662	0.953909417	0.035778758	-	56910	grey	0.230401303	FALSE	STARD7
Region_10485	chr2	96208389	96243353	34964	14	97	119.4242424	12.53556247	0.693930703	0.024379276	+	285033	grey	-0.196675439	FALSE	STARD7-AS1
Region_10486	chr2	96248514	96265997	17483	7	52	69.39393939	8.283215258	0.717827066	0.043598763	-	55654	grey	-0.043459645	FALSE	TMEM127
Region_1048	chr2	96274338	96321271	46933	13	94	115.893939	10.2448918	0.90753524	0.02713104	-	23020	grey	0.084593314	FALSE	SNRNP200

7							4	4	3	7						
Region_10488	chr2	96335766	96377091	41325	8	55	69.96969697	7.472966041	0.853086613	0.045882588	+	23397	grey	0.129224585	FALSE	NCAPH
Region_10489	chr2	96536743	96552634	15891	7	35	48.78787879	6.198052179	0.827911503	0.026980844	+	10865	grey	0.123723605	FALSE	ARID5A
Region_10490	chr2	96593170	96642787	49617	11	67	85.71212121	8.600116549	0.736739358	0.028539878	-	55683	grey	-0.090062742	FALSE	KANSL3
Region_10491	chr2	96642737	96704887	62150	33	231	259.1818182	15.03831935	0.924930283	0.016839086	+	90342	grey	0.093248659	FALSE	FER1L5
Region_10492	chr2	96705929	96740064	34135	19	121	157.9393939	13.60642245	0.961406467	0.021372475	-	81562	grey	-0.064250015	FALSE	LMAN2L
Region_10493	chr2	96760902	96811874	50972	29	199	232.7424242	15.89321152	0.772612603	0.019864924	+	26504	grey	-0.089587117	FALSE	CNNM4
Region_10494	chr2	96816245	96835382	19137	13	87	110.8181818	9.826039332	0.532863227	0.026313152	+	26505	grey	-0.174246682	FALSE	CNNM3
Region_10495	chr2	96824526	96858014	33488	15	87	110.1969697	10.50892616	0.882491316	0.027652139	-	200539	grey	0.085328107	FALSE	ANKRD23
Region_10496	chr2	96847990	96858016	10026	8	46	61.46969697	8.023465004	0.848873182	0.037946007	-	51239	grey	0.080339427	FALSE	ANKRD39
Region_10497	chr2	96859718	96870757	11039	7	16	29.84848485	6.661583143	0.147967617	0.026845195	-	54910	grey	0.094564392	FALSE	SEMA4C
Region_10498	chr2	96875882	96986580	110698	51	336	384.3939394	20.08817393	0.866143669	0.016405419	-	51252	grey	-0.037661805	FALSE	FAM178B
Region_10499	chr2	97113153	97264521	151368	28	184	259	32.42601045	0.772992828	0.029095156	+	375248	grey	-0.325244668	FALSE	ANKRD36

Region_10500	chr2	97492663	97589877	97214	10	62	85.07575758	10.38387893	0.788401923	0.032631748	-	57730	grey	-0.02085259	FALSE	ANKRD36B
Region_10501	chr2	97646062	97648383	2321	3	13	26.28787879	4.586821093	0.653740634	0.032541483	+	1329	grey	0.04460237	FALSE	COX5B
Region_10502	chr2	97664217	97703066	38849	14	104	129.4393939	10.74190756	0.693663294	0.020446258	+	728537	grey	0.100618955	FALSE	C2orf92
Region_10503	chr2	97713576	97739862	26286	12	63	81.36363636	9.481376667	0.898460426	0.038289743	+	7535	grey	-0.029126555	FALSE	ZAP70
Region_10504	chr2	97756333	97995948	239615	48	349	401.3484848	27.4498101	0.800065399	0.019819543	-	23505	grey	0.095152748	FALSE	TMEM131
Region_10505	chr2	98087116	98313299	226183	41	283	324.7575758	17.45416909	0.866144166	0.021825339	+	200403	grey	0.375119544	FALSE	VWA3B
Region_10506	chr2	98346188	98398601	52413	32	248	287.6212121	17.78570312	0.780680322	0.02136406	+	1261	grey	-0.026002558	FALSE	CNGA3
Region_10507	chr2	98444854	98594392	149538	21	143	173.2727273	12.63760622	0.725595219	0.026679514	+	3631	grey	-0.01053292	FALSE	INPP4A
Region_10508	chr2	98599314	98608515	9201	3	16	28.6969697	5.62950933	0.40455961	0.106957169	-	493753	grey	-0.324014405	FALSE	COA5
Region_10509	chr2	98608579	98618515	9936	5	28	42.25757576	7.010564788	0.71550553	0.055916236	+	25972	grey	0.076917876	FALSE	UNC50
Region_10510	chr2	98619106	98731132	112026	24	193	223	13.84863226	0.824392053	0.021689462	-	11320	grey	-0.194206082	FALSE	MGAT4A
Region_10511	chr2	98761098	98772957	11859	4	19	31.48484848	5.239619552	0.476004452	0.08690332	-	101927070	grey	0.033342976	FALSE	LINC02611
Region_1051	chr2	98793846	98936259	142413	27	180	220.015151	16.2001827	0.80589751	0.03307383	-	343990	grey	-0.34602293	FALSE	CRACDL

2							5	4	6	8				9		
Region_10513	chr2	98997261	99154964	157703	35	225	283.969697	22.33863139	0.855997906	0.020241387	-	80705	grey	-0.000613105	FALSE	TSGA10
Region_10514	chr2	99141485	99322733	181248	45	336	386.9393939	22.97616075	0.902575399	0.01556703	+	51263	grey	-0.060962203	FALSE	MRPL30
Region_10515	chr2	99141707	99151669	9962	5	16	34.65151515	7.154326124	0.971716603	0.035123539	+	150590	grey	-0.192816262	FALSE	C2orf15
Region_10516	chr2	99161427	99181058	19631	5	28	43.3030303	6.221475702	0.637146397	0.049139231	-	129531	grey	-0.109456313	FALSE	MITD1
Region_10517	chr2	99284238	99304742	20504	6	40	58.46969697	8.942929647	0.966763657	0.023492037	-	129530	grey	0.175602046	FALSE	LYG1
Region_10518	chr2	99307442	99322741	15299	3	16	26.62121212	4.838197857	0.968822578	0.037622912	+	107985923	grey	-0.125018932	FALSE	LOC107985923
Region_10519	chr2	99318982	99340702	21720	11	66	87.89393939	9.623894602	0.800649811	0.018260828	-	10190	grey	-0.074491229	FALSE	TXNDC9
Region_10520	chr2	99337371	99401326	63955	17	87	112.2878788	11.47004815	0.974499982	0.013125166	+	9669	grey	-0.208335521	FALSE	EIF5B
Region_10521	chr2	99400475	99490035	89560	21	141	171.2727273	13.22532007	0.71200206	0.019617067	-	51455	grey	0.364649824	FALSE	REV1
Region_10522	chr2	99545419	100192428	647009	130	972	1084.545455	38.63684245	0.880432012	0.011254874	-	3899	grey	0.077909076	FALSE	AFF3
Region_10523	chr2	100208254	100251484	43230	12	90	115.3939394	10.27300988	0.878776595	0.027019589	+	150577	grey	0.131461789	FALSE	LINC01104
Region_10524	chr2	100271875	100322501	50626	9	54	79.21212121	9.487668355	0.742550467	0.028399434	-	164832	grey	0.107805385	FALSE	LONRF2

Region_10525	chr2	100391860	100417668	25808	9	54	77.57575758	9.356298752	0.901019213	0.053203105	-	9486	grey	0.192874304	FALSE	CHST10
Region_10526	chr2	100562993	100576739	13746	9	51	69.37878788	11.93542259	0.81746223	0.041672753	+	79031	grey	0.213746496	FALSE	PDCL3
Region_10527	chr2	100820139	100996829	176690	59	457	518.8939394	27.05329713	0.882509891	0.015656916	+	4862	grey	0.240454964	FALSE	NPAS2
Region_10528	chr2	101002229	101024032	21803	9	71	85.45454545	7.66848058	0.600335237	0.029284342	+	6160	grey	0.257327313	FALSE	RPL31
Region_10529	chr2	101007228	101252866	245638	69	472	529.3030303	23.86563006	0.832186877	0.014170857	-	11138	grey	-0.012286391	FALSE	TBC1D8
Region_10530	chr2	101271219	101308701	37482	9	48	65.75757576	7.759977411	0.686563079	0.026354567	-	284996	grey	0.197821227	FALSE	RNF149
Region_10531	chr2	101345550	101387503	41953	6	33	42.92424242	5.905175321	0.634139001	0.076134969	-	200407	grey	-0.131900301	FALSE	CREG2
Region_10532	chr2	101397359	101475112	77753	36	201	252.5757576	21.33690238	0.741598716	0.032328259	-	731220	grey	0.44054652	FALSE	RFX8
Region_10533	chr2	101696850	101894690	197840	43	324	355.6363636	16.78794106	0.818747104	0.019588485	+	9448	grey	0.203327977	FALSE	MAP4K4
Region_10534	chr2	101991960	102028544	36584	15	92	122.530303	12.59819242	0.943989411	0.023767255	+	7850	grey	0.192307996	FALSE	IL1R2
Region_10535	chr2	102037963	102058190	20227	9	56	73.03030303	6.996635888	0.678047155	0.047249744	+	107985926	grey	0.110384532	FALSE	LOC107985926
Region_10536	chr2	102064544	102179874	115330	23	152	185.2424242	16.85052896	0.684233922	0.02449273	+	3554	grey	0.43493448	FALSE	IL1R1
Region_1053	chr2	10218700	10224000	52996	15	90	118.727272	13.5981282	0.74401431	0.05535936	+	8808	grey	0.004290608	FALSE	IL1RL2

7		6	2				7	1	2	6						
Region_10538	chr2	102311502	102352037	40535	9	55	76.28787879	7.824547471	0.925170423	0.045233676	+	9173	grey	-0.090746553	FALSE	IL1RL1
Region_10539	chr2	102311529	102398777	87248	18	132	150.7121212	11.24655866	0.850970386	0.038114752	+	8809	grey	-0.204219548	FALSE	IL18R1
Region_10540	chr2	102473226	102533972	60746	9	61	83.65151515	10.41373712	0.856015569	0.052312441	+	389015	grey	-0.194049294	FALSE	SLC9A4
Region_10541	chr2	102619553	102711355	91802	13	75	93.42424242	8.686081893	0.695915866	0.040128972	+	6549	grey	0.293539856	FALSE	SLC9A2
Region_10542	chr2	102736905	103019900	282995	31	247	287.5151515	15.80481175	0.849504748	0.020767888	+	130827	grey	0.020124202	FALSE	TMEM182
Region_10543	chr2	102873183	102895780	22597	3	19	30.07575758	5.239819745	0.79377864	0.071693171	-	105373518	grey	0.258335876	FALSE	LINC01796
Region_10544	chr2	103855829	103880209	24380	4	28	38.68181818	4.971175657	0.836748357	0.073024549	-	107985927	grey	0.015397827	FALSE	LOC107985927
Region_10545	chr2	103874236	104101537	227301	15	91	115.6666667	10.19401534	0.875564467	0.04404025	+	107985928	grey	0.245972663	FALSE	LINC01965
Region_10546	chr2	104430130	104532747	102617	12	81	101.3333333	8.765901302	0.798841796	0.035849688	+	150568	grey	0.008139134	FALSE	LINC01102
Region_10547	chr2	104488458	104510509	22051	3	10	19.92424242	4.304954591	0.961353349	0.047419641	-	101927360	grey	0.106416026	FALSE	LINC01103
Region_10548	chr2	104583138	104621767	38629	3	14	23	4.564073754	0.806530166	0.083546368	+	105373525	grey	-0.012434443	FALSE	LOC105373525
Region_10549	chr2	104764932	104853183	88251	17	111	138.1515152	12.55908182	0.565391972	0.025637366	-	100506421	grey	0.132685008	FALSE	PANTR1

Region_10550	chr2	104854115	104858574	4459	4	11	19.96969697	5.126011186	0.099097574	0.071726911	+	5455	grey	-0.27730911	FALSE	POU3F3
Region_10551	chr2	104865497	104872653	7156	3	13	22.84848485	4.651690922	0.054835465	0.051200276	-	102682016	grey	-0.216690861	FALSE	LINC01159
Region_10552	chr2	104936239	105038496	102257	16	112	145.6666667	13.07532419	0.814085731	0.034687673	-	102724691	grey	0.088922389	FALSE	MRPS9-AS2
Region_10553	chr2	105038069	105099960	61891	5	27	42.68181818	6.466860113	0.765529287	0.032330626	+	64965	grey	0.18456985	FALSE	MRPS9
Region_10554	chr2	105264391	105329735	65344	21	154	189.0757576	16.47217757	0.899068906	0.018054451	-	9392	grey	-0.143610756	FALSE	TGFBRAP1
Region_10555	chr2	105357712	105438513	80801	22	128	165.1818182	15.01886925	0.624252473	0.030158527	-	2274	grey	-0.213424152	FALSE	FHL2
Region_10556	chr2	105744912	105894274	149362	46	345	390.2575758	23.88968919	0.854570786	0.022615407	+	8440	grey	0.012716485	FALSE	NCK2
Region_10557	chr2	106063246	106078155	14909	5	30	42.81818182	5.932723051	0.772873869	0.041684282	+	84417	grey	0.308076155	FALSE	ECRG4
Region_10558	chr2	106093308	106194301	100993	26	145	189.3939394	16.06322009	0.755819895	0.022267627	-	80146	grey	0.094576868	FALSE	UXS1
Region_10559	chr2	106382110	106393815	11705	4	17	27.96969697	5.756520576	0.868851708	0.090387765	+	285189	grey	-0.091034601	FALSE	PLGLA
Region_10560	chr2	106391290	106468413	77123	19	87	114.3484848	13.60711627	0.941062626	0.023688022	-	653489	grey	-0.044458461	FALSE	RGPD3
Region_10561	chr2	106487364	106544297	56933	22	131	164.9848485	13.43416131	0.816459	0.033021581	+	927	grey	-0.061073825	FALSE	CD8B2
Region_1056	chr2	10680160	10688710	85508	28	184	221.151515	16.3361255	0.50848468	0.02041242	-	84620	grey	-0.05080011	FALSE	ST6GAL2

2		0	8				2	4		9				2		
Region_1056<br>3	chr2	10725469<br>1	10736587<br>3	111182	9	65	85.3484848<br>5	8.30475753<br>2	0.83820437<br>6	0.04249396<br>7	-	10537353<br>6	grey	-0.03178951<br>3	FALSE	LINC01789
Region_1056<br>4	chr2	10738271<br>5	10774827<br>0	365555	40	301	333.575757<br>6	18.9306918<br>9	0.84662775<br>9	0.02515538<br>6	-	10537353<br>8	grey	-0.05840385<br>3	FALSE	LINC01885
Region_1056<br>5	chr2	10775411<br>2	10782212<br>9	68017	10	66	87.1212121<br>2	8.74337578<br>1	0.86349151	0.04134262<br>8	+	10432605<br>7	grey	0.057541647	FALSE	GACAT1
Region_1056<br>6	chr2	10782689<br>2	10789254<br>4	65652	6	15	25.5303030<br>3	5.73577088<br>1	0.83523348<br>6	0.08118380<br>3	+	285190	grey	-0.10825794<br>7	FALSE	RGPD4
Region_1056<br>7	chr2	10816712<br>5	10821788<br>6	50761	4	29	43.2424242<br>4	6.67039522<br>8	0.84905035<br>4	0.09229030<br>9	-	10272477<br>4	grey	-0.36363294<br>4	FALSE	LINC01594
Region_1056<br>8	chr2	10828863<br>9	10830991<br>5	21276	5	22	35.2575757<br>6	6.46008003<br>3	0.92877725<br>7	0.04273917<br>1	+	6819	grey	-0.01274430<br>1	FALSE	SULT1C2
Region_1056<br>9	chr2	10832223<br>8	10835379<br>9	31561	3	13	20.9848484<br>8	4.66242731<br>2	0.89119267	0.06767000<br>5	+	151234	grey	0.265193092	FALSE	SULT1C5P
Region_1057<br>0	chr2	10837791<br>1	10838898<br>9	11078	3	15	23.9242424<br>2	4.78237342<br>4	0.95423187<br>5	0.05042784<br>1	+	27233	grey	-0.07334004	FALSE	SULT1C4
Region_1057<br>1	chr2	10844910<br>7	10850941<br>5	60308	14	80	107.954545<br>5	10.8449659<br>6	0.80769482<br>1	0.03264581<br>8	+	9648	grey	0.251064238	FALSE	GCC2
Region_1057<br>2	chr2	10850751<br>5	10853419<br>6	26681	7	27	41.5909090<br>9	6.10164485<br>4	0.78818387<br>6	0.04983035<br>9	-	644903	grey	-0.29059523<br>8	FALSE	GCC2-AS1
Region_1057<br>3	chr2	10853367<br>1	10868724<br>6	153575	14	96	118.893939<br>4	10.3375771<br>4	0.89833589<br>6	0.03266237<br>3	+	3987	grey	0.06988576	FALSE	LIMS1
Region_1057<br>4	chr2	10871948<br>2	10878581<br>0	66328	6	31	49.0606060<br>6	6.72967889<br>9	0.78119078<br>1	0.04499190<br>8	+	5903	grey	0.06342405	FALSE	RANBP2

Region_10575	chr2	108786757	108885477	98720	16	83	107.7575758	8.670126618	0.85146003	0.024235729	+	165055	grey	0.037312447	FALSE	CCDC138
Region_10576	chr2	108894471	108989372	94901	18	118	138.6515152	9.295975017	0.873335748	0.027720416	-	10913	grey	0.11303486	FALSE	EDAR
Region_10577	chr2	109127327	109128930	1603	4	12	28.09090909	6.406072294	0.005372174	0.013737226	-	100287216	grey	-0.081031502	FALSE	SH3RF3-AS1
Region_10578	chr2	109129205	109504634	375429	123	934	1037.348485	43.47501225	0.858024103	0.011936518	+	344558	grey	0.090030768	FALSE	SH3RF3
Region_10579	chr2	109542799	109614143	71344	33	204	246.469697	17.99591381	0.794460715	0.021057113	-	151011	grey	-0.224689554	FALSE	SEPTIN10
Region_10580	chr2	110022447	110050064	27617	5	34	46.6969697	7.049210969	0.897086126	0.052423674	-	105373985	grey	0.117956904	FALSE	LOC105373985
Region_10581	chr2	110083870	110116022	32152	9	50	68.12121212	8.006757053	0.903875964	0.05074457	-	7851	grey	0.075264593	FALSE	MALL
Region_10582	chr2	110234735	110285856	51121	5	26	44.39393939	7.206575604	0.595637149	0.041498544	+	100507334	grey	0.367825779	FALSE	LOC100507334
Region_10583	chr2	110321402	110348941	27539	3	17	25.27272727	4.334283561	0.917624032	0.082888121	+	105373551	grey	-0.224579986	FALSE	LOC105373551
Region_10584	chr2	110386411	110473075	86664	5	25	51.10606061	9.390877387	0.715265098	0.072383298	-	100288695	grey	-0.011524519	FALSE	LIMS4
Region_10585	chr2	110637528	110678063	40535	8	48	67.86363636	7.739780088	0.743438464	0.025299615	-	699	grey	-0.149975789	FALSE	BUB1
Region_10586	chr2	110732539	111120610	388071	78	549	636.5909091	33.04634381	0.798871553	0.016130687	+	55289	grey	0.257579892	FALSE	ACOXL
Region_1058	chr2	11100601	11169903	693018	128	923	1009.06060	38.2308288	0.75658893	0.01284686	-	541471	grey	0.003707206	FALSE	MIR4435-2HG

7		5	3				6		8	4						
Region_10588	chr2	111098345	111116407	18062	3	15	25.25757576	4.701262044	0.955503622	0.039340214	-	400997	grey	0.024271456	FALSE	ACOXL-AS1
Region_10589	chr2	111119378	111168445	49067	18	117	143.5909091	13.18037851	0.582597215	0.018954397	+	10018	grey	-0.172834591	FALSE	BCL2L11
Region_10590	chr2	111265283	111279880	14597	6	24	44.65151515	6.569140968	0.937368131	0.060822781	+	101927283	grey	0.2826342	FALSE	LOC101927283
Region_10591	chr2	111491272	111570974	79702	13	94	116.7424242	10.58200302	0.828441234	0.034742319	+	105373557	grey	0.166135893	FALSE	SOCAR
Region_10592	chr2	111593594	111631040	37446	10	54	70.84848485	7.340598224	0.606423137	0.058080128	-	107985933	grey	0.148193361	FALSE	LOC107985933
Region_10593	chr2	111611639	111884690	273051	51	328	380.9242424	23.11796545	0.792100662	0.018986277	-	64682	grey	-0.156147559	FALSE	ANAPC1
Region_10594	chr2	111898607	112029561	130954	30	216	251.1212121	13.16348697	0.793448987	0.019791495	+	10461	grey	0.068566275	FALSE	MERTK
Region_10595	chr2	112055269	112119318	64049	13	72	93.68181818	10.07992535	0.95288123	0.030032834	+	84910	grey	0.132677158	FALSE	TMEM87B
Region_10596	chr2	112138385	112244375	105990	30	225	261.5151515	17.11251672	0.85547442	0.021316043	+	129804	grey	-0.021669381	FALSE	FBLN7
Region_10597	chr2	112211529	112255136	43607	12	81	103.3636364	9.940803812	0.800035236	0.018491682	-	84524	grey	-0.014775518	FALSE	ZC3H8
Region_10598	chr2	112275597	112340063	64466	20	129	158.9242424	15.27019279	0.734308021	0.025166142	+	376940	grey	0.363891866	FALSE	ZC3H6
Region_10599	chr2	112368369	112434488	66119	10	46	63.06060606	7.93992596	0.769549048	0.054632875	-	727851	grey	-0.019021742	FALSE	RGPD8

Region_10600	chr2	112482156	112541739	59583	13	74	96.83333333	10.75543989	0.81786291	0.023903941	+	150465	grey	0.472323834	FALSE	TTL
Region_10601	chr2	112541915	112579818	37903	6	28	43.06060606	5.833270063	0.800404795	0.03036724	+	84172	grey	-0.274042714	FALSE	POLR1B
Region_10602	chr2	112584240	112589275	5035	7	41	60.84848485	8.046484993	0.260659944	0.025102317	+	84269	grey	-0.067850584	FALSE	CHCHD5
Region_10603	chr2	112645939	112663825	17886	12	57	82.98484848	11.30893782	0.861888844	0.02846112	+	6574	grey	0.11495558	FALSE	SLC20A1
Region_10604	chr2	112736349	112764664	28315	3	20	30.07575758	5.884296196	0.634861666	0.043956717	-	150468	grey	-0.099006397	FALSE	CKAP2L
Region_10605	chr2	112773925	112784493	10568	5	28	40.78787879	6.227766982	0.968677437	0.027150406	-	3552	grey	0.087181263	FALSE	IL1A
Region_10606	chr2	112829751	112836816	7065	3	11	23.93939394	4.209435511	0.602638986	0.053348214	-	3553	grey	0.20858244	FALSE	IL1B
Region_10607	chr2	112911165	112918882	7717	4	21	29.43939394	4.382232616	0.908322533	0.098108698	+	27178	grey	-0.344352033	FALSE	IL37
Region_10608	chr2	113022089	113052867	30778	16	104	135.530303	12.81972002	0.922815328	0.02788826	-	27177	grey	0.109104057	FALSE	IL36B
Region_10609	chr2	113099315	113134016	34701	6	33	47.98484848	7.66107529	0.933612097	0.037901446	+	3557	grey	0.234341091	FALSE	IL1RN
Region_10610	chr2	113157325	113209396	52071	20	145	172.6212121	13.86280597	0.813798389	0.022383529	+	23550	grey	-0.02648887	FALSE	PSD4
Region_10611	chr2	113211421	113276581	65160	22	125	160.0151515	13.47189944	0.646041215	0.049110135	+	654433	grey	0.206509959	FALSE	PAX8-AS1
Region_1061	chr2	11321599	11327892	62924	28	169	210.787878	16.8618961	0.47982369	0.03751736	-	7849	grey	0.22743338	FALSE	PAX8

2		7	1				8	9	2	1						
Region_10613	chr2	113325009	113430988	105979	25	171	197.7121212	14.5430556	0.817120734	0.022390954	+	101927400	grey	-0.123877158	FALSE	LINC02966
Region_10614	chr2	113370392	113374994	4602	3	23	31.81818182	5.212142896	0.369849411	0.04279366	-	107985936	grey	-0.212782367	FALSE	LOC107985936
Region_10615	chr2	113437691	113496204	58513	9	42	60.54545455	7.526735564	0.94019601	0.038563319	+	150472	grey	-0.038825404	FALSE	ZNG1B
Region_10616	chr2	113583593	113599081	15488	5	39	56.62121212	8.364329301	0.390644167	0.053224987	+	375260	grey	0.421190626	FALSE	WASH2P
Region_10617	chr2	113627229	113643396	16167	4	16	30.6969697	8.074593959	0.55809513	0.065047887	+	11159	grey	0.211684294	FALSE	RABL2A
Region_10618	chr2	113705011	113756693	51682	3	18	30.89393939	4.893099656	0.976378938	0.032096951	-	80255	grey	-0.059274262	FALSE	SLC35F5
Region_10619	chr2	113831049	113891020	59971	11	67	88.68181818	9.052256992	0.796088397	0.043181488	-	101060091	grey	0.081668939	FALSE	ACTR3-AS1
Region_10620	chr2	113890063	113962596	72533	9	56	77.45454545	9.530811279	0.817790456	0.045444248	+	10096	grey	-0.024221201	FALSE	ACTR3
Region_10621	chr2	113970719	114007304	36585	6	24	42.22727273	6.903277827	0.967713148	0.038500289	+	440900	grey	0.245562798	FALSE	LINC01191
Region_10622	chr2	114442299	115845780	1403481	149	1063	1164.909091	42.23477505	0.847067191	0.01809517	+	57628	grey	-0.006362954	FALSE	DPP10
Region_10623	chr2	115126622	115161199	34577	3	18	26.37878788	4.198040391	0.89672748	0.067087695	-	107985937	grey	-0.086324844	FALSE	LOC107985937
Region_10624	chr2	115130935	115161385	30450	3	18	26.37878788	4.198040391	0.89672748	0.067087695	-	389023	grey	-0.086324844	FALSE	DPP10-AS1

Region_10625	chr2	117860333	117934942	74609	9	53	68.07575758	8.980852463	0.939324464	0.034033768	+	101929908	grey	0.041902815	FALSE	LOC101929908
Region_10626	chr2	117915478	118014133	98655	13	98	121.5757576	10.47056035	0.866112239	0.032634993	-	54520	grey	-0.155014221	FALSE	CCDC93
Region_10627	chr2	118031840	118055033	23193	5	36	50.13636364	6.899021923	0.833570991	0.042822325	+	102723413	grey	-0.250247431	FALSE	LOC102723413
Region_10628	chr2	118088452	118110997	22545	3	14	21.77272727	4.543111284	0.97086046	0.040775568	+	51141	grey	0.03809388	FALSE	INSIG2
Region_10629	chr2	118132128	118222250	90122	7	44	62.86363636	7.912767412	0.946339565	0.041131636	-	100506797	grey	0.015667125	FALSE	THORLNC
Region_10630	chr2	118842171	118847648	5477	19	113	155.1515152	15.69835114	0.067369195	0.026454448	-	2019	grey	-0.005195773	FALSE	EN1
Region_10631	chr2	118942194	118994660	52466	6	42	59.71212121	7.607213483	0.772026215	0.04548972	+	8685	grey	0.016275654	FALSE	MARCO
Region_10632	chr2	119223831	119265652	41821	11	55	72.8030303	8.66508413	0.763350544	0.032598979	+	55240	grey	-0.012010934	FALSE	STEAP3
Region_10633	chr2	119244422	119270714	26292	3	14	22.83333333	5.11884402	0.975207558	0.035056532	-	100874111	grey	-0.027910327	FALSE	STEAP3-AS1
Region_10634	chr2	119302225	119366834	64609	11	68	86.98484848	9.645233856	0.935699375	0.033603436	-	130355	grey	0.019987095	FALSE	C2orf76
Region_10635	chr2	119429901	119438504	8603	6	30	48.56060606	6.928489208	0.616700072	0.044266834	+	140738	grey	-0.073319317	FALSE	TMEM37
Region_10636	chr2	119439843	119525301	85458	9	61	80.89393939	8.171486613	0.761400939	0.035593989	-	6344	grey	-0.078919328	FALSE	SCTR
Region_1063	chr2	11954443	11966225	117819	37	254	294.878787	16.9782803	0.83434002	0.02319655	+	200373	grey	0.278772858	FALSE	CFAP221

7		2	1				9	8	9	7						
Region_10638	chr2	119759922	119984899	224977	21	152	179.6060606	13.08537503	0.961781109	0.015879818	+	5775	grey	-0.059963017	FALSE	PTPN4
Region_10639	chr2	120013077	120179119	166042	35	225	263.8181818	16.04983498	0.896834809	0.015383372	+	57669	grey	0.052693805	FALSE	EPB41L5
Region_10640	chr2	120217479	120223400	5921	5	30	47.10606061	7.369644158	0.272562001	0.053930506	-	79134	grey	0.068309846	FALSE	TMEM185B
Region_10641	chr2	120240064	120294710	54646	10	69	90.16666667	9.335759758	0.785138219	0.036700595	+	5899	grey	0.099622003	FALSE	RALB
Region_10642	chr2	120346136	120351803	5667	8	46	64.12121212	8.17409633	0.275474565	0.047183133	+	3625	grey	-0.142117995	FALSE	INHBB
Region_10643	chr2	120542905	120558119	15214	5	30	40.75757576	5.355257966	0.763414041	0.061103176	-	105373585	grey	-0.152435177	FALSE	LOC105373585
Region_10644	chr2	120735623	120992653	257030	88	633	711.1363636	35.78434826	0.903345601	0.015004996	+	2736	grey	-0.040611942	FALSE	GLI2
Region_10645	chr2	121216587	121285202	68615	37	229	293	18.34288795	0.823844971	0.019803624	-	29842	grey	-0.041082172	FALSE	TFCP2L1
Region_10646	chr2	121337776	121649476	311700	95	738	808.6212121	34.94204659	0.905913497	0.009003978	-	23332	grey	0.080896234	FALSE	CLASP1
Region_10647	chr2	121649320	121728563	79243	26	190	224.4090909	13.87413451	0.775677575	0.014947026	+	254128	grey	0.11016575	FALSE	NIFK-AS1
Region_10648	chr2	121726945	121736911	9966	6	37	60.37878788	9.672745456	0.325180044	0.016200627	-	84365	grey	-0.229150888	FALSE	NIFK
Region_10649	chr2	121737103	121767853	30750	18	114	145.1212121	13.77010715	0.704329322	0.026789195	+	7247	grey	0.135330339	FALSE	TSN

Region_10650	chr2	121779133	121809962	30829	7	27	44.37878788	6.201154115	0.788476865	0.058921901	+	101927801	grey	-0.062293231	FALSE	LINC01823
Region_10651	chr2	121902501	122887691	985190	188	1435	1552.666667	47.62233908	0.885689299	0.01394658	+	105373595	grey	0.272320152	FALSE	LOC105373595
Region_10652	chr2	124025287	124921219	895932	85	686	757.030303	27.83631811	0.826055866	0.019301121	+	129684	grey	0.063790834	FALSE	CNTNAP5
Region_10653	chr2	125710969	125765842	54873	5	30	47.8030303	6.530919824	0.833009716	0.077175507	+	105373597	grey	-0.023999585	FALSE	LINC01889
Region_10654	chr2	126656133	126696667	40534	10	63	83.98484848	8.63177034	0.50637451	0.04124258	+	2995	grey	-0.045298644	FALSE	GYPC
Region_10655	chr2	127048027	127107288	59261	13	54	82.01515152	10.7867332	0.696298967	0.045568428	-	274	grey	0.052146053	FALSE	BIN1
Region_10656	chr2	127183832	127220313	36481	13	63	81.51515152	9.543813823	0.80707779	0.025166431	-	339761	grey	-0.116719243	FALSE	CYP27C1
Region_10657	chr2	127257290	127294166	36876	4	22	33.90909091	5.134280783	0.954823281	0.039263781	-	2071	grey	0.159755968	FALSE	ERCC3
Region_10658	chr2	127298668	127388465	89797	14	88	113.4090909	11.73420676	0.965260657	0.022911159	-	10746	grey	0.021882925	FALSE	MAP3K2
Region_10659	chr2	127388173	127406228	18055	4	15	23.95454545	4.602446902	0.939495153	0.050181152	+	100506922	grey	-0.009575907	FALSE	MAP3K2-DT
Region_10660	chr2	127418427	127429242	10815	5	22	36.63636364	6.115896975	0.767643643	0.040449622	+	5624	grey	-0.316597557	FALSE	PROC
Region_10661	chr2	127436207	127486618	50411	6	38	54.62121212	7.900857113	0.939847034	0.058719993	-	105373608	grey	0.625481459	FALSE	LOC105373608
Region_1066	chr2	12753568	12763772	102046	34	188	236.515151	18.9088847	0.94287197	0.01972380	+	4648	grey	0.1187039	FALSE	MYO7B

2		3	9				5			7						
Region_10663	chr2	127638381	127681786	43405	19	95	121.7878788	11.57519251	0.650894513	0.029248079	-	55679	grey	-0.182131476	FALSE	LIMS2
Region_10664	chr2	127701027	127811187	110160	32	234	269.2727273	17.61515559	0.738133193	0.012854078	-	55339	grey	0.044338962	FALSE	WDR33
Region_10665	chr2	127701497	127705242	3745	6	40	55.10606061	7.870350961	0.159270082	0.015977597	+	84826	grey	-0.01151557	FALSE	SFT2D3
Region_10666	chr2	127843553	127858155	14602	15	108	133.1363636	11.60753915	0.701244667	0.022312418	-	5433	grey	0.175443189	FALSE	POLR2D
Region_10667	chr2	127861630	127885967	24337	20	140	170.3636364	13.27071543	0.861541989	0.028668458	-	83607	grey	0.159273537	FALSE	AMMECR1L
Region_10668	chr2	127888829	127892732	3903	3	11	21.53030303	4.427952112	0.710027402	0.076696204	+	107985944	grey	0.110625607	FALSE	LOC107985944
Region_10669	chr2	127941217	128028120	86903	27	186	217.8636364	14.79385149	0.906494948	0.015514094	-	79595	grey	-0.082648823	FALSE	SAP130
Region_10670	chr2	128091200	128195677	104477	37	272	325.4848485	19.31618885	0.858288723	0.02072866	+	56886	grey	-0.1629394	FALSE	UGGT1
Region_10671	chr2	128236716	128318868	82152	29	198	232.3787879	15.3510362	0.830446751	0.018534347	-	9394	grey	0.135444031	FALSE	HS6ST1
Region_10672	chr2	129825126	129877664	52538	8	44	62.77272727	7.513670525	0.926939315	0.050610273	-	105373615	grey	0.277152799	FALSE	LINC02572
Region_10673	chr2	129990456	130051131	60675	8	36	49.43939394	6.850331352	0.711003225	0.083280334	-	440905	grey	0.117548742	FALSE	FAR2P1
Region_10674	chr2	130073535	130129222	55687	4	18	34.83333333	6.685422335	0.924482396	0.053616591	-	728378	grey	-0.084546664	FALSE	POTEF

Region_10675	chr2	130129621	130139417	9796	5	19	30.07575758	5.087875803	0.875626114	0.082246512	+	285103	grey	-0.034047113	FALSE	MED15P9
Region_10676	chr2	130151392	130182750	31358	8	58	73.8030303	7.789973234	0.71235375	0.026030455	-	55627	grey	0.047301853	FALSE	SMPD4
Region_10677	chr2	130181737	130190729	8992	5	32	52.39393939	9.486636411	0.934276575	0.035152524	+	80097	grey	0.103998056	FALSE	MZT2B
Region_10678	chr2	130342877	130347967	5090	7	52	73.3030303	8.118297845	0.966900741	0.022337029	+	92856	grey	-0.112913439	FALSE	IMP4
Region_10679	chr2	130356045	130375405	19360	10	55	79.78787879	12.07988175	0.603244918	0.036986727	+	26469	grey	-0.115023019	FALSE	PTPN18
Region_10680	chr2	130459455	130509707	50252	5	26	41.21212121	9.040958342	0.833992057	0.088561108	-	653269	grey	-0.087931948	FALSE	POTEI
Region_10681	chr2	130611440	130658037	46597	3	10	21.78787879	4.021156173	0.922979588	0.061929464	+	653781	grey	-0.044422373	FALSE	POTEJ
Region_10682	chr2	130755540	130768134	12594	5	17	31.65151515	5.196129993	0.417411504	0.05568393	+	205147	grey	0.045324431	FALSE	AMER3
Region_10683	chr2	130836914	131047263	210349	99	702	783.530303	35.18094751	0.758976624	0.01259706	+	50649	grey	0.010946813	FALSE	ARHGEF4
Region_10684	chr2	131034928	131047253	12325	18	106	145.9545455	16.81430876	0.653216809	0.037275619	+	113523639	grey	0.029002625	FALSE	SMIM39
Region_10685	chr2	131047876	131093460	45584	16	106	126.4545455	10.97154616	0.957629344	0.021306617	-	130074	grey	0.025592742	FALSE	FAM168B
Region_10686	chr2	131104847	131353709	248862	72	513	582.3181818	31.89606153	0.881227682	0.014524468	+	55041	grey	-0.008045608	FALSE	PLEKHB2
Region_1068	chr2	13120953	13126527	55742	4	13	25.5303030	5.21600996	0.92157887	0.05789984	+	445582	grey	0.159122302	FALSE	POTEE

7		6	8				3		4	2						
Region_10688	chr2	131279290	131309068	29778	13	72	91.93939394	9.138050268	0.802356163	0.041909977	+	440910	grey	-0.174211859	FALSE	LOC440910
Region_10689	chr2	131360492	131364168	3676	4	13	22.42424242	4.716780538	0.187061518	0.090742546	-	150786	grey	-0.163165169	FALSE	RAB6D
Region_10690	chr2	131442644	131444587	1943	4	11	33	7.62384922	0.905344544	0.063415288	-	401010	grey	-0.188569346	FALSE	NOC2LP2
Region_10691	chr2	131464900	131492743	27843	7	38	53.53030303	7.864662342	0.97137113	0.033898027	-	653784	grey	0.157821759	FALSE	MZT2A
Region_10692	chr2	131492813	131521573	28760	21	135	169.8181818	16.68892686	0.835482401	0.021050528	+	150776	grey	0.053059804	FALSE	SMPD4BP
Region_10693	chr2	131527675	131533666	5991	8	35	56.09090909	9.037915472	0.701409936	0.037162263	+	90557	grey	-0.166099798	FALSE	CCDC74A
Region_10694	chr2	131591752	131628147	36395	11	67	100.9393939	10.78728423	0.923130438	0.028804272	+	440915	grey	0.00460106	FALSE	POTEKP
Region_10695	chr2	131637025	131649615	12590	5	28	43.63636364	6.465616429	0.703903849	0.079146047	+	101927994	grey	-0.094739734	FALSE	LINC01087
Region_10696	chr2	132147591	132257969	110378	33	219	251.3636364	15.56488787	0.809103124	0.024631306	-	554226	grey	-0.090929364	FALSE	ANKRD30BL
Region_10697	chr2	132416805	132646582	229777	35	278	313.3181818	19.85962978	0.852187811	0.024171534	+	2863	grey	-0.124741499	FALSE	GPR39
Region_10698	chr2	132643286	132671579	28293	5	27	41.18181818	6.304843293	0.319910674	0.072944692	-	116372	grey	0.251551619	FALSE	LYPD1
Region_10699	chr2	132660526	132816896	156370	24	159	206.030303	17.12036097	0.777427089	0.029501266	+	105373623	grey	0.090466136	FALSE	

Region_10700	chr2	132671788	133568463	896675	123	1013	1087.515152	35.68677515	0.846144608	0.019956247	-	344148	grey	-0.042453658	FALSE	NCKAP5
Region_10701	chr2	133264968	133285445	20477	4	21	32.37878788	5.061970281	0.700275088	0.050672344	+	101928161	grey	-0.299167366	FALSE	NCKAP5-AS2
Region_10702	chr2	134119983	134454621	334638	47	344	412.2878788	22.58574442	0.805520582	0.016309717	+	4249	grey	-0.10573498	FALSE	MGAT5
Region_10703	chr2	134455759	134719000	263241	34	249	284.3030303	17.97003463	0.876127657	0.026112454	-	81615	grey	-0.117291566	FALSE	TMEM163
Region_10704	chr2	134718188	134725474	7286	3	10	23.18181818	5.702655497	0.419417361	0.086979088	+	105373629	grey	-0.183687692	FALSE	LOC105373629
Region_10705	chr2	134735464	134918712	183248	18	139	178.9242424	15.92415737	0.862370905	0.028617626	-	100129961	grey	-0.150886421	FALSE	CCNT2-AS1
Region_10706	chr2	134838616	134902034	63418	7	48	62.95454545	7.488312572	0.861610334	0.064804846	+	130013	grey	-0.164650557	FALSE	ACMSD
Region_10707	chr2	134918235	134959342	41107	7	38	50.92424242	6.970952051	0.671448272	0.039795554	+	905	grey	0.015877808	FALSE	CCNT2
Region_10708	chr2	134964485	135047468	82983	15	86	104.6818182	9.728877065	0.845208753	0.03769526	-	80122	grey	0.059928898	FALSE	MAP3K19
Region_10709	chr2	135052289	135176667	124378	11	82	99.21212121	8.518784947	0.805257077	0.014997652	+	22930	grey	-0.214165954	FALSE	RAB3GAP1
Region_10710	chr2	135136916	135531218	394302	69	482	558.7727273	30.64325523	0.963907759	0.009234407	-	84083	grey	0.132719743	FALSE	ZRANB3
Region_10711	chr2	135531455	135725270	193815	25	165	199.0151515	12.85719484	0.927298464	0.016553343	+	23518	grey	-0.01316453	FALSE	R3HDM1
Region_1071	chr2	13574173	13578505	43322	11	66	83.4242424	8.11467920	0.68943010	0.03377255	+	23190	grey	0.25924738	FALSE	UBXN4

2		4	6				2	8	7	3						
Region_10713	chr2	135787850	135837184	49334	12	77	98.18181818	9.900907637	0.809236467	0.033200547	-	3938	grey	-0.088967185	FALSE	LCT
Region_10714	chr2	135839626	135876443	36817	3	11	20.13636364	4.09083916	0.961855312	0.062603976	-	4175	grey	-0.191982949	FALSE	MCM6
Region_10715	chr2	135905881	135986100	80219	9	50	66.63636364	8.450100343	0.837351779	0.039115295	-	1615	grey	0.115189843	FALSE	DARS1
Region_10716	chr2	135985124	136022593	37469	6	38	52.68181818	7.367619574	0.509240134	0.063061806	+	101928243	grey	0.121473572	FALSE	DARS1-AS1
Region_10717	chr2	136114349	136119177	4828	7	44	61.72727273	7.449409795	0.133100581	0.017476696	-	7852	grey	-0.08886051	FALSE	CXCR4
Region_10718	chr2	136765545	137677718	912173	79	611	672.5454545	29.39187843	0.816273656	0.020824953	+	80731	grey	0.224381516	FALSE	THSD7B
Region_10719	chr2	137964020	138016364	52344	4	27	36.48484848	4.971433547	0.8552853	0.052954744	+	3176	grey	0.064507954	FALSE	HNMT
Region_10720	chr2	138418284	138501698	83414	9	54	71.8030303	9.262363375	0.899782548	0.034544	-	105373637	grey	-0.03748872	FALSE	SPOPL-DT
Region_10721	chr2	138501770	138573547	71777	3	10	18.36363636	4.345240946	0.640667956	0.055513995	+	339745	grey	-0.194488129	FALSE	SPOPL
Region_10722	chr2	138669157	138780390	111233	13	77	94.27272727	8.297442525	0.606116765	0.033684477	-	11249	grey	-0.262968895	FALSE	NXPH2
Region_10723	chr2	138917204	138975006	57802	3	13	27.31818182	5.011246094	0.841199502	0.102864514	+	105373640	grey	0.045778418	FALSE	LOC105373640
Region_10724	chr2	139323921	139379147	55226	3	14	22.93939394	4.600597914	0.805208316	0.10214278	+	105373643	grey	-0.093489424	FALSE	LOC105373643

Region_10725	chr2	140231423	142131016	1899593	129	980	1051.121212	41.34452439	0.813630376	0.020868144	-	53353	grey	0.302218439	FALSE	LRP1B
Region_10726	chr2	142877657	143055833	178176	19	138	161.5606061	12.383772	0.934537432	0.023228199	+	8942	grey	-0.145906465	FALSE	KYNU
Region_10727	chr2	143091362	143768352	676990	40	293	325.9090909	20.10107328	0.896614146	0.01554045	+	55843	grey	0.122055362	FALSE	ARHGAP15
Region_10728	chr2	143629133	143741294	112161	12	75	97.92424242	10.35569053	0.889142841	0.028568358	-	105373654	grey	0.081651677	FALSE	ARHGAP15-AS1
Region_10729	chr2	143938068	144332568	394500	29	203	240.969697	16.88237105	0.941970646	0.012879091	-	79712	grey	0.142469043	FALSE	GTDC1
Region_10730	chr2	144364364	144521057	156693	20	138	174.1060606	12.47659814	0.713369794	0.041499185	-	9839	grey	-0.056093295	FALSE	ZEB2
Region_10731	chr2	144521868	144579434	57566	8	48	63.89393939	7.77199863	0.816370771	0.032359388	+	101928455	grey	-0.344439316	FALSE	LINC01412
Region_10732	chr2	144666312	145182656	516344	36	281	322.0454545	21.47730954	0.899394729	0.01767329	+	401014	grey	-0.141458242	FALSE	TEX41
Region_10733	chr2	144667774	145262988	595214	42	324	378.3939394	22.96946387	0.887116144	0.017547597	+	100505498	grey	-0.176452433	FALSE	LOC100505498
Region_10734	chr2	144667985	144810041	142056	9	56	74.71212121	7.763356038	0.848665484	0.037307169	+	105373663	grey	0.033823065	FALSE	LOC105373663
Region_10735	chr2	146682289	146901938	219649	12	81	103.6666667	10.10610377	0.87182472	0.036895135	+	105373669	grey	0.167282243	FALSE	LINC01911
Region_10736	chr2	147844517	147930826	86309	3	18	26.95454545	4.846669969	0.637073105	0.037467866	+	92	grey	0.070136564	FALSE	ACVR2A
Region_1073	chr2	14793039	14802160	91208	5	30	45.1515151	6.68693887	0.95480959	0.05489902	-	5000	grey	-0.00284636	FALSE	ORC4

7		6	4				5	5	9	8				4		
Region_10738	chr2	148021011	148516971	495960	23	154	187.0909091	14.44323347	0.921967426	0.022808506	+	55777	grey	-0.095931438	FALSE	MBD5
Region_10739	chr2	148644440	148787569	143129	14	91	109.9242424	10.38684168	0.681327517	0.027519158	+	26122	grey	-0.154011839	FALSE	EPC2
Region_10740	chr2	148866470	148888823	22353	3	13	25.09090909	4.777277218	0.627536647	0.04181377	-	112267889	grey	0.039426282	FALSE	KIF5C-AS1
Region_10741	chr2	149038107	149215262	177155	19	130	158.8939394	11.19907797	0.895150295	0.024994915	+	130576	grey	0.154086957	FALSE	LYPD6B
Region_10742	chr2	149170561	149184570	14009	4	25	36.36363636	5.255433419	0.953702979	0.039935482	-	105373678	grey	0.144568918	FALSE	
Region_10743	chr2	149329985	149474138	144153	22	167	198.6515152	11.42937164	0.799658798	0.026172956	+	130574	grey	-0.132743955	FALSE	LYPD6
Region_10744	chr2	149587196	150047447	460251	24	192	221.7272727	14.99902095	0.790772563	0.028245674	+	101929231	grey	0.007668948	FALSE	MMADHC-DT
Region_10745	chr2	149745648	149859191	113543	4	28	41.46969697	6.516770547	0.855595719	0.057157423	-	150596	grey	-0.100150878	FALSE	LINC01931
Region_10746	chr2	150169474	150356460	186986	12	69	93.13636364	9.055192074	0.734728603	0.050280461	+	105373684	grey	-0.017766544	FALSE	LINC01818
Region_10747	chr2	150468195	150539011	70816	5	36	47.07575758	5.910383577	0.801909465	0.065971222	-	390	grey	-0.317739765	FALSE	RND3
Region_10748	chr2	150612381	150635818	23437	3	18	26.86363636	4.468303287	0.886866128	0.058357905	+	101929282	grey	0.189817337	FALSE	LINC02612
Region_10749	chr2	151001220	151048774	47554	6	44	60.24242424	6.930557989	0.823964509	0.065122804	-	105373686	grey	-0.089565265	FALSE	LOC105373686

Region_10750	chr2	151409883	151508013	98130	17	101	128.5	18.30615701	0.867805323	0.029637142	+	55183	grey	-0.252802277	FALSE	RIF1
Region_10751	chr2	151485336	151734487	249151	26	177	219.3636364	17.2730438	0.924716174	0.020614035	-	4703	grey	-0.143397127	FALSE	NEB
Region_10752	chr2	151789078	151842014	52936	5	27	42.98484848	7.449300275	0.573370149	0.028933173	-	26225	grey	0.09075197	FALSE	ARL5A
Region_10753	chr2	151802827	152099167	296340	39	286	333.3787879	17.54270846	0.831906209	0.021270614	-	785	grey	0.01959762	FALSE	CACNB4
Region_10754	chr2	152116801	152175763	58962	11	70	86.16666667	9.296125468	0.968164053	0.015518952	-	10254	grey	0.186834259	FALSE	STAM2
Region_10755	chr2	152176020	152225211	49191	14	93	114.6212121	10.59573957	0.860698969	0.041885478	+	105373689	grey	-0.084761272	FALSE	LOC105373689
Region_10756	chr2	152335174	152649826	314652	46	352	392.1363636	18.04429282	0.89242336	0.022195142	+	114793	grey	0.146504091	FALSE	FMNL2
Region_10757	chr2	152651593	152718012	66419	11	63	76.60606061	7.367034239	0.89083326	0.025255992	-	55660	grey	0.220200738	FALSE	PRPF40A
Region_10758	chr2	152717647	152762396	44749	5	27	38.98484848	5.231538748	0.539883114	0.047495066	+	151188	grey	-0.287507517	FALSE	ARL6IP6
Region_10759	chr2	153477338	153478762	1424	3	24	35.12121212	6.417560408	0.0229208	0.032375313	-	56475	grey	-0.035645185	FALSE	RPRM
Region_10760	chr2	153871922	154453979	582057	50	366	401.4242424	20.94686362	0.824727194	0.026634948	+	114805	grey	-0.008874862	FALSE	GALNT13
Region_10761	chr2	154435853	154457438	21585	3	16	26.27272727	5.10970556	0.881428821	0.069762279	-	100144595	grey	-0.190515619	FALSE	GALNT13-AS1
Region_1076	chr2	15469785	15485835	160499	10	63	84.0151515	8.84306320	0.87657074	0.04374324	+	3760	grey	0.067182718	FALSE	KCNJ3

2		5	4				2	8	7	5						
Region_10763	chr2	155525424	155664668	139244	5	32	51.33333333	7.694820103	0.740403439	0.06582267	+	107985953	grey	-0.336567035	FALSE	LOC107985953
Region_10764	chr2	156011530	156254950	243420	17	103	134.9090909	11.87074915	0.818585867	0.037134178	-	101929378	grey	-0.26579149	FALSE	LINC01876
Region_10765	chr2	156324437	156342348	17911	8	53	69.68181818	10.46923565	0.202819791	0.064920805	-	4929	grey	0.163063358	FALSE	NR4A2
Region_10766	chr2	156435290	156613735	178445	13	92	115.8484848	10.0188168	0.727502171	0.02152269	+	2820	grey	0.105733294	FALSE	GPD2
Region_10767	chr2	156803339	156891160	87821	9	63	78.59090909	8.632634454	0.890156849	0.045179492	+	107985955	grey	0.054756756	FALSE	
Region_10768	chr2	157257705	157318491	60786	6	28	42.37878788	7.221261666	0.901508438	0.052558922	+	11227	grey	0.006275338	FALSE	GALNT5
Region_10769	chr2	157414619	157488960	74341	7	40	64.59090909	8.199394486	0.936673632	0.041866152	-	9595	grey	-0.025361095	FALSE	CYTIP
Region_10770	chr2	157430871	157488961	58090	5	30	45.81818182	6.367970552	0.93522244	0.048821406	-	105373712	grey	-0.097845359	FALSE	LOC105373712
Region_10771	chr2	157526767	157628864	102097	12	69	91.42424242	8.837090726	0.748091998	0.034620747	-	130399	grey	0.036716604	FALSE	ACVR1C
Region_10772	chr2	157736251	157876330	140079	13	89	114.6212121	10.21878384	0.829758414	0.039746433	-	90	grey	0.111481715	FALSE	ACVR1
Region_10773	chr2	157876702	158136154	259452	18	115	159.6969697	12.1819156	0.739014812	0.030072304	+	151531	grey	-0.033929049	FALSE	UPP2
Region_10774	chr2	158166650	158236169	69519	4	24	37.04545455	5.965108338	0.938722341	0.044893118	+	554201	grey	0.083725232	FALSE	CCDC148-AS1

Region_10775	chr2	158171073	158456753	285680	16	115	144.3030303	11.54785321	0.904142684	0.032926366	-	130940	grey	0.072625451	FALSE	CCDC148
Region_10776	chr2	158456952	158682879	225927	26	171	204.8939394	13.3348921	0.818547464	0.027574659	+	8502	grey	-0.546334795	FALSE	PKP4
Region_10777	chr2	158658337	158735002	76665	14	93	120.9545455	10.92975025	0.946133376	0.02388791	-	100129029	grey	-0.04441008	FALSE	PKP4-AS1
Region_10778	chr2	158795317	158862781	67464	9	58	79.43939394	8.852863575	0.954925539	0.026057901	+	92196	grey	-0.083057064	FALSE	DAPL1
Region_10779	chr2	158968640	159232659	264019	38	273	317.8484848	20.69503279	0.874262504	0.019953878	+	85461	grey	-0.108564014	FALSE	TANC1
Region_10780	chr2	159235798	159286703	50905	4	14	25.25757576	5.972528407	0.256181407	0.025436309	-	151525	grey	-0.090300039	FALSE	WDSUB1
Region_10781	chr2	159318979	159712444	393465	45	336	370.1515152	18.23457614	0.857671421	0.016500036	-	29994	grey	0.093845427	FALSE	BAZ2B
Region_10782	chr2	159712457	159771027	58570	12	82	104.0151515	10.16402771	0.68138352	0.036151372	+	64844	grey	-0.224775571	FALSE	MARCHF7
Region_10783	chr2	159768628	159798255	29627	5	28	37.65151515	6.491396494	0.788031478	0.044596197	-	9936	grey	0.210995974	FALSE	CD302
Region_10784	chr2	159771851	159904710	132859	21	136	171.2121212	14.91980114	0.863385079	0.02236304	-	100526664	grey	0.179811846	FALSE	LY75-CD302
Region_10785	chr2	159803355	159904756	101401	16	104	133.5606061	12.70024686	0.887092421	0.02797008	-	4065	grey	0.086927056	FALSE	LY75
Region_10786	chr2	159932006	160062615	130609	26	181	212.7878788	15.37481973	0.90733718	0.029829338	-	22925	grey	-0.198186982	FALSE	PLA2R1
Region_1078	chr2	16009966	16027188	172221	21	150	179.060606	13.4254394	0.91707040	0.02495886	-	3694	grey	0.068462337	FALSE	ITGB6

7		7	8				1	4	1							
Region_10788	chr2	160257716	160271880	14164	3	13	25.46969697	5.421381308	0.955212712	0.04670483	-	100505984	grey	0.194657946	FALSE	LINC02478
Region_10789	chr2	160272151	160493807	221656	25	203	235.2424242	15.33729553	0.71882882	0.026665279	-	5937	grey	-0.127402252	FALSE	RBMS1
Region_10790	chr2	161096231	161160224	63993	6	30	44.43939394	6.547175052	0.938645768	0.05077875	-	105373719	grey	0.048351064	FALSE	TANK-AS1
Region_10791	chr2	161136908	161236230	99322	8	64	83.72727273	8.741669361	0.701930466	0.026714364	+	10010	grey	0.18451735	FALSE	TANK
Region_15070	chr5	140807037	141012347	205310	110	831	1041.80303	80.66115635	0.532091292	0.028094665	+	56144	blue	0.94726998	FALSE	PCDHA4
Region_10793	chr2	161308425	161411717	103292	11	55	76.6969697	10.6655011	0.943165314	0.028350045	+	10213	grey	-0.062692035	FALSE	PSMD14
Region_10794	chr2	161416297	161425870	9573	6	37	48.53030303	6.402450725	0.097303235	0.0486478	+	10716	grey	0.15729003	FALSE	TBR1
Region_10795	chr2	161424332	161985282	560950	36	258	302.1212121	15.27342101	0.758375172	0.023101421	+	57282	grey	-0.66565136	FALSE	SLC4A10
Region_10796	chr2	161992245	162074394	82149	9	57	79.28787879	8.66250124	0.815588994	0.043213247	-	1803	grey	-0.25172442	FALSE	DPP4
Region_10797	chr2	162170684	162243456	72772	8	58	72.53030303	8.661532458	0.874918818	0.048554416	-	2191	grey	0.077017589	FALSE	FAP
Region_10798	chr2	162267074	162318684	51610	7	40	65.10606061	8.146974498	0.837023688	0.040942924	-	64135	grey	-0.335649374	FALSE	IFIH1
Region_10799	chr2	162318840	162371595	52755	8	56	76.18181818	9.830735305	0.244018681	0.013974324	+	25801	grey	-0.103017005	FALSE	GCA

Region_10800	chr2	162371407	162838767	467360	22	161	186.7878788	12.6131984	0.868876742	0.030452073	-	90134	grey	-0.587045826	FALSE	KCNH7
Region_10801	chr2	163593396	163736012	142616	9	57	78.59090909	9.139899465	0.814023472	0.05240108	-	55137	grey	0.03930746	FALSE	FIGN
Region_10802	chr2	164492417	164621482	129065	14	93	116.2575758	10.89574456	0.738862317	0.035549547	-	2888	grey	0.26566461	FALSE	GRB14
Region_10803	chr2	164653624	164843679	190055	18	125	149.1818182	12.32871456	0.649459854	0.01971996	-	22837	grey	-0.161711479	FALSE	COBLL1
Region_10804	chr2	164840661	165094117	253456	20	150	184.5909091	13.19437791	0.576281174	0.023693181	+	101929633	grey	0.084177353	FALSE	LOC101929633
Region_10805	chr2	164894354	164955525	61171	3	19	32.07575758	5.744055405	0.485914421	0.094313792	-	151258	grey	0.33407866	FALSE	SLC38A11
Region_10806	chr2	165087526	165204050	116524	7	42	56.86363636	8.236665426	0.888938184	0.044308433	-	6328	grey	-0.112646837	FALSE	SCN3A
Region_10807	chr2	165239372	165392310	152938	13	97	125.9848485	12.24555483	0.83975863	0.033180365	+	6326	grey	-0.000676913	FALSE	SCN2A
Region_10808	chr2	165469647	165689407	219760	28	184	218.6212121	15.33900523	0.91999485	0.025705623	+	80034	grey	0.317303086	FALSE	CSRNP3
Region_10809	chr2	165747588	165794659	47071	5	25	37.66666667	6.526474684	0.766535924	0.032880746	-	2591	grey	0.074844284	FALSE	GALNT3
Region_10810	chr2	165794857	165846091	51234	5	26	36.65151515	5.382285543	0.95544231	0.041515934	-	105373730	grey	0.056750139	FALSE	
Region_10811	chr2	165857478	165953851	96373	4	21	35.22727273	5.721203355	0.774599019	0.052422672	-	79809	grey	-0.425093395	FALSE	TTC21B
Region_1081	chr2	16595718	16617154	214359	11	75	93.0606060	9.66497051	0.88776619	0.04409877	+	10272405	grey	0.502833765	FALSE	LOC102724058

2		8	7				6	3	7	2		8				
Region_10813	chr2	165957401	166390771	433370	23	158	181.3787879	12.38580472	0.823565142	0.032618421	+	101929680	grey	0.300105865	FALSE	SCN1A-AS1
Region_10814	chr2	165984641	166149214	164573	10	71	88.66666667	8.774672176	0.892143001	0.045185858	-	6323	grey	0.488388033	FALSE	SCN1A
Region_10815	chr2	166195185	166376001	180816	10	59	74.78787879	7.523328133	0.808304867	0.038086366	-	6335	grey	0.018193359	FALSE	SCN9A
Region_10816	chr2	166403573	166494326	90753	3	18	30.92424242	5.95446983	0.865100898	0.062510287	-	6332	grey	0.025576208	FALSE	SCN7A
Region_10817	chr2	166477463	166611482	134019	16	98	129.6666667	12.21894284	0.738748471	0.05325085	-	107985958	grey	-0.119039235	FALSE	LOC107985958
Region_10818	chr2	166888480	167259753	371273	28	191	222.8636364	15.96331021	0.913480712	0.024817665	+	129446	grey	-0.057389759	FALSE	XIRP2
Region_10819	chr2	167293001	167874045	581044	46	340	383.7727273	20.07738525	0.88627239	0.027010176	+	8708	grey	0.094223415	FALSE	B3GALT1
Region_10820	chr2	167814757	167941180	126423	8	43	62.18181818	7.509807108	0.930428568	0.034250587	-	105616981	grey	0.034639937	FALSE	B3GALT1-AS1
Region_10821	chr2	167954020	168247595	293575	25	179	208.4545455	13.33379953	0.753975037	0.021085593	-	27347	grey	-0.097734156	FALSE	STK39
Region_10822	chr2	168456249	168775134	318885	29	211	252.7424242	17.04050433	0.80475021	0.016668396	+	253782	grey	-0.170385106	FALSE	CERS6
Region_10823	chr2	169064789	169096167	31378	3	19	30.36363636	5.151956652	0.94251113	0.044733097	+	10170	grey	0.182949897	FALSE	DHRS9
Region_10824	chr2	169127109	169362534	235425	26	160	199.5	16.48425823	0.880866934	0.016770144	-	4036	grey	0.092134797	FALSE	LRP2

Region_10825	chr2	169479480	169525922	46442	8	33	50.65151515	7.076488534	0.980967465	0.021492053	+	129880	grey	-0.369182521	FALSE	BBS5
Region_10826	chr2	169528508	169573875	45367	10	52	73.12121212	7.886727217	0.960451185	0.023129577	-	79675	grey	-0.228054078	FALSE	FASTKD1
Region_10827	chr2	169584342	169641406	57064	7	31	45.93939394	7.425276556	0.818143367	0.032140972	+	9360	grey	-0.054603981	FALSE	PPIG
Region_10828	chr2	169694454	169701708	7254	4	23	35.86363636	5.785622402	0.545509282	0.047443661	+	493911	grey	0.094477516	FALSE	PHOSPHO2
Region_10829	chr2	169694488	169776989	82501	17	117	147.1060606	11.22240724	0.713606418	0.0335873	+	100526832	grey	0.249706727	FALSE	PHOSPHO2-KLHL23
Region_10830	chr2	169733832	169773385	39553	7	42	57.37878788	7.013756047	0.741291618	0.04364864	+	151230	grey	0.181891524	FALSE	KLHL23
Region_10831	chr2	169827454	170084131	256677	29	190	217.0757576	14.29503476	0.968941155	0.01806259	+	130507	grey	0.048778173	FALSE	UBR3
Region_10832	chr2	170178145	170655171	477026	62	446	493.2727273	22.18977273	0.891709194	0.016494613	+	140469	grey	0.299248117	FALSE	MYO3B
Region_10833	chr2	170332085	170408564	76479	5	16	25.74242424	5.982823121	0.885995909	0.074628812	-	101929753	grey	0.24021024	FALSE	MYO3B-AS1
Region_10834	chr2	170640374	170695374	55000	26	179	205.530303	13.60115345	0.926784935	0.020438194	-	100130256	grey	-0.0363426	FALSE	LOC100130256
Region_10835	chr2	170700368	170813670	113302	19	117	146.3787879	11.73917197	0.445465438	0.020869699	-	101926913	grey	0.081189744	FALSE	ERICH2-DT
Region_10836	chr2	170712451	170714567	2116	4	17	29.75757576	5.171104944	0.064368323	0.043938594	-	440925	grey	-0.038359414	FALSE	LINC01124
Region_1083	chr2	17076687	17079897	32099	3	16	25.8181818	4.80034963	0.52646403	0.08139159	+	285141	grey	-0.06281899	FALSE	ERICH2

7		8	7				2	8	3					6		
Region_10838	chr2	170813213	170861151	47938	15	86	117.3484848	10.29935386	0.400463603	0.021556922	+	2571	grey	-0.270649443	FALSE	GAD1
Region_10839	chr2	170928464	170967130	38666	5	33	45.10606061	5.833929373	0.876200015	0.041209308	+	26003	grey	-0.378264015	FALSE	GORASP2
Region_10840	chr2	170990823	171231314	240491	28	197	225.3787879	13.31253331	0.882496157	0.018606713	-	9874	grey	-0.111479682	FALSE	TLK1
Region_10841	chr2	171315746	171434802	119056	15	100	123.969697	11.76954309	0.744561557	0.039957923	-	79828	grey	0.007638883	FALSE	METTL8
Region_10842	chr2	171434217	171485052	50835	5	23	34.25757576	5.063627785	0.748083268	0.059620222	+	80067	grey	-0.394415464	FALSE	DCAF17
Region_10843	chr2	171522247	171558129	35882	11	52	71.92424242	8.289586806	0.652045597	0.034078326	+	79901	grey	0.100364543	FALSE	CYBRD1
Region_10844	chr2	171687409	171750158	62749	10	45	70.13636364	8.73522729	0.795024028	0.052876261	+	1781	grey	0.284863057	FALSE	DYNC1I2
Region_10845	chr2	171783405	171999859	216454	27	174	203.7121212	12.43910107	0.856661288	0.020752489	-	8604	grey	-0.066152301	FALSE	SLC25A12
Region_10846	chr2	171922448	171983686	61238	7	48	72.60606061	8.673352272	0.667986741	0.030379515	+	8520	grey	-0.177592192	FALSE	HAT1
Region_10847	chr2	171999943	172082430	82487	17	97	125.3787879	10.58266384	0.921293181	0.025491422	+	254042	grey	0.028919074	FALSE	METAP1D
Region_10848	chr2	172103006	172109982	6976	7	43	58.74242424	7.84525453	0.113736323	0.050201585	+	104326193	grey	0.13906759	FALSE	DLX2-DT
Region_10849	chr2	172137345	172370401	233056	25	166	208.969697	17.00494923	0.796944155	0.030128441	+	107985960	grey	0.2186543	FALSE	LOC107985960

Region_10850	chr2	172427354	172506459	79105	9	50	79.18181818	10.1586024	0.646557764	0.023071834	+	3655	grey	-0.028767586	FALSE	ITGA6
Region_10851	chr2	172555373	172608669	53296	11	83	102.7575758	10.97838521	0.256004032	0.013256513	+	5163	grey	-0.287403752	FALSE	PDK1
Region_10852	chr2	172677148	172736206	59058	5	24	34.10606061	5.117477708	0.74278624	0.073020896	-	91149	grey	0.033014685	FALSE	RAPGEF4-AS1
Region_10853	chr2	172735274	173052893	317619	64	506	567.8333333	26.26233532	0.894040403	0.016038511	+	11069	grey	0.129448968	FALSE	RAPGEF4
Region_10854	chr2	173075435	173268015	192580	38	265	298.9242424	17.20761067	0.907613376	0.015673852	+	51776	grey	0.010454096	FALSE	MAP3K20
Region_10855	chr2	173166446	173282036	115590	22	162	189.3030303	13.09605895	0.91415067	0.027076602	-	339751	grey	0.13715623	FALSE	MAP3K20-AS1
Region_10856	chr2	173354820	173368997	14177	4	24	37.31818182	6.048926391	0.524303065	0.051058129	+	83879	grey	-0.332387151	FALSE	CDCA7
Region_10857	chr2	173880850	173965373	84523	14	84	105.1666667	10.04234624	0.826730643	0.032075295	-	6670	grey	0.173065951	FALSE	SP3
Region_10858	chr2	174072447	174248599	176152	20	142	167.6212121	11.53825097	0.892909304	0.026543005	-	29789	grey	-0.005141874	FALSE	OLA1
Region_10859	chr2	174334954	174338500	3546	4	17	26.95454545	4.727763557	0.015687217	0.024478049	+	100131390	grey	-0.198160247	FALSE	SP9
Region_10860	chr2	174395730	174429575	33845	7	43	59.45454545	7.187985314	0.838388881	0.022368045	+	79634	grey	-0.054689821	FALSE	SCRN3
Region_10861	chr2	174431571	174487094	55523	9	50	66.21212121	8.048918038	0.960356859	0.03023206	-	151556	grey	0.051015448	FALSE	GPR155
Region_1086	chr2	17455957	17468291	123344	14	83	100.136363	9.48827027	0.77932642	0.04386467	-	7456	grey	0.249546812	FALSE	WIPF1

2		2	6				6	1	6	5						
Region_10863	chr2	174747592	174787935	40343	9	57	76.18181818	9.687278082	0.817376513	0.038899205	-	1134	grey	0.298040197	FALSE	CHRNA1
Region_10864	chr2	174798809	175005381	206572	33	241	274.3181818	15.64919176	0.87467495	0.018861174	-	1123	grey	-0.189077796	FALSE	CHN1
Region_10865	chr2	175072250	175168382	96132	14	76	98.28787879	10.14474499	0.901697847	0.019733399	-	1386	grey	-0.297246882	FALSE	ATF2
Region_10866	chr2	175257362	175463180	205818	25	164	202.6818182	14.56038788	0.915376313	0.021522671	+	105373752	grey	-0.028217958	FALSE	LOC105373752
Region_10867	chr2	175923882	176002839	78957	5	27	37.59090909	5.146999941	0.785645382	0.01922366	-	80856	grey	0.055687715	FALSE	LNPK
Region_10868	chr2	176077472	176083962	6490	3	11	24.40909091	4.634244682	0.018839732	0.027930905	-	344191	grey	0.066072881	FALSE	EVX2
Region_10869	chr2	176092721	176095944	3223	3	12	22.5	5.429973792	0.095541934	0.070670166	+	3239	grey	0.197394916	FALSE	HOXD13
Region_10870	chr2	176121611	176137098	15487	13	70	88.86363636	11.72490625	0.098486629	0.0502577	-	100506783	grey	-0.131333903	FALSE	HOXD-AS2
Region_10871	chr2	176122719	176124937	2218	3	11	19.75757576	4.716780538	0.04130689	0.055046952	+	3235	grey	-0.154542588	FALSE	HOXD9
Region_10872	chr2	176136957	176173102	36145	9	54	73	9.087607792	0.055545151	0.027885907	+	3232	grey	-0.089296591	FALSE	HOXD3
Region_10873	chr2	176164051	176188958	24907	10	63	80.74242424	8.198143516	0.358771557	0.024452131	-	401022	grey	0.179178626	FALSE	HAGLR
Region_10874	chr2	176188668	176190907	2239	4	13	24.31818182	5.384190792	0.038362886	0.050493532	+	3231	grey	-0.126090932	FALSE	HOXD1

Region_10875	chr2	176495255	176819725	324470	27	181	215.6818182	13.2724806	0.789151627	0.024502714	+	102724224	grey	0.246455995	FALSE	LINC01117
Region_10876	chr2	176611437	176637775	26338	4	12	20.62121212	4.898241919	0.236150739	0.043322843	-	105373754	grey	-0.114368111	FALSE	LOC105373754
Region_10877	chr2	176629572	176637931	8359	4	12	20.62121212	4.898241919	0.236150739	0.043322843	-	375295	grey	-0.114368111	FALSE	LINC01116
Region_10878	chr2	176723614	176819310	95696	7	37	53	7.183313998	0.899275748	0.046153027	-	101929963	grey	0.153922749	FALSE	LOC101929963
Region_10879	chr2	176989418	177148864	159446	28	186	226.2424242	15.38536828	0.899476457	0.026308711	-	107985965	grey	-0.189556646	FALSE	LOC107985965
Region_10880	chr2	176990842	177164656	173814	30	201	237.6666667	15.15797158	0.902952922	0.026979813	-	105373760	grey	-0.247428551	FALSE	LOC105373760
Region_10881	chr2	177218667	177392756	174089	17	110	136.4545455	11.5667718	0.737551373	0.025739246	-	4780	grey	0.111263596	FALSE	NFE2L2
Region_10882	chr2	177233250	177392699	159449	16	101	127.0454545	11.6228314	0.72563569	0.026631303	-	100130691	grey	0.112740909	FALSE	LOC100130691
Region_10883	chr2	177392746	177567024	174278	21	144	171.8787879	13.17049751	0.832572091	0.016755065	+	8540	grey	-0.076001942	FALSE	AGPS
Region_10884	chr2	177623244	178108339	485095	49	374	411.5454545	18.37739566	0.862993616	0.025399543	-	50940	grey	0.158351627	FALSE	PDE11A
Region_10885	chr2	177653419	177723289	69870	5	32	43.53030303	6.003126613	0.920430969	0.052986125	+	105373764	grey	0.061745724	FALSE	PDE11A-AS1
Region_10886	chr2	178112424	178139011	26587	6	38	53.31818182	6.661390686	0.949884397	0.043179675	+	129831	grey	0.146729722	FALSE	RBM45
Region_1088	chr2	17819448	17840289	208412	30	219	241.681818	10.9039993	0.90141299	0.02914617	+	114880	grey	-0.05525208	FALSE	OSBPL6

7		1	3				2	9	5	2				2		
Region_1088<br>8	chr2	17841363<br>5	17844024<br>3	26608	3	14	24.8181818<br>2	4.89555242<br>7	0.89065468<br>6	0.07252373<br>7	+	10192702<br>7	grey	0.038689882	FALSE	CHROMR
Region_1088<br>9	chr2	17845134<br>6	17846210<br>2	10756	3	20	31.1969697	5.30665676<br>9	0.62579129<br>4	0.04708023<br>6	+	494513	grey	-0.09955884<br>2	FALSE	PJVK
Region_1089<br>0	chr2	17846366<br>4	17847860<br>0	14936	3	14	27.6666666<br>7	5.56177429<br>7	0.65256863<br>4	0.02668652<br>2	-	51661	grey	0.08700423	FALSE	FKBP7
Region_1089<br>1	chr2	17848045<br>7	17851646<br>3	36006	7	40	52.9393939<br>4	6.17657806<br>2	0.95080297<br>8	0.04620438<br>1	+	65977	grey	0.262713191	FALSE	PLEKHA3
Region_1089<br>2	chr2	17852118<br>3	17877991<br>3	258730	35	292	333.196969<br>7	17.0801006<br>1	0.87189172	0.01701460<br>9	+	10050686<br>6	grey	-0.15934921<br>5	FALSE	TTN-AS1
Region_1089<br>3	chr2	17852322<br>1	17877996<br>3	256742	34	285	327.090909<br>1	16.9694089<br>2	0.86909657	0.01771715<br>5	+	10192705<br>5	grey	-0.18818639<br>4	FALSE	LOC101927055
Region_1089<br>4	chr2	17852598<br>9	17880742<br>3	281434	36	302	341.439393<br>9	18.3647897<br>1	0.87074220<br>6	0.01726108	-	7273	grey	-0.22792034<br>1	FALSE	TTN
Region_1089<br>5	chr2	17880228<br>7	17905013<br>7	247850	27	188	234.727272<br>7	15.8808500<br>6	0.88625619<br>1	0.02568377<br>5	-	285025	grey	0.119590135	FALSE	CCDC141
Region_1089<br>6	chr2	17882834<br>9	17900780<br>7	179458	18	99	139.742424<br>2	12.4533722<br>4	0.87325946<br>6	0.03353053<br>8	+	10537376<br>6	grey	0.130855706	FALSE	LOC105373766
Region_1089<br>7	chr2	17910167<br>8	17926483<br>2	163154	17	86	120.242424<br>2	13.8208990<br>1	0.90520866<br>9	0.02172153<br>8	-	91404	grey	0.134886577	FALSE	SESTD1
Region_1089<br>8	chr2	17944198<br>2	17986161<br>2	419630	38	268	306.484848<br>5	17.6680872	0.80801876<br>9	0.03052190<br>3	-	151126	grey	0.04637734	FALSE	ZNF385B
Region_1089<br>9	chr2	17994487<br>6	18000729<br>7	62421	3	17	29.3636363<br>6	5.33388691<br>8	0.96124520<br>2	0.03472081<br>9	-	57703	grey	0.044118188	FALSE	CWC22

Region_10900	chr2	180692104	180916939	224835	19	145	175.1969697	14.3797076	0.818284271	0.03172087	+	101669767	grey	0.189199281	FALSE	SCHLAP1
Region_10901	chr2	180967248	181076478	109230	6	19	34.89393939	7.300429467	0.490983782	0.038761915	+	10477	grey	-0.019883927	FALSE	UBE2E3
Region_10902	chr2	181101932	181409321	307389	10	63	82.3030303	8.864743819	0.870096147	0.038151549	+	101927156	grey	0.030201256	FALSE	LINC01934
Region_10903	chr2	181457202	181538940	81738	7	39	54.62121212	8.020675497	0.926108948	0.033755654	+	3676	grey	-0.123039965	FALSE	ITGA4
Region_10904	chr2	181535041	181680665	145624	13	97	117.2878788	11.37171275	0.751288071	0.022223912	-	375298	grey	-0.061122915	FALSE	CERKL
Region_10905	chr2	181891730	181930738	39008	6	35	51.39393939	7.528160032	0.545554898	0.040954574	+	6744	grey	0.014743862	FALSE	ITPRID2
Region_10906	chr2	181954241	182131398	177157	11	70	91.28787879	11.02551607	0.898543765	0.037135497	+	151242	grey	0.069631729	FALSE	PPP1R1C
Region_10907	chr2	182139968	182523359	383391	36	266	299.9090909	17.34334479	0.79763424	0.025388178	-	5136	grey	0.140390859	FALSE	PDE1A
Region_10908	chr2	182716255	182794464	78209	4	19	31.03030303	6.558540403	0.94815551	0.065686303	+	54431	grey	-0.133387714	FALSE	DNAJC10
Region_10909	chr2	182833275	182866637	33362	4	26	40.59090909	6.600646292	0.919525237	0.045493366	-	2487	grey	0.259836975	FALSE	FRZB
Region_10910	chr2	182909115	183038858	129743	15	89	121.3939394	11.9162188	0.812029756	0.021744216	-	10787	grey	-0.012087546	FALSE	NCKAP1
Region_10911	chr2	183117513	183161680	44167	4	23	37.92424242	6.753484812	0.477342481	0.046758297	+	129401	grey	0.443204004	FALSE	NUP35
Region_1091	chr2	18404946	18418757	138106	8	52	69.4848484	8.16039671	0.89279617	0.03925473	-	10537377	grey	-0.11066927	FALSE	

2		7	3				8	8	9	4		5		5		
Region_10913	chr2	184204372	184382869	178497	10	61	80.62121212	8.724439857	0.89920017	0.036170705	+	107985968	grey	0.02925447	FALSE	LOC107985968
Region_10914	chr2	184593577	184599008	5431	5	19	28.28787879	5.249952825	0.00854791	0.016404449	-	105373780	grey	0.324103065	FALSE	LOC105373780
Region_10915	chr2	184598529	184939492	340963	14	91	119.1969697	10.00802708	0.755279244	0.030546616	+	91752	grey	-0.234037961	FALSE	ZNF804A
Region_10916	chr2	185738804	185833290	94486	5	35	49.33333333	6.402723779	0.858959586	0.052979029	+	401024	grey	0.325689298	FALSE	FSIP2
Region_10917	chr2	186032884	186486005	453121	27	199	232.4545455	15.12937216	0.910980364	0.01893548	-	101927217	grey	-0.026387229	FALSE	LINC01473
Region_10918	chr2	186590010	186680901	90891	9	59	78.06060606	8.414051961	0.868059101	0.027500679	+	3685	grey	-0.097267993	FALSE	ITGAV
Region_10919	chr2	186694060	186765959	71899	7	35	52.5	7.96772335	0.761852421	0.062464034	+	165215	grey	0.31895534	FALSE	FAM171B
Region_10920	chr2	187003220	187556288	553068	28	195	234.1515152	17.62885958	0.811543897	0.032684956	+	105373786	grey	-0.043211218	FALSE	CALCRL-AS1
Region_10921	chr2	187341964	187448460	106496	5	34	45	6.206696136	0.837232934	0.070703781	-	10203	grey	-0.115735851	FALSE	CALCRL
Region_10922	chr2	187464230	187565760	101530	9	55	78.22727273	8.30161329	0.859324795	0.040246535	-	7035	grey	-0.310101042	FALSE	TFPI
Region_10923	chr2	187712816	188384313	671497	44	316	368.7878788	22.04227767	0.823752833	0.026808679	-	104355152	grey	0.25004979	FALSE	LINC01090
Region_10924	chr2	188291669	188595931	304262	33	233	270.6363636	17.60212956	0.838006621	0.025497847	+	51454	grey	-0.137714423	FALSE	GULP1

Region_10925	chr2	188734155	188790104	55949	4	10	25.6969697	5.256409119	0.884927496	0.088509602	-	116093	grey	-0.058422873	FALSE	DIRC1
Region_10926	chr2	188974373	189012746	38373	5	23	42.04545455	6.889081257	0.910706952	0.0443776	+	1281	grey	-0.17465394	FALSE	COL3A1
Region_10927	chr2	189031898	189225312	193414	12	81	102.469697	10.93648755	0.819982304	0.037377269	-	1290	grey	-0.049288141	FALSE	COL5A2
Region_10928	chr2	189441446	189475552	34106	4	17	28.31818182	5.539117094	0.810097867	0.054736185	+	84128	grey	-0.016552793	FALSE	WDR75
Region_10929	chr2	189674290	189761193	86903	8	49	65.92424242	7.520864529	0.770482903	0.033449228	+	150709	grey	-0.183426574	FALSE	ANKAR
Region_10930	chr2	189770267	189784364	14097	4	22	34.36363636	5.650422349	0.3858815	0.061689779	-	94101	grey	0.266270521	FALSE	ORMDL1
Region_10931	chr2	189784085	189877629	93544	16	112	148.0151515	14.01152539	0.837729743	0.025874014	+	5378	grey	0.090377865	FALSE	PMS1
Region_10932	chr2	189879609	190203484	323875	16	105	126.2878788	9.880462698	0.950153599	0.021229457	+	84281	grey	-0.062931857	FALSE	C2orf88
Region_10933	chr2	190189735	190344193	154458	13	74	99.06060606	10.53620686	0.730786258	0.032218666	-	26275	grey	-0.066710845	FALSE	HIBCH
Region_10934	chr2	190343570	190371665	28095	4	18	29.93939394	5.161629999	0.732301174	0.042050552	+	3628	grey	-0.201216404	FALSE	INPP1
Region_10935	chr2	190408355	190509205	100850	9	53	76.13636364	9.46391749	0.784220653	0.064370709	+	54842	grey	-0.195497717	FALSE	MFSD6
Region_10936	chr2	190504338	190534722	30384	4	19	28.09090909	4.491795162	0.617359293	0.06861368	-	100131211	grey	0.1048904	FALSE	NEMP2
Region_1093	chr2	19053485	19056977	34921	6	37	56.3030303	8.05360826	0.91685174	0.04561806	+	10537674	grey	0.071030084	FALSE	NEMP2-DT

7		5	6					3	7	3		8				
Region_1093 8	chr2	19064674 6	19069276 6	46020	11	42	62.5454545 5	10.5745439 2	0.26380957 9	0.01971929 9	+	4664	grey	-0.04043186 3	FALSE	NAB1
Region_1093 9	chr2	19088082 1	19096555 2	84731	5	40	51.6818181 8	6.05401098	0.95680970 9	0.02960592 2	+	2744	grey	0.202106472	FALSE	GLS
Region_1094 0	chr2	19090846 0	19102096 0	112500	16	121	154.363636 4	12.6766124 6	0.67173482 7	0.0302412	-	6772	grey	-0.11746957 5	FALSE	STAT1
Region_1094 1	chr2	19102957 6	19115159 6	122020	16	116	144.333333 3	13.4362693 2	0.81884783 8	0.03439974 4	-	6775	grey	-0.10849871 4	FALSE	STAT4
Region_1094 2	chr2	19124518 5	19142538 9	180204	30	211	243.015151 5	13.7163489 5	0.80850042 2	0.02860827 3	+	4430	grey	-0.22422708 1	FALSE	MYO1B
Region_1094 3	chr2	19167806 8	19174109 7	63029	4	14	25.3484848 5	3.84904553 3	0.71106868 7	0.07035866	+	64859	grey	0.144788273	FALSE	NABP1
Region_1094 4	chr2	19179279 7	19182025 7	27460	4	21	33.4545454 5	4.94642627 2	0.73387249 3	0.05975900 6	+	729254	grey	0.101637252	FALSE	LOC729254
Region_1094 5	chr2	19183431 0	19184708 8	12778	5	28	42.7121212 1	7.94938827 2	0.67708438 9	0.05674851 2	-	8436	grey	-0.18749593 4	FALSE	CAVIN2
Region_1094 6	chr2	19184653 4	19204452 5	197991	22	164	195.651515 2	13.5629500 1	0.89397846 1	0.01833291 7	+	10537381 3	grey	-0.13079921 3	FALSE	CAVIN2-AS1
Region_1094 7	chr2	19194904 3	19219493 3	245890	23	187	220.818181 8	13.952567	0.86536957 8	0.02899424 6	-	23671	grey	0.049032042	FALSE	TMEFF2
Region_1094 8	chr2	19434419 0	19457657 8	232388	10	66	86.6515151 5	9.35536444 1	0.78951115 4	0.05308420 2	+	10192740 6	grey	0.056835313	FALSE	LINC01821
Region_1094 9	chr2	19557597 7	19573770 2	161725	13	70	89.9848484 8	9.80501037 4	0.54652580 9	0.03640274 6	+	57181	grey	0.028388842	FALSE	SLC39A10

Region_10950	chr2	195737703	196068837	331134	12	75	102.4090909	9.258638	0.930897321	0.033711042	-	56171	grey	-0.272428714	FALSE	DNAH7
Region_10951	chr2	196194072	196593684	399612	54	370	434.1515152	20.40003199	0.837010233	0.020041182	-	57520	grey	0.013417797	FALSE	HECW2
Region_10952	chr2	196639554	196763490	123936	8	44	56.39393939	8.208586101	0.966446191	0.025799519	+	284992	grey	-0.184669648	FALSE	CCDC150
Region_10953	chr2	196833004	196927796	94792	12	72	92.66666667	9.148910542	0.809243616	0.020766823	-	80055	grey	0.063592834	FALSE	PGAP1
Region_10954	chr2	196967017	197311173	344156	54	386	443.1969697	23.98026092	0.904411777	0.014959045	-	91526	grey	0.067999852	FALSE	ANKRD44
Region_10955	chr2	197250858	197302519	51661	6	28	42.39393939	6.2336032	0.963221706	0.027565026	-	101927547	grey	-0.092355791	FALSE	ANKRD44-IT1
Region_10956	chr2	197388515	197435079	46564	6	23	47.87878788	7.263216392	0.81686877	0.030113416	-	23451	grey	0.043328582	FALSE	SF3B1
Region_10957	chr2	197453493	197475310	21817	11	69	99.59090909	10.79882796	0.705975622	0.01892146	+	80219	grey	-0.159761978	FALSE	COQ10B
Region_10958	chr2	197486584	197516737	30153	13	98	131.8484848	12.73063226	0.594673295	0.016260288	-	3329	grey	0.047302417	FALSE	HSPD1
Region_10959	chr2	197500413	197550726	50313	17	117	152.7121212	12.22829651	0.852137914	0.017873967	+	100529241	grey	0.221629652	FALSE	HSPE1-MOB4
Region_10960	chr2	197515571	197553699	38128	11	62	93.16666667	8.647958111	0.882255378	0.02185163	+	25843	grey	0.399893325	FALSE	MOB4
Region_10961	chr2	197568224	197676045	107821	17	123	151.469697	11.69249555	0.922757833	0.02194064	-	130132	grey	-0.23399507	FALSE	RFTN2
Region_1096	chr2	19770536	19770839	3026	3	19	28.7272727	5.15765437	0.41140443	0.07150784	+	92935	grey	-0.13570440	FALSE	MARS2

2		9	5				3		9	7				5		
Region_10963	chr2	197726879	197786574	59695	10	62	84.8030303	8.823429818	0.932744394	0.034805704	-	66037	grey	0.407099226	FALSE	BOLL
Region_10964	chr2	197804593	198149884	345291	40	313	354.9545455	17.92070725	0.789458994	0.02381898	+	5334	grey	0.052074707	FALSE	PLCL1
Region_10965	chr2	198299276	198375490	76214	17	117	142.4848485	12.41617112	0.869360942	0.039238096	-	101927619	grey	-0.432096356	FALSE	LINC01923
Region_10966	chr2	198346829	198572581	225752	29	201	235.3333333	16.64686003	0.858113249	0.029901981	+	105373830	grey	0.00950347	FALSE	LOC105373830
Region_10967	chr2	198493242	198772356	279114	22	167	191.2424242	13.43824692	0.843636628	0.03625892	-	105373831	grey	0.17238178	FALSE	LOC105373831
Region_10968	chr2	199269505	199471266	201761	16	98	134.1515152	13.10459981	0.495157604	0.028216031	-	23314	grey	-0.001089007	FALSE	SATB2
Region_10969	chr2	199457837	199476504	18667	8	53	75.31818182	9.701957124	0.149323256	0.030866728	+	150538	grey	-0.26920364	FALSE	SATB2-AS1
Region_10970	chr2	199608062	199750341	142279	8	48	66.71212121	8.245773092	0.845919922	0.037424479	+	101927641	grey	0.357653155	FALSE	LINC01877
Region_10971	chr2	199760544	199851192	90648	7	36	52.43939394	7.30253652	0.675910276	0.035370512	-	348751	grey	0.0520061	FALSE	FTCDNL1
Region_10972	chr2	199867396	199911159	43763	3	10	26.13636364	5.325949142	0.958633681	0.040623288	-	101927687	grey	-0.036233564	FALSE	LOC101927687
Region_10973	chr2	199911293	199955935	44642	10	67	89.89393939	9.88102889	0.874466882	0.01923857	+	205327	grey	0.029165191	FALSE	C2orf69
Region_10974	chr2	199955317	200008540	53223	6	31	45.03030303	6.950306461	0.948233889	0.03548513	+	79568	grey	-0.070771732	FALSE	MAIP1

Region_10975	chr2	200305881	200482264	176383	44	322	366.4242424	18.97290425	0.809803238	0.021703957	+	26010	grey	-0.101099411	FALSE	SPATS2L
Region_10976	chr2	200390666	200478018	87352	22	144	176.3939394	10.72865149	0.89057305	0.026411145	-	101927741	grey	0.0493116	FALSE	LOC101927741
Region_10977	chr2	200488958	200519784	30826	3	19	24.93939394	4.281907511	0.644637756	0.033540139	-	130535	grey	-0.203192978	FALSE	KCTD18
Region_10978	chr2	200510008	200584096	74088	9	37	59.8030303	8.372518594	0.755957098	0.022503088	+	151246	grey	-0.164470289	FALSE	SGO2
Region_10979	chr2	200586014	200677064	91050	9	62	81.33333333	9.23926959	0.856584787	0.03312039	+	316	grey	0.02941701	FALSE	AOX1
Region_10980	chr2	200678233	200794239	116006	9	57	74.42424242	7.270722334	0.731493739	0.036211925	+	107161151	grey	0.223166552	FALSE	AOX3P-AOX2P
Region_10981	chr2	200711535	200735203	23668	4	25	35.78787879	5.336421018	0.485235836	0.047757898	-	100507140	grey	0.414117694	FALSE	LINC01792
Region_10982	chr2	200780495	200812170	31675	4	18	30.25757576	5.583796905	0.917423128	0.056748887	-	101927795	grey	0.06437001	FALSE	BZW1-AS1
Region_10983	chr2	200810594	200827338	16744	5	27	45.54545455	7.177275717	0.779082391	0.025250068	+	9689	grey	-0.132559012	FALSE	BZW1
Region_10984	chr2	200853009	200864691	11682	4	15	26.5	6.622223656	0.244629837	0.033934245	-	1195	grey	0.302201786	FALSE	CLK1
Region_10985	chr2	200870907	200889303	18396	4	19	32.34848485	6.281823411	0.972183419	0.029182689	-	53938	grey	0.176255677	FALSE	PPIL3
Region_10986	chr2	200889327	200903938	14611	4	23	37.75757576	5.738025943	0.489381641	0.021111731	+	60491	grey	-0.32529265	FALSE	NIF3L1
Region_1098	chr2	20090897	20096368	54703	6	38	57.0606060	7.02496381	0.92261845	0.03624610	-	4999	grey	-0.07250377	FALSE	ORC2

7		7	0				6	1	6	5				6		
Region_10988	chr2	200973718	201071671	97953	6	38	53.43939394	7.058282179	0.974554471	0.024487072	-	285172	grey	-0.227663021	FALSE	HYCC2
Region_10989	chr2	201071433	201085750	14317	4	17	24.68181818	4.054092295	0.977434748	0.038147682	+	4709	grey	-0.081594405	FALSE	NDUFB3
Region_10990	chr2	201116154	201176687	60533	11	74	95.75757576	10.99238982	0.725555871	0.028834405	+	8837	grey	-0.119630302	FALSE	CFLAR
Region_10991	chr2	201182872	201229428	46556	7	53	65.74242424	6.227261668	0.679444339	0.040695383	+	843	grey	0.205379922	FALSE	CASP10
Region_10992	chr2	201233443	201361836	128393	12	90	105.7878788	8.860482973	0.689427209	0.028163983	+	841	grey	-0.141957152	FALSE	CASP8
[truncated: 366,705 more chars]
